# An Efficient Strategy Combining SSR Markers- and Advanced QTL-seq-driven QTL Mapping Unravels Candidate Genes Regulating Grain Weight in Rice

**DOI:** 10.3389/fpls.2016.01535

**Published:** 2016-10-26

**Authors:** Anurag Daware, Sweta Das, Rishi Srivastava, Saurabh Badoni, Ashok K. Singh, Pinky Agarwal, Swarup K. Parida, Akhilesh K. Tyagi

**Affiliations:** ^1^National Institute of Plant Genome Research (NIPGR)New Delhi, India; ^2^Rice Section, Division of Genetics, Indian Agricultural Research Institute (IARI)New Delhi, India

**Keywords:** grain weight, QTL, QTL-seq, rice, SNP, SSR

## Abstract

Development and use of genome-wide informative simple sequence repeat (SSR) markers and novel integrated genomic strategies are vital to drive genomics-assisted breeding applications and for efficient dissection of quantitative trait loci (QTLs) underlying complex traits in rice. The present study developed 6244 genome-wide informative SSR markers exhibiting *in silico* fragment length polymorphism based on repeat-unit variations among genomic sequences of 11 *indica, japonica, aus*, and wild rice accessions. These markers were mapped on diverse coding and non-coding sequence components of known cloned/candidate genes annotated from 12 chromosomes and revealed a much higher amplification (97%) and polymorphic potential (88%) along with wider genetic/functional diversity level (16–74% with a mean 53%) especially among accessions belonging to *indica* cultivar group, suggesting their utility in large-scale genomics-assisted breeding applications in rice. A high-density 3791 SSR markers-anchored genetic linkage map (IR 64 × Sonasal) spanning 2060 cM total map-length with an average inter-marker distance of 0.54 cM was generated. This reference genetic map identified six major genomic regions harboring robust QTLs (31% combined phenotypic variation explained with a 5.7–8.7 LOD) governing grain weight on six rice chromosomes. One strong grain weight major QTL region (*OsqGW5.1*) was narrowed-down by integrating traditional QTL mapping with high-resolution QTL region-specific integrated SSR and single nucleotide polymorphism markers-based QTL-seq analysis and differential expression profiling. This led us to delineate two natural allelic variants in two known *cis*-regulatory elements (RAV1AAT and CARGCW8GAT) of glycosyl hydrolase and serine carboxypeptidase genes exhibiting pronounced seed-specific differential regulation in low (Sonasal) and high (IR 64) grain weight mapping parental accessions. Our genome-wide SSR marker resource (polymorphic within/between diverse cultivar groups) and integrated genomic strategy can efficiently scan functionally relevant potential molecular tags (markers, candidate genes and alleles) regulating complex agronomic traits (grain weight) and expedite marker-assisted genetic enhancement in rice.

## Introduction

A diverse array of random and sequence-based markers such as simple sequence repeats (SSRs)/microsatellites and single nucleotide polymorphism (SNPs) developed hitherto have been successfully employed in manifold genomics-assisted breeding applications of crop plants (Kalia et al., 2011; Gupta et al., 2013; Hayward et al., 2015). However, SSRs remain the most popular and preferred marker for a wide-array of plant genetic analysis. The popularity of SSR markers can be ascribed to a number of desirable genetic attributes including co-dominant inheritance, high reproducibility, robust amplification, multi-allelic nature, and wide genomic distribution in different coding and non-coding sequence components of genome (Li et al., 2004; Varshney et al., 2005; Parida et al., 2006; Miah et al., 2013). Furthermore, amenability of these markers to multiplex high-throughput genotyping makes them the prime choice for various plant structural, functional and comparative genomics applications (Ashkani et al., 2011; Luo et al., 2013; Zhang et al., 2014; Kaur et al., 2015).

Until recently, the development of genic and genomic SSR markers majorly relied upon screening of SSR-enriched/size-fractionated genomic DNA/cDNA libraries and/or mining of SSRs from numerous freely accessible *in silico* ESTs (expressed sequence tags), unigenes and whole genome-sequences of diverse crop accessions including rice (Wu and Tanksley, 1993; Cho et al., 2000; McCouch et al., 2002; Varshney et al., 2002, 2005; International Rice Genome Sequencing Project (IRGSP), 2005; Lawson and Zhang, 2006; Parida et al., 2006; Sharma et al., 2007; Aishwarya and Sharma, 2008; Tang et al., 2008). For instance, the release of early phase draft and gold standard whole genome sequences of rice resulted in generation of large-scale SSR markers including 2240 Rice Microsatellite (RM) markers representing diverse coding and non-coding sequence components of genome (Temnykh et al., 2001; McCouch et al., 2002; International Rice Genome Sequencing Project (IRGSP), 2005). In spite of accessibility to numerous genome-wide SSR markers, significant efforts, time and cost/resources are required for their large-scale validation and genotyping among accessions to select a smaller set of informative polymorphic markers at a whole genome level to be utilized for large-scale genotyping applications in rice. In view of direct correlation between degree of polymorphism and genetic diversity level present among germplasm accessions, the difficulty in selecting such polymorphic informative genome-wide SSR markers increases manifold while dealing with closely related germplasm lines having low level of genetic diversity. The studies pertaining to phylogenetics and domestication of rice have resolved its cultivated accessions belonging to *Oryza sativa* primarily into five different population groups, namely *indica*, tropical and temperate *japonica, aus*, and aromatics, while accessions representing the two wild progenitors (*O. rufipogon* and *O. nivara*) of cultivated species are classified as wild population group (Garris et al., 2005; Caicedo et al., 2007; Parida et al., 2009, 2012; Zhang et al., 2011). Remarkably, these marker-based evolutionary and population genetic structure studies have documented the existence of a very low level of molecular (allelic) diversity among the accessions representing an individual cultivated *O. sativa* population group. Most of the global rice varieties and diverse mapping populations commonly utilized for identification and mapping of quantitative trait loci (QTLs)/genes governing important agronomic traits, have been and/or are being developed by inter-crossing of contrasting accessions belonging to same population-group (*indica* × *indica*) or between related populations (tropical *japonica* × temperate *japonica*). Moreover, the narrow genetic base on account of diverse domestication bottlenecks and modern breeding efforts culminating into low polymorphic potential of markers among cultivated accessions of *indica, japonica, aus* and aromatic populations (association panel) greately impede their efficient utilization in marker-assisted genetic enhancement of rice (Caicedo et al., 2007; Mather et al., 2007). Henceforth, currently the selection of genome-wide informative SSR markers, which exhibit higher degree of polymorphism within different population groups of rice, is the most vital concern in utilization of these markers for large-scale genotyping applications and genomics-assisted crop improvement.

To date, various strategies have been employed to select highly polymorphic informative SSR markers at a genome-wide scale in rice. These approaches include repeat length-based classification of SSRs into longer hyper-variable class I (≥20 bp) and variable class II (12–19 bp) (Temnykh et al., 2001) as well as screening of highly variable SSRs (HvSSR) with repeat length of 51–70 bp (Singh et al., 2010) in rice. To substantiate these efforts, a large-scale genome-wide SSR makers derived from the genic non-coding sequence components (promoters, up/downstream regulatory regions and introns) of rice genes revealing higher polymorphic potential than coding-SSR markers among accessions belonging to diverse *O. sativa* population groups have been developed (Parida et al., 2009). Additionally, the available genome sequences of *japonica* cv. Nipponbare and *indica* cv. 93-11 have paved the way for development of ∼50,000 *in silico* polymorphic genomic and gene-derived SSR markers by analyzing the expansion/contraction of repeat-units between these two accessions (Grover et al., 2007; Zhang et al., 2007). This strategy is advantageous for screening a smaller set of informative SSR markers from large-scale genome-wide markers by assessing their potential to detect fragment length polymorphism between accessions *in silico* prior to synthesis of the primers and thus these markers can be experimentally validated with minimal resource expenses. Furthermore, the degree of *in silico* repeat-length variations predetermined for these polymorphic SSR markers prior to their experimental validation can assist us to select most-suitable genotyping assay for efficient resolution and accurate detection of fragment length polymorphism as well as allelic discrimination among accessions. The implication of *in silico* polymorphic SSR markers developed at a genome-wide scale for manifold high-throughput genetic analyses have been well-demonstrated in crop plants including rice (Zhang et al., 2007; Khajuria et al., 2015; Parida et al., 2015).

In recent years, tremendous efforts on NGS (next-generation sequencing)-based genome and transcriptome sequencing and advent of advanced computational genomics tools have expedited the generation of enormous genomic and transcript sequence resources for multiple rice accessions representing diverse cultivated and wild populations^1^ (Jain et al., 2014; Sakai et al., 2014). Therefore, it is now possible to extend the analysis of *in silico* polymorphic SSR markers development previously documented between *indica* (93-11) and *japonica* (Nipponbare) into a larger set of rice accessions belonging to diverse population groups by comparing their freely accessible genomic sequences at a whole genome level. These efforts will expedite screening of a significant number of informative (high polymorphic potential) *in silico* polymorphic SSR markers differentiating accessions belonging to same and/or related (different) rice populations at a genome-wide scale. Essentially, this will serve as a vital genomic resource to overcome the hindrance of low intra sub-specific polymorphism especially observed within a cultivated population group, which is much required for driving genomics-assisted breeding applications and genetic enhancement in rice. In this regard, primarily the conventional QTL mapping driven by high-throughput genotyping of genome-wide informative *in silico* parental polymorphic SSR markers among mapping individuals of diverse bi-parental populations could prove useful to identify major QTLs governing vital agronomic traits in rice (Fan et al., 2006; Bai et al., 2010; Fuv et al., 2010; Shanmugavadivel et al., 2013; Vemireddy et al., 2015). Aside traditional QTL mapping, recently developed NGS-led QTL-seq assay, dealing with whole genome resequencing of two DNA-bulks/homozygous individuals (with extreme contrasting phenotypic trait values) from a mapping population, appears much promising for quick molecular mapping of major QTLs associated with various quantitative traits in rice (Takagi et al., 2013). Furthermore, the implication of an integrated genomic strategy involving QTL-seq and differential transcript profiling to scale-down traditional QTL mapping-derived long major QTL genomic regions into potential candidate genes is well-documented in many crop plants (Das et al., 2015, 2016; Kujur et al., 2015). Unfortunately, no such afore-mentioned strategy beside fine mapping/map-based cloning is reported so far to identify genes underlying major QTLs controlling traits of agronomic importance in rice. In this prespective, the trait-associated major QTLs identified and mapped on the rice chromosomes using *in silico* polymorphic SSR marker-based traditional QTL mapping can be narrowed-down by combining traditional QTL mapping with QTL region-specific high-resolution integrated SSR and SNP marker-based QTL-seq analysis and differential expression profiling to scan potential candidate genes/alleles regulating complex quantitative traits for marker-assisted genetic improvement of rice.

In view of the above facts, the current study generated numerous genome-wide/gene-derived *in silico* polymorphic SSR markers to evaluate their efficacy for various large-scale genomics-assisted breeding applications in rice. Further, effort has been made to develop an efficient integrated genomic strategy combining *in silico* polymorphic SSR marker-based traditional QTL mapping, QTL region-specific high-resolution integrated SSR and SNP marker-based QTL-seq analysis and differential expression profiling for delineation of functionally relevant candidate genes and natural allelic variants underlying a major QTL regulating grain weight in rice.

## Materials and Methods

### Genome-Wide Discovery and Characterization of *In silico* Polymorphic SSR Markers in Rice

The latest pseudomolecule sequences of two accessions representing *japonica* (Nipponbare) and *aus* (Kasalath) rice populations were retrieved from the MSU rice genome annotation project release ^2^ 7.0 and DDBJ^3^, respectively. The short-read sequences of nine accessions belonging to *aus* (Nagina 22), *indica* (IR 64, Pokkali and Bala), *japonica* (Tainung, Azucena and Moroberekan) and wild (*O. rufipogon* and *O. nivara*) populations were retrieved from the NCBI SRA database^4^ (Supplementary Table S1). The sequences of nine accessions were mapped individually to a Nipponbare reference genome sequence to generate the reference-based genome assembly of each accession using the CLC Genomics workbench (with default parameters) as per Agarwal et al. (2012). Accordingly, genome sequences of nine accessions were constituted individually for further analysis.

The genome sequences of 11 rice accessions including Kasalath and Nipponbare were analyzed with MISA (MIcroSAtellite^5^) for mining SSRs ensuing the criteria (at least six repeats of di-nucleotides and five repeats of tri- to hexa-nucleotides) as described previously (Garg et al., 2011; Jhanwar et al., 2012). The perfect SSRs exhibiting repeat-length variations flanked by conserved genomic sequences (against Nipponbare reference genome) at least between any two of 11 rice accessions were screened and defined as ‘*in silico* polymorphic perfect SSRs’ in accordance with Khajuria et al. (2015) and Parida et al. (2015). To develop *in silico* polymorphic perfect SSR markers at a genome-wide scale, the forward and reverse primer-pairs were designed from the sequences flanking the polymorphic perfect SSR repeats employing the Primer3 interface modules of MISA^6^. These SSR markers were further classified and charcterized into di-, tri-, tetra-, penta-, and hexa-nucleotides based on nature/types of repeat-motifs as well as hypervariable class I (≥20 bp) and potentially variable class II (12–18 bp) based on length of repeats. The developed polymorphic SSR markers were physically mapped on the 12 rice chromosomes as well as structurally and functionally annotated based on their physical locations in the coding and non-coding regions of the rice genome/genes as per the available rice genome annotation (MSU rice genome annotation project release 7.0). The effect of polymorphic SSR markers based on their repeat-unit length variations within CDS for the amino acid sequence alteration of encoded protein was determined using a customized perl script. Additionally, the developed SSR markers were categorized into two groups, namely ‘within population’ and ‘between populations’ based on their polymorphism at least between any two accessions belonging to same population and different populations of rice, respectively. The SSR markers ‘within population’ were further classified into four sub-groups (within *indica*, within *aus*, within *japonica*, and within wild) based on populations within which they are polymorphic. The SSR markers ‘between populations’ were categorized into six different sub-groups (*indica* vs. *aus, indica* vs. *japonica, indica* vs. wild, *aus* vs. *japonica, aus* vs. wild and *japonica* vs. wild) based on polymorphism of these markers between any two accessions belonging two different populations.

### Molecular Diversity and Phylogeny

The allelic data of SSR markers (physically mapped across 12 rice chromosomes) revealing *in silico* polymorphism among 11 rice accessions were analyzed in PowerMarker v3.5 (Liu and Muse, 2005) and MEGA6 (Tamura et al., 2013). Accordingly, determination of molecular diversity and construction of unrooted phylogenetic tree (Nei and Li’s similarity coefficient-based neighbor joining method) among accessions were performed.

### Experimental Validation, Amplification and Polymorphic Potential of SSR Markers

For experimental validation of *in silico* polymorphic SSR markers, the genomic DNA was isolated from 24 rice accessions representing lowland *indica* (three accessions), upland *indica*/*aus* (3), *japonica* (1), long (13)- and short (2)-grained aromatics, and wild (2) population groups. To determine the amplification and polymorphic potential of markers, *in silico* polymorphic SSR markers designed from various known cloned and candidate transcription factor (TF) genes (physically mapped on 12 rice chromosomes) regulating stress tolerance, yield and quality component traits in rice were selected. In addition, SSR markers revealing *in silico* fragment length polymorphism based on repeat-unit variation among accessions especially belonging to *indica* population group were included. These markers were PCR amplified with the genomic DNA of rice accessions using standard PCR constituents and touchdown thermal cycling profiling as described by Jhanwar et al. (2012) and Kujur et al. (2013). The PCR product of SSR markers (with ≥10-bp *in silico* fragment length polymorphism) amplified from accessions were resolved in 3.5% metaphor agarose gel and their fragment size (bp) was estimated against 50-bp DNA ladder size standard. The SSR markers showing 2–9 bp *in silico* fragment length polymorphism were validated and genotyped through PCR amplicon sequencing by automated 96 capillary ABI 3730xl DNA Analyzer (Applied Biosystems, USA) as per Kujur et al. (2013) and Saxena et al. (2014). The genotyping data of experimentally validated SSR markers among accessions was analyzed in PowerMarker to estimate the polymorphic potential (%) and polymorphism information content (PIC) among rice accessions.

### Development and Phenotyping of a Mapping Population

A *F*_4_ mapping population (190 segregating individuals) derived from the bi-parental crosses between high (*indica* accession IR 64 with 1000-grain weight: 25 g) and low (short-grained aromatic accession Sonasal: 10 g) grain weight parental accessions was generated by single seed descent method. The mapping individuals along with their parental accessions were grown in the field (as per randomized complete block design) for two consecutive years during crop growing season at two different geographical regions (New Delhi and Tamil Nadu) of India and *F*_4:5_ individuals were phenotyped for 1000-grain weight (g). The grain weight was estimated by measuring the average weight (g) of 1000-matured seeds from 10 representative plants of each mapping individual and parental accession. The frequency distribution, coefficient of variation (CV) and broad-sense heritability (*H*^2^) of grain weight across years/seasons were determined in the mapping population as per Bajaj et al. (2015a).

### Genetic Linkage Map Construction and Traditional QTL Mapping

The SSR markers exhibiting polymorphism between high and low grain weight parental accessions of a *F*_4_ mapping population (IR 64 × Sonasal) were screened from our marker polymorphism study especially within *indica* population group. These informative markers were PCR amplified and genotyped using the genomic DNA of 190 *F*_4_ mapping individuals and two parental accessions (IR 64 and Sonasal) as per aforementioned agarose gel- and PCR amplicon sequencing-based genotyping assays. The markers exhibiting goodness-of-fit to the expected Mendelian 1:1 segregation ratio (χ^2^-test at *p* < 0.05) were analyzed in JoinMap^7^ 4.1 at a higher LOD threshold (5.0) with Kosambi mapping function to estimate linkage analysis among the markers used. For constructing of a high-density genetic map, the markers were integrated into defined linkage groups (LGs) based on their centi Morgan (cM) genetic distances and corresponding marker physical positions (bp) on the chromosomes, and finally visualized using MapChart v2.2 (Voorrips, 2002).

For identification and molecular mapping of major grain weight QTLs, the genotyping data of informative SSR markers genetically mapped on 12 rice chromosomes was correlated with 1000-grain weight field phenotypic data of 190 mapping individuals and parental accessions employing composite interval mapping (CIM) function (LOD threshold score > 5.0 with 1000 permutations and *p* < 0.05 significance) of MapQTL 6 (Van Ooijen, 2009). The additive effect and phenotypic variation explained (PVE) by each major grain weight QTL at a significant LOD were measured following Bajaj et al. (2015a).

### QTL Interval-Targeted Multiplex-Amplicon Resequencing

For QTL region-specific resequencing analysis, one strong grain weight-associated major genomic region underlying a robust QTL identified by traditional QTL mapping was selected. For this, 10 of each low and high grain weight homozygous individuals (a sum of 20 individuals) representing two utmost ends of 1000-grain weight (g) normal frequency distribution curve were selected from a mapping population (IR 64 × Sonsal) based on the clues that were obtained from our traditional QTL mapping study. From traditional QTL mapping, the genotyping data of 96 genome-wide SSR markers including SSR markers tightly linked to our identified specific major grain weight QTL was correlated with grain weight field phenotypic information to ascertain the homozygous genetic constitution of selected 20 mapping individuals for either of the low and high grain weight trait. The genomic DNA was isolated from the young leaves of 20 low and high grain weight homozygous individuals and parental accessions using QIAGEN DNeasy96 Plant Kit (QIAGEN, USA) according to the manufacturer’s protocol. The genomic DNA isolated from 10 of each low and high mapping individual was quantified to equal concentration and pooled to constitute low grain weight bulk (LGWB) and high grain weight bulk (HGWB). A selected strong grain weight-associated major genomic region harboring a robust QTL was sequenced in parental accessions as well as LGWB and HGWB using the multiplexed amplicon sequencing protocol of TruSeq Custom Amplicon v1.5 in Illumina MiSeq next-generation sequencer (Illumina, USA).

Primarily, the genomic region underlying a QTL was targeted to design and synthesize the custom oligo probes employing Design Studio software (Illumina, USA). All the synthesized probes were pooled into a custom amplicons tube to produce ∼10000 amplicons with an average of 500 bp amplicon size per reaction and further template library was made using TruSeq Custom Amplicon Assay kit v1.5. The common primers from the TruSeq Amplicon Index kit were utilized to tag sample-specific indices to each library by PCR. The uniquely tagged pooled amplicon libraries were normalized and the generated clusters were sequenced through Illumina MiSeq platform. The sequenced amplicons and sequence variants were visualized with Illumina Amplicon Viewer. The high-quality gene amplicon-sequence reads (∼100-fold sequencing depth-coverage) of parental accessions and/or mapping individuals constituting the LGWB and HGWB were mapped on 12 chromosome pseudomolecules of reference Nipponbare rice genome^8^. Accordingly, high-quality non-erroneous and homozygous SNPs (minimum 10 sequence read depth with a SNP base quality ≥ 20) were detected among parental accessions and/or mapping individuals representing LGWB and HGWB as per Jain et al. (2014), Saxena et al. (2014) and Kujur et al. (2015). The polymorphic informative SSRs were identified by comparing the constituted reference genome-based assemblies of parental accessions as well as mapping individuals of LGWB and HGWB following the aforesaid methods. The identified SNPs and SSRs were structurally and functionally annotated with respect to reference Nipponbare genome annotation database (as per Jain et al., 2014).

### QTL Interval-Targeted QTL-seq Analysis

The SNP/SSR-index and Δ (SNP/SSR-index)-based QTL-seq strategy was employed at a target major QTL genomic region to identify major rice GW QTL in accordance with previously defined recommended parameters (Takagi et al., 2013; Lu et al., 2014; Das et al., 2015; Illa-Berenguer et al., 2015). The Δ (SNP/SSR-index) was measured based on subtraction of SNP/SSR-index between LGWB and HGWB. The SNP/SSR-index was measured as ‘1’ and ‘0’ based on the representation of genomic fragments derived from high (IR 64) and low (Sonasal) grain weight mapping parental accessions, respectively, in entire high-quality sequence reads generated. To estimate the average distribution of Δ (SNP/SSR-index) of SNPs physically mapped on specific rice chromosome in a given genomic interval, a 1 Mb window-size and 10 kb increment sliding window strategy was utilized. The Δ (SNP/SSR-index) of LGWB and HGWB, and their corresponding SNP/SSR-index within the specified window size in the graphs were plotted to generate the SNP-index plot. To ascertain the accuracy of major QTL identified by QTL-seq, the statistical confidence interval of Δ (SNP/SSR-index) with a given sequence read-depth under the null hypothesis of no QTLs was estimated following Takagi et al. (2013), Lu et al. (2014) and Das et al. (2015).

### Expression Profiling

To infer differential gene regulatory function, differential expression profiling of genes validated and delineated at a major genomic interval harboring a robust GW QTL by traditional QTL mapping and QTL-seq analysis was performed. For experimental validation, the vegetative 7-days old seedlings (considered as control) and five diverse seed developmental stages [S1: 0–2 days after pollination (DAP), S2: 3–4 DAP, S3: 5–10 DAP, S4: 11–20 DAP and S5: 21–29 DAP, defined as per Agarwal et al. (2011)] of high (IR 64) and low (Sonasal) grain weight rice accessions were collected. The RNA was isolated from these tissues/stages following a modified protocol of Agarwal et al. (2007). The purification of isolated RNA, synthesis of cDNA and amplification of cDNA with the gene-specific primers were performed using the quantitative RT-PCR assay as per Bajaj et al. (2015b). In the quantitative RT-PCR assay, three independent biological replicates of each sample and three technical replicates of each biological replicate with no template and primer as control were included. A house-keeping gene *Actin1* was used as internal control in RT-PCR assay for normalization of expression value across various tissues/seed developmental stages of accessions. The significant difference of gene expression observed in diverse tissues/developmental stages of high and low grain weight rice accessions was compared and correlated among each other to construct a heat map using TIGR MultiExperiment Viewer (MeV^9^).

## Results

### Development, Genomic Distribution and Characterization of *In silico* Polymorphic SSR Markers in Rice Genome

**FIGURE 1:**
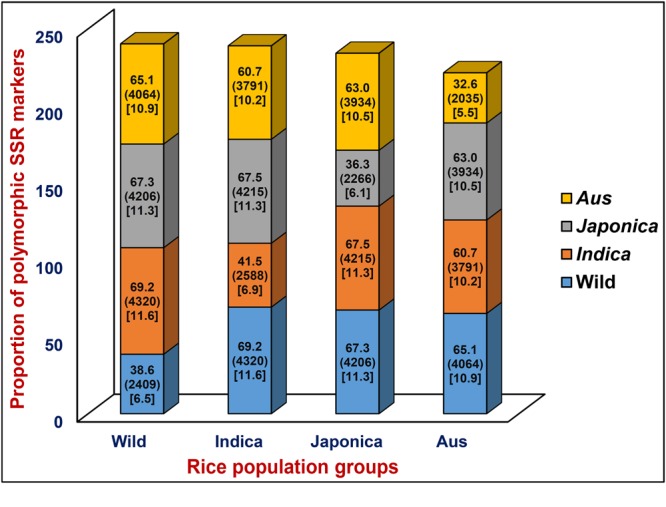
***In silico* potential of polymorphism (denoted by %) detected by genome/gene-derived simple sequence repeat (SSR) markers within and between four different cultivated and wild rice population groups (*indica, aus, japonica*, and wild).** Digits mentioned within the parentheses indicate the polymorphic SSR markers represented by (number) and density [number of markers/Mb]. The markers polymorphic within and between four rice population groups are highlighted with diverse colors.

A total of 6244 *in silico* polymorphic SSR markers based on repeat-unit length variations among the genomic sequences of 11 accessions were developed at a genome-wide scale (Supplementary Tables S1 and S2). These markers were mapped on the 12 rice chromosomes with an average marker density of 16.6 markers/Mb (Supplementary Figures S1A,B). Chromosomes 3 and 4 exhibited highest and lowest marker density of 19.0 and 14.7 markers/Mb, respectively (Supplementary Figure S1B). About 53.1 and 46.9% of the identified 6244 SSR markers were hypervariable class I and variable class II types, respectively. The dinucleotide repeat-motifs were most abundant followed by trinucleotide repeats in both classes I and II SSRs. The structural annotation revealed an occurrence of a highest and lowest proportion of SSR markers in the non-coding and coding sequence components of rice genome with the marker densities of 13.6 and 3.0 markers/Mb (**Table 1**; Supplementary Figure S1B). Among the non-coding SSR markers, 12.4% (632) and 87.6% (4465) markers were derived from the UTR and intronic/intergenic sequence components of genes, respectively. An overall trend of SSR markers distribution observed in the whole rice genome based on relative density of their repeat-motif types and repeat-unit length was almost similar to that obtained within each type of non-coding and coding sequence components of rice genes (**Table 1**; Supplementary Figure S1B). Interestingly, 136 *in silico* polymorphic SSR markers were present in coding and non-coding sequence components of 131 known cloned genes characterized for diverse yield component and abiotic/biotic stress tolerance traits in rice^10^ (Supplementary Table S2; Supplementary Figure S1A). A set of 243 SSR markers were developed from the coding and non-coding regions of 137 TF genes annotated from the rice genome (Supplementary Table S2). The detail structural annotation of developed SSR markers revealed that only 3.1% (36) of total 1147 markers exhibiting *in silico* fragment length polymorphism based on repeat-unit length variation in the coding sequence components of genes led to shift in the reading frames, causing alteration in amino acid sequences of encoded proteins (Supplementary Table S2). The remaining 96.9% (1111) markers caused either insertions or deletions without affecting any shift in the reading frames, which resulted expansion/contraction of similar kinds of amino acid sequences of encoded proteins (Supplementary Table S2). The developed genome-wide SSR markers were classified based on their *in silico* fragment length polymorphism within and between rice population groups (**Figure 1**). A highest proportion of SSR markers detected polymorphism within *indica* population (41.5%, 2588 markers) with an average density of 6.9 markers/Mb. This was followed by wild population (38.6%, 2409 markers) with a mean density of 6.5 markers/Mb. The SSR markers were found most polymorphic between *indica* and wild populations (69.2%, 4320 markers) with an average density 11.6 markers/Mb and least between *indica* and *aus* population (**Figure 1**). Collectively, the developed markers had higher potential to detect polymorphism between populations (65.5% mean polymorphic potential with an average density of 11 markers/Mb) as compared to that within populations (37.2% with 6.2 markers/Mb).

**Table 1:** Characteristics of *in silico* polymorphic SSR markers developed from the rice genome.

SSR repeat-motifs	Class I (≥20 bp)				Class II (12-20 bp)				Grand Total		
	Non-coding		Coding	Total	Non-coding		Coding	Total	Non-coding	Coding	Total
	Intronic/intergenic	UTR	CDS		Intronic/intergenic	UTR	CDS				
Dinucleotides	1861 (93.5)	118 (5.9)	11 (0.6)	1990 (54.2)	1548 (92.0)	110 (6.5)	25 (1.5)	1683 (45.8)	3637 (99)	36 (1.0)	**3673 (58.8)**
Trinucleotides	465 (40.8)	198 (17.4)	476 (41.8)	1139 (47.8)	418 (33.6)	194 (15.6)	631(50.8)	1243 (52.2)	1275 (53.5)	1107 (46.5)	**2382 (38.1)**
Tetranucleotides	143 (93.5)	9 (5.9)	1 (0.6)	153 (100)	-	-	-	-	152 (99.3)	1 (0.7)	**153 (2.5)**
Pentanucleotides	22 (91.7)	2 (8.3)	0 (0.0)	24 (100)	-	-	-	-	24 (100.0)	0 (0.0)	**24 (0.4)**
Hexanucleotides	8 (66.7)	1 (8.3)	3 (25)	12 (100)	-	-	-	-	9 (75.0)	3 (25.0)	**12 (0.2)**
**Total**	2499 (75.3)	328 (9.9)	491 (14.8)	3318 (53.1)	1966 (67.2)	304 (10.4)	656 (22.4)	2926 (46.9)	5097 (81.6)	1147 (18.4)	**6244**
	2827 (85.2)				2270 (77.6)						

*Values within parentheses represent the proportion of SSR markers. UTR, untranslated region; CDS, coding DNA sequence.*

### Wider Genetic Diversity and Expected Phylogenetic Relationship Revealed by Genome-Wide SSR Markers among Rice Accessions

The determination of genetic diversity level among 11 cultivated and wild rice accessions using 6244 *in silico* polymorphic SSR markers (mapped on 12 rice chromosomes) revealed a broad range of diversity (distance) coefficient that varied from 0.12 to 0.85 with an average of 0.69. The level of diversity among nine cultivated accessions varied from 0.14 to 0.78 with a mean of 0.58. In five *indica* accessions, the degree of diversity ranged from 0.16 to 0.74 with an average of 0.53.

The phylogenetic relationship among 11 rice accessions determined using 6244 genome-wide polymorphic SSR markers was depicted by an unrooted phylogenetic tree (Supplementary Figure S2). The SSR markers distinctly differentiated all these accessions from each other and clustered into two major groups, namely *indica* (IR 64, Pokkali, Nagina22, Kasalath and Bala) and *japonica* (Tainung67, Nipponbare, Azucena and Moroberekan). The *indica* group was further gouped into two sub-groups, lowland (IR 64 and Pokkali) and upland/*aus* (Nagina 22, Kasalath and Bala) *indica* (Supplementary Figure S2). One wild accession belonging to *O. nivara* was clustered with *indica*, whereas an accession of *O. rufipogon* grouped separately from *indica* and *japonica* cultivar groups (Supplementary Figure S2).

### High Marker Amplification and Polymorphic Potential Detected by Genome-Wide SSR Markers among Rice Accessions

A set of 379 *in silico* polymorphic SSR markers designed from various known cloned genes (136) and candidate TF genes (243) governing stress tolerance and yield component traits in rice as well as 3791 markers exhibiting *in silico* fragment length polymorphism among accessions belonging to *indica* population group were selected. These 4170 SSR markers (physically mapped on 12 rice chromosomes) were experimentally validated by agarose gel- and PCR amplicon sequencing-based assays using the genomic DNA of two high and low grain weight accessions, namely IR 64 and Sonasal selected as parents to develop a mapping population for constructing a high-density genetic linkage map and grain weight QTL mapping in rice. Of these, 4048 markers amplified single reproducible PCR amplicons in 3.5% Metaphor agarose gel with an average amplification success rate of 97.1% (Supplementary Figure S3A). The gel- and amplicon sequencing-based genotyping of 4048 amplified SSR markers in these rice accessions identified 3819 (94.3%) markers to be polymorphic, which overall detected 9547 alleles with a mean PIC of 0.73. The number of alleles detected by the SSR markers varied from 1 to 3 alleles with a mean of 2.63 alleles per marker between two rice accessions. The sequencing of varied fragment size (bp) amplicons of SSR markers amplified from two rice accessions overall ascertained their correspondence with expected *in silico* fragment length polymorphism based on repeat-unit variation observed among accessions. A selected set of experimentally validated 384 SSR markers were utilized to evaluate their amplification and polymorphic potential among 24 lowland/upland (*aus*) *indica, japonica* and wild rice accessions (Supplementary Figures S3A,B). A higher potential of polymorphism was detected by the markers among the accessions specifically belonging to lowland/upland (*aus*) *indica* (88% polymorphism and mean PIC: 0.70) rice population group. A higher marker polymorphic potential was observed between cultivated *indica* and wild rice (79% polymorphism and mean PIC: 0.69) as compared to that within cultivated (71%, 0.63) and wild (69%, 0.60) rice. The experimentally validated and *in silico* polymorphic SSR markers were utilized in combination for determining genetic diversity and phylogenetics among 11 cultivated and wild rice accessions along with a low grain weight mapping parental accession Sonasal. This clearly differentiated all 12 accessions from each other and further clustered these accessions into aforementioned four *indica, aus, japonica*, and wild rice population groups in accordance with our outcomes obtained in the present study using *in silico* polymorphic SSR markers solely among 11 rice accessions (Supplementary Figure S4). Notably, the short-grain aromatic mapping parental rice accession Sonasal was grouped with three accessions belonging to an upland (*aus*) *indica* population group.

### Construction of a High-Resolution SSR Marker-Based Genetic Linkage Map

**FIGURE 2:**
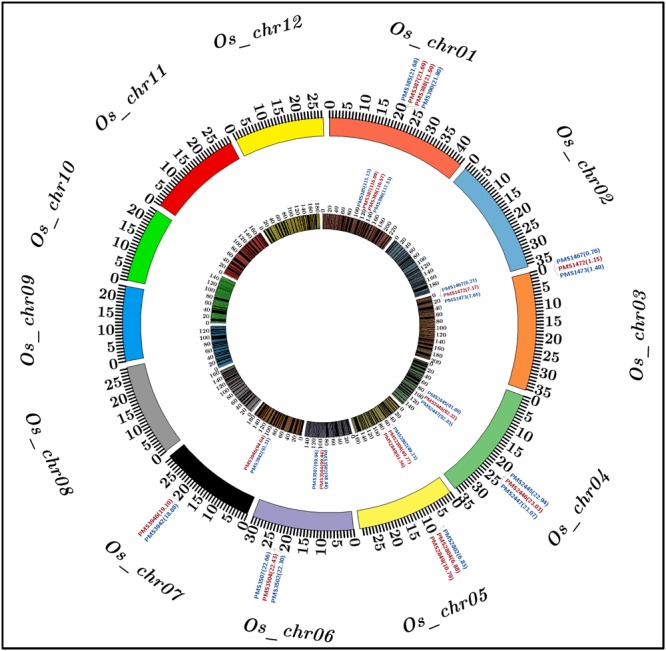
**Molecular mapping of six major grain weight quantitative trait loci (QTLs) on a high-density genetic linkage map (IR 64 × Sonasal) generated by anchoring 3791 genome- and known cloned/candidate genes (TFs)-derived *in silico* polymorphic SSR markers on 12 rice chromosomes, which is depicted by a Circos circular ideogram.** The constructed high-resolution genetic map identified seven SSR markers-containing candidate genes at six major genomic regions harboring grain weight QTLs mapped on six rice chromosomes 1, 3, 4, 5, 6, and 7. The SSR markers flanking and tightly linked to the major grain weight QTLs are highlighted with blue and red color, respectively. The inner circle designates the diverse genetic map-length (cM) (spanning 5 cM uniform genetic distance intervals between bins) of 12 linkage groups/chromosomes coded with multiple colors. The outer circle denotes the various physical sizes (Mb) of 12 chromosomes coded with multiple colors as per the pseudomolecule sizes documented by MSU rice genome annotation project release 7.0. The digits mentioned within the parentheses of inner and outer circles denote genetic (cM) and physical (bp) positions of mapped SSR markers flanking/linked to the major grain weight QTLs, respectively, in rice. The detail information regarding SSR markers are provided in the Supplementary Table S2.

To construct a high-density genetic linkage map, 3819 SSR markers exhibiting polymorphism between two parental accessions (IR 64 and Sonasal) were genotyped among 190 individuals of a *F*_4_ mapping population (IR 64 × Sonasal) using agarose gel- and PCR amplicon sequencing-based genotyping assays. The linkage analysis based on co-dominant inheritance pattern of parental polymorphic SSR markers across segregating mapping individuals led to map 3791 (99.8%) markers across 12 chromosomes of a constructed rice genetic map (Supplementary Table S3; **Figure 2**). The genetic map spanned a total map length of 2060.183 cM with a mean inter-marker distance of 0.543 cM. Highest and lowest number of markers were mapped on chromosomes 1 (485 markers) and 9 (218), respectively (Supplementary Table S3). The chromosome 1 had longest map length spanning 232.216 cM while chromosome 9 contained shortest map length 133.084 cM. The most and least saturated genetic linakge maps were obtained in chromosomes 1 (mean inter-marker distance: 0.479 cM) and 10 (0.639 cM), respectively (Supplementary Table S3).

### Traditional QTL Mapping for Identification of Major Grain Weight QTLs

A significant phenotypic variation for grain weight (1000-grain weight: 9 to 35 g with 83% *H*^2^) trait was observed in 190 mapping individuals (IR 64 × Sonasal) and two parental accessions. A bi-directional transgressive segregation including normal frequency distribution of grain weight trait in mapping individuals and parental accessions was apparent. The genotyping information of 3791 markers genetically mapped on 12 rice chromosomes was integrated with 2 years multi-location field phenotyping data of grain weight for molecular mapping of major QTLs. This analysis enabled to identify six major genomic regions underlying six robust QTLs associated with grain weight, which were mapped on six rice chromosomes (1, 3, 4, 5, 6, and 7) (**Table 2**; **Figure 2**). The individual major QTL explained 11 to 22% phenotypic variation for grain weight at 5.7–8.7 LOD. The PVE measured for all six major QTLs in combination was 31%. The six major genomic regions underlying grain weight QTLs spanned (1.037 cM on chromosome 4 to 2.4 cM on chromosome 1) by 26 SSR markers, were mapped on six rice chromosomes (**Table 2**; **Figure 2**). All six major grain weight QTLs exhibited positive additive gene effects (2.1–4.5) on grain weight with major allelic contribution from a high grain weight parental accession IR 64. The integration of genetic and physical maps specifically at the identified target QTL intervals enabled to detect seven SSR markers-containing genes tightly linked to six major QTLs governing grain weight in rice (**Table 2**; **Figure 2**).

**Table 2:** Molecular mapping of significant quantitative trait loci (QTLs) associated with grain weight in rice.

^∗^QTLs	LGs/Chromosomes	Marker intervals with genetic positions (cM)	QTL physical intervals (bp)	Markers linked with QTLs	Structural annotation	Protein-encoding genes	LOD	PVE (%)	*A*
*OsqGW1.1*	1	PMS385 (115.130) – PMS390 (117.530)	21675678 – 21796054	PMS387 and PMS388	CDS	*bHLH* (basic helix-loop-helix) transcription factor	6.8	14	3.3
*OsqGW3.1*	3	PMS1467 (6.207) – PMS1473 (7.647)	760737 – 1397232	PMS1472	CDS	B3 DNA-binding domain protein	7.6	19	2.9
*OsqGW4.1*	4	PMS2445 (91.797) – PMS2447 (92.834)	22938149 – 23068840	PMS2446	CDS	Myb (myeloblastosis) transcription factor	7.0	12	2.7
*OsqGW5.1*	5	PMS2802 (49.235) – PMS2849 (51.556)	6827053 – 10790507	PMS2804 and PMS2849	CDS	Cytochrome P450 and Serine carboxypeptidase	8.7	22	4.5
*OsqGW6.1*	6	PMS3502 (98.443) – PMS3507 (99.936)	22300982 – 22658873	PMS3504	CDS	Expressed protein	6.5	15	3.0
*OsqGW7.1*	7	PMS3942 (92.308) – PMS3946 (94.039)	18876301 – 19103259	PMS3946	URR	SBP (Squamosa-promoter binding protein) transcription factor	5.7	11	2.1

*^∗^*OsqGW1.1* (*Oryza sativa* QTL for grain weight on chromosome 1 number 1), PVE: phenotypic variation explained by QTLs, A, additive effect; positive additive effect infers alleles from IR 64 with grain weight values. Details regarding PMS (polymorphic SSR) markers are mentioned in the Supplementary Table S2.*

### QTL Region-Specific QTL-seq Analysis Expedites Scale-Down of the Genomic Region Harboring a Major Grain Weight QTL in Rice

**FIGURE 3:**
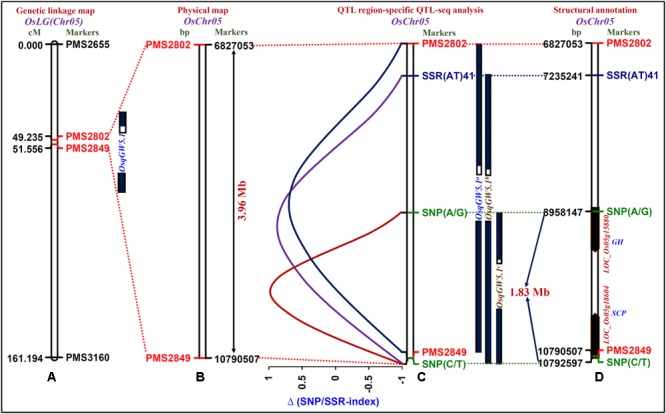
**The integration of genetic linkage map **(A)** with physical **(B)** map of a target genomic region harboring one major grain weight QTL (*OsqGW5.1*) identified and mapped on 3.96 Mb sequence interval (between the flanking markers PMS2802 and PMS2849, indicated with red color) of rice chromosome 5. (C)** The *OsqGW5.1* QTL region-specific high-resolution integrated SSR and SNP marker-based QTL-seq analysis identified three major QTLs, *OsqGW5.1*^a^ (between the flanking markers PMS2802 and PMS2849), *OsqGW5.1*^b^ [SSR(AT)_41_ and SNP (C/T)] and *OsqGW5.1*^c^ [SNP(A/G) and SNP(C/T)]. Subsequently, the integration of traditional QTL mapping with QTL region-specific Δ (SNP/SSR-index)-based QTL-seq analysis narrowed-down the 3.96 Mb *OsqGW5.1* major QTL region into a shorter 1.83 Mb *OsqGW5.1*^c^ sequenced QTL region (between flanking markers SNP (A/G)-SNP (C/T), marked with green color) on chromosome 5. **(D)** Two regulatory SNPs (A/G and C/T)-containing candidate genes [LOC_Os05g15880 (glycosyl hydrolase) and LOC_Os05g18604 (serine carboxypeptidase)] annotated within a novel 1.83 Mb sequenced major *OsqGW5.1^c^* QTL interval exhibited strong association with rice grain weight according to Δ (SNP-index)-based QTL-seq study. The genetic (cM)/physical (bp) distance and identity of the markers mapped on the chromosomes are indicated on the left/right side of the chromosomes, respectively. The details regarding SNPs (green color) and SSRs (blue and black colors) flanking/localized at the major grain weight QTLs are mentioned in the **Table 2** and Supplementary Table S2.

One strong (highest 22% PVE with a 8.7 LOD) major grain weight QTL (*OsqGW5.1*) interval [PMS2802 (49.235 cM) – PMS2849 (61.556 cM)] was selected considering the recombination between two SSR markers flanking this QTL and genetic constitution of low and high grain weight mapping individuals (**Figure 3A**). The integration of genetic linkage map information with physical map defined this major grain weight QTL interval into 3963454 bp (6827053 – 10790507 bp) genomic physical region on chromosome 5 (**Figure 3B**). The targeted sequencing of this 3.96 Mb *OsqGW5.1* QTL interval along with its additional 3 kb flanking genomic region in parental accessions (IR 64 and Sonasal) as well as low (LGWB) and high (HGWB) grain weight homozygous bulks of a mapping population (IR 64 × Sonasal) discovered 455 SSRs and 319 SNPs.

The SNP/SSR-index for individual SNPs/SSRs exhibiting differentiation between high (IR 64 and HGWB) and low (Sonasal and LGWB) grain weight mapping parents and bulks was determined. A mean SNP/SSR-index across a 1 Mb genomic interval was estimated individually in IR 64 and HGWB as well as Sonasal and LGWB employing a 10 kb sliding window approach and was further plotted against rice chromosome 5. The Δ (SNP/SSR-index) was estimated by integrating the SNP/SSR-index information of HGWB and LGWB, and plotted against the genomic position (Mb) of rice chromosome 5. Three major QTLs (*OsqGW5.1*^a^, *OsqGW5.1*^b^, and *OsqGW5.1*^c^) governing grain weight within a *OsqGW5.1* major QTL interval (identified by traditional QTL mapping) on chromosome 5 revealing mean Δ (SNP-index) of 0.6, 0.8, and 0.9, respectively, between HGWB and LGWB were detected following the SNP-index-based QTL-seq estimation criteria as defined by Takagi et al. (2013), Lu et al. (2014), and Das et al. (2015) (**Figure 3C**). These identified QTLs spanned 5747996 [*OsqGW5.1*^a^-6827053 (PMS2802) to 1079057 (PMS2849) bp], 3557266 [*OsqGW5.1*^b^-SSR(AT)_41_: 7235241 bp to SNP (C/T): 10792507 bp] and 1834360 [*OsqGW5.1*^c^-SNP(A/G): 8958147 bp to SNP(C/T): 10792507 bp] bp genomic regions underlying a *OsqGW5.1* QTL on chromosome 5 (**Figure 3C**). These three major genomic regions detected by QTL interval-specific QTL-seq analysis were ascertained by Δ (SNP-index) value, which was significantly different from 0 at a 99% significance level. The comprehensive analysis of these target genomic regions indicated that majority of the SSR and/or SNP alleles derived from high (IR 64) and low (Sonasal) grain weight mapping parental accessions were present in the high and low grain weight mapping individuals constituting the bulks (HGWB and LGWB). The SSRs and SNPs flanking the QTL-seq derived three major grain weight QTLs (*OsqGW5.1*^a^, *OsqGW5.1*^b^, and *OsqGW5.1*^c^) were further validated in accordance with their expected allelic discrimination by resequencing of PCR fragments amplified from the parental accessions (IR 64 and Sonasal) and mapping individuals constituting the HGWB and LGWB. Collectively, the integrated high-resolution SSR and SNP marker-based QTL-seq analysis in a genomic region underlying GW QTL (*OsqGW5.1*) identified one consensus major genomic region with a short physical interval of 1834360 bp [*OsqGW5.1*^c^: SNP(A/G) 8958147 bp to SNP(C/T) 10792507 bp] on chromosome 5 regulating grain weight in rice (**Figure 3D**).

The structural annotation of this 1.83 Mb sequenced *OsqGW5.1*^c^ QTL region with rice genome annotation database identified 271 protein-coding candidate genes. Interestingly, two URR-SNPs [A/G (8958147 bp) and C/T (10792507 bp)] identified in the *cis*-regulatory element [RAV1AAT (CAACA) and CARGCW8GAT (CATAATTATG)] regions of two genes [LOC_Os05g15880 (glycosyl hydrolase) and LOC_Os05g18604 (serine carboxypeptidase)] defining the 1.83 Mb *OsqGW5.1*^c^ QTL interval had high Δ (SNP-index) values of 0.9 based on our QTL-seq analysis in rice (**Figure 3D**). The known binding sites of RAV1AAT (CAACA to CAGCA) and CARGCW8GAT (CATAATTATG TO TATAATTATG) *cis*-regulatory elements of these two rice genes were affected by presence of grain weight-associated regulatory SNPs (A/G and C/T) through substitutions of nucleotides from A to G and C to T, respectively. Henceforth, grain weight-associated two regulatory SNPs-containing genes (glycosyl hydrolase and serine carboxypeptidase) localized at a major GW QTL interval (*OsqGW5.1*^c^) were selected as the potential candidates to decipher their significance in grain weight regulation through differential expression profiling (**Figure 3D**).

### Differential Expression Profiling to Identify Genes Regulating Rice Grain Weight

**FIGURE 4:**
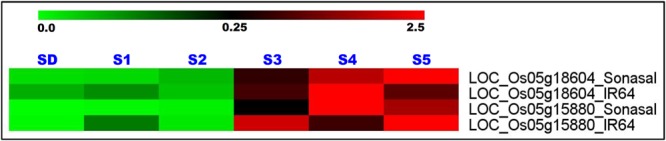
**Differential expression profiles of two genes (glycosyl hydrolase and serine carboxypeptidase) delineated at a novel 1.83 Mb major genomic region underlying the grain weight QTL (*OsqGW5.1*^c^) (detected by integrating traditional QTL mapping with QTL region-specific SSR and SNP marker-based QTL-seq analysis) in diverse vegetative seedlings (SD) and reproductive five seed developmental stages (S1–S5) of low and high grain weight parental accessions of a mapping population (IR 64 × Sonasal).** The average log signal expression value of genes in various tissues and developmental stages is represented at the top with a color scale; in which green, black and red color specify the low, medium and high level of expression, respectively. The tissues/stages and genes/mapping parental accessions utilized for expression profiling are designated on the top and right sides of expression map, respectively.

Two grain weight-associated regulatory SNPs-containing glycosyl hydrolase and serine carboxypeptidase genes delineated at a 1.83 Mb major genomic region underlying a novel *OsqGW5.1*^c^ QTL (validated by traditional QTL mapping and QTL region-specific QTL-seq analysis) were selected for differential expression profiling. The expression profiling of these two genes using RNA isolated from the vegetative (seedlings) and five seed developmental stages of low and high grain weight parental rice accessions (IR 64 and Sonasal) of a mapping population (IR 64 × Sonasal) was performed by quantitative RT-PCR assay. These genes exhibited seed-specific expression in two low and high grain weight mapping parental rice accessions as compared to their respective vegetative seedling tissues (**Figure 4**). Both these genes revealed pronounced differential upregulation specifically in later three seed developmental stages (S3–S5) than that of initial two developmental stages (S1 and S2) of both mapping parental accessions (**Figure 4**). Interestingly, a variable differential regulation of two genes especially in three seed developmental stages (S3–S5) of high (IR 64) and low (Sonasal) grain weight parental accessions was evident. In high grain weight accession IR 64, gene coding for glycosyl hydrolase was relatively more upregulated in S3 and S5 developmental stages as compared to S4. Overall, the levels were also higher in low grain weight accession Sonasal (**Figure 4**). The gene coding for serine carboxypeptidase was upregulated at increasing levels as seed development progressed in low grain weight accession Sonasal. On the other hand, in high grain weight accession IR 64, the upregulation pattern of this gene formed a bell-shaped curve from S3 to S5, being highest in S4 (**Figure 4**).

## Discussion

A diverse array of previous marker development landmark studies in rice have developed numerous high-density SSR markers at a genome-wide scale. The development of these makers are mostly relied either on whole genome sequence information of single *japonica* (Nipponbare) accession or genome sequences of two different *indica* (93-11) and *japonica* (Nipponbare) rice accessions. Although huge in number, these SSR markers exhibited limited polymorphic potential, especially when dealing with closely related accessions belonging to an individual rice population groups (*indica, japonica, aus*, and wild), thereby posing a huge challenge in obtaining the optimum number of genome-wide polymorphic markers for efficient discrimination of these accessions. This essentially makes difficult for user to select a smaller set of genome-wide polymorphic informative markers from these large number of available SSR marker resource and thus limits their utilization in large-scale genotyping applications of rice. With a prime objective to eliminate aforementioned bottleneck associated with previously developed high-density genome-wide SSR markers, the present study has utilized freely accessible whole genome pseudomolecules and genome resequence information of 11 rice accessions belonging to four different populations (*indica, japonica, aus*, and wild rice) for developing 6244 genome-wide *in silico* polymorphic SSR markers based on fragment length polymorphism/variation of their repeat-units at a genome-wide scale in rice. The SSR markers derived from diverse coding and non-coding sequence components of yield and a/biotic stress tolerance-related known/candidate genes/TFs revealed frameshift mutations along with expansion/contraction of amino acid sequences in the proteins encoded by these genes. This suggests functional significance of large-scale polymorphic coding SSR markers developed from the genes at a genome-wide scale in regulating diverse agronomic traits of rice.

To determine the novelty of 6244 *in silico* polymorphic SSR markers developed in the current study, these markers were compared based on their correspondence (physical positions and SSR repeat-motif types) with earlier available high-density genome-wide SSR markers in rice. Interestingly, about 32% of our developed markers were found redundant with the genome-wide RM markers that are most commonly utilized till date in various large-scale genotyping applications of rice (Temnykh et al., 2001; McCouch et al., 2002; International Rice Genome Sequencing Project (IRGSP), 2005). Despite commonality of these markers between past and present study, our current investigations were able to provide additional vital information regarding *in silico* polymorphic potential of the SSR (RM) markers for discrimination of accessions belonging to four different rice population groups (*indica, aus, japonica*, and wild) based on their repeat-unit variations. Therefore, a smaller set of *in silico* polymorphic RM markers including supplemental new polymorphic SSR markers screened by us at a genome-wide scale could be of immense use in various genomics-assisted breeding applications of rice. The genome-wide as well as known cloned/candidate gene-derived SSR markers developed in our study detected a higher degree of polymorphism especially among accessions representing individual *indica, aus, japonica*, and wild population groups. This implicates immense utility of genome/gene-based SSR markers in various high-throughput genetic analyses including association/genetic mapping (fine mapping/map-based isolation) of trait-associated potential genes/QTLs to expedite genomics-assisted crop improvement in rice.

The polymorphic potential detected by the SSR markers (88%) particularly among *indica* rice accessions is much higher than that estimated earlier with genome-wide random RM (rice microsatellite) (18–24%), GNMS (genic non-coding microsatellite) (∼32%) and highly variable SSR (42–54%) markers (Nagaraju et al., 2002; Singh et al., 2004, 2010; Garris et al., 2005; Caicedo et al., 2007; Sang and Ge, 2007; Parida et al., 2009). Notably, *in silico* polymorphic SSR markers designed in our study exhibited almost a comparable level of polymorphic potential among a diverse panel of *indica, aus, japonica*, and wild accessions with that documented earlier using polymorphic SSR markers, developed by comparing the genome sequences between *japonica* (Nipponbare) and *indica* (93-11) rice (Grover et al., 2007; Zhang et al., 2007). The efficient resolution as well as higher average amplification success rate (97%) and polymorphic potential (94%) of SSR markers among cultivated rice accessions and correspondence between *in silico* and experimental fragment length polymorphism detected by SSR markers in gel- and amplicon sequencing-based assay were apparent. These overall infer the broader practical applicability of these markers for large-scale genotyping applications, which can be complemented with user-preference selection of SSR markers according to their predetermined *in silico* frgment length polymorphism (based on repeat-unit variation) by optimal expense of resources in rice. Henceforth, the SSR markers with their simplicity in mining/development *in silico* including robustness in large-scale genotyping and detecting natural/functional allelic variation in the gene sequence components of diverse accessions could serve as a preferred marker resource at a genome-wide scale for their immense use in genomics-assisted breeding applications of rice in laboratories equipped with minimal infrastructural facilities.

The level of molecular diversity estimated specifically among cultivated rice accessions using *in silico* polymorphic genome/gene-derived SSR markers is almost comparable with that documented previously using various sequence-based random SSR markers (Nagaraju et al., 2002; Singh et al., 2004, 2010; Garris et al., 2005; Parida et al., 2009). The observed evolutionary relationship among accessions belonging to *indica, aus, japonica*, and wild rice population groups corresponds well with their previously known species/sub-species-specific origin, pedigree relationship and parentage (Nagaraju et al., 2002; Singh et al., 2004; Garris et al., 2005; Caicedo et al., 2007; Sang and Ge, 2007; Parida et al., 2009; He et al., 2011; Huang et al., 2012; Gross and Zhao, 2014). Notably, the clustering of short-grain aromatic mapping parental rice accession Sonasal with accessions representing an upland (*aus*) *indica* population group is agreed well with the known domestication events and close phylogenetic relationships as documented recently among aromatic, *aus* and *japonica* rice (Civáň et al., 2015). An unique pattern of phylogenetic relationship obtained among accessions representing diverse species/sub-species and population groups is possibly due to combined influence of adaptive selection pressure and complex breeding efforts during domestication of studied accessions from wild progenitors (Garris et al., 2005; Caicedo et al., 2007; Sang and Ge, 2007; He et al., 2011; Huang et al., 2012; Gross and Zhao, 2014). Collectively, a higher potential of *in silico* polymorphic SSR markers for assaying realistic estimation of functional molecular diversity and phylogenetics at a genome/gene level was apparent. This infers significance of these developed markers in establishing distinctness, uniformity and stability (DUS) especially among cultivated rice accessions and thus could be deployed in marker-assisted varietal improvement of rice. Interestingly, these markers are useful in assaying functional allelic varation/diversity at a genome-wide scale based on expansion/contraction of amino acid sequences in the proteins encoded by the genes and thus have potential to be directly correlated with phenotypic trait variation through genetic/association mapping in rice. In this context, informative known/candidate gene-based SSR markers developed could be employed efficiently in selection of trait-associated molecular tags at a whole genome level and desirable cultivar types for genetic improvement of rice.

A 3791 SSR marker-based high-resolution (an average inter-marker distance of 0.543 cM) genetic linkage map (IR 64 × Sonasal) constructed in the present study is highly saturated than that reported yet in diverse *indica* mapping population-led genetic maps of rice (Amarawathi et al., 2008; Zhao and Wu, 2008; Channamallikarjuna et al., 2010; Ngangkham et al., 2010; Meenakshisundaram et al., 2011; Vikram et al., 2011; Yu et al., 2011; Anuradha et al., 2012; Guleria et al., 2012; Marathi et al., 2012; Shanmugavadivel et al., 2013). Therefore, the constructed high-density genetic linkage map has potential to accelerate molecular mapping of high-resolution QTLs/genes controlling diverse agronomic traits including grain size/weight in rice. To acertain the validity and robustness of identified grain weight QTLs, the major genomic regions harboring six grain weight QTLs were compared with that reported by earlier QTL mapping studies utilizing multiple *indica* and aromatic rice-derived mapping populations. None of the grain weight QTLs identified by us showed correspondence with previously documented grain size/weight-associated known cloned and functionally characterized QTLs/genes based on their congruent physical positions on rice chromosomes. This infers that six grain weight QTLs identified by us are novel and possibly exhibit population-specific genomic distribution. The SSR markers-containing genes tightly linked with the major grain weight QTLs mapped on chromosomes could have functional signficance to be deployed in marker-assisted breeding for selecting accessions with higher grain weight and yield in rice.

**FIGURE 5:**
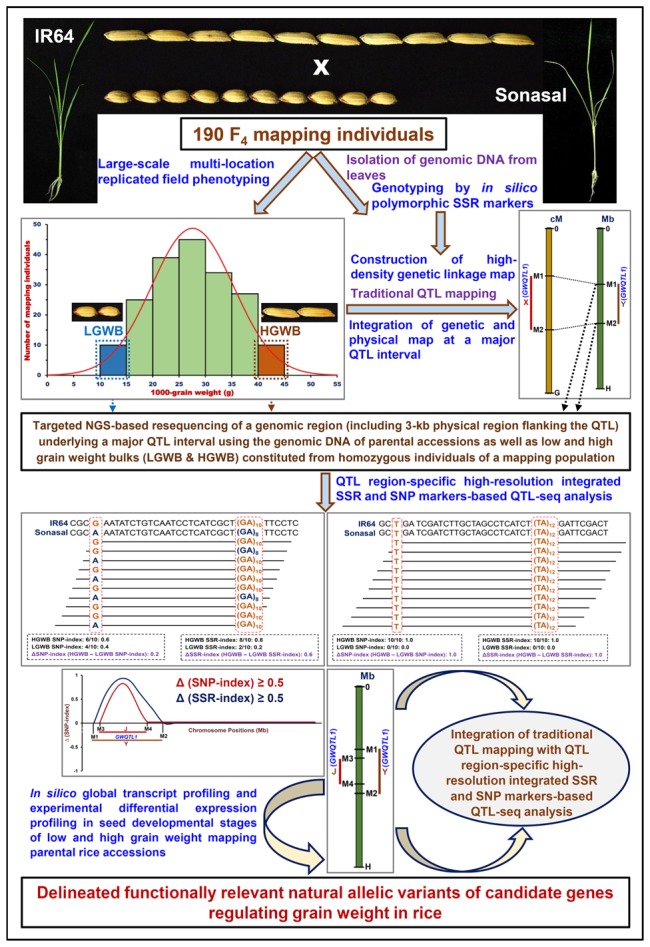
**Schematic illustrating the major steps followed to integrate traditional QTL mapping (genotyping by *in silico* polymorphic SSR markers) and QTL region-specific high-resolution integrated SSR and SNP marker-based QTL-seq analysis in a 190 *F*_4_ mapping population (IR 64 × Sonasal) with differential expression profiling (during seed developmental stages of contrasting low and high grain weight mapping parental accessions) for delineating the functionally relevant natural allelic variants of candidate genes underlying a major QTL associated with grain weight in rice.** LGWB, low grain weight bulk; HGWB, high grain weight bulk.

The integration of traditional QTL mapping (using *in silico* polymorphic SSR markers) with QTL region-specific high-resolution integrated SSR and SNP marker-based QTL-seq analysis (IR 64 × Sonasal) and differential expression profiling (during seed development in contrasting low and high grain weight rice accessions) are found proficient enough to scale-down a 1.83 Mb novel major grain weight QTL (*OsqGW5.1*^c^) region into two functionally relevant regulatory SNP allelic variants (A/G and C/T) in RAV1AAT and CARGCW8GAT *cis*-regulatory elements of two glycosyl hydrolase and serine carboxypeptidase genes governing rice grain weight. The implication of SNP-based QTL-seq led combinatorial approach to delineate the potential candidate genes and alleles at a major QTL region governing diverse agronomic traits is well-demonstrated in multiple crop plants (Saxena et al., 2014; Das et al., 2015, 2016; Kujur et al., 2015). As compared to SNP-based QTL-seq approach, the integrated SSR- and SNP-based QTL-seq strategy utilized in this study appears much efficient and informative enough to narrow-down the specific QTL region rapidly into potential candidate genes and natural alleles regulating complex grain weight quantitative trait in rice. This strategy will be certainly useful for fine-mapping the major QTLs identified by traditional SSR marker-based QTL mapping that are routinely used in molecular breeding laboratories to identify/map the QTLs governing complex agronomic traits in rice. Henceforth, this integrated QTL-seq approach (as illustrated in **Figure 5**) has wider practical applicability for dissection of QTLs regulating various complex quantitative traits not only in chickpea but also in mutiple crop plants.

Multiple evidences on map-based cloning and functional genomic analyses suggest the involvement of serine carboxypeptidase (*SCP*)-encoding known cloned and functionally characterized QTL/gene, namely *GS5*, in controlling positive regulation of grain size/weight in rice (Li et al., 2011; Zuo and Li, 2014). The pronounced expression of *SCP*-encoding *GS5* gene in endosperm of rice seeds for increased grain weight is coherent with that of expression profiles displayed by grain weight-associated *SCP* gene (identified in our study) at later seed maturation developmental stages (S3–S5) of low (Sonasal) and high (IR 64) grain weight rice accessions (Li et al., 2011). In addition to rice, *SCP* genes are known to play crucial role in determining seed size, including seed weight and seed number in *Arabidopsis* (Li et al., 2001; Wu et al., 2008). Most of these *SCP* genes are involved in seed size determination by modulating brassinosteroid (BR) signaling pathways in rice and *Arabidopsis* (Li et al., 2001; Wu et al., 2008; Jiang et al., 2013; Gao et al., 2015). A potential role of *SCP* gene in grain size/weight regulation is clearely evident from its pronounced up-regulation (in global microarray profiling) commonly in the NILs (near isogenic lines) developed by introgressing functional alleles of two major known grain length-regulating QTLs/genes, *GS3* and *qGL3* in rice (Gao et al., 2015). Another grain weight-associated regulatory SNP-containing glycosyl hydrolase (*GH*) candidate gene identified in our study through integrated genomic strategy is known to be involved in structural remodeling of cell wall in plant, which is important determinant of cell size by selective degradation specific polysaccharide constituents (Minic et al., 2006). An endosperm-specific *GH* gene has been reported to influence seed size in *Arabidopsis* (Minic et al., 2006). Interestingly, the strong grain weight-associated SNP allelic variants-containing RAV1AAT and CARGCW8GAT known *cis*-regulatory elements/TF-binding sites of two genes identified are known to be involved in growth and development by transcriptional regulation/interaction and preferential/tissue-specific expression of many important TF genes in seeds of rice (Zheng et al., 2009; Bajaj et al., 2015b). The direct interactions of the RAV1 (related to *ABI3/VP1*) and CArG TF DNA-binding protein with other TF genes including MADS-domain protein-encoding genes, *SEPALLATA3* and AGAMOUS-like 15 (*AGL15*), respectively, have been established to be the key regulators of seed development in *Arabidopsis* (Tang and Perry, 2003; Le et al., 2010; Agarwal et al., 2011; Zhang et al., 2015). Therefore, it would be interesting to study transcriptional regulation and binding/interactions of two *SCP* and *GH* genes at known *cis*-regulatory element regions/TF-binding sites with MADS TF genes in controlling grain weight/size of rice. However, the detail molecular characterization and functional validation of these genes are required to decipher their complex regulatory pathways underlying seed development and grain size/weight determination in rice. The novel functionally relevant grain size/weight-associated genes and natural allelic variants delineated by integrated traditional QTL mapping and QTL-seq strategy could be deployed for genomics-assisted crop improvement to develop genetically tailored varieties with higher grain weight and yield in rice.

Collectively, a novel combinatorial strategy by integrating traditional QTL mapping with high-resolution QTL region-specific integrated SSR and SNP markers-based QTL-seq analysis and expression profiling was deployed in the present study to scale-down one strong major grain weight QTL region into *cis*-regulatory natural allelic variants-containing two seed-specific glycosyl hydrolase and serine carboxypeptidase genes differentially expressed during seed development in low and high seed weight mapping parental accessions. As compared to a SNP-based conventional QTL-seq approach, the QTL region-specific integrated SSR- and SNP marker-based QTL-seq strategy developed in our study has multiple significance in terms of its adopted simpler and cost-efficient methods of detection and realistic practical applications in identification/revalidation and fine-mapping of major QTLs as well as genetic dissection of complex quantitative traits (grain weight in this study) in rice (Supplementary Table S4; **Figure 5**). Primarily, this strategy requires information on strong grain weight-associated known QTL interval identified priorly based on traditional SSR markers-led QTL mapping. Consequently, this involves high genome coverage targeted NGS resequencing of a predetermined known grain weight QTL interval in the parental accessions and preferred bulks constituted from low and high grain weight homozygous individuals (with varied genetic constitution types) of a mapping population, selected by using both phenotyping and genotyping information of genome-wide and QTL-linked SSR markers (identified by traditional SSR marker-based QTL mapping) (**Figure 5**). As compared to a conventional SNP-based QTL-seq approach, our developed QTL-seq strategy thus complemented and enhanced the overall QTL detection efficiency by integrating high-resolution genotyping information of both SSR and SNP markers at a targeted resequenced known grain weight QTL region. Therefore, this strategy expedited the identification including revalidation of non-erroneous known grain weight QTLs (detected by traditional SSR marker-based QTL mapping) as well as delineation of a specific QTL region into potential candidate genes/alleles regulating grain weight in rice (**Figure 5**). In these perspectives, the high-resolution QTL region-specific QTL-seq approach has multiple added-advantages as compared to a traditional QTL-seq strategy especially in fine-mapping the major QTLs that are routinely detected in rice molecular breeding laboratories employing traditional SSR marker-based QTL mapping.

Summarily, our study demonstrated the added advantages of developing *in silico* polymorphic genome-wide SSR markers as compared to other sequence-based markers and their broader practical applicability in acelerating genomics-assisted breeding applications of rice by limited resource expenses. Primarily, this provides user an oppurnunity to select desirable combination of informative SSR markers at a whole genome/gene level as well as suitable marker genotyping assay for precise resolution/estimation of varied polymorphic amplicon fragment sizes based on predetermined marker *in silico* fragment length polymorphism (repeat-unit variation) among accessions belonging to individual rice populations (*indica, aus, japonica*, and wild) to drive large-scale genotyping applications in rice. The efficacy of these SSR markers was ascribed to simplicity in their discovery and greater potential of detecting amplification (97%) and polymorphism (88%) as well as assaying of a wider natural (functional) allelic diversity (16–74%) among accessions especially belonging to *indica* rice population group. Further, the utility of these *in silico* polymorphic SSR markers in constructing a high-density genetic linkage map and molecular mapping of major grain weight QTLs (31% combined PVE) was demonstrated successfully in rice. Considering the above facts, the development of *in silico* polymorphic SSR markers by utilizing/comparing the available whole genome and/genome resequence information of rice accessions seems quite simpler, quicker and cost-efficient. Moreover, these *in silico* polymorphic SSR markers are much more informative than that of other available high-density random genome-wide SSR markers, thereby suitable for various high-throughput genetic analyses in rice. In these perspectives, the designing of such markers seems quite relevant in rice due to availability of genome resequence data for thousands of accessions (for instance, 3000 rice accessions; Alexandrov et al., 2015) in the public domain. Henceforth, the development of *in silico* polymorphic SSR markers by using/comparing freely accessible rather than own generated/sequenced genome resequence information of multiple rice accessions is much more economical and expedient especially in providing informativeness of markers within/between diverse population groups. Collectively, the *in silico* polymorphic genome-wide SSR markers have multiple auxiliary desirable genetic attributes as compared to other sequence-based markers (especially random genome-wide SSR markers), which makes them an attractive alternative to other available markers for accelerating genomics-assisted breeding applications in rice. Beside *in silico* polymorphic genome-wide SSR markers, a high-resolution QTL region-specific integrated SSR- and SNP marker-based QTL-seq strategy developed by us have immense utility in various genomics-assisted breeding applications including delineation of functionally relevant potential molecular tags (markers, QTLs/genes and alleles) at conventional QTL mapping-derived major QTLs regulating vital agronomic traits and marker-assisted genetic enhancement of rice in laboratories equipped with limited infrastructural facilities.

## Author Contributions

AD conducted all experiments and drafted the manuscript. SD involved in gene expression profiling and assisted in manuscript writing. RS helped in computational genomics-related analysis. SB and AS helped in generation of mapping population and their phenotyping. PA, SP, and AT conceived and designed the study, guided data analysis and interpretation, participated in drafting and correcting the manuscript critically and gave the final approval of the version to be published. All authors have read and approved the final manuscript.

## Conflict of Interest Statement

The authors declare that the research was conducted in the absence of any commercial or financial relationships that could be construed as a potential conflict of interest.

## Footnotes

1 http://www.ncbi.nlm.nih.gov/sra/?term=oryza+sativa

2 http://rice.plantbiology.msu.edu/annotation_pseudo_current.shtml

3 http://www.ddbj.nig.ac.jp

4 http://www.ncbi.nlm.nih.gov/sra/?term

5 http://pgrc.ipk-gatersleben.de/misa/

6 http://pgrc.ipk-gatersleben.de/misa/primer3.html

7 http://www.kyazma.nl/index.php/mc.JoinMap

8 http://rice.plantbiology.msu.edu

9 http://mev.tm4.org

10 http://ricencode.github.io/
