# Supplementary material for: An Efficient Strategy Combining SSR Markers- and Advanced QTL-seq-driven QTL Mapping Unravels Candidate Genes Regulating Grain Weight in Rice
Source: Front Plant Sci. 2016 Oct 26;7:1535. doi: 10.3389/fpls.2016.01535 (PMC5080349; doi:10.3389/fpls.2016.01535)
Supplement: Supplementary file 1 [file Data_Sheet_1.PDF]

An efficient strategy combining SSR markers- and advanced QTL-seq-driven QTL mapping unravels candidate genes regulating grain weight in rice

Anurag Daware[1], Sweta Das[1], Rishi Srivastava[1], Saurabh Badoni[1], Ashok K. Singh[2], Pinky Agarwal[1], Swarup K. Parida[1*], Akhilesh K. Tyagi[1a*]

[1]National Institute of Plant Genome Research (NIPGR), Aruna Asaf Ali Marg, New Delhi 110067, India

[2]Division of Genetics, Rice Section, Indian Agricultural Research Institute (IARI), New Delhi 110012, India

*Corresponding authors

Present Address

[a]Department of Plant Molecular Biology, University of Delhi South Campus, New Delhi 110021, India

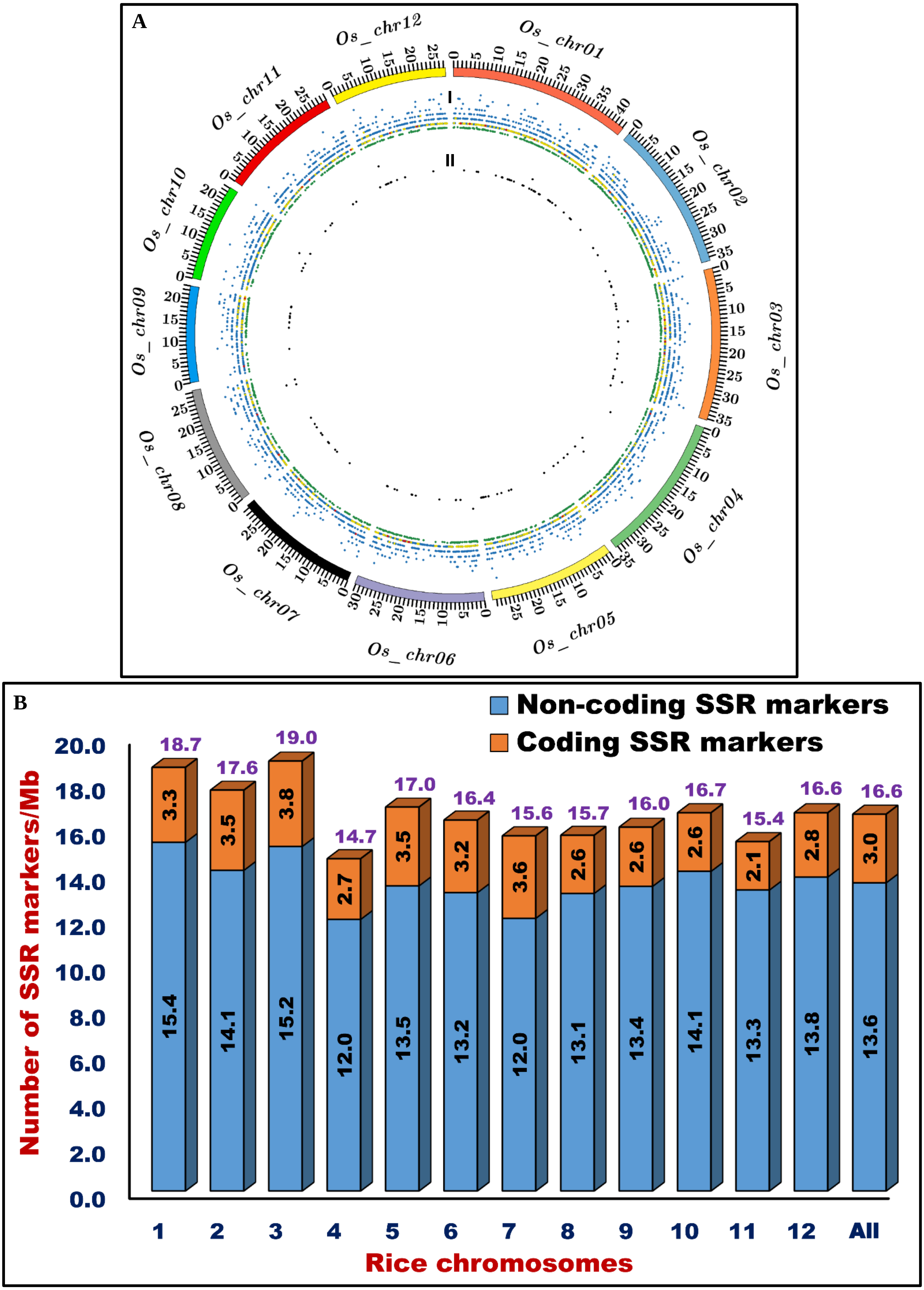

Figure S1. (A) A circos diagram illustrating the relative distribution pattern of 6244 *in silico* polymorphic SSR markers physically mapped on 12 rice chromosomes. The outermost circle represents twelve rice chromosomes coded with different colors. **(I)** Polymorphic SSR markers derived from the introns/intergenic sequences (blue dots), URRs (yellow dots), CDS (green dots) and causing frameshift mutations (red dots). **(II)** Polymorphic SSR markers developed from the known cloned genes characterized for diverse agronomic traits (stress tolerance and yield/quality component traits). **(B)** Relative density (number of SSR markers/Mb) of non-coding and coding SSR markers physically mapped on each of the 12 and all rice chromosomes. The digits mentioned above the bars represent the proportion of total coding and non-coding SSR markers mapped on the individual and all 12 rice chromosomes.

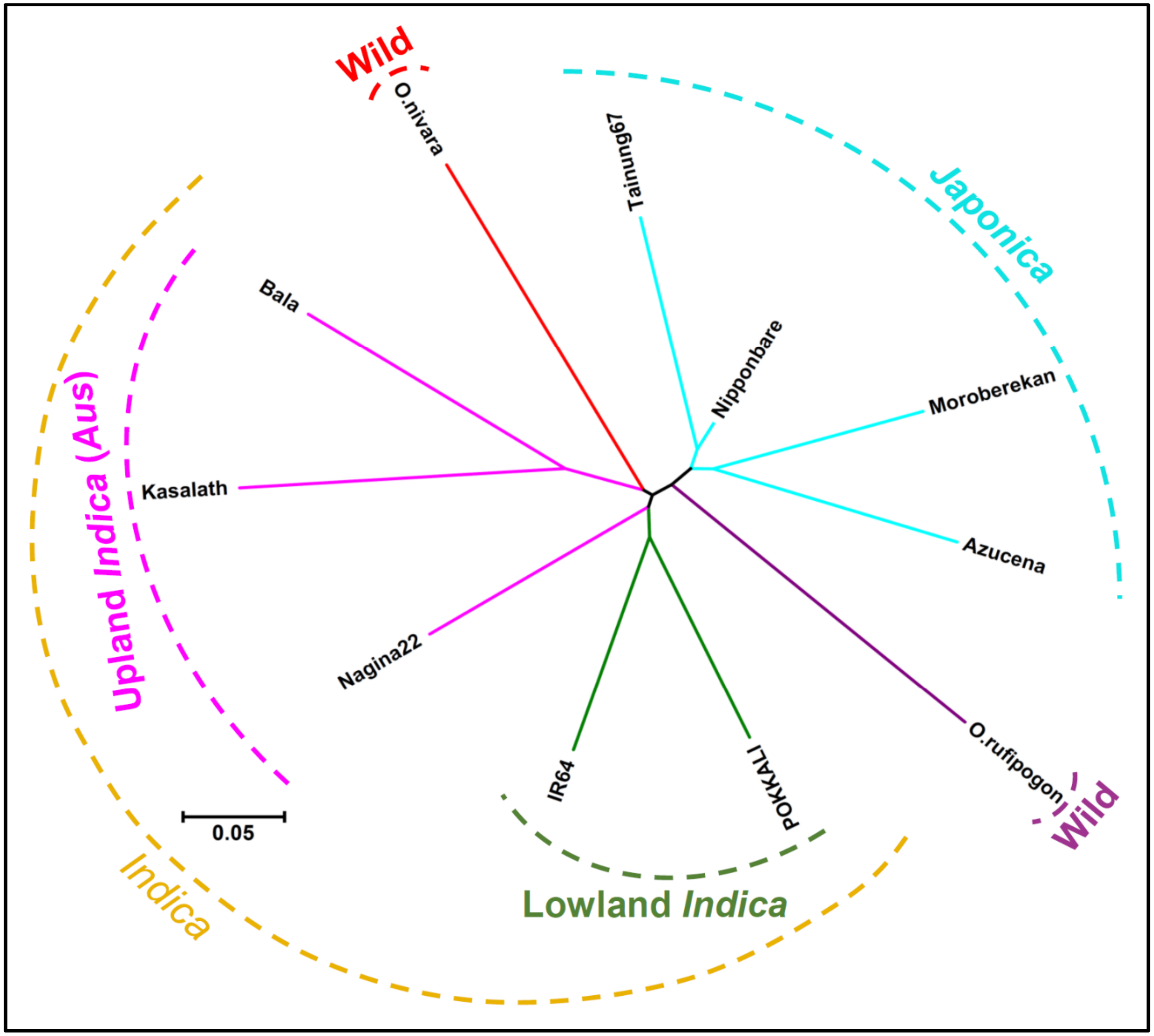

Figure S2. Unrooted phylogenetic tree illustrating the genetic diversity and evolutionary relationships among 11 rice accessions using 6244 genome-wide *in silico* polymorphic SSR markers (physically mapped on 12 chromosomes). All these accessions differentiated into three major groups- *indica* (lowland and upland/*aus*), *japonica* and wild based on their known species/subspecies-specific origin, pedigree relationship and parentage.

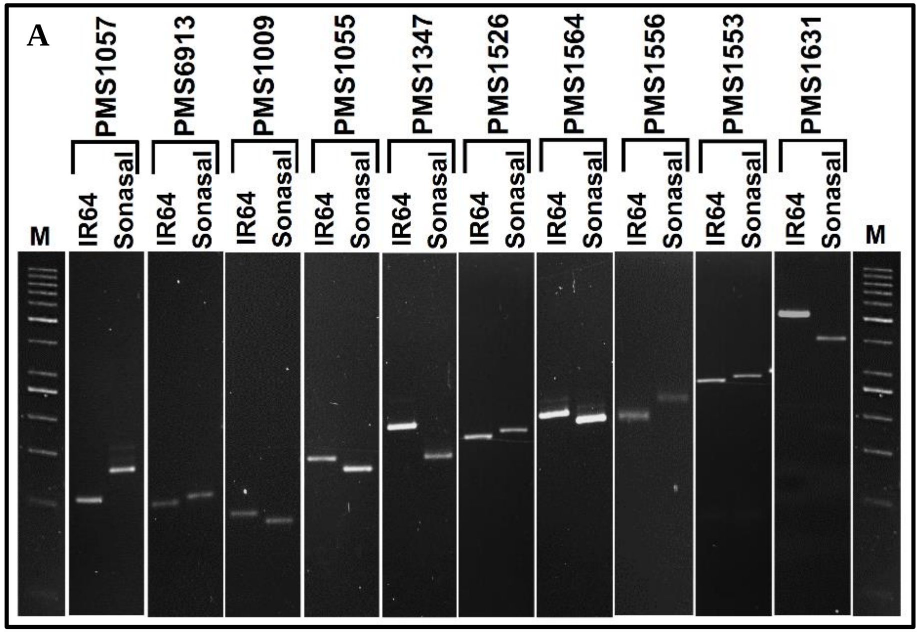

A
PMS1057
PMS6913
PMS1009
PMS1055
PMS1347
PMS1526
PMS1564
PMS1556
PMS1553
PMS1631
M
IR64
Sonasal
M

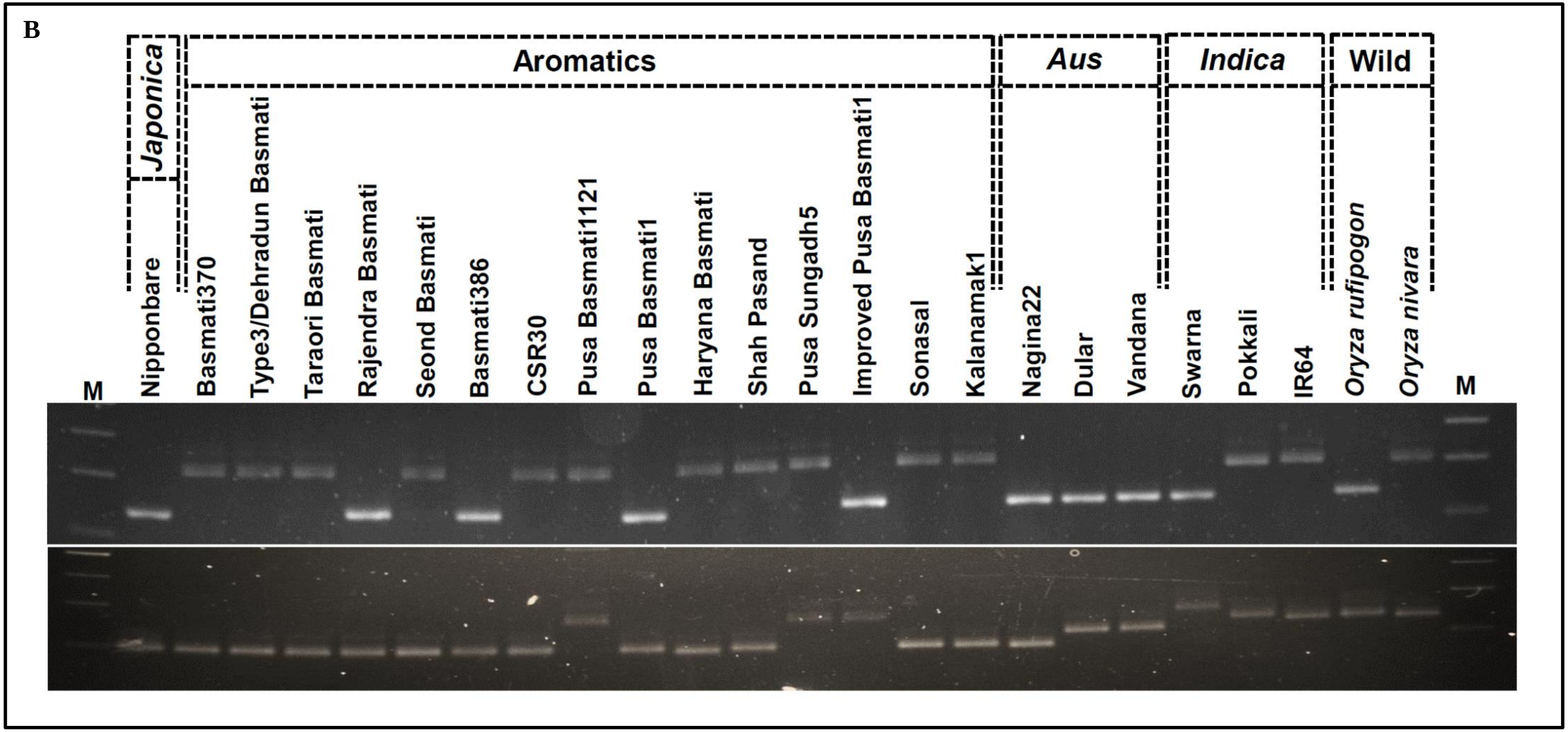

Figure S3. (A) Validation of a representative set of 10 polymorphic SSR markers (physically mapped on 12 rice chromosomes) exhibiting *in silico* fragment length polymorphism between parental accessions (IR 64 and Sonasal) of a F_4 mapping population (IR 64 x Sonasal) using the gel-based assay. **(B)** Amplification and genotyping profiles of two representative SSR markers, PMS1055 and PMS435 in a selected set of 24 *indica*, *aus*, aromatics, *japonica* and wild rice accessions produced two and three polymorphic alleles, respectively among these accessions. The detail information of SSR markers are mentioned in the Table S2. M: 50 bp DNA ladder size standard.

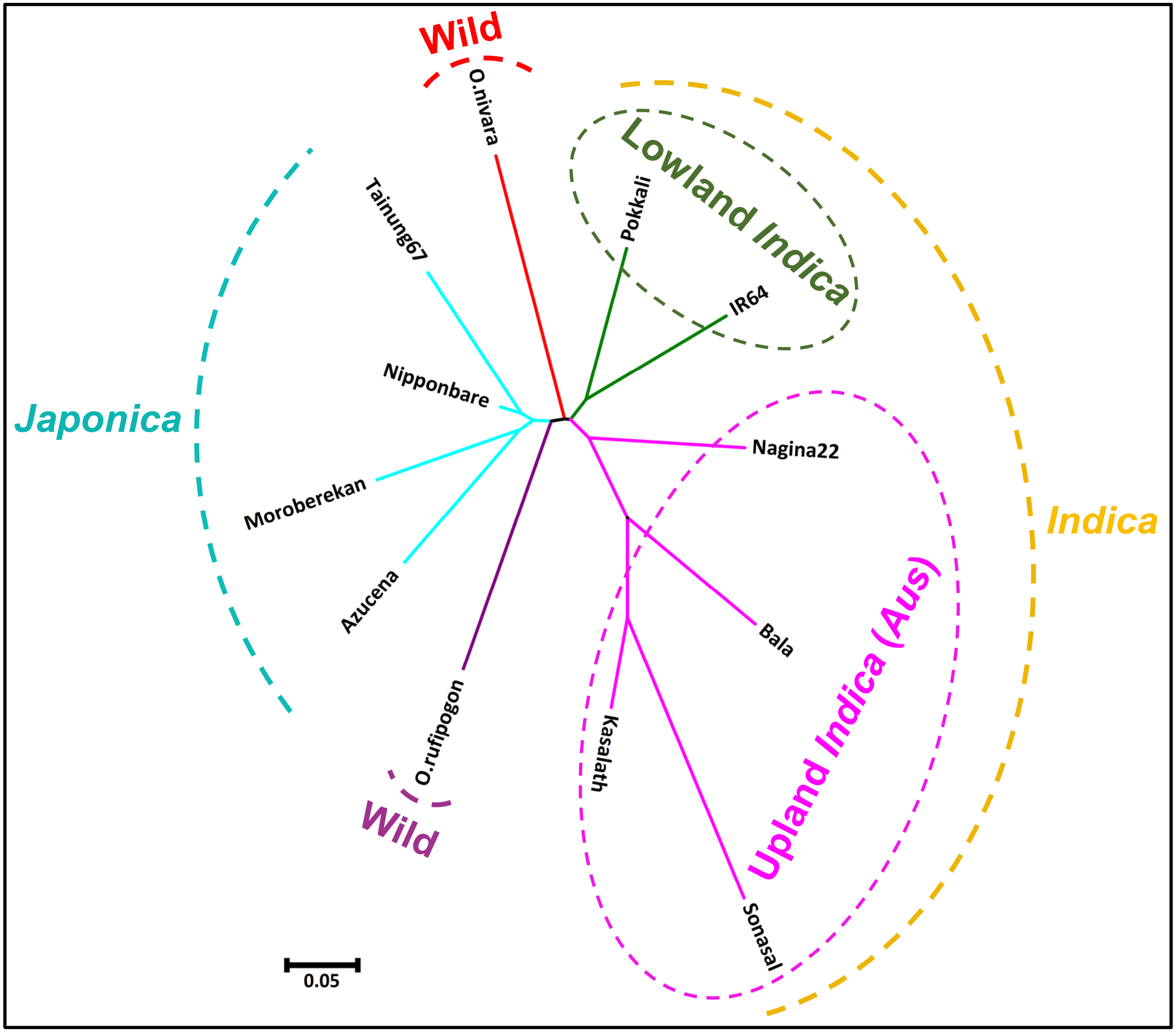

Figure S4. Unrooted phylogenetic tree illustrating the genetic diversity and evolutionary relationships among 12 rice accessions including one low grain weight mapping parental accession Sonasal using genome-wide *in silico* polymorphic and experimentally validated SSR markers (physically mapped on 12 chromosomes). All these accessions differentiated into three major groups- *indica* (lowland and upland/*aus*), *japonica* and wild based on their known species/subspecies-specific origin, pedigree relationship and parentage.

Table S1. Details of genomic sequences of 11 rice accessions used for genome-wide development of *in silico* polymorphic SSR markers

S.N.	Accessions	Database/Accession	Characteristics of sequence resources	Total sequence size (Mb)	Population type
1	Kasalath	DRA000968 and DRA001099 (DDBJ)	Pseudomolecule	330.55	*Aus*
2	Nagina22	SRX272397	Sequence Read Archive (SRA)	7103.00	*Aus*
3	IR64	SRX272395	Sequence Read Archive (SRA)	7243.10	*Indica*
4	Pokkali	SRX272396	Sequence Read Archive (SRA)	7202.31	*Indica*
5	Bala	SRX128324	Sequence Read Archive (SRA)	16799.77	*Indica*
6	Nipponbare	MSU Rice Genome Annotation Project Release 7.0	Pseudomolecule	373.2	Temperate *Japonica*
7	Tainung67	SRX665341	Sequence Read Archive (SRA)	6317.83	Temperate *Japonica*
8	Azucena	SRX128323	Sequence Read Archive (SRA)	9417.93	Tropical *Japonica*
9	Moroberekan	DRX002964	Sequence Read Archive (SRA)	10966.2	Tropical *Japonica*
10	*Oryza rufipogon*	SRX367228	Sequence Read Archive (SRA)	8559.89	Wild
11	*Oryza nivara*	SRX025244	Sequence Read Archive (SRA)	3134.08	Wild

Table S2. Characteristics and annotation of 6244 *in silico* polymorphic SSR markers developed from the rice genome

Polymorphic SSR (PMS) markers	Chromosomes	Start physical positions (bp)	End physical positions (bp)	Nipponbare	Kasalath	Nagina22	IR64	Pokkali	Bala	Tainung67	Azucena	Moroberekan	*Oryza rufipogon*	*Oryza nivara*	MSU gene locus IDs	Structural annotation	Functional annotation	SSR functional effects	Forward primers (5'-3')	Reverse primers (5'-3')	Amplified product size (bp)	Markers exhibiting polymorphism within *indica* rice	Transcrption factor genes-derived markers	Known cloned genes-derived markers	PMS markers corresponding with RM markers
PMS1	Os_Chr01	73110	73127	(GAC)6	(GAC)6	(GAC)6	(GAC)6	(GAC)6	(GAC)6	(GAC)5	(GAC)6	(GAC)6	(GAC)6	(GAC)6	LOC_Os01g01150	CDS	RNA recognition motif, putative, expressed	CODON_DELETION	GAGGAGGCCAAGCTCAAGTC	GACTTGGAGGGGTCGACTG	230	A	A	A	NA
PMS2	Os_Chr01	148166	148183	(TCC)6	(TCC)6	(TCC)6	(TCC)6	(TCC)6	(TCC)6	(TCC)5	(TCC)6	(TCC)6	(TCC)5	(TCC)6	LOC_Os01g01307	CDS	translocon-associated protein beta domain containing protein, expressed	CODON_DELETION	AGAGGAAGGGCATCCAGAAT	AGAGATCGGAGGAGGAGGAG	266	A	A	A	NA
PMS3	Os_Chr01	173088	173111	(GCG)8	(GCG)8	(GCG)6	(GCG)8	(GCG)8	(GCG)8	(GCG)8	(GCG)8	(GCG)8	(GCG)8	(GCG)8	LOC_Os01g01350	URR	SNF7 domain containing protein, putative, expressed	-	CGAGGAGAATACTCGTTCGGTAGC	CCCTTCTCCATCTCATCTCACTCC	172	P	A	A	RM6464
PMS4	Os_Chr01	209481	209504	(GAA)8	(GAA)7	(GAA)8	(GAA)8	(GAA)8	(GAA)7	(GAA)8	(GAA)8	(GAA)8	(GAA)8	(GAA)5	LOC_Os01g01410	CDS	TKL_IRAK_C-LEC.1 - TKL_IRAK_C-LEC has homology to C-type lectin receptors, expressed	CODON_DELETION	CGAGGAGGGAATACATACAGG	GTTCATCATCAAGGAAGGAAGG	162	P	A	A	RM10012
PMS5	Os_Chr01	218626	218643	(AT)9	(AT)8	(AT)9	(AT)9	(AT)9	(AT)9	(AT)9	(AT)9	(AT)9	(AT)9	(AT)9	-	Introns/Intergenic	-	-	AATAACTTGCGCGTGACAGA	TTTCACAAGCAAACGGTTAGG	198	P	A	A	NA
PMS6	Os_Chr01	227223	227240	(CGA)6	(CGA)6	(CGA)5	(CGA)6	(CGA)6	(CGA)6	(CGA)6	(CGA)6	(CGA)6	(CGA)6	(CGA)6	LOC_Os01g01450	CDS	stress responsive protein, putative, expressed	CODON_CHANGE_PLUS_CODON_DELETION	TAAATCTCCTCCATTCCCCC	GAGGAGGATGAGCGTAGTCG	229	P	A	A	NA
PMS7	Os_Chr01	386901	386922	(TA)11	(TA)11	(TA)11	(TA)11	(TA)11	(TA)8	(TA)11	(TA)11	(TA)11	(TA)11	(TA)11	-	Introns/Intergenic	-	-	GACTGATGGTCACTTGCAGTTCG	CTTTCCAAGCACAGCCTAAGTGG	249	P	A	A	RM1843
PMS8	Os_Chr01	389932	389975	(TA)22	(TA)22	(TA)22	(TA)6	(TA)9	(TA)22	(TA)22	(TA)22	(TA)6	(TA)22	(TA)7	-	Introns/Intergenic	-	-	CCGTACCAAACACCAACACTGC	CGGCCGATCATCTAATCTAATCTACC	111	P	A	A	RM4554,RM499
PMS9	Os_Chr01	480232	480249	(GTA)6	(GTA)6	(GTA)6	(GTA)6	(GTA)6	(GTA)6	(GTA)6	(GTA)6	(GTA)6	(GTA)6	(GTA)5	-	Introns/Intergenic	-	-	TTCAGGTCACAGCAGCAATC	TCCTGCACCATCCATCAGTA	279	A	A	A	NA
PMS10	Os_Chr01	514117	514130	(GA)7	(GA)6	(GA)7	(GA)7	(GA)7	(GA)7	(GA)7	(GA)7	(GA)7	(GA)7	(GA)7	LOC_Os01g01960	DRR	transcriptional repressor, putative, expressed	-	CAGAGGGGCCTATACCAACA	AAGCAAAGCATGAGTGAGCA	224	P	A	A	NA
PMS11	Os_Chr01	527836	527853	(GAA)6	(GAA)6	(GAA)6	(GAA)6	(GAA)6	(GAA)5	(GAA)6	(GAA)6	(GAA)6	(GAA)6	(GAA)6	LOC_Os01g01970	URR	expressed protein	-	ATCAACCAAGGTGCACAACA	TCAACCCTAAATTGCTTCCG	210	P	A	A	NA
PMS12	Os_Chr01	597938	597955	(AT)9	(AT)9	(AT)9	(AT)6	(AT)9	(AT)9	(AT)9	(AT)9	(AT)9	(AT)9	(AT)7	-	Introns/Intergenic	-	-	GCCTCATCTTTTGGCTTATGG	AGGACTCCTCCGTTTCGATT	227	P	A	A	NA

Polymorphic SSR (PMS) markers	Chromosomes	Start physical positions (bp)	End physical positions (bp)	Nipponbare	Kasalath	Nagina22	IR64	Pokkali	Bala	Tainung67	Azucena	Moroberekan	*Oryza rufipogon*	*Oryza nivara*	MSU gene locus IDs	Structural annotation	Functional annotation	SSR functional effects	Forward primers (5'-3')	Reverse primers (5'-3')	Amplified product size (bp)	Markers exhibiting polymorphism within *indica* rice	Transcrption factor genes-derived markers	Known cloned genes-derived markers	PMS markers corresponding with RM markers
PMS13	Os_Chr01	615585	615600	(TA)8	(TA)8	(TA)8	(TA)8	(TA)8	(TA)8	(TA)8	(TA)8	(TA)8	(TA)7	(TA)8	LOC_Os01g02120	DRR	osMFT2 MFT-Like2 homologous to Mother of FT and TFL1 gene; contains Pfam profile PF01161: Phosphatidylethanolamine-binding protein, expressed	-	ACCGTCGTGTGGTACGTTTT	GGCGTCAAACATCTAGGGAA	163	A	A	A	NA
PMS14	Os_Chr01	617230	617241	(TA)6	(TA)6	(TA)6	(TA)7	(TA)6	(TA)6	(TA)6	(TA)6	(TA)6	(TA)6	(TA)6	-	Introns/Intergenic	-	-	TTTGAAACGGATATGTAAACTTTTG	CCCTCCAACTGGTTTCCTTT	251	P	A	A	NA
PMS15	Os_Chr01	651848	651863	(AG)8	(AG)8	(AG)8	(AG)8	(AG)8	(AG)8	(AG)8	(AG)7	(AG)8	(AG)8	(AG)8	LOC_Os01g02200	URR	armadillo/beta-catenin repeat family protein, putative, expressed	-	AGAGTTGGTTGGTTCACCTCTCG	AGGAGAGGAGGAGATGGAGTCG	299	A	A	A	RM10044
PMS16	Os_Chr01	673162	673176	(ACG)5	(ACG)5	(ACG)5	(ACG)5	(ACG)5	(ACG)5	(ACG)5	(ACG)5	(ACG)6	(ACG)5	(ACG)5	-	Introns/Intergenic	-	-	GGCGAGGAGAGGAGAAAGAT	TCCTTCCTTGACCCAAACTG	235	A	A	A	NA
PMS17	Os_Chr01	749500	749514	(CGC)5	(CGC)6	(CGC)5	(CGC)5	(CGC)5	(CGC)6	(CGC)5	(CGC)5	(CGC)5	(CGC)5	(CGC)5	-	Introns/Intergenic	-	-	ATGATGCTGCACTGCAAGTC	GCCATTTGCATGCCTAAGAT	247	P	A	A	NA
PMS18	Os_Chr01	796633	796648	(CT)8	(CT)8	(CT)8	(CT)8	(CT)8	(CT)8	(CT)8	(CT)8	(CT)8	(CT)7	(CT)8	-	Introns/Intergenic	-	-	ACATCCTCAAACCGCAAAAC	CTGCCTACCCAAACGAGAAG	220	A	A	A	NA
PMS19	Os_Chr01	1007152	1007165	(AG)7	(AG)7	(AG)7	(AG)7	(AG)7	(AG)7	(AG)6	(AG)7	(AG)7	(AG)7	(AG)7	-	Introns/Intergenic	-	-	GACACCCTGAAGGAGACGAC	TGTGCACGTAGCAAAACCTC	191	A	A	A	NA
PMS20	Os_Chr01	1059540	1059563	(GAA)8	(GAA)8	(GAA)8	(GAA)8	(GAA)8	(GAA)7	(GAA)8	(GAA)8	(GAA)8	(GAA)8	(GAA)8	-	Introns/Intergenic	-	-	GCATGATGCAACGGAGATCG	CTTCCTCATCTCCCTCACCTTCC	156	P	A	A	RM6340
PMS21	Os_Chr01	1100942	1100985	(TA)22	(TA)22	(TA)22	(TA)22	(TA)22	(TA)10	(TA)22	(TA)22	(TA)22	(TA)22	(TA)16	-	Introns/Intergenic	-	-	ACTCTGTAATCACCCAGTGCAACG	ACGAGGGAGGAGGAGATGACTTAGG	210	P	A	A	RM10058
PMS22	Os_Chr01	1111704	1111719	(AC)8	(AC)8	(AC)8	(AC)8	(AC)8	(AC)8	(AC)8	(AC)8	(AC)9	(AC)8	(AC)8	-	Introns/Intergenic	-	-	AAAACTTGCCCTTATGCGTG	TTAATCCCGAACGAAAATCG	275	A	A	A	NA
PMS23	Os_Chr01	1213456	1213521	(ATT)22	(ATT)22	(ATT)6	(ATT)22	(ATT)22	(ATT)22	(ATT)9	(ATT)22	(ATT)22	(ATT)22	(ATT)22	-	Introns/Intergenic	-	-	GAGATGCGTGCAGTGACAAGG	CCAGAATAAATGCCATGCAGTCG	350	P	A	A	RM10060
PMS24	Os_Chr01	1245507	1245526	(TC)10	(TC)8	(TC)10	(TC)10	(TC)10	(TC)10	(TC)10	(TC)10	(TC)10	(TC)10	(TC)10	-	Introns/Intergenic	-	-	GTACTTGTTGTAGCGCCCGTTCC	GGAGAACGAGAAAGAGGGAGAGC	358	P	A	A	RM10062
PMS25	Os_Chr01	1245698	1245711	(CG)7	(CG)6	(CG)7	(CG)7	(CG)7	(CG)7	(CG)7	(CG)7	(CG)7	(CG)7	(CG)7	-	Introns/Intergenic	-	-	TTCTCGTTCTCCTGCTCTCC	GAGCTCTGCCTCCAATCCTT	249	P	A	A	NA

Polymorphic SSR (PMS) markers	Chromosomes	Start physical positions (bp)	End physical positions (bp)	Nipponbare	Kasalath	Nagina22	IR64	Pokkali	Bala	Tainung67	Azucena	Moroberekan	*Oryza rufipogon*	*Oryza nivara*	MSU gene locus IDs	Structural annotation	Functional annotation	SSR functional effects	Forward primers (5'-3')	Reverse primers (5'-3')	Amplified product size (bp)	Markers exhibiting polymorphism within *indica* rice	Transcrption factor genes-derived markers	Known cloned genes-derived markers	PMS markers corresponding with RM markers
PMS26	Os_Chr01	1254692	1254715	(GCG)8	(GCG)8	(GCG)8	(GCG)8	(GCG)8	(GCG)8	(GCG)8	(GCG)8	(GCG)8	(GCG)6	(GCG)8	LOC_Os01g03190	CDS	expressed protein	CODON_CHANGE_PLUS_CODON_DELETION	GCTCGGCTAGTGACGATTTC	GTGGTAGGCGACATAGCTCC	220	A	A	A	NA
PMS27	Os_Chr01	1265068	1265088	(CGG)7	(CGG)7	(CGG)7	(CGG)7	(CGG)7	(CGG)5	(CGG)7	(CGG)7	(CGG)7	(CGG)7	(CGG)7	LOC_Os01g03210	CDS	expressed protein	CODON_CHANGE_PLUS_CODON_DELETION	GCGGTACAGGTGCAAGATGTGG	CATGTCCACCATGACCATGTCC	100	P	A	A	RM10064
PMS28	Os_Chr01	1267380	1267405	(AT)13	(AT)13	(AT)13	(AT)13	(AT)13	(AT)13	(AT)13	(AT)13	(AT)6	(AT)13	(AT)13	-	Introns/Intergenic	-	-	GAGGCAACAGGGAGGGTTAATGG	GCCGCAGAGAGGAAGAGAAGG	138	A	A	A	RM10065
PMS29	Os_Chr01	1315022	1315043	(AT)11	(AT)11	(AT)11	(AT)11	(AT)11	(AT)11	(AT)11	(AT)11	(AT)11	(AT)11	(AT)8	-	Introns/Intergenic	-	-	CGAGGACATAACAACAACAACG	AGCCTAACTACACCGTAATCAGC	145	A	A	A	RM10073
PMS30	Os_Chr01	1347426	1347506	(TTA)27	(TTA)27	(TTA)6	(TTA)16	(TTA)27	(TTA)27	(TTA)27	(TTA)27	(TTA)14	(TTA)27	(TTA)9	-	Introns/Intergenic	-	-	GCCCATGCTCAATGAGATATT	TGCACCCATGTTGAGTTGTC	272	P	A	A	NA
PMS31	Os_Chr01	1420398	1420413	(TA)8	(TA)8	(TA)8	(TA)8	(TA)8	(TA)8	(TA)8	(TA)8	(TA)8	(TA)8	(TA)7	-	Introns/Intergenic	-	-	ACGAGGAAAACGAGAGCGTA	CAGACCAATTTTCAGTGGCA	185	A	A	A	NA
PMS32	Os_Chr01	1481440	1481455	(GA)8	(GA)7	(GA)8	(GA)8	(GA)8	(GA)7	(GA)8	(GA)8	(GA)8	(GA)8	(GA)8	-	Introns/Intergenic	-	-	AGGGGAAGCGGGGAGAGAG	ATTCCTCCGCAAAAGTCTAT	242	P	A	A	NA
PMS33	Os_Chr01	1659105	1659134	(CT)15	(CT)15	(CT)15	(CT)15	(CT)15	(CT)14	(CT)15	(CT)14	(CT)15	(CT)13	(CT)14	-	Introns/Intergenic	-	-	GATTTCGATCTGACTGTCCCTAGC	CTTTCTTTCTCAGCTGCTTGTGC	158	P	A	A	RM1247
PMS34	Os_Chr01	1660242	1660281	(CT)20	(CT)10	(CT)20	(CT)20	(CT)20	(CT)10	(CT)20	(CT)20	(CT)20	(CT)20	(CT)20	-	Introns/Intergenic	-	-	GTGTCATATGCAAGCAACAACTCC	AGTATGTACGTCTCCTCCGTTGC	232	P	A	A	RM8068
PMS35	Os_Chr01	1668714	1668753	(ACAT)10	(ACAT)10	(ACAT)10	(ACAT)10	(ACAT)10	(ACAT)10	(ACAT)10	(ACAT)5	(ACAT)10	(ACAT)10	(ACAT)10	-	Introns/Intergenic	-	-	GGCAGCACTAGTGTAGTGTGTGT	GCTTAAAAGATTCGTTTCGCA	207	A	A	A	NA
PMS36	Os_Chr01	1671811	1671834	(CGA)8	(CGA)8	(CGA)8	(CGA)8	(CGA)8	(CGA)8	(CGA)8	(CGA)8	(CGA)8	(CGA)7	(CGA)8	-	Introns/Intergenic	-	-	GTACAACAGCAAGTGCAAGAGATGC	GTGGCGCATGTGAGCTATCC	178	A	A	A	RM6236
PMS37	Os_Chr01	1683212	1683225	(GA)7	(GA)6	(GA)6	(GA)7	(GA)7	(GA)6	(GA)7	(GA)7	(GA)7	(GA)7	(GA)7	-	Introns/Intergenic	-	-	GCACCAGGATCCTCTGTCAT	CCCTTTGTTCGTGTTTTTAAGC	199	P	A	A	NA
PMS38	Os_Chr01	1698017	1698036	(TA)10	(TA)10	(TA)7	(TA)10	(TA)10	(TA)10	(TA)10	(TA)10	(TA)10	(TA)10	(TA)10	-	Introns/Intergenic	-	-	TGGGATAAACCGTAAGGGCT	TACTAGCGCATCACCAATCG	262	P	A	A	NA

Polymorphic SSR (PMS) markers	Chromosomes	Start physical positions (bp)	End physical positions (bp)	Nipponbare	Kasalath	Nagina22	IR64	Pokkali	Bala	Tainung67	Azucena	Moroberekan	*Oryza rufipogon*	*Oryza nivara*	MSU gene locus IDs	Structural annotation	Functional annotation	SSR functional effects	Forward primers (5'-3')	Reverse primers (5'-3')	Amplified product size (bp)	Markers exhibiting polymorphism within *indica* rice	Transcrption factor genes-derived markers	Known cloned genes-derived markers	PMS markers corresponding with RM markers
PMS39	Os_Chr01	1721103	1721116	(TC)7	(TC)6	(TC)6	(TC)7	(TC)6	(TC)6	(TC)6	(TC)6	(TC)6	(TC)6	(TC)6	-	Introns/Intergenic	-	-	TTAAGACGTGGAATCTGCCC	AGTCGCTGCTTCTTCCATGT	212	P	A	A	NA
PMS40	Os_Chr01	1740852	1740867	(TC)8	(TC)8	(TC)8	(TC)8	(TC)8	(TC)8	(TC)8	(TC)8	(TC)9	(TC)8	(TC)8	-	Introns/Intergenic	-	-	TGTTCAAACCACGCAAGCTA	AGTAATGGTTTTGATCGGCG	218	A	A	A	NA
PMS41	Os_Chr01	1794925	1794964	(ATCT)10	(ATCT)10	(ATCT)10	(ATCT)10	(ATCT)10	(ATCT)10	(ATCT)10	(ATCT)9	(ATCT)10	(ATCT)10	(ATCT)10	-	Introns/Intergenic	-	-	GCTAGTCTGATGAATGCCGGTAGG	GCCATGTGGCAATCTGAGAGC	198	A	A	A	RM7278
PMS42	Os_Chr01	1802280	1802297	(TCG)6	(TCG)6	(TCG)6	(TCG)6	(TCG)6	(TCG)6	(TCG)6	(TCG)6	(TCG)6	(TCG)5	(TCG)6	LOC_Os01g04120	DRR	ZOS1-03 - C2H2 zinc finger protein, expressed	-	TAGCCAGGGAGAGGGATCTT	CTTGACCTCCAGCTCAGCCT	215	A	A	A	NA
PMS43	Os_Chr01	1823313	1823374	(AT)31	(AT)31	(AT)9	(AT)31	(AT)31	(AT)31	(AT)9	(AT)13	(AT)31	(AT)31	(AT)31	-	Introns/Intergenic	-	-	TTGCGTACCGTAATATTAAAAC	AGATTGTTGTACTCAGCATACAT	335	P	A	A	NA
PMS44	Os_Chr01	1825024	1825043	(GA)10	(GA)10	(GA)10	(GA)10	(GA)10	(GA)10	(GA)10	(GA)10	(GA)10	(GA)10	(GA)8	-	Introns/Intergenic	-	-	GGTGGCGTTCGTGGTAGTGG	CGCATCAATTGGGTGAGAGC	149	A	A	A	RM10104
PMS45	Os_Chr01	1941661	1941678	(AC)9	(AC)8	(AC)9	(AC)9	(AC)7	(AC)9	(AC)9	(AC)9	(AC)8	(AC)9	(AC)9	LOC_Os01g04350	DRR	hsp20/alpha crystallin family protein, putative, expressed	-	AGGTGGTCACTGACAAGCAG	CGAGAAAGCATTTCACACCA	255	P	A	A	NA
PMS46	Os_Chr01	1985098	1985123	(TG)13	(TG)13	(TG)12	(TG)13	(TG)13	(TG)12	(TG)13	(TG)13	(TG)13	(TG)13	(TG)13	-	Introns/Intergenic	-	-	GAGACCATCGATTTGCGGTGAGC	ACGGTACATCAGCCAAGCAAGAAAGG	344	P	A	A	RM10109
PMS47	Os_Chr01	2065958	2065975	(CGG)6	(CGG)6	(CGG)6	(CGG)6	(CGG)6	(CGG)6	(CGG)5	(CGG)6	(CGG)6	(CGG)6	(CGG)6	LOC_Os01g04600	URR	expressed protein	-	CTTGAGGGCGGTGTACTTGT	CACCTTCTGAATCGTAGCCG	259	A	A	A	NA
PMS48	Os_Chr01	2182095	2182108	(CG)7	(CG)7	(CG)7	(CG)7	(CG)7	(CG)7	(CG)7	(CG)7	(CG)7	(CG)7	(CG)6	LOC_Os01g04770	CDS	retrotransposon protein, putative, unclassified	FRAME_SHIFT	AAAAGTTAAGATTGGCGGGG	GTTGACGATGTGGTCAGCAG	181	A	A	A	NA
PMS49	Os_Chr01	2211202	2211213	(TC)6	(TC)6	(TC)6	(TC)7	(TC)6	(TC)6	(TC)6	(TC)6	(TC)6	(TC)6	(TC)6	-	Introns/Intergenic	-	-	GAGAACGCGGAATGTGACTT	GTTTCTGCCAGCCTTTTCAA	198	P	A	A	NA
PMS50	Os_Chr01	2229274	2229294	(CGC)7	(CGC)7	(CGC)7	(CGC)7	(CGC)7	(CGC)7	(CGC)7	(CGC)7	(CGC)7	(CGC)7	(CGC)6	LOC_Os01g04840	CDS	vacuolar protein sorting associating protein 4B, putative, expressed	CODON_DELETION	GCCTCTTGCAGTGCCTCCTTGG	GCCGCCAAGTTCACCGAGTACC	206	A	A	A	RM10120
PMS51	Os_Chr01	2233150	2233163	(CT)7	(CT)7	(CT)7	(CT)7	(CT)7	(CT)7	(CT)7	(CT)7	(CT)7	(CT)7	(CT)6	-	Introns/Intergenic	-	-	CTCCAAATCAGCCCTCTCTG	ACCGGCGAGAGAGAGAAATC	196	A	A	A	NA

Polymorphic SSR (PMS) markers	Chromosomes	Start physical positions (bp)	End physical positions (bp)	Nipponbare	Kasalath	Nagina22	IR64	Pokkali	Bala	Tainung67	Azucena	Moroberekan	*Oryza rufipogon*	*Oryza nivara*	MSU gene locus IDs	Structural annotation	Functional annotation	SSR functional effects	Forward primers (5'-3')	Reverse primers (5'-3')	Amplified product size (bp)	Markers exhibiting polymorphism within *indica* rice	Transcrption factor genes-derived markers	Known cloned genes-derived markers	PMS markers corresponding with RM markers
PMS52	Os_Chr01	2261628	2261641	(CG)7	(CG)7	(CG)7	(CG)7	(CG)7	(CG)7	(CG)7	(CG)6	(CG)7	(CG)7	(CG)7	LOC_Os01g04880	URR	retrotransposon protein, putative, unclassified, expressed	-	CTTCACTCCCACTG CTCCTC	CCTCGTCCTTCTCCT TGATG	222	A	A	A	NA
PMS53	Os_Chr01	2453768	2453788	(CGG)7	(CGG)5	(CGG)7	(CGG)7	(CGG)7	(CGG)7	(CGG)7	(CGG)7	(CGG)7	(CGG)7	(CGG)7	-	Introns/Intergenic	-	-	ATGACCTTTGTCCT TGCGTT	GCGATAGCACAGTC AGTGGA	162	P	A	A	NA
PMS54	Os_Chr01	2516936	2516951	(CT)8	(CT)8	(CT)8	(CT)7	(CT)7	(CT)8	(CT)7	(CT)7	(CT)8	(CT)8	(CT)8	-	Introns/Intergenic	-	-	AAGTTGAGGATTGC GGATTG	CCCCTTAGCATGTCG GTTTA	196	P	A	A	NA
PMS55	Os_Chr01	2518283	2518296	(CT)7	(CT)7	(CT)7	(CT)7	(CT)7	(CT)7	(CT)8	(CT)7	(CT)7	(CT)7	(CT)7	-	Introns/Intergenic	-	-	AAGTTGAGGATTGC GGATTG	CCCCTTAGCATGTCG GTTTA	194	A	A	A	NA
PMS56	Os_Chr01	2551375	2551388	(GA)7	(GA)7	(GA)7	(GA)7	(GA)6	(GA)7	(GA)6	(GA)7	(GA)7	(GA)7	(GA)7	-	Introns/Intergenic	-	-	CAGGTCTGAAGGG GAACAGA	ATATGGGTCCCACGT GTCAT	104	P	A	A	NA
PMS57	Os_Chr01	2555069	2555086	(TA)9	(TA)9	(TA)9	(TA)9	(TA)9	(TA)9	(TA)9	(TA)8	(TA)9	(TA)9	(TA)9	-	Introns/Intergenic	-	-	AGTTGCCAAACGCA CAAACT	TTTGTACGTGTCCCC ATCTTC	273	A	A	A	NA
PMS58	Os_Chr01	2566184	2566197	(AT)7	(AT)7	(AT)6	(AT)8	(AT)7	(AT)6	(AT)7	(AT)7	(AT)7	(AT)7	(AT)7	-	Introns/Intergenic	-	-	GCAGGAACGAAATA AGCTGC	ACGGAGATCATTGCC AGAGT	249	P	A	A	NA
PMS59	Os_Chr01	2608563	2608576	(AG)7	(AG)7	(AG)8	(AG)8	(AG)7	(AG)8	(AG)7	(AG)7	(AG)11	(AG)7	(AG)7	-	Introns/Intergenic	-	-	TGATACGGGGATAT GGCATT	TCCAACTGCTAGTGT GGCTG	205	P	A	A	NA
PMS60	Os_Chr01	2731818	2731833	(CT)8	(CT)8	(CT)8	(CT)8	(CT)8	(CT)8	(CT)8	(CT)8	(CT)7	(CT)8	(CT)8	-	Introns/Intergenic	-	-	ATCCCGAATCCAAA TCCTTC	GGAGGAGGAGGGAG AGAGAG	152	A	A	A	NA
PMS61	Os_Chr01	2766661	2766678	(GGT)6	(GGT)6	(GGT)6	(GGT)5	(GGT)6	(GGT)6	(GGT)6	(GGT)6	(GGT)6	(GGT)6	(GGT)6	LOC_Os01g05790	CDS	SLT1 protein, putative, expressed	CODON_DELETION	ACCCTGTCCATGGA GAACAC	TGAACAATTCCCTGT CACCA	166	P	A	A	NA
PMS62	Os_Chr01	2823514	2823537	(AT)12	(AT)12	(AT)12	(AT)7	(AT)6	(AT)12	(AT)7	(AT)12	(AT)12	(AT)12	(AT)12	-	Introns/Intergenic	-	-	TGCACGCTACATCT TGTCTGTCG	GGCACCATGGAGTC CTATCTGG	136	P	A	A	RM10143
PMS63	Os_Chr01	2830015	2830054	(TA)20	(TA)20	(TA)20	(TA)9	(TA)20	(TA)20	(TA)20	(TA)20	(TA)20	(TA)20	(TA)20	-	Introns/Intergenic	-	-	ACCCGTTAATGTGT GATAGCATCC	GGGATGTTCCGATTC GTTGC	192	P	A	A	RM10144
PMS64	Os_Chr01	2983717	2983731	(CGA)5	(CGA)5	(CGA)5	(CGA)5	(CGA)5	(CGA)5	(CGA)5	(CGA)5	(CGA)7	(CGA)5	(CGA)5	LOC_Os01g06280	CDS	TKL_IRAK_CrRLK1L-1.4 - The CrRLK1L-1 subfamily has homology to the CrRLK1L homolog, expressed	CODON_INSERTION	TGTACCTCGGCTAC GACCTC	CACGAACGTGACGA CGAG	194	A	A	A	NA

Polymorphic SSR (PMS) markers	Chromosomes	Start physical positions (bp)	End physical positions (bp)	Nipponbare	Kasalath	Nagina22	IR64	Pokkali	Bala	Tainung67	Azucena	Moroberekan	*Oryza rufipogon*	*Oryza nivara*	MSU gene locus IDs	Structural annotation	Functional annotation	SSR functional effects	Forward primers (5'-3')	Reverse primers (5'-3')	Amplified product size (bp)	Markers exhibiting polymorphism within *indica* rice	Transcrption factor genes-derived markers	Known cloned genes-derived markers	PMS markers corresponding with RM markers
PMS65	Os_Chr01	2989203	2989226	(CGG)8	(CGG)8	(CGG)8	(CGG)8	(CGG)8	(CGG)8	(CGG)8	(CGG)8	(CGG)8	(CGG)6	(CGG)8	LOC_Os01g06290	URR	splicing factor, arginine/serine-rich, putative, expressed	-	GAGAGGAACGAGAAAGAGGATAAGC	CCCTATCTTTACCCTAGTCGATCTCC	193	A	A	A	RM10147
PMS66	Os_Chr01	3005273	3005290	(CTG)6	(CTG)6	(CTG)6	(CTG)6	(CTG)6	(CTG)6	(CTG)6	(CTG)6	(CTG)6	(CTG)6	(CTG)5	LOC_Os01g06320	CDS	MYB family transcription factor, putative, expressed	CODON_DELETION	AGGAAGAACTCCGGGACACT	ATCGATGTGTGTGCAGATCC	259	A	P	A	NA
PMS67	Os_Chr01	3076236	3076261	(AT)13	(AT)13	(AT)13	(AT)13	(AT)13	(AT)13	(AT)13	(AT)13	(AT)13	(AT)13	(AT)12	-	Introns/Intergenic	-	-	TCTTCTCATTCTACACCCAACC	CCAGGATTATTCGTAGTTAGGG	572	A	A	A	RM10152
PMS68	Os_Chr01	3084521	3084538	(GGC)6	(GGC)6	(GGC)6	(GGC)6	(GGC)6	(GGC)6	(GGC)6	(GGC)6	(GGC)5	(GGC)6	(GGC)6	LOC_Os01g06540	CDS	PHD finger protein, putative, expressed	CODON_DELETION	GGAAGTCGCTGAAGATGTCC	TCTGTCACGTTCTCCCTCCT	237	A	A	A	NA
PMS69	Os_Chr01	3268815	3268840	(AG)13	(AG)13	(AG)13	(AG)13	(AG)13	(AG)13	(AG)13	(AG)13	(AG)13	(AG)12	(AG)13	-	Introns/Intergenic	-	-	CCTCACCGAGCCTAGAAACTTAGG	ACCCGCCTATCATACCTTATCTTCC	684	A	A	A	RM1177
PMS70	Os_Chr01	3289427	3289478	(AT)26	(AT)26	(AT)26	(AT)26	(AT)26	(AT)26	(AT)26	(AT)26	(AT)7	(AT)26	(AT)26	-	Introns/Intergenic	-	-	GGGCATGTCATTTCCTACTCCTACCG	CCACAAAGTCGCAATGTCCTGTAGC	250	A	A	A	RM10162
PMS71	Os_Chr01	3324550	3324573	(TGG)8	(TGG)8	(TGG)8	(TGG)8	(TGG)8	(TGG)8	(TGG)8	(TGG)7	(TGG)8	(TGG)7	(TGG)8	LOC_Os01g07070	CDS	transposon protein, putative, CACTA, En/Spm sub-class, expressed	CODON_CHANGE_PLUS_CODON_DELETION	TATGGTGGTGGTCCAGGTTCATATCC	TTAACCTTTGCAGCTGGCTCTGG	254	A	A	A	RM6873
PMS72	Os_Chr01	3355010	3355024	(GCG)5	(GCG)5	(GCG)5	(GCG)5	(GCG)5	(GCG)5	(GCG)5	(GCG)5	(GCG)6	(GCG)5	(GCG)5	LOC_Os01g07110	URR	BRCA2 repeat family protein, expressed	-	CTGCCACCTCCTCTGCAT	AATGGATGTTGATGGGCTGT	259	A	A	A	NA
PMS73	Os_Chr01	3415578	3415595	(CCG)6	(CCG)6	(CCG)6	(CCG)6	(CCG)6	(CCG)6	(CCG)6	(CCG)6	(CCG)6	(CCG)6	(CCG)5	LOC_Os01g07240	CDS	expressed protein	CODON_DELETION	CGTGCTTCCCATACTTCCTT	GGTGTTCTCCAAGCTGCTGT	242	A	A	A	NA
PMS74	Os_Chr01	3518846	3518863	(GCG)6	(GCG)5	(GCG)6	(GCG)6	(GCG)6	(GCG)6	(GCG)6	(GCG)6	(GCG)7	(GCG)6	(GCG)6	LOC_Os01g07420	CDS	hydrolase, alpha/beta fold family protein, putative, expressed	CODON_INSERTION	GAAGCCATGGACGAGCAC	CTCTCATCAGCGCCATGTT	197	P	A	A	NA
PMS75	Os_Chr01	3521919	3521936	(CGG)6	(CGG)6	(CGG)6	(CGG)6	(CGG)6	(CGG)5	(CGG)6	(CGG)5	(CGG)6	(CGG)6	(CGG)6	-	Introns/Intergenic	-	-	GGGTGATGTGGGTGTAGAGC	CGGAAGCCGACGAGTAGTAG	130	P	A	A	NA
PMS76	Os_Chr01	3607018	3607033	(GA)8	(GA)8	(GA)8	(GA)8	(GA)11	(GA)8	(GA)8	(GA)8	(GA)11	(GA)8	(GA)11	-	Introns/Intergenic	-	-	ACATCAAAGTGAACAGGCCC	GATCCTAGATTCTGCACCGC	202	P	A	A	NA
PMS77	Os_Chr01	3689219	3689234	(CT)8	(CT)10	(CT)8	(CT)8	(CT)8	(CT)9	(CT)8	(CT)8	(CT)8	(CT)8	(CT)8	-	Introns/Intergenic	-	-	ATGTTTGGTTCCATGGGCTA	AACGAGGAAACATCCTCTCG	261	P	A	A	NA

Polymorphic SSR (PMS) markers	Chromosomes	Start physical positions (bp)	End physical positions (bp)	Nipponbare	Kasalath	Nagina22	IR64	Pokkali	Bala	Tainung67	Azucena	Moroberekan	*Oryza rufipogon*	*Oryza nivara*	MSU gene locus IDs	Structural annotation	Functional annotation	SSR functional effects	Forward primers (5'-3')	Reverse primers (5'-3')	Amplified product size (bp)	Markers exhibiting polymorphism within *indica* rice	Transcrption factor genes-derived markers	Known cloned genes-derived markers	PMS markers corresponding with RM markers
PMS78	Os_Chr01	3704264	3704284	(GCC)7	(GCC)7	(GCC)7	(GCC)7	(GCC)5	(GCC)7	(GCC)7	(GCC)7	(GCC)7	(GCC)7	(GCC)7	LOC_Os01g07710	CDS	expressed protein	CODON_DELETION	AGTGACTCCTCTTCCCAACACAAACC	TGAGCAGGTCGACGGTCTGG	251	P	A	A	RM10181
PMS79	Os_Chr01	3763313	3763330	(AT)9	(AT)9	(AT)7	(AT)9	(AT)9	(AT)9	(AT)9	(AT)9	(AT)9	(AT)9	(AT)9	-	Introns/Intergenic	-	-	GGTATCGTCTCAGCTCCGTC	CGACCGACGAAAGATGATTT	240	P	A	A	NA
PMS80	Os_Chr01	3804893	3804910	(GGA)6	(GGA)6	(GGA)6	(GGA)6	(GGA)6	(GGA)6	(GGA)6	(GGA)5	(GGA)5	(GGA)6	(GGA)6	LOC_Os01g07870	CDS	ABC transporter family protein, putative, expressed	CODON_DELETION	ACGTGCCCTTGTTCTTCCT	AGGTACCCCTCGTACGCC	229	A	A	A	NA
PMS81	Os_Chr01	3951644	3951675	(AG)16	(AG)16	(AG)16	(AG)16	(AG)16	(AG)16	(AG)15	(AG)16	(AG)16	(AG)16	(AG)16	-	Introns/Intergenic	-	-	CTCACCCCTTTTCAAAGCAA	TCACATCCAACAGACAAGGG	241	A	A	A	NA
PMS82	Os_Chr01	3953354	3953371	(GCG)6	(GCG)6	(GCG)6	(GCG)7	(GCG)7	(GCG)6	(GCG)6	(GCG)6	(GCG)6	(GCG)7	(GCG)6	LOC_Os01g08160	CDS	MYB family transcription factor, putative, expressed	CODON_INSERTION	CGAGGATTGGTGTGTGTTTG	CCGATGTTCTTCACCGGTAT	147	P	P	A	NA
PMS83	Os_Chr01	4004758	4004775	(CGC)6	(CGC)6	(CGC)6	(CGC)6	(CGC)6	(CGC)6	(CGC)6	(CGC)6	(CGC)6	(CGC)5	(CGC)6	LOC_Os01g08220	CDS	d18|OsGA3ox2	CODON_DELETION	GTGCCCGACCAGAAGGAAC	CACTTGAAGAACCCGCTCTG	213	A	A	P	NA
PMS84	Os_Chr01	4033460	4033477	(GCT)6	(GCT)6	(GCT)6	(GCT)6	(GCT)6	(GCT)6	(GCT)6	(GCT)5	(GCT)6	(GCT)6	(GCT)6	-	Introns/Intergenic	-	-	TTGTTGTTTTGCAGTCCTCG	AATATTCGGCGAGCATTTTG	198	A	A	A	NA
PMS85	Os_Chr01	4127326	4127352	(CGC)9	(CGC)9	(CGC)9	(CGC)7	(CGC)9	(CGC)9	(CGC)9	(CGC)9	(CGC)9	(CGC)9	(CGC)9	-	Introns/Intergenic	-	-	AAGCCAAGCCAAGCATCTCTCC	AGTCGCTGGAGTCGGAGACC	293	P	A	A	RM6521
PMS86	Os_Chr01	4303796	4303821	(TA)13	(TA)13	(TA)13	(TA)13	(TA)13	(TA)13	(TA)6	(TA)13	(TA)13	(TA)13	(TA)13	-	Introns/Intergenic	-	-	GGAGGGAGTAGGGTGGATGAGC	CAACGTCGGAGAGGCAGAGC	239	A	A	A	RM10250
PMS87	Os_Chr01	4326125	4326138	(GA)7	(GA)7	(GA)7	(GA)7	(GA)7	(GA)7	(GA)7	(GA)6	(GA)7	(GA)7	(GA)7	LOC_Os01g08680	URR	myb/SANT domain protein, putative, expressed	-	CATTCAACCACGAAATCACG	GAAGCCTTCCGGTGAACTC	277	A	A	A	NA
PMS88	Os_Chr01	4343870	4343887	(GCA)6	(GCA)7	(GCA)7	(GCA)6	(GCA)6	(GCA)7	(GCA)5	(GCA)6	(GCA)6	(GCA)7	(GCA)6	LOC_Os01g08710	CDS	WRKY102, expressed	CODON_DELETION	AATTTGGTTCGACATCTGGC	CAATCTGCTTGCTGGTGTGT	195	P	P	A	NA
PMS89	Os_Chr01	4391461	4391476	(TC)8	(TC)15	(TC)8	(TC)8	(TC)8	(TC)15	(TC)8	(TC)8	(TC)10	(TC)8	(TC)15	-	Introns/Intergenic	-	-	TCTGCAACTGACTCAGCCAC	TGGCTTTGGACCTAGATTGG	262	P	A	A	NA
PMS90	Os_Chr01	4411879	4411954	(TA)38	(TA)38	(TA)38	(TA)38	(TA)12	(TA)13	(TA)38	(TA)38	(TA)13	(TA)13	(TA)7	-	Introns/Intergenic	-	-	ACGCTACACTACCAAGAAATCC	TGAGAAAGGAGGGAGTAGTTAGC	251	P	A	A	RM10261

Polymorphic SSR (PMS) markers	Chromosomes	Start physical positions (bp)	End physical positions (bp)	Nipponbare	Kasalath	Nagina22	IR64	Pokkali	Bala	Tainung67	Azucena	Moroberekan	*Oryza rufipogon*	*Oryza nivara*	MSU gene locus IDs	Structural annotation	Functional annotation	SSR functional effects	Forward primers (5'-3')	Reverse primers (5'-3')	Amplified product size (bp)	Markers exhibiting polymorphism within *indica* rice	Transcrption factor genes-derived markers	Known cloned genes-derived markers	PMS markers corresponding with RM markers
PMS91	Os_Chr01	4433338	4433355	(CGG)6	(CGG)6	(CGG)6	(CGG)6	(CGG)6	(CGG)6	(CGG)6	(CGG)5	(CGG)5	(CGG)6	(CGG)6	LOC_Os01g08820	CDS	PHD-finger domain containing protein, putative, expressed	CODON_DELETION	GACTGAGGGAGAAGCACCAC	GTTTCGCTCTGTGCCTCC	143	A	A	A	NA
PMS92	Os_Chr01	4437871	4437904	(AT)17	(AT)17	(AT)9	(AT)6	(AT)6	(AT)17	(AT)17	(AT)17	(AT)17	(AT)17	(AT)12	LOC_Os01g08830	DRR	OsFBDUF1 - F-box and DUF domain containing protein, expressed	-	CAACTGTCTGAACTTTGGACAACTCG	CCTCCCGCAATACATGAACACG	152	P	A	A	RM10264
PMS93	Os_Chr01	4657777	4657800	(AT)12	(AT)12	(AT)12	(AT)12	(AT)12	(AT)12	(AT)12	(AT)9	(AT)12	(AT)12	(AT)12	-	Introns/Intergenic	-	-	GCTTGTCGAAAAACGAGCAT	TACGCTGAACTAAACACGGC	262	A	A	A	NA
PMS94	Os_Chr01	4673741	4673764	(CGC)8	(CGC)8	(CGC)8	(CGC)8	(CGC)8	(CGC)8	(CGC)6	(CGC)8	(CGC)8	(CGC)8	(CGC)8	LOC_Os01g09246	CDS	glycine-rich protein, putative, expressed	CODON_DELETION	CTTCTAGCGGCTGGAGTCTGATGC	GAGAGGTTGGCGGAGATGACG	408	A	A	A	RM10274
PMS95	Os_Chr01	4688229	4688252	(CGC)8	(CGC)8	(CGC)5	(CGC)8	(CGC)8	(CGC)8	(CGC)8	(CGC)8	(CGC)8	(CGC)8	(CGC)7	LOC_Os01g09252	URR	WD domain, G-beta repeat domain containing protein, expressed	-	TTACCGCCTCCCTCACCTCACG	TTAGGGTTCGGGTTCGGGTTCG	439	P	A	A	RM10275
PMS96	Os_Chr01	4797266	4797292	(CTG)9	(CTG)9	(CTG)9	(CTG)9	(CTG)9	(CTG)5	(CTG)9	(CTG)9	(CTG)9	(CTG)9	(CTG)9	LOC_Os01g09420	CDS	expressed protein	CODON_DELETION	GTTGCATCTTCTCCCCATGT	CCGGAGCCTGTTCTATGGTA	248	P	P	A	NA
PMS97	Os_Chr01	4797825	4797839	(TGC)5	(TGC)6	(TGC)6	(TGC)5	(TGC)5	(TGC)6	(TGC)5	(TGC)5	(TGC)5	(TGC)6	(TGC)5	LOC_Os01g09420	CDS	expressed protein	CODON_INSERTION	AATACATGTGCCTTCCCTGC	CTTCCGCCATCGACTACTTC	146	P	P	A	NA
PMS98	Os_Chr01	4798366	4798392	(GGC)9	(GGC)9	(GGC)9	(GGC)9	(GGC)9	(GGC)9	(GGC)5	(GGC)9	(GGC)9	(GGC)9	(GGC)9	LOC_Os01g09420	URR	expressed protein	-	GGGAATCTCCGTTTCCTTGG	ACCAAACACCAAACACCAAACC	188	A	P	A	RM10279
PMS99	Os_Chr01	4864024	4864037	(CG)7	(CG)6	(CG)7	(CG)7	(CG)7	(CG)7	(CG)7	(CG)7	(CG)7	(CG)7	(CG)7	LOC_Os01g09530	CDS	transposon protein, putative, unclassified, expressed	FRAME_SHIFT	AGTAAAGATTGACGGGGGCT	GGACCCCAACTTCTCTCCAT	247	P	A	A	NA
PMS100	Os_Chr01	4967447	4967488	(AAG)14	(AAG)17	(AAG)17	(AAG)17	(AAG)14	(AAG)17	(AAG)14	(AAG)14	(AAG)17	(AAG)14	(AAG)14	-	Introns/Intergenic	-	-	GTATTCCTTGCTGCTGCTGATGG	GACTGGAGATGTGATCGGAAACC	177	P	A	A	RM5641
PMS101	Os_Chr01	5025635	5025658	(GGC)8	(GGC)8	(GGC)8	(GGC)5	(GGC)8	(GGC)8	(GGC)8	(GGC)7	(GGC)8	(GGC)8	(GGC)8	LOC_Os01g09750	CDS	RNA methyltransferase protein, putative, expressed	CODON_DELETION	GTTGCGCTTCGGGAAGTAGCC	CCGTCCATTCTCCATCCGAACC	226	P	A	A	RM10292
PMS102	Os_Chr01	5088194	5088235	(AT)21	(AT)21	(AT)9	(AT)6	(AT)21	(AT)21	(AT)21	(AT)21	(AT)7	(AT)6	(AT)21	-	Introns/Intergenic	-	-	GAATTACTCTCTCCGTTTCACG	ATTACTTCCTCCGTTTCACG	156	P	A	A	RM10298
PMS103	Os_Chr01	5088949	5088960	(CT)6	(CT)6	(CT)6	(CT)6	(CT)6	(CT)6	(CT)6	(CT)6	(CT)6	(CT)6	(CT)7	-	Introns/Intergenic	-	-	AAAAAGAATGCCTCCCTTGG	CTACATGCAGCTATGGGGGT	166	A	A	A	NA

Polymorphic SSR (PMS) markers	Chromosomes	Start physical positions (bp)	End physical positions (bp)	Nipponbare	Kasalath	Nagina22	IR64	Pokkali	Bala	Tainung67	Azucena	Moroberekan	*Oryza rufipogon*	*Oryza nivara*	MSU gene locus IDs	Structural annotation	Functional annotation	SSR functional effects	Forward primers (5'-3')	Reverse primers (5'-3')	Amplified product size (bp)	Markers exhibiting polymorphism within *indica* rice	Transcrption factor genes-derived markers	Known cloned genes-derived markers	PMS markers corresponding with RM markers
PMS104	Os_Chr01	5140380	5140405	(TC)13	(TC)12	(TC)13	(TC)13	(TC)13	(TC)12	(TC)13	(TC)13	(TC)13	(TC)13	(TC)13	-	Introns/Intergenic	-	-	CAGGAACCAACCTTCTTCTTGACC	GTCAGACTCCGATCTGGGATGG	153	P	A	A	RM3604
PMS105	Os_Chr01	5151000	5151077	(GA)39	(GA)39	(GA)39	(GA)39	(GA)39	(GA)38	(GA)39	(GA)39	(GA)39	(GA)39	(GA)39	-	Introns/Intergenic	-	-	GGAGATTCCCTTCCATGTATGC	ACGAGCTCAGTGTCACCATCG	266	P	A	A	RM3530
PMS106	Os_Chr01	5213026	5213041	(GC)8	(GC)8	(GC)8	(GC)8	(GC)8	(GC)8	(GC)8	(GC)8	(GC)8	(GC)8	(GC)6	-	Introns/Intergenic	-	-	TCAGGTCCCCAAATTTCAGA	GCTCCGCTCAAGGTACAATC	276	A	A	A	NA
PMS107	Os_Chr01	5271589	5271603	(TTA)5	(TTA)6	(TTA)6	(TTA)5	(TTA)5	(TTA)6	(TTA)5	(TTA)5	(TTA)5	(TTA)6	(TTA)5	-	Introns/Intergenic	-	-	ACACGCACAATATGAGGGGT	CTCATCTACCCCATGAACCG	128	P	A	A	NA
PMS108	Os_Chr01	5352615	5352628	(TA)7	(TA)7	(TA)7	(TA)7	(TA)6	(TA)7	(TA)7	(TA)7	(TA)7	(TA)7	(TA)6	LOC_Os01g10180	DRR	avr9/Cf-9 rapidly elicited protein 146, putative, expressed	-	AGGAAAAATTACACGCAGCC	AGCGAGCGCGAATACTAAAG	128	P	A	A	NA
PMS109	Os_Chr01	5369282	5369307	(AT)13	(AT)13	(AT)13	(AT)13	(AT)13	(AT)13	(AT)13	(AT)13	(AT)6	(AT)13	(AT)7	-	Introns/Intergenic	-	-	ACTCCCTCCGTCCCTAAAAA	CTACTCTCTCCCTCCGTCCC	159	A	A	A	NA
PMS110	Os_Chr01	5458646	5458661	(TC)8	(TC)8	(TC)8	(TC)8	(TC)8	(TC)7	(TC)8	(TC)8	(TC)8	(TC)8	(TC)8	-	Introns/Intergenic	-	-	TTCACTTGCATGGTTGCATT	CGTGCAAACTGTATGTGCCT	158	P	A	A	NA
PMS111	Os_Chr01	5547417	5547438	(AG)11	(AG)11	(AG)11	(AG)11	(AG)11	(AG)11	(AG)11	(AG)10	(AG)11	(AG)11	(AG)11	-	Introns/Intergenic	-	-	CTCAAGGTGAGGGAGGGAG	GTATACGTGGCTGCCCAGTT	135	A	A	A	NA
PMS112	Os_Chr01	5593746	5593760	(GCA)5	(GCA)6	(GCA)5	(GCA)6	(GCA)5	(GCA)5	(GCA)5	(GCA)5	(GCA)5	(GCA)5	(GCA)5	LOC_Os01g10530	DRR	expressed protein	-	CAGCCATCTCTCTCCCTGAC	CAACTCCAGATGGGTTTCGT	216	P	A	A	NA
PMS113	Os_Chr01	5802427	5802488	(AG)31	(AG)31	(AG)31	(AG)31	(AG)31	(AG)30	(AG)31	(AG)31	(AG)30	(AG)31	(AG)31	-	Introns/Intergenic	-	-	GCTTGATCTGCCCTTGTTTCTTGG	AACTCGAGCGGCCTTCTCAGC	285	P	A	A	RM10346
PMS114	Os_Chr01	5848514	5848531	(GCG)6	(GCG)5	(GCG)6	(GCG)6	(GCG)6	(GCG)6	(GCG)6	(GCG)6	(GCG)6	(GCG)6	(GCG)6	LOC_Os01g10970	CDS	auxin-induced protein 5NG4, putative, expressed	CODON_DELETION	ACGAGTACCCCTGCAAGATG	GGAGGTAGTAGGAGACCCCG	183	P	A	A	NA
PMS115	Os_Chr01	5877652	5877672	(GGC)7	(GGC)7	(GGC)6	(GGC)7	(GGC)7	(GGC)7	(GGC)7	(GGC)7	(GGC)7	(GGC)7	(GGC)7	LOC_Os01g11010	CDS	peptide-N4-asparagine amidase A, putative, expressed	CODON_DELETION	TGTAGGTGAAACCGAAGGAGTGG	CCTCTCAAGCTTCGGCTATCTGC	190	P	A	A	RM10351
PMS116	Os_Chr01	5983306	5983323	(GA)9	(GA)9	(GA)9	(GA)9	(GA)9	(GA)9	(GA)9	(GA)6	(GA)9	(GA)9	(GA)9	-	Introns/Intergenic	-	-	CATGGCAAATTTGAGTCCCT	AGAGTTTCCCCACCAATGTG	218	A	A	A	NA

Polymorphic SSR (PMS) markers	Chromosomes	Start physical positions (bp)	End physical positions (bp)	Nipponbare	Kasalath	Nagina22	IR64	Pokkali	Bala	Tainung67	Azucena	Moroberekan	*Oryza rufipogon*	*Oryza nivara*	MSU gene locus IDs	Structural annotation	Functional annotation	SSR functional effects	Forward primers (5'-3')	Reverse primers (5'-3')	Amplified product size (bp)	Markers exhibiting polymorphism within *indica* rice	Transcrption factor genes-derived markers	Known cloned genes-derived markers	PMS markers corresponding with RM markers
PMS117	Os_Chr01	6066172	6066189	(GTC)6	(GTC)5	(GTC)6	(GTC)6	(GTC)6	(GTC)6	(GTC)6	(GTC)6	(GTC)5	(GTC)6	(GTC)6	LOC_Os01g11320	CDS	transposon protein, putative, Pong sub-class	CODON_DELETION	ACTGAACCACGACGCTTTCT	TGATCCTGTGATGGCAGTGT	179	P	A	A	NA
PMS118	Os_Chr01	6096247	6096273	(TGC)9	(TGC)9	(TGC)9	(TGC)9	(TGC)9	(TGC)9	(TGC)9	(TGC)9	(TGC)9	(TGC)9	(TGC)6	LOC_Os01g11350	CDS	bZIP transcription factor domain containing protein, expressed	CODON_DELETION	GCTTGGTCGCCGAGAAGTCG	AGATCCCAACCATGGCGACAGC	237	A	P	A	RM10358
PMS119	Os_Chr01	6130923	6130940	(TAA)6	(TAA)5	(TAA)5	(TAA)5	(TAA)5	(TAA)6	(TAA)6	(TAA)6	(TAA)6	(TAA)6	(TAA)6	-	Introns/Intergenic	-	-	GATGGATGGAGCACTGGATT	CCAACAGATCAGCAGGTTCA	109	P	A	A	NA
PMS120	Os_Chr01	6157004	6157071	(CT)34	(CT)34	(CT)34	(CT)34	(CT)34	(CT)34	(CT)34	(CT)34	(CT)34	(CT)34	(CT)33	-	Introns/Intergenic	-	-	GCTATAGACGCCTCCTCCTTATCC	AAAGCTAGCTAGGACCGACATGC	196	A	A	A	RM3873
PMS121	Os_Chr01	6183328	6183345	(CCG)6	(CCG)5	(CCG)6	(CCG)6	(CCG)6	(CCG)5	(CCG)6	(CCG)6	(CCG)6	(CCG)6	(CCG)6	LOC_Os01g11480	CDS	zinc finger, C3HC4 type domain containing protein, expressed	CODON_DELETION	CGTCTCCCCTTCCTCCAC	GCTCGTGGTGTTCTCCCTC	259	P	A	A	NA
PMS122	Os_Chr01	6186004	6186027	(GCC)8	(GCC)8	(GCC)8	(GCC)8	(GCC)8	(GCC)7	(GCC)8	(GCC)8	(GCC)8	(GCC)8	(GCC)8	LOC_Os01g11490	CDS	zinc finger, C3HC4 type domain containing protein, expressed	CODON_DELETION	CGCTTCTTGTTGGTGACGTTCC	TGCTGCTCCACCACCTCCTACC	182	P	A	A	RM6186
PMS123	Os_Chr01	6223465	6223479	(CCG)5	(CCG)5	(CCG)5	(CCG)5	(CCG)5	(CCG)5	(CCG)5	(CCG)5	(CCG)7	(CCG)5	(CCG)5	LOC_Os01g11550	CDS	TCP family transcription factor, putative, expressed	CODON_INSERTION	GTAGAACTGGATGGCGGTGT	GGAGGTGGAGGTGAGATTGT	207	A	P	A	NA
PMS124	Os_Chr01	6390164	6390181	(TGG)6	(TGG)5	(TGG)6	(TGG)6	(TGG)6	(TGG)5	(TGG)6	(TGG)6	(TGG)5	(TGG)6	(TGG)6	-	Introns/Intergenic	-	-	AGCCGTTGTCCGTATCAATC	TCCCTAGCTAGTCCCTTCCC	279	P	A	A	NA
PMS125	Os_Chr01	6390795	6390812	(CGC)6	(CGC)6	(CGC)6	(CGC)6	(CGC)6	(CGC)6	(CGC)6	(CGC)6	(CGC)6	(CGC)6	(CGC)5	LOC_Os01g11810	CDS	powdery mildew resistant protein 5, putative, expressed	CODON_DELETION	TCGTAGAGCGGGTACGACTC	TCACCTCTCCATTGTTGTCG	239	A	A	A	NA
PMS126	Os_Chr01	6517779	6517836	(TA)29	(TA)29	(TA)16	(TA)29	(TA)8	(TA)9	(TA)29	(TA)29	(TA)29	(TA)7	(TA)8	-	Introns/Intergenic	-	-	CCTTCCAAGTATAAGGGTAAGAGG	CCAAGTGACTTCGTAAGATTGG	276	P	A	A	RM10386
PMS127	Os_Chr01	6523670	6523690	(CGC)7	(CGC)7	(CGC)6	(CGC)7	(CGC)7	(CGC)7	(CGC)6	(CGC)7	(CGC)7	(CGC)7	(CGC)7	LOC_Os01g11970	URR	expressed protein	-	AGAACAAGCATGAGAAGGCCACAGC	GTGGAGCGGCGAAGTTGTCG	259	P	A	A	RM10388
PMS128	Os_Chr01	6540832	6540846	(CCG)5	(CCG)6	(CCG)5	(CCG)5	(CCG)5	(CCG)5	(CCG)5	(CCG)5	(CCG)5	(CCG)5	(CCG)5	-	Introns/Intergenic	-	-	CTATAGCCCGTCACTCTCGC	ACTTCGTGTTTGTTTGCGTG	268	P	A	A	NA
PMS129	Os_Chr01	6581847	6581868	(GA)11	(GA)11	(GA)11	(GA)10	(GA)11	(GA)11	(GA)9	(GA)11	(GA)11	(GA)11	(GA)11	-	Introns/Intergenic	-	-	GCAACGTTACGTCTTGGCATGG	CCTCTCGCGTCTCTCTCAACG	90	P	A	A	RM10390

Polymorphic SSR (PMS) markers	Chromosomes	Start physical positions (bp)	End physical positions (bp)	Nipponbare	Kasalath	Nagina22	IR64	Pokkali	Bala	Tainung67	Azucena	Moroberekan	*Oryza rufipogon*	*Oryza nivara*	MSU gene locus IDs	Structural annotation	Functional annotation	SSR functional effects	Forward primers (5'-3')	Reverse primers (5'-3')	Amplified product size (bp)	Markers exhibiting polymorphism within *indica* rice	Transcrption factor genes-derived markers	Known cloned genes-derived markers	PMS markers corresponding with RM markers
PMS130	Os_Chr01	6674948	6674997	(TA)25	(TA)25	(TA)25	(TA)8	(TA)25	(TA)9	(TA)25	(TA)25	(TA)7	(TA)25	(TA)25	-	Introns/Intergenic	-	-	CTCTCTCCTGATGGTAAACTCTCC	ATATAGAGGTGCCTTGTGTGAAGG	482	P	A	A	RM10400
PMS131	Os_Chr01	6683743	6683760	(AGC)6	(AGC)6	(AGC)6	(AGC)6	(AGC)5	(AGC)6	(AGC)6	(AGC)6	(AGC)6	(AGC)6	(AGC)6	-	Introns/Intergenic	-	-	TAGAGGCCAGAGAGAACGGA	GTCCGGCGACTACTGTTGAT	109	P	A	A	NA
PMS132	Os_Chr01	6710630	6710653	(GA)12	(GA)12	(GA)12	(GA)12	(GA)8	(GA)12	(GA)12	(GA)12	(GA)12	(GA)12	(GA)12	-	Introns/Intergenic	-	-	CTGGAGTGGTTTCTCTCCTCTGC	TACTCTGCTCGCGTAACTTCTCC	310	P	A	A	RM10404
PMS133	Os_Chr01	6805749	6805764	(TA)8	(TA)8	(TA)8	(TA)7	(TA)8	(TA)8	(TA)8	(TA)8	(TA)8	(TA)8	(TA)8	-	Introns/Intergenic	-	-	GGCCGGGTAAAAATACCATT	TACCATTTCCGAAGCTCCAC	242	P	A	A	NA
PMS134	Os_Chr01	6823453	6823482	(CT)15	(CT)15	(CT)15	(CT)15	(CT)12	(CT)15	(CT)15	(CT)15	(CT)15	(CT)15	(CT)12	-	Introns/Intergenic	-	-	GAGAATAACTTACGGGTCATAGGC	ACTGAAGAGCTAAATCCAGTGC	247	P	A	A	RM10406
PMS135	Os_Chr01	6845558	6845587	(AT)15	(AT)15	(AT)8	(AT)15	(AT)10	(AT)6	(AT)15	(AT)15	(AT)13	(AT)15	(AT)15	-	Introns/Intergenic	-	-	TCTAGCATTGCCCACATTCA	TCAATTTCAACAATGGTGCC	148	P	A	A	NA
PMS136	Os_Chr01	6915153	6915190	(AT)19	(AT)19	(AT)6	(AT)19	(AT)19	(AT)19	(AT)19	(AT)16	(AT)19	(AT)19	(AT)19	-	Introns/Intergenic	-	-	GCAGTTTGGTGACCTTGATTTGC	AACGGCCTCGACATCATATAACC	166	P	A	A	RM10412
PMS137	Os_Chr01	7009228	7009245	(CGG)6	(CGG)5	(CGG)6	(CGG)6	(CGG)6	(CGG)5	(CGG)6	(CGG)6	(CGG)5	(CGG)6	(CGG)5	LOC_Os01g12690	CDS	plant-specific domain TIGR01568 family protein, expressed	CODON_CHANGE_PLUS_CODON_DELETION	ACTGGTGGTAGCTGCCAATG	ACTCCCTGCCGCAGTAGTC	276	P	A	A	NA
PMS138	Os_Chr01	7038568	7038585	(CGG)6	(CGG)5	(CGG)6	(CGG)6	(CGG)6	(CGG)6	(CGG)6	(CGG)6	(CGG)6	(CGG)6	(CGG)6	LOC_Os01g12720	CDS	protein kinase domain containing protein, expressed	CODON_DELETION	CGAGCAACAACTCGTCGTC	GTGCCGCTTCTTCTTCTTGA	236	P	A	A	NA
PMS139	Os_Chr01	7056281	7056304	(TATG)6	(TATG)5	(TATG)6	(TATG)6	(TATG)6	(TATG)5	(TATG)6	(TATG)5	(TATG)5	(TATG)6	(TATG)5	LOC_Os01g12750	DRR	cytochrome P450, putative, expressed	-	ATTTGTGTGACTGCGTTGATGC	TGAGACGACGAAGGAAGTAATAGGC	254	P	A	A	RM10423
PMS140	Os_Chr01	7061444	7061464	(TCG)7	(TCG)7	(TCG)7	(TCG)7	(TCG)7	(TCG)7	(TCG)7	(TCG)5	(TCG)7	(TCG)7	(TCG)7	LOC_Os01g12760	CDS	cytochrome P450, putative, expressed	CODON_DELETION	GTACTTACGCGGCCACCAAACG	CCTCCTCATTCCACCGTCACC	202	A	A	A	RM10425
PMS141	Os_Chr01	7072176	7072196	(GCG)7	(GCG)7	(GCG)7	(GCG)7	(GCG)7	(GCG)7	(GCG)7	(GCG)7	(GCG)7	(GCG)7	(GCG)6	LOC_Os01g12770	CDS	cytochrome P450, putative, expressed	CODON_CHANGE_PLUS_CODON_DELETION	TCCTCATCGCTTACTCCAACACC	AGTCGTCGAACACCTTCCTCAGC	114	A	A	A	RM10427
PMS142	Os_Chr01	7076918	7076941	(GCC)8	(GCC)8	(GCC)8	(GCC)8	(GCC)8	(GCC)8	(GCC)8	(GCC)5	(GCC)5	(GCC)8	(GCC)8	-	Introns/Intergenic	-	-	CGAACTTCCTCCGGTATATGTGG	TCACAAGGCCATGATTCTTTCG	180	A	A	A	RM6599

Polymorphic SSR (PMS) markers	Chromosomes	Start physical positions (bp)	End physical positions (bp)	Nipponbare	Kasalath	Nagina22	IR64	Pokkali	Bala	Tainung67	Azucena	Moroberekan	*Oryza rufipogon*	*Oryza nivara*	MSU gene locus IDs	Structural annotation	Functional annotation	SSR functional effects	Forward primers (5'-3')	Reverse primers (5'-3')	Amplified product size (bp)	Markers exhibiting polymorphism within *indica* rice	Transcrption factor genes-derived markers	Known cloned genes-derived markers	PMS markers corresponding with RM markers
PMS143	Os_Chr01	7129561	7129581	(GGT)7	(GGT)7	(GGT)7	(GGT)5	(GGT)7	(GGT)7	(GGT)7	(GGT)7	(GGT)7	(GGT)7	(GGT)7	-	Introns/Intergenic	-	-	GGTACGTGTGCTCCTCTTGATCC	CCATCTAGCACTAGCATTCATTCACC	198	P	A	A	RM10432
PMS144	Os_Chr01	7148379	7148392	(CT)7	(CT)7	(CT)7	(CT)6	(CT)7	(CT)7	(CT)7	(CT)7	(CT)7	(CT)7	(CT)6	-	Introns/Intergenic	-	-	CTTGCGCTTTCATTTTCTCC	CAATGGGAAGGTAGATCGGA	202	P	A	A	NA
PMS145	Os_Chr01	7153083	7153098	(TA)8	(TA)6	(TA)6	(TA)8	(TA)8	(TA)8	(TA)8	(TA)10	(TA)8	(TA)8	(TA)7	-	Introns/Intergenic	-	-	TTTTGCGGGTAGACTGTTTTG	TCCAATGCAATATGCGAAAA	259	P	A	A	NA
PMS146	Os_Chr01	7158635	7158662	(TC)14	(TC)14	(TC)14	(TC)12	(TC)14	(TC)14	(TC)14	(TC)14	(TC)14	(TC)14	(TC)14	-	Introns/Intergenic	-	-	GCTACGTACGAGCCCTAGTTACCG	TACCGCGCCACATATACACAACC	189	P	A	A	RM1201
PMS147	Os_Chr01	7162253	7162274	(TG)11	(TG)11	(TG)11	(TG)11	(TG)11	(TG)11	(TG)11	(TG)11	(TG)11	(TG)11	(TG)9	-	Introns/Intergenic	-	-	TACTCAGCGCAGAAGATCAATGC	TATCATGCTCAGCTGGAGAAACC	401	A	A	A	RM10437
PMS148	Os_Chr01	7176123	7176140	(CGG)6	(CGG)5	(CGG)6	(CGG)6	(CGG)6	(CGG)5	(CGG)6	(CGG)6	(CGG)6	(CGG)6	(CGG)6	LOC_Os01g12920	CDS	thioesterase family protein, putative, expressed	CODON_DELETION	AGAGCTTTGCTCGGAGAGTG	AGCCTTCCTCTCTGCTCCTC	174	P	A	A	NA
PMS149	Os_Chr01	7177580	7177594	(GCC)5	(GCC)6	(GCC)5	(GCC)5	(GCC)5	(GCC)5	(GCC)5	(GCC)5	(GCC)5	(GCC)5	(GCC)5	LOC_Os01g12930	CDS	U-box domain containing protein, expressed	CODON_INSERTION	TTCCAAACCTGAATTCTGCC	GCTCGAACTACAGCCTGGTC	253	P	A	A	NA
PMS150	Os_Chr01	7217989	7218000	(AT)6	(AT)6	(AT)6	(AT)6	(AT)6	(AT)6	(AT)6	(AT)6	(AT)7	(AT)6	(AT)6	-	Introns/Intergenic	-	-	TGATCAGCTGAAGCATGGAG	TTCCAGGTATGGACCGTGAT	195	A	A	A	NA
PMS151	Os_Chr01	7243899	7243928	(CT)15	(CT)14	(CT)15	(CT)15	(CT)15	(CT)14	(CT)15	(CT)15	(CT)15	(CT)15	(CT)15	-	Introns/Intergenic	-	-	GGGACTACTCGAGCAAGCTAATGC	GTCCAATCTAATCGACCTCCAAGAGC	396	P	A	A	RM8077,RM1220
PMS152	Os_Chr01	7250339	7250365	(TGG)9	(TGG)9	(TGG)9	(TGG)9	(TGG)9	(TGG)9	(TGG)5	(TGG)9	(TGG)9	(TGG)9	(TGG)9	-	Introns/Intergenic	-	-	CTGCGAAGTAGGTGCCCTTTGC	TCCTTCAAACTCATCACCCATTCG	161	A	A	A	RM5989
PMS153	Os_Chr01	7294418	7294431	(AT)7	(AT)7	(AT)7	(AT)7	(AT)7	(AT)7	(AT)7	(AT)6	(AT)6	(AT)7	(AT)7	-	Introns/Intergenic	-	-	TCGGATTGAAAATGGTCACA	TCGGAAGGTCAGGAATTGAC	182	A	A	A	NA
PMS154	Os_Chr01	7340948	7340965	(TCG)6	(TCG)6	(TCG)6	(TCG)6	(TCG)6	(TCG)6	(TCG)6	(TCG)6	(TCG)6	(TCG)6	(TCG)5	LOC_Os01g13170	CDS	ubiquitin-conjugating enzyme E2, putative, expressed	CODON_DELETION	GGCGTTGTTTTGGGACTTAT	CAAGATCAAACTCGGGGAAA	257	A	A	A	NA
PMS155	Os_Chr01	7348906	7348931	(GA)13	(GA)15	(GA)13	(GA)13	(GA)13	(GA)13	(GA)13	(GA)13	(GA)13	(GA)13	(GA)13	-	Introns/Intergenic	-	-	CACGGTGAGATAGAGACGAGATGG	CAATCACAAATTCACGCAGTGACC	91	P	A	A	RM3598

Polymorphic SSR (PMS) markers	Chromosomes	Start physical positions (bp)	End physical positions (bp)	Nipponbare	Kasalath	Nagina22	IR64	Pokkali	Bala	Tainung67	Azucena	Moroberekan	*Oryza rufipogon*	*Oryza nivara*	MSU gene locus IDs	Structural annotation	Functional annotation	SSR functional effects	Forward primers (5'-3')	Reverse primers (5'-3')	Amplified product size (bp)	Markers exhibiting polymorphism within *indica* rice	Transcrption factor genes-derived markers	Known cloned genes-derived markers	PMS markers corresponding with RM markers
PMS156	Os_Chr01	7465097	7465108	(CA)6	(CA)7	(CA)6	(CA)6	(CA)6	(CA)7	(CA)6	(CA)6	(CA)6	(CA)6	(CA)6	-	Introns/Intergenic	-	-	GATGTTCTCCTCGT GGTGGT	ACCTATGGCGGTTCA ATCTG	278	P	A	A	NA
PMS157	Os_Chr01	7476779	7476802	(GA)12	(GA)12	(GA)11	(GA)12	(GA)12	(GA)12	(GA)12	(GA)12	(GA)12	(GA)12	(GA)12	-	Introns/Intergenic	-	-	GACCGATCGTGAGC TAGGTAGGG	CTCGCTCTCGCACAA ATCAATCC	181	P	A	A	RM10471
PMS158	Os_Chr01	7497964	7497984	(CAA)7	(CAA)8	(CAA)8	(CAA)7	(CAA)7	(CAA)8	(CAA)7	(CAA)7	(CAA)7	(CAA)7	(CAA)8	-	Introns/Intergenic	-	-	GCCAATCAGATTCC ACCCTAAGC	CTTGCTGCACTGTGA GAATTGC	183	P	A	A	RM10472
PMS159	Os_Chr01	7519734	7519751	(CGC)6	(CGC)6	(CGC)6	(CGC)6	(CGC)5	(CGC)6	(CGC)6	(CGC)6	(CGC)6	(CGC)6	(CGC)6	LOC_Os01g13490	URR	expressed protein	-	CGTTGCTGTACCAA CGACAC	GTAGGCGCAGGCGT AGAG	175	P	A	A	NA
PMS160	Os_Chr01	7547412	7547429	(TTG)6	(TTG)6	(TTG)6	(TTG)5	(TTG)5	(TTG)6	(TTG)6	(TTG)6	(TTG)6	(TTG)6	(TTG)5	-	Introns/Intergenic	-	-	CGGGTGTTCTACTT CCTCCA	GTACGGCACCTGGA ACATCT	216	P	A	A	NA
PMS161	Os_Chr01	7673766	7673793	(AC)14	(AC)14	(AC)14	(AC)13	(AC)13	(AC)13	(AC)14	(AC)14	(AC)14	(AC)14	(AC)14	-	Introns/Intergenic	-	-	CATCATCGCAAATT CACCCTAGC	GCTGAACCAGATGG GTATATCAGC	186	P	A	A	RM10483
PMS162	Os_Chr01	7693033	7693059	(GCG)9	(GCG)9	(GCG)9	(GCG)9	(GCG)9	(GCG)9	(GCG)9	(GCG)8	(GCG)9	(GCG)9	(GCG)9	LOC_Os01g13740	CDS	myb-like DNA-binding domain containing protein, expressed	CODON_DELETION	CGTCTTTCTCCTCC GGCAACG	AGGAGCCAAGGAAG ACGTGAAGG	143	A	P	A	RM10485
PMS163	Os_Chr01	7696284	7696331	(TA)24	(TA)24	(TA)24	(TA)24	(TA)24	(TA)24	(TA)7	(TA)16	(TA)24	(TA)24	(TA)14	-	Introns/Intergenic	-	-	TGAGTACACCGATT TAAGTCGAAA	TAGTACGTACGCCTC CCTCC	272	A	A	A	NA
PMS164	Os_Chr01	7847940	7847957	(CCT)6	(CCT)6	(CCT)6	(CCT)6	(CCT)5	(CCT)6	(CCT)6	(CCT)6	(CCT)6	(CCT)5	(CCT)6	LOC_Os01g14010	CDS	ZOS1-05 - C2H2 zinc finger protein, expressed	CODON_DELETION	CTTTCGCTCCTCTC ACCAAC	AACACAGCAAAATGG GGAAG	140	P	P	A	NA
PMS165	Os_Chr01	7870569	7870628	(TTA)20	(TTA)20	(TTA)6	(TTA)20	(TTA)5	(TTA)20	(TTA)20	(TTA)20	(TTA)13	(TTA)20	(TTA)20	-	Introns/Intergenic	-	-	AGGGTACGTGAAGA TAGTACCG	CGATAGTTCGAGAG GTAATTCG	129	P	A	A	RM6685
PMS166	Os_Chr01	7904763	7904783	(GGT)7	(GGT)7	(GGT)7	(GGT)7	(GGT)7	(GGT)7	(GGT)5	(GGT)7	(GGT)7	(GGT)7	(GGT)7	-	Introns/Intergenic	-	-	GTGGTGGTGATTAG CGGAGT	AAATTCCACACCAAA CCGAA	113	A	A	A	NA
PMS167	Os_Chr01	7927455	7927481	(CGG)9	(CGG)9	(CGG)9	(CGG)9	(CGG)9	(CGG)9	(CGG)9	(CGG)9	(CGG)8	(CGG)9	(CGG)9	LOC_Os01g14130	CDS	expressed protein	CODON_DELETION	CAGATGAGCCGTAC ATGAATACG	CTGGGCTATAGTCTT CCAGTGC	97	A	A	A	RM10502
PMS168	Os_Chr01	7974817	7974842	(GA)13	(GA)13	(GA)13	(GA)13	(GA)7	(GA)13	(GA)13	(GA)13	(GA)13	(GA)13	(GA)13	LOC_Os01g14220	URR	expressed protein	-	CAGACTGCAGTTGC ACGATACTACG	GAAAGCTGCAACGAT GTTGTCC	89	P	A	A	RM10505

Polymorphic SSR (PMS) markers	Chromosomes	Start physical positions (bp)	End physical positions (bp)	Nipponbare	Kasalath	Nagina22	IR64	Pokkali	Bala	Tainung67	Azucena	Moroberekan	*Oryza rufipogon*	*Oryza nivara*	MSU gene locus IDs	Structural annotation	Functional annotation	SSR functional effects	Forward primers (5'-3')	Reverse primers (5'-3')	Amplified product size (bp)	Markers exhibiting polymorphism within *indica* rice	Transcrption factor genes-derived markers	Known cloned genes-derived markers	PMS markers corresponding with RM markers
PMS169	Os_Chr01	7978479	7978498	(TA)10	(TA)10	(TA)10	(TA)10	(TA)6	(TA)10	(TA)10	(TA)10	(TA)10	(TA)10	(TA)10	-	Introns/Intergenic	-	-	GCAGAAATAACAAAATGCTAATGG	AATTTATGCACTAAACTTTCGAATTA	249	P	A	A	NA
PMS170	Os_Chr01	8153138	8153158	(CGC)7	(CGC)7	(CGC)6	(CGC)5	(CGC)7	(CGC)7	(CGC)7	(CGC)7	(CGC)7	(CGC)7	(CGC)7	LOC_Os01g14540	DRR	STIGL1 - Stigma-specific Stig1 family protein precursor, expressed	-	TCTCTGATGGATGTGTAGGAACG	GTACTACGACTGCAAGAGGAAGC	388	P	A	A	RM10522
PMS171	Os_Chr01	8160373	8160393	(TAA)7	(TAA)11	(TAA)7	(TAA)7	(TAA)7	(TAA)11	(TAA)7	(TAA)7	(TAA)7	(TAA)7	(TAA)7	-	Introns/Intergenic	-	-	GGAGATGGAGCTCGTCCACAAGG	TTCAGAAACCTCATCCCTGACACG	195	P	A	A	RM10523
PMS172	Os_Chr01	8176974	8176991	(AT)9	(AT)9	(AT)9	(AT)9	(AT)9	(AT)9	(AT)6	(AT)9	(AT)9	(AT)9	(AT)9	-	Introns/Intergenic	-	-	ATTGTCCGGGTCATAGATGC	TGAATAAGACAAATGGTCAAACG	217	A	A	A	NA
PMS173	Os_Chr01	8242898	8242927	(TGG)10	(TGG)7	(TGG)10	(TGG)7	(TGG)6	(TGG)10	(TGG)5	(TGG)6	(TGG)10	(TGG)8	(TGG)8	-	Introns/Intergenic	-	-	TTCATGTTTCGACATCGAGGAACC	AGCTGGTCAGTCCTATCCGAAACC	355	P	A	A	RM10525
PMS174	Os_Chr01	8343300	8343311	(CT)6	(CT)8	(CT)8	(CT)6	(CT)6	(CT)8	(CT)6	(CT)6	(CT)6	(CT)6	(CT)6	-	Introns/Intergenic	-	-	CCAAGATCCTACGGACGAAA	CAGAGAGAGGGATCGAGTGG	152	P	A	A	NA
PMS175	Os_Chr01	8347166	8347177	(GA)6	(GA)7	(GA)7	(GA)6	(GA)6	(GA)7	(GA)6	(GA)6	(GA)6	(GA)6	(GA)7	-	Introns/Intergenic	-	-	AAGAGAGAGGGAGGAGGACG	AACCATCCTGACGGTTCAAG	225	P	A	A	NA
PMS176	Os_Chr01	8411194	8411233	(AG)20	(AG)20	(AG)20	(AG)20	(AG)6	(AG)20	(AG)20	(AG)20	(AG)20	(AG)20	(AG)20	-	Introns/Intergenic	-	-	AGATATACACGGCAATCCGATCC	GTAGGGTTTGAAAGCTTGAAGTGC	297	P	A	A	RM578
PMS177	Os_Chr01	8673214	8673225	(GA)6	(GA)7	(GA)7	(GA)6	(GA)6	(GA)7	(GA)6	(GA)6	(GA)6	(GA)7	(GA)7	-	Introns/Intergenic	-	-	ACTACGCCACATTGTCCTCC	CTAAACATGCCCTTTGCTGC	160	P	A	A	NA
PMS178	Os_Chr01	8680150	8680170	(CCG)7	(CCG)7	(CCG)7	(CCG)7	(CCG)7	(CCG)7	(CCG)6	(CCG)7	(CCG)7	(CCG)7	(CCG)7	-	Introns/Intergenic	-	-	CACTCCATATGCCACCCTCTTCC	CACCGAGGCACGACGATAGC	170	A	A	A	RM10550
PMS179	Os_Chr01	8697989	8698006	(AG)9	(AG)9	(AG)9	(AG)9	(AG)9	(AG)9	(AG)9	(AG)9	(AG)9	(AG)10	(AG)9	-	Introns/Intergenic	-	-	TGGGATGGAAGGAGTACCAG	GGAGACCTAAAGGGCTTGCT	263	A	A	A	NA
PMS180	Os_Chr01	8723187	8723228	(TTA)14	(TTA)14	(TTA)14	(TTA)5	(TTA)14	(TTA)14	(TTA)14	(TTA)14	(TTA)14	(TTA)14	(TTA)9	-	Introns/Intergenic	-	-	CCTAACCTTGGTGTGCGAGAGC	TCTCCAGTTTGTTCCCTGAGAGC	388	P	A	A	RM10556
PMS181	Os_Chr01	8745803	8745818	(TA)8	(TA)8	(TA)8	(TA)8	(TA)8	(TA)8	(TA)8	(TA)8	(TA)8	(TA)8	(TA)7	-	Introns/Intergenic	-	-	GAGAAGCAAAACCCAGCAGT	GGGATTTCGACTTCCTCACT	237	A	A	A	NA

Polymorphic SSR (PMS) markers	Chromosomes	Start physical positions (bp)	End physical positions (bp)	Nipponbare	Kasalath	Nagina22	IR64	Pokkali	Bala	Tainung67	Azucena	Moroberekan	*Oryza rufipogon*	*Oryza nivara*	MSU gene locus IDs	Structural annotation	Functional annotation	SSR functional effects	Forward primers (5'-3')	Reverse primers (5'-3')	Amplified product size (bp)	Markers exhibiting polymorphism within *indica* rice	Transcrption factor genes-derived markers	Known cloned genes-derived markers	PMS markers corresponding with RM markers
PMS182	Os_Chr01	8751674	8751697	(CGA)8	(CGA)8	(CGA)8	(CGA)8	(CGA)8	(CGA)8	(CGA)6	(CGA)8	(CGA)8	(CGA)8	(CGA)8	LOC_Os01g15580	CDS	disease resistance protein RGA2, putative, expressed	CODON_DELETION	ACTCATGCGCGAGACCATCTCC	TCCAGGTTGGTGGTGGTTGTGG	351	A	A	A	RM10559
PMS183	Os_Chr01	8759468	8759485	(GCG)6	(GCG)6	(GCG)6	(GCG)6	(GCG)6	(GCG)6	(GCG)5	(GCG)6	(GCG)6	(GCG)6	(GCG)6	LOC_Os01g15600	URR	PUR ALPHA-1, putative, expressed	-	GCGAGTTTAAACCCAAACCC	GATCTTCAGGTACCTCCCCC	249	A	A	A	NA
PMS184	Os_Chr01	8845987	8846066	(TA)40	(TA)40	(TA)40	(TA)7	(TA)40	(TA)40	(TA)6	(TA)9	(TA)40	(TA)6	(TA)40	-	Introns/Intergenic	-	-	AATTCACACCACCTGTTCATCC	CTTCCTCTTCATCCGATAGTTTGC	175	P	A	A	RM10564
PMS185	Os_Chr01	8913424	8913437	(TG)7	(TG)8	(TG)7	(TG)7	(TG)7	(TG)7	(TG)7	(TG)8	(TG)8	(TG)8	(TG)8	-	Introns/Intergenic	-	-	TGCCTAATTTTGATGATTGAGAGA	GGGGTTATGGAATCGGTTTT	235	P	A	A	NA
PMS186	Os_Chr01	8925121	8925144	(GGC)8	(GGC)6	(GGC)8	(GGC)8	(GGC)8	(GGC)8	(GGC)6	(GGC)8	(GGC)8	(GGC)8	(GGC)8	LOC_Os01g15840	CDS	PPR repeat containing protein, expressed	CODON_DELETION	AGGGGAGAAGAGGAAGATGC	GGTCTTCTCAACGTGCTGC	277	P	A	A	NA
PMS187	Os_Chr01	8952380	8952400	(CCG)7	(CCG)7	(CCG)7	(CCG)7	(CCG)7	(CCG)7	(CCG)7	(CCG)7	(CCG)7	(CCG)7	(CCG)5	LOC_Os01g15900	CDS	RDD1	CODON_DELETION	AATGCGCACGCGATTATTCTCACC	TGACAAGGATCCGGGCATAAAGC	168	A	P	P	RM10571
PMS188	Os_Chr01	9066580	9066603	(CGG)8	(CGG)11	(CGG)8	(CGG)8	(CGG)8	(CGG)8	(CGG)8	(CGG)8	(CGG)8	(CGG)8	(CGG)8	LOC_Os01g16090	CDS	RNA binding protein, putative, expressed	CODON_INSERTION	CAGCCTCACGGGACATCATCC	CACAGCCGTCGAAACCCTAGC	274	P	A	A	RM10575
PMS189	Os_Chr01	9107684	9107707	(GA)12	(GA)12	(GA)12	(GA)12	(GA)12	(GA)12	(GA)12	(GA)11	(GA)12	(GA)12	(GA)12	-	Introns/Intergenic	-	-	ATGCGTGATCAACAATCGAGAACG	CCATAGCCGATGGATTGAAAGTGG	175	A	A	A	RM581
PMS190	Os_Chr01	9117039	9117053	(CGG)5	(CGG)6	(CGG)5	(CGG)5	(CGG)5	(CGG)6	(CGG)5	(CGG)5	(CGG)5	(CGG)5	(CGG)5	LOC_Os01g16146	CDS	phenazine biosynthesis protein, putative, expressed	CODON_INSERTION	ATGAACACAGGGGAGACGAG	GAGTTCAACCTCTCCGAGACC	170	P	A	A	NA
PMS191	Os_Chr01	9121247	9121264	(CGC)6	(CGC)6	(CGC)6	(CGC)6	(CGC)6	(CGC)6	(CGC)5	(CGC)6	(CGC)6	(CGC)6	(CGC)6	LOC_Os01g16152	CDS	peroxiredoxin, putative, expressed	CODON_DELETION	AGAGGGGTGGTGGAGAAGTT	CTGAGGAAGGCGACGGTAG	176	A	A	A	NA
PMS192	Os_Chr01	9181929	9181960	(GA)16	(GA)16	(GA)16	(GA)16	(GA)16	(GA)16	(GA)16	(GA)8	(GA)16	(GA)16	(GA)16	LOC_Os01g16220	URR	Sad1 / UNC-like C-terminal domain containing protein, putative, expressed	-	CTGGTCGAGCAGGAGGAC	CAAAATTTCCCAAATTCCCC	273	A	A	A	NA
PMS193	Os_Chr01	9192223	9192246	(CGG)8	(CGG)9	(CGG)6	(CGG)8	(CGG)8	(CGG)8	(CGG)8	(CGG)8	(CGG)8	(CGG)8	(CGG)8	-	Introns/Intergenic	-	-	TGCTGCTGCTGCTTTACATTGC	CACCTCCTCGAACGAGAACTCC	188	P	A	A	RM6466
PMS194	Os_Chr01	9367672	9367689	(ATG)6	(ATG)5	(ATG)6	(ATG)5	(ATG)5	(ATG)5	(ATG)6	(ATG)6	(ATG)6	(ATG)6	(ATG)6	-	Introns/Intergenic	-	-	TTCTCTATTGCTTCGGGTCG	CTTGAGAACCTGAACGTCGC	223	P	A	A	NA

Polymorphic SSR (PMS) markers	Chromosomes	Start physical positions (bp)	End physical positions (bp)	Nipponbare	Kasalath	Nagina22	IR64	Pokkali	Bala	Tainung67	Azucena	Moroberekan	*Oryza rufipogon*	*Oryza nivara*	MSU gene locus IDs	Structural annotation	Functional annotation	SSR functional effects	Forward primers (5'-3')	Reverse primers (5'-3')	Amplified product size (bp)	Markers exhibiting polymorphism within *indica* rice	Transcrption factor genes-derived markers	Known cloned genes-derived markers	PMS markers corresponding with RM markers
PMS195	Os_Chr01	9464648	9464704	(TTA)19	(TTA)16	(TTA)15	(TTA)12	(TTA)19	(TTA)18	(TTA)19	(TTA)19	(TTA)19	(TTA)19	(TTA)19	-	Introns/Intergenic	-	-	CCTGTTAGGTGGCACATTTATGG	TCGGCCCTGTATATCATTACTCG	296	P	A	A	RM600
PMS196	Os_Chr01	9545986	9545999	(AG)7	(AG)7	(AG)7	(AG)7	(AG)7	(AG)7	(AG)7	(AG)7	(AG)7	(AG)7	(AG)6	LOC_Os01g16800	CDS	expressed protein	FRAME_SHIFT	AGAGAGCTTCATGAGCCTGG	ATGAGGAGGTGTAGGAGGGG	256	A	A	A	NA
PMS197	Os_Chr01	9567143	9567158	(AC)8	(AC)8	(AC)8	(AC)8	(AC)8	(AC)9	(AC)8	(AC)8	(AC)8	(AC)9	(AC)8	-	Introns/Intergenic	-	-	CAGTGACCACTGAGCAAGGA	GCTGCTTTTGCTCCAGGTTA	200	P	A	A	NA
PMS198	Os_Chr01	9568372	9568389	(GCG)6	(GCG)5	(GCG)6	(GCG)6	(GCG)6	(GCG)5	(GCG)6	(GCG)6	(GCG)6	(GCG)6	(GCG)6	LOC_Os01g16810	CDS	CSA	CODON_DELETION	CTCTGGTAGAAGCAGCAGGG	CAGTACAGCGGGTACAGCG	143	P	P	P	NA
PMS199	Os_Chr01	9588771	9588788	(AG)9	(AG)8	(AG)9	(AG)9	(AG)8	(AG)9	(AG)9	(AG)9	(AG)9	(AG)9	(AG)9	-	Introns/Intergenic	-	-	TGGAACGATATGACGGAAAA	ATGGAAACGCTCTCTCATGG	257	P	A	A	NA
PMS200	Os_Chr01	9597344	9597364	(GAG)7	(GAG)5	(GAG)7	(GAG)7	(GAG)7	(GAG)5	(GAG)7	(GAG)7	(GAG)7	(GAG)7	(GAG)7	-	Introns/Intergenic	-	-	AGGGAAAGAAGGAGAGGAGTTATAGG	CATGTGCGGTGAAGAGTACG	132	P	A	A	RM10603
PMS201	Os_Chr01	9606747	9606803	(CTT)19	(CTT)19	(CTT)19	(CTT)19	(CTT)19	(CTT)19	(CTT)19	(CTT)9	(CTT)19	(CTT)19	(CTT)19	-	Introns/Intergenic	-	-	GAAATGGACTCGCTCCTAAACTGG	ACGAACTAGAGCATGGGCACTCC	249	A	A	A	RM8070,RM8067,RM580
PMS202	Os_Chr01	9638885	9638900	(CT)8	(CT)8	(CT)8	(CT)8	(CT)8	(CT)8	(CT)8	(CT)8	(CT)8	(CT)9	(CT)9	LOC_Os01g16870	URR	argonaute, putative, expressed	-	GATTTCCCTCCACCCAAAAT	AAGTGCCGGTTTTACCCTTC	223	A	A	A	NA
PMS203	Os_Chr01	9709515	9709535	(GGA)7	(GGA)7	(GGA)5	(GGA)7	(GGA)7	(GGA)7	(GGA)7	(GGA)7	(GGA)7	(GGA)7	(GGA)7	LOC_Os01g16970	CDS	glucosidase II beta subunit-like domain containing protein, expressed	CODON_DELETION	CAAATCAAGAACCGCATCAAGC	CCTGCTACACTTCCCGTTTCC	281	P	A	A	RM10607
PMS204	Os_Chr01	9863469	9863486	(GGC)6	(GGC)6	(GGC)5	(GGC)6	(GGC)6	(GGC)6	(GGC)6	(GGC)6	(GGC)6	(GGC)6	(GGC)6	-	Introns/Intergenic	-	-	AGCCAGACCAGACCAGAGAG	GACGTCAGCATCTCCATGTG	266	P	A	A	NA
PMS205	Os_Chr01	9894675	9894690	(CT)8	(CT)8	(CT)8	(CT)9	(CT)8	(CT)9	(CT)8	(CT)8	(CT)8	(CT)8	(CT)8	-	Introns/Intergenic	-	-	CCTTTGAAACGGCTTCTCTG	TTCCTCAGCCAATTGTTGGT	189	P	A	A	NA
PMS206	Os_Chr01	10030117	10030134	(AT)9	(AT)9	(AT)6	(AT)9	(AT)13	(AT)9	(AT)9	(AT)9	(AT)9	(AT)9	(AT)9	-	Introns/Intergenic	-	-	GGGGTCAGAAATGATCGAAA	TTGTTAGACATGTTTTCCAAACAAA	172	P	A	A	NA
PMS207	Os_Chr01	10132363	10132378	(CA)8	(CA)8	(CA)8	(CA)8	(CA)8	(CA)8	(CA)6	(CA)8	(CA)8	(CA)8	(CA)8	-	Introns/Intergenic	-	-	ATATCGGATCAGATCGCCAC	CCGTTTCTTTTTCTCCTCCC	197	A	A	A	NA

Polymorphic SSR (PMS) markers	Chromosomes	Start physical positions (bp)	End physical positions (bp)	Nipponbare	Kasalath	Nagina22	IR64	Pokkali	Bala	Tainung67	Azucena	Moroberekan	*Oryza rufipogon*	*Oryza nivara*	MSU gene locus IDs	Structural annotation	Functional annotation	SSR functional effects	Forward primers (5'-3')	Reverse primers (5'-3')	Amplified product size (bp)	Markers exhibiting polymorphism within *indica* rice	Transcrption factor genes-derived markers	Known cloned genes-derived markers	PMS markers corresponding with RM markers
PMS208	Os_Chr01	10140014	10140034	(CCG)7	(CCG)7	(CCG)7	(CCG)5	(CCG)7	(CCG)7	(CCG)7	(CCG)7	(CCG)7	(CCG)7	(CCG)7	LOC_Os01g18100	CDS	OsRhmbd3 - Putative Rhomboid homologue, expressed	CODON_DELETION	AATGATGAGGTGAGGGTTGAAGG	CAGTCAGTGACCCAGTCAATCTCC	280	P	A	A	RM10637
PMS209	Os_Chr01	10172181	10172210	(TA)15	(TA)15	(TA)15	(TA)8	(TA)15	(TA)15	(TA)15	(TA)15	(TA)10	(TA)15	(TA)15	-	Introns/Intergenic	-	-	GAGGCGCTTCTGCTATGTATCG	TGCATCCAGAACCCTAAATCAGC	270	P	A	A	RM10638
PMS210	Os_Chr01	10214241	10214258	(CTC)6	(CTC)5	(CTC)6	(CTC)6	(CTC)6	(CTC)5	(CTC)6	(CTC)6	(CTC)6	(CTC)6	(CTC)6	-	Introns/Intergenic	-	-	ACTTCTCGCTCTCGGTGAGT	GCGGGAAAATTGAGAACTGA	230	P	A	A	NA
PMS211	Os_Chr01	10343418	10343438	(CCG)7	(CCG)7	(CCG)7	(CCG)7	(CCG)7	(CCG)7	(CCG)7	(CCG)7	(CCG)7	(CCG)6	(CCG)7	LOC_Os01g18400	CDS	CHIT10 - Chitinase family protein precursor, expressed	CODON_DELETION	CGAAGGACGCGTAGGTGTAGAACC	CTCGCGCATATCACAGTCACAGC	277	A	A	A	RM10654
PMS212	Os_Chr01	10399893	10399906	(CT)7	(CT)7	(CT)7	(CT)7	(CT)7	(CT)7	(CT)7	(CT)7	(CT)7	(CT)7	(CT)6	-	Introns/Intergenic	-	-	TGCCACCATGCAGAACTACT	CGAGCAAGATGGCTAGTCG	251	A	A	A	NA
PMS213	Os_Chr01	10474189	10474226	(CA)19	(CA)17	(CA)19	(CA)19	(CA)19	(CA)17	(CA)19	(CA)19	(CA)19	(CA)19	(CA)19	-	Introns/Intergenic	-	-	GATCTCAACACACATGCAAACTGG	CTAGCGTATGCAAGGAAGGATGC	321	P	A	A	RM10662
PMS214	Os_Chr01	10526557	10526572	(GT)8	(GT)8	(GT)8	(GT)6	(GT)8	(GT)6	(GT)8	(GT)8	(GT)8	(GT)8	(GT)8	-	Introns/Intergenic	-	-	CATCGAGCAGAAAACAACCA	TACGCATCCACCGTAACAAA	267	P	A	A	NA
PMS215	Os_Chr01	10532605	10532620	(TA)8	(TA)11	(TA)8	(TA)8	(TA)8	(TA)8	(TA)8	(TA)8	(TA)8	(TA)8	(TA)8	-	Introns/Intergenic	-	-	GCCACTCAAAATTCCCACAT	TTTTGGAAGTTGGCCTCAAT	204	P	A	A	NA
PMS216	Os_Chr01	10534973	10534990	(TA)9	(TA)9	(TA)9	(TA)9	(TA)9	(TA)9	(TA)9	(TA)6	(TA)9	(TA)9	(TA)9	-	Introns/Intergenic	-	-	ACGAATCTGTGCCTGAGGAT	ACGGTCTCACCTCCATTCAT	244	A	A	A	NA
PMS217	Os_Chr01	10551899	10551913	(CCT)5	(CCT)6	(CCT)5	(CCT)5	(CCT)5	(CCT)5	(CCT)5	(CCT)5	(CCT)5	(CCT)5	(CCT)6	LOC_Os01g18720	CDS	expressed protein	CODON_CHANGE_PLUS_CODON_INSERTION	TAACCCTAGCTCACCGATGG	CTCTCTTCCTCCCCTCCAAG	278	P	A	A	NA
PMS218	Os_Chr01	10569265	10569290	(TA)13	(TA)13	(TA)6	(TA)13	(TA)13	(TA)13	(TA)13	(TA)13	(TA)13	(TA)13	(TA)13	-	Introns/Intergenic	-	-	GAACCCAGGACCTCATGTGC	TTATCCGACTTGATTCCTCTCTCC	346	P	A	A	RM10668
PMS219	Os_Chr01	10585823	10585840	(GCC)6	(GCC)5	(GCC)6	(GCC)6	(GCC)6	(GCC)6	(GCC)6	(GCC)6	(GCC)6	(GCC)6	(GCC)6	-	Introns/Intergenic	-	-	CCCCTGCTCTCTCTCTCTCTC	TGAAGCTAGCTGTCGCCG	280	P	A	A	NA
PMS220	Os_Chr01	10778068	10778083	(GA)8	(GA)8	(GA)8	(GA)8	(GA)8	(GA)7	(GA)8	(GA)8	(GA)8	(GA)8	(GA)8	-	Introns/Intergenic	-	-	GTTCACGTTCTCGGTGTGG	GCCTCCCTCTCGATCTCTCT	245	P	A	A	NA

Polymorphic SSR (PMS) markers	Chromosomes	Start physical positions (bp)	End physical positions (bp)	Nipponbare	Kasalath	Nagina22	IR64	Pokkali	Bala	Tainung67	Azucena	Moroberekan	*Oryza rufipogon*	*Oryza nivara*	MSU gene locus IDs	Structural annotation	Functional annotation	SSR functional effects	Forward primers (5'-3')	Reverse primers (5'-3')	Amplified product size (bp)	Markers exhibiting polymorphism within *indica* rice	Transcrption factor genes-derived markers	Known cloned genes-derived markers	PMS markers corresponding with RM markers
PMS221	Os_Chr01	10822918	10822933	(TC)8	(TC)8	(TC)8	(TC)8	(TC)8	(TC)6	(TC)8	(TC)8	(TC)8	(TC)8	(TC)8	-	Introns/Intergenic	-	-	CTACCTCCTCCTCCGGGTT	GGTTTTTCCAATCCAAAGCA	220	P	A	A	NA
PMS222	Os_Chr01	10829665	10829685	(CCA)7	(CCA)7	(CCA)7	(CCA)7	(CCA)7	(CCA)7	(CCA)6	(CCA)5	(CCA)7	(CCA)7	(CCA)7	-	Introns/Intergenic	-	-	CGAAGAGCAGCCACATAACAAGC	TAGAGACGGTTGGGAGTTGTTGG	200	A	A	A	RM10681
PMS223	Os_Chr01	10869789	10869822	(TA)17	(TA)17	(TA)17	(TA)6	(TA)17	(TA)16	(TA)17	(TA)9	(TA)6	(TA)6	(TA)17	-	Introns/Intergenic	-	-	CTTTCGATCCCTTCCTTCTTTCC	GACAATAATAGGCCGAACGTAAGTGG	468	P	A	A	RM10683
PMS224	Os_Chr01	10947462	10947523	(TA)31	(TA)31	(TA)31	(TA)31	(TA)31	(TA)31	(TA)13	(TA)31	(TA)8	(TA)31	(TA)31	-	Introns/Intergenic	-	-	CCCCTTTAATCTGTTCTCTTATCC	TGTAGTTTTTGGGTTTTTATACACG	271	A	A	A	NA
PMS225	Os_Chr01	10988057	10988077	(ACG)7	(ACG)7	(ACG)7	(ACG)7	(ACG)7	(ACG)7	(ACG)7	(ACG)7	(ACG)7	(ACG)5	(ACG)7	-	Introns/Intergenic	-	-	TCTGACGTGACGAATGACTCAGC	GTCCTCATCCATGGGAGTTGG	97	A	A	A	RM10700
PMS226	Os_Chr01	10988643	10988656	(AG)7	(AG)7	(AG)7	(AG)7	(AG)7	(AG)8	(AG)7	(AG)7	(AG)7	(AG)7	(AG)7	-	Introns/Intergenic	-	-	ATGGCAGCTGGTGGAGAG	CGCGCTTCTGATACTCTGTG	178	P	A	A	NA
PMS227	Os_Chr01	11028835	11028852	(CCA)6	(CCA)6	(CCA)6	(CCA)6	(CCA)6	(CCA)6	(CCA)5	(CCA)6	(CCA)6	(CCA)6	(CCA)6	-	Introns/Intergenic	-	-	AACTCAGGATGTGGTCAGGG	TATGGTGCTAAATCGTTGCG	204	A	A	A	NA
PMS228	Os_Chr01	11059495	11059510	(GT)8	(GT)8	(GT)8	(GT)8	(GT)8	(GT)8	(GT)7	(GT)8	(GT)8	(GT)7	(GT)8	LOC_Os01g19480	URR	kelch motif family protein, putative, expressed	-	AACAGGGACCTCTTGGTGTG	ATACAGCCACATTGCCTTCC	213	A	A	A	NA
PMS229	Os_Chr01	11129256	11129291	(TA)18	(TA)18	(TA)18	(TA)18	(TA)18	(TA)18	(TA)18	(TA)11	(TA)7	(TA)18	(TA)18	-	Introns/Intergenic	-	-	CACGAGCTCCGTCACATACCTATACC	GACTCTCATTCTGGGAAACCATCG	590	A	A	A	RM10708
PMS230	Os_Chr01	11175610	11175663	(AT)27	(AT)15	(AT)27	(AT)7	(AT)27	(AT)8	(AT)27	(AT)8	(AT)8	(AT)27	(AT)27	-	Introns/Intergenic	-	-	TTGACCGATTCTAACCTGTTGACC	GAGTCGGTAGAGCTCAAGAAGTGC	168	P	A	A	RM10712
PMS231	Os_Chr01	11241983	11242004	(AG)11	(AG)9	(AG)9	(AG)10	(AG)9	(AG)11	(AG)11	(AG)11	(AG)11	(AG)11	(AG)11	-	Introns/Intergenic	-	-	CCTTCTTGCCGCCAACCTAGTCC	TTTGGCTGACATGACGCCTACG	320	P	A	A	RM10716
PMS232	Os_Chr01	11356194	11356217	(CGG)8	(CGG)8	(CGG)8	(CGG)7	(CGG)8	(CGG)7	(CGG)8	(CGG)7	(CGG)7	(CGG)7	(CGG)8	LOC_Os01g19970	CDS	MYB family transcription factor, putative, expressed	CODON_DELETION	GAGGACGAAGGAGAAGGACAAGG	CGAGCCCATCTGATGTGAGACC	164	P	P	A	RM10719
PMS233	Os_Chr01	11369463	11369476	(AT)7	(AT)7	(AT)7	(AT)6	(AT)7	(AT)6	(AT)7	(AT)6	(AT)6	(AT)7	(AT)7	-	Introns/Intergenic	-	-	TCTGTACCGTAAACCACGCA	TACGGCGGATTTAATTAGCG	271	P	A	A	NA

**Polymorphic SSR (PMS) markers**	**Chromosomes**	**Start physical positions (bp)**	**End physical positions (bp)**	**Nipponbare**	**Kasalath**	**Nagina22**	**IR64**	**Pokkali**	**Bala**	**Tainung67**	**Azucena**	**Moroberekan**	***Oryza rufipogon***	***Oryza nivara***	**MSU gene locus IDs**	**Structural annotation**	**Functional annotation**	**SSR functional effects**	**Forward primers (5'-3')**	**Reverse primers (5'-3')**	**Amplified product size (bp)**	**Markers exhibiting polymorphism within *indica* rice**	**Transcrption factor genes-derived markers**	**Known cloned genes-derived markers**	**PMS markers corresponding with RM markers**
PMS234	Os_Chr01	11401855	11401868	(AT)7	(AT)7	(AT)7	(AT)7	(AT)7	(AT)7	(AT)7	(AT)6	(AT)7	(AT)7	(AT)7	-	Introns/Intergenic	-	-	TGTATGCTAAGTCTAACAATCTT	GATTAGCCACGATTTAATATGAG	205	A	A	A	NA
PMS235	Os_Chr01	11584082	11584115	(GA)17	(GA)17	(GA)17	(GA)17	(GA)13	(GA)17	(GA)17	(GA)17	(GA)17	(GA)17	(GA)17	-	Introns/Intergenic	-	-	TGATGGATCTCTGAGGTGTAAAGAGC	TGCACTAATCTTTCTGCCACAGC	100	P	A	A	RM3412
PMS236	Os_Chr01	11636574	11636594	(CTT)7	(CTT)7	(CTT)7	(CTT)7	(CTT)7	(CTT)7	(CTT)7	(CTT)7	(CTT)7	(CTT)8	(CTT)7	-	Introns/Intergenic	-	-	AGGTCGCCTGAGTTTCATACCC	TGTGCGGTTTAAGATAGAGTGAGC	196	A	A	A	RM10741
PMS237	Os_Chr01	11662266	11662279	(TA)7	(TA)7	(TA)7	(TA)7	(TA)7	(TA)7	(TA)7	(TA)6	(TA)7	(TA)7	(TA)7	-	Introns/Intergenic	-	-	CAAACTTTCTATGGACGTATACAAAC	CCCCCTCTAGTCTCTCTCATAGG	248	A	A	A	NA
PMS238	Os_Chr01	11769730	11769744	(GAG)5	(GAG)5	(GAG)5	(GAG)5	(GAG)5	(GAG)5	(GAG)5	(GAG)5	(GAG)5	(GAG)8	(GAG)5	-	Introns/Intergenic	-	-	GAAGAAGGAAGAATTGAATGAGAGA	CCTCTCCAAAATCGAAGGAA	187	A	A	A	NA
PMS239	Os_Chr01	11775126	11775153	(AG)14	(AG)13	(AG)14	(AG)14	(AG)14	(AG)13	(AG)14	(AG)14	(AG)14	(AG)14	(AG)14	-	Introns/Intergenic	-	-	CATCGGTGACCACCTTCTCC	CCTGTCATCTATCTCCCTCAAGC	100	P	A	A	RM10748
PMS240	Os_Chr01	11820859	11820883	(ATAAC)5	(ATAAC)5	(ATAAC)5	(ATAAC)5	(ATAAC)6	(ATAAC)5	(ATAAC)5	(ATAAC)5	(ATAAC)5	(ATAAC)5	(ATAAC)5	-	Introns/Intergenic	-	-	GGTACATAACGATGATGTGCAA	GGCACCCGTGTCTTTAGGTA	126	P	A	A	NA
PMS241	Os_Chr01	11905094	11905135	(AT)21	(AT)21	(AT)21	(AT)21	(AT)21	(AT)6	(AT)21	(AT)21	(AT)21	(AT)21	(AT)21	-	Introns/Intergenic	-	-	GGGCCACCTTCCTAGTTCAT	TTCGACGTCTCACCCTTTCT	246	P	A	A	NA
PMS242	Os_Chr01	11918998	11919057	(TA)30	(TA)30	(TA)30	(TA)30	(TA)30	(TA)30	(TA)30	(TA)30	(TA)30	(TA)9	(TA)30	-	Introns/Intergenic	-	-	TGTATCCCGTACGTATCCAAAA	TAGCGCCTTAGGGTGCTGTA	260	A	A	A	NA
PMS243	Os_Chr01	11955176	11955193	(CCG)6	(CCG)6	(CCG)6	(CCG)6	(CCG)6	(CCG)6	(CCG)6	(CCG)5	(CCG)6	(CCG)6	(CCG)6	LOC_Os01g21410	CDS	expressed protein	CODON_DELETION	ACGTGGAAGTCGGAGCAG	ACAAGAAGCCCTGCCTCTC	238	A	A	A	NA
PMS244	Os_Chr01	12074996	12075010	(CGC)5	(CGC)5	(CGC)5	(CGC)5	(CGC)5	(CGC)5	(CGC)5	(CGC)6	(CGC)6	(CGC)5	(CGC)5	LOC_Os01g21580	CDS	esterase/lipase/thioesterase family protein, putative, expressed	CODON_CHANGE_PLUS_CODON_INSERTION	GGAGAACGTCGACAAGGTGT	GACGCACGTACTTGCTTGAA	189	A	A	A	NA
PMS245	Os_Chr01	12106709	12106723	(CCG)5	(CCG)7	(CCG)5	(CCG)5	(CCG)5	(CCG)7	(CCG)5	(CCG)5	(CCG)5	(CCG)5	(CCG)5	-	Introns/Intergenic	-	-	CCTCCTCTCCTAGCGATGG	GAGAAGTACACGACGGAGGC	248	P	A	A	NA
PMS246	Os_Chr01	12141442	12141485	(AT)22	(AT)22	(AT)22	(AT)22	(AT)22	(AT)22	(AT)7	(AT)22	(AT)22	(AT)22	(AT)7	-	Introns/Intergenic	-	-	AATTTGCAGTTGGCATGGTTGG	ATGGATGGGAATCCACGTATAGAGC	152	A	A	A	RM10770

Polymorphic SSR (PMS) markers	Chromosomes	Start physical positions (bp)	End physical positions (bp)	Nipponbare	Kasalath	Nagina22	IR64	Pokkali	Bala	Tainung67	Azucena	Moroberekan	*Oryza rufipogon*	*Oryza nivara*	MSU gene locus IDs	Structural annotation	Functional annotation	SSR functional effects	Forward primers (5'-3')	Reverse primers (5'-3')	Amplified product size (bp)	Markers exhibiting polymorphism within *indica* rice	Transcrption factor genes-derived markers	Known cloned genes-derived markers	PMS markers corresponding with RM markers
PMS247	Os_Chr01	12161492	12161539	(CTT)16	(CTT)16	(CTT)16	(CTT)16	(CTT)16	(CTT)16	(CTT)16	(CTT)16	(CTT)16	(CTT)15	(CTT)15	LOC_Os01g21670	URR	plant protein of unknown function domain containing protein, expressed	-	GCACACCATGCAAATCAATGC	CAGAAACCTCATCTCCACCTTCC	393	A	A	A	RM10772
PMS248	Os_Chr01	12298641	12298652	(AT)6	(AT)6	(AT)6	(AT)6	(AT)6	(AT)6	(AT)6	(AT)6	(AT)6	(AT)7	(AT)6	-	Introns/Intergenic	-	-	AAGAGGGATGGAGGATGAGG	AAAGGCGAAGACGATGAGAA	209	A	A	A	NA
PMS249	Os_Chr01	12299786	12299806	(CGC)7	(CGC)5	(CGC)7	(CGC)7	(CGC)7	(CGC)6	(CGC)7	(CGC)7	(CGC)7	(CGC)7	(CGC)7	LOC_Os01g21930	CDS	expressed protein	CODON_DELETION	TATGTTGTCGCCCTTCAGAAAGC	AGGAAGACTCCTTCGACGTTGC	283	P	A	A	RM10779
PMS250	Os_Chr01	12333382	12333393	(TA)6	(TA)7	(TA)7	(TA)7	(TA)6	(TA)7	(TA)6	(TA)6	(TA)6	(TA)6	(TA)6	-	Introns/Intergenic	-	-	AGTTGCCAAAACCATCAAGC	TCAAATAACCAAGTGGCAGC	133	P	A	A	NA
PMS251	Os_Chr01	12677758	12677775	(CCG)6	(CCG)6	(CCG)6	(CCG)6	(CCG)6	(CCG)6	(CCG)6	(CCG)6	(CCG)6	(CCG)6	(CCG)5	LOC_Os01g22560	CDS	glycerol-3-phosphate acyltransferase 1, putative, expressed	CODON_DELETION	CTGAACCGCAGCAGGAAC	CACAACCTTTTCGTCTGCAA	272	A	A	A	NA
PMS252	Os_Chr01	12734991	12735012	(AG)11	(AG)11	(AG)11	(AG)11	(AG)11	(AG)11	(AG)11	(AG)8	(AG)11	(AG)11	(AG)11	-	Introns/Intergenic	-	-	CTTCACCCTCTTCAATTTCACC	TTGTGATTCCGCTTTGAGTAGC	312	A	A	A	RM10802
PMS253	Os_Chr01	12871949	12871964	(TC)8	(TC)9	(TC)9	(TC)9	(TC)8	(TC)9	(TC)8	(TC)8	(TC)8	(TC)9	(TC)9	-	Introns/Intergenic	-	-	GCTGTGCCTCTTGATGACAA	AGTTTAATGCCGGTTTGCAC	275	P	A	A	NA
PMS254	Os_Chr01	12946250	12946267	(CTC)6	(CTC)5	(CTC)6	(CTC)6	(CTC)6	(CTC)6	(CTC)6	(CTC)6	(CTC)6	(CTC)6	(CTC)6	-	Introns/Intergenic	-	-	ACCACCGCTCGTCTCCTC	TGGGACCCACCTGTCATACT	270	P	A	A	NA
PMS255	Os_Chr01	13056027	13056044	(CGC)6	(CGC)6	(CGC)6	(CGC)6	(CGC)6	(CGC)6	(CGC)6	(CGC)6	(CGC)6	(CGC)5	(CGC)6	-	Introns/Intergenic	-	-	CTCCCCCTCCCTCTCCTCT	TTCCCCTCTCTTGATTGCAC	219	A	A	A	NA
PMS256	Os_Chr01	13084215	13084232	(CGC)6	(CGC)6	(CGC)6	(CGC)6	(CGC)6	(CGC)6	(CGC)6	(CGC)5	(CGC)6	(CGC)6	(CGC)6	-	Introns/Intergenic	-	-	ACCCGTCATCCCCTTCCT	AAAGGAATCCGAGGGAGAGA	182	A	A	A	NA
PMS257	Os_Chr01	13139642	13139695	(AT)27	(AT)27	(AT)27	(AT)26	(AT)27	(AT)27	(AT)27	(AT)27	(AT)8	(AT)27	(AT)11	-	Introns/Intergenic	-	-	TTGCAAGTAAAGTGTAGAGAAGA	TTACACCATTACATTCGTTGC	291	P	A	A	NA
PMS258	Os_Chr01	13172274	13172325	(AT)26	(AT)26	(AT)26	(AT)6	(AT)26	(AT)26	(AT)26	(AT)16	(AT)26	(AT)9	(AT)26	-	Introns/Intergenic	-	-	TTACACCATTACATTCGTTGC	TTGCAAGTAAAGTGTAGAGAAGA	299	P	A	A	NA
PMS259	Os_Chr01	13345285	13345302	(CGC)6	(CGC)6	(CGC)6	(CGC)6	(CGC)6	(CGC)6	(CGC)6	(CGC)6	(CGC)6	(CGC)6	(CGC)5	LOC_Os01g23710	URR	no apical meristem protein, putative, expressed	-	TTCTCCTTCCCCTTCCACTT	TCTGACCAACTAACTCGCCC	103	A	A	A	NA

**Polymorphic SSR (PMS) markers**	**Chromosomes**	**Start physical positions (bp)**	**End physical positions (bp)**	**Nipponbare**	**Kasalath**	**Nagina22**	**IR64**	**Pokkali**	**Bala**	**Tainung67**	**Azucena**	**Moroberekan**	***Oryza rufipogon***	***Oryza nivara***	**MSU gene locus IDs**	**Structural annotation**	**Functional annotation**	**SSR functional effects**	**Forward primers (5'-3')**	**Reverse primers (5'-3')**	**Amplified product size (bp)**	**Markers exhibiting polymorphism within *indica* rice**	**Transcrption factor genes-derived markers**	**Known cloned genes-derived markers**	**PMS markers corresponding with RM markers**
PMS260	Os_Chr01	13473223	13473236	(AG)7	(AG)8	(AG)7	(AG)7	(AG)7	(AG)7	(AG)7	(AG)7	(AG)7	(AG)7	(AG)7	-	Introns/Intergenic	-	-	TCCCTCTCGTGCTGAGAAAT	AACCGGAGAGGTTTTGGTCT	215	P	A	A	NA
PMS261	Os_Chr01	13491816	13491855	(TA)20	(TA)20	(TA)20	(TA)7	(TA)20	(TA)20	(TA)9	(TA)20	(TA)6	(TA)6	(TA)20	-	Introns/Intergenic	-	-	ACGTGGAGGCTCCTTTTTCT	AGGAGGGGTGAAGATTCCTT	206	P	A	A	NA
PMS262	Os_Chr01	13572125	13572146	(GA)11	(GA)11	(GA)11	(GA)11	(GA)11	(GA)11	(GA)11	(GA)11	(GA)11	(GA)11	(GA)6	-	Introns/Intergenic	-	-	GCAACAGAGCTAACCACCAAACC	GCCTAATGTTGCTACGGGATGG	147	A	A	A	RM10838
PMS263	Os_Chr01	13639409	13639444	(AT)18	(AT)18	(AT)18	(AT)18	(AT)18	(AT)18	(AT)7	(AT)18	(AT)18	(AT)18	(AT)18	-	Introns/Intergenic	-	-	GGTCTACTGGTATCCCCCGT	TTTCTAGATCGACCGTTTCACA	243	A	A	A	NA
PMS264	Os_Chr01	13830848	13830865	(GGC)6	(GGC)6	(GGC)6	(GGC)6	(GGC)6	(GGC)5	(GGC)6	(GGC)6	(GGC)6	(GGC)6	(GGC)6	-	Introns/Intergenic	-	-	GGAGAGATCGAGATGAAGAGA	CACTGCCTAGGGCAAATC	153	P	A	A	NA
PMS265	Os_Chr01	14045451	14045478	(TA)14	(TA)14	(TA)14	(TA)11	(TA)14	(TA)14	(TA)14	(TA)10	(TA)14	(TA)14	(TA)7	-	Introns/Intergenic	-	-	GCATGCTAACTTGAGACGATTGG	ATTTACTGTGGGAGTTCGGTTGG	94	P	A	A	RM10853
PMS266	Os_Chr01	14069824	14069837	(AC)7	(AC)7	(AC)7	(AC)7	(AC)7	(AC)6	(AC)7	(AC)7	(AC)7	(AC)7	(AC)7	-	Introns/Intergenic	-	-	TTAGCGCCATGTGTCAATTT	CTTTTCCTCTTGTTGCACGG	173	P	A	A	NA
PMS267	Os_Chr01	14114499	14114514	(GA)8	(GA)6	(GA)8	(GA)8	(GA)8	(GA)8	(GA)8	(GA)8	(GA)8	(GA)8	(GA)8	-	Introns/Intergenic	-	-	AGTTGGACCACCGCCTCT	TCATATCCCTTCGTCAACCC	190	P	A	A	NA
PMS268	Os_Chr01	14146294	14146311	(GGC)6	(GGC)6	(GGC)6	(GGC)5	(GGC)6	(GGC)6	(GGC)6	(GGC)6	(GGC)6	(GGC)6	(GGC)6	-	Introns/Intergenic	-	-	CCCTTGGAATACTCTCGGGT	GTGCATGATGTTGAAGACCG	257	P	A	A	NA
PMS269	Os_Chr01	14149311	14149328	(GCG)6	(GCG)5	(GCG)6	(GCG)6	(GCG)6	(GCG)5	(GCG)6	(GCG)6	(GCG)6	(GCG)6	(GCG)6	-	Introns/Intergenic	-	-	GAGACTGCGCAGATTTTTCC	TCATACTACCCCGAAGGTGG	276	P	A	A	NA
PMS270	Os_Chr01	14212279	14212318	(AT)20	(AT)20	(AT)20	(AT)20	(AT)8	(AT)20	(AT)20	(AT)20	(AT)20	(AT)20	(AT)13	-	Introns/Intergenic	-	-	CTCCATACTTTGTGCAACTTGG	ACCTGTAGTCCTAATGACAGTTCTGG	405	P	A	A	RM10862
PMS271	Os_Chr01	14271467	14271504	(TA)19	(TA)19	(TA)19	(TA)19	(TA)19	(TA)19	(TA)19	(TA)19	(TA)15	(TA)19	(TA)19	-	Introns/Intergenic	-	-	CCACAACCAAGTTCCTATACGC	TAACACATATGCGGTCCAGTTAGC	196	A	A	A	RM10865
PMS272	Os_Chr01	14332927	14332944	(CGG)6	(CGG)8	(CGG)6	(CGG)6	(CGG)6	(CGG)8	(CGG)6	(CGG)6	(CGG)6	(CGG)8	(CGG)6	LOC_Os01g25370	CDS	SUMO protease, putative, expressed	CODON_INSERTION	AAGGAGGAAGCGTACGAGGT	CTCCAAGAAGCCCAAGCTC	189	P	A	A	NA

Polymorphic SSR (PMS) markers	Chromosomes	Start physical positions (bp)	End physical positions (bp)	Nipponbare	Kasalath	Nagina22	IR64	Pokkali	Bala	Tainung67	Azucena	Moroberekan	*Oryza rufipogon*	*Oryza nivara*	MSU gene locus IDs	Structural annotation	Functional annotation	SSR functional effects	Forward primers (5'-3')	Reverse primers (5'-3')	Amplified product size (bp)	Markers exhibiting polymorphism within *indica* rice	Transcrption factor genes-derived markers	Known cloned genes-derived markers	PMS markers corresponding with RM markers
PMS273	Os_Chr01	14374959	14374978	(AG)10	(AG)8	(AG)10	(AG)10	(AG)10	(AG)10	(AG)10	(AG)10	(AG)10	(AG)10	(AG)10	-	Introns/Intergenic	-	-	GTTGGTCCCGGTTTGTGTTACC	TCAGTGTGCAGTTAACCTTCATCG	423	P	A	A	RM10869
PMS274	Os_Chr01	14455611	14455622	(AG)6	(AG)7	(AG)7	(AG)7	(AG)6	(AG)7	(AG)6	(AG)6	(AG)6	(AG)7	(AG)6	-	Introns/Intergenic	-	-	TCTACGTGGAAGAGAGAGCG	GCCAGCTATAGCACGGACTC	225	P	A	A	NA
PMS275	Os_Chr01	14487107	14487127	(GGA)7	(GGA)7	(GGA)6	(GGA)7	(GGA)7	(GGA)7	(GGA)7	(GGA)7	(GGA)7	(GGA)7	(GGA)7	LOC_Os01g25560	URR	RALFL7 - Rapid ALkalinization Factor RALF family protein precursor, expressed	-	CGGGCTGGCAGTTGTAGTAGG	GTCGACCAAAGCAAGCAACC	370	P	A	A	RM10880
PMS276	Os_Chr01	14570124	14570141	(GGC)6	(GGC)6	(GGC)6	(GGC)6	(GGC)6	(GGC)6	(GGC)6	(GGC)6	(GGC)6	(GGC)5	(GGC)6	LOC_Os01g25700	CDS	transposon protein, putative, CACTA, En/Spm sub-class, expressed	CODON_DELETION	GTTCTCGTTCTCGTTCGTTA	TCCGATCTCGATCTCTCTC	487	A	A	A	NA
PMS277	Os_Chr01	14606122	14606153	(TA)16	(TA)16	(TA)16	(TA)16	(TA)16	(TA)16	(TA)8	(TA)16	(TA)11	(TA)16	(TA)16	-	Introns/Intergenic	-	-	GAGTCATAATTGGCAACTGAGG	CTTCTCCTATAAGCTGGCTTCC	355	A	A	A	RM10885
PMS278	Os_Chr01	14627523	14627561	(AAG)13	(AAG)13	(AAG)13	(AAG)13	(AAG)13	(AAG)13	(AAG)13	(AAG)13	(AAG)13	(AAG)11	(AAG)13	-	Introns/Intergenic	-	-	GGAAAGGAAGAATCAGACACAGAGC	GTACCGTTCCTTTCGTCACTTCC	114	A	A	A	RM562
PMS279	Os_Chr01	14631623	14631644	(AT)11	(AT)11	(AT)10	(AT)11	(AT)11	(AT)9	(AT)11	(AT)10	(AT)11	(AT)11	(AT)11	-	Introns/Intergenic	-	-	AGTGAACGTTGAAAGTGACAGC	CAAGTATTCCTTCCGTATGAGC	376	P	A	A	RM10888
PMS280	Os_Chr01	14714082	14714099	(GTC)6	(GTC)6	(GTC)6	(GTC)6	(GTC)6	(GTC)5	(GTC)6	(GTC)6	(GTC)6	(GTC)6	(GTC)6	-	Introns/Intergenic	-	-	GTTCGTCGTCGTCGTCATC	GAGAACGGCTTGACGGAG	218	P	A	A	NA
PMS281	Os_Chr01	14737980	14737997	(GCC)6	(GCC)6	(GCC)6	(GCC)6	(GCC)6	(GCC)6	(GCC)6	(GCC)6	(GCC)6	(GCC)6	(GCC)5	-	Introns/Intergenic	-	-	AATCAGCGAAAGCAAGGAGA	GTCAGGGACTACTTCGACGC	178	A	A	A	NA
PMS282	Os_Chr01	14738104	14738121	(CTC)6	(CTC)6	(CTC)6	(CTC)6	(CTC)5	(CTC)6	(CTC)6	(CTC)6	(CTC)6	(CTC)6	(CTC)6	-	Introns/Intergenic	-	-	GCGTCGAAGTAGTCCCTGAC	GCCCGTAGTACGCTGCTAAC	275	P	A	A	NA
PMS283	Os_Chr01	14759462	14759479	(GCG)6	(GCG)5	(GCG)6	(GCG)6	(GCG)6	(GCG)6	(GCG)6	(GCG)6	(GCG)6	(GCG)6	(GCG)6	-	Introns/Intergenic	-	-	TGGGAATGGCCTTACATCAT	GGGTTGGTGAGGAGATGCT	278	P	A	A	NA
PMS284	Os_Chr01	14774168	14774237	(TA)35	(TA)35	(TA)10	(TA)12	(TA)35	(TA)35	(TA)35	(TA)35	(TA)24	(TA)35	(TA)35	-	Introns/Intergenic	-	-	TCAATTTCAACAATGGTGCC	ACCCATCGTGTTAGGCAAAG	154	P	A	A	NA
PMS285	Os_Chr01	14796293	14796308	(AT)8	(AT)8	(AT)8	(AT)7	(AT)8	(AT)8	(AT)8	(AT)8	(AT)8	(AT)8	(AT)8	-	Introns/Intergenic	-	-	CGGTTATCTATCGGCCACAT	AATGTTCAGTGCATTCGTCG	268	P	A	A	NA

Polymorphic SSR (PMS) markers	Chromosomes	Start physical positions (bp)	End physical positions (bp)	Nipponbare	Kasalath	Nagina22	IR64	Pokkali	Bala	Tainung67	Azucena	Moroberekan	*Oryza rufipogon*	*Oryza nivara*	MSU gene locus IDs	Structural annotation	Functional annotation	SSR functional effects	Forward primers (5'-3')	Reverse primers (5'-3')	Amplified product size (bp)	Markers exhibiting polymorphism within *indica* rice	Transcrption factor genes-derived markers	Known cloned genes-derived markers	PMS markers corresponding with RM markers
PMS286	Os_Chr01	14946639	14946662	(TA)12	(TA)12	(TA)12	(TA)12	(TA)7	(TA)12	(TA)12	(TA)12	(TA)6	(TA)12	(TA)11	-	Introns/Intergenic	-	-	TCCGACTTTGTTCCTTGAAA	TCAATTTCAACAATGGTGCC	264	P	A	A	NA
PMS287	Os_Chr01	14982949	14982964	(CT)8	(CT)8	(CT)8	(CT)8	(CT)6	(CT)8	(CT)8	(CT)8	(CT)8	(CT)8	(CT)8	-	Introns/Intergenic	-	-	CTCCCGGCTTCTTTCTCC	CTCCTCCCCTCTTCCCTCT	276	P	A	A	NA
PMS288	Os_Chr01	14987912	14987939	(CT)14	(CT)10	(CT)14	(CT)14	(CT)14	(CT)9	(CT)14	(CT)14	(CT)14	(CT)14	(CT)14	-	Introns/Intergenic	-	-	CGGAATTGCCTCTTATCAACCTACG	ATCTTCGGCAAGCTCTATGTCTCC	168	P	A	A	RM10910
PMS289	Os_Chr01	15000670	15000705	(CTTCCC)6	(CTTCCC)6	(CTTCCC)6	(CTTCCC)6	(CTTCCC)6	(CTTCCC)6	(CTTCCC)6	(CTTCCC)6	(CTTCCC)6	(CTTCCC)5	(CTTCCC)6	LOC_Os01g26930	CDS	transposon protein, putative, unclassified, expressed	CODON_DELETION	TTGGGGATGGTTTTCTCTTG	AATGAGCCTTTCCCATCCTT	269	A	A	A	NA
PMS290	Os_Chr01	15059540	15059560	(CCG)7	(CCG)7	(CCG)7	(CCG)7	(CCG)7	(CCG)7	(CCG)5	(CCG)7	(CCG)7	(CCG)7	(CCG)7	LOC_Os01g27020	URR	transposon protein, putative, unclassified, expressed	-	CCTTTCTCGCTAGTCCGATTCC	CCAACCCACCTACTACTGCAACC	121	A	A	A	RM10915
PMS291	Os_Chr01	15105492	15105521	(AT)15	(AT)15	(AT)15	(AT)15	(AT)15	(AT)15	(AT)15	(AT)15	(AT)15	(AT)15	(AT)7	-	Introns/Intergenic	-	-	GCTCCAATTCCTTCTAGGGC	TGTGAACGAGAACGAGAACG	270	A	A	A	NA
PMS292	Os_Chr01	15113003	15113016	(CT)7	(CT)7	(CT)6	(CT)7	(CT)7	(CT)7	(CT)7	(CT)7	(CT)7	(CT)7	(CT)7	-	Introns/Intergenic	-	-	GGTAAAATGACGAAAATACCCC	CCTCGTCATCGAGCCACC	272	P	A	A	NA
PMS293	Os_Chr01	15157283	15157296	(GA)7	(GA)11	(GA)7	(GA)7	(GA)11	(GA)7	(GA)7	(GA)7	(GA)7	(GA)7	(GA)7	-	Introns/Intergenic	-	-	CACTCTCTCTCACTGCGTGC	AAGCGGCACAGCTACAAAAT	268	P	A	A	NA
PMS294	Os_Chr01	15181773	15181796	(ATT)8	(ATT)8	(ATT)7	(ATT)8	(ATT)8	(ATT)8	(ATT)5	(ATT)8	(ATT)8	(ATT)8	(ATT)8	-	Introns/Intergenic	-	-	TTCCGTGCCCAACTTTAATTGC	CCTAACACCATAGGAGTGCAACAGC	270	P	A	A	RM10923
PMS295	Os_Chr01	15215112	15215139	(GA)14	(GA)14	(GA)14	(GA)14	(GA)14	(GA)13	(GA)14	(GA)13	(GA)14	(GA)14	(GA)14	-	Introns/Intergenic	-	-	GGTGATTTCAGCGGCGTC	AAATCCCTCCGCAAAAGTCT	213	P	A	A	NA
PMS296	Os_Chr01	15501379	15501398	(AT)10	(AT)10	(AT)9	(AT)10	(AT)10	(AT)10	(AT)10	(AT)10	(AT)10	(AT)10	(AT)10	-	Introns/Intergenic	-	-	GACAATCGGACTCCTAAATACAGC	AGCTGTTAAGGGTAGCAGTGTGC	375	P	A	A	RM10929
PMS297	Os_Chr01	15547348	15547404	(ATA)19	(ATA)19	(ATA)19	(ATA)13	(ATA)19	(ATA)19	(ATA)14	(ATA)19	(ATA)19	(ATA)19	(ATA)19	-	Introns/Intergenic	-	-	AATTGGACTTTACGAGCCTGTGC	TCTCGTCCTCACCTCAAGAGTGG	273	P	A	A	RM10930
PMS298	Os_Chr01	15729416	15729433	(TA)9	(TA)8	(TA)8	(TA)9	(TA)9	(TA)8	(TA)8	(TA)8	(TA)8	(TA)8	(TA)8	-	Introns/Intergenic	-	-	TTTACGCCGTTGACCTTTTT	GCTCAAGGTTTGTTTTAATCCTG	264	P	A	A	NA

Polymorphic SSR (PMS) markers	Chromosomes	Start physical positions (bp)	End physical positions (bp)	Nipponbare	Kasalath	Nagina22	IR64	Pokkali	Bala	Tainung67	Azucena	Moroberekan	*Oryza rufipogon*	*Oryza nivara*	MSU gene locus IDs	Structural annotation	Functional annotation	SSR functional effects	Forward primers (5'-3')	Reverse primers (5'-3')	Amplified product size (bp)	Markers exhibiting polymorphism within *indica* rice	Transcrption factor genes-derived markers	Known cloned genes-derived markers	PMS markers corresponding with RM markers
PMS299	Os_Chr01	15746436	15746451	(TA)8	(TA)9	(TA)9	(TA)8	(TA)8	(TA)8	(TA)8	(TA)9	(TA)9	(TA)9	(TA)9	-	Introns/Intergenic	-	-	TTTACGCCGTTGAC CTTTTT	GCTCAAGGTTTGTTT TAATCCTG	262	P	A	A	NA
PMS300	Os_Chr01	15759082	15759151	(TA)35	(TA)35	(TA)6	(TA)35	(TA)35	(TA)35	(TA)35	(TA)35	(TA)7	(TA)35	(TA)13	-	Introns/Intergenic	-	-	GTTTTATACCTTGG AGCCCG	TACCACGTGGCAATC CTACA	244	P	A	A	NA
PMS301	Os_Chr01	15847641	15847664	(AG)12	(AG)12	(AG)12	(AG)12	(AG)12	(AG)8	(AG)12	(AG)12	(AG)12	(AG)13	(AG)10	LOC_Os01g28290	CDS	expressed protein	FRAME_SHIFT	GATCCATCGATCCA CTTCTGGAATGC	CCGGCCATCTCCTAC TGCCTTACC	120	P	A	A	RM10942
PMS302	Os_Chr01	15888911	15888928	(AT)9	(AT)9	(AT)9	(AT)9	(AT)9	(AT)9	(AT)9	(AT)7	(AT)9	(AT)9	(AT)9	-	Introns/Intergenic	-	-	TTATTTGCATGGCC CTGTTTGC	TGGTCCTTCCTCTTC TGACATAACG	373	A	A	A	RM23209
PMS303	Os_Chr01	15892676	15892737	(AT)31	(AT)31	(AT)31	(AT)8	(AT)12	(AT)31	(AT)31	(AT)31	(AT)31	(AT)31	(AT)31	-	Introns/Intergenic	-	-	ATCGCGATCAGCCA TCTGTGC	GCGGAAGTGGTACT TAGCCATCG	493	P	A	A	RM10947
PMS304	Os_Chr01	16096103	16096124	(TC)11	(TC)10	(TC)11	(TC)9	(TC)11	(TC)9	(TC)11	(TC)11	(TC)11	(TC)11	(TC)11	-	Introns/Intergenic	-	-	CTATTGGGCGTTGG TCTAGTGG	CAACGAAATCATCCC TAGCTTCC	279	P	A	A	RM513
PMS305	Os_Chr01	16103318	16103361	(AT)22	(AT)22	(AT)9	(AT)9	(AT)22	(AT)6	(AT)22	(AT)22	(AT)22	(AT)8	(AT)22	-	Introns/Intergenic	-	-	GGTTCTACCCGTAC ACCATAAACC	GGCAAGCACCTAAG CATATAGAACC	167	P	A	A	RM25111
PMS306	Os_Chr01	16175892	16175917	(TA)13	(TA)13	(TA)13	(TA)7	(TA)13	(TA)13	(TA)13	(TA)13	(TA)13	(TA)13	(TA)13	-	Introns/Intergenic	-	-	AGAGGTTGTCGCAA TAGCAGAGC	AACATGAGAACTCGT GGAAAGTGG	367	P	A	A	RM10959
PMS307	Os_Chr01	16247409	16247468	(TTA)20	(TTA)20	(TTA)20	(TTA)13	(TTA)20	(TTA)20	(TTA)20	(TTA)20	(TTA)20	(TTA)20	(TTA)20	-	Introns/Intergenic	-	-	TGGAAATGGCACAT GAAAAA	CAGCTGATGTCTCGA TGGAA	276	P	A	A	NA
PMS308	Os_Chr01	16325649	16325672	(GCT)8	(GCT)8	(GCT)8	(GCT)8	(GCT)8	(GCT)8	(GCT)8	(GCT)8	(GCT)8	(GCT)8	(GCT)5	LOC_Os01g29150	DRR	CYP734A6	-	AGCTCAAGGCCTCT AAACTCCTATCC	GCAGACGATGAGGA TCCATGC	82	A	A	P	RM10965
PMS309	Os_Chr01	16326971	16326984	(AG)7	(AG)10	(AG)7	(AG)7	(AG)7	(AG)7	(AG)7	(AG)7	(AG)7	(AG)7	(AG)7	-	Introns/Intergenic	-	-	GACACGGGATCAAT TTGCTT	CAAGGTCGTCTTCAA GCTCC	252	P	A	A	NA
PMS310	Os_Chr01	16432174	16432189	(GA)8	(GA)6	(GA)8	(GA)8	(GA)7	(GA)8	(GA)8	(GA)8	(GA)8	(GA)8	(GA)7	-	Introns/Intergenic	-	-	ACGGAAAAGTTGGT GTGAGC	ATAAACCCCTTCCAC AACCC	268	P	A	A	NA
PMS311	Os_Chr01	16453344	16453367	(ATAG)6	(ATAG)5	(ATAG)6	(ATAG)6	(ATAG)5	(ATAG)6	(ATAG)6	(ATAG)6	(ATAG)6	(ATAG)6	(ATAG)6	-	Introns/Intergenic	-	-	TGGCGACTTGTGAG TGTTTATAGG	CAAATGCTGTTAGGT CGCTACG	363	P	A	A	RM10967

Polymorphic SSR (PMS) markers	Chromosomes	Start physical positions (bp)	End physical positions (bp)	Nipponbare	Kasalath	Nagina22	IR64	Pokkali	Bala	Tainung67	Azucena	Moroberekan	*Oryza rufipogon*	*Oryza nivara*	MSU gene locus IDs	Structural annotation	Functional annotation	SSR functional effects	Forward primers (5'-3')	Reverse primers (5'-3')	Amplified product size (bp)	Markers exhibiting polymorphism within *indica* rice	Transcrption factor genes-derived markers	Known cloned genes-derived markers	PMS markers corresponding with RM markers
PMS312	Os_Chr01	16550726	16550749	(GA)12	(GA)12	(GA)12	(GA)12	(GA)12	(GA)12	(GA)12	(GA)12	(GA)12	(GA)12	(GA)8	-	Introns/Intergenic	-	-	TACCCGAGGGTAGATCAGATGG	CCTTTCACCGTGTTCGCTAGG	346	A	A	A	RM10971
PMS313	Os_Chr01	16649565	16649582	(CGC)6	(CGC)6	(CGC)6	(CGC)6	(CGC)6	(CGC)5	(CGC)6	(CGC)6	(CGC)6	(CGC)6	(CGC)6	LOC_Os01g29710	CDS	transposon protein, putative, CACTA, En/Spm sub-class, expressed	CODON_CHANGE_PLUS_CODON_DELETION	TCCGATCTCGATCTCTCTC	GTTCTCGTTCTCGTTCGTTA	363	P	A	A	NA
PMS314	Os_Chr01	16706285	16706305	(CGC)7	(CGC)7	(CGC)7	(CGC)7	(CGC)7	(CGC)7	(CGC)7	(CGC)7	(CGC)7	(CGC)5	(CGC)7	LOC_Os01g29820	URR	expressed protein	-	ACTCGCGCTCAGGCTTATCTCC	CTGGCAGCCGACAAAGATACG	183	A	A	A	RM10973
PMS315	Os_Chr01	17218496	17218509	(TA)7	(TA)6	(TA)7	(TA)7	(TA)7	(TA)7	(TA)7	(TA)7	(TA)7	(TA)7	(TA)6	-	Introns/Intergenic	-	-	GGACTCAAGAAGATCATCAGC	AAGCTTCTACACTGGAAGTTGG	434	P	A	A	RM10981
PMS316	Os_Chr01	17249834	17249909	(TA)38	(TA)38	(TA)38	(TA)6	(TA)6	(TA)15	(TA)38	(TA)18	(TA)38	(TA)38	(TA)7	-	Introns/Intergenic	-	-	CATGGAAATGTCACACCAACAGC	AGCATTGTTGGGCAAGTCAGC	677	P	A	A	RM10982
PMS317	Os_Chr01	17274754	17274777	(CTGC)6	(CTGC)5	(CTGC)6	(CTGC)6	(CTGC)6	(CTGC)6	(CTGC)6	(CTGC)6	(CTGC)6	(CTGC)6	(CTGC)5	LOC_Os01g31580	URR	BZIP protein, putative, expressed	-	TGGAGAGGAATGGGTGAGAAGC	CCTCTACAGTCCTCCACCAGAGG	160	P	A	A	RM10986
PMS318	Os_Chr01	17530330	17530347	(AAC)6	(AAC)6	(AAC)6	(AAC)6	(AAC)6	(AAC)5	(AAC)6	(AAC)6	(AAC)6	(AAC)6	(AAC)6	-	Introns/Intergenic	-	-	GCCTAGTCACAAGCGTAGCC	GCCAACCAACCCATCATTAC	218	P	A	A	NA
PMS319	Os_Chr01	17583740	17583754	(CGG)5	(CGG)7	(CGG)5	(CGG)7	(CGG)7	(CGG)7	(CGG)5	(CGG)5	(CGG)5	(CGG)5	(CGG)5	LOC_Os01g32130	URR	expressed protein	-	CCCACACTTCTCCACACCTT	GAGGAGGAGGAGGAAGAGGA	210	P	A	A	NA
PMS320	Os_Chr01	17754632	17754685	(TA)27	(TA)27	(TA)6	(TA)27	(TA)27	(TA)27	(TA)27	(TA)27	(TA)7	(TA)27	(TA)23	-	Introns/Intergenic	-	-	ATGGTGCCCCATATGAGTGT	CGCATCATTTTTCGGTTTTT	137	P	A	A	NA
PMS321	Os_Chr01	17950520	17950531	(AT)6	(AT)6	(AT)6	(AT)6	(AT)6	(AT)6	(AT)6	(AT)6	(AT)6	(AT)7	(AT)6	-	Introns/Intergenic	-	-	AGCCCAAGGAGTAAAGATTGA	CGATGCTGCTTGTCATCAAC	275	A	A	A	NA
PMS322	Os_Chr01	18011958	18011972	(GCG)5	(GCG)6	(GCG)5	(GCG)5	(GCG)5	(GCG)5	(GCG)5	(GCG)5	(GCG)5	(GCG)5	(GCG)5	-	Introns/Intergenic	-	-	ATTTCCCCAAATCGAAAACC	GACGATGCCGCTCCTCTC	129	P	A	A	NA
PMS323	Os_Chr01	18042067	18042093	(GTT)9	(GTT)9	(GTT)9	(GTT)9	(GTT)7	(GTT)9	(GTT)9	(GTT)9	(GTT)9	(GTT)9	(GTT)9	-	Introns/Intergenic	-	-	GCGAGGTGACCTTGTCGTGTGG	GCGGTAGGCGGACTTGATCTCC	181	P	A	A	RM11011
PMS324	Os_Chr01	18095159	18095176	(CGC)6	(CGC)6	(CGC)6	(CGC)6	(CGC)6	(CGC)6	(CGC)6	(CGC)6	(CGC)6	(CGC)5	(CGC)6	LOC_Os01g32964	URR	protein phosphotase protein, putative, expressed	-	GAAGTCTTCCCCCTTCTTCG	ACCACCACCACCACCAAC	242	A	A	A	NA

Polymorphic SSR (PMS) markers	Chromosomes	Start physical positions (bp)	End physical positions (bp)	Nipponbare	Kasalath	Nagina22	IR64	Pokkali	Bala	Tainung67	Azucena	Moroberekan	*Oryza rufipogon*	*Oryza nivara*	MSU gene locus IDs	Structural annotation	Functional annotation	SSR functional effects	Forward primers (5'-3')	Reverse primers (5'-3')	Amplified product size (bp)	Markers exhibiting polymorphism within *indica* rice	Transcrption factor genes-derived markers	Known cloned genes-derived markers	PMS markers corresponding with RM markers
PMS325	Os_Chr01	18139740	18139751	(TA)6	(TA)7	(TA)6	(TA)6	(TA)6	(TA)6	(TA)6	(TA)6	(TA)6	(TA)6	(TA)7	-	Introns/Intergenic	-	-	GTGGTCACTTTTTCCTCTCTT	GTGCATTTTGTGAGGTTTTAT	338	P	A	A	NA
PMS326	Os_Chr01	18185618	18185639	(AG)11	(AG)11	(AG)11	(AG)11	(AG)11	(AG)11	(AG)11	(AG)11	(AG)11	(AG)11	(AG)10	-	Introns/Intergenic	-	-	ATTGTTGGAGGTTGAGATCC	GTTTCCTATGTCCCTCATTCC	172	A	A	A	RM8229
PMS327	Os_Chr01	18277326	18277343	(GGC)6	(GGC)6	(GGC)6	(GGC)6	(GGC)6	(GGC)6	(GGC)6	(GGC)5	(GGC)6	(GGC)6	(GGC)5	-	Introns/Intergenic	-	-	CGTTCTCGTTCTCGTTCGTT	TCGATCTCTCTCCGTCGC	280	A	A	A	NA
PMS328	Os_Chr01	18731069	18731100	(AG)16	(AG)16	(AG)16	(AG)16	(AG)13	(AG)16	(AG)16	(AG)16	(AG)16	(AG)16	(AG)16	-	Introns/Intergenic	-	-	TGCAAGGAAACTAGGAGGGAAGC	CTTGACCTCCTCCTCCACAACC	186	P	A	A	RM3375
PMS329	Os_Chr01	18756826	18756857	(TC)16	(TC)16	(TC)16	(TC)16	(TC)16	(TC)16	(TC)16	(TC)16	(TC)16	(TC)16	(TC)15	LOC_Os01g34060	DRR	OsMYBS1	-	CATAGATCAGATGCCAAGAGTGC	AGAGGAGAGGATGAACATGAGG	341	A	A	P	RM5422
PMS330	Os_Chr01	18766784	18766801	(TG)9	(TG)9	(TG)9	(TG)9	(TG)8	(TG)9	(TG)9	(TG)9	(TG)9	(TG)9	(TG)9	-	Introns/Intergenic	-	-	CATGACCTCTAGAGCCTCCG	TGAGTCACCCTAGATTGGGC	269	P	A	A	NA
PMS331	Os_Chr01	18785946	18785985	(TA)20	(TA)20	(TA)10	(TA)9	(TA)20	(TA)20	(TA)20	(TA)20	(TA)20	(TA)20	(TA)20	-	Introns/Intergenic	-	-	ATGGTGCCCCATATGAGTGT	ATGTGGTAGAATGCTTCCCG	148	P	A	A	NA
PMS332	Os_Chr01	18788220	18788239	(TA)10	(TA)10	(TA)10	(TA)10	(TA)10	(TA)10	(TA)10	(TA)10	(TA)7	(TA)10	(TA)10	-	Introns/Intergenic	-	-	CAAGCAGCTGAAATCTGTTTCG	GCGAGCGTAATAGAGGGATTGC	463	A	A	A	RM11039
PMS333	Os_Chr01	18814856	18814885	(GGGGT)6	(GGGGT)6	(GGGGT)6	(GGGGT)6	(GGGGT)6	(GGGGT)6	(GGGGT)6	(GGGGT)6	(GGGGT)5	(GGGGT)6	(GGGGT)6	-	Introns/Intergenic	-	-	CAAACCCAAACTCCGGTAGA	TACCACTCTTTCCGCTTTGC	259	A	A	A	NA
PMS334	Os_Chr01	18855570	18855581	(GC)6	(GC)6	(GC)6	(GC)6	(GC)6	(GC)6	(GC)6	(GC)6	(GC)6	(GC)7	(GC)7	-	Introns/Intergenic	-	-	TCTGCCACATAGCATCAACTG	CTGCCTCAGTTTCCGTCAAT	190	A	A	A	NA
PMS335	Os_Chr01	18889924	18889935	(TA)6	(TA)6	(TA)6	(TA)6	(TA)7	(TA)6	(TA)6	(TA)6	(TA)6	(TA)7	(TA)7	-	Introns/Intergenic	-	-	GGAAAACTGTTGTGCATCCC	CAGATCTCATGCCATCATGC	160	P	A	A	NA
PMS336	Os_Chr01	18968778	18968801	(GAG)8	(GAG)8	(GAG)8	(GAG)8	(GAG)8	(GAG)8	(GAG)7	(GAG)8	(GAG)8	(GAG)8	(GAG)8	LOC_Os01g34390	CDS	prenyltransferase, putative, expressed	CODON_DELETION	GGGCTTTGGAGAGCTCGAAGTAGC	GGCTCTAGCTCCCATCGCATCG	191	A	A	A	RM11049
PMS337	Os_Chr01	19009784	19009807	(GGC)8	(GGC)5	(GGC)8	(GGC)8	(GGC)8	(GGC)8	(GGC)8	(GGC)8	(GGC)8	(GGC)8	(GGC)5	LOC_Os01g34480	URR	OS-CAD	-	CACTCCACGCCATCTCTCTCG	CTCAGACTCGGAGCTCATCTTCC	146	P	A	P	RM129

**Polymorphic SSR (PMS) markers**	**Chromosomes**	**Start physical positions (bp)**	**End physical positions (bp)**	**Nipponbare**	**Kasalath**	**Nagina22**	**IR64**	**Pokkali**	**Bala**	**Tainung67**	**Azucena**	**Moroberekan**	***Oryza rufipogon***	***Oryza nivara***	**MSU gene locus IDs**	**Structural annotation**	**Functional annotation**	**SSR functional effects**	**Forward primers (5'-3')**	**Reverse primers (5'-3')**	**Amplified product size (bp)**	**Markers exhibiting polymorphism within *indica* rice**	**Transcrption factor genes-derived markers**	**Known cloned genes-derived markers**	**PMS markers corresponding with RM markers**
PMS338	Os_Chr01	19010904	19010933	(GTACT)6	(GTACT)6	(GTACT)6	(GTACT)5	(GTACT)5	(GTACT)5	(GTACT)6	(GTACT)6	(GTACT)6	(GTACT)6	(GTACT)5	-	Introns/Intergenic	-	-	CTGTGCATTGCCCTTGGTAT	CAATCCAAATGCACGATCAA	121	P	A	A	NA
PMS339	Os_Chr01	19025343	19025374	(TC)16	(TC)16	(TC)16	(TC)16	(TC)16	(TC)14	(TC)16	(TC)16	(TC)16	(TC)16	(TC)16	-	Introns/Intergenic	-	-	TAAGACGGACGTGGTCTTCAGG	GGATTGATCCAACGTACGAGTGC	153	P	A	A	RM11054
PMS340	Os_Chr01	19077682	19077745	(TA)32	(TA)32	(TA)7	(TA)6	(TA)8	(TA)18	(TA)32	(TA)32	(TA)32	(TA)6	(TA)32	-	Introns/Intergenic	-	-	GGAACAAACGAAGCCTTAGTGG	GGACCCATTGTATCTGGTATTACGG	205	P	A	A	RM8139
PMS341	Os_Chr01	19322446	19322481	(GA)18	(GA)18	(GA)13	(GA)18	(GA)18	(GA)18	(GA)18	(GA)18	(GA)18	(GA)18	(GA)18	-	Introns/Intergenic	-	-	CCGAATCGTGGTGGTGTGAGC	GCGAGCTCGATTCCTTGCTTCC	224	P	A	A	RM11062
PMS342	Os_Chr01	19322611	19322630	(CT)10	(CT)10	(CT)8	(CT)10	(CT)10	(CT)10	(CT)10	(CT)10	(CT)10	(CT)7	(CT)10	-	Introns/Intergenic	-	-	GCTTTATACGCCATGGGAGA	CCGAAACTCGGAGTGAGAAA	232	P	A	A	NA
PMS343	Os_Chr01	19331299	19331328	(TA)15	(TA)15	(TA)15	(TA)8	(TA)15	(TA)15	(TA)15	(TA)15	(TA)15	(TA)15	(TA)15	-	Introns/Intergenic	-	-	AGAGAGGAGCGATGGGAGAGG	GCATGATTCCTACAAGACGAGAGC	312	P	A	A	RM11066
PMS344	Os_Chr01	19364987	19365000	(GA)7	(GA)7	(GA)7	(GA)6	(GA)7	(GA)6	(GA)7	(GA)7	(GA)7	(GA)7	(GA)7	-	Introns/Intergenic	-	-	GTTGCCGCCAAGTTCATC	CTCCCACCTCTCTTTTTCCC	224	P	A	A	NA
PMS345	Os_Chr01	19370830	19370863	(GT)17	(GT)17	(GT)17	(GT)17	(GT)17	(GT)17	(GT)17	(GT)17	(GT)17	(GT)8	(GT)17	-	Introns/Intergenic	-	-	GGAGATCTAGGCTACGACAGTGAGG	TGTTGTTGCCATCCTCTCAAACC	259	A	A	A	RM11068
PMS346	Os_Chr01	19390000	19390017	(GAG)6	(GAG)6	(GAG)6	(GAG)6	(GAG)6	(GAG)6	(GAG)6	(GAG)6	(GAG)6	(GAG)6	(GAG)5	LOC_Os01g35040	URR	ZOS1-09 - C2H2 zinc finger protein, expressed	-	CAACCAGAGCAGAGGAGAGG	CTTGCGCAGCTCGAAATC	109	A	A	A	NA
PMS347	Os_Chr01	19420188	19420208	(TTG)7	(TTG)7	(TTG)7	(TTG)7	(TTG)7	(TTG)9	(TTG)7	(TTG)7	(TTG)7	(TTG)7	(TTG)7	LOC_Os01g35070	CDS	retrotransposon protein, putative, Ty3-gypsy subclass, expressed	CODON_INSERTION	CATGGTTGTTTCCCTGGTTC	TCGGTTTCTCTTCCTGTGCT	197	P	A	A	NA
PMS348	Os_Chr01	19504341	19504408	(TA)34	(TA)34	(TA)34	(TA)34	(TA)34	(TA)34	(TA)34	(TA)34	(TA)6	(TA)34	(TA)34	-	Introns/Intergenic	-	-	TGGAGAACAATCAAGAGGCTTCC	GGGCATCCTATAAATGCTGATTGG	426	A	A	A	RM11072
PMS349	Os_Chr01	19519265	19519314	(AT)25	(AT)25	(AT)14	(AT)25	(AT)25	(AT)25	(AT)16	(AT)17	(AT)6	(AT)25	(AT)25	-	Introns/Intergenic	-	-	TTTTTGCATGGCCCTGTT	GCTAGCTAGGACATATAATGGATTG	280	P	A	A	NA
PMS350	Os_Chr01	19546643	19546662	(GATT)5	(GATT)5	(GATT)5	(GATT)5	(GATT)5	(GATT)5	(GATT)5	(GATT)5	(GATT)5	(GATT)5	(GATT)6	-	Introns/Intergenic	-	-	CAATCAACGCTCGGATTTTT	CGCTGATCTCACCAATGAAA	187	A	A	A	NA

Polymorphic SSR (PMS) markers	Chromosomes	Start physical positions (bp)	End physical positions (bp)	Nipponbare	Kasalath	Nagina22	IR64	Pokkali	Bala	Tainung67	Azucena	Moroberekan	*Oryza rufipogon*	*Oryza nivara*	MSU gene locus IDs	Structural annotation	Functional annotation	SSR functional effects	Forward primers (5'-3')	Reverse primers (5'-3')	Amplified product size (bp)	Markers exhibiting polymorphism within *indica* rice	Transcrption factor genes-derived markers	Known cloned genes-derived markers	PMS markers corresponding with RM markers
PMS351	Os_Chr01	19679542	19679589	(TA)24	(TA)24	(TA)7	(TA)16	(TA)24	(TA)14	(TA)24	(TA)24	(TA)24	(TA)24	(TA)24	-	Introns/Intergenic	-	-	TCAATTTCAACAATGGTGCC	AACAAGCATTTCTAACGCCG	162	P	A	A	NA
PMS352	Os_Chr01	19716606	19716623	(GTT)6	(GTT)6	(GTT)6	(GTT)6	(GTT)6	(GTT)6	(GTT)6	(GTT)6	(GTT)6	(GTT)6	(GTT)5	-	Introns/Intergenic	-	-	TTTCAAGGAGGGTTGGTTTG	AATGAATGTCCAGAGACCCG	159	A	A	A	NA
PMS353	Os_Chr01	19725425	19725510	(AT)43	(AT)43	(AT)43	(AT)43	(AT)43	(AT)43	(AT)43	(AT)43	(AT)6	(AT)8	(AT)43	-	Introns/Intergenic	-	-	AGGTCAAACGCATGTCATCA	GGCTTTTTATTTTTGAAATGCAAG	246	A	A	A	NA
PMS354	Os_Chr01	19880660	19880681	(GT)11	(GT)11	(GT)11	(GT)11	(GT)10	(GT)11	(GT)11	(GT)11	(GT)11	(GT)11	(GT)11	-	Introns/Intergenic	-	-	CCCTCCCTATACACAGGTAGAACC	CCATGCAGTTCTGAAACTGAACC	387	P	A	A	RM11089
PMS355	Os_Chr01	19910404	19910417	(CA)7	(CA)7	(CA)7	(CA)7	(CA)7	(CA)6	(CA)7	(CA)7	(CA)7	(CA)7	(CA)6	-	Introns/Intergenic	-	-	ATCAAATCTCTCTGCACCGC	TGGAGTTGACGAGAAGATGC	194	P	A	A	NA
PMS356	Os_Chr01	19969955	19969981	(CGC)9	(CGC)5	(CGC)9	(CGC)9	(CGC)9	(CGC)9	(CGC)9	(CGC)9	(CGC)9	(CGC)9	(CGC)9	LOC_Os01g36080	URR	protein phosphatase 2C containing protein, expressed	-	GGATTTAGTTACCTTTGGCGATGG	CCATACGATTTCCCTTCTGAATCC	229	P	A	A	RM11092
PMS357	Os_Chr01	19981381	19981395	(GCC)5	(GCC)5	(GCC)6	(GCC)5	(GCC)5	(GCC)5	(GCC)5	(GCC)5	(GCC)5	(GCC)5	(GCC)5	-	Introns/Intergenic	-	-	CACCTCCGATTTGGGTTTC	ACTCGGTTTTATCCGACGTG	247	P	A	A	NA
PMS358	Os_Chr01	19985024	19985051	(CTAT)7	(CTAT)7	(CTAT)9	(CTAT)7	(CTAT)7	(CTAT)7	(CTAT)7	(CTAT)7	(CTAT)7	(CTAT)7	(CTAT)8	-	Introns/Intergenic	-	-	CAACTACAACACGGACTACAAGATGC	ATTCAGATTGATTGGGCCTTCG	248	P	A	A	RM7192
PMS359	Os_Chr01	20027441	20027476	(ATAG)9	(ATAG)9	(ATAG)9	(ATAG)9	(ATAG)9	(ATAG)9	(ATAG)9	(ATAG)9	(ATAG)9	(ATAG)9	(ATAG)6	-	Introns/Intergenic	-	-	AGGGAAGGGAAGTATGTATGTACACG	TATGAGTGATGCCAGCTCAATGG	383	A	A	A	RM11096
PMS360	Os_Chr01	20469413	20469433	(GCG)7	(GCG)7	(GCG)5	(GCG)7	(GCG)7	(GCG)7	(GCG)7	(GCG)7	(GCG)7	(GCG)7	(GCG)7	-	Introns/Intergenic	-	-	GAGAGAGTGCAAGATGGAGACG	TGCGTATATATAGCCACCCTATCC	138	P	A	A	RM11120
PMS361	Os_Chr01	20513128	20513169	(TA)21	(TA)21	(TA)11	(TA)21	(TA)12	(TA)21	(TA)21	(TA)21	(TA)15	(TA)7	(TA)21	-	Introns/Intergenic	-	-	CAGTTGTGTAGTATTGTGGCTTAAA	AATTGGCAACCGATGAGATT	167	P	A	A	NA
PMS362	Os_Chr01	20630882	20630899	(CGG)6	(CGG)7	(CGG)6	(CGG)6	(CGG)6	(CGG)5	(CGG)5	(CGG)6	(CGG)6	(CGG)6	(CGG)6	LOC_Os01g36970	CDS	expressed protein	CODON_DELETION	GCCGTGTCGTACAAGATCAA	CTCTTGACGTGCTTGCTCAG	218	P	A	A	NA
PMS363	Os_Chr01	20652344	20652397	(TTA)18	(TTA)18	(TTA)18	(TTA)18	(TTA)18	(TTA)16	(TTA)7	(TTA)18	(TTA)18	(TTA)18	(TTA)18	-	Introns/Intergenic	-	-	CAGATGGCCACCACGTATAGTACC	GATGTCGCAATCTTTGGTTTCC	252	P	A	A	RM11135

Polymorphic SSR (PMS) markers	Chromosomes	Start physical positions (bp)	End physical positions (bp)	Nipponbare	Kasalath	Nagina22	IR64	Pokkali	Bala	Tainung67	Azucena	Moroberekan	*Oryza rufipogon*	*Oryza nivara*	MSU gene locus IDs	Structural annotation	Functional annotation	SSR functional effects	Forward primers (5'-3')	Reverse primers (5'-3')	Amplified product size (bp)	Markers exhibiting polymorphism within *indica* rice	Transcrption factor genes-derived markers	Known cloned genes-derived markers	PMS markers corresponding with RM markers
PMS364	Os_Chr01	20715777	20715791	(GCA)5	(GCA)5	(GCA)5	(GCA)5	(GCA)5	(GCA)6	(GCA)5	(GCA)5	(GCA)5	(GCA)5	(GCA)5	LOC_Os01g37110	CDS	ABIL2, putative, expressed	CODON_INSERTION	TTGGAAATTTTGGAACGGAG	AGATGGACAAACGTCCAAGC	207	P	A	A	NA
PMS365	Os_Chr01	20729857	20729877	(CGC)7	(CGC)7	(CGC)7	(CGC)7	(CGC)7	(CGC)7	(CGC)7	(CGC)7	(CGC)7	(CGC)5	(CGC)7	-	Introns/Intergenic	-	-	AACGAGAGGACGAGAATCAGAGG	CCATACCTAGGCCCACACAACC	518	A	A	A	RM11141
PMS366	Os_Chr01	20755437	20755452	(TA)8	(TA)8	(TA)6	(TA)8	(TA)8	(TA)8	(TA)8	(TA)8	(TA)8	(TA)8	(TA)8	-	Introns/Intergenic	-	-	GCGGTACAGATGAGAAAGGG	CGAAGGTAACTTTAGCGTGTCA	181	P	A	A	NA
PMS367	Os_Chr01	20782696	20782711	(GA)8	(GA)8	(GA)8	(GA)8	(GA)8	(GA)7	(GA)8	(GA)8	(GA)8	(GA)8	(GA)8	-	Introns/Intergenic	-	-	GAGGAGGTGGGAGTAGGAGG	AGAGCACCGGAAAAATCCTT	247	P	A	A	NA
PMS368	Os_Chr01	20822257	20822304	(TA)24	(TA)22	(TA)18	(TA)24	(TA)24	(TA)24	(TA)24	(TA)19	(TA)10	(TA)24	(TA)24	-	Introns/Intergenic	-	-	GTGCGTATCTTTGTGCGACTGG	GTTGGGTGATGCCGTGATGG	200	P	A	A	RM11145
PMS369	Os_Chr01	20826319	20826392	(TA)37	(TA)37	(TA)37	(TA)37	(TA)37	(TA)37	(TA)7	(TA)18	(TA)13	(TA)37	(TA)37	-	Introns/Intergenic	-	-	TGTACCAAGCAAACCAGCAA	TTTTATACCTTGGAGCCCGA	263	A	A	A	NA
PMS370	Os_Chr01	20834625	20834642	(TA)9	(TA)10	(TA)9	(TA)9	(TA)9	(TA)9	(TA)9	(TA)9	(TA)9	(TA)9	(TA)9	-	Introns/Intergenic	-	-	AACAAACCATGCATGCAGAA	AGTGTGGATCAGATGGGGAG	247	P	A	A	NA
PMS371	Os_Chr01	20843364	20843391	(TA)14	(TA)14	(TA)14	(TA)14	(TA)14	(TA)14	(TA)7	(TA)14	(TA)10	(TA)14	(TA)14	-	Introns/Intergenic	-	-	GTTTTGGGCCTTGGTAAGGA	GCCCACCTATTCGATCTTCA	227	A	A	A	NA
PMS372	Os_Chr01	20844333	20844366	(TA)17	(TA)17	(TA)17	(TA)17	(TA)9	(TA)6	(TA)17	(TA)17	(TA)17	(TA)17	(TA)17	-	Introns/Intergenic	-	-	TTTGGCCATTGGGAGAGAGG	GGGAAGTGGGTGTGACACTTGG	128	P	A	A	RM11150
PMS373	Os_Chr01	21128224	21128247	(GGC)8	(GGC)8	(GGC)8	(GGC)8	(GGC)8	(GGC)8	(GGC)6	(GGC)8	(GGC)8	(GGC)8	(GGC)8	LOC_Os01g37770	URR	RWD domain containing protein, expressed	-	GAGGAGACGAGACGTCAGTAGGG	GTACATCACACACGCGTTATTTGG	161	A	A	A	RM11167
PMS374	Os_Chr01	21195501	21195521	(GCG)7	(GCG)7	(GCG)7	(GCG)7	(GCG)7	(GCG)7	(GCG)6	(GCG)7	(GCG)7	(GCG)7	(GCG)7	LOC_Os01g37870	CDS	WSL	CODON_DELETION	GCCCTATCCCAATTCCCAACG	CGCGCGAATTACTACTACGAAGACG	382	A	A	P	RM11174
PMS375	Os_Chr01	21227331	21227388	(AT)29	(AT)29	(AT)8	(AT)29	(AT)29	(AT)12	(AT)29	(AT)21	(AT)7	(AT)29	(AT)10	-	Introns/Intergenic	-	-	AGAATTGATGAGAGAGGTGAGC	TTGAAAGACGAGTCTACAGTCC	378	P	A	A	RM11175
PMS376	Os_Chr01	21243031	21243057	(TTA)9	(TTA)9	(TTA)7	(TTA)9	(TTA)9	(TTA)9	(TTA)9	(TTA)9	(TTA)9	(TTA)9	(TTA)9	-	Introns/Intergenic	-	-	CGATGGGAGATCCGGCTAGTGC	TCGGAAATCGCAACACAGATTCG	380	P	A	A	RM6889

Polymorphic SSR (PMS) markers	Chromosomes	Start physical positions (bp)	End physical positions (bp)	Nipponbare	Kasalath	Nagina22	IR64	Pokkali	Bala	Tainung67	Azucena	Moroberekan	*Oryza rufipogon*	*Oryza nivara*	MSU gene locus IDs	Structural annotation	Functional annotation	SSR functional effects	Forward primers (5'-3')	Reverse primers (5'-3')	Amplified product size (bp)	Markers exhibiting polymorphism within *indica* rice	Transcrption factor genes-derived markers	Known cloned genes-derived markers	PMS markers corresponding with RM markers
PMS377	Os_Chr01	21275598	21275615	(CT)9	(CT)9	(CT)9	(CT)9	(CT)7	(CT)9	(CT)9	(CT)9	(CT)9	(CT)9	(CT)9	-	Introns/Intergenic	-	-	CCAATCCTGGAAAGCGTAAA	AGTTTCCTGGGACGAAACAA	125	P	A	A	NA
PMS378	Os_Chr01	21351175	21351246	(AT)36	(AT)36	(AT)36	(AT)10	(AT)14	(AT)36	(AT)36	(AT)36	(AT)36	(AT)36	(AT)36	-	Introns/Intergenic	-	-	CCTTCTCTTCCGCTTATTGTG	TTTGCTGCGGGAATTACTAAG	270	P	A	A	NA
PMS379	Os_Chr01	21420684	21420719	(AGAT)9	(AGAT)9	(AGAT)9	(AGAT)8	(AGAT)8	(AGAT)9	(AGAT)9	(AGAT)9	(AGAT)9	(AGAT)8	(AGAT)9	-	Introns/Intergenic	-	-	ATGCTACGTGACGGCGTAGG	CAATGATGATGGTGGACTGTGG	248	P	A	A	RM11179
PMS380	Os_Chr01	21543265	21543285	(CGG)7	(CGG)7	(CGG)7	(CGG)7	(CGG)7	(CGG)7	(CGG)6	(CGG)7	(CGG)7	(CGG)7	(CGG)7	-	Introns/Intergenic	-	-	GCGGAGAAGTAGGAGTCCAAGG	GTCAACCTCCGCTTCCATCG	215	A	A	A	RM11184
PMS381	Os_Chr01	21558895	21558934	(TA)20	(TA)20	(TA)20	(TA)20	(TA)20	(TA)10	(TA)20	(TA)20	(TA)20	(TA)10	(TA)20	-	Introns/Intergenic	-	-	AATTTCAACAATGATGCCCG	AATTACATGCAGGTGTGCCA	231	P	A	A	NA
PMS382	Os_Chr01	21568326	21568345	(CT)10	(CT)10	(CT)10	(CT)10	(CT)10	(CT)10	(CT)8	(CT)10	(CT)10	(CT)10	(CT)10	-	Introns/Intergenic	-	-	AGATCGAGATCTCCCTTCTCCATGC	AGCCCGTTGCAAGATAGTGAGAGAGG	103	A	A	A	RM11187
PMS383	Os_Chr01	21596755	21596772	(TC)9	(TC)8	(TC)9	(TC)9	(TC)9	(TC)9	(TC)9	(TC)9	(TC)9	(TC)9	(TC)9	-	Introns/Intergenic	-	-	AAGCCCTAGTTCATTGGCCT	CTCTCTAGCGGGAGGTTGTG	255	P	A	A	NA
PMS384	Os_Chr01	21597856	21597905	(AT)25	(AT)25	(AT)25	(AT)25	(AT)25	(AT)25	(AT)25	(AT)25	(AT)6	(AT)25	(AT)25	-	Introns/Intergenic	-	-	CCAATGGCAGTTTCCAAGTTTCC	AAACTTCCCGTGTACACTCCATGC	425	A	A	A	RM11188
PMS385	Os_Chr01	21675678	21675695	(GGA)6	(GGA)6	(GGA)5	(GGA)5	(GGA)5	(GGA)5	(GGA)6	(GGA)6	(GGA)6	(GGA)5	(GGA)5	-	Introns/Intergenic	-	-	GATCGAGATCCAGCTCACCT	TAGTCGACACACTCCCTCCC	275	P	A	A	NA
PMS386	Os_Chr01	21687752	21687775	(GTC)8	(GTC)8	(GTC)8	(GTC)8	(GTC)8	(GTC)7	(GTC)8	(GTC)8	(GTC)8	(GTC)8	(GTC)8	LOC_Os01g38610	DRR	helix-loop-helix DNA-binding domain containing protein, expressed	-	GGTCTCACATTGGACAACACATTGC	CTTCCCGGAAACCCTAGGATGC	168	P	P	A	RM11191
PMS387	Os_Chr01	21688281	21688298	(CGC)6	(CGC)6	(CGC)6	(CGC)6	(CGC)6	(CGC)5	(CGC)6	(CGC)6	(CGC)6	(CGC)6	(CGC)6	LOC_Os01g38610	CDS	helix-loop-helix DNA-binding domain containing protein, expressed	CODON_DELETION	GATCATCTCCTTCACCTGCG	ACAACCCTCTCTGCAGCCT	236	P	P	A	NA
PMS388	Os_Chr01	21688727	21688747	(GGT)7	(GGT)7	(GGT)7	(GGT)7	(GGT)7	(GGT)6	(GGT)7	(GGT)6	(GGT)7	(GGT)7	(GGT)7	LOC_Os01g38610	CDS	helix-loop-helix DNA-binding domain containing protein, expressed	CODON_DELETION	CTCCTCTGACTCGTTCGCACTGG	AACACCGGCTGTCACGTTGAGC	209	P	P	A	RM11192
PMS389	Os_Chr01	21719898	21719913	(CT)8	(CT)8	(CT)8	(CT)8	(CT)6	(CT)8	(CT)8	(CT)8	(CT)8	(CT)6	(CT)8	LOC_Os01g38670	URR	OsMST8	-	TTCCATTTCTCCCCTGACTG	CGAGGAAGACGAAGATGGTC	179	P	A	P	NA

Polymorphic SSR (PMS) markers	Chromosomes	Start physical positions (bp)	End physical positions (bp)	Nipponbare	Kasalath	Nagina22	IR64	Pokkali	Bala	Tainung67	Azucena	Moroberekan	*Oryza rufipogon*	*Oryza nivara*	MSU gene locus IDs	Structural annotation	Functional annotation	SSR functional effects	Forward primers (5'-3')	Reverse primers (5'-3')	Amplified product size (bp)	Markers exhibiting polymorphism within *indica* rice	Transcrption factor genes-derived markers	Known cloned genes-derived markers	PMS markers corresponding with RM markers
PMS390	Os_Chr01	21796054	21796067	(AT)7	(AT)7	(AT)6	(AT)7	(AT)7	(AT)7	(AT)7	(AT)7	(AT)7	(AT)8	(AT)9	-	Introns/Intergenic	-	-	TCATGTAATCGTTCCACCAAGG	TTAAACGGACGAAGGAAACG	436	P	A	A	RM11194
PMS391	Os_Chr01	21833837	21833854	(TA)9	(TA)9	(TA)9	(TA)9	(TA)9	(TA)9	(TA)6	(TA)9	(TA)9	(TA)9	(TA)9	-	Introns/Intergenic	-	-	GCCCCATATGAGTGTCCTA	GGGGTATAGAATACGGGTGTA	316	A	A	A	NA
PMS392	Os_Chr01	21887851	21887880	(GA)15	(GA)14	(GA)15	(GA)15	(GA)15	(GA)14	(GA)15	(GA)15	(GA)15	(GA)14	(GA)9	-	Introns/Intergenic	-	-	GTCGTCGCCATCATTGGTATCG	CACCTTTCCCGGATCAAGTACG	195	P	A	A	RM3341
PMS393	Os_Chr01	21933465	21933479	(CCG)5	(CCG)5	(CCG)5	(CCG)5	(CCG)5	(CCG)7	(CCG)5	(CCG)5	(CCG)5	(CCG)5	(CCG)5	LOC_Os01g39010	CDS	50S ribosomal protein, putative, expressed	CODON_INSERTION	GTCCTCGGCAATGTCGTC	ACCGTCTTCGATGATGAACC	280	P	A	A	NA
PMS394	Os_Chr01	21938926	21938946	(TCC)7	(TCC)7	(TCC)7	(TCC)7	(TCC)7	(TCC)7	(TCC)5	(TCC)7	(TCC)7	(TCC)7	(TCC)7	LOC_Os01g39020	CDS	OsHsfA7	CODON_DELETION	GTGGTGAGTCAGGTGGCAATCC	GCCACCATCTCGTACGTCTTCG	427	A	P	P	RM11207
PMS395	Os_Chr01	21944925	21944942	(TCG)6	(TCG)6	(TCG)6	(TCG)6	(TCG)6	(TCG)6	(TCG)6	(TCG)6	(TCG)6	(TCG)6	(TCG)7	LOC_Os01g39040	CDS	DUF260 domain containing protein, putative, expressed	CODON_INSERTION	TCCTCGAAAGGTACCTGCAC	GAGCTCGAGATAGGGACCG	235	A	P	A	NA
PMS396	Os_Chr01	21973584	21973598	(CGC)5	(CGC)5	(CGC)5	(CGC)5	(CGC)5	(CGC)5	(CGC)5	(CGC)5	(CGC)5	(CGC)6	(CGC)5	-	Introns/Intergenic	-	-	ATGGGTTTTCCTCCTCTCGT	TCGGAGCTAGGGTTTAGGGT	112	A	A	A	NA
PMS397	Os_Chr01	21993585	21993602	(CAG)6	(CAG)6	(CAG)6	(CAG)6	(CAG)6	(CAG)7	(CAG)6	(CAG)6	(CAG)6	(CAG)6	(CAG)6	LOC_Os01g39110	CDS	ZOS1-10 - C2H2 zinc finger protein, expressed	CODON_INSERTION	CATCCTACATGCCCTCCTGT	TCCACCAAGAACAATCCTCC	217	P	P	A	NA
PMS398	Os_Chr01	21995221	21995234	(TC)7	(TC)7	(TC)7	(TC)7	(TC)7	(TC)6	(TC)7	(TC)7	(TC)7	(TC)7	(TC)7	-	Introns/Intergenic	-	-	TGCCCATAGGACAAAGGAAG	TAGGCAATTAGGCAGGAGGA	276	P	A	A	NA
PMS399	Os_Chr01	21996021	21996038	(CCG)6	(CCG)6	(CCG)6	(CCG)6	(CCG)6	(CCG)6	(CCG)6	(CCG)6	(CCG)5	(CCG)6	(CCG)6	LOC_Os01g39110	CDS	ZOS1-10 - C2H2 zinc finger protein, expressed	CODON_CHANGE_PLUS_CODON_DELETION	CTACGACCACCACCACCC	ACGCCATGTGGGCGTAGT	227	A	P	A	NA
PMS400	Os_Chr01	21996503	21996517	(CAG)5	(CAG)5	(CAG)5	(CAG)5	(CAG)5	(CAG)5	(CAG)5	(CAG)5	(CAG)5	(CAG)7	(CAG)5	LOC_Os01g39110	CDS	ZOS1-10 - C2H2 zinc finger protein, expressed	CODON_INSERTION	CTACGAGCCACCACCACC	CCCTAGCTAAGAGCAAGGCA	216	A	P	A	NA
PMS401	Os_Chr01	22036910	22036927	(GCC)6	(GCC)6	(GCC)6	(GCC)6	(GCC)6	(GCC)6	(GCC)6	(GCC)6	(GCC)6	(GCC)5	(GCC)6	LOC_Os01g39150	CDS	DUF260 domain containing protein, putative, expressed	CODON_CHANGE_PLUS_CODON_DELETION	AAATATCCACCGCGACACAC	AGGATGTTCTTGATGCCGAA	277	A	A	A	NA
PMS402	Os_Chr01	22042325	22042342	(TA)9	(TA)9	(TA)7	(TA)9	(TA)9	(TA)9	(TA)9	(TA)9	(TA)9	(TA)9	(TA)7	-	Introns/Intergenic	-	-	TCATCGGCTTAATTGGATCG	GCAGTCGAGCTAAGGTCCAT	249	P	A	A	NA

Polymorphic SSR (PMS) markers	Chromosomes	Start physical positions (bp)	End physical positions (bp)	Nipponbare	Kasalath	Nagina22	IR64	Pokkali	Bala	Tainung67	Azucena	Moroberekan	*Oryza rufipogon*	*Oryza nivara*	MSU gene locus IDs	Structural annotation	Functional annotation	SSR functional effects	Forward primers (5'-3')	Reverse primers (5'-3')	Amplified product size (bp)	Markers exhibiting polymorphism within *indica* rice	Transcrption factor genes-derived markers	Known cloned genes-derived markers	PMS markers corresponding with RM markers
PMS403	Os_Chr01	22091530	22091543	(CT)7	(CT)7	(CT)6	(CT)7	(CT)7	(CT)6	(CT)7	(CT)7	(CT)7	(CT)7	(CT)7	-	Introns/Intergenic	-	-	TAATAAGGGATTCCGGCCGATGG	CATCGACCCTTCCATCCAATACG	289	P	A	A	RM11214
PMS404	Os_Chr01	22184271	22184308	(AT)19	(AT)19	(AT)19	(AT)19	(AT)9	(AT)19	(AT)19	(AT)6	(AT)19	(AT)15	(AT)19	-	Introns/Intergenic	-	-	CTTAGTACGACGAATCTGGATAGACC	AACGTTCCCATTATTCCTCACC	274	P	A	A	RM11221
PMS405	Os_Chr01	22279500	22279513	(CT)7	(CT)7	(CT)7	(CT)7	(CT)7	(CT)7	(CT)7	(CT)8	(CT)8	(CT)7	(CT)8	-	Introns/Intergenic	-	-	AGTGACACATTGCCGAGGTT	TGTGAGGAAGAAGGGGAAAA	117	A	A	A	NA
PMS406	Os_Chr01	22336621	22336646	(AG)13	(AG)13	(AG)13	(AG)13	(AG)13	(AG)11	(AG)13	(AG)13	(AG)13	(AG)13	(AG)13	-	Introns/Intergenic	-	-	GAGGCGAGGCTAATGGTGAGAGG	GTCGTCGCCGTGGTTTGAGC	271	P	A	A	RM11228
PMS407	Os_Chr01	22336902	22336913	(TG)6	(TG)6	(TG)6	(TG)6	(TG)6	(TG)7	(TG)6	(TG)6	(TG)6	(TG)6	(TG)6	-	Introns/Intergenic	-	-	GGCTGCTCCCTGCTATTTT	AAAAACGAGACACGGCTGAT	185	P	A	A	NA
PMS408	Os_Chr01	22341783	22341798	(CT)8	(CT)8	(CT)8	(CT)8	(CT)8	(CT)8	(CT)8	(CT)8	(CT)8	(CT)6	(CT)8	-	Introns/Intergenic	-	-	TACAGTAGGGCACCCTCGAC	TTAGGCACCCGTATCAGCTT	160	A	A	A	NA
PMS409	Os_Chr01	22379588	22379619	(TC)16	(TC)16	(TC)16	(TC)16	(TC)16	(TC)16	(TC)16	(TC)16	(TC)16	(TC)16	(TC)15	-	Introns/Intergenic	-	-	GAGGATGTGCTTATTTCCCTACG	ATACGAATCCTACGCACTTCAGG	277	A	A	A	RM11231
PMS410	Os_Chr01	22384795	22384814	(TA)10	(TA)10	(TA)10	(TA)10	(TA)10	(TA)10	(TA)10	(TA)10	(TA)10	(TA)10	(TA)8	-	Introns/Intergenic	-	-	GCCTGGTGGGTTAAACTGAG	TCAGACTAAGGTCCCGTTCG	261	A	A	A	NA
PMS411	Os_Chr01	22445749	22445762	(GA)7	(GA)6	(GA)7	(GA)7	(GA)7	(GA)7	(GA)7	(GA)7	(GA)7	(GA)7	(GA)7	-	Introns/Intergenic	-	-	TTGAATGTTTTCAAAGCTGGG	CGTGGTTTTCGGTGATTGTA	254	P	A	A	NA
PMS412	Os_Chr01	22466549	22466562	(GA)7	(GA)6	(GA)7	(GA)7	(GA)7	(GA)6	(GA)7	(GA)7	(GA)7	(GA)7	(GA)7	LOC_Os01g39830	URR	beta-galactosidase, putative, expressed	-	TAGACAAGAAAACCTGGGCG	GAGTGGAGTGTGGGGAAGAA	182	P	A	A	NA
PMS413	Os_Chr01	22480132	22480149	(TA)9	(TA)9	(TA)9	(TA)9	(TA)6	(TA)9	(TA)9	(TA)9	(TA)9	(TA)9	(TA)9	-	Introns/Intergenic	-	-	CGATGAAAATTAAGGCCCAA	AGGTAGCCATGGGTCTTGTG	274	P	A	A	NA
PMS414	Os_Chr01	22496248	22496265	(AGG)6	(AGG)5	(AGG)6	(AGG)6	(AGG)6	(AGG)6	(AGG)6	(AGG)6	(AGG)6	(AGG)6	(AGG)6	-	Introns/Intergenic	-	-	GCGAGATTCGTAGAGGAC	ATTTTCCCCACCCATACTAC	366	P	A	A	NA
PMS415	Os_Chr01	22535058	22535075	(CCT)6	(CCT)7	(CCT)6	(CCT)6	(CCT)6	(CCT)6	(CCT)6	(CCT)5	(CCT)6	(CCT)6	(CCT)6	LOC_Os01g39960	URR	OsLCYe|OsLCYepsilon	-	TCTCCCTCTCCCTCCTCCTA	AGAACTCCATCGCTTGGTTG	146	P	A	P	NA

Polymorphic SSR (PMS) markers	Chromosomes	Start physical positions (bp)	End physical positions (bp)	Nipponbare	Kasalath	Nagina22	IR64	Pokkali	Bala	Tainung67	Azucena	Moroberekan	*Oryza rufipogon*	*Oryza nivara*	MSU gene locus IDs	Structural annotation	Functional annotation	SSR functional effects	Forward primers (5'-3')	Reverse primers (5'-3')	Amplified product size (bp)	Markers exhibiting polymorphism within *indica* rice	Transcrption factor genes-derived markers	Known cloned genes-derived markers	PMS markers corresponding with RM markers
PMS416	Os_Chr01	22644926	22644947	(CT)11	(CT)11	(CT)11	(CT)11	(CT)11	(CT)11	(CT)11	(CT)11	(CT)11	(CT)10	(CT)11	-	Introns/Intergenic	-	-	TTCCGTCGCTGTGTTCTTCTCC	TGTCGAGGTCCTCTTCGTCTGG	372	A	A	A	RM11240
PMS417	Os_Chr01	22676113	22676130	(GCG)6	(GCG)6	(GCG)6	(GCG)6	(GCG)6	(GCG)6	(GCG)5	(GCG)6	(GCG)6	(GCG)6	(GCG)6	LOC_Os01g40190	URR	retrotransposon protein, putative, unclassified, expressed	-	CGCCACTAACCTCTAGCTGC	ATCAGCGAGAAGAGCAGCAC	280	A	P	A	NA
PMS418	Os_Chr01	22677830	22677844	(ACC)5	(ACC)5	(ACC)5	(ACC)5	(ACC)5	(ACC)5	(ACC)5	(ACC)5	(ACC)5	(ACC)7	(ACC)5	LOC_Os01g40190	CDS	retrotransposon protein, putative, unclassified, expressed	CODON_CHANGE_PLUS_CODON_INSERTION	CATTGGCATCAAGAGCAAGA	CGCCTTTCTTGAAGAGGTTG	215	A	P	A	NA
PMS419	Os_Chr01	22678827	22678850	(AG)12	(AG)12	(AG)12	(AG)12	(AG)12	(AG)12	(AG)12	(AG)12	(AG)10	(AG)12	(AG)12	-	Introns/Intergenic	-	-	CCCATCGGATTTATTCTCCA	GGCCATTTTAAAACAAGCACA	189	A	A	A	NA
PMS420	Os_Chr01	22691245	22691274	(TA)15	(TA)15	(TA)15	(TA)7	(TA)15	(TA)15	(TA)15	(TA)15	(TA)15	(TA)15	(TA)15	-	Introns/Intergenic	-	-	AGCGTGAATGCACACTTCTTTCC	ATTCAAACACGGACGACAGACG	335	P	A	A	RM11245
PMS421	Os_Chr01	22759977	22759991	(GGT)5	(GGT)6	(GGT)5	(GGT)5	(GGT)5	(GGT)5	(GGT)5	(GGT)5	(GGT)6	(GGT)5	(GGT)5	-	Introns/Intergenic	-	-	CCAGTAGGAGGTGAAGCAGC	GAGGAGGAGAGGAGGGTGAC	249	P	A	A	NA
PMS422	Os_Chr01	22763168	22763183	(TA)8	(TA)8	(TA)8	(TA)8	(TA)8	(TA)8	(TA)8	(TA)8	(TA)7	(TA)8	(TA)8	-	Introns/Intergenic	-	-	GGTGCCATTTGCAACAATTC	GTATCCGCTCCGTTTTCATC	223	A	A	A	NA
PMS423	Os_Chr01	22871460	22871477	(CCG)6	(CCG)6	(CCG)6	(CCG)6	(CCG)6	(CCG)6	(CCG)6	(CCG)5	(CCG)6	(CCG)6	(CCG)6	LOC_Os01g40480	CDS	anthranilate phosphoribosyltransferase, putative, expressed	CODON_DELETION	AGAACTGCGACCCGTAGATG	ACGTCTCGCTCTACCACGAC	108	A	A	A	NA
PMS424	Os_Chr01	22931983	22932006	(CTC)8	(CTC)8	(CTC)5	(CTC)8	(CTC)8	(CTC)8	(CTC)8	(CTC)8	(CTC)8	(CTC)8	(CTC)8	LOC_Os01g40610	URR	coiled-coil domain-containing protein, putative, expressed	-	TGGTCGTTCGGTCGGAGTCG	GTTCAGCACCTTGCGCTCACC	383	P	A	A	RM11253
PMS425	Os_Chr01	22954029	22954042	(AG)7	(AG)6	(AG)7	(AG)7	(AG)7	(AG)7	(AG)7	(AG)7	(AG)7	(AG)7	(AG)7	-	Introns/Intergenic	-	-	TCGATATGTACGCGGTTCAA	AGCATGCGTAGAGCGAACTC	200	P	A	A	NA
PMS426	Os_Chr01	22957383	22957403	(CAG)7	(CAG)7	(CAG)7	(CAG)7	(CAG)7	(CAG)7	(CAG)6	(CAG)7	(CAG)7	(CAG)7	(CAG)7	-	Introns/Intergenic	-	-	CACGCAATGCTGTTGGATAGG	AAATCCTTGATGCCCAGAGAGG	329	A	A	A	RM11254
PMS427	Os_Chr01	22958784	22958828	(AAG)15	(AAG)15	(AAG)15	(AAG)15	(AAG)15	(AAG)13	(AAG)15	(AAG)15	(AAG)15	(AAG)15	(AAG)5	-	Introns/Intergenic	-	-	ACGCGCACCAAGAGAGAAACG	CTCGGCAAGGAACTGGTCAGG	213	P	A	A	RM5646
PMS428	Os_Chr01	22983097	22983114	(CGC)6	(CGC)6	(CGC)6	(CGC)6	(CGC)6	(CGC)6	(CGC)6	(CGC)6	(CGC)6	(CGC)5	(CGC)6	LOC_Os01g40640	CDS	S1 RNA binding domain containing protein, expressed	CODON_DELETION	GAGACGGGGAAGAGGAGACT	CCAGCTTTGTGCTGACACTG	233	A	A	A	NA

Polymorphic SSR (PMS) markers	Chromosomes	Start physical positions (bp)	End physical positions (bp)	Nipponbare	Kasalath	Nagina22	IR64	Pokkali	Bala	Tainung67	Azucena	Moroberekan	*Oryza rufipogon*	*Oryza nivara*	MSU gene locus IDs	Structural annotation	Functional annotation	SSR functional effects	Forward primers (5'-3')	Reverse primers (5'-3')	Amplified product size (bp)	Markers exhibiting polymorphism within *indica* rice	Transcrption factor genes-derived markers	Known cloned genes-derived markers	PMS markers corresponding with RM markers
PMS429	Os_Chr01	23073892	23073909	(GCG)6	(GCG)7	(GCG)7	(GCG)6	(GCG)7	(GCG)6	(GCG)6	(GCG)6	(GCG)8	(GCG)6	(GCG)6	LOC_Os01g40820	CDS	peptidase family M41 containing protein, expressed	CODON_CHANGE_PLUS_CODON_INSERTION	GCAGTAATGGAGCAAGGGAG	CTGTACCGGAGATGGTCGAT	162	P	A	A	NA
PMS430	Os_Chr01	23113968	23113991	(TTAA)6	(TTAA)6	(TTAA)6	(TTAA)6	(TTAA)5	(TTAA)6	(TTAA)6	(TTAA)6	(TTAA)6	(TTAA)6	(TTAA)6	-	Introns/Intergenic	-	-	GAGGATAGGCATCTCTCGAAGC	ACGATGAAACGTGTAGCAGTACG	300	P	A	A	RM7056
PMS431	Os_Chr01	23227330	23227345	(GA)8	(GA)8	(GA)8	(GA)8	(GA)8	(GA)8	(GA)8	(GA)8	(GA)11	(GA)8	(GA)8	-	Introns/Intergenic	-	-	CCTCCCAAAACGAATTTGAA	TCGGTTATGAACGAGCTTCAG	138	A	A	A	NA
PMS432	Os_Chr01	23288502	23288521	(AG)10	(AG)10	(AG)10	(AG)10	(AG)10	(AG)6	(AG)10	(AG)10	(AG)10	(AG)10	(AG)9	-	Introns/Intergenic	-	-	CGATCCCTCTCACTGCACAAACG	AGGACGGCCAAACTCTGCTTGC	273	P	A	A	RM11274
PMS433	Os_Chr01	23399104	23399199	(TA)48	(TA)48	(TA)10	(TA)12	(TA)9	(TA)48	(TA)6	(TA)9	(TA)13	(TA)24	(TA)48	-	Introns/Intergenic	-	-	CAATATCAGCCTAGCACAAAGG	CTATCCAACCAGCCATTTATCC	361	P	A	A	RM11288
PMS434	Os_Chr01	23401208	23401227	(TC)10	(TC)10	(TC)10	(TC)10	(TC)10	(TC)10	(TC)10	(TC)10	(TC)10	(TC)10	(TC)11	-	Introns/Intergenic	-	-	TGAAAGATCCCTTTTCTGAATACTG	TCTGTAGACTGCATTTGTTAATTTTTG	270	A	A	A	NA
PMS435	Os_Chr01	23440792	23440821	(ATG)10	(ATG)6	(ATG)10	(ATG)10	(ATG)10	(ATG)6	(ATG)10	(ATG)10	(ATG)6	(ATG)5	(ATG)6	-	Introns/Intergenic	-	-	TTCTATGTATGCACGGAAGACG	CGTGGGTAAGTTTAGTGTCACC	502	P	A	A	RM11292
PMS436	Os_Chr01	23477618	23477631	(GA)7	(GA)7	(GA)7	(GA)7	(GA)7	(GA)7	(GA)7	(GA)6	(GA)7	(GA)7	(GA)7	-	Introns/Intergenic	-	-	GAGGTTGGGGATGACAGGT	AAGTCTTCCTTCTCTCCCGC	183	A	A	A	NA
PMS437	Os_Chr01	23515405	23515422	(CGG)6	(CGG)6	(CGG)6	(CGG)6	(CGG)6	(CGG)6	(CGG)6	(CGG)5	(CGG)6	(CGG)6	(CGG)6	LOC_Os01g41550	CDS	aspartic proteinase, putative, expressed	CODON_CHANGE_PLUS_CODON_DELETION	GACGACTTCGCGGTGAAC	ACCCCGATCTTGGTGAAGTA	215	A	A	A	NA
PMS438	Os_Chr01	23607385	23607399	(CAG)5	(CAG)6	(CAG)5	(CAG)5	(CAG)7	(CAG)7	(CAG)5	(CAG)5	(CAG)5	(CAG)5	(CAG)7	LOC_Os01g41710	URR	CAB2R	-	ACCCCCAAGATTCTCTCGAT	GTCTACCTCGCTCTGCTGCT	244	P	A	P	NA
PMS439	Os_Chr01	23768690	23768707	(TA)9	(TA)9	(TA)9	(TA)9	(TA)9	(TA)6	(TA)7	(TA)9	(TA)9	(TA)9	(TA)9	-	Introns/Intergenic	-	-	TGCAACGGAAAGAAAACACA	TTCCATTGTTTGGGAAATTTTA	246	P	A	A	NA
PMS440	Os_Chr01	23829398	23829411	(TA)7	(TA)6	(TA)6	(TA)7	(TA)6	(TA)7	(TA)7	(TA)7	(TA)7	(TA)6	(TA)6	-	Introns/Intergenic	-	-	CGTAAACACCTGGTGCAATG	ACTTGGACCATGAGAGTGGG	213	P	A	A	NA
PMS441	Os_Chr01	23922655	23922672	(CGG)6	(CGG)5	(CGG)6	(CGG)6	(CGG)6	(CGG)5	(CGG)6	(CGG)6	(CGG)6	(CGG)6	(CGG)6	LOC_Os01g42220	CDS	expressed protein	CODON_DELETION	GTGGGGGAGTACCTCAGCTC	CTGTACGTGCAGCCTTCCTC	265	P	A	A	NA

Polymorphic SSR (PMS) markers	Chromosomes	Start physical positions (bp)	End physical positions (bp)	Nipponbare	Kasalath	Nagina22	IR64	Pokkali	Bala	Tainung67	Azucena	Moroberekan	*Oryza rufipogon*	*Oryza nivara*	MSU gene locus IDs	Structural annotation	Functional annotation	SSR functional effects	Forward primers (5'-3')	Reverse primers (5'-3')	Amplified product size (bp)	Markers exhibiting polymorphism within *indica* rice	Transcrption factor genes-derived markers	Known cloned genes-derived markers	PMS markers corresponding with RM markers
PMS442	Os_Chr01	23960259	23960306	(GTT)16	(GTT)16	(GTT)16	(GTT)16	(GTT)16	(GTT)16	(GTT)16	(GTT)16	(GTT)16	(GTT)16	(GTT)14	LOC_Os01g42270	CDS	transcriptional corepressor LEUNIG, putative, expressed	CODON_DELETION	GGTGTGGTACCTTTATTGATGC	GGGTCTTTGCAGGTAAATAAGC	550	A	A	A	RM11308
PMS443	Os_Chr01	24030850	24030863	(CA)7	(CA)7	(CA)7	(CA)7	(CA)7	(CA)8	(CA)7	(CA)7	(CA)7	(CA)9	(CA)7	-	Introns/Intergenic	-	-	CATCTCAGAGCATTATGAATGTA	ACAACAAGATCTCACATGATT	271	P	A	A	NA
PMS444	Os_Chr01	24118546	24118569	(TA)12	(TA)12	(TA)12	(TA)7	(TA)12	(TA)12	(TA)12	(TA)12	(TA)12	(TA)12	(TA)12	-	Introns/Intergenic	-	-	TCACCTCCGGAGTGTAGTAAGC	TCTCTCTCATCTGTGTCGTTCC	329	P	A	A	RM11319
PMS445	Os_Chr01	24130492	24130513	(TC)11	(TC)11	(TC)11	(TC)11	(TC)8	(TC)8	(TC)11	(TC)11	(TC)11	(TC)11	(TC)11	-	Introns/Intergenic	-	-	ACATACCCAAATACCGGCAA	CATCTCCATGCCTTTGGACT	178	P	A	A	NA
PMS446	Os_Chr01	24153998	24154021	(CGG)8	(CGG)8	(CGG)8	(CGG)8	(CGG)8	(CGG)8	(CGG)8	(CGG)6	(CGG)8	(CGG)8	(CGG)8	LOC_Os01g42480	URR	Yip1 domain containing protein, expressed	-	AGGGTGAGGAGAAAACCCAT	AAAGCAACGAGAGATCCGAA	245	A	A	A	NA
PMS447	Os_Chr01	24191912	24191929	(CCG)6	(CCG)6	(CCG)6	(CCG)6	(CCG)6	(CCG)6	(CCG)5	(CCG)6	(CCG)6	(CCG)6	(CCG)6	LOC_Os01g42540	CDS	ENT domain containing protein, expressed	CODON_DELETION	GTCAGCAGCTCCTCCTTCTC	AATGCCAACCACACATCAAA	268	A	A	A	NA
PMS448	Os_Chr01	24264287	24264318	(AG)16	(AG)16	(AG)15	(AG)16	(AG)16	(AG)16	(AG)16	(AG)16	(AG)11	(AG)16	(AG)16	-	Introns/Intergenic	-	-	CTGTGTATCGGCCCATCTTGG	AGATTTCTCTCTCGCGTCCAACC	179	P	A	A	RM3366
PMS449	Os_Chr01	24360374	24360388	(GTG)5	(GTG)6	(GTG)5	(GTG)5	(GTG)5	(GTG)6	(GTG)5	(GTG)6	(GTG)5	(GTG)5	(GTG)6	LOC_Os01g42800	CDS	MEE18, putative, expressed	CODON_INSERTION	AAGACGCACCTGAGAAGACG	GCAGTGGTACTCGGTGTGAC	277	P	A	A	NA
PMS450	Os_Chr01	24395544	24395561	(CGC)6	(CGC)6	(CGC)6	(CGC)6	(CGC)6	(CGC)5	(CGC)6	(CGC)6	(CGC)6	(CGC)6	(CGC)6	LOC_Os01g42870	CDS	transferase family protein, putative, expressed	CODON_DELETION	TAGTCCACCTCGTTGAACCC	GACGGGTACTACGGCAACTG	239	P	A	A	NA
PMS451	Os_Chr01	24446802	24446828	(ACC)9	(ACC)9	(ACC)9	(ACC)9	(ACC)9	(ACC)9	(ACC)9	(ACC)9	(ACC)9	(ACC)9	(ACC)10	-	Introns/Intergenic	-	-	ATTTATGCCTTGGCCACTCAGC	GCAATGCAAGGGATGTTTATGG	114	A	A	A	RM6875
PMS452	Os_Chr01	24492031	24492056	(AG)13	(AG)13	(AG)6	(AG)6	(AG)6	(AG)13	(AG)13	(AG)7	(AG)13	(AG)13	(AG)6	-	Introns/Intergenic	-	-	ATGAGGATCTGCTTCCGTCTCC	CTCGCGAGCTTTGAGACAAGC	222	P	A	A	RM1180
PMS453	Os_Chr01	24504683	24504702	(GA)10	(GA)10	(GA)10	(GA)10	(GA)10	(GA)10	(GA)6	(GA)10	(GA)10	(GA)10	(GA)10	LOC_Os01g43020	URR	CTP synthase, putative, expressed	-	CACCAGCACGTACTTCATCTCG	ATACTCCCAAAGCCCTCATCC	253	A	A	A	RM11339
PMS454	Os_Chr01	24519954	24519979	(CT)13	(CT)13	(CT)13	(CT)13	(CT)13	(CT)9	(CT)13	(CT)13	(CT)13	(CT)13	(CT)13	-	Introns/Intergenic	-	-	AGGTGGGGATGATGGAGAC	ATAGAGGTGGATTGGGAGGC	181	P	A	A	NA

Polymorphic SSR (PMS) markers	Chromosomes	Start physical positions (bp)	End physical positions (bp)	Nipponbare	Kasalath	Nagina22	IR64	Pokkali	Bala	Tainung67	Azucena	Moroberekan	*Oryza rufipogon*	*Oryza nivara*	MSU gene locus IDs	Structural annotation	Functional annotation	SSR functional effects	Forward primers (5'-3')	Reverse primers (5'-3')	Amplified product size (bp)	Markers exhibiting polymorphism within *indica* rice	Transcrption factor genes-derived markers	Known cloned genes-derived markers	PMS markers corresponding with RM markers
PMS455	Os_Chr01	24553922	24553942	(CGG)7	(CGG)7	(CGG)5	(CGG)7	(CGG)7	(CGG)7	(CGG)7	(CGG)7	(CGG)7	(CGG)7	(CGG)7	LOC_Os01g43040	CDS	amino acid transporter, putative, expressed	CODON_DELETION	GAGGGTGGTGGAC TTGGAT	GTATGCTACGAGGC CCACAT	260	P	A	A	NA
PMS456	Os_Chr01	24606560	24606603	(TA)22	(TA)22	(TA)8	(TA)7	(TA)6	(TA)22	(TA)22	(TA)22	(TA)22	(TA)22	(TA)22	-	Introns/Intergenic	-	-	CAGAGAGTGTAGTC TTCCAACG	ATGCTTGGAGTTGAG ATTGC	346	P	A	A	RM11345
PMS457	Os_Chr01	24623172	24623187	(TC)8	(TC)8	(TC)8	(TC)8	(TC)8	(TC)8	(TC)8	(TC)8	(TC)8	(TC)8	(TC)9	LOC_Os01g43150	URR	OsFtsH9 FtsH protease, homologue of AtFtsH11, expressed	-	CTTCTCCCCCTCCG TTTC	ATCCTACCAACCAAC CCTCC	209	A	A	A	NA
PMS458	Os_Chr01	24646579	24646596	(GGC)6	(GGC)5	(GGC)5	(GGC)6	(GGC)6	(GGC)6	(GGC)6	(GGC)6	(GGC)6	(GGC)6	(GGC)6	-	Introns/Intergenic	-	-	GGGTGCAATGTGTT TTGTTG	GCCTGCGAAAATCCA TAGAA	263	P	A	A	NA
PMS459	Os_Chr01	24720416	24720433	(GCA)6	(GCA)5	(GCA)5	(GCA)5	(GCA)5	(GCA)5	(GCA)6	(GCA)5	(GCA)6	(GCA)6	(GCA)6	-	Introns/Intergenic	-	-	CATGATGAATGCTC GTCGTC	TGTGGCTAACAATGG CTGTG	220	A	A	A	NA
PMS460	Os_Chr01	24742200	24742213	(AG)7	(AG)7	(AG)7	(AG)7	(AG)7	(AG)8	(AG)7	(AG)7	(AG)7	(AG)7	(AG)8	LOC_Os01g43290	CDS	expressed protein	FRAME_SHIFT	CTTGTCGAGGAAGA AAAGCG	TTCTCACTGCTACAC GCCTC	175	P	A	A	NA
PMS461	Os_Chr01	24789144	24789161	(GCC)6	(GCC)7	(GCC)7	(GCC)6	(GCC)6	(GCC)6	(GCC)6	(GCC)7	(GCC)6	(GCC)7	(GCC)7	-	Introns/Intergenic	-	-	TGACATTTTGCGTT GCTTGT	CAACGGATACCAAC GAACCT	183	P	A	A	NA
PMS462	Os_Chr01	24820517	24820530	(TC)7	(TC)7	(TC)6	(TC)7	(TC)7	(TC)7	(TC)7	(TC)7	(TC)7	(TC)7	(TC)7	-	Introns/Intergenic	-	-	AGTAGCAGCAGCAA CAGCAG	ACCACCTTCCTCGTC TTCCT	173	P	A	A	NA
PMS463	Os_Chr01	24894791	24894811	(CGG)7	(CGG)7	(CGG)7	(CGG)7	(CGG)7	(CGG)7	(CGG)7	(CGG)7	(CGG)7	(CGG)6	(CGG)7	LOC_Os01g43480	URR	AAA-type ATPase family protein, putative, expressed	-	TCCAACCCTCCTCT CTATAAATACCG	CTCCATGTGTCCTCC TCGTTCC	184	A	A	A	RM11359
PMS464	Os_Chr01	24919338	24919351	(AG)7	(AG)7	(AG)7	(AG)7	(AG)7	(AG)7	(AG)6	(AG)7	(AG)7	(AG)7	(AG)7	-	Introns/Intergenic	-	-	CCACATGTGCCTGC CTACTA	CAGGGAAGGACCAA GTTGAA	134	A	A	A	NA
PMS465	Os_Chr01	24937988	24938005	(GGT)6	(GGT)5	(GGT)5	(GGT)5	(GGT)5	(GGT)5	(GGT)6	(GGT)5	(GGT)6	(GGT)6	(GGT)5	-	Introns/Intergenic	-	-	CCTCCCCTTCCTTC GATTAG	AAATAGGCGCAACAA ACCAC	147	A	A	A	NA
PMS466	Os_Chr01	24955203	24955230	(AT)14	(AT)7	(AT)14	(AT)14	(AT)14	(AT)14	(AT)6	(AT)14	(AT)14	(AT)7	(AT)14	-	Introns/Intergenic	-	-	TACGTGCGGGCAGT AAATCTGG	GTCGTTCTCTCTCCC TCAAATACACC	478	P	A	A	RM11362
PMS467	Os_Chr01	24989579	24989590	(TA)6	(TA)6	(TA)6	(TA)6	(TA)6	(TA)6	(TA)6	(TA)6	(TA)6	(TA)7	(TA)6	-	Introns/Intergenic	-	-	ATGAAATCCCCTTTT CCCC	CCCTTAGGCCCGTTC TTTTA	237	A	A	A	NA

Polymorphic SSR (PMS) markers	Chromosomes	Start physical positions (bp)	End physical positions (bp)	Nipponbare	Kasalath	Nagina22	IR64	Pokkali	Bala	Tainung67	Azucena	Moroberekan	*Oryza rufipogon*	*Oryza nivara*	MSU gene locus IDs	Structural annotation	Functional annotation	SSR functional effects	Forward primers (5'-3')	Reverse primers (5'-3')	Amplified product size (bp)	Markers exhibiting polymorphism within *indica* rice	Transcrption factor genes-derived markers	Known cloned genes-derived markers	PMS markers corresponding with RM markers
PMS468	Os_Chr01	24994515	24994532	(GGC)6	(GGC)6	(GGC)6	(GGC)6	(GGC)6	(GGC)6	(GGC)6	(GGC)6	(GGC)6	(GGC)5	(GGC)6	-	Introns/Intergenic	-	-	GGGTTAGTTACCGAGCACGA	ACGTTGGTGTCGCTGTAATG	257	A	A	A	NA
PMS469	Os_Chr01	25022534	25022561	(AT)14	(AT)14	(AT)14	(AT)14	(AT)14	(AT)14	(AT)14	(AT)8	(AT)14	(AT)14	(AT)14	-	Introns/Intergenic	-	-	TATTGAGCGGATTGTTGCTACGG	TATTCGTCGGACGGACTATTTGC	193	A	A	A	RM11367
PMS470	Os_Chr01	25162860	25162889	(TCT)10	(TCT)10	(TCT)10	(TCT)9	(TCT)10	(TCT)10	(TCT)10	(TCT)10	(TCT)8	(TCT)10	(TCT)10	-	Introns/Intergenic	-	-	AAACCTAACTCAGTGTCCCAGTCG	GGTGTCCTGATGATGATTTACCC	371	P	A	A	RM11377
PMS471	Os_Chr01	25282035	25282058	(CT)12	(CT)12	(CT)12	(CT)12	(CT)12	(CT)10	(CT)12	(CT)12	(CT)12	(CT)12	(CT)12	LOC_Os01g44110	URR	serine/threonine-protein kinase, putative, expressed	-	CTCTCCTTCACTTCTCCCTGATCC	AGCAATGGCGATGGAAGTAACG	124	P	A	A	RM11383
PMS472	Os_Chr01	25302448	25302465	(CTG)6	(CTG)6	(CTG)6	(CTG)6	(CTG)6	(CTG)6	(CTG)6	(CTG)6	(CTG)6	(CTG)6	(CTG)5	LOC_Os01g44140	CDS	expressed protein	CODON_DELETION	AGTGAGCGGGAGAGGAGG	CCGCACGAGTAAAAACCATT	273	A	A	A	NA
PMS473	Os_Chr01	25334395	25334408	(TC)7	(TC)7	(TC)7	(TC)6	(TC)7	(TC)6	(TC)7	(TC)7	(TC)7	(TC)7	(TC)7	-	Introns/Intergenic	-	-	AGTCCTCTTTTGGCTTTGGG	AGGAGAGAGAGAGGGTGGCT	113	P	A	A	NA
PMS474	Os_Chr01	25340609	25340630	(AT)11	(AT)11	(AT)11	(AT)6	(AT)9	(AT)6	(AT)11	(AT)11	(AT)11	(AT)11	(AT)11	-	Introns/Intergenic	-	-	TGGAATGTCAAAATGCCGTA	TTGCCAACGTCATCATTCTC	254	P	A	A	NA
PMS475	Os_Chr01	25438346	25438361	(TA)8	(TA)8	(TA)8	(TA)8	(TA)8	(TA)7	(TA)8	(TA)8	(TA)8	(TA)8	(TA)8	-	Introns/Intergenic	-	-	TTGCAAGCGGTTTTAGTCCT	TGGGTTTATGAAAAATGATGGTC	148	P	A	A	NA
PMS476	Os_Chr01	25700306	25700323	(AGC)6	(AGC)6	(AGC)6	(AGC)6	(AGC)6	(AGC)6	(AGC)6	(AGC)6	(AGC)6	(AGC)6	(AGC)5	-	Introns/Intergenic	-	-	ATGTTTGCTAGTGATCCGGG	GACGGGGAGGAAGAAGAATC	252	A	A	A	NA
PMS477	Os_Chr01	25826399	25826419	(GCT)7	(GCT)7	(GCT)7	(GCT)7	(GCT)5	(GCT)7	(GCT)7	(GCT)7	(GCT)7	(GCT)7	(GCT)7	LOC_Os01g45510	CDS	DUF617 domain containing protein, expressed	CODON_DELETION	GTAGAGCGTGCCGGTGAG	CACTCCGAGGACTTCGTGTC	186	P	A	A	NA
PMS478	Os_Chr01	25937975	25937988	(CT)7	(CT)7	(CT)7	(CT)7	(CT)7	(CT)7	(CT)7	(CT)7	(CT)6	(CT)7	(CT)7	-	Introns/Intergenic	-	-	GTGGAGTCATCCTCCTTCCA	GTATTACGCTTCTCAGCGGC	276	A	A	A	NA
PMS479	Os_Chr01	25946343	25946358	(CT)8	(CT)8	(CT)8	(CT)6	(CT)8	(CT)8	(CT)8	(CT)8	(CT)8	(CT)8	(CT)8	-	Introns/Intergenic	-	-	GTGCACACGGAAGAGTCTGA	GTATTACGCTTCTCAGCGGC	246	P	A	A	NA
PMS480	Os_Chr01	26014694	26014739	(TA)23	(TA)23	(TA)23	(TA)9	(TA)9	(TA)9	(TA)23	(TA)23	(TA)16	(TA)23	(TA)23	-	Introns/Intergenic	-	-	CGATGGAAGCACCTCTTTTT	GGAGGAGGAAGGGATGAGAG	221	P	A	A	NA

Polymorphic SSR (PMS) markers	Chromosomes	Start physical positions (bp)	End physical positions (bp)	Nipponbare	Kasalath	Nagina22	IR64	Pokkali	Bala	Tainung67	Azucena	Moroberekan	*Oryza rufipogon*	*Oryza nivara*	MSU gene locus IDs	Structural annotation	Functional annotation	SSR functional effects	Forward primers (5'-3')	Reverse primers (5'-3')	Amplified product size (bp)	Markers exhibiting polymorphism within *indica* rice	Transcrption factor genes-derived markers	Known cloned genes-derived markers	PMS markers corresponding with RM markers
PMS481	Os_Chr01	26043528	26043539	(GA)6	(GA)6	(GA)6	(GA)6	(GA)6	(GA)6	(GA)6	(GA)6	(GA)6	(GA)6	(GA)7	LOC_Os01g45850	DRR	expressed protein	-	CTCCACTCCACTCCGCTTC	CGTTAGCCTCCTCTCCCTCT	172	A	A	A	NA
PMS482	Os_Chr01	26087169	26087198	(AT)15	(AT)14	(AT)15	(AT)15	(AT)15	(AT)15	(AT)15	(AT)15	(AT)15	(AT)15	(AT)15	-	Introns/Intergenic	-	-	CGATCTCGTTAATGGAAAGC	CACTCTAGGCTATCAGTTTCTCG	589	P	A	A	RM11418
PMS483	Os_Chr01	26131885	26131920	(TA)18	(TA)18	(TA)18	(TA)18	(TA)18	(TA)18	(TA)18	(TA)6	(TA)18	(TA)18	(TA)18	-	Introns/Intergenic	-	-	TTGATTGGGACCTCTCACATGG	TTCCTTCTGGTTCTCTGTCACTGG	209	A	A	A	RM11423
PMS484	Os_Chr01	26143106	26143129	(CATA)6	(CATA)6	(CATA)6	(CATA)7	(CATA)6	(CATA)7	(CATA)6	(CATA)6	(CATA)7	(CATA)7	(CATA)7	-	Introns/Intergenic	-	-	GATTCCATAGCTGCGTGATTCTCC	AGATGAAACTGGCACGTGTGTCG	115	P	A	A	RM7318
PMS485	Os_Chr01	26149711	26149782	(TA)36	(TA)36	(TA)36	(TA)36	(TA)36	(TA)36	(TA)36	(TA)19	(TA)36	(TA)36	(TA)36	-	Introns/Intergenic	-	-	GGGAGATGATGCATGACACTT	TCGACAAGACTGTTTCCAAGG	266	A	A	A	NA
PMS486	Os_Chr01	26203636	26203667	(TATC)8	(TATC)5	(TATC)8	(TATC)6	(TATC)8	(TATC)5	(TATC)8	(TATC)8	(TATC)5	(TATC)8	(TATC)5	-	Introns/Intergenic	-	-	TTGCTATTAGTTGGTGGGGC	ACGTGGAGGCTCCTTTTTCT	156	P	A	A	NA
PMS487	Os_Chr01	26244607	26244620	(CT)7	(CT)7	(CT)7	(CT)7	(CT)7	(CT)7	(CT)6	(CT)7	(CT)7	(CT)7	(CT)7	-	Introns/Intergenic	-	-	TCTCTCTCTCTCTCAGCGGC	GTCGTCAGATCTGGTGGAGG	234	A	A	A	NA
PMS488	Os_Chr01	26261139	26261154	(GA)8	(GA)8	(GA)8	(GA)8	(GA)8	(GA)8	(GA)8	(GA)8	(GA)8	(GA)8	(GA)7	-	Introns/Intergenic	-	-	GAAGAGGGAGGACTTGGCTC	AAGTCTATTTTGCCTCCCCC	269	A	A	A	NA
PMS489	Os_Chr01	26302888	26302911	(GAC)8	(GAC)9	(GAC)9	(GAC)9	(GAC)9	(GAC)9	(GAC)8	(GAC)9	(GAC)9	(GAC)8	(GAC)8	LOC_Os01g46250	CDS	lipase, putative, expressed	CODON_INSERTION	GGTGAACAAGTTCGACGACTGC	TGGCGATGGTACATATCGAAACC	185	A	A	A	RM11431
PMS490	Os_Chr01	26372918	26372929	(GC)6	(GC)8	(GC)6	(GC)6	(GC)6	(GC)8	(GC)6	(GC)8	(GC)8	(GC)6	(GC)6	-	Introns/Intergenic	-	-	ATACGATTTCCCCCGTTTCT	GCTAACGCTAGTGCCGTTTC	153	P	A	A	NA
PMS491	Os_Chr01	26402713	26402724	(CA)6	(CA)6	(CA)6	(CA)6	(CA)6	(CA)6	(CA)6	(CA)6	(CA)6	(CA)7	(CA)6	-	Introns/Intergenic	-	-	CAAACATGGCAACGTCTCAC	CCCGTGCAGTTCCTACAGTT	199	A	A	A	NA
PMS492	Os_Chr01	26423684	26423701	(CGG)6	(CGG)6	(CGG)6	(CGG)6	(CGG)6	(CGG)6	(CGG)5	(CGG)6	(CGG)6	(CGG)6	(CGG)6	LOC_Os01g46440	CDS	VQ domain containing protein, putative, expressed	CODON_DELETION	CTCTGCACGATGGACTTGAA	GGCACGCGAGGTATAGATGT	144	A	A	A	NA
PMS493	Os_Chr01	26561209	26561226	(CTC)6	(CTC)5	(CTC)6	(CTC)6	(CTC)6	(CTC)5	(CTC)6	(CTC)6	(CTC)5	(CTC)6	(CTC)6	-	Introns/Intergenic	-	-	CTCAAGGACCAGCAAAGAGG	ACCTCCTCATGCGACGATAC	272	P	A	A	NA

**Polymorphic SSR (PMS) markers**	**Chromosomes**	**Start physical positions (bp)**	**End physical positions (bp)**	**Nipponbare**	**Kasalath**	**Nagina22**	**IR64**	**Pokkali**	**Bala**	**Tainung67**	**Azucena**	**Moroberekan**	***Oryza rufipogon***	***Oryza nivara***	**MSU gene locus IDs**	**Structural annotation**	**Functional annotation**	**SSR functional effects**	**Forward primers (5'-3')**	**Reverse primers (5'-3')**	**Amplified product size (bp)**	**Markers exhibiting polymorphism within *indica* rice**	**Transcrption factor genes-derived markers**	**Known cloned genes-derived markers**	**PMS markers corresponding with RM markers**
PMS494	Os_Chr01	26578007	26578042	(GGAGAT)6	(GGAGAT)6	(GGAGAT)6	(GGAGAT)5	(GGAGAT)6	(GGAGAT)6	(GGAGAT)6	(GGAGAT)6	(GGAGAT)6	(GGAGAT)6	(GGAGAT)6	-	Introns/Intergenic	-	-	CAACTGCCAAGAGGAGGAAC	AACTCGGCGACACAACCTAC	279	P	A	A	NA
PMS495	Os_Chr01	26582460	26582489	(TA)15	(TA)15	(TA)9	(TA)15	(TA)15	(TA)15	(TA)15	(TA)15	(TA)15	(TA)15	(TA)15	-	Introns/Intergenic	-	-	AGTGAACCGAGCTGGTGAGGTTCC	TCCCTCCCGCACTCTGAACG	515	P	A	A	RM11444
PMS496	Os_Chr01	26718123	26718140	(GGA)6	(GGA)5	(GGA)6	(GGA)6	(GGA)6	(GGA)6	(GGA)5	(GGA)6	(GGA)6	(GGA)6	(GGA)6	LOC_Os01g46840	CDS	expressed protein	CODON_DELETION	AGGACCTGCGGAGGAAGT	AGGAGGAGGAGGAGAGGTCA	207	P	A	A	NA
PMS497	Os_Chr01	26763057	26763077	(GCG)7	(GCG)6	(GCG)7	(GCG)7	(GCG)7	(GCG)7	(GCG)7	(GCG)7	(GCG)7	(GCG)7	(GCG)5	-	Introns/Intergenic	-	-	TGCGGCCATCATAACTGACG	CTCCTCTCCGCTTCTCCTTCC	119	P	A	A	RM11447
PMS498	Os_Chr01	26821654	26821687	(CA)17	(CA)16	(CA)17	(CA)17	(CA)17	(CA)16	(CA)17	(CA)17	(CA)10	(CA)17	(CA)17	-	Introns/Intergenic	-	-	AAAGCCTGGATAAGATGGTTCG	CTGTAGTTGCTGTTTGCCTGTCC	108	P	A	A	RM3143
PMS499	Os_Chr01	26869613	26869624	(AG)6	(AG)8	(AG)6	(AG)6	(AG)6	(AG)8	(AG)6	(AG)6	(AG)6	(AG)6	(AG)6	-	Introns/Intergenic	-	-	ACTGTCCTCAGTGCGAGGTT	TTACCGACACTTGGTCCTCC	192	P	A	A	NA
PMS500	Os_Chr01	26943194	26943207	(CG)7	(CG)7	(CG)7	(CG)7	(CG)7	(CG)7	(CG)7	(CG)7	(CG)7	(CG)7	(CG)6	-	Introns/Intergenic	-	-	CTTCTCTCTCTTCTCGGCCC	CCTCTATCAATTCAGCCGGA	204	A	A	A	NA
PMS501	Os_Chr01	27004362	27004376	(CGC)5	(CGC)5	(CGC)5	(CGC)5	(CGC)5	(CGC)5	(CGC)5	(CGC)5	(CGC)5	(CGC)6	(CGC)5	LOC_Os01g47256	URR	exosome complex exonuclease RRP40, putative, expressed	-	AAGCCCATAGCATGTGTTCC	GGAGGTCGGGGTAAGGTAAG	264	A	A	A	NA
PMS502	Os_Chr01	27027855	27027874	(TC)10	(TC)10	(TC)10	(TC)10	(TC)10	(TC)10	(TC)10	(TC)10	(TC)10	(TC)11	(TC)10	-	Introns/Intergenic	-	-	ATTGTCAGTCAGAATGGACAGG	CAGTAGTGTTTGTGTTGGAAGC	178	A	A	A	RM11455
PMS503	Os_Chr01	27056736	27056753	(GTG)6	(GTG)5	(GTG)6	(GTG)6	(GTG)6	(GTG)5	(GTG)6	(GTG)5	(GTG)5	(GTG)6	(GTG)5	LOC_Os01g47360	CDS	GATA zinc finger domain containing protein, expressed	CODON_DELETION	AGCTCACCTCAACTCCGGAAGC	CTGGGAACCTCCTCGATGACC	425	P	P	A	RM11456
PMS504	Os_Chr01	27073982	27074007	(AG)13	(AG)13	(AG)9	(AG)13	(AG)8	(AG)13	(AG)13	(AG)13	(AG)13	(AG)8	(AG)13	-	Introns/Intergenic	-	-	AAGCGGCCCAAAGATAAAGAGG	CGAGTACTACTGGCGCTACCTTGC	416	P	A	A	RM3241
PMS505	Os_Chr01	27197442	27197455	(CG)7	(CG)7	(CG)7	(CG)7	(CG)7	(CG)7	(CG)7	(CG)7	(CG)7	(CG)7	(CG)6	-	Introns/Intergenic	-	-	CATTGCAATCATCGCATTCT	CGTCAAGAACAGCCCATACC	146	A	A	A	NA
PMS506	Os_Chr01	27250415	27250434	(AG)10	(AG)10	(AG)10	(AG)10	(AG)10	(AG)11	(AG)10	(AG)10	(AG)10	(AG)10	(AG)10	-	Introns/Intergenic	-	-	GAGGAGCTCATGTTAGTGGAGAAGG	TTCAGCTGGAAGCACAACTTCG	325	P	A	A	RM11475

Polymorphic SSR (PMS) markers	Chromosomes	Start physical positions (bp)	End physical positions (bp)	Nipponbare	Kasalath	Nagina22	IR64	Pokkali	Bala	Tainung67	Azucena	Moroberekan	*Oryza rufipogon*	*Oryza nivara*	MSU gene locus IDs	Structural annotation	Functional annotation	SSR functional effects	Forward primers (5'-3')	Reverse primers (5'-3')	Amplified product size (bp)	Markers exhibiting polymorphism within *indica* rice	Transcrption factor genes-derived markers	Known cloned genes-derived markers	PMS markers corresponding with RM markers
PMS507	Os_Chr01	27278238	27278258	(GGA)7	(GGA)7	(GGA)7	(GGA)7	(GGA)6	(GGA)7	(GGA)7	(GGA)7	(GGA)7	(GGA)7	(GGA)7	LOC_Os01g47690	URR	OsGLYII1|OsETHE1	-	GCGCTATATAATAAGGCGGAGGTACG	CCGATAACACCCAAGCCAAGC	101	P	A	P	RM11476
PMS508	Os_Chr01	27281116	27281135	(CT)10	(CT)10	(CT)10	(CT)10	(CT)10	(CT)10	(CT)9	(CT)10	(CT)10	(CT)10	(CT)10	-	Introns/Intergenic	-	-	GTTGCCCATTTGTAGCCTCATGG	CCGACAATTAAGGGAGGGAAAGG	189	A	A	A	RM11477
PMS509	Os_Chr01	27316174	27316191	(ATA)6	(ATA)6	(ATA)7	(ATA)6	(ATA)6	(ATA)6	(ATA)6	(ATA)5	(ATA)6	(ATA)6	(ATA)6	-	Introns/Intergenic	-	-	CCCCCGTCTCATTCTAAGGT	TGCAACCATAAGTTTCCGTG	214	P	A	A	NA
PMS510	Os_Chr01	27331298	27331311	(GC)7	(GC)7	(GC)7	(GC)7	(GC)7	(GC)7	(GC)7	(GC)6	(GC)7	(GC)7	(GC)7	-	Introns/Intergenic	-	-	TTTAGCCTCATGCGCAATC	AAATCGCAAATCGCACTTCT	280	A	A	A	NA
PMS511	Os_Chr01	27354367	27354384	(GCG)6	(GCG)6	(GCG)6	(GCG)6	(GCG)6	(GCG)6	(GCG)5	(GCG)6	(GCG)6	(GCG)6	(GCG)6	-	Introns/Intergenic	-	-	AGAGCGAGGATTCCGACG	GCCCTACCTCTGCTTCTCCT	178	A	A	A	NA
PMS512	Os_Chr01	27366271	27366300	(TA)15	(TA)15	(TA)15	(TA)15	(TA)12	(TA)10	(TA)8	(TA)15	(TA)15	(TA)15	(TA)15	-	Introns/Intergenic	-	-	TCTAAGAATAGCGTGGGACCTATGG	TGTCTTGAGGAAAGAAGCAAGAGG	201	P	A	A	RM11480
PMS513	Os_Chr01	27511492	27511557	(ATA)22	(ATA)22	(ATA)22	(ATA)13	(ATA)22	(ATA)22	(ATA)22	(ATA)9	(ATA)6	(ATA)22	(ATA)7	-	Introns/Intergenic	-	-	GCAAGTCGATAGGCTAATCAATCC	CATGTGTAGTGGGAAGATGTGTGG	330	P	A	A	RM6738
PMS514	Os_Chr01	27560398	27560415	(CTG)6	(CTG)6	(CTG)6	(CTG)6	(CTG)6	(CTG)6	(CTG)6	(CTG)6	(CTG)6	(CTG)5	(CTG)6	LOC_Os01g48130	CDS	no apical meristem protein, putative, expressed	CODON_DELETION	TCTTGATGAGGTTGACGTGC	CTGCACGATCTCTCGTCTTG	165	A	P	A	NA
PMS515	Os_Chr01	27659024	27659041	(GCC)6	(GCC)6	(GCC)6	(GCC)6	(GCC)6	(GCC)6	(GCC)6	(GCC)6	(GCC)6	(GCC)5	(GCC)5	LOC_Os01g48260	CDS	expressed protein	CODON_DELETION	GCATCATTGCCTCCAAGG	CAGACACAGTAGCTCGCTCG	280	A	A	A	NA
PMS516	Os_Chr01	27671563	27671583	(GAG)7	(GAG)5	(GAG)7	(GAG)7	(GAG)7	(GAG)5	(GAG)7	(GAG)7	(GAG)7	(GAG)7	(GAG)7	LOC_Os01g48280	URR	OsUBC13	-	AGACGCGAACACACAGACAGC	CGTGTATAATAGCGGGCGATCC	357	P	A	P	RM11495
PMS517	Os_Chr01	27676520	27676537	(CCG)6	(CCG)5	(CCG)6	(CCG)6	(CCG)6	(CCG)6	(CCG)6	(CCG)6	(CCG)6	(CCG)6	(CCG)6	LOC_Os01g48290	CDS	dof zinc finger domain containing protein, putative, expressed	CODON_CHANGE_PLUS_CODON_DELETION	ATATAAAGGGTCGCTTCGCC	ACCTTCCCGAAGAGCTTGA	120	P	A	A	NA
PMS518	Os_Chr01	27725565	27725580	(GT)8	(GT)7	(GT)8	(GT)8	(GT)8	(GT)8	(GT)7	(GT)8	(GT)8	(GT)8	(GT)8	-	Introns/Intergenic	-	-	GTTTACCTCGCTCGGAGTTG	AGACCCTCCTCCTCCTCCTC	224	P	A	A	NA
PMS519	Os_Chr01	27754349	27754400	(ATAG)13	(ATAG)13	(ATAG)13	(ATAG)13	(ATAG)13	(ATAG)8	(ATAG)13	(ATAG)13	(ATAG)13	(ATAG)13	(ATAG)13	-	Introns/Intergenic	-	-	AGAATTGCTACGTGACGGCTTAGG	GAAGTATGTGCTTAGGCAGGATGG	377	P	A	A	RM11499

Polymorphic SSR (PMS) markers	Chromosomes	Start physical positions (bp)	End physical positions (bp)	Nipponbare	Kasalath	Nagina22	IR64	Pokkali	Bala	Tainung67	Azucena	Moroberekan	*Oryza rufipogon*	*Oryza nivara*	MSU gene locus IDs	Structural annotation	Functional annotation	SSR functional effects	Forward primers (5'-3')	Reverse primers (5'-3')	Amplified product size (bp)	Markers exhibiting polymorphism within *indica* rice	Transcrption factor genes-derived markers	Known cloned genes-derived markers	PMS markers corresponding with RM markers
PMS520	Os_Chr01	27851210	27851239	(CT)15	(CT)15	(CT)15	(CT)15	(CT)15	(CT)15	(CT)15	(CT)15	(CT)15	(CT)15	(CT)10	-	Introns/Intergenic	-	-	CGGAGGCTGTTTCAAGACTC	ATTCCACGTCTCCAAGATGG	197	A	A	A	NA
PMS521	Os_Chr01	27969442	27969469	(AT)14	(AT)11	(AT)14	(AT)14	(AT)14	(AT)14	(AT)14	(AT)14	(AT)14	(AT)14	(AT)14	-	Introns/Intergenic	-	-	CTTGCTCTGATGCTGACAGATAAACC	GGAGTCCATGTCATATGTGCTTTCC	298	P	A	A	RM11514
PMS522	Os_Chr01	27983850	27983873	(GCC)8	(GCC)8	(GCC)8	(GCC)8	(GCC)8	(GCC)8	(GCC)7	(GCC)8	(GCC)8	(GCC)8	(GCC)6	LOC_Os01g48790	URR	YT521-B-like family domain containing protein, expressed	-	GTGCAGTAGATAGTACACCTTTGAGC	GAACGAGTGAGAAGGATACAGTGG	452	A	A	A	RM11516
PMS523	Os_Chr01	28090722	28090737	(AC)8	(AC)8	(AC)8	(AC)8	(AC)8	(AC)6	(AC)8	(AC)8	(AC)8	(AC)8	(AC)8	-	Introns/Intergenic	-	-	ACCCGTTCAATTCACCTACG	CCAACACAATGCATCGAGAC	161	P	A	A	NA
PMS524	Os_Chr01	28134647	28134668	(GA)11	(GA)11	(GA)11	(GA)9	(GA)9	(GA)11	(GA)6	(GA)11	(GA)11	(GA)11	(GA)11	-	Introns/Intergenic	-	-	AGTTCGACGAGAACGAGAGATGG	ACCTCCCTCTCGGTCGTTGG	175	P	A	A	RM11523
PMS525	Os_Chr01	28135121	28135180	(GATA)15	(GATA)15	(GATA)15	(GATA)15	(GATA)15	(GATA)15	(GATA)15	(GATA)15	(GATA)15	(GATA)15	(GATA)14	-	Introns/Intergenic	-	-	ATCAAAGGATGTGGCGGTAG	CGATCAAATTCCCTCCTTGA	172	A	A	A	NA
PMS526	Os_Chr01	28144883	28144897	(AGG)5	(AGG)5	(AGG)5	(AGG)5	(AGG)5	(AGG)5	(AGG)5	(AGG)5	(AGG)5	(AGG)6	(AGG)5	-	Introns/Intergenic	-	-	TGTACAGAACGTAGCCCGTG	CTTGCTACTGTGCGAGCGTA	177	A	A	A	NA
PMS527	Os_Chr01	28250973	28250993	(CGG)7	(CGG)7	(CGG)7	(CGG)7	(CGG)7	(CGG)7	(CGG)7	(CGG)7	(CGG)7	(CGG)5	(CGG)7	LOC_Os01g49160	CDS	MYB family transcription factor, putative, expressed	CODON_CHANGE_PLUS_CODON_DELETION	CTGGAATACCAAGCTCAAGAAGAAGG	ATCACTCGAGCACTCTCGTTGG	683	A	P	A	RM11528
PMS528	Os_Chr01	28317866	28317879	(TA)7	(TA)6	(TA)7	(TA)7	(TA)7	(TA)7	(TA)7	(TA)7	(TA)7	(TA)7	(TA)7	-	Introns/Intergenic	-	-	AAGATTTTCTAGCATTCGCCA	AAGAACCACCAGTGCAATTT	220	P	A	A	NA
PMS529	Os_Chr01	28370261	28370284	(GGC)8	(GGC)8	(GGC)8	(GGC)8	(GGC)8	(GGC)8	(GGC)8	(GGC)8	(GGC)8	(GGC)5	(GGC)8	LOC_Os01g49340	CDS	arginine/serine-rich coiled-coil protein 1, putative, expressed	CODON_DELETION	AGTACGAGCTCCTCCCGCTTCG	TCCACCTCCTCCTCCTTCCTCTCC	325	A	A	A	RM11535
PMS530	Os_Chr01	28376392	28376407	(TA)8	(TA)8	(TA)8	(TA)8	(TA)8	(TA)8	(TA)8	(TA)8	(TA)8	(TA)8	(TA)7	LOC_Os01g49360	DRR	FAD-binding and arabino-lactone oxidase domains containing protein, putative, expressed	-	TTTAAATCGTAGAGCCCGGA	AAACCTGGTTGACTGCTGAGA	232	A	A	A	NA
PMS531	Os_Chr01	28432004	28432021	(CCG)6	(CCG)6	(CCG)6	(CCG)6	(CCG)6	(CCG)6	(CCG)5	(CCG)6	(CCG)6	(CCG)6	(CCG)6	LOC_Os01g49440	URR	expressed protein	-	CGCATTCCTTCATCACAAAA	ACAAACCCCACCTACGGTC	269	A	A	A	NA
PMS532	Os_Chr01	28618099	28618118	(TC)10	(TC)11	(TC)10	(TC)10	(TC)10	(TC)10	(TC)10	(TC)10	(TC)10	(TC)10	(TC)10	-	Introns/Intergenic	-	-	GGGCTGACTTAGCTCTTATTGTTGC	AGATGCTGCTACTACGAGCTTGC	250	P	A	A	RM11547

Polymorphic SSR (PMS) markers	Chromosomes	Start physical positions (bp)	End physical positions (bp)	Nipponbare	Kasalath	Nagina22	IR64	Pokkali	Bala	Tainung67	Azucena	Moroberekan	*Oryza rufipogon*	*Oryza nivara*	MSU gene locus IDs	Structural annotation	Functional annotation	SSR functional effects	Forward primers (5'-3')	Reverse primers (5'-3')	Amplified product size (bp)	Markers exhibiting polymorphism within *indica* rice	Transcrption factor genes-derived markers	Known cloned genes-derived markers	PMS markers corresponding with RM markers
PMS533	Os_Chr01	28621329	28621346	(GT)9	(GT)9	(GT)8	(GT)9	(GT)8	(GT)8	(GT)9	(GT)9	(GT)9	(GT)9	(GT)8	-	Introns/Intergenic	-	-	CAATGGGGGCTACCATACTG	AAACCAACACTTGCCACTCC	244	P	A	A	NA
PMS534	Os_Chr01	28624136	28624157	(TA)11	(TA)11	(TA)11	(TA)11	(TA)11	(TA)6	(TA)8	(TA)11	(TA)11	(TA)6	(TA)11	-	Introns/Intergenic	-	-	TGGCCTACATTGAAAGGGAA	CCGGGTTGTTGAAGTTTGAC	242	P	A	A	NA
PMS535	Os_Chr01	28809087	28809102	(GA)8	(GA)14	(GA)8	(GA)8	(GA)8	(GA)8	(GA)8	(GA)8	(GA)8	(GA)8	(GA)8	-	Introns/Intergenic	-	-	AGGGATGGGGAGAAGGAGAG	AAATCCCTCCGCAAAAGTCT	263	P	A	A	NA
PMS536	Os_Chr01	28906380	28906395	(AT)8	(AT)8	(AT)7	(AT)8	(AT)8	(AT)8	(AT)8	(AT)8	(AT)8	(AT)8	(AT)7	-	Introns/Intergenic	-	-	GAAAGTGAATGGGGAGCGTA	TTTGAATATGTGACTTTGCACG	156	P	A	A	NA
PMS537	Os_Chr01	28915901	28915914	(GA)7	(GA)6	(GA)7	(GA)6	(GA)7	(GA)6	(GA)7	(GA)7	(GA)7	(GA)6	(GA)7	LOC_Os01g50360	DRR	NAC domain containing protein, putative, expressed	-	GGCTTGCCCATAGAACAAAA	CGGCGTCCCTTAACTAATCA	197	P	A	A	NA
PMS538	Os_Chr01	29155909	29155922	(TC)7	(TC)7	(TC)7	(TC)7	(TC)7	(TC)7	(TC)7	(TC)7	(TC)7	(TC)8	(TC)7	-	Introns/Intergenic	-	-	TAGGCTGAGTTCCTTCCCCT	CACGAATTGCTCCATAGGGT	164	A	A	A	NA
PMS539	Os_Chr01	29199101	29199122	(TA)11	(TA)13	(TA)11	(TA)11	(TA)11	(TA)11	(TA)11	(TA)7	(TA)6	(TA)11	(TA)11	-	Introns/Intergenic	-	-	AACTCTAGCTAGTCGCATACGG	CTCCTGAGTACCTGTGTAACAACC	354	P	A	A	RM11564
PMS540	Os_Chr01	29282502	29282525	(CTC)8	(CTC)8	(CTC)8	(CTC)8	(CTC)10	(CTC)10	(CTC)8	(CTC)8	(CTC)8	(CTC)8	(CTC)8	LOC_Os01g50960	CDS	RNA polymerase II transcriptional coactivator KELP, putative, expressed	CODON_INSERTION	CGCGAAATCTAGCGAAACAAAGG	GAACAGGCCGAGGAGGAAGAGG	163	P	A	A	RM11569
PMS541	Os_Chr01	29363671	29363688	(GCC)6	(GCC)6	(GCC)6	(GCC)6	(GCC)6	(GCC)6	(GCC)6	(GCC)6	(GCC)6	(GCC)5	(GCC)6	-	Introns/Intergenic	-	-	ATCCTTCCTTCCTCTCTCCG	CGACATGGGGAGGATATGAC	194	A	A	A	NA
PMS542	Os_Chr01	29392408	29392421	(CT)7	(CT)14	(CT)7	(CT)7	(CT)7	(CT)7	(CT)7	(CT)7	(CT)7	(CT)7	(CT)7	-	Introns/Intergenic	-	-	CTACTCTCGCCCCTCCTCTT	CTCTAGCTCTGCCACATCGC	204	P	A	A	NA
PMS543	Os_Chr01	29536811	29536834	(CCT)8	(CCT)8	(CCT)8	(CCT)8	(CCT)8	(CCT)8	(CCT)6	(CCT)8	(CCT)8	(CCT)8	(CCT)8	-	Introns/Intergenic	-	-	CTAATCTGACTGGAAACCCAGATCG	GAGCGGAGGAGCTATAGGATCG	164	A	A	A	RM6405
PMS544	Os_Chr01	29606572	29606593	(CT)11	(CT)11	(CT)11	(CT)11	(CT)11	(CT)11	(CT)11	(CT)10	(CT)11	(CT)11	(CT)11	-	Introns/Intergenic	-	-	TGTGGAGGAGATGAGAGGAGAGG	AACCTAGCGGGTGAGATTTATCG	171	A	A	A	RM11593
PMS545	Os_Chr01	29615781	29615798	(AT)9	(AT)9	(AT)6	(AT)9	(AT)7	(AT)9	(AT)9	(AT)9	(AT)8	(AT)9	(AT)9	-	Introns/Intergenic	-	-	CCAAGCCGTTATCTGGTTTC	GCACTATGGAGCACCAAACA	209	P	A	A	NA

Polymorphic SSR (PMS) markers	Chromosomes	Start physical positions (bp)	End physical positions (bp)	Nipponbare	Kasalath	Nagina22	IR64	Pokkali	Bala	Tainung67	Azucena	Moroberekan	*Oryza rufipogon*	*Oryza nivara*	MSU gene locus IDs	Structural annotation	Functional annotation	SSR functional effects	Forward primers (5'-3')	Reverse primers (5'-3')	Amplified product size (bp)	Markers exhibiting polymorphism within *indica* rice	Transcrption factor genes-derived markers	Known cloned genes-derived markers	PMS markers corresponding with RM markers
PMS546	Os_Chr01	29616195	29616212	(TA)9	(TA)9	(TA)9	(TA)9	(TA)9	(TA)9	(TA)9	(TA)9	(TA)7	(TA)9	(TA)9	-	Introns/Intergenic	-	-	ATGGTGCCCCATATGAGTGT	GCTGTGGAGCAATGTGTGTT	232	A	A	A	NA
PMS547	Os_Chr01	29637777	29637800	(GA)12	(GA)12	(GA)12	(GA)6	(GA)12	(GA)12	(GA)12	(GA)12	(GA)12	(GA)12	(GA)12	LOC_Os01g51610	URR	OsLFL1	-	AGTGAACCGAAACCACCAAG	GCTCCTGCCATCTCTCCTC	241	P	P	P	NA
PMS548	Os_Chr01	29638103	29638123	(CCT)7	(CCT)7	(CCT)7	(CCT)7	(CCT)7	(CCT)7	(CCT)7	(CCT)7	(CCT)7	(CCT)7	(CCT)5	LOC_Os01g51610	CDS	OsLFL1	CODON_DELETION	CCCGTCCTCCCTCCTATAAA	GGAGGAGGAGGAGGTGGA	207	A	P	P	NA
PMS549	Os_Chr01	29675569	29675588	(AG)10	(AG)10	(AG)10	(AG)10	(AG)10	(AG)10	(AG)10	(AG)9	(AG)10	(AG)10	(AG)9	-	Introns/Intergenic	-	-	GGTGGGAAGCCAACCAGACTCG	GGGTTTACCTGCGCTAACCCAAGC	387	A	A	A	RM11601
PMS550	Os_Chr01	29702998	29703013	(GA)8	(GA)8	(GA)8	(GA)8	(GA)8	(GA)8	(GA)8	(GA)8	(GA)7	(GA)8	(GA)8	-	Introns/Intergenic	-	-	GAGAGGGAGAGAGGGAGAGG	AAATCCCTCCGCAAAAGTCT	123	A	A	A	NA
PMS551	Os_Chr01	29806781	29806806	(CT)13	(CT)13	(CT)13	(CT)13	(CT)13	(CT)13	(CT)13	(CT)13	(CT)7	(CT)13	(CT)13	LOC_Os01g51840	URR	IQ calmodulin-binding motif family protein, putative, expressed	-	TCAACAAGGCACCAAACACTACG	GAGTCTTAGAGGCCGGATTTGG	183	A	A	A	RM3615
PMS552	Os_Chr01	29926068	29926085	(AT)9	(AT)9	(AT)8	(AT)7	(AT)9	(AT)9	(AT)9	(AT)9	(AT)9	(AT)9	(AT)8	-	Introns/Intergenic	-	-	GCCCGCATTGCTAACATAAT	AAGACGTTCTAGCATTGCCC	261	P	A	A	NA
PMS553	Os_Chr01	29954467	29954482	(CA)8	(CA)6	(CA)8	(CA)8	(CA)8	(CA)8	(CA)8	(CA)8	(CA)7	(CA)8	(CA)8	-	Introns/Intergenic	-	-	ACGTTCTCGATCACCGAAAG	ACGGATCTACCGCTGTGACT	225	P	A	A	NA
PMS554	Os_Chr01	29995623	29995655	(GGT)11	(GGT)11	(GGT)11	(GGT)11	(GGT)11	(GGT)11	(GGT)10	(GGT)11	(GGT)11	(GGT)11	(GGT)11	LOC_Os01g52140	CDS	expressed protein	CODON_DELETION	GACCTCGACTCCTACTCCGACTCC	CCTCGTCCTCAAGTGCCTCTCC	163	A	A	A	RM5954
PMS555	Os_Chr01	30006093	30006108	(CG)8	(CG)8	(CG)8	(CG)8	(CG)8	(CG)8	(CG)7	(CG)8	(CG)8	(CG)8	(CG)8	-	Introns/Intergenic	-	-	GGATCATGTACACTTGCCGA	GCTACACGCGACACAACAAC	260	A	A	A	NA
PMS556	Os_Chr01	30051774	30051791	(CT)9	(CT)9	(CT)9	(CT)9	(CT)8	(CT)9	(CT)9	(CT)9	(CT)9	(CT)9	(CT)9	-	Introns/Intergenic	-	-	TCAATATTGTTTGTTTTTAATTCCGA	TCGCTGTGAGGGCTAGAAAT	261	P	A	A	NA
PMS557	Os_Chr01	30093963	30093988	(AG)13	(AG)15	(AG)15	(AG)13	(AG)19	(AG)13	(AG)13	(AG)13	(AG)14	(AG)13	(AG)13	-	Introns/Intergenic	-	-	TGCTCTTATACCGTTCACATCC	TCAAACTAACCCTTCTGAGAGC	285	P	A	A	RM1152
PMS558	Os_Chr01	30117196	30117211	(GA)8	(GA)8	(GA)8	(GA)8	(GA)8	(GA)8	(GA)7	(GA)8	(GA)8	(GA)8	(GA)8	LOC_Os01g52410	URR	myb-like DNA-binding domain containing protein, putative, expressed	-	ACTTTGCTCTCAGCTCTCGC	TCTCGACGAAGCTCCTGAGT	279	A	P	A	NA

Polymorphic SSR (PMS) markers	Chromosomes	Start physical positions (bp)	End physical positions (bp)	Nipponbare	Kasalath	Nagina22	IR64	Pokkali	Bala	Tainung67	Azucena	Moroberekan	*Oryza rufipogon*	*Oryza nivara*	MSU gene locus IDs	Structural annotation	Functional annotation	SSR functional effects	Forward primers (5'-3')	Reverse primers (5'-3')	Amplified product size (bp)	Markers exhibiting polymorphism within *indica* rice	Transcrption factor genes-derived markers	Known cloned genes-derived markers	PMS markers corresponding with RM markers
PMS559	Os_Chr01	30156026	30156052	(GGC)9	(GGC)9	(GGC)9	(GGC)9	(GGC)9	(GGC)9	(GGC)9	(GGC)9	(GGC)9	(GGC)7	(GGC)8	LOC_Os01g52480	CDS	senescence-associated protein, putative, expressed	CODON_DELETION	CCGGCTGCACTACCTGTTCACG	GGCGCGAAGTCGTTCCAGTACC	255	A	A	A	RM11616
PMS560	Os_Chr01	30166507	30166524	(GGA)6	(GGA)6	(GGA)6	(GGA)6	(GGA)6	(GGA)6	(GGA)6	(GGA)6	(GGA)6	(GGA)5	(GGA)6	LOC_Os01g52500	URR	NADP-ME2	-	ATACTACTCCCGCTTCCGGT	AGTCCTCGCCGTAGGTGTC	248	A	A	P	NA
PMS561	Os_Chr01	30167226	30167241	(TG)8	(TG)6	(TG)8	(TG)8	(TG)8	(TG)8	(TG)8	(TG)8	(TG)8	(TG)8	(TG)8	-	Introns/Intergenic	-	-	AACAGTGCGCTATTTTGGCT	GCAAATTGTAACCACTGCCC	206	P	A	A	NA
PMS562	Os_Chr01	30178143	30178186	(AT)22	(AT)19	(AT)22	(AT)11	(AT)22	(AT)18	(AT)22	(AT)22	(AT)22	(AT)22	(AT)20	-	Introns/Intergenic	-	-	TATATGTTAGGCACGCAGCCTAATGC	GGCAACGAAATCGCACAAGC	258	P	A	A	RM11620
PMS563	Os_Chr01	30224941	30224954	(CT)7	(CT)7	(CT)7	(CT)7	(CT)7	(CT)7	(CT)7	(CT)6	(CT)7	(CT)7	(CT)7	-	Introns/Intergenic	-	-	TTCTACCCTTCTCTTCCCCC	GGATCTACCGTCTCCGGG	165	A	A	A	NA
PMS564	Os_Chr01	30264085	30264105	(GGT)7	(GGT)7	(GGT)7	(GGT)7	(GGT)7	(GGT)7	(GGT)6	(GGT)7	(GGT)7	(GGT)7	(GGT)7	-	Introns/Intergenic	-	-	ACGTACACCCTGCAGTCCTTGC	CTACGCCGGCTACGTCAAGTCC	281	A	A	A	RM11624
PMS565	Os_Chr01	30427363	30427380	(TCC)6	(TCC)6	(TCC)6	(TCC)6	(TCC)6	(TCC)6	(TCC)6	(TCC)6	(TCC)6	(TCC)7	(TCC)6	LOC_Os01g52920	URR	expressed protein	-	AAGCCATTATCCCCAACCTC	CCAATCAAACCGAATCGAAC	170	A	A	A	NA
PMS566	Os_Chr01	30496205	30496219	(GCG)5	(GCG)5	(GCG)5	(GCG)5	(GCG)5	(GCG)6	(GCG)5	(GCG)5	(GCG)5	(GCG)5	(GCG)5	LOC_Os01g53070	CDS	ATP binding protein, putative, expressed	CODON_INSERTION	GGGAGCTGGAGTTGACTGAG	CTGAGAACCCTCAACCCAAG	150	P	A	A	NA
PMS567	Os_Chr01	30524225	30524248	(TGTT)6	(TGTT)5	(TGTT)5	(TGTT)5	(TGTT)5	(TGTT)6	(TGTT)6	(TGTT)6	(TGTT)6	(TGTT)5	(TGTT)5	-	Introns/Intergenic	-	-	GTTCCAAGACGATGATTCTACTCC	CGTACTAGCTTTGTTCCCATGC	196	P	A	A	RM11632
PMS568	Os_Chr01	30551521	30551544	(AT)12	(AT)12	(AT)12	(AT)12	(AT)12	(AT)12	(AT)12	(AT)12	(AT)12	(AT)12	(AT)7	-	Introns/Intergenic	-	-	TTGTCACCCTTACTAGGATCAGC	GTGTGACTCTTGATGTAACTCAGC	294	A	A	A	RM11635
PMS569	Os_Chr01	30871992	30872005	(AT)7	(AT)11	(AT)7	(AT)7	(AT)7	(AT)7	(AT)7	(AT)7	(AT)7	(AT)7	(AT)7	-	Introns/Intergenic	-	-	GGCGGCTCAACGTGTACTAT	GGCTCGGATAATGTGACGAT	136	P	A	A	NA
PMS570	Os_Chr01	31013905	31013919	(TCC)5	(TCC)5	(TCC)5	(TCC)5	(TCC)5	(TCC)6	(TCC)5	(TCC)5	(TCC)5	(TCC)5	(TCC)5	LOC_Os01g53930	URR	OsHXK6	-	AGGAGCAAATCCCCCAGAC	CCGTTCATACGTAAGGGCAT	241	P	A	P	NA
PMS571	Os_Chr01	31376703	31376729	(GGA)9	(GGA)9	(GGA)9	(GGA)9	(GGA)9	(GGA)9	(GGA)5	(GGA)9	(GGA)9	(GGA)9	(GGA)9	LOC_Os01g54560	CDS	trehalose synthase, putative, expressed	CODON_DELETION	GTTGATGACCTCCACGACCT	ATGCTCCCTTTCTCCTCCTC	165	A	A	A	NA

**Polymorphic SSR (PMS) markers**	**Chromosomes**	**Start physical positions (bp)**	**End physical positions (bp)**	**Nipponbare**	**Kasalath**	**Nagina22**	**IR64**	**Pokkali**	**Bala**	**Tainung67**	**Azucena**	**Moroberekan**	***Oryza rufipogon***	***Oryza nivara***	**MSU gene locus IDs**	**Structural annotation**	**Functional annotation**	**SSR functional effects**	**Forward primers (5'-3')**	**Reverse primers (5'-3')**	**Amplified product size (bp)**	**Markers exhibiting polymorphism within *indica* rice**	**Transcrption factor genes-derived markers**	**Known cloned genes-derived markers**	**PMS markers corresponding with RM markers**
PMS572	Os_Chr01	31411075	31411088	(TC)7	(TC)7	(TC)6	(TC)7	(TC)7	(TC)7	(TC)7	(TC)7	(TC)7	(TC)7	(TC)6	-	Introns/Intergenic	-	-	CTGACCGAGCGTAGGAAGAC	CACCATCTCCTCCTCCAAGA	233	P	A	A	NA
PMS573	Os_Chr01	31423683	31423706	(AT)12	(AT)12	(AT)12	(AT)12	(AT)12	(AT)11	(AT)12	(AT)12	(AT)12	(AT)12	(AT)12	-	Introns/Intergenic	-	-	AGTGTCTCTGGAGTTGGGAGTGG	CTGTTCTTCCAGATGGGCTTCC	157	P	A	A	RM11668
PMS574	Os_Chr01	31492479	31492499	(GGC)7	(GGC)7	(GGC)7	(GGC)7	(GGC)7	(GGC)7	(GGC)7	(GGC)6	(GGC)7	(GGC)7	(GGC)7	-	Introns/Intergenic	-	-	AGAGGAGGCATGGTGAGTTTGC	AGCAGAGGAAGAAGCTGCAGAGC	184	A	A	A	RM11670
PMS575	Os_Chr01	31511727	31511753	(CCT)9	(CCT)9	(CCT)9	(CCT)9	(CCT)9	(CCT)9	(CCT)6	(CCT)9	(CCT)9	(CCT)9	(CCT)9	LOC_Os01g54784	URR	expressed protein	-	CCTCCGCACCAAACCCTAGC	GACCTCCGCCGAGATAACAGG	229	A	A	A	RM11674
PMS576	Os_Chr01	31575733	31575750	(CCG)6	(CCG)6	(CCG)6	(CCG)6	(CCG)6	(CCG)6	(CCG)6	(CCG)5	(CCG)6	(CCG)6	(CCG)6	LOC_Os01g54910	URR	GTP-binding protein typA/bipA, putative, expressed	-	CTGTGGTCAGATGGGCTTTT	GAGGTCGGGAAGACAGGG	210	A	A	A	NA
PMS577	Os_Chr01	31650277	31650298	(AG)11	(AG)11	(AG)11	(AG)11	(AG)11	(AG)11	(AG)8	(AG)7	(AG)11	(AG)11	(AG)11	-	Introns/Intergenic	-	-	GGCAGTTTCGCTACTGAGACCAAGG	TCGAGCTTCTCCGGCTCAGG	271	A	A	A	RM11678
PMS578	Os_Chr01	31650550	31650570	(GCC)7	(GCC)7	(GCC)7	(GCC)7	(GCC)7	(GCC)7	(GCC)7	(GCC)7	(GCC)7	(GCC)5	(GCC)7	-	Introns/Intergenic	-	-	GAATCTATGGCAGAGGCAGC	CAGGGAGGAACGAAAACTCA	280	A	A	A	NA
PMS579	Os_Chr01	31657231	31657244	(TA)7	(TA)6	(TA)7	(TA)7	(TA)7	(TA)7	(TA)7	(TA)7	(TA)7	(TA)7	(TA)7	-	Introns/Intergenic	-	-	TGCAAATTTTTACAATCGTCCA	AAACCAACAGAGTGGTAACCG	172	P	A	A	NA
PMS580	Os_Chr01	31719935	31719962	(AG)14	(AG)14	(AG)14	(AG)14	(AG)14	(AG)14	(AG)14	(AG)11	(AG)14	(AG)14	(AG)14	-	Introns/Intergenic	-	-	GGAGCTTGAGTTAAGACTGGTTGG	CTTCCCTCTCGTTCAAATCAATCC	153	A	A	A	RM3324
PMS581	Os_Chr01	31732799	31732813	(TGC)5	(TGC)5	(TGC)5	(TGC)5	(TGC)5	(TGC)5	(TGC)5	(TGC)5	(TGC)5	(TGC)5	(TGC)6	LOC_Os01g55160	CDS	expressed protein	CODON_INSERTION	CACGACCGTTTCTTCTCCTC	TCACAGCCACGTACACAACA	168	A	A	A	NA
PMS582	Os_Chr01	31765319	31765338	(TA)10	(TA)10	(TA)7	(TA)10	(TA)10	(TA)10	(TA)10	(TA)10	(TA)10	(TA)10	(TA)10	-	Introns/Intergenic	-	-	GTTGTGGCTTCACCCTCTGC	AGAAATAAGTTCACCGGCCATCC	299	P	A	A	RM11687
PMS583	Os_Chr01	31790154	31790165	(CG)6	(CG)7	(CG)6	(CG)6	(CG)6	(CG)6	(CG)6	(CG)6	(CG)6	(CG)6	(CG)6	-	Introns/Intergenic	-	-	GACTTTTCTCGGACGGCTAA	GCATAAGCCACGTACTCCCT	213	P	A	A	NA
PMS584	Os_Chr01	31851351	31851371	(CTC)7	(CTC)7	(CTC)7	(CTC)7	(CTC)7	(CTC)7	(CTC)5	(CTC)7	(CTC)7	(CTC)7	(CTC)7	LOC_Os01g55340	CDS	dof zinc finger domain containing protein, putative, expressed	CODON_DELETION	GAAGTAGCAGAACTTGGTGTCACG	CTGCTTGCCTACCTTCCTCTCC	442	A	A	A	RM11691

Polymorphic SSR (PMS) markers	Chromosomes	Start physical positions (bp)	End physical positions (bp)	Nipponbare	Kasalath	Nagina22	IR64	Pokkali	Bala	Tainung67	Azucena	Moroberekan	*Oryza rufipogon*	*Oryza nivara*	MSU gene locus IDs	Structural annotation	Functional annotation	SSR functional effects	Forward primers (5'-3')	Reverse primers (5'-3')	Amplified product size (bp)	Markers exhibiting polymorphism within *indica* rice	Transcrption factor genes-derived markers	Known cloned genes-derived markers	PMS markers corresponding with RM markers
PMS585	Os_Chr01	31896217	31896248	(ATAG)8	(ATAG)8	(ATAG)8	(ATAG)8	(ATAG)8	(ATAG)8	(ATAG)8	(ATAG)8	(ATAG)8	(ATAG)7	(ATAG)8	-	Introns/Intergenic	-	-	ATGTCGGAATTTCTAGCCCC	TTTAATTGTGGAGCATCGGA	123	A	A	A	NA
PMS586	Os_Chr01	31900124	31900145	(AG)11	(AG)10	(AG)11	(AG)11	(AG)11	(AG)11	(AG)11	(AG)11	(AG)11	(AG)11	(AG)11	-	Introns/Intergenic	-	-	TGACACCGGATAGTATAACGTAGGC	GCTACACTTTCCCTTCGATTGC	297	P	A	A	RM11695
PMS587	Os_Chr01	31900598	31900615	(TC)9	(TC)9	(TC)9	(TC)8	(TC)8	(TC)8	(TC)9	(TC)9	(TC)8	(TC)9	(TC)6	-	Introns/Intergenic	-	-	AAATCCCTCCGCAAAAGTCT	GAGAGCGGAGAGGGAGGG	146	P	A	A	NA
PMS588	Os_Chr01	31931403	31931430	(CT)14	(CT)14	(CT)14	(CT)14	(CT)14	(CT)6	(CT)14	(CT)14	(CT)14	(CT)14	(CT)14	-	Introns/Intergenic	-	-	AATTTCTCTCGGCCCATCTT	CGAGGGGAAGGGACACTC	272	P	A	A	NA
PMS589	Os_Chr01	31948529	31948558	(CT)15	(CT)15	(CT)15	(CT)15	(CT)15	(CT)15	(CT)15	(CT)15	(CT)15	(CT)15	(CT)7	LOC_Os01g55450	URR	OsCIPK12	-	ATCAGCATCCCAAAGCTAGAACC	AACCGTATATTGAGGGAGCAAGC	110	A	A	P	RM3709
PMS590	Os_Chr01	32102782	32102799	(TG)9	(TG)9	(TG)10	(TG)9	(TG)9	(TG)9	(TG)9	(TG)9	(TG)9	(TG)9	(TG)10	-	Introns/Intergenic	-	-	GAATCCCTTTCCTTCCTCCA	GGCCCATTTGGAATGTAATG	280	P	A	A	NA
PMS591	Os_Chr01	32137913	32137936	(ACT)8	(ACT)8	(ACT)8	(ACT)8	(ACT)8	(ACT)8	(ACT)8	(ACT)8	(ACT)8	(ACT)8	(ACT)6	-	Introns/Intergenic	-	-	CTTTGACCAGGACAATCTAGC	CTATTATACCGTGAGGTGTGTACC	221	A	A	A	RM11706
PMS592	Os_Chr01	32144949	32144962	(CG)7	(CG)6	(CG)7	(CG)7	(CG)7	(CG)6	(CG)7	(CG)6	(CG)6	(CG)6	(CG)6	-	Introns/Intergenic	-	-	GCGATGTACTGCTGCATGAT	GAGCTCCAGCTCTTCCTCAA	258	P	A	A	NA
PMS593	Os_Chr01	32168026	32168043	(CCG)6	(CCG)5	(CCG)6	(CCG)6	(CCG)6	(CCG)6	(CCG)6	(CCG)6	(CCG)6	(CCG)5	(CCG)6	LOC_Os01g55860	CDS	expressed protein	CODON_DELETION	CCTCTCCAAGTCCAAGACGA	GAGAAGCACGGGAGAGTCTG	237	P	A	A	NA
PMS594	Os_Chr01	32203662	32203703	(AT)21	(AT)21	(AT)21	(AT)21	(AT)21	(AT)21	(AT)21	(AT)10	(AT)6	(AT)21	(AT)21	-	Introns/Intergenic	-	-	CTCACATGATTATACTTTGATG	CAGAGCATTATGAATGTATAGGA	296	A	A	A	NA
PMS595	Os_Chr01	32272189	32272206	(TG)9	(TG)9	(TG)9	(TG)9	(TG)9	(TG)9	(TG)6	(TG)9	(TG)9	(TG)9	(TG)9	-	Introns/Intergenic	-	-	GATCTCCGTCAGAATCACCG	GTTACACTGTGCGTGGCTTG	224	A	A	A	NA
PMS596	Os_Chr01	32322749	32322766	(GCG)6	(GCG)6	(GCG)6	(GCG)6	(GCG)6	(GCG)6	(GCG)6	(GCG)5	(GCG)6	(GCG)6	(GCG)6	-	Introns/Intergenic	-	-	CACCTCAGATATCGCCCACT	GCTGAGGAGAGTTGGGATTG	252	A	A	A	NA
PMS597	Os_Chr01	32325424	32325437	(TC)7	(TC)6	(TC)6	(TC)6	(TC)6	(TC)6	(TC)7	(TC)6	(TC)6	(TC)7	(TC)6	-	Introns/Intergenic	-	-	GCTATGCTCTTCTGGTTGCC	GTTCCGTCCTGACAATGCTT	227	A	A	A	NA

Polymorphic SSR (PMS) markers	Chromosomes	Start physical positions (bp)	End physical positions (bp)	Nipponbare	Kasalath	Nagina22	IR64	Pokkali	Bala	Tainung67	Azucena	Moroberekan	*Oryza rufipogon*	*Oryza nivara*	MSU gene locus IDs	Structural annotation	Functional annotation	SSR functional effects	Forward primers (5'-3')	Reverse primers (5'-3')	Amplified product size (bp)	Markers exhibiting polymorphism within *indica* rice	Transcrption factor genes-derived markers	Known cloned genes-derived markers	PMS markers corresponding with RM markers
PMS598	Os_Chr01	32340875	32340894	(CT)10	(CT)10	(CT)10	(CT)10	(CT)13	(CT)13	(CT)10	(CT)10	(CT)10	(CT)10	(CT)10	-	Introns/Intergenic	-	-	TCAATAGAACCCATACGTCAGTCG	CATGCCACGGTTACTCCTACG	200	P	A	A	RM11717
PMS599	Os_Chr01	32367962	32367979	(TC)9	(TC)9	(TC)9	(TC)9	(TC)9	(TC)9	(TC)9	(TC)9	(TC)9	(TC)8	(TC)9	LOC_Os01g56200	URR	NH2	-	TTTGACCCCCACTGTTCTTC	TACCAAACACTTTCGACCCC	184	A	A	P	NA
PMS600	Os_Chr01	32392076	32392089	(TA)7	(TA)7	(TA)7	(TA)7	(TA)6	(TA)7	(TA)7	(TA)7	(TA)7	(TA)7	(TA)7	-	Introns/Intergenic	-	-	TTGGTGTTTGGTATAGCTGTGG	TGTTGAATCATTTTATTCTTATGCG	247	P	A	A	NA
PMS601	Os_Chr01	32549219	32549242	(GAC)8	(GAC)6	(GAC)8	(GAC)8	(GAC)8	(GAC)7	(GAC)8	(GAC)8	(GAC)7	(GAC)8	(GAC)7	-	Introns/Intergenic	-	-	CCAATCCTTTCCAAATCCAACG	GCCAAATATCTAGCCTCCTATTGAGC	156	P	A	A	RM6387
PMS602	Os_Chr01	32601548	32601565	(GAG)6	(GAG)6	(GAG)6	(GAG)6	(GAG)6	(GAG)6	(GAG)6	(GAG)6	(GAG)6	(GAG)5	(GAG)6	LOC_Os01g56550	CDS	OsTFIIF2-2	CODON_DELETION	AGGGAGACGATGACCTTGG	CTGAGTTTCCTGCCTCCATC	235	A	A	P	NA
PMS603	Os_Chr01	32703084	32703097	(CA)7	(CA)8	(CA)8	(CA)7	(CA)7	(CA)7	(CA)7	(CA)8	(CA)8	(CA)7	(CA)7	-	Introns/Intergenic	-	-	ACAGGCCCAGAGTTTGGACT	CCAAGCATAAACATGGGAAA	280	P	A	A	NA
PMS604	Os_Chr01	32725195	32725214	(TTCT)5	(TTCT)6	(TTCT)6	(TTCT)6	(TTCT)6	(TTCT)6	(TTCT)5	(TTCT)6	(TTCT)6	(TTCT)6	(TTCT)6	-	Introns/Intergenic	-	-	CACATGCATCACTCCACACA	AGTTCCATGCTCAGTTGCAG	217	A	A	A	NA
PMS605	Os_Chr01	32866275	32866290	(TA)8	(TA)8	(TA)8	(TA)8	(TA)8	(TA)8	(TA)7	(TA)6	(TA)8	(TA)8	(TA)8	-	Introns/Intergenic	-	-	ATATCCGCACACATTGAGCA	CTCCATCAGATGTCGTTTGAA	247	A	A	A	NA
PMS606	Os_Chr01	32879101	32879118	(GA)9	(GA)8	(GA)9	(GA)9	(GA)9	(GA)9	(GA)9	(GA)9	(GA)9	(GA)10	(GA)9	-	Introns/Intergenic	-	-	GATTCTCTTGCGTGGGCTAC	CACGCGGTTGCAGTACAC	233	P	A	A	NA
PMS607	Os_Chr01	32928583	32928650	(TA)34	(TA)23	(TA)34	(TA)9	(TA)10	(TA)34	(TA)34	(TA)34	(TA)14	(TA)34	(TA)34	-	Introns/Intergenic	-	-	AGGCTCATGCGAGATTATCTGACC	AAATGGTCTTCAGTGCTCCAGTTCG	229	P	A	A	RM11738
PMS608	Os_Chr01	33193177	33193190	(TG)7	(TG)7	(TG)7	(TG)7	(TG)7	(TG)6	(TG)7	(TG)7	(TG)7	(TG)7	(TG)7	-	Introns/Intergenic	-	-	AATTACGTTGCTGACAGGTGG	AAATCCACCAGGTGATCGAG	222	P	A	A	NA
PMS609	Os_Chr01	33238783	33238832	(AG)25	(AG)24	(AG)25	(AG)25	(AG)25	(AG)25	(AG)25	(AG)25	(AG)25	(AG)25	(AG)25	-	Introns/Intergenic	-	-	CTCCTACATGTCCACGATAACAGC	AGGAGGGTGAGTGAATGTGTCC	197	P	A	A	RM11745
PMS610	Os_Chr01	33307368	33307415	(AT)24	(AT)24	(AT)24	(AT)24	(AT)24	(AT)24	(AT)24	(AT)24	(AT)15	(AT)24	(AT)24	-	Introns/Intergenic	-	-	ACCTCTCAGCCTATCAAGTACCC	AGGTAAAGTGCACCTTGTTTCG	513	A	A	A	RM11753

Polymorphic SSR (PMS) markers	Chromosomes	Start physical positions (bp)	End physical positions (bp)	Nipponbare	Kasalath	Nagina22	IR64	Pokkali	Bala	Tainung67	Azucena	Moroberekan	*Oryza rufipogon*	*Oryza nivara*	MSU gene locus IDs	Structural annotation	Functional annotation	SSR functional effects	Forward primers (5'-3')	Reverse primers (5'-3')	Amplified product size (bp)	Markers exhibiting polymorphism within *indica* rice	Transcrption factor genes-derived markers	Known cloned genes-derived markers	PMS markers corresponding with RM markers
PMS611	Os_Chr01	33345259	33345282	(TA)12	(TA)12	(TA)12	(TA)12	(TA)12	(TA)12	(TA)12	(TA)12	(TA)9	(TA)12	(TA)12	-	Introns/Intergenic	-	-	GTCCTTCTTCTTCC GATGTCACC	TCTTTAGTCCCGGTT AGTGTCACC	277	A	A	A	RM11754
PMS612	Os_Chr01	33481170	33481190	(GGC)7	(GGC)7	(GGC)7	(GGC)7	(GGC)7	(GGC)7	(GGC)5	(GGC)7	(GGC)7	(GGC)7	(GGC)7	LOC_Os01g57900	CDS	PPR repeat domain containing protein, putative, expressed	CODON_DELETION	CTCTTCTCGAGCAG GAACACG	TCTTGAGACTAGGTT GGCAAAGG	479	A	A	A	RM11760
PMS613	Os_Chr01	33644340	33644355	(GC)8	(GC)8	(GC)8	(GC)8	(GC)8	(GC)6	(GC)8	(GC)8	(GC)8	(GC)8	(GC)8	-	Introns/Intergenic	-	-	GGTTAGTGGCTGCA AAAGGA	CCCTGTTGACCAGTT GAGGT	148	P	A	A	NA
PMS614	Os_Chr01	33716216	33716275	(TA)30	(TA)30	(TA)11	(TA)7	(TA)30	(TA)30	(TA)8	(TA)30	(TA)8	(TA)30	(TA)7	-	Introns/Intergenic	-	-	GGCTGTGGTTGGTT TGATTTCC	TTGGAGGGATATAGC ATGGATGG	250	P	A	A	RM8138
PMS615	Os_Chr01	33820645	33820662	(GCG)6	(GCG)6	(GCG)5	(GCG)6	(GCG)6	(GCG)6	(GCG)6	(GCG)6	(GCG)6	(GCG)6	(GCG)6	LOC_Os01g58520	URR	disease resistance protein, putative, expressed	-	GGACACGAGCTTAA TCGACG	GAGGGTAAAAACCTC GCTCC	223	P	A	A	NA
PMS616	Os_Chr01	33830818	33830889	(TA)36	(TA)36	(TA)36	(TA)36	(TA)36	(TA)28	(TA)36	(TA)36	(TA)8	(TA)36	(TA)36	-	Introns/Intergenic	-	-	CACCATGGAACACC AAACAA	ACAATATGGGGCTG GAACAG	163	P	A	A	NA
PMS617	Os_Chr01	33979758	33979773	(CG)8	(CG)8	(CG)8	(CG)8	(CG)8	(CG)8	(CG)7	(CG)8	(CG)8	(CG)8	(CG)8	-	Introns/Intergenic	-	-	CCCTGTTGACCAGT TGAGGT	GAGAAAGAACGTGG ACGAGC	159	A	A	A	NA
PMS618	Os_Chr01	34012418	34012439	(CT)11	(CT)11	(CT)11	(CT)11	(CT)11	(CT)11	(CT)11	(CT)11	(CT)10	(CT)11	(CT)11	-	Introns/Intergenic	-	-	TCGAGTTCAATTCC GAATCC	GGGGGAGAGATGGG AGAG	182	A	A	A	NA
PMS619	Os_Chr01	34064089	34064124	(GA)18	(GA)19	(GA)18	(GA)18	(GA)18	(GA)18	(GA)18	(GA)11	(GA)18	(GA)18	(GA)18	-	Introns/Intergenic	-	-	AGCGGAGAAGAGG GAGAGAG	AAATCCCTCCGCAAA AGTCT	147	P	A	A	NA
PMS620	Os_Chr01	34077166	34077177	(CT)6	(CT)7	(CT)6	(CT)6	(CT)6	(CT)6	(CT)6	(CT)6	(CT)6	(CT)6	(CT)6	-	Introns/Intergenic	-	-	GTTCGATGGGCCGA ATCT	AGGCGCGGCGGATT TGAG	300	P	A	A	NA
PMS621	Os_Chr01	34103711	34103760	(TA)25	(TA)25	(TA)25	(TA)11	(TA)25	(TA)10	(TA)7	(TA)25	(TA)6	(TA)25	(TA)25	-	Introns/Intergenic	-	-	ATGCTGAGACACGG AGATTACC	GGGCTATATTGGCAC AATCG	646	P	A	A	RM11775
PMS622	Os_Chr01	34117934	34117953	(CT)10	(CT)12	(CT)10	(CT)10	(CT)10	(CT)10	(CT)10	(CT)9	(CT)10	(CT)10	(CT)10	-	Introns/Intergenic	-	-	GCAGGAGTTGGCG ACGAAGG	GTACAATAACCGGC GGTACTGCTTCC	185	P	A	A	RM11778
PMS623	Os_Chr01	34119553	34119566	(GA)7	(GA)7	(GA)7	(GA)7	(GA)7	(GA)7	(GA)6	(GA)7	(GA)7	(GA)7	(GA)7	-	Introns/Intergenic	-	-	CAACCCCGACTTGT CGTC	TCCCTCCAATGGCTG AATAG	235	A	A	A	NA

Polymorphic SSR (PMS) markers	Chromosomes	Start physical positions (bp)	End physical positions (bp)	Nipponbare	Kasalath	Nagina22	IR64	Pokkali	Bala	Tainung67	Azucena	Moroberekan	*Oryza rufipogon*	*Oryza nivara*	MSU gene locus IDs	Structural annotation	Functional annotation	SSR functional effects	Forward primers (5'-3')	Reverse primers (5'-3')	Amplified product size (bp)	Markers exhibiting polymorphism within *indica* rice	Transcrption factor genes-derived markers	Known cloned genes-derived markers	PMS markers corresponding with RM markers
PMS624	Os_Chr01	34126305	34126358	(TA)27	(TA)27	(TA)9	(TA)27	(TA)12	(TA)14	(TA)7	(TA)27	(TA)27	(TA)27	(TA)27	-	Introns/Intergenic	-	-	CGACGAACCTTCAATCTCAGACTTCG	ATGGGCCATCGTGCGTTTCC	198	P	A	A	RM8061
PMS625	Os_Chr01	34224022	34224035	(GA)7	(GA)7	(GA)7	(GA)7	(GA)7	(GA)6	(GA)7	(GA)7	(GA)7	(GA)7	(GA)7	-	Introns/Intergenic	-	-	CATCAAGGTCGTGGACGTAA	CCCACTTGTCATCCCTCTTC	202	P	A	A	NA
PMS626	Os_Chr01	34336672	34336697	(CT)13	(CT)14	(CT)14	(CT)14	(CT)14	(CT)14	(CT)14	(CT)14	(CT)15	(CT)13	(CT)11	-	Introns/Intergenic	-	-	CTCTTCTTTCGCCAGCCTCT	ACGACTGCAAAAGGACGG	265	A	A	A	NA
PMS627	Os_Chr01	34343919	34343946	(CT)14	(CT)13	(CT)13	(CT)13	(CT)13	(CT)13	(CT)13	(CT)13	(CT)13	(CT)13	(CT)11	-	Introns/Intergenic	-	-	CTCTTCTTTCGCCAGCCTCTCC	GGGACGGTTTGAAGGCAAGC	250	A	A	A	RM12334
PMS628	Os_Chr01	34379107	34379127	(GGT)7	(GGT)7	(GGT)7	(GGT)7	(GGT)7	(GGT)7	(GGT)7	(GGT)7	(GGT)7	(GGT)6	(GGT)7	LOC_Os01g59440	URR	OsLRR1	-	GCAAAGCACCCAAACCAAACC	GAAGAGGAGGAACACGAGGAAGG	225	A	A	P	RM11790
PMS629	Os_Chr01	34402674	34402701	(TA)14	(TA)14	(TA)12	(TA)6	(TA)14	(TA)14	(TA)14	(TA)14	(TA)7	(TA)14	(TA)14	-	Introns/Intergenic	-	-	CCCGAACATCCTATGTGACACC	TCTTCAGGTCGCTCTCGATGG	467	P	A	A	RM11791
PMS630	Os_Chr01	34404694	34404707	(CT)7	(CT)7	(CT)7	(CT)7	(CT)7	(CT)7	(CT)7	(CT)7	(CT)7	(CT)6	(CT)7	-	Introns/Intergenic	-	-	GCTCCCTCATCCCTCTTTCT	GTAATGTGCGTCGCGTTATG	146	A	A	A	NA
PMS631	Os_Chr01	34437785	34437850	(TA)33	(TA)10	(TA)33	(TA)33	(TA)33	(TA)33	(TA)33	(TA)33	(TA)33	(TA)33	(TA)33	-	Introns/Intergenic	-	-	GAGACTACCAAACTCCTAACTACCG	GATTCATAGGCCGAGACTGC	333	P	A	A	RM11793
PMS632	Os_Chr01	34506714	34506733	(CG)10	(CG)10	(CG)10	(CG)10	(CG)10	(CG)10	(CG)10	(CG)10	(CG)10	(CG)7	(CG)10	-	Introns/Intergenic	-	-	GATCTGCGCGTTAGTGTGATTCG	GAGGCGAGAAACGCACAGTGG	188	A	A	A	RM11795
PMS633	Os_Chr01	34513983	34513996	(AC)7	(AC)12	(AC)7	(AC)7	(AC)7	(AC)7	(AC)7	(AC)7	(AC)7	(AC)7	(AC)7	-	Introns/Intergenic	-	-	GATGGACCCTCAACCAGCTA	TCTGTATGGCCTGACACCTG	214	P	A	A	NA
PMS634	Os_Chr01	34515221	34515256	(TAC)12	(TAC)5	(TAC)9	(TAC)12	(TAC)12	(TAC)12	(TAC)12	(TAC)12	(TAC)12	(TAC)12	(TAC)12	-	Introns/Intergenic	-	-	GCTTTCCTCTCCTCTCCTCTCC	CAAATCAGTGTCGTATGCAGTGG	179	P	A	A	RM6703
PMS635	Os_Chr01	34546039	34546053	(GAG)5	(GAG)5	(GAG)5	(GAG)5	(GAG)5	(GAG)5	(GAG)5	(GAG)5	(GAG)6	(GAG)5	(GAG)5	LOC_Os01g59710	CDS	expressed protein	CODON_INSERTION	GACGTTGCCGGAATTAACAT	AGAGACTGGCCATCCTGAAG	280	A	A	A	NA
PMS636	Os_Chr01	34550018	34550031	(AT)7	(AT)7	(AT)7	(AT)7	(AT)7	(AT)7	(AT)7	(AT)6	(AT)7	(AT)7	(AT)7	-	Introns/Intergenic	-	-	CACTTTGGGCTGGTGACATA	CACGGTCGAAAGAATAGGGA	123	A	A	A	NA

Polymorphic SSR (PMS) markers	Chromosomes	Start physical positions (bp)	End physical positions (bp)	Nipponbare	Kasalath	Nagina22	IR64	Pokkali	Bala	Tainung67	Azucena	Moroberekan	*Oryza rufipogon*	*Oryza nivara*	MSU gene locus IDs	Structural annotation	Functional annotation	SSR functional effects	Forward primers (5'-3')	Reverse primers (5'-3')	Amplified product size (bp)	Markers exhibiting polymorphism within *indica* rice	Transcrption factor genes-derived markers	Known cloned genes-derived markers	PMS markers corresponding with RM markers
PMS637	Os_Chr01	34556364	34556379	(CT)8	(CT)8	(CT)8	(CT)8	(CT)8	(CT)8	(CT)7	(CT)8	(CT)8	(CT)8	(CT)8	-	Introns/Intergenic	-	-	ATCAAGGTGGCAGGCAAG	CCGTGCGTAAGCAACTAATG	261	A	A	A	NA
PMS638	Os_Chr01	34561476	34561505	(AG)15	(AG)16	(AG)16	(AG)15	(AG)15	(AG)15	(AG)15	(AG)15	(AG)15	(AG)15	(AG)15	-	Introns/Intergenic	-	-	CCGTAGTATGTTACGGACATGAGG	TGTTATGGCAGAGTGGTAGATGC	199	P	A	A	RM11799
PMS639	Os_Chr01	34694321	34694347	(GCT)9	(GCT)9	(GCT)9	(GCT)9	(GCT)6	(GCT)9	(GCT)9	(GCT)9	(GCT)9	(GCT)9	(GCT)7	LOC_Os01g60020	URR	OsNAC4	-	TCCATCCTTCTCCTCTCGTG	AGCCACCCCCATATATAGCC	165	P	P	P	NA
PMS640	Os_Chr01	34770243	34770275	(TTA)11	(TTA)9	(TTA)8	(TTA)11	(TTA)11	(TTA)9	(TTA)11	(TTA)11	(TTA)11	(TTA)11	(TTA)10	-	Introns/Intergenic	-	-	TCTGCAGCTTCTTGAGTTCTTCC	ATCAGTGCAGGCTTTAGGTTTGC	252	P	A	A	RM11808
PMS641	Os_Chr01	34823151	34823164	(CG)7	(CG)6	(CG)7	(CG)7	(CG)7	(CG)6	(CG)7	(CG)7	(CG)7	(CG)7	(CG)7	LOC_Os01g60210	CDS	transposon protein, putative, unclassified	FRAME_SHIFT	GGACCCCAACTTCTCTCCAT	AAAAGTAAAGATTGGCGGGG	250	P	A	A	NA
PMS642	Os_Chr01	34835383	34835400	(GAA)6	(GAA)6	(GAA)6	(GAA)6	(GAA)6	(GAA)6	(GAA)6	(GAA)6	(GAA)6	(GAA)5	(GAA)6	LOC_Os01g60230	URR	auxin efflux carrier component, putative, expressed	-	GTCACTCATGTTGGCAGGAA	CGGCTGGAGGATATTGACAT	269	A	A	A	NA
PMS643	Os_Chr01	34887288	34887319	(AT)16	(AT)16	(AT)16	(AT)16	(AT)16	(AT)16	(AT)16	(AT)9	(AT)16	(AT)16	(AT)14	-	Introns/Intergenic	-	-	AAATCGTTCTGGACAACGACACG	GATGATTCGCTCGGTTGTTTAGG	293	A	A	A	RM11813
PMS644	Os_Chr01	34947253	34947270	(GCG)6	(GCG)6	(GCG)6	(GCG)6	(GCG)6	(GCG)6	(GCG)6	(GCG)6	(GCG)6	(GCG)5	(GCG)6	LOC_Os01g60430	CDS	pentatricopeptide repeat-containing protein, putative, expressed	CODON_CHANGE_PLUS_CODON_DELETION	CTGCCTCGTGTCTGTCTACG	GAGATCACCGCGTTCCAC	117	A	A	A	NA
PMS645	Os_Chr01	34999106	34999121	(AC)8	(AC)7	(AC)8	(AC)7	(AC)7	(AC)7	(AC)8	(AC)8	(AC)8	(AC)8	(AC)8	-	Introns/Intergenic	-	-	GCGTACGTTGCACACACATT	TGAAGGATGTAGAGACCGGG	239	P	A	A	NA
PMS646	Os_Chr01	35028658	35028675	(CT)9	(CT)9	(CT)9	(CT)9	(CT)9	(CT)9	(CT)9	(CT)6	(CT)9	(CT)9	(CT)9	-	Introns/Intergenic	-	-	AAATAAGAGCACCCGCAATG	GGGTACCCACAATGGTTATCT	278	A	A	A	NA
PMS647	Os_Chr01	35049622	35049641	(CT)10	(CT)6	(CT)10	(CT)10	(CT)10	(CT)10	(CT)10	(CT)10	(CT)10	(CT)10	(CT)10	-	Introns/Intergenic	-	-	ATTTTACCTCCCTCGGGTTC	ACCGGTTTGGAGTGGAGC	280	P	A	A	NA
PMS648	Os_Chr01	35149619	35149636	(CGG)6	(CGG)5	(CGG)6	(CGG)6	(CGG)6	(CGG)5	(CGG)6	(CGG)6	(CGG)6	(CGG)6	(CGG)6	-	Introns/Intergenic	-	-	TTCCCCTTCTGTCTTCGCTA	GTAATGATATCCCCGGTCCC	153	P	A	A	NA
PMS649	Os_Chr01	35263114	35263131	(TA)9	(TA)9	(TA)9	(TA)9	(TA)6	(TA)9	(TA)9	(TA)9	(TA)9	(TA)9	(TA)9	-	Introns/Intergenic	-	-	TCCTCCCCTTCTTCTCTTCC	TTTGTACACCCCAAATTTACACA	275	P	A	A	NA

Polymorphic SSR (PMS) markers	Chromosomes	Start physical positions (bp)	End physical positions (bp)	Nipponbare	Kasalath	Nagina22	IR64	Pokkali	Bala	Tainung67	Azucena	Moroberekan	*Oryza rufipogon*	*Oryza nivara*	MSU gene locus IDs	Structural annotation	Functional annotation	SSR functional effects	Forward primers (5'-3')	Reverse primers (5'-3')	Amplified product size (bp)	Markers exhibiting polymorphism within *indica* rice	Transcrption factor genes-derived markers	Known cloned genes-derived markers	PMS markers corresponding with RM markers
PMS650	Os_Chr01	35323115	35323135	(CGG)7	(CGG)7	(CGG)7	(CGG)6	(CGG)7	(CGG)6	(CGG)7	(CGG)7	(CGG)7	(CGG)7	(CGG)7	LOC_Os01g61044	CDS	transmembrane amino acid transporter protein, putative, expressed	CODON_DELETION	GGGTGTACCGGAGCATGAAGTCC	AAGAAACGCGCACCAAACTCTCG	338	P	A	A	RM11829
PMS651	Os_Chr01	35463226	35463241	(TC)8	(TC)9	(TC)8	(TC)9	(TC)8	(TC)9	(TC)8	(TC)8	(TC)8	(TC)9	(TC)9	-	Introns/Intergenic	-	-	CAATGGCAGTGGTTATGACG	GAAACATTGGCAAGGACACA	273	P	A	A	NA
PMS652	Os_Chr01	35491258	35491302	(TTC)15	(TTC)15	(TTC)15	(TTC)15	(TTC)15	(TTC)15	(TTC)15	(TTC)13	(TTC)15	(TTC)15	(TTC)15	-	Introns/Intergenic	-	-	CGACATCACTAGGGCCATTT	TTCATTAAATAATGTAAACAACCCAC	278	A	A	A	NA
PMS653	Os_Chr01	35553688	35553703	(GC)8	(GC)8	(GC)8	(GC)8	(GC)8	(GC)8	(GC)8	(GC)8	(GC)8	(GC)6	(GC)8	-	Introns/Intergenic	-	-	ATCACGTCTCGTCTCGCTCT	GGCTCTCTTCCGTGACAAAG	223	A	A	A	NA
PMS654	Os_Chr01	35637831	35637850	(AT)10	(AT)10	(AT)10	(AT)10	(AT)10	(AT)10	(AT)10	(AT)10	(AT)10	(AT)10	(AT)7	-	Introns/Intergenic	-	-	GTGGGAGTACTGATCAAATCG	GACAATCAGTACACTGTTCTCAGG	630	A	A	A	RM11840
PMS655	Os_Chr01	35747664	35747675	(AC)6	(AC)7	(AC)6	(AC)6	(AC)6	(AC)6	(AC)6	(AC)6	(AC)6	(AC)6	(AC)6	-	Introns/Intergenic	-	-	GCATACACCTAGCAGCACCA	GCCTCCTCAACATCAGATCA	209	P	A	A	NA
PMS656	Os_Chr01	35770113	35770136	(CT)12	(CT)12	(CT)12	(CT)12	(CT)12	(CT)11	(CT)12	(CT)12	(CT)12	(CT)12	(CT)12	-	Introns/Intergenic	-	-	TCTTCATTGCATTGCTGCTACG	AACCAACCGTACTGTTTCTTCTCC	283	P	A	A	RM11847
PMS657	Os_Chr01	35836082	35836108	(CTC)9	(CTC)9	(CTC)9	(CTC)9	(CTC)9	(CTC)9	(CTC)9	(CTC)7	(CTC)9	(CTC)9	(CTC)9	LOC_Os01g61910	URR	KIP1, putative, expressed	-	CACCACCACGAGTAGGCGTTGC	CCGCCAGTAGCGGTAGCATTAGC	171	A	A	A	RM11848
PMS658	Os_Chr01	35912287	35912307	(CGG)7	(CGG)7	(CGG)7	(CGG)7	(CGG)5	(CGG)7	(CGG)7	(CGG)7	(CGG)7	(CGG)7	(CGG)7	LOC_Os01g62060	CDS	plant-specific domain TIGR01589 family protein, expressed	CODON_DELETION	AGTAGGCAAGCCTCAATCGATCC	GACGTACCAATCTGATGTACGAAGC	151	P	A	A	RM11851
PMS659	Os_Chr01	36059121	36059138	(CGG)6	(CGG)5	(CGG)6	(CGG)6	(CGG)6	(CGG)6	(CGG)6	(CGG)6	(CGG)6	(CGG)6	(CGG)6	LOC_Os01g62310	CDS	homeobox domain containing protein, expressed	CODON_DELETION	AAAGGTTTGGCTTACCACCA	AGAAGCAGCAGAGCTTCGAC	189	P	A	A	NA
PMS660	Os_Chr01	36253248	36253265	(CT)9	(CT)10	(CT)9	(CT)9	(CT)9	(CT)9	(CT)9	(CT)9	(CT)9	(CT)9	(CT)9	-	Introns/Intergenic	-	-	TGAAGCTGTGGAGTGTGGAC	AGTACACTGCGAGCCCATTT	213	P	A	A	NA
PMS661	Os_Chr01	36299000	36299051	(AT)26	(AT)26	(AT)7	(AT)26	(AT)26	(AT)26	(AT)26	(AT)26	(AT)26	(AT)18	(AT)24	-	Introns/Intergenic	-	-	AAGACGAGTGGTCAAACGCT	CCATCCGGATATTCACTGCT	179	P	A	A	NA
PMS662	Os_Chr01	36479385	36479396	(TA)6	(TA)7	(TA)6	(TA)6	(TA)7	(TA)7	(TA)6	(TA)6	(TA)6	(TA)6	(TA)6	-	Introns/Intergenic	-	-	TGTGCAACTCCTTGTTGGAG	TTCAATAAATCTGCTTCCATAAATGT	223	P	A	A	NA

Polymorphic SSR (PMS) markers	Chromosomes	Start physical positions (bp)	End physical positions (bp)	Nipponbare	Kasalath	Nagina22	IR64	Pokkali	Bala	Tainung67	Azucena	Moroberekan	*Oryza rufipogon*	*Oryza nivara*	MSU gene locus IDs	Structural annotation	Functional annotation	SSR functional effects	Forward primers (5'-3')	Reverse primers (5'-3')	Amplified product size (bp)	Markers exhibiting polymorphism within *indica* rice	Transcrption factor genes-derived markers	Known cloned genes-derived markers	PMS markers corresponding with RM markers
PMS663	Os_Chr01	36508498	36508539	(TA)21	(TA)20	(TA)21	(TA)21	(TA)21	(TA)21	(TA)21	(TA)8	(TA)21	(TA)21	(TA)21	-	Introns/Intergenic	-	-	GATTACGCAAGCACAACGAA	TGTTTTGCCCTAGCCAGAAT	241	P	A	A	NA
PMS664	Os_Chr01	36595682	36595703	(GA)11	(GA)12	(GA)12	(GA)11	(GA)11	(GA)11	(GA)11	(GA)11	(GA)11	(GA)11	(GA)11	-	Introns/Intergenic	-	-	GGTTTGGAGCGGAGCAAC	ATTCCTCTCGGCCCATCT	293	P	A	A	NA
PMS665	Os_Chr01	36608052	36608069	(CGG)6	(CGG)6	(CGG)6	(CGG)6	(CGG)6	(CGG)6	(CGG)6	(CGG)6	(CGG)6	(CGG)5	(CGG)6	LOC_Os01g63160	CDS	MYB family transcription factor, putative, expressed	CODON_DELETION	AGGAGGACGACGATGAGGAG	AACTAGCACCAACCCCACAG	194	A	P	A	NA
PMS666	Os_Chr01	36716299	36716313	(GCT)5	(GCT)5	(GCT)5	(GCT)5	(GCT)5	(GCT)5	(GCT)5	(GCT)7	(GCT)5	(GCT)5	(GCT)5	-	Introns/Intergenic	-	-	GAGCGGTCGTTTCGTGTAGT	CAAAAGAAGCGGCGATTAAA	148	A	A	A	NA
PMS667	Os_Chr01	36735199	36735228	(CA)15	(CA)15	(CA)15	(CA)11	(CA)15	(CA)11	(CA)15	(CA)15	(CA)15	(CA)15	(CA)15	-	Introns/Intergenic	-	-	AAGCTCATTTGAGGGTATCG	AGGGCAAGAGAAACTATTGG	431	P	A	A	RM315
PMS668	Os_Chr01	36793289	36793342	(AT)27	(AT)27	(AT)27	(AT)6	(AT)12	(AT)7	(AT)27	(AT)27	(AT)27	(AT)6	(AT)27	-	Introns/Intergenic	-	-	CCTTACTTCCTCCGTTTCACA	ACTCCTTCCGTTTCACAATG	184	P	A	A	NA
PMS669	Os_Chr01	36831471	36831484	(GA)7	(GA)7	(GA)7	(GA)7	(GA)7	(GA)6	(GA)7	(GA)7	(GA)7	(GA)7	(GA)7	-	Introns/Intergenic	-	-	TGGTTGTTTCCTCGCTCTCT	CAAGCTAATGATGCCAGCAA	232	P	A	A	NA
PMS670	Os_Chr01	37033242	37033257	(AT)8	(AT)8	(AT)8	(AT)8	(AT)8	(AT)8	(AT)8	(AT)8	(AT)8	(AT)7	(AT)8	-	Introns/Intergenic	-	-	AAACAACTTTCCGTTGGCTG	TCACGGGTTGTGCTTGTCTA	231	A	A	A	NA
PMS671	Os_Chr01	37088912	37088929	(AG)9	(AG)9	(AG)7	(AG)9	(AG)9	(AG)9	(AG)9	(AG)9	(AG)9	(AG)9	(AG)10	LOC_Os01g63910	URR	AT hook motif family protein, expressed	-	GGTGACTCGTCAAATACGCA	TTAATTGGGCCGATCTCAAG	267	P	A	A	NA
PMS672	Os_Chr01	37108116	37108131	(TG)8	(TG)8	(TG)8	(TG)8	(TG)8	(TG)7	(TG)8	(TG)8	(TG)8	(TG)8	(TG)8	-	Introns/Intergenic	-	-	AGGCGACAGGGATGGATAC	CGCTCGTTTTCCTGAAGTGT	153	P	A	A	NA
PMS673	Os_Chr01	37138453	37138470	(GT)9	(GT)10	(GT)10	(GT)10	(GT)9	(GT)10	(GT)9	(GT)9	(GT)9	(GT)9	(GT)10	-	Introns/Intergenic	-	-	TGTCCAAAGGCAGAGTTTGA	AGAGGAGGTGGTTCCGAGTT	217	P	A	A	NA
PMS674	Os_Chr01	37199462	37199479	(GCG)6	(GCG)6	(GCG)6	(GCG)6	(GCG)6	(GCG)6	(GCG)6	(GCG)6	(GCG)6	(GCG)5	(GCG)6	-	Introns/Intergenic	-	-	GTCCATCCCTTCTCGCCT	CAACTGGCACGCTCATACAT	189	A	A	A	NA
PMS675	Os_Chr01	37398380	37398415	(GA)18	(GA)18	(GA)18	(GA)18	(GA)18	(GA)18	(GA)18	(GA)12	(GA)18	(GA)18	(GA)18	-	Introns/Intergenic	-	-	AATGTAATGGTGCTGCTACAGG	TCAATGTGCCATTCCATAGG	272	A	A	A	RM5448

Polymorphic SSR (PMS) markers	Chromosomes	Start physical positions (bp)	End physical positions (bp)	Nipponbare	Kasalath	Nagina22	IR64	Pokkali	Bala	Tainung67	Azucena	Moroberekan	*Oryza rufipogon*	*Oryza nivara*	MSU gene locus IDs	Structural annotation	Functional annotation	SSR functional effects	Forward primers (5'-3')	Reverse primers (5'-3')	Amplified product size (bp)	Markers exhibiting polymorphism within *indica* rice	Transcrption factor genes-derived markers	Known cloned genes-derived markers	PMS markers corresponding with RM markers
PMS676	Os_Chr01	37433454	37433465	(TC)6	(TC)11	(TC)6	(TC)6	(TC)6	(TC)6	(TC)6	(TC)6	(TC)6	(TC)6	(TC)6	-	Introns/Intergenic	-	-	ATAAGAAGGCGAGGGACACA	CGCCTCTCTCAAGTGCTTCT	219	P	A	A	NA
PMS677	Os_Chr01	37559409	37559429	(GCA)7	(GCA)7	(GCA)7	(GCA)7	(GCA)7	(GCA)7	(GCA)7	(GCA)7	(GCA)7	(GCA)7	(GCA)5	LOC_Os01g64730	CDS	bZIP transcription factor domain containing protein, expressed	CODON_DELETION	GTGACCACAGCCTCGTCTTCC	GGAGGTCTGGAAGGAGATAACTGG	189	A	P	A	RM11927
PMS678	Os_Chr01	37588002	37588019	(CCT)6	(CCT)6	(CCT)6	(CCT)6	(CCT)6	(CCT)6	(CCT)5	(CCT)6	(CCT)6	(CCT)6	(CCT)6	LOC_Os01g64780	URR	COV1, putative, expressed	-	GTTAGATCCAGACCTTGCGG	ATCAACCGCTTGCTGCTACT	268	A	A	A	NA
PMS679	Os_Chr01	37603803	37603830	(AG)14	(AG)15	(AG)14	(AG)14	(AG)14	(AG)16	(AG)14	(AG)14	(AG)14	(AG)15	(AG)14	-	Introns/Intergenic	-	-	CTTCCCGGAAACACACACTT	GCCATGTATTGCTTCCGTTT	299	P	A	A	RM1198
PMS680	Os_Chr01	37641816	37641830	(GCG)5	(GCG)7	(GCG)5	(GCG)5	(GCG)5	(GCG)7	(GCG)5	(GCG)5	(GCG)5	(GCG)5	(GCG)5	LOC_Os01g64840	CDS	aspartic proteinase nepenthesin-1 precursor, putative, expressed	CODON_CHANGE_PLUS_CODON_INSERTION	CGAGGACGAATAAGCAGCTC	GCCGTAGCTGAAGACGTACA	212	P	A	A	NA
PMS681	Os_Chr01	37699998	37700025	(TA)14	(TA)14	(TA)14	(TA)6	(TA)6	(TA)14	(TA)14	(TA)14	(TA)14	(TA)7	(TA)14	-	Introns/Intergenic	-	-	GTAAATTCAGTAAAGCCTAAAAG	AGTAATCACCTCAGAGCATCA	196	P	A	A	NA
PMS682	Os_Chr01	37798854	37798891	(TA)19	(TA)19	(TA)19	(TA)13	(TA)19	(TA)19	(TA)19	(TA)10	(TA)10	(TA)19	(TA)19	-	Introns/Intergenic	-	-	CGGCTAGCATCATTATATTTT	AAAAGACGAGTCTAGAGTCCATA	264	P	A	A	NA
PMS683	Os_Chr01	37897156	37897211	(GA)28	(GA)28	(GA)28	(GA)28	(GA)28	(GA)28	(GA)28	(GA)24	(GA)28	(GA)28	(GA)28	-	Introns/Intergenic	-	-	ACAACTCGACGCATCACATC	CTCCTCCCATCCTCGTCC	141	A	A	A	NA
PMS684	Os_Chr01	37897382	37897402	(CGC)7	(CGC)7	(CGC)7	(CGC)7	(CGC)7	(CGC)7	(CGC)6	(CGC)7	(CGC)7	(CGC)7	(CGC)7	LOC_Os01g65320	URR	expressed protein	-	AGAGAGAGAGAGCTCACGGG	TAGGCCATTAGCCAAAGCAT	253	A	A	A	NA
PMS685	Os_Chr01	37953637	37953657	(CGG)7	(CGG)6	(CGG)7	(CGG)6	(CGG)7	(CGG)6	(CGG)7	(CGG)7	(CGG)7	(CGG)7	(CGG)7	-	Introns/Intergenic	-	-	GAAACGGAGTGAGTGCTACTGATAGG	CTGTCGCGTTCCAATTTATTAGCC	158	P	A	A	RM11954
PMS686	Os_Chr01	37962787	37962807	(GGT)7	(GGT)6	(GGT)7	(GGT)7	(GGT)7	(GGT)7	(GGT)7	(GGT)7	(GGT)7	(GGT)7	(GGT)7	LOC_Os01g65410	URR	serine hydroxymethyltransferase, mitochondrial precursor, putative, expressed	-	GGTTCTCAGATTCCACCCAA	CAACCCTAGCCCCTCTAACC	227	P	A	A	NA
PMS687	Os_Chr01	38009730	38009753	(TCT)8	(TCT)7	(TCT)8	(TCT)8	(TCT)8	(TCT)8	(TCT)8	(TCT)8	(TCT)8	(TCT)8	(TCT)8	LOC_Os01g65480	URR	dnaJ domain containing protein, expressed	-	ACTCACTCACTCACCCACACAGC	TTGGAGAGGGAAGAGAAGACACG	187	P	A	A	RM6333
PMS688	Os_Chr01	38044671	38044684	(CT)7	(CT)7	(CT)7	(CT)7	(CT)7	(CT)7	(CT)7	(CT)7	(CT)7	(CT)7	(CT)11	-	Introns/Intergenic	-	-	CAGCTCTTCTCTCCCCTCCT	CACAGCGTGAACATGACTCC	170	A	A	A	NA

Polymorphic SSR (PMS) markers	Chromosomes	Start physical positions (bp)	End physical positions (bp)	Nipponbare	Kasalath	Nagina22	IR64	Pokkali	Bala	Tainung67	Azucena	Moroberekan	*Oryza rufipogon*	*Oryza nivara*	MSU gene locus IDs	Structural annotation	Functional annotation	SSR functional effects	Forward primers (5'-3')	Reverse primers (5'-3')	Amplified product size (bp)	Markers exhibiting polymorphism within *indica* rice	Transcrption factor genes-derived markers	Known cloned genes-derived markers	PMS markers corresponding with RM markers
PMS689	Os_Chr01	38049702	38049719	(TC)9	(TC)9	(TC)9	(TC)8	(TC)9	(TC)8	(TC)9	(TC)9	(TC)9	(TC)9	(TC)8	-	Introns/Intergenic	-	-	CCACAAGGTCTCCC TTACCA	AAAGCTTCCATGCCA TTCAC	160	P	A	A	NA
PMS690	Os_Chr01	38054912	38054937	(AG)13	(AG)12	(AG)13	(AG)13	(AG)11	(AG)12	(AG)13	(AG)13	(AG)13	(AG)13	(AG)11	-	Introns/Intergenic	-	-	GTCATGTCTCTCCC ATCAGTTCC	CCATCTTCGTATAGA ACTGGACATGC	311	P	A	A	RM5382
PMS691	Os_Chr01	38075351	38075364	(AG)7	(AG)11	(AG)7	(AG)7	(AG)7	(AG)11	(AG)7	(AG)7	(AG)7	(AG)7	(AG)7	-	Introns/Intergenic	-	-	TCTATGAGCTCCTC GGTTGG	AACGAGGAGACAGT GGCG	235	P	A	A	NA
PMS692	Os_Chr01	38205487	38205502	(AT)8	(AT)10	(AT)8	(AT)8	(AT)8	(AT)8	(AT)8	(AT)8	(AT)8	(AT)8	(AT)8	-	Introns/Intergenic	-	-	AAAAGACGAGTCTA GAGCCCA	AAATTTTGCAATAAC TTTACGAAAC	155	P	A	A	NA
PMS693	Os_Chr01	38209722	38209751	(GA)15	(GA)16	(GA)15	(GA)15	(GA)15	(GA)15	(GA)15	(GA)15	(GA)15	(GA)15	(GA)15	-	Introns/Intergenic	-	-	CTCGTCGTTGGGAG TAGGTGACG	GCCGCTCGTCGTTGT CTTCC	341	P	A	A	RM11965
PMS694	Os_Chr01	38291516	38291529	(CT)7	(CT)7	(CT)7	(CT)7	(CT)7	(CT)7	(CT)7	(CT)7	(CT)7	(CT)6	(CT)7	-	Introns/Intergenic	-	-	TCCTCTCCTCTCCC TTCCTC	CGACTACTTCTCGTG GCTCC	235	A	A	A	NA
PMS695	Os_Chr01	38326196	38326216	(CTC)7	(CTC)7	(CTC)7	(CTC)7	(CTC)7	(CTC)7	(CTC)7	(CTC)7	(CTC)7	(CTC)6	(CTC)7	-	Introns/Intergenic	-	-	TAGAGCGCCTTCCT CCCTCTCC	TGAGCAGCTTCTTCC ACGACACG	321	A	A	A	RM11972
PMS696	Os_Chr01	38338613	38338644	(TA)16	(TA)16	(TA)16	(TA)16	(TA)16	(TA)16	(TA)6	(TA)16	(TA)6	(TA)16	(TA)11	-	Introns/Intergenic	-	-	GATCTCTCGGTGCA CATTCTCC	AGGAGTGGTTGAGA TTGGTGTGG	280	A	A	A	RM11973
PMS697	Os_Chr01	38424600	38424617	(GA)9	(GA)9	(GA)9	(GA)9	(GA)9	(GA)9	(GA)9	(GA)9	(GA)9	(GA)8	(GA)9	LOC_Os01g66140	URR	plus-3 domain containing protein, putative, expressed	-	AAACCCTCGTCGTC TTCTCC	GGACGCAGAAATAG AAAGCG	250	A	A	A	NA
PMS698	Os_Chr01	38440022	38440045	(CCT)8	(CCT)8	(CCT)8	(CCT)6	(CCT)8	(CCT)7	(CCT)8	(CCT)8	(CCT)8	(CCT)8	(CCT)8	LOC_Os01g66170	CDS	SNARE associated Golgi protein, putative, expressed	CODON_DELETION	GGGTGAGGAAGGT GTTGATCTGG	GGTCCTCTCCTCCCA CTTCTCG	383	P	A	A	RM11980
PMS699	Os_Chr01	38452511	38452526	(AG)8	(AG)7	(AG)8	(AG)8	(AG)8	(AG)8	(AG)6	(AG)8	(AG)8	(AG)8	(AG)7	-	Introns/Intergenic	-	-	AAACCGAAACCTCT CCCACT	ACTAAACACACCCGG AGACG	267	P	A	A	NA
PMS700	Os_Chr01	38475157	38475180	(CCT)8	(CCT)6	(CCT)8	(CCT)8	(CCT)6	(CCT)6	(CCT)8	(CCT)8	(CCT)8	(CCT)8	(CCT)8	LOC_Os01g66230	CDS	csAtPR5, putative, expressed	CODON_DELETION	CGCGTTCTCCCTTC TTTCTACTCG	GTTACATGGGCCTCC AGCATAGG	195	P	A	A	RM11982
PMS701	Os_Chr01	38504572	38504592	(GCG)7	(GCG)7	(GCG)7	(GCG)7	(GCG)7	(GCG)7	(GCG)5	(GCG)7	(GCG)7	(GCG)7	(GCG)7	LOC_Os01g66290	CDS	OsMADS21	CODON_DELETION	CGGCGACGTTCGAC ACAAGG	GCCGGAGTCGACCT TGAAGTAGC	146	A	P	P	RM11984

Polymorphic SSR (PMS) markers	Chromosomes	Start physical positions (bp)	End physical positions (bp)	Nipponbare	Kasalath	Nagina22	IR64	Pokkali	Bala	Tainung67	Azucena	Moroberekan	*Oryza rufipogon*	*Oryza nivara*	MSU gene locus IDs	Structural annotation	Functional annotation	SSR functional effects	Forward primers (5'-3')	Reverse primers (5'-3')	Amplified product size (bp)	Markers exhibiting polymorphism within *indica* rice	Transcrption factor genes-derived markers	Known cloned genes-derived markers	PMS markers corresponding with RM markers
PMS702	Os_Chr01	38597672	38597689	(CT)9	(CT)9	(CT)9	(CT)9	(CT)8	(CT)9	(CT)9	(CT)9	(CT)9	(CT)9	(CT)9	-	Introns/Intergenic	-	-	GACGGAGCGAGAG AGGAAG	TAATTCCTCCCCAAG TTCCC	197	P	A	A	NA
PMS703	Os_Chr01	38611898	38611918	(GGA)7	(GGA)7	(GGA)7	(GGA)7	(GGA)7	(GGA)7	(GGA)7	(GGA)6	(GGA)7	(GGA)7	(GGA)7	LOC_Os01g66490	CDS	no apical meristem protein, putative, expressed	CODON_DELETION	AACTAGCCCTAGCG ACGGATGC	AGGTCATCCTCATGC CCATCTACC	200	A	P	A	RM11987
PMS704	Os_Chr01	38616723	38616740	(CG)9	(CG)7	(CG)7	(CG)9	(CG)7	(CG)9	(CG)9	(CG)9	(CG)9	(CG)7	(CG)7	-	Introns/Intergenic	-	-	ACCTCCCCTCGCAT TACAC	CTAGCTGCCTCACCT CACCT	217	P	A	A	NA
PMS705	Os_Chr01	38617322	38617333	(GA)6	(GA)8	(GA)6	(GA)6	(GA)6	(GA)6	(GA)6	(GA)6	(GA)6	(GA)7	(GA)6	-	Introns/Intergenic	-	-	ACTGATCTCGATCT GGACGG	GGATCTCTTGCCAGT TGCC	199	P	A	A	NA
PMS706	Os_Chr01	38675211	38675238	(TA)14	(TA)14	(TA)14	(TA)14	(TA)14	(TA)14	(TA)14	(TA)6	(TA)6	(TA)8	(TA)14	-	Introns/Intergenic	-	-	CTCAAGGGTTAACC ACAATGG	ATACACCAACGACTT CAACACC	454	A	A	A	RM11993
PMS707	Os_Chr01	38675519	38675562	(TA)22	(TA)22	(TA)22	(TA)22	(TA)22	(TA)22	(TA)10	(TA)22	(TA)14	(TA)18	(TA)22	-	Introns/Intergenic	-	-	TCGCTCATCGATCT TATAGCA	CACCAACGACTTCAA CACCA	230	A	A	A	NA
PMS708	Os_Chr01	38787107	38787178	(AT)36	(AT)36	(AT)36	(AT)36	(AT)36	(AT)36	(AT)36	(AT)36	(AT)7	(AT)36	(AT)36	-	Introns/Intergenic	-	-	GGGAACTCCATACT GCTATGTCG	AGTAGGGTATCGTC GGTGTCAGG	184	A	A	A	RM11998
PMS709	Os_Chr01	38799865	38799878	(AT)7	(AT)8	(AT)7	(AT)7	(AT)7	(AT)7	(AT)7	(AT)7	(AT)7	(AT)7	(AT)7	-	Introns/Intergenic	-	-	GATGCAGTTCAGGC TGTGTT	GCGAGACGAATCTTT TGAGC	259	P	A	A	NA
PMS710	Os_Chr01	38810540	38810557	(GAG)6	(GAG)5	(GAG)5	(GAG)5	(GAG)5	(GAG)5	(GAG)6	(GAG)5	(GAG)6	(GAG)5	(GAG)5	-	Introns/Intergenic	-	-	AGAACACGTACGAG GTGGGT	CTACGGCGACAGAT CACAGA	235	A	A	A	NA
PMS711	Os_Chr01	38875579	38875600	(AT)11	(AT)11	(AT)11	(AT)11	(AT)11	(AT)11	(AT)11	(AT)11	(AT)7	(AT)11	(AT)11	-	Introns/Intergenic	-	-	CCATGTTCCCAACC TATCTTCC	TGTGTTCGGATTCTT GTGATGG	228	A	A	A	RM12007
PMS712	Os_Chr01	38887576	38887589	(GA)7	(GA)7	(GA)7	(GA)7	(GA)7	(GA)7	(GA)7	(GA)6	(GA)7	(GA)7	(GA)7	LOC_Os01g66940	URR	OsFKI	-	GTCTATCAGCATCT CCCCGA	GCCATCCGATCTCAT CTCAT	266	A	A	P	NA
PMS713	Os_Chr01	38903054	38903069	(TA)8	(TA)8	(TA)6	(TA)8	(TA)8	(TA)8	(TA)8	(TA)8	(TA)8	(TA)8	(TA)8	-	Introns/Intergenic	-	-	ACTTTAAATGGGCC TTTGGG	AACAAGGTGATACG GCTTCA	248	P	A	A	NA
PMS714	Os_Chr01	38986718	38986741	(CCG)8	(CCG)8	(CCG)5	(CCG)8	(CCG)8	(CCG)8	(CCG)8	(CCG)8	(CCG)8	(CCG)8	(CCG)8	LOC_Os01g67170	URR	expressed protein	-	ATGTGAATTGGGTG GCAAAT	GAGAGGCGGTAGTA TGCTCG	137	P	A	A	NA

Polymorphic SSR (PMS) markers	Chromosomes	Start physical positions (bp)	End physical positions (bp)	Nipponbare	Kasalath	Nagina22	IR64	Pokkali	Bala	Tainung67	Azucena	Moroberekan	*Oryza rufipogon*	*Oryza nivara*	MSU gene locus IDs	Structural annotation	Functional annotation	SSR functional effects	Forward primers (5'-3')	Reverse primers (5'-3')	Amplified product size (bp)	Markers exhibiting polymorphism within *indica* rice	Transcrption factor genes-derived markers	Known cloned genes-derived markers	PMS markers corresponding with RM markers
PMS715	Os_Chr01	39147562	39147581	(CT)10	(CT)9	(CT)10	(CT)9	(CT)10	(CT)9	(CT)9	(CT)10	(CT)10	(CT)10	(CT)9	-	Introns/Intergenic	-	-	AAGGAGAAGTGGAGGTTAAGATAGGG	GTTAATTTACTGACCGGCCATGC	279	P	A	A	RM12024
PMS716	Os_Chr01	39151379	39151394	(CT)8	(CT)8	(CT)8	(CT)8	(CT)8	(CT)8	(CT)8	(CT)8	(CT)7	(CT)8	(CT)8	-	Introns/Intergenic	-	-	GGACCATCAAAAAGCAGCTC	GCGGGTGATGATGTGATATG	212	A	A	A	NA
PMS717	Os_Chr01	39174890	39174917	(TCCA)7	(TCCA)7	(TCCA)7	(TCCA)7	(TCCA)7	(TCCA)6	(TCCA)7	(TCCA)7	(TCCA)7	(TCCA)6	(TCCA)7	-	Introns/Intergenic	-	-	GGAATGATCAAGGCATCTCC	ATTTAATTGGGTGGGGAAGG	210	P	A	A	NA
PMS718	Os_Chr01	39176827	39176856	(GA)15	(GA)15	(GA)15	(GA)15	(GA)8	(GA)15	(GA)15	(GA)15	(GA)15	(GA)15	(GA)15	-	Introns/Intergenic	-	-	AGGTTAGATTGAGACAGAGACACC	AAGTTAGCCTCGCTCTTCTACC	309	P	A	A	RM12030
PMS719	Os_Chr01	39187264	39187279	(AT)8	(AT)8	(AT)7	(AT)7	(AT)8	(AT)7	(AT)7	(AT)8	(AT)8	(AT)7	(AT)7	-	Introns/Intergenic	-	-	GATCCTTCAGAGCAAGCAGC	TGTTCGTAAGGGTTATGCAAGA	198	P	A	A	NA
PMS720	Os_Chr01	39243852	39243866	(CGG)5	(CGG)5	(CGG)5	(CGG)5	(CGG)5	(CGG)5	(CGG)5	(CGG)5	(CGG)5	(CGG)7	(CGG)5	LOC_Os01g67510	URR	recA protein, putative, expressed	-	GCTGCCAGTAGGAACACTCC	CCTAAACCCTAGCAATCCCC	256	A	A	A	NA
PMS721	Os_Chr01	39310714	39310731	(TA)9	(TA)9	(TA)8	(TA)6	(TA)9	(TA)9	(TA)9	(TA)9	(TA)9	(TA)9	(TA)9	-	Introns/Intergenic	-	-	GATCGGAAACCGGTAACAAA	TCTCGTGGAAATTACCGTTTCT	277	P	A	A	NA
PMS722	Os_Chr01	39490525	39490542	(TAT)6	(TAT)6	(TAT)6	(TAT)7	(TAT)6	(TAT)7	(TAT)6	(TAT)6	(TAT)6	(TAT)6	(TAT)6	-	Introns/Intergenic	-	-	TGGAATCCCTTTCCAGTTTG	ATGCTGCTTCGAGACCTTGT	266	P	A	A	NA
PMS723	Os_Chr01	39508571	39508591	(CGC)7	(CGC)5	(CGC)7	(CGC)7	(CGC)7	(CGC)7	(CGC)7	(CGC)7	(CGC)7	(CGC)7	(CGC)7	-	Introns/Intergenic	-	-	CTCTCGTCGCACTCGACACC	GAGAAACACGACAGGTTGTTACCG	196	P	A	A	RM12046
PMS724	Os_Chr01	39518589	39518626	(TA)19	(TA)19	(TA)19	(TA)19	(TA)19	(TA)19	(TA)19	(TA)19	(TA)19	(TA)12	(TA)19	-	Introns/Intergenic	-	-	ACGGGCACCTTTGCTTATTT	AGTGCCAATGTTTTGTGCTG	279	A	A	A	NA
PMS725	Os_Chr01	39525033	39525053	(GGC)7	(GGC)7	(GGC)7	(GGC)7	(GGC)7	(GGC)7	(GGC)7	(GGC)5	(GGC)7	(GGC)7	(GGC)7	LOC_Os01g68000	CDS	PLA2|LHD2	CODON_DELETION	GGCTTCAGCATCGTCGTCTACC	CTCGGGTTCTTTGAACAGGAAGC	130	A	A	P	RM12049
PMS726	Os_Chr01	39569722	39569742	(CCG)7	(CCG)5	(CCG)7	(CCG)7	(CCG)7	(CCG)5	(CCG)7	(CCG)7	(CCG)5	(CCG)7	(CCG)7	LOC_Os01g68060	CDS	copine, putative, expressed	CODON_DELETION	GCCGTAGTAGCCTCCTCCATGC	GATCGATCGGTTGGTTGGTTGG	224	P	A	A	RM12054
PMS727	Os_Chr01	39606147	39606164	(CGG)6	(CGG)6	(CGG)6	(CGG)5	(CGG)6	(CGG)6	(CGG)6	(CGG)6	(CGG)6	(CGG)6	(CGG)6	-	Introns/Intergenic	-	-	AAAGAGGATGTGCGTAACGG	CCATTTTGACGTAGTGGCCT	276	P	A	A	NA

Polymorphic SSR (PMS) markers	Chromosomes	Start physical positions (bp)	End physical positions (bp)	Nipponbare	Kasalath	Nagina22	IR64	Pokkali	Bala	Tainung67	Azucena	Moroberekan	*Oryza rufipogon*	*Oryza nivara*	MSU gene locus IDs	Structural annotation	Functional annotation	SSR functional effects	Forward primers (5'-3')	Reverse primers (5'-3')	Amplified product size (bp)	Markers exhibiting polymorphism within *indica* rice	Transcrption factor genes-derived markers	Known cloned genes-derived markers	PMS markers corresponding with RM markers
PMS728	Os_Chr01	39613820	39613837	(GCC)6	(GCC)6	(GCC)6	(GCC)6	(GCC)6	(GCC)6	(GCC)6	(GCC)6	(GCC)8	(GCC)6	(GCC)6	LOC_Os01g68140	CDS	expressed protein	CODON_INSERTION	ACGTAGTAGTCGGACAGCGG	GACAAGATCGGCAGGGAGTA	239	A	A	A	NA
PMS729	Os_Chr01	39644806	39644826	(CCG)7	(CCG)9	(CCG)7	(CCG)9	(CCG)7	(CCG)9	(CCG)7	(CCG)7	(CCG)9	(CCG)9	(CCG)7	LOC_Os01g68210	CDS	expressed protein	CODON_INSERTION	GAGTCATCCAGCACCAAGTACAGG	TCACGATATAGCACGGGATCAGC	180	P	A	A	RM12056
PMS730	Os_Chr01	39679691	39679708	(CA)9	(CA)9	(CA)9	(CA)9	(CA)9	(CA)9	(CA)9	(CA)9	(CA)6	(CA)9	(CA)9	LOC_Os01g68290	DRR	expressed protein	-	ACACACCATGTACACGACCG	CAGTACGACTCCTGCACCG	138	A	A	A	NA
PMS731	Os_Chr01	39736986	39737003	(CT)9	(CT)8	(CT)9	(CT)9	(CT)9	(CT)9	(CT)9	(CT)9	(CT)9	(CT)8	(CT)9	-	Introns/Intergenic	-	-	ACTGGTTACACCGGAAGTGG	ATTTAGCCTTGGCTTCGGAT	237	P	A	A	NA
PMS732	Os_Chr01	39793227	39793295	(TTA)23	(TTA)23	(TTA)9	(TTA)23	(TTA)23	(TTA)23	(TTA)5	(TTA)23	(TTA)23	(TTA)23	(TTA)19	-	Introns/Intergenic	-	-	GTCGGTTTGGGAATTTGACTAGTAGG	TAATTGTGGACTGCTCGTTTCTGG	392	P	A	A	RM12061
PMS733	Os_Chr01	39810245	39810264	(CACG)5	(CACG)5	(CACG)5	(CACG)5	(CACG)5	(CACG)5	(CACG)5	(CACG)7	(CACG)5	(CACG)5	(CACG)5	-	Introns/Intergenic	-	-	TCCGTTTCGCAATAAACACA	CGCGTATGTGAAAGTAGGCA	235	A	A	A	NA
PMS734	Os_Chr01	39824122	39824185	(CT)32	(CT)32	(CT)32	(CT)32	(CT)32	(CT)32	(CT)32	(CT)23	(CT)32	(CT)32	(CT)32	-	Introns/Intergenic	-	-	TCCTGGCTGCTATTGTGGTTGG	AAGTCGTTCGTTGCATGAAGAGG	147	A	A	A	RM3523
PMS735	Os_Chr01	39897671	39897688	(GTG)6	(GTG)6	(GTG)6	(GTG)5	(GTG)6	(GTG)5	(GTG)6	(GTG)6	(GTG)6	(GTG)6	(GTG)6	LOC_Os01g68700	URR	BHLH transcription factor, putative, expressed	-	ATTGGGAGTGTGAGCCTACG	TCACTCACCTCTTGCCTGTG	219	P	P	A	NA
PMS736	Os_Chr01	39928691	39928710	(CT)10	(CT)10	(CT)11	(CT)11	(CT)10	(CT)11	(CT)10	(CT)10	(CT)10	(CT)10	(CT)10	-	Introns/Intergenic	-	-	CTCAGACACGCATGCATTAAACC	CTATGGGCGTTCATTAGCTAGACC	229	P	A	A	RM12071
PMS737	Os_Chr01	40037384	40037401	(GGT)6	(GGT)6	(GGT)6	(GGT)5	(GGT)5	(GGT)6	(GGT)6	(GGT)6	(GGT)6	(GGT)6	(GGT)6	LOC_Os01g68900	URR	zinc finger, C3HC4 type family protein, expressed	-	CTCCATCTCTCAGCTCGCTC	TTCCCTCCTCTCCTCTCCTC	193	P	A	A	NA
PMS738	Os_Chr01	40138555	40138590	(AT)18	(AT)18	(AT)18	(AT)18	(AT)18	(AT)18	(AT)10	(AT)18	(AT)18	(AT)18	(AT)18	-	Introns/Intergenic	-	-	TAGACAAAAACCTTTGGGCG	AGTGCTTTTAGCCCTTGCTG	173	A	A	A	NA
PMS739	Os_Chr01	40230317	40230334	(CCG)6	(CCG)6	(CCG)6	(CCG)6	(CCG)6	(CCG)6	(CCG)5	(CCG)6	(CCG)6	(CCG)6	(CCG)6	LOC_Os01g69220	URR	exostosin family domain containing protein, expressed	-	ACAAGGAGAGAGAGGGAGGC	AAGACGAGGAGCAGGAGGAG	264	A	A	A	NA
PMS740	Os_Chr01	40282863	40282883	(CCA)7	(CCA)5	(CCA)5	(CCA)5	(CCA)7	(CCA)5	(CCA)6	(CCA)7	(CCA)7	(CCA)7	(CCA)5	-	Introns/Intergenic	-	-	TCCTCCTCCTCCTCCTCTTC	CACTTGTTGGTGGTTGGTTG	194	P	A	A	NA

Polymorphic SSR (PMS) markers	Chromosomes	Start physical positions (bp)	End physical positions (bp)	Nipponbare	Kasalath	Nagina22	IR64	Pokkali	Bala	Tainung67	Azucena	Moroberekan	*Oryza rufipogon*	*Oryza nivara*	MSU gene locus IDs	Structural annotation	Functional annotation	SSR functional effects	Forward primers (5'-3')	Reverse primers (5'-3')	Amplified product size (bp)	Markers exhibiting polymorphism within *indica* rice	Transcrption factor genes-derived markers	Known cloned genes-derived markers	PMS markers corresponding with RM markers
PMS741	Os_Chr01	40365868	40365891	(AAG)8	(AAG)8	(AAG)8	(AAG)8	(AAG)8	(AAG)8	(AAG)8	(AAG)8	(AAG)8	(AAG)5	(AAG)8	-	Introns/Intergenic	-	-	GCATTGCTGGGTTGACCTGTCC	CGATCTGCGTCTTCTTCCATTCC	235	A	A	A	RM12105
PMS742	Os_Chr01	40409601	40409616	(CT)8	(CT)6	(CT)8	(CT)8	(CT)8	(CT)6	(CT)8	(CT)8	(CT)8	(CT)8	(CT)8	LOC_Os01g69920	URR	OHK2|OsHk3	-	CACAACAGTGAGAGAGGCCA	GTGGTGAGGTGAGGTGGAGT	163	P	A	P	NA
PMS743	Os_Chr01	40425808	40425819	(CA)6	(CA)7	(CA)7	(CA)6	(CA)6	(CA)6	(CA)6	(CA)6	(CA)6	(CA)6	(CA)6	-	Introns/Intergenic	-	-	GCTAGCAACCTCTGAATCGC	ACAATGGGGGAAATCGTACA	227	P	A	A	NA
PMS744	Os_Chr01	40434152	40434173	(AT)11	(AT)11	(AT)7	(AT)11	(AT)11	(AT)11	(AT)11	(AT)11	(AT)11	(AT)11	(AT)11	-	Introns/Intergenic	-	-	TCCAGATGGATGAGAGTAGTGACC	GTACATCCATCATCGATCATCACC	377	P	A	A	RM12112
PMS745	Os_Chr01	40475113	40475132	(AG)10	(AG)14	(AG)10	(AG)10	(AG)10	(AG)10	(AG)10	(AG)10	(AG)10	(AG)10	(AG)10	-	Introns/Intergenic	-	-	TTATTCCCTCTCCTCTCACTCACC	GCAATACGCATTCACTTCATCG	500	P	A	A	RM12120
PMS746	Os_Chr01	40475675	40475698	(TTTA)6	(TTTA)6	(TTTA)6	(TTTA)6	(TTTA)6	(TTTA)6	(TTTA)6	(TTTA)6	(TTTA)6	(TTTA)6	(TTTA)5	-	Introns/Intergenic	-	-	TCGGATCATCGGAATAGCAGACC	CCGGTACTACGAACTTGCACTCG	442	A	A	A	RM12121
PMS747	Os_Chr01	40478359	40478379	(CAG)7	(CAG)6	(CAG)7	(CAG)7	(CAG)7	(CAG)6	(CAG)7	(CAG)7	(CAG)6	(CAG)7	(CAG)6	-	Introns/Intergenic	-	-	GTCGCTCAGCCAGTTCTACC	CCCATGAAGAACAACCCAAC	277	P	A	A	NA
PMS748	Os_Chr01	40500771	40500790	(AT)10	(AT)9	(AT)10	(AT)10	(AT)10	(AT)10	(AT)10	(AT)10	(AT)10	(AT)10	(AT)10	-	Introns/Intergenic	-	-	GTGTTCGATTCCTTGCTTTAGAGG	ACGAGACGAGACGATACGAGAGC	658	P	A	A	RM12125
PMS749	Os_Chr01	40509244	40509264	(CGG)7	(CGG)5	(CGG)7	(CGG)7	(CGG)7	(CGG)7	(CGG)7	(CGG)7	(CGG)7	(CGG)7	(CGG)7	-	Introns/Intergenic	-	-	TCTCCAACCTCGACCAGAAC	GCAGTCCACGATAAGCTTCC	191	P	A	A	NA
PMS750	Os_Chr01	40537051	40537120	(AT)35	(AT)35	(AT)35	(AT)35	(AT)35	(AT)35	(AT)6	(AT)35	(AT)35	(AT)35	(AT)35	-	Introns/Intergenic	-	-	TCTTGCGTGTGAAGATCTTGTT	TGAGGAGGATGTACCGTTGA	267	A	A	A	NA
PMS751	Os_Chr01	40556876	40556896	(GCC)7	(GCC)6	(GCC)7	(GCC)7	(GCC)7	(GCC)6	(GCC)7	(GCC)6	(GCC)6	(GCC)6	(GCC)7	LOC_Os01g70080	CDS	NB-ARC domain containing protein, expressed	CODON_DELETION	TAGGCTCGGGATGGTGAACACG	CTGATCCTGAAGGGCAGGAACG	354	P	P	A	RM12133
PMS752	Os_Chr01	40561036	40561050	(GCG)5	(GCG)6	(GCG)5	(GCG)5	(GCG)5	(GCG)6	(GCG)5	(GCG)5	(GCG)6	(GCG)5	(GCG)5	LOC_Os01g70080	CDS	NB-ARC domain containing protein, expressed	CODON_INSERTION	GCGTGTGCTCGATGTTGTAG	CTCGACGACTTCCTCCACC	220	P	P	A	NA
PMS753	Os_Chr01	40574179	40574199	(GAT)7	(GAT)6	(GAT)6	(GAT)6	(GAT)6	(GAT)6	(GAT)7	(GAT)7	(GAT)7	(GAT)7	(GAT)6	-	Introns/Intergenic	-	-	TAAAGTGGGTGAAGAGGAGAAAGC	ACAGGTTCCATATTCCTGTTGC	183	A	A	A	RM12136

Polymorphic SSR (PMS) markers	Chromosomes	Start physical positions (bp)	End physical positions (bp)	Nipponbare	Kasalath	Nagina22	IR64	Pokkali	Bala	Tainung67	Azucena	Moroberekan	*Oryza rufipogon*	*Oryza nivara*	MSU gene locus IDs	Structural annotation	Functional annotation	SSR functional effects	Forward primers (5'-3')	Reverse primers (5'-3')	Amplified product size (bp)	Markers exhibiting polymorphism within *indica* rice	Transcrption factor genes-derived markers	Known cloned genes-derived markers	PMS markers corresponding with RM markers
PMS754	Os_Chr01	40687181	40687194	(TC)7	(TC)9	(TC)7	(TC)7	(TC)7	(TC)9	(TC)7	(TC)7	(TC)7	(TC)7	(TC)9	-	Introns/Intergenic	-	-	GCAACTCAGTAGTGGGCCTC	AAGCACATCCATCCATCCAT	134	P	A	A	NA
PMS755	Os_Chr01	40701059	40701088	(GGGGAT)5	(GGGGAT)5	(GGGGAT)5	(GGGGAT)5	(GGGGAT)5	(GGGGAT)5	(GGGGAT)5	(GGGGAT)5	(GGGGAT)5	(GGGGAT)6	(GGGGAT)6	-	Introns/Intergenic	-	-	GGTGTGGAAAAGCAGAAAGC	TGATAAGGGAGAGGAGGCAA	242	A	A	A	NA
PMS756	Os_Chr01	40712794	40712808	(CTC)5	(CTC)6	(CTC)5	(CTC)5	(CTC)5	(CTC)5	(CTC)5	(CTC)5	(CTC)5	(CTC)5	(CTC)5	LOC_Os01g70300	CDS	aspartokinase 3, chloroplast precursor, putative, expressed	CODON_INSERTION	ACAGCGAGCGGAGTCATC	CTCCCCTCTCCCTCTCTCAC	262	P	A	A	NA
PMS757	Os_Chr01	40749957	40749977	(TGG)7	(TGG)6	(TGG)7	(TGG)6	(TGG)7	(TGG)7	(TGG)6	(TGG)7	(TGG)7	(TGG)7	(TGG)7	LOC_Os01g70340	CDS	expressed protein	CODON_DELETION	ATCGCCGGAGCTCCTCAACG	CGCCGCCAAGCAGTACAACG	157	P	A	A	RM12150
PMS758	Os_Chr01	40751029	40751044	(TA)8	(TA)9	(TA)8	(TA)8	(TA)8	(TA)8	(TA)7	(TA)8	(TA)8	(TA)8	(TA)8	-	Introns/Intergenic	-	-	GGAGCCTAGCTTCTCACCCT	TGCACCACCCTTAATCCTTC	260	P	A	A	NA
PMS759	Os_Chr01	40790624	40790637	(GT)7	(GT)6	(GT)6	(GT)6	(GT)6	(GT)6	(GT)7	(GT)7	(GT)7	(GT)7	(GT)6	-	Introns/Intergenic	-	-	TCCTGTTCTTCAGGTGGGTC	TTACCTGCAACAGCATCAGC	217	A	A	A	NA
PMS760	Os_Chr01	40820406	40820451	(TA)23	(TA)23	(TA)6	(TA)23	(TA)23	(TA)23	(TA)9	(TA)23	(TA)6	(TA)23	(TA)23	-	Introns/Intergenic	-	-	GCAATCAGTTGGAACTTGGAAGC	CAGATCACCTGAGCTGTGTTTGC	349	P	A	A	RM12157
PMS761	Os_Chr01	40831415	40831440	(CT)13	(CT)13	(CT)8	(CT)8	(CT)8	(CT)13	(CT)13	(CT)13	(CT)13	(CT)13	(CT)13	-	Introns/Intergenic	-	-	TCCTCAACCGTAAAACCAGG	TCGAAGTTTGTGTGTGCTCC	233	P	A	A	NA
PMS762	Os_Chr01	40940464	40940481	(GT)9	(GT)9	(GT)9	(GT)6	(GT)9	(GT)9	(GT)9	(GT)9	(GT)9	(GT)9	(GT)9	LOC_Os01g70720	URR	expressed protein	-	CACCCCTACTTGAACTGGGA	AGGGAGAGTGGTCATGATGC	193	P	A	A	NA
PMS763	Os_Chr01	40942694	40942711	(GGC)6	(GGC)6	(GGC)6	(GGC)6	(GGC)6	(GGC)5	(GGC)6	(GGC)6	(GGC)6	(GGC)6	(GGC)5	LOC_Os01g70730	CDS	flowering promoting factor-like 1, putative, expressed	CODON_DELETION	CTTGTGGAACTGGAGCATCATGG	ATTTATACATGTCGGGCGTGTGG	269	P	P	A	RM12162
PMS764	Os_Chr01	40942842	40942871	(CCG)10	(CCG)10	(CCG)5	(CCG)10	(CCG)10	(CCG)10	(CCG)10	(CCG)10	(CCG)10	(CCG)8	(CCG)10	LOC_Os01g70730	CDS	flowering promoting factor-like 1, putative, expressed	CODON_DELETION	CTTGTGGAACTGGAGCATCA	TTATACATGTCGGGCGTGTG	276	P	P	A	NA
PMS765	Os_Chr01	40994263	40994283	(GCA)7	(GCA)7	(GCA)7	(GCA)7	(GCA)7	(GCA)7	(GCA)7	(GCA)7	(GCA)7	(GCA)7	(GCA)5	-	Introns/Intergenic	-	-	TTTCTCTCGCGAAAACGTC	CATCGCGGAGCAGAAAAG	102	A	A	A	NA
PMS766	Os_Chr01	40994619	40994632	(AC)7	(AC)8	(AC)7	(AC)7	(AC)7	(AC)7	(AC)7	(AC)7	(AC)7	(AC)8	(AC)7	-	Introns/Intergenic	-	-	CTCCGACAATGGATTCGGTA	ATTGAAGTCAACCGAGCCAC	202	P	A	A	NA

Polymorphic SSR (PMS) markers	Chromosomes	Start physical positions (bp)	End physical positions (bp)	Nipponbare	Kasalath	Nagina22	IR64	Pokkali	Bala	Tainung67	Azucena	Moroberekan	*Oryza rufipogon*	*Oryza nivara*	MSU gene locus IDs	Structural annotation	Functional annotation	SSR functional effects	Forward primers (5'-3')	Reverse primers (5'-3')	Amplified product size (bp)	Markers exhibiting polymorphism within *indica* rice	Transcrption factor genes-derived markers	Known cloned genes-derived markers	PMS markers corresponding with RM markers
PMS767	Os_Chr01	40999323	40999337	(CGG)5	(CGG)6	(CGG)6	(CGG)5	(CGG)6	(CGG)5	(CGG)5	(CGG)5	(CGG)5	(CGG)6	(CGG)5	-	Introns/Intergenic	-	-	GTCCAACAGGCCCATCTAAA	ACGCTATTAGTTGGCTGGGA	142	P	A	A	NA
PMS768	Os_Chr01	41021570	41021593	(CGG)8	(CGG)7	(CGG)8	(CGG)8	(CGG)8	(CGG)7	(CGG)8	(CGG)8	(CGG)8	(CGG)8	(CGG)8	LOC_Os01g70880	CDS	histone-like transcription factor and archaeal histone, putative, expressed	CODON_DELETION	GCTCTCACGTCGATCTGTATGG	AGGAGACGATCCAGGAGTGC	288	P	A	A	RM6198
PMS769	Os_Chr01	41052499	41052513	(CTC)5	(CTC)5	(CTC)5	(CTC)5	(CTC)5	(CTC)5	(CTC)5	(CTC)5	(CTC)5	(CTC)7	(CTC)5	-	Introns/Intergenic	-	-	GCGCACTTCTCCTGGTTAGA	AGATTGCTCGGAACACAACC	133	A	A	A	NA
PMS770	Os_Chr01	41088118	41088143	(TC)13	(TC)13	(TC)13	(TC)13	(TC)13	(TC)13	(TC)13	(TC)12	(TC)13	(TC)8	(TC)13	LOC_Os01g70980	URR	expressed protein	-	GAGCGCTAGGGCTTTGGATCG	GCCCATGTACGATGACGTGTACC	160	A	A	A	RM5362
PMS771	Os_Chr01	41114046	41114069	(TCG)8	(TCG)6	(TCG)8	(TCG)8	(TCG)5	(TCG)6	(TCG)8	(TCG)8	(TCG)8	(TCG)8	(TCG)8	LOC_Os01g71040	CDS	expressed protein	CODON_DELETION	GTGATGCTTTCGTCGTCGTTGG	GGAGGACAAGAACAAGAACAAGAACG	150	P	A	A	RM12171
PMS772	Os_Chr01	41193269	41193280	(AG)6	(AG)6	(AG)6	(AG)6	(AG)6	(AG)9	(AG)6	(AG)6	(AG)6	(AG)6	(AG)6	-	Introns/Intergenic	-	-	ACAACCTCCCTCCCTTGTCT	TTGTGGGTTTAGGTGGTGGT	187	P	A	A	NA
PMS773	Os_Chr01	41199744	41199759	(AT)8	(AT)9	(AT)8	(AT)8	(AT)8	(AT)8	(AT)8	(AT)8	(AT)8	(AT)8	(AT)8	-	Introns/Intergenic	-	-	CCGTAGTGCATCATCAGGAA	CACAGCCAAGTCGTTTTAGG	280	P	A	A	NA
PMS774	Os_Chr01	41289165	41289180	(TG)8	(TG)10	(TG)10	(TG)8	(TG)8	(TG)8	(TG)8	(TG)8	(TG)8	(TG)8	(TG)8	-	Introns/Intergenic	-	-	CATGCATCAAACCTGTGGTC	ACCCTTTCTTTTCGCCCTAA	235	P	A	A	NA
PMS775	Os_Chr01	41324819	41324836	(AT)9	(AT)9	(AT)9	(AT)9	(AT)9	(AT)9	(AT)9	(AT)6	(AT)9	(AT)9	(AT)9	-	Introns/Intergenic	-	-	CCATTAGTGAAAGGGAGCAAACC	CGAAAGCTGTCTCTGGAAATTCG	157	A	A	A	RM12179
PMS776	Os_Chr01	41362206	41362217	(GA)6	(GA)6	(GA)6	(GA)6	(GA)6	(GA)6	(GA)6	(GA)6	(GA)6	(GA)7	(GA)6	-	Introns/Intergenic	-	-	AGACTTTGTTCCAAGGAGCG	CTTCCCTCTCTCTCTCCCGT	207	A	A	A	NA
PMS777	Os_Chr01	41363953	41363980	(GA)14	(GA)14	(GA)7	(GA)14	(GA)14	(GA)9	(GA)14	(GA)14	(GA)9	(GA)14	(GA)14	-	Introns/Intergenic	-	-	GCGACCGGCGAATTCAAACG	TTTCCTCGGCCCATCTCTCTCC	156	P	A	A	RM12181
PMS778	Os_Chr01	41585042	41585059	(CCG)6	(CCG)5	(CCG)6	(CCG)6	(CCG)6	(CCG)6	(CCG)5	(CCG)6	(CCG)6	(CCG)6	(CCG)6	LOC_Os01g71790	CDS	NAM, putative, expressed	CODON_CHANGE_PLUS_CODON_DELETION	CCAAGATCTCTCCAGCCAAG	GGAATGTATGGGCAGATGCT	198	P	A	A	NA
PMS779	Os_Chr01	41669365	41669410	(AT)23	(AT)23	(AT)23	(AT)23	(AT)23	(AT)23	(AT)23	(AT)16	(AT)6	(AT)23	(AT)23	-	Introns/Intergenic	-	-	CCTTCCTCTTCTGACGTAACACC	CGGCTGATCCATCTTTAACTGG	435	A	A	A	RM8086

Polymorphic SSR (PMS) markers	Chromosomes	Start physical positions (bp)	End physical positions (bp)	Nipponbare	Kasalath	Nagina22	IR64	Pokkali	Bala	Tainung67	Azucena	Moroberekan	*Oryza rufipogon*	*Oryza nivara*	MSU gene locus IDs	Structural annotation	Functional annotation	SSR functional effects	Forward primers (5'-3')	Reverse primers (5'-3')	Amplified product size (bp)	Markers exhibiting polymorphism within *indica* rice	Transcrption factor genes-derived markers	Known cloned genes-derived markers	PMS markers corresponding with RM markers
PMS780	Os_Chr01	41686223	41686270	(TA)24	(TA)24	(TA)24	(TA)24	(TA)24	(TA)11	(TA)24	(TA)24	(TA)24	(TA)24	(TA)24	-	Introns/Intergenic	-	-	TTGCCAAGTGACTCCACAAG	CGCTGGAAATGAAGTTTACG	185	P	A	A	NA
PMS781	Os_Chr01	41703152	41703163	(AT)6	(AT)6	(AT)6	(AT)6	(AT)7	(AT)6	(AT)6	(AT)6	(AT)6	(AT)7	(AT)6	-	Introns/Intergenic	-	-	CATGCTTGACATTTGGCACT	GAACAACCGTGTGTCCAGATT	144	P	A	A	NA
PMS782	Os_Chr01	41771335	41771356	(AG)11	(AG)8	(AG)11	(AG)11	(AG)11	(AG)11	(AG)11	(AG)11	(AG)11	(AG)11	(AG)11	-	Introns/Intergenic	-	-	TTTATGTGCGCATTTGCATT	AAGGTAGGCAGAGAGAGGGC	228	P	A	A	NA
PMS783	Os_Chr01	41775216	41775249	(AT)17	(AT)17	(AT)17	(AT)6	(AT)17	(AT)17	(AT)7	(AT)17	(AT)17	(AT)7	(AT)17	-	Introns/Intergenic	-	-	TGTCGACTAGCTACTGCCCC	TCAATTTCAACAATGGTGCC	277	P	A	A	NA
PMS784	Os_Chr01	41775644	41775675	(TA)16	(TA)16	(TA)11	(TA)6	(TA)6	(TA)10	(TA)16	(TA)16	(TA)16	(TA)16	(TA)16	-	Introns/Intergenic	-	-	ATGGTGCCCCATATGAGTGT	GATGGACGATTAAAGTTAGGCA	117	P	A	A	NA
PMS785	Os_Chr01	41866699	41866713	(TCA)5	(TCA)6	(TCA)5	(TCA)5	(TCA)5	(TCA)6	(TCA)5	(TCA)6	(TCA)6	(TCA)6	(TCA)5	-	Introns/Intergenic	-	-	GCCCAAGTGACTTTCAGCTC	AACCAACAATGGTCATGCAA	195	P	A	A	NA
PMS786	Os_Chr01	42009654	42009671	(TGG)6	(TGG)6	(TGG)6	(TGG)6	(TGG)6	(TGG)6	(TGG)6	(TGG)6	(TGG)5	(TGG)5	(TGG)6	LOC_Os01g72430	URR	NADPH quinone oxidoreductase, putative, expressed	-	TGATTAAGCCCAAAACTCGC	CGTCACCTACTCAGCAGCCT	235	A	A	A	NA
PMS787	Os_Chr01	42013492	42013509	(CAG)6	(CAG)5	(CAG)6	(CAG)6	(CAG)6	(CAG)6	(CAG)6	(CAG)6	(CAG)6	(CAG)6	(CAG)5	LOC_Os01g72450	CDS	DNA binding protein, putative, expressed	CODON_DELETION	TGATGAGGACGACGAGGC	GTAACGACTCGTGGCAGGAT	259	P	A	A	NA
PMS788	Os_Chr01	42055960	42055977	(GT)9	(GT)9	(GT)9	(GT)9	(GT)9	(GT)9	(GT)9	(GT)9	(GT)9	(GT)8	(GT)9	-	Introns/Intergenic	-	-	ATTTTAAAGCAGGTGCACGG	ACTGAGGCAGGGTCTTGATG	192	A	A	A	NA
PMS789	Os_Chr01	42074044	42074123	(TA)40	(TA)40	(TA)10	(TA)12	(TA)14	(TA)40	(TA)9	(TA)13	(TA)40	(TA)40	(TA)40	LOC_Os01g72530	DRR	OsMSR2	-	TCAATTTCAACAATGGTGCC	TGGATCTGTAAATGCTTCATGG	184	P	A	P	NA
PMS790	Os_Chr01	42125315	42125332	(TC)9	(TC)9	(TC)9	(TC)9	(TC)9	(TC)7	(TC)9	(TC)9	(TC)9	(TC)9	(TC)9	-	Introns/Intergenic	-	-	CACCAAGGTTCAGGCAGAAT	AGCCTACACCTACCCCTGCT	254	P	A	A	NA
PMS791	Os_Chr01	42268457	42268474	(CGC)6	(CGC)6	(CGC)6	(CGC)6	(CGC)6	(CGC)6	(CGC)6	(CGC)6	(CGC)5	(CGC)6	(CGC)6	LOC_Os01g72870	URR	BAS1, putative, expressed	-	TGGGGATTCGAGTTGCTTAC	TCTTGTCCGTTGGTTCTTCC	210	A	A	A	NA
PMS792	Os_Chr01	42377167	42377190	(TCC)8	(TCC)8	(TCC)8	(TCC)8	(TCC)5	(TCC)8	(TCC)8	(TCC)8	(TCC)8	(TCC)8	(TCC)8	-	Introns/Intergenic	-	-	CAACCAGTTCCTCCTCCTATGACC	TTTGAAATGGTGGAGTCCAAGG	218	P	A	A	RM6407

Polymorphic SSR (PMS) markers	Chromosomes	Start physical positions (bp)	End physical positions (bp)	Nipponbare	Kasalath	Nagina22	IR64	Pokkali	Bala	Tainung67	Azucena	Moroberekan	*Oryza rufipogon*	*Oryza nivara*	MSU gene locus IDs	Structural annotation	Functional annotation	SSR functional effects	Forward primers (5'-3')	Reverse primers (5'-3')	Amplified product size (bp)	Markers exhibiting polymorphism within *indica* rice	Transcrption factor genes-derived markers	Known cloned genes-derived markers	PMS markers corresponding with RM markers
PMS793	Os_Chr01	42504127	42504152	(CT)13	(CT)13	(CT)13	(CT)13	(CT)13	(CT)13	(CT)13	(CT)13	(CT)13	(CT)13	(CT)12	-	Introns/Intergenic	-	-	AATCTCACTCGGCCCATCTT	GGGAAGCGTCTTTGCGAG	247	A	A	A	NA
PMS794	Os_Chr01	42505753	42505764	(CG)6	(CG)6	(CG)6	(CG)6	(CG)6	(CG)6	(CG)6	(CG)6	(CG)7	(CG)6	(CG)6	-	Introns/Intergenic	-	-	GGTCCACCGGACTCTCTCTC	ATTAGACTTTTCCTCGGGCG	136	A	A	A	NA
PMS795	Os_Chr01	42559904	42559917	(AG)7	(AG)7	(AG)7	(AG)7	(AG)7	(AG)7	(AG)6	(AG)7	(AG)7	(AG)7	(AG)7	LOC_Os01g73440	CDS	expressed protein	FRAME_SHIFT	TTGGCCTGCGATAGAAGAGT	CAGATATTCGGCGTCCCTTA	195	A	A	A	NA
PMS796	Os_Chr01	42567966	42567992	(CGT)9	(CGT)9	(CGT)9	(CGT)9	(CGT)9	(CGT)9	(CGT)9	(CGT)9	(CGT)8	(CGT)6	(CGT)9	LOC_Os01g73460	CDS	ATXR, putative, expressed	CODON_DELETION	TCTCCCATCTCCCATATTCCCATCC	GCGCATGACGTCGGAGATCG	300	A	A	A	RM6237
PMS797	Os_Chr01	42800170	42800197	(TA)14	(TA)14	(TA)9	(TA)14	(TA)14	(TA)14	(TA)7	(TA)14	(TA)14	(TA)14	(TA)14	-	Introns/Intergenic	-	-	GAACACCACCTAAATCGCCT	GGCTCAAGAGGAGGAGGAAA	234	P	A	A	NA
PMS798	Os_Chr01	42852504	42852533	(AAG)10	(AAG)10	(AAG)10	(AAG)10	(AAG)10	(AAG)10	(AAG)10	(AAG)10	(AAG)10	(AAG)5	(AAG)10	-	Introns/Intergenic	-	-	TGTGAACTGGCGACCAACTA	CCCAATTTCTTTCTTCACGC	276	A	A	A	NA
PMS799	Os_Chr01	42884353	42884364	(AT)6	(AT)6	(AT)6	(AT)6	(AT)6	(AT)6	(AT)6	(AT)6	(AT)6	(AT)8	(AT)6	-	Introns/Intergenic	-	-	ACGTACGTTGCAATTGGTGA	TTGGAAATATGCAACGAGGC	196	A	A	A	NA
PMS800	Os_Chr01	42947565	42947580	(CT)8	(CT)8	(CT)8	(CT)8	(CT)8	(CT)8	(CT)8	(CT)8	(CT)8	(CT)8	(CT)7	-	Introns/Intergenic	-	-	TCGCAGATGATGGGTACAAA	TAATTAATTGAGCCGCCTGG	179	A	A	A	NA
PMS801	Os_Chr01	42963086	42963103	(AT)9	(AT)9	(AT)9	(AT)9	(AT)6	(AT)9	(AT)9	(AT)9	(AT)9	(AT)9	(AT)9	-	Introns/Intergenic	-	-	AATCGCAGTTGTCCCTATGC	TTTTGGCCGCTAGAAAAATG	278	P	A	A	NA
PMS802	Os_Chr01	42987202	42987219	(AT)9	(AT)9	(AT)9	(AT)9	(AT)8	(AT)9	(AT)9	(AT)7	(AT)9	(AT)9	(AT)9	-	Introns/Intergenic	-	-	TCCAGTTGACTAGTTCCTCCG	CTTCTGAGCTAGCAGCGAGT	279	P	A	A	NA
PMS803	Os_Chr01	43056654	43056679	(GA)13	(GA)10	(GA)13	(GA)13	(GA)13	(GA)13	(GA)13	(GA)13	(GA)13	(GA)10	(GA)13	-	Introns/Intergenic	-	-	GCAAACTTCAGCTATGACTTCG	GGGACCATCTACTTCTTATGTAGG	576	P	A	A	RM12279
PMS804	Os_Chr01	43058573	43058592	(GA)10	(GA)10	(GA)10	(GA)10	(GA)10	(GA)10	(GA)10	(GA)10	(GA)11	(GA)10	(GA)10	-	Introns/Intergenic	-	-	GTTGATGGGTTGGCGATATAATCC	AGTTAGGGCGAGGCTATCTAGGG	376	A	A	A	RM12280
PMS805	Os_Chr01	43080353	43080370	(TC)9	(TC)9	(TC)9	(TC)9	(TC)9	(TC)7	(TC)9	(TC)9	(TC)9	(TC)9	(TC)9	LOC_Os01g74370	URR	domain of unknown function DUF966 domain containing protein, expressed	-	CAACCAACACTACCGTGTGC	GATGCGTCATGTGAACTGCT	250	P	A	A	NA

Polymorphic SSR (PMS) markers	Chromosomes	Start physical positions (bp)	End physical positions (bp)	Nipponbare	Kasalath	Nagina22	IR64	Pokkali	Bala	Tainung67	Azucena	Moroberekan	*Oryza rufipogon*	*Oryza nivara*	MSU gene locus IDs	Structural annotation	Functional annotation	SSR functional effects	Forward primers (5'-3')	Reverse primers (5'-3')	Amplified product size (bp)	Markers exhibiting polymorphism within *indica* rice	Transcrption factor genes-derived markers	Known cloned genes-derived markers	PMS markers corresponding with RM markers
PMS806	Os_Chr01	43172146	43172172	(TTC)9	(TTC)9	(TTC)9	(TTC)9	(TTC)9	(TTC)10	(TTC)9	(TTC)9	(TTC)9	(TTC)9	(TTC)9	LOC_Os01g74540	DRR	GATA zinc finger domain containing protein, expressed	-	CAGCACGAACAGACAGGATGG	AGGAAGAAGAAGCAGCGATTCC	339	P	A	A	RM12284
PMS807	Os_Chr01	43224323	43224398	(TA)38	(TA)38	(TA)7	(TA)38	(TA)16	(TA)9	(TA)13	(TA)8	(TA)38	(TA)38	(TA)8	-	Introns/Intergenic	-	-	ATGAGACGATGAAAGCCTCAAGC	GTGGGACAAGCAAATTGAAACG	536	P	A	A	RM12292
PMS808	Os_Chr01	43239255	43239275	(GAG)7	(GAG)6	(GAG)5	(GAG)6	(GAG)6	(GAG)6	(GAG)7	(GAG)7	(GAG)7	(GAG)7	(GAG)6	-	Introns/Intergenic	-	-	CAGGGCCTTAGACTTGGTCAGC	CCAACAATCGAGGCGTATGTCC	349	P	A	A	RM12293
PMS809	Os_Chr02	48351	48382	(AT)16	(AT)16	(AT)16	(AT)15	(AT)16	(AT)16	(AT)7	(AT)11	(AT)16	(AT)16	(AT)16	-	Introns/Intergenic	-	-	GGGTGCGGTACTTAGCTACTCC	CTGTCTACTTGCATTGCTATCTCC	275	P	A	A	RM12298
PMS810	Os_Chr02	48884	48907	(CT)12	(CT)12	(CT)12	(CT)12	(CT)12	(CT)9	(CT)12	(CT)12	(CT)12	(CT)12	(CT)12	-	Introns/Intergenic	-	-	TGCCTCCTCCCTCCTACTACTGC	ATGGTGTTGTCTGCCTGACTGC	164	P	A	A	RM12299
PMS811	Os_Chr02	49732	49745	(GA)7	(GA)7	(GA)6	(GA)7	(GA)7	(GA)7	(GA)7	(GA)7	(GA)7	(GA)7	(GA)7	-	Introns/Intergenic	-	-	CCACACACCGACAGCTCTAA	ACAAGTTGGCCATATCGGAG	247	P	A	A	NA
PMS812	Os_Chr02	54953	54970	(TGC)6	(TGC)6	(TGC)5	(TGC)5	(TGC)5	(TGC)5	(TGC)6	(TGC)6	(TGC)6	(TGC)5	(TGC)5	LOC_Os02g01100	CDS	transmembrane amino acid transporter protein, putative, expressed	CODON_DELETION	CCTGGGTGTAGGAGCATTGT	GCAGGCTCTCCAACTCATTC	264	P	A	A	NA
PMS813	Os_Chr02	82072	82093	(AG)11	(AG)11	(AG)11	(AG)11	(AG)11	(AG)12	(AG)11	(AG)11	(AG)11	(AG)11	(AG)12	-	Introns/Intergenic	-	-	AGTCTGACCCTTGAGCAAGTTCC	GGAGGCAGATCAGTTCATGAGG	174	P	A	A	RM12305
PMS814	Os_Chr02	85789	85802	(TC)7	(TC)7	(TC)7	(TC)7	(TC)7	(TC)7	(TC)7	(TC)7	(TC)7	(TC)6	(TC)7	-	Introns/Intergenic	-	-	GGAGGAGGAAACCCTAGACG	ACGGTCGTTCCTATGTTTCG	229	A	A	A	NA
PMS815	Os_Chr02	183362	183393	(CT)16	(CT)13	(CT)16	(CT)16	(CT)16	(CT)16	(CT)16	(CT)16	(CT)16	(CT)16	(CT)16	LOC_Os02g01332	URR	ribosomal protein L6, putative, expressed	-	AGCCAAGATCGTCTTCATCTCTCG	TCGTCTCCTTCTTCCTTCTTCTTCC	113	P	A	A	RM109
PMS816	Os_Chr02	200175	200190	(AT)8	(AT)8	(AT)8	(AT)8	(AT)10	(AT)8	(AT)8	(AT)8	(AT)8	(AT)8	(AT)8	-	Introns/Intergenic	-	-	GGTCCCTTCTGTTAGCTTTCC	GGAGGGGATGTGTGCTAGTT	177	P	A	A	NA
PMS817	Os_Chr02	212724	212747	(GA)12	(GA)12	(GA)12	(GA)11	(GA)10	(GA)11	(GA)12	(GA)12	(GA)12	(GA)12	(GA)12	-	Introns/Intergenic	-	-	TGTGTGTCTCTGCTCTCGCT	CATGGATGGATCGAGATGTG	276	P	A	A	NA
PMS818	Os_Chr02	222384	222401	(GGT)6	(GGT)6	(GGT)6	(GGT)6	(GGT)6	(GGT)6	(GGT)6	(GGT)6	(GGT)6	(GGT)5	(GGT)6	-	Introns/Intergenic	-	-	GAGGCCGAGATGGTGGTG	CTCAGATGCCTCCCCACTT	218	A	A	A	NA

Polymorphic SSR (PMS) markers	Chromosomes	Start physical positions (bp)	End physical positions (bp)	Nipponbare	Kasalath	Nagina22	IR64	Pokkali	Bala	Tainung67	Azucena	Moroberekan	*Oryza rufipogon*	*Oryza nivara*	MSU gene locus IDs	Structural annotation	Functional annotation	SSR functional effects	Forward primers (5'-3')	Reverse primers (5'-3')	Amplified product size (bp)	Markers exhibiting polymorphism within *indica* rice	Transcrption factor genes-derived markers	Known cloned genes-derived markers	PMS markers corresponding with RM markers
PMS819	Os_Chr02	254906	254921	(AT)8	(AT)8	(AT)8	(AT)8	(AT)8	(AT)8	(AT)8	(AT)8	(AT)8	(AT)7	(AT)8	-	Introns/Intergenic	-	-	AAGTGGATAAATCGTAATAAGCAAA	TCATGCATGTGGCATTTACA	251	A	A	A	NA
PMS820	Os_Chr02	386212	386241	(AG)15	(AG)15	(AG)19	(AG)15	(AG)15	(AG)15	(AG)15	(AG)15	(AG)15	(AG)15	(AG)15	-	Introns/Intergenic	-	-	GAGAGAGACACCAAATGATCCATCC	ACTGATTTGGCCCTTGTTCTTGG	152	P	A	A	RM3340
PMS821	Os_Chr02	426903	426950	(TAA)16	(TAA)16	(TAA)16	(TAA)16	(TAA)16	(TAA)16	(TAA)16	(TAA)16	(TAA)16	(TAA)16	(TAA)9	-	Introns/Intergenic	-	-	GCATGCACTGCAGATACCAA	TTTGCAGCAGAGCAGAAAAA	199	A	A	A	NA
PMS822	Os_Chr02	571608	571637	(GA)15	(GA)15	(GA)17	(GA)17	(GA)15	(GA)15	(GA)15	(GA)15	(GA)15	(GA)15	(GA)15	-	Introns/Intergenic	-	-	AGGAAGAGGCGAAGGTAGATCG	CCAATCATGCTGTGTTTCAAGG	240	P	A	A	RM12329
PMS823	Os_Chr02	709261	709290	(AT)15	(AT)15	(AT)15	(AT)15	(AT)12	(AT)15	(AT)15	(AT)15	(AT)6	(AT)15	(AT)15	-	Introns/Intergenic	-	-	CGAAGGGTCAAATATTATGAGAAAA	TGTGCCCTTTTAAATGATGGA	184	P	A	A	NA
PMS824	Os_Chr02	723452	723463	(TC)6	(TC)8	(TC)8	(TC)8	(TC)8	(TC)8	(TC)6	(TC)6	(TC)6	(TC)6	(TC)8	-	Introns/Intergenic	-	-	AACGGTGAACACAGCAAACA	TTAGGGCATGTGCAATGGTA	243	A	A	A	NA
PMS825	Os_Chr02	776357	776374	(GCG)6	(GCG)6	(GCG)6	(GCG)6	(GCG)6	(GCG)6	(GCG)6	(GCG)5	(GCG)6	(GCG)6	(GCG)6	LOC_Os02g02340	CDS	glycerol-3-phosphate acyltransferase, putative, expressed	CODON_DELETION	ATGGACACGATGATGGGG	CCACACCTACCGCCTCAC	132	A	A	A	NA
PMS826	Os_Chr02	886500	886517	(CGG)6	(CGG)6	(CGG)6	(CGG)6	(CGG)6	(CGG)6	(CGG)5	(CGG)6	(CGG)6	(CGG)6	(CGG)6	LOC_Os02g02490	CDS	phytosulfokine receptor precursor, putative, expressed	CODON_CHANGE_PLUS_CODON_DELETION	ACGTCGCGTACAACAACCTC	GTTGTCCTCCTGCCACCTC	272	A	A	A	NA
PMS827	Os_Chr02	1040565	1040588	(CCA)8	(CCA)8	(CCA)7	(CCA)8	(CCA)8	(CCA)8	(CCA)5	(CCA)8	(CCA)8	(CCA)8	(CCA)7	-	Introns/Intergenic	-	-	TCCTTTTGTTCCCTCCTCCT	CCGATCGGAACAAGAAAGAA	232	P	A	A	NA
PMS828	Os_Chr02	1040697	1040714	(CTT)6	(CTT)6	(CTT)6	(CTT)6	(CTT)6	(CTT)9	(CTT)6	(CTT)6	(CTT)6	(CTT)6	(CTT)9	-	Introns/Intergenic	-	-	TCCTTTTGTTCCCTCCTCCT	CCGATCGGAACAAGAAAGAA	232	P	A	A	NA
PMS829	Os_Chr02	1083920	1083961	(CT)21	(CT)21	(CT)21	(CT)21	(CT)12	(CT)21	(CT)21	(CT)21	(CT)21	(CT)21	(CT)21	LOC_Os02g02840	URR	OsRacB	-	GACGGTGACGCACTTTATGAACC	CGATCTGCGAGAAACCCTCTCC	271	P	A	P	RM154,OSR11
PMS830	Os_Chr02	1097225	1097248	(GATG)6	(GATG)6	(GATG)6	(GATG)6	(GATG)6	(GATG)6	(GATG)6	(GATG)6	(GATG)6	(GATG)6	(GATG)5	-	Introns/Intergenic	-	-	GAGGGCAAGGAAATGCATAA	GGCCAATACCAAAGACGAGA	187	A	A	A	NA
PMS831	Os_Chr02	1097896	1097909	(TA)7	(TA)7	(TA)7	(TA)7	(TA)7	(TA)7	(TA)7	(TA)7	(TA)7	(TA)8	(TA)8	-	Introns/Intergenic	-	-	TGAAATCAGAACACGCAAGG	CCTGTGGTCCGTTCTTCATT	214	A	A	A	NA

Polymorphic SSR (PMS) markers	Chromosomes	Start physical positions (bp)	End physical positions (bp)	Nipponbare	Kasalath	Nagina22	IR64	Pokkali	Bala	Tainung67	Azucena	Moroberekan	*Oryza rufipogon*	*Oryza nivara*	MSU gene locus IDs	Structural annotation	Functional annotation	SSR functional effects	Forward primers (5'-3')	Reverse primers (5'-3')	Amplified product size (bp)	Markers exhibiting polymorphism within *indica* rice	Transcrption factor genes-derived markers	Known cloned genes-derived markers	PMS markers corresponding with RM markers
PMS832	Os_Chr02	1124012	1124032	(CCG)7	(CCG)7	(CCG)7	(CCG)7	(CCG)7	(CCG)8	(CCG)6	(CCG)7	(CCG)7	(CCG)7	(CCG)7	LOC_Os02g02900	CDS	expressed protein	CODON_DELETION	TTGTCACGCCGGATCTGTTGC	AGGAAGAGGAGGTGAATCGGAAGC	271	P	A	A	RM12358
PMS833	Os_Chr02	1162235	1162252	(CCA)6	(CCA)6	(CCA)6	(CCA)6	(CCA)6	(CCA)6	(CCA)6	(CCA)6	(CCA)6	(CCA)5	(CCA)6	LOC_Os02g02970	CDS	expressed protein	CODON_DELETION	GATGGATTCTTCCGGTTCCT	GCGGAGGTATCGAGAGAGG	205	A	A	A	NA
PMS834	Os_Chr02	1173220	1173234	(GCC)5	(GCC)5	(GCC)5	(GCC)5	(GCC)5	(GCC)5	(GCC)5	(GCC)5	(GCC)5	(GCC)6	(GCC)5	LOC_Os02g02980	CDS	MATE domain containing protein, expressed	CODON_INSERTION	GAGCTGGAGGGAGGAAGTCT	GCTTACCGAGCCACAGCC	241	A	A	A	NA
PMS835	Os_Chr02	1292379	1292396	(GCG)6	(GCG)5	(GCG)6	(GCG)6	(GCG)6	(GCG)6	(GCG)6	(GCG)6	(GCG)5	(GCG)6	(GCG)6	LOC_Os02g03230	URR	membrane related protein CP5, putative, expressed	-	CGACAGCACGTACCCCAT	GACTCGATAGGCCACAAAGC	114	P	A	A	NA
PMS836	Os_Chr02	1302333	1302350	(CGC)6	(CGC)6	(CGC)6	(CGC)5	(CGC)6	(CGC)6	(CGC)6	(CGC)6	(CGC)6	(CGC)6	(CGC)6	LOC_Os02g03250	URR	expressed protein	-	CCTCAACTGACTCAGCCTCC	GGACTTCCTCGCCTTCTTCT	230	P	A	A	NA
PMS837	Os_Chr02	1344993	1345007	(CGG)5	(CGG)5	(CGG)5	(CGG)5	(CGG)5	(CGG)5	(CGG)5	(CGG)5	(CGG)5	(CGG)6	(CGG)5	LOC_Os02g03330	CDS	expressed protein	CODON_INSERTION	GACCTCCTCACCCTCTCCTC	CTTCTCGAACTGCCTGAGGT	252	A	A	A	NA
PMS838	Os_Chr02	1402704	1402721	(GCG)6	(GCG)7	(GCG)6	(GCG)6	(GCG)6	(GCG)6	(GCG)6	(GCG)6	(GCG)6	(GCG)6	(GCG)6	LOC_Os02g03450	CDS	vesicle transport v-SNARE protein, putative, expressed	CODON_INSERTION	CCAATTCTCCTCTTCTCCCC	CGTAGGAGGAGAGCTTGACG	259	P	A	A	NA
PMS839	Os_Chr02	1450405	1450425	(CGG)7	(CGG)7	(CGG)7	(CGG)7	(CGG)7	(CGG)7	(CGG)7	(CGG)7	(CGG)7	(CGG)7	(CGG)5	LOC_Os02g03570	CDS	hsp20/alpha crystallin family protein, putative, expressed	CODON_CHANGE_PLUS_CODON_DELETION	GACATCATGGAGCCGTTCAT	ACCTTCTCCTCCTCCACCTC	197	A	A	A	NA
PMS840	Os_Chr02	1489767	1489792	(AG)13	(AG)12	(AG)13	(AG)12	(AG)13	(AG)13	(AG)13	(AG)13	(AG)13	(AG)13	(AG)12	-	Introns/Intergenic	-	-	GCTCCCAACTAGTGCAAGTACACG	CATGGCTAACTAGATCGACACACG	142	P	A	A	RM12380
PMS841	Os_Chr02	1516707	1516720	(TG)7	(TG)6	(TG)6	(TG)7	(TG)7	(TG)7	(TG)7	(TG)7	(TG)7	(TG)7	(TG)7	-	Introns/Intergenic	-	-	ACATGTTGGTGCTTTATGCG	CAAGCTGAAAGAAACAGGGC	203	P	A	A	NA
PMS842	Os_Chr02	1521357	1521382	(AT)13	(AT)13	(AT)6	(AT)13	(AT)13	(AT)13	(AT)13	(AT)13	(AT)13	(AT)13	(AT)13	-	Introns/Intergenic	-	-	TCTCCACCTACTCTCTTATCTCAACC	GAAGAATATTCGAACGGTAGGG	86	P	A	A	RM12384
PMS843	Os_Chr02	1530232	1530257	(AT)13	(AT)13	(AT)13	(AT)13	(AT)13	(AT)13	(AT)13	(AT)7	(AT)13	(AT)13	(AT)13	-	Introns/Intergenic	-	-	CCATGCAGTGGTGAGTACGATGG	GTATGCCTGCCTGCTAGCATTGG	392	A	A	A	RM12386
PMS844	Os_Chr02	1579099	1579119	(CGC)7	(CGC)7	(CGC)7	(CGC)7	(CGC)7	(CGC)7	(CGC)7	(CGC)7	(CGC)7	(CGC)6	(CGC)7	LOC_Os02g03760	URR	expressed protein	-	AACCGTAACCTCGTCAATGC	AGCAGAGAGCAAAGCGAAAG	263	A	A	A	NA

Polymorphic SSR (PMS) markers	Chromosomes	Start physical positions (bp)	End physical positions (bp)	Nipponbare	Kasalath	Nagina22	IR64	Pokkali	Bala	Tainung67	Azucena	Moroberekan	*Oryza rufipogon*	*Oryza nivara*	MSU gene locus IDs	Structural annotation	Functional annotation	SSR functional effects	Forward primers (5'-3')	Reverse primers (5'-3')	Amplified product size (bp)	Markers exhibiting polymorphism within *indica* rice	Transcrption factor genes-derived markers	Known cloned genes-derived markers	PMS markers corresponding with RM markers
PMS845	Os_Chr02	1636859	1636878	(AT)10	(AT)11	(AT)10	(AT)10	(AT)10	(AT)10	(AT)10	(AT)10	(AT)10	(AT)10	(AT)10	-	Introns/Intergenic	-	-	CGGAGGAACACGACGACTAT	ACACCATGGAGCACAAAACA	165	P	A	A	NA
PMS846	Os_Chr02	1693222	1693277	(TA)28	(TA)28	(TA)28	(TA)28	(TA)6	(TA)28	(TA)28	(TA)28	(TA)28	(TA)28	(TA)28	-	Introns/Intergenic	-	-	CCAACAGGAGAACCAAGGTC	CATTTCTGTTTTGATCCGGC	220	P	A	A	NA
PMS847	Os_Chr02	1949350	1949376	(GCG)9	(GCG)9	(GCG)9	(GCG)9	(GCG)9	(GCG)9	(GCG)5	(GCG)9	(GCG)9	(GCG)9	(GCG)9	LOC_Os02g04420	URR	expressed protein	-	ATCCACCGGATTTCTCCTACTCC	CCATTCGAGCCCAAACATAACC	186	A	A	A	RM6151
PMS848	Os_Chr02	1974627	1974644	(CGC)6	(CGC)6	(CGC)6	(CGC)5	(CGC)6	(CGC)6	(CGC)6	(CGC)6	(CGC)6	(CGC)6	(CGC)6	LOC_Os02g04460	CDS	ribosomal protein L3, putative, expressed	CODON_DELETION	CGAAGTAGGTCATCATCCCG	GACACTGCTGGAGTGAGCAA	256	P	A	A	NA
PMS849	Os_Chr02	2064642	2064659	(CCG)6	(CCG)6	(CCG)6	(CCG)6	(CCG)6	(CCG)7	(CCG)6	(CCG)6	(CCG)6	(CCG)6	(CCG)6	LOC_Os02g04610	CDS	expressed protein	CODON_INSERTION	AGCAATGAGGCAGTTCGTCT	AGCATGAAGTGCATGACCC	231	P	A	A	NA
PMS850	Os_Chr02	2331735	2331755	(CCG)7	(CCG)7	(CCG)7	(CCG)7	(CCG)7	(CCG)7	(CCG)7	(CCG)7	(CCG)7	(CCG)7	(CCG)6	LOC_Os02g04970	URR	expressed protein	-	CCCAAATTTCTCCGAACATTCC	GGAGGAGACGACGTGGAACC	289	A	A	A	RM12446
PMS851	Os_Chr02	2413192	2413209	(CCG)6	(CCG)5	(CCG)6	(CCG)6	(CCG)6	(CCG)5	(CCG)6	(CCG)5	(CCG)6	(CCG)5	(CCG)6	LOC_Os02g05070	CDS	expressed protein	CODON_DELETION	GAGCGACACGGTCAAGAACT	CGTCACGTCTCTTCTACCCC	260	P	A	A	NA
PMS852	Os_Chr02	2416235	2416258	(CGG)8	(CGG)8	(CGG)8	(CGG)8	(CGG)8	(CGG)5	(CGG)8	(CGG)5	(CGG)5	(CGG)8	(CGG)8	LOC_Os02g05080	CDS	expressed protein	CODON_CHANGE_PLUS_CODON_DELETION	CGACCCATCAAGTCTCTTCTACG	CTTCATCCACGTGAGGTTGTCC	143	P	A	A	RM12452
PMS853	Os_Chr02	2606889	2606915	(GTC)9	(GTC)9	(GTC)9	(GTC)8	(GTC)9	(GTC)9	(GTC)9	(GTC)9	(GTC)9	(GTC)9	(GTC)9	-	Introns/Intergenic	-	-	GACAGCGCACTTTCTCTTCA	TCAGAACACTCGGAAACACG	212	P	A	A	NA
PMS854	Os_Chr02	2633763	2633794	(TC)16	(TC)17	(TC)16	(TC)16	(TC)16	(TC)16	(TC)16	(TC)16	(TC)16	(TC)16	(TC)16	-	Introns/Intergenic	-	-	TGGCACTACAGTGACAACAAACC	AGGGACTTTATCCAAAGGACACG	169	P	A	A	RM12460
PMS855	Os_Chr02	2698818	2698831	(GA)7	(GA)6	(GA)7	(GA)7	(GA)7	(GA)6	(GA)7	(GA)7	(GA)7	(GA)7	(GA)7	-	Introns/Intergenic	-	-	TGCGTAAACCAAACACCAAA	CCCTAACGAGATCGGACAGA	212	P	A	A	NA
PMS856	Os_Chr02	2701485	2701508	(TA)12	(TA)12	(TA)12	(TA)12	(TA)12	(TA)12	(TA)7	(TA)7	(TA)9	(TA)8	(TA)12	-	Introns/Intergenic	-	-	TCAATTTCAACAATGGTGCC	ATTAGATACCATGGTGCCCG	122	A	A	A	NA
PMS857	Os_Chr02	2701902	2701957	(TA)28	(TA)10	(TA)28	(TA)28	(TA)28	(TA)8	(TA)28	(TA)28	(TA)9	(TA)21	(TA)8	-	Introns/Intergenic	-	-	TCAATTTCAACAATGGTGCC	TTTGGCGGATCGATCTTTAG	146	P	A	A	NA

Polymorphic SSR (PMS) markers	Chromosomes	Start physical positions (bp)	End physical positions (bp)	Nipponbare	Kasalath	Nagina22	IR64	Pokkali	Bala	Tainung67	Azucena	Moroberekan	*Oryza rufipogon*	*Oryza nivara*	MSU gene locus IDs	Structural annotation	Functional annotation	SSR functional effects	Forward primers (5'-3')	Reverse primers (5'-3')	Amplified product size (bp)	Markers exhibiting polymorphism within *indica* rice	Transcrption factor genes-derived markers	Known cloned genes-derived markers	PMS markers corresponding with RM markers
PMS858	Os_Chr02	2758677	2758694	(CCG)6	(CCG)6	(CCG)6	(CCG)6	(CCG)6	(CCG)6	(CCG)5	(CCG)6	(CCG)6	(CCG)6	(CCG)6	LOC_Os02g05640	CDS	homeobox associated leucine zipper, putative, expressed	CODON_CHANGE_PLUS_CODON_DELETION	AGGACGAAGCTGAAGCAGAC	GAGCCCAGCTCGATCAGTTA	259	A	A	A	NA
PMS859	Os_Chr02	2798196	2798257	(AT)31	(AT)31	(AT)31	(AT)7	(AT)31	(AT)31	(AT)31	(AT)11	(AT)31	(AT)31	(AT)31	-	Introns/Intergenic	-	-	TCTTACTCCCTACAGGCTGATACTCC	TTGGACAGAGCACGGTCTCC	424	P	A	A	RM12477
PMS860	Os_Chr02	2799060	2799092	(GAG)11	(GAG)11	(GAG)11	(GAG)11	(GAG)11	(GAG)11	(GAG)11	(GAG)9	(GAG)11	(GAG)11	(GAG)11	LOC_Os02g05700	CDS	OsFBO8 - F-box and other domain containing protein, expressed	CODON_DELETION	ATGGAGTGGGACAGCGACT	GGTTGACGTAGATGATGGGG	217	A	A	A	NA
PMS861	Os_Chr02	2812182	2812215	(CT)17	(CT)16	(CT)17	(CT)6	(CT)17	(CT)17	(CT)17	(CT)17	(CT)17	(CT)17	(CT)17	-	Introns/Intergenic	-	-	CGCTTTTACCACCACCATCT	CTCACTTCAAGCTCCCCGT	278	P	A	A	NA
PMS862	Os_Chr02	2837275	2837288	(TC)7	(TC)11	(TC)7	(TC)7	(TC)7	(TC)7	(TC)7	(TC)7	(TC)7	(TC)7	(TC)7	-	Introns/Intergenic	-	-	AACGCAATGCCAGAGTTTTT	CACCCCCGGAGAGTAGAAAT	198	P	A	A	NA
PMS863	Os_Chr02	2865096	2865125	(CGC)10	(CGC)10	(CGC)10	(CGC)10	(CGC)10	(CGC)10	(CGC)10	(CGC)8	(CGC)10	(CGC)10	(CGC)10	LOC_Os02g05810	URR	expressed protein	-	TCAAGATCAACGCACTCCTCTCC	TCGTACGAGCAACAGGTGGTAGC	259	A	A	A	RM6616
PMS864	Os_Chr02	2918948	2918963	(CG)8	(CG)8	(CG)7	(CG)8	(CG)8	(CG)8	(CG)8	(CG)8	(CG)8	(CG)7	(CG)7	LOC_Os02g05880	URR	RNA polymerase Rpb1, domain 2 family protein, expressed	-	ATTTCTGCGAGTAACCCAGC	CTGCACCTGAGACCTGACCT	280	P	A	A	NA
PMS865	Os_Chr02	2943666	2943679	(TA)7	(TA)7	(TA)7	(TA)7	(TA)7	(TA)6	(TA)7	(TA)7	(TA)7	(TA)7	(TA)7	-	Introns/Intergenic	-	-	CGGTGATATGAGTGGGACCT	ATCGAGATCACCCAATGAGC	276	P	A	A	NA
PMS866	Os_Chr02	3091773	3091793	(CGC)7	(CGC)6	(CGC)7	(CGC)7	(CGC)7	(CGC)7	(CGC)7	(CGC)7	(CGC)7	(CGC)7	(CGC)5	-	Introns/Intergenic	-	-	AGACGGCCTGCTAAACTTTGACC	CGGAAATGATTCTACGGTGAGC	200	P	A	A	RM12491
PMS867	Os_Chr02	3160941	3160964	(CT)12	(CT)11	(CT)12	(CT)12	(CT)11	(CT)12	(CT)12	(CT)12	(CT)12	(CT)12	(CT)11	-	Introns/Intergenic	-	-	CACGGTCAAATCCGAAGTCT	AGCAACAAACCCAAGCAACT	274	P	A	A	NA
PMS868	Os_Chr02	3161689	3161703	(TCT)5	(TCT)7	(TCT)7	(TCT)5	(TCT)5	(TCT)5	(TCT)5	(TCT)5	(TCT)5	(TCT)5	(TCT)5	-	Introns/Intergenic	-	-	GCTTGCTTGCTATCCGAGTC	TGCTTTTCACTTGTTGGTGC	193	P	A	A	NA
PMS869	Os_Chr02	3226417	3226434	(GCG)6	(GCG)6	(GCG)6	(GCG)6	(GCG)6	(GCG)6	(GCG)6	(GCG)5	(GCG)6	(GCG)6	(GCG)6	-	Introns/Intergenic	-	-	GGAGAAGTAGTCGGCCATCA	GGCTCTTCAATGACGTGGAT	222	A	A	A	NA
PMS870	Os_Chr02	3251365	3251382	(AGG)6	(AGG)6	(AGG)6	(AGG)6	(AGG)6	(AGG)8	(AGG)6	(AGG)8	(AGG)6	(AGG)6	(AGG)6	-	Introns/Intergenic	-	-	CGACATTGGCTCTAAGCTCC	AATGGCAATGAGTCGAGGAC	127	P	A	A	NA

Polymorphic SSR (PMS) markers	Chromosomes	Start physical positions (bp)	End physical positions (bp)	Nipponbare	Kasalath	Nagina22	IR64	Pokkali	Bala	Tainung67	Azucena	Moroberekan	*Oryza rufipogon*	*Oryza nivara*	MSU gene locus IDs	Structural annotation	Functional annotation	SSR functional effects	Forward primers (5'-3')	Reverse primers (5'-3')	Amplified product size (bp)	Markers exhibiting polymorphism within *indica* rice	Transcrption factor genes-derived markers	Known cloned genes-derived markers	PMS markers corresponding with RM markers
PMS871	Os_Chr02	3451675	3451695	(GGC)7	(GGC)5	(GGC)7	(GGC)7	(GGC)7	(GGC)7	(GGC)7	(GGC)7	(GGC)7	(GGC)7	(GGC)7	-	Introns/Intergenic	-	-	CCTACCCTCCTCGACCGT	CTGACTGCCGTTCCCATT	144	P	A	A	NA
PMS872	Os_Chr02	3458199	3458216	(CCG)6	(CCG)6	(CCG)6	(CCG)5	(CCG)5	(CCG)5	(CCG)5	(CCG)6	(CCG)6	(CCG)6	(CCG)6	-	Introns/Intergenic	-	-	CTTTTTCCAGTTTCCCTCCC	GATCCTCGAGTCCCTGAGC	244	P	A	A	NA
PMS873	Os_Chr02	3460014	3460031	(CGC)6	(CGC)6	(CGC)6	(CGC)6	(CGC)6	(CGC)6	(CGC)5	(CGC)6	(CGC)6	(CGC)6	(CGC)6	LOC_Os02g06860	CDS	OsMADS80 - MADS-box family gene with M alpha type-box, expressed	CODON_DELETION	AGGCGATGACACACTACACG	GTACCCAAATCCATGGCTGA	212	A	P	A	NA
PMS874	Os_Chr02	3487408	3487431	(TGG)8	(TGG)8	(TGG)8	(TGG)8	(TGG)8	(TGG)8	(TGG)7	(TGG)8	(TGG)8	(TGG)5	(TGG)8	LOC_Os02g06910	URR	auxin response factor 6, putative, expressed	-	AAGAAGGTTTGGCCGGTTTGG	GTCGTGTCATGGAGCAAGCAACC	88	A	P	A	RM5984
PMS875	Os_Chr02	3496497	3496512	(AT)8	(AT)7	(AT)7	(AT)8	(AT)8	(AT)8	(AT)8	(AT)8	(AT)8	(AT)8	(AT)7	-	Introns/Intergenic	-	-	CATGGAAAGAGCGGTAAGGA	GCTTTTCTCTGGCTCTACGC	255	P	A	A	NA
PMS876	Os_Chr02	3610888	3610919	(TTTA)8	(TTTA)8	(TTTA)8	(TTTA)8	(TTTA)8	(TTTA)8	(TTTA)8	(TTTA)5	(TTTA)8	(TTTA)8	(TTTA)8	-	Introns/Intergenic	-	-	CAGGACATCCCTGAACAGACAGC	CTCACCTTGGTGATCTTGAGAAGG	182	A	A	A	RM12517
PMS877	Os_Chr02	3639735	3639748	(TG)7	(TG)6	(TG)6	(TG)7	(TG)7	(TG)7	(TG)7	(TG)7	(TG)7	(TG)7	(TG)7	-	Introns/Intergenic	-	-	GCTGCTGCATTCATCTACGA	AAAAGGATGCATGCAAAAGG	260	P	A	A	NA
PMS878	Os_Chr02	3655667	3655690	(CCG)8	(CCG)6	(CCG)8	(CCG)8	(CCG)8	(CCG)8	(CCG)8	(CCG)8	(CCG)8	(CCG)6	(CCG)8	LOC_Os02g07110	CDS	OsSAUR6 - Auxin-responsive SAUR gene family member, expressed	CODON_CHANGE_PLUS_CODON_DELETION	AGTACGGGTACGGCCACC	GGCAGAAGAGCGTAAAATGC	204	P	A	A	NA
PMS879	Os_Chr02	3850851	3850862	(AC)6	(AC)6	(AC)6	(AC)6	(AC)7	(AC)6	(AC)6	(AC)7	(AC)7	(AC)6	(AC)6	-	Introns/Intergenic	-	-	CCCTAGCTCCCTTGTTTGTTGC	GGTACAATGTGCAGTGGCAGTCC	151	P	A	A	RM1285
PMS880	Os_Chr02	4044395	4044416	(GA)11	(GA)13	(GA)11	(GA)11	(GA)11	(GA)9	(GA)11	(GA)11	(GA)9	(GA)11	(GA)11	-	Introns/Intergenic	-	-	TGAAGCCCACTAAAGGATGG	GGGAAATTGGAAGATGATGG	230	P	A	A	NA
PMS881	Os_Chr02	4058484	4058507	(GTC)8	(GTC)8	(GTC)8	(GTC)8	(GTC)8	(GTC)8	(GTC)8	(GTC)8	(GTC)8	(GTC)7	(GTC)8	LOC_Os02g07770	CDS	Myb transcription factor, putative, expressed	CODON_DELETION	TGTGATTACCCGTAGGATAGGTTCC	CTGCAAGACCTCCACATGTACG	491	A	P	A	RM12546
PMS882	Os_Chr02	4086519	4086536	(CCG)6	(CCG)6	(CCG)6	(CCG)6	(CCG)6	(CCG)5	(CCG)6	(CCG)6	(CCG)6	(CCG)6	(CCG)6	LOC_Os02g07810	CDS	inactive receptor kinase At2g26730 precursor, putative, expressed	CODON_CHANGE_PLUS_CODON_DELETION	CAAAAGCATAGCCACCCACT	GAACGCGATCAGCACGTC	257	P	P	A	NA
PMS883	Os_Chr02	4089125	4089142	(CGA)6	(CGA)6	(CGA)6	(CGA)6	(CGA)6	(CGA)6	(CGA)6	(CGA)6	(CGA)6	(CGA)5	(CGA)6	LOC_Os02g07810	CDS	inactive receptor kinase At2g26730 precursor, putative, expressed	CODON_CHANGE_PLUS_CODON_DELETION	CAGAGGTGGGAGCTCAAGAC	TTACGTTACGACCGGGTCTC	152	A	P	A	NA

Polymorphic SSR (PMS) markers	Chromosomes	Start physical positions (bp)	End physical positions (bp)	Nipponbare	Kasalath	Nagina22	IR64	Pokkali	Bala	Tainung67	Azucena	Moroberekan	*Oryza rufipogon*	*Oryza nivara*	MSU gene locus IDs	Structural annotation	Functional annotation	SSR functional effects	Forward primers (5'-3')	Reverse primers (5'-3')	Amplified product size (bp)	Markers exhibiting polymorphism within *indica* rice	Transcrption factor genes-derived markers	Known cloned genes-derived markers	PMS markers corresponding with RM markers
PMS884	Os_Chr02	4154753	4154768	(TG)8	(TG)8	(TG)8	(TG)8	(TG)8	(TG)8	(TG)8	(TG)8	(TG)8	(TG)9	(TG)8	-	Introns/Intergenic	-	-	GCCCGTTAAAAGATTCCCAT	AAACCCTATGATCACGCGAC	239	A	A	A	NA
PMS885	Os_Chr02	4247867	4247884	(CGG)6	(CGG)6	(CGG)6	(CGG)6	(CGG)6	(CGG)6	(CGG)6	(CGG)5	(CGG)6	(CGG)6	(CGG)6	LOC_Os02g08070	CDS	OsSPL5 - SBP-box gene family member, expressed	CODON_CHANGE_PLUS_CODON_DELETION	CAGATGCCGATGATGATGTC	GCGGGGGAGAAGTAGGTC	176	A	P	A	NA
PMS886	Os_Chr02	4285827	4285844	(TAT)6	(TAT)6	(TAT)6	(TAT)7	(TAT)7	(TAT)7	(TAT)6	(TAT)6	(TAT)6	(TAT)7	(TAT)7	LOC_Os02g08100	DRR	Os4CL3	-	TTGGTTTGTTGGTTGGTTGA	TTTTCTCCCTGAAATGCCAC	270	P	A	P	NA
PMS887	Os_Chr02	4344128	4344143	(CA)8	(CA)7	(CA)8	(CA)8	(CA)8	(CA)8	(CA)8	(CA)8	(CA)8	(CA)8	(CA)8	-	Introns/Intergenic	-	-	GTGAAACGCGTGATAGCAAA	CCAAATGAGAGGGTACCATTTTT	134	P	A	A	NA
PMS888	Os_Chr02	4355430	4355480	(ATA)17	(ATA)17	(ATA)17	(ATA)5	(ATA)17	(ATA)17	(ATA)17	(ATA)17	(ATA)17	(ATA)17	(ATA)17	-	Introns/Intergenic	-	-	CCGCGTTCCATCGTGTACTGC	CGAGACTGCACCTACGGCAAGC	266	P	A	A	RM12576
PMS889	Os_Chr02	4362320	4362337	(GA)9	(GA)9	(GA)8	(GA)9	(GA)9	(GA)9	(GA)9	(GA)9	(GA)9	(GA)9	(GA)9	LOC_Os02g08230	URR	WAX2, putative, expressed	-	GGCTGCTTGCTTAGTTTGCT	CGTCCCCTTCTTCTCCTTCT	219	P	A	A	NA
PMS890	Os_Chr02	4423163	4423177	(TTC)5	(TTC)5	(TTC)6	(TTC)5	(TTC)5	(TTC)5	(TTC)5	(TTC)5	(TTC)5	(TTC)5	(TTC)5	LOC_Os02g08320	URR	haemolysin-III, putative, expressed	-	CAGACAAGACCAAGTGGCAA	ATCCAATGCCACCTTCATTC	265	P	A	A	NA
PMS891	Os_Chr02	4426540	4426557	(GA)9	(GA)9	(GA)9	(GA)6	(GA)9	(GA)9	(GA)8	(GA)9	(GA)9	(GA)9	(GA)9	LOC_Os02g08330	URR	gp176, putative, expressed	-	TGTTGAAGTTGGCGTCCAT	GTCCTCCTCCACCAACACAT	230	P	A	A	NA
PMS892	Os_Chr02	4457721	4457746	(AG)13	(AG)12	(AG)12	(AG)13	(AG)13	(AG)12	(AG)13	(AG)13	(AG)13	(AG)13	(AG)13	LOC_Os02g08370	URR	ubiquitin carboxyl-terminal hydrolase, family 1, putative, expressed	-	CGATGGGTGAGATTGGACTGG	TTGTGGCCTTCTCTCTACTGTTGG	387	P	A	A	RM12584
PMS893	Os_Chr02	4460382	4460399	(CGC)6	(CGC)6	(CGC)6	(CGC)6	(CGC)6	(CGC)6	(CGC)6	(CGC)6	(CGC)6	(CGC)6	(CGC)5	LOC_Os02g08380	URR	CR084 protein, putative, expressed	-	ATCCCAAAAATCGAGCCTTT	GGAGGGAGAGGATGGCTTAC	167	A	A	A	NA
PMS894	Os_Chr02	4465403	4465418	(AT)8	(AT)8	(AT)8	(AT)8	(AT)8	(AT)8	(AT)8	(AT)8	(AT)8	(AT)9	(AT)8	-	Introns/Intergenic	-	-	CGCATGCTTTCCCAAATAAC	ATCACGGCTTTTCGTCAAAC	138	A	A	A	NA
PMS895	Os_Chr02	4546790	4546809	(GACC)5	(GACC)5	(GACC)5	(GACC)5	(GACC)5	(GACC)7	(GACC)5	(GACC)5	(GACC)5	(GACC)5	(GACC)5	-	Introns/Intergenic	-	-	CCAGTCGCTCACCTCAGTCACC	ATTGGATGGGATCAGCATTCAGC	183	P	A	A	RM12591
PMS896	Os_Chr02	4571098	4571117	(GA)10	(GA)10	(GA)10	(GA)10	(GA)10	(GA)10	(GA)10	(GA)10	(GA)13	(GA)13	(GA)10	-	Introns/Intergenic	-	-	ACAATACGCTGTGCCAATCTGC	TATTGGCCGTGATGATGAAGTGC	258	A	A	A	RM12593

Polymorphic SSR (PMS) markers	Chromosomes	Start physical positions (bp)	End physical positions (bp)	Nipponbare	Kasalath	Nagina22	IR64	Pokkali	Bala	Tainung67	Azucena	Moroberekan	*Oryza rufipogon*	*Oryza nivara*	MSU gene locus IDs	Structural annotation	Functional annotation	SSR functional effects	Forward primers (5'-3')	Reverse primers (5'-3')	Amplified product size (bp)	Markers exhibiting polymorphism within *indica* rice	Transcrption factor genes-derived markers	Known cloned genes-derived markers	PMS markers corresponding with RM markers
PMS897	Os_Chr02	4607136	4607153	(GCG)6	(GCG)5	(GCG)6	(GCG)6	(GCG)6	(GCG)5	(GCG)6	(GCG)6	(GCG)5	(GCG)6	(GCG)6	LOC_Os02g08530	CDS	protein kinase family protein, putative, expressed	CODON_DELETION	AATGGGTGAAGGAAACATCG	ATCTTCTCCTCGAATGGCAG	238	P	A	A	NA
PMS898	Os_Chr02	4607935	4607960	(GA)13	(GA)13	(GA)13	(GA)13	(GA)18	(GA)13	(GA)13	(GA)13	(GA)13	(GA)13	(GA)13	-	Introns/Intergenic	-	-	GCATTGTTTCTTGTTGGGATGG	ACTGAAGTTGGTGTGGCTCTGC	149	P	A	A	RM12595
PMS899	Os_Chr02	4656267	4656287	(GGA)7	(GGA)7	(GGA)6	(GGA)7	(GGA)7	(GGA)7	(GGA)7	(GGA)7	(GGA)7	(GGA)7	(GGA)7	LOC_Os02g09090	CDS	expressed protein	CODON_DELETION	GGTGGCCAAGTACTGCCTGTGC	CACCATCCTGCCTCGACTTCC	206	P	A	A	RM12598
PMS900	Os_Chr02	4698988	4699023	(AG)18	(AG)18	(AG)14	(AG)18	(AG)18	(AG)18	(AG)18	(AG)18	(AG)18	(AG)18	(AG)18	-	Introns/Intergenic	-	-	ACACACGCAAACCACGTAAA	CACAACGTCGTCTCCAAGTG	220	P	A	A	NA
PMS901	Os_Chr02	4699351	4699400	(AG)25	(AG)25	(AG)25	(AG)25	(AG)25	(AG)25	(AG)25	(AG)8	(AG)25	(AG)25	(AG)25	-	Introns/Intergenic	-	-	AGAGAGGGTTGCTCTGCTCACC	ACTCTGCTGCGTACTCCTCTTGC	462	A	A	A	RM3497
PMS902	Os_Chr02	4716358	4716375	(GCC)6	(GCC)6	(GCC)6	(GCC)6	(GCC)6	(GCC)6	(GCC)6	(GCC)6	(GCC)6	(GCC)6	(GCC)5	LOC_Os02g09170	DRR	sucrose-phosphate synthase, putative, expressed	-	GGATGCTCTGCCATCTTCAT	GTGGCTTTTCACTCGATTCC	223	A	A	A	NA
PMS903	Os_Chr02	4873935	4873955	(GGC)7	(GGC)7	(GGC)7	(GGC)6	(GGC)6	(GGC)6	(GGC)6	(GGC)7	(GGC)7	(GGC)7	(GGC)7	LOC_Os02g09490	URR	GH2|OsCAD2	-	TCGTCTCTGGACACGAACAC	AGCGAGAGAGAGCGAATCAG	128	P	A	P	NA
PMS904	Os_Chr02	4944783	4944800	(CTC)6	(CTC)7	(CTC)7	(CTC)6	(CTC)6	(CTC)6	(CTC)6	(CTC)7	(CTC)7	(CTC)6	(CTC)6	LOC_Os02g09620	CDS	GDSL-like lipase/acylhydrolase, putative, expressed	CODON_INSERTION	CACTTCACTGCTTACACCGC	GTCCCGATCTGGTTGTTGTT	220	P	A	A	NA
PMS905	Os_Chr02	4950330	4950344	(GCC)5	(GCC)6	(GCC)5	(GCC)5	(GCC)5	(GCC)5	(GCC)5	(GCC)5	(GCC)5	(GCC)5	(GCC)5	LOC_Os02g09630	CDS	GA20292-PA, putative, expressed	CODON_INSERTION	CGTTCTGCTCGTCCCACAC	GGTACAGCAGCAACAGCAAC	234	P	A	A	NA
PMS906	Os_Chr02	4958981	4959001	(GCC)7	(GCC)7	(GCC)7	(GCC)5	(GCC)7	(GCC)7	(GCC)7	(GCC)7	(GCC)7	(GCC)7	(GCC)7	LOC_Os02g09650	CDS	AP2 domain containing protein, expressed	CODON_DELETION	AGGATCATGTCGGCTACGAG	AGTTCGACCTCAACCTGCCT	189	P	A	A	NA
PMS907	Os_Chr02	5006705	5006740	(AATT)9	(AATT)9	(AATT)9	(AATT)9	(AATT)9	(AATT)9	(AATT)5	(AATT)9	(AATT)9	(AATT)9	(AATT)6	-	Introns/Intergenic	-	-	ACATGCTGTCCTGCCTTTCTTCAACC	CAGATAGTAGCTAGCCACCGGCAAGG	339	A	A	A	RM12624
PMS908	Os_Chr02	5009363	5009398	(TA)18	(TA)12	(TA)6	(TA)6	(TA)10	(TA)16	(TA)18	(TA)6	(TA)18	(TA)18	(TA)18	-	Introns/Intergenic	-	-	GGCACCATTGTTGAAATTGA	ATCTACCGAGCCATGCATTC	257	P	A	A	NA
PMS909	Os_Chr02	5080319	5080398	(TA)40	(TA)40	(TA)40	(TA)6	(TA)40	(TA)40	(TA)40	(TA)36	(TA)40	(TA)12	(TA)40	-	Introns/Intergenic	-	-	GGCTTTATTTGTTCTGGCCTTGTGG	CCAAATCAAATGGGTCCTTTGTGC	452	P	A	A	RM12632

Polymorphic SSR (PMS) markers	Chromosomes	Start physical positions (bp)	End physical positions (bp)	Nipponbare	Kasalath	Nagina22	IR64	Pokkali	Bala	Tainung67	Azucena	Moroberekan	*Oryza rufipogon*	*Oryza nivara*	MSU gene locus IDs	Structural annotation	Functional annotation	SSR functional effects	Forward primers (5'-3')	Reverse primers (5'-3')	Amplified product size (bp)	Markers exhibiting polymorphism within *indica* rice	Transcrption factor genes-derived markers	Known cloned genes-derived markers	PMS markers corresponding with RM markers
PMS910	Os_Chr02	5086263	5086284	(TA)11	(TA)11	(TA)11	(TA)11	(TA)11	(TA)11	(TA)11	(TA)11	(TA)11	(TA)11	(TA)7	-	Introns/Intergenic	-	-	ATGGTGCCCCATATGAGTGT	TGAGAAATGGTTGAGATGATCG	132	A	A	A	NA
PMS911	Os_Chr02	5087399	5087410	(CT)6	(CT)7	(CT)6	(CT)6	(CT)6	(CT)6	(CT)6	(CT)6	(CT)6	(CT)6	(CT)6	-	Introns/Intergenic	-	-	TACCCTTTTTGCCCCTATCC	GTGACCCACTGCACATGAAC	228	P	A	A	NA
PMS912	Os_Chr02	5121417	5121437	(CGG)7	(CGG)6	(CGG)7	(CGG)7	(CGG)7	(CGG)6	(CGG)7	(CGG)7	(CGG)7	(CGG)6	(CGG)7	LOC_Os02g09890	URR	protein kinase PKN/PRK1, effector, putative, expressed	-	CAGCATGTCGTACGGGAAC	TCAGTGAGAAATCCGAAGCC	277	P	P	A	NA
PMS913	Os_Chr02	5121850	5121870	(GGC)7	(GGC)7	(GGC)5	(GGC)7	(GGC)7	(GGC)7	(GGC)7	(GGC)7	(GGC)7	(GGC)7	(GGC)7	LOC_Os02g09890	URR	protein kinase PKN/PRK1, effector, putative, expressed	-	GGAGGTGAGGCCGTGATTTGG	AGCTGCAGTTGCCGGAGTCG	118	P	P	A	RM12639
PMS914	Os_Chr02	5131451	5131468	(CCT)6	(CCT)5	(CCT)6	(CCT)6	(CCT)6	(CCT)6	(CCT)6	(CCT)6	(CCT)6	(CCT)6	(CCT)6	LOC_Os02g09910	URR	PHD-finger domain containing protein, putative, expressed	-	GCTTAACCCTCTCCTCGCTT	AGGGCTACGATCTAGGGCAT	145	P	A	A	NA
PMS915	Os_Chr02	5138258	5138277	(TA)10	(TA)8	(TA)8	(TA)10	(TA)10	(TA)10	(TA)10	(TA)10	(TA)6	(TA)8	(TA)10	-	Introns/Intergenic	-	-	GTTTGGCACGTGAGAATTGTAGC	GAGATGGATTGCAAGGAGTCTAGG	293	P	A	A	RM12642
PMS916	Os_Chr02	5209338	5209355	(CGG)6	(CGG)6	(CGG)6	(CGG)6	(CGG)6	(CGG)6	(CGG)5	(CGG)6	(CGG)6	(CGG)6	(CGG)6	LOC_Os02g10020	CDS	Mad3/BUB1 homology region 1 domain containing protein, expressed	CODON_CHANGE_PLUS_CODON_DELETION	GGCCCACATAAACTTTCCAA	ACCCCATCAGCATCAGAGTC	258	A	A	A	NA
PMS917	Os_Chr02	5316207	5316221	(CGG)5	(CGG)5	(CGG)5	(CGG)5	(CGG)5	(CGG)5	(CGG)5	(CGG)5	(CGG)5	(CGG)6	(CGG)5	LOC_Os02g10140	CDS	bZIP transcription factor domain containing protein, expressed	CODON_CHANGE_PLUS_CODON_INSERTION	CTTCTCCCAAGGGATCATCA	TTGAGCCACTCAGAATGTCG	262	A	P	A	NA
PMS918	Os_Chr02	5418553	5418577	(TCGTG)5	(TCGTG)5	(TCGTG)5	(TCGTG)6	(TCGTG)5	(TCGTG)5	(TCGTG)5	(TCGTG)5	(TCGTG)5	(TCGTG)5	(TCGTG)5	LOC_Os02g10310	URR	fumarylacetoacetase, putative, expressed	-	GAGGTTCTGGATGGGGAAGT	ATCTGCGCCTATTTATTGCG	197	P	A	A	NA
PMS919	Os_Chr02	5464180	5464197	(GGC)6	(GGC)6	(GGC)6	(GGC)6	(GGC)6	(GGC)5	(GGC)6	(GGC)6	(GGC)6	(GGC)6	(GGC)5	-	Introns/Intergenic	-	-	ACACAGCGAAGTCACACGTC	GACCTGAAACCCTAGACCCC	209	P	A	A	NA
PMS920	Os_Chr02	5548734	5548751	(CGG)6	(CGG)6	(CGG)6	(CGG)6	(CGG)6	(CGG)6	(CGG)5	(CGG)6	(CGG)6	(CGG)6	(CGG)6	LOC_Os02g10530	CDS	expressed protein	CODON_DELETION	CCCTCATCAACCAGTCCTTC	GGCTTCAGCTCCACCTTGT	103	A	A	A	NA
PMS921	Os_Chr02	5552036	5552056	(GGC)7	(GGC)7	(GGC)7	(GGC)7	(GGC)7	(GGC)7	(GGC)7	(GGC)7	(GGC)7	(GGC)7	(GGC)6	LOC_Os02g10540	URR	ribosomal protein L7/L12 C-terminal domain containing protein, expressed	-	CTAGGAGAGAAGCTAGGGCTAACG	AAAGGATCGAGCAGAGAAAGC	293	A	A	A	RM12666
PMS922	Os_Chr02	5571633	5571646	(TA)7	(TA)7	(TA)7	(TA)7	(TA)6	(TA)6	(TA)7	(TA)7	(TA)7	(TA)7	(TA)7	-	Introns/Intergenic	-	-	AATCGCTCGATGAACCTGTC	AAGGTGAAATTTACCGTGTGA	232	P	A	A	NA

Polymorphic SSR (PMS) markers	Chromosomes	Start physical positions (bp)	End physical positions (bp)	Nipponbare	Kasalath	Nagina22	IR64	Pokkali	Bala	Tainung67	Azucena	Moroberekan	*Oryza rufipogon*	*Oryza nivara*	MSU gene locus IDs	Structural annotation	Functional annotation	SSR functional effects	Forward primers (5'-3')	Reverse primers (5'-3')	Amplified product size (bp)	Markers exhibiting polymorphism within *indica* rice	Transcrption factor genes-derived markers	Known cloned genes-derived markers	PMS markers corresponding with RM markers
PMS923	Os_Chr02	5630995	5631009	(CAA)5	(CAA)5	(CAA)5	(CAA)5	(CAA)5	(CAA)6	(CAA)5	(CAA)5	(CAA)5	(CAA)7	(CAA)6	-	Introns/Intergenic	-	-	ACACGAGACACCCTCCATCT	CTATGGACTCGGAGGTGAGC	184	P	A	A	NA
PMS924	Os_Chr02	5644394	5644411	(CCT)6	(CCT)6	(CCT)6	(CCT)5	(CCT)6	(CCT)6	(CCT)6	(CCT)6	(CCT)6	(CCT)6	(CCT)5	LOC_Os02g10720	URR	expressed protein	-	CACCTCCACTCGAAACCCTA	TCGTGGTTGTGTTCGTCTGT	115	P	A	A	NA
PMS925	Os_Chr02	5806263	5806283	(CGG)7	(CGG)7	(CGG)7	(CGG)6	(CGG)7	(CGG)7	(CGG)7	(CGG)7	(CGG)7	(CGG)7	(CGG)7	-	Introns/Intergenic	-	-	GCACCTGCTCTCTGGAGACTCG	GCCTGATCGAGATCGCTTCTGC	104	P	A	A	RM12680
PMS926	Os_Chr02	5852348	5852371	(GCA)8	(GCA)7	(GCA)7	(GCA)8	(GCA)7	(GCA)8	(GCA)8	(GCA)8	(GCA)8	(GCA)8	(GCA)8	-	Introns/Intergenic	-	-	CGATAGCTTGCTGCTGTTTCTGC	TCAGATCAGCTCAAGGACATTAGCC	96	P	A	A	RM12682
PMS927	Os_Chr02	5853963	5853984	(TC)11	(TC)9	(TC)11	(TC)11	(TC)11	(TC)9	(TC)11	(TC)11	(TC)11	(TC)11	(TC)9	-	Introns/Intergenic	-	-	TGTGTCTGTGCTAGCAGCCT	CAGAGAGAGAGCCCAGAGGA	193	P	A	A	NA
PMS928	Os_Chr02	5857444	5857461	(TA)9	(TA)9	(TA)6	(TA)9	(TA)9	(TA)9	(TA)9	(TA)8	(TA)9	(TA)9	(TA)9	-	Introns/Intergenic	-	-	AAAGCTGGCTTCTCTTGTCG	TCGGCAGGGAAATGATTTAG	188	P	A	A	NA
PMS929	Os_Chr02	5924681	5924700	(CA)10	(CA)10	(CA)10	(CA)10	(CA)10	(CA)10	(CA)9	(CA)10	(CA)10	(CA)10	(CA)10	-	Introns/Intergenic	-	-	TCCTCAATCAAGGAACAAGACAGC	TTTATATAGCGCGTCGGAGATGG	180	A	A	A	RM12685
PMS930	Os_Chr02	5957808	5957875	(AT)34	(AT)32	(AT)9	(AT)15	(AT)21	(AT)33	(AT)34	(AT)34	(AT)34	(AT)34	(AT)6	-	Introns/Intergenic	-	-	TGTTAGCGGCATTCTCAAAA	GCAGTTTGAAAAGAGTGCCA	278	P	A	A	NA
PMS931	Os_Chr02	5972255	5972312	(AT)29	(AT)29	(AT)8	(AT)9	(AT)10	(AT)29	(AT)6	(AT)29	(AT)29	(AT)29	(AT)29	-	Introns/Intergenic	-	-	ACCTTTTAGGCACCTCCGAT	GTGCTACCAACCGGGACTAA	216	P	A	A	NA
PMS932	Os_Chr02	6053850	6053883	(AT)17	(AT)17	(AT)17	(AT)17	(AT)17	(AT)15	(AT)17	(AT)9	(AT)17	(AT)17	(AT)8	-	Introns/Intergenic	-	-	AAATTGAACAAATGTTGCTGGA	GTCGAAACAATGGAGGTGGT	237	P	A	A	NA
PMS933	Os_Chr02	6089004	6089024	(CAG)7	(CAG)5	(CAG)7	(CAG)7	(CAG)7	(CAG)5	(CAG)7	(CAG)7	(CAG)7	(CAG)7	(CAG)7	LOC_Os02g11780	CDS	transcription factor S-II, central domain containing protein, expressed	CODON_DELETION	AGCTTGTCACCCAACAACAGC	GTTGCTGGGTATCTGAGGTTGC	188	P	P	A	RM12692
PMS934	Os_Chr02	6089192	6089206	(CGC)5	(CGC)5	(CGC)5	(CGC)5	(CGC)5	(CGC)5	(CGC)5	(CGC)5	(CGC)5	(CGC)6	(CGC)5	LOC_Os02g11780	CDS	transcription factor S-II, central domain containing protein, expressed	CODON_CHANGE_PLUS_CODON_INSERTION	CTATCCCCCAGGCTTACCTC	CACACCATACTGCGACTGCT	176	A	P	A	NA
PMS935	Os_Chr02	6322800	6322855	(AT)28	(AT)14	(AT)28	(AT)28	(AT)28	(AT)28	(AT)7	(AT)9	(AT)28	(AT)28	(AT)28	-	Introns/Intergenic	-	-	GTAAGTATATCGGTCGTTGTCG	CATGCATCGTACCAGTAAGG	630	P	A	A	RM2483

Polymorphic SSR (PMS) markers	Chromosomes	Start physical positions (bp)	End physical positions (bp)	Nipponbare	Kasalath	Nagina22	IR64	Pokkali	Bala	Tainung67	Azucena	Moroberekan	*Oryza rufipogon*	*Oryza nivara*	MSU gene locus IDs	Structural annotation	Functional annotation	SSR functional effects	Forward primers (5'-3')	Reverse primers (5'-3')	Amplified product size (bp)	Markers exhibiting polymorphism within *indica* rice	Transcrption factor genes-derived markers	Known cloned genes-derived markers	PMS markers corresponding with RM markers
PMS936	Os_Chr02	6329557	6329572	(TA)8	(TA)8	(TA)8	(TA)8	(TA)8	(TA)8	(TA)8	(TA)6	(TA)8	(TA)8	(TA)8	-	Introns/Intergenic	-	-	ACGCATATGGTTGGTTTGGT	AGTGCAAAACCAGGGAAAAA	147	A	A	A	NA
PMS937	Os_Chr02	6405198	6405212	(CAA)5	(CAA)6	(CAA)5	(CAA)5	(CAA)5	(CAA)5	(CAA)5	(CAA)5	(CAA)5	(CAA)5	(CAA)5	LOC_Os02g12310	CDS	no apical meristem protein, putative, expressed	CODON_CHANGE_PLUS_CODON_INSERTION	CCGGTACTTCTTCACGATGG	GGAAGACGATTCACCGAAGA	279	P	P	A	NA
PMS938	Os_Chr02	6421176	6421193	(GCG)6	(GCG)6	(GCG)6	(GCG)6	(GCG)6	(GCG)5	(GCG)6	(GCG)6	(GCG)6	(GCG)6	(GCG)6	-	Introns/Intergenic	-	-	ACAAGAGTTACCCACGGTCG	TTCCGGCCCAACACTTATAG	252	P	A	A	NA
PMS939	Os_Chr02	6445868	6445905	(TA)19	(TA)19	(TA)11	(TA)19	(TA)15	(TA)19	(TA)19	(TA)19	(TA)8	(TA)19	(TA)19	-	Introns/Intergenic	-	-	TGGTGTTGGTAACTAATCCCTTGC	CATTTCCCACCTCACATTTCTCC	266	P	A	A	RM12713
PMS940	Os_Chr02	6475156	6475176	(CCG)7	(CCG)7	(CCG)7	(CCG)7	(CCG)7	(CCG)6	(CCG)7	(CCG)7	(CCG)7	(CCG)7	(CCG)7	-	Introns/Intergenic	-	-	GTCGTCGACAGCCTCTCC	TCCTCTCTCTCACTCGAGGC	192	P	A	A	NA
PMS941	Os_Chr02	6550390	6550405	(GA)8	(GA)6	(GA)8	(GA)8	(GA)8	(GA)8	(GA)8	(GA)8	(GA)8	(GA)8	(GA)8	-	Introns/Intergenic	-	-	CCCTTCAGAGCTCCTCCAA	GACCCCCTTTCCACTACTCC	253	P	A	A	NA
PMS942	Os_Chr02	6653762	6653782	(AAT)7	(AAT)7	(AAT)7	(AAT)7	(AAT)7	(AAT)6	(AAT)7	(AAT)7	(AAT)7	(AAT)7	(AAT)7	-	Introns/Intergenic	-	-	GAATGGTGCAACGTTGTGATTGG	GCAATGCATCTACAACCACACTGC	368	P	A	A	RM12722
PMS943	Os_Chr02	6696412	6696473	(TA)31	(TA)31	(TA)10	(TA)12	(TA)31	(TA)31	(TA)31	(TA)31	(TA)31	(TA)31	(TA)31	-	Introns/Intergenic	-	-	ACACCTTTCGAAGCACTTTCACG	GCTGAAATTAAGGGCCATGTAGC	274	P	A	A	RM12724
PMS944	Os_Chr02	6702871	6702888	(TC)9	(TC)9	(TC)9	(TC)9	(TC)9	(TC)7	(TC)9	(TC)9	(TC)9	(TC)9	(TC)7	-	Introns/Intergenic	-	-	ATGGAGGGAGTATGTGCCAG	TCCCATCGGATCTAGCAGAG	256	P	A	A	NA
PMS945	Os_Chr02	6710928	6710967	(AT)20	(AT)20	(AT)20	(AT)20	(AT)20	(AT)6	(AT)20	(AT)20	(AT)7	(AT)20	(AT)20	-	Introns/Intergenic	-	-	CGGAGGATCAAATTCCAAGA	TTTGCCCTTTCATGGACCTA	227	P	A	A	NA
PMS946	Os_Chr02	6958507	6958521	(CGA)5	(CGA)5	(CGA)5	(CGA)5	(CGA)5	(CGA)5	(CGA)5	(CGA)6	(CGA)5	(CGA)5	(CGA)5	LOC_Os02g13100	CDS	protein phosphatase 2C, putative, expressed	CODON_INSERTION	GTTCATGATCCTGAGCCACC	CCTCCACTCCTCCAGCATCT	175	A	A	A	NA
PMS947	Os_Chr02	7017266	7017295	(GA)15	(GA)15	(GA)15	(GA)15	(GA)15	(GA)15	(GA)15	(GA)12	(GA)15	(GA)15	(GA)15	-	Introns/Intergenic	-	-	GGAGGGACACCTACCTCGAACG	CTCCCATCTCGCCCTTAGTCC	254	A	A	A	RM12745
PMS948	Os_Chr02	7063678	7063701	(AGC)8	(AGC)8	(AGC)8	(AGC)8	(AGC)8	(AGC)8	(AGC)8	(AGC)8	(AGC)8	(AGC)8	(AGC)6	LOC_Os02g13260	CDS	F-box family protein, putative, expressed	CODON_CHANGE_PLUS_CODON_DELETION	GAGAAGTTCCCCAAGACCGT	TGTACTGGTGCTCGTCCTTG	214	A	A	A	NA

Polymorphic SSR (PMS) markers	Chromosomes	Start physical positions (bp)	End physical positions (bp)	Nipponbare	Kasalath	Nagina22	IR64	Pokkali	Bala	Tainung67	Azucena	Moroberekan	*Oryza rufipogon*	*Oryza nivara*	MSU gene locus IDs	Structural annotation	Functional annotation	SSR functional effects	Forward primers (5'-3')	Reverse primers (5'-3')	Amplified product size (bp)	Markers exhibiting polymorphism within *indica* rice	Transcrption factor genes-derived markers	Known cloned genes-derived markers	PMS markers corresponding with RM markers
PMS949	Os_Chr02	7074066	7074121	(AT)28	(AT)22	(AT)9	(AT)28	(AT)27	(AT)28	(AT)28	(AT)28	(AT)28	(AT)28	(AT)28	-	Introns/Intergenic	-	-	TGTTCGATCGTATCGGCATA	CGGTCCAAAATTCGTATGGT	267	P	A	A	NA
PMS950	Os_Chr02	7137680	7137701	(TC)11	(TC)11	(TC)11	(TC)11	(TC)11	(TC)10	(TC)11	(TC)11	(TC)11	(TC)11	(TC)11	-	Introns/Intergenic	-	-	TGGGAAGCCTTAGATAAACAGAGC	AAGGACGGTGTGAGATTTCTTGG	163	P	A	A	RM12750
PMS951	Os_Chr02	7143776	7143791	(AG)8	(AG)8	(AG)8	(AG)8	(AG)6	(AG)8	(AG)8	(AG)8	(AG)8	(AG)8	(AG)8	LOC_Os02g13390	CDS	expressed protein	FRAME_SHIFT	TACTCGTCGTTCGCTGACTG	AGCTCCATGGGGAAATCTCT	234	P	A	A	NA
PMS952	Os_Chr02	7171966	7172001	(TA)18	(TA)18	(TA)7	(TA)18	(TA)18	(TA)18	(TA)8	(TA)18	(TA)18	(TA)18	(TA)18	-	Introns/Intergenic	-	-	CACCCAGGCTTTCAACACTCC	ATGCCTCATAGCCGATAATCTCC	177	P	A	A	RM12752
PMS953	Os_Chr02	7230786	7230801	(TG)8	(TG)8	(TG)8	(TG)8	(TG)8	(TG)8	(TG)8	(TG)8	(TG)8	(TG)8	(TG)7	-	Introns/Intergenic	-	-	CCCACCATTACAATATTCCCC	TTGCAGCCACCCTAATAACC	117	A	A	A	NA
PMS954	Os_Chr02	7251154	7251165	(GA)6	(GA)7	(GA)6	(GA)6	(GA)6	(GA)7	(GA)6	(GA)6	(GA)6	(GA)6	(GA)7	-	Introns/Intergenic	-	-	CATTTTGGACTTTGGAGGGA	CGACGTGACGTGGTTGTCTA	267	P	A	A	NA
PMS955	Os_Chr02	7285819	7285840	(CT)11	(CT)11	(CT)11	(CT)11	(CT)11	(CT)6	(CT)11	(CT)11	(CT)11	(CT)11	(CT)11	-	Introns/Intergenic	-	-	GAAAGCGAGGTGAAACGAAGC	CTAGGCCCAAGGTTTACCAAACG	285	P	A	A	RM492
PMS956	Os_Chr02	7321477	7321497	(CGC)7	(CGC)5	(CGC)5	(CGC)5	(CGC)5	(CGC)5	(CGC)7	(CGC)7	(CGC)7	(CGC)7	(CGC)5	LOC_Os02g13650	URR	expressed protein	-	AAGCAGGCGTAGAGGAGATGACG	CCTCTCCTTGACCCACCACTCG	653	A	A	A	RM12765
PMS957	Os_Chr02	7338296	7338310	(CCG)5	(CCG)6	(CCG)5	(CCG)5	(CCG)5	(CCG)6	(CCG)5	(CCG)5	(CCG)5	(CCG)6	(CCG)5	-	Introns/Intergenic	-	-	GGGAGAGGGGAGAGAAGC	TCTATCTCCTCCTTTCAGACG	411	P	A	A	NA
PMS958	Os_Chr02	7353256	7353283	(TC)14	(TC)11	(TC)11	(TC)11	(TC)14	(TC)11	(TC)14	(TC)14	(TC)14	(TC)14	(TC)14	-	Introns/Intergenic	-	-	GAAGCAGAAGGCGTAACCTATCG	CACAGCAGAAAGAGACACACAGC	152	P	A	A	RM12769
PMS959	Os_Chr02	7365952	7365966	(GCG)5	(GCG)6	(GCG)5	(GCG)5	(GCG)5	(GCG)5	(GCG)5	(GCG)5	(GCG)5	(GCG)5	(GCG)5	LOC_Os02g13710	CDS	ethylene-responsive transcription factor TINY, putative, expressed	CODON_INSERTION	CAAGAACGGCGGTGTCAG	GTTCCATAAGAGGGCACCAA	280	P	A	A	NA
PMS960	Os_Chr02	7384889	7384912	(AAT)8	(AAT)9	(AAT)8	(AAT)8	(AAT)8	(AAT)8	(AAT)8	(AAT)8	(AAT)8	(AAT)9	(AAT)8	-	Introns/Intergenic	-	-	TTCCTCCACTAGAGCCATCTCG	ACCGTGGTCTTGCCTATGTGG	154	P	A	A	RM12775
PMS961	Os_Chr02	7523483	7523512	(ATATAG)5	(ATATAG)6	(ATATAG)6	(ATATAG)6	(ATATAG)6	(ATATAG)6	(ATATAG)5	(ATATAG)5	(ATATAG)6	(ATATAG)6	(ATATAG)6	-	Introns/Intergenic	-	-	CACAAATGACTAACCGCGAA	TATGAAAGCATACACCCGCA	229	A	A	A	NA

Polymorphic SSR (PMS) markers	Chromosomes	Start physical positions (bp)	End physical positions (bp)	Nipponbare	Kasalath	Nagina22	IR64	Pokkali	Bala	Tainung67	Azucena	Moroberekan	*Oryza rufipogon*	*Oryza nivara*	MSU gene locus IDs	Structural annotation	Functional annotation	SSR functional effects	Forward primers (5'-3')	Reverse primers (5'-3')	Amplified product size (bp)	Markers exhibiting polymorphism within *indica* rice	Transcrption factor genes-derived markers	Known cloned genes-derived markers	PMS markers corresponding with RM markers
PMS962	Os_Chr02	7568900	7568920	(CGG)7	(CGG)7	(CGG)7	(CGG)7	(CGG)7	(CGG)7	(CGG)5	(CGG)7	(CGG)7	(CGG)6	(CGG)7	-	Introns/Intergenic	-	-	GGGCCTCTGTCAGTCTCAAGG	AACTGTCAACCACAGGCTACTTCG	109	A	A	A	RM12791
PMS963	Os_Chr02	7689214	7689234	(GCG)7	(GCG)7	(GCG)7	(GCG)7	(GCG)7	(GCG)7	(GCG)5	(GCG)7	(GCG)7	(GCG)7	(GCG)7	-	Introns/Intergenic	-	-	AGGACACCACCACTGACTCC	GACGAGGTCCTTTTCGCAG	226	A	A	A	NA
PMS964	Os_Chr02	7706972	7707033	(TC)31	(TC)31	(TC)15	(TC)31	(TC)31	(TC)31	(TC)31	(TC)31	(TC)31	(TC)31	(TC)31	-	Introns/Intergenic	-	-	CACTGCTTTGCTCTCCAGTCTCC	CAACATCTCCGTCAGAATCAAGC	337	P	A	A	RM145,OSR9A
PMS965	Os_Chr02	7987817	7987840	(CGG)8	(CGG)8	(CGG)8	(CGG)5	(CGG)5	(CGG)8	(CGG)8	(CGG)8	(CGG)8	(CGG)8	(CGG)5	LOC_Os02g14490	CDS	MYB family transcription factor, putative, expressed	CODON_CHANGE_PLUS_CODON_DELETION	GATCACCATCGTCTTCCGTT	AAACGGAGTACAGCGTTCGT	204	P	P	A	NA
PMS966	Os_Chr02	8077914	8077959	(TA)23	(TA)23	(TA)23	(TA)23	(TA)23	(TA)23	(TA)23	(TA)7	(TA)6	(TA)23	(TA)23	-	Introns/Intergenic	-	-	CGTCCCTTAGTTGTAGCCGA	TGACATGTAGCCCCCACATA	230	A	A	A	NA
PMS967	Os_Chr02	8106448	8106489	(TA)21	(TA)20	(TA)21	(TA)8	(TA)21	(TA)21	(TA)6	(TA)21	(TA)10	(TA)21	(TA)21	-	Introns/Intergenic	-	-	TGATTTTGTGCATTTGAAACC	TCCTCGCCTCAATTGTTTTT	180	P	A	A	NA
PMS968	Os_Chr02	8108438	8108458	(AGG)7	(AGG)7	(AGG)7	(AGG)7	(AGG)7	(AGG)5	(AGG)7	(AGG)7	(AGG)7	(AGG)7	(AGG)7	LOC_Os02g14700	CDS	expressed protein	CODON_DELETION	CCTTGAGAAACCAGAGGCAG	CCCTTAGTCTCCTGTCAGCG	198	P	A	A	NA
PMS969	Os_Chr02	8139533	8139550	(CA)9	(CA)9	(CA)9	(CA)11	(CA)9	(CA)9	(CA)9	(CA)9	(CA)9	(CA)10	(CA)10	-	Introns/Intergenic	-	-	AATGTGGGGTTGAGAGAAGC	CAAACACACATGCACACCCT	268	P	A	A	NA
PMS970	Os_Chr02	8160035	8160048	(AG)7	(AG)7	(AG)7	(AG)7	(AG)7	(AG)7	(AG)7	(AG)6	(AG)7	(AG)7	(AG)7	-	Introns/Intergenic	-	-	ATTTCAACCCGCTTCGTATG	CGTGGCATGATATGGTAACG	202	A	A	A	NA
PMS971	Os_Chr02	8186015	8186080	(AGA)22	(AGA)22	(AGA)22	(AGA)22	(AGA)22	(AGA)22	(AGA)22	(AGA)10	(AGA)22	(AGA)22	(AGA)22	-	Introns/Intergenic	-	-	CTTATTCCTTGGCCCCAAAT	TCATGGACGAATGAAAGCAA	248	A	A	A	NA
PMS972	Os_Chr02	8271420	8271433	(TA)7	(TA)8	(TA)8	(TA)7	(TA)7	(TA)8	(TA)7	(TA)7	(TA)7	(TA)7	(TA)7	-	Introns/Intergenic	-	-	TGAGAGCGAACGTAGAGGCT	TTACGTGACCAACGATCGAG	144	P	A	A	NA
PMS973	Os_Chr02	8321982	8322065	(TTA)28	(TTA)28	(TTA)28	(TTA)28	(TTA)28	(TTA)28	(TTA)28	(TTA)26	(TTA)28	(TTA)28	(TTA)28	-	Introns/Intergenic	-	-	TTTCAAATCTCAGCTCCCTAGC	AGGGCTTCAAATTAAGGTGTGG	258	A	A	A	RM12843
PMS974	Os_Chr02	8338747	8338767	(GAT)7	(GAT)7	(GAT)7	(GAT)7	(GAT)7	(GAT)7	(GAT)7	(GAT)7	(GAT)7	(GAT)6	(GAT)7	-	Introns/Intergenic	-	-	GGCTGGGAAAGAAGTGTCAG	TGAGACTCATCGGACTGTGC	225	A	A	A	NA

Polymorphic SSR (PMS) markers	Chromosomes	Start physical positions (bp)	End physical positions (bp)	Nipponbare	Kasalath	Nagina22	IR64	Pokkali	Bala	Tainung67	Azucena	Moroberekan	*Oryza rufipogon*	*Oryza nivara*	MSU gene locus IDs	Structural annotation	Functional annotation	SSR functional effects	Forward primers (5'-3')	Reverse primers (5'-3')	Amplified product size (bp)	Markers exhibiting polymorphism within *indica* rice	Transcrption factor genes-derived markers	Known cloned genes-derived markers	PMS markers corresponding with RM markers
PMS975	Os_Chr02	8375519	8375536	(CCG)6	(CCG)6	(CCG)6	(CCG)6	(CCG)6	(CCG)6	(CCG)6	(CCG)6	(CCG)6	(CCG)5	(CCG)6	LOC_Os02g15010	CDS	zinc finger, C3HC4 type domain containing protein, expressed	CODON_DELETION	CTTCACCATCTCCCCCTTCT	GCACATGTACGTGCTGGAAC	172	A	A	A	NA
PMS976	Os_Chr02	8398393	8398416	(GCG)8	(GCG)8	(GCG)8	(GCG)8	(GCG)8	(GCG)6	(GCG)8	(GCG)8	(GCG)8	(GCG)8	(GCG)8	-	Introns/Intergenic	-	-	CTTGCGCGGCGAATGATATAGG	AACTTGACCGGAAACAAACACACG	137	P	A	A	RM12845
PMS977	Os_Chr02	8563847	8563880	(AT)17	(AT)17	(AT)17	(AT)17	(AT)17	(AT)17	(AT)17	(AT)7	(AT)17	(AT)17	(AT)17	-	Introns/Intergenic	-	-	GCTTGCACGAATGGTTCCTAGC	GTAGGATCGAGCCAAGCTTCTCC	370	A	A	A	RM12850
PMS978	Os_Chr02	8578531	8578606	(TA)38	(TA)38	(TA)13	(TA)11	(TA)9	(TA)38	(TA)38	(TA)38	(TA)38	(TA)38	(TA)38	-	Introns/Intergenic	-	-	CGCTACGTATAAACAAGCAACACG	CACTTATTTGTCAGTGGTCGAAGC	510	P	A	A	RM12852
PMS979	Os_Chr02	8601666	8601683	(CGG)6	(CGG)6	(CGG)6	(CGG)6	(CGG)6	(CGG)6	(CGG)6	(CGG)6	(CGG)5	(CGG)5	(CGG)6	LOC_Os02g15360	CDS	expressed protein	CODON_DELETION	GAGGAGATGAGGGTGGAAGC	AGTCCAAACTCAACCGCAAC	243	A	A	A	NA
PMS980	Os_Chr02	8607833	8607856	(CAG)8	(CAG)5	(CAG)8	(CAG)8	(CAG)8	(CAG)5	(CAG)8	(CAG)8	(CAG)8	(CAG)8	(CAG)8	LOC_Os02g15390	CDS	BED zinc finger family protein, expressed	CODON_DELETION	AACTGCTTGTCTTCGACCATCG	CGTCTTGCAGAATAATGCATCG	153	P	A	A	RM12855
PMS981	Os_Chr02	8631285	8631306	(TC)11	(TC)11	(TC)11	(TC)11	(TC)11	(TC)11	(TC)11	(TC)11	(TC)6	(TC)11	(TC)9	-	Introns/Intergenic	-	-	TTGTCGTCTAGTGTCCGTGC	ATAGAGGCGGGGAGAGAGAG	279	A	A	A	NA
PMS982	Os_Chr02	8710229	8710252	(AGAT)6	(AGAT)6	(AGAT)6	(AGAT)6	(AGAT)6	(AGAT)6	(AGAT)5	(AGAT)6	(AGAT)6	(AGAT)6	(AGAT)6	-	Introns/Intergenic	-	-	TCAGTTAGCACGAAACCAACC	AACATCCAGATTCTAGAGGCTTCC	92	A	A	A	RM12866
PMS983	Os_Chr02	8730243	8730257	(CCA)5	(CCA)6	(CCA)5	(CCA)5	(CCA)5	(CCA)6	(CCA)5	(CCA)5	(CCA)5	(CCA)5	(CCA)5	LOC_Os02g15550	CDS	spermidine synthase, putative, expressed	CODON_INSERTION	ACAACAACGTCACATACCGC	ACTTCGAGGAGGAGCAGGAT	259	P	A	A	NA
PMS984	Os_Chr02	8741300	8741320	(ATC)7	(ATC)6	(ATC)7	(ATC)7	(ATC)7	(ATC)6	(ATC)7	(ATC)7	(ATC)7	(ATC)7	(ATC)7	LOC_Os02g15570	CDS	expressed protein	CODON_DELETION	ACTCATCAACATCGCGAAACAGC	CTGACTCCGACAACAGCTATGAACC	144	P	A	A	RM12868
PMS985	Os_Chr02	8882816	8882831	(AG)8	(AG)8	(AG)6	(AG)8	(AG)8	(AG)8	(AG)8	(AG)8	(AG)8	(AG)7	(AG)8	-	Introns/Intergenic	-	-	ATCGATTGATCTCCGCAAAC	GTGCTACACATGGACAACGG	197	P	A	A	NA
PMS986	Os_Chr02	8927663	8927690	(TC)14	(TC)14	(TC)14	(TC)14	(TC)14	(TC)14	(TC)14	(TC)13	(TC)14	(TC)14	(TC)14	-	Introns/Intergenic	-	-	TCTCCTCCATCCACCTGTAAAGC	GCTCGAGTGTCACGTACTTAATTGG	188	A	A	A	RM12885
PMS987	Os_Chr02	8927995	8928012	(CGG)6	(CGG)5	(CGG)6	(CGG)6	(CGG)6	(CGG)5	(CGG)6	(CGG)6	(CGG)6	(CGG)6	(CGG)6	LOC_Os02g15800	CDS	expressed protein	CODON_CHANGE_PLUS_CODON_DELETION	TCGAGCTCCATCATCACAAG	TAGCCGTAGTAGGCCTCGAA	250	P	A	A	NA

**Polymorphic SSR (PMS) markers**	**Chromosomes**	**Start physical positions (bp)**	**End physical positions (bp)**	**Nipponbare**	**Kasalath**	**Nagina22**	**IR64**	**Pokkali**	**Bala**	**Tainung67**	**Azucena**	**Moroberekan**	***Oryza rufipogon***	***Oryza nivara***	**MSU gene locus IDs**	**Structural annotation**	**Functional annotation**	**SSR functional effects**	**Forward primers (5'-3')**	**Reverse primers (5'-3')**	**Amplified product size (bp)**	**Markers exhibiting polymorphism within *indica* rice**	**Transcrption factor genes-derived markers**	**Known cloned genes-derived markers**	**PMS markers corresponding with RM markers**
PMS988	Os_Chr02	8935452	8935469	(CGC)6	(CGC)5	(CGC)5	(CGC)5	(CGC)5	(CGC)5	(CGC)6	(CGC)6	(CGC)6	(CGC)6	(CGC)5	LOC_Os02g15810	CDS	HMG1/2, putative, expressed	CODON_DELETION	GAAGAGAATCCTCGTCAGCG	CAAGAGGGACATGGAGGAGT	148	A	A	A	NA
PMS989	Os_Chr02	8956929	8956952	(AGC)8	(AGC)5	(AGC)8	(AGC)8	(AGC)8	(AGC)5	(AGC)8	(AGC)7	(AGC)7	(AGC)5	(AGC)5	-	Introns/Intergenic	-	-	TGCATTTCTTGCCACAGGTAGG	TCAACACGCACATCCTGTACCC	184	P	A	A	RM6853
PMS990	Os_Chr02	9082822	9082848	(CCT)9	(CCT)9	(CCT)9	(CCT)9	(CCT)9	(CCT)9	(CCT)9	(CCT)8	(CCT)9	(CCT)9	(CCT)7	LOC_Os02g16000	URR	E1-BTB1 - Bric-a-Brac, Tramtrack, and Broad Complex domain with E1 subfamily conserved sequence, expressed	-	CTTGTCGTCCAACCCTCCTCTCC	CGAGTTGAAGGCGAACTCGAAGC	193	A	A	A	RM12899
PMS991	Os_Chr02	9163004	9163029	(TC)13	(TC)13	(TC)13	(TC)13	(TC)10	(TC)13	(TC)13	(TC)13	(TC)13	(TC)9	(TC)13	-	Introns/Intergenic	-	-	TTTGCTCATTCCTTATCCCC	CTCTCTGCCACCGATCCTAC	148	P	A	A	NA
PMS992	Os_Chr02	9314806	9314821	(GA)8	(GA)6	(GA)8	(GA)8	(GA)8	(GA)6	(GA)8	(GA)8	(GA)8	(GA)8	(GA)8	-	Introns/Intergenic	-	-	ATTCAAGAGGGAAATTGGGG	CAGGTCGTCATCGTCTTCCT	174	P	A	A	NA
PMS993	Os_Chr02	9362040	9362055	(GA)8	(GA)6	(GA)8	(GA)8	(GA)8	(GA)6	(GA)8	(GA)8	(GA)8	(GA)8	(GA)8	-	Introns/Intergenic	-	-	ATTCAAGAGGGAAATTGGGG	CAGGTCGTCATCGTCTTCCT	174	P	A	A	NA
PMS994	Os_Chr02	9418480	9418497	(GA)9	(GA)8	(GA)8	(GA)8	(GA)8	(GA)8	(GA)9	(GA)9	(GA)9	(GA)9	(GA)9	-	Introns/Intergenic	-	-	ACAACCAGGGAACCAGTCAG	GTCAGCGACGGTGAGGATA	152	A	A	A	NA
PMS995	Os_Chr02	9463485	9463499	(CTC)5	(CTC)5	(CTC)5	(CTC)5	(CTC)5	(CTC)5	(CTC)5	(CTC)5	(CTC)5	(CTC)6	(CTC)5	LOC_Os02g16550	URR	Ser/Thr protein phosphatase family protein, putative, expressed	-	AGCATGGACTTGAACTTGGC	GGGAAGGAGGGGAGGAGTA	118	A	A	A	NA
PMS996	Os_Chr02	9464892	9464905	(TA)7	(TA)7	(TA)7	(TA)7	(TA)6	(TA)7	(TA)7	(TA)8	(TA)8	(TA)8	(TA)7	-	Introns/Intergenic	-	-	TTGACCGTTCGTCCTATTCA	GAACCTTGGAGGGTCAAACA	237	P	A	A	NA
PMS997	Os_Chr02	9503007	9503086	(CT)40	(CT)39	(CT)40	(CT)40	(CT)40	(CT)40	(CT)40	(CT)40	(CT)40	(CT)40	(CT)40	-	Introns/Intergenic	-	-	TCGTATTTCCCGGTGTCTCAGG	ACTAGTACTCGGTGCAGGGAATCG	269	P	A	A	RM12921
PMS998	Os_Chr02	9563429	9563455	(CGA)9	(CGA)9	(CGA)9	(CGA)9	(CGA)9	(CGA)9	(CGA)8	(CGA)9	(CGA)9	(CGA)8	(CGA)9	LOC_Os02g16760	CDS	OsFBX41 - F-box domain containing protein, expressed	CODON_DELETION	GTGGACTTGGCGAGATGCTACG	GTTAAGGGCAGCCACCAGATCG	243	A	A	A	RM452
PMS999	Os_Chr02	9610959	9611010	(TA)26	(TA)26	(TA)26	(TA)8	(TA)26	(TA)26	(TA)26	(TA)7	(TA)10	(TA)26	(TA)22	-	Introns/Intergenic	-	-	TCAATTTCAACAATGGTGCC	GGCCTTCACTTCCTTTTTCC	233	P	A	A	NA
PMS1000	Os_Chr02	9863221	9863248	(CT)14	(CT)14	(CT)14	(CT)14	(CT)14	(CT)14	(CT)14	(CT)10	(CT)14	(CT)14	(CT)14	-	Introns/Intergenic	-	-	CCTCACCCAACCTATGCTTATCTCC	TCAATTTGGCGGAGAGGAGAGG	185	A	A	A	RM12939

Polymorphic SSR (PMS) markers	Chromosomes	Start physical positions (bp)	End physical positions (bp)	Nipponbare	Kasalath	Nagina22	IR64	Pokkali	Bala	Tainung67	Azucena	Moroberekan	*Oryza rufipogon*	*Oryza nivara*	MSU gene locus IDs	Structural annotation	Functional annotation	SSR functional effects	Forward primers (5'-3')	Reverse primers (5'-3')	Amplified product size (bp)	Markers exhibiting polymorphism within *indica* rice	Transcrption factor genes-derived markers	Known cloned genes-derived markers	PMS markers corresponding with RM markers
PMS1001	Os_Chr02	9893562	9893588	(CGG)9	(CGG)9	(CGG)9	(CGG)7	(CGG)7	(CGG)9	(CGG)9	(CGG)9	(CGG)9	(CGG)6	(CGG)9	LOC_Os02g17250	URR	expressed protein	-	AACACGATCCCGAGGCTGACC	CCATGCCAAACAACCGGTACG	442	P	A	A	RM12942
PMS1002	Os_Chr02	9933218	9933235	(TAA)6	(TAA)6	(TAA)6	(TAA)6	(TAA)6	(TAA)6	(TAA)6	(TAA)6	(TAA)6	(TAA)5	(TAA)6	-	Introns/Intergenic	-	-	GCTCAATTTTGATCGTCCGT	TGGAAGTACTCCATCCGTCC	205	A	A	A	NA
PMS1003	Os_Chr02	10013366	10013389	(GGC)8	(GGC)8	(GGC)8	(GGC)8	(GGC)8	(GGC)8	(GGC)6	(GGC)8	(GGC)8	(GGC)8	(GGC)6	LOC_Os02g17400	CDS	leucine rich repeat protein, putative, expressed	CODON_DELETION	CTGGTGAAGCCAGCTTTGAT	ATTAAGCTGAGAGCCCGAGA	216	A	A	A	NA
PMS1004	Os_Chr02	10063175	10063190	(CT)8	(CT)8	(CT)7	(CT)8	(CT)8	(CT)8	(CT)8	(CT)8	(CT)8	(CT)8	(CT)8	-	Introns/Intergenic	-	-	TTCCGTCTGACCCTCTTTTG	CACGCACGAGTGAGAGAGAG	172	P	A	A	NA
PMS1005	Os_Chr02	10099909	10099954	(TA)23	(TA)23	(TA)23	(TA)23	(TA)23	(TA)23	(TA)23	(TA)23	(TA)7	(TA)10	(TA)23	-	Introns/Intergenic	-	-	TCAATTTCAACAATGGTGCC	GGAATTTTGGAGCAATTGGA	197	A	A	A	NA
PMS1006	Os_Chr02	10135305	10135326	(TA)11	(TA)11	(TA)11	(TA)11	(TA)11	(TA)12	(TA)11	(TA)11	(TA)6	(TA)11	(TA)11	LOC_Os02g17620	DRR	isochorismatase family protein, putative, expressed	-	CTCGTGGCTGGTGTAAGACAACC	TATACGCAAGACCACCACCTTCC	363	P	A	A	RM12962
PMS1007	Os_Chr02	10185621	10185668	(CT)24	(CT)23	(CT)24	(CT)24	(CT)24	(CT)24	(CT)24	(CT)24	(CT)24	(CT)24	(CT)24	-	Introns/Intergenic	-	-	AAAGGTGTGTGATGGCTTGTGG	AGGGCTCTACTATGCACCAATGC	350	P	A	A	RM1358
PMS1008	Os_Chr02	10193849	10193869	(GAC)7	(GAC)7	(GAC)7	(GAC)7	(GAC)7	(GAC)7	(GAC)7	(GAC)7	(GAC)7	(GAC)7	(GAC)6	-	Introns/Intergenic	-	-	GGCTTCCTCCTCCTCGAC	AGATTCACCTGCCTGCAAAG	216	A	A	A	NA
PMS1009	Os_Chr02	10235648	10235675	(TA)14	(TA)14	(TA)14	(TA)14	(TA)14	(TA)14	(TA)14	(TA)14	(TA)14	(TA)14	(TA)9	-	Introns/Intergenic	-	-	ATAGGCTAGCAGCCAGCAATGG	TACGTACCCGAGGGAGGTTGG	81	A	A	A	RM12969
PMS1010	Os_Chr02	10283524	10283538	(CCG)5	(CCG)6	(CCG)5	(CCG)5	(CCG)5	(CCG)5	(CCG)5	(CCG)5	(CCG)5	(CCG)5	(CCG)5	LOC_Os02g17780	CDS	OsCPS|OsCPS1	CODON_INSERTION	ATGCACAACCACCTTTAGCC	ATCGCATCTCCATCATCTCC	241	P	A	P	NA
PMS1011	Os_Chr02	10312746	10312805	(TA)30	(TA)30	(TA)6	(TA)30	(TA)9	(TA)30	(TA)30	(TA)30	(TA)17	(TA)30	(TA)13	-	Introns/Intergenic	-	-	AAACGGAGGGAGTAGTAGATGACC	ACACCACACCAACTAACCTGACC	290	P	A	A	RM12977
PMS1012	Os_Chr02	10320517	10320546	(CATCC)6	(CATCC)6	(CATCC)6	(CATCC)5	(CATCC)5	(CATCC)6	(CATCC)6	(CATCC)6	(CATCC)6	(CATCC)6	(CATCC)6	-	Introns/Intergenic	-	-	CCTGAACCAAACACCCCTAA	GAGGCATCTGACAAAGCACA	247	P	A	A	NA
PMS1013	Os_Chr02	10335909	10335930	(AT)11	(AT)9	(AT)11	(AT)11	(AT)11	(AT)11	(AT)11	(AT)11	(AT)11	(AT)11	(AT)11	-	Introns/Intergenic	-	-	CAAGAAGGCAAGAATAGCCG	TTTTTGGGCCATTATTCGAG	262	P	A	A	NA

Polymorphic SSR (PMS) markers	Chromosomes	Start physical positions (bp)	End physical positions (bp)	Nipponbare	Kasalath	Nagina22	IR64	Pokkali	Bala	Tainung67	Azucena	Moroberekan	*Oryza rufipogon*	*Oryza nivara*	MSU gene locus IDs	Structural annotation	Functional annotation	SSR functional effects	Forward primers (5'-3')	Reverse primers (5'-3')	Amplified product size (bp)	Markers exhibiting polymorphism within *indica* rice	Transcrption factor genes-derived markers	Known cloned genes-derived markers	PMS markers corresponding with RM markers
PMS1014	Os_Chr02	10468846	10468869	(TC)12	(TC)12	(TC)12	(TC)12	(TC)12	(TC)12	(TC)11	(TC)12	(TC)12	(TC)12	(TC)12	-	Introns/Intergenic	-	-	CCTGATCTCGGCTTCTCCTT	GTCGGTGTCGTTGGCAGTA	268	A	A	A	NA
PMS1015	Os_Chr02	10621795	10621810	(CG)8	(CG)8	(CG)8	(CG)8	(CG)8	(CG)8	(CG)8	(CG)8	(CG)8	(CG)7	(CG)8	-	Introns/Intergenic	-	-	CCACCTCTGGATTTAAGCGA	GGTTAGTGGCTGCAAAAGGA	183	A	A	A	NA
PMS1016	Os_Chr02	10679091	10679126	(AT)18	(AT)18	(AT)18	(AT)18	(AT)18	(AT)9	(AT)8	(AT)18	(AT)18	(AT)18	(AT)18	-	Introns/Intergenic	-	-	ATCCCTCCTCTTTGGGATGT	ATATGCCACGGTCGAAAGAA	211	P	A	A	NA
PMS1017	Os_Chr02	10737848	10737859	(CT)6	(CT)6	(CT)6	(CT)6	(CT)7	(CT)6	(CT)6	(CT)6	(CT)6	(CT)6	(CT)6	-	Introns/Intergenic	-	-	GCCAAAGCAACCAGCTAGAC	CGTCGACATGAGTGGTAGGA	225	P	A	A	NA
PMS1018	Os_Chr02	10792913	10793000	(TA)44	(TA)44	(TA)6	(TA)16	(TA)10	(TA)44	(TA)44	(TA)44	(TA)44	(TA)44	(TA)7	-	Introns/Intergenic	-	-	GTTTTTGCGCAGGTCATTTT	CATTGGACTAGGGACCAACAA	269	P	A	A	NA
PMS1019	Os_Chr02	10807209	10807236	(TC)14	(TC)14	(TC)14	(TC)15	(TC)15	(TC)14	(TC)14	(TC)14	(TC)14	(TC)14	(TC)15	-	Introns/Intergenic	-	-	ATGACCCAATTTCTGACTCTAGCC	CATGGGTGGTGCTGTAGATGG	475	P	A	A	RM290,RM5390,RM521
PMS1020	Os_Chr02	10826769	10826814	(AT)23	(AT)23	(AT)23	(AT)23	(AT)7	(AT)6	(AT)23	(AT)23	(AT)23	(AT)17	(AT)23	-	Introns/Intergenic	-	-	TTCACAACAAAATCTTACGTGATT	CGTACATCCAAGGGTAGAGGG	280	P	A	A	NA
PMS1021	Os_Chr02	10879955	10879968	(GA)7	(GA)7	(GA)7	(GA)7	(GA)7	(GA)7	(GA)7	(GA)7	(GA)7	(GA)6	(GA)7	-	Introns/Intergenic	-	-	AGCCGCGAGAGAGAAAAGA	CCCATGAACCATAAAAACGG	217	A	A	A	NA
PMS1022	Os_Chr02	10921682	10921705	(TA)12	(TA)12	(TA)12	(TA)12	(TA)12	(TA)12	(TA)12	(TA)12	(TA)11	(TA)15	(TA)12	-	Introns/Intergenic	-	-	ACGATCGATCTGTTGCATTG	CCCATACTCCATGTAGCACCA	190	A	A	A	NA
PMS1023	Os_Chr02	11004951	11004968	(CTC)6	(CTC)6	(CTC)6	(CTC)6	(CTC)6	(CTC)6	(CTC)6	(CTC)6	(CTC)6	(CTC)5	(CTC)5	-	Introns/Intergenic	-	-	GCTGAAGCTAAGCGCCAC	GTGAGGTCGTCGTCGGAT	246	A	A	A	NA
PMS1024	Os_Chr02	11193693	11193710	(GAG)6	(GAG)5	(GAG)6	(GAG)6	(GAG)6	(GAG)5	(GAG)6	(GAG)6	(GAG)6	(GAG)6	(GAG)6	LOC_Os02g19200	CDS	OsFBX46 - F-box domain containing protein, expressed	CODON_DELETION	GATCTCGCGGAGGAGGTC	CCCAAGTCTCCATTTGTTGG	172	P	A	A	NA
PMS1025	Os_Chr02	11199407	11199424	(TC)9	(TC)9	(TC)9	(TC)8	(TC)9	(TC)9	(TC)9	(TC)6	(TC)6	(TC)8	(TC)9	-	Introns/Intergenic	-	-	TAAGGCCCATTTCGAGTTTG	ATGCTTTCGTTCCTCGTCAC	257	P	A	A	NA
PMS1026	Os_Chr02	11245860	11245911	(ATAG)13	(ATAG)12	(ATAG)14	(ATAG)14	(ATAG)14	(ATAG)14	(ATAG)13	(ATAG)13	(ATAG)13	(ATAG)13	(ATAG)14	-	Introns/Intergenic	-	-	GAGTATCACTTGATAGCTTGGGAACG	TCCTGCTATAGGCTAGGTTGTAACG	358	P	A	A	RM13016

Polymorphic SSR (PMS) markers	Chromosomes	Start physical positions (bp)	End physical positions (bp)	Nipponbare	Kasalath	Nagina22	IR64	Pokkali	Bala	Tainung67	Azucena	Moroberekan	*Oryza rufipogon*	*Oryza nivara*	MSU gene locus IDs	Structural annotation	Functional annotation	SSR functional effects	Forward primers (5'-3')	Reverse primers (5'-3')	Amplified product size (bp)	Markers exhibiting polymorphism within *indica* rice	Transcrption factor genes-derived markers	Known cloned genes-derived markers	PMS markers corresponding with RM markers
PMS1027	Os_Chr02	11361535	11361552	(CCG)6	(CCG)5	(CCG)6	(CCG)5	(CCG)6	(CCG)5	(CCG)6	(CCG)6	(CCG)6	(CCG)6	(CCG)6	LOC_Os02g19450	CDS	ATPase, AAA family protein, expressed	CODON_DELETION	CCACCGGCAATAATAATCCA	GGGAGAGGATGCAGACCAC	175	P	A	A	NA
PMS1028	Os_Chr02	11431756	11431771	(CT)8	(CT)8	(CT)11	(CT)11	(CT)8	(CT)11	(CT)8	(CT)8	(CT)8	(CT)8	(CT)8	-	Introns/Intergenic	-	-	CTCTGTGTATCCGCGTTGAA	TCTCCCTCCAACCCTTCTTT	267	P	A	A	NA
PMS1029	Os_Chr02	11463346	11463365	(GA)10	(GA)9	(GA)9	(GA)9	(GA)10	(GA)9	(GA)10	(GA)10	(GA)10	(GA)10	(GA)9	-	Introns/Intergenic	-	-	GGGCTAATCGGGAGACAGTTCACC	GCGTCCATCAAGGTGCTCAACG	93	P	A	A	RM13024
PMS1030	Os_Chr02	11466624	11466655	(TCTG)8	(TCTG)8	(TCTG)6	(TCTG)6	(TCTG)8	(TCTG)6	(TCTG)8	(TCTG)8	(TCTG)8	(TCTG)8	(TCTG)6	-	Introns/Intergenic	-	-	CTGTGAGTGGGTCTTGCTGA	TGACGCGCAGTACATACACA	132	P	A	A	NA
PMS1031	Os_Chr02	11592773	11592790	(GGC)6	(GGC)6	(GGC)5	(GGC)5	(GGC)5	(GGC)5	(GGC)6	(GGC)6	(GGC)6	(GGC)6	(GGC)5	LOC_Os02g19820	CDS	nodulin MtN3 family protein, putative, expressed	CODON_DELETION	CTGGTGAAGTAAAGAGGGCG	CTACAAGTCGACGCAGCAGA	169	P	A	A	NA
PMS1032	Os_Chr02	11596625	11596644	(CT)10	(CT)10	(CT)10	(CT)7	(CT)10	(CT)7	(CT)10	(CT)10	(CT)10	(CT)10	(CT)7	-	Introns/Intergenic	-	-	TGACTCCGGTCTTTCTCTTGG	GGTGTCCCGTTGTCAGTACG	283	P	A	A	RM13036
PMS1033	Os_Chr02	11674075	11674130	(TA)28	(TA)28	(TA)28	(TA)28	(TA)28	(TA)28	(TA)7	(TA)28	(TA)28	(TA)28	(TA)28	-	Introns/Intergenic	-	-	ATGGGAGAGCAATGTAGATAGG	CGTGCTATCCTTATACTTCTGG	244	A	A	A	RM13039
PMS1034	Os_Chr02	11684889	11684916	(TA)14	(TA)13	(TA)14	(TA)14	(TA)13	(TA)13	(TA)14	(TA)14	(TA)14	(TA)14	(TA)9	-	Introns/Intergenic	-	-	ATCTAATACGTGGCGCCGGTAGC	TACGAAGATGGGCAGGATGTACG	190	P	A	A	RM13041
PMS1035	Os_Chr02	11775175	11775190	(AC)8	(AC)10	(AC)8	(AC)8	(AC)8	(AC)8	(AC)8	(AC)8	(AC)8	(AC)8	(AC)8	-	Introns/Intergenic	-	-	CTGTGCTGTCGATTGAGCTG	GCATTCCAATGCAAAGTAACC	207	P	A	A	NA
PMS1036	Os_Chr02	12091238	12091265	(CT)14	(CT)14	(CT)14	(CT)14	(CT)15	(CT)15	(CT)14	(CT)14	(CT)14	(CT)14	(CT)15	-	Introns/Intergenic	-	-	GTTGGATTGCAGGAGGAGAA	GATCCAATCCAAGCCGTAAA	150	P	A	A	NA
PMS1037	Os_Chr02	12093586	12093599	(TA)7	(TA)7	(TA)7	(TA)7	(TA)7	(TA)8	(TA)7	(TA)7	(TA)7	(TA)7	(TA)7	-	Introns/Intergenic	-	-	CACACAAAGCATAGGGAGCA	TCCCTTCTCTTTGGGCTGT	170	P	A	A	NA
PMS1038	Os_Chr02	12229314	12229339	(TA)13	(TA)13	(TA)13	(TA)7	(TA)13	(TA)13	(TA)13	(TA)13	(TA)13	(TA)13	(TA)13	-	Introns/Intergenic	-	-	CGTACTCGCGCCGTTCTTAGTCC	GAAAGGCAGAGGACGACGTGTGG	606	P	A	A	RM13062
PMS1039	Os_Chr02	12348001	12348014	(AC)7	(AC)7	(AC)7	(AC)7	(AC)7	(AC)8	(AC)7	(AC)7	(AC)7	(AC)7	(AC)7	-	Introns/Intergenic	-	-	GGAAAAACTTTCAGCAAGCG	ATAAACCTATGCCCTTGGGG	159	P	A	A	NA

Polymorphic SSR (PMS) markers	Chromosomes	Start physical positions (bp)	End physical positions (bp)	Nipponbare	Kasalath	Nagina22	IR64	Pokkali	Bala	Tainung67	Azucena	Moroberekan	*Oryza rufipogon*	*Oryza nivara*	MSU gene locus IDs	Structural annotation	Functional annotation	SSR functional effects	Forward primers (5'-3')	Reverse primers (5'-3')	Amplified product size (bp)	Markers exhibiting polymorphism within *indica* rice	Transcrption factor genes-derived markers	Known cloned genes-derived markers	PMS markers corresponding with RM markers
PMS1040	Os_Chr02	12353500	12353517	(TA)9	(TA)8	(TA)9	(TA)9	(TA)9	(TA)9	(TA)9	(TA)9	(TA)9	(TA)9	(TA)9	-	Introns/Intergenic	-	-	TTCAGAAGATTCAAACACCACC	AGGTCAAGTTGGGACATCCA	279	P	A	A	NA
PMS1041	Os_Chr02	12490335	12490352	(GCG)6	(GCG)5	(GCG)6	(GCG)6	(GCG)6	(GCG)6	(GCG)6	(GCG)6	(GCG)6	(GCG)6	(GCG)6	-	Introns/Intergenic	-	-	ATCCCAATCCTCACCTCTCC	GCTCCCTCTCTCCGCTTC	241	P	A	A	NA
PMS1042	Os_Chr02	12514397	12514410	(GA)7	(GA)6	(GA)7	(GA)7	(GA)7	(GA)6	(GA)7	(GA)7	(GA)7	(GA)7	(GA)6	-	Introns/Intergenic	-	-	ATTCCCGTAGCCACTGTCAC	CCGCCTAGAAGTCTGACCTG	178	P	A	A	NA
PMS1043	Os_Chr02	12517484	12517539	(CT)28	(CT)27	(CT)28	(CT)28	(CT)28	(CT)28	(CT)28	(CT)23	(CT)28	(CT)28	(CT)14	-	Introns/Intergenic	-	-	GGACTTGGCCATATCTCCTATCACG	CCAAAGGCGGTGAGATACGG	410	P	A	A	RM13067
PMS1044	Os_Chr02	12651406	12651423	(GGA)6	(GGA)5	(GGA)5	(GGA)5	(GGA)5	(GGA)5	(GGA)6	(GGA)6	(GGA)6	(GGA)6	(GGA)6	LOC_Os02g21310	URR	expressed protein	-	GACACCGGAGAACATGGAGT	AATCTTCCCACTTGCCCTCT	201	A	A	A	NA
PMS1045	Os_Chr02	12666920	12667005	(TA)43	(TA)43	(TA)7	(TA)8	(TA)7	(TA)43	(TA)43	(TA)10	(TA)9	(TA)43	(TA)8	-	Introns/Intergenic	-	-	AGAGCAGTTGGAGACGACTTTCC	ATGTGGACATGCCCTCAGTAAGC	256	P	A	A	RM5210
PMS1046	Os_Chr02	12800407	12800424	(GA)9	(GA)8	(GA)9	(GA)9	(GA)9	(GA)9	(GA)9	(GA)9	(GA)9	(GA)9	(GA)9	-	Introns/Intergenic	-	-	TGCCATGGATCAAAAACTCA	CCATCTCATCCCCATTCATC	205	P	A	A	NA
PMS1047	Os_Chr02	12860235	12860252	(CGG)6	(CGG)6	(CGG)6	(CGG)6	(CGG)6	(CGG)6	(CGG)6	(CGG)6	(CGG)6	(CGG)5	(CGG)6	LOC_Os02g21650	CDS	prefoldin, putative, expressed	CODON_DELETION	CTCTACAGCATGGCCGAGAT	ATAAGGGTATCCCCAACCCA	215	A	A	A	NA
PMS1048	Os_Chr02	12998324	12998341	(AT)9	(AT)9	(AT)9	(AT)9	(AT)8	(AT)9	(AT)9	(AT)9	(AT)9	(AT)9	(AT)9	-	Introns/Intergenic	-	-	TTATACCGTTGTGTTCGTTGC	TGCATTCATAATGCTCTAAATTG	200	P	A	A	NA
PMS1049	Os_Chr02	13085920	13085939	(AT)10	(AT)10	(AT)10	(AT)7	(AT)10	(AT)10	(AT)10	(AT)10	(AT)10	(AT)10	(AT)10	-	Introns/Intergenic	-	-	TTCCCATCTTTAACACCTTCAGA	GAAAACAAGGAACGAGAGCG	180	P	A	A	NA
PMS1050	Os_Chr02	13127299	13127322	(GAG)8	(GAG)7	(GAG)8	(GAG)8	(GAG)8	(GAG)7	(GAG)8	(GAG)8	(GAG)8	(GAG)8	(GAG)8	LOC_Os02g22060	URR	expressed protein	-	ACCAGCTTGGGCGAGTTAGAGG	CTCCACCTCCACTTCCTCATCG	84	P	A	A	RM13090
PMS1051	Os_Chr02	13458427	13458468	(TCTCTA)7	(TCTCTA)7	(TCTCTA)7	(TCTCTA)7	(TCTCTA)7	(TCTCTA)7	(TCTCTA)7	(TCTCTA)7	(TCTCTA)7	(TCTCTA)7	(TCTCTA)6	-	Introns/Intergenic	-	-	ACCACCATCACCACCGTAAT	TTTTGGAAAGCGAGCAAACT	251	A	A	A	NA
PMS1052	Os_Chr02	13779607	13779624	(CT)9	(CT)9	(CT)9	(CT)9	(CT)9	(CT)7	(CT)9	(CT)9	(CT)9	(CT)9	(CT)9	-	Introns/Intergenic	-	-	TAACCCCCTCTTTCCCTTTG	GGAGGGAGAGGGAGAGAAAA	113	P	A	A	NA

Polymorphic SSR (PMS) markers	Chromosomes	Start physical positions (bp)	End physical positions (bp)	Nipponbare	Kasalath	Nagina22	IR64	Pokkali	Bala	Tainung67	Azucena	Moroberekan	*Oryza rufipogon*	*Oryza nivara*	MSU gene locus IDs	Structural annotation	Functional annotation	SSR functional effects	Forward primers (5'-3')	Reverse primers (5'-3')	Amplified product size (bp)	Markers exhibiting polymorphism within *indica* rice	Transcrption factor genes-derived markers	Known cloned genes-derived markers	PMS markers corresponding with RM markers
PMS1053	Os_Chr02	13830234	13830277	(AT)22	(AT)22	(AT)22	(AT)22	(AT)22	(AT)22	(AT)22	(AT)8	(AT)9	(AT)9	(AT)9	-	Introns/Intergenic	-	-	ATCCTAACCAGTGGTAATAGGG	AGTAGCGAGCCATATACAGAGG	274	A	A	A	RM13110
PMS1054	Os_Chr02	13849614	13849643	(TA)15	(TA)15	(TA)15	(TA)15	(TA)15	(TA)15	(TA)15	(TA)10	(TA)15	(TA)10	(TA)15	-	Introns/Intergenic	-	-	GTGCATAGAAAAAGACAGACG	ATGTCCTATGTAAACTTCGTAT	233	A	A	A	NA
PMS1055	Os_Chr02	13959138	13959179	(CT)21	(CT)21	(CT)21	(CT)21	(CT)21	(CT)21	(CT)21	(CT)21	(CT)21	(CT)15	(CT)21	-	Introns/Intergenic	-	-	ATGGCCGGGATTTGAATTT	GCGGTGATGCTTAAATAGGG	155	A	A	A	NA
PMS1056	Os_Chr02	13959550	13959567	(CT)9	(CT)9	(CT)9	(CT)9	(CT)9	(CT)9	(CT)9	(CT)7	(CT)9	(CT)9	(CT)9	-	Introns/Intergenic	-	-	ACCTCTCCTCCGTCGGAAT	CCACCTAGCGAACATGATGA	155	A	A	A	NA
PMS1057	Os_Chr02	13962991	13963014	(AT)12	(AT)12	(AT)12	(AT)12	(AT)12	(AT)12	(AT)12	(AT)12	(AT)12	(AT)12	(AT)6	-	Introns/Intergenic	-	-	TGCTATGGTGGTAGGGAGGA	TGCCATGGCTGAATGTGTAT	140	A	A	A	NA
PMS1058	Os_Chr02	14010542	14010553	(CT)6	(CT)6	(CT)6	(CT)6	(CT)6	(CT)6	(CT)6	(CT)6	(CT)6	(CT)6	(CT)7	-	Introns/Intergenic	-	-	CGACGAGTAGGACCACCCT	CTGCTAGCGGGAGAGAGAGA	264	A	A	A	NA
PMS1059	Os_Chr02	14133159	14133174	(AG)8	(AG)8	(AG)8	(AG)8	(AG)8	(AG)8	(AG)8	(AG)8	(AG)8	(AG)6	(AG)8	-	Introns/Intergenic	-	-	CAGCCATAGAAACCATCCGT	TGGTAGTTGAAGCTGTTGCG	171	A	A	A	NA
PMS1060	Os_Chr02	14202492	14202509	(GTC)6	(GTC)6	(GTC)6	(GTC)6	(GTC)6	(GTC)6	(GTC)6	(GTC)5	(GTC)6	(GTC)6	(GTC)6	-	Introns/Intergenic	-	-	GTCGTCTTCACCGTTCATCC	TGCTGCACACTTGACGAAAC	244	A	A	A	NA
PMS1061	Os_Chr02	14302451	14302464	(GA)7	(GA)7	(GA)7	(GA)7	(GA)7	(GA)7	(GA)6	(GA)7	(GA)7	(GA)7	(GA)7	-	Introns/Intergenic	-	-	GGAGGGAGAGGGAGAGAAAA	CCTTGGTTTAAAAGGAGCCC	222	A	A	A	NA
PMS1062	Os_Chr02	14334728	14334785	(AT)29	(AT)29	(AT)14	(AT)29	(AT)10	(AT)14	(AT)8	(AT)11	(AT)6	(AT)29	(AT)29	-	Introns/Intergenic	-	-	AGTAGGTGGATGAGCAGATGTGG	CATCACTCCTCTCCAAGAAAGAGC	492	P	A	A	RM13123
PMS1063	Os_Chr02	14336881	14336904	(GGC)8	(GGC)8	(GGC)8	(GGC)8	(GGC)8	(GGC)8	(GGC)6	(GGC)8	(GGC)8	(GGC)8	(GGC)8	-	Introns/Intergenic	-	-	CTGAGGGAAAGGGAATGGATAGG	CATGCGGCCAACAACTTTCC	269	A	A	A	RM13125
PMS1064	Os_Chr02	14443959	14443986	(CT)14	(CT)14	(CT)14	(CT)14	(CT)14	(CT)14	(CT)14	(CT)14	(CT)14	(CT)14	(CT)12	LOC_Os02g24920	CDS	expressed protein	FRAME_SHIFT	GTTGACCGGTTGACATAGTGAGC	GGAGCTCTGACTTGGTGATTTACG	155	A	A	A	RM13129
PMS1065	Os_Chr02	14509329	14509348	(CT)10	(CT)10	(CT)10	(CT)10	(CT)10	(CT)10	(CT)10	(CT)10	(CT)10	(CT)13	(CT)10	LOC_Os02g25020	DRR	DNA binding protein, putative, expressed	-	GAGGATTTCCGTGGGAATTATGC	AGTCAACAGCGAGATCAGTTAAGAGG	176	A	A	A	RM13133

Polymorphic SSR (PMS) markers	Chromosomes	Start physical positions (bp)	End physical positions (bp)	Nipponbare	Kasalath	Nagina22	IR64	Pokkali	Bala	Tainung67	Azucena	Moroberekan	*Oryza rufipogon*	*Oryza nivara*	MSU gene locus IDs	Structural annotation	Functional annotation	SSR functional effects	Forward primers (5'-3')	Reverse primers (5'-3')	Amplified product size (bp)	Markers exhibiting polymorphism within *indica* rice	Transcrption factor genes-derived markers	Known cloned genes-derived markers	PMS markers corresponding with RM markers
PMS1066	Os_Chr02	14592151	14592178	(AC)14	(AC)8	(AC)14	(AC)14	(AC)14	(AC)8	(AC)14	(AC)14	(AC)14	(AC)11	(AC)14	-	Introns/Intergenic	-	-	CTACCTCGAGCCGGAAGTGAGG	TCGTCGACCTTGGTCAGTTAAGG	292	P	A	A	RM1037
PMS1067	Os_Chr02	14689447	14689544	(TA)49	(TA)49	(TA)10	(TA)49	(TA)49	(TA)49	(TA)49	(TA)49	(TA)11	(TA)49	(TA)49	-	Introns/Intergenic	-	-	ACGGACGACGGAAGCATTAT	ACGTCTGCCGTTCTGTTTTT	279	P	A	A	NA
PMS1068	Os_Chr02	14827720	14827747	(AT)14	(AT)14	(AT)9	(AT)9	(AT)14	(AT)14	(AT)14	(AT)14	(AT)14	(AT)15	(AT)14	-	Introns/Intergenic	-	-	CATGCAGGTGTTCAAAGATTCG	TAAACCCAACATGTTCCTCTGC	510	P	A	A	RM13148
PMS1069	Os_Chr02	14945140	14945163	(CTAC)6	(CTAC)5	(CTAC)5	(CTAC)5	(CTAC)5	(CTAC)5	(CTAC)6	(CTAC)6	(CTAC)6	(CTAC)5	(CTAC)5	-	Introns/Intergenic	-	-	AGAGGGTAGCGAAGCGAAGTAGG	CTCTTGCGTGCTTAACTCAGTGG	135	A	A	A	RM13152
PMS1070	Os_Chr02	15397254	15397293	(TA)20	(TA)20	(TA)20	(TA)20	(TA)20	(TA)20	(TA)20	(TA)6	(TA)7	(TA)20	(TA)20	-	Introns/Intergenic	-	-	GGGATTAACACGACAAGCTAATGG	CTGCCAGAGATCAAGCAGAAAGG	571	A	A	A	RM13159
PMS1071	Os_Chr02	15481641	15481661	(ACT)7	(ACT)5	(ACT)7	(ACT)7	(ACT)7	(ACT)7	(ACT)7	(ACT)7	(ACT)7	(ACT)7	(ACT)7	-	Introns/Intergenic	-	-	ATGCCAAAAACCACCTCAAG	CCTGACCCCTTTTCCTTCTC	158	P	A	A	NA
PMS1072	Os_Chr02	15667514	15667583	(TA)35	(TA)35	(TA)9	(TA)7	(TA)10	(TA)7	(TA)35	(TA)35	(TA)6	(TA)33	(TA)35	-	Introns/Intergenic	-	-	GGTTAACTAATGGATGTCAACCG	TGCCATTAACTAATAGATGCCCA	264	P	A	A	NA
PMS1073	Os_Chr02	15708616	15708630	(GAG)5	(GAG)6	(GAG)5	(GAG)5	(GAG)5	(GAG)6	(GAG)5	(GAG)5	(GAG)5	(GAG)5	(GAG)5	LOC_Os02g26740	CDS	transposon protein, putative, Pong sub-class, expressed	CODON_CHANGE_PLUS_CODON_INSERTION	CTCATGTTTCAGGGTCGGAT	TTGCTAGATCGGCAAGACCT	209	P	A	A	NA
PMS1074	Os_Chr02	15805658	15805707	(TA)25	(TA)25	(TA)7	(TA)10	(TA)8	(TA)7	(TA)25	(TA)25	(TA)25	(TA)25	(TA)10	-	Introns/Intergenic	-	-	AAGAGTACACATGGCAAACGATGG	AATTTCGGTCAGACTGCGATCC	359	P	A	A	RM13171
PMS1075	Os_Chr02	15829744	15829787	(AT)22	(AT)15	(AT)6	(AT)6	(AT)6	(AT)15	(AT)22	(AT)22	(AT)6	(AT)6	(AT)22	-	Introns/Intergenic	-	-	CATTGACTCATTGAGGTTAGGG	CCTCGCTAACTGCCTTACTAGC	361	P	A	A	RM13172
PMS1076	Os_Chr02	15969017	15969044	(AATG)7	(AATG)7	(AATG)5	(AATG)5	(AATG)7	(AATG)7	(AATG)7	(AATG)7	(AATG)7	(AATG)7	(AATG)6	-	Introns/Intergenic	-	-	TAGGAAGGCAAGCAATGAACAGG	CGAGTGTATGGTCAGGTTGATGG	259	P	A	A	RM13181
PMS1077	Os_Chr02	15996364	15996381	(GCC)6	(GCC)5	(GCC)6	(GCC)6	(GCC)6	(GCC)6	(GCC)6	(GCC)6	(GCC)6	(GCC)6	(GCC)6	LOC_Os02g27180	CDS	retrotransposon, putative, centromere-specific, expressed	CODON_DELETION	TCCCTCTTTCTCCCCTTAGC	GGACCACGTCCACAAAGG	268	P	A	A	NA
PMS1078	Os_Chr02	16037174	16037195	(AT)11	(AT)10	(AT)11	(AT)11	(AT)11	(AT)10	(AT)11	(AT)11	(AT)11	(AT)11	(AT)11	-	Introns/Intergenic	-	-	CGCCAACACTTTCATCTGAACC	AAGTTCATGAAAGCCAGACACTCC	407	P	A	A	RM13183

Polymorphic SSR (PMS) markers	Chromosomes	Start physical positions (bp)	End physical positions (bp)	Nipponbare	Kasalath	Nagina22	IR64	Pokkali	Bala	Tainung67	Azucena	Moroberekan	*Oryza rufipogon*	*Oryza nivara*	MSU gene locus IDs	Structural annotation	Functional annotation	SSR functional effects	Forward primers (5'-3')	Reverse primers (5'-3')	Amplified product size (bp)	Markers exhibiting polymorphism within *indica* rice	Transcrption factor genes-derived markers	Known cloned genes-derived markers	PMS markers corresponding with RM markers
PMS1079	Os_Chr02	16095256	16095271	(AT)8	(AT)8	(AT)7	(AT)8	(AT)8	(AT)8	(AT)8	(AT)9	(AT)9	(AT)8	(AT)8	-	Introns/Intergenic	-	-	CTCTTGCTCCAATTCCCTCTCTTTCC	CTTGCTTCACCTGTCAGACTCATGG	293	P	A	A	RM13186
PMS1080	Os_Chr02	16202552	16202572	(CGG)7	(CGG)7	(CGG)7	(CGG)7	(CGG)7	(CGG)7	(CGG)5	(CGG)7	(CGG)7	(CGG)7	(CGG)5	-	Introns/Intergenic	-	-	AAAGATACCGGGCCCACACAGC	GCTCACCTGGTTGAGGCTCTCC	455	A	A	A	RM13190
PMS1081	Os_Chr02	16318959	16318974	(AT)8	(AT)8	(AT)8	(AT)7	(AT)8	(AT)8	(AT)8	(AT)8	(AT)8	(AT)8	(AT)8	-	Introns/Intergenic	-	-	TCCATTGTAGAGGTAAAAATATG	CCCAATAGCTAACCTTCAAAT	293	P	A	A	NA
PMS1082	Os_Chr02	16370817	16370838	(TA)11	(TA)11	(TA)11	(TA)11	(TA)11	(TA)11	(TA)11	(TA)11	(TA)11	(TA)11	(TA)7	-	Introns/Intergenic	-	-	ATTAGTGGGAGCTGTTGGGCTAGG	ACGCAAGCATTGATGGAGTGG	481	A	A	A	RM13194
PMS1083	Os_Chr02	16542175	16542186	(CA)6	(CA)7	(CA)7	(CA)7	(CA)7	(CA)7	(CA)6	(CA)7	(CA)6	(CA)7	(CA)7	-	Introns/Intergenic	-	-	CCCTCCACTAGTTCCACACA	GCATCTTGCTGAGGTAAGCC	184	A	A	A	NA
PMS1084	Os_Chr02	16547474	16547491	(TG)9	(TG)9	(TG)9	(TG)9	(TG)9	(TG)10	(TG)9	(TG)9	(TG)9	(TG)9	(TG)10	-	Introns/Intergenic	-	-	GGATGAGCCCCTTAACAACA	CATTCCCGATTGGAGTGATT	272	P	A	A	NA
PMS1085	Os_Chr02	16621154	16621185	(TA)16	(TA)16	(TA)16	(TA)16	(TA)16	(TA)9	(TA)16	(TA)16	(TA)16	(TA)16	(TA)16	-	Introns/Intergenic	-	-	TTGGAGCCCATACTCCATGT	AAGGCGTACACATGCACATT	120	P	A	A	NA
PMS1086	Os_Chr02	16689112	16689125	(TA)7	(TA)7	(TA)7	(TA)7	(TA)7	(TA)7	(TA)7	(TA)6	(TA)7	(TA)7	(TA)7	-	Introns/Intergenic	-	-	AACATGCAAAAATGCCACCT	TCCCACACTTTTATTATATGGATGA	189	A	A	A	NA
PMS1087	Os_Chr02	16761356	16761385	(CTCGC)6	(CTCGC)6	(CTCGC)6	(CTCGC)5	(CTCGC)5	(CTCGC)6	(CTCGC)5	(CTCGC)6	(CTCGC)6	(CTCGC)6	(CTCGC)6	-	Introns/Intergenic	-	-	AGCCACTTGAAGCATGTGTG	GCAGCAAAACAAAACTGCAA	195	P	A	A	NA
PMS1088	Os_Chr02	16774255	16774276	(AT)11	(AT)11	(AT)8	(AT)11	(AT)11	(AT)7	(AT)11	(AT)11	(AT)8	(AT)11	(AT)11	-	Introns/Intergenic	-	-	ACCTTTTAGGCACCTCCGAT	ACCGATCTTTAGTCCCGGTT	179	P	A	A	NA
PMS1089	Os_Chr02	16854405	16854420	(GA)8	(GA)8	(GA)8	(GA)8	(GA)8	(GA)8	(GA)7	(GA)8	(GA)8	(GA)8	(GA)8	-	Introns/Intergenic	-	-	AAATGGGAAAAGGGAGAGGA	ATCTCCCGCAACTGCTACC	207	A	A	A	NA
PMS1090	Os_Chr02	16923247	16923270	(GA)12	(GA)11	(GA)12	(GA)12	(GA)12	(GA)11	(GA)12	(GA)12	(GA)12	(GA)12	(GA)12	-	Introns/Intergenic	-	-	CCAGGGATCAGAGAAGAAGAAGAGG	CCCTGAACCGCAATATCATACACC	347	P	A	A	RM13218
PMS1091	Os_Chr02	16923723	16923748	(AT)13	(AT)13	(AT)13	(AT)13	(AT)6	(AT)13	(AT)13	(AT)13	(AT)13	(AT)13	(AT)13	-	Introns/Intergenic	-	-	GCGCCGTTGATAAGAAAATC	TTGGACACGATGCAAGTTTT	255	P	A	A	NA

Polymorphic SSR (PMS) markers	Chromosomes	Start physical positions (bp)	End physical positions (bp)	Nipponbare	Kasalath	Nagina22	IR64	Pokkali	Bala	Tainung67	Azucena	Moroberekan	*Oryza rufipogon*	*Oryza nivara*	MSU gene locus IDs	Structural annotation	Functional annotation	SSR functional effects	Forward primers (5'-3')	Reverse primers (5'-3')	Amplified product size (bp)	Markers exhibiting polymorphism within *indica* rice	Transcrption factor genes-derived markers	Known cloned genes-derived markers	PMS markers corresponding with RM markers
PMS1092	Os_Chr02	16957820	16957895	(AT)38	(AT)38	(AT)38	(AT)38	(AT)38	(AT)6	(AT)38	(AT)38	(AT)38	(AT)38	(AT)38	-	Introns/Intergenic	-	-	GGTCTAGCCTACAGATTGATAGC	CACATCTTAGCCTTCTCTCTCC	330	P	A	A	RM13222
PMS1093	Os_Chr02	17243751	17243764	(TA)7	(TA)7	(TA)6	(TA)6	(TA)7	(TA)7	(TA)7	(TA)7	(TA)7	(TA)7	(TA)7	-	Introns/Intergenic	-	-	CGCGTAAGAAAAATATTGGCA	TTACGATTTTAGGGCGATGG	213	P	A	A	NA
PMS1094	Os_Chr02	17365378	17365395	(AT)9	(AT)9	(AT)9	(AT)8	(AT)9	(AT)9	(AT)9	(AT)9	(AT)9	(AT)9	(AT)9	-	Introns/Intergenic	-	-	CGCATGCTTCCCAAACTACT	TTATTTGGCTCTGTTCGGCT	207	P	A	A	NA
PMS1095	Os_Chr02	17543871	17543916	(TA)23	(TA)23	(TA)23	(TA)8	(TA)23	(TA)23	(TA)23	(TA)23	(TA)8	(TA)23	(TA)23	-	Introns/Intergenic	-	-	GCCCGATTTTCTTGTTCGTA	CCATGGAGCACCAAACAAAT	269	P	A	A	NA
PMS1096	Os_Chr02	17668060	17668073	(GA)7	(GA)7	(GA)7	(GA)7	(GA)6	(GA)7	(GA)7	(GA)7	(GA)7	(GA)7	(GA)7	-	Introns/Intergenic	-	-	GAGTTCATGCGAGGAAAAGG	CCCTCGAGAAATCTCACACC	235	P	A	A	NA
PMS1097	Os_Chr02	17733245	17733260	(GA)8	(GA)8	(GA)8	(GA)6	(GA)8	(GA)8	(GA)8	(GA)8	(GA)8	(GA)8	(GA)8	-	Introns/Intergenic	-	-	GAGGAGGAGAGGGAGAGGAA	CCTTGGTCTAAAAGGAGCCC	214	P	A	A	NA
PMS1098	Os_Chr02	17778003	17778018	(AG)8	(AG)8	(AG)8	(AG)8	(AG)8	(AG)8	(AG)8	(AG)8	(AG)8	(AG)8	(AG)6	-	Introns/Intergenic	-	-	CTCAGTGCTTTTTGCGAGTG	CCCCAAAAGAGTATGACCGA	179	A	A	A	NA
PMS1099	Os_Chr02	17846300	17846326	(CCG)9	(CCG)9	(CCG)5	(CCG)9	(CCG)5	(CCG)9	(CCG)9	(CCG)9	(CCG)9	(CCG)9	(CCG)9	LOC_Os02g30050	URR	ribosomal protein L29, putative, expressed	-	ATGAAAGCCCATCTCAACTCAGC	GAAGAACGGAATGTTGTAGCTTACCC	191	P	A	A	RM6611
PMS1100	Os_Chr02	17873808	17873822	(GGC)5	(GGC)5	(GGC)5	(GGC)5	(GGC)5	(GGC)5	(GGC)5	(GGC)5	(GGC)5	(GGC)6	(GGC)5	LOC_Os02g30100	CDS	cytochrome P450, putative, expressed	CODON_INSERTION	CGCCATTGCAGCAGTATAAA	TCCGGAACAGGTGCAGGT	276	A	A	A	NA
PMS1101	Os_Chr02	17875413	17875432	(TA)10	(TA)10	(TA)10	(TA)10	(TA)10	(TA)10	(TA)10	(TA)10	(TA)9	(TA)7	(TA)10	-	Introns/Intergenic	-	-	CAGAAGCCTTCATCCCAGATAGG	CAAACGGGATCACCATCTTCC	469	A	A	A	RM13254
PMS1102	Os_Chr02	17944155	17944172	(GA)9	(GA)8	(GA)9	(GA)9	(GA)8	(GA)8	(GA)9	(GA)9	(GA)9	(GA)9	(GA)8	-	Introns/Intergenic	-	-	TCAAACAATTGAGAGAGGGAGA	TTTGATCCCTTCCAGCTTTC	236	P	A	A	NA
PMS1103	Os_Chr02	18039199	18039219	(GAG)7	(GAG)7	(GAG)7	(GAG)6	(GAG)7	(GAG)7	(GAG)7	(GAG)7	(GAG)7	(GAG)7	(GAG)7	LOC_Os02g30310	URR	ThiF family domain containing protein, putative, expressed	-	CTCAACTCGATCAGCCTCTCTCG	AGATCCACACAGGTCAGAAGAAGC	266	P	A	A	RM13258
PMS1104	Os_Chr02	18068018	18068035	(CT)9	(CT)9	(CT)9	(CT)9	(CT)9	(CT)6	(CT)9	(CT)9	(CT)9	(CT)9	(CT)9	-	Introns/Intergenic	-	-	CTCCAACAGAATGGCCAAAT	AGGGAGGGAGCAGCAGAT	234	P	A	A	NA

Polymorphic SSR (PMS) markers	Chromosomes	Start physical positions (bp)	End physical positions (bp)	Nipponbare	Kasalath	Nagina22	IR64	Pokkali	Bala	Tainung67	Azucena	Moroberekan	*Oryza rufipogon*	*Oryza nivara*	MSU gene locus IDs	Structural annotation	Functional annotation	SSR functional effects	Forward primers (5'-3')	Reverse primers (5'-3')	Amplified product size (bp)	Markers exhibiting polymorphism within *indica* rice	Transcrption factor genes-derived markers	Known cloned genes-derived markers	PMS markers corresponding with RM markers
PMS1105	Os_Chr02	18183330	18183399	(TA)35	(TA)35	(TA)7	(TA)13	(TA)13	(TA)10	(TA)35	(TA)35	(TA)35	(TA)35	(TA)35	-	Introns/Intergenic	-	-	TAAGGAGCAGTTACAGACTTGC	GCATGTCTTCGAATTAGAGC	364	P	A	A	RM13271
PMS1106	Os_Chr02	18277244	18277329	(AT)43	(AT)43	(AT)43	(AT)43	(AT)43	(AT)6	(AT)43	(AT)6	(AT)6	(AT)43	(AT)6	-	Introns/Intergenic	-	-	GCAATCTGGGAGCGTTTGTAGG	TGTGGATCGGGAGGTAAACACG	619	P	A	A	RM13272
PMS1107	Os_Chr02	18378994	18379005	(CA)6	(CA)6	(CA)6	(CA)6	(CA)6	(CA)6	(CA)6	(CA)7	(CA)7	(CA)6	(CA)6	-	Introns/Intergenic	-	-	CCGCGGTAGTTCAGAGGATA	AATTGGTGCCATTTTCACAT	208	A	A	A	NA
PMS1108	Os_Chr02	18385235	18385252	(CCG)6	(CCG)6	(CCG)6	(CCG)6	(CCG)6	(CCG)6	(CCG)5	(CCG)6	(CCG)6	(CCG)6	(CCG)6	LOC_Os02g30840	URR	RNA pseudouridine synthase, putative, expressed	-	CTAAAATCCTCCACTGCCGA	AAGATGTAGTCCTGCCGCTC	266	A	A	A	NA
PMS1109	Os_Chr02	18454300	18454325	(CT)13	(CT)13	(CT)13	(CT)13	(CT)13	(CT)7	(CT)13	(CT)13	(CT)13	(CT)13	(CT)13	-	Introns/Intergenic	-	-	GATCGACCTCCACACACTTGTCC	CTCAGCTTACCGTGCGACACC	168	P	A	A	RM3284
PMS1110	Os_Chr02	18521266	18521277	(GA)6	(GA)6	(GA)6	(GA)6	(GA)6	(GA)6	(GA)6	(GA)6	(GA)7	(GA)6	(GA)6	-	Introns/Intergenic	-	-	CGCTGTGTACATGAGACGGA	TTCTTCACCCCCAACTATGC	229	A	A	A	NA
PMS1111	Os_Chr02	18535708	18535759	(TA)26	(TA)26	(TA)26	(TA)7	(TA)26	(TA)26	(TA)26	(TA)26	(TA)26	(TA)26	(TA)26	-	Introns/Intergenic	-	-	ATCACTCCTGCTACCACATGG	CAGATTCATCACGAGAGCACA	252	P	A	A	RM4499
PMS1112	Os_Chr02	18621323	18621342	(AG)10	(AG)9	(AG)10	(AG)10	(AG)9	(AG)10	(AG)10	(AG)10	(AG)10	(AG)10	(AG)10	-	Introns/Intergenic	-	-	ACATGGACAACCCTCCAAAG	GGAAGCAACTCCAGCTCAAC	123	P	A	A	NA
PMS1113	Os_Chr02	18639733	18639759	(GCT)9	(GCT)9	(GCT)9	(GCT)9	(GCT)9	(GCT)9	(GCT)9	(GCT)6	(GCT)9	(GCT)9	(GCT)9	LOC_Os02g31140	CDS	CFL1	CODON_DELETION	CACCATGAAGTACATGAAGCAAGC	CAGCTATGGCTACGGCTACGG	253	A	A	P	RM13290
PMS1114	Os_Chr02	18664178	18664197	(AG)10	(AG)10	(AG)10	(AG)10	(AG)10	(AG)10	(AG)10	(AG)7	(AG)9	(AG)10	(AG)10	-	Introns/Intergenic	-	-	GATTTGGGCATGATCCTAAGAGG	GTGCATGGAACAATGGAAGAGC	92	A	A	A	RM13297
PMS1115	Os_Chr02	18909799	18909819	(TCT)7	(TCT)7	(TCT)7	(TCT)5	(TCT)5	(TCT)5	(TCT)7	(TCT)7	(TCT)7	(TCT)7	(TCT)5	-	Introns/Intergenic	-	-	CCACATTTTCTGAAGCACCA	AAACAACCCCAAAATCTCCC	230	P	A	A	NA
PMS1116	Os_Chr02	18924263	18924283	(GCT)7	(GCT)7	(GCT)7	(GCT)6	(GCT)6	(GCT)6	(GCT)5	(GCT)7	(GCT)7	(GCT)6	(GCT)6	LOC_Os02g32040	CDS	AP2 domain containing protein, expressed	CODON_DELETION	CGAGGTATCTCGCCTCGTTCC	CCAACGACCACAACTACTTCTTCTCC	167	P	A	A	RM13312
PMS1117	Os_Chr02	19009045	19009059	(AGG)5	(AGG)5	(AGG)5	(AGG)5	(AGG)5	(AGG)5	(AGG)5	(AGG)5	(AGG)5	(AGG)6	(AGG)5	LOC_Os02g32180	CDS	expressed protein	CODON_CHANGE_PLUS_CODON_INSERTION	TATTCTGGGCATCTTGGGAG	TCCCCTTACCATGCTGATGT	259	A	A	A	NA

Polymorphic SSR (PMS) markers	Chromosomes	Start physical positions (bp)	End physical positions (bp)	Nipponbare	Kasalath	Nagina22	IR64	Pokkali	Bala	Tainung67	Azucena	Moroberekan	*Oryza rufipogon*	*Oryza nivara*	MSU gene locus IDs	Structural annotation	Functional annotation	SSR functional effects	Forward primers (5'-3')	Reverse primers (5'-3')	Amplified product size (bp)	Markers exhibiting polymorphism within *indica* rice	Transcrption factor genes-derived markers	Known cloned genes-derived markers	PMS markers corresponding with RM markers
PMS1118	Os_Chr02	19065101	19065156	(TA)28	(TA)28	(TA)10	(TA)28	(TA)15	(TA)28	(TA)6	(TA)28	(TA)10	(TA)28	(TA)28	-	Introns/Intergenic	-	-	TTGATCGTCGCGTTACTACG	TCAATTTCAACAATGGTGCC	237	P	A	A	NA
PMS1119	Os_Chr02	19085887	19085970	(TA)42	(TA)42	(TA)42	(TA)42	(TA)42	(TA)7	(TA)42	(TA)42	(TA)42	(TA)8	(TA)41	-	Introns/Intergenic	-	-	ATCTTTCCTTACGGCTTTCACG	TTAGTTATAGCACACCCTGGATGC	278	P	A	A	RM13321
PMS1120	Os_Chr02	19088779	19088800	(TA)11	(TA)11	(TA)11	(TA)11	(TA)11	(TA)10	(TA)11	(TA)9	(TA)11	(TA)11	(TA)11	-	Introns/Intergenic	-	-	TCAATTTCAACAATGGTGCC	AGTAATCCGCATCCCACCTA	253	P	A	A	NA
PMS1121	Os_Chr02	19138071	19138108	(TA)19	(TA)19	(TA)19	(TA)19	(TA)19	(TA)19	(TA)19	(TA)19	(TA)12	(TA)19	(TA)19	-	Introns/Intergenic	-	-	GGCCCGACACATAAACAATAGGG	TATTTGCCATCCCTCCTCTTTGG	210	A	A	A	RM13327
PMS1122	Os_Chr02	19210060	19210115	(AT)28	(AT)28	(AT)9	(AT)28	(AT)28	(AT)16	(AT)28	(AT)28	(AT)28	(AT)28	(AT)16	-	Introns/Intergenic	-	-	GCCAAAAGTTTACATACCCGA	TACCATGGAGCACCAAACAA	133	P	A	A	NA
PMS1123	Os_Chr02	19230924	19230941	(AG)9	(AG)9	(AG)9	(AG)9	(AG)9	(AG)11	(AG)9	(AG)9	(AG)9	(AG)9	(AG)9	-	Introns/Intergenic	-	-	AAAAGCAAAGCAAAACCGAA	AAATTTTTAGGCACCTGCGA	200	P	A	A	NA
PMS1124	Os_Chr02	19271922	19271939	(CGT)6	(CGT)6	(CGT)6	(CGT)6	(CGT)6	(CGT)6	(CGT)6	(CGT)6	(CGT)6	(CGT)5	(CGT)6	LOC_Os02g32550	CDS	expressed protein	CODON_DELETION	GGAGAGCCAGTAGGAGGGAG	GACTTCCTGGAGCGCATCT	231	A	A	A	NA
PMS1125	Os_Chr02	19314006	19314029	(CCCT)6	(CCCT)6	(CCCT)6	(CCCT)6	(CCCT)6	(CCCT)6	(CCCT)6	(CCCT)5	(CCCT)6	(CCCT)6	(CCCT)6	-	Introns/Intergenic	-	-	TGCGAGACCGACGGTATATT	TAGGAAAGTAATGCGGTGCC	244	A	A	A	NA
PMS1126	Os_Chr02	19485267	19485287	(CCG)7	(CCG)7	(CCG)7	(CCG)7	(CCG)7	(CCG)7	(CCG)7	(CCG)7	(CCG)7	(CCG)7	(CCG)5	-	Introns/Intergenic	-	-	GCCATGGATAGGGTGAGGT	GCAGACAAGGGGTAGGAGG	150	A	A	A	NA
PMS1127	Os_Chr02	19587302	19587321	(CA)10	(CA)10	(CA)10	(CA)11	(CA)11	(CA)10	(CA)10	(CA)10	(CA)10	(CA)10	(CA)10	-	Introns/Intergenic	-	-	TCTAGGACTTGAAGGGATGTTCC	GCTTCTAGACTCTGGAGTCACTGG	288	P	A	A	RM13355
PMS1128	Os_Chr02	19651246	19651257	(CT)6	(CT)6	(CT)6	(CT)6	(CT)6	(CT)6	(CT)6	(CT)6	(CT)6	(CT)7	(CT)6	-	Introns/Intergenic	-	-	CCAAAATTGTTTGGCCTGAT	TCTCCTCAGCAAAATCTCTGG	111	A	A	A	NA
PMS1129	Os_Chr02	19699298	19699309	(TA)6	(TA)6	(TA)6	(TA)8	(TA)6	(TA)6	(TA)6	(TA)6	(TA)6	(TA)6	(TA)6	-	Introns/Intergenic	-	-	AGCAGCAATCCAATCCACAT	CGTAAATATTCCACGCCGTT	266	P	A	A	NA
PMS1130	Os_Chr02	19715827	19715846	(AG)10	(AG)10	(AG)10	(AG)10	(AG)10	(AG)10	(AG)10	(AG)10	(AG)10	(AG)10	(AG)9	-	Introns/Intergenic	-	-	TAGCATTCCACTTGCAGAACAGC	AATTGCCTTACCATCCGTCTGC	282	A	A	A	RM13356

Polymorphic SSR (PMS) markers	Chromosomes	Start physical positions (bp)	End physical positions (bp)	Nipponbare	Kasalath	Nagina22	IR64	Pokkali	Bala	Tainung67	Azucena	Moroberekan	*Oryza rufipogon*	*Oryza nivara*	MSU gene locus IDs	Structural annotation	Functional annotation	SSR functional effects	Forward primers (5'-3')	Reverse primers (5'-3')	Amplified product size (bp)	Markers exhibiting polymorphism within *indica* rice	Transcrption factor genes-derived markers	Known cloned genes-derived markers	PMS markers corresponding with RM markers
PMS1131	Os_Chr02	19723791	19723808	(CA)9	(CA)9	(CA)9	(CA)9	(CA)8	(CA)9	(CA)9	(CA)9	(CA)9	(CA)9	(CA)9	-	Introns/Intergenic	-	-	AAGGACCTCCTTTTCGGGTA	GCGCGGCTATACGTGTCTAC	198	P	A	A	NA
PMS1132	Os_Chr02	19828833	19828847	(AGG)5	(AGG)5	(AGG)5	(AGG)6	(AGG)5	(AGG)5	(AGG)5	(AGG)5	(AGG)5	(AGG)5	(AGG)5	LOC_Os02g33370	CDS	retrotransposon protein, putative, Ty3-gypsy subclass, expressed	CODON_INSERTION	GGAGCAGCTGGTTGTTCTTC	GCTCCGCCAGTAAGACATTC	181	P	A	A	NA
PMS1133	Os_Chr02	19898360	19898377	(GAC)6	(GAC)6	(GAC)6	(GAC)6	(GAC)6	(GAC)6	(GAC)6	(GAC)6	(GAC)6	(GAC)6	(GAC)5	LOC_Os02g33460	CDS	transposon protein, putative, Mutator sub-class, expressed	CODON_DELETION	CTCGTCCAAGAGCTTCGTGT	GACGCGCTCCATCAGAAT	235	A	A	A	NA
PMS1134	Os_Chr02	20068476	20068493	(CTT)6	(CTT)5	(CTT)6	(CTT)6	(CTT)6	(CTT)5	(CTT)6	(CTT)6	(CTT)6	(CTT)6	(CTT)5	-	Introns/Intergenic	-	-	ATCAACATTTTCTCACCGGG	TTCACACGGCTTTTGTCTTG	172	P	A	A	NA
PMS1135	Os_Chr02	20152867	20152887	(CGG)7	(CGG)7	(CGG)7	(CGG)7	(CGG)7	(CGG)7	(CGG)7	(CGG)7	(CGG)7	(CGG)7	(CGG)5	LOC_Os02g33780	CDS	serine-aspartate repeat containing protein I precursor, putative, expressed	CODON_CHANGE_PLUS_CODON_DELETION	CTGAGGAGAAGGAGAAGAACG	AGCTCAAGCTTGCTACTGACG	179	A	A	A	RM13375
PMS1136	Os_Chr02	20182006	20182026	(CCG)7	(CCG)7	(CCG)7	(CCG)7	(CCG)7	(CCG)7	(CCG)7	(CCG)5	(CCG)7	(CCG)7	(CCG)7	LOC_Os02g33850	CDS	elongation factor Tu family protein, putative, expressed	CODON_DELETION	AGGCCGACTTCATGGTGAT	TATATCCGTACGCCGGAGAG	278	A	A	A	NA
PMS1137	Os_Chr02	20190935	20190952	(GAC)6	(GAC)6	(GAC)6	(GAC)6	(GAC)6	(GAC)6	(GAC)6	(GAC)6	(GAC)6	(GAC)6	(GAC)5	-	Introns/Intergenic	-	-	GACGAACGCCACGACAAC	CACCTCTCCCTCCTCTCCTC	166	A	A	A	NA
PMS1138	Os_Chr02	20203972	20203993	(CT)11	(CT)11	(CT)11	(CT)11	(CT)11	(CT)11	(CT)11	(CT)11	(CT)11	(CT)8	(CT)10	-	Introns/Intergenic	-	-	TTCCAAATCAAATTCCTCCG	AGGAGGAGGAGCACTCGG	278	A	A	A	NA
PMS1139	Os_Chr02	20265661	20265714	(AT)27	(AT)27	(AT)27	(AT)27	(AT)27	(AT)27	(AT)27	(AT)27	(AT)6	(AT)27	(AT)27	-	Introns/Intergenic	-	-	ATCTTTCTACTTGCCTCGATCC	AACAGGGATGCATTAGTGTGC	351	A	A	A	RM13383
PMS1140	Os_Chr02	20272537	20272566	(AT)15	(AT)15	(AT)15	(AT)15	(AT)15	(AT)10	(AT)15	(AT)15	(AT)15	(AT)15	(AT)6	-	Introns/Intergenic	-	-	GCTCGATATTTAGTCCATGTGTGC	TCTGGACGGATTCTGAATTTGC	244	P	A	A	RM13384
PMS1141	Os_Chr02	20292398	20292415	(GGC)6	(GGC)6	(GGC)6	(GGC)6	(GGC)6	(GGC)5	(GGC)6	(GGC)6	(GGC)6	(GGC)6	(GGC)6	LOC_Os02g33990	CDS	transposon protein, putative, CACTA, En/Spm sub-class, expressed	CODON_DELETION	AGAGATCGAGATGCATGAAG	GTATCTCGCCGTCGTCTC	120	P	A	A	NA
PMS1142	Os_Chr02	20360825	20360836	(CT)6	(CT)6	(CT)6	(CT)6	(CT)6	(CT)6	(CT)6	(CT)6	(CT)6	(CT)7	(CT)6	-	Introns/Intergenic	-	-	GTAGTCACCTGGTTGGCGAT	ACGTCAGTGTTGCCTCACAC	175	A	A	A	NA
PMS1143	Os_Chr02	20405051	20405092	(AT)21	(AT)21	(AT)9	(AT)21	(AT)21	(AT)21	(AT)21	(AT)21	(AT)21	(AT)21	(AT)21	-	Introns/Intergenic	-	-	GGAAGGAAAGCACGGAGATA	ACGGTGGGATTAGACTGTGC	233	P	A	A	NA

Polymorphic SSR (PMS) markers	Chromosomes	Start physical positions (bp)	End physical positions (bp)	Nipponbare	Kasalath	Nagina22	IR64	Pokkali	Bala	Tainung67	Azucena	Moroberekan	*Oryza rufipogon*	*Oryza nivara*	MSU gene locus IDs	Structural annotation	Functional annotation	SSR functional effects	Forward primers (5'-3')	Reverse primers (5'-3')	Amplified product size (bp)	Markers exhibiting polymorphism within *indica* rice	Transcrption factor genes-derived markers	Known cloned genes-derived markers	PMS markers corresponding with RM markers
PMS1144	Os_Chr02	20476253	20476266	(CG)7	(CG)6	(CG)7	(CG)7	(CG)7	(CG)7	(CG)6	(CG)7	(CG)7	(CG)7	(CG)7	-	Introns/Intergenic	-	-	CTTCTCCCTCCCTCTCTGCT	CAGATGGAGGAGGAGCTCTG	268	P	A	A	NA
PMS1145	Os_Chr02	20566810	20566865	(TA)28	(TA)28	(TA)28	(TA)28	(TA)28	(TA)27	(TA)28	(TA)18	(TA)11	(TA)28	(TA)28	-	Introns/Intergenic	-	-	ATGCACAAGAAAGCAAAGCC	TTCCATGAAAGACTCAATGAAAG	263	P	A	A	NA
PMS1146	Os_Chr02	20585151	20585174	(TC)12	(TC)12	(TC)12	(TC)12	(TC)11	(TC)12	(TC)12	(TC)12	(TC)12	(TC)12	(TC)12	-	Introns/Intergenic	-	-	CACGAATCTTGAGGCACATTGG	AATTTGCTTAGCTTGGCCTCTCG	220	P	A	A	RM13400
PMS1147	Os_Chr02	20620936	20620957	(AT)11	(AT)11	(AT)11	(AT)11	(AT)11	(AT)11	(AT)11	(AT)11	(AT)11	(AT)11	(AT)9	-	Introns/Intergenic	-	-	GGGCATGCTCTACCACTCTTACC	CTCACCAAAGATTCGGTATGTGC	245	A	A	A	RM13402
PMS1148	Os_Chr02	20677275	20677292	(AGC)6	(AGC)6	(AGC)6	(AGC)6	(AGC)6	(AGC)6	(AGC)5	(AGC)6	(AGC)6	(AGC)6	(AGC)6	LOC_Os02g34500	CDS	expressed protein	CODON_DELETION	TCCTTCTCCTCCTCCTCCTC	CCCTCTCCTGTAATCCCCAT	241	A	A	A	NA
PMS1149	Os_Chr02	20754849	20754878	(AT)15	(AT)14	(AT)15	(AT)7	(AT)9	(AT)15	(AT)15	(AT)15	(AT)15	(AT)7	(AT)10	-	Introns/Intergenic	-	-	TTTCCCATCTCTTCTCTCTTCACC	GTGAGTGAAATTCGACCAGTGC	356	P	A	A	RM13407
PMS1150	Os_Chr02	20787761	20787781	(GGA)7	(GGA)5	(GGA)7	(GGA)7	(GGA)7	(GGA)7	(GGA)5	(GGA)7	(GGA)7	(GGA)7	(GGA)7	-	Introns/Intergenic	-	-	TTTCTCCGGTGTGATTTGATCG	GAGGAGCATACGAGGATGAGAGG	312	P	A	A	RM13410
PMS1151	Os_Chr02	20800897	20800914	(CT)9	(CT)11	(CT)9	(CT)9	(CT)9	(CT)9	(CT)9	(CT)9	(CT)9	(CT)9	(CT)9	-	Introns/Intergenic	-	-	CAGTACTTCCCTCTGCTCCG	ATCTATGGCTGCTCCCACTC	253	P	A	A	NA
PMS1152	Os_Chr02	20932670	20932684	(GGA)5	(GGA)7	(GGA)5	(GGA)5	(GGA)5	(GGA)5	(GGA)5	(GGA)5	(GGA)5	(GGA)5	(GGA)5	LOC_Os02g34884	URR	SacI homology domain containing protein, expressed	-	GGCGTCCGATCCATATCTAA	CTAGTCGTCTCGTCGTGCTG	269	P	A	A	NA
PMS1153	Os_Chr02	20955734	20955751	(CGA)6	(CGA)5	(CGA)6	(CGA)6	(CGA)6	(CGA)6	(CGA)6	(CGA)6	(CGA)6	(CGA)6	(CGA)6	LOC_Os02g34930	CDS	expressed protein	CODON_CHANGE_PLUS_CODON_DELETION	CTGCTGTGGGAGGACTTCA	GTACAGCCCTCCTCCTCCTC	177	P	A	A	NA
PMS1154	Os_Chr02	20960938	20960951	(AT)7	(AT)7	(AT)7	(AT)6	(AT)7	(AT)7	(AT)7	(AT)7	(AT)7	(AT)7	(AT)6	-	Introns/Intergenic	-	-	TCGTTTGGCTCTTTCCCTAA	GCTCTCATTGGATGGCCTAA	219	P	A	A	NA
PMS1155	Os_Chr02	20977571	20977594	(CGG)8	(CGG)5	(CGG)8	(CGG)6	(CGG)8	(CGG)8	(CGG)8	(CGG)8	(CGG)8	(CGG)8	(CGG)8	-	Introns/Intergenic	-	-	TGAGTGTTTGCGGGAGGAGAGC	CAGGGTGAGAGGTTCCAGTCAGG	275	P	A	A	RM6023
PMS1156	Os_Chr02	20980984	20981011	(AT)14	(AT)14	(AT)14	(AT)14	(AT)14	(AT)10	(AT)14	(AT)14	(AT)14	(AT)14	(AT)14	-	Introns/Intergenic	-	-	AGGTACTTCCTCCGTTTCACA	TGGTACTACCTCCGTTTCAGG	188	P	A	A	NA

Polymorphic SSR (PMS) markers	Chromosomes	Start physical positions (bp)	End physical positions (bp)	Nipponbare	Kasalath	Nagina22	IR64	Pokkali	Bala	Tainung67	Azucena	Moroberekan	*Oryza rufipogon*	*Oryza nivara*	MSU gene locus IDs	Structural annotation	Functional annotation	SSR functional effects	Forward primers (5'-3')	Reverse primers (5'-3')	Amplified product size (bp)	Markers exhibiting polymorphism within *indica* rice	Transcrption factor genes-derived markers	Known cloned genes-derived markers	PMS markers corresponding with RM markers
PMS1157	Os_Chr02	20986154	20986210	(TCT)19	(TCT)19	(TCT)19	(TCT)19	(TCT)19	(TCT)18	(TCT)19	(TCT)10	(TCT)19	(TCT)19	(TCT)19	-	Introns/Intergenic	-	-	CTTCTTCCTCCCATGGTCTCTCC	CGCGTCGTCTTACATCTCACG	149	P	A	A	RM13418
PMS1158	Os_Chr02	21054406	21054419	(CT)7	(CT)7	(CT)7	(CT)7	(CT)7	(CT)7	(CT)7	(CT)7	(CT)7	(CT)6	(CT)7	LOC_Os02g35080	URR	T-complex protein 11, putative, expressed	-	CAAAATCACCGAAACACCCT	GAGATGAATGTTTCGAGGCG	112	A	A	A	NA
PMS1159	Os_Chr02	21131577	21131590	(AT)7	(AT)7	(AT)7	(AT)6	(AT)7	(AT)7	(AT)7	(AT)7	(AT)7	(AT)7	(AT)7	-	Introns/Intergenic	-	-	TGGGAATCCTCCTCTCCTCT	GCACGTAGCATAGTGGTTGC	246	P	A	A	NA
PMS1160	Os_Chr02	21192471	21192496	(AT)13	(AT)13	(AT)13	(AT)13	(AT)13	(AT)13	(AT)13	(AT)13	(AT)13	(AT)13	(AT)10	-	Introns/Intergenic	-	-	CCACCCAAGCAACCAAATAAGG	AGGGCAGGGATTAGAGGTAATGG	156	A	A	A	RM13432
PMS1161	Os_Chr02	21349371	21349406	(TA)18	(TA)17	(TA)18	(TA)18	(TA)10	(TA)18	(TA)18	(TA)8	(TA)18	(TA)11	(TA)18	-	Introns/Intergenic	-	-	TTCCTTCCATCCAAAAGTGC	AGGGCACACGATGAAAGATT	256	P	A	A	NA
PMS1162	Os_Chr02	21372442	21372463	(TC)11	(TC)11	(TC)11	(TC)11	(TC)11	(TC)11	(TC)11	(TC)11	(TC)11	(TC)10	(TC)11	-	Introns/Intergenic	-	-	GATGCACATGCAGTCAAACATCC	CTTCTTGTGTCGTTGCTACCTTCC	83	A	A	A	RM13441
PMS1163	Os_Chr02	21380163	21380176	(AG)7	(AG)8	(AG)7	(AG)8	(AG)7	(AG)8	(AG)7	(AG)7	(AG)7	(AG)8	(AG)8	-	Introns/Intergenic	-	-	TGACTACCAGTCCACCACCA	CCTACATGCAGCAAAATGGA	128	P	A	A	NA
PMS1164	Os_Chr02	21435182	21435199	(GCG)6	(GCG)6	(GCG)6	(GCG)6	(GCG)6	(GCG)6	(GCG)5	(GCG)6	(GCG)6	(GCG)6	(GCG)6	LOC_Os02g35650	URR	late embryogenesis abundant protein, putative, expressed	-	GTCCTTCTCGTACTGGGCTG	GCGAGAGGTGTCAGGAGAAG	275	A	A	A	NA
PMS1165	Os_Chr02	21438756	21438770	(GCT)5	(GCT)5	(GCT)6	(GCT)5	(GCT)5	(GCT)5	(GCT)5	(GCT)5	(GCT)5	(GCT)5	(GCT)5	LOC_Os02g35660	CDS	PTF1, putative, expressed	CODON_INSERTION	TTACCACTGGCGATGAATGA	CTTTGGCAGCCTCTTCAATG	188	P	P	A	NA
PMS1166	Os_Chr02	21443833	21443844	(TA)6	(TA)6	(TA)6	(TA)6	(TA)7	(TA)7	(TA)6	(TA)6	(TA)6	(TA)6	(TA)6	-	Introns/Intergenic	-	-	CCAATTAGGCACACAGAATGAAGG	CTCTCTCCTCCCGGTCTTCTCC	217	P	A	A	RM13447
PMS1167	Os_Chr02	21458410	21458431	(GA)11	(GA)11	(GA)8	(GA)11	(GA)11	(GA)11	(GA)11	(GA)11	(GA)11	(GA)11	(GA)11	-	Introns/Intergenic	-	-	AGGTCACTGACGCCCTTAAATCC	CTCCCAAATGAGGTTAGCTTTGC	106	P	A	A	RM13449
PMS1168	Os_Chr02	21461448	21461481	(AG)17	(AG)17	(AG)17	(AG)7	(AG)9	(AG)16	(AG)17	(AG)17	(AG)17	(AG)17	(AG)8	-	Introns/Intergenic	-	-	CTAGCTGACGATATCCGAACAAGAGC	TTTGGAAGAGGCAGATTCATGG	407	P	A	A	RM13450
PMS1169	Os_Chr02	21536807	21536834	(AG)14	(AG)14	(AG)14	(AG)14	(AG)14	(AG)14	(AG)14	(AG)14	(AG)14	(AG)14	(AG)10	-	Introns/Intergenic	-	-	CCCTTCTCTCCTCTATCGCC	GCAAAAGGGGTCTCAAAAGA	267	A	A	A	NA

Polymorphic SSR (PMS) markers	Chromosomes	Start physical positions (bp)	End physical positions (bp)	Nipponbare	Kasalath	Nagina22	IR64	Pokkali	Bala	Tainung67	Azucena	Moroberekan	*Oryza rufipogon*	*Oryza nivara*	MSU gene locus IDs	Structural annotation	Functional annotation	SSR functional effects	Forward primers (5'-3')	Reverse primers (5'-3')	Amplified product size (bp)	Markers exhibiting polymorphism within *indica* rice	Transcrption factor genes-derived markers	Known cloned genes-derived markers	PMS markers corresponding with RM markers
PMS1170	Os_Chr02	21574069	21574089	(GGT)7	(GGT)5	(GGT)7	(GGT)7	(GGT)7	(GGT)7	(GGT)7	(GGT)7	(GGT)7	(GGT)7	(GGT)7	LOC_Os02g35910	URR	protein phosphotase protein, putative, expressed	-	TTCTTGGCCTGGCTGAAGAGC	ACCACCACCATTCTCCCTCTCC	286	P	A	A	RM13455
PMS1171	Os_Chr02	21596630	21596647	(GGC)6	(GGC)6	(GGC)5	(GGC)6	(GGC)6	(GGC)6	(GGC)6	(GGC)6	(GGC)6	(GGC)6	(GGC)6	LOC_Os02g35950	URR	RNA recognition motif containing protein, putative, expressed	-	AGTTCGTCAAGCACTTCGC	CCAAAGCAGTTGTGCAGGTA	190	P	A	A	NA
PMS1172	Os_Chr02	21832911	21832926	(CT)8	(CT)8	(CT)8	(CT)8	(CT)8	(CT)8	(CT)8	(CT)8	(CT)8	(CT)7	(CT)8	-	Introns/Intergenic	-	-	TACCTGGAAAAGGACGGAGA	GGTGTCCTGGGTCTAACAGTG	206	A	A	A	NA
PMS1173	Os_Chr02	21847589	21847648	(TA)30	(TA)30	(TA)7	(TA)30	(TA)14	(TA)30	(TA)30	(TA)8	(TA)30	(TA)30	(TA)8	LOC_Os02g36210	DRR	OsCPS2|OsCyc2	-	ATGGCTCGTAAAGTGATGACTGC	GAGCGACACGGTAGTATGTTTGC	368	P	A	P	RM13467
PMS1174	Os_Chr02	21881980	21882031	(TA)26	(TA)26	(TA)26	(TA)6	(TA)7	(TA)26	(TA)26	(TA)26	(TA)26	(TA)26	(TA)26	-	Introns/Intergenic	-	-	TGTAATGGGGCCATCAATTT	TGCTTCCTTTCTTTTCCTTTTT	269	P	A	A	NA
PMS1175	Os_Chr02	21928734	21928754	(GGA)7	(GGA)7	(GGA)7	(GGA)7	(GGA)7	(GGA)7	(GGA)7	(GGA)6	(GGA)7	(GGA)7	(GGA)7	LOC_Os02g36330	CDS	RING-H2 finger protein ATL1O, putative, expressed	CODON_DELETION	ACGGAGAAGGCGAGGAGGATGG	ATGGCGCCTGCTACGACTGTCC	133	A	A	A	RM13477
PMS1176	Os_Chr02	21957048	21957065	(AG)9	(AG)7	(AG)9	(AG)7	(AG)7	(AG)9	(AG)9	(AG)7	(AG)9	(AG)9	(AG)7	LOC_Os02g36360	DRR	ZOS2-11 - C2H2 zinc finger protein, expressed	-	TGCATAACAGGCTAACAGCG	TGCAGGCCTATGGTAGGAAG	153	P	P	A	NA
PMS1177	Os_Chr02	21962250	21962265	(TA)8	(TA)8	(TA)8	(TA)8	(TA)8	(TA)12	(TA)8	(TA)8	(TA)8	(TA)8	(TA)8	-	Introns/Intergenic	-	-	CGCTAATGATGCACGAGTGT	CTTTCTTTCCTGAAAGCCCC	273	P	A	A	NA
PMS1178	Os_Chr02	21962559	21962582	(CGCA)6	(CGCA)6	(CGCA)5	(CGCA)6	(CGCA)6	(CGCA)6	(CGCA)6	(CGCA)6	(CGCA)6	(CGCA)6	(CGCA)6	-	Introns/Intergenic	-	-	GTAATTCTGCGTACGACGGTTGG	GAGTGTCAGCGAGAGGAGTTGG	277	P	A	A	RM13478
PMS1179	Os_Chr02	22009570	22009607	(AT)19	(AT)19	(AT)6	(AT)10	(AT)19	(AT)13	(AT)19	(AT)19	(AT)7	(AT)19	(AT)19	-	Introns/Intergenic	-	-	GGCGATGGCATATTGTAGAACG	ATGGTATCGAGGTTTCCGATGG	428	P	A	A	RM13485
PMS1180	Os_Chr02	22095973	22096000	(TC)14	(TC)14	(TC)14	(TC)14	(TC)14	(TC)14	(TC)14	(TC)14	(TC)14	(TC)14	(TC)11	-	Introns/Intergenic	-	-	CAGCTTGACGGCCATAAATACG	GGAATCTCAAACGGAGGAGACG	115	A	A	A	RM13489
PMS1181	Os_Chr02	22180963	22180986	(GGC)8	(GGC)6	(GGC)8	(GGC)8	(GGC)8	(GGC)8	(GGC)8	(GGC)8	(GGC)8	(GGC)8	(GGC)8	LOC_Os02g36780	CDS	mTERF domain containing protein, expressed	CODON_DELETION	CTCCGAAGAGAGGAAGTAGAGCTTGC	GATCCGACGAGCAGTGGATAACG	227	P	A	A	RM13491
PMS1182	Os_Chr02	22203843	22203866	(CGC)8	(CGC)9	(CGC)8	(CGC)8	(CGC)8	(CGC)8	(CGC)8	(CGC)8	(CGC)8	(CGC)8	(CGC)8	LOC_Os02g36810	CDS	cytokinin-O-glucosyltransferase 2, putative, expressed	CODON_CHANGE_PLUS_CODON_INSERTION	ACGTGAACTACGGGAGCATC	TCGTCGCCTCCATGAACT	180	P	A	A	NA

Polymorphic SSR (PMS) markers	Chromosomes	Start physical positions (bp)	End physical positions (bp)	Nipponbare	Kasalath	Nagina22	IR64	Pokkali	Bala	Tainung67	Azucena	Moroberekan	*Oryza rufipogon*	*Oryza nivara*	MSU gene locus IDs	Structural annotation	Functional annotation	SSR functional effects	Forward primers (5'-3')	Reverse primers (5'-3')	Amplified product size (bp)	Markers exhibiting polymorphism within *indica* rice	Transcrption factor genes-derived markers	Known cloned genes-derived markers	PMS markers corresponding with RM markers
PMS1183	Os_Chr02	22206583	22206604	(GA)11	(GA)11	(GA)11	(GA)11	(GA)11	(GA)11	(GA)11	(GA)6	(GA)11	(GA)9	(GA)11	-	Introns/Intergenic	-	-	GTGATGGCGGCTCGATCTACG	CATCAGCCTCAACATCCACATCG	237	A	A	A	RM13493
PMS1184	Os_Chr02	22248183	22248222	(TA)20	(TA)20	(TA)20	(TA)6	(TA)6	(TA)20	(TA)20	(TA)20	(TA)7	(TA)20	(TA)12	-	Introns/Intergenic	-	-	AGATGGCTTGCACGAACTAGAGG	TAGCGGCGATCAGATTAGATTCC	573	P	A	A	RM13497
PMS1185	Os_Chr02	22258902	22258933	(CT)16	(CT)16	(CT)16	(CT)16	(CT)12	(CT)12	(CT)16	(CT)16	(CT)16	(CT)16	(CT)16	-	Introns/Intergenic	-	-	CCTTAACCACTTCATCACTTCTCTCG	CCAACACCAACCATTAAGCAAAGG	187	P	A	A	RM13500
PMS1186	Os_Chr02	22284810	22284821	(GA)6	(GA)7	(GA)6	(GA)7	(GA)7	(GA)7	(GA)6	(GA)6	(GA)6	(GA)6	(GA)7	-	Introns/Intergenic	-	-	AGCTTGCATTGAGGTCTTGC	GACCGCACAGAAAAATCGTT	226	P	A	A	NA
PMS1187	Os_Chr02	22286519	22286534	(TC)8	(TC)8	(TC)8	(TC)8	(TC)8	(TC)8	(TC)8	(TC)6	(TC)8	(TC)8	(TC)8	-	Introns/Intergenic	-	-	ATGTCTCTAGGGACGTTGCG	TGGTGATTGTTGTGTGCTGA	280	A	A	A	NA
PMS1188	Os_Chr02	22339284	22339301	(ATT)6	(ATT)6	(ATT)6	(ATT)6	(ATT)6	(ATT)6	(ATT)6	(ATT)6	(ATT)6	(ATT)7	(ATT)6	-	Introns/Intergenic	-	-	TTTTCTGATGATCTCGCACG	TTCATTTGCATTGCCACATT	129	A	A	A	NA
PMS1189	Os_Chr02	22367392	22367406	(AAT)5	(AAT)6	(AAT)6	(AAT)5	(AAT)5	(AAT)5	(AAT)5	(AAT)5	(AAT)5	(AAT)6	(AAT)5	-	Introns/Intergenic	-	-	AGGATAATACGGCGTGGAGA	GTATTGGCACCGACCGTATC	234	P	A	A	NA
PMS1190	Os_Chr02	22439525	22439539	(ATT)5	(ATT)5	(ATT)5	(ATT)5	(ATT)5	(ATT)5	(ATT)5	(ATT)5	(ATT)5	(ATT)6	(ATT)5	-	Introns/Intergenic	-	-	AACGGTTAAGCAGCAGCAAT	TGCTTCAGTTTGTCGTCAGG	263	A	A	A	NA
PMS1191	Os_Chr02	22440657	22440690	(AG)17	(AG)16	(AG)17	(AG)17	(AG)17	(AG)17	(AG)17	(AG)17	(AG)17	(AG)17	(AG)17	-	Introns/Intergenic	-	-	AAGGGAGAGCTTAACCAGCAAGC	TGCCGAGTTTATCTGAACTTGAGG	401	P	A	A	RM13508
PMS1192	Os_Chr02	22462497	22462532	(GA)18	(GA)18	(GA)18	(GA)18	(GA)18	(GA)18	(GA)18	(GA)17	(GA)18	(GA)18	(GA)18	-	Introns/Intergenic	-	-	CCACTAGAATCGGAGAAGTTCACG	TCTACCCTCTATCTCTGGCTCTTCG	412	A	A	A	RM3762
PMS1193	Os_Chr02	22477580	22477597	(TTC)6	(TTC)6	(TTC)6	(TTC)5	(TTC)6	(TTC)6	(TTC)6	(TTC)6	(TTC)6	(TTC)6	(TTC)5	-	Introns/Intergenic	-	-	CGAGAACGGCCCAATATAGA	CCTTCAGTCCGAACACCTTT	134	P	A	A	NA
PMS1194	Os_Chr02	22482202	22482217	(TG)8	(TG)8	(TG)8	(TG)8	(TG)8	(TG)8	(TG)8	(TG)8	(TG)8	(TG)8	(TG)9	-	Introns/Intergenic	-	-	TCAAACCGACGAGCACAATA	TTGCTTGTTTTCGCTCTCCT	264	A	A	A	NA
PMS1195	Os_Chr02	22522670	22522725	(AGAT)14	(AGAT)14	(AGAT)14	(AGAT)14	(AGAT)14	(AGAT)14	(AGAT)15	(AGAT)14	(AGAT)14	(AGAT)14	(AGAT)13	-	Introns/Intergenic	-	-	CGGCTTAGGAGCGTTTGTAG	CGTCCAAGGCAGCTTAATTT	255	A	A	A	NA

Polymorphic SSR (PMS) markers	Chromosomes	Start physical positions (bp)	End physical positions (bp)	Nipponbare	Kasalath	Nagina22	IR64	Pokkali	Bala	Tainung67	Azucena	Moroberekan	*Oryza rufipogon*	*Oryza nivara*	MSU gene locus IDs	Structural annotation	Functional annotation	SSR functional effects	Forward primers (5'-3')	Reverse primers (5'-3')	Amplified product size (bp)	Markers exhibiting polymorphism within *indica* rice	Transcrption factor genes-derived markers	Known cloned genes-derived markers	PMS markers corresponding with RM markers
PMS1196	Os_Chr02	22540459	22540474	(TC)8	(TC)11	(TC)8	(TC)8	(TC)8	(TC)6	(TC)8	(TC)8	(TC)8	(TC)8	(TC)8	-	Introns/Intergenic	-	-	CTCGCACGTGTGTTGCAC	CTCTGCCTCCGGGAGTTG	265	P	A	A	NA
PMS1197	Os_Chr02	22556664	22556677	(AT)7	(AT)7	(AT)7	(AT)7	(AT)7	(AT)7	(AT)6	(AT)7	(AT)7	(AT)7	(AT)7	-	Introns/Intergenic	-	-	GTGCAGATCTCATGCCACAC	TGTTCTCAATCACCCATCAAA	200	A	A	A	NA
PMS1198	Os_Chr02	22560331	22560348	(GAC)6	(GAC)5	(GAC)6	(GAC)5	(GAC)5	(GAC)5	(GAC)6	(GAC)6	(GAC)6	(GAC)6	(GAC)5	-	Introns/Intergenic	-	-	ATCTTGGCCCATCAATCAAG	AGCCCAAAAGCAACTTCAGA	194	P	A	A	NA
PMS1199	Os_Chr02	22572256	22572281	(TA)13	(TA)13	(TA)6	(TA)13	(TA)13	(TA)13	(TA)13	(TA)13	(TA)13	(TA)13	(TA)13	LOC_Os02g37360	DRR	expressed protein	-	AGATTAAGAGTGCGGCGAAA	TACACGGCTGATCATGGAGA	257	P	A	A	NA
PMS1200	Os_Chr02	22612126	22612143	(CT)9	(CT)8	(CT)9	(CT)9	(CT)9	(CT)8	(CT)9	(CT)9	(CT)9	(CT)9	(CT)9	-	Introns/Intergenic	-	-	GTCCACCCAACTCCCGTC	GGAGAGAGAGAGGAAGGGGA	253	P	A	A	NA
PMS1201	Os_Chr02	22684925	22684940	(GA)8	(GA)8	(GA)8	(GA)8	(GA)8	(GA)8	(GA)8	(GA)8	(GA)8	(GA)7	(GA)8	-	Introns/Intergenic	-	-	CGTGCCCTTCTAAACAATCG	TCCTATGTGCGTGTGTTGCT	218	A	A	A	NA
PMS1202	Os_Chr02	22697699	22697716	(GTG)6	(GTG)9	(GTG)6	(GTG)6	(GTG)6	(GTG)9	(GTG)6	(GTG)6	(GTG)6	(GTG)6	(GTG)6	LOC_Os02g37610	URR	expressed protein	-	GGATGGAAGGAAGCACACAT	GTGCACGCCAACCTTTTTAT	211	P	A	A	NA
PMS1203	Os_Chr02	22732062	22732095	(AT)17	(AT)17	(AT)17	(AT)17	(AT)17	(AT)15	(AT)17	(AT)17	(AT)17	(AT)10	(AT)13	-	Introns/Intergenic	-	-	CACAAGGTAATACTCCCTCCG	CCCTTGGATGGTTGATCTAGG	279	P	A	A	NA
PMS1204	Os_Chr02	22760126	22760221	(TAA)32	(TAA)20	(TAA)32	(TAA)32	(TAA)32	(TAA)32	(TAA)32	(TAA)28	(TAA)32	(TAA)32	(TAA)32	-	Introns/Intergenic	-	-	AAGGAGTTGGGTCGTGTGAAGG	TACTTCTTTATAGCCCGCTCGTTGC	235	P	A	A	RM13534
PMS1205	Os_Chr02	22762340	22762357	(TC)9	(TC)12	(TC)9	(TC)9	(TC)9	(TC)9	(TC)9	(TC)9	(TC)9	(TC)9	(TC)9	-	Introns/Intergenic	-	-	CGATCTATCGTCAAGTTCAAT	AGAGCGGGGGAGAGGGAGAG	168	P	A	A	NA
PMS1206	Os_Chr02	22987500	22987515	(GA)8	(GA)8	(GA)8	(GA)8	(GA)6	(GA)8	(GA)8	(GA)8	(GA)8	(GA)8	(GA)8	LOC_Os02g38050	URR	joka2, putative, expressed	-	ACAACAATCCCCCAACAGAA	ACCGTCTTCTCTGGTTGTCG	252	P	A	A	NA
PMS1207	Os_Chr02	23030738	23030759	(CA)11	(CA)10	(CA)11	(CA)11	(CA)11	(CA)11	(CA)11	(CA)11	(CA)11	(CA)11	(CA)11	-	Introns/Intergenic	-	-	ACAGCCTCCTCCAGCTACAGACC	GGGATGACAAGATGGGTGAGG	148	P	A	A	RM13547
PMS1208	Os_Chr02	23095586	23095601	(AG)8	(AG)8	(AG)8	(AG)8	(AG)8	(AG)8	(AG)8	(AG)8	(AG)8	(AG)9	(AG)8	-	Introns/Intergenic	-	-	CGAACCAAACACATGCCTTA	TCATGTGTAGGGCGAGCATA	253	A	A	A	NA

Polymorphic SSR (PMS) markers	Chromosomes	Start physical positions (bp)	End physical positions (bp)	Nipponbare	Kasalath	Nagina22	IR64	Pokkali	Bala	Tainung67	Azucena	Moroberekan	*Oryza rufipogon*	*Oryza nivara*	MSU gene locus IDs	Structural annotation	Functional annotation	SSR functional effects	Forward primers (5'-3')	Reverse primers (5'-3')	Amplified product size (bp)	Markers exhibiting polymorphism within *indica* rice	Transcrption factor genes-derived markers	Known cloned genes-derived markers	PMS markers corresponding with RM markers
PMS1209	Os_Chr02	23096338	23096353	(GT)8	(GT)7	(GT)8	(GT)8	(GT)8	(GT)7	(GT)8	(GT)8	(GT)8	(GT)8	(GT)8	-	Introns/Intergenic	-	-	ATCACCACAGTAGCACGCAC	GCTTCTTCCAAGTCGTCGTC	181	P	A	A	NA
PMS1210	Os_Chr02	23143549	23143566	(GCG)6	(GCG)5	(GCG)6	(GCG)6	(GCG)6	(GCG)5	(GCG)6	(GCG)6	(GCG)6	(GCG)6	(GCG)6	LOC_Os02g38260	CDS	glycosyl hydrolase family 5 protein, putative, expressed	CODON_DELETION	TACACCGTGCTGTTCCAC	CTTGAACCACTGGGTCTCT	459	P	A	A	NA
PMS1211	Os_Chr02	23279380	23279397	(CAG)6	(CAG)6	(CAG)6	(CAG)6	(CAG)6	(CAG)6	(CAG)6	(CAG)5	(CAG)6	(CAG)6	(CAG)6	-	Introns/Intergenic	-	-	AGATGGCTATGACCTGCCTG	ATCGATCACATCATGTTGGC	163	A	A	A	NA
PMS1212	Os_Chr02	23325848	23325865	(CAG)6	(CAG)6	(CAG)6	(CAG)6	(CAG)6	(CAG)6	(CAG)6	(CAG)5	(CAG)6	(CAG)6	(CAG)6	LOC_Os02g38574	CDS	EMB2423, putative, expressed	CODON_CHANGE_PLUS_CODON_DELETION	AGATGGCTATGACCTGCCTG	ATCGATCACATCATGTTGGC	163	A	A	A	NA
PMS1213	Os_Chr02	23397984	23398007	(CAG)8	(CAG)8	(CAG)8	(CAG)8	(CAG)8	(CAG)8	(CAG)7	(CAG)8	(CAG)5	(CAG)8	(CAG)5	-	Introns/Intergenic	-	-	CCATTGGCATGTTTGTCTTG	ATCGATCACATCATGTTGGC	192	A	A	A	NA
PMS1214	Os_Chr02	23412466	23412491	(TC)13	(TC)13	(TC)13	(TC)13	(TC)13	(TC)13	(TC)6	(TC)13	(TC)12	(TC)13	(TC)13	-	Introns/Intergenic	-	-	CTTGTCGTCGATGAGGCAC	CCCGAGGGTAGATCGGAC	256	A	A	A	NA
PMS1215	Os_Chr02	23448200	23448217	(GA)9	(GA)9	(GA)9	(GA)9	(GA)9	(GA)9	(GA)8	(GA)9	(GA)9	(GA)9	(GA)9	-	Introns/Intergenic	-	-	GCCTGCCAGAGAGAAGAGTG	TACACCGAAGGTCCCTCAAC	234	A	A	A	NA
PMS1216	Os_Chr02	23490582	23490595	(CT)7	(CT)6	(CT)7	(CT)7	(CT)7	(CT)7	(CT)7	(CT)7	(CT)7	(CT)7	(CT)7	-	Introns/Intergenic	-	-	AACAGGCGACAAGCAATAAA	AGGGTGAACCCTACGAAACC	161	P	A	A	NA
PMS1217	Os_Chr02	23506726	23506757	(ATAG)8	(ATAG)7	(ATAG)8	(ATAG)8	(ATAG)8	(ATAG)8	(ATAG)8	(ATAG)8	(ATAG)8	(ATAG)8	(ATAG)8	-	Introns/Intergenic	-	-	GCATCCACCTGTCTTCTTTCA	GCTGAGTCAGGTTTTTCGATG	155	P	A	A	NA
PMS1218	Os_Chr02	23573079	23573108	(CT)15	(CT)17	(CT)15	(CT)15	(CT)15	(CT)15	(CT)15	(CT)15	(CT)15	(CT)15	(CT)17	-	Introns/Intergenic	-	-	TTCACTCCTCTCCTTTCCCA	AGCCTGACGAGCACAGAAGT	239	P	A	A	NA
PMS1219	Os_Chr02	23649328	23649351	(AT)12	(AT)12	(AT)12	(AT)12	(AT)12	(AT)12	(AT)12	(AT)12	(AT)12	(AT)11	(AT)12	-	Introns/Intergenic	-	-	CAAGGCTTCTTGATCTCTTCATCC	GATATTGCGTACGTACCCTCTCC	388	A	A	A	RM13575
PMS1220	Os_Chr02	23839906	23839919	(GA)7	(GA)6	(GA)7	(GA)7	(GA)7	(GA)7	(GA)7	(GA)7	(GA)7	(GA)7	(GA)7	-	Introns/Intergenic	-	-	GAGACCATGTTTTCATGGGG	AGCTCGCCAGATCCAGTG	269	P	A	A	NA
PMS1221	Os_Chr02	23875311	23875328	(TA)9	(TA)9	(TA)9	(TA)9	(TA)9	(TA)9	(TA)6	(TA)9	(TA)9	(TA)9	(TA)9	-	Introns/Intergenic	-	-	TGTGTTTAAAAGTTAATGGCATCAT	TCCGCATTAGAATTGGCTTC	206	A	A	A	NA

Polymorphic SSR (PMS) markers	Chromosomes	Start physical positions (bp)	End physical positions (bp)	Nipponbare	Kasalath	Nagina22	IR64	Pokkali	Bala	Tainung67	Azucena	Moroberekan	*Oryza rufipogon*	*Oryza nivara*	MSU gene locus IDs	Structural annotation	Functional annotation	SSR functional effects	Forward primers (5'-3')	Reverse primers (5'-3')	Amplified product size (bp)	Markers exhibiting polymorphism within *indica* rice	Transcrption factor genes-derived markers	Known cloned genes-derived markers	PMS markers corresponding with RM markers
PMS1222	Os_Chr02	24006144	24006164	(AGG)7	(AGG)7	(AGG)7	(AGG)7	(AGG)7	(AGG)7	(AGG)7	(AGG)7	(AGG)7	(AGG)6	(AGG)7	-	Introns/Intergenic	-	-	GCGTCAGAGTGGTCACAGGTAGG	GACATCGCCTCCTCGACACG	299	A	A	A	RM13591
PMS1223	Os_Chr02	24174213	24174226	(GA)7	(GA)11	(GA)7	(GA)7	(GA)7	(GA)7	(GA)7	(GA)7	(GA)7	(GA)7	(GA)7	LOC_Os02g39970	URR	regulatory subunit, putative, expressed	-	AGAGATCAGCCACCTCCTCA	TAACAGTTTTCCGCCCACTC	138	P	A	A	NA
PMS1224	Os_Chr02	24195270	24195283	(CT)7	(CT)7	(CT)7	(CT)7	(CT)7	(CT)7	(CT)7	(CT)7	(CT)7	(CT)6	(CT)7	-	Introns/Intergenic	-	-	CATTTCAATTTGGACCACCC	AGGGACTCAGGGAGGTAGGA	197	A	A	A	NA
PMS1225	Os_Chr02	24202840	24202855	(CT)8	(CT)7	(CT)8	(CT)8	(CT)7	(CT)8	(CT)8	(CT)8	(CT)8	(CT)8	(CT)7	-	Introns/Intergenic	-	-	ATCACGCGTTTCAAAGGAAC	CAAATCTCCCCACCCTTTTT	193	P	A	A	NA
PMS1226	Os_Chr02	24290611	24290632	(TC)11	(TC)11	(TC)11	(TC)11	(TC)11	(TC)11	(TC)7	(TC)11	(TC)11	(TC)11	(TC)11	-	Introns/Intergenic	-	-	ACGACCTGGGTTCGAAGCTTTACC	TTGTTGTGTGCCACAGCATAATGG	351	A	A	A	RM13601
PMS1227	Os_Chr02	24326122	24326139	(AT)9	(AT)9	(AT)9	(AT)9	(AT)9	(AT)9	(AT)9	(AT)9	(AT)9	(AT)9	(AT)7	-	Introns/Intergenic	-	-	GCATGGATGGGAAAAGCTAA	TTTTATACCTTGGAGCCCGA	106	A	A	A	NA
PMS1228	Os_Chr02	24555647	24555736	(TA)45	(TA)45	(TA)45	(TA)45	(TA)45	(TA)45	(TA)8	(TA)45	(TA)45	(TA)45	(TA)45	-	Introns/Intergenic	-	-	ACTGCAGGTGGTACCTTGATCC	GTGTTGTTCACTTGTTCAGCTTCC	587	A	A	A	RM13604
PMS1229	Os_Chr02	24561058	24561075	(CCG)6	(CCG)6	(CCG)6	(CCG)6	(CCG)6	(CCG)6	(CCG)6	(CCG)5	(CCG)5	(CCG)6	(CCG)6	-	Introns/Intergenic	-	-	GATGTACTCATCCTGGCCGT	AAGGGAAAATCAGCACGATG	241	A	A	A	NA
PMS1230	Os_Chr02	24679828	24679849	(CT)11	(CT)9	(CT)11	(CT)11	(CT)11	(CT)11	(CT)11	(CT)11	(CT)11	(CT)11	(CT)11	-	Introns/Intergenic	-	-	CTTTACTTTGACTCGTCCTGTGG	CTCACTGAATGTGAGTGAATGC	83	P	A	A	RM13606
PMS1231	Os_Chr02	24709730	24709753	(AT)12	(AT)12	(AT)12	(AT)12	(AT)12	(AT)12	(AT)12	(AT)12	(AT)12	(AT)12	(AT)10	-	Introns/Intergenic	-	-	TGCTCTCAAGGCCTAGAAATTACC	CTATGCGCAACTCCATTAAAGC	182	A	A	A	RM13607
PMS1232	Os_Chr02	24816933	24816950	(TCG)6	(TCG)5	(TCG)5	(TCG)6	(TCG)6	(TCG)6	(TCG)6	(TCG)6	(TCG)6	(TCG)6	(TCG)6	LOC_Os02g41450	CDS	no apical meristem protein, putative, expressed	CODON_DELETION	TGCGTATACGAATGCAGAGG	GGAACTCGTGCATAACCCAG	266	P	P	A	NA
PMS1233	Os_Chr02	24833304	24833369	(TTA)22	(TTA)22	(TTA)5	(TTA)22	(TTA)22	(TTA)22	(TTA)6	(TTA)10	(TTA)22	(TTA)22	(TTA)22	-	Introns/Intergenic	-	-	CATTTCATGTTTATGGCAGAT	TCACTCTTTCTTAATTGGGATT	320	P	A	A	NA
PMS1234	Os_Chr02	24885992	24886039	(AT)24	(AT)24	(AT)24	(AT)24	(AT)10	(AT)24	(AT)24	(AT)9	(AT)24	(AT)6	(AT)9	-	Introns/Intergenic	-	-	TGGCTCCGTCGAGTCAAACTGG	TGATGCTTTGGCCTTCAAGTAGCC	409	P	A	A	RM13619

**Polymorphic SSR (PMS) markers**	**Chromosomes**	**Start physical positions (bp)**	**End physical positions (bp)**	**Nipponbare**	**Kasalath**	**Nagina22**	**IR64**	**Pokkali**	**Bala**	**Tainung67**	**Azucena**	**Moroberekan**	***Oryza rufipogon***	***Oryza nivara***	**MSU gene locus IDs**	**Structural annotation**	**Functional annotation**	**SSR functional effects**	**Forward primers (5'-3')**	**Reverse primers (5'-3')**	**Amplified product size (bp)**	**Markers exhibiting polymorphism within *indica* rice**	**Transcrption factor genes-derived markers**	**Known cloned genes-derived markers**	**PMS markers corresponding with RM markers**
PMS1235	Os_Chr02	24909016	24909091	(TA)38	(TA)38	(TA)11	(TA)38	(TA)38	(TA)38	(TA)38	(TA)38	(TA)38	(TA)6	(TA)14	-	Introns/Intergenic	-	-	GCTGGGAACTAGGACGAAACC	GCTTGCATTAACCACCTAAGTTCC	198	P	A	A	RM13622
PMS1236	Os_Chr02	24945228	24945247	(AG)10	(AG)10	(AG)10	(AG)10	(AG)9	(AG)10	(AG)10	(AG)10	(AG)10	(AG)10	(AG)10	-	Introns/Intergenic	-	-	AGCGTGTGTCAAGAGCAAGACC	GAGATGAAGAAGAATGCCCAAGC	145	P	A	A	RM13623
PMS1237	Os_Chr02	24953331	24953348	(TC)9	(TC)9	(TC)9	(TC)9	(TC)9	(TC)7	(TC)9	(TC)9	(TC)9	(TC)9	(TC)9	LOC_Os02g41610	URR	expressed protein	-	AACACAGAACACACCACCCC	TAAATTGCACCACCACAGGA	279	P	A	A	NA
PMS1238	Os_Chr02	25023800	25023815	(CT)8	(CT)8	(CT)8	(CT)8	(CT)8	(CT)8	(CT)8	(CT)8	(CT)8	(CT)7	(CT)8	-	Introns/Intergenic	-	-	ATCCCGAATCCAAATCCTTC	GGCAGAGCGGAGAGAGAGAG	148	A	A	A	NA
PMS1239	Os_Chr02	25052114	25052127	(TA)7	(TA)6	(TA)7	(TA)6	(TA)7	(TA)7	(TA)7	(TA)7	(TA)7	(TA)7	(TA)7	-	Introns/Intergenic	-	-	CGTAGCACCGCTCTTGTTTA	TAACTGATTGGCACAAGGGC	148	P	A	A	NA
PMS1240	Os_Chr02	25185348	25185419	(TA)36	(TA)36	(TA)36	(TA)9	(TA)36	(TA)36	(TA)36	(TA)12	(TA)36	(TA)36	(TA)36	-	Introns/Intergenic	-	-	ATAATGATGTTAGCCGCGCA	AGGCTAACCTTGCGCTATGA	269	P	A	A	NA
PMS1241	Os_Chr02	25326728	25326739	(TA)6	(TA)7	(TA)6	(TA)6	(TA)6	(TA)6	(TA)6	(TA)6	(TA)6	(TA)6	(TA)6	-	Introns/Intergenic	-	-	TGCACGTAAAATGGAGCAAC	TGGCCCCTAGTTAAGGGAGT	260	P	A	A	NA
PMS1242	Os_Chr02	25440679	25440766	(TA)44	(TA)44	(TA)44	(TA)44	(TA)44	(TA)44	(TA)44	(TA)8	(TA)9	(TA)44	(TA)44	-	Introns/Intergenic	-	-	TTTATATGCGGCCCTACTCTTCC	ACTAAGACAAGCAAGTCAGGATGC	214	A	A	A	RM13646
PMS1243	Os_Chr02	25505314	25505331	(GCG)6	(GCG)6	(GCG)6	(GCG)6	(GCG)6	(GCG)6	(GCG)6	(GCG)6	(GCG)6	(GCG)5	(GCG)6	LOC_Os02g42390	CDS	expressed protein	CODON_DELETION	TGGAACTCCAAGATCTTCCTGAACG	CAAGCCGAGTAGGCCCAAGAGG	178	A	A	A	RM13649
PMS1244	Os_Chr02	25528676	25528693	(GTC)6	(GTC)6	(GTC)6	(GTC)6	(GTC)6	(GTC)6	(GTC)5	(GTC)6	(GTC)6	(GTC)6	(GTC)6	LOC_Os02g42440	CDS	GEM, putative, expressed	CODON_DELETION	CCTTCCTGCCATATTTGCTC	TTTCGTTGTTGGTTCCTTCC	189	A	A	A	NA
PMS1245	Os_Chr02	25626565	25626612	(TA)24	(TA)24	(TA)24	(TA)8	(TA)7	(TA)7	(TA)24	(TA)24	(TA)24	(TA)20	(TA)7	-	Introns/Intergenic	-	-	ATGGTGCCCCATATGAGTGT	CAATTTTGGTAGGTGGTGGG	164	P	A	A	NA
PMS1246	Os_Chr02	25803405	25803422	(CCG)6	(CCG)6	(CCG)6	(CCG)6	(CCG)6	(CCG)6	(CCG)5	(CCG)6	(CCG)6	(CCG)6	(CCG)6	LOC_Os02g42900	CDS	expressed protein	CODON_DELETION	CTGTCGCGTCAGACCCAT	AGCATGGGTTCCAGGCAT	255	A	A	A	NA
PMS1247	Os_Chr02	25855822	25855842	(CGG)7	(CGG)7	(CGG)7	(CGG)7	(CGG)7	(CGG)7	(CGG)7	(CGG)7	(CGG)5	(CGG)7	(CGG)7	LOC_Os02g42960	CDS	thylakoid lumenal protein, putative, expressed	CODON_DELETION	GGACGGCAATTCGTTGAG	TCCGCCTCGAACTTGTTAAG	147	A	A	A	NA

Polymorphic SSR (PMS) markers	Chromosomes	Start physical positions (bp)	End physical positions (bp)	Nipponbare	Kasalath	Nagina22	IR64	Pokkali	Bala	Tainung67	Azucena	Moroberekan	*Oryza rufipogon*	*Oryza nivara*	MSU gene locus IDs	Structural annotation	Functional annotation	SSR functional effects	Forward primers (5'-3')	Reverse primers (5'-3')	Amplified product size (bp)	Markers exhibiting polymorphism within *indica* rice	Transcrption factor genes-derived markers	Known cloned genes-derived markers	PMS markers corresponding with RM markers
PMS1248	Os_Chr02	25863746	25863761	(TC)8	(TC)7	(TC)8	(TC)8	(TC)8	(TC)8	(TC)8	(TC)8	(TC)8	(TC)8	(TC)8	-	Introns/Intergenic	-	-	GGAGGTGGAGTGGAGTGAAA	GCCAAACACGAGAGATCGTT	149	P	A	A	NA
PMS1249	Os_Chr02	25997459	25997479	(GGC)7	(GGC)7	(GGC)7	(GGC)7	(GGC)7	(GGC)7	(GGC)7	(GGC)6	(GGC)7	(GGC)7	(GGC)7	LOC_Os02g43150	CDS	GATA zinc finger domain containing protein, expressed	CODON_CHANGE_PLUS_CODON_DELETION	AGGAGCTCTGTGAGGTGGACAGG	TGAGCCATGGGAGTCACTAGACC	110	A	P	A	RM13671
PMS1250	Os_Chr02	26002796	26002840	(AAT)15	(AAT)15	(AAT)15	(AAT)15	(AAT)11	(AAT)15	(AAT)7	(AAT)14	(AAT)14	(AAT)15	(AAT)9	-	Introns/Intergenic	-	-	TGTTTACGGCCTACCGCTTCC	ACAGTGCAGTTCCTCCAAATTGC	121	P	A	A	RM13672
PMS1251	Os_Chr02	26109536	26109553	(TA)9	(TA)9	(TA)9	(TA)9	(TA)9	(TA)9	(TA)9	(TA)9	(TA)9	(TA)6	(TA)9	-	Introns/Intergenic	-	-	GAGACAAAGGCCGAAGACAG	TGATGGATCGAAAACAAGCA	277	A	A	A	NA
PMS1252	Os_Chr02	26123964	26123999	(CCTCTC)6	(CCTCTC)6	(CCTCTC)6	(CCTCTC)6	(CCTCTC)6	(CCTCTC)6	(CCTCTC)6	(CCTCTC)6	(CCTCTC)6	(CCTCTC)5	(CCTCTC)6	-	Introns/Intergenic	-	-	GGCTCTCCTTCCCGTCTC	GATCCACCCTCGGACGAA	231	A	A	A	NA
PMS1253	Os_Chr02	26212525	26212542	(CGG)6	(CGG)6	(CGG)6	(CGG)6	(CGG)6	(CGG)6	(CGG)6	(CGG)6	(CGG)6	(CGG)6	(CGG)5	LOC_Os02g43430	CDS	protein kinase domain containing protein, expressed	CODON_DELETION	GACACCGTGGAGGTGGAGT	TACTGTTCCTCGGAGATGGG	155	A	A	A	NA
PMS1254	Os_Chr02	26242438	26242463	(CT)13	(CT)12	(CT)13	(CT)13	(CT)13	(CT)12	(CT)13	(CT)13	(CT)13	(CT)13	(CT)13	-	Introns/Intergenic	-	-	CCACTGATACAGAAGCTGATGAGG	AGCACAATGACTCCCTTCTTTGC	186	P	A	A	RM3730
PMS1255	Os_Chr02	26288867	26288884	(ACT)6	(ACT)6	(ACT)6	(ACT)6	(ACT)6	(ACT)6	(ACT)6	(ACT)6	(ACT)6	(ACT)7	(ACT)6	-	Introns/Intergenic	-	-	GACGTGATCAGGCAGAGACA	TTTGGGACGGCAGTAGTAGG	236	A	A	A	NA
PMS1256	Os_Chr02	26320330	26320353	(GGC)8	(GGC)8	(GGC)8	(GGC)8	(GGC)8	(GGC)8	(GGC)5	(GGC)8	(GGC)8	(GGC)8	(GGC)8	LOC_Os02g43600	CDS	50S ribosomal protein L19, chloroplast precursor, putative, expressed	CODON_DELETION	GGGAGTAACGCACCGGAGATGG	ACCCACTTTACAGCGGCACAGC	177	A	A	A	RM13692
PMS1257	Os_Chr02	26348884	26348927	(CT)22	(CT)21	(CT)22	(CT)22	(CT)22	(CT)22	(CT)22	(CT)22	(CT)22	(CT)22	(CT)22	LOC_Os02g43670	DRR	transferase family protein, putative, expressed	-	CTCTCCAGATCCACATCCTCTCC	GAGTCACCGCTGCCAGATCC	358	P	A	A	RM13694
PMS1258	Os_Chr02	26384872	26384891	(AAAT)5	(AAAT)6	(AAAT)5	(AAAT)5	(AAAT)5	(AAAT)5	(AAAT)5	(AAAT)5	(AAAT)5	(AAAT)5	(AAAT)5	-	Introns/Intergenic	-	-	GCTAGCTCCAGATTCAGGATGACC	AATCCTAGGAAGACGCTGTGTGC	214	P	A	A	RM13701
PMS1259	Os_Chr02	26425870	26425915	(CT)23	(CT)23	(CT)23	(CT)23	(CT)23	(CT)23	(CT)23	(CT)11	(CT)23	(CT)23	(CT)23	-	Introns/Intergenic	-	-	CCGCAAAAGTCTAGTTTGCC	CGGAGGAGGAGGAGCACT	267	A	A	A	NA
PMS1260	Os_Chr02	26435930	26435947	(CT)9	(CT)9	(CT)6	(CT)9	(CT)9	(CT)9	(CT)9	(CT)9	(CT)9	(CT)9	(CT)8	-	Introns/Intergenic	-	-	CCGCAAAAGTCTAGTTTGCC	CGGAGGAGGAGGAGCACT	241	P	A	A	NA

Polymorphic SSR (PMS) markers	Chromosomes	Start physical positions (bp)	End physical positions (bp)	Nipponbare	Kasalath	Nagina22	IR64	Pokkali	Bala	Tainung67	Azucena	Moroberekan	*Oryza rufipogon*	*Oryza nivara*	MSU gene locus IDs	Structural annotation	Functional annotation	SSR functional effects	Forward primers (5'-3')	Reverse primers (5'-3')	Amplified product size (bp)	Markers exhibiting polymorphism within *indica* rice	Transcrption factor genes-derived markers	Known cloned genes-derived markers	PMS markers corresponding with RM markers
PMS1261	Os_Chr02	26450907	26450926	(AT)10	(AT)10	(AT)10	(AT)10	(AT)10	(AT)10	(AT)8	(AT)10	(AT)10	(AT)10	(AT)10	-	Introns/Intergenic	-	-	GAGAAGGGATTGAGGGGAAG	ACGGTGCATTTTGCAAGTTT	274	A	A	A	NA
PMS1262	Os_Chr02	26653109	26653128	(GC)10	(GC)10	(GC)10	(GC)6	(GC)6	(GC)10	(GC)10	(GC)10	(GC)10	(GC)10	(GC)10	-	Introns/Intergenic	-	-	ACCATCACCAACCAACCAACC	CCGTTGCTTTCAGTCTTTCACC	171	P	A	A	RM13718
PMS1263	Os_Chr02	26780155	26780175	(AGG)7	(AGG)7	(AGG)7	(AGG)7	(AGG)7	(AGG)7	(AGG)6	(AGG)7	(AGG)7	(AGG)7	(AGG)7	LOC_Os02g44250	CDS	expressed protein	CODON_DELETION	GAGGAGGAGGAGGAGGAGAG	TCCCTCCGAATCTCCAATC	249	A	A	A	NA
PMS1264	Os_Chr02	27115118	27115141	(CGC)8	(CGC)8	(CGC)8	(CGC)8	(CGC)7	(CGC)8	(CGC)8	(CGC)8	(CGC)8	(CGC)8	(CGC)8	-	Introns/Intergenic	-	-	CTGCAGCTCTTCCCAGAGAACC	CGCGGCAGAGTAGCAGAACC	100	P	A	A	RM599
PMS1265	Os_Chr02	27155518	27155535	(GCG)6	(GCG)5	(GCG)6	(GCG)6	(GCG)6	(GCG)5	(GCG)6	(GCG)6	(GCG)6	(GCG)6	(GCG)6	LOC_Os02g44840	CDS	expressed protein	CODON_DELETION	TTCTCTGGCTTCTTCTCTGGCTCTGG	GCTCCAGCCAATCCACCTTGC	178	P	A	A	RM13744
PMS1266	Os_Chr02	27262359	27262376	(CCG)6	(CCG)5	(CCG)6	(CCG)6	(CCG)6	(CCG)5	(CCG)6	(CCG)6	(CCG)6	(CCG)6	(CCG)5	LOC_Os02g45000	CDS	expressed protein	CODON_DELETION	CTCCAGGCTCTTCTTCTGCC	GGTGAAGGGGTGGAGGAG	175	P	A	A	NA
PMS1267	Os_Chr02	27311771	27311828	(AT)29	(AT)29	(AT)10	(AT)29	(AT)11	(AT)11	(AT)29	(AT)29	(AT)29	(AT)29	(AT)29	-	Introns/Intergenic	-	-	AACAGGCACCTATCATTTCTCC	CTAGTAGTGTACACAGCGAAGAAACC	475	P	A	A	RM13748
PMS1268	Os_Chr02	27319447	27319466	(TA)10	(TA)10	(TA)9	(TA)10	(TA)10	(TA)10	(TA)10	(TA)10	(TA)10	(TA)10	(TA)10	-	Introns/Intergenic	-	-	AATTCGTTGGGCTGTCTGTC	AACCCATCTTTTTATGGTTGGA	241	P	A	A	NA
PMS1269	Os_Chr02	27455811	27455890	(TA)40	(TA)40	(TA)36	(TA)40	(TA)40	(TA)40	(TA)40	(TA)15	(TA)40	(TA)36	(TA)40	-	Introns/Intergenic	-	-	ATGGGTCATAGCGAATGCTT	ATTAGATACCATGGTGCCCG	236	P	A	A	NA
PMS1270	Os_Chr02	27500509	27500524	(GA)8	(GA)8	(GA)8	(GA)8	(GA)8	(GA)8	(GA)8	(GA)8	(GA)8	(GA)8	(GA)7	-	Introns/Intergenic	-	-	CTGAGAAGCTGGTGGTGGA	CGCCTGCAAAAATCGTATCT	127	A	A	A	NA
PMS1271	Os_Chr02	27559374	27559391	(GCG)6	(GCG)6	(GCG)6	(GCG)6	(GCG)6	(GCG)6	(GCG)5	(GCG)6	(GCG)6	(GCG)6	(GCG)6	LOC_Os02g45344	URR	O-acyltransferase, putative, expressed	-	TCTTGTATCACCCGCATCTG	GAACGAGAGGTAGGAAAGCG	238	A	A	A	NA
PMS1272	Os_Chr02	27612283	27612300	(TCG)6	(TCG)6	(TCG)5	(TCG)6	(TCG)6	(TCG)6	(TCG)6	(TCG)6	(TCG)6	(TCG)6	(TCG)5	LOC_Os02g45400	CDS	transposon protein, putative, Pong sub-class	CODON_DELETION	TTATCTCACGACCTGGGACC	TGATCTTGTGATGGCAGTGTT	202	P	A	A	NA
PMS1273	Os_Chr02	27640836	27640875	(AT)20	(AT)20	(AT)20	(AT)10	(AT)9	(AT)20	(AT)20	(AT)14	(AT)20	(AT)14	(AT)20	-	Introns/Intergenic	-	-	GGTCGAAACGCTAAATACGG	AAAGTAATCACCTTAGAGCATTATGAA	233	P	A	A	NA

Polymorphic SSR (PMS) markers	Chromosomes	Start physical positions (bp)	End physical positions (bp)	Nipponbare	Kasalath	Nagina22	IR64	Pokkali	Bala	Tainung67	Azucena	Moroberekan	*Oryza rufipogon*	*Oryza nivara*	MSU gene locus IDs	Structural annotation	Functional annotation	SSR functional effects	Forward primers (5'-3')	Reverse primers (5'-3')	Amplified product size (bp)	Markers exhibiting polymorphism within *indica* rice	Transcrption factor genes-derived markers	Known cloned genes-derived markers	PMS markers corresponding with RM markers
PMS1274	Os_Chr02	27775008	27775028	(CCA)7	(CCA)7	(CCA)7	(CCA)7	(CCA)7	(CCA)7	(CCA)7	(CCA)7	(CCA)7	(CCA)7	(CCA)6	-	Introns/Intergenic	-	-	TCACAGCTTCCCTCATGAACACC	CACCGCCTCCTCACTCTTCC	552	A	A	A	RM13774
PMS1275	Os_Chr02	27791872	27791886	(CCT)5	(CCT)5	(CCT)6	(CCT)6	(CCT)6	(CCT)6	(CCT)5	(CCT)5	(CCT)5	(CCT)5	(CCT)6	LOC_Os02g45680	CDS	retrotransposon protein, putative, unclassified, expressed	CODON_INSERTION	GGGCAAAATTTGAAGAAGCA	GTAAAGCTTCATGCCCGGTA	271	P	A	A	NA
PMS1276	Os_Chr02	27792547	27792570	(AT)12	(AT)12	(AT)12	(AT)12	(AT)12	(AT)12	(AT)12	(AT)12	(AT)12	(AT)12	(AT)6	-	Introns/Intergenic	-	-	GTGAACTTGTGCGATGATGG	ATGGAAATCAAAAGCTCCCC	222	A	A	A	NA
PMS1277	Os_Chr02	27843944	27843963	(GA)10	(GA)10	(GA)10	(GA)10	(GA)10	(GA)8	(GA)10	(GA)10	(GA)10	(GA)10	(GA)10	-	Introns/Intergenic	-	-	GAGCTTAGCAGACAACACTTCC	TTTCCTCCTACACAACTTGTCC	93	P	A	A	RM13780
PMS1278	Os_Chr02	27918830	27918847	(GGC)6	(GGC)5	(GGC)6	(GGC)6	(GGC)6	(GGC)6	(GGC)6	(GGC)6	(GGC)5	(GGC)6	(GGC)6	-	Introns/Intergenic	-	-	AGTCCGACTGCAAAAATCGT	AGATCTAGGAGGGAGGGGG	262	P	A	A	NA
PMS1279	Os_Chr02	27920960	27920977	(GA)9	(GA)9	(GA)9	(GA)9	(GA)9	(GA)8	(GA)9	(GA)9	(GA)9	(GA)9	(GA)9	-	Introns/Intergenic	-	-	TCAATCTGATCGATCCCCTC	ATGCTTTTGTTCCCATGCTC	257	P	A	A	NA
PMS1280	Os_Chr02	27936077	27936090	(TA)7	(TA)7	(TA)7	(TA)7	(TA)7	(TA)7	(TA)7	(TA)7	(TA)7	(TA)7	(TA)6	-	Introns/Intergenic	-	-	TTATTCGTCGCTGATGTCGT	CGCCCTATAATTTAGGATGGA	133	A	A	A	NA
PMS1281	Os_Chr02	27962505	27962522	(CTC)6	(CTC)6	(CTC)5	(CTC)5	(CTC)5	(CTC)5	(CTC)6	(CTC)6	(CTC)6	(CTC)6	(CTC)5	-	Introns/Intergenic	-	-	CCTCCTTCGTAAGCATCTCG	ACCAACCCAGTTGGAGTACG	188	P	A	A	NA
PMS1282	Os_Chr02	27965951	27965974	(CCG)8	(CCG)8	(CCG)8	(CCG)8	(CCG)8	(CCG)8	(CCG)8	(CCG)8	(CCG)8	(CCG)5	(CCG)8	LOC_Os02g45880	CDS	expressed protein	CODON_CHANGE_PLUS_CODON_DELETION	GAAGCTAATCGTCGAGACCG	CTGCCCAGAACGGAGTAGAG	261	A	A	A	NA
PMS1283	Os_Chr02	28051080	28051097	(CT)9	(CT)9	(CT)9	(CT)7	(CT)9	(CT)9	(CT)9	(CT)9	(CT)9	(CT)9	(CT)9	-	Introns/Intergenic	-	-	TTTTGCACCCTTCTCTTGCT	AATGTCAAACCGGAGTCGAG	141	P	A	A	NA
PMS1284	Os_Chr02	28084008	28084028	(TCC)7	(TCC)7	(TCC)7	(TCC)7	(TCC)7	(TCC)7	(TCC)7	(TCC)7	(TCC)7	(TCC)5	(TCC)8	-	Introns/Intergenic	-	-	TCCCGCTTCGACTTCTTTCTCTTCC	CGTTAGGAGAAGAGCCGGAGATTGG	135	A	A	A	RM13787
PMS1285	Os_Chr02	28113283	28113303	(GCG)7	(GCG)7	(GCG)7	(GCG)7	(GCG)7	(GCG)7	(GCG)7	(GCG)7	(GCG)7	(GCG)6	(GCG)7	LOC_Os02g46120	URR	regulatory protein, putative, expressed	-	GAAGTGACGGAGGGAGGTG	CGTTTACGGAAACCACGAGT	203	A	A	A	NA
PMS1286	Os_Chr02	28161314	28161334	(TGC)7	(TGC)7	(TGC)7	(TGC)7	(TGC)7	(TGC)6	(TGC)7	(TGC)7	(TGC)7	(TGC)7	(TGC)6	LOC_Os02g46220	CDS	expressed protein	CODON_DELETION	CACAAGGCTTTCAGGCGTTTGC	ACTCGTGTCCACTGGCCTTCG	87	P	A	A	RM13791

Polymorphic SSR (PMS) markers	Chromosomes	Start physical positions (bp)	End physical positions (bp)	Nipponbare	Kasalath	Nagina22	IR64	Pokkali	Bala	Tainung67	Azucena	Moroberekan	*Oryza rufipogon*	*Oryza nivara*	MSU gene locus IDs	Structural annotation	Functional annotation	SSR functional effects	Forward primers (5'-3')	Reverse primers (5'-3')	Amplified product size (bp)	Markers exhibiting polymorphism within *indica* rice	Transcrption factor genes-derived markers	Known cloned genes-derived markers	PMS markers corresponding with RM markers
PMS1287	Os_Chr02	28165372	28165415	(AG)22	(AG)22	(AG)22	(AG)22	(AG)22	(AG)23	(AG)22	(AG)22	(AG)22	(AG)22	(AG)22	-	Introns/Intergenic	-	-	ATGGCGATTGAAGCAAGAAACC	GTTGTTTGCAAGGAAGGTAGATGC	243	P	A	A	RM1342
PMS1288	Os_Chr02	28176912	28176935	(TC)12	(TC)13	(TC)12	(TC)12	(TC)12	(TC)12	(TC)12	(TC)12	(TC)13	(TC)12	(TC)13	-	Introns/Intergenic	-	-	TGCTGCATCTCCATATCACAAGG	TCCCTCTCACTTCACACAGATGG	162	P	A	A	RM5305
PMS1289	Os_Chr02	28215131	28215182	(TA)26	(TA)26	(TA)6	(TA)6	(TA)6	(TA)6	(TA)6	(TA)9	(TA)26	(TA)6	(TA)7	-	Introns/Intergenic	-	-	CATGCACTGACGTAATGCAGACC	TCTGACATGAGCTGTGGAGAAGG	137	P	A	A	RM13794
PMS1290	Os_Chr02	28273512	28273547	(AAG)12	(AAG)12	(AAG)12	(AAG)6	(AAG)12	(AAG)7	(AAG)12	(AAG)12	(AAG)12	(AAG)12	(AAG)12	LOC_Os02g46410	CDS	expressed protein	CODON_DELETION	GTTGGTATTCCCTGATGAGTTTGC	GAGACAGAATCCTTACGCTGTGC	175	P	A	A	RM5631,RM525
PMS1291	Os_Chr02	28407521	28407534	(CT)7	(CT)7	(CT)7	(CT)7	(CT)7	(CT)7	(CT)7	(CT)7	(CT)7	(CT)7	(CT)6	-	Introns/Intergenic	-	-	CTGTCAGGTCGTCCCCTTC	GGAAGCTTTATAGGGGCTCG	279	A	A	A	NA
PMS1292	Os_Chr02	28414958	28414973	(TA)8	(TA)8	(TA)7	(TA)8	(TA)8	(TA)8	(TA)8	(TA)8	(TA)8	(TA)8	(TA)8	-	Introns/Intergenic	-	-	ATTATCCAACGCGGCATTAC	ACGTTGGGGAGTGATTTGAG	181	P	A	A	NA
PMS1293	Os_Chr02	28540677	28540746	(TA)35	(TA)35	(TA)9	(TA)35	(TA)35	(TA)35	(TA)35	(TA)35	(TA)35	(TA)35	(TA)35	-	Introns/Intergenic	-	-	CGGAATTTGTCTTAGTGAGGATGC	GACTGCGGCAATTTGTATCAGG	303	P	A	A	RM13807
PMS1294	Os_Chr02	28608133	28608152	(GA)10	(GA)10	(GA)10	(GA)10	(GA)10	(GA)7	(GA)10	(GA)10	(GA)10	(GA)10	(GA)10	-	Introns/Intergenic	-	-	GATCGTTCGTTCGTTCATGTGG	GATCGATCTCTCTCGGCTTCTCC	481	P	A	A	RM13815
PMS1295	Os_Chr02	28652814	28652853	(TC)20	(TC)20	(TC)20	(TC)20	(TC)20	(TC)20	(TC)20	(TC)20	(TC)20	(TC)20	(TC)17	LOC_Os02g46940	URR	OsKANADI1|OsKANADI2	-	CACACTCCTGTCCTTTCCATTCC	AGGAGTTCTTGCTTGGAAGATGG	117	A	P	P	RM13819
PMS1296	Os_Chr02	28654367	28654382	(AG)8	(AG)8	(AG)8	(AG)8	(AG)8	(AG)8	(AG)8	(AG)8	(AG)8	(AG)7	(AG)7	-	Introns/Intergenic	-	-	AAGTTTCACTCCCCACATGC	GCTGCTATAGCTTTGTGCCC	157	A	A	A	NA
PMS1297	Os_Chr02	28738508	28738579	(AT)36	(AT)36	(AT)36	(AT)8	(AT)7	(AT)8	(AT)36	(AT)36	(AT)36	(AT)36	(AT)36	-	Introns/Intergenic	-	-	CTCAATTTCAGCAATGGTGC	AGATCATCATGGGAAGCGAC	174	P	A	A	NA
PMS1298	Os_Chr02	28743516	28743553	(TA)19	(TA)19	(TA)10	(TA)6	(TA)19	(TA)19	(TA)19	(TA)19	(TA)19	(TA)10	(TA)14	-	Introns/Intergenic	-	-	CGTGAACACAAGAATATGCCAAGG	GACCCTTACCAACTTCCCATTCG	377	P	A	A	RM13834
PMS1299	Os_Chr02	28752929	28752946	(TC)9	(TC)9	(TC)9	(TC)9	(TC)9	(TC)10	(TC)9	(TC)9	(TC)9	(TC)9	(TC)9	LOC_Os02g47090	DRR	peptide transporter PTR2, putative, expressed	-	AAGTTGCGATTGGTGAAAGG	GTTGCTGACATGCCATAGGA	186	P	A	A	NA

Polymorphic SSR (PMS) markers	Chromosomes	Start physical positions (bp)	End physical positions (bp)	Nipponbare	Kasalath	Nagina22	IR64	Pokkali	Bala	Tainung67	Azucena	Moroberekan	*Oryza rufipogon*	*Oryza nivara*	MSU gene locus IDs	Structural annotation	Functional annotation	SSR functional effects	Forward primers (5'-3')	Reverse primers (5'-3')	Amplified product size (bp)	Markers exhibiting polymorphism within *indica* rice	Transcrption factor genes-derived markers	Known cloned genes-derived markers	PMS markers corresponding with RM markers
PMS1300	Os_Chr02	28872631	28872678	(ATA)16	(ATA)16	(ATA)16	(ATA)16	(ATA)16	(ATA)10	(ATA)16	(ATA)16	(ATA)14	(ATA)16	(ATA)16	-	Introns/Intergenic	-	-	GCGAACAGACTAGCCAGACAACC	ATATTGTAACAGTGCCGCTGTGC	331	P	A	A	RM8248,RM5833
PMS1301	Os_Chr02	29033953	29033985	(GTG)11	(GTG)11	(GTG)11	(GTG)11	(GTG)11	(GTG)11	(GTG)11	(GTG)11	(GTG)11	(GTG)11	(GTG)9	LOC_Os02g47520	CDS	expressed protein	CODON_CHANGE_PLUS_CODON_DELETION	GCTGCTTGTGTTGTTGTTGTCG	CACAGGCTCCTCTTCACCTATGG	199	A	A	A	RM497
PMS1302	Os_Chr02	29086610	29086624	(GGA)5	(GGA)5	(GGA)5	(GGA)5	(GGA)5	(GGA)5	(GGA)5	(GGA)5	(GGA)5	(GGA)7	(GGA)5	LOC_Os02g47600	URR	soluble inorganic pyrophosphatase, putative, expressed	-	AACTCACAAACACGCACGAG	TTTACCCATCTCCCCTCCTC	280	A	A	A	NA
PMS1303	Os_Chr02	29109109	29109138	(AGT)10	(AGT)10	(AGT)10	(AGT)10	(AGT)10	(AGT)7	(AGT)5	(AGT)10	(AGT)10	(AGT)10	(AGT)10	LOC_Os02g47640	URR	OsFBT4 - F-box and tubby domain containing protein, expressed	-	CGTCAGTAATGGCGGATCTTGC	CTTCGCTATCACCGAAGGAACC	86	P	A	A	RM13854
PMS1304	Os_Chr02	29132583	29132645	(TAA)21	(TAA)21	(TAA)6	(TAA)13	(TAA)21	(TAA)21	(TAA)21	(TAA)21	(TAA)21	(TAA)21	(TAA)21	-	Introns/Intergenic	-	-	ATGAGAGCGAACGTGGAGAC	GCTAGAGCCACTGGCGAC	257	P	A	A	NA
PMS1305	Os_Chr02	29219635	29219652	(TC)9	(TC)10	(TC)10	(TC)9	(TC)9	(TC)9	(TC)9	(TC)10	(TC)10	(TC)9	(TC)9	-	Introns/Intergenic	-	-	TGTGCACTGGATGCTCTACC	CCTCCGAAACAAAGAAACGA	117	P	A	A	NA
PMS1306	Os_Chr02	29265467	29265484	(AAG)6	(AAG)6	(AAG)6	(AAG)6	(AAG)6	(AAG)6	(AAG)6	(AAG)6	(AAG)6	(AAG)5	(AAG)6	LOC_Os02g47840	URR	universal stress protein domain containing protein, putative, expressed	-	GGAGGAAGAGGAGGTGGAAG	GAGCAGATTGCCTTTTGTCC	275	A	A	A	NA
PMS1307	Os_Chr02	29313483	29313584	(ATA)34	(ATA)34	(ATA)34	(ATA)26	(ATA)34	(ATA)34	(ATA)34	(ATA)24	(ATA)6	(ATA)34	(ATA)34	-	Introns/Intergenic	-	-	AATGCCTAGCACTCATCCTTGC	AGGCACCCTACGATGAAATAGTGG	460	P	A	A	RM6933
PMS1308	Os_Chr02	29331001	29331018	(GCC)6	(GCC)6	(GCC)6	(GCC)6	(GCC)6	(GCC)6	(GCC)6	(GCC)6	(GCC)6	(GCC)5	(GCC)6	LOC_Os02g47950	CDS	expressed protein	CODON_DELETION	CGAAGCACCTCTCTTTCCAC	GCAGATCACCGTGTTCCTCT	278	A	A	A	NA
PMS1309	Os_Chr02	29393395	29393440	(TA)23	(TA)23	(TA)23	(TA)23	(TA)15	(TA)23	(TA)23	(TA)23	(TA)9	(TA)23	(TA)23	-	Introns/Intergenic	-	-	CCAGGCTGCCCACACTATTGG	TTGAGATGATCAGGCTCCTAACTGC	385	P	A	A	RM13874
PMS1310	Os_Chr02	29438295	29438308	(CG)7	(CG)7	(CG)7	(CG)7	(CG)7	(CG)7	(CG)9	(CG)9	(CG)9	(CG)7	(CG)7	-	Introns/Intergenic	-	-	ATCATGCGTTCTTTGGCTCT	GGCATCGTCTCTCTCTCGTC	240	A	A	A	NA
PMS1311	Os_Chr02	29481413	29481433	(CGG)7	(CGG)7	(CGG)7	(CGG)7	(CGG)7	(CGG)7	(CGG)7	(CGG)5	(CGG)7	(CGG)7	(CGG)7	-	Introns/Intergenic	-	-	TTAGAGCCAACAAACCGACC	CATCTTCAGCGAGGAGAACC	203	A	A	A	NA
PMS1312	Os_Chr02	29481764	29481784	(TGC)7	(TGC)7	(TGC)7	(TGC)7	(TGC)7	(TGC)7	(TGC)7	(TGC)6	(TGC)7	(TGC)7	(TGC)7	LOC_Os02g48170	CDS	class E vacuolar protein-sorting machinery protein HSE1, putative, expressed	CODON_DELETION	TGAAGATGGTGGTGAGCCTGTTGG	CGCCAACTCCGTCTACGTGATCC	545	A	A	A	RM13880

Polymorphic SSR (PMS) markers	Chromosomes	Start physical positions (bp)	End physical positions (bp)	Nipponbare	Kasalath	Nagina22	IR64	Pokkali	Bala	Tainung67	Azucena	Moroberekan	*Oryza rufipogon*	*Oryza nivara*	MSU gene locus IDs	Structural annotation	Functional annotation	SSR functional effects	Forward primers (5'-3')	Reverse primers (5'-3')	Amplified product size (bp)	Markers exhibiting polymorphism within *indica* rice	Transcrption factor genes-derived markers	Known cloned genes-derived markers	PMS markers corresponding with RM markers
PMS1313	Os_Chr02	29503662	29503715	(TA)27	(TA)27	(TA)27	(TA)27	(TA)27	(TA)27	(TA)27	(TA)27	(TA)27	(TA)27	(TA)25	-	Introns/Intergenic	-	-	CTTGCCCAAAGATCAAGAATGC	GGCCCATAATTGTAGGAAACTGC	378	A	A	A	RM13881
PMS1314	Os_Chr02	29512367	29512386	(TA)10	(TA)10	(TA)10	(TA)10	(TA)10	(TA)8	(TA)10	(TA)10	(TA)10	(TA)10	(TA)7	-	Introns/Intergenic	-	-	GCTTCTAGCAGCATAATGTGAGG	CTCGTGTTTATGTTCGAGATGG	432	P	A	A	RM13882
PMS1315	Os_Chr02	29516973	29516986	(TA)7	(TA)6	(TA)7	(TA)7	(TA)7	(TA)7	(TA)7	(TA)7	(TA)7	(TA)7	(TA)6	-	Introns/Intergenic	-	-	ACTAGCAACAGCCTGCCATT	TTGGAACTTGGAACAGCCAT	237	P	A	A	NA
PMS1316	Os_Chr02	29528774	29528791	(CGC)6	(CGC)5	(CGC)6	(CGC)6	(CGC)6	(CGC)6	(CGC)6	(CGC)6	(CGC)6	(CGC)6	(CGC)6	-	Introns/Intergenic	-	-	CCTCCACAGATGCGATTCTC	ACCTGCAGTCGTCCCTTGTA	271	P	A	A	NA
PMS1317	Os_Chr02	29594819	29594836	(GCA)6	(GCA)6	(GCA)6	(GCA)6	(GCA)5	(GCA)6	(GCA)6	(GCA)6	(GCA)6	(GCA)6	(GCA)6	LOC_Os02g48340	CDS	RNA recognition motif containing protein, putative, expressed	CODON_DELETION	CTTTTCCCCCTTCCAAAATC	CTTGGTGTAGGTGGTGTCCC	263	P	A	A	NA
PMS1318	Os_Chr02	29695161	29695172	(TC)6	(TC)7	(TC)6	(TC)6	(TC)6	(TC)6	(TC)6	(TC)6	(TC)6	(TC)7	(TC)7	-	Introns/Intergenic	-	-	TTGCTTGTTGTCAGTCTCCG	CGGGAGAACGAGAGAGAGAG	269	P	A	A	NA
PMS1319	Os_Chr02	29703154	29703169	(TA)8	(TA)8	(TA)8	(TA)8	(TA)8	(TA)7	(TA)8	(TA)8	(TA)8	(TA)8	(TA)7	-	Introns/Intergenic	-	-	AGAGAGAGCACCAGACCCAA	TCAAAGCACTTGTACAGCCG	194	P	A	A	NA
PMS1320	Os_Chr02	29724451	29724464	(GA)7	(GA)7	(GA)7	(GA)7	(GA)6	(GA)7	(GA)7	(GA)7	(GA)7	(GA)7	(GA)7	-	Introns/Intergenic	-	-	CCCTCGATCTACTTGTCGGA	CACTTTTCATCTCCCCTCCA	209	P	A	A	NA
PMS1321	Os_Chr02	29752367	29752382	(TC)8	(TC)10	(TC)10	(TC)8	(TC)8	(TC)8	(TC)8	(TC)8	(TC)8	(TC)8	(TC)8	-	Introns/Intergenic	-	-	AAAAAGCTTCATGAGAAGAACCA	GGTTTCAGCAAAGATTGGGA	203	P	A	A	NA
PMS1322	Os_Chr02	29805515	29805535	(CTG)7	(CTG)7	(CTG)7	(CTG)7	(CTG)7	(CTG)7	(CTG)6	(CTG)7	(CTG)7	(CTG)7	(CTG)7	-	Introns/Intergenic	-	-	GAGGCGAGAGGAAGAAGAGATGG	ATCTCCCTCCTCAGGCAAAGC	72	A	A	A	RM13895
PMS1323	Os_Chr02	29854224	29854244	(CCG)7	(CCG)7	(CCG)7	(CCG)7	(CCG)7	(CCG)6	(CCG)7	(CCG)7	(CCG)7	(CCG)7	(CCG)7	LOC_Os02g48780	CDS	IQ calmodulin-binding and BAG domain containing protein, putative, expressed	CODON_DELETION	GAGGGGATCTGGGGAGAGT	TATTTCCGGCATATTTCCCA	191	P	A	A	NA
PMS1324	Os_Chr02	29881509	29881524	(TC)8	(TC)7	(TC)8	(TC)8	(TC)8	(TC)8	(TC)8	(TC)8	(TC)8	(TC)8	(TC)8	-	Introns/Intergenic	-	-	TGTTCAGTGCAACATTGGGT	ATGTTTGGATCCATGGGCTA	123	P	A	A	NA
PMS1325	Os_Chr02	29896931	29896958	(TA)14	(TA)14	(TA)14	(TA)11	(TA)9	(TA)14	(TA)6	(TA)14	(TA)14	(TA)14	(TA)14	-	Introns/Intergenic	-	-	TTCTAATACAGTCCTCCTCCATCC	GTGTCACATCCAATCAAAGTACCC	215	P	A	A	RM13903

Polymorphic SSR (PMS) markers	Chromosomes	Start physical positions (bp)	End physical positions (bp)	Nipponbare	Kasalath	Nagina22	IR64	Pokkali	Bala	Tainung67	Azucena	Moroberekan	*Oryza rufipogon*	*Oryza nivara*	MSU gene locus IDs	Structural annotation	Functional annotation	SSR functional effects	Forward primers (5'-3')	Reverse primers (5'-3')	Amplified product size (bp)	Markers exhibiting polymorphism within *indica* rice	Transcrption factor genes-derived markers	Known cloned genes-derived markers	PMS markers corresponding with RM markers
PMS1326	Os_Chr02	30043935	30043948	(CT)7	(CT)7	(CT)7	(CT)7	(CT)7	(CT)7	(CT)7	(CT)8	(CT)7	(CT)7	(CT)7	-	Introns/Intergenic	-	-	ACAGCCTGTCCAGGTGACAT	AGCAGTAGGTCAAACCAAGCA	255	A	A	A	NA
PMS1327	Os_Chr02	30103908	30103925	(GCG)6	(GCG)5	(GCG)6	(GCG)6	(GCG)6	(GCG)6	(GCG)6	(GCG)6	(GCG)6	(GCG)6	(GCG)6	LOC_Os02g49250	CDS	myb-like DNA-binding domain containing protein, expressed	CODON_DELETION	CGACATGAACGCGAAATCTA	GACTCGGAGTACATGGGGAG	171	P	P	A	NA
PMS1328	Os_Chr02	30104359	30104382	(CGC)8	(CGC)8	(CGC)8	(CGC)8	(CGC)7	(CGC)7	(CGC)8	(CGC)8	(CGC)8	(CGC)8	(CGC)8	LOC_Os02g49250	CDS	myb-like DNA-binding domain containing protein, expressed	CODON_DELETION	ACCATTGCCGGAAGAGGTTCTCC	ATACCCTTGCTCTCGCTGCAACC	191	P	P	A	RM13913
PMS1329	Os_Chr02	30159188	30159208	(CGG)7	(CGG)7	(CGG)7	(CGG)7	(CGG)7	(CGG)7	(CGG)7	(CGG)7	(CGG)7	(CGG)7	(CGG)6	LOC_Os02g49360	URR	RNA methyltransferase domain-containing protein 2, putative, expressed	-	CTCTCCAAACCGAGCCACTGC	CTCCTGCTTCAGCAGCTTCTTCC	164	A	A	A	RM13920
PMS1330	Os_Chr02	30167871	30167884	(TA)7	(TA)7	(TA)6	(TA)7	(TA)7	(TA)7	(TA)7	(TA)7	(TA)7	(TA)7	(TA)7	-	Introns/Intergenic	-	-	TTGTCCCATATGCTGACGTG	GCTCCATACGCTAAAATGGC	241	P	A	A	NA
PMS1331	Os_Chr02	30199755	30199775	(GAG)7	(GAG)6	(GAG)7	(GAG)7	(GAG)7	(GAG)7	(GAG)7	(GAG)7	(GAG)7	(GAG)7	(GAG)7	LOC_Os02g49420	URR	fasciclin-like arabinogalactan precursor protein, putative, expressed	-	AAGAGGAGGAGCATGACGTG	AACGGCGACACGTAAGTTTT	238	P	A	A	NA
PMS1332	Os_Chr02	30236846	30236857	(CG)6	(CG)7	(CG)6	(CG)6	(CG)6	(CG)7	(CG)6	(CG)6	(CG)6	(CG)7	(CG)6	-	Introns/Intergenic	-	-	TTTGCTAGCTACGCTTGCCT	CTTCGGTTGGAGGCTGTAAA	146	P	A	A	NA
PMS1333	Os_Chr02	30282900	30282917	(GGA)6	(GGA)6	(GGA)6	(GGA)6	(GGA)6	(GGA)6	(GGA)6	(GGA)6	(GGA)6	(GGA)5	(GGA)6	LOC_Os02g49550	CDS	zinc finger, C3HC4 type domain containing protein, expressed	CODON_DELETION	GAGGTGGAGTTGCCTCTCTG	GTCGATTCCACCGTCGTAGT	240	A	A	A	NA
PMS1334	Os_Chr02	30428095	30428114	(TG)10	(TG)10	(TG)10	(TG)10	(TG)10	(TG)10	(TG)10	(TG)9	(TG)9	(TG)10	(TG)10	-	Introns/Intergenic	-	-	CTTCTTCGCTCCTTTGCTGATGG	AGGAACCCACTCACCACCTCTGC	85	A	A	A	RM13929
PMS1335	Os_Chr02	30434560	30434583	(TCG)8	(TCG)8	(TCG)5	(TCG)8	(TCG)7	(TCG)8	(TCG)8	(TCG)8	(TCG)8	(TCG)8	(TCG)8	-	Introns/Intergenic	-	-	TACGTGGCTACGGACCTGTTTGG	CGACTCGGATTCCTTGGTTCG	649	P	A	A	RM13930
PMS1336	Os_Chr02	30475429	30475446	(CGT)6	(CGT)6	(CGT)6	(CGT)6	(CGT)6	(CGT)6	(CGT)5	(CGT)6	(CGT)6	(CGT)6	(CGT)6	LOC_Os02g49880	CDS	CCT/B-box zinc finger protein, putative, expressed	CODON_DELETION	ACGCCGCCCTCTCTCTTC	CTGTTTGTTCTTTTGTTTCGT	322	A	P	A	NA
PMS1337	Os_Chr02	30541504	30541519	(AT)8	(AT)8	(AT)7	(AT)8	(AT)8	(AT)8	(AT)8	(AT)8	(AT)8	(AT)8	(AT)8	-	Introns/Intergenic	-	-	CACCAACGGAAACGGAGTAT	GTGTTGCATAGTTGTTGGCG	256	P	A	A	NA
PMS1338	Os_Chr02	30563529	30563543	(TAT)5	(TAT)5	(TAT)5	(TAT)5	(TAT)5	(TAT)5	(TAT)5	(TAT)5	(TAT)5	(TAT)6	(TAT)6	-	Introns/Intergenic	-	-	GACTCACCGGAAGCAGAGAC	CTTTAACGAACCCCAACGAA	145	A	A	A	NA

Polymorphic SSR (PMS) markers	Chromosomes	Start physical positions (bp)	End physical positions (bp)	Nipponbare	Kasalath	Nagina22	IR64	Pokkali	Bala	Tainung67	Azucena	Moroberekan	*Oryza rufipogon*	*Oryza nivara*	MSU gene locus IDs	Structural annotation	Functional annotation	SSR functional effects	Forward primers (5'-3')	Reverse primers (5'-3')	Amplified product size (bp)	Markers exhibiting polymorphism within *indica* rice	Transcrption factor genes-derived markers	Known cloned genes-derived markers	PMS markers corresponding with RM markers
PMS1339	Os_Chr02	30576345	30576362	(TC)9	(TC)9	(TC)10	(TC)9	(TC)9	(TC)9	(TC)9	(TC)9	(TC)9	(TC)9	(TC)9	-	Introns/Intergenic	-	-	CTGATGGGGAAGGACGAGTA	AAGAGCTGCGGTTCTAGCTG	176	P	A	A	NA
PMS1340	Os_Chr02	30606807	30606828	(AG)11	(AG)10	(AG)11	(AG)11	(AG)11	(AG)10	(AG)11	(AG)11	(AG)11	(AG)11	(AG)11	-	Introns/Intergenic	-	-	ACTGTTGAACGAACAACACTGCTACC	GCGCTTGGTGAGTTCTTCTCG	206	P	A	A	RM13935
PMS1341	Os_Chr02	30699168	30699185	(CCG)6	(CCG)6	(CCG)6	(CCG)6	(CCG)6	(CCG)6	(CCG)6	(CCG)6	(CCG)6	(CCG)6	(CCG)5	LOC_Os02g50280	CDS	pentatricopeptide, putative, expressed	CODON_DELETION	ACTCCTCGACCTACCCACCT	GCATTCTGTCGAACAGCGTA	181	A	A	A	NA
PMS1342	Os_Chr02	30744293	30744392	(TA)50	(TA)50	(TA)50	(TA)50	(TA)50	(TA)44	(TA)50	(TA)50	(TA)50	(TA)23	(TA)8	-	Introns/Intergenic	-	-	ACGTTAGGGGGCTCTCGTAT	TCAATTTCAACAATGGTGCC	252	P	A	A	NA
PMS1343	Os_Chr02	30823001	30823024	(TAC)8	(TAC)8	(TAC)8	(TAC)8	(TAC)8	(TAC)5	(TAC)8	(TAC)8	(TAC)8	(TAC)8	(TAC)8	-	Introns/Intergenic	-	-	TTGCACACGAGATGACGAGAAGG	GAATTCGCGACACCACACAGC	366	P	A	A	RM13944
PMS1344	Os_Chr02	30834924	30834949	(TA)13	(TA)13	(TA)13	(TA)13	(TA)6	(TA)13	(TA)13	(TA)13	(TA)13	(TA)13	(TA)13	-	Introns/Intergenic	-	-	AAGGGAATGATGACTTGCTGA	GCTTTCTGGATTCCTAAACGG	265	P	A	A	NA
PMS1345	Os_Chr02	30843785	30843802	(GA)9	(GA)9	(GA)9	(GA)10	(GA)9	(GA)9	(GA)9	(GA)9	(GA)9	(GA)9	(GA)9	-	Introns/Intergenic	-	-	AGGCGCGACAGAGGGTGAC	ATCTCTCTCGGCCCATCT	294	P	A	A	NA
PMS1346	Os_Chr02	30870893	30870910	(GGC)6	(GGC)6	(GGC)6	(GGC)6	(GGC)6	(GGC)6	(GGC)6	(GGC)6	(GGC)6	(GGC)6	(GGC)5	-	Introns/Intergenic	-	-	AAAACCGAATGCCACAAAAG	CGGCAACGTCGTAAGTGTTT	228	A	A	A	NA
PMS1347	Os_Chr02	30895928	30895981	(AT)27	(AT)27	(AT)27	(AT)27	(AT)27	(AT)27	(AT)11	(AT)27	(AT)6	(AT)27	(AT)27	-	Introns/Intergenic	-	-	GTAGGCTTGTCACCTTCCCTTCC	GGTCCTGCAGAAAGAATCAAACC	157	A	A	A	RM13947
PMS1348	Os_Chr02	31075074	31075085	(AT)6	(AT)8	(AT)6	(AT)6	(AT)6	(AT)6	(AT)6	(AT)6	(AT)6	(AT)6	(AT)6	-	Introns/Intergenic	-	-	GACCATAAGGAGGAGCACCA	GGCTGAGGTCTGTTCCAAAA	108	P	A	A	NA
PMS1349	Os_Chr02	31145820	31145849	(CCG)10	(CCG)10	(CCG)10	(CCG)10	(CCG)10	(CCG)10	(CCG)10	(CCG)10	(CCG)10	(CCG)5	(CCG)10	LOC_Os02g50940	CDS	NHL repeat-containing protein, putative, expressed	CODON_DELETION	GAGCTTCCGGCCGTAGTTGTGC	AGACCTTCATCCGGCGGTTCG	77	A	A	A	RM6535
PMS1350	Os_Chr02	31190885	31190934	(CT)25	(CT)22	(CT)25	(CT)25	(CT)25	(CT)22	(CT)25	(CT)25	(CT)25	(CT)25	(CT)12	LOC_Os02g50990	URR	RING-H2 finger protein ATL1Q, putative, expressed	-	AGAGCCTCTCCCTCTCCTCTCC	CAGCAAAGAACGAGCTCTCACG	404	P	A	A	RM3857
PMS1351	Os_Chr02	31284802	31284819	(GGC)6	(GGC)6	(GGC)6	(GGC)5	(GGC)6	(GGC)6	(GGC)6	(GGC)6	(GGC)6	(GGC)6	(GGC)6	LOC_Os02g51130	CDS	glycosyl transferase 8 domain containing protein, putative, expressed	CODON_DELETION	ATGAGTCACTGGCAGAAGGG	CAAGCAAGAGGAGCGTGAT	220	P	A	A	NA

Polymorphic SSR (PMS) markers	Chromosomes	Start physical positions (bp)	End physical positions (bp)	Nipponbare	Kasalath	Nagina22	IR64	Pokkali	Bala	Tainung67	Azucena	Moroberekan	*Oryza rufipogon*	*Oryza nivara*	MSU gene locus IDs	Structural annotation	Functional annotation	SSR functional effects	Forward primers (5'-3')	Reverse primers (5'-3')	Amplified product size (bp)	Markers exhibiting polymorphism within *indica* rice	Transcrption factor genes-derived markers	Known cloned genes-derived markers	PMS markers corresponding with RM markers
PMS1352	Os_Chr02	31398089	31398112	(CAG)8	(CAG)7	(CAG)8	(CAG)8	(CAG)8	(CAG)7	(CAG)8	(CAG)8	(CAG)8	(CAG)7	(CAG)7	LOC_Os02g51280	CDS	TCP-domain protein, putative, expressed	CODON_DELETION	CCGCTTCTGTTGAACTTCCACTCG	GGCATTTGGCTCTGCGTGTACC	206	P	P	A	RM13966
PMS1353	Os_Chr02	31398542	31398559	(GGC)6	(GGC)6	(GGC)6	(GGC)6	(GGC)6	(GGC)6	(GGC)5	(GGC)6	(GGC)6	(GGC)6	(GGC)6	LOC_Os02g51280	CDS	TCP-domain protein, putative, expressed	CODON_DELETION	TCAATGCTTACCGGACACAG	TGGTGCTTATGCTCTGATGC	135	A	P	A	NA
PMS1354	Os_Chr02	31399811	31399834	(AGC)8	(AGC)5	(AGC)8	(AGC)8	(AGC)8	(AGC)8	(AGC)8	(AGC)8	(AGC)8	(AGC)5	(AGC)5	-	Introns/Intergenic	-	-	CGTGAAGTACGCACCAAGTCACC	ATAGGAGGAAGGGAGGGAGATCG	280	P	A	A	RM13967
PMS1355	Os_Chr02	31401057	31401080	(TC)12	(TC)11	(TC)12	(TC)11	(TC)11	(TC)11	(TC)12	(TC)12	(TC)12	(TC)12	(TC)12	-	Introns/Intergenic	-	-	CACACAGGCTTCGCAATTTA	CCCTCCTTGTGAGCCTACAG	199	P	A	A	NA
PMS1356	Os_Chr02	31487064	31487089	(TA)13	(TA)13	(TA)13	(TA)13	(TA)13	(TA)13	(TA)13	(TA)6	(TA)13	(TA)6	(TA)13	-	Introns/Intergenic	-	-	GGGGTAGGTAGAGGGCGTAA	GAGGGAGATGCATGGAGAAA	226	A	A	A	NA
PMS1357	Os_Chr02	31503070	31503085	(AG)8	(AG)9	(AG)8	(AG)8	(AG)8	(AG)8	(AG)8	(AG)8	(AG)8	(AG)8	(AG)8	-	Introns/Intergenic	-	-	TCAGCAAATGTTGCGACAAG	TCACCGATGTGTGATTTGGT	262	P	A	A	NA
PMS1358	Os_Chr02	31512343	31512360	(GGA)6	(GGA)6	(GGA)6	(GGA)6	(GGA)6	(GGA)6	(GGA)5	(GGA)6	(GGA)6	(GGA)6	(GGA)6	LOC_Os02g51430	URR	SEC6, putative, expressed	-	CAGATCCTCCATGACAGCAC	TCTTCACGATCTCCGTCTCC	178	A	A	A	NA
PMS1359	Os_Chr02	31564979	31564996	(GCT)6	(GCT)6	(GCT)6	(GCT)6	(GCT)6	(GCT)6	(GCT)6	(GCT)6	(GCT)6	(GCT)6	(GCT)5	LOC_Os02g51540	CDS	eukaryotic aspartyl protease domain containing protein, expressed	CODON_CHANGE_PLUS_CODON_DELETION	CATTACCCCCAGATCCACC	CGGAAGTAATCCCCGCTC	225	A	P	A	NA
PMS1360	Os_Chr02	31571538	31571558	(CGC)7	(CGC)7	(CGC)7	(CGC)7	(CGC)7	(CGC)7	(CGC)7	(CGC)7	(CGC)7	(CGC)7	(CGC)6	LOC_Os02g51540	DRR	eukaryotic aspartyl protease domain containing protein, expressed	-	AACTATGGCCCCAGTACCCT	GCTGACGGACAGTAGGTGGT	221	A	P	A	NA
PMS1361	Os_Chr02	31707862	31707875	(CG)7	(CG)7	(CG)7	(CG)7	(CG)7	(CG)7	(CG)7	(CG)7	(CG)7	(CG)7	(CG)6	LOC_Os02g51770	URR	TLD family protein, putative, expressed	-	TGACTTGGCAGTTGTGTTCG	CCGCTAAGTTTAGGAGGCG	227	A	A	A	NA
PMS1362	Os_Chr02	31781270	31781281	(GA)6	(GA)7	(GA)6	(GA)7	(GA)7	(GA)7	(GA)6	(GA)6	(GA)6	(GA)6	(GA)7	-	Introns/Intergenic	-	-	CTCACGGCCAATCTGAAAAT	AAGACTCGGAGGAGGGAGAG	258	P	A	A	NA
PMS1363	Os_Chr02	31796640	31796657	(CGC)6	(CGC)6	(CGC)6	(CGC)6	(CGC)5	(CGC)6	(CGC)6	(CGC)6	(CGC)6	(CGC)6	(CGC)6	-	Introns/Intergenic	-	-	CCCCCATCAAAACCACTAAA	GTTCCTTCTCCCTCCTCACC	166	P	A	A	NA
PMS1364	Os_Chr02	31933148	31933171	(CGC)8	(CGC)8	(CGC)8	(CGC)8	(CGC)8	(CGC)8	(CGC)8	(CGC)8	(CGC)8	(CGC)6	(CGC)8	LOC_Os02g52170	CDS	expressed protein	CODON_DELETION	GTTCCTCATCAGAAGCGACAGC	CGGATGAGCCGATAATAATAGACG	415	A	A	A	RM13981

Polymorphic SSR (PMS) markers	Chromosomes	Start physical positions (bp)	End physical positions (bp)	Nipponbare	Kasalath	Nagina22	IR64	Pokkali	Bala	Tainung67	Azucena	Moroberekan	*Oryza rufipogon*	*Oryza nivara*	MSU gene locus IDs	Structural annotation	Functional annotation	SSR functional effects	Forward primers (5'-3')	Reverse primers (5'-3')	Amplified product size (bp)	Markers exhibiting polymorphism within *indica* rice	Transcrption factor genes-derived markers	Known cloned genes-derived markers	PMS markers corresponding with RM markers
PMS1365	Os_Chr02	32040478	32040569	(TA)46	(TA)46	(TA)46	(TA)46	(TA)10	(TA)6	(TA)46	(TA)46	(TA)7	(TA)46	(TA)46	-	Introns/Intergenic	-	-	CCTGTCCATCACTTCATCGT	TGATGCAAATTAACAAACCTGAA	201	P	A	A	NA
PMS1366	Os_Chr02	32088235	32088246	(AC)6	(AC)8	(AC)8	(AC)10	(AC)8	(AC)8	(AC)6	(AC)6	(AC)6	(AC)6	(AC)8	LOC_Os02g52430	DRR	regulator of ribonuclease, putative, expressed	-	GCAGAAGCAGCACTCCATTT	GAGTGGCTCTACGCCGATAC	121	P	A	A	NA
PMS1367	Os_Chr02	32153961	32153972	(CT)6	(CT)6	(CT)6	(CT)10	(CT)6	(CT)6	(CT)6	(CT)6	(CT)6	(CT)6	(CT)6	-	Introns/Intergenic	-	-	AATAAAATCGAACCGAGCCC	GGACAAACCCGTAACTTTGAA	209	P	A	A	NA
PMS1368	Os_Chr02	32256052	32256111	(AT)30	(AT)30	(AT)30	(AT)30	(AT)30	(AT)30	(AT)30	(AT)30	(AT)30	(AT)11	(AT)30	-	Introns/Intergenic	-	-	AATTAAAGGACCACGAGCCC	CTGTCATTGGTTGGTTGGTG	255	A	A	A	NA
PMS1369	Os_Chr02	32267126	32267161	(CTCCCC)6	(CTCCCC)7	(CTCCCC)6	(CTCCCC)6	(CTCCCC)6	(CTCCCC)5	(CTCCCC)6	(CTCCCC)6	(CTCCCC)6	(CTCCCC)6	(CTCCCC)6	LOC_Os02g52744	CDS	DCL, chloroplast precursor, putative, expressed	CODON_DELETION	GATGAGGTCCTCCCAATC	CCGTACCATGCCGTGACC	410	P	A	A	NA
PMS1370	Os_Chr02	32273733	32273746	(TA)7	(TA)7	(TA)7	(TA)7	(TA)7	(TA)7	(TA)7	(TA)6	(TA)7	(TA)7	(TA)7	-	Introns/Intergenic	-	-	GGCGATTTAAACGGCAATAA	GGCGATGGGATTTTTATCCT	255	A	A	A	NA
PMS1371	Os_Chr02	32303843	32303869	(GCC)9	(GCC)9	(GCC)9	(GCC)9	(GCC)9	(GCC)9	(GCC)9	(GCC)9	(GCC)9	(GCC)7	(GCC)9	LOC_Os02g52830	CDS	lipase, putative, expressed	CODON_DELETION	ACCACAGCAGGTGGAACAG	TGCATTGCATGACCACTTCT	261	A	A	A	NA
PMS1372	Os_Chr02	32323205	32323218	(CT)7	(CT)7	(CT)7	(CT)7	(CT)7	(CT)7	(CT)7	(CT)7	(CT)7	(CT)6	(CT)7	LOC_Os02g52860	URR	phosphate carrier protein, mitochondrial precursor, putative, expressed	-	AGGTGTAGAGGAAGCTCGGG	CCAAAAGATCGATGCCCTAA	256	A	A	A	NA
PMS1373	Os_Chr02	32345024	32345039	(AG)8	(AG)11	(AG)8	(AG)8	(AG)8	(AG)11	(AG)8	(AG)8	(AG)8	(AG)8	(AG)8	-	Introns/Intergenic	-	-	TCCCCTCTTTTCCTTCTGGT	GATCGAAGTTGAGCCGAGAG	234	P	A	A	NA
PMS1374	Os_Chr02	32430880	32430897	(CT)9	(CT)12	(CT)12	(CT)9	(CT)9	(CT)9	(CT)9	(CT)9	(CT)9	(CT)9	(CT)9	-	Introns/Intergenic	-	-	AGAGATGCCAGATTGCCTGT	CTCGTTTGTCCACCCAAACT	268	P	A	A	NA
PMS1375	Os_Chr02	32622670	32622691	(GA)11	(GA)11	(GA)11	(GA)11	(GA)11	(GA)15	(GA)11	(GA)11	(GA)11	(GA)11	(GA)11	-	Introns/Intergenic	-	-	AGAGAGGACATCCACCACTAGCC	AAATCTCTCCCAAGTCCCAATCC	237	P	A	A	RM14008
PMS1376	Os_Chr02	32666623	32666640	(CCG)6	(CCG)6	(CCG)6	(CCG)6	(CCG)6	(CCG)6	(CCG)6	(CCG)6	(CCG)6	(CCG)5	(CCG)6	LOC_Os02g53360	CDS	expressed protein	CODON_DELETION	GTACGGCCTCTTGGAGAGGAAGC	CAAGGACCAGGAGGAGAGAAAGC	300	A	A	A	RM14010
PMS1377	Os_Chr02	32747334	32747357	(GGT)8	(GGT)8	(GGT)8	(GGT)8	(GGT)8	(GGT)8	(GGT)6	(GGT)8	(GGT)8	(GGT)8	(GGT)8	-	Introns/Intergenic	-	-	ATCTGTGCATCTACATGGCTTCG	ATGCCAATTGGTTTGCTCTGC	240	A	A	A	RM6627

**Polymorphic SSR (PMS) markers**	**Chromosomes**	**Start physical positions (bp)**	**End physical positions (bp)**	**Nipponbare**	**Kasalath**	**Nagina22**	**IR64**	**Pokkali**	**Bala**	**Tainung67**	**Azucena**	**Moroberekan**	***Oryza rufipogon***	***Oryza nivara***	**MSU gene locus IDs**	**Structural annotation**	**Functional annotation**	**SSR functional effects**	**Forward primers (5'-3')**	**Reverse primers (5'-3')**	**Amplified product size (bp)**	**Markers exhibiting polymorphism within *indica* rice**	**Transcrption factor genes-derived markers**	**Known cloned genes-derived markers**	**PMS markers corresponding with RM markers**
PMS1378	Os_Chr02	32770916	32770935	(AT)10	(AT)10	(AT)10	(AT)10	(AT)10	(AT)9	(AT)10	(AT)10	(AT)10	(AT)10	(AT)10	-	Introns/Intergenic	-	-	AACTGGTTGGAGTACCCTTGTCG	TGCTGGCATTAAGGGATAAGACG	481	P	A	A	RM14023
PMS1379	Os_Chr02	32780260	32780313	(AG)27	(AG)27	(AG)27	(AG)27	(AG)27	(AG)27	(AG)27	(AG)23	(AG)27	(AG)27	(AG)27	-	Introns/Intergenic	-	-	GTTCAAACCAAGCTGATCACAAGC	GGCGTCAGAGTCAGAGATGAAGG	170	A	A	A	RM250
PMS1380	Os_Chr02	32841204	32841227	(TGC)8	(TGC)8	(TGC)6	(TGC)8	(TGC)8	(TGC)8	(TGC)8	(TGC)8	(TGC)8	(TGC)8	(TGC)8	LOC_Os02g53690	CDS	OsGRF1|rhd1	CODON_DELETION	AAGAAGTGGCGGAGAGGTTT	GACGATGAGAAAGAGAGGCG	140	P	P	P	NA
PMS1381	Os_Chr02	32856036	32856055	(CAGG)5	(CAGG)7	(CAGG)7	(CAGG)7	(CAGG)7	(CAGG)7	(CAGG)5	(CAGG)5	(CAGG)5	(CAGG)7	(CAGG)5	-	Introns/Intergenic	-	-	CTGATTCACAGATGCATGGC	GACGAGAAGACGCCACTGTC	263	A	A	A	NA
PMS1382	Os_Chr02	33351083	33351106	(AT)12	(AT)12	(AT)11	(AT)12	(AT)12	(AT)12	(AT)12	(AT)12	(AT)12	(AT)12	(AT)7	-	Introns/Intergenic	-	-	GACAGAGGGATGGATAGTTAGC	TGAACATGCTCTGTGTATGC	146	P	A	A	RM14056
PMS1383	Os_Chr02	33354967	33354984	(CCG)6	(CCG)6	(CCG)5	(CCG)6	(CCG)6	(CCG)6	(CCG)5	(CCG)6	(CCG)6	(CCG)6	(CCG)6	LOC_Os02g54450	CDS	hypro1, putative, expressed	CODON_DELETION	GTGAGGATGCCGAAGAACAT	CGCAAAGTGCACCTCACTAA	225	P	A	A	NA
PMS1384	Os_Chr02	33400317	33400334	(ACC)6	(ACC)6	(ACC)6	(ACC)6	(ACC)6	(ACC)5	(ACC)6	(ACC)6	(ACC)6	(ACC)6	(ACC)6	LOC_Os02g54520	CDS	MYB family transcription factor, putative, expressed	CODON_DELETION	AATTCTCACAAGCCCACACC	CGTCGTCGTCTATCTCAGCA	261	P	P	A	NA
PMS1385	Os_Chr02	33402203	33402217	(GCG)5	(GCG)6	(GCG)5	(GCG)5	(GCG)5	(GCG)6	(GCG)5	(GCG)5	(GCG)5	(GCG)6	(GCG)5	LOC_Os02g54520	CDS	MYB family transcription factor, putative, expressed	CODON_INSERTION	GAGAACGGACAACGACATCA	TGTTGTAGAGGCCTTGGAGG	245	P	P	A	NA
PMS1386	Os_Chr02	33402477	33402494	(CCG)6	(CCG)6	(CCG)6	(CCG)6	(CCG)6	(CCG)6	(CCG)5	(CCG)6	(CCG)6	(CCG)6	(CCG)6	LOC_Os02g54520	CDS	MYB family transcription factor, putative, expressed	CODON_DELETION	AACACCCCCATCATTTGGT	ACGACGACGAGGTCATCTCT	168	A	P	A	NA
PMS1387	Os_Chr02	33469271	33469291	(CGG)7	(CGG)6	(CGG)7	(CGG)7	(CGG)7	(CGG)7	(CGG)7	(CGG)7	(CGG)7	(CGG)6	(CGG)7	LOC_Os02g54640	CDS	glutamate receptor, putative, expressed	CODON_DELETION	TATTGTGATGCGGGTGTAGATGG	CGACGTCAAGTTCAGGATCACC	172	P	A	A	RM14064
PMS1388	Os_Chr02	33491680	33491700	(CGG)7	(CGG)7	(CGG)7	(CGG)7	(CGG)6	(CGG)7	(CGG)7	(CGG)7	(CGG)7	(CGG)7	(CGG)7	LOC_Os02g54690	CDS	RNA recognition motif containing protein, putative, expressed	CODON_DELETION	ACGGCTAAGCACCAGACGATGC	ACTGCTATACCTGGTGCGCTTGG	362	P	A	A	RM14065
PMS1389	Os_Chr02	33604271	33604294	(TC)12	(TC)11	(TC)12	(TC)12	(TC)12	(TC)12	(TC)12	(TC)12	(TC)12	(TC)12	(TC)12	-	Introns/Intergenic	-	-	TCTCGGCCCAGAATAAATTTCG	GCTAGGACTAGGAGGACTAGGAGAGG	363	P	A	A	RM14068
PMS1390	Os_Chr02	33693959	33693976	(TCC)6	(TCC)5	(TCC)5	(TCC)5	(TCC)5	(TCC)5	(TCC)6	(TCC)6	(TCC)6	(TCC)6	(TCC)5	-	Introns/Intergenic	-	-	AGGAGGCGAAGAGGGAGAG	CAAATCCGAACAAGCCAAAG	248	A	A	A	NA

Polymorphic SSR (PMS) markers	Chromosomes	Start physical positions (bp)	End physical positions (bp)	Nipponbare	Kasalath	Nagina22	IR64	Pokkali	Bala	Tainung67	Azucena	Moroberekan	*Oryza rufipogon*	*Oryza nivara*	MSU gene locus IDs	Structural annotation	Functional annotation	SSR functional effects	Forward primers (5'-3')	Reverse primers (5'-3')	Amplified product size (bp)	Markers exhibiting polymorphism within *indica* rice	Transcrption factor genes-derived markers	Known cloned genes-derived markers	PMS markers corresponding with RM markers
PMS1391	Os_Chr02	33742331	33742345	(GGC)5	(GGC)5	(GGC)5	(GGC)5	(GGC)5	(GGC)5	(GGC)5	(GGC)5	(GGC)7	(GGC)5	(GGC)5	-	Introns/Intergenic	-	-	TTATATAGGCTTCGGGCGTG	CCATCCGGGCTATTGTCTTA	200	A	A	A	NA
PMS1392	Os_Chr02	33762560	33762583	(CGG)8	(CGG)8	(CGG)7	(CGG)6	(CGG)8	(CGG)8	(CGG)8	(CGG)8	(CGG)8	(CGG)8	(CGG)8	LOC_Os02g55120	URR	translation initiation factor IF-3, putative, expressed	-	ACGACGAGGAGGCGTTGTACC	CGGAGACACGGAGCAGATAAGG	227	P	A	A	RM14081
PMS1393	Os_Chr02	33806074	33806094	(GAG)7	(GAG)8	(GAG)7	(GAG)7	(GAG)7	(GAG)7	(GAG)7	(GAG)7	(GAG)7	(GAG)7	(GAG)7	LOC_Os02g55190	CDS	expressed protein	CODON_INSERTION	ATGACACGGACGGTGCTGACG	CTTGCTATCTGAGGCGGGAGTCG	261	P	A	A	RM14088
PMS1394	Os_Chr02	33853731	33853754	(AG)12	(AG)11	(AG)12	(AG)12	(AG)12	(AG)12	(AG)12	(AG)12	(AG)12	(AG)12	(AG)7	LOC_Os02g55290	CDS	expressed protein	FRAME_SHIFT	CACGGTGCGAGATACCTAGAGC	CCATGTCACCACCCTTTATTTCC	264	P	A	A	RM1092
PMS1395	Os_Chr02	33867085	33867102	(GCT)6	(GCT)6	(GCT)5	(GCT)5	(GCT)5	(GCT)5	(GCT)6	(GCT)6	(GCT)6	(GCT)6	(GCT)6	-	Introns/Intergenic	-	-	CGCACACACAAATCACCTCT	TATGCCCTCCTGCTACTGCT	241	P	A	A	NA
PMS1396	Os_Chr02	33928370	33928387	(CGC)6	(CGC)6	(CGC)6	(CGC)6	(CGC)6	(CGC)6	(CGC)6	(CGC)5	(CGC)6	(CGC)6	(CGC)6	-	Introns/Intergenic	-	-	CTTCTTCCACACTTCACGCA	TTCGCCAGCTTCTTCTTGAT	214	A	A	A	NA
PMS1397	Os_Chr02	34048246	34048259	(TC)7	(TC)7	(TC)6	(TC)7	(TC)6	(TC)7	(TC)7	(TC)7	(TC)7	(TC)8	(TC)7	-	Introns/Intergenic	-	-	ACTGCACCTGCACTGTTCAC	TGTGGAGCCCATGTATGAAA	258	P	A	A	NA
PMS1398	Os_Chr02	34077500	34077529	(AC)15	(AC)15	(AC)15	(AC)15	(AC)7	(AC)15	(AC)15	(AC)15	(AC)15	(AC)15	(AC)15	-	Introns/Intergenic	-	-	TCTAACCAGGAAAATTGGCG	AGATTGCCCTTGCAGTTAGC	227	P	A	A	NA
PMS1399	Os_Chr02	34101116	34101154	(AGA)13	(AGA)12	(AGA)12	(AGA)12	(AGA)12	(AGA)12	(AGA)12	(AGA)12	(AGA)12	(AGA)12	(AGA)12	-	Introns/Intergenic	-	-	GCGTGTTAGCTAGGGTTTCG	GTGGTGAAGAAGGAGATCGG	187	A	A	A	NA
PMS1400	Os_Chr02	34101296	34101319	(CT)12	(CT)12	(CT)11	(CT)12	(CT)12	(CT)12	(CT)12	(CT)12	(CT)12	(CT)9	(CT)12	-	Introns/Intergenic	-	-	GAAGAAGACCTTGTGGACGC	AGAGGTGGAGACGATCCTGA	260	P	A	A	NA
PMS1401	Os_Chr02	34125668	34125703	(AGA)12	(AGA)13	(AGA)13	(AGA)13	(AGA)13	(AGA)13	(AGA)13	(AGA)13	(AGA)13	(AGA)13	(AGA)13	-	Introns/Intergenic	-	-	GCGTGTTAGCTAGGGTTTCG	GTGGTGAAGAAGGAGATCGG	184	A	A	A	NA
PMS1402	Os_Chr02	34125845	34125868	(CT)12	(CT)13	(CT)10	(CT)12	(CT)12	(CT)13	(CT)13	(CT)13	(CT)13	(CT)13	(CT)13	-	Introns/Intergenic	-	-	GAAGAAGACCTTGTGGACGC	AGAGGTGGAGACGATCCTGA	260	P	A	A	NA
PMS1403	Os_Chr02	34155385	34155420	(AGA)12	(AGA)13	(AGA)13	(AGA)13	(AGA)13	(AGA)13	(AGA)13	(AGA)13	(AGA)13	(AGA)13	(AGA)13	-	Introns/Intergenic	-	-	TCAGCGTGTTAGCTAGGGTTTCG	GCGTGGTGAAGAAGGAGATCG	189	A	A	A	RM14119

Polymorphic SSR (PMS) markers	Chromosomes	Start physical positions (bp)	End physical positions (bp)	Nipponbare	Kasalath	Nagina22	IR64	Pokkali	Bala	Tainung67	Azucena	Moroberekan	*Oryza rufipogon*	*Oryza nivara*	MSU gene locus IDs	Structural annotation	Functional annotation	SSR functional effects	Forward primers (5'-3')	Reverse primers (5'-3')	Amplified product size (bp)	Markers exhibiting polymorphism within *indica* rice	Transcrption factor genes-derived markers	Known cloned genes-derived markers	PMS markers corresponding with RM markers
PMS1404	Os_Chr02	34155562	34155587	(CT)13	(CT)12	(CT)10	(CT)12	(CT)13	(CT)12	(CT)12	(CT)12	(CT)12	(CT)12	(CT)12	-	Introns/Intergenic	-	-	GAAGAAGACCTTGTGGACGC	AGAGGTGGAGACGATCCTGA	262	P	A	A	NA
PMS1405	Os_Chr02	34276332	34276352	(CGA)7	(CGA)7	(CGA)7	(CGA)7	(CGA)7	(CGA)7	(CGA)7	(CGA)5	(CGA)7	(CGA)7	(CGA)7	LOC_Os02g56010	CDS	anthocyanidin 3-O-glucosyltransferase, putative, expressed	CODON_DELETION	CCAGCTGTTCGACTTCCTCACC	GCGACGTAGATGACGGACTTGG	165	A	A	A	RM14131
PMS1406	Os_Chr02	34296385	34296405	(GGC)7	(GGC)7	(GGC)7	(GGC)7	(GGC)7	(GGC)7	(GGC)7	(GGC)5	(GGC)7	(GGC)7	(GGC)7	LOC_Os02g56014	URR	40S ribosomal protein S30, putative, expressed	-	AAGAGAGGACTCGGAGGAGATGG	CTAGGGCTTCGTCTCATCACACC	114	A	A	A	RM14135
PMS1407	Os_Chr02	34298677	34298696	(TG)10	(TG)10	(TG)10	(TG)10	(TG)11	(TG)10	(TG)10	(TG)10	(TG)10	(TG)10	(TG)10	-	Introns/Intergenic	-	-	TGCATTCTTTGTCCAGAGCTTCC	GCTAGTACCTCCTCCAAGACAACAGC	232	P	A	A	RM14136
PMS1408	Os_Chr02	34356561	34356576	(TC)8	(TC)8	(TC)8	(TC)8	(TC)8	(TC)9	(TC)8	(TC)8	(TC)8	(TC)8	(TC)8	LOC_Os02g56140	URR	helix-loop-helix DNA-binding domain containing protein, expressed	-	GTCATGAGCTGCCCATACCT	GAGAGAGAAGCTGAAGCCGA	205	P	P	A	NA
PMS1409	Os_Chr02	34377596	34377610	(CTG)5	(CTG)5	(CTG)5	(CTG)6	(CTG)6	(CTG)6	(CTG)5	(CTG)5	(CTG)5	(CTG)6	(CTG)6	LOC_Os02g56200	CDS	ELMO/CED-12 family protein, putative, expressed	CODON_INSERTION	TGCTTGGTGTAAGCAACCTG	TCGAAGCCCTGAAGCTGTAT	224	P	A	A	NA
PMS1410	Os_Chr02	34505725	34505738	(TC)7	(TC)6	(TC)7	(TC)7	(TC)7	(TC)7	(TC)7	(TC)7	(TC)7	(TC)7	(TC)7	-	Introns/Intergenic	-	-	GGGGCTTTTCTGAAACAACA	TCGTTGTCGACGTACTGAGC	276	P	A	A	NA
PMS1411	Os_Chr02	34515874	34515905	(CT)16	(CT)16	(CT)16	(CT)16	(CT)16	(CT)15	(CT)16	(CT)16	(CT)16	(CT)16	(CT)7	-	Introns/Intergenic	-	-	TCTCTAATGGCCTTCCAACAAGG	AACGGAGGACTTCTGGTAGACACC	237	P	A	A	RM1251
PMS1412	Os_Chr02	34548958	34548973	(TC)8	(TC)8	(TC)8	(TC)8	(TC)8	(TC)8	(TC)8	(TC)10	(TC)9	(TC)8	(TC)8	-	Introns/Intergenic	-	-	TGCCTCGAAAAGGACAAGTT	ACCAACACTGACCCCTGAAG	168	A	A	A	NA
PMS1413	Os_Chr02	34632748	34632765	(AGG)6	(AGG)6	(AGG)6	(AGG)6	(AGG)6	(AGG)8	(AGG)6	(AGG)6	(AGG)6	(AGG)6	(AGG)6	LOC_Os02g56540	CDS	kinesin motor domain containing protein, putative, expressed	CODON_CHANGE_PLUS_CODON_INSERTION	GGAAGAGCCACACCATGTTC	CGTTTCCTTTAGCATTCGCT	249	P	A	A	NA
PMS1414	Os_Chr02	34683962	34683979	(TTC)6	(TTC)6	(TTC)6	(TTC)5	(TTC)5	(TTC)5	(TTC)6	(TTC)6	(TTC)6	(TTC)6	(TTC)5	-	Introns/Intergenic	-	-	ATTGAATGACATCGCCAACA	TGACGTGTGAACGAAACACA	244	P	A	A	NA
PMS1415	Os_Chr02	34689086	34689106	(TGG)7	(TGG)7	(TGG)5	(TGG)5	(TGG)5	(TGG)5	(TGG)5	(TGG)7	(TGG)7	(TGG)5	(TGG)5	LOC_Os02g56610	CDS	BLS1|BSG1|OsG1L6|TH1	CODON_DELETION	GAAGAAGCTGTCCTGCGTG	GGAAGCAGATTCATCCGGTA	262	P	A	P	NA
PMS1416	Os_Chr02	34738869	34738908	(AT)20	(AT)20	(AT)20	(AT)20	(AT)20	(AT)20	(AT)20	(AT)20	(AT)20	(AT)18	(AT)20	-	Introns/Intergenic	-	-	TCCTCCCACTAGCACCTCTGC	CTGCTACTATTCCAGCCCATGC	589	A	A	A	RM14154

Polymorphic SSR (PMS) markers	Chromosomes	Start physical positions (bp)	End physical positions (bp)	Nipponbare	Kasalath	Nagina22	IR64	Pokkali	Bala	Tainung67	Azucena	Moroberekan	*Oryza rufipogon*	*Oryza nivara*	MSU gene locus IDs	Structural annotation	Functional annotation	SSR functional effects	Forward primers (5'-3')	Reverse primers (5'-3')	Amplified product size (bp)	Markers exhibiting polymorphism within *indica* rice	Transcrption factor genes-derived markers	Known cloned genes-derived markers	PMS markers corresponding with RM markers
PMS1417	Os_Chr02	34850037	34850060	(AT)12	(AT)12	(AT)12	(AT)12	(AT)12	(AT)8	(AT)12	(AT)12	(AT)6	(AT)12	(AT)12	-	Introns/Intergenic	-	-	ACGAGAGTTCGTGG TGACATCG	CGCACTCGCAGCTAT AAATACACG	125	P	A	A	RM14159
PMS1418	Os_Chr02	34857817	34857866	(TA)25	(TA)25	(TA)12	(TA)25	(TA)12	(TA)11	(TA)25	(TA)25	(TA)9	(TA)25	(TA)22	-	Introns/Intergenic	-	-	AGGGAGGGAGAGA TCGAGAG	CCATGGAGCACCAA ACAAAT	255	P	A	A	NA
PMS1419	Os_Chr02	34858259	34858294	(AT)18	(AT)18	(AT)9	(AT)7	(AT)10	(AT)9	(AT)18	(AT)18	(AT)18	(AT)8	(AT)18	-	Introns/Intergenic	-	-	ATTAGATACCATGG TGCCCG	GCGAGAACAAATGG GAAAAA	259	P	A	A	NA
PMS1420	Os_Chr02	34859207	34859286	(TA)40	(TA)40	(TA)40	(TA)40	(TA)15	(TA)40	(TA)40	(TA)8	(TA)9	(TA)40	(TA)40	-	Introns/Intergenic	-	-	GGAGGTAGCAGACT GGGCTAGG	TCCTCATCTCATCTC ATCTCATCC	288	P	A	A	RM14163
PMS1421	Os_Chr02	34958856	34958873	(AGC)6	(AGC)6	(AGC)6	(AGC)6	(AGC)6	(AGC)6	(AGC)6	(AGC)6	(AGC)6	(AGC)6	(AGC)5	LOC_Os02g57090	CDS	anthranilate phosphoribosyltransferase, putative, expressed	CODON_CHANGE_PLUS_CODON_DELETION	GTTGTCGTCAAGTC CAAGCC	TCCTTGAGAGAGAAG TCGCC	263	A	A	A	NA
PMS1422	Os_Chr02	34982604	34982661	(TA)29	(TA)29	(TA)10	(TA)16	(TA)29	(TA)29	(TA)29	(TA)7	(TA)29	(TA)29	(TA)29	-	Introns/Intergenic	-	-	CAAGAGATGCCTCA CTTCTTGC	ATGCTGTTAGATGAT GCCATGC	439	P	A	A	RM14169
PMS1423	Os_Chr02	34991251	34991268	(CCT)6	(CCT)5	(CCT)6	(CCT)6	(CCT)6	(CCT)5	(CCT)6	(CCT)6	(CCT)5	(CCT)6	(CCT)6	LOC_Os02g57140	URR	expressed protein	-	TGTACTACGCCGCT TCACTG	AGGAGAGAGATGGG GAGGAA	116	P	A	A	NA
PMS1424	Os_Chr02	35036713	35036732	(TA)10	(TA)10	(TA)9	(TA)10	(TA)7	(TA)10	(TA)10	(TA)10	(TA)10	(TA)10	(TA)10	-	Introns/Intergenic	-	-	GGTCTTTCAGCGTT ACTTGAGC	AGTGGCATATTGCTA CTTCAGC	300	P	A	A	RM14172
PMS1425	Os_Chr02	35086149	35086175	(CTT)9	(CTT)8	(CTT)9	(CTT)9	(CTT)7	(CTT)8	(CTT)9	(CTT)9	(CTT)8	(CTT)9	(CTT)9	LOC_Os02g57270	DRR	RTBP1	-	GCCTCACCTCATCA TCACAACG	TGGTCCTTCCTACTG TTGGTTCG	186	P	A	P	RM6307
PMS1426	Os_Chr02	35105665	35105702	(AC)19	(AC)19	(AC)19	(AC)19	(AC)19	(AC)19	(AC)19	(AC)19	(AC)12	(AC)19	(AC)19	-	Introns/Intergenic	-	-	TTGATCCACTATCG TGGCCTACC	TGGCAAGTGCAACT GCAAGG	190	A	A	A	RM1063
PMS1427	Os_Chr02	35141709	35141732	(CT)12	(CT)15	(CT)12	(CT)12	(CT)15	(CT)15	(CT)12	(CT)12	(CT)12	(CT)10	(CT)15	-	Introns/Intergenic	-	-	AGTACCACCACCAT TCTCTGCAAGC	TCGATTGGCCATGAG TTCTCG	282	P	A	A	RM208
PMS1428	Os_Chr02	35144500	35144523	(GA)12	(GA)12	(GA)12	(GA)12	(GA)12	(GA)12	(GA)12	(GA)12	(GA)12	(GA)12	(GA)11	-	Introns/Intergenic	-	-	GTGATGCATCTTGA GAAGAGATGG	CACCCTTTCAGTGCC TCTAAACC	202	A	A	A	RM3542
PMS1429	Os_Chr02	35223089	35223106	(CGG)6	(CGG)6	(CGG)6	(CGG)6	(CGG)6	(CGG)6	(CGG)6	(CGG)5	(CGG)5	(CGG)5	(CGG)6	LOC_Os02g57480	CDS	transferase family protein, putative, expressed	CODON_DELETION	TTGAGCTTGTCGAT GCTGTC	CCAGCTTCATGCACT TCGT	262	A	A	A	NA

**Polymorphic SSR (PMS) markers**	**Chromosomes**	**Start physical positions (bp)**	**End physical positions (bp)**	**Nipponbare**	**Kasalath**	**Nagina22**	**IR64**	**Pokkali**	**Bala**	**Tainung67**	**Azucena**	**Moroberekan**	***Oryza rufipogon***	***Oryza nivara***	**MSU gene locus IDs**	**Structural annotation**	**Functional annotation**	**SSR functional effects**	**Forward primers (5'-3')**	**Reverse primers (5'-3')**	**Amplified product size (bp)**	**Markers exhibiting polymorphism within *indica* rice**	**Transcrption factor genes-derived markers**	**Known cloned genes-derived markers**	**PMS markers corresponding with RM markers**
PMS1430	Os_Chr02	35242016	35242047	(TC)16	(TC)16	(TC)16	(TC)16	(TC)16	(TC)16	(TC)16	(TC)10	(TC)16	(TC)16	(TC)16	-	Introns/Intergenic	-	-	GTGTGCTCCTTGGGAAGAAAGG	GGGAGAAAGGTGGACATGTAAGC	125	A	A	A	RM406
PMS1431	Os_Chr02	35265104	35265118	(GAG)5	(GAG)5	(GAG)5	(GAG)5	(GAG)5	(GAG)5	(GAG)5	(GAG)5	(GAG)5	(GAG)6	(GAG)5	LOC_Os02g57560	CDS	tyrosine protein kinase domain containing protein, putative, expressed	CODON_INSERTION	ACAATTTACATGGCGGCTTC	GTTGTGGTTCAGCTCGCTCT	137	A	A	A	NA
PMS1432	Os_Chr02	35288153	35288170	(CCA)6	(CCA)7	(CCA)6	(CCA)6	(CCA)6	(CCA)7	(CCA)6	(CCA)6	(CCA)6	(CCA)6	(CCA)7	LOC_Os02g57620	CDS	Citrate transporter protein, putative, expressed	CODON_CHANGE_PLUS_CODON_INSERTION	CCATCCTGCTCTGCCTCTAC	CTTCACTGGCTCGAACACCT	221	P	A	A	NA
PMS1433	Os_Chr02	35361937	35361950	(CT)7	(CT)7	(CT)7	(CT)7	(CT)7	(CT)7	(CT)7	(CT)7	(CT)7	(CT)9	(CT)7	-	Introns/Intergenic	-	-	TGCACCAAGAGCTGAAAATG	TCGGTATATTGAGTCCCCCA	200	A	A	A	NA
PMS1434	Os_Chr02	35375437	35375494	(AG)29	(AG)29	(AG)29	(AG)29	(AG)29	(AG)27	(AG)29	(AG)29	(AG)29	(AG)29	(AG)29	-	Introns/Intergenic	-	-	ATCCTAGTGGATAAGGCACAGACTGG	CCCTTGCTCTTCCACCTCATCC	342	P	A	A	RM207
PMS1435	Os_Chr02	35389697	35389717	(CGG)7	(CGG)7	(CGG)7	(CGG)6	(CGG)7	(CGG)6	(CGG)7	(CGG)7	(CGG)7	(CGG)7	(CGG)7	LOC_Os02g57790	CDS	ZOS2-19 - C2H2 zinc finger protein, expressed	CODON_DELETION	AGCTGCAGAATTGGCTTCTTGC	GGACACCAAGAAAGAGGGAAGC	165	P	A	A	RM14198
PMS1436	Os_Chr02	35431694	35431741	(GA)24	(GA)13	(GA)24	(GA)24	(GA)24	(GA)24	(GA)24	(GA)22	(GA)12	(GA)24	(GA)24	-	Introns/Intergenic	-	-	GCCTCGAGAAAGAAGCATGTCC	AAGCGACCTGTACCTCAACTTCG	285	P	A	A	RM3850
PMS1437	Os_Chr02	35436186	35436199	(TC)7	(TC)7	(TC)7	(TC)6	(TC)6	(TC)6	(TC)7	(TC)7	(TC)7	(TC)7	(TC)7	-	Introns/Intergenic	-	-	TCCCTCAATTAGCAAAACCG	TCCTCTCCCACCTCACAGAC	205	P	A	A	NA
PMS1438	Os_Chr02	35464218	35464229	(GT)6	(GT)6	(GT)6	(GT)6	(GT)6	(GT)6	(GT)6	(GT)7	(GT)7	(GT)6	(GT)6	-	Introns/Intergenic	-	-	CACCTACCTCGTTTTCTGCG	AGGGCATGTTTAAGGGTGTG	187	A	A	A	NA
PMS1439	Os_Chr02	35483918	35483935	(GGC)6	(GGC)5	(GGC)6	(GGC)6	(GGC)6	(GGC)6	(GGC)6	(GGC)6	(GGC)6	(GGC)6	(GGC)6	LOC_Os02g57940	URR	OsFBX74 - F-box domain containing protein, expressed	-	CTTGCGTCGACTACCTTTCC	TCGAGAGGAGGCCCATAATA	255	P	A	A	NA
PMS1440	Os_Chr02	35623027	35623040	(AG)7	(AG)7	(AG)7	(AG)7	(AG)7	(AG)7	(AG)7	(AG)7	(AG)7	(AG)8	(AG)7	-	Introns/Intergenic	-	-	AGAACGATGGAACGGAAAAA	ATCACGGAGGGGACATGTAA	267	A	A	A	NA
PMS1441	Os_Chr02	35709597	35709614	(GCT)6	(GCT)6	(GCT)6	(GCT)6	(GCT)6	(GCT)6	(GCT)6	(GCT)6	(GCT)6	(GCT)5	(GCT)6	LOC_Os02g58390	CDS	inactive receptor kinase At2g26730 precursor, putative, expressed	CODON_DELETION	CCTCTCCCACGAGAACTCAC	ACGTGGATAAGGGAGCAATG	152	A	A	A	NA
PMS1442	Os_Chr02	35822836	35822857	(CT)11	(CT)11	(CT)6	(CT)11	(CT)11	(CT)11	(CT)11	(CT)11	(CT)11	(CT)11	(CT)11	LOC_Os02g58610	DRR	protein kinase, putative, expressed	-	CCCAACCTTAATTCCTCCATCTGC	CCCATGGCCTTCCAAATTAAGG	118	P	A	A	RM14229

Polymorphic SSR (PMS) markers	Chromosomes	Start physical positions (bp)	End physical positions (bp)	Nipponbare	Kasalath	Nagina22	IR64	Pokkali	Bala	Tainung67	Azucena	Moroberekan	*Oryza rufipogon*	*Oryza nivara*	MSU gene locus IDs	Structural annotation	Functional annotation	SSR functional effects	Forward primers (5'-3')	Reverse primers (5'-3')	Amplified product size (bp)	Markers exhibiting polymorphism within *indica* rice	Transcrption factor genes-derived markers	Known cloned genes-derived markers	PMS markers corresponding with RM markers
PMS1443	Os_Chr02	35930096	35930111	(TA)8	(TA)8	(TA)8	(TA)7	(TA)7	(TA)7	(TA)8	(TA)8	(TA)8	(TA)8	(TA)8	-	Introns/Intergenic	-	-	CTTGCTTCACCTCATCGACA	TTGTTCATCGCGGTAACTCC	273	P	A	A	NA
PMS1444	Os_Chr03	121365	121408	(ATAG)11	(ATAG)11	(ATAG)11	(ATAG)11	(ATAG)11	(ATAG)11	(ATAG)11	(ATAG)11	(ATAG)11	(ATAG)11	(ATAG)6	-	Introns/Intergenic	-	-	GCTCAAAGAATGACACCGATGC	CATGGTGAGTTTGGAGTGATTGG	184	A	A	A	RM14240
PMS1445	Os_Chr03	125890	125913	(TGTC)6	(TGTC)6	(TGTC)5	(TGTC)6	(TGTC)6	(TGTC)5	(TGTC)6	(TGTC)6	(TGTC)6	(TGTC)6	(TGTC)6	-	Introns/Intergenic	-	-	GGGTTCCAAACACATCAATTCC	GCATTGAGGCCAATGTATGTAGG	95	P	A	A	RM14241
PMS1446	Os_Chr03	135780	135849	(TA)35	(TA)35	(TA)35	(TA)35	(TA)9	(TA)35	(TA)6	(TA)35	(TA)10	(TA)35	(TA)35	-	Introns/Intergenic	-	-	CAATTTCAAACCAGGACTGACAGG	ACACAGAAGCAGATCGGAGAACG	302	P	A	A	RM14243
PMS1447	Os_Chr03	139316	139365	(AT)25	(AT)25	(AT)25	(AT)14	(AT)25	(AT)7	(AT)25	(AT)25	(AT)25	(AT)25	(AT)25	-	Introns/Intergenic	-	-	TGAGCTAGCGTTGAGTTCTTAGCC	TGGCTTAACCTGCACTTTCTTCC	380	P	A	A	RM14244
PMS1448	Os_Chr03	192627	192648	(AT)11	(AT)11	(AT)11	(AT)11	(AT)9	(AT)11	(AT)11	(AT)11	(AT)11	(AT)11	(AT)11	-	Introns/Intergenic	-	-	TTCTTCATCACGCACCACCAACG	ACCATGGAGTCCTGCGGTTCG	397	P	A	A	RM14248
PMS1449	Os_Chr03	197253	197270	(TTG)6	(TTG)6	(TTG)6	(TTG)6	(TTG)6	(TTG)6	(TTG)6	(TTG)6	(TTG)6	(TTG)6	(TTG)7	-	Introns/Intergenic	-	-	CCTCCATCATCCACTCCAAT	TAGCAGTAGCCAAGCAAGCA	133	A	A	A	NA
PMS1450	Os_Chr03	216315	216437	(TAT)41	(TAT)41	(TAT)41	(TAT)12	(TAT)14	(TAT)41	(TAT)41	(TAT)41	(TAT)19	(TAT)41	(TAT)39	-	Introns/Intergenic	-	-	AGGAGGGATTACTGCCGATT	CATACGGATCGATCAGCACAT	263	P	A	A	NA
PMS1451	Os_Chr03	303011	303030	(GT)10	(GT)10	(GT)10	(GT)10	(GT)10	(GT)10	(GT)10	(GT)10	(GT)10	(GT)10	(GT)8	-	Introns/Intergenic	-	-	GCGATCGTGCTTGGTAATTT	CACAGGTTCAATGCAACCAC	208	A	A	A	NA
PMS1452	Os_Chr03	374213	374237	(AGGAG)5	(AGGAG)5	(AGGAG)5	(AGGAG)5	(AGGAG)6	(AGGAG)5	(AGGAG)5	(AGGAG)5	(AGGAG)5	(AGGAG)5	(AGGAG)5	-	Introns/Intergenic	-	-	AATCCACAAGGCAATTCAGC	ACAACCTCCTCACCCTCCTC	225	P	A	A	NA
PMS1453	Os_Chr03	387763	387798	(TA)18	(TA)18	(TA)18	(TA)7	(TA)18	(TA)18	(TA)6	(TA)10	(TA)16	(TA)18	(TA)18	LOC_Os03g01610	DRR	expansin precursor, putative, expressed	-	GCAAGGAAGAAACCAGCGTA	TTGTGGGCAAAAACAAATGA	271	P	A	A	NA
PMS1454	Os_Chr03	430621	430638	(AG)9	(AG)9	(AG)9	(AG)9	(AG)9	(AG)9	(AG)9	(AG)9	(AG)9	(AG)9	(AG)10	LOC_Os03g01700	DRR	Lsi2	-	GCCAACTGCCACAGAGAGTT	GCATGTTCCTCAAAATACATGG	280	A	A	P	NA
PMS1455	Os_Chr03	470217	470244	(TTCC)7	(TTCC)7	(TTCC)7	(TTCC)7	(TTCC)7	(TTCC)7	(TTCC)7	(TTCC)7	(TTCC)7	(TTCC)6	(TTCC)7	-	Introns/Intergenic	-	-	AAGAAGAGGAAGCTGTGGGTCAGG	ATGTGATGGGAAATGGAGAAGACG	124	A	A	A	RM14272

Polymorphic SSR (PMS) markers	Chromosomes	Start physical positions (bp)	End physical positions (bp)	Nipponbare	Kasalath	Nagina22	IR64	Pokkali	Bala	Tainung67	Azucena	Moroberekan	*Oryza rufipogon*	*Oryza nivara*	MSU gene locus IDs	Structural annotation	Functional annotation	SSR functional effects	Forward primers (5'-3')	Reverse primers (5'-3')	Amplified product size (bp)	Markers exhibiting polymorphism within *indica* rice	Transcrption factor genes-derived markers	Known cloned genes-derived markers	PMS markers corresponding with RM markers
PMS1456	Os_Chr03	479380	479400	(TCC)7	(TCC)7	(TCC)7	(TCC)7	(TCC)7	(TCC)7	(TCC)7	(TCC)7	(TCC)7	(TCC)7	(TCC)5	LOC_Os03g01760	CDS	exostosin family domain containing protein, expressed	CODON_DELETION	ATGAGAGGAGATCCCAAGCA	CGTAGACGTAGATCCGGAGG	232	A	A	A	NA
PMS1457	Os_Chr03	485171	485184	(CT)7	(CT)7	(CT)7	(CT)7	(CT)7	(CT)7	(CT)6	(CT)7	(CT)7	(CT)7	(CT)7	-	Introns/Intergenic	-	-	TGCTTGCAGCTTCCAAGTAG	TGTGACGACGAAGACCATGT	193	A	A	A	NA
PMS1458	Os_Chr03	485364	485381	(CAG)6	(CAG)6	(CAG)6	(CAG)5	(CAG)5	(CAG)6	(CAG)6	(CAG)6	(CAG)6	(CAG)5	(CAG)5	LOC_Os03g01780	CDS	transposon protein, putative, unclassified, expressed	CODON_DELETION	ACGCAGCTGGTTGAAGAAGT	ACATTGTGAAACGGAGGGAG	138	P	A	A	NA
PMS1459	Os_Chr03	504216	504227	(AT)6	(AT)6	(AT)6	(AT)6	(AT)6	(AT)8	(AT)6	(AT)6	(AT)6	(AT)6	(AT)6	-	Introns/Intergenic	-	-	CGTTCAATTTCGTTCCTGCT	TACAGAGACGCAGCGAGAGA	210	P	A	A	NA
PMS1460	Os_Chr03	510228	510251	(GGC)8	(GGC)8	(GGC)5	(GGC)8	(GGC)8	(GGC)8	(GGC)6	(GGC)8	(GGC)8	(GGC)8	(GGC)8	LOC_Os03g01840	CDS	knotted 1-binding protein 36, putative, expressed	CODON_DELETION	GAGGGAGAGAGCAGTAGCCAGTAGG	TAATTCCGTTCCGAGCAGATGG	143	P	A	A	RM14276
PMS1461	Os_Chr03	514943	514960	(CT)9	(CT)8	(CT)9	(CT)9	(CT)9	(CT)9	(CT)9	(CT)9	(CT)9	(CT)9	(CT)9	-	Introns/Intergenic	-	-	TTTGGTATTTGGCATTTGCT	TGTGTAACAGCAACGAAGGG	247	P	A	A	NA
PMS1462	Os_Chr03	536562	536589	(TA)14	(TA)14	(TA)7	(TA)14	(TA)14	(TA)14	(TA)14	(TA)14	(TA)7	(TA)14	(TA)14	-	Introns/Intergenic	-	-	CATGCATGCAACTCTGCTAAACG	ATCAGGATCCAGGAAATCGAACC	231	P	A	A	RM4108
PMS1463	Os_Chr03	651035	651054	(TC)10	(TC)10	(TC)8	(TC)8	(TC)8	(TC)8	(TC)10	(TC)10	(TC)10	(TC)10	(TC)10	-	Introns/Intergenic	-	-	GTACCGCAGCTTGATCCCTAGC	CCCAAATAGCGTGACAAAGTGC	191	P	A	A	RM14288
PMS1464	Os_Chr03	752947	752967	(TCG)7	(TCG)7	(TCG)7	(TCG)7	(TCG)7	(TCG)7	(TCG)7	(TCG)7	(TCG)7	(TCG)6	(TCG)7	LOC_Os03g02240	CDS	AT-GTL1, putative, expressed	CODON_DELETION	TCGTACTGCACCTGCATCTTGC	ATGAACCACAACTACGGCAACG	140	A	P	A	RM14292
PMS1465	Os_Chr03	755642	755659	(CCG)6	(CCG)6	(CCG)6	(CCG)6	(CCG)6	(CCG)6	(CCG)6	(CCG)6	(CCG)6	(CCG)5	(CCG)6	LOC_Os03g02240	CDS	AT-GTL1, putative, expressed	CODON_DELETION	ATCTCCGACCGGATCTTGA	TGTTAATTTGTTGGCATGCAG	260	A	P	A	NA
PMS1466	Os_Chr03	758787	758806	(TA)10	(TA)10	(TA)10	(TA)10	(TA)10	(TA)10	(TA)10	(TA)10	(TA)10	(TA)6	(TA)10	-	Introns/Intergenic	-	-	TCAGAAGATCCTCAAAGGCTACTGG	AACAGATCACTGTTGCCTTCACC	115	A	A	A	RM14296
PMS1467	Os_Chr03	760737	760764	(TC)14	(TC)14	(TC)13	(TC)14	(TC)14	(TC)13	(TC)14	(TC)14	(TC)14	(TC)14	(TC)14	-	Introns/Intergenic	-	-	GCACAGTGTACATTGCTGATCG	GTACAACATGCCTGCCTTTACG	277	P	A	A	RM14297
PMS1468	Os_Chr03	842748	842759	(TC)6	(TC)6	(TC)8	(TC)8	(TC)6	(TC)8	(TC)6	(TC)8	(TC)8	(TC)6	(TC)7	LOC_Os03g02380	URR	major facilitator superfamily domain-containing protein 5, putative, expressed	-	TATAAATCCCACCGGCTTGA	TAGAACACCTCCATGGCTCC	112	P	A	A	NA

Polymorphic SSR (PMS) markers	Chromosomes	Start physical positions (bp)	End physical positions (bp)	Nipponbare	Kasalath	Nagina22	IR64	Pokkali	Bala	Tainung67	Azucena	Moroberekan	*Oryza rufipogon*	*Oryza nivara*	MSU gene locus IDs	Structural annotation	Functional annotation	SSR functional effects	Forward primers (5'-3')	Reverse primers (5'-3')	Amplified product size (bp)	Markers exhibiting polymorphism within *indica* rice	Transcrption factor genes-derived markers	Known cloned genes-derived markers	PMS markers corresponding with RM markers
PMS1469	Os_Chr03	870761	870781	(CCA)7	(CCA)7	(CCA)7	(CCA)7	(CCA)7	(CCA)7	(CCA)5	(CCA)7	(CCA)7	(CCA)7	(CCA)7	LOC_Os03g02440	URR	WD repeat-containing protein 44, putative, expressed	-	CAACCGATCCCAGAGATCAAGG	GCAAGAGAGGGAAGAGGAAGAGG	87	A	A	A	RM14302
PMS1470	Os_Chr03	921750	921763	(GC)7	(GC)7	(GC)7	(GC)7	(GC)7	(GC)7	(GC)7	(GC)7	(GC)7	(GC)7	(GC)6	-	Introns/Intergenic	-	-	AGATCTCAGGCGAAGGTGTG	CCCTGTTGACCAGTTGAGGT	139	A	A	A	NA
PMS1471	Os_Chr03	1125828	1125839	(CT)6	(CT)6	(CT)6	(CT)6	(CT)6	(CT)6	(CT)6	(CT)7	(CT)6	(CT)6	(CT)6	-	Introns/Intergenic	-	-	GCCATGTTTAGTTGCTCCAAA	TGGTGCATAGATGCATGGTAA	215	A	A	A	NA
PMS1472	Os_Chr03	1153906	1153932	(GCC)9	(GCC)11	(GCC)9	(GCC)9	(GCC)9	(GCC)6	(GCC)6	(GCC)6	(GCC)11	(GCC)9	(GCC)5	LOC_Os03g02900	CDS	B3 DNA binding domain containing protein, expressed	CODON_DELETION	GGGAGGCTCTTCATCGACTGG	GAGGAGGGAGGCAGGAGAAACC	179	P	P	A	RM14310
PMS1473	Os_Chr03	1397232	1397287	(TA)28	(TA)28	(TA)8	(TA)6	(TA)6	(TA)28	(TA)6	(TA)28	(TA)6	(TA)28	(TA)28	-	Introns/Intergenic	-	-	CAATTTGCTATGGACGGTTGTTCG	AATGTGGTCCGGGAGAACAATGC	104	P	A	A	RM4853
PMS1474	Os_Chr03	1479930	1479943	(AG)7	(AG)7	(AG)7	(AG)7	(AG)7	(AG)6	(AG)7	(AG)7	(AG)7	(AG)7	(AG)7	-	Introns/Intergenic	-	-	ATCACCAGGAGAACCGACAC	AGATCCGGCAGTGTGGAG	174	P	A	A	NA
PMS1475	Os_Chr03	1511453	1511473	(TTC)7	(TTC)6	(TTC)7	(TTC)7	(TTC)7	(TTC)7	(TTC)7	(TTC)7	(TTC)7	(TTC)7	(TTC)7	LOC_Os03g03500	URR	heavy metal-associated domain containing protein, expressed	-	GCCGCTGCTTCTGATCATCTCC	ATACGAACGCTCGCAATGTGTAACG	233	P	P	A	RM14328
PMS1476	Os_Chr03	1512288	1512308	(GCC)7	(GCC)7	(GCC)7	(GCC)7	(GCC)7	(GCC)7	(GCC)6	(GCC)7	(GCC)7	(GCC)7	(GCC)7	LOC_Os03g03500	CDS	heavy metal-associated domain containing protein, expressed	CODON_DELETION	GATGGGAAGAAGGAAGGTGA	ATTCGATCAGCAAATGGCAC	279	A	P	A	NA
PMS1477	Os_Chr03	1520616	1520651	(CT)18	(CT)18	(CT)18	(CT)18	(CT)18	(CT)18	(CT)18	(CT)18	(CT)18	(CT)13	(CT)18	-	Introns/Intergenic	-	-	GTTGACAAACAGGCCCTGAAACC	GTATGGTCTGGAGACGACGATCC	179	A	A	A	RM22
PMS1478	Os_Chr03	1705726	1705746	(AGC)7	(AGC)5	(AGC)7	(AGC)5	(AGC)5	(AGC)7	(AGC)7	(AGC)7	(AGC)7	(AGC)7	(AGC)5	-	Introns/Intergenic	-	-	TAGTCTCGCAAGCAGAAGCA	GCCAACGAAACAGTGTGAGA	233	P	A	A	NA
PMS1479	Os_Chr03	1765973	1765996	(CTT)8	(CTT)6	(CTT)8	(CTT)8	(CTT)8	(CTT)6	(CTT)8	(CTT)8	(CTT)8	(CTT)8	(CTT)8	LOC_Os03g03880	URR	protein kinase, putative, expressed	-	CTCTCCTCTCGCTCGCTGTTCC	GAGTGGTAATTGGGCTTGGATGG	177	P	A	A	RM6297
PMS1480	Os_Chr03	1804615	1804632	(CCT)6	(CCT)6	(CCT)6	(CCT)6	(CCT)6	(CCT)6	(CCT)6	(CCT)6	(CCT)6	(CCT)5	(CCT)6	-	Introns/Intergenic	-	-	TTCCTCCCTTCATCAATTCG	AAACCACGGAGAGTCAGAGC	148	A	A	A	NA
PMS1481	Os_Chr03	1850913	1850948	(AT)18	(AT)18	(AT)18	(AT)11	(AT)18	(AT)18	(AT)18	(AT)18	(AT)18	(AT)18	(AT)18	-	Introns/Intergenic	-	-	GTATACTACCCGAATTGTCACG	GATCTGTGAGTTTGGGATGG	189	P	A	A	RM14345

Polymorphic SSR (PMS) markers	Chromosomes	Start physical positions (bp)	End physical positions (bp)	Nipponbare	Kasalath	Nagina22	IR64	Pokkali	Bala	Tainung67	Azucena	Moroberekan	*Oryza rufipogon*	*Oryza nivara*	MSU gene locus IDs	Structural annotation	Functional annotation	SSR functional effects	Forward primers (5'-3')	Reverse primers (5'-3')	Amplified product size (bp)	Markers exhibiting polymorphism within *indica* rice	Transcrption factor genes-derived markers	Known cloned genes-derived markers	PMS markers corresponding with RM markers
PMS1482	Os_Chr03	1909279	1909310	(GA)16	(GA)16	(GA)16	(GA)16	(GA)16	(GA)14	(GA)16	(GA)16	(GA)16	(GA)16	(GA)16	LOC_Os03g04169	URR	ATP phosphoribosyltransferase, putative, expressed	-	GCTGCGTCAGATTTCTCCTC	GACATTCTCGCTTGCTCCTC	239	P	A	A	NA
PMS1483	Os_Chr03	1938583	1938600	(TGC)6	(TGC)5	(TGC)6	(TGC)5	(TGC)5	(TGC)5	(TGC)6	(TGC)6	(TGC)6	(TGC)6	(TGC)6	LOC_Os03g04220	CDS	glutathione S-transferase, putative, expressed	CODON_DELETION	ATCGTCGATATGCCCATGTT	GGAGTACGTCAAGAGCCTGC	191	P	A	A	NA
PMS1484	Os_Chr03	2089800	2089820	(TGG)7	(TGG)7	(TGG)6	(TGG)7	(TGG)7	(TGG)7	(TGG)7	(TGG)7	(TGG)7	(TGG)7	(TGG)7	-	Introns/Intergenic	-	-	TTTGCACTGGTCTCTCTAGTTGG	TCTAAACGATTGGGAGATGAGG	97	P	A	A	RM14363
PMS1485	Os_Chr03	2165654	2165701	(TA)24	(TA)24	(TA)24	(TA)24	(TA)24	(TA)24	(TA)24	(TA)12	(TA)24	(TA)8	(TA)6	-	Introns/Intergenic	-	-	CACGCTGGAGATGGATTGTA	CGTGTTAGTTTGAGATGATCGG	280	A	A	A	NA
PMS1486	Os_Chr03	2183970	2183981	(TC)6	(TC)6	(TC)6	(TC)6	(TC)6	(TC)8	(TC)6	(TC)6	(TC)6	(TC)6	(TC)6	-	Introns/Intergenic	-	-	ACGACCATCCTCCTACTCCC	TGAATCCATGGCAACACACT	230	P	A	A	NA
PMS1487	Os_Chr03	2301947	2301964	(AT)9	(AT)9	(AT)9	(AT)9	(AT)9	(AT)9	(AT)7	(AT)9	(AT)9	(AT)9	(AT)9	-	Introns/Intergenic	-	-	ATGCACAGTTGACTCGCTTG	TCAATTTCAACAATGGTGCC	169	A	A	A	NA
PMS1488	Os_Chr03	2316559	2316572	(CG)7	(CG)7	(CG)7	(CG)7	(CG)7	(CG)7	(CG)7	(CG)7	(CG)7	(CG)6	(CG)7	-	Introns/Intergenic	-	-	GGTCCACCGGACTCTCTCTC	GAAAAGGAAGGAGGGAGGAA	156	A	A	A	NA
PMS1489	Os_Chr03	2412537	2412557	(CTG)7	(CTG)6	(CTG)7	(CTG)7	(CTG)7	(CTG)7	(CTG)7	(CTG)7	(CTG)7	(CTG)7	(CTG)7	LOC_Os03g04980	CDS	PHD-finger domain containing protein, putative, expressed	CODON_CHANGE_PLUS_CODON_DELETION	GGTTGATGCTCATATCCAACAGC	ACAGTAGCAGCAACAGGCGTACC	253	P	A	A	RM14375
PMS1490	Os_Chr03	2432850	2432879	(CCT)10	(CCT)10	(CCT)10	(CCT)10	(CCT)10	(CCT)5	(CCT)10	(CCT)10	(CCT)10	(CCT)10	(CCT)10	-	Introns/Intergenic	-	-	AGTAGGTGGCTGCATTGGTTTGC	GTGGCTTACCTCGAGGGAGAAGG	220	P	A	A	RM14378
PMS1491	Os_Chr03	2700645	2700662	(GTG)6	(GTG)6	(GTG)6	(GTG)6	(GTG)6	(GTG)6	(GTG)6	(GTG)6	(GTG)6	(GTG)5	(GTG)6	-	Introns/Intergenic	-	-	AATCGAAGTCAGGAGGGGAC	CATCCGCCTCTCGTAGTCTC	244	A	A	A	NA
PMS1492	Os_Chr03	2869512	2869565	(TTA)18	(TTA)18	(TTA)18	(TTA)18	(TTA)18	(TTA)18	(TTA)5	(TTA)18	(TTA)18	(TTA)18	(TTA)18	-	Introns/Intergenic	-	-	TTGGGTTCAACTGGAACTTTGG	GCTCAAATCCTACTGCTGATTTGC	183	A	A	A	RM14396
PMS1493	Os_Chr03	2880419	2880451	(GAA)11	(GAA)7	(GAA)11	(GAA)11	(GAA)11	(GAA)7	(GAA)11	(GAA)11	(GAA)11	(GAA)11	(GAA)11	LOC_Os03g05770	DRR	peroxidase precursor, putative, expressed	-	CGATGAAGAGCCAATCCTTC	CCCTTCTACAAACAGGCGAG	190	P	A	A	NA
PMS1494	Os_Chr03	2934883	2934900	(CGT)6	(CGT)6	(CGT)6	(CGT)6	(CGT)6	(CGT)6	(CGT)5	(CGT)6	(CGT)6	(CGT)6	(CGT)6	LOC_Os03g05840	CDS	FAD dependent oxidoreductase domain containing protein, expressed	CODON_CHANGE_PLUS_CODON_DELETION	GCCTGTGGCTAAAGAAGCTG	TGCCAACACAGAGAGCAGTC	247	A	A	A	NA

Polymorphic SSR (PMS) markers	Chromosomes	Start physical positions (bp)	End physical positions (bp)	Nipponbare	Kasalath	Nagina22	IR64	Pokkali	Bala	Tainung67	Azucena	Moroberekan	*Oryza rufipogon*	*Oryza nivara*	MSU gene locus IDs	Structural annotation	Functional annotation	SSR functional effects	Forward primers (5'-3')	Reverse primers (5'-3')	Amplified product size (bp)	Markers exhibiting polymorphism within *indica* rice	Transcrption factor genes-derived markers	Known cloned genes-derived markers	PMS markers corresponding with RM markers
PMS1495	Os_Chr03	3014715	3014728	(CT)7	(CT)9	(CT)7	(CT)7	(CT)7	(CT)7	(CT)7	(CT)7	(CT)7	(CT)7	(CT)7	-	Introns/Intergenic	-	-	AATTCCTCTCGGCCCATCT	GTTGTGACGACGGCGATG	267	P	A	A	NA
PMS1496	Os_Chr03	3052005	3052022	(TGG)6	(TGG)5	(TGG)6	(TGG)6	(TGG)6	(TGG)5	(TGG)6	(TGG)6	(TGG)6	(TGG)6	(TGG)5	-	Introns/Intergenic	-	-	TTTGGAAGAGGAGAGGAGGG	TGATAGCTTATCGTGACAGCG	260	P	A	A	NA
PMS1497	Os_Chr03	3104528	3104545	(GGC)6	(GGC)6	(GGC)5	(GGC)6	(GGC)6	(GGC)6	(GGC)5	(GGC)6	(GGC)6	(GGC)6	(GGC)6	LOC_Os03g06190	CDS	3-5 exonuclease family protein, putative, expressed	CODON_CHANGE_PLUS_CODON_DELETION	GTAAGAAGAAGAAGCGGCCC	ATCGTGGAAAGACACCTTCG	131	P	A	A	NA
PMS1498	Os_Chr03	3222516	3222565	(TA)25	(TA)25	(TA)25	(TA)16	(TA)12	(TA)25	(TA)25	(TA)25	(TA)25	(TA)25	(TA)9	-	Introns/Intergenic	-	-	GTGACTCGTCACTCACGCAT	ATTAGATACCATGGTGCCCG	271	P	A	A	NA
PMS1499	Os_Chr03	3253853	3253870	(CCG)6	(CCG)6	(CCG)6	(CCG)6	(CCG)6	(CCG)6	(CCG)6	(CCG)6	(CCG)6	(CCG)6	(CCG)5	LOC_Os03g06490	CDS	expressed protein	CODON_DELETION	TTGATTTGGTCCTCCTCGTC	AGCAAACCAAACGTACGACC	251	A	A	A	NA
PMS1500	Os_Chr03	3311725	3311745	(GCG)7	(GCG)7	(GCG)7	(GCG)7	(GCG)7	(GCG)7	(GCG)7	(GCG)5	(GCG)7	(GCG)7	(GCG)7	LOC_Os03g06570	CDS	IQ calmodulin-binding motif family protein, putative, expressed	CODON_CHANGE_PLUS_CODON_DELETION	TGTGCTGCCCGAGCTACATGG	CGGAAGTCGTAACTCACCTCGAATCC	337	A	A	A	RM14425
PMS1501	Os_Chr03	3404399	3404416	(AT)9	(AT)9	(AT)9	(AT)9	(AT)9	(AT)9	(AT)7	(AT)9	(AT)9	(AT)9	(AT)9	-	Introns/Intergenic	-	-	GTTTTATACCTTGGAGCCCG	ACAGCCTTAGCAGCCAAAGA	126	A	A	A	NA
PMS1502	Os_Chr03	3461029	3461042	(GC)7	(GC)7	(GC)7	(GC)8	(GC)7	(GC)7	(GC)7	(GC)7	(GC)7	(GC)9	(GC)7	-	Introns/Intergenic	-	-	AATTAAGTTGATGCCTGCCG	AATTCTCAGCCCAGTTCCCT	228	P	A	A	NA
PMS1503	Os_Chr03	3550654	3550668	(GCG)5	(GCG)6	(GCG)5	(GCG)6	(GCG)5	(GCG)6	(GCG)5	(GCG)5	(GCG)5	(GCG)5	(GCG)5	LOC_Os03g06950	CDS	ubiquitin carboxyl-terminal hydrolase domain containing protein, expressed	CODON_INSERTION	GAGGGGAGGGAGGAGTAGG	CGATTCGATTGCCATTTTCT	133	P	A	A	NA
PMS1504	Os_Chr03	3644940	3644960	(AAT)7	(AAT)7	(AAT)7	(AAT)6	(AAT)6	(AAT)7	(AAT)7	(AAT)7	(AAT)7	(AAT)7	(AAT)7	-	Introns/Intergenic	-	-	GTTCCATGGATCTCTCGTTTGG	CAACTACTACAGTTGAACTCCCAAGG	200	P	A	A	RM14446
PMS1505	Os_Chr03	3669987	3670016	(TA)15	(TA)15	(TA)15	(TA)15	(TA)6	(TA)7	(TA)15	(TA)15	(TA)15	(TA)15	(TA)15	-	Introns/Intergenic	-	-	GGAATCTGGTTGGTGGATTTAAGG	CCACAGCAACAGAGTTCAAAGG	331	P	A	A	RM14448
PMS1506	Os_Chr03	3783479	3783500	(AT)11	(AT)12	(AT)6	(AT)11	(AT)11	(AT)11	(AT)11	(AT)11	(AT)11	(AT)11	(AT)11	-	Introns/Intergenic	-	-	CCAGTACGTACGACACACTGAGC	TCGAAACAATTGGTCCAAGC	401	P	A	A	RM14454
PMS1507	Os_Chr03	3834130	3834143	(GA)7	(GA)7	(GA)7	(GA)7	(GA)7	(GA)7	(GA)6	(GA)7	(GA)7	(GA)7	(GA)7	LOC_Os03g07530	URR	OsFBK12	-	GAGCCAAAACAGACGGAGAG	TACTGCTGCCCGCTTCTAAT	252	A	A	P	NA

Polymorphic SSR (PMS) markers	Chromosomes	Start physical positions (bp)	End physical positions (bp)	Nipponbare	Kasalath	Nagina22	IR64	Pokkali	Bala	Tainung67	Azucena	Moroberekan	*Oryza rufipogon*	*Oryza nivara*	MSU gene locus IDs	Structural annotation	Functional annotation	SSR functional effects	Forward primers (5'-3')	Reverse primers (5'-3')	Amplified product size (bp)	Markers exhibiting polymorphism within *indica* rice	Transcrption factor genes-derived markers	Known cloned genes-derived markers	PMS markers corresponding with RM markers
PMS1508	Os_Chr03	3881803	3881818	(AT)8	(AT)7	(AT)8	(AT)8	(AT)8	(AT)7	(AT)8	(AT)8	(AT)8	(AT)8	(AT)7	-	Introns/Intergenic	-	-	TGGCTCTATTACTCCCTCCG	TTTTCATCTACCTCCGTTTTAGG	273	P	A	A	NA
PMS1509	Os_Chr03	3904160	3904175	(TA)8	(TA)11	(TA)8	(TA)8	(TA)8	(TA)8	(TA)8	(TA)8	(TA)8	(TA)8	(TA)8	-	Introns/Intergenic	-	-	GCTAGCGAGCAAATAAATTGT	TTGCTGCATGGTTGATTAT	327	P	A	A	NA
PMS1510	Os_Chr03	3934938	3934993	(GA)28	(GA)28	(GA)28	(GA)6	(GA)6	(GA)28	(GA)28	(GA)28	(GA)28	(GA)6	(GA)28	-	Introns/Intergenic	-	-	ACGTCACAACAGCACCTCATTCC	CTGTGGCATATGTTCGATTCAGG	226	P	A	A	RM14473
PMS1511	Os_Chr03	3989930	3989953	(GCG)8	(GCG)8	(GCG)8	(GCG)8	(GCG)8	(GCG)8	(GCG)8	(GCG)8	(GCG)8	(GCG)8	(GCG)5	LOC_Os03g07840	CDS	adenylosuccinate synthetase, chloroplast precursor, putative, expressed	CODON_DELETION	AGCCATCAAGCATGGATTAGAGC	ATGTCGCTCTCCACTGTCAACC	358	A	A	A	RM14476
PMS1512	Os_Chr03	4072146	4072157	(GA)6	(GA)6	(GA)6	(GA)7	(GA)7	(GA)6	(GA)7	(GA)7	(GA)7	(GA)6	(GA)6	-	Introns/Intergenic	-	-	CCCCTGCAACATCAAAAGTT	CGACGATTCTGCCAGGTC	239	P	A	A	NA
PMS1513	Os_Chr03	4230200	4230219	(CG)10	(CG)10	(CG)10	(CG)9	(CG)10	(CG)10	(CG)10	(CG)10	(CG)10	(CG)10	(CG)10	-	Introns/Intergenic	-	-	GATCGATGAGCGAGATCGAC	ATCTGCATATGGCATCCTCC	266	P	A	A	NA
PMS1514	Os_Chr03	4244239	4244250	(CT)6	(CT)9	(CT)6	(CT)6	(CT)6	(CT)6	(CT)6	(CT)6	(CT)6	(CT)6	(CT)6	-	Introns/Intergenic	-	-	ATCCCGAATCCAAATCCTTC	GAGAAGGAGAGCGAGGGG	270	P	A	A	NA
PMS1515	Os_Chr03	4256114	4256191	(ATA)26	(ATA)26	(ATA)26	(ATA)26	(ATA)18	(ATA)26	(ATA)26	(ATA)21	(ATA)26	(ATA)26	(ATA)9	-	Introns/Intergenic	-	-	AATATGCTCGTGCGCTGC	TCCTCAATTCTTGGTTCCATC	280	P	A	A	NA
PMS1516	Os_Chr03	4301155	4301194	(AT)20	(AT)20	(AT)8	(AT)20	(AT)20	(AT)16	(AT)20	(AT)20	(AT)20	(AT)20	(AT)20	-	Introns/Intergenic	-	-	TGGTCATTGGTACAACACATCC	AAACAGGAGCAAGACACTGAAGC	177	P	A	A	RM14486
PMS1517	Os_Chr03	4306303	4306318	(TA)8	(TA)8	(TA)8	(TA)8	(TA)6	(TA)8	(TA)8	(TA)8	(TA)8	(TA)8	(TA)8	-	Introns/Intergenic	-	-	CTCTAGATCGATCATCCGGC	GCAATGACGACCTGATTGAA	151	P	A	A	NA
PMS1518	Os_Chr03	4373702	4373721	(TG)10	(TG)10	(TG)10	(TG)10	(TG)10	(TG)10	(TG)9	(TG)9	(TG)9	(TG)10	(TG)9	-	Introns/Intergenic	-	-	TAAAGAAATCGCGGCGAAAGAGG	CTCCACGAGCCCATCTCCTTCC	272	A	A	A	RM14491
PMS1519	Os_Chr03	4420482	4420496	(CGC)5	(CGC)6	(CGC)6	(CGC)6	(CGC)5	(CGC)5	(CGC)5	(CGC)5	(CGC)5	(CGC)5	(CGC)5	-	Introns/Intergenic	-	-	CCCTGCTCTTGTCGCTCTC	TTCTTTGGCATCATCCTTCC	270	P	A	A	NA
PMS1520	Os_Chr03	4467502	4467513	(TC)6	(TC)7	(TC)6	(TC)6	(TC)6	(TC)7	(TC)6	(TC)7	(TC)7	(TC)6	(TC)6	-	Introns/Intergenic	-	-	GTCATGCCTCCACTTCACCT	CAATGCTACCTCCTTGCCTC	145	P	A	A	NA

Polymorphic SSR (PMS) markers	Chromosomes	Start physical positions (bp)	End physical positions (bp)	Nipponbare	Kasalath	Nagina22	IR64	Pokkali	Bala	Tainung67	Azucena	Moroberekan	*Oryza rufipogon*	*Oryza nivara*	MSU gene locus IDs	Structural annotation	Functional annotation	SSR functional effects	Forward primers (5'-3')	Reverse primers (5'-3')	Amplified product size (bp)	Markers exhibiting polymorphism within *indica* rice	Transcrption factor genes-derived markers	Known cloned genes-derived markers	PMS markers corresponding with RM markers
PMS1521	Os_Chr03	4482707	4482730	(CT)12	(CT)12	(CT)12	(CT)12	(CT)12	(CT)12	(CT)12	(CT)12	(CT)12	(CT)12	(CT)7	-	Introns/Intergenic	-	-	ACCGGAAGAAAGCCCATCACG	CCGGTAGCGTCTGAGCTGTTGG	199	A	A	A	RM3195
PMS1522	Os_Chr03	4483060	4483077	(GCG)6	(GCG)6	(GCG)6	(GCG)6	(GCG)6	(GCG)5	(GCG)6	(GCG)6	(GCG)6	(GCG)6	(GCG)6	LOC_Os03g08700	CDS	expressed protein	CODON_CHANGE_PLUS_CODON_DELETION	ATGTCCACCTCCAGCGTCT	GCGGAGATGTGGTAGATCGT	244	P	A	A	NA
PMS1523	Os_Chr03	4514030	4514047	(CGG)6	(CGG)5	(CGG)6	(CGG)6	(CGG)6	(CGG)5	(CGG)7	(CGG)6	(CGG)7	(CGG)6	(CGG)5	LOC_Os03g08740	CDS	expressed protein	CODON_CHANGE_PLUS_CODON_INSERTION	GATTTCTTGCAGGGAGTTGC	CAGTCTCTCGACACCACCAA	255	P	A	A	NA
PMS1524	Os_Chr03	4537886	4537935	(TA)25	(TA)25	(TA)25	(TA)10	(TA)25	(TA)25	(TA)6	(TA)6	(TA)6	(TA)9	(TA)22	-	Introns/Intergenic	-	-	CTGCCAGGACGATAGGAGAG	TTTTGAAAAATTTGAGTGATTTTG	217	P	A	A	NA
PMS1525	Os_Chr03	4562534	4562567	(TC)17	(TC)12	(TC)12	(TC)17	(TC)12	(TC)16	(TC)17	(TC)17	(TC)17	(TC)17	(TC)17	-	Introns/Intergenic	-	-	GAATTCAAAGGGCTCACTTCTGC	CCATATAAAGGTGGCACGACAGG	262	P	A	A	RM1278
PMS1526	Os_Chr03	4606747	4606762	(GC)8	(GC)8	(GC)8	(GC)8	(GC)8	(GC)8	(GC)8	(GC)8	(GC)8	(GC)8	(GC)7	-	Introns/Intergenic	-	-	TTAGTTGGCACCAAACACCA	AGCTACAGCTAGGCACGCAC	152	A	A	A	NA
PMS1527	Os_Chr03	4653549	4653566	(GAG)6	(GAG)5	(GAG)5	(GAG)5	(GAG)5	(GAG)5	(GAG)6	(GAG)6	(GAG)6	(GAG)5	(GAG)5	LOC_Os03g08960	CDS	homeobox associated leucine zipper, putative, expressed	CODON_DELETION	GAAAGATGAAGCCGAGATGG	GTCCAGCTTGTTGTCCGTGT	187	A	P	A	NA
PMS1528	Os_Chr03	4664826	4664841	(GA)8	(GA)8	(GA)8	(GA)6	(GA)6	(GA)8	(GA)8	(GA)8	(GA)8	(GA)8	(GA)8	-	Introns/Intergenic	-	-	TCCTTGCCTGGTTGATTTTC	CCCATGGGTCAGTGACAGTT	207	P	A	A	NA
PMS1529	Os_Chr03	4743160	4743173	(TC)7	(TC)7	(TC)7	(TC)7	(TC)7	(TC)7	(TC)7	(TC)7	(TC)7	(TC)10	(TC)7	-	Introns/Intergenic	-	-	AATCCAAATCTTTCCCAAAT	GTGCAACGCACGGGCAGAG	148	A	A	A	NA
PMS1530	Os_Chr03	4829447	4829473	(GCG)9	(GCG)9	(GCG)7	(GCG)9	(GCG)9	(GCG)9	(GCG)9	(GCG)9	(GCG)9	(GCG)9	(GCG)9	LOC_Os03g09250	URR	OsINO1-1|OsINO1	-	CCGTCGTGTCGTACTGGTAGTCC	TCCACAGCCTACCTAGCTTCTCC	194	P	A	P	RM6038
PMS1531	Os_Chr03	4898411	4898432	(TA)11	(TA)11	(TA)11	(TA)11	(TA)11	(TA)11	(TA)11	(TA)11	(TA)7	(TA)11	(TA)11	-	Introns/Intergenic	-	-	CCGGTGGCATAGAAGGTAGA	TTTCTAGCATTGCCAGCATTT	207	A	A	A	NA
PMS1532	Os_Chr03	4975982	4976017	(AT)18	(AT)16	(AT)6	(AT)11	(AT)18	(AT)18	(AT)7	(AT)18	(AT)13	(AT)18	(AT)18	-	Introns/Intergenic	-	-	GCACTTATCCATTTCCAGCTTGC	CCTACTCGTCTACCAACAACAATACG	196	P	A	A	RM14524
PMS1533	Os_Chr03	5014995	5015006	(GT)6	(GT)6	(GT)6	(GT)6	(GT)6	(GT)6	(GT)6	(GT)6	(GT)6	(GT)7	(GT)6	-	Introns/Intergenic	-	-	GCTAGAGCTAATTGGGGGCT	CCGTCCATTGGAGTTGAGTT	203	A	A	A	NA

Polymorphic SSR (PMS) markers	Chromosomes	Start physical positions (bp)	End physical positions (bp)	Nipponbare	Kasalath	Nagina22	IR64	Pokkali	Bala	Tainung67	Azucena	Moroberekan	*Oryza rufipogon*	*Oryza nivara*	MSU gene locus IDs	Structural annotation	Functional annotation	SSR functional effects	Forward primers (5'-3')	Reverse primers (5'-3')	Amplified product size (bp)	Markers exhibiting polymorphism within *indica* rice	Transcrption factor genes-derived markers	Known cloned genes-derived markers	PMS markers corresponding with RM markers
PMS1534	Os_Chr03	5054073	5054086	(AG)7	(AG)7	(AG)7	(AG)7	(AG)7	(AG)7	(AG)7	(AG)6	(AG)7	(AG)7	(AG)7	-	Introns/Intergenic	-	-	GTAGCAGACGCAAGAGAGGG	AAGTTGGCACGGGTATTAAAAA	210	A	A	A	NA
PMS1535	Os_Chr03	5056536	5056547	(TA)6	(TA)7	(TA)7	(TA)7	(TA)7	(TA)7	(TA)6	(TA)6	(TA)6	(TA)6	(TA)7	-	Introns/Intergenic	-	-	AAAACGTCTGCGTTCCAAAC	CGACCCAAAAGAGACTGCTC	244	A	A	A	NA
PMS1536	Os_Chr03	5066144	5066159	(AC)8	(AC)8	(AC)8	(AC)7	(AC)8	(AC)8	(AC)8	(AC)8	(AC)8	(AC)8	(AC)8	-	Introns/Intergenic	-	-	ACATGTGTGTGCAGTTGTGC	TCAAAACTGATGCCGAAGAA	215	P	A	A	NA
PMS1537	Os_Chr03	5183853	5183868	(AG)8	(AG)8	(AG)8	(AG)8	(AG)8	(AG)8	(AG)8	(AG)8	(AG)8	(AG)8	(AG)7	-	Introns/Intergenic	-	-	GTGGGCATTCAAGTTTGGTT	CCCGTTGCTCTTCTCTCTCA	258	A	A	A	NA
PMS1538	Os_Chr03	5282318	5282337	(GA)10	(GA)8	(GA)8	(GA)10	(GA)10	(GA)8	(GA)10	(GA)10	(GA)10	(GA)10	(GA)7	-	Introns/Intergenic	-	-	TGCCATGTGAGAGAATGGATTAGG	AAAGAGTAGCCGTCCTAGTGATTGG	280	P	A	A	RM14543
PMS1539	Os_Chr03	5434590	5434611	(AT)11	(AT)11	(AT)11	(AT)11	(AT)11	(AT)8	(AT)9	(AT)11	(AT)11	(AT)11	(AT)11	-	Introns/Intergenic	-	-	GGACCCAATTGAAAGGGAAAGG	CAGATGAACTAATAAGGCCTGACAGC	403	P	A	A	RM14551
PMS1540	Os_Chr03	5459348	5459368	(CCA)7	(CCA)6	(CCA)7	(CCA)7	(CCA)7	(CCA)7	(CCA)7	(CCA)7	(CCA)7	(CCA)7	(CCA)7	LOC_Os03g10680	CDS	formin-like protein 6 precursor, putative, expressed	CODON_DELETION	AAGTTGAATACAGCGCCGAAACC	GCTTTGGGAGATTGTTGTTTGACG	79	P	A	A	RM14553
PMS1541	Os_Chr03	5496874	5496894	(GGC)7	(GGC)7	(GGC)7	(GGC)7	(GGC)7	(GGC)7	(GGC)7	(GGC)7	(GGC)6	(GGC)7	(GGC)7	LOC_Os03g10770	CDS	helix-loop-helix DNA-binding domain containing protein, expressed	CODON_DELETION	ATCCTTGTGATCCTCAACCTTCC	AGCAGCATTACGGGTGCTACG	227	A	P	A	RM14554
PMS1542	Os_Chr03	5503268	5503323	(TA)28	(TA)28	(TA)7	(TA)6	(TA)28	(TA)7	(TA)28	(TA)28	(TA)12	(TA)7	(TA)28	-	Introns/Intergenic	-	-	TATGTGTGTCCACCTGCGTT	TCATGCCACATCCACTCAAT	257	P	A	A	NA
PMS1543	Os_Chr03	5530617	5530638	(CT)11	(CT)10	(CT)10	(CT)11	(CT)11	(CT)10	(CT)11	(CT)11	(CT)11	(CT)11	(CT)11	LOC_Os03g10800	URR	BTBN4 - Bric-a-Brac, Tramtrack, Broad Complex BTB domain with non-phototropic hypocotyl 3 NPH3 domain, expressed	-	GTTGTCCCTCCTGGCTCCTTGC	GAGGAGCACAAGCACCTCAATGG	94	P	A	A	RM14557
PMS1544	Os_Chr03	5591446	5591466	(GCC)7	(GCC)7	(GCC)7	(GCC)7	(GCC)7	(GCC)7	(GCC)7	(GCC)7	(GCC)7	(GCC)6	(GCC)7	LOC_Os03g10880	CDS	BTBN5 - Bric-a-Brac, Tramtrack, Broad Complex BTB domain with non-phototropic hypocotyl 3 NPH3 and coiled-coil domains, expressed	CODON_DELETION	CGCGCAGAGCTCAGAAACTAACC	GACGGCCAGTTCAGCATGTCG	256	A	A	A	RM14560
PMS1545	Os_Chr03	5641385	5641399	(GGA)5	(GGA)5	(GGA)5	(GGA)5	(GGA)5	(GGA)5	(GGA)5	(GGA)5	(GGA)5	(GGA)6	(GGA)5	LOC_Os03g10980	CDS	expressed protein	CODON_CHANGE_PLUS_CODON_INSERTION	TGCACAAGCACAAGAACACA	TCATCCTCACAGTACGGCAA	264	A	A	A	NA
PMS1546	Os_Chr03	5687206	5687232	(CCG)9	(CCG)8	(CCG)9	(CCG)9	(CCG)9	(CCG)8	(CCG)9	(CCG)9	(CCG)9	(CCG)9	(CCG)9	LOC_Os03g11060	CDS	MFP1 attachment factor 1, putative, expressed	CODON_CHANGE_PLUS_CODON_DELETION	GCTGCAGCTCTACTCCAAGG	AAGCCACATTTCTCCAAACG	238	P	A	A	NA

Polymorphic SSR (PMS) markers	Chromosomes	Start physical positions (bp)	End physical positions (bp)	Nipponbare	Kasalath	Nagina22	IR64	Pokkali	Bala	Tainung67	Azucena	Moroberekan	*Oryza rufipogon*	*Oryza nivara*	MSU gene locus IDs	Structural annotation	Functional annotation	SSR functional effects	Forward primers (5'-3')	Reverse primers (5'-3')	Amplified product size (bp)	Markers exhibiting polymorphism within *indica* rice	Transcrption factor genes-derived markers	Known cloned genes-derived markers	PMS markers corresponding with RM markers
PMS1547	Os_Chr03	5738309	5738330	(TA)11	(TA)11	(TA)11	(TA)11	(TA)6	(TA)11	(TA)9	(TA)7	(TA)11	(TA)11	(TA)11	-	Introns/Intergenic	-	-	TTTGTTGGGAGCTTCTTCATGC	TGCATACCATACAGCATGCTTCG	461	P	A	A	RM14567
PMS1548	Os_Chr03	5755156	5755173	(AT)9	(AT)9	(AT)9	(AT)9	(AT)9	(AT)9	(AT)9	(AT)8	(AT)9	(AT)9	(AT)9	-	Introns/Intergenic	-	-	ATTTGGGGATCGAATTGTTG	CAGAGGATCTGGATTCGACC	271	A	A	A	NA
PMS1549	Os_Chr03	5789097	5789120	(GCG)8	(GCG)8	(GCG)8	(GCG)8	(GCG)8	(GCG)8	(GCG)8	(GCG)7	(GCG)8	(GCG)8	(GCG)8	-	Introns/Intergenic	-	-	AACCCAAAAACCCTACACCC	CCTCTCCTCTCCTCTCCTCC	277	A	A	A	NA
PMS1550	Os_Chr03	5812726	5812741	(TC)8	(TC)7	(TC)8	(TC)8	(TC)8	(TC)7	(TC)8	(TC)8	(TC)8	(TC)8	(TC)7	-	Introns/Intergenic	-	-	AAGAGAGGTGATGGCTTGGA	CTCGACGAAGAGAGGACACC	220	P	A	A	NA
PMS1551	Os_Chr03	5856461	5856488	(CA)14	(CA)12	(CA)12	(CA)14	(CA)14	(CA)12	(CA)14	(CA)14	(CA)14	(CA)14	(CA)12	-	Introns/Intergenic	-	-	TCTCTGCACCCTGTTCACATGC	GCCTTTCCCAATGGAATATCAGG	162	P	A	A	RM3131
PMS1552	Os_Chr03	5872732	5872767	(TG)18	(TG)18	(TG)9	(TG)18	(TG)18	(TG)18	(TG)18	(TG)18	(TG)18	(TG)18	(TG)18	-	Introns/Intergenic	-	-	GGTGTTCCTGTTCCAATTCTTGC	TCAAACTTGCTAGCCAAAGTAGGG	196	P	A	A	RM5570
PMS1553	Os_Chr03	5959864	5959941	(GTA)26	(GTA)26	(GTA)26	(GTA)26	(GTA)26	(GTA)12	(GTA)26	(GTA)26	(GTA)26	(GTA)26	(GTA)26	-	Introns/Intergenic	-	-	CATCAGACACCGGCCTTAAT	GGAATCGTTGCTCTCGTTTC	255	P	A	A	NA
PMS1554	Os_Chr03	5965994	5966023	(AT)15	(AT)15	(AT)15	(AT)9	(AT)15	(AT)15	(AT)15	(AT)15	(AT)15	(AT)15	(AT)15	-	Introns/Intergenic	-	-	CATCATTGTGTTCGTTACAATTAAA	TCGCAAGTAAAGTGTAGAGAAGATTT	277	P	A	A	NA
PMS1555	Os_Chr03	6024348	6024361	(AT)7	(AT)7	(AT)7	(AT)7	(AT)7	(AT)7	(AT)7	(AT)7	(AT)6	(AT)7	(AT)7	-	Introns/Intergenic	-	-	CCATGCCTTCTTGTTTCGAT	GAGGTTGGGACTCGTGTCAT	208	A	A	A	NA
PMS1556	Os_Chr03	6028254	6028268	(TAG)5	(TAG)5	(TAG)5	(TAG)5	(TAG)6	(TAG)5	(TAG)5	(TAG)5	(TAG)5	(TAG)5	(TAG)5	-	Introns/Intergenic	-	-	AGCGCGAAACCTAGTCATGT	GCTTGATGGGATTTGAGGAA	243	P	A	A	NA
PMS1557	Os_Chr03	6036946	6036969	(TCGA)6	(TCGA)6	(TCGA)6	(TCGA)5	(TCGA)6	(TCGA)6	(TCGA)6	(TCGA)6	(TCGA)6	(TCGA)6	(TCGA)6	-	Introns/Intergenic	-	-	GCATGCTCTCCTTAGGCTTATCG	GCACTAAGCTCGGACACACTTGG	278	P	A	A	RM7565
PMS1558	Os_Chr03	6048652	6048671	(TA)10	(TA)10	(TA)10	(TA)10	(TA)9	(TA)10	(TA)10	(TA)10	(TA)10	(TA)10	(TA)10	LOC_Os03g11600	DRR	DL	-	CTCATCGTTCCCCTTTAGCA	AAAACCTGCAAACATGCGTA	279	P	P	P	NA
PMS1559	Os_Chr03	6079300	6079331	(TAGA)8	(TAGA)12	(TAGA)10	(TAGA)10	(TAGA)8	(TAGA)12	(TAGA)10	(TAGA)8	(TAGA)8	(TAGA)8	(TAGA)12	-	Introns/Intergenic	-	-	GTGGGAAGAAGAACATCAACTGG	GCACACAAGATAAACCCAATCAGC	129	P	A	A	RM7576

Polymorphic SSR (PMS) markers	Chromosomes	Start physical positions (bp)	End physical positions (bp)	Nipponbare	Kasalath	Nagina22	IR64	Pokkali	Bala	Tainung67	Azucena	Moroberekan	*Oryza rufipogon*	*Oryza nivara*	MSU gene locus IDs	Structural annotation	Functional annotation	SSR functional effects	Forward primers (5'-3')	Reverse primers (5'-3')	Amplified product size (bp)	Markers exhibiting polymorphism within *indica* rice	Transcrption factor genes-derived markers	Known cloned genes-derived markers	PMS markers corresponding with RM markers
PMS1560	Os_Chr03	6167112	6167141	(AG)15	(AG)15	(AG)15	(AG)15	(AG)15	(AG)15	(AG)15	(AG)6	(AG)15	(AG)15	(AG)15	LOC_Os03g11790	URR	OsFBX79 - F-box domain containing protein, expressed	-	CAGCTCCTTCCTATCCGTCTCC	TCAGATCTAGCCGAGAAATCAAGG	184	A	A	A	RM517
PMS1561	Os_Chr03	6211980	6211993	(CG)7	(CG)7	(CG)6	(CG)7	(CG)7	(CG)7	(CG)7	(CG)7	(CG)7	(CG)7	(CG)7	-	Introns/Intergenic	-	-	CCCTGTTGACCAGTTGAGGT	AATCGTGGAACGTGGGTTAG	173	P	A	A	NA
PMS1562	Os_Chr03	6321259	6321286	(TA)14	(TA)14	(TA)14	(TA)14	(TA)7	(TA)7	(TA)14	(TA)14	(TA)14	(TA)8	(TA)14	-	Introns/Intergenic	-	-	GTGACCCCTCTGTCACGAAT	TCACATCAATTGTTTTTAGTGCTT	222	P	A	A	NA
PMS1563	Os_Chr03	6411900	6411941	(GA)21	(GA)16	(GA)21	(GA)21	(GA)21	(GA)21	(GA)21	(GA)10	(GA)21	(GA)21	(GA)21	-	Introns/Intergenic	-	-	GCGTGAGGGAGAGAGAGG	TTCAGGAGCAGGAGAGAG	300	P	A	A	NA
PMS1564	Os_Chr03	6428704	6428729	(TC)13	(TC)13	(TC)13	(TC)13	(TC)9	(TC)13	(TC)13	(TC)13	(TC)13	(TC)13	(TC)13	-	Introns/Intergenic	-	-	GTGCGCCGGAATTACGCTTCC	GCAGCACGCAGTTCTCCTTCTCC	167	P	A	A	RM5347
PMS1565	Os_Chr03	6477462	6477477	(AG)8	(AG)8	(AG)6	(AG)8	(AG)8	(AG)6	(AG)8	(AG)8	(AG)7	(AG)6	(AG)8	-	Introns/Intergenic	-	-	GTTTTATACCTTGGAGCCCG	TGTTACGGTACCATCGCAAA	253	P	A	A	NA
PMS1566	Os_Chr03	6594498	6594545	(AT)24	(AT)24	(AT)24	(AT)24	(AT)24	(AT)24	(AT)24	(AT)24	(AT)24	(AT)24	(AT)9	-	Introns/Intergenic	-	-	GACCTGGCACGAAGTTCAGAGATGC	CGGGCCGGCACATAAAGAATAGG	467	A	A	A	RM14618
PMS1567	Os_Chr03	6624070	6624097	(GA)14	(GA)14	(GA)14	(GA)14	(GA)14	(GA)14	(GA)14	(GA)14	(GA)14	(GA)13	(GA)14	-	Introns/Intergenic	-	-	CTTGGAGACTAGCACGTAGAGTGG	ATCCCGAATCCAGATCCTTCC	434	A	A	A	RM14622
PMS1568	Os_Chr03	6737063	6737086	(TA)12	(TA)12	(TA)12	(TA)12	(TA)12	(TA)8	(TA)12	(TA)12	(TA)12	(TA)12	(TA)12	-	Introns/Intergenic	-	-	TATCCGCAGGAGACCTAACTTGC	GCAGGATGTGGATGTGAGAAGC	284	P	A	A	RM14626
PMS1569	Os_Chr03	6743029	6743058	(AACTG)6	(AACTG)6	(AACTG)6	(AACTG)6	(AACTG)6	(AACTG)6	(AACTG)5	(AACTG)6	(AACTG)6	(AACTG)6	(AACTG)6	-	Introns/Intergenic	-	-	AAGGACGTGCACTACAAGGG	TCCATCTCCAGGGATTGAAG	221	A	A	A	NA
PMS1570	Os_Chr03	6762601	6762614	(GT)7	(GT)7	(GT)7	(GT)7	(GT)7	(GT)7	(GT)7	(GT)6	(GT)7	(GT)7	(GT)7	-	Introns/Intergenic	-	-	ACCAGCTCCTGCCCCTAC	AATACCGGACGAATTACCCC	200	A	A	A	NA
PMS1571	Os_Chr03	6856327	6856344	(GCG)6	(GCG)6	(GCG)6	(GCG)6	(GCG)6	(GCG)6	(GCG)6	(GCG)6	(GCG)6	(GCG)5	(GCG)6	LOC_Os03g12790	CDS	MATE efflux family protein, putative, expressed	CODON_DELETION	TCACGCCATCAAAATTACCA	GCTTTTTGTGTACTCCCCGA	253	A	A	A	NA
PMS1572	Os_Chr03	7050809	7050824	(AG)8	(AG)11	(AG)8	(AG)8	(AG)8	(AG)11	(AG)8	(AG)8	(AG)8	(AG)8	(AG)8	LOC_Os03g13040	URR	haemolysin-III, putative, expressed	-	AGGATGTACTCGTTGTCCCG	GCATAACACCATTGTCGCC	209	P	A	A	NA

Polymorphic SSR (PMS) markers	Chromosomes	Start physical positions (bp)	End physical positions (bp)	Nipponbare	Kasalath	Nagina22	IR64	Pokkali	Bala	Tainung67	Azucena	Moroberekan	*Oryza rufipogon*	*Oryza nivara*	MSU gene locus IDs	Structural annotation	Functional annotation	SSR functional effects	Forward primers (5'-3')	Reverse primers (5'-3')	Amplified product size (bp)	Markers exhibiting polymorphism within *indica* rice	Transcrption factor genes-derived markers	Known cloned genes-derived markers	PMS markers corresponding with RM markers
PMS1573	Os_Chr03	7143774	7143791	(TA)9	(TA)9	(TA)9	(TA)9	(TA)9	(TA)9	(TA)9	(TA)8	(TA)9	(TA)9	(TA)9	-	Introns/Intergenic	-	-	AAAACGTTCTGGGTTTGTGC	AAAGAACATGTCCCGTCTCG	190	A	A	A	NA
PMS1574	Os_Chr03	7202088	7202099	(GA)6	(GA)6	(GA)6	(GA)6	(GA)8	(GA)6	(GA)6	(GA)6	(GA)6	(GA)6	(GA)6	-	Introns/Intergenic	-	-	TTTGACCAGAGTGGGAGGAC	CTCCTCTGCCTCAGCTCTGT	142	P	A	A	NA
PMS1575	Os_Chr03	7334923	7334940	(TTC)6	(TTC)6	(TTC)6	(TTC)6	(TTC)9	(TTC)6	(TTC)6	(TTC)6	(TTC)6	(TTC)9	(TTC)9	LOC_Os03g13560	URR	hydroxyproline-rich glycoprotein family protein, putative, expressed	-	ATAGAAAGGCGAACCCCAAC	AGTTTCGGGAATCTTCCGTT	239	P	A	A	NA
PMS1576	Os_Chr03	7377244	7377257	(TA)7	(TA)7	(TA)7	(TA)7	(TA)6	(TA)7	(TA)7	(TA)7	(TA)7	(TA)7	(TA)7	-	Introns/Intergenic	-	-	GTTTCGCTCTCCAAATCCACAGG	AGGGATTTGTGCCCTAGGAAGG	591	P	A	A	RM14657
PMS1577	Os_Chr03	7389863	7389888	(GA)13	(GA)13	(GA)13	(GA)13	(GA)13	(GA)13	(GA)13	(GA)13	(GA)13	(GA)13	(GA)12	-	Introns/Intergenic	-	-	CATTCTCTCCACTCCATCTCC	TCTTCTTCCTCTCTTTCTCTCG	294	A	A	A	RM14658
PMS1578	Os_Chr03	7404427	7404454	(TA)14	(TA)12	(TA)14	(TA)14	(TA)14	(TA)14	(TA)14	(TA)14	(TA)8	(TA)14	(TA)14	-	Introns/Intergenic	-	-	ACCTGGGAGTACAGAAGAGTCG	GGACATGTGGATGTATCTGATCG	147	P	A	A	RM14659
PMS1579	Os_Chr03	7417106	7417127	(AT)11	(AT)11	(AT)6	(AT)11	(AT)11	(AT)11	(AT)11	(AT)11	(AT)11	(AT)11	(AT)10	-	Introns/Intergenic	-	-	CGAACGGATGGTGGATGAAACTAGG	ATATTCAGTCCGAGCGAGCGAACG	154	P	A	A	RM14661
PMS1580	Os_Chr03	7449906	7449920	(GAG)5	(GAG)6	(GAG)6	(GAG)6	(GAG)6	(GAG)5	(GAG)5	(GAG)5	(GAG)5	(GAG)6	(GAG)6	LOC_Os03g13760	URR	LSM domain containing protein, expressed	-	TCCAAGACAAAACCGGAGAG	GTCTTGAGCTCCACCGTCAC	261	P	A	A	NA
PMS1581	Os_Chr03	7467903	7467920	(TCC)6	(TCC)6	(TCC)6	(TCC)6	(TCC)6	(TCC)6	(TCC)6	(TCC)6	(TCC)6	(TCC)6	(TCC)5	-	Introns/Intergenic	-	-	CTCCCAACTGTAGCTCTCCG	ATACACCACCTTGCAGCACA	173	A	A	A	NA
PMS1582	Os_Chr03	7468628	7468645	(CTG)6	(CTG)7	(CTG)6	(CTG)6	(CTG)6	(CTG)7	(CTG)6	(CTG)6	(CTG)6	(CTG)6	(CTG)6	-	Introns/Intergenic	-	-	GATCATCCTCACCTTGTGGG	TGCAAAATCACCGGAGAAAG	266	P	A	A	NA
PMS1583	Os_Chr03	7469656	7469675	(TA)10	(TA)10	(TA)6	(TA)10	(TA)10	(TA)10	(TA)10	(TA)10	(TA)10	(TA)10	(TA)10	LOC_Os03g13790	URR	myb/SANT domain protein, putative, expressed	-	ATGTCAGCAACCAGCGTAACTGC	AAAGAGCATGAGGCATACTGATCG	405	P	P	A	RM14663
PMS1584	Os_Chr03	7785463	7785542	(AT)40	(AT)40	(AT)6	(AT)8	(AT)40	(AT)40	(AT)40	(AT)40	(AT)40	(AT)6	(AT)40	-	Introns/Intergenic	-	-	TGGTCAGGCAAGGTTCTTTT	CGAATGCTTACCAGAATAGCC	278	P	A	A	NA
PMS1585	Os_Chr03	7799763	7799776	(GA)7	(GA)7	(GA)7	(GA)7	(GA)7	(GA)7	(GA)7	(GA)6	(GA)7	(GA)7	(GA)7	-	Introns/Intergenic	-	-	GACGGTGGAGAGAGAGGAAA	CTACGAGGGGAGGAGGACAT	279	A	A	A	NA

Polymorphic SSR (PMS) markers	Chromosomes	Start physical positions (bp)	End physical positions (bp)	Nipponbare	Kasalath	Nagina22	IR64	Pokkali	Bala	Tainung67	Azucena	Moroberekan	*Oryza rufipogon*	*Oryza nivara*	MSU gene locus IDs	Structural annotation	Functional annotation	SSR functional effects	Forward primers (5'-3')	Reverse primers (5'-3')	Amplified product size (bp)	Markers exhibiting polymorphism within *indica* rice	Transcrption factor genes-derived markers	Known cloned genes-derived markers	PMS markers corresponding with RM markers
PMS1586	Os_Chr03	7918027	7918044	(TCC)6	(TCC)6	(TCC)6	(TCC)6	(TCC)6	(TCC)6	(TCC)6	(TCC)6	(TCC)6	(TCC)6	(TCC)5	LOC_Os03g14590	CDS	calcium-binding EF hand family protein, putative, expressed	CODON_DELETION	GTATCTCCGACCCG TCCAC	GCAACTTTTTCTCCG TCGAG	176	A	A	A	NA
PMS1587	Os_Chr03	8120087	8120120	(TA)17	(TA)17	(TA)17	(TA)7	(TA)17	(TA)17	(TA)6	(TA)17	(TA)9	(TA)17	(TA)17	-	Introns/Intergenic	-	-	TTATTATGGACCTG GTGGGG	TGGGTGCATGCATTG TATTT	259	P	A	A	NA
PMS1588	Os_Chr03	8164443	8164454	(TA)6	(TA)6	(TA)6	(TA)6	(TA)6	(TA)6	(TA)6	(TA)6	(TA)6	(TA)7	(TA)6	-	Introns/Intergenic	-	-	TATCTTGATTCTCCC AGCCG	CATCCCATTTTGGGT GAGAC	190	A	A	A	NA
PMS1589	Os_Chr03	8201817	8201855	(AAG)13	(AAG)13	(AAG)13	(AAG)13	(AAG)13	(AAG)13	(AAG)13	(AAG)13	(AAG)13	(AAG)13	(AAG)12	LOC_Os03g15020	CDS	beta-galactosidase precursor, putative, expressed	CODON_DELETION	AGGAGGAAGGAAG AACAGAGTTGC	CTGAGTGCGTGCCAT TTATTTCC	141	A	A	A	RM5639
PMS1590	Os_Chr03	8223601	8223620	(AG)10	(AG)9	(AG)9	(AG)10	(AG)9	(AG)9	(AG)10	(AG)10	(AG)10	(AG)10	(AG)10	-	Introns/Intergenic	-	-	TTCAGATTGCTCGT TAGGAAGC	TCCAGTCCATCAGGT GACTCC	107	P	A	A	RM14687
PMS1591	Os_Chr03	8330289	8330306	(GCC)6	(GCC)6	(GCC)6	(GCC)6	(GCC)6	(GCC)6	(GCC)6	(GCC)6	(GCC)6	(GCC)6	(GCC)5	LOC_Os03g15230	CDS	DUF292 domain containing protein, expressed	CODON_DELETION	CACCGGATGCACCT TCTC	TGTAACAGGTTCAGG GGAGG	236	A	A	A	NA
PMS1592	Os_Chr03	8429687	8429707	(GGC)7	(GGC)7	(GGC)7	(GGC)7	(GGC)7	(GGC)7	(GGC)5	(GGC)5	(GGC)7	(GGC)7	(GGC)7	LOC_Os03g15410	CDS	expressed protein	CODON_DELETION	CGCAAGAATTCATT CGGTCTCG	GCGATGGCGTAGGA GAAGAGC	288	A	A	A	RM14697
PMS1593	Os_Chr03	8456897	8456912	(CG)8	(CG)8	(CG)8	(CG)8	(CG)8	(CG)8	(CG)8	(CG)8	(CG)8	(CG)7	(CG)8	-	Introns/Intergenic	-	-	GCTAGCTCGAACCA CCACTC	CGGGCGTAATCTCGT ATCTC	206	A	A	A	NA
PMS1594	Os_Chr03	8484176	8484203	(AT)14	(AT)14	(AT)7	(AT)14	(AT)14	(AT)14	(AT)8	(AT)14	(AT)14	(AT)14	(AT)8	-	Introns/Intergenic	-	-	CAAGTTAATGCACC GTGGGT	TGGAATTTGCAATGA ACAGC	204	P	A	A	NA
PMS1595	Os_Chr03	8492004	8492021	(AG)9	(AG)8	(AG)9	(AG)7	(AG)9	(AG)8	(AG)9	(AG)9	(AG)9	(AG)9	(AG)6	-	Introns/Intergenic	-	-	GGACGACGATGAC GATACAA	CCCCACCTCTCTCTC CTCTT	132	P	A	A	NA
PMS1596	Os_Chr03	8511426	8511455	(TA)15	(TA)14	(TA)15	(TA)15	(TA)15	(TA)14	(TA)15	(TA)15	(TA)15	(TA)8	(TA)15	-	Introns/Intergenic	-	-	AAACAACCGACGTG TTAGATCTGG	GATGGAATATGAGC GATGACAGC	237	P	A	A	RM14701
PMS1597	Os_Chr03	8623448	8623465	(GCG)6	(GCG)6	(GCG)6	(GCG)6	(GCG)6	(GCG)6	(GCG)5	(GCG)5	(GCG)6	(GCG)6	(GCG)6	LOC_Os03g15630	URR	harpin-induced protein 1 domain containing protein, expressed	-	CATTAGCTCCCGAA ATCCAA	AGGTGAGCAGCAGG AAGATG	243	A	A	A	NA
PMS1598	Os_Chr03	8827884	8827904	(TCG)7	(TCG)7	(TCG)7	(TCG)7	(TCG)7	(TCG)7	(TCG)7	(TCG)7	(TCG)7	(TCG)7	(TCG)5	LOC_Os03g16010	URR	BRASSINOSTEROID INSENSITIVE 1-associated receptor kinase 1 precursor, putative, expressed	-	GCACTGGCCGCCTA ATCTCTCC	TTCACCACTCAGCAA CACACAAGC	283	A	A	A	RM14708

Polymorphic SSR (PMS) markers	Chromosomes	Start physical positions (bp)	End physical positions (bp)	Nipponbare	Kasalath	Nagina22	IR64	Pokkali	Bala	Tainung67	Azucena	Moroberekan	*Oryza rufipogon*	*Oryza nivara*	MSU gene locus IDs	Structural annotation	Functional annotation	SSR functional effects	Forward primers (5'-3')	Reverse primers (5'-3')	Amplified product size (bp)	Markers exhibiting polymorphism within *indica* rice	Transcrption factor genes-derived markers	Known cloned genes-derived markers	PMS markers corresponding with RM markers
PMS1599	Os_Chr03	8851990	8852010	(CGG)7	(CGG)5	(CGG)7	(CGG)7	(CGG)7	(CGG)5	(CGG)7	(CGG)7	(CGG)7	(CGG)7	(CGG)7	LOC_Os03g16060	CDS	expressed protein	CODON_CHANGE_PLUS_CODON_DELETION	GCTGATTGGCTCATCCATTT	CCGTTCTTGCTGCTCTTCTT	236	P	A	A	NA
PMS1600	Os_Chr03	8912726	8912743	(AG)9	(AG)9	(AG)9	(AG)9	(AG)9	(AG)9	(AG)8	(AG)9	(AG)9	(AG)9	(AG)9	LOC_Os03g16150	URR	mannose-1-phosphate guanyltransferase, putative, expressed	-	GCGGGAATTGAGAAAGAAGA	CTCGCAGGGATTGATTAGGA	242	A	A	A	NA
PMS1601	Os_Chr03	8947686	8947711	(AG)13	(AG)8	(AG)13	(AG)13	(AG)13	(AG)13	(AG)13	(AG)13	(AG)13	(AG)13	(AG)13	-	Introns/Intergenic	-	-	ATCACACGGATGGCACACAGC	GAAGATAAAGCGAAAGGGACCAAACC	247	P	A	A	RM14710
PMS1602	Os_Chr03	8952517	8952544	(TA)14	(TA)14	(TA)7	(TA)9	(TA)14	(TA)14	(TA)14	(TA)14	(TA)14	(TA)14	(TA)14	-	Introns/Intergenic	-	-	CTCCCCTTATTCTCTTTCTACTACAG	AAAAGTTTACATACCCGATTCAAA	279	P	A	A	NA
PMS1603	Os_Chr03	8991174	8991191	(GA)9	(GA)9	(GA)9	(GA)9	(GA)9	(GA)9	(GA)7	(GA)9	(GA)9	(GA)7	(GA)9	-	Introns/Intergenic	-	-	GAAGCTTTATAGGGGCTCGG	ACCTGTCGGTCGTCCCTAAC	278	A	A	A	NA
PMS1604	Os_Chr03	9063795	9063808	(GT)7	(GT)7	(GT)7	(GT)6	(GT)7	(GT)7	(GT)7	(GT)7	(GT)7	(GT)7	(GT)7	-	Introns/Intergenic	-	-	GACGCTGCTGTGATAACGAA	ACTCCCACGACTCGATCAAC	230	P	A	A	NA
PMS1605	Os_Chr03	9210850	9210867	(TC)9	(TC)9	(TC)9	(TC)9	(TC)9	(TC)8	(TC)9	(TC)9	(TC)9	(TC)9	(TC)9	-	Introns/Intergenic	-	-	GTAGCACATCCGACAGCTTG	AAAATCAAACCCCAAATCCC	239	P	A	A	NA
PMS1606	Os_Chr03	9274147	9274179	(TCT)11	(TCT)11	(TCT)11	(TCT)11	(TCT)11	(TCT)11	(TCT)11	(TCT)11	(TCT)11	(TCT)10	(TCT)11	-	Introns/Intergenic	-	-	CTCAGCGGACTAGAATGCAAGC	AAACGGATACATCTTCGCAGAACG	235	A	A	A	RM14728
PMS1607	Os_Chr03	9276829	9276846	(CAC)6	(CAC)5	(CAC)5	(CAC)6	(CAC)6	(CAC)5	(CAC)6	(CAC)6	(CAC)6	(CAC)6	(CAC)5	-	Introns/Intergenic	-	-	CCAAAGACGATCCAGAGAGC	TACGTACGACAGATCGGCAG	268	P	A	A	NA
PMS1608	Os_Chr03	9315945	9315977	(CCT)11	(CCT)11	(CCT)11	(CCT)11	(CCT)11	(CCT)11	(CCT)5	(CCT)11	(CCT)11	(CCT)11	(CCT)11	LOC_Os03g16800	URR	clathrin assembly protein, putative, expressed	-	ATGCTGGTCTGGTCCTTCACC	TTCAGATCTAAACCGAGCAAGAGG	164	A	P	A	RM6783
PMS1609	Os_Chr03	9316534	9316559	(CT)13	(CT)13	(CT)13	(CT)13	(CT)13	(CT)13	(CT)12	(CT)13	(CT)13	(CT)13	(CT)13	LOC_Os03g16800	URR	clathrin assembly protein, putative, expressed	-	CGATCCAAGCGAAAGATTTACC	GCTTCTTCCTCCCTCGTCTCC	212	A	P	A	RM3278
PMS1610	Os_Chr03	9329660	9329683	(AGG)8	(AGG)8	(AGG)8	(AGG)7	(AGG)8	(AGG)8	(AGG)8	(AGG)8	(AGG)8	(AGG)8	(AGG)8	LOC_Os03g16824	CDS	spotted leaf 11, putative, expressed	CODON_CHANGE_PLUS_CODON_DELETION	GAAGCGAAGCCGAACCCTAACG	GGCGATCTCTTCCACCAACTCC	189	P	A	A	RM5803
PMS1611	Os_Chr03	9402459	9402476	(CCA)6	(CCA)5	(CCA)6	(CCA)6	(CCA)6	(CCA)6	(CCA)6	(CCA)6	(CCA)6	(CCA)6	(CCA)6	-	Introns/Intergenic	-	-	CCGTCCATCAATCTACCACC	GGAAGGGGAGAGACGGTAAC	268	P	A	A	NA

Polymorphic SSR (PMS) markers	Chromosomes	Start physical positions (bp)	End physical positions (bp)	Nipponbare	Kasalath	Nagina22	IR64	Pokkali	Bala	Tainung67	Azucena	Moroberekan	*Oryza rufipogon*	*Oryza nivara*	MSU gene locus IDs	Structural annotation	Functional annotation	SSR functional effects	Forward primers (5'-3')	Reverse primers (5'-3')	Amplified product size (bp)	Markers exhibiting polymorphism within *indica* rice	Transcrption factor genes-derived markers	Known cloned genes-derived markers	PMS markers corresponding with RM markers
PMS1612	Os_Chr03	9458362	9458382	(CCG)7	(CCG)7	(CCG)6	(CCG)7	(CCG)7	(CCG)7	(CCG)7	(CCG)7	(CCG)7	(CCG)7	(CCG)7	LOC_Os03g17020	CDS	GTPase-activating protein, putative, expressed	CODON_DELETION	AAATCGTCGTCCCAGTCATCC	GTCGCCAAGTACAACTCCAACG	203	P	A	A	RM14743
PMS1613	Os_Chr03	9744188	9744219	(CT)16	(CT)16	(CT)16	(CT)16	(CT)16	(CT)16	(CT)16	(CT)15	(CT)16	(CT)16	(CT)16	-	Introns/Intergenic	-	-	TTCTTCTTCGTCTTCTGGTTCC	CAAAGCCATACTAAACCCTAGCC	279	A	A	A	RM1256
PMS1614	Os_Chr03	9783450	9783535	(TA)43	(TA)43	(TA)43	(TA)6	(TA)9	(TA)43	(TA)43	(TA)43	(TA)6	(TA)43	(TA)9	-	Introns/Intergenic	-	-	GAGCGCCCGATTTGTAGATGC	ATATCCTTTCCACCTGACATCCTTGC	149	P	A	A	RM14761
PMS1615	Os_Chr03	9829606	9829637	(GA)16	(GA)16	(GA)16	(GA)16	(GA)16	(GA)16	(GA)16	(GA)16	(GA)16	(GA)15	(GA)16	-	Introns/Intergenic	-	-	TCTTGGAGCGGAGGCTTATACC	CTATGTAAGCAAGCCCAGGAGACG	165	A	A	A	RM14764
PMS1616	Os_Chr03	9889654	9889681	(ATAG)7	(ATAG)9	(ATAG)7	(ATAG)8	(ATAG)9	(ATAG)8	(ATAG)7	(ATAG)7	(ATAG)7	(ATAG)7	(ATAG)9	-	Introns/Intergenic	-	-	CGAACATGGTGCCTCATTTCAAAGC	GTTCTGTGAGAACCATCCATCCATCC	82	P	A	A	RM7197
PMS1617	Os_Chr03	9915433	9915474	(AT)21	(AT)20	(AT)21	(AT)21	(AT)9	(AT)21	(AT)21	(AT)13	(AT)21	(AT)21	(AT)8	-	Introns/Intergenic	-	-	CATGCGTTTCCTAGTTCCTACACG	AGACCAACTCCCGTCGTTCG	400	P	A	A	RM14770
PMS1618	Os_Chr03	9949891	9949920	(AG)15	(AG)14	(AG)15	(AG)15	(AG)15	(AG)14	(AG)15	(AG)15	(AG)15	(AG)15	(AG)15	-	Introns/Intergenic	-	-	GGCGCTAGGTGATCATTTCTCC	TTACCCTCTCCCACCAAATTAGC	166	P	A	A	RM14772
PMS1619	Os_Chr03	10046060	10046113	(TA)27	(TA)27	(TA)27	(TA)7	(TA)27	(TA)6	(TA)27	(TA)27	(TA)27	(TA)27	(TA)27	-	Introns/Intergenic	-	-	CTGGGAGAGGACTACGGTTAGGC	TGCTCTTTGATTGCCGATAGGG	545	P	A	A	RM14776
PMS1620	Os_Chr03	10107465	10107485	(CGG)7	(CGG)7	(CGG)7	(CGG)7	(CGG)5	(CGG)7	(CGG)7	(CGG)7	(CGG)7	(CGG)7	(CGG)7	LOC_Os03g18110	CDS	proteins of unknown function domain containing protein, putative, expressed	CODON_CHANGE_PLUS_CODON_DELETION	GAAGAACTGCTACGGCGAGACG	GAGATGTCGAGGAGGCGAACG	153	P	A	A	RM14779
PMS1621	Os_Chr03	10142632	10142655	(CGC)8	(CGC)8	(CGC)8	(CGC)8	(CGC)6	(CGC)8	(CGC)8	(CGC)6	(CGC)8	(CGC)8	(CGC)8	-	Introns/Intergenic	-	-	TACATGATTCCCTGCCAACAAGG	GTACGTCTGCTGTGTCATGTGTCC	333	P	A	A	RM6496
PMS1622	Os_Chr03	10170180	10170261	(AT)41	(AT)40	(AT)7	(AT)7	(AT)7	(AT)41	(AT)41	(AT)16	(AT)6	(AT)41	(AT)41	-	Introns/Intergenic	-	-	CCGCCTACGTAACGGTGTTGACC	ATCGTGGACCCAACATGTCAGTGG	114	P	A	A	RM4321
PMS1623	Os_Chr03	10346124	10346137	(TC)7	(TC)7	(TC)7	(TC)7	(TC)7	(TC)7	(TC)7	(TC)7	(TC)7	(TC)7	(TC)6	-	Introns/Intergenic	-	-	ATGGCCCAAATATCTTGACG	TCCCACCTTTTGCAGATTTC	214	A	A	A	NA
PMS1624	Os_Chr03	10358591	10358662	(AG)36	(AG)36	(AG)36	(AG)36	(AG)36	(AG)36	(AG)36	(AG)24	(AG)36	(AG)36	(AG)36	-	Introns/Intergenic	-	-	ATTCAATCCGGTGTTTCGAC	GGTTGCTGTCCTGTTTGTCA	191	A	A	A	NA

Polymorphic SSR (PMS) markers	Chromosomes	Start physical positions (bp)	End physical positions (bp)	Nipponbare	Kasalath	Nagina22	IR64	Pokkali	Bala	Tainung67	Azucena	Moroberekan	*Oryza rufipogon*	*Oryza nivara*	MSU gene locus IDs	Structural annotation	Functional annotation	SSR functional effects	Forward primers (5'-3')	Reverse primers (5'-3')	Amplified product size (bp)	Markers exhibiting polymorphism within *indica* rice	Transcrption factor genes-derived markers	Known cloned genes-derived markers	PMS markers corresponding with RM markers
PMS1625	Os_Chr03	10359090	10359104	(CCG)5	(CCG)6	(CCG)5	(CCG)5	(CCG)5	(CCG)6	(CCG)5	(CCG)5	(CCG)6	(CCG)5	(CCG)5	LOC_Os03g18454	CDS	gamma-interferon-inducible lysosomal thiol reductase precursor, putative, expressed	CODON_INSERTION	CTCGTAGTAGAGCGCGAGTG	CCGAGAGCTTAGCTTGAGGA	213	P	A	A	NA
PMS1626	Os_Chr03	10372501	10372524	(CGC)8	(CGC)8	(CGC)8	(CGC)8	(CGC)7	(CGC)8	(CGC)8	(CGC)8	(CGC)8	(CGC)8	(CGC)8	LOC_Os03g18500	URR	mitochondrial import inner membrane translocase subunit Tim17, putative, expressed	-	GGAGAGGGAACCAACAACAA	ATCACACCGATCCGAATCTC	145	P	A	A	NA
PMS1627	Os_Chr03	10436935	10436952	(CT)9	(CT)9	(CT)7	(CT)9	(CT)9	(CT)9	(CT)9	(CT)9	(CT)9	(CT)9	(CT)9	-	Introns/Intergenic	-	-	AGTGCCAGCAGTTGACAAAA	GCCGCAGTAAAGAAGAGTGG	219	P	A	A	NA
PMS1628	Os_Chr03	10562512	10562529	(TA)9	(TA)9	(TA)9	(TA)9	(TA)9	(TA)9	(TA)8	(TA)9	(TA)9	(TA)9	(TA)9	-	Introns/Intergenic	-	-	AGGCAGTTCAACTTCACCTCA	TGCAGAAAGAACAGCAGGTG	189	A	A	A	NA
PMS1629	Os_Chr03	10570619	10570638	(TA)10	(TA)10	(TA)6	(TA)10	(TA)6	(TA)10	(TA)10	(TA)10	(TA)10	(TA)10	(TA)10	-	Introns/Intergenic	-	-	TAAGCTTAAAGCCAACGGATCG	CGTTCACCTTTGTTACTACTCTGTCC	206	P	A	A	RM14804
PMS1630	Os_Chr03	10617292	10617312	(CCA)7	(CCA)7	(CCA)7	(CCA)7	(CCA)7	(CCA)7	(CCA)5	(CCA)7	(CCA)7	(CCA)7	(CCA)7	-	Introns/Intergenic	-	-	ACGAAAGCCTACTACCACCCATCC	AGAAGCTTCGAGGAAGCAGAGAACG	86	A	A	A	RM14807
PMS1631	Os_Chr03	10658230	10658267	(GA)19	(GA)19	(GA)19	(GA)18	(GA)19	(GA)18	(GA)19	(GA)11	(GA)19	(GA)19	(GA)19	-	Introns/Intergenic	-	-	CGTGGTACCAATTGTGTAGTTGACC	GGCACAAAGTTCTCCATTCATCC	343	P	A	A	RM5178,RM3803
PMS1632	Os_Chr03	10661122	10661133	(CT)6	(CT)8	(CT)8	(CT)6	(CT)6	(CT)6	(CT)6	(CT)6	(CT)6	(CT)6	(CT)6	-	Introns/Intergenic	-	-	CCCAAACCACAAAACCATTC	GTCGGTCGTCTGAGAGTGTG	246	P	A	A	NA
PMS1633	Os_Chr03	10684798	10684818	(GGA)7	(GGA)6	(GGA)6	(GGA)6	(GGA)6	(GGA)6	(GGA)7	(GGA)7	(GGA)6	(GGA)6	(GGA)6	LOC_Os03g19080	CDS	SSD1	CODON_DELETION	GAGAAAGGTGGAGCAAAGATTGG	TCCAGTCAAACAGGTTGAACAGC	85	A	A	P	RM14814
PMS1634	Os_Chr03	10750429	10750446	(TGC)6	(TGC)6	(TGC)6	(TGC)6	(TGC)6	(TGC)6	(TGC)6	(TGC)6	(TGC)6	(TGC)5	(TGC)6	LOC_Os03g19180	CDS	GCRP8 - Glycine and cysteine rich family protein precursor, putative, expressed	CODON_DELETION	ACGAGGCAGATCAAGGTCAC	CATCCTCTCCAACTTCCAGG	278	A	A	A	NA
PMS1635	Os_Chr03	10775713	10775748	(CT)18	(CT)18	(CT)18	(CT)18	(CT)18	(CT)18	(CT)18	(CT)18	(CT)18	(CT)19	(CT)18	LOC_Os03g19200	URR	DNAJ heat shock N-terminal domain-containing protein, putative, expressed	-	AGAGAAATGCCAGCTTTGACTGC	CCAGCTAGGATGTTGAAGGATCG	124	A	A	A	RM3434
PMS1636	Os_Chr03	10812794	10812807	(GT)7	(GT)7	(GT)7	(GT)7	(GT)7	(GT)7	(GT)7	(GT)7	(GT)8	(GT)7	(GT)7	-	Introns/Intergenic	-	-	ATTTCTGGAGGACATGGTGG	GCTCAAATTCATTCCGCATT	209	A	A	A	NA
PMS1637	Os_Chr03	10827464	10827475	(GT)6	(GT)7	(GT)6	(GT)7	(GT)6	(GT)7	(GT)6	(GT)6	(GT)7	(GT)6	(GT)6	LOC_Os03g19250	URR	AMP-binding enzyme, putative, expressed	-	AGATCGTCGATGTCCCTCTC	AAGTCTCTCGCCTGCCATTA	275	P	A	A	NA

Polymorphic SSR (PMS) markers	Chromosomes	Start physical positions (bp)	End physical positions (bp)	Nipponbare	Kasalath	Nagina22	IR64	Pokkali	Bala	Tainung67	Azucena	Moroberekan	*Oryza rufipogon*	*Oryza nivara*	MSU gene locus IDs	Structural annotation	Functional annotation	SSR functional effects	Forward primers (5'-3')	Reverse primers (5'-3')	Amplified product size (bp)	Markers exhibiting polymorphism within *indica* rice	Transcrption factor genes-derived markers	Known cloned genes-derived markers	PMS markers corresponding with RM markers
PMS1638	Os_Chr03	10844927	10844938	(TC)6	(TC)7	(TC)6	(TC)7	(TC)7	(TC)7	(TC)6	(TC)7	(TC)7	(TC)6	(TC)7	-	Introns/Intergenic	-	-	TTGATGTGCAGAAATGCCTC	TGCAGGTGAGTGTAACCAGG	231	P	A	A	NA
PMS1639	Os_Chr03	10906330	10906350	(CGG)7	(CGG)7	(CGG)7	(CGG)7	(CGG)7	(CGG)5	(CGG)7	(CGG)7	(CGG)7	(CGG)7	(CGG)7	LOC_Os03g19370	CDS	CPuORF4 - conserved peptide uORF-containing transcript, expressed	CODON_CHANGE_PLUS_CODON_DELETION	CGGTGAGTGTTAGTTTCTCTTTGTGC	GTCCACGTACTGCTGCTTCTTTATGC	190	P	A	A	RM14826
PMS1640	Os_Chr03	10929929	10929946	(GCG)6	(GCG)7	(GCG)6	(GCG)6	(GCG)6	(GCG)7	(GCG)6	(GCG)7	(GCG)7	(GCG)6	(GCG)7	LOC_Os03g19427	CDS	OsNAS1	CODON_INSERTION	GCTGTTAGACGGACAGCTCC	TGATCAACTCCGTCATCGTC	198	P	A	P	NA
PMS1641	Os_Chr03	10969655	10969669	(GGT)5	(GGT)6	(GGT)6	(GGT)5	(GGT)5	(GGT)5	(GGT)5	(GGT)6	(GGT)6	(GGT)5	(GGT)5	LOC_Os03g19510	CDS	OsClpP5	CODON_INSERTION	GTAGAGTGAGGAGGGAGGGG	GATAAGAGGCGCAACCAAAG	194	P	A	P	NA
PMS1642	Os_Chr03	11131473	11131484	(TA)6	(TA)7	(TA)6	(TA)6	(TA)6	(TA)6	(TA)6	(TA)6	(TA)10	(TA)6	(TA)6	-	Introns/Intergenic	-	-	GCCGTTAGATCATGATGGGT	CCGGCTAGCTTCAAAAGATG	248	P	A	A	NA
PMS1643	Os_Chr03	11158468	11158485	(GAG)6	(GAG)8	(GAG)8	(GAG)6	(GAG)6	(GAG)8	(GAG)6	(GAG)6	(GAG)8	(GAG)8	(GAG)6	LOC_Os03g19830	CDS	expressed protein	CODON_INSERTION	GTAGCAGGGATTCAGGTTCG	GAAGGCTGAGGAAGTTGGC	269	P	A	A	NA
PMS1644	Os_Chr03	11160385	11160400	(GA)8	(GA)8	(GA)8	(GA)7	(GA)7	(GA)7	(GA)8	(GA)8	(GA)8	(GA)8	(GA)7	-	Introns/Intergenic	-	-	GTCGCCGACTCATGTCAAG	GGGACCCATTTGTCATTCAC	144	P	A	A	NA
PMS1645	Os_Chr03	11179019	11179050	(AT)16	(AT)16	(AT)16	(AT)16	(AT)11	(AT)7	(AT)6	(AT)8	(AT)9	(AT)16	(AT)8	-	Introns/Intergenic	-	-	GCGATGGTTTATCTTCTCTTCTCTGC	GTGACAAACAAACCCTCAAGTTGG	477	P	A	A	RM14831
PMS1646	Os_Chr03	11236908	11236955	(TAT)16	(TAT)16	(TAT)16	(TAT)16	(TAT)16	(TAT)16	(TAT)5	(TAT)16	(TAT)16	(TAT)16	(TAT)16	-	Introns/Intergenic	-	-	GTTTAGGGACAGGTGACGGA	TTCTTTACAATTACGGTTCGATTG	113	A	A	A	NA
PMS1647	Os_Chr03	11271135	11271162	(TA)14	(TA)11	(TA)14	(TA)6	(TA)13	(TA)14	(TA)7	(TA)14	(TA)6	(TA)6	(TA)14	-	Introns/Intergenic	-	-	CTATGTCAACCAAGACCGCA	CGGATCCAACCATTTAATCA	256	P	A	A	NA
PMS1648	Os_Chr03	11336538	11336565	(AT)14	(AT)14	(AT)14	(AT)14	(AT)14	(AT)14	(AT)6	(AT)8	(AT)14	(AT)14	(AT)14	-	Introns/Intergenic	-	-	GAGCACTTTACGTGTTCCAATCC	GAAAGTTCTCCGGTTCCTTTCG	292	A	A	A	RM14843
PMS1649	Os_Chr03	11461756	11461767	(CT)6	(CT)7	(CT)7	(CT)7	(CT)7	(CT)7	(CT)6	(CT)6	(CT)6	(CT)6	(CT)7	LOC_Os03g20280	CDS	retrotransposon protein, putative, unclassified, expressed	FRAME_SHIFT	TGACTCCGGTTTTATCCGAC	GTGGTCTTGACATCTCGCCT	276	A	A	A	NA
PMS1650	Os_Chr03	11502642	11502669	(CAAA)7	(CAAA)5	(CAAA)7	(CAAA)7	(CAAA)7	(CAAA)5	(CAAA)7	(CAAA)7	(CAAA)5	(CAAA)7	(CAAA)6	-	Introns/Intergenic	-	-	GGTCTAACATGAGCAAGCAAGC	CTACGCAACCATGTGCTATTGG	125	P	A	A	RM14850

Polymorphic SSR (PMS) markers	Chromosomes	Start physical positions (bp)	End physical positions (bp)	Nipponbare	Kasalath	Nagina22	IR64	Pokkali	Bala	Tainung67	Azucena	Moroberekan	*Oryza rufipogon*	*Oryza nivara*	MSU gene locus IDs	Structural annotation	Functional annotation	SSR functional effects	Forward primers (5'-3')	Reverse primers (5'-3')	Amplified product size (bp)	Markers exhibiting polymorphism within *indica* rice	Transcrption factor genes-derived markers	Known cloned genes-derived markers	PMS markers corresponding with RM markers
PMS1651	Os_Chr03	11559132	11559197	(TA)33	(TA)33	(TA)33	(TA)7	(TA)8	(TA)7	(TA)33	(TA)7	(TA)33	(TA)7	(TA)6	-	Introns/Intergenic	-	-	GCCATTGTTGGACTCTGACACG	ACCAACTCAAATACGAACGACGTACC	384	P	A	A	RM14852
PMS1652	Os_Chr03	11590650	11590665	(AC)8	(AC)8	(AC)7	(AC)7	(AC)7	(AC)7	(AC)8	(AC)7	(AC)7	(AC)7	(AC)8	-	Introns/Intergenic	-	-	ATGCTTTATCACGGGAATGG	GAGCTTGATCTCAACTCGCC	221	P	A	A	NA
PMS1653	Os_Chr03	11654535	11654705	(ATT)57	(ATT)57	(ATT)6	(ATT)6	(ATT)6	(ATT)6	(ATT)57	(ATT)6	(ATT)6	(ATT)8	(ATT)6	-	Introns/Intergenic	-	-	GGAAGGTGATTTCATCCGGTAGC	TGGCATGTTTAATGCTGGTTCG	142	P	A	A	RM14860
PMS1654	Os_Chr03	11665898	11665949	(TA)26	(TA)26	(TA)26	(TA)10	(TA)26	(TA)26	(TA)26	(TA)26	(TA)26	(TA)26	(TA)26	-	Introns/Intergenic	-	-	TGAGATGTGACCCATCCTTG	CAAATCAGGGGGCTAATTCA	231	P	A	A	NA
PMS1655	Os_Chr03	11855068	11855089	(TA)11	(TA)9	(TA)11	(TA)11	(TA)11	(TA)11	(TA)11	(TA)11	(TA)11	(TA)11	(TA)10	-	Introns/Intergenic	-	-	GCAGTGTTTGGCTGCTTAAATGC	TGCCACTGCACAAGTCACAACC	273	P	A	A	RM14875
PMS1656	Os_Chr03	11864078	11864095	(AG)9	(AG)9	(AG)9	(AG)9	(AG)9	(AG)9	(AG)9	(AG)7	(AG)9	(AG)9	(AG)9	-	Introns/Intergenic	-	-	CTGATGCTCCTGATGCTTGA	GGGAGTCTCCTGGGAAAAAG	137	A	A	A	NA
PMS1657	Os_Chr03	11910335	11910352	(CT)9	(CT)10	(CT)9	(CT)9	(CT)9	(CT)10	(CT)9	(CT)9	(CT)9	(CT)9	(CT)9	-	Introns/Intergenic	-	-	ATCCGAGGTGGGATGATTTT	ATCCAAAAGAACGCATCACC	250	P	A	A	NA
PMS1658	Os_Chr03	11939659	11939685	(ACT)9	(ACT)9	(ACT)5	(ACT)9	(ACT)9	(ACT)9	(ACT)9	(ACT)9	(ACT)9	(ACT)9	(ACT)9	-	Introns/Intergenic	-	-	CGTGCTAATCCTGATTGCCT	GTGGGGAAGAGCAGTGAAAA	104	P	A	A	NA
PMS1659	Os_Chr03	11959416	11959433	(CGC)6	(CGC)6	(CGC)5	(CGC)6	(CGC)6	(CGC)6	(CGC)6	(CGC)6	(CGC)6	(CGC)6	(CGC)6	-	Introns/Intergenic	-	-	CTGAAGCGCATGTCCTTGTA	TCCCGAAGAGAGCAAGATGT	228	P	A	A	NA
PMS1660	Os_Chr03	11986996	11987013	(CT)9	(CT)7	(CT)9	(CT)9	(CT)9	(CT)7	(CT)9	(CT)9	(CT)9	(CT)9	(CT)7	-	Introns/Intergenic	-	-	CTGAGTCAGCACAAAACCCA	CTCCATGTCGTCACTGGTGT	251	P	A	A	NA
PMS1661	Os_Chr03	12047082	12047105	(GT)12	(GT)12	(GT)12	(GT)12	(GT)12	(GT)12	(GT)11	(GT)12	(GT)12	(GT)12	(GT)12	LOC_Os03g21140	URR	RNA-binding zinc finger protein, putative, expressed	-	TGCGCTACTTAAGGCTGTAGG	GAGCAACAAGTTCTCCTTCTCC	184	A	P	A	RM14886
PMS1662	Os_Chr03	12047373	12047390	(CCG)6	(CCG)6	(CCG)6	(CCG)6	(CCG)6	(CCG)6	(CCG)6	(CCG)5	(CCG)6	(CCG)6	(CCG)6	LOC_Os03g21140	CDS	RNA-binding zinc finger protein, putative, expressed	CODON_DELETION	CGGCGACAAGGAGATGATAC	ACCACCTCATCCACGGAC	279	A	P	A	NA
PMS1663	Os_Chr03	12157518	12157545	(CT)14	(CT)14	(CT)14	(CT)14	(CT)14	(CT)14	(CT)14	(CT)14	(CT)14	(CT)15	(CT)14	LOC_Os03g21270	URR	expressed protein	-	GGACTTCAAAAACGAACCCA	GGCACGTAAATACCTCGGAA	205	A	A	A	NA

Polymorphic SSR (PMS) markers	Chromosomes	Start physical positions (bp)	End physical positions (bp)	Nipponbare	Kasalath	Nagina22	IR64	Pokkali	Bala	Tainung67	Azucena	Moroberekan	*Oryza rufipogon*	*Oryza nivara*	MSU gene locus IDs	Structural annotation	Functional annotation	SSR functional effects	Forward primers (5'-3')	Reverse primers (5'-3')	Amplified product size (bp)	Markers exhibiting polymorphism within *indica* rice	Transcrption factor genes-derived markers	Known cloned genes-derived markers	PMS markers corresponding with RM markers
PMS1664	Os_Chr03	12209002	12209015	(GA)7	(GA)7	(GA)7	(GA)7	(GA)7	(GA)7	(GA)7	(GA)6	(GA)7	(GA)7	(GA)7	-	Introns/Intergenic	-	-	ACCTGTCAGCTACAGCGAGG	CAACATAAAATGGGGCCAGA	265	A	A	A	NA
PMS1665	Os_Chr03	12308848	12308865	(GA)9	(GA)9	(GA)9	(GA)9	(GA)9	(GA)9	(GA)8	(GA)9	(GA)9	(GA)6	(GA)9	LOC_Os03g21540	URR	TKL_IRAK_CrRLK1L-1.8 - The CrRLK1L-1 subfamily has homology to the CrRLK1L homolog, expressed	-	TAGCCTCCCACTCCTCTTCA	CGGCCACAAACCTAGAACTC	180	A	A	A	NA
PMS1666	Os_Chr03	12408722	12408739	(GA)9	(GA)9	(GA)9	(GA)9	(GA)9	(GA)9	(GA)9	(GA)9	(GA)9	(GA)9	(GA)11	-	Introns/Intergenic	-	-	GCCAAGACCAACAGGGTAAA	ATATCATGCCTCGCTACGCT	236	A	A	A	NA
PMS1667	Os_Chr03	12434714	12434729	(AT)8	(AT)8	(AT)8	(AT)8	(AT)8	(AT)8	(AT)7	(AT)7	(AT)7	(AT)8	(AT)8	-	Introns/Intergenic	-	-	TGCTCGTGCTAAACGCTATG	AATCCCATGTTTCCCCTCTC	131	A	A	A	NA
PMS1668	Os_Chr03	12501288	12501305	(AC)9	(AC)9	(AC)9	(AC)9	(AC)9	(AC)9	(AC)6	(AC)7	(AC)9	(AC)9	(AC)9	-	Introns/Intergenic	-	-	GTGCGTAGCTTTAGATGGGG	GTAGTTGAGGCAGGAGGCTG	271	A	A	A	NA
PMS1669	Os_Chr03	12544600	12544617	(CCG)6	(CCG)6	(CCG)6	(CCG)6	(CCG)6	(CCG)6	(CCG)5	(CCG)6	(CCG)6	(CCG)6	(CCG)6	LOC_Os03g21940	URR	60S ribosomal protein L19-3, putative, expressed	-	GAGGGGAGTTCTTACGGGAG	ACACACTCCCACCCAGGTAA	197	A	A	A	NA
PMS1670	Os_Chr03	12558564	12558577	(CG)7	(CG)9	(CG)9	(CG)9	(CG)7	(CG)9	(CG)7	(CG)7	(CG)7	(CG)6	(CG)7	-	Introns/Intergenic	-	-	AGTTCCGGAGCGATATGATG	CTCTACCTCAACCACGCCAT	265	P	A	A	NA
PMS1671	Os_Chr03	12669990	12670003	(TA)7	(TA)6	(TA)7	(TA)7	(TA)7	(TA)7	(TA)7	(TA)7	(TA)7	(TA)7	(TA)7	-	Introns/Intergenic	-	-	CATGTTTGACCGTTCATCTTCT	CTCGATTCGTTTTTGCATCA	235	P	A	A	NA
PMS1672	Os_Chr03	12724669	12724689	(GGC)7	(GGC)7	(GGC)7	(GGC)7	(GGC)7	(GGC)7	(GGC)7	(GGC)7	(GGC)7	(GGC)5	(GGC)7	LOC_Os03g22190	CDS	expressed protein	CODON_DELETION	AGGAGAGGACGTCGTCGAG	CTCCTGGAGGGGAAAACC	247	A	A	A	NA
PMS1673	Os_Chr03	12728031	12728060	(TA)15	(TA)15	(TA)12	(TA)15	(TA)10	(TA)15	(TA)15	(TA)13	(TA)6	(TA)15	(TA)15	-	Introns/Intergenic	-	-	TCCGCTAGCAGCAATTGAAACG	TTGAGTGACCACTACTGCTGTCACG	184	P	A	A	RM14912
PMS1674	Os_Chr03	12739263	12739290	(AT)14	(AT)14	(AT)14	(AT)14	(AT)7	(AT)14	(AT)14	(AT)14	(AT)14	(AT)14	(AT)14	LOC_Os03g22210	DRR	POEI46 - Pollen Ole e I allergen and extensin family protein precursor, expressed	-	TGGGAATGCCCTAACACTTC	GATTACCCTGCACCGCTTTA	196	P	A	A	NA
PMS1675	Os_Chr03	12742802	12742833	(AT)16	(AT)16	(AT)16	(AT)7	(AT)9	(AT)16	(AT)8	(AT)16	(AT)11	(AT)16	(AT)16	-	Introns/Intergenic	-	-	AAGCACCCATGTGTAGAGCAACC	GGGCATCTTAAGAAACCGTATGC	369	P	A	A	RM14916
PMS1676	Os_Chr03	12822862	12822885	(TA)12	(TA)12	(TA)10	(TA)12	(TA)12	(TA)12	(TA)12	(TA)12	(TA)12	(TA)12	(TA)12	-	Introns/Intergenic	-	-	ATAAAAGGCACCGCACAGAG	AATTTTGTGCATTTGAAACCA	276	P	A	A	NA

Polymorphic SSR (PMS) markers	Chromosomes	Start physical positions (bp)	End physical positions (bp)	Nipponbare	Kasalath	Nagina22	IR64	Pokkali	Bala	Tainung67	Azucena	Moroberekan	*Oryza rufipogon*	*Oryza nivara*	MSU gene locus IDs	Structural annotation	Functional annotation	SSR functional effects	Forward primers (5'-3')	Reverse primers (5'-3')	Amplified product size (bp)	Markers exhibiting polymorphism within *indica* rice	Transcrption factor genes-derived markers	Known cloned genes-derived markers	PMS markers corresponding with RM markers
PMS1677	Os_Chr03	12869450	12869467	(CGC)6	(CGC)5	(CGC)6	(CGC)6	(CGC)6	(CGC)6	(CGC)6	(CGC)6	(CGC)6	(CGC)5	(CGC)5	LOC_Os03g22460	URR	expressed protein	-	GCCGAAGCTAGGGTTTTTCT	GGAGAACAGGAAGCAGATCG	170	P	A	A	NA
PMS1678	Os_Chr03	12906302	12906319	(CGG)6	(CGG)6	(CGG)6	(CGG)6	(CGG)5	(CGG)6	(CGG)6	(CGG)6	(CGG)6	(CGG)6	(CGG)5	LOC_Os03g22490	DRR	heavy metal-associated domain containing protein, expressed	-	CGGTGAAGGAGAGCTTTGAC	AGGACGAGTACCGCATGTTC	185	P	A	A	NA
PMS1679	Os_Chr03	12929592	12929687	(TTA)32	(TTA)32	(TTA)32	(TTA)7	(TTA)32	(TTA)32	(TTA)32	(TTA)22	(TTA)9	(TTA)32	(TTA)32	-	Introns/Intergenic	-	-	TTCTTTCGAGGGTACGTAGAGG	CTAGCTAGCCAGTAGCTGATCG	182	P	A	A	RM6929
PMS1680	Os_Chr03	12930155	12930184	(TG)15	(TG)14	(TG)15	(TG)15	(TG)15	(TG)15	(TG)15	(TG)15	(TG)15	(TG)15	(TG)15	LOC_Os03g22510	DRR	OsSIN	-	TAGTCCGGCCGTACATGAACACG	CTTGTGTGACGCGTGGAGAACG	121	P	A	P	RM5551
PMS1681	Os_Chr03	12945976	12945993	(GGC)6	(GGC)5	(GGC)6	(GGC)6	(GGC)6	(GGC)6	(GGC)6	(GGC)6	(GGC)6	(GGC)6	(GGC)6	LOC_Os03g22540	CDS	jmjC domain containing protein, expressed	CODON_CHANGE_PLUS_CODON_DELETION	TTTACCCCTTCCTGGTTTCC	GCCTGGACGTAGTGCTTCTC	265	P	A	A	NA
PMS1682	Os_Chr03	12959660	12959689	(TA)15	(TA)15	(TA)8	(TA)15	(TA)15	(TA)7	(TA)15	(TA)15	(TA)15	(TA)15	(TA)15	-	Introns/Intergenic	-	-	ATCAACAGGCACAAATGCAG	ATTTGATACCATGGTGCCCG	270	P	A	A	NA
PMS1683	Os_Chr03	12966925	12966951	(GGT)9	(GGT)9	(GGT)9	(GGT)9	(GGT)9	(GGT)9	(GGT)9	(GGT)8	(GGT)9	(GGT)9	(GGT)5	-	Introns/Intergenic	-	-	GGAGAGGAGAGAATCACCTTAGCC	TCTCGTCTCCTCTTCTCTCTTTGC	399	A	A	A	RM14939
PMS1684	Os_Chr03	13025512	13025529	(CCG)6	(CCG)5	(CCG)6	(CCG)6	(CCG)6	(CCG)6	(CCG)6	(CCG)6	(CCG)6	(CCG)6	(CCG)6	LOC_Os03g22600	CDS	BTBN6 - Bric-a-Brac, Tramtrack, Broad Complex BTB domain with non-phototropic hypocotyl 3 NPH3 and coiled-coil domains, expressed	CODON_DELETION	ATCTCGGCGAGGTAGCTGT	CCTGGAGGACATCCTCGTC	258	P	A	A	NA
PMS1685	Os_Chr03	13027630	13027649	(AT)10	(AT)10	(AT)10	(AT)6	(AT)10	(AT)10	(AT)10	(AT)10	(AT)10	(AT)10	(AT)10	-	Introns/Intergenic	-	-	TGATGATGCAGGGTAAGAACTGC	TCCCTGTTCATTGTTCCTGTGC	146	P	A	A	RM14948
PMS1686	Os_Chr03	13098308	13098321	(GT)7	(GT)7	(GT)7	(GT)6	(GT)7	(GT)7	(GT)7	(GT)7	(GT)7	(GT)7	(GT)7	-	Introns/Intergenic	-	-	CTAGTGAGGAGGCGAGCTGT	CTGCAGCTGATCTCAATCCA	105	P	A	A	NA
PMS1687	Os_Chr03	13105908	13105955	(AT)24	(AT)24	(AT)14	(AT)24	(AT)8	(AT)24	(AT)24	(AT)24	(AT)24	(AT)9	(AT)24	-	Introns/Intergenic	-	-	CACAGGCTTGCAACAACTAAACC	CCTGGTGATGCTAACCGAATAGG	291	P	A	A	RM14951
PMS1688	Os_Chr03	13205239	13205253	(GGC)5	(GGC)5	(GGC)5	(GGC)5	(GGC)5	(GGC)5	(GGC)5	(GGC)5	(GGC)7	(GGC)5	(GGC)5	LOC_Os03g22840	CDS	retrotransposon protein, putative, unclassified, expressed	CODON_INSERTION	AGGAGCTCCAGGTTGAGATG	ATAGGTGACTCGCAACGAGC	269	A	A	A	NA
PMS1689	Os_Chr03	13236377	13236397	(CTA)7	(CTA)7	(CTA)7	(CTA)6	(CTA)7	(CTA)7	(CTA)7	(CTA)7	(CTA)7	(CTA)7	(CTA)5	-	Introns/Intergenic	-	-	CTTGGTTTCTCTCACCAAATGC	AGGCGTGGAGATTAGGGTTAGG	182	P	A	A	RM14956

Polymorphic SSR (PMS) markers	Chromosomes	Start physical positions (bp)	End physical positions (bp)	Nipponbare	Kasalath	Nagina22	IR64	Pokkali	Bala	Tainung67	Azucena	Moroberekan	*Oryza rufipogon*	*Oryza nivara*	MSU gene locus IDs	Structural annotation	Functional annotation	SSR functional effects	Forward primers (5'-3')	Reverse primers (5'-3')	Amplified product size (bp)	Markers exhibiting polymorphism within *indica* rice	Transcrption factor genes-derived markers	Known cloned genes-derived markers	PMS markers corresponding with RM markers
PMS1690	Os_Chr03	13272695	13272720	(AG)13	(AG)13	(AG)13	(AG)13	(AG)13	(AG)13	(AG)13	(AG)11	(AG)13	(AG)13	(AG)13	-	Introns/Intergenic	-	-	CTATCCGGAATTGGACCCAGACC	ACTGGCGGTCATGGAGATACAGC	477	A	A	A	RM3257
PMS1691	Os_Chr03	13282473	13282526	(TAA)18	(TAA)18	(TAA)18	(TAA)18	(TAA)18	(TAA)18	(TAA)10	(TAA)14	(TAA)18	(TAA)18	(TAA)18	-	Introns/Intergenic	-	-	TGTCTAGGTTCGTAGCATGTGTTCC	CCACTGAAAGCACCGTTAAATCC	239	A	A	A	RM5903
PMS1692	Os_Chr03	13462693	13462713	(CCA)7	(CCA)5	(CCA)5	(CCA)5	(CCA)5	(CCA)5	(CCA)5	(CCA)5	(CCA)5	(CCA)5	(CCA)5	LOC_Os03g23760	CDS	retrotransposon protein, putative, Ty3-gypsy subclass, expressed	CODON_DELETION	AATGTTAGCACCGGTGAAGG	TTAGAGTGTTGGGGAGGTGG	203	A	A	A	NA
PMS1693	Os_Chr03	13470692	13470707	(AG)8	(AG)8	(AG)8	(AG)6	(AG)8	(AG)8	(AG)8	(AG)8	(AG)8	(AG)8	(AG)8	-	Introns/Intergenic	-	-	GTATTACGCTTCTGAGCGGC	TTTTCCGCTGCAGTTTTCTT	247	P	A	A	NA
PMS1694	Os_Chr03	13496160	13496177	(TGG)6	(TGG)6	(TGG)6	(TGG)6	(TGG)6	(TGG)6	(TGG)6	(TGG)6	(TGG)5	(TGG)6	(TGG)6	LOC_Os03g23800	CDS	retrotransposon protein, putative, Ty3-gypsy subclass, expressed	CODON_DELETION	GTCATCATGTTGGCTTGCTG	CTGGTTCTGTGTGGGGTTTT	193	A	A	A	NA
PMS1695	Os_Chr03	13585389	13585406	(GAC)6	(GAC)6	(GAC)5	(GAC)6	(GAC)6	(GAC)6	(GAC)6	(GAC)6	(GAC)6	(GAC)6	(GAC)6	LOC_Os03g23940	URR	expressed protein	-	CAAGCTGGTCTTCTTCTCCG	GAAACGTCCTCCATCGTGTC	159	P	A	A	NA
PMS1696	Os_Chr03	13609257	13609272	(AC)8	(AC)8	(AC)8	(AC)8	(AC)8	(AC)8	(AC)8	(AC)7	(AC)7	(AC)8	(AC)8	-	Introns/Intergenic	-	-	CCTTCGCCCTGAAGTGATAA	GTGGTGGCAGAAGCCTGTAT	249	A	A	A	NA
PMS1697	Os_Chr03	13672281	13672294	(GA)7	(GA)7	(GA)7	(GA)7	(GA)7	(GA)7	(GA)6	(GA)7	(GA)7	(GA)7	(GA)7	-	Introns/Intergenic	-	-	ATGGGGATTCTGGAGGAGTT	GGGAGTTTGTCCCACAAAAA	187	A	A	A	NA
PMS1698	Os_Chr03	13708936	13708956	(GTC)7	(GTC)7	(GTC)7	(GTC)7	(GTC)7	(GTC)5	(GTC)7	(GTC)7	(GTC)7	(GTC)7	(GTC)7	-	Introns/Intergenic	-	-	CTGTTCCTCGATCTCCCTGA	AGTGGTCAAGATTTCCGGTG	210	P	A	A	NA
PMS1699	Os_Chr03	13881908	13881927	(TC)10	(TC)10	(TC)10	(TC)10	(TC)10	(TC)10	(TC)10	(TC)10	(TC)10	(TC)9	(TC)10	-	Introns/Intergenic	-	-	TATCTGCAGGTGCGTGTAAATGG	GCATATAGAGCGAGTAAGCGAGAGG	232	A	A	A	RM14978
PMS1700	Os_Chr03	13883854	13883886	(TGG)11	(TGG)11	(TGG)11	(TGG)11	(TGG)11	(TGG)11	(TGG)5	(TGG)11	(TGG)11	(TGG)10	(TGG)10	-	Introns/Intergenic	-	-	CGGTAAAGAGGGTGCTCACTGC	TTTGACTGTGGTTGGTTCACTGC	186	A	A	A	RM14979
PMS1701	Os_Chr03	13917344	13917357	(GC)7	(GC)7	(GC)7	(GC)7	(GC)7	(GC)7	(GC)6	(GC)7	(GC)7	(GC)7	(GC)7	LOC_Os03g24430	CDS	cytokinin-O-glucosyltransferase 3, putative, expressed	FRAME_SHIFT	GGTGTACCAGAGGGTGAGGA	TGTGATCGTCTCATTGACGC	278	A	A	A	NA
PMS1702	Os_Chr03	13922880	13922897	(CGG)6	(CGG)6	(CGG)6	(CGG)6	(CGG)6	(CGG)6	(CGG)6	(CGG)5	(CGG)6	(CGG)6	(CGG)6	LOC_Os03g24440	CDS	IPP transferase, putative, expressed	CODON_DELETION	GTCGCCGCGTATCCTACTTA	GTGGAGGGAGGTGGTGTG	198	A	A	A	NA

Polymorphic SSR (PMS) markers	Chromosomes	Start physical positions (bp)	End physical positions (bp)	Nipponbare	Kasalath	Nagina22	IR64	Pokkali	Bala	Tainung67	Azucena	Moroberekan	*Oryza rufipogon*	*Oryza nivara*	MSU gene locus IDs	Structural annotation	Functional annotation	SSR functional effects	Forward primers (5'-3')	Reverse primers (5'-3')	Amplified product size (bp)	Markers exhibiting polymorphism within *indica* rice	Transcrption factor genes-derived markers	Known cloned genes-derived markers	PMS markers corresponding with RM markers
PMS1703	Os_Chr03	13951121	13951144	(CCT)8	(CCT)8	(CCT)8	(CCT)8	(CCT)8	(CCT)8	(CCT)8	(CCT)8	(CCT)8	(CCT)7	(CCT)8	-	Introns/Intergenic	-	-	ACCTCGTCCTCACAGTCGTCACC	AGTACGGGAGCGACGTCCTTGG	395	A	A	A	RM14986
PMS1704	Os_Chr03	13982047	13982061	(GAG)5	(GAG)5	(GAG)5	(GAG)7	(GAG)5	(GAG)7	(GAG)5	(GAG)5	(GAG)5	(GAG)5	(GAG)5	-	Introns/Intergenic	-	-	AGGAGGACGAGGAGGAGAAG	CGGCTTTGAGTAAGCCAGTC	129	P	A	A	NA
PMS1705	Os_Chr03	14029837	14029892	(TA)28	(TA)28	(TA)6	(TA)28	(TA)28	(TA)9	(TA)28	(TA)8	(TA)28	(TA)28	(TA)28	-	Introns/Intergenic	-	-	GGAGCCATCGTTTCACCTTA	AGGGCCTTATCTTTTCGCTT	219	P	A	A	NA
PMS1706	Os_Chr03	14030081	14030170	(TA)45	(TA)45	(TA)45	(TA)45	(TA)8	(TA)9	(TA)45	(TA)45	(TA)45	(TA)45	(TA)22	-	Introns/Intergenic	-	-	AGGCCCTTGATGTAGCATATTGTTGC	TGGTGACATCGACCGGCTTACC	493	P	A	A	RM14992
PMS1707	Os_Chr03	14107087	14107108	(CT)11	(CT)11	(CT)11	(CT)11	(CT)11	(CT)11	(CT)11	(CT)11	(CT)12	(CT)11	(CT)11	-	Introns/Intergenic	-	-	TGTGATGCGTTAGGACCTTAATACCC	GAATCCGACAGCGTATGAGTCAGC	156	A	A	A	RM14999
PMS1708	Os_Chr03	14195190	14195213	(TA)12	(TA)12	(TA)12	(TA)9	(TA)12	(TA)12	(TA)12	(TA)12	(TA)12	(TA)12	(TA)12	-	Introns/Intergenic	-	-	ATCACTGTTGGGACGGTGAGC	CGCACGTGATAACCACGATAACC	462	P	A	A	RM15002
PMS1709	Os_Chr03	14249787	14249802	(GA)8	(GA)8	(GA)8	(GA)8	(GA)8	(GA)8	(GA)8	(GA)8	(GA)8	(GA)6	(GA)8	-	Introns/Intergenic	-	-	CCGACGGAGAGAGAGTGAGT	AAATCTGCCACCTTCACCTC	198	A	A	A	NA
PMS1710	Os_Chr03	14447211	14447228	(GGC)6	(GGC)5	(GGC)5	(GGC)5	(GGC)5	(GGC)5	(GGC)6	(GGC)6	(GGC)5	(GGC)5	(GGC)6	LOC_Os03g25304	CDS	myb-like DNA-binding domain containing protein, expressed	CODON_DELETION	ACCAGCAACGGAGAAGAAGA	GATCCTGATGCGGAGGAC	178	A	A	A	NA
PMS1711	Os_Chr03	14480947	14480998	(TA)26	(TA)26	(TA)26	(TA)26	(TA)26	(TA)25	(TA)26	(TA)26	(TA)26	(TA)26	(TA)11	-	Introns/Intergenic	-	-	GTCAATTGGCTGGCTACTTACCG	GCACAATTGTTGGGATTTGACC	551	P	A	A	RM15025
PMS1712	Os_Chr03	14494526	14494561	(TAA)12	(TAA)12	(TAA)12	(TAA)12	(TAA)12	(TAA)12	(TAA)6	(TAA)9	(TAA)12	(TAA)12	(TAA)12	-	Introns/Intergenic	-	-	AGAGGAGAGATACAGATTGAGACG	GACCCTTGATGTGAGTAGTTGG	308	A	A	A	RM6676
PMS1713	Os_Chr03	14538785	14538802	(GGC)6	(GGC)6	(GGC)6	(GGC)6	(GGC)6	(GGC)6	(GGC)5	(GGC)6	(GGC)6	(GGC)6	(GGC)6	LOC_Os03g25430	CDS	transcription regulator, putative, expressed	CODON_DELETION	GAGCCGGTGGGTAAGGCT	GTCCCCTTCCTCCACAAGAG	122	A	P	A	NA
PMS1714	Os_Chr03	14552480	14552497	(CGG)6	(CGG)6	(CGG)6	(CGG)6	(CGG)6	(CGG)6	(CGG)6	(CGG)6	(CGG)6	(CGG)5	(CGG)6	LOC_Os03g25470	CDS	ctr copper transporter family protein, putative, expressed	CODON_DELETION	ACTTCCTCTGCCTCCTCCTC	AGCATCACCAGGTAGGCG	187	A	A	A	NA
PMS1715	Os_Chr03	14568062	14568077	(CT)8	(CT)8	(CT)8	(CT)7	(CT)8	(CT)8	(CT)8	(CT)8	(CT)8	(CT)8	(CT)8	-	Introns/Intergenic	-	-	TTGCGTTTGGCTCTTTCTTT	ACGATTAAACGTTGGACGGA	195	P	A	A	NA

Polymorphic SSR (PMS) markers	Chromosomes	Start physical positions (bp)	End physical positions (bp)	Nipponbare	Kasalath	Nagina22	IR64	Pokkali	Bala	Tainung67	Azucena	Moroberekan	*Oryza rufipogon*	*Oryza nivara*	MSU gene locus IDs	Structural annotation	Functional annotation	SSR functional effects	Forward primers (5'-3')	Reverse primers (5'-3')	Amplified product size (bp)	Markers exhibiting polymorphism within *indica* rice	Transcrption factor genes-derived markers	Known cloned genes-derived markers	PMS markers corresponding with RM markers
PMS1716	Os_Chr03	14577640	14577729	(TA)45	(TA)31	(TA)6	(TA)6	(TA)45	(TA)10	(TA)45	(TA)24	(TA)45	(TA)6	(TA)6	-	Introns/Intergenic	-	-	TTCGTGATCGGTCATCCATCC	AGATTCATATGCTGCGCTGTTACC	330	P	A	A	RM15035
PMS1717	Os_Chr03	14667890	14667904	(CGG)5	(CGG)6	(CGG)5	(CGG)5	(CGG)5	(CGG)5	(CGG)5	(CGG)5	(CGG)5	(CGG)5	(CGG)5	LOC_Os03g25610	CDS	heavy metal-associated domain containing protein, expressed	CODON_INSERTION	TCCACCTCGTAGAGGACGAC	GATGAGAAGGGGACGATGAC	275	P	A	A	NA
PMS1718	Os_Chr03	14698814	14698837	(CTCG)6	(CTCG)6	(CTCG)6	(CTCG)6	(CTCG)6	(CTCG)6	(CTCG)6	(CTCG)6	(CTCG)6	(CTCG)5	(CTCG)6	LOC_Os03g25680	URR	expressed protein	-	GATCGTATCTCGCACGGTGTTCG	CTGGCCGCTTGCTAATATTTCATACC	279	A	A	A	RM7369
PMS1719	Os_Chr03	14781548	14781623	(AT)38	(AT)38	(AT)38	(AT)6	(AT)9	(AT)38	(AT)38	(AT)19	(AT)38	(AT)10	(AT)10	-	Introns/Intergenic	-	-	GCAAATGGTCAAAATACCACC	ATCCTCCTCCCTCTCCTTTG	280	P	A	A	NA
PMS1720	Os_Chr03	14861879	14861904	(AG)13	(AG)12	(AG)13	(AG)13	(AG)13	(AG)13	(AG)13	(AG)13	(AG)13	(AG)13	(AG)13	-	Introns/Intergenic	-	-	TTTCTGGCGACGTGATTTGTCG	CAATTCGGAAGAGCAAACATGACC	87	P	A	A	RM1164
PMS1721	Os_Chr03	14937020	14937043	(CAA)8	(CAA)6	(CAA)8	(CAA)6	(CAA)8	(CAA)8	(CAA)8	(CAA)8	(CAA)8	(CAA)8	(CAA)8	LOC_Os03g26070	CDS	expressed protein	CODON_DELETION	GAGCTGGAGAGGATCAGAGTGC	TAGTGTAATGCTGCTGCTTCTGC	380	P	P	A	RM15059
PMS1722	Os_Chr03	14937944	14937961	(CGA)6	(CGA)7	(CGA)7	(CGA)7	(CGA)6	(CGA)6	(CGA)6	(CGA)6	(CGA)6	(CGA)6	(CGA)6	LOC_Os03g26070	CDS	expressed protein	CODON_CHANGE_PLUS_CODON_INSERTION	TGCAGGAGCTATACAGGATGC	TTTGATCGCGTGGTTGTCTA	223	P	P	A	NA
PMS1723	Os_Chr03	14969265	14969314	(TA)25	(TA)25	(TA)9	(TA)25	(TA)25	(TA)25	(TA)25	(TA)25	(TA)25	(TA)25	(TA)25	-	Introns/Intergenic	-	-	CCAAAATATCGGCAAAAACA	AAGCTATTGGGAAACAACGG	280	P	A	A	NA
PMS1724	Os_Chr03	15079995	15080018	(GCAG)6	(GCAG)6	(GCAG)5	(GCAG)6	(GCAG)6	(GCAG)6	(GCAG)6	(GCAG)6	(GCAG)6	(GCAG)6	(GCAG)6	-	Introns/Intergenic	-	-	GCCGCAGTTGAGAGAACTCTTCC	GAGACGCGGATGACGAGACG	169	P	A	A	RM15066
PMS1725	Os_Chr03	15130294	15130305	(AT)6	(AT)8	(AT)6	(AT)6	(AT)6	(AT)8	(AT)6	(AT)6	(AT)6	(AT)6	(AT)6	-	Introns/Intergenic	-	-	TGGATGGGAGAAGCCATAAG	ATGAAGCCATCCAGTTTTGG	244	P	A	A	NA
PMS1726	Os_Chr03	15151581	15151594	(AC)7	(AC)8	(AC)7	(AC)7	(AC)7	(AC)6	(AC)7	(AC)6	(AC)6	(AC)7	(AC)8	LOC_Os03g26530	DRR	3-ketoacyl-CoA synthase, putative, expressed	-	CCCACCCATCTCCACAAAT	TGCACCTACGTACTCGATCCT	280	P	P	A	NA
PMS1727	Os_Chr03	15151948	15151971	(TA)12	(TA)11	(TA)12	(TA)11	(TA)12	(TA)12	(TA)12	(TA)12	(TA)12	(TA)12	(TA)12	LOC_Os03g26530	DRR	3-ketoacyl-CoA synthase, putative, expressed	-	GGCCATGTGTTGTTTCAGTGG	TGTAGCTGCGATCTACTTCACTCTCC	382	P	P	A	RM15068
PMS1728	Os_Chr03	15156811	15156826	(CT)8	(CT)8	(CT)8	(CT)8	(CT)8	(CT)8	(CT)8	(CT)8	(CT)6	(CT)8	(CT)8	-	Introns/Intergenic	-	-	TTTGAAATCAGCAATGCAGC	AGAGGGAGAGAGAGAGGCGA	250	A	A	A	NA

Polymorphic SSR (PMS) markers	Chromosomes	Start physical positions (bp)	End physical positions (bp)	Nipponbare	Kasalath	Nagina22	IR64	Pokkali	Bala	Tainung67	Azucena	Moroberekan	*Oryza rufipogon*	*Oryza nivara*	MSU gene locus IDs	Structural annotation	Functional annotation	SSR functional effects	Forward primers (5'-3')	Reverse primers (5'-3')	Amplified product size (bp)	Markers exhibiting polymorphism within *indica* rice	Transcrption factor genes-derived markers	Known cloned genes-derived markers	PMS markers corresponding with RM markers
PMS1729	Os_Chr03	15159940	15159957	(CT)9	(CT)9	(CT)9	(CT)9	(CT)9	(CT)8	(CT)9	(CT)9	(CT)6	(CT)9	(CT)6	-	Introns/Intergenic	-	-	TTTTTCCCTCCTCCTCCATT	GGAGGTCGGGGAACGAAG	234	P	A	A	NA
PMS1730	Os_Chr03	15323878	15323892	(CGG)5	(CGG)5	(CGG)5	(CGG)5	(CGG)6	(CGG)5	(CGG)5	(CGG)5	(CGG)6	(CGG)5	(CGG)5	LOC_Os03g26860	CDS	ribosomal protein, putative, expressed	CODON_INSERTION	CAGAGCACAGGTGTACTTCCC	TGAAAACCCTATGCACTCCC	101	P	A	A	NA
PMS1731	Os_Chr03	15342693	15342714	(TG)11	(TG)11	(TG)11	(TG)11	(TG)11	(TG)11	(TG)11	(TG)11	(TG)11	(TG)10	(TG)11	-	Introns/Intergenic	-	-	GGAGCAGGCTCATACACACATAGC	GTGTACCTCCTACGACATGTCAATCC	228	A	A	A	RM15073
PMS1732	Os_Chr03	15437125	15437145	(CGC)7	(CGC)7	(CGC)7	(CGC)7	(CGC)7	(CGC)7	(CGC)7	(CGC)7	(CGC)7	(CGC)6	(CGC)7	LOC_Os03g26990	CDS	VQ domain containing protein, putative, expressed	CODON_CHANGE_PLUS_CODON_DELETION	CCGCTCAAGTACACGCACAGC	GAAGGAGGTGGTGGTGCAAGC	81	A	A	A	RM15083
PMS1733	Os_Chr03	15512679	15512699	(CGC)7	(CGC)5	(CGC)7	(CGC)7	(CGC)7	(CGC)7	(CGC)7	(CGC)7	(CGC)7	(CGC)7	(CGC)7	LOC_Os03g27090	CDS	MYB family transcription factor, putative, expressed	CODON_DELETION	CAAATCATCGATGGCGTTC	GCGACTCCTCTCCCTTCTCT	222	P	P	A	NA
PMS1734	Os_Chr03	15524153	15524172	(GA)10	(GA)10	(GA)10	(GA)10	(GA)10	(GA)10	(GA)9	(GA)10	(GA)10	(GA)10	(GA)10	-	Introns/Intergenic	-	-	ACGCTTGCACAAGCTGAAGTAGACC	GGTTGACACCGAGCAACATCG	434	A	A	A	RM15094
PMS1735	Os_Chr03	15524677	15524690	(GA)7	(GA)7	(GA)7	(GA)7	(GA)8	(GA)8	(GA)7	(GA)7	(GA)8	(GA)8	(GA)7	-	Introns/Intergenic	-	-	CGCCGCATAGAGAGAGAACT	AACTCCCTAGGCCCACTTGT	237	P	A	A	NA
PMS1736	Os_Chr03	15634728	15634761	(TA)17	(TA)16	(TA)7	(TA)17	(TA)17	(TA)17	(TA)14	(TA)17	(TA)9	(TA)17	(TA)17	-	Introns/Intergenic	-	-	CCCTAGGGTTGTATGTTGTTGG	ACCCGTAGATAACCCATACCTAACC	141	P	A	A	RM15107
PMS1737	Os_Chr03	15638053	15638070	(GC)9	(GC)9	(GC)9	(GC)6	(GC)9	(GC)9	(GC)9	(GC)9	(GC)9	(GC)9	(GC)9	-	Introns/Intergenic	-	-	CTAGAAGCTTCCATCGCCAC	GGTAGAGGAGACGGGGAGAC	252	P	A	A	NA
PMS1738	Os_Chr03	15714723	15714742	(TC)10	(TC)10	(TC)9	(TC)10	(TC)10	(TC)10	(TC)10	(TC)10	(TC)10	(TC)10	(TC)8	-	Introns/Intergenic	-	-	ATCTAGCTTGCATTCCGAGTGG	GAATGTGCTGTACGATCTGAACC	405	P	A	A	RM15121
PMS1739	Os_Chr03	15790679	15790726	(TAT)16	(TAT)16	(TAT)16	(TAT)16	(TAT)16	(TAT)16	(TAT)16	(TAT)16	(TAT)16	(TAT)16	(TAT)7	-	Introns/Intergenic	-	-	TGCAAATGCACCGATAGTGT	TTACAAAATATGGCCGGAAAA	205	A	A	A	NA
PMS1740	Os_Chr03	15792269	15792284	(AG)8	(AG)8	(AG)8	(AG)8	(AG)8	(AG)7	(AG)8	(AG)8	(AG)8	(AG)8	(AG)8	-	Introns/Intergenic	-	-	CCTCTGCAGCTAGCACTCG	ATCGACAAAAGTAGTGGCGG	247	P	A	A	NA
PMS1741	Os_Chr03	15859445	15859496	(TA)26	(TA)26	(TA)26	(TA)26	(TA)26	(TA)26	(TA)26	(TA)26	(TA)12	(TA)26	(TA)26	-	Introns/Intergenic	-	-	CGGCATAATTTGCTCGATTT	TTGAAGATGCGACTTTGCAC	235	A	A	A	NA

Polymorphic SSR (PMS) markers	Chromosomes	Start physical positions (bp)	End physical positions (bp)	Nipponbare	Kasalath	Nagina22	IR64	Pokkali	Bala	Tainung67	Azucena	Moroberekan	*Oryza rufipogon*	*Oryza nivara*	MSU gene locus IDs	Structural annotation	Functional annotation	SSR functional effects	Forward primers (5'-3')	Reverse primers (5'-3')	Amplified product size (bp)	Markers exhibiting polymorphism within *indica* rice	Transcrption factor genes-derived markers	Known cloned genes-derived markers	PMS markers corresponding with RM markers
PMS1742	Os_Chr03	15868681	15868704	(AG)12	(AG)12	(AG)12	(AG)12	(AG)12	(AG)7	(AG)12	(AG)12	(AG)12	(AG)12	(AG)12	-	Introns/Intergenic	-	-	CGAGTTCAGTCTTGGAACCATCC	TGAGCAGTGGAAGACAGTTAGGG	202	P	A	A	RM15136
PMS1743	Os_Chr03	15888795	15888850	(AT)28	(AT)28	(AT)28	(AT)16	(AT)28	(AT)28	(AT)28	(AT)28	(AT)28	(AT)28	(AT)28	-	Introns/Intergenic	-	-	GGCTAAGCCATCTTTAGTCC	CATATGTTTCTATATGCATGTGT	281	P	A	A	NA
PMS1744	Os_Chr03	15901945	15901965	(GCG)7	(GCG)6	(GCG)7	(GCG)7	(GCG)7	(GCG)7	(GCG)7	(GCG)7	(GCG)7	(GCG)7	(GCG)7	LOC_Os03g27740	CDS	expressed protein	CODON_CHANGE_PLUS_CODON_DELETION	CAGCAGCAGAGACACCTGCTATCG	CTACAAGCAGCCTCCTGCCAACC	258	P	A	A	RM15141
PMS1745	Os_Chr03	15972384	15972417	(AT)17	(AT)17	(AT)17	(AT)17	(AT)17	(AT)17	(AT)17	(AT)6	(AT)17	(AT)17	(AT)12	-	Introns/Intergenic	-	-	TACACCATTGTGTTCGTTGC	TCAACACAAATTATGGAATTGACT	249	A	A	A	NA
PMS1746	Os_Chr03	15977807	15977822	(AG)8	(AG)13	(AG)8	(AG)8	(AG)8	(AG)8	(AG)8	(AG)8	(AG)8	(AG)8	(AG)8	-	Introns/Intergenic	-	-	CAACACTGGATGTGTCCTGC	TCTGTCAATCGGTCACTTGC	179	P	A	A	NA
PMS1747	Os_Chr03	15999259	15999272	(CA)7	(CA)6	(CA)7	(CA)7	(CA)7	(CA)7	(CA)7	(CA)7	(CA)7	(CA)7	(CA)7	-	Introns/Intergenic	-	-	AACCCCACAAGCTCAGAGAC	TCAAATGGATGTGGTCGTTG	106	P	A	A	NA
PMS1748	Os_Chr03	16024625	16024642	(AGC)6	(AGC)6	(AGC)7	(AGC)6	(AGC)6	(AGC)6	(AGC)6	(AGC)6	(AGC)6	(AGC)6	(AGC)6	-	Introns/Intergenic	-	-	GCAATGTGGATAGAGCTTTCG	GGTACGTAGTTCCTGTTTGCG	248	P	A	A	NA
PMS1749	Os_Chr03	16035388	16035405	(CT)9	(CT)9	(CT)9	(CT)9	(CT)9	(CT)12	(CT)9	(CT)9	(CT)9	(CT)9	(CT)9	-	Introns/Intergenic	-	-	CCTCATCCTGCCTTATCCAA	ATGCCAAATCTATACCGCCA	270	P	A	A	NA
PMS1750	Os_Chr03	16039345	16039365	(TGT)7	(TGT)5	(TGT)7	(TGT)7	(TGT)7	(TGT)7	(TGT)7	(TGT)7	(TGT)7	(TGT)7	(TGT)7	-	Introns/Intergenic	-	-	TTTGTAGGGTTTCGGCATCTCC	ATAGGAATCTTACGCCGGAATCG	176	P	A	A	RM15152
PMS1751	Os_Chr03	16125834	16125845	(AT)6	(AT)8	(AT)6	(AT)8	(AT)8	(AT)8	(AT)6	(AT)8	(AT)6	(AT)6	(AT)6	-	Introns/Intergenic	-	-	CGACACGTTCTCCAAAACAC	CCCAACTTCCTTCTTGACCA	252	P	A	A	NA
PMS1752	Os_Chr03	16140021	16140044	(AT)12	(AT)12	(AT)12	(AT)12	(AT)9	(AT)8	(AT)12	(AT)12	(AT)6	(AT)12	(AT)12	-	Introns/Intergenic	-	-	TATAGGTGGTACGCTTCCCG	CCCAACAATTCTACCGAGGA	193	P	A	A	NA
PMS1753	Os_Chr03	16147453	16147514	(TA)31	(TA)31	(TA)14	(TA)10	(TA)31	(TA)31	(TA)31	(TA)31	(TA)31	(TA)31	(TA)31	-	Introns/Intergenic	-	-	TGTCGTGACAAAATTACTTCCG	TCTTCAAAACAAGATCTTACATGATT	273	P	A	A	NA
PMS1754	Os_Chr03	16179112	16179126	(AGC)5	(AGC)5	(AGC)5	(AGC)5	(AGC)5	(AGC)5	(AGC)5	(AGC)5	(AGC)5	(AGC)6	(AGC)5	-	Introns/Intergenic	-	-	GATGGATGGATGGATGCTTT	GGCGGTAGCAATAGCACACT	269	A	A	A	NA

Polymorphic SSR (PMS) markers	Chromosomes	Start physical positions (bp)	End physical positions (bp)	Nipponbare	Kasalath	Nagina22	IR64	Pokkali	Bala	Tainung67	Azucena	Moroberekan	*Oryza rufipogon*	*Oryza nivara*	MSU gene locus IDs	Structural annotation	Functional annotation	SSR functional effects	Forward primers (5'-3')	Reverse primers (5'-3')	Amplified product size (bp)	Markers exhibiting polymorphism within *indica* rice	Transcrption factor genes-derived markers	Known cloned genes-derived markers	PMS markers corresponding with RM markers
PMS1755	Os_Chr03	16243711	16243731	(CCG)7	(CCG)7	(CCG)7	(CCG)7	(CCG)7	(CCG)7	(CCG)7	(CCG)7	(CCG)7	(CCG)7	(CCG)6	-	Introns/Intergenic	-	-	CATCTGCCGCTCTCTGCTACC	ATATGGGTGGTTTCGCTGACG	317	A	A	A	RM15171
PMS1756	Os_Chr03	16297063	16297086	(CGG)8	(CGG)8	(CGG)8	(CGG)8	(CGG)8	(CGG)8	(CGG)8	(CGG)8	(CGG)8	(CGG)7	(CGG)8	LOC_Os03g28310	URR	dnaJ domain containing protein, expressed	-	ATGTGCTCACCAGGATGGAT	TGCTGAGTTATCCTGACCCC	259	A	A	A	NA
PMS1757	Os_Chr03	16362264	16362275	(GA)6	(GA)6	(GA)6	(GA)7	(GA)7	(GA)6	(GA)6	(GA)7	(GA)7	(GA)6	(GA)7	LOC_Os03g28400	URR	transport protein particle component, Bet3, domain containing protein, expressed	-	GGAGGTTTCTGACTTGCTGC	CGTCTCGGCCCATCTAGTAA	276	P	A	A	NA
PMS1758	Os_Chr03	16379094	16379108	(GGA)5	(GGA)5	(GGA)6	(GGA)6	(GGA)6	(GGA)5	(GGA)5	(GGA)6	(GGA)6	(GGA)5	(GGA)6	-	Introns/Intergenic	-	-	GGGTTGGAGAAAACATTGGA	TTAAAAATTGCGTCGGGTTT	130	P	A	A	NA
PMS1759	Os_Chr03	16391024	16391041	(GTC)6	(GTC)6	(GTC)6	(GTC)6	(GTC)6	(GTC)6	(GTC)6	(GTC)5	(GTC)6	(GTC)6	(GTC)6	-	Introns/Intergenic	-	-	GTCGTCTTCACCGTTCATCC	TGCTGCACACTTGACGAAAC	244	A	A	A	NA
PMS1760	Os_Chr03	16531657	16531672	(CG)8	(CG)8	(CG)8	(CG)8	(CG)8	(CG)8	(CG)8	(CG)8	(CG)8	(CG)7	(CG)8	-	Introns/Intergenic	-	-	CCCTGTTGACCAGTTGAGGT	GAAGCGAGGAACTCAGATCG	220	A	A	A	NA
PMS1761	Os_Chr03	16547966	16547979	(CG)7	(CG)7	(CG)7	(CG)7	(CG)6	(CG)7	(CG)7	(CG)7	(CG)7	(CG)7	(CG)7	-	Introns/Intergenic	-	-	TTGACTAGTTGAGGTGGGGG	GGTTAGTGGCTGCAAAAGGA	157	P	A	A	NA
PMS1762	Os_Chr03	16706888	16706927	(TA)20	(TA)20	(TA)20	(TA)13	(TA)20	(TA)20	(TA)7	(TA)20	(TA)20	(TA)20	(TA)10	-	Introns/Intergenic	-	-	ATCACCTCAGAGCATTATGAA	GTAAATTCAGTAAAGCCTAAAAC	203	P	A	A	NA
PMS1763	Os_Chr03	16747973	16748004	(CT)16	(CT)16	(CT)16	(CT)16	(CT)16	(CT)15	(CT)16	(CT)16	(CT)16	(CT)16	(CT)16	-	Introns/Intergenic	-	-	CCTACCTGTCATTCCGTCAATCC	AAGCTGGAGCTGTGGACTACTGG	192	P	A	A	RM15191
PMS1764	Os_Chr03	16753777	16753794	(CGC)6	(CGC)6	(CGC)6	(CGC)6	(CGC)6	(CGC)6	(CGC)5	(CGC)6	(CGC)6	(CGC)6	(CGC)6	LOC_Os03g29410	CDS	tyrosine protein kinase domain containing protein, putative, expressed	CODON_DELETION	GAACACGTGCAGGTTCGAC	GCAGGATGACGAGGAAGAAG	248	A	A	A	NA
PMS1765	Os_Chr03	16756261	16756284	(TA)12	(TA)12	(TA)12	(TA)12	(TA)12	(TA)12	(TA)6	(TA)12	(TA)12	(TA)12	(TA)12	-	Introns/Intergenic	-	-	CCATACACTCCTCCCTCCTCTCC	CTCTCCCTCCACCTGGTTTCC	323	A	A	A	RM15193
PMS1766	Os_Chr03	16864577	16864606	(GGC)10	(GGC)10	(GGC)10	(GGC)10	(GGC)10	(GGC)10	(GGC)6	(GGC)10	(GGC)10	(GGC)10	(GGC)10	LOC_Os03g29584	CDS	expressed protein	CODON_DELETION	AAGCGGGTGTACAAGCACAAGC	GGAGGTTGTGGCGAGGATGG	213	A	A	A	RM15199
PMS1767	Os_Chr03	16926067	16926122	(AT)28	(AT)28	(AT)28	(AT)17	(AT)28	(AT)28	(AT)28	(AT)28	(AT)28	(AT)28	(AT)28	-	Introns/Intergenic	-	-	TAGGTGATTCTCAGCGCTTTGC	AAACGTTAGCCGAGCCATCC	459	P	A	A	RM15204

Polymorphic SSR (PMS) markers	Chromosomes	Start physical positions (bp)	End physical positions (bp)	Nipponbare	Kasalath	Nagina22	IR64	Pokkali	Bala	Tainung67	Azucena	Moroberekan	*Oryza rufipogon*	*Oryza nivara*	MSU gene locus IDs	Structural annotation	Functional annotation	SSR functional effects	Forward primers (5'-3')	Reverse primers (5'-3')	Amplified product size (bp)	Markers exhibiting polymorphism within *indica* rice	Transcrption factor genes-derived markers	Known cloned genes-derived markers	PMS markers corresponding with RM markers
PMS1768	Os_Chr03	16940769	16940786	(GGA)6	(GGA)6	(GGA)5	(GGA)6	(GGA)6	(GGA)6	(GGA)6	(GGA)6	(GGA)6	(GGA)6	(GGA)6	LOC_Os03g29730	CDS	expressed protein	CODON_DELETION	GATGTAGTCGAGGATGCGGT	CACCACCACCAGATATGCC	209	P	A	A	NA
PMS1769	Os_Chr03	17266028	17266047	(CT)10	(CT)10	(CT)10	(CT)10	(CT)10	(CT)10	(CT)10	(CT)9	(CT)10	(CT)10	(CT)10	LOC_Os03g30260	URR	COBRA-like protein precursor, putative, expressed	-	GGAGTAGTAAAGCGAAGCAGTAGC	GGAGTCCCTCTTTCTCTTTGG	168	A	A	A	RM15214
PMS1770	Os_Chr03	17273909	17273922	(GT)7	(GT)7	(GT)7	(GT)7	(GT)7	(GT)7	(GT)7	(GT)7	(GT)7	(GT)6	(GT)6	-	Introns/Intergenic	-	-	ATCTAAGAGGTGGCTTCGCC	ACGAGCTCGACACCAATCTT	270	A	A	A	NA
PMS1771	Os_Chr03	17285924	17285947	(GCC)8	(GCC)7	(GCC)8	(GCC)6	(GCC)8	(GCC)7	(GCC)8	(GCC)8	(GCC)8	(GCC)8	(GCC)8	LOC_Os03g30290	URR	expressed protein	-	CTCTTTGCGGCATGGGAGTAGC	GTAACATGTCCTTGATGGCGAAGG	169	P	A	A	RM15216
PMS1772	Os_Chr03	17294828	17294879	(TA)26	(TA)14	(TA)26	(TA)26	(TA)12	(TA)26	(TA)26	(TA)26	(TA)26	(TA)26	(TA)15	-	Introns/Intergenic	-	-	CATGGATTAGCTCCCTCAATTAGC	GCATATTCACCCTCCCTCTACG	169	P	A	A	RM15218
PMS1773	Os_Chr03	17397427	17397438	(TG)6	(TG)8	(TG)8	(TG)6	(TG)6	(TG)8	(TG)6	(TG)6	(TG)6	(TG)6	(TG)6	-	Introns/Intergenic	-	-	ACAGGGCTAAATCCCCTCAT	CGAGGGACCTAGTTTGCACT	263	P	A	A	NA
PMS1774	Os_Chr03	17415483	17415494	(CA)6	(CA)9	(CA)6	(CA)6	(CA)6	(CA)6	(CA)6	(CA)6	(CA)6	(CA)9	(CA)9	-	Introns/Intergenic	-	-	CTTCTCGTCGGAATTTGAGG	AATTGGCTGGAAATGTGCTC	256	P	A	A	NA
PMS1775	Os_Chr03	17576386	17576401	(CT)8	(CT)8	(CT)8	(CT)8	(CT)8	(CT)8	(CT)8	(CT)8	(CT)8	(CT)8	(CT)6	LOC_Os03g30840	URR	expressed protein	-	ATTCCTCAATCCTCGTGCC	GTCATCCTCTTCCTCCACCA	239	A	A	A	NA
PMS1776	Os_Chr03	17600743	17600754	(AT)6	(AT)6	(AT)6	(AT)6	(AT)6	(AT)6	(AT)6	(AT)6	(AT)6	(AT)7	(AT)6	-	Introns/Intergenic	-	-	CCAGCTGGCAGTAGCATTTT	GTTGGTGTCCTATGCCTCGT	188	A	A	A	NA
PMS1777	Os_Chr03	17714933	17714956	(CGG)8	(CGG)8	(CGG)8	(CGG)8	(CGG)8	(CGG)8	(CGG)8	(CGG)6	(CGG)8	(CGG)8	(CGG)5	-	Introns/Intergenic	-	-	CACTCCCTCCTCTCTCCTCTCC	GTTGGTTGGTCGGTTGCTTACC	170	A	A	A	RM15236
PMS1778	Os_Chr03	17773032	17773047	(TC)8	(TC)6	(TC)8	(TC)8	(TC)8	(TC)8	(TC)8	(TC)8	(TC)8	(TC)8	(TC)8	-	Introns/Intergenic	-	-	GAGCAACCTCCTCCCTTTTC	CCAATGCAAGAAAGAGCACA	140	P	A	A	NA
PMS1779	Os_Chr03	17778327	17778344	(CCG)6	(CCG)5	(CCG)6	(CCG)6	(CCG)6	(CCG)6	(CCG)6	(CCG)6	(CCG)6	(CCG)6	(CCG)5	-	Introns/Intergenic	-	-	ATCCTCTATCCCGGACACCT	TAGTGGGGCCCATATGTCAG	211	P	A	A	NA
PMS1780	Os_Chr03	17788604	17788624	(CGC)7	(CGC)7	(CGC)7	(CGC)7	(CGC)7	(CGC)7	(CGC)7	(CGC)5	(CGC)7	(CGC)7	(CGC)7	LOC_Os03g31240	CDS	ZOS3-10 - C2H2 zinc finger protein, expressed	CODON_DELETION	GACGACGACGACAGGAGATCG	CGGATATGGGTTAAGGGAGAATCC	135	A	P	A	RM15242

Polymorphic SSR (PMS) markers	Chromosomes	Start physical positions (bp)	End physical positions (bp)	Nipponbare	Kasalath	Nagina22	IR64	Pokkali	Bala	Tainung67	Azucena	Moroberekan	*Oryza rufipogon*	*Oryza nivara*	MSU gene locus IDs	Structural annotation	Functional annotation	SSR functional effects	Forward primers (5'-3')	Reverse primers (5'-3')	Amplified product size (bp)	Markers exhibiting polymorphism within *indica* rice	Transcrption factor genes-derived markers	Known cloned genes-derived markers	PMS markers corresponding with RM markers
PMS1781	Os_Chr03	17796807	17796828	(TC)11	(TC)11	(TC)11	(TC)11	(TC)11	(TC)11	(TC)11	(TC)11	(TC)11	(TC)11	(TC)10	-	Introns/Intergenic	-	-	CAATTGTTGCACTCGATCTCTCC	TAAGCATCATTGCCTGTGACTCG	100	A	A	A	RM15244
PMS1782	Os_Chr03	17883579	17883592	(GA)7	(GA)7	(GA)7	(GA)7	(GA)7	(GA)7	(GA)6	(GA)7	(GA)7	(GA)7	(GA)6	LOC_Os03g31390	CDS	expressed protein	FRAME_SHIFT	CATCCCAAATCAACACACCA	GCTCCATCTCCCTATCTCCC	263	A	A	A	NA
PMS1783	Os_Chr03	17975781	17975800	(AT)10	(AT)10	(AT)10	(AT)10	(AT)10	(AT)10	(AT)10	(AT)10	(AT)10	(AT)10	(AT)9	-	Introns/Intergenic	-	-	AAACGTGGAAAATGCTAGAATGA	GAGTGGTGCCAGACCAAAAT	261	A	A	A	NA
PMS1784	Os_Chr03	18112971	18113000	(CCTCG)6	(CCTCG)5	(CCTCG)5	(CCTCG)5	(CCTCG)5	(CCTCG)5	(CCTCG)6	(CCTCG)5	(CCTCG)6	(CCTCG)6	(CCTCG)5	-	Introns/Intergenic	-	-	ACGTTTCACATCCAACGACA	GCTTCAATCCGGTTGTGAGT	233	A	A	A	NA
PMS1785	Os_Chr03	18310302	18310331	(AT)15	(AT)15	(AT)15	(AT)15	(AT)15	(AT)14	(AT)15	(AT)15	(AT)15	(AT)15	(AT)15	-	Introns/Intergenic	-	-	TTTTCAAACTGTTAAATGGTGCAT	GAGGAGGAAGATTTGCATCG	174	P	A	A	NA
PMS1786	Os_Chr03	18314941	18314960	(AT)10	(AT)10	(AT)10	(AT)10	(AT)10	(AT)6	(AT)10	(AT)10	(AT)10	(AT)10	(AT)10	-	Introns/Intergenic	-	-	TCGGCTGATGATGATGTGAT	GGGGTGCTAGCCGATAAGTT	254	P	A	A	NA
PMS1787	Os_Chr03	18369730	18369744	(GGC)5	(GGC)6	(GGC)5	(GGC)5	(GGC)5	(GGC)5	(GGC)5	(GGC)5	(GGC)5	(GGC)5	(GGC)5	-	Introns/Intergenic	-	-	TCTCACTCTCTCTCTCCTCCT	CTCCTCCGCTCCCGTCACT	166	P	A	A	NA
PMS1788	Os_Chr03	18487344	18487365	(TA)11	(TA)11	(TA)6	(TA)11	(TA)11	(TA)11	(TA)7	(TA)11	(TA)6	(TA)7	(TA)11	LOC_Os03g32314	URR	OsAOC	-	GGCATCTTTAGTTGGTCCAAAGC	ACAACCATTCCGTTCAGTGTGC	224	P	A	P	RM15269
PMS1789	Os_Chr03	18601191	18601244	(TA)27	(TA)27	(TA)27	(TA)27	(TA)7	(TA)11	(TA)27	(TA)7	(TA)27	(TA)27	(TA)27	-	Introns/Intergenic	-	-	TCAATTTCAACAATGGTGCC	CGCTGTACAAAGTTCGTGGT	204	P	A	A	NA
PMS1790	Os_Chr03	18604728	18604741	(AG)7	(AG)7	(AG)7	(AG)7	(AG)7	(AG)7	(AG)7	(AG)6	(AG)7	(AG)7	(AG)7	-	Introns/Intergenic	-	-	AGCATTTGCACAGGAAAACC	AGGTTGGTGACCCAGTTCAG	203	A	A	A	NA
PMS1791	Os_Chr03	18629278	18629295	(CT)9	(CT)9	(CT)9	(CT)9	(CT)9	(CT)10	(CT)6	(CT)10	(CT)9	(CT)9	(CT)9	-	Introns/Intergenic	-	-	CACAGATATGTTCATCGGCG	TAGATCCACCCACGACAACA	246	P	A	A	NA
PMS1792	Os_Chr03	18635896	18635916	(CCA)7	(CCA)7	(CCA)7	(CCA)7	(CCA)7	(CCA)7	(CCA)6	(CCA)7	(CCA)7	(CCA)7	(CCA)7	LOC_Os03g32580	URR	BRASSINOSTEROID INSENSITIVE 1-associated receptor kinase 1 precursor, putative, expressed	-	AGTTGAAGTGTTGACCGCAATCC	GTAGGTAGAGGCTCTGGCAGTCG	176	A	A	A	RM15280
PMS1793	Os_Chr03	18859441	18859456	(CT)8	(CT)8	(CT)8	(CT)8	(CT)8	(CT)8	(CT)8	(CT)8	(CT)8	(CT)6	(CT)8	-	Introns/Intergenic	-	-	TCCTTGGTCTAAAAAGAGCCC	GGGAGAGAAGGAGAGGGAGA	242	A	A	A	NA

Polymorphic SSR (PMS) markers	Chromosomes	Start physical positions (bp)	End physical positions (bp)	Nipponbare	Kasalath	Nagina22	IR64	Pokkali	Bala	Tainung67	Azucena	Moroberekan	*Oryza rufipogon*	*Oryza nivara*	MSU gene locus IDs	Structural annotation	Functional annotation	SSR functional effects	Forward primers (5'-3')	Reverse primers (5'-3')	Amplified product size (bp)	Markers exhibiting polymorphism within *indica* rice	Transcrption factor genes-derived markers	Known cloned genes-derived markers	PMS markers corresponding with RM markers
PMS1794	Os_Chr03	19059164	19059179	(CT)8	(CT)8	(CT)9	(CT)9	(CT)9	(CT)8	(CT)8	(CT)8	(CT)8	(CT)8	(CT)8	-	Introns/Intergenic	-	-	CCTTGGTCTAAAATGAGCCCT	GGGAGAGAAGGAGAGGGAGA	229	P	A	A	NA
PMS1795	Os_Chr03	19146692	19146705	(GA)7	(GA)8	(GA)7	(GA)7	(GA)7	(GA)8	(GA)7	(GA)7	(GA)7	(GA)7	(GA)7	-	Introns/Intergenic	-	-	ACAACGATGAGAAGCACACG	CGCCACCTGGTTGAATTAGT	110	P	A	A	NA
PMS1796	Os_Chr03	19243397	19243444	(TA)24	(TA)24	(TA)6	(TA)24	(TA)12	(TA)11	(TA)24	(TA)24	(TA)24	(TA)18	(TA)9	-	Introns/Intergenic	-	-	TTGCTTACACTGTTTGACCACTCG	CACCTCTTTCTTTGGTGCCTTCC	301	P	A	A	RM15289
PMS1797	Os_Chr03	19362932	19362952	(GAC)7	(GAC)7	(GAC)7	(GAC)7	(GAC)7	(GAC)7	(GAC)7	(GAC)7	(GAC)7	(GAC)7	(GAC)5	-	Introns/Intergenic	-	-	GGACGAGACGGACGAGAAC	CCTCCATCCAAACCCCTC	278	A	A	A	NA
PMS1798	Os_Chr03	19412817	19412866	(CT)25	(CT)25	(CT)25	(CT)25	(CT)25	(CT)25	(CT)25	(CT)18	(CT)25	(CT)25	(CT)25	-	Introns/Intergenic	-	-	ATCGGCAAGCAATGTCATCAAGC	GTGCATGTGCAAGACACGTTCC	343	A	A	A	RM15293
PMS1799	Os_Chr03	19520244	19520267	(CCA)8	(CCA)8	(CCA)8	(CCA)8	(CCA)8	(CCA)8	(CCA)8	(CCA)8	(CCA)8	(CCA)7	(CCA)8	-	Introns/Intergenic	-	-	GAGAGTAGTGGTGGTTATCGTTCTGC	TAGCTATAAATGCCCGTGCTTGC	378	A	A	A	RM15296
PMS1800	Os_Chr03	19526434	19526457	(GGC)8	(GGC)8	(GGC)8	(GGC)8	(GGC)8	(GGC)5	(GGC)8	(GGC)8	(GGC)8	(GGC)8	(GGC)5	-	Introns/Intergenic	-	-	AGGCGACGATGGAAGGCTAGGG	TGACGATGTCTCCTCTTCGTTTCTCC	208	P	A	A	RM15297
PMS1801	Os_Chr03	19871482	19871547	(TA)33	(TA)33	(TA)33	(TA)33	(TA)33	(TA)33	(TA)33	(TA)10	(TA)33	(TA)33	(TA)33	-	Introns/Intergenic	-	-	CAAGCTAATCATGACCGATCAACC	GTGATATATCCAATAGGGACCCATGC	252	A	A	A	RM15302
PMS1802	Os_Chr03	20155760	20155773	(CT)7	(CT)7	(CT)6	(CT)7	(CT)7	(CT)7	(CT)7	(CT)7	(CT)7	(CT)7	(CT)7	-	Introns/Intergenic	-	-	CTCCCTCCTGGCTTCTTTCT	TCCCTTCTCCTCTCCCTCTC	143	P	A	A	NA
PMS1803	Os_Chr03	20296389	20296406	(GAA)6	(GAA)5	(GAA)6	(GAA)6	(GAA)6	(GAA)5	(GAA)6	(GAA)6	(GAA)6	(GAA)6	(GAA)5	LOC_Os03g36600	CDS	retrotransposon protein, putative, unclassified, expressed	CODON_DELETION	TCACCGACAGCTGTGTTCTC	TAGGTTGGTTCTCCCCCTTT	259	P	A	A	NA
PMS1804	Os_Chr03	20321077	20321092	(GA)8	(GA)8	(GA)8	(GA)8	(GA)8	(GA)8	(GA)7	(GA)8	(GA)8	(GA)8	(GA)8	-	Introns/Intergenic	-	-	AGAGACACGGCCATTTTGAC	GCTTGACAATCCAGTCAGCA	259	A	A	A	NA
PMS1805	Os_Chr03	20390889	20391032	(AT)72	(AT)72	(AT)72	(AT)72	(AT)9	(AT)72	(AT)72	(AT)7	(AT)13	(AT)72	(AT)72	-	Introns/Intergenic	-	-	CCATTGTGTTCGTTACAATTA	GACAAAGTTACTTCCGTTTTG	382	P	A	A	NA
PMS1806	Os_Chr03	20403093	20403113	(CGC)7	(CGC)9	(CGC)7	(CGC)7	(CGC)7	(CGC)9	(CGC)7	(CGC)7	(CGC)7	(CGC)7	(CGC)7	LOC_Os03g36780	URR	elongation factor, putative, expressed	-	CTCGACCAAACAGACCAGTAGGG	ATCGCCGCTTAGGAGAATCTGG	108	P	A	A	RM15315

Polymorphic SSR (PMS) markers	Chromosomes	Start physical positions (bp)	End physical positions (bp)	Nipponbare	Kasalath	Nagina22	IR64	Pokkali	Bala	Tainung67	Azucena	Moroberekan	*Oryza rufipogon*	*Oryza nivara*	MSU gene locus IDs	Structural annotation	Functional annotation	SSR functional effects	Forward primers (5'-3')	Reverse primers (5'-3')	Amplified product size (bp)	Markers exhibiting polymorphism within *indica* rice	Transcrption factor genes-derived markers	Known cloned genes-derived markers	PMS markers corresponding with RM markers
PMS1807	Os_Chr03	20439095	20439130	(TA)18	(TA)18	(TA)18	(TA)18	(TA)10	(TA)18	(TA)18	(TA)18	(TA)10	(TA)18	(TA)18	-	Introns/Intergenic	-	-	GTTTTATACCTTGGAGCCCG	GAGCTGAGAAGAGGTGGGTG	221	P	A	A	NA
PMS1808	Os_Chr03	20459216	20459229	(GA)7	(GA)6	(GA)7	(GA)7	(GA)7	(GA)7	(GA)7	(GA)7	(GA)7	(GA)7	(GA)7	-	Introns/Intergenic	-	-	GAAGGAGGGAGAGGAGGAGA	CATGAATTTTCAGAGCACCG	190	P	A	A	NA
PMS1809	Os_Chr03	20637176	20637189	(CT)7	(CT)7	(CT)7	(CT)7	(CT)8	(CT)7	(CT)7	(CT)7	(CT)7	(CT)10	(CT)7	-	Introns/Intergenic	-	-	CAGGATGCCACGTATCACAC	CTCCTCAGTCCTCCCTACCC	134	P	A	A	NA
PMS1810	Os_Chr03	20803564	20803595	(TA)16	(TA)16	(TA)16	(TA)16	(TA)16	(TA)16	(TA)16	(TA)16	(TA)7	(TA)16	(TA)16	-	Introns/Intergenic	-	-	CTGTGTGTAGTGGCATGTTACC	AACATTCCCACCTCTATCTAGC	376	A	A	A	RM15327
PMS1811	Os_Chr03	20820038	20820053	(CT)8	(CT)11	(CT)8	(CT)8	(CT)8	(CT)8	(CT)8	(CT)8	(CT)8	(CT)8	(CT)8	-	Introns/Intergenic	-	-	GGTCAGCTGAAATTCTTCGG	GCTACCTACCCAACCAAGGA	176	P	A	A	NA
PMS1812	Os_Chr03	20847603	20847622	(AT)10	(AT)9	(AT)10	(AT)10	(AT)10	(AT)9	(AT)10	(AT)10	(AT)10	(AT)10	(AT)10	-	Introns/Intergenic	-	-	CAACTTTTATGGGACGGAGG	TCAAATTGGGGTGTGACAAG	272	P	A	A	NA
PMS1813	Os_Chr03	20984577	20984674	(TA)49	(TA)47	(TA)49	(TA)10	(TA)49	(TA)49	(TA)8	(TA)10	(TA)9	(TA)49	(TA)49	-	Introns/Intergenic	-	-	GTACAGTATTCCCACGCACTGG	TCAGTCTGCCAAATTGATCAGC	286	P	A	A	RM15332
PMS1814	Os_Chr03	20987395	20987434	(TA)20	(TA)20	(TA)20	(TA)20	(TA)20	(TA)20	(TA)7	(TA)6	(TA)20	(TA)20	(TA)20	-	Introns/Intergenic	-	-	AGAGACTGTGGCCCAATATTCC	GCCAGAGTATCATTCAAACACACC	378	A	A	A	RM15333
PMS1815	Os_Chr03	21069004	21069033	(TA)15	(TA)15	(TA)15	(TA)6	(TA)15	(TA)15	(TA)15	(TA)15	(TA)13	(TA)7	(TA)15	-	Introns/Intergenic	-	-	AAACATCCTCATAGGTGCCG	AGAGTGAGCGAGAGAGGTGG	280	P	A	A	NA
PMS1816	Os_Chr03	21150553	21150576	(CTGC)6	(CTGC)5	(CTGC)5	(CTGC)6	(CTGC)6	(CTGC)6	(CTGC)6	(CTGC)6	(CTGC)6	(CTGC)6	(CTGC)6	-	Introns/Intergenic	-	-	CTTCAATAACCGTTTGCCTCACC	CCTTTCCAAACATACCCTACATCG	329	P	A	A	RM7370
PMS1817	Os_Chr03	21192266	21192327	(TA)31	(TA)31	(TA)13	(TA)11	(TA)31	(TA)31	(TA)31	(TA)6	(TA)12	(TA)27	(TA)6	-	Introns/Intergenic	-	-	ATGGTGCCCCATATGAGTGT	GACTCGTGAACCCCAGAAAA	113	P	A	A	NA
PMS1818	Os_Chr03	21273053	21273073	(CCG)7	(CCG)6	(CCG)6	(CCG)6	(CCG)7	(CCG)6	(CCG)7	(CCG)7	(CCG)7	(CCG)5	(CCG)6	-	Introns/Intergenic	-	-	AGAAGATTCTGCCGCTGGT	CTTATGTTGGGCCTTCCAGA	230	P	A	A	NA
PMS1819	Os_Chr03	21364128	21364148	(CCG)7	(CCG)7	(CCG)7	(CCG)7	(CCG)7	(CCG)7	(CCG)7	(CCG)5	(CCG)7	(CCG)5	(CCG)7	LOC_Os03g38490	URR	DAG protein, chloroplast precursor, putative, expressed	-	GCCACATCATCGCTAATCCATCC	AATCCGCTGCTACGGACTTGC	153	A	A	A	RM15350

Polymorphic SSR (PMS) markers	Chromosomes	Start physical positions (bp)	End physical positions (bp)	Nipponbare	Kasalath	Nagina22	IR64	Pokkali	Bala	Tainung67	Azucena	Moroberekan	*Oryza rufipogon*	*Oryza nivara*	MSU gene locus IDs	Structural annotation	Functional annotation	SSR functional effects	Forward primers (5'-3')	Reverse primers (5'-3')	Amplified product size (bp)	Markers exhibiting polymorphism within *indica* rice	Transcrption factor genes-derived markers	Known cloned genes-derived markers	PMS markers corresponding with RM markers
PMS1820	Os_Chr03	21392928	21392973	(AG)23	(AG)23	(AG)23	(AG)23	(AG)23	(AG)23	(AG)23	(AG)15	(AG)23	(AG)23	(AG)23	LOC_Os03g38534	CDS	expressed protein	FRAME_SHIFT	GGGTCAGCTGCACACATAGAACC	TCTTCAGCTCTCCCTCTCCTTCC	117	A	A	A	RM5488
PMS1821	Os_Chr03	21469697	21469714	(AGA)6	(AGA)6	(AGA)6	(AGA)6	(AGA)6	(AGA)6	(AGA)6	(AGA)6	(AGA)6	(AGA)5	(AGA)6	-	Introns/Intergenic	-	-	ACCGAAACAAAGATCGGATG	TACAGTGCATGGGTGAGAGC	119	A	A	A	NA
PMS1822	Os_Chr03	21645005	21645022	(GA)9	(GA)9	(GA)9	(GA)9	(GA)9	(GA)9	(GA)9	(GA)8	(GA)9	(GA)9	(GA)9	-	Introns/Intergenic	-	-	CCAAACGAGCTCTAACCCAC	TGGCATATGCTATGGTGGTG	274	A	A	A	NA
PMS1823	Os_Chr03	21685756	21685773	(GCC)6	(GCC)6	(GCC)6	(GCC)5	(GCC)6	(GCC)6	(GCC)6	(GCC)6	(GCC)6	(GCC)6	(GCC)5	LOC_Os03g39010	CDS	possible lysine decarboxylase domain containing protein, expressed	CODON_DELETION	CTCGTCTGTAGGAGCAGCG	GATCGAAGCACCTCGCTATC	255	P	A	A	NA
PMS1824	Os_Chr03	21756875	21756895	(CGC)7	(CGC)7	(CGC)7	(CGC)7	(CGC)6	(CGC)7	(CGC)7	(CGC)7	(CGC)7	(CGC)7	(CGC)7	LOC_Os03g39170	CDS	frigida, putative, expressed	CODON_CHANGE_PLUS_CODON_DELETION	CGTTTCACACTTCTCCGCTCTCG	GCCATCAGGATCGACTCCAACG	174	P	A	A	RM15364
PMS1825	Os_Chr03	21924018	21924041	(CAA)8	(CAA)8	(CAA)8	(CAA)8	(CAA)8	(CAA)8	(CAA)8	(CAA)8	(CAA)8	(CAA)6	(CAA)6	LOC_Os03g39450	CDS	retrotransposon protein, putative, Ty3-gypsy subclass, expressed	CODON_DELETION	ACACTTGCAGCAATGGAAATGG	GGCCTGTTGAGCTAGGACTTGG	92	A	A	A	RM15367
PMS1826	Os_Chr03	21931934	21931949	(TC)8	(TC)8	(TC)6	(TC)6	(TC)8	(TC)7	(TC)8	(TC)8	(TC)8	(TC)6	(TC)8	-	Introns/Intergenic	-	-	TCCTTCTTTTGGTTTTGGGC	GGTGGAAGACGACCCTAACA	156	P	A	A	NA
PMS1827	Os_Chr03	21984995	21985012	(GA)9	(GA)9	(GA)9	(GA)9	(GA)9	(GA)9	(GA)9	(GA)6	(GA)9	(GA)9	(GA)9	-	Introns/Intergenic	-	-	GAAAGGCCGATGACGAAG	AACGAAAATCAGTATGTGACAA	379	A	A	A	NA
PMS1828	Os_Chr03	21997851	21997878	(CT)14	(CT)14	(CT)14	(CT)14	(CT)10	(CT)14	(CT)14	(CT)14	(CT)14	(CT)14	(CT)14	-	Introns/Intergenic	-	-	GACACCGAGGTCCACAAGAGG	CTTGACACGGCAAGGCTATCG	387	P	A	A	RM3646
PMS1829	Os_Chr03	22007235	22007272	(TA)19	(TA)17	(TA)19	(TA)19	(TA)19	(TA)19	(TA)19	(TA)19	(TA)19	(TA)13	(TA)19	-	Introns/Intergenic	-	-	CAAGCGACAGTTCCTTGAGATCC	CCTCTACTCCGTCTTCCATCAGC	587	P	A	A	RM15373
PMS1830	Os_Chr03	22015970	22015981	(TA)6	(TA)6	(TA)6	(TA)6	(TA)6	(TA)6	(TA)6	(TA)6	(TA)6	(TA)7	(TA)6	-	Introns/Intergenic	-	-	TGGCAAATTTACGGGATAGG	ATGTGCTCCAGTACAACGCA	205	A	A	A	NA
PMS1831	Os_Chr03	22116316	22116333	(GCG)6	(GCG)6	(GCG)6	(GCG)6	(GCG)6	(GCG)6	(GCG)6	(GCG)5	(GCG)6	(GCG)6	(GCG)6	LOC_Os03g39740	URR	expressed protein	-	GCGATGTAATTTGCAGGGAT	GCAGTAGGGAGGAAAAACCC	189	A	A	A	NA
PMS1832	Os_Chr03	22249365	22249385	(TTG)7	(TTG)7	(TTG)7	(TTG)7	(TTG)7	(TTG)7	(TTG)7	(TTG)7	(TTG)7	(TTG)7	(TTG)5	-	Introns/Intergenic	-	-	GCTTGTCTTGGCGTGTGTTAGC	CTCGGCAGGACCCACTATGTACC	275	A	A	A	RM15386

Polymorphic SSR (PMS) markers	Chromosomes	Start physical positions (bp)	End physical positions (bp)	Nipponbare	Kasalath	Nagina22	IR64	Pokkali	Bala	Tainung67	Azucena	Moroberekan	*Oryza rufipogon*	*Oryza nivara*	MSU gene locus IDs	Structural annotation	Functional annotation	SSR functional effects	Forward primers (5'-3')	Reverse primers (5'-3')	Amplified product size (bp)	Markers exhibiting polymorphism within *indica* rice	Transcrption factor genes-derived markers	Known cloned genes-derived markers	PMS markers corresponding with RM markers
PMS1833	Os_Chr03	22258843	22258866	(GATC)6	(GATC)6	(GATC)5	(GATC)6	(GATC)6	(GATC)6	(GATC)6	(GATC)6	(GATC)6	(GATC)6	(GATC)6	-	Introns/Intergenic	-	-	GTTGTAGTGCGGGCAGTTCTCC	CGTACTGTTTGCCTCTGCTTGC	134	P	A	A	RM7395
PMS1834	Os_Chr03	22274057	22274074	(AAG)6	(AAG)7	(AAG)6	(AAG)6	(AAG)7	(AAG)7	(AAG)6	(AAG)6	(AAG)6	(AAG)6	(AAG)6	-	Introns/Intergenic	-	-	CGAAAAGTACTGTCACCCCA	CTTTTTGAGCAAAGACCAGGA	235	P	A	A	NA
PMS1835	Os_Chr03	22297806	22297823	(AT)9	(AT)9	(AT)9	(AT)9	(AT)9	(AT)9	(AT)8	(AT)9	(AT)9	(AT)10	(AT)9	-	Introns/Intergenic	-	-	TCCTCCGTTTCACAACGTAA	TGAACTACTCACTCCGTTTCACA	155	A	A	A	NA
PMS1836	Os_Chr03	22323689	22323704	(AG)8	(AG)8	(AG)8	(AG)8	(AG)8	(AG)8	(AG)8	(AG)8	(AG)8	(AG)6	(AG)7	-	Introns/Intergenic	-	-	GGGAAGCTACTGCCATCAAT	AAGACGGACGGTCAAACATT	184	A	A	A	NA
PMS1837	Os_Chr03	22340971	22340988	(CAA)6	(CAA)6	(CAA)6	(CAA)6	(CAA)6	(CAA)6	(CAA)6	(CAA)6	(CAA)6	(CAA)6	(CAA)5	-	Introns/Intergenic	-	-	TTTGGTTGGTCGTTTTGGTT	CCTTGCCCTTGCTGTAAAAG	278	A	A	A	NA
PMS1838	Os_Chr03	22392947	22392970	(ATC)8	(ATC)5	(ATC)8	(ATC)8	(ATC)8	(ATC)8	(ATC)8	(ATC)8	(ATC)8	(ATC)8	(ATC)8	-	Introns/Intergenic	-	-	AGGGAGTAACTAACAAGGTCACAAGG	GCATAAATTCAGCTCCGTACAAGG	191	P	A	A	RM5864
PMS1839	Os_Chr03	22517847	22517862	(GA)8	(GA)6	(GA)8	(GA)8	(GA)8	(GA)8	(GA)8	(GA)8	(GA)8	(GA)6	(GA)8	-	Introns/Intergenic	-	-	TCGTTCTTCGTTCGTTCAC	CATCTCTGGGGTGAGAGCC	280	P	A	A	NA
PMS1840	Os_Chr03	22586232	22586247	(AT)8	(AT)8	(AT)8	(AT)8	(AT)8	(AT)8	(AT)8	(AT)8	(AT)8	(AT)8	(AT)6	-	Introns/Intergenic	-	-	AGGGTTTAGGAATCAGGAGAAGG	ATCTCTCCTTGTAACGCATGAGC	355	A	A	A	RM15416
PMS1841	Os_Chr03	22603703	22603726	(CCT)8	(CCT)8	(CCT)8	(CCT)8	(CCT)8	(CCT)8	(CCT)6	(CCT)8	(CCT)8	(CCT)9	(CCT)8	LOC_Os03g40650	CDS	bromodomain associated family protein, expressed	CODON_INSERTION	GAACTGTATGACGTCGCACTCG	CCCTAAACCCTAGATTTCCATCC	496	A	A	A	RM15417
PMS1842	Os_Chr03	22607474	22607530	(TTA)19	(TTA)19	(TTA)19	(TTA)19	(TTA)19	(TTA)19	(TTA)16	(TTA)6	(TTA)19	(TTA)19	(TTA)19	-	Introns/Intergenic	-	-	GGCGAGAGGGAGAGAGAAGAAAGG	TTTGACCAAGAGCTTGCACACG	157	A	A	A	RM15418
PMS1843	Os_Chr03	22668976	22668993	(CGG)6	(CGG)6	(CGG)6	(CGG)6	(CGG)6	(CGG)6	(CGG)6	(CGG)6	(CGG)6	(CGG)5	(CGG)6	LOC_Os03g40770	CDS	expressed protein	CODON_CHANGE_PLUS_CODON_DELETION	TTCGATGTGCTACATGACGG	TAGCACGCTACCTACTGCCA	217	A	A	A	NA
PMS1844	Os_Chr03	22683493	22683524	(AT)16	(AT)6	(AT)6	(AT)16	(AT)16	(AT)6	(AT)16	(AT)16	(AT)16	(AT)6	(AT)16	-	Introns/Intergenic	-	-	CAGCCCATATATTTGTCGTGAACC	CTAATGTCCCAAGATCGTGTTGC	259	P	A	A	RM15422
PMS1845	Os_Chr03	22708892	22708903	(GA)6	(GA)6	(GA)6	(GA)7	(GA)7	(GA)7	(GA)6	(GA)6	(GA)6	(GA)6	(GA)6	-	Introns/Intergenic	-	-	AACAATGAGATGGTGTGGCA	TGCGTCCAAGATGTCTGTTC	173	P	A	A	NA

Polymorphic SSR (PMS) markers	Chromosomes	Start physical positions (bp)	End physical positions (bp)	Nipponbare	Kasalath	Nagina22	IR64	Pokkali	Bala	Tainung67	Azucena	Moroberekan	*Oryza rufipogon*	*Oryza nivara*	MSU gene locus IDs	Structural annotation	Functional annotation	SSR functional effects	Forward primers (5'-3')	Reverse primers (5'-3')	Amplified product size (bp)	Markers exhibiting polymorphism within *indica* rice	Transcrption factor genes-derived markers	Known cloned genes-derived markers	PMS markers corresponding with RM markers
PMS1846	Os_Chr03	22759607	22759663	(TAT)19	(TAT)19	(TAT)14	(TAT)19	(TAT)19	(TAT)19	(TAT)19	(TAT)18	(TAT)19	(TAT)19	(TAT)19	-	Introns/Intergenic	-	-	ACTAAACTCCCTTCGCCCAACC	TCGTATGCACGTATGTCTTCTTGG	489	P	A	A	RM15424
PMS1847	Os_Chr03	22807361	22807376	(CT)8	(CT)10	(CT)10	(CT)8	(CT)8	(CT)8	(CT)8	(CT)8	(CT)8	(CT)8	(CT)8	-	Introns/Intergenic	-	-	GGCCAGTATTCTCCGTCAAA	CCAGAATTCGAGACGTTGGT	149	P	A	A	NA
PMS1848	Os_Chr03	22826514	22826537	(CGG)8	(CGG)6	(CGG)8	(CGG)8	(CGG)8	(CGG)8	(CGG)8	(CGG)8	(CGG)8	(CGG)8	(CGG)8	LOC_Os03g41070	CDS	metal transporter Nramp2, putative, expressed	CODON_DELETION	GTCGGAGTAGGCGGAGAACAGG	GAGAAGATGGCTTGAGATAGCATTGG	315	P	A	A	RM15427
PMS1849	Os_Chr03	22853180	22853195	(AC)8	(AC)8	(AC)8	(AC)6	(AC)6	(AC)8	(AC)8	(AC)8	(AC)8	(AC)8	(AC)6	-	Introns/Intergenic	-	-	AAATTTAGTCCCCACCCCAC	GCAACCCTGTCGTTGAAACT	215	P	A	A	NA
PMS1850	Os_Chr03	22906323	22906382	(AT)30	(AT)30	(AT)11	(AT)30	(AT)9	(AT)30	(AT)30	(AT)30	(AT)30	(AT)30	(AT)9	-	Introns/Intergenic	-	-	ATATAAGATTAGCCGCGATTT	TTGCGTACCGTAATATTAAAAC	382	P	A	A	NA
PMS1851	Os_Chr03	23068857	23068874	(CTA)6	(CTA)6	(CTA)6	(CTA)7	(CTA)7	(CTA)5	(CTA)6	(CTA)6	(CTA)6	(CTA)7	(CTA)6	LOC_Os03g41460	DRR	SAPK10	-	CTGGGGGCATCTCTTATTCA	CGCCGTTGATTTGTGTATTG	149	P	A	P	NA
PMS1852	Os_Chr03	23099210	23099241	(TA)16	(TA)16	(TA)7	(TA)16	(TA)6	(TA)16	(TA)16	(TA)16	(TA)16	(TA)9	(TA)16	-	Introns/Intergenic	-	-	ATTGGAGAAGAAGTGGAGGTAGG	GGTCTCCGGTCTCAAGTACTCC	483	P	A	A	RM15443
PMS1853	Os_Chr03	23112791	23112868	(TA)39	(TA)39	(TA)39	(TA)7	(TA)7	(TA)13	(TA)8	(TA)6	(TA)39	(TA)39	(TA)8	-	Introns/Intergenic	-	-	GCTGTGACAAACATGCCCTA	CAACCATTGGGCTCAATTAAA	279	P	A	A	NA
PMS1854	Os_Chr03	23125840	23125893	(TTA)18	(TTA)18	(TTA)18	(TTA)18	(TTA)18	(TTA)9	(TTA)5	(TTA)14	(TTA)18	(TTA)18	(TTA)18	-	Introns/Intergenic	-	-	GGGTTTGTTGACATACATAGGCTTGC	GTGTAACAGGTGATCGACATTGAGG	477	P	A	A	RM15446
PMS1855	Os_Chr03	23334906	23334925	(AT)10	(AT)10	(AT)10	(AT)10	(AT)10	(AT)10	(AT)10	(AT)10	(AT)10	(AT)10	(AT)8	-	Introns/Intergenic	-	-	AGCGTTCGAATTGCAGAAGTGG	GAGGCTAACAGAGGATGGGAAGG	627	A	A	A	RM15454
PMS1856	Os_Chr03	23423068	23423095	(TA)14	(TA)6	(TA)14	(TA)6	(TA)14	(TA)11	(TA)14	(TA)9	(TA)14	(TA)14	(TA)14	-	Introns/Intergenic	-	-	ATCCCTCACCCTTATCCTCTCC	GGTAAGTCAAACATCTAGCGAAGG	408	P	A	A	RM15458
PMS1857	Os_Chr03	23446121	23446138	(CGG)6	(CGG)6	(CGG)6	(CGG)6	(CGG)6	(CGG)6	(CGG)6	(CGG)6	(CGG)6	(CGG)7	(CGG)6	LOC_Os03g42120	CDS	expressed protein	CODON_INSERTION	GTGGTGCATGACGGAGTTC	CTGCAGGCTTGCCTCTACTT	127	A	A	A	NA
PMS1858	Os_Chr03	23452166	23452179	(GT)7	(GT)7	(GT)7	(GT)7	(GT)7	(GT)7	(GT)7	(GT)7	(GT)7	(GT)7	(GT)6	LOC_Os03g42130	URR	gibberellin 20 oxidase 2, putative, expressed	-	GCACAAGTGTTCGTCTCGTG	TCATTGAATCCAGCCATGAA	128	A	A	A	NA

Polymorphic SSR (PMS) markers	Chromosomes	Start physical positions (bp)	End physical positions (bp)	Nipponbare	Kasalath	Nagina22	IR64	Pokkali	Bala	Tainung67	Azucena	Moroberekan	*Oryza rufipogon*	*Oryza nivara*	MSU gene locus IDs	Structural annotation	Functional annotation	SSR functional effects	Forward primers (5'-3')	Reverse primers (5'-3')	Amplified product size (bp)	Markers exhibiting polymorphism within *indica* rice	Transcrption factor genes-derived markers	Known cloned genes-derived markers	PMS markers corresponding with RM markers
PMS1859	Os_Chr03	23485597	23485614	(CCG)6	(CCG)6	(CCG)6	(CCG)6	(CCG)6	(CCG)6	(CCG)5	(CCG)6	(CCG)6	(CCG)6	(CCG)6	LOC_Os03g42220	URR	T-complex protein, putative, expressed	-	TTTCCAAGAGCAAAAATGGG	CAATTACGCTGCACTGTTGG	261	A	A	A	NA
PMS1860	Os_Chr03	23514729	23514743	(AGG)5	(AGG)5	(AGG)5	(AGG)6	(AGG)6	(AGG)6	(AGG)5	(AGG)5	(AGG)5	(AGG)5	(AGG)5	-	Introns/Intergenic	-	-	AGAAGCCGATCCCATCTACA	CTTCATCAGCAGCGGTAGTG	202	P	A	A	NA
PMS1861	Os_Chr03	23579700	23579765	(AT)33	(AT)33	(AT)6	(AT)33	(AT)10	(AT)14	(AT)33	(AT)33	(AT)9	(AT)33	(AT)7	-	Introns/Intergenic	-	-	TTAGGACGAAGGGAGTATATCG	CTTCTTCTTCCTTCTCTTCTGC	278	P	A	A	RM15467
PMS1862	Os_Chr03	23611388	23611405	(GGA)6	(GGA)6	(GGA)6	(GGA)6	(GGA)6	(GGA)6	(GGA)5	(GGA)6	(GGA)6	(GGA)6	(GGA)6	LOC_Os03g42420	CDS	B3 DNA binding domain containing protein, expressed	CODON_DELETION	TGGTGCATTTCTTTTGGTCA	ACCAACCTGAGCTCGAAGAG	239	A	P	A	NA
PMS1863	Os_Chr03	23671317	23671340	(TC)12	(TC)10	(TC)12	(TC)12	(TC)12	(TC)12	(TC)12	(TC)12	(TC)12	(TC)12	(TC)12	-	Introns/Intergenic	-	-	GAGGCAGACAGATTTGTGCAAGC	GGCAGCAAGGCCATACAATAAGC	405	P	A	A	RM15476
PMS1864	Os_Chr03	23755827	23755838	(TA)6	(TA)6	(TA)6	(TA)6	(TA)6	(TA)6	(TA)6	(TA)6	(TA)6	(TA)7	(TA)6	-	Introns/Intergenic	-	-	ATGTGAATGGTACACGGGCT	GATTGTAAAATTACTCGCATATTGG	106	A	A	A	NA
PMS1865	Os_Chr03	23827669	23827695	(GAG)9	(GAG)7	(GAG)9	(GAG)7	(GAG)9	(GAG)8	(GAG)9	(GAG)8	(GAG)8	(GAG)9	(GAG)7	LOC_Os03g42780	CDS	zinc finger protein-related, putative, expressed	CODON_DELETION	ACGAGGAGTGCTTCTACGACTACG	GGCTTTACTCACCCAGCAATCG	146	P	A	A	RM6425
PMS1866	Os_Chr03	23884469	23884482	(AG)7	(AG)7	(AG)7	(AG)7	(AG)7	(AG)7	(AG)7	(AG)7	(AG)7	(AG)6	(AG)7	-	Introns/Intergenic	-	-	GTGGTGTAGTGATTGCACGG	GGCAGTAATCTTTGTCCCCA	238	A	A	A	NA
PMS1867	Os_Chr03	23888855	23888872	(CGC)6	(CGC)6	(CGC)5	(CGC)6	(CGC)6	(CGC)6	(CGC)6	(CGC)6	(CGC)6	(CGC)5	(CGC)6	LOC_Os03g42840	URR	calcineurin B, putative, expressed	-	ATCCAATCCACTCCACCGTA	AGGAAGCGCGAAGTAGGAG	112	P	A	A	NA
PMS1868	Os_Chr03	23900825	23900842	(CGC)6	(CGC)6	(CGC)6	(CGC)6	(CGC)6	(CGC)5	(CGC)6	(CGC)6	(CGC)6	(CGC)6	(CGC)6	-	Introns/Intergenic	-	-	GCCACAGCAAGCCTCATATC	AAGAATAGCAGGGAGCAGCC	262	P	A	A	NA
PMS1869	Os_Chr03	23901627	23901656	(TA)15	(TA)15	(TA)9	(TA)15	(TA)15	(TA)15	(TA)15	(TA)9	(TA)15	(TA)15	(TA)15	-	Introns/Intergenic	-	-	GCAGAGATCTATAGGTGCCACTCC	CGTACTGTTGCTGCCTTAGATCG	242	P	A	A	RM15495
PMS1870	Os_Chr03	23903929	23903944	(TA)8	(TA)8	(TA)7	(TA)8	(TA)8	(TA)8	(TA)8	(TA)8	(TA)8	(TA)8	(TA)8	-	Introns/Intergenic	-	-	CATGACCCAGCAAAGATCAA	TCGATAGCAAGATGCATGTGA	270	P	A	A	NA
PMS1871	Os_Chr03	23921362	23921379	(AT)9	(AT)7	(AT)6	(AT)7	(AT)8	(AT)9	(AT)9	(AT)9	(AT)9	(AT)9	(AT)9	-	Introns/Intergenic	-	-	GAAAGTCACAAGTCACTTCAACAA	GCTCATGTGTGTATGCTCCG	279	P	A	A	NA

Polymorphic SSR (PMS) markers	Chromosomes	Start physical positions (bp)	End physical positions (bp)	Nipponbare	Kasalath	Nagina22	IR64	Pokkali	Bala	Tainung67	Azucena	Moroberekan	*Oryza rufipogon*	*Oryza nivara*	MSU gene locus IDs	Structural annotation	Functional annotation	SSR functional effects	Forward primers (5'-3')	Reverse primers (5'-3')	Amplified product size (bp)	Markers exhibiting polymorphism within *indica* rice	Transcrption factor genes-derived markers	Known cloned genes-derived markers	PMS markers corresponding with RM markers
PMS1872	Os_Chr03	24068697	24068714	(CT)9	(CT)9	(CT)9	(CT)9	(CT)9	(CT)7	(CT)9	(CT)9	(CT)9	(CT)9	(CT)9	-	Introns/Intergenic	-	-	TGCCTCTGCCTTTCTGATCT	GCTGGAATGGAGAGCGTTAG	241	P	A	A	NA
PMS1873	Os_Chr03	24112223	24112243	(TTC)7	(TTC)7	(TTC)7	(TTC)7	(TTC)7	(TTC)7	(TTC)7	(TTC)7	(TTC)7	(TTC)7	(TTC)5	LOC_Os03g43240	CDS	retrotransposon protein, putative, Ty3-gypsy subclass	CODON_DELETION	ATCGGCTAACTCATCCGGAATTTGG	GACACCAGGTGACATCGGCTACC	296	A	A	A	RM15509
PMS1874	Os_Chr03	24281462	24281511	(TA)25	(TA)25	(TA)25	(TA)25	(TA)25	(TA)25	(TA)25	(TA)8	(TA)6	(TA)25	(TA)25	-	Introns/Intergenic	-	-	GGTGAAGAACCTTCCTCCTTTATACC	TCATACTGTCCTGGACCACATCC	166	A	A	A	RM15521
PMS1875	Os_Chr03	24302802	24302815	(AG)7	(AG)8	(AG)7	(AG)7	(AG)7	(AG)8	(AG)7	(AG)7	(AG)7	(AG)7	(AG)7	-	Introns/Intergenic	-	-	CGTCATCGTCGTCCTTCTC	ATGCCGTGGTGTAGGTTCTC	273	P	A	A	NA
PMS1876	Os_Chr03	24311832	24311851	(GA)10	(GA)10	(GA)10	(GA)10	(GA)7	(GA)10	(GA)10	(GA)8	(GA)10	(GA)10	(GA)10	-	Introns/Intergenic	-	-	GCAGGGAGTGAGGAAGAGTTGG	CAAAGCCCATTAGCCCACTAAGC	95	P	A	A	RM15522
PMS1877	Os_Chr03	24362604	24362624	(CTG)7	(CTG)6	(CTG)5	(CTG)5	(CTG)5	(CTG)5	(CTG)7	(CTG)7	(CTG)7	(CTG)7	(CTG)7	-	Introns/Intergenic	-	-	GTACTCCAACGCACAGTTGATCG	AGAAGGTCGATGCAGAAGAGACG	97	P	A	A	RM15526
PMS1878	Os_Chr03	24420109	24420132	(TA)12	(TA)12	(TA)12	(TA)12	(TA)12	(TA)11	(TA)12	(TA)12	(TA)12	(TA)12	(TA)12	-	Introns/Intergenic	-	-	CCTGGATTACTTTACCATGCATCC	CCTGCCTTGATTATGTCGAAAGC	241	P	A	A	RM15528
PMS1879	Os_Chr03	24442941	24442954	(CT)7	(CT)7	(CT)7	(CT)7	(CT)7	(CT)7	(CT)7	(CT)7	(CT)8	(CT)7	(CT)7	-	Introns/Intergenic	-	-	GACGCATACGTGGCAGTCTA	GCTCTGCTTATGCCTCTGCT	280	A	A	A	NA
PMS1880	Os_Chr03	24514011	24514034	(AT)12	(AT)11	(AT)12	(AT)12	(AT)12	(AT)12	(AT)12	(AT)12	(AT)8	(AT)12	(AT)12	-	Introns/Intergenic	-	-	CACAGCTCGATCGGCACTTCG	CAGGCCCGACACAAACAATAGGG	384	P	A	A	RM15531
PMS1881	Os_Chr03	24536482	24536497	(AT)8	(AT)8	(AT)8	(AT)8	(AT)8	(AT)8	(AT)8	(AT)7	(AT)8	(AT)8	(AT)8	-	Introns/Intergenic	-	-	CAGGAAGGAAGGCATGTGAT	CAAACCCTGACCACACACAC	248	A	A	A	NA
PMS1882	Os_Chr03	24645804	24645833	(TA)15	(TA)15	(TA)6	(TA)7	(TA)15	(TA)15	(TA)6	(TA)13	(TA)7	(TA)10	(TA)11	-	Introns/Intergenic	-	-	GGCTATTTGTGGTCATCTGTTGG	CCAGAGACCAAGGAAGAAAGAAAGG	186	P	A	A	RM15542
PMS1883	Os_Chr03	24660633	24660646	(AG)7	(AG)6	(AG)7	(AG)7	(AG)7	(AG)7	(AG)7	(AG)7	(AG)7	(AG)7	(AG)6	-	Introns/Intergenic	-	-	GGAAGGGGTGTTACAGTGGA	CCCATCCTAAATGGCTCAAA	154	P	A	A	NA
PMS1884	Os_Chr03	24682317	24682332	(AG)8	(AG)8	(AG)8	(AG)8	(AG)8	(AG)8	(AG)8	(AG)8	(AG)8	(AG)10	(AG)8	-	Introns/Intergenic	-	-	GTCACTGCCATGATGAGAGC	ATCCCATAGAGGTGTGGCTG	217	A	A	A	NA

Polymorphic SSR (PMS) markers	Chromosomes	Start physical positions (bp)	End physical positions (bp)	Nipponbare	Kasalath	Nagina22	IR64	Pokkali	Bala	Tainung67	Azucena	Moroberekan	*Oryza rufipogon*	*Oryza nivara*	MSU gene locus IDs	Structural annotation	Functional annotation	SSR functional effects	Forward primers (5'-3')	Reverse primers (5'-3')	Amplified product size (bp)	Markers exhibiting polymorphism within *indica* rice	Transcrption factor genes-derived markers	Known cloned genes-derived markers	PMS markers corresponding with RM markers
PMS1885	Os_Chr03	24733203	24733218	(TA)8	(TA)8	(TA)8	(TA)7	(TA)7	(TA)7	(TA)8	(TA)7	(TA)7	(TA)8	(TA)8	-	Introns/Intergenic	-	-	ACCCCTGCCCTGATTAATTT	GTTTGACGCCGTTGACTTTT	247	P	A	A	NA
PMS1886	Os_Chr03	24733821	24733854	(AG)17	(AG)18	(AG)17	(AG)17	(AG)17	(AG)17	(AG)17	(AG)17	(AG)17	(AG)17	(AG)17	-	Introns/Intergenic	-	-	GAGTTGTGTCAGTGAAGGTTGTCG	GATGCCCTTGATTTCTGAACAGG	188	P	A	A	RM15555
PMS1887	Os_Chr03	24743297	24743314	(CT)9	(CT)9	(CT)9	(CT)9	(CT)9	(CT)9	(CT)9	(CT)9	(CT)9	(CT)6	(CT)9	-	Introns/Intergenic	-	-	GATGTGGGAGACAGCAGTGA	TCATCGGAATTGTGAACCCT	280	A	A	A	NA
PMS1888	Os_Chr03	24775339	24775358	(AT)10	(AT)10	(AT)10	(AT)10	(AT)10	(AT)10	(AT)10	(AT)10	(AT)10	(AT)9	(AT)10	-	Introns/Intergenic	-	-	AACGAGCTAGGGAACGTGAA	GCTCCAATTCCTTCTAGGGC	184	A	A	A	NA
PMS1889	Os_Chr03	24797867	24797884	(CGT)6	(CGT)7	(CGT)6	(CGT)6	(CGT)6	(CGT)6	(CGT)6	(CGT)6	(CGT)6	(CGT)6	(CGT)6	LOC_Os03g44140	CDS	OsWAK26 - OsWAK receptor-like protein kinase, expressed	CODON_INSERTION	CTTGTCCGTCTCCATGCTCT	AGGTGTTGAGGTGGTGGATG	115	P	A	A	NA
PMS1890	Os_Chr03	24854211	24854228	(GGT)6	(GGT)6	(GGT)6	(GGT)6	(GGT)6	(GGT)6	(GGT)6	(GGT)6	(GGT)6	(GGT)5	(GGT)6	LOC_Os03g44230	URR	OsProT|ProT	-	CCCGTGCCATATAGGTAGGA	GGGAAAGACGGGAAAATCTC	166	A	A	P	NA
PMS1891	Os_Chr03	24866348	24866380	(TCT)11	(TCT)11	(TCT)11	(TCT)11	(TCT)6	(TCT)11	(TCT)11	(TCT)11	(TCT)11	(TCT)11	(TCT)11	-	Introns/Intergenic	-	-	GGACGCCACCTTCCTCTTCTGC	CGGTCATAAACGCCATTAGACCAAGC	94	P	A	A	RM5626
PMS1892	Os_Chr03	24895137	24895148	(CT)6	(CT)11	(CT)6	(CT)6	(CT)6	(CT)6	(CT)6	(CT)6	(CT)6	(CT)11	(CT)11	-	Introns/Intergenic	-	-	CCCTCAGTCGGTTCACATTT	ACCTGCCCACTCAAGAACAC	211	P	A	A	NA
PMS1893	Os_Chr03	24918186	24918223	(GA)19	(GA)19	(GA)19	(GA)19	(GA)19	(GA)19	(GA)19	(GA)17	(GA)19	(GA)19	(GA)19	-	Introns/Intergenic	-	-	TCAGAAGCAGTGAGAGGGGT	GGCATCACATGACCAACCTA	216	A	A	A	NA
PMS1894	Os_Chr03	24994836	24994847	(CT)6	(CT)6	(CT)6	(CT)6	(CT)6	(CT)6	(CT)6	(CT)6	(CT)6	(CT)7	(CT)6	-	Introns/Intergenic	-	-	CCCATCACGCTTCCAATAGT	ATTAGCTCGAATCCCCGTCT	176	A	A	A	NA
PMS1895	Os_Chr03	25020389	25020414	(GT)13	(GT)13	(GT)6	(GT)6	(GT)6	(GT)13	(GT)13	(GT)13	(GT)13	(GT)13	(GT)13	-	Introns/Intergenic	-	-	CACTATGTGGCCGGTCTGA	GCAAAGGGAAAGAAGACGGT	257	P	A	A	NA
PMS1896	Os_Chr03	25030596	25030627	(GT)16	(GT)16	(GT)16	(GT)16	(GT)16	(GT)6	(GT)16	(GT)16	(GT)16	(GT)16	(GT)16	-	Introns/Intergenic	-	-	GTCGGCTCTGTATCAGCCATCC	GCAAAGCAAAGGGAAAGAAGAAGG	369	P	A	A	RM15574
PMS1897	Os_Chr03	25101734	25101757	(TAT)8	(TAT)8	(TAT)8	(TAT)8	(TAT)8	(TAT)5	(TAT)8	(TAT)8	(TAT)8	(TAT)8	(TAT)5	-	Introns/Intergenic	-	-	ACCCAACAGTGGGTATATTAGG	TGTTTCTTAGTCGGTCTTGC	217	P	A	A	RM15575

Polymorphic SSR (PMS) markers	Chromosomes	Start physical positions (bp)	End physical positions (bp)	Nipponbare	Kasalath	Nagina22	IR64	Pokkali	Bala	Tainung67	Azucena	Moroberekan	*Oryza rufipogon*	*Oryza nivara*	MSU gene locus IDs	Structural annotation	Functional annotation	SSR functional effects	Forward primers (5'-3')	Reverse primers (5'-3')	Amplified product size (bp)	Markers exhibiting polymorphism within *indica* rice	Transcrption factor genes-derived markers	Known cloned genes-derived markers	PMS markers corresponding with RM markers
PMS1898	Os_Chr03	25139685	25139699	(CGG)5	(CGG)6	(CGG)6	(CGG)5	(CGG)5	(CGG)5	(CGG)5	(CGG)5	(CGG)5	(CGG)5	(CGG)5	LOC_Os03g44650	CDS	glutaredoxin, putative, expressed	CODON_INSERTION	CAGTGCTTGCAAGAATGAGC	AGCGGCAGTCATAGGCTCT	137	P	A	A	NA
PMS1899	Os_Chr03	25179679	25179766	(TA)44	(TA)44	(TA)44	(TA)44	(TA)9	(TA)44	(TA)9	(TA)8	(TA)44	(TA)44	(TA)44	-	Introns/Intergenic	-	-	CCCAAATAGTCACCAGCATTATCG	TTGCCTGTGCAACCTTATGAACC	103	P	A	A	RM15583
PMS1900	Os_Chr03	25228174	25228197	(GGT)8	(GGT)7	(GGT)8	(GGT)8	(GGT)8	(GGT)8	(GGT)8	(GGT)8	(GGT)8	(GGT)7	(GGT)8	LOC_Os03g44750	CDS	pollen preferential protein, putative, expressed	CODON_DELETION	AGGAGGTCTCGGAGCGGT	CCAATAATGGGGAGAGGCTT	244	P	A	A	NA
PMS1901	Os_Chr03	25233380	25233397	(AT)9	(AT)11	(AT)11	(AT)9	(AT)9	(AT)9	(AT)8	(AT)9	(AT)9	(AT)9	(AT)9	-	Introns/Intergenic	-	-	TGCTACGGTAACAAGGGGTC	GCTACACCCGTTTCGGTAAA	262	P	A	A	NA
PMS1902	Os_Chr03	25383027	25383058	(CT)16	(CT)16	(CT)16	(CT)9	(CT)16	(CT)16	(CT)16	(CT)16	(CT)16	(CT)16	(CT)16	-	Introns/Intergenic	-	-	CTGGAAGGAGAGCTGCACAGG	GCATTTGCTTGACCACCAGTCC	173	P	A	A	RM15602
PMS1903	Os_Chr03	25410499	25410512	(GA)7	(GA)6	(GA)7	(GA)7	(GA)7	(GA)7	(GA)7	(GA)7	(GA)7	(GA)7	(GA)7	-	Introns/Intergenic	-	-	GGAGGAACGAAGCTAGGGTT	ACTCCCTCCTCCTCCCTCTC	234	P	A	A	NA
PMS1904	Os_Chr03	25527867	25527890	(TA)12	(TA)12	(TA)9	(TA)9	(TA)12	(TA)12	(TA)12	(TA)12	(TA)6	(TA)12	(TA)12	-	Introns/Intergenic	-	-	AGCTTGCTGAACACCGTCAT	CTCCGAGGTAACGAGAGCTG	274	P	A	A	NA
PMS1905	Os_Chr03	25566469	25566492	(CGG)8	(CGG)6	(CGG)8	(CGG)8	(CGG)8	(CGG)8	(CGG)8	(CGG)8	(CGG)8	(CGG)5	(CGG)8	-	Introns/Intergenic	-	-	CTATCCCGTCCTCCGTCAATTCG	ACACACCCTGCAGCTGTTCTGC	180	P	A	A	RM15608
PMS1906	Os_Chr03	25613905	25613918	(CT)7	(CT)6	(CT)7	(CT)7	(CT)7	(CT)7	(CT)7	(CT)7	(CT)7	(CT)6	(CT)7	LOC_Os03g45370	URR	sodium/calcium exchanger protein, putative, expressed	-	CAATCCCCAATCTTTTTCCA	GAGCTCACCTCAGCTCATCA	231	P	A	A	NA
PMS1907	Os_Chr03	25759448	25759459	(AG)6	(AG)7	(AG)6	(AG)6	(AG)6	(AG)6	(AG)6	(AG)6	(AG)6	(AG)6	(AG)6	-	Introns/Intergenic	-	-	CCTCTGGTGGGAGAGAAGAA	CAAAACATCTCCTGGCCACT	180	P	A	A	NA
PMS1908	Os_Chr03	25879995	25880024	(AT)15	(AT)15	(AT)6	(AT)15	(AT)15	(AT)15	(AT)15	(AT)15	(AT)6	(AT)15	(AT)15	-	Introns/Intergenic	-	-	TTTGTTTAGCAGGAGGACCATGC	AGCCGGTGATCACGATTTGG	212	P	A	A	RM15620
PMS1909	Os_Chr03	25908975	25908992	(CGT)6	(CGT)6	(CGT)6	(CGT)6	(CGT)6	(CGT)6	(CGT)6	(CGT)6	(CGT)6	(CGT)6	(CGT)5	LOC_Os03g45870	CDS	expressed protein	CODON_DELETION	GCCGTCTTCACCTTCTCGAT	CCGACAGACCTCAACCTCTT	158	A	A	A	NA
PMS1910	Os_Chr03	25912420	25912443	(CT)12	(CT)12	(CT)12	(CT)12	(CT)8	(CT)12	(CT)12	(CT)12	(CT)12	(CT)12	(CT)12	-	Introns/Intergenic	-	-	TCCTCCTCTTCCTCTAACTTGTCTGG	CGCCACTAGGCACTCACATCG	286	P	A	A	RM15621

Polymorphic SSR (PMS) markers	Chromosomes	Start physical positions (bp)	End physical positions (bp)	Nipponbare	Kasalath	Nagina22	IR64	Pokkali	Bala	Tainung67	Azucena	Moroberekan	*Oryza rufipogon*	*Oryza nivara*	MSU gene locus IDs	Structural annotation	Functional annotation	SSR functional effects	Forward primers (5'-3')	Reverse primers (5'-3')	Amplified product size (bp)	Markers exhibiting polymorphism within *indica* rice	Transcrption factor genes-derived markers	Known cloned genes-derived markers	PMS markers corresponding with RM markers
PMS1911	Os_Chr03	25941276	25941307	(TA)16	(TA)16	(TA)16	(TA)7	(TA)7	(TA)16	(TA)16	(TA)10	(TA)16	(TA)16	(TA)16	-	Introns/Intergenic	-	-	TCTTAGGGTATTCTTCCGTTCC	CACTAGTTCTCTACTTGCACATAGCC	376	P	A	A	RM15622
PMS1912	Os_Chr03	25991024	25991037	(AG)7	(AG)7	(AG)7	(AG)7	(AG)6	(AG)6	(AG)7	(AG)7	(AG)7	(AG)7	(AG)7	-	Introns/Intergenic	-	-	GCTACCCTCCCCATTGCTTA	TGGTTTTGATCCTATGCGGT	166	P	A	A	NA
PMS1913	Os_Chr03	26041232	26041249	(AT)9	(AT)9	(AT)9	(AT)9	(AT)7	(AT)9	(AT)9	(AT)9	(AT)9	(AT)9	(AT)9	-	Introns/Intergenic	-	-	AAAGCGTACAAGAGTTTTACAGCA	CAAAGTTTGTATGTGCGTATTTTT	234	P	A	A	NA
PMS1914	Os_Chr03	26143054	26143079	(AT)13	(AT)13	(AT)6	(AT)13	(AT)13	(AT)13	(AT)13	(AT)13	(AT)13	(AT)13	(AT)13	-	Introns/Intergenic	-	-	TGTGTGTGCATGTGAGCTTG	ATTAGATACCATGGTGCCCG	107	P	A	A	NA
PMS1915	Os_Chr03	26232668	26232697	(TA)15	(TA)15	(TA)9	(TA)8	(TA)15	(TA)15	(TA)15	(TA)15	(TA)13	(TA)13	(TA)15	-	Introns/Intergenic	-	-	GTTAATGGTGGTGCATGCTATGG	TGTATTCGGGAGGATGGTTTGG	411	P	A	A	RM15640
PMS1916	Os_Chr03	26292134	26292148	(GCT)5	(GCT)5	(GCT)5	(GCT)6	(GCT)5	(GCT)5	(GCT)5	(GCT)5	(GCT)5	(GCT)6	(GCT)5	-	Introns/Intergenic	-	-	TGTTGGTGTGCTTCCCATTA	GCTCAGCTCAGCAACACAGT	277	P	A	A	NA
PMS1917	Os_Chr03	26300145	26300159	(CGC)5	(CGC)5	(CGC)5	(CGC)5	(CGC)5	(CGC)5	(CGC)5	(CGC)5	(CGC)5	(CGC)5	(CGC)6	-	Introns/Intergenic	-	-	GCTTGGGACACTTGTTGGTT	CAGATCGACGAGGGAAAGTC	213	A	A	A	NA
PMS1918	Os_Chr03	26357262	26357275	(AT)7	(AT)7	(AT)7	(AT)7	(AT)7	(AT)7	(AT)7	(AT)7	(AT)7	(AT)8	(AT)7	-	Introns/Intergenic	-	-	TTTGGGTTCCTAGCAGCAGT	TGTATACCCCAAGCAGTGGC	195	A	A	A	NA
PMS1919	Os_Chr03	26358126	26358141	(TG)8	(TG)8	(TG)6	(TG)8	(TG)8	(TG)8	(TG)8	(TG)8	(TG)8	(TG)8	(TG)8	-	Introns/Intergenic	-	-	CCTATCCACGTACTGTGCCC	TGTCGTGTGTCCACCAGAGT	276	P	A	A	NA
PMS1920	Os_Chr03	26359804	26359829	(AT)13	(AT)13	(AT)13	(AT)8	(AT)9	(AT)13	(AT)13	(AT)13	(AT)13	(AT)13	(AT)13	-	Introns/Intergenic	-	-	GAGTAAGAATCCTCGGCCATTATCC	AAAGAGCCGCAATAGCGTTCG	488	P	A	A	RM15643
PMS1921	Os_Chr03	26365204	26365219	(AT)8	(AT)8	(AT)8	(AT)8	(AT)8	(AT)8	(AT)8	(AT)7	(AT)8	(AT)8	(AT)8	-	Introns/Intergenic	-	-	TCATGCAAGAAGAAGCAATCA	TGTCGATCGGCGACTTAGAT	262	A	A	A	NA
PMS1922	Os_Chr03	26460813	26460830	(GGC)6	(GGC)6	(GGC)6	(GGC)6	(GGC)6	(GGC)6	(GGC)6	(GGC)6	(GGC)5	(GGC)6	(GGC)6	LOC_Os03g46770	CDS	RNA recognition motif containing protein, expressed	CODON_DELETION	GCAACATCACCGTCAATGAG	CCACCAATCAGTTCCTCCAG	267	A	A	A	NA
PMS1923	Os_Chr03	26525705	26525755	(GAT)17	(GAT)15	(GAT)17	(GAT)17	(GAT)17	(GAT)16	(GAT)17	(GAT)16	(GAT)16	(GAT)17	(GAT)17	-	Introns/Intergenic	-	-	GGGAGATGCATTCAAGCATGG	ATCTGCAGAAGCTTGCTACAACG	300	P	A	A	RM15652

**Polymorphic SSR (PMS) markers**	**Chromosomes**	**Start physical positions (bp)**	**End physical positions (bp)**	**Nipponbare**	**Kasalath**	**Nagina22**	**IR64**	**Pokkali**	**Bala**	**Tainung67**	**Azucena**	**Moroberekan**	***Oryza rufipogon***	***Oryza nivara***	**MSU gene locus IDs**	**Structural annotation**	**Functional annotation**	**SSR functional effects**	**Forward primers (5'-3')**	**Reverse primers (5'-3')**	**Amplified product size (bp)**	**Markers exhibiting polymorphism within *indica* rice**	**Transcrption factor genes-derived markers**	**Known cloned genes-derived markers**	**PMS markers corresponding with RM markers**
PMS1924	Os_Chr03	26586570	26586591	(GA)11	(GA)11	(GA)11	(GA)16	(GA)11	(GA)11	(GA)11	(GA)11	(GA)11	(GA)11	(GA)11	-	Introns/Intergenic	-	-	TGCTCCATGGGTGTACTTATCTCC	CCACAACAACTATTGTTCCCTTCC	301	P	A	A	RM15657
PMS1925	Os_Chr03	26646497	26646512	(CT)8	(CT)8	(CT)8	(CT)8	(CT)8	(CT)8	(CT)8	(CT)8	(CT)8	(CT)8	(CT)6	-	Introns/Intergenic	-	-	AGCTCCATGGGGAAATCTCT	TACTCGTCGTTCGCTGACTG	235	A	A	A	NA
PMS1926	Os_Chr03	26706431	26706448	(TC)9	(TC)9	(TC)9	(TC)9	(TC)9	(TC)9	(TC)9	(TC)9	(TC)9	(TC)9	(TC)10	LOC_Os03g47190	DRR	expressed protein	-	ATGAAAAACATGGAGCTCGG	CGTCTGTGACATGCTTGCTT	234	A	A	A	NA
PMS1927	Os_Chr03	26710512	26710529	(GT)9	(GT)9	(GT)9	(GT)9	(GT)11	(GT)9	(GT)9	(GT)9	(GT)9	(GT)9	(GT)9	-	Introns/Intergenic	-	-	ACTCAGAGGAGCGGTGTTGT	TCAAAGAAAGGCTTGCTGGT	173	P	A	A	NA
PMS1928	Os_Chr03	26740686	26740703	(CCG)6	(CCG)6	(CCG)6	(CCG)6	(CCG)6	(CCG)6	(CCG)6	(CCG)6	(CCG)6	(CCG)5	(CCG)6	LOC_Os03g47260	CDS	expressed protein	CODON_CHANGE_PLUS_CODON_DELETION	AAGACGATGGTGAGATTGGG	CCGTCTTGAGCAGCCAGTA	193	A	A	A	NA
PMS1929	Os_Chr03	26863102	26863119	(CGC)6	(CGC)6	(CGC)5	(CGC)6	(CGC)6	(CGC)6	(CGC)6	(CGC)6	(CGC)6	(CGC)6	(CGC)6	LOC_Os03g47500	URR	zinc finger, C3HC4 type domain containing protein, expressed	-	TATACTCCTCGTCCTCCCCC	ATACAAGCACCCAAGCAACC	220	P	A	A	NA
PMS1930	Os_Chr03	26881031	26881058	(TATC)7	(TATC)7	(TATC)7	(TATC)7	(TATC)7	(TATC)7	(TATC)7	(TATC)7	(TATC)7	(TATC)7	(TATC)6	-	Introns/Intergenic	-	-	GGCCATTATGTGCATCTCTCAGC	GGATCGATCGACATCAATCTTGG	185	A	A	A	RM7097
PMS1931	Os_Chr03	26962768	26962788	(GGT)7	(GGT)8	(GGT)7	(GGT)7	(GGT)7	(GGT)7	(GGT)7	(GGT)7	(GGT)5	(GGT)7	(GGT)7	LOC_Os03g47620	CDS	ankyrin, putative, expressed	CODON_DELETION	CATGGAGAAGAAGGATAAGGGAAGG	ACCCAACCCACCATTAGAGATCC	321	P	A	A	RM15668
PMS1932	Os_Chr03	26978259	26978272	(CT)7	(CT)6	(CT)7	(CT)6	(CT)7	(CT)6	(CT)7	(CT)7	(CT)7	(CT)7	(CT)7	-	Introns/Intergenic	-	-	ACCTACCATTTTACGCGCAG	CGAAGCACCTTTGCCTTTAG	271	P	A	A	NA
PMS1933	Os_Chr03	27140592	27140671	(TA)40	(TA)40	(TA)18	(TA)40	(TA)40	(TA)40	(TA)40	(TA)40	(TA)40	(TA)40	(TA)9	-	Introns/Intergenic	-	-	GTGGTTAATGGGCTGGTGAT	CCCAAAGTTTATATACCCGATTCA	258	P	A	A	NA
PMS1934	Os_Chr03	27238747	27238767	(TGG)7	(TGG)7	(TGG)6	(TGG)7	(TGG)7	(TGG)7	(TGG)5	(TGG)7	(TGG)7	(TGG)7	(TGG)7	LOC_Os03g47920	CDS	expressed protein	CODON_DELETION	TTACCACCACCACCAGGAAAGC	AAGAGGAAGCGCATATCCAAGC	299	P	A	A	RM15684
PMS1935	Os_Chr03	27281827	27281847	(GGC)7	(GGC)7	(GGC)6	(GGC)7	(GGC)7	(GGC)7	(GGC)7	(GGC)7	(GGC)7	(GGC)7	(GGC)7	LOC_Os03g47980	CDS	expressed protein	CODON_DELETION	CGTCGTCGTCGTCATCCTCTTCC	CCACCAAACCACCTCCGATGG	465	P	A	A	RM15685
PMS1936	Os_Chr03	27283172	27283192	(GCG)7	(GCG)7	(GCG)7	(GCG)6	(GCG)7	(GCG)7	(GCG)5	(GCG)7	(GCG)7	(GCG)7	(GCG)7	LOC_Os03g47990	CDS	OB-fold nucleic acid binding domain containing protein, putative, expressed	CODON_DELETION	GAGAGTTGAGACCCGAGAGAGC	GAGGGTGTAGTCCATGTTGTTCC	128	P	A	A	RM15686

Polymorphic SSR (PMS) markers	Chromosomes	Start physical positions (bp)	End physical positions (bp)	Nipponbare	Kasalath	Nagina22	IR64	Pokkali	Bala	Tainung67	Azucena	Moroberekan	*Oryza rufipogon*	*Oryza nivara*	MSU gene locus IDs	Structural annotation	Functional annotation	SSR functional effects	Forward primers (5'-3')	Reverse primers (5'-3')	Amplified product size (bp)	Markers exhibiting polymorphism within *indica* rice	Transcrption factor genes-derived markers	Known cloned genes-derived markers	PMS markers corresponding with RM markers
PMS1937	Os_Chr03	27345029	27345058	(TA)15	(TA)15	(TA)15	(TA)6	(TA)15	(TA)15	(TA)15	(TA)6	(TA)15	(TA)15	(TA)15	-	Introns/Intergenic	-	-	GCTGGTTATTCATG AGGCAGTGG	AAGTGCAAGTCCCTT CTTTCTGG	284	P	A	A	RM15693
PMS1938	Os_Chr03	27364325	27364346	(GT)11	(GT)11	(GT)11	(GT)11	(GT)11	(GT)11	(GT)11	(GT)7	(GT)11	(GT)11	(GT)11	LOC_Os03g48104	URR	expressed protein	-	GGCTTCTCAACTCA CCCGTAGC	GTCAGTCACACAGCC ACACAGG	187	A	A	A	RM15695
PMS1939	Os_Chr03	27387941	27387970	(CGG)10	(CGG)10	(CGG)10	(CGG)10	(CGG)10	(CGG)10	(CGG)10	(CGG)10	(CGG)10	(CGG)5	(CGG)7	-	Introns/Intergenic	-	-	CAACTTCTCTCTTCC TCCTATGC	AGTAACCTTCCGATT GACACC	379	A	A	A	RM6053
PMS1940	Os_Chr03	27486595	27486646	(TA)26	(TA)26	(TA)26	(TA)26	(TA)6	(TA)26	(TA)26	(TA)26	(TA)6	(TA)8	(TA)11	-	Introns/Intergenic	-	-	TGCAGATGACCGTG AGAGAG	TACCCCTCCTTACCC AATCC	204	P	A	A	NA
PMS1941	Os_Chr03	27607366	27607415	(TA)25	(TA)20	(TA)25	(TA)25	(TA)25	(TA)25	(TA)25	(TA)18	(TA)25	(TA)25	(TA)25	-	Introns/Intergenic	-	-	CAGCGAGTACTACT ACGTCGAAA	GATCGATCTTTACTC CCGGTT	277	P	A	A	NA
PMS1942	Os_Chr03	27669078	27669095	(CTC)6	(CTC)6	(CTC)6	(CTC)6	(CTC)6	(CTC)5	(CTC)6	(CTC)6	(CTC)6	(CTC)6	(CTC)6	LOC_Os03g48540	CDS	expressed protein	CODON_DELETION	AGATACGACTCTCC CCCTCC	ACGCCGGAATCACA CTACTC	246	P	A	A	NA
PMS1943	Os_Chr03	27694595	27694615	(GCA)7	(GCA)7	(GCA)7	(GCA)7	(GCA)7	(GCA)7	(GCA)7	(GCA)7	(GCA)7	(GCA)7	(GCA)6	-	Introns/Intergenic	-	-	GATTCTTGGGCACT GTCATTGG	CGAATCAGCATGATC AGCATACC	184	A	A	A	RM15719
PMS1944	Os_Chr03	27703804	27703818	(GCC)5	(GCC)6	(GCC)5	(GCC)5	(GCC)5	(GCC)5	(GCC)5	(GCC)5	(GCC)5	(GCC)5	(GCC)5	LOC_Os03g48590	CDS	protein transport protein Sec61 subunit alpha, putative, expressed	CODON_INSERTION	GAGGTGTAGAGGA GGCGGAC	ACCAGGGCCAGAAA GCTG	215	P	A	A	NA
PMS1945	Os_Chr03	27709739	27709800	(AG)31	(AG)31	(AG)31	(AG)31	(AG)31	(AG)28	(AG)31	(AG)31	(AG)31	(AG)31	(AG)31	-	Introns/Intergenic	-	-	GGGAGAGAGACAG ACAGAGAATCG	TTCATCTCACCTTGC ACTCTTGC	269	P	A	A	RM15722
PMS1946	Os_Chr03	27823097	27823120	(CGC)8	(CGC)8	(CGC)8	(CGC)8	(CGC)8	(CGC)6	(CGC)8	(CGC)8	(CGC)8	(CGC)8	(CGC)8	LOC_Os03g48840	URR	ribosomal L18p/L5e family protein, putative, expressed	-	CCTCTCTCCCAAAT CCCAATCC	ATACATACCGTCGAT GGGTAGTCG	94	P	A	A	RM15729
PMS1947	Os_Chr03	27860910	27860924	(CCG)5	(CCG)8	(CCG)8	(CCG)8	(CCG)5	(CCG)8	(CCG)5	(CCG)5	(CCG)5	(CCG)5	(CCG)5	LOC_Os03g48930	URR	peptidase, T1 family, putative, expressed	-	ACCATCACCTTGTC CTCGTC	GACGAAGCGAACTC AACTCC	255	P	A	A	NA
PMS1948	Os_Chr03	27905003	27905017	(TTG)5	(TTG)5	(TTG)5	(TTG)5	(TTG)5	(TTG)5	(TTG)5	(TTG)5	(TTG)5	(TTG)7	(TTG)5	LOC_Os03g48990	CDS	retrotransposon protein, putative, Ty3-gypsy subclass, expressed	CODON_INSERTION	TATTTCCCTGGTTCT GCTGG	TAGTTTCCCCCGGTT TCTCT	206	A	A	A	NA
PMS1949	Os_Chr03	27943367	27943384	(CT)9	(CT)9	(CT)9	(CT)9	(CT)9	(CT)9	(CT)9	(CT)9	(CT)9	(CT)11	(CT)9	-	Introns/Intergenic	-	-	GCCGGGAAAAGTCT TACACA	CAACCTTGTCGTGGT TAGCA	101	A	A	A	NA

Polymorphic SSR (PMS) markers	Chromosomes	Start physical positions (bp)	End physical positions (bp)	Nipponbare	Kasalath	Nagina22	IR64	Pokkali	Bala	Tainung67	Azucena	Moroberekan	*Oryza rufipogon*	*Oryza nivara*	MSU gene locus IDs	Structural annotation	Functional annotation	SSR functional effects	Forward primers (5'-3')	Reverse primers (5'-3')	Amplified product size (bp)	Markers exhibiting polymorphism within *indica* rice	Transcrption factor genes-derived markers	Known cloned genes-derived markers	PMS markers corresponding with RM markers
PMS1950	Os_Chr03	27988122	27988145	(CGG)8	(CGG)6	(CGG)8	(CGG)8	(CGG)8	(CGG)8	(CGG)8	(CGG)8	(CGG)8	(CGG)8	(CGG)8	LOC_Os03g49132	CDS	ZOS3-16 - C2H2 zinc finger protein, expressed	CODON_CHANGE_PLUS_CODON_DELETION	GTGTGTCCCAAGAAGATCGC	AGCGCTTGGAAGCTCTCTC	217	P	P	A	NA
PMS1951	Os_Chr03	28225264	28225284	(GTG)7	(GTG)7	(GTG)7	(GTG)7	(GTG)5	(GTG)7	(GTG)7	(GTG)7	(GTG)7	(GTG)7	(GTG)7	LOC_Os03g49560	CDS	expressed protein	CODON_DELETION	CCTTCACGATGAGCCAAGTC	CCCTAGCGCTGTTGCATATT	189	P	A	A	NA
PMS1952	Os_Chr03	28259645	28259665	(CCG)7	(CCG)7	(CCG)7	(CCG)5	(CCG)7	(CCG)7	(CCG)7	(CCG)7	(CCG)7	(CCG)7	(CCG)7	-	Introns/Intergenic	-	-	TCTCCCACTTCACCTCCTCACC	ATCCACCTAACCAACCAAGTTGC	188	P	A	A	RM15756
PMS1953	Os_Chr03	28297239	28297253	(CCT)5	(CCT)5	(CCT)5	(CCT)6	(CCT)6	(CCT)6	(CCT)5	(CCT)5	(CCT)5	(CCT)5	(CCT)5	LOC_Os03g49700	URR	SNARE associated Golgi protein, putative, expressed	-	CTCGTTCAGCAGGTCAAACA	GCAGCTTCATGCCTTCATC	188	P	A	A	NA
PMS1954	Os_Chr03	28321591	28321617	(AGT)9	(AGT)9	(AGT)9	(AGT)9	(AGT)9	(AGT)5	(AGT)9	(AGT)9	(AGT)9	(AGT)9	(AGT)9	-	Introns/Intergenic	-	-	CAGGATCGGACAGGATCACAGG	GCTCCTGGCGCAGCTATAGACC	90	P	A	A	RM15759
PMS1955	Os_Chr03	28327121	28327134	(CG)7	(CG)7	(CG)7	(CG)7	(CG)7	(CG)7	(CG)7	(CG)7	(CG)7	(CG)7	(CG)6	-	Introns/Intergenic	-	-	GAAAGTGGGGTTGGCACTT	AAAGCTAAGCCAGCAGCAAC	270	A	A	A	NA
PMS1956	Os_Chr03	28378244	28378257	(CT)7	(CT)7	(CT)7	(CT)7	(CT)7	(CT)7	(CT)7	(CT)7	(CT)7	(CT)6	(CT)7	-	Introns/Intergenic	-	-	TGTCAAAACGAACCAACCAA	GGTCTTTTCCACCCCACTTT	139	A	A	A	NA
PMS1957	Os_Chr03	28391563	28391580	(CGA)6	(CGA)5	(CGA)6	(CGA)6	(CGA)6	(CGA)6	(CGA)6	(CGA)6	(CGA)6	(CGA)6	(CGA)6	LOC_Os03g49830	CDS	expressed protein	CODON_CHANGE_PLUS_CODON_DELETION	CTCCACCTCCACCACGAC	GAGCTTAGGAGGTGGCAATG	254	P	A	A	NA
PMS1958	Os_Chr03	28397920	28397955	(TCTT)9	(TCTT)9	(TCTT)9	(TCTT)9	(TCTT)9	(TCTT)7	(TCTT)9	(TCTT)9	(TCTT)9	(TCTT)9	(TCTT)9	-	Introns/Intergenic	-	-	CACACTCGTGGGCTTCTAATTCC	TTTGTCGCTGAAAGGATGAAGG	179	P	A	A	RM15766
PMS1959	Os_Chr03	28446689	28446702	(CT)7	(CT)7	(CT)7	(CT)7	(CT)7	(CT)7	(CT)7	(CT)7	(CT)7	(CT)6	(CT)7	-	Introns/Intergenic	-	-	TTCAAGCCGGCTGTAAGTTT	CGGATGGAGGAAATAGATCG	130	A	A	A	NA
PMS1960	Os_Chr03	28466295	28466316	(TA)11	(TA)11	(TA)11	(TA)6	(TA)11	(TA)11	(TA)11	(TA)11	(TA)11	(TA)11	(TA)11	-	Introns/Intergenic	-	-	CTTTGGCTCTCTCGTTCCTC	CTCGTGCACATAGATGGAGC	239	P	A	A	NA
PMS1961	Os_Chr03	28516340	28516353	(CT)7	(CT)7	(CT)7	(CT)7	(CT)7	(CT)7	(CT)6	(CT)7	(CT)7	(CT)7	(CT)7	-	Introns/Intergenic	-	-	GAAGGCAGCGGTAGAAGTTG	GCTAGAGAAGGGGGTGTCCT	242	A	A	A	NA
PMS1962	Os_Chr03	28578944	28578957	(TA)7	(TA)7	(TA)7	(TA)7	(TA)6	(TA)6	(TA)7	(TA)7	(TA)6	(TA)6	(TA)7	-	Introns/Intergenic	-	-	TGGCGCCATAAATATAGTCAC	GCTGATTTGAGAAAAAGGCG	225	P	A	A	NA

Polymorphic SSR (PMS) markers	Chromosomes	Start physical positions (bp)	End physical positions (bp)	Nipponbare	Kasalath	Nagina22	IR64	Pokkali	Bala	Tainung67	Azucena	Moroberekan	*Oryza rufipogon*	*Oryza nivara*	MSU gene locus IDs	Structural annotation	Functional annotation	SSR functional effects	Forward primers (5'-3')	Reverse primers (5'-3')	Amplified product size (bp)	Markers exhibiting polymorphism within *indica* rice	Transcrption factor genes-derived markers	Known cloned genes-derived markers	PMS markers corresponding with RM markers
PMS1963	Os_Chr03	28617377	28617428	(TA)26	(TA)26	(TA)6	(TA)26	(TA)26	(TA)26	(TA)26	(TA)9	(TA)26	(TA)26	(TA)26	-	Introns/Intergenic	-	-	GAATGGAACATATCCTGTAGTACGAA	ATGATGCGTAAGATGGGCTC	243	P	A	A	NA
PMS1964	Os_Chr03	28683436	28683481	(AG)23	(AG)20	(AG)23	(AG)23	(AG)23	(AG)23	(AG)23	(AG)23	(AG)23	(AG)20	(AG)20	-	Introns/Intergenic	-	-	AGGAACACCCAAGAGAGTCATGC	GCAAGAAAGCTCTGCTCCATGC	234	P	A	A	RM1350
PMS1965	Os_Chr03	28688919	28688936	(CCG)6	(CCG)6	(CCG)6	(CCG)6	(CCG)6	(CCG)6	(CCG)6	(CCG)6	(CCG)6	(CCG)6	(CCG)5	LOC_Os03g50310	CDS	CCT/B-box zinc finger protein, putative, expressed	CODON_DELETION	CAAGAAGATCCGGTACGAGG	TGCATGTAGCTAGACGACGC	260	A	P	A	NA
PMS1966	Os_Chr03	28872686	28872703	(CCT)6	(CCT)5	(CCT)5	(CCT)5	(CCT)6	(CCT)6	(CCT)6	(CCT)6	(CCT)6	(CCT)6	(CCT)5	-	Introns/Intergenic	-	-	TAGCTTGCATTGTAGCGTGC	GGATTTGATGCGCTATTGGT	242	P	A	A	NA
PMS1967	Os_Chr03	28918140	28918181	(AT)21	(AT)21	(AT)7	(AT)21	(AT)21	(AT)21	(AT)21	(AT)12	(AT)21	(AT)7	(AT)21	-	Introns/Intergenic	-	-	GTTGTGTCGTCCCAAACTCAGC	GTTCTTCGATCCGTTCTTTCTGC	142	P	A	A	RM15801
PMS1968	Os_Chr03	28955934	28955953	(AG)10	(AG)9	(AG)10	(AG)10	(AG)10	(AG)7	(AG)10	(AG)10	(AG)9	(AG)10	(AG)10	-	Introns/Intergenic	-	-	CACTAACGGTGTCACTGCTTGC	ACCTCTTCCATCCCTCCTTTCG	96	P	A	A	RM15805
PMS1969	Os_Chr03	28977162	28977179	(CT)9	(CT)9	(CT)9	(CT)9	(CT)9	(CT)9	(CT)9	(CT)9	(CT)9	(CT)14	(CT)9	-	Introns/Intergenic	-	-	TCTCATTTCTTCCTCGCTCG	CATCAGGTGGTTTCGTTGTG	207	A	A	A	NA
PMS1970	Os_Chr03	29060035	29060056	(AT)11	(AT)11	(AT)11	(AT)6	(AT)11	(AT)11	(AT)11	(AT)11	(AT)6	(AT)11	(AT)11	-	Introns/Intergenic	-	-	CACACGACAAATGGCTCATCC	GGAGTTAGTAGGCGGGTTTGACC	198	P	A	A	RM15810
PMS1971	Os_Chr03	29061506	29061525	(AT)10	(AT)10	(AT)10	(AT)10	(AT)10	(AT)10	(AT)9	(AT)10	(AT)10	(AT)10	(AT)10	-	Introns/Intergenic	-	-	AGCGAGCGCTCCAATTCTATACC	GAGGCTAGCTTGGTTGGGTAAGC	195	A	A	A	RM15811
PMS1972	Os_Chr03	29103494	29103511	(GGC)6	(GGC)6	(GGC)6	(GGC)6	(GGC)6	(GGC)6	(GGC)5	(GGC)6	(GGC)6	(GGC)6	(GGC)6	LOC_Os03g50940	CDS	COX11	CODON_DELETION	TCCTCCTCGAGCTCTTCTCC	GTAGCGTCGAACACCGTACA	275	A	A	P	NA
PMS1973	Os_Chr03	29114930	29114950	(GCC)7	(GCC)7	(GCC)6	(GCC)7	(GCC)7	(GCC)7	(GCC)7	(GCC)7	(GCC)7	(GCC)7	(GCC)5	LOC_Os03g50970	CDS	retrotransposon protein, putative, Ty1-copia subclass, expressed	CODON_DELETION	ATCAAATCCGCCGTCACAAACG	CGTTCAACTACGTCGCCGTGTCC	200	P	A	A	RM15814
PMS1974	Os_Chr03	29153279	29153299	(CCG)7	(CCG)7	(CCG)7	(CCG)7	(CCG)7	(CCG)6	(CCG)7	(CCG)7	(CCG)7	(CCG)7	(CCG)7	LOC_Os03g51010	CDS	hydrolase, alpha/beta fold family domain containing protein, expressed	CODON_DELETION	CTTTCACGGAAGGTCGTCATCG	GGATTGGGAGAGGCATTTCTAGG	576	P	A	A	RM15819
PMS1975	Os_Chr03	29176220	29176293	(AT)37	(AT)37	(AT)37	(AT)9	(AT)37	(AT)21	(AT)37	(AT)37	(AT)37	(AT)37	(AT)37	-	Introns/Intergenic	-	-	GTGAGGTGTCGCGGATTTAC	TCAATTTCAACAATGGTGCC	191	P	A	A	NA

Polymorphic SSR (PMS) markers	Chromosomes	Start physical positions (bp)	End physical positions (bp)	Nipponbare	Kasalath	Nagina22	IR64	Pokkali	Bala	Tainung67	Azucena	Moroberekan	*Oryza rufipogon*	*Oryza nivara*	MSU gene locus IDs	Structural annotation	Functional annotation	SSR functional effects	Forward primers (5'-3')	Reverse primers (5'-3')	Amplified product size (bp)	Markers exhibiting polymorphism within *indica* rice	Transcrption factor genes-derived markers	Known cloned genes-derived markers	PMS markers corresponding with RM markers
PMS1976	Os_Chr03	29240432	29240449	(GA)9	(GA)10	(GA)9	(GA)10	(GA)9	(GA)10	(GA)9	(GA)9	(GA)9	(GA)9	(GA)9	-	Introns/Intergenic	-	-	AATCAAAACCAAATCGCCC	ATATCGATCGTACAGCGCCT	279	P	A	A	NA
PMS1977	Os_Chr03	29291435	29291466	(CT)16	(CT)16	(CT)16	(CT)16	(CT)16	(CT)16	(CT)16	(CT)14	(CT)16	(CT)16	(CT)16	-	Introns/Intergenic	-	-	AATTTAACCTTGCCACGTCACC	CGAAGGTTGGAAGCTTCTGG	197	A	A	A	RM15828
PMS1978	Os_Chr03	29384280	29384293	(AT)7	(AT)7	(AT)7	(AT)7	(AT)7	(AT)7	(AT)7	(AT)6	(AT)6	(AT)7	(AT)7	-	Introns/Intergenic	-	-	ATATCGTGTGTGTGGCTGGA	ACTCCCTCCGTTTCACAATG	188	A	A	A	NA
PMS1979	Os_Chr03	29402892	29402912	(GCT)7	(GCT)7	(GCT)7	(GCT)7	(GCT)7	(GCT)7	(GCT)6	(GCT)7	(GCT)7	(GCT)6	(GCT)7	LOC_Os03g51390	CDS	expressed protein	CODON_DELETION	CGTAGCGCTTCTCCTCCTC	GAGAGAGACGGTGCATGGTT	124	A	A	A	NA
PMS1980	Os_Chr03	29533097	29533168	(AT)36	(AT)36	(AT)36	(AT)36	(AT)6	(AT)36	(AT)36	(AT)36	(AT)36	(AT)32	(AT)36	-	Introns/Intergenic	-	-	TGTGGAGTCAACTTATGCTCATGC	CTAGGACACTGAGGCCCAACC	350	P	A	A	RM15843
PMS1981	Os_Chr03	29534296	29534330	(AAAAG)7	(AAAAG)7	(AAAAG)7	(AAAAG)7	(AAAAG)5	(AAAAG)5	(AAAAG)7	(AAAAG)7	(AAAAG)7	(AAAAG)7	(AAAAG)7	-	Introns/Intergenic	-	-	AGTTTGGACGCTCTCGAAAA	CAGACGGCAACACAAACACT	267	P	A	A	NA
PMS1982	Os_Chr03	29703834	29703881	(TA)24	(TA)24	(TA)14	(TA)7	(TA)15	(TA)24	(TA)24	(TA)11	(TA)24	(TA)10	(TA)24	-	Introns/Intergenic	-	-	GTCATTCTGGCATTTCCCAT	ATTTGATATCATGGTGCCCG	139	P	A	A	NA
PMS1983	Os_Chr03	29755026	29755040	(CAA)5	(CAA)6	(CAA)6	(CAA)5	(CAA)5	(CAA)5	(CAA)5	(CAA)5	(CAA)5	(CAA)5	(CAA)5	-	Introns/Intergenic	-	-	CTAGCCATGGCAGCAGTACA	ATGCTCTTGCTTTGCTTGGT	226	P	A	A	NA
PMS1984	Os_Chr03	29766174	29766191	(GCC)6	(GCC)5	(GCC)5	(GCC)6	(GCC)5	(GCC)5	(GCC)6	(GCC)6	(GCC)6	(GCC)6	(GCC)6	LOC_Os03g51900	CDS	DEAD-box ATP-dependent RNA helicase, putative, expressed	CODON_DELETION	CTATTAACCGAGCCGAGCTG	GCCTCGTCGTGGTTCTCTAC	152	P	A	A	NA
PMS1985	Os_Chr03	29820294	29820385	(TA)46	(TA)25	(TA)46	(TA)46	(TA)26	(TA)46	(TA)7	(TA)46	(TA)6	(TA)10	(TA)46	-	Introns/Intergenic	-	-	GGAGTTTAGAAATATGGGCTCTGG	TGGTTGATGTCTGAACCGTATAGC	230	P	A	A	RM15855
PMS1986	Os_Chr03	29988996	29989011	(AT)8	(AT)8	(AT)6	(AT)8	(AT)8	(AT)8	(AT)6	(AT)8	(AT)8	(AT)8	(AT)8	-	Introns/Intergenic	-	-	ACCGTTTGTTTTGGATGCTC	CAAGTCCGTGGGTTTTAGGA	269	P	A	A	NA
PMS1987	Os_Chr03	29995856	29995893	(TA)19	(TA)19	(TA)8	(TA)11	(TA)19	(TA)19	(TA)19	(TA)19	(TA)19	(TA)19	(TA)19	-	Introns/Intergenic	-	-	GCACCACACCAGTGTATACTCTTTCC	GCACCAGATAAGATGATGTCTACGG	308	P	A	A	RM15862
PMS1988	Os_Chr03	30007903	30007917	(AGC)5	(AGC)5	(AGC)5	(AGC)5	(AGC)5	(AGC)5	(AGC)5	(AGC)5	(AGC)5	(AGC)6	(AGC)6	-	Introns/Intergenic	-	-	ACATGTGCGCCTGTGTGTAT	AAAGCTTGGCACAAAGGAGA	205	A	A	A	NA

Polymorphic SSR (PMS) markers	Chromosomes	Start physical positions (bp)	End physical positions (bp)	Nipponbare	Kasalath	Nagina22	IR64	Pokkali	Bala	Tainung67	Azucena	Moroberekan	*Oryza rufipogon*	*Oryza nivara*	MSU gene locus IDs	Structural annotation	Functional annotation	SSR functional effects	Forward primers (5'-3')	Reverse primers (5'-3')	Amplified product size (bp)	Markers exhibiting polymorphism within *indica* rice	Transcrption factor genes-derived markers	Known cloned genes-derived markers	PMS markers corresponding with RM markers
PMS1989	Os_Chr03	30076891	30076908	(CGC)6	(CGC)6	(CGC)6	(CGC)6	(CGC)6	(CGC)6	(CGC)6	(CGC)5	(CGC)6	(CGC)6	(CGC)6	LOC_Os03g52380	DRR	PIII5 - Proteinase inhibitor II family protein precursor, expressed	-	GCTCCTCCTACAAGTGTCGC	GTGAGGTAAGCTGGCGCTAC	143	A	A	A	NA
PMS1990	Os_Chr03	30125163	30125204	(TA)21	(TA)21	(TA)21	(TA)8	(TA)8	(TA)20	(TA)21	(TA)10	(TA)21	(TA)21	(TA)21	-	Introns/Intergenic	-	-	CTAGAGGTGTTTGCCTGTGTGG	ACCGAAGTCCTTGTTGATTTGC	299	P	A	A	RM15879
PMS1991	Os_Chr03	30194635	30194648	(CT)7	(CT)6	(CT)6	(CT)7	(CT)7	(CT)7	(CT)7	(CT)7	(CT)7	(CT)7	(CT)7	-	Introns/Intergenic	-	-	GTCATCCTCCCTCTCCTTCC	TCGAAGTCCTCCTTGCAGAC	255	P	A	A	NA
PMS1992	Os_Chr03	30312910	30312925	(CA)8	(CA)8	(CA)8	(CA)8	(CA)8	(CA)8	(CA)8	(CA)8	(CA)8	(CA)7	(CA)7	-	Introns/Intergenic	-	-	GAGTAGTACGCCGCATGTGA	AGGAGAGAGCAAGCGTGATT	265	A	A	A	NA
PMS1993	Os_Chr03	30333341	30333356	(CT)8	(CT)8	(CT)8	(CT)6	(CT)8	(CT)8	(CT)8	(CT)8	(CT)8	(CT)6	(CT)8	-	Introns/Intergenic	-	-	AGGCCGAGAAGGTACTGGAT	ACCGGATCTAGCAGAGGGAG	216	P	A	A	NA
PMS1994	Os_Chr03	30395692	30395712	(CCG)7	(CCG)7	(CCG)7	(CCG)7	(CCG)7	(CCG)7	(CCG)7	(CCG)7	(CCG)7	(CCG)7	(CCG)6	LOC_Os03g53000	URR	XRCC4, putative, expressed	-	ACGGCCCATACGAATTCAAAGC	GAAAGAACGAACCCTTCGATTGC	388	A	A	A	RM15891
PMS1995	Os_Chr03	30434910	30434931	(TA)11	(TA)11	(TA)11	(TA)11	(TA)11	(TA)11	(TA)7	(TA)11	(TA)11	(TA)9	(TA)11	-	Introns/Intergenic	-	-	AGCCTCACCAGAAGAGACGA	TGATACCATGGTGCCCCC	208	A	A	A	NA
PMS1996	Os_Chr03	30445064	30445078	(CCA)5	(CCA)6	(CCA)5	(CCA)6	(CCA)5	(CCA)6	(CCA)5	(CCA)5	(CCA)5	(CCA)5	(CCA)5	LOC_Os03g53080	URR	zinc finger, C3HC4 type domain containing protein, expressed	-	CGGGATAGATGGGAGACAGA	CTGAGTTTGGACGAGGTTGG	256	P	A	A	NA
PMS1997	Os_Chr03	30522162	30522173	(TA)6	(TA)7	(TA)7	(TA)6	(TA)6	(TA)6	(TA)6	(TA)6	(TA)6	(TA)6	(TA)6	-	Introns/Intergenic	-	-	CCCCCTTGCACGTAGTTAGT	GGTTGGCTTTTAGTCCGTGT	278	P	A	A	NA
PMS1998	Os_Chr03	30522883	30522906	(GCG)8	(GCG)7	(GCG)8	(GCG)8	(GCG)8	(GCG)7	(GCG)8	(GCG)5	(GCG)8	(GCG)8	(GCG)8	-	Introns/Intergenic	-	-	CGTCTTCCCTCCAAACCAAACC	ATGGATCGGCTGGTGGATTACC	172	P	A	A	RM5992
PMS1999	Os_Chr03	30618815	30618862	(TA)24	(TA)24	(TA)24	(TA)7	(TA)24	(TA)24	(TA)24	(TA)7	(TA)11	(TA)24	(TA)24	-	Introns/Intergenic	-	-	GCCAGCGCTGAAAACTGT	ATGGTGCCCCATATGAGTGT	172	P	A	A	NA
PMS2000	Os_Chr03	30694855	30694872	(CCG)6	(CCG)6	(CCG)6	(CCG)6	(CCG)6	(CCG)6	(CCG)5	(CCG)6	(CCG)6	(CCG)6	(CCG)6	LOC_Os03g53520	CDS	expressed protein	CODON_DELETION	GTGTAGTCCTCCGATGACGG	AGTTCGGCGTCAAGAACAAG	121	A	A	A	NA
PMS2001	Os_Chr03	30708347	30708361	(TCG)5	(TCG)6	(TCG)5	(TCG)5	(TCG)5	(TCG)5	(TCG)5	(TCG)5	(TCG)5	(TCG)5	(TCG)5	LOC_Os03g53540	CDS	expressed protein	CODON_INSERTION	AAGTACGAGGCCGGGAAG	ATCAGATGAGCTGGACCGAG	274	P	A	A	NA

Polymorphic SSR (PMS) markers	Chromosomes	Start physical positions (bp)	End physical positions (bp)	Nipponbare	Kasalath	Nagina22	IR64	Pokkali	Bala	Tainung67	Azucena	Moroberekan	*Oryza rufipogon*	*Oryza nivara*	MSU gene locus IDs	Structural annotation	Functional annotation	SSR functional effects	Forward primers (5'-3')	Reverse primers (5'-3')	Amplified product size (bp)	Markers exhibiting polymorphism within *indica* rice	Transcrption factor genes-derived markers	Known cloned genes-derived markers	PMS markers corresponding with RM markers
PMS2002	Os_Chr03	30741038	30741053	(CA)8	(CA)8	(CA)8	(CA)8	(CA)8	(CA)8	(CA)7	(CA)8	(CA)8	(CA)8	(CA)8	-	Introns/Intergenic	-	-	CTCGCTAAAAGCTACGGGTC	CCAAGTCCGCTTTCTTTTGA	252	A	A	A	NA
PMS2003	Os_Chr03	30777970	30777987	(GGA)6	(GGA)6	(GGA)6	(GGA)6	(GGA)6	(GGA)6	(GGA)6	(GGA)5	(GGA)6	(GGA)6	(GGA)6	LOC_Os03g53670	URR	YT521-B-like family domain containing protein, expressed	-	AATTTTCGCCTTCTTGGGAT	TAAAAAGGATACGGTCGCCA	135	A	A	A	NA
PMS2004	Os_Chr03	30797269	30797286	(CGC)6	(CGC)6	(CGC)5	(CGC)5	(CGC)6	(CGC)6	(CGC)6	(CGC)6	(CGC)6	(CGC)6	(CGC)6	LOC_Os03g53710	CDS	aldose 1-epimerase, putative, expressed	CODON_DELETION	TACCCTGTCGATGATGGTGA	ACTACCGCGCCATAGATCC	260	P	A	A	NA
PMS2005	Os_Chr03	30808398	30808412	(CGC)5	(CGC)6	(CGC)5	(CGC)5	(CGC)5	(CGC)5	(CGC)5	(CGC)5	(CGC)5	(CGC)5	(CGC)5	LOC_Os03g53730	CDS	flavoprotein wrbA, putative, expressed	CODON_INSERTION	AGGGAAGCCGAAGAGGAAC	TTCTACTCCATGTACGGGCA	234	P	A	A	NA
PMS2006	Os_Chr03	30819948	30819977	(TA)15	(TA)15	(TA)15	(TA)15	(TA)15	(TA)15	(TA)7	(TA)15	(TA)8	(TA)15	(TA)15	-	Introns/Intergenic	-	-	CAGAAGAATGTTGGGAAAGTGC	CAAATCACAACGCTTGTTGAGG	89	A	A	A	RM15921
PMS2007	Os_Chr03	30851581	30851600	(AG)10	(AG)11	(AG)10	(AG)10	(AG)10	(AG)10	(AG)10	(AG)10	(AG)10	(AG)10	(AG)10	-	Introns/Intergenic	-	-	CCCACTGGACAGTGAGAGTTGG	AACGCGAGGCTTTATTGACAACC	200	P	A	A	RM15925
PMS2008	Os_Chr03	30885065	30885082	(CGG)6	(CGG)6	(CGG)6	(CGG)6	(CGG)5	(CGG)6	(CGG)6	(CGG)6	(CGG)6	(CGG)6	(CGG)6	-	Introns/Intergenic	-	-	TCCTCTCTGCATTCTTGGCT	CAGGAGCACGATGAGAAACC	146	P	A	A	NA
PMS2009	Os_Chr03	31119537	31119560	(GA)12	(GA)12	(GA)8	(GA)7	(GA)8	(GA)7	(GA)12	(GA)12	(GA)12	(GA)8	(GA)8	-	Introns/Intergenic	-	-	GGCGAGAGGGAGAGAGAGAT	AGCTAGCTTCGCTATTCCCC	208	P	A	A	NA
PMS2010	Os_Chr03	31133822	31133835	(AT)7	(AT)7	(AT)7	(AT)7	(AT)7	(AT)8	(AT)7	(AT)7	(AT)7	(AT)7	(AT)7	-	Introns/Intergenic	-	-	CATGCAGTGTTAGGGGTGTG	GTTCCTTTTTGGCTCTTCCC	229	P	A	A	NA
PMS2011	Os_Chr03	31143858	31143877	(GCAT)5	(GCAT)5	(GCAT)6	(GCAT)5	(GCAT)6	(GCAT)5	(GCAT)5	(GCAT)5	(GCAT)5	(GCAT)5	(GCAT)6	-	Introns/Intergenic	-	-	TTGCTAGCTACTCCCTCCGTTCC	CAACCACCCTTAACCCAAAGACC	255	P	A	A	RM15936
PMS2012	Os_Chr03	31202963	31202976	(CT)7	(CT)6	(CT)7	(CT)7	(CT)7	(CT)7	(CT)7	(CT)7	(CT)7	(CT)7	(CT)7	-	Introns/Intergenic	-	-	TGTCATTCTCACCCCTCCTC	CCCGAACTGTCAAAACTGGT	197	P	A	A	NA
PMS2013	Os_Chr03	31304772	31304792	(CCT)7	(CCT)7	(CCT)7	(CCT)7	(CCT)7	(CCT)7	(CCT)7	(CCT)7	(CCT)7	(CCT)6	(CCT)7	LOC_Os03g55050	DRR	UDP-glucoronosyl and UDP-glucosyl transferase domain containing protein, expressed	-	CAAGATATCCTTGCCGTTCTCG	GTTCAGTTCCAGATTTGCCTTGC	265	A	A	A	RM15943
PMS2014	Os_Chr03	31338838	31338853	(CG)8	(CG)8	(CG)8	(CG)8	(CG)6	(CG)8	(CG)8	(CG)8	(CG)8	(CG)8	(CG)8	-	Introns/Intergenic	-	-	CCCTAACGACCGAAAACAAA	CGTAATGTGGTTCGAATTGC	145	P	A	A	NA

Polymorphic SSR (PMS) markers	Chromosomes	Start physical positions (bp)	End physical positions (bp)	Nipponbare	Kasalath	Nagina22	IR64	Pokkali	Bala	Tainung67	Azucena	Moroberekan	*Oryza rufipogon*	*Oryza nivara*	MSU gene locus IDs	Structural annotation	Functional annotation	SSR functional effects	Forward primers (5'-3')	Reverse primers (5'-3')	Amplified product size (bp)	Markers exhibiting polymorphism within *indica* rice	Transcrption factor genes-derived markers	Known cloned genes-derived markers	PMS markers corresponding with RM markers
PMS2015	Os_Chr03	31345181	31345198	(CGG)6	(CGG)6	(CGG)6	(CGG)6	(CGG)6	(CGG)6	(CGG)6	(CGG)6	(CGG)6	(CGG)5	(CGG)6	LOC_Os03g55100	CDS	cyclic nucleotide-gated ion channel 2, putative, expressed	CODON_CHANGE_PLUS_CODON_DELETION	GATGTGTGTGATGGGGTGAA	ACGTCGTGGAGTGGAACG	178	A	A	A	NA
PMS2016	Os_Chr03	31399396	31399443	(TC)24	(TC)23	(TC)24	(TC)24	(TC)24	(TC)23	(TC)24	(TC)24	(TC)24	(TC)24	(TC)24	LOC_Os03g55180	DRR	DUF1336 domain containing protein, expressed	-	TGCTCCTGTTGTCATTCTTTGG	ATAAGATCGACTTTGCCGACAGC	250	P	A	A	RM8267,RM8203
PMS2017	Os_Chr03	31458770	31458790	(CGG)7	(CGG)7	(CGG)7	(CGG)7	(CGG)7	(CGG)7	(CGG)5	(CGG)7	(CGG)7	(CGG)7	(CGG)7	LOC_Os03g55270	URR	TIP41, putative, expressed	-	CTCTTCAGGACTGCGCCTCAGC	ACGCGAGCCCATAATCAAGTCG	175	A	A	A	RM15953
PMS2018	Os_Chr03	31503880	31503894	(CGG)5	(CGG)7	(CGG)5	(CGG)5	(CGG)5	(CGG)5	(CGG)5	(CGG)5	(CGG)5	(CGG)5	(CGG)5	LOC_Os03g55380	CDS	oxidoreductase, putative, expressed	CODON_CHANGE_PLUS_CODON_INSERTION	CACAAGTCACAACTCCGACG	TACCTGGGTGAAGAGGATGG	228	P	A	A	NA
PMS2019	Os_Chr03	31566006	31566029	(CT)12	(CT)12	(CT)12	(CT)12	(CT)12	(CT)12	(CT)7	(CT)12	(CT)12	(CT)12	(CT)12	-	Introns/Intergenic	-	-	GAACAGCCCACTTCTCCATCG	ACCAGGCCATGTACCTCTCTTCC	178	A	A	A	RM15957
PMS2020	Os_Chr03	31598366	31598389	(TACA)6	(TACA)6	(TACA)6	(TACA)6	(TACA)6	(TACA)6	(TACA)6	(TACA)6	(TACA)6	(TACA)6	(TACA)5	-	Introns/Intergenic	-	-	CTACTCCAAGGTCGCCTACATCC	ACAGTTGGACGCTTCTTGATTGG	134	A	A	A	RM15961
PMS2021	Os_Chr03	31623386	31623409	(GAG)8	(GAG)10	(GAG)10	(GAG)8	(GAG)8	(GAG)8	(GAG)8	(GAG)8	(GAG)8	(GAG)8	(GAG)8	LOC_Os03g55570	CDS	upstream activation factor subunit spp27, putative, expressed	CODON_INSERTION	GGGAAGAAGAGGAGGAGGAA	CCCTACAGAACGCATCGAAT	140	P	A	A	NA
PMS2022	Os_Chr03	31660252	31660271	(CT)10	(CT)9	(CT)10	(CT)10	(CT)10	(CT)10	(CT)10	(CT)10	(CT)10	(CT)10	(CT)10	-	Introns/Intergenic	-	-	ATGCTTGCAGCATTGTGCTTCG	CCTTATGGGTTGGTGGAGTGAAGG	99	P	A	A	RM15968
PMS2023	Os_Chr03	31665257	31665272	(TA)8	(TA)8	(TA)8	(TA)9	(TA)8	(TA)8	(TA)8	(TA)8	(TA)8	(TA)6	(TA)8	-	Introns/Intergenic	-	-	ACTCCATCCGTTTCACAATG	TGCACCAATTTTCGTATTGC	208	P	A	A	NA
PMS2024	Os_Chr03	31672375	31672416	(TA)21	(TA)21	(TA)14	(TA)21	(TA)21	(TA)21	(TA)21	(TA)21	(TA)21	(TA)7	(TA)8	-	Introns/Intergenic	-	-	TTGTGAGGGGGTTTTCTGTC	TGATGAAGGGAGTTGGTGGT	259	P	A	A	NA
PMS2025	Os_Chr03	31751255	31751275	(TGG)7	(TGG)7	(TGG)7	(TGG)7	(TGG)7	(TGG)7	(TGG)7	(TGG)7	(TGG)7	(TGG)6	(TGG)7	LOC_Os03g55760	CDS	OsKANADI4	CODON_CHANGE_PLUS_CODON_DELETION	GCGGCTTAAATTGACCATTGC	AGCTACAGATCCCAACCATGTCG	179	A	P	P	RM15975
PMS2026	Os_Chr03	31849543	31849563	(GCG)7	(GCG)7	(GCG)7	(GCG)7	(GCG)7	(GCG)5	(GCG)7	(GCG)7	(GCG)7	(GCG)7	(GCG)7	LOC_Os03g55940	CDS	expressed protein	CODON_DELETION	GTCGTCGAGGTGGAGGACT	AGAGGGACGCTGTGGAGAG	217	P	A	A	NA
PMS2027	Os_Chr03	31934209	31934229	(CGC)7	(CGC)7	(CGC)7	(CGC)7	(CGC)7	(CGC)7	(CGC)7	(CGC)7	(CGC)7	(CGC)6	(CGC)7	LOC_Os03g56060	URR	CSLC9 - cellulose synthase-like family C, expressed	-	TTGGGGTTCTCCATCTTGAC	GGTGAAGTGTGGGAGGCTAA	210	A	A	A	NA

Polymorphic SSR (PMS) markers	Chromosomes	Start physical positions (bp)	End physical positions (bp)	Nipponbare	Kasalath	Nagina22	IR64	Pokkali	Bala	Tainung67	Azucena	Moroberekan	*Oryza rufipogon*	*Oryza nivara*	MSU gene locus IDs	Structural annotation	Functional annotation	SSR functional effects	Forward primers (5'-3')	Reverse primers (5'-3')	Amplified product size (bp)	Markers exhibiting polymorphism within *indica* rice	Transcrption factor genes-derived markers	Known cloned genes-derived markers	PMS markers corresponding with RM markers
PMS2028	Os_Chr03	31977967	31977984	(GCC)6	(GCC)6	(GCC)5	(GCC)5	(GCC)5	(GCC)5	(GCC)6	(GCC)6	(GCC)6	(GCC)6	(GCC)5	-	Introns/Intergenic	-	-	GCTACTCCTCCCCTGCGT	CTTTGGTTTTGACGGCAGTT	261	P	A	A	NA
PMS2029	Os_Chr03	31991501	31991524	(GCG)8	(GCG)8	(GCG)8	(GCG)8	(GCG)8	(GCG)8	(GCG)8	(GCG)5	(GCG)8	(GCG)8	(GCG)8	LOC_Os03g56140	CDS	homeobox protein rough sheath 1, putative, expressed	CODON_DELETION	ATGACCTTCCCGACAGTGGAAGC	GGCTGATCATGGGAACATCATGC	190	A	P	A	RM6508
PMS2030	Os_Chr03	31996713	31996730	(CTC)6	(CTC)6	(CTC)6	(CTC)5	(CTC)6	(CTC)6	(CTC)6	(CTC)6	(CTC)6	(CTC)6	(CTC)6	-	Introns/Intergenic	-	-	TTCCCCTCTCTCCCTCTGAT	CAGAGAAGTGGACAGCACGA	248	P	A	A	NA
PMS2031	Os_Chr03	32008690	32008710	(CGG)7	(CGG)5	(CGG)5	(CGG)7	(CGG)7	(CGG)5	(CGG)6	(CGG)7	(CGG)7	(CGG)5	(CGG)5	LOC_Os03g56160	CDS	lectin-like receptor kinase 7, putative, expressed	CODON_DELETION	GTTCGTCTACGCCGGCTTCACC	CTTCCCGACGAAGAAGACGATGC	256	P	A	A	RM15995
PMS2032	Os_Chr03	32015010	32015027	(CGC)6	(CGC)6	(CGC)6	(CGC)6	(CGC)6	(CGC)6	(CGC)6	(CGC)6	(CGC)6	(CGC)6	(CGC)5	LOC_Os03g56180	CDS	legume lectins beta domain containing protein, expressed	CODON_DELETION	AACGACGACATGATGCTGAA	GTCGACGAGTTCGCCTACA	227	A	A	A	NA
PMS2033	Os_Chr03	32027535	32027555	(AGC)7	(AGC)7	(AGC)7	(AGC)7	(AGC)7	(AGC)7	(AGC)6	(AGC)7	(AGC)7	(AGC)7	(AGC)7	-	Introns/Intergenic	-	-	TAGTCGCCAATCCCAACAAACC	AGTGGTGGTGGAGTGGAGTACG	153	A	A	A	RM15996
PMS2034	Os_Chr03	32032942	32032959	(GCT)6	(GCT)6	(GCT)6	(GCT)6	(GCT)6	(GCT)6	(GCT)6	(GCT)6	(GCT)6	(GCT)6	(GCT)5	-	Introns/Intergenic	-	-	GCTCGCTTCCACCTAGTACG	GATGCAGATGGGAATCATGG	219	A	A	A	NA
PMS2035	Os_Chr03	32095363	32095422	(GA)30	(GA)30	(GA)30	(GA)30	(GA)30	(GA)30	(GA)30	(GA)30	(GA)30	(GA)30	(GA)25	-	Introns/Intergenic	-	-	TCGGAGCAGGAATACTCGAT	GAACCCTATCACCCCTCCTC	138	A	A	A	NA
PMS2036	Os_Chr03	32134841	32134862	(TC)11	(TC)11	(TC)11	(TC)11	(TC)11	(TC)7	(TC)11	(TC)11	(TC)11	(TC)11	(TC)11	-	Introns/Intergenic	-	-	ACATGCAATCGAATGATCCA	GCGCGAGCATAGGGTATCTA	138	P	A	A	NA
PMS2037	Os_Chr03	32142501	32142518	(CCG)6	(CCG)5	(CCG)6	(CCG)6	(CCG)6	(CCG)5	(CCG)6	(CCG)6	(CCG)6	(CCG)6	(CCG)5	LOC_Os03g56380	CDS	methyltransferase, putative, expressed	CODON_CHANGE_PLUS_CODON_DELETION	CCCACAACCATACTGCCACT	TCTAGCTCAGTCCCTGGCTC	226	P	A	A	NA
PMS2038	Os_Chr03	32187761	32187790	(GCT)10	(GCT)10	(GCT)10	(GCT)10	(GCT)10	(GCT)5	(GCT)10	(GCT)10	(GCT)10	(GCT)5	(GCT)10	LOC_Os03g56480	CDS	expressed protein	CODON_DELETION	GAATCACCATGCACCTGCTACC	AGCCTGTCCATGTGCTTACTGC	140	P	A	A	RM16003
PMS2039	Os_Chr03	32230268	32230510	(ATA)81	(ATA)81	(ATA)7	(ATA)7	(ATA)8	(ATA)81	(ATA)8	(ATA)7	(ATA)81	(ATA)8	(ATA)81	-	Introns/Intergenic	-	-	CCCGAGTCTTCATAGAGATATTCC	ATCCCTAGCTAGCCTTCCTTCC	487	P	A	A	RM6759
PMS2040	Os_Chr03	32257652	32257672	(CCT)7	(CCT)7	(CCT)7	(CCT)7	(CCT)7	(CCT)7	(CCT)7	(CCT)7	(CCT)7	(CCT)7	(CCT)5	-	Introns/Intergenic	-	-	AGCTTCGACACCCTCCACTTCC	CACACTCTCTCGCCTCCTCTCG	403	A	A	A	RM16007

Polymorphic SSR (PMS) markers	Chromosomes	Start physical positions (bp)	End physical positions (bp)	Nipponbare	Kasalath	Nagina22	IR64	Pokkali	Bala	Tainung67	Azucena	Moroberekan	*Oryza rufipogon*	*Oryza nivara*	MSU gene locus IDs	Structural annotation	Functional annotation	SSR functional effects	Forward primers (5'-3')	Reverse primers (5'-3')	Amplified product size (bp)	Markers exhibiting polymorphism within *indica* rice	Transcrption factor genes-derived markers	Known cloned genes-derived markers	PMS markers corresponding with RM markers
PMS2041	Os_Chr03	32265740	32265760	(GCT)7	(GCT)7	(GCT)7	(GCT)7	(GCT)7	(GCT)7	(GCT)7	(GCT)6	(GCT)7	(GCT)7	(GCT)7	LOC_Os03g56660	URR	calmodulin binding protein, putative, expressed	-	CAGTCCATCCCACTCAGAATCC	GGTGGTGTGGTGGTAGTAGTTGG	86	A	A	A	RM16008
PMS2042	Os_Chr03	32348979	32348994	(CT)8	(CT)8	(CT)8	(CT)8	(CT)8	(CT)7	(CT)8	(CT)8	(CT)8	(CT)8	(CT)8	-	Introns/Intergenic	-	-	CGAATCCAAATCCTTCCAAA	AGAGCGAGGTTAAAGGGAGG	269	P	A	A	NA
PMS2043	Os_Chr03	32541258	32541278	(CGC)7	(CGC)7	(CGC)7	(CGC)7	(CGC)7	(CGC)7	(CGC)7	(CGC)7	(CGC)7	(CGC)5	(CGC)7	LOC_Os03g57080	CDS	PLA IIIA/PLP7, putative, expressed	CODON_DELETION	GTTCTTCTGCGACAGCATCTCC	CTCCATTGGTGTCTTTGATCTTGC	134	A	A	A	RM16018
PMS2044	Os_Chr03	32575906	32575923	(GCC)6	(GCC)6	(GCC)6	(GCC)6	(GCC)6	(GCC)6	(GCC)6	(GCC)6	(GCC)6	(GCC)5	(GCC)6	LOC_Os03g57130	CDS	expressed protein	CODON_DELETION	AGACCTTGGAGAGGTCGAGG	TCCTCCTGTCCTGTCTCCC	273	A	A	A	NA
PMS2045	Os_Chr03	32578089	32578106	(GCG)6	(GCG)5	(GCG)6	(GCG)6	(GCG)6	(GCG)6	(GCG)5	(GCG)6	(GCG)6	(GCG)6	(GCG)6	-	Introns/Intergenic	-	-	AAAAGGGCCAAAAATTCAGG	ACCCGCATCAAGATTCTACG	276	P	A	A	NA
PMS2046	Os_Chr03	32645957	32645977	(GCG)7	(GCG)7	(GCG)7	(GCG)7	(GCG)7	(GCG)7	(GCG)7	(GCG)7	(GCG)7	(GCG)7	(GCG)6	LOC_Os03g57240	DRR	DST/ REG1	-	TGGAGCTTGGAGAAGAAGAACATGG	CAACAACGGCGAGGAGAACG	154	A	A	P	RM16027
PMS2047	Os_Chr03	32655606	32655620	(CTG)5	(CTG)5	(CTG)5	(CTG)5	(CTG)5	(CTG)5	(CTG)5	(CTG)5	(CTG)6	(CTG)5	(CTG)5	-	Introns/Intergenic	-	-	GGGGCAAACAATAAGCACAT	AAGGCCACAGAAGCTCACAT	216	A	A	A	NA
PMS2048	Os_Chr03	32713991	32714012	(AG)11	(AG)13	(AG)11	(AG)11	(AG)11	(AG)11	(AG)11	(AG)11	(AG)11	(AG)15	(AG)11	-	Introns/Intergenic	-	-	GCGAACTATGAGCATGCCAACC	GGATTACCTGGTGTGTGCAGTGTCC	104	P	A	A	RM16030
PMS2049	Os_Chr03	32730801	32730816	(TA)8	(TA)8	(TA)8	(TA)8	(TA)8	(TA)8	(TA)8	(TA)8	(TA)8	(TA)8	(TA)6	-	Introns/Intergenic	-	-	AAACCCTAGTTCTCCCCACC	AAATCGGAGCAGCAGAAAGA	134	A	A	A	NA
PMS2050	Os_Chr03	32736557	32736577	(GGC)7	(GGC)5	(GGC)7	(GGC)7	(GGC)7	(GGC)6	(GGC)7	(GGC)7	(GGC)7	(GGC)7	(GGC)7	LOC_Os03g57410	CDS	RING-H2 finger protein ATL5D, putative, expressed	CODON_DELETION	CCTCCTCATCACCACCATCACG	CCGTAGTTGTCGAGGCAGATCG	318	P	A	A	RM16031
PMS2051	Os_Chr03	32901341	32901354	(TA)7	(TA)6	(TA)6	(TA)6	(TA)6	(TA)6	(TA)7	(TA)7	(TA)7	(TA)7	(TA)7	-	Introns/Intergenic	-	-	AGCGTTTGACGGGATTTCTA	ACCATTAGCAGCTTTGCCAC	219	A	A	A	NA
PMS2052	Os_Chr03	32989319	32989336	(GCG)6	(GCG)6	(GCG)6	(GCG)5	(GCG)6	(GCG)6	(GCG)6	(GCG)6	(GCG)6	(GCG)6	(GCG)6	LOC_Os03g57930	CDS	expressed protein	CODON_DELETION	GGCTCCTCCCTTCTCTTCTC	CTCGGACTTCTACCCGCAG	189	P	P	A	NA
PMS2053	Os_Chr03	32989555	32989572	(CGG)6	(CGG)6	(CGG)6	(CGG)6	(CGG)6	(CGG)6	(CGG)5	(CGG)6	(CGG)6	(CGG)6	(CGG)6	LOC_Os03g57930	CDS	expressed protein	CODON_DELETION	CTGCGGGTAGAAGTCCGAG	GAGGAGACCGATGGTGAGAC	198	A	P	A	NA

Polymorphic SSR (PMS) markers	Chromosomes	Start physical positions (bp)	End physical positions (bp)	Nipponbare	Kasalath	Nagina22	IR64	Pokkali	Bala	Tainung67	Azucena	Moroberekan	*Oryza rufipogon*	*Oryza nivara*	MSU gene locus IDs	Structural annotation	Functional annotation	SSR functional effects	Forward primers (5'-3')	Reverse primers (5'-3')	Amplified product size (bp)	Markers exhibiting polymorphism within *indica* rice	Transcrption factor genes-derived markers	Known cloned genes-derived markers	PMS markers corresponding with RM markers
PMS2054	Os_Chr03	33033116	33033129	(AT)7	(AT)11	(AT)7	(AT)7	(AT)7	(AT)8	(AT)7	(AT)7	(AT)7	(AT)7	(AT)7	-	Introns/Intergenic	-	-	GACACGAGATTCATCCGAGC	GAATCCCTCTAGGTGATCGCT	280	P	A	A	NA
PMS2055	Os_Chr03	33091564	33091584	(CGG)7	(CGG)6	(CGG)7	(CGG)7	(CGG)7	(CGG)7	(CGG)7	(CGG)7	(CGG)7	(CGG)7	(CGG)7	LOC_Os03g58120	CDS	PPR repeat domain containing protein, putative, expressed	CODON_DELETION	ATGTGCTGCTCTTTCTCTGATGC	CGAACTCAGAAGAACACCCTCTAGC	94	P	A	A	RM16046
PMS2056	Os_Chr03	33105981	33106007	(CCT)9	(CCT)9	(CCT)9	(CCT)9	(CCT)9	(CCT)9	(CCT)9	(CCT)9	(CCT)9	(CCT)9	(CCT)5	LOC_Os03g58160	URR	OsHsfA2e	-	ATCTCCTGAGCTCGAAGCTCTCC	ACAGAATCAGAAAGCGCACACC	194	A	P	P	RM16048
PMS2057	Os_Chr03	33139058	33139075	(CGG)6	(CGG)6	(CGG)6	(CGG)6	(CGG)6	(CGG)6	(CGG)5	(CGG)6	(CGG)6	(CGG)6	(CGG)6	LOC_Os03g58190	CDS	expressed protein	CODON_DELETION	CTGAGCTGCAGCAAGTGTTC	AAGCTCCAATCTCTCGGCTT	275	A	A	A	NA
PMS2058	Os_Chr03	33173358	33173379	(AG)11	(AG)10	(AG)11	(AG)11	(AG)11	(AG)11	(AG)11	(AG)11	(AG)11	(AG)11	(AG)11	-	Introns/Intergenic	-	-	AACTGATGTGATCTTGCGTCAGG	AATCTTTCCATCACCGCTCACC	136	P	A	A	RM16053
PMS2059	Os_Chr03	33191277	33191297	(CGC)7	(CGC)7	(CGC)7	(CGC)7	(CGC)7	(CGC)7	(CGC)7	(CGC)7	(CGC)7	(CGC)7	(CGC)5	LOC_Os03g58250	CDS	REB	CODON_DELETION	CGAGGTCCTTCTCCAGCTTCTGC	GTGGTGCCGGTCGATGTTAAGC	117	A	P	P	RM16055
PMS2060	Os_Chr03	33193642	33193653	(TC)6	(TC)6	(TC)6	(TC)6	(TC)6	(TC)6	(TC)6	(TC)6	(TC)6	(TC)7	(TC)7	-	Introns/Intergenic	-	-	GTTTCGCCATGTAGGAAAGG	AAATCCCCCGAGAAAAAGAA	192	A	A	A	NA
PMS2061	Os_Chr03	33227732	33227755	(TA)12	(TA)10	(TA)12	(TA)12	(TA)6	(TA)12	(TA)12	(TA)12	(TA)7	(TA)12	(TA)9	-	Introns/Intergenic	-	-	GATGGCTTCACTCCTGAAAGTGG	CGGTGGTGCTCTTCTCTTACTTCG	533	P	A	A	RM16058
PMS2062	Os_Chr03	33231866	33231897	(AT)16	(AT)16	(AT)16	(AT)16	(AT)16	(AT)16	(AT)16	(AT)16	(AT)16	(AT)10	(AT)16	-	Introns/Intergenic	-	-	ATTTGAATCATGGAGCCCAG	GAGCGTACGTGCACTACCAA	229	A	A	A	NA
PMS2063	Os_Chr03	33250363	33250384	(TC)11	(TC)7	(TC)11	(TC)11	(TC)11	(TC)11	(TC)11	(TC)11	(TC)11	(TC)11	(TC)11	-	Introns/Intergenic	-	-	AATGACGCTGCAGGCTAGATACG	GTCAGACACGAGATCACGAGAGG	79	P	A	A	RM16060
PMS2064	Os_Chr03	33262499	33262514	(TA)8	(TA)8	(TA)8	(TA)8	(TA)8	(TA)8	(TA)8	(TA)8	(TA)7	(TA)8	(TA)8	-	Introns/Intergenic	-	-	TTTGTCATGAATATGGTGGTTG	GCAAACTTAGATTTTTAATAACAGCTT	266	A	A	A	NA
PMS2065	Os_Chr03	33310440	33310461	(CT)11	(CT)11	(CT)11	(CT)11	(CT)8	(CT)11	(CT)11	(CT)11	(CT)11	(CT)11	(CT)11	LOC_Os03g58480	URR	seed specific protein Bn15D14A, putative, expressed	-	CCACGCACATTTTCACACTT	TAGCTACAGGAGAGACCGGG	242	P	A	A	NA
PMS2066	Os_Chr03	33368241	33368261	(GAT)7	(GAT)7	(GAT)7	(GAT)7	(GAT)7	(GAT)5	(GAT)7	(GAT)7	(GAT)7	(GAT)7	(GAT)7	LOC_Os03g58590	CDS	expressed protein	CODON_DELETION	ACGGAAGCATCTACATCGTCTGG	TCGCTAGAGTTACAGCATGGAAGG	189	P	A	A	RM16066

Polymorphic SSR (PMS) markers	Chromosomes	Start physical positions (bp)	End physical positions (bp)	Nipponbare	Kasalath	Nagina22	IR64	Pokkali	Bala	Tainung67	Azucena	Moroberekan	*Oryza rufipogon*	*Oryza nivara*	MSU gene locus IDs	Structural annotation	Functional annotation	SSR functional effects	Forward primers (5'-3')	Reverse primers (5'-3')	Amplified product size (bp)	Markers exhibiting polymorphism within *indica* rice	Transcrption factor genes-derived markers	Known cloned genes-derived markers	PMS markers corresponding with RM markers
PMS2067	Os_Chr03	33417843	33417860	(GGC)6	(GGC)6	(GGC)6	(GGC)6	(GGC)6	(GGC)6	(GGC)6	(GGC)5	(GGC)6	(GGC)6	(GGC)6	LOC_Os03g58670	CDS	LTPL119 - Protease inhibitor/seed storage/LTP family protein precursor, expressed	CODON_DELETION	AGCGTAGAACACATGACCCC	GTCGACGCCATTCGAGAC	244	A	A	A	NA
PMS2068	Os_Chr03	33480110	33480127	(CGC)6	(CGC)6	(CGC)6	(CGC)7	(CGC)6	(CGC)7	(CGC)6	(CGC)6	(CGC)6	(CGC)5	(CGC)6	LOC_Os03g58780	URR	expressed protein	-	GTACCCCCTCCCGTCCTC	GTCTCGTACTCTCGCTTCCG	207	P	A	A	NA
PMS2069	Os_Chr03	33528475	33528501	(GAG)9	(GAG)9	(GAG)9	(GAG)9	(GAG)9	(GAG)9	(GAG)9	(GAG)9	(GAG)9	(GAG)8	(GAG)7	LOC_Os03g58900	CDS	galactosyltransferase family protein, putative, expressed	CODON_DELETION	ATCGATGGAAGACGAGTTGC	ACCTCCTCTTCCTGCTCCTC	245	A	A	A	NA
PMS2070	Os_Chr03	33558091	33558134	(AT)22	(AT)13	(AT)22	(AT)22	(AT)22	(AT)22	(AT)22	(AT)22	(AT)22	(AT)22	(AT)12	-	Introns/Intergenic	-	-	TCTACGTGTAACCAACACATCACC	CGATGAACGCTACTATCCAGAGG	639	P	A	A	RM16082
PMS2071	Os_Chr03	33576026	33576043	(CGC)6	(CGC)6	(CGC)5	(CGC)6	(CGC)6	(CGC)6	(CGC)6	(CGC)6	(CGC)6	(CGC)6	(CGC)6	LOC_Os03g58960	URR	DHHC zinc finger domain containing protein, expressed	-	ATCTGTGCATCTCTCCCCAC	GAGAGAAGACGACCTGACCG	231	P	A	A	NA
PMS2072	Os_Chr03	33604082	33604127	(TA)23	(TA)23	(TA)8	(TA)23	(TA)17	(TA)13	(TA)23	(TA)23	(TA)23	(TA)6	(TA)23	-	Introns/Intergenic	-	-	ATGATGCGGGTCGAATTTAAGG	TCTCCTTTCTTTGTGACCCATGC	616	P	A	A	RM16084
PMS2073	Os_Chr03	33638733	33638756	(CGC)8	(CGC)8	(CGC)8	(CGC)8	(CGC)8	(CGC)8	(CGC)7	(CGC)8	(CGC)8	(CGC)8	(CGC)8	LOC_Os03g59090	CDS	retrotransposon protein, putative, unclassified, expressed	CODON_CHANGE_PLUS_CODON_DELETION	AGTAGCCATGGATGCCCTCTCC	AGTCGAACCGCTCCTCCTTACC	231	A	A	A	RM16086
PMS2074	Os_Chr03	33653824	33653891	(TATC)17	(TATC)17	(TATC)5	(TATC)17	(TATC)17	(TATC)17	(TATC)17	(TATC)17	(TATC)17	(TATC)17	(TATC)17	-	Introns/Intergenic	-	-	CCAGGACTACGTCCATGCTT	TGCCTTCTTTTCGAGCTGTT	203	P	A	A	NA
PMS2075	Os_Chr03	33686587	33686607	(GAA)7	(GAA)5	(GAA)5	(GAA)7	(GAA)7	(GAA)7	(GAA)7	(GAA)7	(GAA)7	(GAA)7	(GAA)7	LOC_Os03g59152	CDS	lethal leaf spot 1, putative, expressed	CODON_DELETION	TACTCTGATGATGCAGGCTGACG	GGTGAACAACACCACCCTCACC	73	P	A	A	RM16090
PMS2076	Os_Chr03	33729563	33729576	(GA)7	(GA)7	(GA)7	(GA)7	(GA)7	(GA)7	(GA)7	(GA)7	(GA)7	(GA)7	(GA)6	-	Introns/Intergenic	-	-	TTTCCAAAGCTCACCGTACC	AGTGGGTCCCATCTTACGTG	194	A	A	A	NA
PMS2077	Os_Chr03	33764560	33764609	(TA)25	(TA)25	(TA)25	(TA)25	(TA)25	(TA)25	(TA)25	(TA)25	(TA)6	(TA)25	(TA)25	-	Introns/Intergenic	-	-	GGAAGGAACACTAAATTTTGGAA	AGTTTCGCAGAAGATGGACC	179	A	A	A	NA
PMS2078	Os_Chr03	33772173	33772190	(GCA)6	(GCA)6	(GCA)6	(GCA)6	(GCA)5	(GCA)6	(GCA)6	(GCA)6	(GCA)6	(GCA)6	(GCA)6	LOC_Os03g59320	CDS	expressed protein	CODON_DELETION	GAAGAGAGTGGCAGGGTGAG	GGATGTCCTCGAAACCTTCA	265	P	A	A	NA
PMS2079	Os_Chr03	33833684	33833701	(GGC)6	(GGC)6	(GGC)6	(GGC)6	(GGC)6	(GGC)6	(GGC)5	(GGC)6	(GGC)6	(GGC)6	(GGC)6	-	Introns/Intergenic	-	-	TGCCATGATTTTTGCTTCAG	CCGGAGTTGAACATGTCGAG	183	A	A	A	NA

Polymorphic SSR (PMS) markers	Chromosomes	Start physical positions (bp)	End physical positions (bp)	Nipponbare	Kasalath	Nagina22	IR64	Pokkali	Bala	Tainung67	Azucena	Moroberekan	*Oryza rufipogon*	*Oryza nivara*	MSU gene locus IDs	Structural annotation	Functional annotation	SSR functional effects	Forward primers (5'-3')	Reverse primers (5'-3')	Amplified product size (bp)	Markers exhibiting polymorphism within *indica* rice	Transcrption factor genes-derived markers	Known cloned genes-derived markers	PMS markers corresponding with RM markers
PMS2080	Os_Chr03	33902580	33902597	(CTA)6	(CTA)6	(CTA)6	(CTA)6	(CTA)6	(CTA)6	(CTA)6	(CTA)6	(CTA)6	(CTA)5	(CTA)6	-	Introns/Intergenic	-	-	CCACCACTACCACTACGGCT	AGGCAAGCACGTACGCTTAT	134	A	A	A	NA
PMS2081	Os_Chr03	33914774	33914831	(AT)29	(AT)29	(AT)29	(AT)29	(AT)29	(AT)11	(AT)29	(AT)29	(AT)29	(AT)29	(AT)29	-	Introns/Intergenic	-	-	ATGGCAACCTAACCTAAGCG	GAAATTTGGATGCCAATGGT	204	P	A	A	NA
PMS2082	Os_Chr03	33953376	33953389	(CT)7	(CT)7	(CT)7	(CT)7	(CT)6	(CT)6	(CT)7	(CT)7	(CT)7	(CT)7	(CT)7	-	Introns/Intergenic	-	-	AACGAGAGAGCGAGAGAACG	TCTCCAAATCAAACCTTCCG	191	P	A	A	NA
PMS2083	Os_Chr03	33962413	33962430	(GGC)6	(GGC)5	(GGC)6	(GGC)6	(GGC)5	(GGC)6	(GGC)6	(GGC)6	(GGC)6	(GGC)6	(GGC)5	LOC_Os03g59650	URR	BRCA1 C Terminus domain containing protein, expressed	-	ACAAAAGAAAGAGGGGGAGG	TACAGACCAACGCACTCAGC	235	P	A	A	NA
PMS2084	Os_Chr03	34010309	34010374	(TA)33	(TA)9	(TA)9	(TA)10	(TA)9	(TA)9	(TA)9	(TA)9	(TA)7	(TA)9	(TA)9	-	Introns/Intergenic	-	-	TCAATTTCAACAATGGTGCC	CAATGGGTCTTATCTTTTCGC	246	P	A	A	NA
PMS2085	Os_Chr03	34100266	34100289	(CT)12	(CT)12	(CT)12	(CT)12	(CT)12	(CT)12	(CT)11	(CT)12	(CT)12	(CT)12	(CT)11	-	Introns/Intergenic	-	-	TCGAGTTCAATTCCGAATCC	CTCTCGCACACGAGAGCA	240	A	A	A	NA
PMS2086	Os_Chr03	34201844	34201861	(GA)9	(GA)9	(GA)9	(GA)9	(GA)9	(GA)9	(GA)8	(GA)9	(GA)9	(GA)9	(GA)9	-	Introns/Intergenic	-	-	CTCGTCGACCTCCTCCAC	GGAAACACAGTGGTTGCAGA	244	A	A	A	NA
PMS2087	Os_Chr03	34226198	34226241	(TA)22	(TA)20	(TA)22	(TA)22	(TA)22	(TA)22	(TA)22	(TA)22	(TA)22	(TA)22	(TA)22	-	Introns/Intergenic	-	-	TGAGCACGGAATTTATGGTTGC	ACATTTAGGCACCGGATAGTTGG	439	P	A	A	RM16129
PMS2088	Os_Chr03	34279136	34279167	(AG)16	(AG)16	(AG)16	(AG)16	(AG)16	(AG)15	(AG)16	(AG)16	(AG)16	(AG)16	(AG)16	-	Introns/Intergenic	-	-	CAGCATTGCAGTCTTGCTTGC	GGGTAGCAGTAGGTAGTCAGAGTTGG	211	P	A	A	RM16131
PMS2089	Os_Chr03	34407587	34407600	(TG)7	(TG)7	(TG)7	(TG)7	(TG)7	(TG)6	(TG)7	(TG)7	(TG)7	(TG)7	(TG)7	-	Introns/Intergenic	-	-	CTCACTCCCTCGATCTACGC	ATGCTTCGATTCATTCACCC	164	P	A	A	NA
PMS2090	Os_Chr03	34438254	34438283	(GGGGA)6	(GGGGA)6	(GGGGA)6	(GGGGA)6	(GGGGA)6	(GGGGA)6	(GGGGA)6	(GGGGA)6	(GGGGA)6	(GGGGA)5	(GGGGA)6	LOC_Os03g60600	URR	zinc knuckle domain containing protein, expressed	-	ACCAGCAAATCAGGATCGAG	CAGTCACCTCGCAATCTCAC	271	A	A	A	NA
PMS2091	Os_Chr03	34456146	34456163	(CGC)6	(CGC)6	(CGC)6	(CGC)6	(CGC)6	(CGC)6	(CGC)6	(CGC)6	(CGC)6	(CGC)5	(CGC)6	LOC_Os03g60630	CDS	dof zinc finger domain containing protein, putative, expressed	CODON_DELETION	CTAGGGTTTCCCGGATTGAG	CTCAAGAAGGGGAATTGCTG	128	A	P	A	NA
PMS2092	Os_Chr03	34520883	34520912	(CTCAT)6	(CTCAT)5	(CTCAT)6	(CTCAT)6	(CTCAT)6	(CTCAT)6	(CTCAT)6	(CTCAT)6	(CTCAT)6	(CTCAT)6	(CTCAT)6	-	Introns/Intergenic	-	-	TATCAGGAGGAGGTAGCCGA	ATCGATCAAAACCTTGCTGC	278	P	A	A	NA

Polymorphic SSR (PMS) markers	Chromosomes	Start physical positions (bp)	End physical positions (bp)	Nipponbare	Kasalath	Nagina22	IR64	Pokkali	Bala	Tainung67	Azucena	Moroberekan	*Oryza rufipogon*	*Oryza nivara*	MSU gene locus IDs	Structural annotation	Functional annotation	SSR functional effects	Forward primers (5'-3')	Reverse primers (5'-3')	Amplified product size (bp)	Markers exhibiting polymorphism within *indica* rice	Transcrption factor genes-derived markers	Known cloned genes-derived markers	PMS markers corresponding with RM markers
PMS2093	Os_Chr03	34535361	34535374	(GA)7	(GA)7	(GA)7	(GA)7	(GA)7	(GA)7	(GA)7	(GA)6	(GA)7	(GA)7	(GA)7	-	Introns/Intergenic	-	-	GACGGCCTCCTCCTCTCC	ATGACGGCTTAGTCAGCGTT	153	A	A	A	NA
PMS2094	Os_Chr03	34688626	34688643	(CGG)6	(CGG)6	(CGG)6	(CGG)5	(CGG)6	(CGG)6	(CGG)5	(CGG)5	(CGG)6	(CGG)6	(CGG)6	LOC_Os03g61060	CDS	protein kinase domain containing protein, expressed	CODON_DELETION	TTGTTAGACCCAACCCCTCA	GGTCGGTCAGGAGGGAATAC	252	P	A	A	NA
PMS2095	Os_Chr03	34814790	34814807	(CGC)6	(CGC)6	(CGC)6	(CGC)6	(CGC)6	(CGC)6	(CGC)6	(CGC)6	(CGC)6	(CGC)6	(CGC)5	LOC_Os03g61319	CDS	RD26, putative, expressed	CODON_DELETION	CGCCATAGGAGAACTTCTGG	TGGTACTTCTTCTGCTCCCC	178	A	P	A	NA
PMS2096	Os_Chr03	34828846	34828863	(CCG)6	(CCG)6	(CCG)6	(CCG)6	(CCG)6	(CCG)6	(CCG)6	(CCG)6	(CCG)6	(CCG)6	(CCG)5	LOC_Os03g61360	CDS	hydrolase, alpha/beta fold family domain containing protein, expressed	CODON_DELETION	TACCAGAGCTCCGGGAATC	GAAGTGTGCAAACACACGCT	222	A	A	A	NA
PMS2097	Os_Chr03	34829175	34829188	(GA)7	(GA)7	(GA)7	(GA)7	(GA)7	(GA)7	(GA)7	(GA)7	(GA)7	(GA)7	(GA)6	-	Introns/Intergenic	-	-	CATCCGAACTCTCGTGTGAA	CAGCCGCTATGATCACACTC	181	A	A	A	NA
PMS2098	Os_Chr03	34857145	34857162	(GCG)6	(GCG)6	(GCG)5	(GCG)6	(GCG)6	(GCG)6	(GCG)6	(GCG)6	(GCG)6	(GCG)6	(GCG)6	LOC_Os03g61430	CDS	uncharacterized Cys-rich domain containing protein, putative, expressed	CODON_DELETION	CCGAATACGAGAGGTGATCG	GCTCATCCAGGAGTACAGGG	163	P	A	A	NA
PMS2099	Os_Chr03	34873979	34873996	(GCG)6	(GCG)6	(GCG)6	(GCG)6	(GCG)6	(GCG)5	(GCG)6	(GCG)6	(GCG)6	(GCG)5	(GCG)6	LOC_Os03g61480	CDS	uncharacterized Cys-rich domain containing protein, putative, expressed	CODON_DELETION	AGCTTTTTCTAACGGCCCAT	GGATGTACCTGCACCTACCG	245	P	A	A	NA
PMS2100	Os_Chr03	34894451	34894468	(GTC)6	(GTC)6	(GTC)6	(GTC)6	(GTC)6	(GTC)6	(GTC)6	(GTC)6	(GTC)6	(GTC)6	(GTC)5	-	Introns/Intergenic	-	-	TCACTGTTAATAGGCGTGCG	CCTCACCCTACCCTTCTTCC	237	A	A	A	NA
PMS2101	Os_Chr03	34912430	34912444	(TGC)5	(TGC)6	(TGC)5	(TGC)5	(TGC)5	(TGC)5	(TGC)5	(TGC)5	(TGC)5	(TGC)5	(TGC)5	-	Introns/Intergenic	-	-	ATTTTCGCAAAGCAGCAAAC	GCCGACACTAGAACCCAAAA	162	P	A	A	NA
PMS2102	Os_Chr03	34969024	34969103	(AT)40	(AT)35	(AT)40	(AT)40	(AT)11	(AT)10	(AT)6	(AT)14	(AT)40	(AT)40	(AT)40	-	Introns/Intergenic	-	-	TCCATGGGCTTCTTAGTAATGC	GTTGAGAAAGAGAACGGGAAGG	382	P	A	A	RM16150
PMS2103	Os_Chr03	35036284	35036297	(TC)7	(TC)11	(TC)7	(TC)7	(TC)7	(TC)7	(TC)7	(TC)7	(TC)7	(TC)7	(TC)7	-	Introns/Intergenic	-	-	TGGAATCCAAAGACGAGACC	CACTCATCTCGTCAGCCTCA	164	P	A	A	NA
PMS2104	Os_Chr03	35146692	35146707	(TG)8	(TG)8	(TG)8	(TG)8	(TG)8	(TG)8	(TG)8	(TG)8	(TG)8	(TG)9	(TG)8	-	Introns/Intergenic	-	-	GATTAATTGTGCACGGATGC	TCTTGAATGGAAAGGATGGC	267	A	A	A	NA
PMS2105	Os_Chr03	35165829	35165861	(TAT)11	(TAT)11	(TAT)11	(TAT)5	(TAT)11	(TAT)11	(TAT)11	(TAT)11	(TAT)11	(TAT)11	(TAT)11	-	Introns/Intergenic	-	-	AAGTTAGGCGCTGAAACTCATGG	TGCATCTTTCCATCTTCCAAGC	372	P	A	A	RM16158

Polymorphic SSR (PMS) markers	Chromosomes	Start physical positions (bp)	End physical positions (bp)	Nipponbare	Kasalath	Nagina22	IR64	Pokkali	Bala	Tainung67	Azucena	Moroberekan	*Oryza rufipogon*	*Oryza nivara*	MSU gene locus IDs	Structural annotation	Functional annotation	SSR functional effects	Forward primers (5'-3')	Reverse primers (5'-3')	Amplified product size (bp)	Markers exhibiting polymorphism within *indica* rice	Transcrption factor genes-derived markers	Known cloned genes-derived markers	PMS markers corresponding with RM markers
PMS2106	Os_Chr03	35201157	35201177	(CCG)7	(CCG)7	(CCG)7	(CCG)7	(CCG)7	(CCG)7	(CCG)5	(CCG)7	(CCG)7	(CCG)7	(CCG)7	LOC_Os03g62120	CDS	heat shock protein DnaJ, putative, expressed	CODON_DELETION	GTGAACTGCCTCGACTGCAACC	GACACCATGGAGAAGGCTTTGC	257	A	A	A	RM16159
PMS2107	Os_Chr03	35204021	35204060	(TAGA)10	(TAGA)10	(TAGA)10	(TAGA)10	(TAGA)10	(TAGA)10	(TAGA)10	(TAGA)10	(TAGA)10	(TAGA)10	(TAGA)5	-	Introns/Intergenic	-	-	CTCGGGTACTCTGCTTCTGG	AAAAATGACTATAGTGGACCGACA	235	A	A	A	NA
PMS2108	Os_Chr03	35233819	35233842	(AAT)8	(AAT)8	(AAT)8	(AAT)8	(AAT)8	(AAT)8	(AAT)8	(AAT)8	(AAT)8	(AAT)8	(AAT)6	-	Introns/Intergenic	-	-	CCAGCATCATCATTGTCATCATCG	ATCCATCCAGCAGGAGAAACAGG	388	A	A	A	RM6712
PMS2109	Os_Chr03	35240038	35240057	(TC)10	(TC)10	(TC)10	(TC)10	(TC)10	(TC)10	(TC)10	(TC)10	(TC)10	(TC)7	(TC)10	-	Introns/Intergenic	-	-	GTGGCAACTAATTAAACACGCACACG	CAACCAACCTAGATTCCGATGAACG	167	A	A	A	RM16163
PMS2110	Os_Chr03	35264437	35264460	(GGC)8	(GGC)8	(GGC)8	(GGC)8	(GGC)8	(GGC)5	(GGC)8	(GGC)8	(GGC)8	(GGC)8	(GGC)6	LOC_Os03g62260	URR	FAD binding domain containing protein, expressed	-	AGGAGGCAGTAAGGCTCAATCG	CCACAAACTCGCAAACCAAACC	165	P	A	A	RM6135
PMS2111	Os_Chr03	35271931	35271962	(AT)16	(AT)16	(AT)16	(AT)16	(AT)16	(AT)16	(AT)16	(AT)16	(AT)16	(AT)9	(AT)16	-	Introns/Intergenic	-	-	TAGGACGAATGGAGTACTAAGCAACC	GAGTGAGCATGCATTTCTGTCC	405	A	A	A	RM16167
PMS2112	Os_Chr03	35288531	35288554	(AC)12	(AC)12	(AC)13	(AC)12	(AC)12	(AC)12	(AC)12	(AC)12	(AC)12	(AC)12	(AC)12	-	Introns/Intergenic	-	-	CTTCTCAGATTGATCTCCCATTCC	GGGAGAGAGGAAGAAGACAAGG	495	P	A	A	RM514
PMS2113	Os_Chr03	35421863	35421944	(TA)41	(TA)41	(TA)8	(TA)10	(TA)8	(TA)41	(TA)41	(TA)14	(TA)41	(TA)41	(TA)41	-	Introns/Intergenic	-	-	TTAGCTCCTGCTGGTACATACGC	CTGAGAGGCGCGCTACTTCC	470	P	A	A	RM16174
PMS2114	Os_Chr03	35468291	35468311	(CGG)7	(CGG)7	(CGG)6	(CGG)7	(CGG)6	(CGG)7	(CGG)7	(CGG)7	(CGG)7	(CGG)7	(CGG)6	LOC_Os03g62670	CDS	OsHOL1	CODON_CHANGE_PLUS_CODON_DELETION	ATATCTGTGACACGGCCATCC	GTAGTCGAAGATGAGATGGAACG	242	P	A	P	RM16176
PMS2115	Os_Chr03	35485931	35485957	(AAT)9	(AAT)9	(AAT)9	(AAT)8	(AAT)9	(AAT)9	(AAT)9	(AAT)9	(AAT)8	(AAT)9	(AAT)9	-	Introns/Intergenic	-	-	GTACTATGCGAGCGTAGCAATCC	CCCAATCCATCCTGGTTATTAGC	95	P	A	A	RM5665
PMS2116	Os_Chr03	35537823	35537846	(AT)12	(AT)12	(AT)12	(AT)12	(AT)12	(AT)12	(AT)12	(AT)12	(AT)6	(AT)12	(AT)12	-	Introns/Intergenic	-	-	CAATCTACCTAGCACCTGCTGTTCC	TCCTCAAACACACCCTAAGTTGC	659	A	A	A	RM16180
PMS2117	Os_Chr03	35581906	35581919	(TC)7	(TC)6	(TC)7	(TC)7	(TC)6	(TC)7	(TC)7	(TC)7	(TC)7	(TC)7	(TC)7	-	Introns/Intergenic	-	-	CGAGACATACCCGTGGTTTT	CGGTGGAGATGGAGAGAGAG	197	P	A	A	NA
PMS2118	Os_Chr03	35658008	35658028	(CGG)7	(CGG)7	(CGG)7	(CGG)7	(CGG)7	(CGG)7	(CGG)7	(CGG)7	(CGG)7	(CGG)5	(CGG)7	LOC_Os03g63060	CDS	P21-Rho-binding domain containing protein, putative, expressed	CODON_CHANGE_PLUS_CODON_DELETION	GCACAGAGTGAGTTCTTGATCACC	CTCCCTCTCCTCTTCGTCTTCC	285	A	A	A	RM16190

Polymorphic SSR (PMS) markers	Chromosomes	Start physical positions (bp)	End physical positions (bp)	Nipponbare	Kasalath	Nagina22	IR64	Pokkali	Bala	Tainung67	Azucena	Moroberekan	*Oryza rufipogon*	*Oryza nivara*	MSU gene locus IDs	Structural annotation	Functional annotation	SSR functional effects	Forward primers (5'-3')	Reverse primers (5'-3')	Amplified product size (bp)	Markers exhibiting polymorphism within *indica* rice	Transcrption factor genes-derived markers	Known cloned genes-derived markers	PMS markers corresponding with RM markers
PMS2119	Os_Chr03	35676799	35676828	(AG)15	(AG)14	(AG)15	(AG)15	(AG)15	(AG)15	(AG)15	(AG)15	(AG)15	(AG)15	(AG)15	LOC_Os03g63110	URR	prefoldin, putative, expressed	-	GTGGGCAGATTATGGTACGATGC	AAGCATGTCCCTGCAACTTTAGG	273	P	A	A	RM1221
PMS2120	Os_Chr03	35830134	35830154	(GCG)7	(GCG)7	(GCG)7	(GCG)7	(GCG)7	(GCG)5	(GCG)7	(GCG)7	(GCG)7	(GCG)7	(GCG)7	LOC_Os03g63410	URR	elongation factor Tu, putative, expressed	-	GATCTGATCCAAGAAGGACTCACG	CCGTCTCTCTCCTCCTCTCACC	277	P	A	A	RM16197
PMS2121	Os_Chr03	35862681	35862698	(TCG)6	(TCG)6	(TCG)6	(TCG)6	(TCG)6	(TCG)7	(TCG)6	(TCG)6	(TCG)6	(TCG)6	(TCG)6	-	Introns/Intergenic	-	-	TCCTCGACGAGATCGAGAAC	GCTTCAGGGTCGTTCCAATA	169	P	A	A	NA
PMS2122	Os_Chr03	35887968	35887991	(GA)12	(GA)12	(GA)12	(GA)12	(GA)12	(GA)12	(GA)11	(GA)12	(GA)12	(GA)12	(GA)12	-	Introns/Intergenic	-	-	GTGGTAGGGCGAAATGATCTGC	ATCACGCGCTCCTACCTCACC	232	A	A	A	RM16200
PMS2123	Os_Chr03	35948483	35948497	(CTT)5	(CTT)6	(CTT)5	(CTT)6	(CTT)6	(CTT)5	(CTT)5	(CTT)6	(CTT)6	(CTT)5	(CTT)5	LOC_Os03g63644	CDS	expressed protein	CODON_INSERTION	CCTTCCTTTTTGTTTGCTGC	TACGGTGGTGCAGATGGTTA	167	P	A	A	NA
PMS2124	Os_Chr03	35954458	35954473	(TC)8	(TC)8	(TC)8	(TC)9	(TC)8	(TC)8	(TC)8	(TC)8	(TC)8	(TC)8	(TC)8	-	Introns/Intergenic	-	-	TACGTTCTTGCCTGCATCTG	AGAACGATTCCCTCTGCTCA	262	P	A	A	NA
PMS2125	Os_Chr03	36067593	36067643	(AAG)17	(AAG)17	(AAG)17	(AAG)17	(AAG)17	(AAG)17	(AAG)17	(AAG)17	(AAG)17	(AAG)18	(AAG)17	-	Introns/Intergenic	-	-	AATGCTAATGGCGACTGACTTCG	ATGGGCTTGTTTGATTGCATCC	219	A	A	A	RM16211
PMS2126	Os_Chr03	36132915	36132928	(AT)7	(AT)6	(AT)7	(AT)7	(AT)7	(AT)7	(AT)7	(AT)7	(AT)7	(AT)7	(AT)7	-	Introns/Intergenic	-	-	GCTTCCCTGGAATTTCCTTC	TCCCTCTGTTTCAGGTTATAAGA	150	P	A	A	NA
PMS2127	Os_Chr03	36150616	36150631	(AG)8	(AG)8	(AG)6	(AG)8	(AG)8	(AG)6	(AG)8	(AG)8	(AG)8	(AG)8	(AG)8	-	Introns/Intergenic	-	-	AGCAGAGTGGGCTAAGCAAA	CTTATATACACGCTGCGCCA	214	P	A	A	NA
PMS2128	Os_Chr03	36197059	36197080	(TA)11	(TA)11	(TA)11	(TA)11	(TA)12	(TA)11	(TA)11	(TA)11	(TA)11	(TA)11	(TA)11	-	Introns/Intergenic	-	-	CTGCATGGAGAATAATGTGTGTCG	CGCAGACTAAACTCTGGCAACG	438	P	A	A	RM16223
PMS2129	Os_Chr03	36203152	36203175	(GA)12	(GA)12	(GA)12	(GA)12	(GA)12	(GA)12	(GA)12	(GA)10	(GA)12	(GA)12	(GA)12	-	Introns/Intergenic	-	-	GAGGGAGCGAAGAGGAGG	TAGTGTCCGTGCCATCTCAG	279	A	A	A	NA
PMS2130	Os_Chr03	36203680	36203693	(CT)7	(CT)7	(CT)7	(CT)7	(CT)7	(CT)7	(CT)6	(CT)7	(CT)7	(CT)7	(CT)7	-	Introns/Intergenic	-	-	CGGGTGAAAACCTAGTCCAA	CCTCACAGGAGGTTTGAGGA	196	A	A	A	NA
PMS2131	Os_Chr03	36206110	36206139	(CT)15	(CT)15	(CT)15	(CT)15	(CT)15	(CT)15	(CT)15	(CT)15	(CT)15	(CT)15	(CT)17	-	Introns/Intergenic	-	-	AGTCATCGTCGTCTTGTCCATCG	GGTCCAGAAAGAGGAGATGAGAGG	284	A	A	A	RM16229

Polymorphic SSR (PMS) markers	Chromosomes	Start physical positions (bp)	End physical positions (bp)	Nipponbare	Kasalath	Nagina22	IR64	Pokkali	Bala	Tainung67	Azucena	Moroberekan	*Oryza rufipogon*	*Oryza nivara*	MSU gene locus IDs	Structural annotation	Functional annotation	SSR functional effects	Forward primers (5'-3')	Reverse primers (5'-3')	Amplified product size (bp)	Markers exhibiting polymorphism within *indica* rice	Transcrption factor genes-derived markers	Known cloned genes-derived markers	PMS markers corresponding with RM markers
PMS2132	Os_Chr03	36293326	36293340	(ACA)5	(ACA)6	(ACA)6	(ACA)6	(ACA)6	(ACA)6	(ACA)5	(ACA)5	(ACA)5	(ACA)6	(ACA)6	LOC_Os03g64230	CDS	expressed protein	CODON_INSERTION	TCAAGTGCCTCATCTGCATC	ATCTCTGACGATGGAATCCG	278	A	A	A	NA
PMS2133	Os_Chr03	36408462	36408475	(GA)7	(GA)7	(GA)7	(GA)7	(GA)7	(GA)7	(GA)7	(GA)7	(GA)7	(GA)8	(GA)7	-	Introns/Intergenic	-	-	AGACTTTGTTCCAAGGAGCG	GCTCCTCCCCTCTCTCTCTC	234	A	A	A	NA
PMS2134	Os_Chr04	50485	50502	(GGC)6	(GGC)6	(GGC)6	(GGC)6	(GGC)6	(GGC)7	(GGC)6	(GGC)6	(GGC)6	(GGC)6	(GGC)6	-	Introns/Intergenic	-	-	GACGTGTTCGTTCTCGTTCA	GATCTCTCTCTCCGTCGCAC	254	P	A	A	NA
PMS2135	Os_Chr04	58335	58374	(AT)20	(AT)20	(AT)7	(AT)7	(AT)20	(AT)20	(AT)20	(AT)20	(AT)20	(AT)20	(AT)20	-	Introns/Intergenic	-	-	TCCTCTTGTTGTTGTCTGGTTG	GCGTGCATGTAGCCAGATTA	250	P	A	A	NA
PMS2136	Os_Chr04	72460	72498	(TGA)13	(TGA)11	(TGA)13	(TGA)13	(TGA)13	(TGA)11	(TGA)13	(TGA)13	(TGA)13	(TGA)13	(TGA)11	-	Introns/Intergenic	-	-	TTAGCTCGTGATGCTGATGC	TTATCATGAGGATTTGGCCC	180	P	A	A	NA
PMS2137	Os_Chr04	128086	128115	(AT)15	(AT)15	(AT)15	(AT)15	(AT)15	(AT)15	(AT)15	(AT)15	(AT)9	(AT)8	(AT)15	-	Introns/Intergenic	-	-	GCATGAACAACCACGATTCAACC	TAACACCTACTCCCTCCGGTTCC	510	A	A	A	RM16257
PMS2138	Os_Chr04	143877	143903	(ATG)9	(ATG)8	(ATG)9	(ATG)9	(ATG)9	(ATG)9	(ATG)9	(ATG)9	(ATG)9	(ATG)9	(ATG)9	-	Introns/Intergenic	-	-	CGCACAAAAAGCTGCTGATA	ACTTTTGCGGGTTTTAGGCT	272	P	A	A	NA
PMS2139	Os_Chr04	186309	186335	(GGC)9	(GGC)9	(GGC)9	(GGC)9	(GGC)9	(GGC)9	(GGC)9	(GGC)9	(GGC)9	(GGC)5	(GGC)9	LOC_Os04g01240	URR	serine-type peptidase, putative, expressed	-	CCCTAGCCCCTAAGAGGAGA	TGTTGTACCCCGAATCCAAT	132	A	A	A	NA
PMS2140	Os_Chr04	245676	245732	(AAT)19	(AAT)18	(AAT)19	(AAT)19	(AAT)6	(AAT)18	(AAT)19	(AAT)19	(AAT)18	(AAT)19	(AAT)18	-	Introns/Intergenic	-	-	CGAACTTGTAAAGCGTACATCG	GACATGTAGATGACGGCATGG	373	P	A	A	RM16266
PMS2141	Os_Chr04	250446	250461	(CT)8	(CT)8	(CT)8	(CT)8	(CT)8	(CT)8	(CT)8	(CT)8	(CT)8	(CT)8	(CT)6	LOC_Os04g01330	URR	expressed protein	-	GGAGCCAAATGACAGCCTAA	CGGGCGATTTTTCTAAGTGT	226	A	A	A	NA
PMS2142	Os_Chr04	258379	258410	(TA)16	(TA)16	(TA)16	(TA)7	(TA)16	(TA)8	(TA)16	(TA)16	(TA)16	(TA)16	(TA)16	-	Introns/Intergenic	-	-	GGTTTGAGAAATCGCGAAAA	ACTCACAGCACATGCCTACG	226	P	A	A	NA
PMS2143	Os_Chr04	299590	299607	(AT)9	(AT)9	(AT)9	(AT)9	(AT)9	(AT)9	(AT)9	(AT)9	(AT)9	(AT)9	(AT)6	-	Introns/Intergenic	-	-	AGATGCTTCAATCTCCCCCT	GGGGAGCTTTTGATTTCCAT	119	A	A	A	NA
PMS2144	Os_Chr04	359114	359128	(TAA)5	(TAA)7	(TAA)5	(TAA)5	(TAA)5	(TAA)7	(TAA)5	(TAA)5	(TAA)5	(TAA)5	(TAA)7	-	Introns/Intergenic	-	-	GGTCTGCTATCCGATTCCAA	AACGACACCCAAATCCTCAG	240	P	A	A	NA

Polymorphic SSR (PMS) markers	Chromosomes	Start physical positions (bp)	End physical positions (bp)	Nipponbare	Kasalath	Nagina22	IR64	Pokkali	Bala	Tainung67	Azucena	Moroberekan	*Oryza rufipogon*	*Oryza nivara*	MSU gene locus IDs	Structural annotation	Functional annotation	SSR functional effects	Forward primers (5'-3')	Reverse primers (5'-3')	Amplified product size (bp)	Markers exhibiting polymorphism within *indica* rice	Transcrption factor genes-derived markers	Known cloned genes-derived markers	PMS markers corresponding with RM markers
PMS2145	Os_Chr04	368583	368600	(GA)9	(GA)9	(GA)6	(GA)9	(GA)9	(GA)9	(GA)9	(GA)9	(GA)9	(GA)9	(GA)9	-	Introns/Intergenic	-	-	GTCAACGCTGGAGAGGTAGC	CCTCCCTTTCATCTTCCTCC	196	P	A	A	NA
PMS2146	Os_Chr04	373725	373740	(CA)8	(CA)8	(CA)8	(CA)7	(CA)8	(CA)8	(CA)8	(CA)7	(CA)7	(CA)8	(CA)8	-	Introns/Intergenic	-	-	TGTCATTCCATTAGCACCCA	TCACTGGCAACAACTCAAGC	241	P	A	A	NA
PMS2147	Os_Chr04	552723	552743	(GTC)7	(GTC)7	(GTC)7	(GTC)7	(GTC)6	(GTC)7	(GTC)7	(GTC)7	(GTC)7	(GTC)7	(GTC)7	LOC_Os04g01874	CDS	protein kinase, putative, expressed	CODON_DELETION	GCTCAAGATGGAATAACGATGG	GAGCTTTCATCGGATTCTCG	101	P	A	A	RM16285
PMS2148	Os_Chr04	679594	679611	(GCG)6	(GCG)6	(GCG)6	(GCG)6	(GCG)6	(GCG)6	(GCG)6	(GCG)6	(GCG)6	(GCG)5	(GCG)6	LOC_Os04g02100	CDS	expressed protein	CODON_CHANGE_PLUS_CODON_DELETION	CTCCCTCCTGGCTTCTTTCT	CTCCCCTCTACCCTCTCCAC	246	A	A	A	NA
PMS2149	Os_Chr04	842675	842686	(CG)6	(CG)6	(CG)6	(CG)6	(CG)6	(CG)6	(CG)6	(CG)6	(CG)7	(CG)6	(CG)6	-	Introns/Intergenic	-	-	CACCTGTCATCCCCTTCCT	ATAGCCTCAAGAGTCGACGG	260	A	A	A	NA
PMS2150	Os_Chr04	954057	954110	(TA)27	(TA)27	(TA)27	(TA)27	(TA)8	(TA)27	(TA)27	(TA)27	(TA)27	(TA)27	(TA)27	-	Introns/Intergenic	-	-	CAACGCCATTGAAACGACTAGG	GCAAGCACAGTTAGGCTCTTAATTCC	723	P	A	A	RM2416
PMS2151	Os_Chr04	984512	984527	(TA)8	(TA)8	(TA)8	(TA)8	(TA)8	(TA)6	(TA)8	(TA)8	(TA)8	(TA)8	(TA)8	-	Introns/Intergenic	-	-	TCTACCTAAAATGCGGGCAC	GAAGACGACGGTGAGGTAGG	268	P	A	A	NA
PMS2152	Os_Chr04	1190372	1190392	(TGC)7	(TGC)6	(TGC)7	(TGC)7	(TGC)7	(TGC)6	(TGC)7	(TGC)7	(TGC)7	(TGC)7	(TGC)7	-	Introns/Intergenic	-	-	TGTGAGAAAAAGTTAACGGCG	AGAACAGCGGCAAGATGACT	279	P	A	A	NA
PMS2153	Os_Chr04	1212765	1212782	(GGC)6	(GGC)6	(GGC)6	(GGC)6	(GGC)6	(GGC)6	(GGC)6	(GGC)6	(GGC)5	(GGC)6	(GGC)6	-	Introns/Intergenic	-	-	AGTGGAAGGAGGGAGGAGG	CACAGATCCACCACAGGAGA	208	A	A	A	NA
PMS2154	Os_Chr04	1276071	1276114	(AGAT)11	(AGAT)11	(AGAT)11	(AGAT)11	(AGAT)11	(AGAT)11	(AGAT)11	(AGAT)11	(AGAT)11	(AGAT)11	(AGAT)8	-	Introns/Intergenic	-	-	GCCATGTGGTGGCATGAGAGC	CCAACAGCCCACTTACTCCACTCTCC	147	A	A	A	RM16314
PMS2155	Os_Chr04	1310769	1310788	(TA)10	(TA)9	(TA)10	(TA)10	(TA)10	(TA)10	(TA)10	(TA)10	(TA)7	(TA)7	(TA)10	-	Introns/Intergenic	-	-	CACTGGTACAAACAGGCATCG	CTGGATACATCCGTTAGGAGAGC	291	P	A	A	RM16315
PMS2156	Os_Chr04	1312942	1313009	(TA)34	(TA)34	(TA)24	(TA)34	(TA)9	(TA)7	(TA)34	(TA)34	(TA)34	(TA)34	(TA)34	-	Introns/Intergenic	-	-	AACCTTGTAGAGCAGGACCTTGTTGG	TCGTGTGCTTCTTCCATTCTCG	287	P	A	A	RM16316
PMS2157	Os_Chr04	1344702	1344716	(CAA)5	(CAA)8	(CAA)5	(CAA)5	(CAA)5	(CAA)5	(CAA)5	(CAA)5	(CAA)5	(CAA)5	(CAA)5	LOC_Os04g03190	CDS	retrotransposon protein, putative, Ty3-gypsy subclass, expressed	CODON_INSERTION	GTCAGATGGTGAACACCCG	CCAGCCTCAACTGGATTGAT	247	P	A	A	NA

Polymorphic SSR (PMS) markers	Chromosomes	Start physical positions (bp)	End physical positions (bp)	Nipponbare	Kasalath	Nagina22	IR64	Pokkali	Bala	Tainung67	Azucena	Moroberekan	*Oryza rufipogon*	*Oryza nivara*	MSU gene locus IDs	Structural annotation	Functional annotation	SSR functional effects	Forward primers (5'-3')	Reverse primers (5'-3')	Amplified product size (bp)	Markers exhibiting polymorphism within *indica* rice	Transcrption factor genes-derived markers	Known cloned genes-derived markers	PMS markers corresponding with RM markers
PMS2158	Os_Chr04	1349873	1349887	(CGC)5	(CGC)5	(CGC)5	(CGC)5	(CGC)5	(CGC)5	(CGC)5	(CGC)5	(CGC)5	(CGC)6	(CGC)5	-	Introns/Intergenic	-	-	CATCCCCTAGCCCCTTTAGA	GACAACTTAGCGAACCCGAC	199	A	A	A	NA
PMS2159	Os_Chr04	1415998	1416045	(TA)24	(TA)24	(TA)24	(TA)12	(TA)24	(TA)12	(TA)24	(TA)24	(TA)24	(TA)24	(TA)24	-	Introns/Intergenic	-	-	CACGTAAGACACAGCGTTACTGC	GATATTGTTGGAGCCCATACTCC	296	P	A	A	RM16320
PMS2160	Os_Chr04	1564128	1564143	(AT)8	(AT)8	(AT)8	(AT)8	(AT)8	(AT)8	(AT)6	(AT)8	(AT)8	(AT)8	(AT)8	-	Introns/Intergenic	-	-	TGCTAACTTGAGACGATCGGA	ATGCTTCGTAACGGGTGAAG	151	A	A	A	NA
PMS2161	Os_Chr04	1609840	1609855	(AG)8	(AG)8	(AG)7	(AG)8	(AG)8	(AG)8	(AG)8	(AG)8	(AG)8	(AG)8	(AG)8	-	Introns/Intergenic	-	-	ATTGAATTCTGGTCCGTTGC	ATCAGTAGGGTTGACACCCG	169	P	A	A	NA
PMS2162	Os_Chr04	1652958	1652981	(CT)12	(CT)7	(CT)12	(CT)12	(CT)12	(CT)12	(CT)12	(CT)12	(CT)12	(CT)12	(CT)12	LOC_Os04g03740	CDS	expressed protein	FRAME_SHIFT	GGCTACGAGCCTCCTCTTTCTCC	ATCCCGCCTCCACTTTCTACACC	209	P	A	A	RM16332
PMS2163	Os_Chr04	1657038	1657061	(AT)12	(AT)12	(AT)12	(AT)12	(AT)12	(AT)12	(AT)8	(AT)12	(AT)12	(AT)12	(AT)12	-	Introns/Intergenic	-	-	TATGGTCCTCCCATTGTTGAACG	TGTATGGCCACCTGCTTATTTGC	261	A	A	A	RM16333
PMS2164	Os_Chr04	1683503	1683518	(TC)8	(TC)7	(TC)7	(TC)8	(TC)7	(TC)8	(TC)8	(TC)8	(TC)8	(TC)7	(TC)8	-	Introns/Intergenic	-	-	GAGTGGCCTTCTATGCTTCG	GCCGTTGCTTTACGAGAGAG	237	P	A	A	NA
PMS2165	Os_Chr04	1701704	1701757	(AT)27	(AT)13	(AT)6	(AT)27	(AT)27	(AT)27	(AT)27	(AT)27	(AT)27	(AT)14	(AT)7	-	Introns/Intergenic	-	-	GATTGGATCCTCAAACCTATGC	GTAGGTGCAGTGTGTGTAATGC	467	P	A	A	RM16334
PMS2166	Os_Chr04	1842793	1842812	(TA)10	(TA)10	(TA)10	(TA)10	(TA)10	(TA)10	(TA)10	(TA)10	(TA)10	(TA)10	(TA)7	-	Introns/Intergenic	-	-	TCGACTCATACACAGATGACGTACC	GCCCAGTTGTAAACAGGAAATAGC	283	A	A	A	RM16344
PMS2167	Os_Chr04	1879899	1879922	(GA)12	(GA)12	(GA)12	(GA)12	(GA)12	(GA)12	(GA)12	(GA)10	(GA)12	(GA)12	(GA)12	-	Introns/Intergenic	-	-	GGCCATGTGGTGAGAAGAAGAAGG	GAGGTGGCGGTGCACTACTACAGG	390	A	A	A	RM16345
PMS2168	Os_Chr04	1904992	1905023	(GA)16	(GA)21	(GA)16	(GA)16	(GA)16	(GA)16	(GA)16	(GA)16	(GA)16	(GA)16	(GA)16	-	Introns/Intergenic	-	-	TGAGCCACACTAGCAGGACTACG	TGGCCTTCTGACAATTCAACTCC	200	P	A	A	RM16349
PMS2169	Os_Chr04	1947935	1947952	(CCG)6	(CCG)6	(CCG)6	(CCG)6	(CCG)6	(CCG)6	(CCG)6	(CCG)6	(CCG)6	(CCG)6	(CCG)5	LOC_Os04g04180	CDS	transposon protein, putative, CACTA, En/Spm sub-class, expressed	CODON_DELETION	TTCTCGTGGCTCGTTTTCTT	CTCTTTCTCTCCGCGAGTGT	172	A	A	A	NA
PMS2170	Os_Chr04	1965173	1965190	(AG)9	(AG)8	(AG)9	(AG)9	(AG)9	(AG)9	(AG)9	(AG)9	(AG)9	(AG)9	(AG)9	-	Introns/Intergenic	-	-	TCTCATCAAGGTCACGAGGA	CCATATGTCAGCCTCGCTCT	217	P	A	A	NA

Polymorphic SSR (PMS) markers	Chromosomes	Start physical positions (bp)	End physical positions (bp)	Nipponbare	Kasalath	Nagina22	IR64	Pokkali	Bala	Tainung67	Azucena	Moroberekan	*Oryza rufipogon*	*Oryza nivara*	MSU gene locus IDs	Structural annotation	Functional annotation	SSR functional effects	Forward primers (5'-3')	Reverse primers (5'-3')	Amplified product size (bp)	Markers exhibiting polymorphism within *indica* rice	Transcrption factor genes-derived markers	Known cloned genes-derived markers	PMS markers corresponding with RM markers
PMS2171	Os_Chr04	1986406	1986419	(AT)7	(AT)6	(AT)7	(AT)6	(AT)6	(AT)6	(AT)7	(AT)6	(AT)6	(AT)6	(AT)7	-	Introns/Intergenic	-	-	ACCACCATGCCATAGACCAT	TTTCCATTGCACAATGTCGT	269	P	A	A	NA
PMS2172	Os_Chr04	2061694	2061717	(CT)12	(CT)10	(CT)13	(CT)12	(CT)12	(CT)12	(CT)12	(CT)12	(CT)12	(CT)12	(CT)12	-	Introns/Intergenic	-	-	CCACTGAACAAGTGTTGTAGTTCTGC	CTAGCTGACCACATGCTTCATCC	291	P	A	A	RM16355
PMS2173	Os_Chr04	2219237	2219254	(GA)9	(GA)9	(GA)9	(GA)9	(GA)9	(GA)9	(GA)9	(GA)9	(GA)9	(GA)9	(GA)7	-	Introns/Intergenic	-	-	GAGCAGCTACACGGAGATCG	TTTGGTTGTTTCTCCCTTGC	272	A	A	A	NA
PMS2174	Os_Chr04	2336764	2336784	(CCA)7	(CCA)7	(CCA)7	(CCA)7	(CCA)7	(CCA)7	(CCA)7	(CCA)5	(CCA)7	(CCA)7	(CCA)7	LOC_Os04g04850	CDS	retrotransposon protein, putative, Ty3-gypsy subclass	CODON_DELETION	GTGTGGGGTTTTCAGATGGT	TTAGAGTGTTGGGGAGGTGG	260	A	A	A	NA
PMS2175	Os_Chr04	2487583	2487600	(AG)9	(AG)8	(AG)8	(AG)7	(AG)7	(AG)9	(AG)9	(AG)7	(AG)8	(AG)9	(AG)7	-	Introns/Intergenic	-	-	TTTGCTTGGGCTTACAATCG	ATCTCTTCCACCCACCACAC	157	P	A	A	NA
PMS2176	Os_Chr04	2544062	2544100	(GGA)13	(GGA)13	(GGA)13	(GGA)13	(GGA)13	(GGA)13	(GGA)13	(GGA)13	(GGA)13	(GGA)13	(GGA)8	-	Introns/Intergenic	-	-	GCGAAGATAGGAGAGGGGAG	GGCGTAGTACTGTTGGACGG	183	A	A	A	NA
PMS2177	Os_Chr04	2569776	2569799	(GCA)8	(GCA)8	(GCA)8	(GCA)8	(GCA)8	(GCA)8	(GCA)8	(GCA)8	(GCA)8	(GCA)8	(GCA)6	LOC_Os04g05160	CDS	retrotransposon protein, putative, Ty3-gypsy subclass, expressed	CODON_CHANGE_PLUS_CODON_DELETION	TCCGCTACACCAATCCCTAC	TTGCTGATTCTGCTGCTGTT	131	A	A	A	NA
PMS2178	Os_Chr04	2578691	2578714	(GCA)8	(GCA)8	(GCA)8	(GCA)8	(GCA)8	(GCA)8	(GCA)8	(GCA)8	(GCA)8	(GCA)8	(GCA)6	LOC_Os04g05170	CDS	retrotransposon protein, putative, Ty3-gypsy subclass, expressed	CODON_CHANGE_PLUS_CODON_DELETION	TCCGCTACACCAATCCCTAC	TTGCTGATTCTGCTGCTGTT	131	A	A	A	NA
PMS2179	Os_Chr04	2825309	2825326	(GCC)6	(GCC)6	(GCC)6	(GCC)6	(GCC)6	(GCC)6	(GCC)6	(GCC)6	(GCC)5	(GCC)6	(GCC)6	-	Introns/Intergenic	-	-	ACCCACAGCTACCCCTTGT	CTCGTCTCGATCCCTTCAAC	213	A	A	A	NA
PMS2180	Os_Chr04	3013971	3014004	(TA)17	(TA)17	(TA)8	(TA)8	(TA)11	(TA)17	(TA)17	(TA)17	(TA)17	(TA)17	(TA)17	-	Introns/Intergenic	-	-	ATGGTGCCCCATATGAGTGT	GCTAGCGAGCAAATGAATTGT	235	P	A	A	NA
PMS2181	Os_Chr04	3133415	3133453	(ATA)13	(ATA)13	(ATA)13	(ATA)13	(ATA)13	(ATA)13	(ATA)13	(ATA)13	(ATA)13	(ATA)13	(ATA)10	-	Introns/Intergenic	-	-	CGGAGAAGATTAGCAGTGATGG	GCACGTACTACTCCATCTGTTTCG	577	A	A	A	RM16388
PMS2182	Os_Chr04	3228520	3228537	(TAA)6	(TAA)5	(TAA)6	(TAA)6	(TAA)6	(TAA)5	(TAA)6	(TAA)6	(TAA)6	(TAA)6	(TAA)6	-	Introns/Intergenic	-	-	AGACCGTGCAGCTGAAGAAT	AATGCCACACATTGGACTCA	258	P	A	A	NA
PMS2183	Os_Chr04	3418863	3418883	(AGA)7	(AGA)7	(AGA)7	(AGA)7	(AGA)7	(AGA)7	(AGA)7	(AGA)7	(AGA)6	(AGA)7	(AGA)7	LOC_Os04g06510	CDS	expressed protein	CODON_DELETION	ACCCAGTTGAAGACGTGGAG	GGACTTGGACGATTCTGCAT	257	A	A	A	NA

Polymorphic SSR (PMS) markers	Chromosomes	Start physical positions (bp)	End physical positions (bp)	Nipponbare	Kasalath	Nagina22	IR64	Pokkali	Bala	Tainung67	Azucena	Moroberekan	*Oryza rufipogon*	*Oryza nivara*	MSU gene locus IDs	Structural annotation	Functional annotation	SSR functional effects	Forward primers (5'-3')	Reverse primers (5'-3')	Amplified product size (bp)	Markers exhibiting polymorphism within *indica* rice	Transcrption factor genes-derived markers	Known cloned genes-derived markers	PMS markers corresponding with RM markers
PMS2184	Os_Chr04	3474134	3474159	(TA)13	(TA)13	(TA)7	(TA)13	(TA)13	(TA)13	(TA)7	(TA)13	(TA)13	(TA)13	(TA)13	-	Introns/Intergenic	-	-	TGTGTTTTATACCTTGGAGCCC	TGGATGCACAGACAACTTCG	236	P	A	A	NA
PMS2185	Os_Chr04	3491565	3491591	(ATT)9	(ATT)5	(ATT)9	(ATT)9	(ATT)9	(ATT)9	(ATT)9	(ATT)9	(ATT)9	(ATT)9	(ATT)9	-	Introns/Intergenic	-	-	GCATGTAACTTGAGAGTCATGG	ACGATCTTTAGTCCAGGTTGG	337	P	A	A	RM16398
PMS2186	Os_Chr04	3537942	3537953	(AT)6	(AT)8	(AT)6	(AT)6	(AT)6	(AT)6	(AT)6	(AT)6	(AT)6	(AT)6	(AT)8	-	Introns/Intergenic	-	-	AGGAGTGTGGGTAGTGTCGG	AGTGAGACCAACGTGAACCC	101	P	A	A	NA
PMS2187	Os_Chr04	3539617	3539637	(CCG)7	(CCG)7	(CCG)7	(CCG)7	(CCG)7	(CCG)7	(CCG)7	(CCG)6	(CCG)7	(CCG)7	(CCG)7	-	Introns/Intergenic	-	-	GCAAAGACTTCGCTCCTCTGTACTGC	CCTGTGACAGCGGTGGAGTTCG	460	A	A	A	RM16401
PMS2188	Os_Chr04	3739485	3739496	(AG)6	(AG)6	(AG)6	(AG)6	(AG)6	(AG)6	(AG)6	(AG)6	(AG)7	(AG)6	(AG)6	-	Introns/Intergenic	-	-	AACGTCGAATCCATAGAGCG	CGAGTTGCGTTGACTTCCTT	245	A	A	A	NA
PMS2189	Os_Chr04	3804547	3804564	(CGC)6	(CGC)6	(CGC)6	(CGC)6	(CGC)6	(CGC)6	(CGC)5	(CGC)6	(CGC)6	(CGC)6	(CGC)6	LOC_Os04g07170	CDS	transposon protein, putative, CACTA, En/Spm sub-class, expressed	CODON_CHANGE_PLUS_CODON_DELETION	TCCGATCTCGATCTCTCTC	GTTCTCGTTCTCGTTCGTTA	337	A	A	A	NA
PMS2190	Os_Chr04	3936265	3936285	(CGG)7	(CGG)7	(CGG)7	(CGG)7	(CGG)7	(CGG)7	(CGG)7	(CGG)7	(CGG)7	(CGG)5	(CGG)7	-	Introns/Intergenic	-	-	TTCCCCCTTATTGATTGCAC	ATCCCTCTTCCCTCTCTTGG	260	A	A	A	NA
PMS2191	Os_Chr04	3988902	3988917	(CG)8	(CG)8	(CG)8	(CG)8	(CG)8	(CG)8	(CG)8	(CG)7	(CG)8	(CG)8	(CG)8	-	Introns/Intergenic	-	-	AGATCCCGGAGATTGTTTCC	AAAGTCGGCCCAAAATCTCT	233	A	A	A	NA
PMS2192	Os_Chr04	4025695	4025726	(ATAG)8	(ATAG)6	(ATAG)8	(ATAG)8	(ATAG)8	(ATAG)8	(ATAG)8	(ATAG)8	(ATAG)6	(ATAG)8	(ATAG)8	-	Introns/Intergenic	-	-	CGTCTTACCGCAAGGAAGAG	CAAGTAATCGGCACAGAGCA	225	P	A	A	NA
PMS2193	Os_Chr04	4042564	4042595	(ATCT)8	(ATCT)11	(ATCT)11	(ATCT)11	(ATCT)11	(ATCT)8	(ATCT)8	(ATCT)11	(ATCT)13	(ATCT)11	(ATCT)11	-	Introns/Intergenic	-	-	GCTGCACTATGCAGTTGCTGAGG	TGATCGATGGTGACGATGATACGC	437	P	A	A	RM7200
PMS2194	Os_Chr04	4066937	4066957	(GCA)7	(GCA)7	(GCA)7	(GCA)7	(GCA)7	(GCA)7	(GCA)7	(GCA)7	(GCA)7	(GCA)7	(GCA)6	LOC_Os04g07650	CDS	retrotransposon protein, putative, Ty3-gypsy subclass, expressed	CODON_CHANGE_PLUS_CODON_DELETION	ACTTCCAACGACTGGTGGAC	GCACGAGTCCCTTGTTGATT	253	A	A	A	NA
PMS2195	Os_Chr04	4188756	4188767	(CA)6	(CA)6	(CA)6	(CA)6	(CA)6	(CA)7	(CA)6	(CA)6	(CA)6	(CA)6	(CA)6	-	Introns/Intergenic	-	-	ATTACACTCGTCCCCTCACG	TTGCATATGGAGGCCGTAGT	138	P	A	A	NA
PMS2196	Os_Chr04	4225620	4225633	(AC)7	(AC)7	(AC)7	(AC)7	(AC)7	(AC)7	(AC)7	(AC)7	(AC)8	(AC)7	(AC)7	-	Introns/Intergenic	-	-	ATCATTTTCTTCACCGGCAC	CCACTTGAATACCTTGCCGT	245	A	A	A	NA

Polymorphic SSR (PMS) markers	Chromosomes	Start physical positions (bp)	End physical positions (bp)	Nipponbare	Kasalath	Nagina22	IR64	Pokkali	Bala	Tainung67	Azucena	Moroberekan	*Oryza rufipogon*	*Oryza nivara*	MSU gene locus IDs	Structural annotation	Functional annotation	SSR functional effects	Forward primers (5'-3')	Reverse primers (5'-3')	Amplified product size (bp)	Markers exhibiting polymorphism within *indica* rice	Transcrption factor genes-derived markers	Known cloned genes-derived markers	PMS markers corresponding with RM markers
PMS2197	Os_Chr04	4244619	4244672	(TA)27	(TA)27	(TA)27	(TA)27	(TA)23	(TA)27	(TA)6	(TA)27	(TA)27	(TA)27	(TA)27	-	Introns/Intergenic	-	-	CTGTTTGCTGCATG GTTGAT	GCTAGCGAGCAAAT GAATTGT	253	P	A	A	NA
PMS2198	Os_Chr04	4358724	4358747	(AATT)6	(AATT)6	(AATT)6	(AATT)6	(AATT)6	(AATT)6	(AATT)6	(AATT)6	(AATT)6	(AATT)5	(AATT)6	-	Introns/Intergenic	-	-	GGGACAAACTGACA GAGGGA	GGAGGAGGAGGAAA GATGCT	202	A	A	A	NA
PMS2199	Os_Chr04	4463092	4463115	(TCC)8	(TCC)8	(TCC)8	(TCC)8	(TCC)8	(TCC)8	(TCC)8	(TCC)8	(TCC)8	(TCC)7	(TCC)8	LOC_Os04g08360	URR	expressed protein	-	CGAAAATAGCCCAC ACACCT	GCCACCGCACTTGTC TATG	260	A	A	A	NA
PMS2200	Os_Chr04	4697874	4697893	(TA)10	(TA)10	(TA)8	(TA)10	(TA)10	(TA)10	(TA)10	(TA)7	(TA)6	(TA)10	(TA)10	-	Introns/Intergenic	-	-	CGTGTATTCAGATG TGTCCTTTGG	CTGTAAGACGAGAG CAACAAAGTGG	389	P	A	A	RM16439
PMS2201	Os_Chr04	4764387	4764418	(AT)16	(AT)16	(AT)6	(AT)6	(AT)16	(AT)16	(AT)16	(AT)13	(AT)16	(AT)16	(AT)16	-	Introns/Intergenic	-	-	AGAGCACGCCTAGT CCTGATTCC	CGATAAGTGGAACAC GACACTTTCC	417	P	A	A	RM16444
PMS2202	Os_Chr04	4806895	4806906	(CT)6	(CT)6	(CT)6	(CT)6	(CT)6	(CT)6	(CT)6	(CT)6	(CT)8	(CT)7	(CT)6	-	Introns/Intergenic	-	-	GGAGAGAGGTGCG AAGAATC	CGAATGCATGTTTCG ACAAG	271	A	A	A	NA
PMS2203	Os_Chr04	4870932	4870943	(AT)6	(AT)6	(AT)6	(AT)6	(AT)6	(AT)7	(AT)6	(AT)6	(AT)6	(AT)6	(AT)6	-	Introns/Intergenic	-	-	CACTTGATGAGGTC AAGGCA	TCAAAATGAACGGAA CCACA	235	P	A	A	NA
PMS2204	Os_Chr04	4914524	4914541	(AT)9	(AT)9	(AT)9	(AT)9	(AT)9	(AT)9	(AT)9	(AT)9	(AT)6	(AT)9	(AT)9	-	Introns/Intergenic	-	-	CACAGCCACAACCA GCTCTA	TGGCATGTGAGGTTC AGAAA	204	A	A	A	NA
PMS2205	Os_Chr04	5078812	5078825	(AG)7	(AG)7	(AG)7	(AG)7	(AG)7	(AG)7	(AG)7	(AG)7	(AG)7	(AG)7	(AG)6	-	Introns/Intergenic	-	-	CTTTATAGGGGCTC GGGAGA	CCATTCTCTCTTTCC TCCCC	274	A	A	A	NA
PMS2206	Os_Chr04	5113582	5113599	(GGC)6	(GGC)6	(GGC)6	(GGC)6	(GGC)6	(GGC)6	(GGC)6	(GGC)6	(GGC)6	(GGC)5	(GGC)6	LOC_Os04g09530	URR	pentatricopeptide, putative, expressed	-	GGGGTAGGAGTAG GAGAGGG	CAGAAAGCCCACTAA TCCCA	254	A	A	A	NA
PMS2207	Os_Chr04	5145980	5146017	(TA)19	(TA)19	(TA)19	(TA)19	(TA)19	(TA)9	(TA)19	(TA)19	(TA)19	(TA)19	(TA)13	-	Introns/Intergenic	-	-	CAACATCCTGAGTC CAACACTGG	GGTATTTCTTGGAGC CCATACTCC	279	P	A	A	RM16453
PMS2208	Os_Chr04	5149558	5149579	(TA)11	(TA)11	(TA)11	(TA)11	(TA)11	(TA)11	(TA)11	(TA)6	(TA)11	(TA)11	(TA)11	-	Introns/Intergenic	-	-	ATGAATTTGAAGAT GCGACT	GTAAATTCAGTAAAG GCTAAAAG	274	A	A	A	NA
PMS2209	Os_Chr04	5151275	5151288	(CT)7	(CT)8	(CT)7	(CT)7	(CT)8	(CT)8	(CT)7	(CT)7	(CT)7	(CT)7	(CT)7	-	Introns/Intergenic	-	-	CCATTGAACAACCA CCTCCT	CGATCTAAAAGCAAA TGCTGAA	216	P	A	A	NA

Polymorphic SSR (PMS) markers	Chromosomes	Start physical positions (bp)	End physical positions (bp)	Nipponbare	Kasalath	Nagina22	IR64	Pokkali	Bala	Tainung67	Azucena	Moroberekan	*Oryza rufipogon*	*Oryza nivara*	MSU gene locus IDs	Structural annotation	Functional annotation	SSR functional effects	Forward primers (5'-3')	Reverse primers (5'-3')	Amplified product size (bp)	Markers exhibiting polymorphism within *indica* rice	Transcrption factor genes-derived markers	Known cloned genes-derived markers	PMS markers corresponding with RM markers
PMS2210	Os_Chr04	5189791	5189846	(TA)28	(TA)28	(TA)28	(TA)28	(TA)28	(TA)28	(TA)12	(TA)28	(TA)28	(TA)14	(TA)14	-	Introns/Intergenic	-	-	AGAGGAGCGATGGGAGAGAGG	CTTGCGGACAACCTACATCTTCG	332	A	A	A	RM16458
PMS2211	Os_Chr04	5393700	5393793	(AT)47	(AT)31	(AT)47	(AT)47	(AT)44	(AT)47	(AT)47	(AT)12	(AT)47	(AT)47	(AT)47	-	Introns/Intergenic	-	-	GCTCCAATTCCTTCTAGGGC	CGTGAATGAGAACGTAGCGA	268	P	A	A	NA
PMS2212	Os_Chr04	5453932	5453955	(TGC)8	(TGC)8	(TGC)8	(TGC)8	(TGC)8	(TGC)7	(TGC)8	(TGC)8	(TGC)8	(TGC)8	(TGC)7	LOC_Os04g10100	CDS	retrotransposon protein, putative, Ty3-gypsy subclass, expressed	CODON_DELETION	GCACGAGTCCCTTGTTGATT	GTCCGCTACACCAATCCCTA	143	P	A	A	NA
PMS2213	Os_Chr04	5533342	5533353	(TC)6	(TC)8	(TC)8	(TC)8	(TC)8	(TC)8	(TC)6	(TC)6	(TC)6	(TC)6	(TC)6	-	Introns/Intergenic	-	-	TTGGGGATGAATCAAATGCT	CAGCAAATATGTGGGTGTGC	267	A	A	A	NA
PMS2214	Os_Chr04	5560803	5560817	(CCG)5	(CCG)5	(CCG)5	(CCG)5	(CCG)5	(CCG)5	(CCG)5	(CCG)5	(CCG)5	(CCG)6	(CCG)5	-	Introns/Intergenic	-	-	ACCATGTCTGCCACCTACG	TCACCTAGAGGAATGCCCAC	280	A	A	A	NA
PMS2215	Os_Chr04	5576261	5576274	(TC)7	(TC)6	(TC)6	(TC)6	(TC)6	(TC)6	(TC)7	(TC)7	(TC)7	(TC)7	(TC)7	-	Introns/Intergenic	-	-	AAATGGCACTGTGTACGGGT	TCAAATTAATCCCTCCGCTG	203	A	A	A	NA
PMS2216	Os_Chr04	5600263	5600302	(TATG)10	(TATG)16	(TATG)16	(TATG)16	(TATG)16	(TATG)16	(TATG)10	(TATG)10	(TATG)10	(TATG)10	(TATG)16	-	Introns/Intergenic	-	-	CAGTGAAAGCTGCATAGGTTTGG	TCCTTCCATCTAGGTCCATTCTAAGG	452	A	A	A	RM16464
PMS2217	Os_Chr04	5685939	5685956	(TA)9	(TA)9	(TA)9	(TA)9	(TA)9	(TA)9	(TA)9	(TA)9	(TA)9	(TA)9	(TA)8	-	Introns/Intergenic	-	-	CGGATCTAACATATCATGCCC	AAAACCACTTATCCACCCCA	278	A	A	A	NA
PMS2218	Os_Chr04	5733818	5733843	(AT)13	(AT)13	(AT)13	(AT)13	(AT)13	(AT)8	(AT)6	(AT)13	(AT)13	(AT)13	(AT)6	-	Introns/Intergenic	-	-	GCACATGCGTAAGCGAAATA	TGTGCAAGAACTAAAACCACG	255	P	A	A	NA
PMS2219	Os_Chr04	6008314	6008329	(AG)8	(AG)8	(AG)8	(AG)8	(AG)8	(AG)6	(AG)8	(AG)8	(AG)6	(AG)8	(AG)8	-	Introns/Intergenic	-	-	CTTTCTGGTGGAGCCAACTC	AAGAGAGAAACCGCGTGAAA	130	P	A	A	NA
PMS2220	Os_Chr04	6055174	6055205	(TA)16	(TA)16	(TA)16	(TA)8	(TA)16	(TA)16	(TA)16	(TA)16	(TA)16	(TA)16	(TA)16	-	Introns/Intergenic	-	-	GAAGAACTTGCGTGATGTCTCC	TAAAGTGCAGCAAGGAAGATGC	723	P	A	A	RM16481
PMS2221	Os_Chr04	6209397	6209414	(TA)9	(TA)9	(TA)9	(TA)7	(TA)8	(TA)9	(TA)9	(TA)9	(TA)8	(TA)9	(TA)9	-	Introns/Intergenic	-	-	TGTGCACTTACCATTGTTTGAA	CCGCGAAGATGTAAACCCTA	247	P	A	A	NA
PMS2222	Os_Chr04	6222749	6222760	(AT)6	(AT)7	(AT)6	(AT)6	(AT)6	(AT)7	(AT)6	(AT)6	(AT)6	(AT)6	(AT)6	-	Introns/Intergenic	-	-	TTGGACCGGGATGATACATT	GCATTACCCGAAAATTGTTCTC	233	P	A	A	NA

Polymorphic SSR (PMS) markers	Chromosomes	Start physical positions (bp)	End physical positions (bp)	Nipponbare	Kasalath	Nagina22	IR64	Pokkali	Bala	Tainung67	Azucena	Moroberekan	*Oryza rufipogon*	*Oryza nivara*	MSU gene locus IDs	Structural annotation	Functional annotation	SSR functional effects	Forward primers (5'-3')	Reverse primers (5'-3')	Amplified product size (bp)	Markers exhibiting polymorphism within *indica* rice	Transcrption factor genes-derived markers	Known cloned genes-derived markers	PMS markers corresponding with RM markers
PMS2223	Os_Chr04	6244212	6244223	(AG)6	(AG)8	(AG)6	(AG)6	(AG)6	(AG)6	(AG)6	(AG)6	(AG)6	(AG)6	(AG)6	-	Introns/Intergenic	-	-	AACAATCGTTGCTG CATCTG	TGCCCATCAAAGATG ACAAA	262	P	A	A	NA
PMS2224	Os_Chr04	6314644	6314685	(GA)21	(GA)21	(GA)21	(GA)21	(GA)21	(GA)21	(GA)21	(GA)21	(GA)21	(GA)13	(GA)21	-	Introns/Intergenic	-	-	AGAAACAGAGGGA GGGAGCAGAGG	GATCCCGACAGATG GTGACTTGC	149	A	A	A	RM3471
PMS2225	Os_Chr04	6344365	6344382	(TA)9	(TA)9	(TA)9	(TA)7	(TA)9	(TA)9	(TA)9	(TA)9	(TA)9	(TA)7	(TA)9	-	Introns/Intergenic	-	-	TCGTAACCCCCTTT AGTCCC	TCAATTTCAACAATG GTGCC	262	P	A	A	NA
PMS2226	Os_Chr04	6431865	6431898	(AT)17	(AT)17	(AT)8	(AT)8	(AT)8	(AT)8	(AT)17	(AT)17	(AT)17	(AT)9	(AT)17	-	Introns/Intergenic	-	-	GCCCGGCCGTTTCT ATAACACC	GTGTGTGCTCACGTG TATGGTAGTCC	684	P	A	A	RM16494
PMS2227	Os_Chr04	6495201	6495218	(CGG)6	(CGG)7	(CGG)6	(CGG)6	(CGG)6	(CGG)7	(CGG)6	(CGG)6	(CGG)6	(CGG)6	(CGG)6	LOC_Os04g11830	CDS	PCF1	CODON_INSERTION	GAGGAGCGCGGTG TAGTAAG	GAGTGGCTACTCCG CCAG	177	P	P	P	NA
PMS2228	Os_Chr04	6495619	6495636	(TGG)6	(TGG)6	(TGG)6	(TGG)6	(TGG)6	(TGG)6	(TGG)6	(TGG)6	(TGG)6	(TGG)5	(TGG)5	LOC_Os04g11830	URR	PCF1	-	TAGAGGATGAGGTC GGAGGA	AGACAGTCCAACCG AACCAG	199	A	P	P	NA
PMS2229	Os_Chr04	6579013	6579028	(CT)8	(CT)7	(CT)7	(CT)8	(CT)8	(CT)8	(CT)8	(CT)8	(CT)8	(CT)8	(CT)8	-	Introns/Intergenic	-	-	ATCGCATCGCATCC TCTACT	ATGGTGTACCATCGC CAAAT	142	P	A	A	NA
PMS2230	Os_Chr04	6932701	6932712	(AT)6	(AT)6	(AT)6	(AT)6	(AT)6	(AT)6	(AT)6	(AT)7	(AT)7	(AT)6	(AT)6	-	Introns/Intergenic	-	-	AGTGCTCAAAGGGG TAGGGT	CTTGTTGACAGAAGG TGCCA	210	A	A	A	NA
PMS2231	Os_Chr04	6936896	6936921	(AT)13	(AT)13	(AT)13	(AT)13	(AT)6	(AT)13	(AT)13	(AT)13	(AT)13	(AT)13	(AT)13	-	Introns/Intergenic	-	-	TTCACATCCATTCA GCGACATGC	GGGTGCTACATTCAG CATCAATCC	306	P	A	A	RM16507
PMS2232	Os_Chr04	7029512	7029541	(TA)15	(TA)15	(TA)7	(TA)15	(TA)15	(TA)15	(TA)8	(TA)15	(TA)15	(TA)15	(TA)15	-	Introns/Intergenic	-	-	GAGACGATCGGACT CTTAACTGC	GATAGATGGAGTTTC GGAGAAGG	241	P	A	A	RM16510
PMS2233	Os_Chr04	7104997	7105011	(AAG)5	(AAG)5	(AAG)5	(AAG)5	(AAG)5	(AAG)6	(AAG)5	(AAG)5	(AAG)5	(AAG)5	(AAG)5	-	Introns/Intergenic	-	-	CCGTAACTTCCCAT CAAGGA	TTGCCAATTTCTATT GGTTGC	177	P	A	A	NA
PMS2234	Os_Chr04	7251799	7251842	(TATC)11	(TATC)9	(TATC)9	(TATC)1 1	(TATC)11	(TATC)1 1	(TATC)11	(TATC)11	(TATC)11	(TATC)9	(TATC)1 1	LOC_Os04g13140	DRR	vignain precursor, putative, expressed	-	GGCTACGTCAGGAT GGAGAGG	GGATGTTACATGTCA GCTTGAGAGC	371	P	A	A	RM16514
PMS2235	Os_Chr04	7299859	7299872	(GA)7	(GA)7	(GA)7	(GA)6	(GA)6	(GA)7	(GA)7	(GA)7	(GA)7	(GA)7	(GA)7	-	Introns/Intergenic	-	-	GGAGACCACTCCTC GCTTC	GGGTCCCACATGTAC CTCTC	266	P	A	A	NA

Polymorphic SSR (PMS) markers	Chromosomes	Start physical positions (bp)	End physical positions (bp)	Nipponbare	Kasalath	Nagina22	IR64	Pokkali	Bala	Tainung67	Azucena	Moroberekan	*Oryza rufipogon*	*Oryza nivara*	MSU gene locus IDs	Structural annotation	Functional annotation	SSR functional effects	Forward primers (5'-3')	Reverse primers (5'-3')	Amplified product size (bp)	Markers exhibiting polymorphism within *indica* rice	Transcrption factor genes-derived markers	Known cloned genes-derived markers	PMS markers corresponding with RM markers
PMS2236	Os_Chr04	7423796	7423807	(CT)6	(CT)6	(CT)6	(CT)9	(CT)6	(CT)9	(CT)6	(CT)6	(CT)9	(CT)6	(CT)6	-	Introns/Intergenic	-	-	GCCGTCATCGAGTCTTTCTC	GAGTGGATAGGGAAGAGGGG	270	P	A	A	NA
PMS2237	Os_Chr04	7504051	7504071	(CCT)7	(CCT)7	(CCT)7	(CCT)7	(CCT)7	(CCT)7	(CCT)7	(CCT)7	(CCT)7	(CCT)5	(CCT)7	LOC_Os04g13470	URR	expressed protein	-	ACCTGACCGCCAGATCTGACACC	GTTGAGGAGCGCGGTGATGG	255	A	A	A	RM16519
PMS2238	Os_Chr04	7581413	7581428	(CT)8	(CT)7	(CT)7	(CT)8	(CT)8	(CT)8	(CT)8	(CT)8	(CT)8	(CT)8	(CT)8	-	Introns/Intergenic	-	-	CCCAAAGACTCACTTGCACA	CATTGCGTGCTGTTCATCTT	109	P	A	A	NA
PMS2239	Os_Chr04	7731831	7731844	(CT)7	(CT)7	(CT)7	(CT)7	(CT)7	(CT)7	(CT)7	(CT)7	(CT)7	(CT)7	(CT)6	-	Introns/Intergenic	-	-	GTCTTCTTTTGGCTTTGGGC	ACGAAGACTGACAAGTGGGC	140	A	A	A	NA
PMS2240	Os_Chr04	7745795	7745821	(GAT)9	(GAT)9	(GAT)9	(GAT)9	(GAT)9	(GAT)9	(GAT)9	(GAT)9	(GAT)6	(GAT)9	(GAT)9	LOC_Os04g13880	CDS	retrotransposon protein, putative, unclassified, expressed	CODON_DELETION	AACGGTTCTCTACTGATGACG	TGAACTCCAATCCTCTTTCC	99	A	A	A	RM16524
PMS2241	Os_Chr04	7888252	7888305	(TA)27	(TA)27	(TA)8	(TA)27	(TA)27	(TA)27	(TA)27	(TA)27	(TA)27	(TA)8	(TA)27	-	Introns/Intergenic	-	-	TGTCGTGACAAAATTACTTCCG	TTCAATCTTCACAACAAGATCTTACA	279	P	A	A	NA
PMS2242	Os_Chr04	7946667	7946704	(AT)19	(AT)19	(AT)19	(AT)19	(AT)19	(AT)18	(AT)19	(AT)19	(AT)19	(AT)18	(AT)11	-	Introns/Intergenic	-	-	TGTCCTTTCTAATGCAGCGA	CGATCATATGTGTGCGATCC	160	P	A	A	NA
PMS2243	Os_Chr04	7954551	7954598	(TA)24	(TA)24	(TA)7	(TA)24	(TA)24	(TA)24	(TA)24	(TA)24	(TA)24	(TA)7	(TA)24	-	Introns/Intergenic	-	-	CGTGACAAAATTACTTCCGTTTT	CGTTACAATTAAATCTTCACAACAAGA	280	P	A	A	NA
PMS2244	Os_Chr04	7968860	7968875	(AG)8	(AG)8	(AG)8	(AG)8	(AG)8	(AG)8	(AG)8	(AG)8	(AG)8	(AG)8	(AG)6	-	Introns/Intergenic	-	-	AATGCGGAAGAGAGGGAGAT	TCACACATCTAACGGTGCAAA	192	A	A	A	NA
PMS2245	Os_Chr04	7976204	7976259	(AT)28	(AT)21	(AT)28	(AT)28	(AT)28	(AT)28	(AT)28	(AT)28	(AT)28	(AT)28	(AT)28	-	Introns/Intergenic	-	-	CAGTGCAGGAACAAGATTCAGG	CATTGCAGTTGGGTTCTATTGG	369	P	A	A	RM16531
PMS2246	Os_Chr04	8038779	8038799	(AGG)7	(AGG)7	(AGG)7	(AGG)7	(AGG)7	(AGG)5	(AGG)7	(AGG)7	(AGG)7	(AGG)7	(AGG)7	-	Introns/Intergenic	-	-	GGAGCGAGGACGAGGAGTATGG	TGCCCGAGAATTGTGTAACCTTCG	215	P	A	A	RM16534
PMS2247	Os_Chr04	8071990	8072010	(CTC)7	(CTC)7	(CTC)7	(CTC)7	(CTC)7	(CTC)7	(CTC)5	(CTC)5	(CTC)7	(CTC)7	(CTC)7	LOC_Os04g14410	CDS	expressed protein	CODON_DELETION	ACGCGGTAGTCCTCTTCAATGTCG	GGCGCCAACCCTTCCTACTACC	177	A	A	A	RM16535
PMS2248	Os_Chr04	8111649	8111666	(TTG)6	(TTG)6	(TTG)6	(TTG)6	(TTG)6	(TTG)6	(TTG)6	(TTG)6	(TTG)6	(TTG)6	(TTG)5	LOC_Os04g14500	CDS	retrotransposon, putative, centromere-specific, expressed	CODON_DELETION	TGGTCCGTACTTGATCCTCC	ACATGTGAGGTCTGTGGCAA	170	A	A	A	NA

Polymorphic SSR (PMS) markers	Chromosomes	Start physical positions (bp)	End physical positions (bp)	Nipponbare	Kasalath	Nagina22	IR64	Pokkali	Bala	Tainung67	Azucena	Moroberekan	*Oryza rufipogon*	*Oryza nivara*	MSU gene locus IDs	Structural annotation	Functional annotation	SSR functional effects	Forward primers (5'-3')	Reverse primers (5'-3')	Amplified product size (bp)	Markers exhibiting polymorphism within *indica* rice	Transcrption factor genes-derived markers	Known cloned genes-derived markers	PMS markers corresponding with RM markers
PMS2249	Os_Chr04	8156931	8156946	(AT)8	(AT)8	(AT)8	(AT)8	(AT)8	(AT)8	(AT)8	(AT)8	(AT)8	(AT)6	(AT)8	-	Introns/Intergenic	-	-	#REF!	#REF!	239	A	A	A	NA
PMS2250	Os_Chr04	8276161	8276181	(CCT)7	(CCT)7	(CCT)7	(CCT)7	(CCT)7	(CCT)7	(CCT)7	(CCT)7	(CCT)7	(CCT)7	(CCT)5	-	Introns/Intergenic	-	-	TTGGGAAATTAGTTCAGCGG	ATTCTCCCTCGAGGTGCC	274	A	A	A	NA
PMS2251	Os_Chr04	8421576	8421589	(GT)7	(GT)6	(GT)7	(GT)7	(GT)7	(GT)6	(GT)7	(GT)7	(GT)7	(GT)7	(GT)7	-	Introns/Intergenic	-	-	GGATCATGTGCAAGTGGTGT	GAGGAAGAGTTTGCCTCCAA	187	P	A	A	NA
PMS2252	Os_Chr04	8588610	8588623	(TA)7	(TA)8	(TA)7	(TA)7	(TA)7	(TA)7	(TA)7	(TA)7	(TA)7	(TA)7	(TA)7	-	Introns/Intergenic	-	-	GCGTTGATGGCTACAGACAG	TCTTAGGGATGACAGGTGGG	257	P	A	A	NA
PMS2253	Os_Chr04	8605261	8605308	(AT)24	(AT)24	(AT)24	(AT)24	(AT)13	(AT)24	(AT)24	(AT)7	(AT)24	(AT)24	(AT)7	-	Introns/Intergenic	-	-	#REF!	#REF!	557	P	A	A	NA
PMS2254	Os_Chr04	8869719	8869744	(TA)13	(TA)13	(TA)9	(TA)12	(TA)13	(TA)13	(TA)7	(TA)13	(TA)13	(TA)11	(TA)13	-	Introns/Intergenic	-	-	TTGGACCAGGAGATCAATGAAGG	GTGCGCACACTCTTCTATGTGC	391	P	A	A	RM16556
PMS2255	Os_Chr04	8907463	8907480	(CCT)6	(CCT)5	(CCT)5	(CCT)5	(CCT)5	(CCT)5	(CCT)6	(CCT)5	(CCT)5	(CCT)5	(CCT)5	LOC_Os04g16390	CDS	retrotransposon protein, putative, Ty3-gypsy subclass, expressed	CODON_DELETION	TGAGGAAGAAATCAGCAGCA	CCGATGGAGTTTGGTCACTT	172	A	A	A	NA
PMS2256	Os_Chr04	9278892	9278909	(AG)9	(AG)9	(AG)9	(AG)9	(AG)9	(AG)9	(AG)9	(AG)9	(AG)6	(AG)9	(AG)9	-	Introns/Intergenic	-	-	CATGGACCCACTTGTCATCC	TGGAGAGAGGGCAAGGTTTA	250	A	A	A	NA
PMS2257	Os_Chr04	9557657	9557672	(CT)8	(CT)7	(CT)8	(CT)8	(CT)8	(CT)8	(CT)8	(CT)8	(CT)8	(CT)8	(CT)8	-	Introns/Intergenic	-	-	GTCCCCACGTGCAATAAAGT	TAAATAGGGTTGGAAGGGGG	204	P	A	A	NA
PMS2258	Os_Chr04	9567569	9567616	(TA)24	(TA)24	(TA)24	(TA)11	(TA)24	(TA)24	(TA)24	(TA)24	(TA)24	(TA)24	(TA)22	-	Introns/Intergenic	-	-	GTTTTATACCTTGGAGCCCG	GCACCATGGAGCATAAGACA	127	P	A	A	NA
PMS2259	Os_Chr04	9569504	9569555	(TA)26	(TA)26	(TA)26	(TA)26	(TA)26	(TA)10	(TA)26	(TA)7	(TA)7	(TA)26	(TA)26	-	Introns/Intergenic	-	-	TCAAAGATACCCACATATTAGTTGAAA	GATTTAAATGGTAAATTAAATGGGTG	235	P	A	A	NA
PMS2260	Os_Chr04	9675721	9675744	(AT)12	(AT)11	(AT)12	(AT)12	(AT)12	(AT)12	(AT)7	(AT)12	(AT)12	(AT)12	(AT)12	-	Introns/Intergenic	-	-	TCCACAAGACAAAGCACGAG	ACCGCGAAAACATCTTCAAT	214	P	A	A	NA
PMS2261	Os_Chr04	9683752	9683779	(TA)14	(TA)14	(TA)14	(TA)14	(TA)6	(TA)14	(TA)14	(TA)7	(TA)6	(TA)14	(TA)10	-	Introns/Intergenic	-	-	CAACGCACGGGTATCACTG	GGATGGTTTGCGAGATCCTA	244	P	A	A	NA

Polymorphic SSR (PMS) markers	Chromosomes	Start physical positions (bp)	End physical positions (bp)	Nipponbare	Kasalath	Nagina22	IR64	Pokkali	Bala	Tainung67	Azucena	Moroberekan	*Oryza rufipogon*	*Oryza nivara*	MSU gene locus IDs	Structural annotation	Functional annotation	SSR functional effects	Forward primers (5'-3')	Reverse primers (5'-3')	Amplified product size (bp)	Markers exhibiting polymorphism within *indica* rice	Transcrption factor genes-derived markers	Known cloned genes-derived markers	PMS markers corresponding with RM markers
PMS2262	Os_Chr04	9997073	9997110	(AT)19	(AT)19	(AT)19	(AT)6	(AT)16	(AT)19	(AT)19	(AT)19	(AT)19	(AT)19	(AT)19	-	Introns/Intergenic	-	-	TCTGTCCACTTCATGGGAGTACG	CGAACCTTCCACATACAGAGAGC	488	P	A	A	RM16574
PMS2263	Os_Chr04	10030740	10030769	(CT)15	(CT)14	(CT)15	(CT)15	(CT)15	(CT)15	(CT)15	(CT)15	(CT)16	(CT)15	(CT)15	-	Introns/Intergenic	-	-	CACCAACTACACTCCTACACTCC	CTAGATCATAGGCGGTCACG	254	P	A	A	RM16575
PMS2264	Os_Chr04	10212003	10212020	(GAT)6	(GAT)6	(GAT)5	(GAT)6	(GAT)6	(GAT)6	(GAT)6	(GAT)6	(GAT)6	(GAT)6	(GAT)6	-	Introns/Intergenic	-	-	GAACAGAGGTTTGCAGAGGC	TGGTTGTCCTTCCTTATCCG	263	P	A	A	NA
PMS2265	Os_Chr04	10714282	10714299	(GCG)6	(GCG)6	(GCG)6	(GCG)6	(GCG)6	(GCG)6	(GCG)5	(GCG)6	(GCG)6	(GCG)5	(GCG)6	-	Introns/Intergenic	-	-	AGAGAGAGGGAAAAATGGGG	CTCTCTCTCCTCTTCCCCG	263	A	A	A	NA
PMS2266	Os_Chr04	10811501	10811515	(GTC)5	(GTC)5	(GTC)5	(GTC)5	(GTC)5	(GTC)6	(GTC)5	(GTC)5	(GTC)5	(GTC)5	(GTC)5	-	Introns/Intergenic	-	-	CTGCTAGCTGTCCCTCGTTG	ATCCTTGGATGAAGGCGAG	218	P	A	A	NA
PMS2267	Os_Chr04	10833989	10834002	(AT)7	(AT)7	(AT)7	(AT)7	(AT)7	(AT)7	(AT)7	(AT)8	(AT)8	(AT)7	(AT)7	-	Introns/Intergenic	-	-	GCCAGGTAGGCCAACAATTA	TTATTGCATTTTGTGGGGTG	248	A	A	A	NA
PMS2268	Os_Chr04	10884122	10884151	(TA)15	(TA)15	(TA)11	(TA)15	(TA)15	(TA)10	(TA)8	(TA)15	(TA)15	(TA)15	(TA)15	-	Introns/Intergenic	-	-	AAACATACGATCGGCGAAAC	CCACCATTTCCATGTTTTCC	121	P	A	A	NA
PMS2269	Os_Chr04	11037998	11038037	(TA)20	(TA)20	(TA)20	(TA)20	(TA)20	(TA)20	(TA)20	(TA)20	(TA)11	(TA)20	(TA)20	-	Introns/Intergenic	-	-	ATGCATGAAGCGAGCACAACC	TGGTGAACTTTGTTTCCCTCACG	207	A	A	A	RM16584
PMS2270	Os_Chr04	11120413	11120428	(TC)8	(TC)7	(TC)7	(TC)7	(TC)8	(TC)7	(TC)8	(TC)7	(TC)7	(TC)7	(TC)7	-	Introns/Intergenic	-	-	TTCCATTGTTGTCCCCTCTC	CCAAACTTTTGACCGTCGAT	248	P	A	A	NA
PMS2271	Os_Chr04	11278563	11278586	(CGC)8	(CGC)8	(CGC)8	(CGC)8	(CGC)8	(CGC)8	(CGC)8	(CGC)8	(CGC)8	(CGC)7	(CGC)8	-	Introns/Intergenic	-	-	TTATCTTAATCCCGTGGGAAGC	TAACCCTAACCCTCGATTCTCC	340	A	A	A	RM16591
PMS2272	Os_Chr04	11295489	11295506	(AT)9	(AT)9	(AT)9	(AT)9	(AT)6	(AT)9	(AT)9	(AT)9	(AT)9	(AT)9	(AT)9	-	Introns/Intergenic	-	-	AAAATAATCGACTTAGAGCATTATGAA	TCGATCTATCATCCGATTACACC	216	P	A	A	NA
PMS2273	Os_Chr04	11344197	11344212	(TA)8	(TA)8	(TA)6	(TA)8	(TA)8	(TA)8	(TA)8	(TA)8	(TA)8	(TA)8	(TA)8	-	Introns/Intergenic	-	-	CTCCGCAAACCCTTTGTAGT	ACGGCCTCATCTTTCTCTCA	274	P	A	A	NA
PMS2274	Os_Chr04	11408651	11408716	(AT)33	(AT)33	(AT)33	(AT)33	(AT)33	(AT)33	(AT)33	(AT)33	(AT)33	(AT)22	(AT)12	LOC_Os04g20400	DRR	cytokinin-O-glucosyltransferase 1, putative, expressed	-	AGGTTATTTCAGGCACCACG	GCTGACGCCGAGAGAAATAA	271	A	A	A	NA

Polymorphic SSR (PMS) markers	Chromosomes	Start physical positions (bp)	End physical positions (bp)	Nipponbare	Kasalath	Nagina22	IR64	Pokkali	Bala	Tainung67	Azucena	Moroberekan	*Oryza rufipogon*	*Oryza nivara*	MSU gene locus IDs	Structural annotation	Functional annotation	SSR functional effects	Forward primers (5'-3')	Reverse primers (5'-3')	Amplified product size (bp)	Markers exhibiting polymorphism within *indica* rice	Transcrption factor genes-derived markers	Known cloned genes-derived markers	PMS markers corresponding with RM markers
PMS2275	Os_Chr04	11483186	11483215	(AT)15	(AT)15	(AT)7	(AT)9	(AT)15	(AT)9	(AT)15	(AT)15	(AT)15	(AT)15	(AT)8	-	Introns/Intergenic	-	-	CATGGTGGGACTCACATATACC	AAGAACTGTGCTGCAGATAACC	135	P	A	A	RM16605
PMS2276	Os_Chr04	11498898	11498915	(CGA)6	(CGA)6	(CGA)6	(CGA)6	(CGA)6	(CGA)6	(CGA)6	(CGA)6	(CGA)6	(CGA)5	(CGA)6	-	Introns/Intergenic	-	-	AGTACGACCTTCAAGGGCCA	GGAGGTCGCTGGAGGACT	263	A	A	A	NA
PMS2277	Os_Chr04	11651576	11651591	(TA)8	(TA)8	(TA)6	(TA)6	(TA)8	(TA)8	(TA)8	(TA)8	(TA)8	(TA)8	(TA)11	-	Introns/Intergenic	-	-	GCTTCCCGTGTGGTACAAGT	TTTGATGTTGGGAAGGAGGT	258	P	A	A	NA
PMS2278	Os_Chr04	11652193	11652206	(CT)7	(CT)7	(CT)7	(CT)12	(CT)7	(CT)12	(CT)7	(CT)7	(CT)7	(CT)7	(CT)7	-	Introns/Intergenic	-	-	TTATTCTTCCTCCACCGCAC	GCACAAGGATGACGGGTACT	234	P	A	A	NA
PMS2279	Os_Chr04	11666863	11666900	(AT)19	(AT)19	(AT)6	(AT)19	(AT)19	(AT)10	(AT)19	(AT)9	(AT)8	(AT)19	(AT)11	-	Introns/Intergenic	-	-	TTAAATCGCGGCTAATCTTAT	GGCAAGTGATGTAAAGAAGTG	405	P	A	A	NA
PMS2280	Os_Chr04	11791632	11791647	(AG)8	(AG)8	(AG)8	(AG)8	(AG)8	(AG)7	(AG)8	(AG)8	(AG)8	(AG)8	(AG)6	-	Introns/Intergenic	-	-	TCCAGATCGGGTGATTTTTC	TAACTGGGCAACGAGGAACT	264	P	A	A	NA
PMS2281	Os_Chr04	11811106	11811123	(TGC)6	(TGC)5	(TGC)5	(TGC)6	(TGC)6	(TGC)5	(TGC)6	(TGC)6	(TGC)6	(TGC)6	(TGC)6	-	Introns/Intergenic	-	-	GAAGGGAAAGGGGAAGGAG	ACCTCCCTCGGGTAGGTATG	243	P	A	A	NA
PMS2282	Os_Chr04	11889263	11889280	(AG)9	(AG)10	(AG)9	(AG)9	(AG)9	(AG)10	(AG)9	(AG)9	(AG)9	(AG)9	(AG)9	-	Introns/Intergenic	-	-	TTTACCCCAACTAACGGCAG	ACCTGGACGGAGGGAGTAAT	162	P	A	A	NA
PMS2283	Os_Chr04	11896396	11896439	(ATAG)11	(ATAG)11	(ATAG)11	(ATAG)11	(ATAG)6	(ATAG)11	(ATAG)11	(ATAG)9	(ATAG)11	(ATAG)11	(ATAG)7	-	Introns/Intergenic	-	-	CGGAATTTGGAAACTTTTCTTG	TGACTTAGAAACAAAAGACAAACGA	212	P	A	A	NA
PMS2284	Os_Chr04	11947850	11947870	(GCA)7	(GCA)7	(GCA)7	(GCA)6	(GCA)7	(GCA)7	(GCA)7	(GCA)7	(GCA)7	(GCA)7	(GCA)7	LOC_Os04g21200	CDS	retrotransposon protein, putative, Ty3-gypsy subclass, expressed	CODON_CHANGE_PLUS_CODON_DELETION	GTCCGCTACACCAATCCCTA	CGAAGCAGTTGAAGCACAGA	226	P	A	A	NA
PMS2285	Os_Chr04	12028572	12028607	(AT)18	(AT)18	(AT)18	(AT)18	(AT)18	(AT)18	(AT)18	(AT)7	(AT)18	(AT)18	(AT)18	-	Introns/Intergenic	-	-	ATTGTCCAAGCGAGCCTTTA	GACGTGGCATTGAAAGATGA	158	A	A	A	NA
PMS2286	Os_Chr04	12138569	12138582	(CT)7	(CT)7	(CT)7	(CT)7	(CT)7	(CT)7	(CT)7	(CT)7	(CT)7	(CT)7	(CT)8	-	Introns/Intergenic	-	-	TCCGGGTGTCTTAACAGGTC	AGAGGAGAGGGAGAGATGCC	141	A	A	A	NA
PMS2287	Os_Chr04	12142344	12142357	(CT)7	(CT)7	(CT)7	(CT)7	(CT)7	(CT)8	(CT)7	(CT)7	(CT)7	(CT)7	(CT)8	-	Introns/Intergenic	-	-	CGTTGGATCCTCTCCATCAT	CTCTTGGGGTCAGAGTGGTG	222	P	A	A	NA

Polymorphic SSR (PMS) markers	Chromosomes	Start physical positions (bp)	End physical positions (bp)	Nipponbare	Kasalath	Nagina22	IR64	Pokkali	Bala	Tainung67	Azucena	Moroberekan	*Oryza rufipogon*	*Oryza nivara*	MSU gene locus IDs	Structural annotation	Functional annotation	SSR functional effects	Forward primers (5'-3')	Reverse primers (5'-3')	Amplified product size (bp)	Markers exhibiting polymorphism within *indica* rice	Transcrption factor genes-derived markers	Known cloned genes-derived markers	PMS markers corresponding with RM markers
PMS2288	Os_Chr04	12228496	12228513	(GA)9	(GA)9	(GA)9	(GA)9	(GA)9	(GA)9	(GA)9	(GA)9	(GA)9	(GA)9	(GA)8	-	Introns/Intergenic	-	-	CGATTAGTAATTCAGGGGCG	CCGCATCCTCTTTCTCATTC	212	A	A	A	NA
PMS2289	Os_Chr04	12280648	12280665	(CCG)6	(CCG)5	(CCG)6	(CCG)6	(CCG)5	(CCG)5	(CCG)6	(CCG)6	(CCG)5	(CCG)5	(CCG)6	LOC_Os04g21710	URR	expressed protein	-	CTGACGATCGCTTGAGGTTC	TTCACTCAGGTGTTCTTGCG	222	P	A	A	NA
PMS2290	Os_Chr04	12410680	12410697	(AT)9	(AT)13	(AT)9	(AT)9	(AT)9	(AT)9	(AT)9	(AT)9	(AT)9	(AT)9	(AT)9	-	Introns/Intergenic	-	-	TTGATTGTCATGTCGCACG	GGGGACATTGAAAGGAAAAA	221	P	A	A	NA
PMS2291	Os_Chr04	12441392	12441443	(TA)26	(TA)26	(TA)26	(TA)26	(TA)26	(TA)7	(TA)26	(TA)11	(TA)7	(TA)26	(TA)26	-	Introns/Intergenic	-	-	AGGGAGTAGCTAGCTCATGAAGG	CACAATGGACCACAAATACTCC	598	P	A	A	RM16623
PMS2292	Os_Chr04	12448380	12448409	(CTT)10	(CTT)10	(CTT)10	(CTT)10	(CTT)10	(CTT)8	(CTT)10	(CTT)10	(CTT)10	(CTT)10	(CTT)10	LOC_Os04g21990	CDS	retrotransposon protein, putative, unclassified, expressed	CODON_DELETION	CGTTTCTGTTTGGTCTGCAA	GGGCTTTGCTATCAATTTCG	261	P	A	A	NA
PMS2293	Os_Chr04	12551646	12551659	(AG)7	(AG)7	(AG)7	(AG)7	(AG)7	(AG)6	(AG)7	(AG)7	(AG)7	(AG)7	(AG)7	-	Introns/Intergenic	-	-	GAGGAAGGGAGAGAAGAGGG	AATAGGTCTTTCGGCGGTTT	139	P	A	A	NA
PMS2294	Os_Chr04	12880317	12880338	(TG)11	(TG)11	(TG)11	(TG)11	(TG)11	(TG)11	(TG)11	(TG)11	(TG)11	(TG)9	(TG)11	-	Introns/Intergenic	-	-	GATTGCAGCAGTGAAGATGAAGG	AGGATCCGGATAATGTCGATGG	263	A	A	A	RM16628
PMS2295	Os_Chr04	12943089	12943102	(AG)7	(AG)7	(AG)7	(AG)6	(AG)6	(AG)7	(AG)7	(AG)7	(AG)7	(AG)7	(AG)7	-	Introns/Intergenic	-	-	AACGGAGCTCGGATGATTTA	CTCTCTCTTGAGCCGCTGAC	222	P	A	A	NA
PMS2296	Os_Chr04	13224455	13224472	(CGG)6	(CGG)6	(CGG)6	(CGG)6	(CGG)6	(CGG)6	(CGG)5	(CGG)6	(CGG)6	(CGG)6	(CGG)6	LOC_Os04g23220	CDS	expressed protein	CODON_DELETION	GGATGGAGATGATGAGCTGG	CTCATCCAGCCTAACCCGAC	208	A	A	A	NA
PMS2297	Os_Chr04	13394435	13394470	(AT)18	(AT)18	(AT)9	(AT)18	(AT)18	(AT)18	(AT)18	(AT)18	(AT)7	(AT)18	(AT)18	-	Introns/Intergenic	-	-	CAACAGTGGCTCCTAACAGAACG	ATGGGATTTGCTCGTCCTATGG	315	P	A	A	RM16642
PMS2298	Os_Chr04	13475858	13475929	(TA)36	(TA)36	(TA)36	(TA)36	(TA)9	(TA)36	(TA)36	(TA)36	(TA)36	(TA)36	(TA)36	-	Introns/Intergenic	-	-	TTTACTCCCGGTTGGACTAACG	CGACGGAAACACAATTCATAGG	389	P	A	A	RM16646
PMS2299	Os_Chr04	13566407	13566424	(GCG)6	(GCG)6	(GCG)6	(GCG)6	(GCG)6	(GCG)6	(GCG)5	(GCG)5	(GCG)6	(GCG)6	(GCG)5	-	Introns/Intergenic	-	-	CATGTTCATACATGGCCCAC	TGATACCTTGGGTTTTTGCC	262	A	A	A	NA
PMS2300	Os_Chr04	13605422	13605601	(AT)90	(AT)90	(AT)8	(AT)9	(AT)10	(AT)90	(AT)90	(AT)90	(AT)7	(AT)90	(AT)7	-	Introns/Intergenic	-	-	TGACATTAGTTGTGGCAGATCC	CCTAGAATCTCATCTGTCTTCTGG	398	P	A	A	RM16652

Polymorphic SSR (PMS) markers	Chromosomes	Start physical positions (bp)	End physical positions (bp)	Nipponbare	Kasalath	Nagina22	IR64	Pokkali	Bala	Tainung67	Azucena	Moroberekan	*Oryza rufipogon*	*Oryza nivara*	MSU gene locus IDs	Structural annotation	Functional annotation	SSR functional effects	Forward primers (5'-3')	Reverse primers (5'-3')	Amplified product size (bp)	Markers exhibiting polymorphism within *indica* rice	Transcrption factor genes-derived markers	Known cloned genes-derived markers	PMS markers corresponding with RM markers
PMS2301	Os_Chr04	13605953	13605976	(TA)12	(TA)12	(TA)12	(TA)6	(TA)12	(TA)12	(TA)12	(TA)12	(TA)12	(TA)12	(TA)7	-	Introns/Intergenic	-	-	TGAAGCACCTCATGTTTGACTAGC	CATGTGGTTTCATCACTAGGGTTAGC	161	P	A	A	RM16653
PMS2302	Os_Chr04	13620389	13620406	(CCG)6	(CCG)5	(CCG)6	(CCG)6	(CCG)5	(CCG)6	(CCG)6	(CCG)6	(CCG)6	(CCG)6	(CCG)6	-	Introns/Intergenic	-	-	CATATCCAACGCTCCCAGTT	GAGAGAAACGGTCGAGAGGG	250	P	A	A	NA
PMS2303	Os_Chr04	13654897	13654917	(GGT)7	(GGT)7	(GGT)7	(GGT)7	(GGT)7	(GGT)7	(GGT)6	(GGT)7	(GGT)7	(GGT)7	(GGT)7	-	Introns/Intergenic	-	-	CGATACCAATCCAATCAGTCAGG	GAGCTACCGTCGTGCTCACC	351	A	A	A	RM16657
PMS2304	Os_Chr04	13883348	13883397	(TA)25	(TA)25	(TA)6	(TA)25	(TA)8	(TA)25	(TA)25	(TA)25	(TA)10	(TA)25	(TA)25	-	Introns/Intergenic	-	-	TGAACTCGAGTGAAAGATGAGG	CTACAAGGAAACCTCCCTATTTGG	138	P	A	A	RM16670
PMS2305	Os_Chr04	14007149	14007164	(TA)8	(TA)8	(TA)8	(TA)6	(TA)8	(TA)8	(TA)8	(TA)8	(TA)8	(TA)8	(TA)8	-	Introns/Intergenic	-	-	TCCATCGTTGAAGGAGTATGT	TCCTTACATACCGGATGCTT	201	P	A	A	NA
PMS2306	Os_Chr04	14265575	14265592	(CGC)6	(CGC)6	(CGC)6	(CGC)6	(CGC)6	(CGC)6	(CGC)6	(CGC)6	(CGC)5	(CGC)6	(CGC)6	LOC_Os04g24820	CDS	OsFBX127 - F-box domain containing protein, expressed	CODON_DELETION	ACCAGCAACACGTACACCAG	GTGAATCCGTGCAACTCCTC	145	A	A	A	NA
PMS2307	Os_Chr04	14387026	14387077	(AT)26	(AT)26	(AT)6	(AT)11	(AT)26	(AT)26	(AT)10	(AT)26	(AT)26	(AT)26	(AT)9	-	Introns/Intergenic	-	-	GCTCCAATTCCTTCTAGGGC	CGTGAATGAGAACAAGCGAA	256	P	A	A	NA
PMS2308	Os_Chr04	14441764	14441778	(TGC)5	(TGC)5	(TGC)5	(TGC)5	(TGC)5	(TGC)5	(TGC)5	(TGC)5	(TGC)7	(TGC)5	(TGC)5	LOC_Os04g25040	CDS	retrotransposon protein, putative, Ty3-gypsy subclass, expressed	CODON_INSERTION	CTCGAAGCAGTTGAAGCACA	TCCGCTACACCAATCCCTAC	227	A	A	A	NA
PMS2309	Os_Chr04	14497247	14497262	(TG)8	(TG)7	(TG)8	(TG)8	(TG)8	(TG)7	(TG)8	(TG)8	(TG)7	(TG)7	(TG)8	-	Introns/Intergenic	-	-	TTAACTGTACCGCATTGGCTC	TGTCCCACTCTCCCTCTGTT	180	P	A	A	NA
PMS2310	Os_Chr04	14507204	14507227	(AT)12	(AT)11	(AT)12	(AT)12	(AT)12	(AT)12	(AT)12	(AT)12	(AT)12	(AT)12	(AT)7	-	Introns/Intergenic	-	-	TAGCCGCGGATTTACAATAGC	GGCCTCATGTTGTAAAGAAACC	154	P	A	A	RM16681
PMS2311	Os_Chr04	14551538	14551557	(TC)10	(TC)10	(TC)10	(TC)10	(TC)10	(TC)10	(TC)10	(TC)8	(TC)10	(TC)10	(TC)10	-	Introns/Intergenic	-	-	CGGACATACAAAACAAGGTGG	ACCTACCCAGCCCAAACTCT	279	A	A	A	NA
PMS2312	Os_Chr04	14596772	14596823	(AT)26	(AT)26	(AT)6	(AT)17	(AT)26	(AT)26	(AT)26	(AT)26	(AT)26	(AT)26	(AT)16	-	Introns/Intergenic	-	-	GGGAAGAGAATGTTGGTGGA	CTCCGGTTACGACACGATTT	218	P	A	A	NA
PMS2313	Os_Chr04	14620731	14620748	(GA)9	(GA)9	(GA)9	(GA)9	(GA)9	(GA)9	(GA)9	(GA)6	(GA)9	(GA)9	(GA)6	-	Introns/Intergenic	-	-	ATGCCCGTACTGCTTGACTC	GTCGGGGCTTTAATTTCCTC	116	A	A	A	NA

Polymorphic SSR (PMS) markers	Chromosomes	Start physical positions (bp)	End physical positions (bp)	Nipponbare	Kasalath	Nagina22	IR64	Pokkali	Bala	Tainung67	Azucena	Moroberekan	*Oryza rufipogon*	*Oryza nivara*	MSU gene locus IDs	Structural annotation	Functional annotation	SSR functional effects	Forward primers (5'-3')	Reverse primers (5'-3')	Amplified product size (bp)	Markers exhibiting polymorphism within *indica* rice	Transcrption factor genes-derived markers	Known cloned genes-derived markers	PMS markers corresponding with RM markers
PMS2314	Os_Chr04	14637917	14637928	(GA)6	(GA)6	(GA)6	(GA)6	(GA)6	(GA)6	(GA)6	(GA)6	(GA)7	(GA)6	(GA)6	-	Introns/Intergenic	-	-	GGGCATTTATAGCAGAGCCA	CCGTGGCATCAGTGAGTAGA	213	A	A	A	NA
PMS2315	Os_Chr04	14722006	14722029	(CCG)8	(CCG)8	(CCG)8	(CCG)6	(CCG)8	(CCG)8	(CCG)8	(CCG)8	(CCG)8	(CCG)8	(CCG)8	LOC_Os04g25410	URR	pentatricopeptide repeat domain containing protein, putative, expressed	-	GCCTCGTCGACGTACTTCTTGC	CAACAAGCCGAGTGTGGTAAGG	197	P	A	A	RM16685
PMS2316	Os_Chr04	14727969	14727998	(GGA)10	(GGA)10	(GGA)10	(GGA)10	(GGA)10	(GGA)10	(GGA)10	(GGA)10	(GGA)10	(GGA)9	(GGA)8	-	Introns/Intergenic	-	-	GGCACTGCTTGCATATGGATCG	TGCCGGCGAACTTATCCTCTCC	94	A	A	A	RM16686
PMS2317	Os_Chr04	14743878	14743895	(GCG)6	(GCG)6	(GCG)6	(GCG)6	(GCG)6	(GCG)6	(GCG)6	(GCG)5	(GCG)6	(GCG)6	(GCG)6	LOC_Os04g25440	CDS	cytokinin-O-glucosyltransferase 2, putative, expressed	CODON_DELETION	CGAGCAGGTGTTTGTAGTGG	GGCTTCCCAGGTTCATGTC	219	A	A	A	NA
PMS2318	Os_Chr04	14745231	14745294	(AT)32	(AT)32	(AT)23	(AT)32	(AT)8	(AT)32	(AT)32	(AT)6	(AT)8	(AT)32	(AT)12	-	Introns/Intergenic	-	-	CCCTAATATTTCGCCCCTGT	TAACTTGGAGCTCAGGCACC	188	P	A	A	NA
PMS2319	Os_Chr04	14898260	14898292	(TAA)11	(TAA)11	(TAA)11	(TAA)10	(TAA)11	(TAA)11	(TAA)11	(TAA)11	(TAA)11	(TAA)11	(TAA)11	-	Introns/Intergenic	-	-	TGCTCACACTTTTGCTGTGT	TTTTAGTTGTACCACAATGGCA	237	P	A	A	NA
PMS2320	Os_Chr04	15023170	15023185	(AT)8	(AT)8	(AT)8	(AT)8	(AT)8	(AT)8	(AT)8	(AT)8	(AT)7	(AT)8	(AT)8	-	Introns/Intergenic	-	-	TGAATCCAACAGGTCCATCA	AGCTGGATACAACGATTGGC	201	A	A	A	NA
PMS2321	Os_Chr04	15063401	15063416	(AT)8	(AT)8	(AT)6	(AT)8	(AT)8	(AT)8	(AT)8	(AT)8	(AT)8	(AT)8	(AT)8	-	Introns/Intergenic	-	-	TCGTTTAGAACAGCGACACG	CTGTGCTCTCTTTGTGTGTGTG	276	P	A	A	NA
PMS2322	Os_Chr04	15126285	15126302	(CGG)6	(CGG)6	(CGG)6	(CGG)6	(CGG)6	(CGG)6	(CGG)6	(CGG)6	(CGG)6	(CGG)6	(CGG)5	-	Introns/Intergenic	-	-	AATCGGTTCTGGAGCACG	CCGATCTCGGAAAAGAGGAT	252	A	A	A	NA
PMS2323	Os_Chr04	15405154	15405199	(AT)23	(AT)23	(AT)23	(AT)23	(AT)23	(AT)23	(AT)23	(AT)10	(AT)23	(AT)23	(AT)23	-	Introns/Intergenic	-	-	CGACAACACGTCTTTTCTGC	AGACAAGCAAGAACGAGGGA	241	A	A	A	NA
PMS2324	Os_Chr04	15419147	15419228	(TA)41	(TA)31	(TA)41	(TA)41	(TA)7	(TA)41	(TA)41	(TA)9	(TA)7	(TA)41	(TA)41	-	Introns/Intergenic	-	-	GACTCAAACACGGATGACAGACC	GCTGGTTTGACCGAGGTAGG	271	P	A	A	RM16716
PMS2325	Os_Chr04	16122325	16122358	(AT)17	(AT)17	(AT)17	(AT)17	(AT)17	(AT)17	(AT)17	(AT)17	(AT)12	(AT)17	(AT)17	-	Introns/Intergenic	-	-	GAGTCTGTACCCCTGTGCG	GGTAGGTTGTGAGGGGGAGT	143	A	A	A	NA
PMS2326	Os_Chr04	16243413	16243432	(CT)10	(CT)10	(CT)10	(CT)10	(CT)10	(CT)10	(CT)10	(CT)10	(CT)10	(CT)7	(CT)10	-	Introns/Intergenic	-	-	ATCCCGAATCCAAATCCTTTCC	AACGACGACAGCGAACAGAGC	374	A	A	A	RM16730

Polymorphic SSR (PMS) markers	Chromosomes	Start physical positions (bp)	End physical positions (bp)	Nipponbare	Kasalath	Nagina22	IR64	Pokkali	Bala	Tainung67	Azucena	Moroberekan	*Oryza rufipogon*	*Oryza nivara*	MSU gene locus IDs	Structural annotation	Functional annotation	SSR functional effects	Forward primers (5'-3')	Reverse primers (5'-3')	Amplified product size (bp)	Markers exhibiting polymorphism within *indica* rice	Transcrption factor genes-derived markers	Known cloned genes-derived markers	PMS markers corresponding with RM markers
PMS2327	Os_Chr04	16449618	16449638	(GGC)7	(GGC)7	(GGC)7	(GGC)7	(GGC)7	(GGC)7	(GGC)6	(GGC)7	(GGC)7	(GGC)7	(GGC)5	LOC_Os04g27850	URR	oxidoreductase, putative, expressed	-	GGAAGAGACCGCGAGAGACACG	CGGCGACTCACCTAGGATCATGC	263	A	A	A	RM16735
PMS2328	Os_Chr04	16497408	16497425	(CCA)6	(CCA)6	(CCA)6	(CCA)6	(CCA)6	(CCA)6	(CCA)5	(CCA)6	(CCA)6	(CCA)6	(CCA)6	LOC_Os04g27950	CDS	expressed protein	CODON_DELETION	CTACCTCATCCGCCTCTCTG	GCCAGGCTGCTAAAACTGTC	265	A	A	A	NA
PMS2329	Os_Chr04	16509474	16509491	(GCC)6	(GCC)6	(GCC)6	(GCC)6	(GCC)6	(GCC)6	(GCC)6	(GCC)6	(GCC)6	(GCC)5	(GCC)6	LOC_Os04g27960	CDS	B3 DNA binding domain containing protein, expressed	CODON_DELETION	AGAAGGCACGAGAACCTTGA	TTGAATAGACGACGACACGC	206	A	P	A	NA
PMS2330	Os_Chr04	16512669	16512682	(TA)7	(TA)7	(TA)7	(TA)7	(TA)7	(TA)7	(TA)7	(TA)7	(TA)7	(TA)6	(TA)7	-	Introns/Intergenic	-	-	TTCATATATGCAACACGCAAA	CACAAAGCTAGTTGTATTTGGACC	265	A	A	A	NA
PMS2331	Os_Chr04	16593887	16593904	(GA)9	(GA)9	(GA)9	(GA)9	(GA)9	(GA)8	(GA)9	(GA)9	(GA)7	(GA)9	(GA)9	LOC_Os04g28100	URR	spotted leaf 11, putative, expressed	-	CTTCGCCATGTCGGTGAT	CGAGCGACTGAAGGAGAAAG	197	P	A	A	NA
PMS2332	Os_Chr04	16598028	16598090	(TAA)21	(TAA)21	(TAA)21	(TAA)21	(TAA)21	(TAA)21	(TAA)21	(TAA)9	(TAA)21	(TAA)21	(TAA)21	-	Introns/Intergenic	-	-	TTGCAAACACAGCCTCAATC	AGGCTGGGGAGTTTCTGAAT	259	A	A	A	NA
PMS2333	Os_Chr04	16685202	16685216	(CGG)5	(CGG)5	(CGG)5	(CGG)5	(CGG)5	(CGG)5	(CGG)5	(CGG)5	(CGG)5	(CGG)6	(CGG)5	LOC_Os04g28234	CDS	Rf1, mitochondrial precursor, putative, expressed	CODON_CHANGE_PLUS_CODON_INSERTION	ACATATGGGCCGCTATTGTC	GCGAGGATGGAGGAGAGAC	235	A	A	A	NA
PMS2334	Os_Chr04	16723673	16723726	(TTA)18	(TTA)18	(TTA)18	(TTA)18	(TTA)5	(TTA)18	(TTA)18	(TTA)18	(TTA)18	(TTA)18	(TTA)18	-	Introns/Intergenic	-	-	ACGACCACGATAGTTCAGGG	CGACCAAATTCACGGTTAAA	209	P	A	A	NA
PMS2335	Os_Chr04	16727156	16727173	(AT)9	(AT)9	(AT)9	(AT)9	(AT)9	(AT)9	(AT)9	(AT)7	(AT)9	(AT)8	(AT)9	-	Introns/Intergenic	-	-	TGATTTTTGGGTTCATTCATCA	GGGTTAGACCCATCCCACTT	187	A	A	A	NA
PMS2336	Os_Chr04	16807616	16807639	(CGA)8	(CGA)8	(CGA)8	(CGA)8	(CGA)8	(CGA)8	(CGA)8	(CGA)8	(CGA)8	(CGA)8	(CGA)5	LOC_Os04g28410	CDS	expressed protein	CODON_DELETION	GGACTCTCTACCTCGACCACTCC	GGCCGTACAAGTGCTGTAGCC	296	A	A	A	RM16746
PMS2337	Os_Chr04	16857100	16857115	(CT)8	(CT)7	(CT)7	(CT)8	(CT)8	(CT)7	(CT)8	(CT)8	(CT)8	(CT)8	(CT)8	-	Introns/Intergenic	-	-	CCATACATCGCATCCCTCTT	CGCTCGGTTCAACCTTACC	161	P	A	A	NA
PMS2338	Os_Chr04	16917054	16917069	(AC)8	(AC)6	(AC)8	(AC)8	(AC)8	(AC)6	(AC)8	(AC)8	(AC)8	(AC)8	(AC)6	-	Introns/Intergenic	-	-	AGATACTGCGAGTTCAGCCG	GGCTGTCGAAATGGTTCACT	252	P	A	A	NA
PMS2339	Os_Chr04	16929140	16929163	(TA)12	(TA)12	(TA)12	(TA)12	(TA)12	(TA)12	(TA)12	(TA)7	(TA)12	(TA)12	(TA)7	-	Introns/Intergenic	-	-	CACTGAGCCAAACCCGTTAT	GGCAAACAACTCTCATGGCT	158	A	A	A	NA

Polymorphic SSR (PMS) markers	Chromosomes	Start physical positions (bp)	End physical positions (bp)	Nipponbare	Kasalath	Nagina22	IR64	Pokkali	Bala	Tainung67	Azucena	Moroberekan	*Oryza rufipogon*	*Oryza nivara*	MSU gene locus IDs	Structural annotation	Functional annotation	SSR functional effects	Forward primers (5'-3')	Reverse primers (5'-3')	Amplified product size (bp)	Markers exhibiting polymorphism within *indica* rice	Transcrption factor genes-derived markers	Known cloned genes-derived markers	PMS markers corresponding with RM markers
PMS2340	Os_Chr04	16961579	16961594	(GA)8	(GA)8	(GA)8	(GA)8	(GA)8	(GA)8	(GA)8	(GA)7	(GA)8	(GA)8	(GA)8	-	Introns/Intergenic	-	-	ACGCGCTGAGAAAGTGAGAG	ACCAGCACCTATCCAAATCG	110	A	A	A	NA
PMS2341	Os_Chr04	17044243	17044254	(TA)6	(TA)7	(TA)6	(TA)6	(TA)6	(TA)6	(TA)6	(TA)6	(TA)6	(TA)6	(TA)7	-	Introns/Intergenic	-	-	ACCCAGTGCTGTCCAAAATC	GAGAAGCAGCCCGATGAATA	267	P	A	A	NA
PMS2342	Os_Chr04	17278892	17278911	(TA)10	(TA)10	(TA)10	(TA)10	(TA)10	(TA)10	(TA)10	(TA)10	(TA)10	(TA)9	(TA)10	-	Introns/Intergenic	-	-	CCCTAGTAGAGGGTGATTGATCC	CGTAGATCCTTGTACCAACGTACC	322	A	A	A	RM16765
PMS2343	Os_Chr04	17563281	17563294	(TC)7	(TC)7	(TC)7	(TC)7	(TC)7	(TC)7	(TC)7	(TC)7	(TC)7	(TC)6	(TC)7	-	Introns/Intergenic	-	-	CGTGGCGCTTATTTGGTATT	GCTCATCTCAAGGGGAGAAA	188	A	A	A	NA
PMS2344	Os_Chr04	17685794	17685817	(GA)12	(GA)12	(GA)12	(GA)12	(GA)12	(GA)12	(GA)12	(GA)12	(GA)12	(GA)12	(GA)9	-	Introns/Intergenic	-	-	GGTGGGAGAGAGGGAGAGA	CCCGATCTATCGTCGAGTTC	179	A	A	A	NA
PMS2345	Os_Chr04	17768290	17768313	(AT)12	(AT)12	(AT)12	(AT)12	(AT)12	(AT)12	(AT)12	(AT)12	(AT)12	(AT)12	(AT)6	-	Introns/Intergenic	-	-	CCGTTTCAGGTTATAAGACTTTCT	CCGGCGTATCCTTTCTTACA	280	A	A	A	NA
PMS2346	Os_Chr04	17824211	17824224	(AT)7	(AT)7	(AT)7	(AT)7	(AT)7	(AT)7	(AT)7	(AT)7	(AT)6	(AT)7	(AT)7	-	Introns/Intergenic	-	-	TCATGCATGTGGCATTTACA	AAGTGGATAAATCGTAATAAGCAAA	249	A	A	A	NA
PMS2347	Os_Chr04	17838189	17838203	(CGG)5	(CGG)5	(CGG)5	(CGG)5	(CGG)5	(CGG)5	(CGG)5	(CGG)5	(CGG)5	(CGG)5	(CGG)6	LOC_Os04g29920	CDS	reticulon domain containing protein, putative, expressed	CODON_INSERTION	CGAGAAATCTCATTTGGGGA	GGATCGAGATGCCTACCAAA	236	A	A	A	NA
PMS2348	Os_Chr04	17921276	17921291	(TA)8	(TA)8	(TA)8	(TA)8	(TA)6	(TA)8	(TA)8	(TA)8	(TA)8	(TA)8	(TA)8	-	Introns/Intergenic	-	-	ATTAGTCGTGGGTTCTTCGTA	TCTTATATTTTGGCACCACAT	336	P	A	A	NA
PMS2349	Os_Chr04	17935621	17935674	(TA)27	(TA)27	(TA)27	(TA)8	(TA)27	(TA)27	(TA)8	(TA)27	(TA)15	(TA)27	(TA)27	-	Introns/Intergenic	-	-	CCATGGAGCACCAAACAAAT	ACTGATTCGTTCTCGGTTGG	264	P	A	A	NA
PMS2350	Os_Chr04	18077125	18077208	(AGAT)21	(AGAT)21	(AGAT)21	(AGAT)9	(AGAT)20	(AGAT)21	(AGAT)21	(AGAT)21	(AGAT)21	(AGAT)21	(AGAT)20	-	Introns/Intergenic	-	-	ATGTCAACGTAGGAGCGTTTGTAGG	AAAGCTATATCAGGAAGCCGAAGC	217	P	A	A	RM16789
PMS2351	Os_Chr04	18112284	18112301	(GA)9	(GA)12	(GA)9	(GA)9	(GA)9	(GA)9	(GA)9	(GA)9	(GA)9	(GA)9	(GA)9	-	Introns/Intergenic	-	-	TTATGCCCAGAAGGTGGTTC	TTCCCTCAGTCCTCTCCTCTC	121	P	A	A	NA
PMS2352	Os_Chr04	18188256	18188271	(AT)8	(AT)8	(AT)8	(AT)8	(AT)8	(AT)8	(AT)8	(AT)7	(AT)8	(AT)8	(AT)8	-	Introns/Intergenic	-	-	AAAAATGCCGTACTACTATAACA	TATCATATTTGCCCTTTGGTA	195	A	A	A	NA

Polymorphic SSR (PMS) markers	Chromosomes	Start physical positions (bp)	End physical positions (bp)	Nipponbare	Kasalath	Nagina22	IR64	Pokkali	Bala	Tainung67	Azucena	Moroberekan	*Oryza rufipogon*	*Oryza nivara*	MSU gene locus IDs	Structural annotation	Functional annotation	SSR functional effects	Forward primers (5'-3')	Reverse primers (5'-3')	Amplified product size (bp)	Markers exhibiting polymorphism within *indica* rice	Transcrption factor genes-derived markers	Known cloned genes-derived markers	PMS markers corresponding with RM markers
PMS2353	Os_Chr04	18330058	18330097	(ATCT)10	(ATCT)9	(ATCT)10	(ATCT)10	(ATCT)10	(ATCT)9	(ATCT)10	(ATCT)10	(ATCT)10	(ATCT)10	(ATCT)5	-	Introns/Intergenic	-	-	AGTCATGCTGTGGTTTGTCAAGG	GAACGAAAGCCAGCAATACATACC	379	P	A	A	RM7113
PMS2354	Os_Chr04	18350635	18350682	(TA)24	(TA)24	(TA)7	(TA)24	(TA)24	(TA)24	(TA)24	(TA)24	(TA)24	(TA)24	(TA)6	-	Introns/Intergenic	-	-	TCAATTTCAACAATGGTGCC	TTGGAGCCCATACTCCATGT	134	P	A	A	NA
PMS2355	Os_Chr04	18366060	18366077	(CGC)6	(CGC)6	(CGC)6	(CGC)6	(CGC)6	(CGC)6	(CGC)6	(CGC)6	(CGC)5	(CGC)6	(CGC)6	LOC_Os04g30720	CDS	kinesin motor domain containing protein, putative, expressed	CODON_DELETION	GGGATGAAGGAGATTAGGGC	GATCTCCACAACCCCAAAAA	203	A	A	A	NA
PMS2356	Os_Chr04	18419934	18419951	(CCG)6	(CCG)5	(CCG)6	(CCG)6	(CCG)6	(CCG)6	(CCG)6	(CCG)6	(CCG)6	(CCG)6	(CCG)6	LOC_Os04g30810	CDS	OsFBX129 - F-box domain containing protein, expressed	CODON_DELETION	CTTCCAGTCCCAGTACCGC	CACATCCACGTCCAAGAAGA	255	P	A	A	NA
PMS2357	Os_Chr04	18489736	18489757	(GA)11	(GA)11	(GA)11	(GA)6	(GA)6	(GA)11	(GA)11	(GA)11	(GA)11	(GA)7	(GA)11	-	Introns/Intergenic	-	-	AAACCGGAACCCAGGTTGGAAGG	ATTTCCGGGCAACTTCCTCTTGC	75	P	A	A	RM16815
PMS2358	Os_Chr04	18499991	18500008	(TC)9	(TC)9	(TC)9	(TC)9	(TC)9	(TC)9	(TC)9	(TC)9	(TC)7	(TC)9	(TC)9	-	Introns/Intergenic	-	-	CATCCACAATGCAATGAAAAA	CTCCATGCATGTGTCCAAAG	272	A	A	A	NA
PMS2359	Os_Chr04	18787007	18787020	(TG)7	(TG)7	(TG)7	(TG)7	(TG)7	(TG)7	(TG)7	(TG)7	(TG)7	(TG)8	(TG)7	LOC_Os04g31410	DRR	RALFL37 - Rapid ALkalinization Factor RALF family protein precursor, expressed	-	CAGTGCATCTACCACAACCG	TCACGGCAATCAATTCGTAA	227	A	A	A	NA
PMS2360	Os_Chr04	18825721	18825744	(TC)12	(TC)12	(TC)12	(TC)12	(TC)12	(TC)13	(TC)12	(TC)12	(TC)12	(TC)8	(TC)12	-	Introns/Intergenic	-	-	AACGTGAGACCCACATGTCA	CATAGCTTGGTGGCGAAGAC	279	P	A	A	NA
PMS2361	Os_Chr04	18913822	18913845	(AT)12	(AT)12	(AT)12	(AT)12	(AT)12	(AT)12	(AT)12	(AT)12	(AT)6	(AT)12	(AT)12	-	Introns/Intergenic	-	-	TAATCCAGCCGTCCGTTTCTTGC	TTGTGTTTCATTATGGCGCTACGG	460	A	A	A	RM16832
PMS2362	Os_Chr04	18965381	18965402	(TA)11	(TA)11	(TA)11	(TA)11	(TA)11	(TA)8	(TA)11	(TA)10	(TA)11	(TA)11	(TA)11	-	Introns/Intergenic	-	-	ATTAGATACCATGGTGCCCG	TTGCACAGCGGAGTAAACAG	100	P	A	A	NA
PMS2363	Os_Chr04	18976082	18976102	(CGC)7	(CGC)5	(CGC)7	(CGC)7	(CGC)7	(CGC)5	(CGC)7	(CGC)7	(CGC)7	(CGC)7	(CGC)7	LOC_Os04g31670	CDS	expressed protein	CODON_DELETION	GCGCGATTGCAAGCTTCTTCC	GGCGGCGATCTCCTTCATGC	492	P	A	A	RM16836
PMS2364	Os_Chr04	19169197	19169214	(GCC)6	(GCC)6	(GCC)6	(GCC)6	(GCC)6	(GCC)6	(GCC)6	(GCC)5	(GCC)6	(GCC)7	(GCC)6	LOC_Os04g32010	CDS	thiamine pyrophosphate enzyme, C-terminal TPP binding domain containing protein, expressed	CODON_INSERTION	TCACCAAACCAGTGATGAGC	CGTCGTCGAGCACCTCTC	258	A	A	A	NA
PMS2365	Os_Chr04	19183781	19183818	(CT)19	(CT)19	(CT)19	(CT)19	(CT)19	(CT)19	(CT)19	(CT)19	(CT)19	(CT)21	(CT)19	-	Introns/Intergenic	-	-	GTAGCCTTGCACTCGACCGTACC	ACCAACTCTGGCAATGCATCC	97	A	A	A	RM16852

Polymorphic SSR (PMS) markers	Chromosomes	Start physical positions (bp)	End physical positions (bp)	Nipponbare	Kasalath	Nagina22	IR64	Pokkali	Bala	Tainung67	Azucena	Moroberekan	*Oryza rufipogon*	*Oryza nivara*	MSU gene locus IDs	Structural annotation	Functional annotation	SSR functional effects	Forward primers (5'-3')	Reverse primers (5'-3')	Amplified product size (bp)	Markers exhibiting polymorphism within *indica* rice	Transcrption factor genes-derived markers	Known cloned genes-derived markers	PMS markers corresponding with RM markers
PMS2366	Os_Chr04	19189484	19189501	(CCG)6	(CCG)6	(CCG)6	(CCG)6	(CCG)6	(CCG)6	(CCG)6	(CCG)6	(CCG)6	(CCG)7	(CCG)6	LOC_Os04g32030	CDS	heavy metal-associated domain containing protein, expressed	CODON_INSERTION	AGGAAGGCAAGAAGGAGGAG	GGGAGCAAAGAACACAGCAT	277	A	A	A	NA
PMS2367	Os_Chr04	19273700	19273714	(CGC)5	(CGC)5	(CGC)5	(CGC)5	(CGC)5	(CGC)6	(CGC)5	(CGC)5	(CGC)5	(CGC)5	(CGC)5	LOC_Os04g32120	CDS	expressed protein	CODON_INSERTION	CTACCGTCGCGTTCTTGTTC	TACCTCCTCTTCCTCAGCCA	190	P	A	A	NA
PMS2368	Os_Chr04	19282413	19282424	(AG)6	(AG)7	(AG)6	(AG)7	(AG)7	(AG)7	(AG)6	(AG)6	(AG)6	(AG)6	(AG)7	-	Introns/Intergenic	-	-	CCCTAGCTCATTACCACCCA	TGAACCTACACCGAGCTGC	100	P	A	A	NA
PMS2369	Os_Chr04	19293178	19293198	(CTA)7	(CTA)7	(CTA)7	(CTA)7	(CTA)7	(CTA)7	(CTA)7	(CTA)7	(CTA)7	(CTA)7	(CTA)6	-	Introns/Intergenic	-	-	ACGATAACGGCTCTGTTTCTTCG	CGTATCTCGTGGTTGCAGATCG	119	A	A	A	RM16858
PMS2370	Os_Chr04	19314153	19314173	(GGA)7	(GGA)7	(GGA)6	(GGA)6	(GGA)7	(GGA)6	(GGA)7	(GGA)7	(GGA)7	(GGA)7	(GGA)7	LOC_Os04g32220	CDS	expressed protein	CODON_DELETION	GTTGCGGATAACTTCCTGAACG	ACCGTGAAGACTCGTTGCTTCC	404	P	A	A	RM16859
PMS2371	Os_Chr04	19329350	19329367	(GGC)6	(GGC)6	(GGC)6	(GGC)6	(GGC)6	(GGC)6	(GGC)6	(GGC)6	(GGC)6	(GGC)6	(GGC)5	LOC_Os04g32260	CDS	expressed protein	CODON_DELETION	AGCAGCAGACTCAGGGATTC	GTGAGACGGGAGATTGATCG	246	A	A	A	NA
PMS2372	Os_Chr04	19349662	19349676	(TGC)5	(TGC)5	(TGC)5	(TGC)5	(TGC)5	(TGC)5	(TGC)5	(TGC)5	(TGC)5	(TGC)6	(TGC)5	-	Introns/Intergenic	-	-	TATGGGTGGGTCGAGAAAAG	GAGTGAGTGGCTAAGGCAGG	277	A	A	A	NA
PMS2373	Os_Chr04	19366128	19366145	(GGA)6	(GGA)6	(GGA)6	(GGA)6	(GGA)6	(GGA)6	(GGA)5	(GGA)6	(GGA)6	(GGA)6	(GGA)6	LOC_Os04g32310	URR	serine/threonine-protein kinase NAK, putative, expressed	-	CCGGAGGTGATTCTGTTTTC	CATGCGGAGATCTTCTCGAC	258	A	A	A	NA
PMS2374	Os_Chr04	19371282	19371302	(GCG)7	(GCG)7	(GCG)7	(GCG)7	(GCG)7	(GCG)7	(GCG)7	(GCG)7	(GCG)7	(GCG)7	(GCG)5	-	Introns/Intergenic	-	-	ATTGTGGTCGTCGTCATCAACC	TTGCTCAGATACCTCGGCTTCG	139	A	A	A	RM16861
PMS2375	Os_Chr04	19378447	19378464	(CCG)6	(CCG)6	(CCG)6	(CCG)6	(CCG)5	(CCG)6	(CCG)6	(CCG)6	(CCG)6	(CCG)5	(CCG)6	LOC_Os04g32330	URR	dihydrolipoyllysine-residue succinyltransferase component of 2-oxoglutarate dehydrogenase complex, mitochondrial precursor, putative	-	ATCCATTCCCCTTCTCCTTC	CGTCTTCCCCAAGACAAAAA	250	P	A	A	NA
PMS2376	Os_Chr04	19472305	19472324	(TC)10	(TC)8	(TC)10	(TC)10	(TC)10	(TC)10	(TC)8	(TC)8	(TC)10	(TC)10	(TC)10	-	Introns/Intergenic	-	-	CCCTAATGCCAGCACTGTTT	CAACAAAGAGCAAAGAGCCC	224	P	A	A	NA
PMS2377	Os_Chr04	19477796	19477827	(AG)16	(AG)16	(AG)16	(AG)16	(AG)16	(AG)16	(AG)16	(AG)7	(AG)16	(AG)16	(AG)16	-	Introns/Intergenic	-	-	TAGCAAGCTTGGAGAAGTGATGG	CAGAAGAAGTCAGCTCTATGCTTGG	141	A	A	A	RM16874
PMS2378	Os_Chr04	19485252	19485275	(AT)12	(AT)12	(AT)12	(AT)12	(AT)6	(AT)12	(AT)12	(AT)12	(AT)6	(AT)12	(AT)12	-	Introns/Intergenic	-	-	CTATACGAAGGGTGAGCGTGTGC	AAGTTGCCTATACCACACCAATGC	276	P	A	A	RM16876

Polymorphic SSR (PMS) markers	Chromosomes	Start physical positions (bp)	End physical positions (bp)	Nipponbare	Kasalath	Nagina22	IR64	Pokkali	Bala	Tainung67	Azucena	Moroberekan	*Oryza rufipogon*	*Oryza nivara*	MSU gene locus IDs	Structural annotation	Functional annotation	SSR functional effects	Forward primers (5'-3')	Reverse primers (5'-3')	Amplified product size (bp)	Markers exhibiting polymorphism within *indica* rice	Transcrption factor genes-derived markers	Known cloned genes-derived markers	PMS markers corresponding with RM markers
PMS2379	Os_Chr04	19493005	19493022	(CGC)6	(CGC)6	(CGC)5	(CGC)6	(CGC)6	(CGC)6	(CGC)6	(CGC)6	(CGC)6	(CGC)6	(CGC)6	LOC_Os04g32480	CDS	zinc-finger protein, putative, expressed	CODON_CHANGE_PLUS_CODON_DELETION	CGATCTGAGTCGATTGCTTG	ACACCTTATCGTGGGAGACG	229	P	A	A	NA
PMS2380	Os_Chr04	19517138	19517201	(AT)32	(AT)32	(AT)32	(AT)7	(AT)8	(AT)32	(AT)32	(AT)18	(AT)9	(AT)11	(AT)32	-	Introns/Intergenic	-	-	ATATACTCAATTTGAATCTTGGAGC	CTCTTGGAGGGAGTGGAGGT	145	P	A	A	NA
PMS2381	Os_Chr04	19571015	19571038	(CAG)8	(CAG)7	(CAG)8	(CAG)8	(CAG)8	(CAG)8	(CAG)8	(CAG)8	(CAG)8	(CAG)8	(CAG)7	-	Introns/Intergenic	-	-	AGGGAATTCCAGCAAAGGAACC	GTTGCATTGCATAGCGACTCAGG	98	P	A	A	RM16888
PMS2382	Os_Chr04	19607680	19607699	(AG)10	(AG)10	(AG)10	(AG)10	(AG)10	(AG)10	(AG)10	(AG)10	(AG)10	(AG)10	(AG)8	-	Introns/Intergenic	-	-	TGCCATTTCTCCGACGAGACC	CCCTTCCTCCTCTCCCATCTCC	414	A	A	A	RM16891
PMS2383	Os_Chr04	19610527	19610550	(GGA)8	(GGA)7	(GGA)8	(GGA)8	(GGA)8	(GGA)8	(GGA)8	(GGA)8	(GGA)8	(GGA)8	(GGA)8	LOC_Os04g32580	CDS	expressed protein	CODON_DELETION	CCAACCCAAACCCTTGTACAAATCC	CTTCACCCGCTCCTCGAACC	155	P	A	A	RM16892
PMS2384	Os_Chr04	19615852	19615878	(GAA)9	(GAA)9	(GAA)9	(GAA)9	(GAA)9	(GAA)9	(GAA)9	(GAA)9	(GAA)9	(GAA)9	(GAA)8	-	Introns/Intergenic	-	-	GCACCAGTATCTCCTGCCTC	AGGCCACTCCCTCCTAATGT	215	A	A	A	NA
PMS2385	Os_Chr04	19635199	19635226	(AG)14	(AG)14	(AG)14	(AG)14	(AG)13	(AG)13	(AG)14	(AG)14	(AG)14	(AG)14	(AG)14	-	Introns/Intergenic	-	-	CAATCACAGAGCAACACGTACCC	GCAGAGGCAGCTGAGAAGTATAGC	159	P	A	A	RM1205
PMS2386	Os_Chr04	19656869	19656886	(AC)9	(AC)8	(AC)9	(AC)8	(AC)8	(AC)9	(AC)9	(AC)9	(AC)9	(AC)9	(AC)6	-	Introns/Intergenic	-	-	CAGCCATGCACAACTCAGTAA	TGAGTGGATTTGGTGGTGAA	219	P	A	A	NA
PMS2387	Os_Chr04	19685971	19685982	(TA)6	(TA)7	(TA)6	(TA)6	(TA)6	(TA)7	(TA)6	(TA)6	(TA)6	(TA)6	(TA)6	-	Introns/Intergenic	-	-	TGACCTAGCTGCATACGACG	CAAGGGTGGATTCCAAAAGA	210	P	A	A	NA
PMS2388	Os_Chr04	19784631	19784654	(AG)12	(AG)12	(AG)13	(AG)12	(AG)13	(AG)13	(AG)12	(AG)12	(AG)12	(AG)12	(AG)12	-	Introns/Intergenic	-	-	ATCGAGGACCACTGAAGCAAGC	GAGGCGATATTCAGAACGACAGG	479	P	A	A	RM16910
PMS2389	Os_Chr04	19791154	19791171	(CGG)6	(CGG)7	(CGG)6	(CGG)6	(CGG)6	(CGG)7	(CGG)6	(CGG)7	(CGG)7	(CGG)6	(CGG)6	LOC_Os04g32790	CDS	AP2 domain containing protein, expressed	CODON_CHANGE_PLUS_CODON_INSERTION	AGCAGCGACGAATACTCCAG	TAGTAGGACGACGACGAGCC	269	P	A	A	NA
PMS2390	Os_Chr04	19812433	19812453	(CGG)7	(CGG)7	(CGG)7	(CGG)7	(CGG)5	(CGG)6	(CGG)7	(CGG)7	(CGG)7	(CGG)7	(CGG)7	LOC_Os04g32810	CDS	expressed protein	CODON_DELETION	CGTCGACGTCAAACCAAACAGC	GCTGCAACAAACCTGCAGTCC	246	P	P	A	RM16912
PMS2391	Os_Chr04	19812580	19812597	(CGG)6	(CGG)6	(CGG)6	(CGG)6	(CGG)6	(CGG)5	(CGG)6	(CGG)6	(CGG)6	(CGG)6	(CGG)6	LOC_Os04g32810	CDS	expressed protein	CODON_DELETION	GGACTGCAGGTTTGTTGCAG	CAAGGTCGTCAAGGACATCA	239	P	P	A	NA

Polymorphic SSR (PMS) markers	Chromosomes	Start physical positions (bp)	End physical positions (bp)	Nipponbare	Kasalath	Nagina22	IR64	Pokkali	Bala	Tainung67	Azucena	Moroberekan	*Oryza rufipogon*	*Oryza nivara*	MSU gene locus IDs	Structural annotation	Functional annotation	SSR functional effects	Forward primers (5'-3')	Reverse primers (5'-3')	Amplified product size (bp)	Markers exhibiting polymorphism within *indica* rice	Transcrption factor genes-derived markers	Known cloned genes-derived markers	PMS markers corresponding with RM markers
PMS2392	Os_Chr04	19833699	19833716	(TC)9	(TC)9	(TC)9	(TC)9	(TC)9	(TC)9	(TC)9	(TC)9	(TC)9	(TC)8	(TC)9	-	Introns/Intergenic	-	-	GGACCACATCACCACACTGA	TAGTGGTTGTGGTTGGCAAG	100	A	A	A	NA
PMS2393	Os_Chr04	19861540	19861557	(AG)9	(AG)9	(AG)7	(AG)9	(AG)9	(AG)9	(AG)9	(AG)7	(AG)9	(AG)9	(AG)9	-	Introns/Intergenic	-	-	AGCAACCAACCCACTGCTAC	CCCACCTGTCACAGACACAC	279	P	A	A	NA
PMS2394	Os_Chr04	19904048	19904069	(AT)11	(AT)8	(AT)8	(AT)11	(AT)11	(AT)11	(AT)11	(AT)11	(AT)11	(AT)11	(AT)11	-	Introns/Intergenic	-	-	AATATTCGTCAGTGTCACGGTAGCC	CTGCTAAGCGAGCACACATGC	269	P	A	A	RM16916
PMS2395	Os_Chr04	19916706	19916739	(TC)17	(TC)17	(TC)9	(TC)17	(TC)9	(TC)17	(TC)17	(TC)16	(TC)17	(TC)17	(TC)17	LOC_Os04g32950	URR	OsCNX	-	GCAAATTCTCCACCAAGGAA	TCCAGTTCCGCCTCTTCTTA	273	P	A	P	NA
PMS2396	Os_Chr04	19980942	19980959	(GA)9	(GA)9	(GA)9	(GA)9	(GA)9	(GA)9	(GA)9	(GA)9	(GA)9	(GA)6	(GA)9	-	Introns/Intergenic	-	-	GTAACCGGAACGACCACATC	ATTCCTGAAAGCCACCACAC	240	A	A	A	NA
PMS2397	Os_Chr04	19992937	19992956	(GA)10	(GA)10	(GA)10	(GA)10	(GA)10	(GA)8	(GA)10	(GA)10	(GA)10	(GA)10	(GA)10	-	Introns/Intergenic	-	-	GTGGCACGTACTGCTTCTACTGG	CCACCACCCTGTACACATGACC	192	P	A	A	RM16925
PMS2398	Os_Chr04	20196024	20196044	(GGC)7	(GGC)7	(GGC)7	(GGC)7	(GGC)5	(GGC)7	(GGC)7	(GGC)7	(GGC)7	(GGC)7	(GGC)7	LOC_Os04g33350	CDS	expressed protein	CODON_DELETION	CGACGATCTGGAACAATCTCAGC	ACAGATCAAGCGATCTTGGATGC	150	P	A	A	RM16936
PMS2399	Os_Chr04	20339082	20339103	(CT)11	(CT)11	(CT)11	(CT)11	(CT)11	(CT)11	(CT)11	(CT)11	(CT)11	(CT)11	(CT)8	-	Introns/Intergenic	-	-	TGGAGTTTCTTCTCCTCCCACTCG	CAGAGAGCTAACGCCGGAGAGACC	186	A	A	A	RM16940
PMS2400	Os_Chr04	20494337	20494357	(GGA)7	(GGA)7	(GGA)7	(GGA)7	(GGA)7	(GGA)7	(GGA)7	(GGA)7	(GGA)7	(GGA)7	(GGA)5	-	Introns/Intergenic	-	-	GGAAGAGCTCAACTGCATGTCTGC	CTAAGTTCCCGCCCGTGTGC	469	A	A	A	RM16946
PMS2401	Os_Chr04	20531125	20531142	(GCG)6	(GCG)6	(GCG)6	(GCG)6	(GCG)6	(GCG)6	(GCG)6	(GCG)6	(GCG)6	(GCG)5	(GCG)6	LOC_Os04g33890	CDS	expressed protein	CODON_DELETION	GGAGCTCTCCTCGAACTCCT	CAGCGACAACTTCTCCTCCT	208	A	A	A	NA
PMS2402	Os_Chr04	20534056	20534069	(GC)7	(GC)6	(GC)7	(GC)7	(GC)7	(GC)6	(GC)7	(GC)7	(GC)7	(GC)7	(GC)7	-	Introns/Intergenic	-	-	ATCCCCCTGATCCCATTAAC	TCGCTCCAGAAGAAGGTCAT	268	P	A	A	NA
PMS2403	Os_Chr04	20575272	20575287	(CA)8	(CA)8	(CA)8	(CA)8	(CA)8	(CA)8	(CA)8	(CA)8	(CA)8	(CA)8	(CA)7	-	Introns/Intergenic	-	-	CTCCCGCCTCTCTAGTTGTG	TGGATATGATGGTGTAATCGGA	229	A	A	A	NA
PMS2404	Os_Chr04	20599591	20599622	(TC)16	(TC)15	(TC)15	(TC)16	(TC)16	(TC)15	(TC)16	(TC)16	(TC)16	(TC)16	(TC)15	-	Introns/Intergenic	-	-	TAAAGGTGTCCGACAAGAACACG	GATCGATCTGGAGGATTGAAAGG	201	P	A	A	RM5424

Polymorphic SSR (PMS) markers	Chromosomes	Start physical positions (bp)	End physical positions (bp)	Nipponbare	Kasalath	Nagina22	IR64	Pokkali	Bala	Tainung67	Azucena	Moroberekan	*Oryza rufipogon*	*Oryza nivara*	MSU gene locus IDs	Structural annotation	Functional annotation	SSR functional effects	Forward primers (5'-3')	Reverse primers (5'-3')	Amplified product size (bp)	Markers exhibiting polymorphism within *indica* rice	Transcrption factor genes-derived markers	Known cloned genes-derived markers	PMS markers corresponding with RM markers
PMS2405	Os_Chr04	20636180	20636197	(GCC)6	(GCC)6	(GCC)6	(GCC)6	(GCC)6	(GCC)6	(GCC)5	(GCC)6	(GCC)6	(GCC)6	(GCC)6	-	Introns/Intergenic	-	-	AGGTGAATGAGCGGAGGAG	GGTACGCAGAGAGCGAAATC	230	A	A	A	NA
PMS2406	Os_Chr04	20661483	20661500	(CAG)6	(CAG)5	(CAG)5	(CAG)5	(CAG)5	(CAG)5	(CAG)6	(CAG)6	(CAG)6	(CAG)6	(CAG)5	-	Introns/Intergenic	-	-	AAGAACCCGAGGAAATGGTT	TCTCCGATCCTCATCTTGCT	235	A	A	A	NA
PMS2407	Os_Chr04	20729102	20729116	(CCG)5	(CCG)6	(CCG)5	(CCG)5	(CCG)5	(CCG)5	(CCG)5	(CCG)5	(CCG)5	(CCG)5	(CCG)5	LOC_Os04g34220	CDS	expressed protein	CODON_INSERTION	CTTAGCACTGCCATCACTC	TCGAATTTTCAGGGTGTTAC	383	P	A	A	NA
PMS2408	Os_Chr04	20730680	20730701	(GA)11	(GA)11	(GA)11	(GA)11	(GA)11	(GA)11	(GA)6	(GA)11	(GA)11	(GA)11	(GA)11	-	Introns/Intergenic	-	-	CATCCTCACCTCCTCTCCTGTACC	TCCCGGTTTCTCTCATCCTTCC	284	A	A	A	RM16961
PMS2409	Os_Chr04	20873904	20873923	(TC)10	(TC)9	(TC)9	(TC)9	(TC)9	(TC)9	(TC)10	(TC)10	(TC)10	(TC)10	(TC)9	-	Introns/Intergenic	-	-	GAGGGGGCTCTTCCTCTTC	AGTTGACAATCAGCAGCAGG	242	A	A	A	NA
PMS2410	Os_Chr04	20905229	20905249	(CAG)7	(CAG)7	(CAG)7	(CAG)7	(CAG)7	(CAG)6	(CAG)7	(CAG)7	(CAG)7	(CAG)7	(CAG)5	LOC_Os04g34540	CDS	expressed protein	CODON_DELETION	CAACGCGCACTTACCTGTTCG	GCATCGAGGAATGAGGAAAGATATGC	156	P	A	A	RM16966
PMS2411	Os_Chr04	20924884	20924901	(CGG)6	(CGG)5	(CGG)6	(CGG)6	(CGG)6	(CGG)5	(CGG)6	(CGG)6	(CGG)6	(CGG)6	(CGG)6	LOC_Os04g34580	CDS	FAD binding domain containing protein, expressed	CODON_DELETION	CACCATCCGGTTCTCGTC	AACTGCCTGAGCTCCTTCCT	261	P	A	A	NA
PMS2412	Os_Chr04	20930145	20930174	(TA)15	(TA)15	(TA)15	(TA)15	(TA)11	(TA)15	(TA)15	(TA)8	(TA)15	(TA)15	(TA)15	-	Introns/Intergenic	-	-	TGTCTGCATGTATATTGGCGA	AGGGCCTGTGTGCATAGGAT	239	P	A	A	NA
PMS2413	Os_Chr04	20930553	20930612	(AT)30	(AT)29	(AT)23	(AT)30	(AT)30	(AT)30	(AT)30	(AT)30	(AT)10	(AT)30	(AT)30	-	Introns/Intergenic	-	-	GGGCCTGTGTGCATAGGATA	AACGGTGGATGATTTTGGAG	138	P	A	A	NA
PMS2414	Os_Chr04	20974080	20974107	(TA)14	(TA)10	(TA)14	(TA)14	(TA)14	(TA)13	(TA)7	(TA)14	(TA)14	(TA)14	(TA)14	-	Introns/Intergenic	-	-	CGCTGTACAAAGTTCGTGGTAGG	AAAGTCCCAGTGCCTCCTAGTCC	179	P	A	A	RM16979
PMS2415	Os_Chr04	21024001	21024042	(AT)21	(AT)21	(AT)6	(AT)21	(AT)21	(AT)21	(AT)21	(AT)21	(AT)21	(AT)21	(AT)7	-	Introns/Intergenic	-	-	TGGCTCATTTTCTTCCATCC	TCATGCATGTTGGTTTGAGG	278	P	A	A	NA
PMS2416	Os_Chr04	21032273	21032286	(GT)7	(GT)6	(GT)6	(GT)6	(GT)6	(GT)6	(GT)7	(GT)7	(GT)7	(GT)7	(GT)6	-	Introns/Intergenic	-	-	AGAGGCATCTAGTGTGGCGT	AATTTCCGACGCATCCTATG	187	A	A	A	NA
PMS2417	Os_Chr04	21042734	21042753	(CT)10	(CT)10	(CT)6	(CT)10	(CT)10	(CT)10	(CT)10	(CT)10	(CT)10	(CT)10	(CT)7	-	Introns/Intergenic	-	-	CCTCAGATCTCAAATCCCCA	GAGGGAGCGGAGAGGGAG	254	P	A	A	NA

Polymorphic SSR (PMS) markers	Chromosomes	Start physical positions (bp)	End physical positions (bp)	Nipponbare	Kasalath	Nagina22	IR64	Pokkali	Bala	Tainung67	Azucena	Moroberekan	*Oryza rufipogon*	*Oryza nivara*	MSU gene locus IDs	Structural annotation	Functional annotation	SSR functional effects	Forward primers (5'-3')	Reverse primers (5'-3')	Amplified product size (bp)	Markers exhibiting polymorphism within *indica* rice	Transcrption factor genes-derived markers	Known cloned genes-derived markers	PMS markers corresponding with RM markers
PMS2418	Os_Chr04	21057527	21057544	(CTC)6	(CTC)6	(CTC)6	(CTC)6	(CTC)6	(CTC)6	(CTC)6	(CTC)5	(CTC)6	(CTC)6	(CTC)6	-	Introns/Intergenic	-	-	ATCCAAGAGTGGGAGAAGCA	GGGCAAAAGGAAAAAGGAAG	115	A	A	A	NA
PMS2419	Os_Chr04	21214922	21214937	(CT)8	(CT)8	(CT)8	(CT)8	(CT)8	(CT)8	(CT)8	(CT)8	(CT)8	(CT)8	(CT)6	-	Introns/Intergenic	-	-	AGTCACCAGGAGCGAAAGAA	CGCCCTGTGAGAGAGAGTGT	196	A	A	A	NA
PMS2420	Os_Chr04	21387362	21387379	(CGC)6	(CGC)6	(CGC)6	(CGC)6	(CGC)6	(CGC)6	(CGC)6	(CGC)5	(CGC)6	(CGC)6	(CGC)6	LOC_Os04g35200	URR	expressed protein	-	GAGATGTAGGAGCAGGTGGG	CTACCTCCAGTACCGCCTCC	228	A	A	A	NA
PMS2421	Os_Chr04	21405293	21405313	(CGG)7	(CGG)6	(CGG)7	(CGG)7	(CGG)7	(CGG)7	(CGG)7	(CGG)7	(CGG)7	(CGG)7	(CGG)7	LOC_Os04g35220	URR	expressed protein	-	CGATTCGAACCGAGATGGATGC	CATGGAACCGTGGCTGTTTGG	329	P	A	A	RM16998
PMS2422	Os_Chr04	21448530	21448547	(CGC)6	(CGC)6	(CGC)6	(CGC)6	(CGC)6	(CGC)6	(CGC)6	(CGC)6	(CGC)6	(CGC)5	(CGC)6	-	Introns/Intergenic	-	-	CCGACACCGTAAGTACACGA	GATGAAAATGTCCTCGTGCC	173	A	A	A	NA
PMS2423	Os_Chr04	21492336	21492356	(GGC)7	(GGC)7	(GGC)6	(GGC)7	(GGC)6	(GGC)6	(GGC)7	(GGC)7	(GGC)7	(GGC)7	(GGC)7	LOC_Os04g35340	CDS	MrBTB1 - Bric-a-Brac, Tramtrack, Broad Complex BTB domain with Meprin and TRAF Homology MATH-related domain, expressed	CODON_DELETION	TCTTCCCGCTGACCTCGAACG	TGCGACCTCACCGTCTTCATGG	178	P	A	A	RM17009
PMS2424	Os_Chr04	21501108	21501119	(TA)6	(TA)6	(TA)7	(TA)6	(TA)6	(TA)6	(TA)6	(TA)6	(TA)6	(TA)6	(TA)6	-	Introns/Intergenic	-	-	AACTTGGCAGCTCAGCTAGG	CGGATAGGTTCGGACTTCAG	218	P	A	A	NA
PMS2425	Os_Chr04	21525540	21525560	(CGC)7	(CGC)7	(CGC)7	(CGC)5	(CGC)7	(CGC)7	(CGC)7	(CGC)7	(CGC)7	(CGC)7	(CGC)7	LOC_Os04g35390	CDS	expressed protein	CODON_DELETION	CTCCATAAAAACCCAAGCGA	GAAGGAGAGGCTGGACGAC	255	P	A	A	NA
PMS2426	Os_Chr04	21536978	21537017	(CT)20	(CT)20	(CT)20	(CT)20	(CT)20	(CT)20	(CT)20	(CT)20	(CT)20	(CT)20	(CT)9	-	Introns/Intergenic	-	-	CGACACTTCCTAAATCCCCA	AGCAAAGGAAGGGAGGAGAG	137	A	A	A	NA
PMS2427	Os_Chr04	21544970	21544987	(GTG)6	(GTG)6	(GTG)6	(GTG)6	(GTG)6	(GTG)6	(GTG)6	(GTG)6	(GTG)6	(GTG)5	(GTG)6	-	Introns/Intergenic	-	-	AACGAGCAGCATCCTTTTGT	CGGTGCAGGTAGGTCGTC	174	A	A	A	NA
PMS2428	Os_Chr04	21567407	21567430	(CT)12	(CT)12	(CT)12	(CT)12	(CT)12	(CT)8	(CT)12	(CT)12	(CT)12	(CT)12	(CT)12	-	Introns/Intergenic	-	-	GAAGGCACTTCACTCACTCTGC	AGAGTACCACCCAACATCCATCC	244	P	A	A	RM1142
PMS2429	Os_Chr04	21679262	21679273	(AC)6	(AC)6	(AC)6	(AC)6	(AC)8	(AC)6	(AC)6	(AC)6	(AC)6	(AC)6	(AC)6	-	Introns/Intergenic	-	-	ATTTCATGGATCGGAGTTCG	TTTGTCCTCGTTCATCCTCC	255	P	A	A	NA
PMS2430	Os_Chr04	21728158	21728184	(GGC)9	(GGC)5	(GGC)9	(GGC)9	(GGC)9	(GGC)6	(GGC)9	(GGC)9	(GGC)9	(GGC)9	(GGC)9	LOC_Os04g35650	CDS	pentatricopeptide, putative, expressed	CODON_DELETION	CTGGGAGGAGGAGTATGACG	GTTCAAGAATCAAGGCCCAA	224	P	A	A	NA

Polymorphic SSR (PMS) markers	Chromosomes	Start physical positions (bp)	End physical positions (bp)	Nipponbare	Kasalath	Nagina22	IR64	Pokkali	Bala	Tainung67	Azucena	Moroberekan	*Oryza rufipogon*	*Oryza nivara*	MSU gene locus IDs	Structural annotation	Functional annotation	SSR functional effects	Forward primers (5'-3')	Reverse primers (5'-3')	Amplified product size (bp)	Markers exhibiting polymorphism within *indica* rice	Transcrption factor genes-derived markers	Known cloned genes-derived markers	PMS markers corresponding with RM markers
PMS2431	Os_Chr04	21747351	21747368	(GCT)6	(GCT)6	(GCT)6	(GCT)6	(GCT)5	(GCT)5	(GCT)6	(GCT)6	(GCT)6	(GCT)5	(GCT)6	LOC_Os04g35680	CDS	U-box domain containing protein, expressed	CODON_DELETION	TGATGAACTCGAGCAACGAC	CATGGATTTGAGCAGCTTGA	194	P	A	A	NA
PMS2432	Os_Chr04	21987210	21987233	(GTG)8	(GTG)7	(GTG)7	(GTG)8	(GTG)8	(GTG)7	(GTG)8	(GTG)8	(GTG)8	(GTG)8	(GTG)8	-	Introns/Intergenic	-	-	TGATGGGTCAGCATCATGTGG	CTCCTTCGTGTGTTCCTTCTGG	400	P	A	A	RM17040
PMS2433	Os_Chr04	22141412	22141427	(GA)8	(GA)9	(GA)8	(GA)8	(GA)8	(GA)8	(GA)8	(GA)8	(GA)8	(GA)9	(GA)8	-	Introns/Intergenic	-	-	GGGGAGAGGTTGAGCGAG	ATCCCGAATCCAATTCCTTC	193	P	A	A	NA
PMS2434	Os_Chr04	22180070	22180101	(AT)16	(AT)16	(AT)7	(AT)16	(AT)16	(AT)16	(AT)16	(AT)16	(AT)16	(AT)16	(AT)16	-	Introns/Intergenic	-	-	GTGAAACGAGAGAAGTGAAGTTGG	CCCACAACTGTGTTTACTATGAGACG	187	P	A	A	RM17056
PMS2435	Os_Chr04	22243935	22243961	(GCG)9	(GCG)9	(GCG)9	(GCG)9	(GCG)9	(GCG)9	(GCG)9	(GCG)9	(GCG)6	(GCG)9	(GCG)9	LOC_Os04g36850	CDS	ORG4, putative, expressed	CODON_CHANGE_PLUS_CODON_DELETION	GCGCCACTCATTTCTAGATTACG	GCACCACGTTGATCTTGTACAGC	260	A	A	A	RM17061
PMS2436	Os_Chr04	22258519	22258533	(GCC)5	(GCC)7	(GCC)5	(GCC)5	(GCC)5	(GCC)5	(GCC)5	(GCC)5	(GCC)5	(GCC)5	(GCC)5	-	Introns/Intergenic	-	-	TCTCCAACCCAGAGTTCCAC	CAAACCGAAAACGAGGAGAG	196	P	A	A	NA
PMS2437	Os_Chr04	22393808	22393828	(CGC)7	(CGC)7	(CGC)7	(CGC)7	(CGC)7	(CGC)7	(CGC)7	(CGC)7	(CGC)7	(CGC)5	(CGC)7	LOC_Os04g37660	CDS	protein phosphotase protein, putative, expressed	CODON_DELETION	AGTCATTGCAGTGGAGGAGACG	GGTCGGGATCTGAGGAGAGAGG	354	A	A	A	RM17066
PMS2438	Os_Chr04	22442443	22442463	(CGC)7	(CGC)7	(CGC)7	(CGC)7	(CGC)7	(CGC)7	(CGC)7	(CGC)7	(CGC)7	(CGC)7	(CGC)5	LOC_Os04g37740	CDS	zinc finger, C3HC4 type domain containing protein, expressed	CODON_DELETION	CTCGAGACCTTCAGGCAAGACG	GGAACGCCAAGAAGAAGAAGAGG	285	A	A	A	RM17069
PMS2439	Os_Chr04	22508280	22508319	(TA)20	(TA)20	(TA)10	(TA)10	(TA)20	(TA)20	(TA)20	(TA)20	(TA)20	(TA)20	(TA)20	-	Introns/Intergenic	-	-	ATCTCTAGGGCCCTGATTACG	GTTGTTTGAGCTTGAGCTTACC	461	P	A	A	RM17071
PMS2440	Os_Chr04	22514514	22514527	(AT)7	(AT)7	(AT)7	(AT)7	(AT)7	(AT)7	(AT)7	(AT)7	(AT)7	(AT)6	(AT)7	-	Introns/Intergenic	-	-	TGACATGTGGGTCGCAGTAT	GGGAGAACCGGTGTGATAGA	150	A	A	A	NA
PMS2441	Os_Chr04	22614959	22614978	(GA)10	(GA)10	(GA)10	(GA)10	(GA)10	(GA)10	(GA)10	(GA)8	(GA)8	(GA)8	(GA)10	-	Introns/Intergenic	-	-	GTTTGGAGCGGAGCGACGAC	ATCCTTCTTGGCCCATCT	273	A	A	A	NA
PMS2442	Os_Chr04	22846214	22846249	(TA)18	(TA)18	(TA)12	(TA)18	(TA)18	(TA)9	(TA)18	(TA)18	(TA)18	(TA)9	(TA)7	-	Introns/Intergenic	-	-	CCGGATATATTATGCAGCCA	ATGCAATTAATCCGTGCGTA	272	P	A	A	NA
PMS2443	Os_Chr04	22860562	22860585	(CCG)8	(CCG)7	(CCG)8	(CCG)8	(CCG)8	(CCG)7	(CCG)8	(CCG)8	(CCG)8	(CCG)8	(CCG)8	LOC_Os04g38450	CDS	gamma-glutamyltranspeptidase 1 precursor, putative, expressed	CODON_DELETION	TGACTACACCGGATTGTACGCTACC	GCTGGTGCTCCTCCTCTTAGCC	369	P	A	A	RM17086

Polymorphic SSR (PMS) markers	Chromosomes	Start physical positions (bp)	End physical positions (bp)	Nipponbare	Kasalath	Nagina22	IR64	Pokkali	Bala	Tainung67	Azucena	Moroberekan	*Oryza rufipogon*	*Oryza nivara*	MSU gene locus IDs	Structural annotation	Functional annotation	SSR functional effects	Forward primers (5'-3')	Reverse primers (5'-3')	Amplified product size (bp)	Markers exhibiting polymorphism within *indica* rice	Transcrption factor genes-derived markers	Known cloned genes-derived markers	PMS markers corresponding with RM markers
PMS2444	Os_Chr04	22860723	22860740	(CG)9	(CG)9	(CG)6	(CG)9	(CG)9	(CG)6	(CG)9	(CG)9	(CG)9	(CG)9	(CG)9	-	Introns/Intergenic	-	-	AAAAGCTTGGCGAATTGGTA	TTCCAGCTCTCTTGGCAAAT	215	P	A	A	NA
PMS2445	Os_Chr04	22938149	22938166	(CT)9	(CT)9	(CT)9	(CT)7	(CT)9	(CT)9	(CT)9	(CT)9	(CT)9	(CT)9	(CT)9	-	Introns/Intergenic	-	-	GATCCTCGACCAGAAGTTCG	TAGGAGTGGGTGGTGGTCAT	144	P	A	A	NA
PMS2446	Os_Chr04	23025935	23025955	(CGG)7	(CGG)6	(CGG)7	(CGG)7	(CGG)7	(CGG)7	(CGG)7	(CGG)7	(CGG)7	(CGG)7	(CGG)7	LOC_Os04g38740	CDS	MYB family transcription factor, putative, expressed	CODON_CHANGE_PLUS_CODON_DELETION	CTGGAACACGCACCTAAAGAAGC	TCAAGGAGCCTTCGAGGAGTAGG	539	P	P	A	RM17095
PMS2447	Os_Chr04	23068840	23068897	(AT)29	(AT)29	(AT)8	(AT)29	(AT)6	(AT)29	(AT)29	(AT)29	(AT)29	(AT)29	(AT)29	-	Introns/Intergenic	-	-	ATTTGGCTAGGTTTGACGGATGC	GATTGGGCTGAGCCGATACG	262	P	A	A	RM17099
PMS2448	Os_Chr04	23097552	23097605	(AT)27	(AT)27	(AT)27	(AT)9	(AT)27	(AT)27	(AT)7	(AT)12	(AT)27	(AT)27	(AT)27	-	Introns/Intergenic	-	-	TCGTCAACACCAACCTAATACCG	TCCGCGAGTGTTGTAACTTTAACC	331	P	A	A	RM2439
PMS2449	Os_Chr04	23182080	23182097	(CCG)6	(CCG)8	(CCG)6	(CCG)6	(CCG)6	(CCG)6	(CCG)6	(CCG)6	(CCG)6	(CCG)6	(CCG)6	LOC_Os04g39030	CDS	peptide transporter PTR2, putative, expressed	CODON_INSERTION	GGACACGTTCGCCTTCAG	CAGCACACAACACAGCATGA	241	P	A	A	NA
PMS2450	Os_Chr04	23186146	23186163	(GGC)6	(GGC)6	(GGC)6	(GGC)6	(GGC)6	(GGC)6	(GGC)6	(GGC)6	(GGC)6	(GGC)5	(GGC)6	LOC_Os04g39040	CDS	UBA and UBX domain-containing protein, putative, expressed	CODON_DELETION	AGCCAAACCCTACCTTCCAC	TGTAGTACTCCTGGGGCTCG	226	A	A	A	NA
PMS2451	Os_Chr04	23197289	23197310	(AG)11	(AG)10	(AG)11	(AG)10	(AG)11	(AG)11	(AG)11	(AG)11	(AG)11	(AG)11	(AG)11	-	Introns/Intergenic	-	-	CTGCTCTAGTACTACCCACTTGTGC	TTGTATGTGTCAGCTTGGAGTGG	109	P	A	A	RM17104
PMS2452	Os_Chr04	23361639	23361656	(CCG)6	(CCG)6	(CCG)6	(CCG)6	(CCG)6	(CCG)6	(CCG)5	(CCG)6	(CCG)6	(CCG)6	(CCG)6	LOC_Os04g39260	URR	CAF1 family ribonuclease containing protein, putative, expressed	-	GGAGCTTGAGGTAGCGGAC	GCCGTCCAGAACCCTACTCT	257	A	A	A	NA
PMS2453	Os_Chr04	23378370	23378387	(CA)9	(CA)8	(CA)9	(CA)9	(CA)9	(CA)9	(CA)9	(CA)9	(CA)9	(CA)9	(CA)9	-	Introns/Intergenic	-	-	AGAGCAGGCAGCACAGTACA	GTGTGGCATTTTTCTCGCTC	162	P	A	A	NA
PMS2454	Os_Chr04	23420490	23420516	(GAA)9	(GAA)11	(GAA)9	(GAA)9	(GAA)9	(GAA)9	(GAA)9	(GAA)9	(GAA)9	(GAA)9	(GAA)9	-	Introns/Intergenic	-	-	AGTGATGCACTTCTGTTTGTCC	GGTCCTCTTGTTCAAGTCAAACC	108	P	A	A	RM17116
PMS2455	Os_Chr04	23481368	23481382	(CCA)5	(CCA)6	(CCA)5	(CCA)5	(CCA)5	(CCA)5	(CCA)5	(CCA)5	(CCA)5	(CCA)5	(CCA)5	-	Introns/Intergenic	-	-	CCTGCACTGACCATCTTTCA	CGGAGGAGTACGAGAGTGGA	147	P	A	A	NA
PMS2456	Os_Chr04	23504434	23504445	(CT)6	(CT)6	(CT)6	(CT)6	(CT)6	(CT)6	(CT)6	(CT)6	(CT)6	(CT)8	(CT)6	-	Introns/Intergenic	-	-	AATTTTCCATGGGACCATCA	ACATGTTTGAATCCATGGGC	104	A	A	A	NA

Polymorphic SSR (PMS) markers	Chromosomes	Start physical positions (bp)	End physical positions (bp)	Nipponbare	Kasalath	Nagina22	IR64	Pokkali	Bala	Tainung67	Azucena	Moroberekan	*Oryza rufipogon*	*Oryza nivara*	MSU gene locus IDs	Structural annotation	Functional annotation	SSR functional effects	Forward primers (5'-3')	Reverse primers (5'-3')	Amplified product size (bp)	Markers exhibiting polymorphism within *indica* rice	Transcrption factor genes-derived markers	Known cloned genes-derived markers	PMS markers corresponding with RM markers
PMS2457	Os_Chr04	23551558	23551578	(GGT)7	(GGT)7	(GGT)7	(GGT)7	(GGT)7	(GGT)7	(GGT)5	(GGT)7	(GGT)7	(GGT)6	(GGT)7	-	Introns/Intergenic	-	-	ACACGATCGACCAGCATCTCTCC	CTGCAACACGGACTTCGAGACG	404	A	A	A	RM17128
PMS2458	Os_Chr04	23559985	23559999	(GAA)5	(GAA)7	(GAA)5	(GAA)5	(GAA)5	(GAA)5	(GAA)5	(GAA)5	(GAA)5	(GAA)6	(GAA)6	-	Introns/Intergenic	-	-	ACCCAAGAGTGGATGACAGG	GTGCTGGATGGATGGATCTT	177	P	A	A	NA
PMS2459	Os_Chr04	23626646	23626669	(CAG)8	(CAG)8	(CAG)8	(CAG)8	(CAG)8	(CAG)8	(CAG)6	(CAG)8	(CAG)8	(CAG)8	(CAG)6	-	Introns/Intergenic	-	-	ACTTGTCATTCATTGCGCTTCG	CGTCAGGAGTGTCGTAGCCTAGC	87	A	A	A	RM6540
PMS2460	Os_Chr04	23626963	23626980	(CGG)6	(CGG)5	(CGG)6	(CGG)6	(CGG)6	(CGG)6	(CGG)6	(CGG)6	(CGG)6	(CGG)5	(CGG)6	LOC_Os04g39650	CDS	receptor protein kinase CLAVATA1 precursor, putative, expressed	CODON_DELETION	CTGCTCAGCTTCAGGTCCTC	GACGGTCTGGTTGAAGGTGT	243	P	A	A	NA
PMS2461	Os_Chr04	23697460	23697477	(CGT)6	(CGT)5	(CGT)6	(CGT)5	(CGT)6	(CGT)6	(CGT)6	(CGT)6	(CGT)6	(CGT)5	(CGT)5	LOC_Os04g39770	CDS	FCP1|OsCLE402	CODON_DELETION	TCTCATGGGATTCTGGGTTC	CTTTTCATGGCTTTCTTGCC	266	P	P	P	NA
PMS2462	Os_Chr04	23697768	23697788	(GCG)7	(GCG)7	(GCG)7	(GCG)7	(GCG)7	(GCG)7	(GCG)7	(GCG)6	(GCG)7	(GCG)7	(GCG)7	LOC_Os04g39770	CDS	FCP1|OsCLE402	CODON_DELETION	TACAAGAAAAGACCCCGCAC	CTGGATGGCTCATCCTCATT	182	A	P	P	NA
PMS2463	Os_Chr04	23877448	23877465	(CGG)6	(CGG)5	(CGG)6	(CGG)6	(CGG)6	(CGG)6	(CGG)6	(CGG)6	(CGG)6	(CGG)6	(CGG)6	LOC_Os04g40100	CDS	BTBN11 - Bric-a-Brac, Tramtrack, Broad Complex BTB domain with non-phototropic hypocotyl 3 NPH3 domain, expressed	CODON_DELETION	CTCGGGGTTGACCTCTACTG	CGGGAGCAAGCTCACTATCT	237	P	A	A	NA
PMS2464	Os_Chr04	23945137	23945166	(AGATT)6	(AGATT)5	(AGATT)6	(AGATT)6	(AGATT)6	(AGATT)6	(AGATT)6	(AGATT)6	(AGATT)6	(AGATT)6	(AGATT)6	-	Introns/Intergenic	-	-	ATCCCGTCATACGTGATGGT	CAAGGTGATTTGGAGAGGGA	260	P	A	A	NA
PMS2465	Os_Chr04	23972022	23972039	(GTC)6	(GTC)6	(GTC)6	(GTC)6	(GTC)6	(GTC)6	(GTC)5	(GTC)6	(GTC)6	(GTC)6	(GTC)6	LOC_Os04g40320	CDS	expressed protein	CODON_DELETION	GACGGAGATGGAGAGGGAG	AGAAAGCTTCCCCCAGAAAA	242	A	A	A	NA
PMS2466	Os_Chr04	24021723	24021740	(GGA)6	(GGA)6	(GGA)6	(GGA)6	(GGA)6	(GGA)6	(GGA)6	(GGA)6	(GGA)6	(GGA)5	(GGA)6	-	Introns/Intergenic	-	-	GAATCGAGGTTTCCTCCTCC	CTCGTGGATCTCATCCCACT	171	A	A	A	NA
PMS2467	Os_Chr04	24024384	24024409	(AT)13	(AT)13	(AT)13	(AT)13	(AT)13	(AT)13	(AT)13	(AT)6	(AT)13	(AT)13	(AT)6	-	Introns/Intergenic	-	-	TATCTGTTGGTGCAGTTGATCC	AGCTGCCCTTACTTGATATGTGG	459	A	A	A	RM17139
PMS2468	Os_Chr04	24030966	24030980	(GAC)5	(GAC)5	(GAC)5	(GAC)6	(GAC)5	(GAC)5	(GAC)5	(GAC)5	(GAC)5	(GAC)6	(GAC)5	-	Introns/Intergenic	-	-	CCTGACGAGGATGAAATCGT	GATGCAGTAGCAGCAGATGG	265	P	A	A	NA
PMS2469	Os_Chr04	24033140	24033160	(CGG)7	(CGG)7	(CGG)7	(CGG)7	(CGG)5	(CGG)7	(CGG)7	(CGG)7	(CGG)7	(CGG)7	(CGG)7	LOC_Os04g40440	CDS	expressed protein	CODON_CHANGE_PLUS_CODON_DELETION	AACCGAGCTTCAGAGCTGTAATGG	AAGGTGAGGAGCTGGAGCTTGG	151	P	A	A	RM17140

Polymorphic SSR (PMS) markers	Chromosomes	Start physical positions (bp)	End physical positions (bp)	Nipponbare	Kasalath	Nagina22	IR64	Pokkali	Bala	Tainung67	Azucena	Moroberekan	*Oryza rufipogon*	*Oryza nivara*	MSU gene locus IDs	Structural annotation	Functional annotation	SSR functional effects	Forward primers (5'-3')	Reverse primers (5'-3')	Amplified product size (bp)	Markers exhibiting polymorphism within *indica* rice	Transcrption factor genes-derived markers	Known cloned genes-derived markers	PMS markers corresponding with RM markers
PMS2470	Os_Chr04	24044171	24044202	(GA)16	(GA)16	(GA)16	(GA)15	(GA)11	(GA)16	(GA)16	(GA)16	(GA)16	(GA)16	(GA)16	-	Introns/Intergenic	-	-	TCTTTACCCAATCCTGCACC	ATATGGAAGCCGTCGTGAAG	100	P	A	A	NA
PMS2471	Os_Chr04	24178900	24178920	(GGC)7	(GGC)7	(GGC)7	(GGC)7	(GGC)7	(GGC)7	(GGC)7	(GGC)6	(GGC)7	(GGC)5	(GGC)7	LOC_Os04g40730	URR	oxidoreductase, short chain dehydrogenase/reductase family, putative, expressed	-	GTCCATTTCTGGAGACGGG	CGTCCCCTCCTCTCTCTCTC	167	A	A	A	NA
PMS2472	Os_Chr04	24201563	24201576	(AT)7	(AT)7	(AT)7	(AT)7	(AT)7	(AT)7	(AT)7	(AT)7	(AT)7	(AT)7	(AT)6	-	Introns/Intergenic	-	-	ATGCACGAATGACGTACCAA	CGCATGCTAACTTGAGACGA	268	A	A	A	NA
PMS2473	Os_Chr04	24225850	24225863	(GA)7	(GA)8	(GA)7	(GA)7	(GA)7	(GA)7	(GA)7	(GA)7	(GA)7	(GA)7	(GA)7	-	Introns/Intergenic	-	-	CCCTCTTCCCCTTCTCCAC	CTTTTTCTCTGCTCCCTCCC	279	P	A	A	NA
PMS2474	Os_Chr04	24241268	24241333	(TG)33	(TG)33	(TG)33	(TG)33	(TG)20	(TG)33	(TG)33	(TG)33	(TG)33	(TG)33	(TG)33	-	Introns/Intergenic	-	-	CACAAGCTGTTCCTGCTCTCG	ATATCTGACGCCAAGCACATCG	287	P	A	A	RM17148
PMS2475	Os_Chr04	24247689	24247726	(GA)19	(GA)19	(GA)19	(GA)19	(GA)8	(GA)19	(GA)19	(GA)19	(GA)19	(GA)19	(GA)19	LOC_Os04g40860	URR	transposon protein, putative, unclassified, expressed	-	GCAAGCAGCAAGAGCGAAGAGG	CTCAAGGCCGCCTCTCAAATCC	140	P	A	A	RM3785
PMS2476	Os_Chr04	24253503	24253520	(CAG)6	(CAG)7	(CAG)6	(CAG)6	(CAG)7	(CAG)6	(CAG)6	(CAG)6	(CAG)6	(CAG)6	(CAG)6	LOC_Os04g40870	CDS	alveolar soft part sarcoma chromosome region, candidate 1, putative, expressed	CODON_INSERTION	CGCACCAGATCACATACACC	TATGTCCAGCGATGAAGCAG	261	P	A	A	NA
PMS2477	Os_Chr04	24259464	24259484	(TGC)7	(TGC)8	(TGC)7	(TGC)7	(TGC)7	(TGC)7	(TGC)7	(TGC)7	(TGC)7	(TGC)7	(TGC)7	-	Introns/Intergenic	-	-	CACAGTTCAGTCATCCAGTGTGG	CAGGTTCCAGCACACAGATCG	87	P	A	A	RM17150
PMS2478	Os_Chr04	24275656	24275711	(TA)28	(TA)28	(TA)6	(TA)28	(TA)28	(TA)12	(TA)28	(TA)28	(TA)6	(TA)28	(TA)9	-	Introns/Intergenic	-	-	ATTGTGGAACGGAGGGAGTAGC	AGCTCCGTTCTTATTAGGTTGTGG	361	P	A	A	RM17152
PMS2479	Os_Chr04	24312822	24312842	(CCT)7	(CCT)6	(CCT)7	(CCT)7	(CCT)7	(CCT)7	(CCT)7	(CCT)7	(CCT)7	(CCT)6	(CCT)7	LOC_Os04g40970	CDS	OsDBH	CODON_DELETION	ATCCCTCCTCTCCGACGTCTCC	GTGGGCTTGAGCTTGGGTTTGG	141	P	A	P	RM17156
PMS2480	Os_Chr04	24371912	24371929	(CCG)6	(CCG)6	(CCG)6	(CCG)5	(CCG)6	(CCG)6	(CCG)6	(CCG)6	(CCG)6	(CCG)6	(CCG)6	LOC_Os04g41100	URR	cyclin-dependent kinase G-2, putative, expressed	-	CTTCCTCTCCCTACCTCGCT	AAGCCAAACCCTAGATCCGT	204	P	A	A	NA
PMS2481	Os_Chr04	24608415	24608426	(AT)6	(AT)6	(AT)6	(AT)6	(AT)6	(AT)6	(AT)6	(AT)6	(AT)6	(AT)7	(AT)6	-	Introns/Intergenic	-	-	TATACAGTCGGCGTTCCAAA	GACTTCAAAAACCAAACGAAAAA	233	A	A	A	NA
PMS2482	Os_Chr04	24658943	24658963	(TGG)7	(TGG)7	(TGG)7	(TGG)7	(TGG)7	(TGG)7	(TGG)7	(TGG)7	(TGG)7	(TGG)6	(TGG)7	-	Introns/Intergenic	-	-	GGGAGAGGTGTGGAACAAAA	GCGGTTTAGGTGAAGAGCTG	248	A	A	A	NA

Polymorphic SSR (PMS) markers	Chromosomes	Start physical positions (bp)	End physical positions (bp)	Nipponbare	Kasalath	Nagina22	IR64	Pokkali	Bala	Tainung67	Azucena	Moroberekan	*Oryza rufipogon*	*Oryza nivara*	MSU gene locus IDs	Structural annotation	Functional annotation	SSR functional effects	Forward primers (5'-3')	Reverse primers (5'-3')	Amplified product size (bp)	Markers exhibiting polymorphism within *indica* rice	Transcrption factor genes-derived markers	Known cloned genes-derived markers	PMS markers corresponding with RM markers
PMS2483	Os_Chr04	24659527	24659552	(CT)13	(CT)13	(CT)11	(CT)13	(CT)13	(CT)11	(CT)13	(CT)13	(CT)11	(CT)13	(CT)13	-	Introns/Intergenic	-	-	GGACGAACACCATGCATACTCC	TTAGGCTGTTGACCTGTTGTTAGC	192	P	A	A	RM17169
PMS2484	Os_Chr04	24662443	24662462	(TA)10	(TA)7	(TA)10	(TA)10	(TA)10	(TA)10	(TA)10	(TA)10	(TA)10	(TA)10	(TA)10	-	Introns/Intergenic	-	-	CCACAACGCATACACATACAACG	TAGATTTACCGTGTCCCAGTTGC	403	P	A	A	RM17170
PMS2485	Os_Chr04	24779655	24779668	(TC)7	(TC)7	(TC)6	(TC)7	(TC)7	(TC)7	(TC)6	(TC)7	(TC)7	(TC)7	(TC)7	-	Introns/Intergenic	-	-	TCTTCTCTTTCCCGCTACGA	AAGCATCCACAGGGAGAAAA	199	P	A	A	NA
PMS2486	Os_Chr04	24783963	24783982	(AT)10	(AT)10	(AT)10	(AT)10	(AT)10	(AT)10	(AT)7	(AT)10	(AT)10	(AT)10	(AT)10	-	Introns/Intergenic	-	-	CTCGCGAGCTTTACCGAGTCTGG	CCGAGCTCAAGTCAAGCCTATTGC	323	A	A	A	RM17175
PMS2487	Os_Chr04	24899671	24899691	(GCG)7	(GCG)8	(GCG)7	(GCG)7	(GCG)7	(GCG)7	(GCG)7	(GCG)7	(GCG)7	(GCG)7	(GCG)7	-	Introns/Intergenic	-	-	GGAGACAACTCTGGTCTTGACAGC	GGACCTCGCGTAGTTGAAGTCG	157	P	A	A	RM17180
PMS2488	Os_Chr04	24902047	24902070	(AT)12	(AT)12	(AT)12	(AT)6	(AT)12	(AT)12	(AT)12	(AT)12	(AT)12	(AT)12	(AT)12	-	Introns/Intergenic	-	-	CACCATTGTGTTCGCTACAA	TCGTGACAAAGTTACTTCCGTTT	268	P	A	A	NA
PMS2489	Os_Chr04	24905303	24905323	(AGA)7	(AGA)7	(AGA)7	(AGA)7	(AGA)7	(AGA)7	(AGA)6	(AGA)7	(AGA)7	(AGA)7	(AGA)7	LOC_Os04g42050	CDS	expressed protein	CODON_CHANGE_PLUS_CODON_DELETION	TGATGATGAGGAGGATGATGTCC	GTGGAAAGAAAGGGAGCTCAGG	172	A	A	A	RM17183
PMS2490	Os_Chr04	24906952	24906967	(AC)8	(AC)7	(AC)8	(AC)8	(AC)8	(AC)8	(AC)8	(AC)8	(AC)8	(AC)8	(AC)8	-	Introns/Intergenic	-	-	GTCCTGCAAATGTCCCATTC	ACTGTACCGCATTCTCTCGC	190	P	A	A	NA
PMS2491	Os_Chr04	24932322	24932342	(GCG)7	(GCG)7	(GCG)7	(GCG)7	(GCG)7	(GCG)7	(GCG)6	(GCG)7	(GCG)7	(GCG)7	(GCG)7	LOC_Os04g42110	CDS	cell division control protein 48 homolog B, putative, expressed	CODON_CHANGE_PLUS_CODON_DELETION	GACGAGGCGAATGGAAGAGGTTAGG	GTGCGTAGAGGACAGGATACATCACG	179	A	A	A	RM17185
PMS2492	Os_Chr04	25175449	25175466	(GCG)6	(GCG)7	(GCG)6	(GCG)6	(GCG)6	(GCG)6	(GCG)6	(GCG)6	(GCG)6	(GCG)7	(GCG)6	LOC_Os04g42540	CDS	expressed protein	CODON_INSERTION	GTGGAGGTTCAGGAATGAGC	CTTCGACTACTCCCAGAGCG	232	P	A	A	NA
PMS2493	Os_Chr04	25186098	25186115	(CGG)6	(CGG)6	(CGG)6	(CGG)6	(CGG)6	(CGG)6	(CGG)6	(CGG)5	(CGG)6	(CGG)6	(CGG)6	LOC_Os04g42570	CDS	AP2/EREBP transcription factor BABY BOOM, putative, expressed	CODON_DELETION	CCACCCACAACTTCTTCCAG	GTCGTACCCCACGACGAG	182	A	P	A	NA
PMS2494	Os_Chr04	25187914	25187931	(CCG)6	(CCG)5	(CCG)6	(CCG)6	(CCG)6	(CCG)6	(CCG)6	(CCG)6	(CCG)6	(CCG)5	(CCG)5	LOC_Os04g42580	CDS	carboxyvinyl-carboxyphosphonate phosphorylmutase, putative, expressed	CODON_DELETION	AAAACGGCAGTGAGGCTCTA	CAGGCGTAACCGTATCAACC	224	P	A	A	NA
PMS2495	Os_Chr04	25349161	25349178	(TA)9	(TA)7	(TA)6	(TA)6	(TA)7	(TA)7	(TA)9	(TA)9	(TA)9	(TA)9	(TA)7	-	Introns/Intergenic	-	-	GTTAAGGCCTCATCGTTTGG	AGGCGACAAAGGACTATGGA	240	P	A	A	NA

Polymorphic SSR (PMS) markers	Chromosomes	Start physical positions (bp)	End physical positions (bp)	Nipponbare	Kasalath	Nagina22	IR64	Pokkali	Bala	Tainung67	Azucena	Moroberekan	*Oryza rufipogon*	*Oryza nivara*	MSU gene locus IDs	Structural annotation	Functional annotation	SSR functional effects	Forward primers (5'-3')	Reverse primers (5'-3')	Amplified product size (bp)	Markers exhibiting polymorphism within *indica* rice	Transcrption factor genes-derived markers	Known cloned genes-derived markers	PMS markers corresponding with RM markers
PMS2496	Os_Chr04	25405685	25405702	(GAC)6	(GAC)6	(GAC)6	(GAC)6	(GAC)6	(GAC)6	(GAC)5	(GAC)6	(GAC)6	(GAC)6	(GAC)6	LOC_Os04g42950	CDS	MYB family transcription factor, putative, expressed	CODON_DELETION	TAGTCCTCGCTGGTAGAGGC	CGCAGAGCAAGGACATCAT	277	A	P	A	NA
PMS2497	Os_Chr04	25429187	25429224	(TC)19	(TC)17	(TC)19	(TC)19	(TC)19	(TC)19	(TC)19	(TC)19	(TC)19	(TC)19	(TC)19	LOC_Os04g42980	URR	zinc finger family protein, putative, expressed	-	GTTTGGCTGCGGCTTCTATCTCC	CTTCTCTCCCACGAGGTCAAACC	82	P	A	A	RM17215
PMS2498	Os_Chr04	25651923	25651940	(CT)9	(CT)6	(CT)9	(CT)9	(CT)9	(CT)6	(CT)9	(CT)8	(CT)9	(CT)6	(CT)9	-	Introns/Intergenic	-	-	GAGTTCAATTCCGAACCCAA	GAAAGGAGGGCTCGGCTC	258	P	A	A	NA
PMS2499	Os_Chr04	25672536	25672549	(GA)7	(GA)7	(GA)7	(GA)7	(GA)7	(GA)7	(GA)7	(GA)7	(GA)7	(GA)7	(GA)6	-	Introns/Intergenic	-	-	CTTGAAAAGCCACCACGTTT	ATCATCGATCATTCGATCCC	238	A	A	A	NA
PMS2500	Os_Chr04	25687652	25687679	(TA)14	(TA)14	(TA)14	(TA)14	(TA)9	(TA)14	(TA)14	(TA)14	(TA)14	(TA)14	(TA)14	-	Introns/Intergenic	-	-	TGAACACATCCCAATTGCTT	GGAGAAGAAAACTCCGACCC	165	P	A	A	NA
PMS2501	Os_Chr04	25692610	25692621	(GA)6	(GA)6	(GA)6	(GA)6	(GA)6	(GA)6	(GA)6	(GA)6	(GA)7	(GA)6	(GA)6	-	Introns/Intergenic	-	-	GGGAGAGGGAGAGAGGGAG	TGCTTGTCGTCTAGTGTCCG	245	A	A	A	NA
PMS2502	Os_Chr04	25693048	25693069	(TA)11	(TA)11	(TA)7	(TA)11	(TA)11	(TA)7	(TA)11	(TA)6	(TA)11	(TA)11	(TA)11	-	Introns/Intergenic	-	-	GCTACGGCGCTGAAATACTCC	GTCCAAACATCCAATGTGAGTCC	363	P	A	A	RM17227
PMS2503	Os_Chr04	25772640	25772663	(GA)12	(GA)12	(GA)12	(GA)12	(GA)12	(GA)12	(GA)12	(GA)12	(GA)12	(GA)12	(GA)9	-	Introns/Intergenic	-	-	AATGGCAACAACGAGACTAGG	GTCTCTGAATCCTAACCCTAACC	91	A	A	A	RM17231
PMS2504	Os_Chr04	25846430	25846445	(AT)8	(AT)8	(AT)8	(AT)8	(AT)8	(AT)8	(AT)8	(AT)8	(AT)8	(AT)8	(AT)7	-	Introns/Intergenic	-	-	TTTATGTGGGGCTCTGAAGG	TTTCGTAGGCGGATATTTGG	257	A	A	A	NA
PMS2505	Os_Chr04	26299084	26299129	(AG)23	(AG)22	(AG)23	(AG)23	(AG)23	(AG)22	(AG)23	(AG)22	(AG)23	(AG)23	(AG)23	-	Introns/Intergenic	-	-	TCGTTGGTCCAAGCTCACACG	GGCCGGCAAAGGTATCTGTCG	165	P	A	A	RM1354
PMS2506	Os_Chr04	26428131	26428148	(TC)9	(TC)9	(TC)9	(TC)9	(TC)9	(TC)9	(TC)9	(TC)9	(TC)9	(TC)9	(TC)6	-	Introns/Intergenic	-	-	GTGGCCATATCTGGTCATCG	GTATGGAGCTCGTGGGTACG	240	A	A	A	NA
PMS2507	Os_Chr04	26441901	26441920	(AT)10	(AT)10	(AT)10	(AT)10	(AT)7	(AT)10	(AT)10	(AT)10	(AT)8	(AT)10	(AT)10	-	Introns/Intergenic	-	-	AATCGCGGTTATGAAAGGCTACG	GTGGTTTGATGCTGTTTGCTTCC	187	P	A	A	RM17274
PMS2508	Os_Chr04	26528095	26528124	(CCT)10	(CCT)9	(CCT)10	(CCT)10	(CCT)10	(CCT)10	(CCT)10	(CCT)10	(CCT)10	(CCT)9	(CCT)10	LOC_Os04g44800	CDS	expressed protein	CODON_DELETION	CTGAGTCTGTCTCGCTCTCTGACC	CCGCAGCAAGTCCATAGAAGC	314	P	A	A	RM17278

Polymorphic SSR (PMS) markers	Chromosomes	Start physical positions (bp)	End physical positions (bp)	Nipponbare	Kasalath	Nagina22	IR64	Pokkali	Bala	Tainung67	Azucena	Moroberekan	*Oryza rufipogon*	*Oryza nivara*	MSU gene locus IDs	Structural annotation	Functional annotation	SSR functional effects	Forward primers (5'-3')	Reverse primers (5'-3')	Amplified product size (bp)	Markers exhibiting polymorphism within *indica* rice	Transcrption factor genes-derived markers	Known cloned genes-derived markers	PMS markers corresponding with RM markers
PMS2509	Os_Chr04	26594066	26594099	(TA)17	(TA)17	(TA)17	(TA)17	(TA)17	(TA)6	(TA)17	(TA)17	(TA)17	(TA)12	(TA)17	-	Introns/Intergenic	-	-	CTCAATGAATGTGGGCAATG	AAAATAAATAATATTGGAAGAAGAGCA	258	P	A	A	NA
PMS2510	Os_Chr04	26634298	26634319	(TA)11	(TA)11	(TA)11	(TA)8	(TA)11	(TA)11	(TA)11	(TA)11	(TA)6	(TA)7	(TA)11	-	Introns/Intergenic	-	-	GTACGCGTGGCAGTTCAGTCAGC	CATACGCGTAGCAGTCCAGTCAGC	77	P	A	A	RM17281
PMS2511	Os_Chr04	26758443	26758468	(CA)13	(CA)13	(CA)13	(CA)13	(CA)13	(CA)13	(CA)13	(CA)13	(CA)13	(CA)13	(CA)14	-	Introns/Intergenic	-	-	ACTAGCCACAGCCCATCTGTTCC	CCCGCTCAAATTCTTCACAATCC	152	A	A	A	RM3125
PMS2512	Os_Chr04	26774606	26774625	(CT)10	(CT)10	(CT)10	(CT)10	(CT)10	(CT)10	(CT)10	(CT)10	(CT)10	(CT)10	(CT)8	-	Introns/Intergenic	-	-	TCCAAATCATATCCATCCGC	GAGAGGGAGGACTCGGCTT	241	A	A	A	NA
PMS2513	Os_Chr04	26959695	26959715	(CCG)7	(CCG)7	(CCG)7	(CCG)7	(CCG)7	(CCG)7	(CCG)7	(CCG)7	(CCG)7	(CCG)7	(CCG)5	LOC_Os04g45570	CDS	expressed protein	CODON_DELETION	GCACACCACCGTCCATCTCTCC	GTGGACATCCTCCTGGCTCACG	90	A	A	A	RM17293
PMS2514	Os_Chr04	27217302	27217317	(GC)8	(GC)7	(GC)8	(GC)7	(GC)8	(GC)8	(GC)8	(GC)8	(GC)8	(GC)8	(GC)8	-	Introns/Intergenic	-	-	CAGCACGTACTCCATCAGGA	GTAACGTTCGGTGTGTGTGC	243	P	A	A	NA
PMS2515	Os_Chr04	27241513	27241530	(GGC)6	(GGC)6	(GGC)6	(GGC)6	(GGC)6	(GGC)6	(GGC)5	(GGC)6	(GGC)6	(GGC)6	(GGC)6	LOC_Os04g45980	CDS	glycine-rich cell wall protein, putative, expressed	CODON_DELETION	GAGGTGGAGGAGGCGTTG	AAAATGTCCAGCGGTTTCAG	245	A	A	A	NA
PMS2516	Os_Chr04	27274504	27274539	(CT)18	(CT)18	(CT)18	(CT)18	(CT)9	(CT)18	(CT)18	(CT)18	(CT)18	(CT)18	(CT)18	-	Introns/Intergenic	-	-	TAGCGATTGGATGGAGGCTGAGG	GGTCGCCTCGCCATTAGTTACG	119	P	A	A	RM17303
PMS2517	Os_Chr04	27443315	27443326	(TA)6	(TA)6	(TA)6	(TA)6	(TA)6	(TA)6	(TA)6	(TA)6	(TA)6	(TA)7	(TA)6	-	Introns/Intergenic	-	-	ATCCACTTTTGGGGCCTATT	TTAGAACCCTCGAAGCCTCC	224	A	A	A	NA
PMS2518	Os_Chr04	27485280	27485301	(TA)11	(TA)11	(TA)11	(TA)11	(TA)11	(TA)11	(TA)11	(TA)11	(TA)7	(TA)11	(TA)11	-	Introns/Intergenic	-	-	CATATGATGCTCTGCAACACAAGC	TAGCTCGCGATATGATTCTGACG	191	A	A	A	RM17311
PMS2519	Os_Chr04	27519680	27519697	(GCA)6	(GCA)6	(GCA)6	(GCA)6	(GCA)6	(GCA)6	(GCA)6	(GCA)6	(GCA)6	(GCA)6	(GCA)5	-	Introns/Intergenic	-	-	TGGCTCGCCATCTATACCTT	TAACCGTACATGCGTGTGGT	278	A	A	A	NA
PMS2520	Os_Chr04	27648334	27648357	(CGC)8	(CGC)8	(CGC)5	(CGC)8	(CGC)8	(CGC)8	(CGC)8	(CGC)8	(CGC)8	(CGC)8	(CGC)8	-	Introns/Intergenic	-	-	GAGGTCCCTCGGTGGTTTAGAAGG	GGCTGTTGCGTCGGTGTAGAGG	355	P	A	A	RM6590
PMS2521	Os_Chr04	27704151	27704220	(TA)35	(TA)35	(TA)6	(TA)10	(TA)35	(TA)17	(TA)6	(TA)35	(TA)35	(TA)13	(TA)35	-	Introns/Intergenic	-	-	CCCTTGAAGGTCCAACCATAGG	TGGGTGTTCCATTGAACTTGACC	349	P	A	A	RM17318

**Polymorphic SSR (PMS) markers**	**Chromosomes**	**Start physical positions (bp)**	**End physical positions (bp)**	**Nipponbare**	**Kasalath**	**Nagina22**	**IR64**	**Pokkali**	**Bala**	**Tainung67**	**Azucena**	**Moroberekan**	***Oryza rufipogon***	***Oryza nivara***	**MSU gene locus IDs**	**Structural annotation**	**Functional annotation**	**SSR functional effects**	**Forward primers (5'-3')**	**Reverse primers (5'-3')**	**Amplified product size (bp)**	**Markers exhibiting polymorphism within *indica* rice**	**Transcrption factor genes-derived markers**	**Known cloned genes-derived markers**	**PMS markers corresponding with RM markers**
PMS2522	Os_Chr04	27724734	27724751	(CCG)6	(CCG)7	(CCG)6	(CCG)6	(CCG)6	(CCG)7	(CCG)6	(CCG)6	(CCG)6	(CCG)6	(CCG)6	LOC_Os04g46750	URR	glycosyl transferase 8 domain containing protein, putative, expressed	-	CATGTACATCATCGCCGGTA	GTAGGCAGATCTGGGCGTAG	205	P	A	A	NA
PMS2523	Os_Chr04	27745950	27745961	(GA)6	(GA)7	(GA)7	(GA)7	(GA)6	(GA)7	(GA)6	(GA)6	(GA)6	(GA)6	(GA)7	-	Introns/Intergenic	-	-	CATTTGCTAGCTGACTGCCA	CATGAGCTCCAACCGTTCTT	238	P	A	A	NA
PMS2524	Os_Chr04	27764588	27764599	(AG)6	(AG)6	(AG)6	(AG)6	(AG)6	(AG)6	(AG)6	(AG)6	(AG)6	(AG)7	(AG)7	-	Introns/Intergenic	-	-	TAGGCAGAGATGGGGAAAGA	GGAACAGCGAAGATGGAGAG	236	A	A	A	NA
PMS2525	Os_Chr04	27765896	27765913	(GCG)6	(GCG)6	(GCG)6	(GCG)6	(GCG)6	(GCG)6	(GCG)5	(GCG)6	(GCG)6	(GCG)6	(GCG)6	LOC_Os04g46860	CDS	scarecrow, putative, expressed	CODON_DELETION	TACATAGCGCGGTACCCTTC	GGGCGTCCTTTAGGTAGGAG	243	A	P	A	NA
PMS2526	Os_Chr04	27931463	27931478	(AG)8	(AG)8	(AG)8	(AG)9	(AG)8	(AG)8	(AG)8	(AG)8	(AG)8	(AG)8	(AG)8	-	Introns/Intergenic	-	-	AATAGACGCGCTCTTTGCAT	CTCATGGCATCGTCCCTTAT	214	P	A	A	NA
PMS2527	Os_Chr04	27978540	27978571	(GA)16	(GA)16	(GA)16	(GA)16	(GA)7	(GA)16	(GA)16	(GA)16	(GA)16	(GA)16	(GA)16	-	Introns/Intergenic	-	-	GGGGAGAGGGAGAGCGAG	ATCCCGAATCCAAATCCTTC	211	P	A	A	NA
PMS2528	Os_Chr04	28006641	28006652	(GA)6	(GA)9	(GA)6	(GA)6	(GA)6	(GA)6	(GA)6	(GA)6	(GA)6	(GA)9	(GA)6	-	Introns/Intergenic	-	-	GAAATTACCGGAGGGGAGAG	AGAACGTATTTGGCGGTCAG	189	P	A	A	NA
PMS2529	Os_Chr04	28092278	28092295	(CGA)6	(CGA)6	(CGA)6	(CGA)6	(CGA)6	(CGA)6	(CGA)5	(CGA)6	(CGA)6	(CGA)6	(CGA)6	LOC_Os04g47320	CDS	uncharacterized mscS family protein, putative, expressed	CODON_DELETION	AAGTCGTTCGTCGGCAAG	GACCCGAGAGCTTCTTGTTG	280	A	A	A	NA
PMS2530	Os_Chr04	28110145	28110162	(TTG)6	(TTG)6	(TTG)5	(TTG)6	(TTG)6	(TTG)6	(TTG)6	(TTG)6	(TTG)6	(TTG)6	(TTG)6	-	Introns/Intergenic	-	-	AGCAGAGTCCGAGAGAGACG	AGCAACACGTCAGGTTCCTC	108	P	A	A	NA
PMS2531	Os_Chr04	28175909	28175932	(TA)12	(TA)12	(TA)12	(TA)7	(TA)12	(TA)12	(TA)12	(TA)12	(TA)12	(TA)12	(TA)12	-	Introns/Intergenic	-	-	GTCTCTCTTCTCTACGGCAAT	CATACGCATATGCAAACTT	267	P	A	A	NA
PMS2532	Os_Chr04	28189528	28189539	(AG)6	(AG)6	(AG)6	(AG)6	(AG)7	(AG)6	(AG)6	(AG)6	(AG)6	(AG)6	(AG)6	-	Introns/Intergenic	-	-	GACATTGCACAGGTGTCGTC	AGCAAGCCTGTGTTACCCAC	191	P	A	A	NA
PMS2533	Os_Chr04	28264890	28264907	(AAG)6	(AAG)6	(AAG)6	(AAG)6	(AAG)6	(AAG)6	(AAG)6	(AAG)6	(AAG)6	(AAG)6	(AAG)5	-	Introns/Intergenic	-	-	CAGCGAGAAAGGAAGTGGTC	CAAGCTGATCCGGAAATGAT	238	A	A	A	NA
PMS2534	Os_Chr04	28305036	28305056	(CCA)7	(CCA)7	(CCA)7	(CCA)7	(CCA)7	(CCA)7	(CCA)7	(CCA)7	(CCA)7	(CCA)6	(CCA)7	-	Introns/Intergenic	-	-	TTACCTTCTTGGAGGCCTTCTCC	GGATTCTTTGGTTGGTGTTCTTCG	97	A	A	A	RM17340

Polymorphic SSR (PMS) markers	Chromosomes	Start physical positions (bp)	End physical positions (bp)	Nipponbare	Kasalath	Nagina22	IR64	Pokkali	Bala	Tainung67	Azucena	Moroberekan	*Oryza rufipogon*	*Oryza nivara*	MSU gene locus IDs	Structural annotation	Functional annotation	SSR functional effects	Forward primers (5'-3')	Reverse primers (5'-3')	Amplified product size (bp)	Markers exhibiting polymorphism within *indica* rice	Transcrption factor genes-derived markers	Known cloned genes-derived markers	PMS markers corresponding with RM markers
PMS2535	Os_Chr04	28311026	28311046	(GGC)7	(GGC)7	(GGC)7	(GGC)7	(GGC)7	(GGC)7	(GGC)7	(GGC)6	(GGC)7	(GGC)7	(GGC)7	LOC_Os04g47710	CDS	retrotransposon protein, putative, unclassified, expressed	CODON_DELETION	CGGCATTAGTCGCGAGAT	ACCAGTATAGGTGACCCGCA	229	A	A	A	NA
PMS2536	Os_Chr04	28401967	28401984	(CT)9	(CT)9	(CT)9	(CT)9	(CT)9	(CT)9	(CT)9	(CT)9	(CT)9	(CT)7	(CT)9	-	Introns/Intergenic	-	-	CCACTGCCTTGCTCTCCTAC	GGCCTGTTCCAAGGACATAA	184	A	A	A	NA
PMS2537	Os_Chr04	28403309	28403392	(TA)42	(TA)42	(TA)42	(TA)42	(TA)8	(TA)6	(TA)42	(TA)11	(TA)42	(TA)11	(TA)42	-	Introns/Intergenic	-	-	TTTAGCTGTTTCGCACACACG	AAGGACATCCTTCATCCTCTTGC	225	P	A	A	RM17345
PMS2538	Os_Chr04	28414469	28414483	(GCG)5	(GCG)5	(GCG)5	(GCG)5	(GCG)5	(GCG)5	(GCG)5	(GCG)5	(GCG)5	(GCG)6	(GCG)5	LOC_Os04g47860	CDS	OsIDD10	CODON_CHANGE_PLUS_CODON_INSERTION	GCTCGACCACCTGCTGTC	AAGCCGAGGTTGAAGAGGTT	243	A	P	P	NA
PMS2539	Os_Chr04	28571427	28571450	(GAT)8	(GAT)8	(GAT)8	(GAT)6	(GAT)8	(GAT)8	(GAT)8	(GAT)8	(GAT)8	(GAT)8	(GAT)8	-	Introns/Intergenic	-	-	TGCTCTGCATTCCGTATCAT	GCCAACGCCTAATAACTGGA	203	P	A	A	NA
PMS2540	Os_Chr04	28606120	28606133	(AC)7	(AC)7	(AC)7	(AC)7	(AC)7	(AC)7	(AC)7	(AC)7	(AC)7	(AC)7	(AC)6	-	Introns/Intergenic	-	-	GACCGCAGCTTATCGTTCTC	ATGGTGCAGTGCAGTTCTTG	131	A	A	A	NA
PMS2541	Os_Chr04	28716400	28716421	(AT)11	(AT)11	(AT)11	(AT)8	(AT)11	(AT)11	(AT)11	(AT)11	(AT)11	(AT)11	(AT)11	-	Introns/Intergenic	-	-	ATCCTGCATGTTACACTCAAGC	TATTTATCCGGATCCTCTCACC	430	P	A	A	RM17360
PMS2542	Os_Chr04	28758977	28759014	(AT)19	(AT)19	(AT)8	(AT)19	(AT)19	(AT)6	(AT)19	(AT)19	(AT)9	(AT)19	(AT)19	-	Introns/Intergenic	-	-	GCACCTAAATTATAAATGGGGGA	TGCGTAAAGTTGTGGGGAG	280	P	A	A	NA
PMS2543	Os_Chr04	29096784	29096795	(TA)6	(TA)6	(TA)6	(TA)7	(TA)7	(TA)6	(TA)6	(TA)6	(TA)6	(TA)7	(TA)6	-	Introns/Intergenic	-	-	CACGATTAACCATCGTGAGCTACC	ATCGGGTGGTGAGCCTTTCC	237	P	A	A	RM16546
PMS2544	Os_Chr04	29198897	29198910	(CT)7	(CT)7	(CT)7	(CT)7	(CT)7	(CT)7	(CT)6	(CT)7	(CT)7	(CT)7	(CT)7	-	Introns/Intergenic	-	-	TTTACCCTGGTTTTTGCCAC	GAGAGGAGAACGAAGAGGGC	112	A	A	A	NA
PMS2545	Os_Chr04	29221687	29221726	(TA)20	(TA)20	(TA)20	(TA)7	(TA)20	(TA)20	(TA)7	(TA)9	(TA)20	(TA)7	(TA)11	-	Introns/Intergenic	-	-	TCGGACTCAGCACTACGTTACCC	GAGCACGTAATGGAATCTGATGG	197	P	A	A	RM17379
PMS2546	Os_Chr04	29312813	29312824	(TA)6	(TA)7	(TA)6	(TA)6	(TA)6	(TA)6	(TA)6	(TA)6	(TA)6	(TA)6	(TA)6	-	Introns/Intergenic	-	-	GCCAATTAGCACCCTAGCTG	ATTTGCACGGTTCTTTCAAT	124	P	A	A	NA
PMS2547	Os_Chr04	29377650	29377665	(TA)8	(TA)8	(TA)8	(TA)8	(TA)8	(TA)8	(TA)6	(TA)8	(TA)8	(TA)8	(TA)8	-	Introns/Intergenic	-	-	TGGATCCTTGCATCTCTTTTT	TTGACTAAGGTGGGAGGTGG	228	A	A	A	NA

Polymorphic SSR (PMS) markers	Chromosomes	Start physical positions (bp)	End physical positions (bp)	Nipponbare	Kasalath	Nagina22	IR64	Pokkali	Bala	Tainung67	Azucena	Moroberekan	*Oryza rufipogon*	*Oryza nivara*	MSU gene locus IDs	Structural annotation	Functional annotation	SSR functional effects	Forward primers (5'-3')	Reverse primers (5'-3')	Amplified product size (bp)	Markers exhibiting polymorphism within *indica* rice	Transcrption factor genes-derived markers	Known cloned genes-derived markers	PMS markers corresponding with RM markers
PMS2548	Os_Chr04	29429982	29430005	(AG)12	(AG)10	(AG)12	(AG)12	(AG)12	(AG)12	(AG)12	(AG)12	(AG)12	(AG)12	(AG)12	-	Introns/Intergenic	-	-	GGAGATATCAACCGGCAGAA	ATCATGGGGAAGTGCAAGAG	118	P	A	A	NA
PMS2549	Os_Chr04	29548501	29548576	(TA)38	(TA)38	(TA)6	(TA)38	(TA)8	(TA)38	(TA)38	(TA)38	(TA)38	(TA)38	(TA)38	-	Introns/Intergenic	-	-	ACTATGCCGTGTGGGTATGTGG	GTGTACTGGTGAGCTGAGAGTTCG	644	P	A	A	RM17393
PMS2550	Os_Chr04	29564777	29564788	(AT)6	(AT)6	(AT)6	(AT)6	(AT)12	(AT)6	(AT)6	(AT)6	(AT)6	(AT)6	(AT)6	-	Introns/Intergenic	-	-	TTTGATTGTTCGTCGGCATA	TGCTAACTCCGTTTCACAACA	247	P	A	A	NA
PMS2551	Os_Chr04	29606671	29606688	(GGT)6	(GGT)6	(GGT)5	(GGT)6	(GGT)6	(GGT)6	(GGT)6	(GGT)6	(GGT)6	(GGT)6	(GGT)6	LOC_Os04g49650	CDS	DUF581 domain containing protein, expressed	CODON_DELETION	GCACCAGAAGTCCCGAAAAT	CAGCAGATCGAGGTGGACAG	263	P	A	A	NA
PMS2552	Os_Chr04	29608548	29608561	(CT)7	(CT)7	(CT)7	(CT)7	(CT)7	(CT)7	(CT)7	(CT)8	(CT)7	(CT)7	(CT)7	-	Introns/Intergenic	-	-	CCAAAACGCGATGTGTGTAT	GGAACAATGATGCAGGCAC	203	A	A	A	NA
PMS2553	Os_Chr04	29641174	29641200	(CGC)9	(CGC)9	(CGC)9	(CGC)8	(CGC)9	(CGC)9	(CGC)9	(CGC)9	(CGC)9	(CGC)9	(CGC)9	LOC_Os04g49700	CDS	zinc finger, C3HC4 type domain containing protein, expressed	CODON_CHANGE_PLUS_CODON_DELETION	GTTCTACTAGCGTCGGTCTCGATCC	CTCGACGTTGTGCACCTCACG	117	P	A	A	RM6148
PMS2554	Os_Chr04	29751933	29751948	(TG)8	(TG)8	(TG)8	(TG)9	(TG)8	(TG)8	(TG)8	(TG)8	(TG)8	(TG)8	(TG)8	-	Introns/Intergenic	-	-	CCAATGTGGCTCACACTCAC	GCGAAGCTGAATCAACAACA	155	P	A	A	NA
PMS2555	Os_Chr04	29836122	29836139	(AT)9	(AT)9	(AT)9	(AT)9	(AT)9	(AT)9	(AT)9	(AT)9	(AT)9	(AT)9	(AT)7	-	Introns/Intergenic	-	-	TCAATTTCAACAATGGTGCC	GATCCACGACGTCTGTCCTT	249	A	A	A	NA
PMS2556	Os_Chr04	29883424	29883439	(AG)8	(AG)8	(AG)8	(AG)8	(AG)8	(AG)9	(AG)8	(AG)8	(AG)8	(AG)8	(AG)8	LOC_Os04g50100	DRR	RING-H2 finger protein ATL5G, putative, expressed	-	AATGCCAATGCGTAGAAACC	AATCGATCCTCTGCAGTGCT	233	P	A	A	NA
PMS2557	Os_Chr04	29894350	29894391	(GA)21	(GA)21	(GA)18	(GA)21	(GA)21	(GA)21	(GA)21	(GA)21	(GA)21	(GA)21	(GA)21	-	Introns/Intergenic	-	-	ACCTCACCTTTCCCTCGATTGG	GTTGGTTGCTTCCTCCCATACG	272	P	A	A	RM3474
PMS2558	Os_Chr04	29897817	29897830	(CT)7	(CT)7	(CT)7	(CT)7	(CT)6	(CT)7	(CT)7	(CT)7	(CT)7	(CT)7	(CT)7	-	Introns/Intergenic	-	-	CGGATGCTTCCTTTCACATT	TGTATACGTCGCCGTGTCTC	205	P	A	A	NA
PMS2559	Os_Chr04	29911516	29911529	(CG)7	(CG)6	(CG)7	(CG)7	(CG)7	(CG)7	(CG)7	(CG)7	(CG)7	(CG)7	(CG)7	-	Introns/Intergenic	-	-	GTGCTAGCGAGTTACCAGGC	AAAAGTAAAGATTGGCGGGG	273	P	A	A	NA
PMS2560	Os_Chr04	29918642	29918657	(CT)8	(CT)8	(CT)8	(CT)8	(CT)6	(CT)8	(CT)8	(CT)8	(CT)8	(CT)8	(CT)8	-	Introns/Intergenic	-	-	GGCCATTAGGCTGAACAAGA	TGTATACGTCGCCGTGTCTC	246	P	A	A	NA

Polymorphic SSR (PMS) markers	Chromosomes	Start physical positions (bp)	End physical positions (bp)	Nipponbare	Kasalath	Nagina22	IR64	Pokkali	Bala	Tainung67	Azucena	Moroberekan	*Oryza rufipogon*	*Oryza nivara*	MSU gene locus IDs	Structural annotation	Functional annotation	SSR functional effects	Forward primers (5'-3')	Reverse primers (5'-3')	Amplified product size (bp)	Markers exhibiting polymorphism within *indica* rice	Transcrption factor genes-derived markers	Known cloned genes-derived markers	PMS markers corresponding with RM markers
PMS2561	Os_Chr04	29973272	29973285	(CT)7	(CT)7	(CT)7	(CT)7	(CT)7	(CT)7	(CT)7	(CT)7	(CT)8	(CT)7	(CT)7	-	Introns/Intergenic	-	-	TCCCCTCTTCTCTCCTTTCC	CTTCATCGACCTCTTACGCC	180	A	A	A	NA
PMS2562	Os_Chr04	29989920	29989943	(CT)12	(CT)11	(CT)12	(CT)12	(CT)12	(CT)12	(CT)12	(CT)10	(CT)12	(CT)12	(CT)12	-	Introns/Intergenic	-	-	CGGTATGTAGCATTCCCGGTTACG	GCCACTGCCACCATGAACTGG	519	P	A	A	RM17412
PMS2563	Os_Chr04	30028691	30028710	(TA)10	(TA)10	(TA)10	(TA)9	(TA)10	(TA)10	(TA)10	(TA)10	(TA)10	(TA)10	(TA)10	-	Introns/Intergenic	-	-	TGGCATCTGGGATATGGACACC	GAATCAACGACCAAGTACCGAAGG	485	P	A	A	RM17416
PMS2564	Os_Chr04	30096923	30096956	(TA)17	(TA)17	(TA)8	(TA)6	(TA)12	(TA)17	(TA)17	(TA)17	(TA)17	(TA)17	(TA)17	-	Introns/Intergenic	-	-	TGGACAGTTTGTTGATCCGA	TGGTGCCCTATATGAGTGTCC	230	P	A	A	NA
PMS2565	Os_Chr04	30141711	30141742	(ATAG)8	(ATAG)8	(ATAG)8	(ATAG)8	(ATAG)8	(ATAG)5	(ATAG)8	(ATAG)8	(ATAG)8	(ATAG)8	(ATAG)8	-	Introns/Intergenic	-	-	GCCAATGTGTGAGAAGAGGATAGG	GAGGCAAGCTTTCTACCATTATGC	369	P	A	A	RM7208
PMS2566	Os_Chr04	30270947	30270960	(AC)7	(AC)8	(AC)8	(AC)7	(AC)7	(AC)8	(AC)7	(AC)7	(AC)7	(AC)7	(AC)6	-	Introns/Intergenic	-	-	CAAATTTGTGGTGCTGTTGG	CCATGATCAATCCAAATCCA	229	P	A	A	NA
PMS2567	Os_Chr04	30282587	30282604	(CGG)6	(CGG)5	(CGG)6	(CGG)6	(CGG)6	(CGG)5	(CGG)6	(CGG)6	(CGG)6	(CGG)6	(CGG)6	LOC_Os04g51130	URR	expressed protein	-	TTCGTGTTGCTCGATCTTTG	AAACGAACTGACTGATGGGC	274	P	A	A	NA
PMS2568	Os_Chr04	30333674	30333695	(CT)11	(CT)11	(CT)11	(CT)11	(CT)11	(CT)10	(CT)11	(CT)11	(CT)9	(CT)11	(CT)11	-	Introns/Intergenic	-	-	CCGCAAAAGTCTAGTTTGCC	GGGGAGAGATGGGAGAGC	153	P	A	A	NA
PMS2569	Os_Chr04	30410184	30410201	(GTC)6	(GTC)6	(GTC)6	(GTC)6	(GTC)6	(GTC)6	(GTC)6	(GTC)6	(GTC)6	(GTC)5	(GTC)6	LOC_Os04g51350	URR	MPR25	-	CACCCCCATATTGGCTATTG	CGAGGAGAGGACATGAGAGG	256	A	A	P	NA
PMS2570	Os_Chr04	30547148	30547161	(CT)7	(CT)6	(CT)7	(CT)7	(CT)7	(CT)7	(CT)7	(CT)7	(CT)7	(CT)7	(CT)7	-	Introns/Intergenic	-	-	AAATTTTGCGCCTCTCCTTT	TGATGAGGTGCGGTCAACTA	152	P	A	A	NA
PMS2571	Os_Chr04	30645616	30645630	(CCG)5	(CCG)5	(CCG)5	(CCG)5	(CCG)5	(CCG)5	(CCG)5	(CCG)5	(CCG)7	(CCG)5	(CCG)5	LOC_Os04g51710	URR	expressed protein	-	GAGAAAAGGAATGGCATGGA	CTTCCTCTCTGTCTCCAGCG	277	A	A	A	NA
PMS2572	Os_Chr04	30682452	30682469	(GGT)6	(GGT)6	(GGT)6	(GGT)6	(GGT)6	(GGT)6	(GGT)6	(GGT)6	(GGT)6	(GGT)5	(GGT)6	-	Introns/Intergenic	-	-	AGACCGGTTCTTGTTCGATG	AAACACCCATAAGAACCCCC	133	A	A	A	NA
PMS2573	Os_Chr04	30770074	30770097	(AAC)8	(AAC)8	(AAC)8	(AAC)8	(AAC)8	(AAC)8	(AAC)8	(AAC)8	(AAC)8	(AAC)8	(AAC)7	-	Introns/Intergenic	-	-	TATTATGCACCACCACTCTACC	GCGTCACAGTGTAGTAGAATTACC	193	A	A	A	RM6992

Polymorphic SSR (PMS) markers	Chromosomes	Start physical positions (bp)	End physical positions (bp)	Nipponbare	Kasalath	Nagina22	IR64	Pokkali	Bala	Tainung67	Azucena	Moroberekan	*Oryza rufipogon*	*Oryza nivara*	MSU gene locus IDs	Structural annotation	Functional annotation	SSR functional effects	Forward primers (5'-3')	Reverse primers (5'-3')	Amplified product size (bp)	Markers exhibiting polymorphism within *indica* rice	Transcrption factor genes-derived markers	Known cloned genes-derived markers	PMS markers corresponding with RM markers
PMS2574	Os_Chr04	30786923	30786936	(TA)7	(TA)6	(TA)6	(TA)7	(TA)7	(TA)6	(TA)7	(TA)6	(TA)6	(TA)7	(TA)6	-	Introns/Intergenic	-	-	GCAAGGACAGACGTAGCGTA	TGCTCAAGGAGCTGGAGATT	246	P	A	A	NA
PMS2575	Os_Chr04	30901667	30901706	(CT)20	(CT)20	(CT)20	(CT)20	(CT)20	(CT)20	(CT)20	(CT)20	(CT)20	(CT)20	(CT)17	-	Introns/Intergenic	-	-	CCCTTTCCTCCTTGAATTGTGC	GTTTGATGTCAGCCATCTCTTGC	163	A	A	A	RM17470
PMS2576	Os_Chr04	31164147	31164170	(GA)12	(GA)12	(GA)12	(GA)6	(GA)12	(GA)12	(GA)12	(GA)12	(GA)12	(GA)12	(GA)12	-	Introns/Intergenic	-	-	TGAGCTTCGTCTACAAGCGA	CAGCTCCCACCATCTCTCTC	128	P	A	A	NA
PMS2577	Os_Chr04	31212020	31212031	(TG)6	(TG)6	(TG)6	(TG)6	(TG)6	(TG)6	(TG)6	(TG)6	(TG)6	(TG)7	(TG)6	-	Introns/Intergenic	-	-	TTAACAGGGTCCAGGAGGTG	ATGATGGCATGCTGTGCTTA	239	A	A	A	NA
PMS2578	Os_Chr04	31254015	31254032	(TGG)6	(TGG)6	(TGG)6	(TGG)6	(TGG)6	(TGG)6	(TGG)5	(TGG)6	(TGG)6	(TGG)6	(TGG)6	LOC_Os04g52560	URR	transposon protein, putative, unclassified, expressed	-	TTGGAGGAGAGGTCGAGGTA	TGAGAGATGAGTGCCTCACG	213	A	P	A	NA
PMS2579	Os_Chr04	31262214	31262245	(AG)16	(AG)16	(AG)16	(AG)16	(AG)16	(AG)16	(AG)16	(AG)16	(AG)16	(AG)16	(AG)13	-	Introns/Intergenic	-	-	TGGAATCACAAACCACGACTAGG	CTACCTCAAGCTCCACGACTTCC	194	A	A	A	RM17486
PMS2580	Os_Chr04	31335651	31335666	(CT)8	(CT)7	(CT)8	(CT)8	(CT)8	(CT)8	(CT)8	(CT)8	(CT)8	(CT)8	(CT)8	-	Introns/Intergenic	-	-	ATTCCTCTCGGCCCATCTT	AGTTTGGAACGGAGCGGT	271	P	A	A	NA
PMS2581	Os_Chr04	31357583	31357600	(CGG)6	(CGG)6	(CGG)6	(CGG)6	(CGG)6	(CGG)6	(CGG)5	(CGG)6	(CGG)6	(CGG)6	(CGG)6	LOC_Os04g52684	CDS	expressed protein	CODON_DELETION	ATCAATACACCGCCACCATC	TCCTGGTCGTACCCGTACTC	259	A	A	A	NA
PMS2582	Os_Chr04	31384642	31384683	(AT)21	(AT)21	(AT)10	(AT)6	(AT)6	(AT)21	(AT)7	(AT)21	(AT)21	(AT)12	(AT)6	-	Introns/Intergenic	-	-	GAAGACCCACCTAGAAATACACG	ACTAAGTGTAGCACCAGCAACG	472	P	A	A	RM17489
PMS2583	Os_Chr04	31654653	31654666	(AT)7	(AT)7	(AT)7	(AT)7	(AT)7	(AT)7	(AT)7	(AT)7	(AT)7	(AT)6	(AT)7	-	Introns/Intergenic	-	-	ATTTGCCGTCCCTCTTCTTT	GCTAGGCCCGACACATAAAC	189	A	A	A	NA
PMS2584	Os_Chr04	31684570	31684589	(CT)10	(CT)10	(CT)10	(CT)10	(CT)10	(CT)10	(CT)10	(CT)10	(CT)10	(CT)10	(CT)7	-	Introns/Intergenic	-	-	GTTGCTGTCTCCCTTGTCGATGG	GTGGATGGAGAGGAGGAGGAAGG	190	A	A	A	RM17504
PMS2585	Os_Chr04	31714277	31714297	(GCA)7	(GCA)7	(GCA)7	(GCA)6	(GCA)6	(GCA)7	(GCA)7	(GCA)6	(GCA)5	(GCA)7	(GCA)7	-	Introns/Intergenic	-	-	AGGTCGTCACGCTAGGAGATGC	ATGCCTGCATAGGCTGTAGACG	190	P	A	A	RM17506
PMS2586	Os_Chr04	31744665	31744688	(GGT)8	(GGT)7	(GGT)8	(GGT)8	(GGT)8	(GGT)8	(GGT)5	(GGT)8	(GGT)7	(GGT)8	(GGT)8	-	Introns/Intergenic	-	-	CCGTAATGCCATTGTTGTCAGC	TTATTATGGGCGGTCGCTAACC	291	P	A	A	RM6629

Polymorphic SSR (PMS) markers	Chromosomes	Start physical positions (bp)	End physical positions (bp)	Nipponbare	Kasalath	Nagina22	IR64	Pokkali	Bala	Tainung67	Azucena	Moroberekan	*Oryza rufipogon*	*Oryza nivara*	MSU gene locus IDs	Structural annotation	Functional annotation	SSR functional effects	Forward primers (5'-3')	Reverse primers (5'-3')	Amplified product size (bp)	Markers exhibiting polymorphism within *indica* rice	Transcrption factor genes-derived markers	Known cloned genes-derived markers	PMS markers corresponding with RM markers
PMS2587	Os_Chr04	31773153	31773166	(AG)7	(AG)7	(AG)7	(AG)6	(AG)7	(AG)7	(AG)7	(AG)7	(AG)6	(AG)7	(AG)6	LOC_Os04g53350	URR	expressed protein	-	GGCTGGCTACTTGGCTAGTG	AAACTCCACACCTCCCCTCT	111	P	A	A	NA
PMS2588	Os_Chr04	31836410	31836427	(GGA)6	(GGA)5	(GGA)6	(GGA)6	(GGA)6	(GGA)6	(GGA)6	(GGA)6	(GGA)6	(GGA)6	(GGA)6	LOC_Os04g53460	URR	AT hook motif family protein, expressed	-	GTTGATACGGCCACTAAGGG	CAAACGATTTCCATCCACG	229	P	A	A	NA
PMS2589	Os_Chr04	31907200	31907229	(GA)15	(GA)15	(GA)15	(GA)15	(GA)15	(GA)15	(GA)15	(GA)11	(GA)15	(GA)15	(GA)15	-	Introns/Intergenic	-	-	GCAATCGCATTTATGGAGAAGG	TTGTTCTTGGCTATGAGGTCTCC	265	A	A	A	RM17513
PMS2590	Os_Chr04	31924424	31924437	(TG)7	(TG)7	(TG)7	(TG)7	(TG)7	(TG)7	(TG)7	(TG)7	(TG)7	(TG)6	(TG)6	-	Introns/Intergenic	-	-	TTGGGGAAATCAAGCACTTC	TCTCTGTTTGCCTGGTTGTG	272	A	A	A	NA
PMS2591	Os_Chr04	31943816	31943831	(CG)8	(CG)8	(CG)8	(CG)8	(CG)8	(CG)8	(CG)8	(CG)7	(CG)8	(CG)8	(CG)8	-	Introns/Intergenic	-	-	CCTCCCCAAACCCTAGCAC	TCTAGACTTTCCTCGGACGG	232	A	A	A	NA
PMS2592	Os_Chr04	31953908	31953923	(CG)8	(CG)8	(CG)8	(CG)8	(CG)8	(CG)8	(CG)8	(CG)7	(CG)8	(CG)8	(CG)8	-	Introns/Intergenic	-	-	CCTCCCCAAACCCTAGCAC	TCTAGACTTTCCTCGGACGG	232	A	A	A	NA
PMS2593	Os_Chr04	32075529	32075546	(CT)9	(CT)9	(CT)9	(CT)9	(CT)8	(CT)9	(CT)9	(CT)9	(CT)9	(CT)9	(CT)9	LOC_Os04g53820	CDS	transposon protein, putative, unclassified, expressed	FRAME_SHIFT	ACGCCACCTCATCTCTTCC	GGCTTCCAACAGGAGAAAAG	223	P	A	A	NA
PMS2594	Os_Chr04	32133538	32133565	(AT)14	(AT)14	(AT)14	(AT)14	(AT)8	(AT)14	(AT)14	(AT)7	(AT)14	(AT)14	(AT)14	-	Introns/Intergenic	-	-	GCTTTCTCTAATCGACACCACTGC	CGGCTTCAATAAGCTCTTTCTGC	180	P	A	A	RM17523
PMS2595	Os_Chr04	32177956	32177979	(GCG)8	(GCG)8	(GCG)8	(GCG)8	(GCG)8	(GCG)8	(GCG)8	(GCG)8	(GCG)6	(GCG)8	(GCG)8	-	Introns/Intergenic	-	-	AGGTTGAAGCGGAGGGACAAGG	TCGGAAGCAATGGTTTACGATCC	177	A	A	A	RM17526
PMS2596	Os_Chr04	32365831	32365848	(CTT)6	(CTT)6	(CTT)5	(CTT)5	(CTT)5	(CTT)5	(CTT)6	(CTT)6	(CTT)6	(CTT)5	(CTT)5	LOC_Os04g54400	URR	BTBN12 - Bric-a-Brac, Tramtrack, Broad Complex BTB domain with non-phototropic hypocotyl 3 NPH3 and coiled-coil domains, expressed	-	TCACTGACGAACGAACAAGC	TCCGATCTCAAACCCTCAAC	202	P	P	A	NA
PMS2597	Os_Chr04	32369486	32369503	(CGA)6	(CGA)6	(CGA)6	(CGA)6	(CGA)6	(CGA)6	(CGA)5	(CGA)6	(CGA)6	(CGA)5	(CGA)5	LOC_Os04g54400	CDS	BTBN12 - Bric-a-Brac, Tramtrack, Broad Complex BTB domain with non-phototropic hypocotyl 3 NPH3 and coiled-coil domains, expressed	CODON_CHANGE_PLUS_CODON_DELETION	GTGGTGCAGGTGCTCTTCTT	CTCCTCGAGCTCCACCAC	270	A	P	A	NA
PMS2598	Os_Chr04	32444891	32444904	(AT)7	(AT)7	(AT)7	(AT)7	(AT)6	(AT)7	(AT)7	(AT)7	(AT)7	(AT)7	(AT)7	-	Introns/Intergenic	-	-	ACACCTAAGTTCCCGTGACTTGC	CGAACTAACACCCATCACCATAGG	200	P	A	A	RM17476
PMS2599	Os_Chr04	32454093	32454156	(AT)32	(AT)32	(AT)32	(AT)32	(AT)32	(AT)6	(AT)10	(AT)32	(AT)32	(AT)32	(AT)6	-	Introns/Intergenic	-	-	TCCACCACGAAAGGGTTACT	GGTAAGTTGTAGAAGTTGAATTTGAGC	167	P	A	A	NA

Polymorphic SSR (PMS) markers	Chromosomes	Start physical positions (bp)	End physical positions (bp)	Nipponbare	Kasalath	Nagina22	IR64	Pokkali	Bala	Tainung67	Azucena	Moroberekan	*Oryza rufipogon*	*Oryza nivara*	MSU gene locus IDs	Structural annotation	Functional annotation	SSR functional effects	Forward primers (5'-3')	Reverse primers (5'-3')	Amplified product size (bp)	Markers exhibiting polymorphism within *indica* rice	Transcrption factor genes-derived markers	Known cloned genes-derived markers	PMS markers corresponding with RM markers
PMS2600	Os_Chr04	32558831	32558854	(CCT)8	(CCT)8	(CCT)8	(CCT)7	(CCT)8	(CCT)8	(CCT)8	(CCT)8	(CCT)8	(CCT)8	(CCT)8	-	Introns/Intergenic	-	-	CTTGCGGTTCTTCTTGAAATAGGC	TATTCTTCACCGATGGCGTACTGG	239	P	A	A	RM17532
PMS2601	Os_Chr04	32593687	32593701	(CAA)5	(CAA)5	(CAA)5	(CAA)5	(CAA)5	(CAA)5	(CAA)5	(CAA)5	(CAA)5	(CAA)6	(CAA)5	-	Introns/Intergenic	-	-	CATGTTCGCTGCGTCATACT	AGGATCATATGCCTCGCATC	180	A	A	A	NA
PMS2602	Os_Chr04	32691938	32691961	(AG)12	(AG)10	(AG)12	(AG)12	(AG)12	(AG)12	(AG)12	(AG)12	(AG)12	(AG)10	(AG)12	-	Introns/Intergenic	-	-	TACAGGAGGTGAATTTCGGG	GGCATCCTCTCCGGTAAAA	228	P	A	A	NA
PMS2603	Os_Chr04	32697677	32697688	(CT)6	(CT)6	(CT)6	(CT)6	(CT)6	(CT)6	(CT)6	(CT)6	(CT)6	(CT)9	(CT)6	-	Introns/Intergenic	-	-	CGAACCTAGGGTTTGGGATT	GACCCAGCAGTCAGTGACAA	171	A	A	A	NA
PMS2604	Os_Chr04	32719289	32719310	(CT)11	(CT)11	(CT)6	(CT)8	(CT)11	(CT)11	(CT)11	(CT)11	(CT)11	(CT)11	(CT)11	-	Introns/Intergenic	-	-	TCGAGTTCAATTCCGAATCC	GGAGGGAGAGAGGGAGAGAG	218	P	A	A	NA
PMS2605	Os_Chr04	32779181	32779200	(GA)10	(GA)10	(GA)10	(GA)10	(GA)10	(GA)10	(GA)10	(GA)10	(GA)10	(GA)11	(GA)10	-	Introns/Intergenic	-	-	GACAGCGACATGGAGGTGAGC	AAAGCCCATGTCGTCATCAAGC	437	A	A	A	RM17549
PMS2606	Os_Chr04	32788824	32788847	(CGG)8	(CGG)8	(CGG)5	(CGG)8	(CGG)8	(CGG)8	(CGG)8	(CGG)8	(CGG)8	(CGG)5	(CGG)7	-	Introns/Intergenic	-	-	GTCTCCTCCATAGACAATCCCGTTCC	CGCCGAAGATGAGGTTGATGC	309	P	A	A	RM17550
PMS2607	Os_Chr04	32826738	32826758	(GAG)7	(GAG)7	(GAG)7	(GAG)7	(GAG)7	(GAG)7	(GAG)6	(GAG)7	(GAG)7	(GAG)7	(GAG)7	-	Introns/Intergenic	-	-	ACCACTCTCCTCGGCTTGTGTCG	CTGGGAGGTGGTGTTGGACTGG	271	A	A	A	RM17552
PMS2608	Os_Chr04	32834730	32834749	(AG)10	(AG)12	(AG)10	(AG)12	(AG)10	(AG)10	(AG)10	(AG)10	(AG)10	(AG)10	(AG)10	-	Introns/Intergenic	-	-	ATCTGGCCTTCCCTCCTCTCC	CATTCGACGGTCACGAGAGAGC	140	P	A	A	RM17554
PMS2609	Os_Chr04	33030852	33030869	(GCC)6	(GCC)6	(GCC)6	(GCC)6	(GCC)6	(GCC)6	(GCC)6	(GCC)6	(GCC)6	(GCC)6	(GCC)5	LOC_Os04g55520	DRR	AP2 domain containing protein, expressed	-	CAAAACCGCATCGAAAAATC	AGCGTATGATCACCTCCACC	128	A	P	A	NA
PMS2610	Os_Chr04	33031144	33031170	(GGT)9	(GGT)9	(GGT)5	(GGT)8	(GGT)9	(GGT)9	(GGT)9	(GGT)9	(GGT)9	(GGT)9	(GGT)8	LOC_Os04g55520	CDS	AP2 domain containing protein, expressed	CODON_DELETION	ATACGCTGGCTGCATGTTGACG	TACGACACGGCGGTGTTCTACC	469	P	P	A	RM5950
PMS2611	Os_Chr04	33113355	33113368	(GT)7	(GT)7	(GT)6	(GT)9	(GT)7	(GT)7	(GT)6	(GT)7	(GT)7	(GT)7	(GT)7	-	Introns/Intergenic	-	-	TGTGGAGTGGAGTGAAGTGG	AACCGATCAAGTGCCAAAAG	162	P	A	A	NA
PMS2612	Os_Chr04	33179094	33179111	(CGG)6	(CGG)6	(CGG)6	(CGG)6	(CGG)6	(CGG)6	(CGG)6	(CGG)5	(CGG)6	(CGG)6	(CGG)6	LOC_Os04g55730	URR	alpha-N-acetylglucosaminidase, putative, expressed	-	CAGGAGCACCCGGAGTAAG	AGTTAATCCCTCCCCACACC	138	A	A	A	NA

Polymorphic SSR (PMS) markers	Chromosomes	Start physical positions (bp)	End physical positions (bp)	Nipponbare	Kasalath	Nagina22	IR64	Pokkali	Bala	Tainung67	Azucena	Moroberekan	*Oryza rufipogon*	*Oryza nivara*	MSU gene locus IDs	Structural annotation	Functional annotation	SSR functional effects	Forward primers (5'-3')	Reverse primers (5'-3')	Amplified product size (bp)	Markers exhibiting polymorphism within *indica* rice	Transcrption factor genes-derived markers	Known cloned genes-derived markers	PMS markers corresponding with RM markers
PMS2613	Os_Chr04	33214623	33214649	(CGA)9	(CGA)9	(CGA)9	(CGA)9	(CGA)9	(CGA)9	(CGA)9	(CGA)9	(CGA)9	(CGA)9	(CGA)7	-	Introns/Intergenic	-	-	CTCGGCTAGCTTGCGTTTCTTGC	CCGTTAGCGAGGTTTCCGTTCC	132	A	A	A	RM17571
PMS2614	Os_Chr04	33306723	33306737	(CAG)5	(CAG)6	(CAG)6	(CAG)6	(CAG)6	(CAG)6	(CAG)5	(CAG)5	(CAG)5	(CAG)6	(CAG)6	LOC_Os04g55920	CDS	OsJAZ1|OsTIFY3|EG2	CODON_INSERTION	AAGGAGGAAGAGGTGGTGGT	AGATGCATTTCCCGAACAAC	209	A	A	P	NA
PMS2615	Os_Chr04	33324531	33324576	(TA)23	(TA)23	(TA)23	(TA)23	(TA)12	(TA)23	(TA)23	(TA)14	(TA)23	(TA)23	(TA)17	-	Introns/Intergenic	-	-	CCTTTACTCACTTGCAAGCAACC	TGGAGTGTGGAGCTAAGGTATGG	316	P	A	A	RM17574
PMS2616	Os_Chr04	33337075	33337092	(CA)9	(CA)7	(CA)9	(CA)9	(CA)7	(CA)9	(CA)9	(CA)9	(CA)9	(CA)8	(CA)9	-	Introns/Intergenic	-	-	CATGTGAAGCTTAGCACCGA	TTGCCACTAGCATGAAGCTG	242	P	A	A	NA
PMS2617	Os_Chr04	33376129	33376146	(CT)9	(CT)9	(CT)9	(CT)9	(CT)6	(CT)9	(CT)9	(CT)9	(CT)9	(CT)9	(CT)9	-	Introns/Intergenic	-	-	CGTCACCACCCAATCTTTTT	ACCTTCTCTCCCTCCTCACC	202	P	A	A	NA
PMS2618	Os_Chr04	33475328	33475349	(TA)11	(TA)11	(TA)11	(TA)11	(TA)11	(TA)11	(TA)11	(TA)11	(TA)11	(TA)11	(TA)10	-	Introns/Intergenic	-	-	GTAGGTCCGGCACTGTTGTT	ATCATCAGGGGGACACAATC	260	A	A	A	NA
PMS2619	Os_Chr04	33500759	33500776	(AT)9	(AT)8	(AT)9	(AT)9	(AT)9	(AT)9	(AT)9	(AT)9	(AT)9	(AT)9	(AT)8	-	Introns/Intergenic	-	-	TGTGCACCGTCAATATCAGAA	GTACAGGCACAGACCGGAAC	238	P	A	A	NA
PMS2620	Os_Chr04	33530268	33530288	(GTC)7	(GTC)7	(GTC)7	(GTC)7	(GTC)7	(GTC)7	(GTC)7	(GTC)7	(GTC)7	(GTC)6	(GTC)7	LOC_Os04g56240	URR	lipase, putative, expressed	-	CCTGCATAGCGGAGAGGTAG	CGCAGCACCATGTAGTCATT	182	A	A	A	NA
PMS2621	Os_Chr04	33532357	33532384	(AT)14	(AT)14	(AT)14	(AT)14	(AT)14	(AT)14	(AT)14	(AT)9	(AT)14	(AT)14	(AT)14	-	Introns/Intergenic	-	-	TCAACTTCTACATCTCGCAACG	CGGTCAACATCAGTACATCACC	483	A	A	A	RM17587
PMS2622	Os_Chr04	33591755	33591772	(CGG)6	(CGG)6	(CGG)6	(CGG)6	(CGG)6	(CGG)6	(CGG)6	(CGG)5	(CGG)6	(CGG)6	(CGG)6	LOC_Os04g56350	CDS	nucleolar protein,Nop52 containing protein, expressed	CODON_DELETION	GACTGGTAGAGCGGCTTGTC	TAGGGTTTCCACCTCTCCCT	254	A	A	A	NA
PMS2623	Os_Chr04	33682345	33682362	(CT)9	(CT)9	(CT)9	(CT)9	(CT)9	(CT)9	(CT)8	(CT)9	(CT)9	(CT)9	(CT)9	-	Introns/Intergenic	-	-	ACCAACATCGCAGATACAGC	TGGGAGCACAATGAATTGAA	257	A	A	A	NA
PMS2624	Os_Chr04	33726605	33726634	(TA)15	(TA)15	(TA)15	(TA)6	(TA)15	(TA)10	(TA)10	(TA)15	(TA)9	(TA)15	(TA)15	-	Introns/Intergenic	-	-	GATGGATGACTTCGTGACATGC	GCCTCTTTGCCTCCCTAATGG	591	P	A	A	RM17598
PMS2625	Os_Chr04	33733834	33733854	(GAC)7	(GAC)7	(GAC)7	(GAC)7	(GAC)7	(GAC)5	(GAC)7	(GAC)7	(GAC)5	(GAC)7	(GAC)7	LOC_Os04g56570	CDS	heavy metal-associated domain containing protein, expressed	CODON_DELETION	TGCTTCTTGATCTGCTGCTG	CGATATCGACTCGGAGAAGG	180	P	A	A	NA

Polymorphic SSR (PMS) markers	Chromosomes	Start physical positions (bp)	End physical positions (bp)	Nipponbare	Kasalath	Nagina22	IR64	Pokkali	Bala	Tainung67	Azucena	Moroberekan	*Oryza rufipogon*	*Oryza nivara*	MSU gene locus IDs	Structural annotation	Functional annotation	SSR functional effects	Forward primers (5'-3')	Reverse primers (5'-3')	Amplified product size (bp)	Markers exhibiting polymorphism within *indica* rice	Transcrption factor genes-derived markers	Known cloned genes-derived markers	PMS markers corresponding with RM markers
PMS2626	Os_Chr04	33782253	33782276	(CCG)8	(CCG)8	(CCG)8	(CCG)8	(CCG)8	(CCG)7	(CCG)8	(CCG)8	(CCG)7	(CCG)8	(CCG)6	LOC_Os04g56654	CDS	BSD domain-containing protein, putative, expressed	CODON_CHANGE_PLUS_CODON_DELETION	CCTATGGCCTTCTCCTCTTTCTCG	ATGGAGATGGCGAGTGTGTGG	239	P	A	A	RM17601
PMS2627	Os_Chr04	33809679	33809693	(CGG)5	(CGG)5	(CGG)5	(CGG)5	(CGG)5	(CGG)5	(CGG)5	(CGG)5	(CGG)5	(CGG)6	(CGG)5	LOC_Os04g56700	CDS	naringenin,2-oxoglutarate 3-dioxygenase, putative, expressed	CODON_INSERTION	CTTCGATTTTCGACGGAAGA	TCAAGAAGAAGGCCAAGGAG	258	A	A	A	NA
PMS2628	Os_Chr04	33894334	33894357	(CTG)8	(CTG)8	(CTG)8	(CTG)8	(CTG)8	(CTG)8	(CTG)8	(CTG)8	(CTG)8	(CTG)8	(CTG)7	LOC_Os04g56850	URR	auxin response factor, putative, expressed	-	AGATGGCAATGATACGGAGG	GCAAATGAGGAATCATGGCT	203	A	P	A	NA
PMS2629	Os_Chr04	33990257	33990280	(CGC)8	(CGC)8	(CGC)8	(CGC)8	(CGC)8	(CGC)8	(CGC)8	(CGC)8	(CGC)8	(CGC)8	(CGC)5	LOC_Os04g57030	CDS	VQ domain containing protein, putative, expressed	CODON_DELETION	CGGCCTCCTCCACCTTTATTTCC	ACAAGCTCTCCCACGCCATCG	279	A	A	A	RM17608
PMS2630	Os_Chr04	34063231	34063269	(CAT)13	(CAT)13	(CAT)13	(CAT)13	(CAT)13	(CAT)13	(CAT)13	(CAT)11	(CAT)13	(CAT)13	(CAT)13	LOC_Os04g57160	DRR	flavonol synthase/flavanone 3-hydroxylase, putative, expressed	-	GAGCAAATCCAGACCAGAAGTGC	ACACCTGGCAGCCAAGATATGG	239	A	A	A	RM17611
PMS2631	Os_Chr04	34295232	34295263	(CGTG)8	(CGTG)8	(CGTG)8	(CGTG)8	(CGTG)8	(CGTG)8	(CGTG)8	(CGTG)8	(CGTG)8	(CGTG)6	(CGTG)8	-	Introns/Intergenic	-	-	ACGCTCTCAAATCGATCAGTGG	CACCACCACCATACTACACATACTCC	99	A	A	A	RM7410
PMS2632	Os_Chr04	34302613	34302628	(CT)8	(CT)8	(CT)8	(CT)6	(CT)6	(CT)6	(CT)8	(CT)8	(CT)8	(CT)6	(CT)6	LOC_Os04g57630	URR	phytosulfokine receptor precursor, putative, expressed	-	GAGGAGAGTGGGGGAAAGTC	CACCATATCAAATGCGCAAC	250	P	A	A	NA
PMS2633	Os_Chr04	34338506	34338523	(CTC)6	(CTC)6	(CTC)6	(CTC)6	(CTC)5	(CTC)5	(CTC)6	(CTC)6	(CTC)6	(CTC)6	(CTC)6	LOC_Os04g57680	URR	expressed protein	-	GCCAAGGCCAAATAAACAAA	GCTTGGCTTGACTTGCTTCT	270	P	A	A	NA
PMS2634	Os_Chr04	34504050	34504105	(TAGA)14	(TAGA)14	(TAGA)14	(TAGA)13	(TAGA)13	(TAGA)13	(TAGA)14	(TAGA)14	(TAGA)14	(TAGA)14	(TAGA)13	-	Introns/Intergenic	-	-	TTTTGGTACGGACAGGAAAG	CAGTCCACGTCCACTCTTCA	129	P	A	A	NA
PMS2635	Os_Chr04	34534363	34534383	(CGG)7	(CGG)7	(CGG)7	(CGG)7	(CGG)7	(CGG)7	(CGG)7	(CGG)7	(CGG)7	(CGG)7	(CGG)6	LOC_Os04g57970	CDS	expressed protein	CODON_DELETION	ATCGCTTGTTGTCAAGCCCAAGG	CAGAGAACTCCCGCTCGAGAACC	248	A	A	A	RM17637
PMS2636	Os_Chr04	34554573	34554596	(GA)12	(GA)12	(GA)12	(GA)9	(GA)9	(GA)9	(GA)12	(GA)12	(GA)12	(GA)12	(GA)12	-	Introns/Intergenic	-	-	GGTTTAGGGTAGAGGAGCACACG	CATCAAGAAAGCTTCTCCCATCG	219	P	A	A	RM5307
PMS2637	Os_Chr04	34595116	34595139	(ATAG)6	(ATAG)6	(ATAG)6	(ATAG)6	(ATAG)6	(ATAG)6	(ATAG)6	(ATAG)6	(ATAG)5	(ATAG)6	(ATAG)5	-	Introns/Intergenic	-	-	GAATTTCTAGCCACTACAGCGAACG	GCTACGAGCTGTGGTGGTTGC	190	A	A	A	RM7172
PMS2638	Os_Chr04	34672968	34672985	(GGC)6	(GGC)6	(GGC)6	(GGC)6	(GGC)6	(GGC)6	(GGC)6	(GGC)6	(GGC)6	(GGC)6	(GGC)5	LOC_Os04g58230	CDS	transporter family protein, putative, expressed	CODON_DELETION	CAGCACAAGAAAGACGGTGA	AGACAGAGCAGGCAAAGACG	224	A	A	A	NA

**Polymorphic SSR (PMS) markers**	**Chromosomes**	**Start physical positions (bp)**	**End physical positions (bp)**	**Nipponbare**	**Kasalath**	**Nagina22**	**IR64**	**Pokkali**	**Bala**	**Tainung67**	**Azucena**	**Moroberekan**	***Oryza rufipogon***	***Oryza nivara***	**MSU gene locus IDs**	**Structural annotation**	**Functional annotation**	**SSR functional effects**	**Forward primers (5'-3')**	**Reverse primers (5'-3')**	**Amplified product size (bp)**	**Markers exhibiting polymorphism within *indica* rice**	**Transcrption factor genes-derived markers**	**Known cloned genes-derived markers**	**PMS markers corresponding with RM markers**
PMS2639	Os_Chr04	34686053	34686118	(TA)33	(TA)33	(TA)33	(TA)33	(TA)6	(TA)33	(TA)33	(TA)33	(TA)33	(TA)33	(TA)33	-	Introns/Intergenic	-	-	GCTACTACTGCTGGTATTGTATCC	AGCACAGGTGAGTTATTGTAGC	393	P	A	A	RM17644
PMS2640	Os_Chr04	34690952	34691011	(AT)30	(AT)30	(AT)30	(AT)30	(AT)7	(AT)30	(AT)30	(AT)30	(AT)30	(AT)30	(AT)30	-	Introns/Intergenic	-	-	GCTTTGTTGGGTGATCGTCTAGG	GGCGATCTACTGTTCTTGTCACC	255	P	A	A	RM17645
PMS2641	Os_Chr04	34703837	34703857	(TTC)7	(TTC)7	(TTC)6	(TTC)7	(TTC)7	(TTC)6	(TTC)7	(TTC)7	(TTC)7	(TTC)7	(TTC)7	-	Introns/Intergenic	-	-	CACACAAAGCAAGTCCTCTCATCC	CAAGGCCCAGAACAAGAGTAAGG	126	P	A	A	RM17646
PMS2642	Os_Chr04	34711765	34711779	(TTA)5	(TTA)6	(TTA)5	(TTA)5	(TTA)5	(TTA)5	(TTA)5	(TTA)5	(TTA)5	(TTA)5	(TTA)5	-	Introns/Intergenic	-	-	ATCTACGGTCCAAACTATCTCC	ACCATGTGTGTAGTTTCAGAGC	297	P	A	A	RM10431
PMS2643	Os_Chr04	34751752	34751775	(GCC)8	(GCC)8	(GCC)8	(GCC)8	(GCC)8	(GCC)8	(GCC)6	(GCC)8	(GCC)8	(GCC)8	(GCC)8	LOC_Os04g58410	CDS	smr domain containing protein, expressed	CODON_DELETION	CGAAACCCAACCTTCTTCTTCC	TCTCTACCCGCATCTGCTTCG	186	A	A	A	RM17654
PMS2644	Os_Chr04	34911435	34911464	(CT)15	(CT)15	(CT)15	(CT)15	(CT)15	(CT)15	(CT)15	(CT)15	(CT)15	(CT)9	(CT)15	-	Introns/Intergenic	-	-	CGGTTATTTGTGGCTCAAAGTGC	GCTTGTTGGCACCTTACAATTTGG	289	A	A	A	RM3333
PMS2645	Os_Chr04	34924944	34924963	(GA)10	(GA)10	(GA)10	(GA)7	(GA)10	(GA)10	(GA)10	(GA)10	(GA)10	(GA)10	(GA)10	LOC_Os04g58730	URR	AT hook motif domain containing protein, expressed	-	GGTGTTGAAGATGAAGAGTGAGTTGG	AAATCTTATCGCCTCCTTGTGTGG	291	P	A	A	RM124
PMS2646	Os_Chr04	34957749	34957808	(TA)30	(TA)30	(TA)30	(TA)30	(TA)30	(TA)30	(TA)30	(TA)30	(TA)7	(TA)30	(TA)30	-	Introns/Intergenic	-	-	AGAGCCAAATCCAACGGTATGTAGC	CCAACATTCAGGCGACAGAGG	182	A	A	A	RM17669
PMS2647	Os_Chr04	35078395	35078418	(GGA)8	(GGA)8	(GGA)8	(GGA)8	(GGA)8	(GGA)8	(GGA)8	(GGA)8	(GGA)8	(GGA)8	(GGA)5	-	Introns/Intergenic	-	-	CCAAATCGCCCTCCATAATCC	CCTCAGCGAGACATCACTTCTCC	337	A	A	A	RM6246
PMS2648	Os_Chr04	35106702	35106722	(GGC)7	(GGC)5	(GGC)5	(GGC)7	(GGC)7	(GGC)7	(GGC)7	(GGC)7	(GGC)7	(GGC)7	(GGC)7	LOC_Os04g59010	CDS	F-box domain containing protein, expressed	CODON_DELETION	AGGAAGTTGTGGGAGAGGGT	GTAGTTGCGCACCGCTATCT	253	P	A	A	NA
PMS2649	Os_Chr04	35124080	35124094	(GAA)5	(GAA)5	(GAA)5	(GAA)6	(GAA)6	(GAA)6	(GAA)5	(GAA)5	(GAA)5	(GAA)5	(GAA)5	LOC_Os04g59040	URR	soluble inorganic pyrophosphatase, putative, expressed	-	ATAGCTTCCGCTCAGATTGG	AACGTTGGAAGGAGGGAGTT	254	P	A	A	NA
PMS2650	Os_Chr04	35327300	35327320	(AGA)7	(AGA)7	(AGA)7	(AGA)6	(AGA)6	(AGA)6	(AGA)7	(AGA)7	(AGA)7	(AGA)7	(AGA)6	LOC_Os04g59420	URR	expressed protein	-	GCAACTCCAGATACCGACCTAGC	TCCTGCTACTCCATCCTTCATGG	114	P	A	A	RM17691
PMS2651	Os_Chr04	35358664	35358684	(CAG)7	(CAG)7	(CAG)7	(CAG)7	(CAG)7	(CAG)7	(CAG)7	(CAG)7	(CAG)7	(CAG)6	(CAG)7	LOC_Os04g59470	CDS	no apical meristem protein, putative, expressed	CODON_DELETION	CAAGTGAAGTGGTGGTGGTG	GACGAGGAGGATGAATCCAA	186	A	A	A	NA

Polymorphic SSR (PMS) markers	Chromosomes	Start physical positions (bp)	End physical positions (bp)	Nipponbare	Kasalath	Nagina22	IR64	Pokkali	Bala	Tainung67	Azucena	Moroberekan	*Oryza rufipogon*	*Oryza nivara*	MSU gene locus IDs	Structural annotation	Functional annotation	SSR functional effects	Forward primers (5'-3')	Reverse primers (5'-3')	Amplified product size (bp)	Markers exhibiting polymorphism within *indica* rice	Transcrption factor genes-derived markers	Known cloned genes-derived markers	PMS markers corresponding with RM markers
PMS2652	Os_Chr04	35421394	35421414	(CCG)7	(CCG)7	(CCG)7	(CCG)7	(CCG)7	(CCG)7	(CCG)6	(CCG)7	(CCG)7	(CCG)5	(CCG)7	LOC_Os04g59550	URR	OsUGT1	-	GCAGGCAAGCTTGAGCAAATGG	GGCTGAGGCTCCTCTTCCTCTTCC	86	A	A	P	RM17706
PMS2653	Os_Chr04	35439381	35439398	(GCG)6	(GCG)6	(GCG)6	(GCG)6	(GCG)6	(GCG)6	(GCG)6	(GCG)6	(GCG)6	(GCG)6	(GCG)5	LOC_Os04g59600	URR	pentatricopeptide, putative, expressed	-	CCAGATAGGTAGGCAGGCAG	GGTGGTGGTGGTGGTAGAGT	172	A	A	A	NA
PMS2654	Os_Chr05	41290	41322	(CAA)11	(CAA)11	(CAA)11	(CAA)11	(CAA)11	(CAA)11	(CAA)11	(CAA)11	(CAA)11	(CAA)10	(CAA)11	-	Introns/Intergenic	-	-	GCCAAGAGAACAGGACAAATATGC	GCTTGCTAAGCATGTTCCTAGAGC	188	A	A	A	RM17709
PMS2655	Os_Chr05	99740	99757	(GAA)6	(GAA)5	(GAA)6	(GAA)6	(GAA)6	(GAA)5	(GAA)6	(GAA)6	(GAA)6	(GAA)6	(GAA)5	LOC_Os05g01110	URR	ribosomal protein L28 protein, putative, expressed	-	AGCCGAGAGAGGAAGAGGAG	TAATGAGAGTTGGGCTTGGG	262	P	A	A	NA
PMS2656	Os_Chr05	143868	143888	(GCC)7	(GCC)7	(GCC)7	(GCC)6	(GCC)7	(GCC)7	(GCC)7	(GCC)5	(GCC)7	(GCC)7	(GCC)7	LOC_Os05g01190	URR	transposon protein, putative, unclassified, expressed	-	GACCGGATCGAGTTGTTCAT	AGGGACCTTGGACTTGGAGT	263	P	A	A	NA
PMS2657	Os_Chr05	169905	169955	(ATA)17	(ATA)17	(ATA)17	(ATA)17	(ATA)17	(ATA)18	(ATA)17	(ATA)17	(ATA)17	(ATA)17	(ATA)17	-	Introns/Intergenic	-	-	CGTGTCACATCCATTGAAGG	TCTTACAAACTGAAACGGAAGGA	279	P	A	A	NA
PMS2658	Os_Chr05	193352	193381	(AAC)10	(AAC)10	(AAC)10	(AAC)8	(AAC)10	(AAC)10	(AAC)10	(AAC)10	(AAC)10	(AAC)10	(AAC)7	LOC_Os05g01290	URR	expressed protein	-	GCTCGCCTCGCTTACTTACTACC	CCTCCTATTTCTTTCCCGATAGAGC	95	P	A	A	RM17730
PMS2659	Os_Chr05	194175	194234	(ATGT)15	(ATGT)10	(ATGT)15	(ATGT)15	(ATGT)11	(ATGT)5	(ATGT)15	(ATGT)15	(ATGT)12	(ATGT)15	(ATGT)6	-	Introns/Intergenic	-	-	TTTAAAGGCGTGTGCTCAGA	TTCCCTGTCCTGATGGACTC	259	P	A	A	NA
PMS2660	Os_Chr05	214356	214373	(CAG)6	(CAG)6	(CAG)6	(CAG)6	(CAG)6	(CAG)6	(CAG)6	(CAG)5	(CAG)6	(CAG)6	(CAG)6	LOC_Os05g01350	CDS	retrotransposon protein, putative, unclassified, expressed	CODON_DELETION	CCCCTTCACCAACTACCCTT	GGAAAGGAAGGAGAGGAGGA	193	A	A	A	NA
PMS2661	Os_Chr05	250776	250799	(CCG)8	(CCG)5	(CCG)8	(CCG)8	(CCG)8	(CCG)8	(CCG)8	(CCG)8	(CCG)8	(CCG)8	(CCG)8	-	Introns/Intergenic	-	-	CCGTCACTCGTTTCATTCTCG	GGGAGGTGGACAGAAAGAGTAAGC	153	P	A	A	RM17736
PMS2662	Os_Chr05	304358	304371	(TA)7	(TA)7	(TA)7	(TA)7	(TA)7	(TA)6	(TA)7	(TA)7	(TA)7	(TA)7	(TA)7	LOC_Os05g01520	DRR	ECT protein, putative, expressed	-	CTTGTGCTTTGTTCTTGCCA	TGATCCAACGACGGGATAAT	214	P	A	A	NA
PMS2663	Os_Chr05	324421	324432	(AG)6	(AG)6	(AG)6	(AG)6	(AG)7	(AG)6	(AG)6	(AG)6	(AG)6	(AG)6	(AG)6	-	Introns/Intergenic	-	-	GTAGTAGTAGCCCCCTGCCC	AGGAATGTTGTTGTTTCGGC	243	P	A	A	NA
PMS2664	Os_Chr05	344529	344552	(AT)12	(AT)12	(AT)12	(AT)6	(AT)12	(AT)12	(AT)12	(AT)12	(AT)12	(AT)12	(AT)12	-	Introns/Intergenic	-	-	AGCATGCATGTGGAAAACAA	TCCTAGACATCTTATCGTCCGT	277	P	A	A	NA

Polymorphic SSR (PMS) markers	Chromosomes	Start physical positions (bp)	End physical positions (bp)	Nipponbare	Kasalath	Nagina22	IR64	Pokkali	Bala	Tainung67	Azucena	Moroberekan	*Oryza rufipogon*	*Oryza nivara*	MSU gene locus IDs	Structural annotation	Functional annotation	SSR functional effects	Forward primers (5'-3')	Reverse primers (5'-3')	Amplified product size (bp)	Markers exhibiting polymorphism within *indica* rice	Transcrption factor genes-derived markers	Known cloned genes-derived markers	PMS markers corresponding with RM markers
PMS2665	Os_Chr05	347274	347297	(CAG)8	(CAG)7	(CAG)8	(CAG)8	(CAG)8	(CAG)8	(CAG)8	(CAG)8	(CAG)8	(CAG)8	(CAG)8	LOC_Os05g01580	CDS	integral membrane protein DUF6 containing protein, expressed	CODON_DELETION	GATGACGATGAATGGCAATG	GAAGCGGTGATTTTGTTGGT	170	P	A	A	NA
PMS2666	Os_Chr05	524618	524643	(TC)13	(TC)13	(TC)13	(TC)13	(TC)14	(TC)13	(TC)13	(TC)11	(TC)13	(TC)13	(TC)13	-	Introns/Intergenic	-	-	ACATTTCGAAACGGATGGAG	ATGCAGATGATAGCCCAAGG	235	P	A	A	NA
PMS2667	Os_Chr05	600304	600327	(CTT)8	(CTT)8	(CTT)8	(CTT)8	(CTT)8	(CTT)7	(CTT)8	(CTT)8	(CTT)8	(CTT)8	(CTT)8	LOC_Os05g02060	URR	mitochondrial import inner membrane translocase subunit Tim17, putative, expressed	-	TTTGCTTTCGTGCCTTGTCAGC	TCTCTCTTGTTGGTGATGGTGAGG	91	P	A	A	RM6300
PMS2668	Os_Chr05	629892	629912	(CGG)7	(CGG)7	(CGG)7	(CGG)7	(CGG)7	(CGG)7	(CGG)5	(CGG)7	(CGG)7	(CGG)7	(CGG)7	LOC_Os05g02110	CDS	protein phosphatase 2C, putative, expressed	CODON_DELETION	CAACAGCATCGGTCACATTC	AAGGAGTTGGAGCTGTCGAA	189	A	A	A	NA
PMS2669	Os_Chr05	655557	655572	(TG)8	(TG)8	(TG)8	(TG)8	(TG)8	(TG)8	(TG)8	(TG)8	(TG)8	(TG)9	(TG)8	-	Introns/Intergenic	-	-	CCGTACCTGCATCCTCTCTC	TTTAACCCATGCATCCAAGA	188	A	A	A	NA
PMS2670	Os_Chr05	706806	706819	(CT)7	(CT)6	(CT)7	(CT)7	(CT)7	(CT)7	(CT)7	(CT)7	(CT)7	(CT)7	(CT)6	LOC_Os05g02230	URR	expressed protein	-	CCCACATGTCAGCCTCACTA	ATGCGCTTCTTCGGTCAC	216	P	A	A	NA
PMS2671	Os_Chr05	711639	711653	(CCG)5	(CCG)5	(CCG)5	(CCG)6	(CCG)5	(CCG)5	(CCG)5	(CCG)5	(CCG)5	(CCG)5	(CCG)5	LOC_Os05g02240	URR	ATCHX20, putative, expressed	-	CACATCACATGCACGAGACA	AGGAGGAGGATGAGGAGAGC	255	P	A	A	NA
PMS2672	Os_Chr05	742920	742937	(AG)9	(AG)8	(AG)8	(AG)9	(AG)9	(AG)9	(AG)9	(AG)9	(AG)9	(AG)9	(AG)9	LOC_Os05g02310	URR	soluble inorganic pyrophosphatase, putative, expressed	-	GATCTGAATCTGGGGACGAA	AAGCAACGAGGACCAAAGAA	222	P	A	A	NA
PMS2673	Os_Chr05	775333	775346	(TC)7	(TC)13	(TC)7	(TC)7	(TC)7	(TC)7	(TC)7	(TC)7	(TC)7	(TC)7	(TC)7	-	Introns/Intergenic	-	-	TCTCCCTCCAGTTCCTTC	CTGCGGGAGAGAGAGAAA	286	P	A	A	NA
PMS2674	Os_Chr05	828377	828396	(GATG)5	(GATG)5	(GATG)5	(GATG)5	(GATG)6	(GATG)5	(GATG)5	(GATG)5	(GATG)5	(GATG)5	(GATG)5	-	Introns/Intergenic	-	-	AAGCAAGTCCCAAGAACCCT	TGGGGAGAGAGAAGAGGACA	265	P	A	A	NA
PMS2675	Os_Chr05	836698	836717	(AG)10	(AG)10	(AG)10	(AG)10	(AG)10	(AG)10	(AG)10	(AG)10	(AG)10	(AG)10	(AG)9	-	Introns/Intergenic	-	-	GTTTGGCACTGTAGTGAGATGC	GTTGATGATGAGGAGACACAAGG	280	A	A	A	RM17786
PMS2676	Os_Chr05	898374	898387	(AG)7	(AG)7	(AG)7	(AG)7	(AG)7	(AG)7	(AG)7	(AG)7	(AG)7	(AG)8	(AG)7	-	Introns/Intergenic	-	-	AATGCAAACACACTTGCAGC	AGGAGGAAACACACAAAGCC	228	A	A	A	NA
PMS2677	Os_Chr05	962004	962019	(TC)8	(TC)10	(TC)8	(TC)8	(TC)8	(TC)8	(TC)8	(TC)8	(TC)8	(TC)8	(TC)8	-	Introns/Intergenic	-	-	CTGCACTACAGGGTTTGGGT	CAGTTCTCCTCTCCCTCACG	247	P	A	A	NA

Polymorphic SSR (PMS) markers	Chromosomes	Start physical positions (bp)	End physical positions (bp)	Nipponbare	Kasalath	Nagina22	IR64	Pokkali	Bala	Tainung67	Azucena	Moroberekan	*Oryza rufipogon*	*Oryza nivara*	MSU gene locus IDs	Structural annotation	Functional annotation	SSR functional effects	Forward primers (5'-3')	Reverse primers (5'-3')	Amplified product size (bp)	Markers exhibiting polymorphism within *indica* rice	Transcrption factor genes-derived markers	Known cloned genes-derived markers	PMS markers corresponding with RM markers
PMS2678	Os_Chr05	980543	980562	(TA)10	(TA)10	(TA)10	(TA)6	(TA)10	(TA)10	(TA)10	(TA)10	(TA)6	(TA)10	(TA)10	-	Introns/Intergenic	-	-	TGGTATTAATTGTTGAGCACCG	GTCCGCTCGTTAATCCGATA	156	P	A	A	NA
PMS2679	Os_Chr05	1003835	1003848	(GA)7	(GA)7	(GA)7	(GA)7	(GA)7	(GA)7	(GA)6	(GA)7	(GA)7	(GA)7	(GA)7	-	Introns/Intergenic	-	-	CTACTTGAGGCCCGTTTCTG	AGCAGTAGCACAAGAGGGGA	155	A	A	A	NA
PMS2680	Os_Chr05	1049201	1049216	(GC)8	(GC)6	(GC)6	(GC)6	(GC)6	(GC)6	(GC)8	(GC)8	(GC)8	(GC)8	(GC)8	-	Introns/Intergenic	-	-	GACGCGAAGCTCTACAAACC	TGTTGTTCGATTCCGTTCCT	193	A	A	A	NA
PMS2681	Os_Chr05	1103131	1103144	(TC)7	(TC)7	(TC)7	(TC)7	(TC)7	(TC)7	(TC)7	(TC)7	(TC)10	(TC)7	(TC)7	-	Introns/Intergenic	-	-	CCCACATGGTCATCCTCTCT	GACCAGAACTCTAGTGCGGC	204	A	A	A	NA
PMS2682	Os_Chr05	1181020	1181057	(AT)19	(AT)19	(AT)19	(AT)19	(AT)19	(AT)19	(AT)7	(AT)19	(AT)6	(AT)7	(AT)19	-	Introns/Intergenic	-	-	GCAATACACGCCAATCTTCTAGG	TTGGGATGGAGGTAGTATTGTGC	210	A	A	A	RM2010
PMS2683	Os_Chr05	1231417	1231434	(TGG)6	(TGG)6	(TGG)6	(TGG)6	(TGG)6	(TGG)6	(TGG)6	(TGG)6	(TGG)6	(TGG)6	(TGG)5	LOC_Os05g03110	CDS	PWWP domain containing protein, expressed	CODON_DELETION	TGCACTTCTTGTGCACCTTC	ATCACTGGCAATGTGGATGA	240	A	A	A	NA
PMS2684	Os_Chr05	1258726	1258751	(TC)13	(TC)13	(TC)13	(TC)13	(TC)13	(TC)17	(TC)13	(TC)13	(TC)13	(TC)13	(TC)13	LOC_Os05g03140	DRR	tetraspanin family protein, putative, expressed	-	GGGTCATCGTCGTCCTCTCC	GCCTCGTCCTCTACCTTCTCTCC	217	P	A	A	RM5401
PMS2685	Os_Chr05	1457063	1457080	(TC)9	(TC)9	(TC)9	(TC)9	(TC)9	(TC)9	(TC)9	(TC)9	(TC)9	(TC)9	(TC)8	LOC_Os05g03450	DRR	lectin protein kinase family protein, putative, expressed	-	CCGGTGAATTTTACTGCCTT	TGCGAGAATAATGGATTACTGG	231	A	A	A	NA
PMS2686	Os_Chr05	1464350	1464379	(TA)15	(TA)15	(TA)15	(TA)15	(TA)15	(TA)15	(TA)8	(TA)15	(TA)15	(TA)9	(TA)15	-	Introns/Intergenic	-	-	TGATAGGCATCCATAGTGCAA	TTGGTTTTAAATCGGACGTTCT	250	A	A	A	NA
PMS2687	Os_Chr05	1465197	1465212	(TA)8	(TA)8	(TA)8	(TA)8	(TA)8	(TA)8	(TA)8	(TA)8	(TA)8	(TA)8	(TA)7	-	Introns/Intergenic	-	-	AAAATTTGGGCGAATTTGTG	TGCCAATATGGGTCATCAGA	221	A	A	A	NA
PMS2688	Os_Chr05	1490324	1490341	(GCG)6	(GCG)6	(GCG)6	(GCG)6	(GCG)6	(GCG)6	(GCG)6	(GCG)5	(GCG)6	(GCG)6	(GCG)6	LOC_Os05g03520	CDS	expressed protein	CODON_DELETION	ACGTCATGGAAAACACCCTC	AAGCAGTAGGTGCCCACATC	244	A	A	A	NA
PMS2689	Os_Chr05	1507419	1507436	(CCA)6	(CCA)6	(CCA)6	(CCA)5	(CCA)6	(CCA)6	(CCA)6	(CCA)6	(CCA)6	(CCA)6	(CCA)6	LOC_Os05g03550	CDS	MYB family transcription factor, putative, expressed	CODON_DELETION	TGGTTTCTGGAGAAGTCGCT	GCTCTGCTATTTCAGACGGC	259	P	P	A	NA
PMS2690	Os_Chr05	1507896	1507916	(GTC)7	(GTC)7	(GTC)7	(GTC)7	(GTC)7	(GTC)6	(GTC)7	(GTC)7	(GTC)7	(GTC)7	(GTC)7	-	Introns/Intergenic	-	-	CTGTACAGGAAGTCCCTCCG	TGGATTTCTGGTGGTTGGTT	235	P	A	A	NA

Polymorphic SSR (PMS) markers	Chromosomes	Start physical positions (bp)	End physical positions (bp)	Nipponbare	Kasalath	Nagina22	IR64	Pokkali	Bala	Tainung67	Azucena	Moroberekan	*Oryza rufipogon*	*Oryza nivara*	MSU gene locus IDs	Structural annotation	Functional annotation	SSR functional effects	Forward primers (5'-3')	Reverse primers (5'-3')	Amplified product size (bp)	Markers exhibiting polymorphism within *indica* rice	Transcrption factor genes-derived markers	Known cloned genes-derived markers	PMS markers corresponding with RM markers
PMS2691	Os_Chr05	1510598	1510611	(TC)7	(TC)9	(TC)7	(TC)7	(TC)9	(TC)9	(TC)7	(TC)7	(TC)7	(TC)7	(TC)7	-	Introns/Intergenic	-	-	TGATTTTGGTCTACGTGGCA	CACGATGTCGTCGTCTGC	222	P	A	A	NA
PMS2692	Os_Chr05	1522667	1522702	(CTT)12	(CTT)11	(CTT)12	(CTT)12	(CTT)12	(CTT)11	(CTT)12	(CTT)12	(CTT)12	(CTT)12	(CTT)11	LOC_Os05g03590	URR	expressed protein	-	GGAGACAGTGGAGAGGCTACTGG	CATCATCAACTACCAACCCATCC	190	P	A	A	RM6317
PMS2693	Os_Chr05	1645149	1645180	(CTAT)8	(CTAT)8	(CTAT)8	(CTAT)8	(CTAT)8	(CTAT)8	(CTAT)8	(CTAT)8	(CTAT)8	(CTAT)8	(CTAT)6	-	Introns/Intergenic	-	-	CATGCCTTTGCATGATCAATCG	GGTGGACTCATGGTGAGAAGAGAGG	100	A	A	A	RM17822
PMS2694	Os_Chr05	1646513	1646542	(AT)15	(AT)15	(AT)15	(AT)7	(AT)15	(AT)15	(AT)15	(AT)15	(AT)15	(AT)15	(AT)15	-	Introns/Intergenic	-	-	AATCGTGCGTGGTCAGTAACG	GGAATCAAGTGCAGATGGACTAAGC	192	P	A	A	RM17824
PMS2695	Os_Chr05	1663222	1663239	(GGC)6	(GGC)6	(GGC)6	(GGC)6	(GGC)6	(GGC)6	(GGC)6	(GGC)6	(GGC)6	(GGC)5	(GGC)6	LOC_Os05g03760	CDS	zinc finger family protein, putative, expressed	CODON_DELETION	ATCTGCTTCTTCGCCCACA	GCTTCTGCTGGTACTGGTCC	253	A	P	A	NA
PMS2696	Os_Chr05	1709082	1709111	(CTT)10	(CTT)9	(CTT)10	(CTT)10	(CTT)10	(CTT)9	(CTT)10	(CTT)10	(CTT)10	(CTT)10	(CTT)10	-	Introns/Intergenic	-	-	TCACCACCAACTCTATCGCA	GGATTCAAGCGTGTTCATCA	179	P	A	A	NA
PMS2697	Os_Chr05	1720021	1720042	(CT)11	(CT)12	(CT)11	(CT)12	(CT)11	(CT)11	(CT)11	(CT)11	(CT)11	(CT)11	(CT)11	-	Introns/Intergenic	-	-	CCCACCATTTTCTTTGTTCC	TCTCCACCAATCATTCCGAT	206	P	A	A	NA
PMS2698	Os_Chr05	1771725	1771756	(TA)16	(TA)16	(TA)7	(TA)16	(TA)16	(TA)16	(TA)6	(TA)16	(TA)16	(TA)16	(TA)16	-	Introns/Intergenic	-	-	TCATGCATGTGGCATTTACA	AAGTGGATAAATCGTAATAAGCAAA	267	P	A	A	NA
PMS2699	Os_Chr05	1833091	1833108	(AGC)6	(AGC)6	(AGC)5	(AGC)6	(AGC)6	(AGC)6	(AGC)6	(AGC)6	(AGC)6	(AGC)6	(AGC)6	-	Introns/Intergenic	-	-	GAAGAAGGCGAGGAGGAAGT	TCGTTGTTAAGCAAGATGCG	158	P	A	A	NA
PMS2700	Os_Chr05	1850552	1850567	(AG)8	(AG)9	(AG)8	(AG)8	(AG)8	(AG)8	(AG)8	(AG)8	(AG)8	(AG)8	(AG)8	-	Introns/Intergenic	-	-	TCAGAGGATCTGCTTCCACC	TAGGTCCTTCGGTGGTTTTG	204	P	A	A	NA
PMS2701	Os_Chr05	1920855	1920866	(TA)6	(TA)6	(TA)6	(TA)6	(TA)6	(TA)6	(TA)6	(TA)6	(TA)6	(TA)8	(TA)6	-	Introns/Intergenic	-	-	GGAGTCACGTCCATAGTTCCA	GTGGGTAGCAATGTCGGAAT	109	A	A	A	NA
PMS2702	Os_Chr05	1926869	1926886	(ACG)6	(ACG)7	(ACG)7	(ACG)7	(ACG)7	(ACG)7	(ACG)6	(ACG)7	(ACG)7	(ACG)7	(ACG)7	-	Introns/Intergenic	-	-	TCGGTCACAAACGAATTTCA	CGAGATCGGTTTGTTGGATT	144	A	A	A	NA
PMS2703	Os_Chr05	1996885	1996916	(CT)16	(CT)15	(CT)15	(CT)16	(CT)7	(CT)15	(CT)16	(CT)16	(CT)16	(CT)16	(CT)16	LOC_Os05g04360	DRR	protein phosphatase 2C, putative, expressed	-	GGACTAGCTTCCCTTGAGCA	TGGTCTTTCAAGAATGGGACA	116	P	A	A	NA

Polymorphic SSR (PMS) markers	Chromosomes	Start physical positions (bp)	End physical positions (bp)	Nipponbare	Kasalath	Nagina22	IR64	Pokkali	Bala	Tainung67	Azucena	Moroberekan	*Oryza rufipogon*	*Oryza nivara*	MSU gene locus IDs	Structural annotation	Functional annotation	SSR functional effects	Forward primers (5'-3')	Reverse primers (5'-3')	Amplified product size (bp)	Markers exhibiting polymorphism within *indica* rice	Transcrption factor genes-derived markers	Known cloned genes-derived markers	PMS markers corresponding with RM markers
PMS2704	Os_Chr05	2002340	2002354	(CAC)5	(CAC)6	(CAC)6	(CAC)6	(CAC)6	(CAC)6	(CAC)5	(CAC)6	(CAC)5	(CAC)6	(CAC)6	LOC_Os05g04370	CDS	expressed protein	CODON_INSERTION	ATGATCTCCACCTTCCTCCC	CACCGCGTGTACGACCTC	245	A	A	A	NA
PMS2705	Os_Chr05	2011788	2011817	(GA)15	(GA)15	(GA)15	(GA)15	(GA)15	(GA)14	(GA)15	(GA)15	(GA)15	(GA)15	(GA)14	-	Introns/Intergenic	-	-	CAGAGCTTCTTTGCTTCCTATGG	ATTCATGACTGAGGAGCTAGTGG	199	P	A	A	RM3345
PMS2706	Os_Chr05	2109476	2109537	(AT)31	(AT)31	(AT)31	(AT)8	(AT)31	(AT)31	(AT)31	(AT)6	(AT)6	(AT)31	(AT)31	-	Introns/Intergenic	-	-	TCAATTTGAACCATGGAGCC	GAGGGTGAGCTATGCTGAGG	235	P	A	A	NA
PMS2707	Os_Chr05	2137722	2137741	(TA)10	(TA)7	(TA)10	(TA)10	(TA)10	(TA)7	(TA)10	(TA)10	(TA)10	(TA)10	(TA)10	-	Introns/Intergenic	-	-	GTTCCAACGAGATCCCGTACAATAGC	CGCCGGTTTCTACACTATTTCTGACG	684	P	A	A	RM17860
PMS2708	Os_Chr05	2284385	2284411	(CCT)9	(CCT)9	(CCT)9	(CCT)9	(CCT)9	(CCT)8	(CCT)9	(CCT)9	(CCT)9	(CCT)9	(CCT)9	-	Introns/Intergenic	-	-	TCCAAGGGTATGCGAAAATC	CTGACGACGACGATGATGAG	187	P	A	A	NA
PMS2709	Os_Chr05	2286156	2286229	(TA)37	(TA)37	(TA)12	(TA)6	(TA)10	(TA)9	(TA)37	(TA)7	(TA)37	(TA)37	(TA)7	-	Introns/Intergenic	-	-	TCACTGTTCATACACGATCACG	ACAAGAAACTCACGACCGCT	154	P	A	A	NA
PMS2710	Os_Chr05	2289144	2289209	(AT)33	(AT)33	(AT)33	(AT)9	(AT)17	(AT)9	(AT)8	(AT)33	(AT)33	(AT)17	(AT)33	-	Introns/Intergenic	-	-	TCTAGGCGACATCAAGGTCC	TGCAACATTGGATTGTGAAA	271	P	A	A	NA
PMS2711	Os_Chr05	2296499	2296586	(TA)44	(TA)44	(TA)44	(TA)6	(TA)44	(TA)10	(TA)44	(TA)16	(TA)7	(TA)44	(TA)6	-	Introns/Intergenic	-	-	GAATCCCCACACACATATATTCA	TAATATAGGGCCACGCCAAC	257	P	A	A	NA
PMS2712	Os_Chr05	2502866	2502892	(GGC)9	(GGC)9	(GGC)9	(GGC)9	(GGC)9	(GGC)9	(GGC)6	(GGC)9	(GGC)9	(GGC)9	(GGC)9	LOC_Os05g05140	CDS	expressed protein	CODON_DELETION	ATCGAAGAGGCAACACGAAC	GATTGGGGGAATTCTTGGTT	260	A	A	A	NA
PMS2713	Os_Chr05	2564338	2564355	(GGA)6	(GGA)6	(GGA)6	(GGA)6	(GGA)6	(GGA)6	(GGA)6	(GGA)6	(GGA)6	(GGA)5	(GGA)6	LOC_Os05g05240	URR	OsPPKL2	-	CTTTGGTTACTACCGCCTCG	CCTTCCACCTCCCTGTACG	164	A	A	P	NA
PMS2714	Os_Chr05	2705244	2705264	(CGG)7	(CGG)7	(CGG)7	(CGG)7	(CGG)7	(CGG)7	(CGG)7	(CGG)5	(CGG)7	(CGG)7	(CGG)7	-	Introns/Intergenic	-	-	GGAGATCGGCGGAGATGATCG	CAAGATACGCCGACCAAATCACC	325	A	A	A	RM17890
PMS2715	Os_Chr05	2723131	2723162	(GA)16	(GA)11	(GA)16	(GA)16	(GA)16	(GA)11	(GA)16	(GA)16	(GA)16	(GA)16	(GA)16	-	Introns/Intergenic	-	-	GCATCTGCAAACCACAGAAA	ACGTGCTCAAGACCAACCTT	278	P	A	A	NA
PMS2716	Os_Chr05	2734150	2734170	(CGC)7	(CGC)7	(CGC)7	(CGC)7	(CGC)7	(CGC)7	(CGC)5	(CGC)7	(CGC)7	(CGC)7	(CGC)7	-	Introns/Intergenic	-	-	ACCGTTGCTTTCTGATCTGACG	AGGCAAGTCGGGAGTACTGTTCC	181	A	A	A	RM17893

Polymorphic SSR (PMS) markers	Chromosomes	Start physical positions (bp)	End physical positions (bp)	Nipponbare	Kasalath	Nagina22	IR64	Pokkali	Bala	Tainung67	Azucena	Moroberekan	*Oryza rufipogon*	*Oryza nivara*	MSU gene locus IDs	Structural annotation	Functional annotation	SSR functional effects	Forward primers (5'-3')	Reverse primers (5'-3')	Amplified product size (bp)	Markers exhibiting polymorphism within *indica* rice	Transcrption factor genes-derived markers	Known cloned genes-derived markers	PMS markers corresponding with RM markers
PMS2717	Os_Chr05	2738633	2738653	(CGG)7	(CGG)7	(CGG)7	(CGG)6	(CGG)7	(CGG)7	(CGG)6	(CGG)7	(CGG)7	(CGG)7	(CGG)7	LOC_Os05g05500	CDS	OsFBX159 - F-box domain containing protein, expressed	CODON_DELETION	GGGAGCAGGAGGAAGACCT	AGGAGACTTGGACTTGGTCG	171	P	A	A	NA
PMS2718	Os_Chr05	2798220	2798244	(GCCAG)5	(GCCAG)5	(GCCAG)5	(GCCAG)5	(GCCAG)5	(GCCAG)5	(GCCAG)6	(GCCAG)5	(GCCAG)5	(GCCAG)5	(GCCAG)5	-	Introns/Intergenic	-	-	GCAGCAAAACAAAACTGCAA	AGCCACTTGAAGCATGTGTG	203	A	A	A	NA
PMS2719	Os_Chr05	2969619	2969642	(CCG)8	(CCG)8	(CCG)8	(CCG)8	(CCG)8	(CCG)8	(CCG)8	(CCG)8	(CCG)8	(CCG)7	(CCG)6	LOC_Os05g05930	CDS	peripheral-type benzodiazepine receptor, putative, expressed	CODON_CHANGE_PLUS_CODON_DELETION	CGGATCAGAGCACAGCAGTA	GACCCGGAGCCGTAGAAC	244	A	A	A	NA
PMS2720	Os_Chr05	2972521	2972560	(AG)20	(AG)20	(AG)20	(AG)20	(AG)20	(AG)20	(AG)20	(AG)18	(AG)20	(AG)20	(AG)20	-	Introns/Intergenic	-	-	ACCACCACCACCAAGAACTC	CGCCTCAATTAAGTCTTCGC	261	A	A	A	NA
PMS2721	Os_Chr05	3042445	3042465	(GGC)7	(GGC)7	(GGC)7	(GGC)7	(GGC)7	(GGC)7	(GGC)7	(GGC)7	(GGC)7	(GGC)7	(GGC)5	LOC_Os05g06080	CDS	E3 ubiquitin-protein ligase SINA-like, putative, expressed	CODON_DELETION	CCTCTCTCCATCTGTATGCGTAACC	GAGGAAGAAGACAGACACCATCG	179	A	A	A	RM17910
PMS2722	Os_Chr05	3050777	3050794	(TTC)6	(TTC)6	(TTC)6	(TTC)6	(TTC)6	(TTC)6	(TTC)6	(TTC)5	(TTC)6	(TTC)6	(TTC)6	LOC_Os05g06110	URR	villin, putative, expressed	-	TAAAAAGCAACGCACGTCAG	GAAGCTGGTGGTGGTAGGAG	257	A	A	A	NA
PMS2723	Os_Chr05	3062635	3062732	(TA)49	(TA)49	(TA)49	(TA)28	(TA)49	(TA)21	(TA)49	(TA)49	(TA)49	(TA)35	(TA)49	-	Introns/Intergenic	-	-	ATTATGGGACGGATGGAGTAGG	TGCCCACTACCACTAGAGTATCG	502	P	A	A	RM17916
PMS2724	Os_Chr05	3101294	3101313	(AT)10	(AT)10	(AT)7	(AT)10	(AT)10	(AT)10	(AT)10	(AT)10	(AT)10	(AT)10	(AT)10	-	Introns/Intergenic	-	-	TGAAGCTTCTTGTTCACTGACTCC	GATCTTATGAACCTTCGCACAACG	331	P	A	A	RM17920
PMS2725	Os_Chr05	3125274	3125315	(AG)21	(AG)19	(AG)21	(AG)21	(AG)21	(AG)21	(AG)21	(AG)21	(AG)21	(AG)21	(AG)21	-	Introns/Intergenic	-	-	TAATGCCACTGGAAGTAAGCCACTGC	GAAACTTCCTCCATGGCTCCACTAGC	423	P	A	A	RM17922
PMS2726	Os_Chr05	3167882	3167901	(TA)10	(TA)10	(TA)8	(TA)10	(TA)6	(TA)6	(TA)10	(TA)7	(TA)10	(TA)10	(TA)10	-	Introns/Intergenic	-	-	TAGTTATTGCGCACCTCATACGC	AAACACAGATCCTTCTCCTTGTCC	196	P	A	A	RM17927
PMS2727	Os_Chr05	3206149	3206166	(TC)9	(TC)8	(TC)8	(TC)8	(TC)8	(TC)8	(TC)9	(TC)9	(TC)9	(TC)9	(TC)8	-	Introns/Intergenic	-	-	CAAATTGCCCGCTTACAAAT	CTAGCACAAGTCCATTGCCA	224	A	A	A	NA
PMS2728	Os_Chr05	3366132	3366149	(AG)9	(AG)6	(AG)9	(AG)9	(AG)9	(AG)9	(AG)9	(AG)9	(AG)9	(AG)9	(AG)9	-	Introns/Intergenic	-	-	TAGGACCAACCCAAAACGAC	ATGGCATGGTTCCAATTTTC	272	P	A	A	NA
PMS2729	Os_Chr05	3384037	3384084	(GGTGAT)8	(GGTGAT)9	(GGTGAT)9	(GGTGAT)8	(GGTGAT)8	(GGTGAT)8	(GGTGAT)8	(GGTGAT)8	(GGTGAT)8	(GGTGAT)8	(GGTGAT)8	LOC_Os05g06560	CDS	expressed protein	CODON_INSERTION	GTAAAGCCGGATACGCAATC	ATCGTCATTCTCCTCGTCGT	249	P	A	A	NA

Polymorphic SSR (PMS) markers	Chromosomes	Start physical positions (bp)	End physical positions (bp)	Nipponbare	Kasalath	Nagina22	IR64	Pokkali	Bala	Tainung67	Azucena	Moroberekan	*Oryza rufipogon*	*Oryza nivara*	MSU gene locus IDs	Structural annotation	Functional annotation	SSR functional effects	Forward primers (5'-3')	Reverse primers (5'-3')	Amplified product size (bp)	Markers exhibiting polymorphism within *indica* rice	Transcrption factor genes-derived markers	Known cloned genes-derived markers	PMS markers corresponding with RM markers
PMS2730	Os_Chr05	3391998	3392023	(TA)13	(TA)13	(TA)7	(TA)13	(TA)13	(TA)13	(TA)13	(TA)13	(TA)13	(TA)13	(TA)13	-	Introns/Intergenic	-	-	TTTGCCTTCTGTGTGTCTGG	CCCAGGTTGCCTTGATAGAA	267	P	A	A	NA
PMS2731	Os_Chr05	3450959	3450980	(CT)11	(CT)11	(CT)11	(CT)10	(CT)11	(CT)10	(CT)11	(CT)11	(CT)11	(CT)11	(CT)11	-	Introns/Intergenic	-	-	AAACTAGCCACGGTTTGGTAGGG	AGGGTGGCAGGGATGTAATTTCC	267	P	A	A	RM574
PMS2732	Os_Chr05	3502640	3502673	(AG)17	(AG)16	(AG)16	(AG)17	(AG)17	(AG)16	(AG)17	(AG)17	(AG)17	(AG)17	(AG)16	-	Introns/Intergenic	-	-	GTTCTTGCCTTCTCGGCATGTGG	GGTCACTGATTCCACCATTCACACC	192	P	A	A	RM17947
PMS2733	Os_Chr05	3506879	3506902	(GGA)8	(GGA)8	(GGA)8	(GGA)8	(GGA)8	(GGA)8	(GGA)8	(GGA)8	(GGA)8	(GGA)8	(GGA)6	-	Introns/Intergenic	-	-	AAAAACAGAACGCATACGCA	GGGTGGTGGTGTTCAACTTC	261	A	A	A	NA
PMS2734	Os_Chr05	3522047	3522060	(AG)7	(AG)7	(AG)7	(AG)8	(AG)8	(AG)8	(AG)7	(AG)7	(AG)7	(AG)7	(AG)7	-	Introns/Intergenic	-	-	AACAGTGCCGGAAGAAAAGA	ATTTCAGCCGTCCATTTCAG	271	P	A	A	NA
PMS2735	Os_Chr05	3571253	3571267	(CCG)5	(CCG)6	(CCG)5	(CCG)5	(CCG)5	(CCG)6	(CCG)5	(CCG)5	(CCG)5	(CCG)5	(CCG)5	LOC_Os05g06814	CDS	expressed protein	CODON_INSERTION	CAGCAGCACTTGCATCTCC	ACTCCACACTCCACAGTCCC	254	P	A	A	NA
PMS2736	Os_Chr05	3572268	3572279	(TC)6	(TC)7	(TC)7	(TC)7	(TC)7	(TC)7	(TC)6	(TC)6	(TC)6	(TC)6	(TC)6	-	Introns/Intergenic	-	-	TAATTTGTGTGGGGGAGACG	GGCCTCTCCTCCATTTATCC	203	A	A	A	NA
PMS2737	Os_Chr05	3580597	3580614	(CT)9	(CT)7	(CT)9	(CT)9	(CT)9	(CT)9	(CT)9	(CT)9	(CT)9	(CT)9	(CT)9	LOC_Os05g06840	URR	POLE2A - Putative DNA polymerase epsilon complex subunit, expressed	-	ACAGGCGATCGGTACATTTC	AGCCTCGCAGCTTGAACTT	179	P	A	A	NA
PMS2738	Os_Chr05	3607734	3607757	(CGT)8	(CGT)8	(CGT)8	(CGT)8	(CGT)8	(CGT)8	(CGT)8	(CGT)7	(CGT)8	(CGT)8	(CGT)8	LOC_Os05g06890	CDS	relA-SpoT like protein RSH4, putative, expressed	CODON_CHANGE_PLUS_CODON_DELETION	CCACGTCGCCTCCAATTCATCC	TCGAGCTACAGGATGCAATCATTCG	491	A	A	A	RM17953
PMS2739	Os_Chr05	3703177	3703188	(TA)6	(TA)7	(TA)6	(TA)6	(TA)6	(TA)6	(TA)6	(TA)6	(TA)6	(TA)6	(TA)6	-	Introns/Intergenic	-	-	TGAATAAGACGAAAGGTCAAACG	TCCATTAACCATTAGCGCATC	226	P	A	A	NA
PMS2740	Os_Chr05	3759467	3759487	(GGA)7	(GGA)7	(GGA)7	(GGA)7	(GGA)7	(GGA)7	(GGA)7	(GGA)5	(GGA)7	(GGA)7	(GGA)7	LOC_Os05g07120	CDS	basic helix-loop-helix, putative, expressed	CODON_CHANGE_PLUS_CODON_DELETION	GAGGTGACCGTCCAATGCTACG	AGTAACGCGACTCCATCATCAGC	352	A	P	A	RM17955
PMS2741	Os_Chr05	3761558	3761584	(TAT)9	(TAT)5	(TAT)9	(TAT)9	(TAT)9	(TAT)9	(TAT)9	(TAT)11	(TAT)9	(TAT)9	(TAT)5	-	Introns/Intergenic	-	-	AGATCTCGTTCATCCCAAGAAGG	TGACCCATGACAGTGAAGAGAGC	394	P	A	A	RM17956
PMS2742	Os_Chr05	3782974	3782987	(GA)7	(GA)8	(GA)7	(GA)8	(GA)8	(GA)8	(GA)7	(GA)7	(GA)7	(GA)8	(GA)7	-	Introns/Intergenic	-	-	TCAATTCCTTTTGACCCCAA	AGGAGGACGAGGGCAATAGT	233	P	A	A	NA

Polymorphic SSR (PMS) markers	Chromosomes	Start physical positions (bp)	End physical positions (bp)	Nipponbare	Kasalath	Nagina22	IR64	Pokkali	Bala	Tainung67	Azucena	Moroberekan	*Oryza rufipogon*	*Oryza nivara*	MSU gene locus IDs	Structural annotation	Functional annotation	SSR functional effects	Forward primers (5'-3')	Reverse primers (5'-3')	Amplified product size (bp)	Markers exhibiting polymorphism within *indica* rice	Transcrption factor genes-derived markers	Known cloned genes-derived markers	PMS markers corresponding with RM markers
PMS2743	Os_Chr05	3806505	3806576	(ATT)24	(ATT)24	(ATT)18	(ATT)24	(ATT)24	(ATT)24	(ATT)24	(ATT)24	(ATT)24	(ATT)24	(ATT)19	-	Introns/Intergenic	-	-	GAGCGAACCTAGGCCAAACT	TTTGATCCTTGTTCGCACG	257	P	A	A	NA
PMS2744	Os_Chr05	3858327	3858341	(CGC)5	(CGC)6	(CGC)5	(CGC)5	(CGC)5	(CGC)6	(CGC)5	(CGC)6	(CGC)5	(CGC)5	(CGC)5	LOC_Os05g07280	CDS	expressed protein	CODON_INSERTION	TGGCGTACAAAATGCAAAGA	AGAGGGGGCTCGTGAAAG	213	P	A	A	NA
PMS2745	Os_Chr05	4093811	4093828	(CCG)6	(CCG)6	(CCG)6	(CCG)6	(CCG)6	(CCG)6	(CCG)6	(CCG)6	(CCG)6	(CCG)5	(CCG)6	LOC_Os05g07626	CDS	expressed protein	CODON_DELETION	GTCATCATGTTGGCGATCAG	ATCGTCAACACCTGCACCTT	134	A	A	A	NA
PMS2746	Os_Chr05	4151164	4151179	(TA)8	(TA)7	(TA)8	(TA)8	(TA)8	(TA)7	(TA)8	(TA)8	(TA)8	(TA)8	(TA)7	-	Introns/Intergenic	-	-	TTATGTCACGCAATGTGCAA	TGCAAATAACCTTGGGTGGT	222	P	A	A	NA
PMS2747	Os_Chr05	4226450	4226470	(GCT)7	(GCT)7	(GCT)7	(GCT)7	(GCT)7	(GCT)7	(GCT)7	(GCT)7	(GCT)7	(GCT)6	(GCT)7	LOC_Os05g07840	CDS	expressed protein	CODON_DELETION	CGGCTCTGATCCTTCCAGTGC	GACACTGATCATCGTCCCTCTCG	195	A	A	A	RM17973
PMS2748	Os_Chr05	4310544	4310561	(GGC)6	(GGC)6	(GGC)6	(GGC)6	(GGC)5	(GGC)6	(GGC)6	(GGC)6	(GGC)6	(GGC)6	(GGC)6	-	Introns/Intergenic	-	-	TTGGATTTCTCTCTCGCCAT	ACACTCTCTCTCCCCCTCGT	218	P	A	A	NA
PMS2749	Os_Chr05	4489518	4489571	(TA)27	(TA)20	(TA)9	(TA)27	(TA)27	(TA)27	(TA)27	(TA)27	(TA)27	(TA)9	(TA)27	-	Introns/Intergenic	-	-	AGGCAAACTTATGCCGAGAAGG	TGTCCTGTTACTGCACGATGACC	288	P	A	A	RM17979
PMS2750	Os_Chr05	4493652	4493709	(TA)29	(TA)29	(TA)11	(TA)12	(TA)12	(TA)20	(TA)16	(TA)10	(TA)29	(TA)29	(TA)29	-	Introns/Intergenic	-	-	GAAGGGGATTTTGGACCCTA	TGCACACGTATGGACTTGGT	234	P	A	A	NA
PMS2751	Os_Chr05	4498392	4498417	(TA)13	(TA)13	(TA)8	(TA)13	(TA)13	(TA)13	(TA)13	(TA)12	(TA)8	(TA)13	(TA)13	-	Introns/Intergenic	-	-	GTTGCGCCATTTAGTTTCGT	CATGGCAAAACTTTTCGGTT	250	P	A	A	NA
PMS2752	Os_Chr05	4559281	4559301	(GCG)7	(GCG)7	(GCG)7	(GCG)7	(GCG)7	(GCG)7	(GCG)7	(GCG)5	(GCG)7	(GCG)7	(GCG)7	LOC_Os05g08360	URR	rRNA 2-O-methyltransferase fibrillarin 2, putative, expressed	-	ATCAACACACCCTAGCCGTC	TAAGGGTGGAAGTGGAATCG	267	A	A	A	NA
PMS2753	Os_Chr05	4610483	4610498	(CT)8	(CT)8	(CT)8	(CT)8	(CT)8	(CT)8	(CT)7	(CT)8	(CT)8	(CT)8	(CT)8	-	Introns/Intergenic	-	-	AAACAATCAAGCGAAGTCCG	CTCCTCTCGTCGATTTGCTC	224	A	A	A	NA
PMS2754	Os_Chr05	4803126	4803146	(CCG)7	(CCG)5	(CCG)7	(CCG)7	(CCG)7	(CCG)7	(CCG)7	(CCG)7	(CCG)6	(CCG)7	(CCG)7	-	Introns/Intergenic	-	-	CCTCCCGCTAGATCCTCC	TCTCATCTCTCTCTTCGCCG	278	P	A	A	NA
PMS2755	Os_Chr05	4803938	4803977	(TGTA)10	(TGTA)8	(TGTA)10	(TGTA)10	(TGTA)10	(TGTA)10	(TGTA)10	(TGTA)10	(TGTA)10	(TGTA)10	(TGTA)5	-	Introns/Intergenic	-	-	GATCTGTACGCTTCCCGGT	GGGACTCCGAACACAAGAAA	114	P	A	A	NA

Polymorphic SSR (PMS) markers	Chromosomes	Start physical positions (bp)	End physical positions (bp)	Nipponbare	Kasalath	Nagina22	IR64	Pokkali	Bala	Tainung67	Azucena	Moroberekan	*Oryza rufipogon*	*Oryza nivara*	MSU gene locus IDs	Structural annotation	Functional annotation	SSR functional effects	Forward primers (5'-3')	Reverse primers (5'-3')	Amplified product size (bp)	Markers exhibiting polymorphism within *indica* rice	Transcrption factor genes-derived markers	Known cloned genes-derived markers	PMS markers corresponding with RM markers
PMS2756	Os_Chr05	4926334	4926365	(CT)16	(CT)12	(CT)16	(CT)16	(CT)16	(CT)16	(CT)16	(CT)16	(CT)16	(CT)16	(CT)16	-	Introns/Intergenic	-	-	GAACACTAGGCGCATCCATTCC	ATTAGGGAGCGTTGGATTGTTTCC	178	P	A	A	RM17998
PMS2757	Os_Chr05	4937388	4937405	(GGC)6	(GGC)6	(GGC)6	(GGC)6	(GGC)6	(GGC)6	(GGC)6	(GGC)6	(GGC)6	(GGC)5	(GGC)6	LOC_Os05g08920	URR	late embryogenesis abundant protein, putative, expressed	-	GTTCACCTCGTCGACATCCT	GAGAACAACTTGCCGAAAGC	277	A	A	A	NA
PMS2758	Os_Chr05	4951769	4951789	(GGA)7	(GGA)7	(GGA)5	(GGA)6	(GGA)7	(GGA)7	(GGA)7	(GGA)7	(GGA)7	(GGA)6	(GGA)7	LOC_Os05g08950	CDS	phosphoribosyl transferase, putative, expressed	CODON_DELETION	GAGCTAGGGTTAGGGTTCCG	CTTCCATCGTGATGGTGTTG	205	P	A	A	NA
PMS2759	Os_Chr05	4965460	4965483	(CT)12	(CT)12	(CT)12	(CT)12	(CT)12	(CT)12	(CT)11	(CT)12	(CT)12	(CT)12	(CT)12	LOC_Os05g08970	URR	SSRP1-like FACT complex subunit, putative, expressed	-	GGGGCCGATATTTTACACCT	CGACGTGGAGGAGAGAAGAG	240	A	A	A	NA
PMS2760	Os_Chr05	5012082	5012095	(CG)7	(CG)6	(CG)7	(CG)7	(CG)7	(CG)7	(CG)7	(CG)7	(CG)7	(CG)7	(CG)7	LOC_Os05g09030	CDS	transposon protein, putative, unclassified, expressed	FRAME_SHIFT	GTGCTAGCGAGTTACCAGGC	AAAAGTAAAGATTGGCGGGG	273	P	A	A	NA
PMS2761	Os_Chr05	5031077	5031090	(TA)7	(TA)11	(TA)7	(TA)7	(TA)7	(TA)7	(TA)7	(TA)7	(TA)7	(TA)7	(TA)7	-	Introns/Intergenic	-	-	TTTACGTGTGACGTCGTTGG	GATGTCGGTCACAACCTCCT	276	P	A	A	NA
PMS2762	Os_Chr05	5166354	5166383	(TA)15	(TA)14	(TA)15	(TA)15	(TA)15	(TA)15	(TA)15	(TA)15	(TA)15	(TA)15	(TA)15	-	Introns/Intergenic	-	-	CTTCACTCGTTGTAACGCACA	AAACTCAAAGTGGGCAGTGG	198	P	A	A	NA
PMS2763	Os_Chr05	5167372	5167397	(AT)13	(AT)13	(AT)13	(AT)13	(AT)13	(AT)9	(AT)13	(AT)13	(AT)13	(AT)13	(AT)13	-	Introns/Intergenic	-	-	CACCATGAAGGGAACCAATC	CGAATTCTGTGTGGATGACG	216	P	A	A	NA
PMS2764	Os_Chr05	5203893	5203924	(AT)16	(AT)16	(AT)16	(AT)16	(AT)16	(AT)16	(AT)16	(AT)16	(AT)16	(AT)16	(AT)15	-	Introns/Intergenic	-	-	GGCAAGTGATGTAAAGAAGTG	GGTCATATTTGCTAAGCGTAA	300	A	A	A	NA
PMS2765	Os_Chr05	5262037	5262057	(CGG)7	(CGG)7	(CGG)7	(CGG)7	(CGG)7	(CGG)7	(CGG)5	(CGG)7	(CGG)7	(CGG)7	(CGG)7	LOC_Os05g09370	URR	kinase, pfkB family, putative, expressed	-	CGTGGTTCTAGAGATGAGAAAGAGG	GGTACTACAGTCCCAGAAGAAAGTGG	235	A	A	A	RM18019
PMS2766	Os_Chr05	5292421	5292436	(AT)8	(AT)6	(AT)8	(AT)8	(AT)8	(AT)8	(AT)8	(AT)8	(AT)8	(AT)8	(AT)8	-	Introns/Intergenic	-	-	TTTGACTCTCTCGCGGTTTT	AAGAAGGGGAGGTGAGAGGA	222	P	A	A	NA
PMS2767	Os_Chr05	5305063	5305090	(GGTG)7	(GGTG)7	(GGTG)7	(GGTG)7	(GGTG)7	(GGTG)7	(GGTG)5	(GGTG)7	(GGTG)7	(GGTG)7	(GGTG)7	-	Introns/Intergenic	-	-	AGCACCTGACCTGACCTGACTCC	GACCTGACCGGCTTAAACTATCTCC	244	A	A	A	RM18032
PMS2768	Os_Chr05	5310445	5310462	(TC)9	(TC)8	(TC)9	(TC)9	(TC)9	(TC)9	(TC)9	(TC)9	(TC)9	(TC)9	(TC)9	-	Introns/Intergenic	-	-	CCTTTCCTTGCTTGCATCTC	CACCAATTGATTCCATGCAG	126	P	A	A	NA

Polymorphic SSR (PMS) markers	Chromosomes	Start physical positions (bp)	End physical positions (bp)	Nipponbare	Kasalath	Nagina22	IR64	Pokkali	Bala	Tainung67	Azucena	Moroberekan	*Oryza rufipogon*	*Oryza nivara*	MSU gene locus IDs	Structural annotation	Functional annotation	SSR functional effects	Forward primers (5'-3')	Reverse primers (5'-3')	Amplified product size (bp)	Markers exhibiting polymorphism within *indica* rice	Transcrption factor genes-derived markers	Known cloned genes-derived markers	PMS markers corresponding with RM markers
PMS2769	Os_Chr05	5356223	5356288	(AC)33	(AC)32	(AC)33	(AC)33	(AC)33	(AC)33	(AC)33	(AC)11	(AC)33	(AC)6	(AC)33	-	Introns/Intergenic	-	-	TCGATACCCCAAAAGAAATCC	GCAAAGATTAAAGCATTTCAAGG	279	P	A	A	NA
PMS2770	Os_Chr05	5438041	5438054	(AC)7	(AC)6	(AC)6	(AC)7	(AC)7	(AC)7	(AC)7	(AC)7	(AC)7	(AC)7	(AC)6	-	Introns/Intergenic	-	-	TTTGGCAGATAAAGGTTGGC	TGGGCTCAGGGAAATGATAG	204	P	A	A	NA
PMS2771	Os_Chr05	5446882	5446896	(CCT)5	(CCT)6	(CCT)6	(CCT)5	(CCT)5	(CCT)5	(CCT)5	(CCT)5	(CCT)6	(CCT)5	(CCT)5	LOC_Os05g09640	CDS	uncharacterized protein At4g06744 precursor, putative, expressed	CODON_CHANGE_PLUS_CODON_INSERTION	TCAATCAAAGCATGCAAAGC	GCCTCGTACTCCTGGCTCT	272	P	A	A	NA
PMS2772	Os_Chr05	5503055	5503116	(TA)31	(TA)31	(TA)15	(TA)31	(TA)8	(TA)10	(TA)31	(TA)31	(TA)8	(TA)28	(TA)8	-	Introns/Intergenic	-	-	TTGACCACCATGAGCTACCA	CCGGGTGGTGTGAACTTATT	202	P	A	A	NA
PMS2773	Os_Chr05	5517936	5517947	(TA)6	(TA)6	(TA)6	(TA)6	(TA)6	(TA)6	(TA)6	(TA)8	(TA)6	(TA)6	(TA)6	-	Introns/Intergenic	-	-	TCGATACCCCAAAAGAAATCC	ATGCATGCACGCCTTATATT	231	A	A	A	NA
PMS2774	Os_Chr05	5526595	5526606	(TC)6	(TC)6	(TC)6	(TC)6	(TC)6	(TC)6	(TC)6	(TC)7	(TC)6	(TC)6	(TC)7	-	Introns/Intergenic	-	-	ACGAGCAAACCTGAGACCAA	GTGCACCATGGAGTCCCATA	226	A	A	A	NA
PMS2775	Os_Chr05	5592371	5592388	(TC)9	(TC)9	(TC)9	(TC)9	(TC)9	(TC)9	(TC)9	(TC)9	(TC)9	(TC)9	(TC)7	-	Introns/Intergenic	-	-	ATGCAAACCGGACGAAATAC	GTCGTCATCATCCTCATCCC	250	A	A	A	NA
PMS2776	Os_Chr05	5623034	5623069	(TA)18	(TA)18	(TA)6	(TA)18	(TA)18	(TA)18	(TA)18	(TA)18	(TA)18	(TA)9	(TA)6	-	Introns/Intergenic	-	-	TGAATCGGGTATATAAACTTTTGAC	GGCACCGCATTACTTTCCTA	280	P	A	A	NA
PMS2777	Os_Chr05	5751023	5751062	(TA)20	(TA)20	(TA)20	(TA)10	(TA)20	(TA)20	(TA)20	(TA)20	(TA)20	(TA)20	(TA)20	-	Introns/Intergenic	-	-	GTTTCTGTCCAAGGGAAATCAGC	AGGACCATTAACCTGAGGATTGG	325	P	A	A	RM18047
PMS2778	Os_Chr05	5853764	5853783	(AC)10	(AC)10	(AC)10	(AC)10	(AC)10	(AC)10	(AC)10	(AC)9	(AC)9	(AC)10	(AC)9	-	Introns/Intergenic	-	-	GGAGACAAAGAGAGGAGGGC	CCTTCCTTAGGTCTCCCGAC	172	A	A	A	NA
PMS2779	Os_Chr05	5983047	5983062	(CT)8	(CT)10	(CT)8	(CT)8	(CT)8	(CT)9	(CT)8	(CT)9	(CT)9	(CT)9	(CT)9	-	Introns/Intergenic	-	-	TGGCCTTGTGTTCTTCAGTG	GCTTGATGCATTGACTCGAA	232	P	A	A	NA
PMS2780	Os_Chr05	5997224	5997241	(GCG)6	(GCG)7	(GCG)6	(GCG)6	(GCG)6	(GCG)6	(GCG)6	(GCG)7	(GCG)6	(GCG)6	(GCG)6	LOC_Os05g10840	CDS	calmodulin-binding protein, putative, expressed	CODON_INSERTION	ACAGGTTGACGTGCTCCAG	AAAGAAGACGAGCACAAGGC	175	P	A	A	NA
PMS2781	Os_Chr05	6032104	6032139	(ATAG)9	(ATAG)9	(ATAG)9	(ATAG)9	(ATAG)9	(ATAG)9	(ATAG)9	(ATAG)9	(ATAG)9	(ATAG)5	(ATAG)9	-	Introns/Intergenic	-	-	CCACAGTGAACGTGGTGCTTCC	ATAACACGATTGCGACAGTGAACAGC	411	A	A	A	RM18064

Polymorphic SSR (PMS) markers	Chromosomes	Start physical positions (bp)	End physical positions (bp)	Nipponbare	Kasalath	Nagina22	IR64	Pokkali	Bala	Tainung67	Azucena	Moroberekan	*Oryza rufipogon*	*Oryza nivara*	MSU gene locus IDs	Structural annotation	Functional annotation	SSR functional effects	Forward primers (5'-3')	Reverse primers (5'-3')	Amplified product size (bp)	Markers exhibiting polymorphism within *indica* rice	Transcrption factor genes-derived markers	Known cloned genes-derived markers	PMS markers corresponding with RM markers
PMS2782	Os_Chr05	6109914	6109931	(CT)9	(CT)10	(CT)9	(CT)9	(CT)9	(CT)9	(CT)9	(CT)9	(CT)9	(CT)9	(CT)9	-	Introns/Intergenic	-	-	TCCTCCTCCTTCCTCTCCTC	ACGCACGTGACATGATGTTT	228	P	A	A	NA
PMS2783	Os_Chr05	6207712	6207726	(CGG)5	(CGG)5	(CGG)5	(CGG)5	(CGG)5	(CGG)6	(CGG)5	(CGG)5	(CGG)5	(CGG)5	(CGG)5	LOC_Os05g11070	CDS	helix-loop-helix DNA-binding domain containing protein, expressed	CODON_INSERTION	GACAGAGGGCGTTCAAGAAG	CTATCGTTGAGCTTCTCCCG	199	P	P	A	NA
PMS2784	Os_Chr05	6211985	6212002	(GAT)6	(GAT)6	(GAT)6	(GAT)6	(GAT)6	(GAT)6	(GAT)6	(GAT)5	(GAT)5	(GAT)6	(GAT)6	-	Introns/Intergenic	-	-	TTTGGACTGGCATTACATGG	TGGAGCTAGCTGAAAAACCC	225	A	A	A	NA
PMS2785	Os_Chr05	6270833	6270852	(AT)10	(AT)10	(AT)10	(AT)10	(AT)10	(AT)10	(AT)10	(AT)10	(AT)6	(AT)10	(AT)10	-	Introns/Intergenic	-	-	CGGATTAATAACACTTGTCATTTTG	TTTTGCACAAGGCTGATGTC	227	A	A	A	NA
PMS2786	Os_Chr05	6316577	6316594	(CT)9	(CT)7	(CT)9	(CT)9	(CT)9	(CT)9	(CT)9	(CT)9	(CT)9	(CT)11	(CT)9	-	Introns/Intergenic	-	-	TGTCGATGGAGCATGGTTTA	AAAGAGACGCGAGAGATGGA	212	P	A	A	NA
PMS2787	Os_Chr05	6349317	6349334	(GAC)6	(GAC)5	(GAC)6	(GAC)6	(GAC)6	(GAC)6	(GAC)6	(GAC)6	(GAC)6	(GAC)6	(GAC)6	LOC_Os05g11250	CDS	lachrymatory factor synthase, putative, expressed	CODON_DELETION	ACCTCCGCGAGCTAAGGAT	GTTGACATCCACGTCCTCG	220	P	P	A	NA
PMS2788	Os_Chr05	6349653	6349670	(GCC)6	(GCC)6	(GCC)6	(GCC)6	(GCC)6	(GCC)6	(GCC)6	(GCC)6	(GCC)6	(GCC)6	(GCC)5	LOC_Os05g11250	CDS	lachrymatory factor synthase, putative, expressed	CODON_DELETION	CGTGAAGGGGTGGACAAG	GTCTTCTACGGCAATGTCGG	118	A	P	A	NA
PMS2789	Os_Chr05	6376391	6376404	(CT)7	(CT)7	(CT)7	(CT)7	(CT)7	(CT)7	(CT)7	(CT)8	(CT)8	(CT)7	(CT)7	-	Introns/Intergenic	-	-	GCTAATGCATGCTCACCTCA	CACAGCTTCAAACATTCTTTCG	105	A	A	A	NA
PMS2790	Os_Chr05	6380566	6380583	(CGG)6	(CGG)8	(CGG)6	(CGG)6	(CGG)6	(CGG)6	(CGG)6	(CGG)8	(CGG)7	(CGG)8	(CGG)6	LOC_Os05g11300	CDS	expressed protein	CODON_INSERTION	GTAGTGGCAGTGGCTTCCTC	ACACCCAGTCTCCGTAGTGC	163	P	P	A	NA
PMS2791	Os_Chr05	6380733	6380756	(GCG)8	(GCG)8	(GCG)8	(GCG)8	(GCG)8	(GCG)8	(GCG)8	(GCG)8	(GCG)8	(GCG)5	(GCG)8	LOC_Os05g11300	CDS	expressed protein	CODON_CHANGE_PLUS_CODON_DELETION	ACTACGGAGACTGGGTGTGC	CCTCGTCGTCGAGTTGAAGT	137	A	P	A	NA
PMS2792	Os_Chr05	6405871	6405894	(AT)12	(AT)11	(AT)12	(AT)12	(AT)12	(AT)12	(AT)12	(AT)12	(AT)12	(AT)12	(AT)12	LOC_Os05g11330	DRR	RALFL19 - Rapid ALkalinization Factor RALF family protein precursor, expressed	-	GCGAAACTCATCGACTGATCG	GGTGATCACATCATTGTCCATCC	278	P	A	A	RM18080
PMS2793	Os_Chr05	6431034	6431051	(AG)9	(AG)9	(AG)9	(AG)9	(AG)9	(AG)9	(AG)9	(AG)6	(AG)9	(AG)9	(AG)9	-	Introns/Intergenic	-	-	GTCCAAGTGACCAATGGCTT	TGGTTAAGGTCTCTGCCTGC	116	A	A	A	NA
PMS2794	Os_Chr05	6453358	6453391	(AT)17	(AT)17	(AT)10	(AT)9	(AT)17	(AT)17	(AT)6	(AT)17	(AT)17	(AT)17	(AT)17	-	Introns/Intergenic	-	-	TCCCAAATTGCAAAGATCAA	TCGCCAAAAGGATAGCTTACA	278	P	A	A	NA

Polymorphic SSR (PMS) markers	Chromosomes	Start physical positions (bp)	End physical positions (bp)	Nipponbare	Kasalath	Nagina22	IR64	Pokkali	Bala	Tainung67	Azucena	Moroberekan	*Oryza rufipogon*	*Oryza nivara*	MSU gene locus IDs	Structural annotation	Functional annotation	SSR functional effects	Forward primers (5'-3')	Reverse primers (5'-3')	Amplified product size (bp)	Markers exhibiting polymorphism within *indica* rice	Transcrption factor genes-derived markers	Known cloned genes-derived markers	PMS markers corresponding with RM markers
PMS2795	Os_Chr05	6455478	6455517	(TA)20	(TA)20	(TA)20	(TA)9	(TA)20	(TA)20	(TA)20	(TA)20	(TA)8	(TA)8	(TA)20	-	Introns/Intergenic	-	-	TCTATCGTACGTATTGACCAGTCC	TACCCTGCACAACACTATCTAGC	151	P	A	A	RM18084
PMS2796	Os_Chr05	6487770	6487781	(AG)6	(AG)6	(AG)6	(AG)7	(AG)6	(AG)6	(AG)6	(AG)6	(AG)6	(AG)6	(AG)6	-	Introns/Intergenic	-	-	GGAAAATATGCTGACATGGATCT	CCTAACGAATTATATGTTTGGATGC	274	P	A	A	NA
PMS2797	Os_Chr05	6516381	6516398	(CGG)6	(CGG)6	(CGG)6	(CGG)6	(CGG)5	(CGG)6	(CGG)6	(CGG)6	(CGG)6	(CGG)6	(CGG)6	LOC_Os05g11510	CDS	B-box zinc finger family protein, putative, expressed	CODON_CHANGE_PLUS_CODON_DELETION	GGCAGGTACCAGAGATCCAG	GCAGTCACACTCACATGGCT	274	P	P	A	NA
PMS2798	Os_Chr05	6516519	6516539	(GAC)7	(GAC)7	(GAC)7	(GAC)7	(GAC)7	(GAC)7	(GAC)7	(GAC)7	(GAC)7	(GAC)7	(GAC)5	LOC_Os05g11510	DRR	B-box zinc finger family protein, putative, expressed	-	GCCGGAGGCAATAATGCTA	ACGTCAGCGTCTCGAGCTAT	119	A	P	A	NA
PMS2799	Os_Chr05	6574484	6574551	(AT)34	(AT)34	(AT)10	(AT)34	(AT)15	(AT)17	(AT)34	(AT)34	(AT)23	(AT)34	(AT)34	-	Introns/Intergenic	-	-	CGTACACTTGGACGTAGCGA	AAAGCAAGTTTGAACTGAATTTGA	256	P	A	A	NA
PMS2800	Os_Chr05	6576436	6576456	(CGC)7	(CGC)7	(CGC)7	(CGC)7	(CGC)6	(CGC)7	(CGC)7	(CGC)7	(CGC)7	(CGC)7	(CGC)6	LOC_Os05g11610	CDS	expressed protein	CODON_DELETION	CTCATCTTCTTGGCGTCCTC	AGTTCAGTTTCCTATGCCCG	276	P	A	A	NA
PMS2801	Os_Chr05	6671658	6671672	(GAG)5	(GAG)5	(GAG)5	(GAG)5	(GAG)5	(GAG)5	(GAG)5	(GAG)5	(GAG)5	(GAG)5	(GAG)6	LOC_Os05g11750	CDS	protein kinase, putative, expressed	CODON_INSERTION	CGGTAGCTGAACACCTGGAC	CTACTGCTACATCACCCGCA	243	A	A	A	NA
PMS2802	Os_Chr05	6827053	6827118	(GA)33	(GA)33	(GA)33	(GA)9	(GA)33	(GA)33	(GA)33	(GA)33	(GA)33	(GA)33	(GA)33	LOC_Os05g11950	DRR	GDSL-like lipase/acylhydrolase, putative, expressed	-	CGTATGGACTTGCCTTGAGTCG	TCCAATCTGCCAAGCTTTACACC	295	P	A	A	RM18107
PMS2803	Os_Chr05	6831346	6831359	(GC)7	(GC)7	(GC)7	(GC)7	(GC)7	(GC)7	(GC)7	(GC)7	(GC)7	(GC)6	(GC)7	-	Introns/Intergenic	-	-	TTTGTGCTTTGCTTGATTGC	GACCCGCACATTTAACTCAGA	233	A	A	A	NA
PMS2804	Os_Chr05	6883591	6883614	(CCG)8	(CCG)7	(CCG)8	(CCG)8	(CCG)8	(CCG)7	(CCG)8	(CCG)8	(CCG)8	(CCG)8	(CCG)8	LOC_Os05g12040	CDS	cytochrome P450 51, putative, expressed	CODON_DELETION	ATCAGGCACTGCAGCATGTCG	TTGAACAACTCGTCACCCTCATCG	286	P	A	A	RM5994
PMS2805	Os_Chr05	7017233	7017288	(TA)28	(TA)28	(TA)12	(TA)8	(TA)8	(TA)19	(TA)28	(TA)9	(TA)7	(TA)8	(TA)28	-	Introns/Intergenic	-	-	CAAACAACCACCATCATCTTACCC	CTCTCTCGTTAAGCCGACCATCC	437	P	A	A	RM4837
PMS2806	Os_Chr05	7032659	7032670	(AT)6	(AT)7	(AT)6	(AT)6	(AT)6	(AT)6	(AT)6	(AT)6	(AT)6	(AT)6	(AT)6	-	Introns/Intergenic	-	-	ATCATTGGTTTTGCGGCTAC	GTCAGGCCGACAGCTACTTC	237	P	A	A	NA
PMS2807	Os_Chr05	7205926	7205946	(CCA)7	(CCA)7	(CCA)7	(CCA)7	(CCA)7	(CCA)7	(CCA)5	(CCA)7	(CCA)7	(CCA)7	(CCA)6	-	Introns/Intergenic	-	-	TGGGACCCACCTATCAGATT	TGCTGCTCCTGTCACTGCTA	238	A	A	A	NA

Polymorphic SSR (PMS) markers	Chromosomes	Start physical positions (bp)	End physical positions (bp)	Nipponbare	Kasalath	Nagina22	IR64	Pokkali	Bala	Tainung67	Azucena	Moroberekan	*Oryza rufipogon*	*Oryza nivara*	MSU gene locus IDs	Structural annotation	Functional annotation	SSR functional effects	Forward primers (5'-3')	Reverse primers (5'-3')	Amplified product size (bp)	Markers exhibiting polymorphism within *indica* rice	Transcrption factor genes-derived markers	Known cloned genes-derived markers	PMS markers corresponding with RM markers
PMS2808	Os_Chr05	7235240	7235321	(AT)41	(AT)31	(AT)9	(AT)10	(AT)11	(AT)41	(AT)41	(AT)41	(AT)6	(AT)9	(AT)13	-	Introns/Intergenic	-	-	CACCATCGCCACTACACATC	CATTCCTTTAGTGGATATGCGA	204	P	A	A	NA
PMS2809	Os_Chr05	7357502	7357519	(CGT)6	(CGT)6	(CGT)6	(CGT)6	(CGT)6	(CGT)6	(CGT)5	(CGT)6	(CGT)6	(CGT)6	(CGT)6	LOC_Os05g12808	CDS	DUF623 domain containing protein, expressed	CODON_DELETION	ATCCTTCTTCTCCTCCTCCG	AGCTCCTCCTCCTCCACCT	189	A	A	A	NA
PMS2810	Os_Chr05	7373750	7373764	(CCT)5	(CCT)5	(CCT)5	(CCT)5	(CCT)5	(CCT)5	(CCT)5	(CCT)5	(CCT)5	(CCT)7	(CCT)5	LOC_Os05g13300	URR	helicase, putative, expressed	-	CGAATTCCGTCTTCTTGAGC	GGTTGAGGAGTACGAGAGCG	206	A	A	A	NA
PMS2811	Os_Chr05	7406949	7406962	(TA)7	(TA)7	(TA)7	(TA)7	(TA)7	(TA)7	(TA)7	(TA)6	(TA)6	(TA)7	(TA)7	-	Introns/Intergenic	-	-	GCACACTCCATCAGCACTGT	CATGCATGCGCTATGATTTT	231	A	A	A	NA
PMS2812	Os_Chr05	7484122	7484151	(AT)15	(AT)15	(AT)8	(AT)15	(AT)15	(AT)7	(AT)15	(AT)15	(AT)15	(AT)15	(AT)15	-	Introns/Intergenic	-	-	CGGCCTCTTTGAATTAGGAA	GTGTATAGAGGGGATGGGGG	258	P	A	A	NA
PMS2813	Os_Chr05	7498053	7498076	(CT)12	(CT)13	(CT)12	(CT)12	(CT)12	(CT)12	(CT)12	(CT)12	(CT)12	(CT)12	(CT)12	LOC_Os05g13520	URR	white-brown complex homolog protein 12, putative, expressed	-	CACCTCCTCCAAGATCCTTATGC	CTCTCTGTCTCGCTGTCTGTTGC	144	P	A	A	RM169
PMS2814	Os_Chr05	7556345	7556358	(CT)7	(CT)7	(CT)7	(CT)7	(CT)7	(CT)7	(CT)7	(CT)7	(CT)7	(CT)6	(CT)7	-	Introns/Intergenic	-	-	CAGACCCATTCTCACCCAAC	CGGTGTGCTTTCTATGCTGA	181	A	A	A	NA
PMS2815	Os_Chr05	7765811	7765838	(TA)14	(TA)14	(TA)7	(TA)6	(TA)14	(TA)14	(TA)14	(TA)14	(TA)14	(TA)14	(TA)7	-	Introns/Intergenic	-	-	CAAAAGGACTGCCCAGAAAG	TGATTTTGTGCATTTGAAACC	226	P	A	A	NA
PMS2816	Os_Chr05	7806078	7806093	(AG)8	(AG)7	(AG)7	(AG)8	(AG)7	(AG)8	(AG)8	(AG)8	(AG)8	(AG)8	(AG)8	-	Introns/Intergenic	-	-	ACGTGTGTAGGGCATCTTCTCG	GGCTCTGAGCTACCCTTACATGG	199	P	A	A	RM18142
PMS2817	Os_Chr05	7834837	7834880	(ATAG)11	(ATAG)11	(ATAG)11	(ATAG)11	(ATAG)11	(ATAG)11	(ATAG)11	(ATAG)11	(ATAG)11	(ATAG)11	(ATAG)6	-	Introns/Intergenic	-	-	ATGTGGCGGTATAGAAGCGT	ATAGTTCGGACAAGGGGGAG	180	A	A	A	NA
PMS2818	Os_Chr05	7924212	7924245	(TA)17	(TA)17	(TA)17	(TA)11	(TA)17	(TA)17	(TA)17	(TA)17	(TA)8	(TA)17	(TA)9	-	Introns/Intergenic	-	-	AAAATTTGAGTGATTTTGTGCATT	GAATCGGAGGGAGGAGTGAT	213	P	A	A	NA
PMS2819	Os_Chr05	7927228	7927248	(CTC)7	(CTC)7	(CTC)7	(CTC)7	(CTC)7	(CTC)7	(CTC)7	(CTC)7	(CTC)7	(CTC)6	(CTC)7	LOC_Os05g14170	CDS	expressed protein	CODON_DELETION	GACTCCCCAAAGTCCACAAA	GAGAGAGAACGCACCAGTCC	262	A	A	A	NA
PMS2820	Os_Chr05	7944151	7944165	(AGT)5	(AGT)6	(AGT)5	(AGT)5	(AGT)5	(AGT)5	(AGT)5	(AGT)5	(AGT)5	(AGT)5	(AGT)5	-	Introns/Intergenic	-	-	TAATGGATCGCTCCGTTTTC	TTGTACTGCTGGAGTGCTGG	162	P	A	A	NA

Polymorphic SSR (PMS) markers	Chromosomes	Start physical positions (bp)	End physical positions (bp)	Nipponbare	Kasalath	Nagina22	IR64	Pokkali	Bala	Tainung67	Azucena	Moroberekan	*Oryza rufipogon*	*Oryza nivara*	MSU gene locus IDs	Structural annotation	Functional annotation	SSR functional effects	Forward primers (5'-3')	Reverse primers (5'-3')	Amplified product size (bp)	Markers exhibiting polymorphism within *indica* rice	Transcrption factor genes-derived markers	Known cloned genes-derived markers	PMS markers corresponding with RM markers
PMS2821	Os_Chr05	7950910	7950925	(CA)8	(CA)8	(CA)8	(CA)8	(CA)8	(CA)7	(CA)8	(CA)8	(CA)8	(CA)8	(CA)8	LOC_Os05g14180	URR	OsIAA17 - Auxin-responsive Aux/IAA gene family member, expressed	-	CTTCACCACCTCAGCTCCTC	GCGAGAGGCCTATGTAGTCG	173	P	P	A	NA
PMS2822	Os_Chr05	7951243	7951266	(CCG)8	(CCG)8	(CCG)8	(CCG)6	(CCG)8	(CCG)8	(CCG)8	(CCG)8	(CCG)8	(CCG)8	(CCG)8	LOC_Os05g14180	CDS	OsIAA17 - Auxin-responsive Aux/IAA gene family member, expressed	CODON_CHANGE_PLUS_CODON_DELETION	ACAACGACCTGAAGGGCAC	CCTTCTTGTCCTCCTCCTCC	237	P	P	A	NA
PMS2823	Os_Chr05	8009027	8009040	(AT)7	(AT)7	(AT)7	(AT)7	(AT)7	(AT)7	(AT)7	(AT)7	(AT)7	(AT)6	(AT)7	-	Introns/Intergenic	-	-	GCAGGTGTCTACCATGATGC	CGGCTCGGTATTGTGAGGTA	173	A	A	A	NA
PMS2824	Os_Chr05	8225509	8225520	(CT)6	(CT)7	(CT)6	(CT)6	(CT)6	(CT)6	(CT)6	(CT)6	(CT)6	(CT)6	(CT)6	-	Introns/Intergenic	-	-	CAGTCCAGTCAGCACTCTATT	CCGCACGACGCATTTGTG	116	P	A	A	NA
PMS2825	Os_Chr05	8232961	8232976	(TG)8	(TG)7	(TG)7	(TG)7	(TG)7	(TG)8	(TG)8	(TG)8	(TG)8	(TG)7	(TG)7	-	Introns/Intergenic	-	-	CCTTGAGATATGTCCACACCAA	TGATGATGATTCCAGCCAGA	101	P	A	A	NA
PMS2826	Os_Chr05	8305421	8305444	(AT)12	(AT)12	(AT)12	(AT)12	(AT)12	(AT)12	(AT)12	(AT)12	(AT)12	(AT)9	(AT)12	-	Introns/Intergenic	-	-	GCTTAAGCAGCTGTGAGATTTGAGC	ATTCAGGGATGAGCTTGTCTTGG	465	A	A	A	RM18171
PMS2827	Os_Chr05	8433842	8433857	(CT)8	(CT)8	(CT)8	(CT)8	(CT)8	(CT)8	(CT)8	(CT)7	(CT)8	(CT)8	(CT)8	-	Introns/Intergenic	-	-	AAGCGGTTTTGTCCTACGTG	GCTAGTTGCACCGGCTGT	224	A	A	A	NA
PMS2828	Os_Chr05	8613910	8613924	(CGC)5	(CGC)5	(CGC)5	(CGC)5	(CGC)5	(CGC)5	(CGC)5	(CGC)5	(CGC)5	(CGC)6	(CGC)5	LOC_Os05g15170	CDS	zinc finger, C3HC4 type domain containing protein, expressed	CODON_INSERTION	ACCCTCTTGTACTCGAACGC	CGTGCTGTCCTTCCTCTACC	201	A	A	A	NA
PMS2829	Os_Chr05	8902640	8902655	(AC)8	(AC)7	(AC)8	(AC)8	(AC)8	(AC)8	(AC)8	(AC)8	(AC)8	(AC)8	(AC)6	LOC_Os05g15770	DRR	glycosyl hydrolase, putative, expressed	-	CCGTCCTGATCACCTCACTT	CTGGATTCATGCACGAGCTA	174	P	A	A	NA
PMS2830	Os_Chr05	8921583	8921598	(CG)8	(CG)8	(CG)8	(CG)8	(CG)8	(CG)8	(CG)8	(CG)8	(CG)8	(CG)6	(CG)8	-	Introns/Intergenic	-	-	CCACCTCAGGATTTAAGCGA	GGTTAGTGGCTGCAAAAGGA	197	A	A	A	NA
PMS2831	Os_Chr05	9063147	9063166	(AT)10	(AT)10	(AT)10	(AT)9	(AT)10	(AT)10	(AT)10	(AT)10	(AT)10	(AT)10	(AT)10	-	Introns/Intergenic	-	-	AGTAAACGACCACAAGCGCAAGC	TGAACCAGCCAACAAGAGAGTGG	85	P	A	A	RM18185
PMS2832	Os_Chr05	9111302	9111313	(AG)6	(AG)7	(AG)7	(AG)7	(AG)7	(AG)7	(AG)7	(AG)7	(AG)7	(AG)7	(AG)7	-	Introns/Intergenic	-	-	GCTGAGGGGAAAGGGAGTTA	ACACACACTGAGCCCCCTAC	279	A	A	A	NA
PMS2833	Os_Chr05	9123067	9123080	(AG)7	(AG)7	(AG)7	(AG)6	(AG)7	(AG)6	(AG)6	(AG)6	(AG)6	(AG)6	(AG)6	-	Introns/Intergenic	-	-	GCTGAGGGGAAAGGGAGTTA	TATTTGGATGGAGGGAGTGC	273	P	A	A	NA

Polymorphic SSR (PMS) markers	Chromosomes	Start physical positions (bp)	End physical positions (bp)	Nipponbare	Kasalath	Nagina22	IR64	Pokkali	Bala	Tainung67	Azucena	Moroberekan	*Oryza rufipogon*	*Oryza nivara*	MSU gene locus IDs	Structural annotation	Functional annotation	SSR functional effects	Forward primers (5'-3')	Reverse primers (5'-3')	Amplified product size (bp)	Markers exhibiting polymorphism within *indica* rice	Transcrption factor genes-derived markers	Known cloned genes-derived markers	PMS markers corresponding with RM markers
PMS2834	Os_Chr05	9149538	9149603	(ATA)22	(ATA)22	(ATA)22	(ATA)22	(ATA)22	(ATA)22	(ATA)14	(ATA)22	(ATA)22	(ATA)22	(ATA)22	-	Introns/Intergenic	-	-	GGCATTCAGCTATGCATCCTA	AGGGGACGAGAGAGAGAAGG	210	A	A	A	NA
PMS2835	Os_Chr05	9163412	9163435	(GGA)8	(GGA)8	(GGA)8	(GGA)8	(GGA)8	(GGA)8	(GGA)8	(GGA)8	(GGA)8	(GGA)8	(GGA)7	-	Introns/Intergenic	-	-	GATTCCATGTACAGCGGGAG	ATCTGTCGCTCGATGAGGAG	273	A	A	A	NA
PMS2836	Os_Chr05	9241157	9241184	(CGTG)7	(CGTG)5	(CGTG)5	(CGTG)7	(CGTG)5	(CGTG)7	(CGTG)7	(CGTG)7	(CGTG)7	(CGTG)5	(CGTG)5	-	Introns/Intergenic	-	-	CTACGTGTCGATGGCTGCTTGC	CGATCACCGATCGATAACAGATGC	140	P	A	A	RM7409
PMS2837	Os_Chr05	9400824	9400851	(AAAG)7	(AAAG)7	(AAAG)7	(AAAG)7	(AAAG)7	(AAAG)7	(AAAG)7	(AAAG)7	(AAAG)7	(AAAG)7	(AAAG)6	-	Introns/Intergenic	-	-	GCAATTTCCGAAGCCCATGACG	GCCCATGGGTACGTGCTATGATCC	149	A	A	A	RM7588
PMS2838	Os_Chr05	9950313	9950333	(GAC)7	(GAC)7	(GAC)7	(GAC)7	(GAC)7	(GAC)7	(GAC)7	(GAC)7	(GAC)7	(GAC)7	(GAC)5	-	Introns/Intergenic	-	-	GGACGAGACGGACGAGAA	ATCGTCGTCTCCGATCGTT	249	A	A	A	NA
PMS2839	Os_Chr05	10015577	10015597	(GTC)7	(GTC)7	(GTC)7	(GTC)7	(GTC)7	(GTC)7	(GTC)7	(GTC)7	(GTC)7	(GTC)6	(GTC)7	-	Introns/Intergenic	-	-	CTCCGTCATCGTCATCGTAG	ATCCTTGGACAAAGGCGAG	278	A	A	A	NA
PMS2840	Os_Chr05	10022471	10022491	(GTC)7	(GTC)7	(GTC)7	(GTC)7	(GTC)7	(GTC)7	(GTC)7	(GTC)7	(GTC)7	(GTC)6	(GTC)7	-	Introns/Intergenic	-	-	CTCCGTCATCGTCATCGTAG	ATCCTTGGACAAAGGCGAG	278	A	A	A	NA
PMS2841	Os_Chr05	10133375	10133406	(TA)16	(TA)16	(TA)16	(TA)7	(TA)16	(TA)16	(TA)16	(TA)16	(TA)16	(TA)16	(TA)16	-	Introns/Intergenic	-	-	TGATTCCTCTATATGCAGCCTTGG	TATCGTGGTTTCATCGTGTGTGC	279	P	A	A	RM18222
PMS2842	Os_Chr05	10163789	10163824	(TA)18	(TA)18	(TA)9	(TA)6	(TA)18	(TA)18	(TA)18	(TA)18	(TA)18	(TA)18	(TA)18	-	Introns/Intergenic	-	-	GAATGGACGGCTAGGAATCA	TGTCTAAATTACTTTTAGATTTCACGA	224	P	A	A	NA
PMS2843	Os_Chr05	10176461	10176481	(GAC)7	(GAC)7	(GAC)7	(GAC)7	(GAC)7	(GAC)7	(GAC)7	(GAC)7	(GAC)7	(GAC)5	(GAC)7	-	Introns/Intergenic	-	-	CTTGGACGAAGGCGAGAC	CTCCGTCATCATCATCGCT	274	A	A	A	NA
PMS2844	Os_Chr05	10385497	10385517	(GAC)7	(GAC)7	(GAC)7	(GAC)7	(GAC)7	(GAC)7	(GAC)7	(GAC)7	(GAC)7	(GAC)6	(GAC)5	-	Introns/Intergenic	-	-	ATCCTTGGACAAAGGCGAG	CTCCGTCATCGTCATCGTAG	278	A	A	A	NA
PMS2845	Os_Chr05	10392661	10392681	(GAC)7	(GAC)7	(GAC)7	(GAC)7	(GAC)7	(GAC)7	(GAC)7	(GAC)7	(GAC)7	(GAC)6	(GAC)5	-	Introns/Intergenic	-	-	ATCCTTGGACAAAGGCGAG	CTCCGTCATCGTCATCGTAG	278	A	A	A	NA
PMS2846	Os_Chr05	10530017	10530040	(AT)12	(AT)12	(AT)6	(AT)12	(AT)12	(AT)12	(AT)12	(AT)12	(AT)12	(AT)12	(AT)12	-	Introns/Intergenic	-	-	CTGATACCAACGATCCCCAT	TTGAGTGATTTTGTGCATTTGA	251	P	A	A	NA

Polymorphic SSR (PMS) markers	Chromosomes	Start physical positions (bp)	End physical positions (bp)	Nipponbare	Kasalath	Nagina22	IR64	Pokkali	Bala	Tainung67	Azucena	Moroberekan	*Oryza rufipogon*	*Oryza nivara*	MSU gene locus IDs	Structural annotation	Functional annotation	SSR functional effects	Forward primers (5'-3')	Reverse primers (5'-3')	Amplified product size (bp)	Markers exhibiting polymorphism within *indica* rice	Transcrption factor genes-derived markers	Known cloned genes-derived markers	PMS markers corresponding with RM markers
PMS2847	Os_Chr05	10530433	10530490	(TA)29	(TA)29	(TA)29	(TA)18	(TA)29	(TA)29	(TA)6	(TA)29	(TA)6	(TA)29	(TA)29	-	Introns/Intergenic	-	-	TCAATTTCAACAATGGTGCC	TAGTAGGCAGTTTGACCGGG	270	P	A	A	NA
PMS2848	Os_Chr05	10549270	10549283	(TA)7	(TA)7	(TA)7	(TA)7	(TA)7	(TA)7	(TA)7	(TA)7	(TA)7	(TA)6	(TA)7	-	Introns/Intergenic	-	-	CATGAGCACGACCTTCAAAA	CTCTAGCCAAGCACACCACA	249	A	A	A	NA
PMS2849	Os_Chr05	10790507	10790524	(CGC)6	(CGC)5	(CGC)6	(CGC)6	(CGC)5	(CGC)6	(CGC)6	(CGC)6	(CGC)6	(CGC)5	(CGC)6	LOC_Os05g18604	CDS	OsSCP28 - Putative Serine Carboxypeptidase homologue, expressed	CODON_DELETION	GCCGGAGTACATGTCGAAGT	TCAACTCGTTGTCTTGCTCG	139	P	A	A	NA
PMS2850	Os_Chr05	11087855	11087872	(GGC)6	(GGC)6	(GGC)6	(GGC)6	(GGC)6	(GGC)6	(GGC)6	(GGC)5	(GGC)6	(GGC)7	(GGC)6	LOC_Os05g19110	CDS	expressed protein	CODON_INSERTION	AAACTAGGCCACGCATTGAC	GCGAGGAAGATGGTAGTGGA	190	A	A	A	NA
PMS2851	Os_Chr05	11137458	11137475	(TGC)6	(TGC)6	(TGC)6	(TGC)6	(TGC)6	(TGC)5	(TGC)6	(TGC)6	(TGC)6	(TGC)6	(TGC)5	LOC_Os05g19190	CDS	retrotransposon protein, putative, Ty3-gypsy subclass, expressed	CODON_DELETION	GCACGAGTCCCTTGTTGATT	ACTTCCAACGTCTGGTGGAC	250	P	A	A	NA
PMS2852	Os_Chr05	11181720	11181740	(CCT)7	(CCT)7	(CCT)7	(CCT)7	(CCT)7	(CCT)7	(CCT)7	(CCT)7	(CCT)7	(CCT)8	(CCT)7	-	Introns/Intergenic	-	-	ACTCCACAATCAGTGCCTCC	CTAGCCACCAAATCTGGCTC	280	A	A	A	NA
PMS2853	Os_Chr05	11291749	11291769	(GTC)7	(GTC)7	(GTC)7	(GTC)7	(GTC)7	(GTC)7	(GTC)7	(GTC)7	(GTC)7	(GTC)7	(GTC)6	-	Introns/Intergenic	-	-	ATCATCGTTTCCTCGTCACC	ACGACAGACGAGAACGGC	254	A	A	A	NA
PMS2854	Os_Chr05	11328247	11328267	(GCG)7	(GCG)7	(GCG)7	(GCG)7	(GCG)7	(GCG)7	(GCG)5	(GCG)7	(GCG)7	(GCG)7	(GCG)7	-	Introns/Intergenic	-	-	TTCATCCTCTGCTTCCTCGT	GCTGCTTCAACAACGAGTGT	220	A	A	A	NA
PMS2855	Os_Chr05	11671406	11671431	(GA)13	(GA)12	(GA)13	(GA)13	(GA)13	(GA)13	(GA)13	(GA)13	(GA)13	(GA)13	(GA)13	-	Introns/Intergenic	-	-	GAAAGGCGCTATCACAAAGATAGG	GTCACGATTGGGAGGAAACTAGG	181	P	A	A	RM18271
PMS2856	Os_Chr05	12065518	12065553	(TA)18	(TA)18	(TA)8	(TA)18	(TA)7	(TA)18	(TA)6	(TA)8	(TA)6	(TA)18	(TA)18	-	Introns/Intergenic	-	-	ACGGTTGTAGTCGGATCGTC	GGCTCCACTGGTCCTAACAA	276	P	A	A	NA
PMS2857	Os_Chr05	12136480	12136495	(GA)8	(GA)8	(GA)8	(GA)8	(GA)8	(GA)8	(GA)8	(GA)8	(GA)8	(GA)8	(GA)6	-	Introns/Intergenic	-	-	CCCAAGAGAGAGAGGGAAGG	CCTTGGTCTAAAAGGAGCCC	226	A	A	A	NA
PMS2858	Os_Chr05	12877775	12877814	(TA)20	(TA)20	(TA)20	(TA)20	(TA)20	(TA)6	(TA)20	(TA)20	(TA)20	(TA)9	(TA)20	-	Introns/Intergenic	-	-	TGTGGAATGCGTGTGTTCTT	TCCTGCTCTTCATGTTGCAC	239	P	A	A	NA
PMS2859	Os_Chr05	12995594	12995611	(AT)9	(AT)9	(AT)6	(AT)9	(AT)9	(AT)9	(AT)9	(AT)9	(AT)8	(AT)9	(AT)7	-	Introns/Intergenic	-	-	TTGCCCCTTCTCTTACATCG	TGCGCACAATCTAGGAGGTA	249	P	A	A	NA

Polymorphic SSR (PMS) markers	Chromosomes	Start physical positions (bp)	End physical positions (bp)	Nipponbare	Kasalath	Nagina22	IR64	Pokkali	Bala	Tainung67	Azucena	Moroberekan	*Oryza rufipogon*	*Oryza nivara*	MSU gene locus IDs	Structural annotation	Functional annotation	SSR functional effects	Forward primers (5'-3')	Reverse primers (5'-3')	Amplified product size (bp)	Markers exhibiting polymorphism within *indica* rice	Transcrption factor genes-derived markers	Known cloned genes-derived markers	PMS markers corresponding with RM markers
PMS2860	Os_Chr05	13061208	13061222	(GAT)5	(GAT)6	(GAT)6	(GAT)5	(GAT)6	(GAT)5	(GAT)5	(GAT)5	(GAT)5	(GAT)5	(GAT)6	-	Introns/Intergenic	-	-	GCCGGATTTAGGAGTCACAG	GGGCAGTTTCCCCTTAAAAA	279	P	A	A	NA
PMS2861	Os_Chr05	13202046	13202091	(TA)23	(TA)23	(TA)10	(TA)11	(TA)23	(TA)12	(TA)23	(TA)10	(TA)23	(TA)23	(TA)23	-	Introns/Intergenic	-	-	TCAATTTCAACAATGGTGCC	ATTGTGTCTGCCTCAAGCCT	152	P	A	A	NA
PMS2862	Os_Chr05	13277768	13277793	(AT)13	(AT)12	(AT)13	(AT)13	(AT)13	(AT)13	(AT)13	(AT)13	(AT)13	(AT)13	(AT)13	-	Introns/Intergenic	-	-	ACAGGAGCCGTATTGGCTTGC	TCTCATCTCTGATCATCCGTGACC	233	P	A	A	RM18318
PMS2863	Os_Chr05	13297687	13297704	(CT)9	(CT)11	(CT)9	(CT)9	(CT)9	(CT)9	(CT)9	(CT)9	(CT)9	(CT)9	(CT)9	-	Introns/Intergenic	-	-	ATCCCGAATCCAAATCCTTC	GAGCTCGCTAGGGATGGAG	241	P	A	A	NA
PMS2864	Os_Chr05	13405362	13405385	(GCG)8	(GCG)8	(GCG)5	(GCG)8	(GCG)7	(GCG)8	(GCG)5	(GCG)8	(GCG)8	(GCG)8	(GCG)8	LOC_Os05g23440	URR	solute carrier family 35 member F1, putative, expressed	-	AACGGATGGATGGATGCTCACC	CTTGCACCATAGTCGCAATCACC	187	P	A	A	RM18326
PMS2865	Os_Chr05	13476308	13476328	(GGC)7	(GGC)7	(GGC)7	(GGC)7	(GGC)5	(GGC)7	(GGC)7	(GGC)7	(GGC)7	(GGC)7	(GGC)7	LOC_Os05g23530	CDS	expressed protein	CODON_DELETION	CAGCTGGAGTGGAGGTGAAC	GTAGGTGGGAGGAGGAGGAG	215	P	A	A	NA
PMS2866	Os_Chr05	13537325	13537388	(TA)32	(TA)32	(TA)32	(TA)32	(TA)32	(TA)32	(TA)32	(TA)32	(TA)6	(TA)32	(TA)14	-	Introns/Intergenic	-	-	ATGCAGGCCAAATTGTTTGTGG	CCTACCAGCTCAGAGTGATGGTTCC	386	A	A	A	RM8014,RM2676
PMS2867	Os_Chr05	13606445	13606480	(CCG)12	(CCG)12	(CCG)12	(CCG)12	(CCG)9	(CCG)12	(CCG)12	(CCG)12	(CCG)12	(CCG)12	(CCG)12	LOC_Os05g23700	URR	DNA-binding storekeeper protein-related, putative, expressed	-	ATCCCTTCACACTGCTCCAC	AAAGAATCCAGCACCCCC	232	P	A	A	NA
PMS2868	Os_Chr05	13632051	13632064	(TC)7	(TC)7	(TC)7	(TC)7	(TC)7	(TC)7	(TC)6	(TC)7	(TC)7	(TC)7	(TC)7	-	Introns/Intergenic	-	-	TAGAAAACCGACAACGAGCC	CAGATCTGGCGGAGGAGG	206	A	A	A	NA
PMS2869	Os_Chr05	13702770	13702803	(CT)17	(CT)17	(CT)17	(CT)8	(CT)17	(CT)17	(CT)17	(CT)10	(CT)17	(CT)17	(CT)17	LOC_Os05g23840	URR	expressed protein	-	CGTTGCATAAACGGCTGATA	AAGGGATCGATGGGGTTC	266	P	P	A	NA
PMS2870	Os_Chr05	13702920	13702940	(GCG)7	(GCG)5	(GCG)7	(GCG)7	(GCG)7	(GCG)7	(GCG)7	(GCG)7	(GCG)7	(GCG)7	(GCG)7	LOC_Os05g23840	CDS	expressed protein	CODON_DELETION	CTCTCTCTTCCCCCAAATCC	CGGGGGATGATAGGGTTTC	195	P	P	A	NA
PMS2871	Os_Chr05	13703186	13703203	(GCC)6	(GCC)6	(GCC)6	(GCC)6	(GCC)6	(GCC)6	(GCC)6	(GCC)6	(GCC)6	(GCC)5	(GCC)6	LOC_Os05g23840	CDS	expressed protein	CODON_DELETION	AGTACGTCTGGGAGCTCGG	GCCTCTTGCTGATATAGGCG	258	A	P	A	NA
PMS2872	Os_Chr05	13789462	13789475	(TA)7	(TA)7	(TA)7	(TA)7	(TA)6	(TA)7	(TA)7	(TA)7	(TA)7	(TA)7	(TA)7	-	Introns/Intergenic	-	-	TCGTGACAAAATTACTGCCG	TCTTCACAACAAGATCTTACATGATT	235	P	A	A	NA

Polymorphic SSR (PMS) markers	Chromosomes	Start physical positions (bp)	End physical positions (bp)	Nipponbare	Kasalath	Nagina22	IR64	Pokkali	Bala	Tainung67	Azucena	Moroberekan	*Oryza rufipogon*	*Oryza nivara*	MSU gene locus IDs	Structural annotation	Functional annotation	SSR functional effects	Forward primers (5'-3')	Reverse primers (5'-3')	Amplified product size (bp)	Markers exhibiting polymorphism within *indica* rice	Transcrption factor genes-derived markers	Known cloned genes-derived markers	PMS markers corresponding with RM markers
PMS2873	Os_Chr05	13907746	13907757	(CT)6	(CT)7	(CT)6	(CT)7	(CT)7	(CT)6	(CT)6	(CT)6	(CT)7	(CT)6	(CT)6	-	Introns/Intergenic	-	-	TGAATTTTCAGAGCATGGGA	AGAGGAGAGGGAGGGGGAG	254	P	A	A	NA
PMS2874	Os_Chr05	14007128	14007171	(TA)22	(TA)21	(TA)22	(TA)22	(TA)11	(TA)22	(TA)22	(TA)15	(TA)22	(TA)22	(TA)10	-	Introns/Intergenic	-	-	AGATCTCACTATTGAGTAGCCCATGC	CACCTTGCCCTTAAATACCAACC	287	P	A	A	RM18353
PMS2875	Os_Chr05	14047563	14047610	(TA)24	(TA)24	(TA)17	(TA)24	(TA)7	(TA)12	(TA)24	(TA)24	(TA)24	(TA)24	(TA)24	-	Introns/Intergenic	-	-	TTCGAATATCATCGGGTGGT	CACGATTAACCATCGTGAGC	259	P	A	A	NA
PMS2876	Os_Chr05	14057013	14057028	(TC)8	(TC)8	(TC)8	(TC)8	(TC)8	(TC)6	(TC)8	(TC)8	(TC)8	(TC)8	(TC)8	-	Introns/Intergenic	-	-	TGGGGGTCTTAGTTTCAGTCA	TTCTTTTATGAAAACCGAAGGG	268	P	A	A	NA
PMS2877	Os_Chr05	14153353	14153373	(AGG)7	(AGG)7	(AGG)7	(AGG)7	(AGG)7	(AGG)7	(AGG)7	(AGG)7	(AGG)7	(AGG)6	(AGG)7	-	Introns/Intergenic	-	-	ATGGCTCCCTGTAGCCGATGG	ATCGCCAACAACAACTCTCAAACTGG	179	A	A	A	RM18359
PMS2878	Os_Chr05	14167821	14167841	(GAA)7	(GAA)7	(GAA)7	(GAA)8	(GAA)7	(GAA)7	(GAA)7	(GAA)7	(GAA)7	(GAA)7	(GAA)7	-	Introns/Intergenic	-	-	GATGAAGAAGCATAGCAAGCATCG	CATCTACTCTTGGAGAACTGCCTACC	99	P	A	A	RM18361
PMS2879	Os_Chr05	14181639	14181653	(CTT)5	(CTT)6	(CTT)6	(CTT)6	(CTT)6	(CTT)6	(CTT)5	(CTT)6	(CTT)6	(CTT)6	(CTT)6	-	Introns/Intergenic	-	-	CCATCCGTTTCCCATCTATC	AAGCAGCTTCCAGATCCAAA	101	A	A	A	NA
PMS2880	Os_Chr05	14325196	14325255	(TA)30	(TA)30	(TA)30	(TA)30	(TA)8	(TA)20	(TA)30	(TA)30	(TA)30	(TA)18	(TA)13	-	Introns/Intergenic	-	-	ATGGTGCCCCATATGAGTGT	CGGGACGACGATGCTACTAT	232	P	A	A	NA
PMS2881	Os_Chr05	14327142	14327159	(AT)9	(AT)9	(AT)7	(AT)9	(AT)9	(AT)9	(AT)9	(AT)8	(AT)9	(AT)9	(AT)9	-	Introns/Intergenic	-	-	CTAAGTGACACCCGCACTCA	TTTGCCATCAAGATCAACGA	200	P	A	A	NA
PMS2882	Os_Chr05	14364399	14364424	(TA)13	(TA)13	(TA)13	(TA)13	(TA)13	(TA)13	(TA)13	(TA)10	(TA)13	(TA)13	(TA)13	-	Introns/Intergenic	-	-	TGGTGTGGTGTGCATGTGTA	GAAGCATTGCATCAGTTCCA	141	A	A	A	NA
PMS2883	Os_Chr05	14510002	14510019	(AT)9	(AT)9	(AT)9	(AT)7	(AT)9	(AT)9	(AT)9	(AT)9	(AT)9	(AT)9	(AT)9	-	Introns/Intergenic	-	-	GGCTATCTTCAAGGCAACCA	CAAGTTCCAAGGATCCCAAA	212	P	A	A	NA
PMS2884	Os_Chr05	14525370	14525383	(GA)7	(GA)13	(GA)7	(GA)7	(GA)7	(GA)13	(GA)7	(GA)7	(GA)7	(GA)7	(GA)7	-	Introns/Intergenic	-	-	GGGAGAAAGGGGAAATTTTG	CCTAAGGCAGAATTAGGGGC	153	P	A	A	NA
PMS2885	Os_Chr05	14552992	14553013	(CT)11	(CT)9	(CT)11	(CT)11	(CT)11	(CT)11	(CT)11	(CT)11	(CT)11	(CT)11	(CT)11	-	Introns/Intergenic	-	-	GTGGGTGGCACTTAACCCATACC	GAGAATTCCTTGCTCTTCGATTCC	214	P	A	A	RM18374

Polymorphic SSR (PMS) markers	Chromosomes	Start physical positions (bp)	End physical positions (bp)	Nipponbare	Kasalath	Nagina22	IR64	Pokkali	Bala	Tainung67	Azucena	Moroberekan	*Oryza rufipogon*	*Oryza nivara*	MSU gene locus IDs	Structural annotation	Functional annotation	SSR functional effects	Forward primers (5'-3')	Reverse primers (5'-3')	Amplified product size (bp)	Markers exhibiting polymorphism within *indica* rice	Transcrption factor genes-derived markers	Known cloned genes-derived markers	PMS markers corresponding with RM markers
PMS2886	Os_Chr05	14574422	14574484	(AAT)21	(AAT)20	(AAT)21	(AAT)21	(AAT)21	(AAT)21	(AAT)8	(AAT)21	(AAT)21	(AAT)21	(AAT)21	-	Introns/Intergenic	-	-	GTGGTGCCTGATGAAGGCTACG	CAACTGACAAGTTCAGTGGCATGG	354	P	A	A	RM5705
PMS2887	Os_Chr05	14839503	14839516	(CT)7	(CT)7	(CT)7	(CT)7	(CT)7	(CT)7	(CT)7	(CT)6	(CT)6	(CT)7	(CT)7	-	Introns/Intergenic	-	-	GGTTCCTTTTGAACGCAAGA	AAGAACAGAAAGTCTCTCACCGA	213	A	A	A	NA
PMS2888	Os_Chr05	14858128	14858148	(CTC)7	(CTC)7	(CTC)7	(CTC)7	(CTC)7	(CTC)7	(CTC)7	(CTC)5	(CTC)7	(CTC)7	(CTC)7	LOC_Os05g25570	CDS	retrotransposon protein, putative, unclassified, expressed	CODON_DELETION	ACAAGTTACTCAACGGCCCAAGC	CCTCCTTCGTCAGGGACTCTAGC	389	A	A	A	RM18389
PMS2889	Os_Chr05	14922929	14922944	(TC)8	(TC)6	(TC)8	(TC)8	(TC)8	(TC)8	(TC)8	(TC)8	(TC)8	(TC)8	(TC)8	-	Introns/Intergenic	-	-	GGCGTCCCTCAACTAACAAA	CCTAACACCACACGCTGAGA	194	P	A	A	NA
PMS2890	Os_Chr05	14946683	14946696	(AT)7	(AT)7	(AT)7	(AT)7	(AT)7	(AT)7	(AT)7	(AT)7	(AT)7	(AT)6	(AT)7	-	Introns/Intergenic	-	-	CAACGGGTGAAGGTTGTTTT	CGTCATTTGTGTGTGATTCG	241	A	A	A	NA
PMS2891	Os_Chr05	14948182	14948199	(CGG)6	(CGG)6	(CGG)6	(CGG)5	(CGG)6	(CGG)5	(CGG)6	(CGG)6	(CGG)6	(CGG)6	(CGG)6	-	Introns/Intergenic	-	-	CTTGCAACCATTGTTCATGC	ACCTCGTCCATCTCCCAAC	159	P	A	A	NA
PMS2892	Os_Chr05	14987610	14987633	(CCT)8	(CCT)8	(CCT)8	(CCT)8	(CCT)8	(CCT)8	(CCT)5	(CCT)8	(CCT)8	(CCT)8	(CCT)8	-	Introns/Intergenic	-	-	CACCGCCACTTTCCTCTTAG	CTTGGAGAGGGAGAAGGGAG	167	A	A	A	NA
PMS2893	Os_Chr05	14997921	14997941	(GCC)7	(GCC)7	(GCC)7	(GCC)7	(GCC)6	(GCC)7	(GCC)7	(GCC)5	(GCC)7	(GCC)7	(GCC)7	LOC_Os05g25780	CDS	rhodanese-like domain containing protein, putative, expressed	CODON_DELETION	TGCGACTCCAAACCTTACTAGGC	AATCACCAGCATCCAGTGTAGCC	389	P	A	A	RM18396
PMS2894	Os_Chr05	15090213	15090230	(GCG)6	(GCG)6	(GCG)6	(GCG)6	(GCG)6	(GCG)6	(GCG)6	(GCG)6	(GCG)6	(GCG)5	(GCG)6	LOC_Os05g25960	CDS	no apical meristem protein, expressed	CODON_DELETION	CGACGAGGCCTTCTTCTTC	GTACTCGTGCATCACCCATC	253	A	P	A	NA
PMS2895	Os_Chr05	15107006	15107032	(GAT)9	(GAT)9	(GAT)9	(GAT)9	(GAT)9	(GAT)9	(GAT)9	(GAT)9	(GAT)6	(GAT)9	(GAT)9	LOC_Os05g26000	CDS	retrotransposon protein, putative, unclassified, expressed	CODON_DELETION	GTAATGTTGCCGGTGAAGGT	AGGCACCAAAAAGCAAGAAA	261	A	A	A	NA
PMS2896	Os_Chr05	15148339	15148358	(AT)10	(AT)10	(AT)10	(AT)10	(AT)8	(AT)10	(AT)10	(AT)10	(AT)10	(AT)10	(AT)6	-	Introns/Intergenic	-	-	ACTCAAAACAGTCATGACATACA	ACTGTTCGTATGGTCTACGTG	291	P	A	A	NA
PMS2897	Os_Chr05	15249834	15249855	(AG)11	(AG)11	(AG)7	(AG)11	(AG)11	(AG)11	(AG)11	(AG)11	(AG)11	(AG)11	(AG)11	-	Introns/Intergenic	-	-	GGCACTCTGGTTCCTCAATGG	TCTGTATCATCAACGGCAACTCC	179	P	A	A	RM18408
PMS2898	Os_Chr05	15319816	15319829	(GA)7	(GA)8	(GA)8	(GA)7	(GA)7	(GA)7	(GA)7	(GA)7	(GA)7	(GA)7	(GA)7	-	Introns/Intergenic	-	-	TTCTTTTATGAAAACCGAAGGG	TTGCTAAAATTACGGGGGTC	280	P	A	A	NA

**Polymorphic SSR (PMS) markers**	**Chromosomes**	**Start physical positions (bp)**	**End physical positions (bp)**	**Nipponbare**	**Kasalath**	**Nagina22**	**IR64**	**Pokkali**	**Bala**	**Tainung67**	**Azucena**	**Moroberekan**	***Oryza rufipogon***	***Oryza nivara***	**MSU gene locus IDs**	**Structural annotation**	**Functional annotation**	**SSR functional effects**	**Forward primers (5'-3')**	**Reverse primers (5'-3')**	**Amplified product size (bp)**	**Markers exhibiting polymorphism within *indica* rice**	**Transcrption factor genes-derived markers**	**Known cloned genes-derived markers**	**PMS markers corresponding with RM markers**
PMS2899	Os_Chr05	15461725	15461742	(GAC)6	(GAC)6	(GAC)6	(GAC)6	(GAC)6	(GAC)6	(GAC)6	(GAC)6	(GAC)6	(GAC)5	(GAC)6	-	Introns/Intergenic	-	-	AGAGATCCTTACCACCACGC	ACCGTCATCGTCATTGCAG	228	A	A	A	NA
PMS2900	Os_Chr05	15755953	15755968	(AC)8	(AC)8	(AC)6	(AC)6	(AC)6	(AC)6	(AC)8	(AC)8	(AC)8	(AC)8	(AC)7	-	Introns/Intergenic	-	-	TTGGAGGTGAGCTGAAGACA	TCAATTGTACGCTTGCTTGC	200	P	A	A	NA
PMS2901	Os_Chr05	15839709	15839764	(AT)28	(AT)28	(AT)28	(AT)6	(AT)28	(AT)28	(AT)28	(AT)28	(AT)28	(AT)28	(AT)28	-	Introns/Intergenic	-	-	GGGACTAAAGATCGAGCTTACC	TTCTGAGCTAGCGACTACTACTACG	421	P	A	A	RM18415
PMS2902	Os_Chr05	15896285	15896296	(CT)6	(CT)6	(CT)6	(CT)6	(CT)6	(CT)7	(CT)6	(CT)6	(CT)6	(CT)6	(CT)6	LOC_Os05g27350	URR	expressed protein	-	AAGACGGGCACATGAAGAAC	ATTCACTTTCTTGGCTCCCC	157	P	A	A	NA
PMS2903	Os_Chr05	15935522	15935541	(AAAT)5	(AAAT)6	(AAAT)6	(AAAT)5	(AAAT)5	(AAAT)6	(AAAT)5	(AAAT)5	(AAAT)5	(AAAT)5	(AAAT)5	-	Introns/Intergenic	-	-	ACAATGTGACACGCCAAAAA	ATAAGGTCGAGGCCCTGAAT	230	P	A	A	NA
PMS2904	Os_Chr05	15990361	15990386	(AT)13	(AT)13	(AT)11	(AT)13	(AT)13	(AT)13	(AT)13	(AT)13	(AT)10	(AT)13	(AT)13	-	Introns/Intergenic	-	-	AACAGAGCACGCTCTACCTTCTTCC	GAGATTTAGGCCCTGTCTGTTTCAGC	377	P	A	A	RM18431
PMS2905	Os_Chr05	16099095	16099116	(AT)11	(AT)11	(AT)11	(AT)11	(AT)6	(AT)7	(AT)11	(AT)7	(AT)11	(AT)11	(AT)11	-	Introns/Intergenic	-	-	GAAGGAATAGAACCCTCGGC	GGTTTCATTTGTTGGGATGG	172	P	A	A	NA
PMS2906	Os_Chr05	16128617	16128644	(TA)14	(TA)14	(TA)9	(TA)14	(TA)14	(TA)14	(TA)14	(TA)14	(TA)14	(TA)14	(TA)8	-	Introns/Intergenic	-	-	CTTCGTTTAATACGTAGCACCTTA	CGTAGCTAGCAAGGACATAATGAA	279	P	A	A	NA
PMS2907	Os_Chr05	16177244	16177257	(CG)7	(CG)7	(CG)7	(CG)6	(CG)7	(CG)7	(CG)7	(CG)7	(CG)7	(CG)7	(CG)7	-	Introns/Intergenic	-	-	CCCTGTTGACCAGTTGAGGT	GGTTAGTGGCTGCAAAAGGA	144	P	A	A	NA
PMS2908	Os_Chr05	16194183	16194250	(TA)34	(TA)34	(TA)34	(TA)34	(TA)34	(TA)34	(TA)34	(TA)34	(TA)11	(TA)27	(TA)34	-	Introns/Intergenic	-	-	GGGAGATGAGAGCGCTTGTA	CCATGGAGCACCAAACAAAT	227	A	A	A	NA
PMS2909	Os_Chr05	16382163	16382184	(TC)11	(TC)9	(TC)11	(TC)9	(TC)11	(TC)11	(TC)11	(TC)11	(TC)11	(TC)11	(TC)9	-	Introns/Intergenic	-	-	CCGGTGTCTTCTTCTTCTCTCACC	ACGTAATCTCGATCTCCCTGTGC	172	P	A	A	RM509
PMS2910	Os_Chr05	16413410	16413427	(AG)9	(AG)9	(AG)7	(AG)9	(AG)9	(AG)9	(AG)9	(AG)9	(AG)9	(AG)9	(AG)9	-	Introns/Intergenic	-	-	CTCCCTGTCCATCTCTTCCA	CCCATCTATTGACAGACCCG	221	P	A	A	NA
PMS2911	Os_Chr05	16473887	16473904	(CCG)6	(CCG)6	(CCG)6	(CCG)6	(CCG)6	(CCG)5	(CCG)6	(CCG)6	(CCG)5	(CCG)6	(CCG)6	-	Introns/Intergenic	-	-	AGTAAGGCGAAAGTGGCGT	AAGCGCCAGAATCACACC	256	P	A	A	NA

Polymorphic SSR (PMS) markers	Chromosomes	Start physical positions (bp)	End physical positions (bp)	Nipponbare	Kasalath	Nagina22	IR64	Pokkali	Bala	Tainung67	Azucena	Moroberekan	*Oryza rufipogon*	*Oryza nivara*	MSU gene locus IDs	Structural annotation	Functional annotation	SSR functional effects	Forward primers (5'-3')	Reverse primers (5'-3')	Amplified product size (bp)	Markers exhibiting polymorphism within *indica* rice	Transcrption factor genes-derived markers	Known cloned genes-derived markers	PMS markers corresponding with RM markers
PMS2912	Os_Chr05	16500208	16500225	(ATT)6	(ATT)6	(ATT)6	(ATT)5	(ATT)6	(ATT)5	(ATT)6	(ATT)5	(ATT)5	(ATT)6	(ATT)6	-	Introns/Intergenic	-	-	GCGTGGATAGTGAGGTGGAT	TCGTGTGATTTGAAAAGGGA	245	P	A	A	NA
PMS2913	Os_Chr05	16506548	16506568	(CGG)7	(CGG)7	(CGG)7	(CGG)7	(CGG)5	(CGG)7	(CGG)7	(CGG)7	(CGG)7	(CGG)7	(CGG)7	LOC_Os05g28190	URR	ranBP1 domain containing protein, expressed	-	AACAAGCCCGTACTCACCAG	AATCCCCCAACCCTAAATTG	261	P	A	A	NA
PMS2914	Os_Chr05	16559938	16559955	(CCG)6	(CCG)6	(CCG)6	(CCG)6	(CCG)6	(CCG)6	(CCG)6	(CCG)6	(CCG)6	(CCG)5	(CCG)5	LOC_Os05g28290	CDS	ranBP1 domain containing protein, expressed	CODON_CHANGE_PLUS_CODON_DELETION	CGTTAGCTCTTCTCCTCCGA	CACGAGCTACAAGAGAGGGG	258	A	A	A	NA
PMS2915	Os_Chr05	16566623	16566638	(TC)8	(TC)8	(TC)8	(TC)8	(TC)8	(TC)8	(TC)8	(TC)8	(TC)8	(TC)11	(TC)8	LOC_Os05g28300	URR	expressed protein	-	GGTTCAGAGCTTTCAGACCG	AGCTCTAGGGCTTACCGAGC	236	A	A	A	NA
PMS2916	Os_Chr05	16578500	16578529	(TA)15	(TA)15	(TA)15	(TA)15	(TA)7	(TA)15	(TA)15	(TA)15	(TA)15	(TA)15	(TA)15	-	Introns/Intergenic	-	-	CGGAAGAAGAATATACACGACCATGC	CCCGCTGTACGGATCGTAATACC	301	P	A	A	RM18461
PMS2917	Os_Chr05	16601108	16601128	(CCG)7	(CCG)8	(CCG)7	(CCG)7	(CCG)7	(CCG)8	(CCG)7	(CCG)7	(CCG)7	(CCG)7	(CCG)7	LOC_Os05g28350	CDS	OsABI4/Osh69	CODON_INSERTION	GGAATCGGTCACTTGAGGAA	GTTGCAGCTGCTGTGTCTTC	206	P	A	P	NA
PMS2918	Os_Chr05	16603497	16603546	(TA)25	(TA)25	(TA)20	(TA)25	(TA)25	(TA)25	(TA)25	(TA)13	(TA)7	(TA)25	(TA)25	-	Introns/Intergenic	-	-	CCTCAATCTGATGGCCAGTT	TTCCTACAAGGGATGGATGC	215	P	A	A	NA
PMS2919	Os_Chr05	16639546	16639571	(TA)13	(TA)13	(TA)6	(TA)13	(TA)13	(TA)13	(TA)13	(TA)13	(TA)13	(TA)13	(TA)13	-	Introns/Intergenic	-	-	AATGTGATCCAACGGCAGAT	AGTCTTGTTATGGGCCGTGA	279	P	A	A	NA
PMS2920	Os_Chr05	16662749	16662766	(CGC)6	(CGC)6	(CGC)5	(CGC)6	(CGC)6	(CGC)6	(CGC)6	(CGC)6	(CGC)6	(CGC)6	(CGC)6	LOC_Os05g28460	URR	MEE12, putative, expressed	-	AAATACACGCTTTTTCCCCA	TGGGAGGGATTTTTGTTTGA	159	P	A	A	NA
PMS2921	Os_Chr05	16702652	16702669	(CGC)6	(CGC)6	(CGC)6	(CGC)6	(CGC)6	(CGC)6	(CGC)6	(CGC)6	(CGC)6	(CGC)5	(CGC)6	LOC_Os05g28520	CDS	MRH1, putative, expressed	CODON_DELETION	CATCTCCAGCAGCAGCATC	CGTCTACCTCACCGACGACT	142	A	A	A	NA
PMS2922	Os_Chr05	16718386	16718412	(CCG)9	(CCG)7	(CCG)9	(CCG)9	(CCG)9	(CCG)9	(CCG)9	(CCG)9	(CCG)9	(CCG)9	(CCG)9	LOC_Os05g28530	CDS	glutaredoxin, putative, expressed	CODON_DELETION	GGTGAAGTAGAGCACCACGC	CAGTCCCCGTCCTTCTCC	273	P	A	A	NA
PMS2923	Os_Chr05	16778563	16778586	(CT)12	(CT)9	(CT)12	(CT)12	(CT)12	(CT)9	(CT)12	(CT)12	(CT)12	(CT)12	(CT)12	-	Introns/Intergenic	-	-	AACGCCCTCCCTTTCAGTAGG	CGACAACACAAGAACATCACAAGC	386	P	A	A	RM18473
PMS2924	Os_Chr05	16786252	16786269	(CTC)6	(CTC)6	(CTC)6	(CTC)6	(CTC)6	(CTC)6	(CTC)6	(CTC)6	(CTC)6	(CTC)5	(CTC)6	LOC_Os05g28630	CDS	leaf senescence related protein, putative, expressed	CODON_DELETION	AGAGAACAGCGAGGAACAGC	GTCTCCGTGCTCTACGACAG	197	A	A	A	NA

Polymorphic SSR (PMS) markers	Chromosomes	Start physical positions (bp)	End physical positions (bp)	Nipponbare	Kasalath	Nagina22	IR64	Pokkali	Bala	Tainung67	Azucena	Moroberekan	*Oryza rufipogon*	*Oryza nivara*	MSU gene locus IDs	Structural annotation	Functional annotation	SSR functional effects	Forward primers (5'-3')	Reverse primers (5'-3')	Amplified product size (bp)	Markers exhibiting polymorphism within *indica* rice	Transcrption factor genes-derived markers	Known cloned genes-derived markers	PMS markers corresponding with RM markers
PMS2925	Os_Chr05	16809101	16809122	(TA)11	(TA)11	(TA)10	(TA)11	(TA)11	(TA)11	(TA)11	(TA)11	(TA)7	(TA)11	(TA)11	-	Introns/Intergenic	-	-	CCATTTGTTGGAGCCTGTCT	CACTGGGTTGTAGCAGGTGA	212	P	A	A	NA
PMS2926	Os_Chr05	16846523	16846543	(GCC)7	(GCC)7	(GCC)7	(GCC)7	(GCC)7	(GCC)7	(GCC)7	(GCC)5	(GCC)7	(GCC)7	(GCC)7	LOC_Os05g28720	CDS	PPR repeat containing protein, expressed	CODON_DELETION	GTTTCCACCTGCTCGGCATCC	TGCAGTACGTGATGGTGGTGTGC	331	A	A	A	RM18479
PMS2927	Os_Chr05	16883812	16883827	(AG)8	(AG)8	(AG)8	(AG)8	(AG)8	(AG)10	(AG)8	(AG)8	(AG)8	(AG)8	(AG)8	-	Introns/Intergenic	-	-	ATGTCACCCCAACAGGACAT	CGCATGTAAAAATGGTCCCT	240	P	A	A	NA
PMS2928	Os_Chr05	16993984	16994001	(TC)9	(TC)8	(TC)8	(TC)8	(TC)8	(TC)8	(TC)9	(TC)9	(TC)9	(TC)9	(TC)9	LOC_Os05g28980	URR	drought induced 19 protein, putative, expressed	-	AGGAGGATGAGGAGGAGGAG	GAAGCGGTAACAAAGCCAAC	231	A	A	A	NA
PMS2929	Os_Chr05	17038886	17038900	(TGT)5	(TGT)5	(TGT)5	(TGT)5	(TGT)5	(TGT)5	(TGT)5	(TGT)5	(TGT)5	(TGT)6	(TGT)5	-	Introns/Intergenic	-	-	TCGGACGATGATGATGAAAA	AGTTGCTCTGGGAATGGATG	275	A	A	A	NA
PMS2930	Os_Chr05	17041733	17041768	(ATCT)9	(ATCT)11	(ATCT)11	(ATCT)11	(ATCT)11	(ATCT)11	(ATCT)9	(ATCT)11	(ATCT)9	(ATCT)11	(ATCT)11	-	Introns/Intergenic	-	-	GGAAATTTTCGTTGGGTGTG	ATGCATCCATGCACACAAGT	273	A	A	A	NA
PMS2931	Os_Chr05	17045350	17045364	(TAA)5	(TAA)7	(TAA)5	(TAA)5	(TAA)5	(TAA)5	(TAA)5	(TAA)5	(TAA)5	(TAA)5	(TAA)5	-	Introns/Intergenic	-	-	TTGGGTTTTGATGTTCAACG	CCGAGATCAATGAATACGCC	248	P	A	A	NA
PMS2932	Os_Chr05	17086553	17086570	(TA)9	(TA)9	(TA)9	(TA)9	(TA)9	(TA)9	(TA)6	(TA)9	(TA)9	(TA)9	(TA)9	-	Introns/Intergenic	-	-	CTACAACAAGCCCCTAACGG	ACGTGGAACACATTCAAGCA	271	A	A	A	NA
PMS2933	Os_Chr05	17275212	17275247	(AT)18	(AT)18	(AT)9	(AT)18	(AT)7	(AT)10	(AT)18	(AT)18	(AT)12	(AT)18	(AT)7	-	Introns/Intergenic	-	-	GGCATGACCTTCCCTCTTTT	GGTAATACCAACCGGGGACT	227	P	A	A	NA
PMS2934	Os_Chr05	17289596	17289619	(TG)12	(TG)9	(TG)12	(TG)12	(TG)12	(TG)12	(TG)12	(TG)12	(TG)12	(TG)12	(TG)12	-	Introns/Intergenic	-	-	ATCCGTACAACCCCTCCTCT	CATGGAACATGGAGACATGG	274	P	A	A	NA
PMS2935	Os_Chr05	17359055	17359075	(CAA)7	(CAA)6	(CAA)6	(CAA)7	(CAA)7	(CAA)5	(CAA)7	(CAA)7	(CAA)5	(CAA)7	(CAA)5	-	Introns/Intergenic	-	-	CCATGAGGAACACACACGTC	AATCATCATGGAGCACACGA	245	P	A	A	NA
PMS2936	Os_Chr05	17379788	17379827	(GA)20	(GA)20	(GA)20	(GA)20	(GA)20	(GA)20	(GA)20	(GA)15	(GA)20	(GA)20	(GA)20	-	Introns/Intergenic	-	-	GGGATGAAGGTGAGTAGGCA	CAGATGTGTGCGATCAGGAG	256	A	A	A	NA
PMS2937	Os_Chr05	17446160	17446177	(CCG)6	(CCG)6	(CCG)6	(CCG)6	(CCG)6	(CCG)6	(CCG)5	(CCG)6	(CCG)6	(CCG)6	(CCG)6	LOC_Os05g30140	URR	RNA recognition motif containing protein, expressed	-	GACCCCGTCTCCTTTTTCAT	TCGCGCAGGAGTACTAGGAT	201	A	A	A	NA

Polymorphic SSR (PMS) markers	Chromosomes	Start physical positions (bp)	End physical positions (bp)	Nipponbare	Kasalath	Nagina22	IR64	Pokkali	Bala	Tainung67	Azucena	Moroberekan	*Oryza rufipogon*	*Oryza nivara*	MSU gene locus IDs	Structural annotation	Functional annotation	SSR functional effects	Forward primers (5'-3')	Reverse primers (5'-3')	Amplified product size (bp)	Markers exhibiting polymorphism within *indica* rice	Transcrption factor genes-derived markers	Known cloned genes-derived markers	PMS markers corresponding with RM markers
PMS2938	Os_Chr05	17649535	17649552	(GGC)6	(GGC)6	(GGC)6	(GGC)6	(GGC)6	(GGC)6	(GGC)6	(GGC)6	(GGC)6	(GGC)5	(GGC)6	LOC_Os05g30454	URR	thiamin pyrophosphokinase 1, putative, expressed	-	GCTGGTTGAGGACGAGGAG	ACCCCAAATTTTCAAACACG	274	A	A	A	NA
PMS2939	Os_Chr05	17661490	17661504	(GCC)5	(GCC)5	(GCC)5	(GCC)5	(GCC)5	(GCC)5	(GCC)5	(GCC)6	(GCC)5	(GCC)5	(GCC)5	LOC_Os05g30480	CDS	OsBiP3|BiP3	CODON_INSERTION	CCATTCCGGTAGACACCAAC	CGTCTCTCACACACAGCCAT	194	A	A	P	NA
PMS2940	Os_Chr05	17758872	17758889	(GAC)6	(GAC)8	(GAC)6	(GAC)6	(GAC)6	(GAC)6	(GAC)6	(GAC)6	(GAC)6	(GAC)6	(GAC)6	LOC_Os05g30660	CDS	leucine zipper protein-like, putative, expressed	CODON_INSERTION	GTTACTGGATCTTGCCGAGC	GAAGTCGACCTCTCAATCGC	233	P	A	A	NA
PMS2941	Os_Chr05	17768438	17768469	(CTAT)8	(CTAT)8	(CTAT)8	(CTAT)8	(CTAT)8	(CTAT)8	(CTAT)8	(CTAT)8	(CTAT)8	(CTAT)8	(CTAT)5	-	Introns/Intergenic	-	-	GCAAAGCAAACACTCCATCA	TAGATGTGCCATGTGGTGGT	119	A	A	A	NA
PMS2942	Os_Chr05	17874179	17874192	(TA)7	(TA)7	(TA)7	(TA)7	(TA)7	(TA)7	(TA)6	(TA)7	(TA)7	(TA)7	(TA)7	-	Introns/Intergenic	-	-	CACTACTCCCTCCGGTTTCA	ACGTTTTGGACAATGACACG	246	A	A	A	NA
PMS2943	Os_Chr05	17968290	17968309	(AT)10	(AT)10	(AT)10	(AT)10	(AT)10	(AT)10	(AT)7	(AT)10	(AT)10	(AT)10	(AT)10	-	Introns/Intergenic	-	-	TACCAACCTTGAATGCAACA	GGACCAAATATGAGCAGAGGA	234	A	A	A	NA
PMS2944	Os_Chr05	18016628	18016657	(TC)15	(TC)14	(TC)15	(TC)15	(TC)15	(TC)15	(TC)15	(TC)15	(TC)15	(TC)15	(TC)15	LOC_Os05g31020	URR	eukaryotic peptide chain release factor subunit 1-1, putative, expressed	-	GGTTGGTGAACAGCACACAT	CCGCGAGCTTTAGAAGAGAA	157	P	A	A	NA
PMS2945	Os_Chr05	18081414	18081449	(GA)18	(GA)16	(GA)18	(GA)18	(GA)18	(GA)16	(GA)18	(GA)8	(GA)18	(GA)18	(GA)18	-	Introns/Intergenic	-	-	GTGGCGAATAAATAGGCGAG	TTATGACCCTCTCCTGCCAC	103	P	A	A	NA
PMS2946	Os_Chr05	18125209	18125222	(AG)7	(AG)8	(AG)7	(AG)7	(AG)7	(AG)7	(AG)7	(AG)7	(AG)7	(AG)7	(AG)7	-	Introns/Intergenic	-	-	AGCCACTCATCCGAAACAAC	ATATGGTGGAGTTGAGGCCA	256	P	A	A	NA
PMS2947	Os_Chr05	18232417	18232428	(CA)6	(CA)7	(CA)7	(CA)7	(CA)7	(CA)7	(CA)6	(CA)6	(CA)6	(CA)6	(CA)7	-	Introns/Intergenic	-	-	CTTTTGACCAATAATGCCCG	CATTATGACGGGTGGGCTAC	140	A	A	A	NA
PMS2948	Os_Chr05	18361671	18361686	(TC)8	(TC)8	(TC)8	(TC)8	(TC)8	(TC)8	(TC)7	(TC)8	(TC)8	(TC)8	(TC)8	-	Introns/Intergenic	-	-	AGTCTCTTTTGGCTTTGGGC	AATTGATTTCACCGGAAACG	225	A	A	A	NA
PMS2949	Os_Chr05	18407224	18407241	(GCG)6	(GCG)6	(GCG)6	(GCG)6	(GCG)6	(GCG)6	(GCG)6	(GCG)6	(GCG)6	(GCG)5	(GCG)6	LOC_Os05g31620	CDS	OsCML15 - Calmodulin-related calcium sensor protein, expressed	CODON_DELETION	GTCGAACTTCCTGAACACCC	TCACCTCTGCTTGTTGATCG	235	A	A	A	NA
PMS2950	Os_Chr05	18456781	18456801	(CGC)7	(CGC)7	(CGC)7	(CGC)7	(CGC)5	(CGC)7	(CGC)7	(CGC)7	(CGC)7	(CGC)7	(CGC)7	-	Introns/Intergenic	-	-	AAATCGTTTCTTTCCCCCAC	TGTGTCTTCCTCATTGCCTG	247	P	A	A	NA

Polymorphic SSR (PMS) markers	Chromosomes	Start physical positions (bp)	End physical positions (bp)	Nipponbare	Kasalath	Nagina22	IR64	Pokkali	Bala	Tainung67	Azucena	Moroberekan	*Oryza rufipogon*	*Oryza nivara*	MSU gene locus IDs	Structural annotation	Functional annotation	SSR functional effects	Forward primers (5'-3')	Reverse primers (5'-3')	Amplified product size (bp)	Markers exhibiting polymorphism within *indica* rice	Transcrption factor genes-derived markers	Known cloned genes-derived markers	PMS markers corresponding with RM markers
PMS2951	Os_Chr05	18500928	18500945	(AGC)6	(AGC)6	(AGC)6	(AGC)6	(AGC)6	(AGC)6	(AGC)6	(AGC)6	(AGC)6	(AGC)6	(AGC)5	LOC_Os05g31760	CDS	annexin, putative, expressed	CODON_CHANGE_PLUS_CODON_DELETION	CTCTTCCTCCATTTCTCCCC	GGGTTTGGCAAAGAACAGTG	280	A	A	A	NA
PMS2952	Os_Chr05	18621821	18621838	(TA)9	(TA)6	(TA)9	(TA)9	(TA)9	(TA)9	(TA)9	(TA)9	(TA)9	(TA)9	(TA)9	-	Introns/Intergenic	-	-	TCCCTCCGTACTCATAAAGGAA	TCGTTTAGAACAGCGACACG	241	P	A	A	NA
PMS2953	Os_Chr05	18658015	18658046	(CT)16	(CT)16	(CT)16	(CT)16	(CT)16	(CT)16	(CT)16	(CT)16	(CT)16	(CT)16	(CT)11	-	Introns/Intergenic	-	-	GTCGTCAATCATCGTTGGTG	GGTGGCTAAGCGGCTAGG	112	A	A	A	NA
PMS2954	Os_Chr05	18701786	18701811	(TC)13	(TC)12	(TC)13	(TC)13	(TC)13	(TC)13	(TC)13	(TC)13	(TC)13	(TC)13	(TC)13	-	Introns/Intergenic	-	-	ACGCACCTTCTCTCGTCTTC	ACATATCTAGGGGGCCATCC	260	P	A	A	NA
PMS2955	Os_Chr05	18704074	18704091	(TA)9	(TA)9	(TA)8	(TA)9	(TA)9	(TA)9	(TA)9	(TA)6	(TA)9	(TA)9	(TA)9	-	Introns/Intergenic	-	-	CAAACATTTTAAAACGGAGGGA	TTTTTACAACAAGATCTCACATGATT	261	P	A	A	NA
PMS2956	Os_Chr05	18710122	18710137	(CT)8	(CT)11	(CT)8	(CT)8	(CT)8	(CT)8	(CT)8	(CT)8	(CT)8	(CT)7	(CT)8	-	Introns/Intergenic	-	-	GGCGTCCCTCAACTAACAAA	CATCACACTCGTGCAGAGAAG	190	P	A	A	NA
PMS2957	Os_Chr05	18757934	18757948	(CAG)5	(CAG)6	(CAG)5	(CAG)5	(CAG)5	(CAG)6	(CAG)5	(CAG)5	(CAG)5	(CAG)5	(CAG)5	LOC_Os05g32170	CDS	TKL_IRAK_CR4L.5 - The CR4L subfamily has homology with Crinkly4, expressed	CODON_INSERTION	GAGATCCGCATCCTCTCCTC	TGGAATGGAGCAGCTCATAG	169	P	A	A	NA
PMS2958	Os_Chr05	18789685	18789705	(CCA)7	(CCA)7	(CCA)7	(CCA)7	(CCA)7	(CCA)7	(CCA)7	(CCA)7	(CCA)7	(CCA)6	(CCA)7	LOC_Os05g32220	URR	L1P family of ribosomal proteins domain containing protein, expressed	-	GAGAGAGAGCCCTATCCGCT	GACGAGGAGGAGCAGCACT	260	A	A	A	NA
PMS2959	Os_Chr05	18887560	18887586	(TCC)9	(TCC)6	(TCC)9	(TCC)9	(TCC)9	(TCC)9	(TCC)9	(TCC)9	(TCC)9	(TCC)9	(TCC)6	LOC_Os05g32380	CDS	phospholipase, putative, expressed	CODON_DELETION	GTCCTGGCTATCGTGGTGAT	CCACACGCACCTCTCCTT	235	P	A	A	NA
PMS2960	Os_Chr05	18999089	18999112	(TA)12	(TA)12	(TA)12	(TA)12	(TA)12	(TA)6	(TA)12	(TA)12	(TA)12	(TA)12	(TA)12	-	Introns/Intergenic	-	-	CAGTAAGCATGCAAAACGGA	GGAACCCAGAAGAACACCCT	197	P	A	A	NA
PMS2961	Os_Chr05	19067755	19067784	(TGA)10	(TGA)10	(TGA)5	(TGA)10	(TGA)10	(TGA)10	(TGA)10	(TGA)10	(TGA)10	(TGA)10	(TGA)10	-	Introns/Intergenic	-	-	GGGTTTGGGAAGGAGGAATGG	GAAGAGGAGAGAGCCTTGCTTGTCG	120	P	A	A	RM8211
PMS2962	Os_Chr05	19178686	19178706	(GCC)7	(GCC)7	(GCC)7	(GCC)7	(GCC)7	(GCC)7	(GCC)7	(GCC)5	(GCC)7	(GCC)7	(GCC)7	LOC_Os05g32760	CDS	BZIP protein, putative, expressed	CODON_CHANGE_PLUS_CODON_DELETION	GGAGGCAGTGGATGAAGT	GTCGTCGTCGTCGGTTGG	163	A	A	A	NA
PMS2963	Os_Chr05	19215936	19215959	(TC)12	(TC)18	(TC)12	(TC)12	(TC)12	(TC)12	(TC)12	(TC)12	(TC)12	(TC)12	(TC)12	-	Introns/Intergenic	-	-	ATGCGGGATCGTCTATGAAC	TGGACTTGCCACTTGTGTGT	266	P	A	A	NA

Polymorphic SSR (PMS) markers	Chromosomes	Start physical positions (bp)	End physical positions (bp)	Nipponbare	Kasalath	Nagina22	IR64	Pokkali	Bala	Tainung67	Azucena	Moroberekan	*Oryza rufipogon*	*Oryza nivara*	MSU gene locus IDs	Structural annotation	Functional annotation	SSR functional effects	Forward primers (5'-3')	Reverse primers (5'-3')	Amplified product size (bp)	Markers exhibiting polymorphism within *indica* rice	Transcrption factor genes-derived markers	Known cloned genes-derived markers	PMS markers corresponding with RM markers
PMS2964	Os_Chr05	19264630	19264691	(GA)31	(GA)31	(GA)31	(GA)31	(GA)31	(GA)31	(GA)31	(GA)27	(GA)31	(GA)31	(GA)31	-	Introns/Intergenic	-	-	CGTGCATTTGCAACTTAAACA	TAAGGAGCTGCACGAGAGGT	259	A	A	A	NA
PMS2965	Os_Chr05	19273505	19273536	(TA)16	(TA)16	(TA)16	(TA)16	(TA)16	(TA)16	(TA)6	(TA)16	(TA)16	(TA)6	(TA)6	-	Introns/Intergenic	-	-	CAGAAGATCTTGGGGGAATG	TTTGGAAAAATGTGCATAGCC	197	A	A	A	NA
PMS2966	Os_Chr05	19278738	19278749	(GT)6	(GT)6	(GT)6	(GT)6	(GT)6	(GT)6	(GT)7	(GT)6	(GT)7	(GT)6	(GT)6	-	Introns/Intergenic	-	-	TATGCCGGAACAAGACAACA	CGCATGCACACAAATACACA	253	A	A	A	NA
PMS2967	Os_Chr05	19325614	19325629	(CT)8	(CT)7	(CT)8	(CT)8	(CT)8	(CT)8	(CT)8	(CT)8	(CT)8	(CT)8	(CT)6	-	Introns/Intergenic	-	-	CTCTCAAAAGAGAGGACACGC	AGGACGACGACGAGGATG	267	P	A	A	NA
PMS2968	Os_Chr05	19328121	19328138	(GCT)6	(GCT)6	(GCT)6	(GCT)6	(GCT)6	(GCT)6	(GCT)5	(GCT)6	(GCT)6	(GCT)6	(GCT)6	LOC_Os05g32970	CDS	expressed protein	CODON_DELETION	TGCTCTTGCCCTTTTTCTTG	ACAGCTAGCCCATTTGGTTG	278	A	A	A	NA
PMS2969	Os_Chr05	19353759	19353779	(GGA)7	(GGA)8	(GGA)8	(GGA)8	(GGA)7	(GGA)8	(GGA)7	(GGA)8	(GGA)7	(GGA)7	(GGA)7	LOC_Os05g33010	CDS	heat shock protein DnaJ, putative, expressed	CODON_INSERTION	CATACGAACGCCACACAGAC	GGTCCAGTATCCCCCTCC	276	P	A	A	NA
PMS2970	Os_Chr05	19452664	19452681	(TC)9	(TC)9	(TC)9	(TC)9	(TC)9	(TC)9	(TC)9	(TC)9	(TC)9	(TC)6	(TC)9	LOC_Os05g33170	CDS	expressed protein	CODON_DELETION	AGTGTCCACGTCAGCCTTCT	CGACGTTGACGAAGATGATG	116	A	A	A	NA
PMS2971	Os_Chr05	19480819	19480836	(CAG)6	(CAG)5	(CAG)6	(CAG)6	(CAG)6	(CAG)6	(CAG)6	(CAG)6	(CAG)5	(CAG)6	(CAG)6	LOC_Os05g33220	URR	AWPM-19-like membrane family protein, putative, expressed	-	GCGTATATATTGGCTCCCGA	TGCTGATCTTCCCTCTTGCT	127	P	A	A	NA
PMS2972	Os_Chr05	19653795	19653824	(TC)15	(TC)13	(TC)8	(TC)15	(TC)15	(TC)15	(TC)10	(TC)15	(TC)15	(TC)15	(TC)15	-	Introns/Intergenic	-	-	CCGGTCATACCTCCTCTTCA	ACCAACGCCTACCTCAACC	181	P	A	A	NA
PMS2973	Os_Chr05	19724504	19724524	(CGC)7	(CGC)7	(CGC)7	(CGC)7	(CGC)7	(CGC)5	(CGC)7	(CGC)7	(CGC)7	(CGC)7	(CGC)7	-	Introns/Intergenic	-	-	CTGGCCTTATCTTACCAGCG	GACCACCTCCCTCCTCCTAC	191	P	A	A	NA
PMS2974	Os_Chr05	19732780	19732830	(TTA)17	(TTA)17	(TTA)17	(TTA)17	(TTA)17	(TTA)17	(TTA)17	(TTA)17	(TTA)17	(TTA)17	(TTA)5	-	Introns/Intergenic	-	-	CATCATGTGGTAAGTGTGCAACG	GGTTGCGATGAGATTACGAGACC	317	A	A	A	RM18639
PMS2975	Os_Chr05	19776692	19776712	(GGA)7	(GGA)7	(GGA)7	(GGA)7	(GGA)7	(GGA)7	(GGA)7	(GGA)7	(GGA)7	(GGA)7	(GGA)5	LOC_Os05g33620	CDS	expressed protein	CODON_DELETION	CGGATAGCGGATAACCAGAA	GATGGAGGTGATCTTCCGC	236	A	A	A	NA
PMS2976	Os_Chr05	19820623	19820678	(AT)28	(AT)28	(AT)7	(AT)28	(AT)28	(AT)7	(AT)28	(AT)6	(AT)28	(AT)28	(AT)28	-	Introns/Intergenic	-	-	TAAAAGGGCATCCACATGGT	AAAAATTTACTCACTCCCTATTGTG	209	P	A	A	NA

Polymorphic SSR (PMS) markers	Chromosomes	Start physical positions (bp)	End physical positions (bp)	Nipponbare	Kasalath	Nagina22	IR64	Pokkali	Bala	Tainung67	Azucena	Moroberekan	*Oryza rufipogon*	*Oryza nivara*	MSU gene locus IDs	Structural annotation	Functional annotation	SSR functional effects	Forward primers (5'-3')	Reverse primers (5'-3')	Amplified product size (bp)	Markers exhibiting polymorphism within *indica* rice	Transcrption factor genes-derived markers	Known cloned genes-derived markers	PMS markers corresponding with RM markers
PMS2977	Os_Chr05	19828949	19828970	(AG)11	(AG)11	(AG)8	(AG)13	(AG)11	(AG)11	(AG)11	(AG)13	(AG)13	(AG)11	(AG)13	LOC_Os05g33690	URR	receptor-like protein kinase precursor, putative, expressed	-	TTCTTCGTCCACGAGAAAGC	TGCAGCAAGGAAGAACAATG	243	P	A	A	NA
PMS2978	Os_Chr05	19853979	19854034	(ATAG)14	(ATAG)15	(ATAG)14	(ATAG)14	(ATAG)14	(ATAG)15	(ATAG)14	(ATAG)14	(ATAG)14	(ATAG)15	(ATAG)14	-	Introns/Intergenic	-	-	CGGTAAATGTGGTGCCTTCT	TTTTTATCCATTGTTGATGCG	162	P	A	A	NA
PMS2979	Os_Chr05	19906260	19906313	(TA)27	(TA)27	(TA)23	(TA)24	(TA)23	(TA)18	(TA)27	(TA)11	(TA)11	(TA)8	(TA)27	-	Introns/Intergenic	-	-	ACTCACGGAATTGAAGGTGG	CGAAGATGGCATGTAAACGA	132	P	A	A	NA
PMS2980	Os_Chr05	19983882	19983899	(CCG)6	(CCG)5	(CCG)6	(CCG)5	(CCG)6	(CCG)5	(CCG)5	(CCG)6	(CCG)5	(CCG)6	(CCG)6	LOC_Os05g33880	CDS	cysteine proteinase inhibitor precursor protein, putative, expressed	CODON_DELETION	CGGGATCGGAGCAGTAGTAG	AGAAAGCGGACCTGGTTCTT	280	P	A	A	NA
PMS2981	Os_Chr05	19999446	19999466	(CTG)7	(CTG)6	(CTG)7	(CTG)7	(CTG)7	(CTG)7	(CTG)6	(CTG)7	(CTG)7	(CTG)7	(CTG)7	LOC_Os05g33900	CDS	auxin-induced protein 5NG4, putative, expressed	CODON_DELETION	CTTGGTGATCACGTTCATGC	TACACGATCGAGCACCAGAC	153	P	A	A	NA
PMS2982	Os_Chr05	20080235	20080262	(TC)14	(TC)11	(TC)14	(TC)14	(TC)14	(TC)14	(TC)14	(TC)12	(TC)11	(TC)14	(TC)14	-	Introns/Intergenic	-	-	ATCCCGAATCCAATTCCTTC	CGGGCAAAGCGAGATAGG	146	P	A	A	NA
PMS2983	Os_Chr05	20168243	20168262	(TA)10	(TA)10	(TA)10	(TA)8	(TA)8	(TA)10	(TA)10	(TA)10	(TA)10	(TA)10	(TA)10	-	Introns/Intergenic	-	-	GGGTCGGATGGAATATGTATG	GACAAGTGTGGAGATGCGTG	198	P	A	A	NA
PMS2984	Os_Chr05	20222982	20223005	(ATA)8	(ATA)9	(ATA)8	(ATA)8	(ATA)8	(ATA)8	(ATA)8	(ATA)8	(ATA)8	(ATA)8	(ATA)8	-	Introns/Intergenic	-	-	GGTTGAAGCTAGGAAGTGATACGC	CAGTGGTAGTTGGGTGTACTTGTCG	678	P	A	A	RM18665
PMS2985	Os_Chr05	20400878	20400897	(GA)10	(GA)8	(GA)10	(GA)10	(GA)10	(GA)10	(GA)10	(GA)10	(GA)10	(GA)10	(GA)10	-	Introns/Intergenic	-	-	CACGTACATTCGTTCGCGTTTCG	ATCGTATCTCGCCGTTGTCTCACG	449	P	A	A	RM18680
PMS2986	Os_Chr05	20471110	20471127	(CT)9	(CT)9	(CT)9	(CT)9	(CT)9	(CT)9	(CT)9	(CT)9	(CT)9	(CT)9	(CT)8	-	Introns/Intergenic	-	-	TCGATTGATCACCTCACCTCT	CTCGTCGTGCGTGTGAAG	235	A	A	A	NA
PMS2987	Os_Chr05	20508910	20508973	(TA)32	(TA)32	(TA)32	(TA)32	(TA)32	(TA)32	(TA)32	(TA)10	(TA)32	(TA)32	(TA)32	-	Introns/Intergenic	-	-	CAACCCTCCACCACTCACTT	AATTGGGCTAAATGGTGCAT	246	A	A	A	NA
PMS2988	Os_Chr05	20519963	20519980	(AT)9	(AT)9	(AT)9	(AT)9	(AT)8	(AT)9	(AT)9	(AT)9	(AT)9	(AT)9	(AT)9	-	Introns/Intergenic	-	-	GCAAACGGACCAAAGACCTA	TTGGCCAATTAGATATGGTTCC	221	P	A	A	NA
PMS2989	Os_Chr05	20524458	20524539	(CT)41	(CT)41	(CT)41	(CT)41	(CT)41	(CT)40	(CT)41	(CT)41	(CT)41	(CT)41	(CT)41	-	Introns/Intergenic	-	-	ATCGGTTTGACTTTTGTCCG	TAAGTTTTTGTCTTGGCCCC	250	P	A	A	NA

Polymorphic SSR (PMS) markers	Chromosomes	Start physical positions (bp)	End physical positions (bp)	Nipponbare	Kasalath	Nagina22	IR64	Pokkali	Bala	Tainung67	Azucena	Moroberekan	*Oryza rufipogon*	*Oryza nivara*	MSU gene locus IDs	Structural annotation	Functional annotation	SSR functional effects	Forward primers (5'-3')	Reverse primers (5'-3')	Amplified product size (bp)	Markers exhibiting polymorphism within *indica* rice	Transcrption factor genes-derived markers	Known cloned genes-derived markers	PMS markers corresponding with RM markers
PMS2990	Os_Chr05	20539692	20539718	(GGC)9	(GGC)9	(GGC)9	(GGC)9	(GGC)9	(GGC)9	(GGC)9	(GGC)8	(GGC)9	(GGC)9	(GGC)9	LOC_Os05g34630	CDS	hydrolase, alpha/beta fold family domain containing protein, expressed	CODON_DELETION	GTGGTGGTGAACTACTCGTGTCC	AACACGTACCCTCTCCTCCATCC	198	A	A	A	RM18702
PMS2991	Os_Chr05	20601817	20601834	(CCG)6	(CCG)6	(CCG)6	(CCG)6	(CCG)6	(CCG)6	(CCG)6	(CCG)5	(CCG)6	(CCG)6	(CCG)6	LOC_Os05g34730	CDS	SERF1	CODON_DELETION	ATCTCCGACACCCACTTCC	AAGACGGTACGTACATGGGC	115	A	A	P	NA
PMS2992	Os_Chr05	20602816	20602865	(TA)25	(TA)25	(TA)12	(TA)10	(TA)10	(TA)25	(TA)25	(TA)25	(TA)25	(TA)10	(TA)11	-	Introns/Intergenic	-	-	CCAGGAAACTCCTGTCAACATATCC	GTCTCGTTTGACGTTGATTAGCC	523	P	A	A	RM18707
PMS2993	Os_Chr05	20735575	20735592	(GGC)6	(GGC)6	(GGC)6	(GGC)6	(GGC)6	(GGC)6	(GGC)5	(GGC)6	(GGC)6	(GGC)6	(GGC)6	LOC_Os05g34900	URR	wound-responsive family protein, putative, expressed	-	CAGCAGTGGCTGCAGGAT	TACCTCACCTGCCTTCCTTG	227	A	A	A	NA
PMS2994	Os_Chr05	20945286	20945303	(GCG)6	(GCG)6	(GCG)6	(GCG)6	(GCG)6	(GCG)6	(GCG)5	(GCG)6	(GCG)6	(GCG)6	(GCG)5	LOC_Os05g35266	CDS	galactosyltransferase, putative, expressed	CODON_DELETION	GCTGCTCTTCCTTGTGGCTA	AAGTCCGTCGACAGGATGAG	159	A	A	A	NA
PMS2995	Os_Chr05	20959805	20959824	(CT)10	(CT)10	(CT)10	(CT)10	(CT)10	(CT)9	(CT)10	(CT)10	(CT)10	(CT)10	(CT)10	-	Introns/Intergenic	-	-	GAGGCCATCCAACCCAGTTGC	CATGACTCTGCTGCTGGAGGAACC	481	P	A	A	RM18731
PMS2996	Os_Chr05	20975192	20975225	(AT)17	(AT)17	(AT)16	(AT)17	(AT)17	(AT)17	(AT)17	(AT)8	(AT)17	(AT)17	(AT)17	-	Introns/Intergenic	-	-	ACTAGCAAGACCTACTCCATTCG	GCTAACCAGGTCTTTCTTACACG	421	P	A	A	RM18733
PMS2997	Os_Chr05	21031326	21031351	(GA)13	(GA)13	(GA)13	(GA)13	(GA)13	(GA)13	(GA)13	(GA)10	(GA)13	(GA)13	(GA)10	-	Introns/Intergenic	-	-	GGGGAGAGATGGGAGAGC	TCGAGTTCAATTCCGAATCC	185	A	A	A	NA
PMS2998	Os_Chr05	21063527	21063540	(TC)7	(TC)12	(TC)7	(TC)7	(TC)7	(TC)12	(TC)7	(TC)7	(TC)7	(TC)7	(TC)12	-	Introns/Intergenic	-	-	CCTTCCGAACCTGTAGACCA	CCGATCCAGCATAGCTCATT	138	P	A	A	NA
PMS2999	Os_Chr05	21083930	21083947	(CGG)6	(CGG)6	(CGG)6	(CGG)6	(CGG)6	(CGG)6	(CGG)6	(CGG)6	(CGG)6	(CGG)6	(CGG)5	LOC_Os05g35480	URR	anthranilate phosphoribosyltransferase, putative, expressed	-	GTAGGTGGACGCGATCTTG	ACACTAGAGGCGAGACCCAA	262	A	A	A	NA
PMS3000	Os_Chr05	21231136	21231156	(GAG)7	(GAG)6	(GAG)7	(GAG)7	(GAG)6	(GAG)6	(GAG)7	(GAG)7	(GAG)7	(GAG)7	(GAG)6	-	Introns/Intergenic	-	-	CACACCCGCACCTACCTAGT	CCTCCACCTGTATCCCTCAA	146	P	A	A	NA
PMS3001	Os_Chr05	21247875	21247912	(TC)19	(TC)19	(TC)19	(TC)19	(TC)19	(TC)19	(TC)19	(TC)19	(TC)19	(TC)13	(TC)19	-	Introns/Intergenic	-	-	AGGCGGCAAAGTTCACTGTA	GATGCACAATCCTGCAAAGA	198	A	A	A	NA
PMS3002	Os_Chr05	21252013	21252044	(TG)16	(TG)16	(TG)7	(TG)7	(TG)7	(TG)16	(TG)16	(TG)16	(TG)16	(TG)16	(TG)16	-	Introns/Intergenic	-	-	CCGTGTGTTGGCTTAGAATCAAGG	GCCACTTTCCAAACATCAGAAAGC	126	P	A	A	RM18751

Polymorphic SSR (PMS) markers	Chromosomes	Start physical positions (bp)	End physical positions (bp)	Nipponbare	Kasalath	Nagina22	IR64	Pokkali	Bala	Tainung67	Azucena	Moroberekan	*Oryza rufipogon*	*Oryza nivara*	MSU gene locus IDs	Structural annotation	Functional annotation	SSR functional effects	Forward primers (5'-3')	Reverse primers (5'-3')	Amplified product size (bp)	Markers exhibiting polymorphism within *indica* rice	Transcrption factor genes-derived markers	Known cloned genes-derived markers	PMS markers corresponding with RM markers
PMS3003	Os_Chr05	21393861	21393876	(TA)8	(TA)8	(TA)8	(TA)8	(TA)8	(TA)8	(TA)6	(TA)8	(TA)8	(TA)8	(TA)8	-	Introns/Intergenic	-	-	CTCCCTCCGGTGTC ATTTTA	ATCAAAGTGAACAGG CCCAT	162	A	A	A	NA
PMS3004	Os_Chr05	21404781	21404798	(GCG)6	(GCG)6	(GCG)6	(GCG)6	(GCG)6	(GCG)6	(GCG)6	(GCG)5	(GCG)6	(GCG)6	(GCG)6	LOC_Os05g36100	CDS	AP2 domain containing protein, expressed	CODON_DELETION	GGCTCGATCCCAGT AACAAT	ATCGTGGACATCTCC TGAGC	257	A	P	A	NA
PMS3005	Os_Chr05	21410357	21410380	(CTG)8	(CTG)7	(CTG)8	(CTG)8	(CTG)8	(CTG)7	(CTG)8	(CTG)8	(CTG)8	(CTG)8	(CTG)8	LOC_Os05g36110	URR	protein of unknown function DUF502 domain containing protein, expressed	-	CCTTCCTCCCTCTC GCTTAC	GATGGTAGCTAGTG GCCGTG	159	P	A	A	NA
PMS3006	Os_Chr05	21466886	21466903	(AAG)6	(AAG)5	(AAG)6	(AAG)6	(AAG)6	(AAG)5	(AAG)6	(AAG)6	(AAG)6	(AAG)6	(AAG)6	LOC_Os05g36200	URR	retrotransposon protein, putative, Ty1-copia subclass, expressed	-	AGCTCAGCAGACCA CCAACT	TTACCATACCCGCTT CCATC	210	P	A	A	NA
PMS3007	Os_Chr05	21528441	21528458	(GCA)6	(GCA)5	(GCA)6	(GCA)6	(GCA)6	(GCA)5	(GCA)6	(GCA)6	(GCA)6	(GCA)6	(GCA)6	LOC_Os05g36370	CDS	expressed protein	CODON_DELETION	TACAGGAAGCCGGA GAGCTA	GTGCGTAGTACGGC TTGACA	232	P	A	A	NA
PMS3008	Os_Chr05	21641013	21641030	(CA)9	(CA)9	(CA)9	(CA)9	(CA)8	(CA)9	(CA)9	(CA)9	(CA)9	(CA)9	(CA)9	LOC_Os05g37030	DRR	expressed protein	-	ACGCACGCGAAATT ATTACC	TTCTACTGCATGCGT CTTCG	163	P	A	A	NA
PMS3009	Os_Chr05	21651071	21651086	(TC)8	(TC)8	(TC)8	(TC)8	(TC)8	(TC)8	(TC)8	(TC)8	(TC)8	(TC)7	(TC)8	-	Introns/Intergenic	-	-	ACGCCATTCCTTTG TTTGAG	ACCTTTATAAGCCGC CCATT	177	A	A	A	NA
PMS3010	Os_Chr05	21787801	21787852	(TA)26	(TA)26	(TA)26	(TA)6	(TA)6	(TA)26	(TA)26	(TA)10	(TA)26	(TA)26	(TA)9	-	Introns/Intergenic	-	-	AAATTTCAATCATG GTGCCC	CGATTACACCACTGT GTTCGTT	201	P	A	A	NA
PMS3011	Os_Chr05	22003868	22003885	(GA)9	(GA)9	(GA)9	(GA)9	(GA)9	(GA)9	(GA)9	(GA)9	(GA)6	(GA)9	(GA)9	-	Introns/Intergenic	-	-	GCTCCAGCCCTTGT CTGTC	GGAAGAGAAATCGT CGGGAT	195	A	A	A	NA
PMS3012	Os_Chr05	22009677	22009756	(TA)40	(TA)40	(TA)40	(TA)40	(TA)40	(TA)40	(TA)8	(TA)40	(TA)13	(TA)16	(TA)12	-	Introns/Intergenic	-	-	GCTGGGAACCAGAA CGAAACC	ACACCTAAGTTCCCG TGACTTGC	198	A	A	A	RM18791
PMS3013	Os_Chr05	22078778	22078807	(TA)15	(TA)15	(TA)9	(TA)15	(TA)7	(TA)7	(TA)15	(TA)15	(TA)6	(TA)15	(TA)15	-	Introns/Intergenic	-	-	TGGCGTCAAATATT TAGGGA	ATGAAGCATGAGTTA CCCCG	164	P	A	A	NA
PMS3014	Os_Chr05	22113897	22113936	(TA)20	(TA)20	(TA)9	(TA)20	(TA)20	(TA)20	(TA)6	(TA)13	(TA)20	(TA)20	(TA)20	-	Introns/Intergenic	-	-	ATTGGATCCTTCGC TGTCAC	CGTGTGCGACGAAT AAAAGA	257	P	A	A	NA
PMS3015	Os_Chr05	22246988	22247029	(AAG)14	(AAG)13	(AAG)14	(AAG)14	(AAG)14	(AAG)14	(AAG)14	(AAG)14	(AAG)14	(AAG)14	(AAG)14	-	Introns/Intergenic	-	-	GGCTAATCCCTCCG TTTGTA	GGGATGGTTTGAAG CATTGT	238	P	A	A	NA

Polymorphic SSR (PMS) markers	Chromosomes	Start physical positions (bp)	End physical positions (bp)	Nipponbare	Kasalath	Nagina22	IR64	Pokkali	Bala	Tainung67	Azucena	Moroberekan	*Oryza rufipogon*	*Oryza nivara*	MSU gene locus IDs	Structural annotation	Functional annotation	SSR functional effects	Forward primers (5'-3')	Reverse primers (5'-3')	Amplified product size (bp)	Markers exhibiting polymorphism within *indica* rice	Transcrption factor genes-derived markers	Known cloned genes-derived markers	PMS markers corresponding with RM markers
PMS3016	Os_Chr05	22266381	22266392	(GA)6	(GA)7	(GA)6	(GA)7	(GA)7	(GA)6	(GA)6	(GA)6	(GA)6	(GA)6	(GA)7	-	Introns/Intergenic	-	-	CTAGCAAGCATCCATCACCA	ACGTCGATGTCAGTTCCTCC	227	P	A	A	NA
PMS3017	Os_Chr05	22289805	22289832	(TC)14	(TC)14	(TC)14	(TC)14	(TC)14	(TC)14	(TC)13	(TC)14	(TC)14	(TC)14	(TC)14	-	Introns/Intergenic	-	-	GTGGATCCTTATCGCCGTC	CCAGCTTGCTTTTTCCAGAG	177	A	A	A	NA
PMS3018	Os_Chr05	22309639	22309660	(CT)11	(CT)12	(CT)11	(CT)12	(CT)12	(CT)10	(CT)11	(CT)11	(CT)11	(CT)11	(CT)11	-	Introns/Intergenic	-	-	GCTTAGCATCCAGTCTTTACCTATCG	TGAACGTCTCTCACTGGTTCAGG	106	P	A	A	RM18806
PMS3019	Os_Chr05	22354876	22354896	(GGC)7	(GGC)7	(GGC)7	(GGC)7	(GGC)7	(GGC)7	(GGC)7	(GGC)6	(GGC)7	(GGC)7	(GGC)5	LOC_Os05g38120	CDS	SH5	CODON_DELETION	CAGATCCCGAGCCAGAACT	TCTACGTCGAGCAAACCCTC	260	A	P	P	NA
PMS3020	Os_Chr05	22415407	22415427	(CGA)7	(CGA)6	(CGA)7	(CGA)7	(CGA)7	(CGA)6	(CGA)7	(CGA)7	(CGA)7	(CGA)6	(CGA)7	-	Introns/Intergenic	-	-	TGATAAGATTGTGTCGGGGG	ATCTAGGCCCATGTAGCGTG	229	P	A	A	NA
PMS3021	Os_Chr05	22494914	22494941	(CT)14	(CT)14	(CT)14	(CT)14	(CT)14	(CT)14	(CT)14	(CT)14	(CT)14	(CT)9	(CT)14	LOC_Os05g38370	URR	peptidyl-prolyl cis-trans isomerase, FKBP-type, putative, expressed	-	GGGCAACAGCAGACACGTACC	CGCATCACTATCTTCCCAATCTCG	190	A	A	A	RM3327
PMS3022	Os_Chr05	22569017	22569034	(GGT)6	(GGT)6	(GGT)6	(GGT)6	(GGT)6	(GGT)6	(GGT)5	(GGT)6	(GGT)6	(GGT)6	(GGT)6	LOC_Os05g38470	URR	expressed protein	-	GTATGGTCGTACCAGCAGCA	CGCCTTCTCCTTCTCCTTCT	258	A	A	A	NA
PMS3023	Os_Chr05	22716286	22716301	(AC)8	(AC)8	(AC)8	(AC)8	(AC)8	(AC)8	(AC)6	(AC)8	(AC)8	(AC)8	(AC)8	-	Introns/Intergenic	-	-	CAAAATCCTGAGGGGGAGAG	GTTTCGATCGGTAGTCCTGC	256	A	A	A	NA
PMS3024	Os_Chr05	22745425	22745442	(GTC)6	(GTC)6	(GTC)6	(GTC)5	(GTC)6	(GTC)6	(GTC)6	(GTC)6	(GTC)6	(GTC)5	(GTC)6	LOC_Os05g38770	CDS	protein kinase APK1B, chloroplast precursor, putative, expressed	CODON_DELETION	TCCCCTTCCTCTTCTTCGTT	GTGCTTGCAGGAGGAAGACT	219	P	P	A	NA
PMS3025	Os_Chr05	22747250	22747270	(CCG)7	(CCG)7	(CCG)7	(CCG)7	(CCG)7	(CCG)7	(CCG)6	(CCG)7	(CCG)7	(CCG)7	(CCG)7	LOC_Os05g38770	URR	protein kinase APK1B, chloroplast precursor, putative, expressed	-	TCAGGCTCTTCTTCTTTCCG	TCCGTGCGTCTTTCTTTTTC	150	A	P	A	NA
PMS3026	Os_Chr05	22773935	22773952	(GA)9	(GA)9	(GA)8	(GA)9	(GA)9	(GA)13	(GA)9	(GA)9	(GA)9	(GA)9	(GA)9	-	Introns/Intergenic	-	-	CCCAAACCCAAACCCTAGAT	CCGCTTCCTCTCTCTCTCCT	222	P	A	A	NA
PMS3027	Os_Chr05	22789340	22789354	(CCG)5	(CCG)7	(CCG)5	(CCG)5	(CCG)5	(CCG)5	(CCG)5	(CCG)5	(CCG)5	(CCG)7	(CCG)5	LOC_Os05g38850	URR	MCM8 - Putative minichromosome maintenance MCM family subunit 8, expressed	-	AATTTGTGATTGGGCTCGAA	TCGTACATGTTCGCCTGAGA	253	P	A	A	NA
PMS3028	Os_Chr05	22802600	22802659	(TCT)20	(TCT)20	(TCT)20	(TCT)13	(TCT)20	(TCT)20	(TCT)20	(TCT)20	(TCT)20	(TCT)20	(TCT)20	LOC_Os05g38870	CDS	conserved hypothetical protein	CODON_DELETION	TGACGTGGCATCCTAGTCAG	GCCCACCAGCCTCACTTA	202	P	A	A	NA

Polymorphic SSR (PMS) markers	Chromosomes	Start physical positions (bp)	End physical positions (bp)	Nipponbare	Kasalath	Nagina22	IR64	Pokkali	Bala	Tainung67	Azucena	Moroberekan	*Oryza rufipogon*	*Oryza nivara*	MSU gene locus IDs	Structural annotation	Functional annotation	SSR functional effects	Forward primers (5'-3')	Reverse primers (5'-3')	Amplified product size (bp)	Markers exhibiting polymorphism within *indica* rice	Transcrption factor genes-derived markers	Known cloned genes-derived markers	PMS markers corresponding with RM markers
PMS3029	Os_Chr05	22806480	22806527	(AT)24	(AT)11	(AT)7	(AT)6	(AT)6	(AT)24	(AT)24	(AT)24	(AT)24	(AT)6	(AT)24	-	Introns/Intergenic	-	-	TCCATTTACTTCAACGCACAA	GAGTGCGTGTCCATTACGTG	270	P	A	A	NA
PMS3030	Os_Chr05	22811691	22811710	(AG)10	(AG)10	(AG)10	(AG)10	(AG)10	(AG)10	(AG)10	(AG)10	(AG)10	(AG)8	(AG)10	-	Introns/Intergenic	-	-	CCGGTGCCTCTCTTGGATCTCG	GCTCCCTCTACTGTTGCTCGTTCG	278	A	A	A	RM18831
PMS3031	Os_Chr05	22812417	22812430	(GA)7	(GA)7	(GA)7	(GA)7	(GA)9	(GA)7	(GA)7	(GA)7	(GA)7	(GA)7	(GA)9	-	Introns/Intergenic	-	-	GACCCTGCGAGAACAGAAAA	GCTTTATCCAACGTGGCAGT	151	P	A	A	NA
PMS3032	Os_Chr05	22889018	22889037	(CT)10	(CT)10	(CT)10	(CT)10	(CT)10	(CT)8	(CT)10	(CT)10	(CT)10	(CT)10	(CT)10	-	Introns/Intergenic	-	-	CCTTGGTCTAAAAGGAGCCC	GAAGGAGGGAGAGGAGGAGA	236	P	A	A	NA
PMS3033	Os_Chr05	22961344	22961364	(CTA)7	(CTA)5	(CTA)6	(CTA)6	(CTA)6	(CTA)6	(CTA)7	(CTA)7	(CTA)7	(CTA)6	(CTA)6	LOC_Os05g39150	URR	expressed protein	-	CTCTCGGTTTCTCCCACAAA	ACCGGTCCATCTTCGACA	255	P	A	A	NA
PMS3034	Os_Chr05	22970194	22970211	(CAC)6	(CAC)6	(CAC)6	(CAC)6	(CAC)6	(CAC)6	(CAC)6	(CAC)6	(CAC)6	(CAC)5	(CAC)6	-	Introns/Intergenic	-	-	CAACTGGACCCGACAGTTTT	CTCACCGGAGAGGCCAAG	237	A	A	A	NA
PMS3035	Os_Chr05	22973692	22973715	(GA)12	(GA)12	(GA)12	(GA)12	(GA)12	(GA)14	(GA)12	(GA)12	(GA)12	(GA)12	(GA)14	-	Introns/Intergenic	-	-	CTTTAAAAATCAGACGCCCG	TAGGCCTCAAACCAAACCAG	112	P	A	A	NA
PMS3036	Os_Chr05	23054027	23054044	(CCT)6	(CCT)6	(CCT)6	(CCT)6	(CCT)6	(CCT)6	(CCT)6	(CCT)6	(CCT)6	(CCT)6	(CCT)5	-	Introns/Intergenic	-	-	TTCTCTCCTTCGTTAAAAACC	GGCTCGCTTCGTCACTGC	176	A	A	A	NA
PMS3037	Os_Chr05	23111355	23111372	(GCG)6	(GCG)6	(GCG)6	(GCG)5	(GCG)6	(GCG)6	(GCG)6	(GCG)6	(GCG)6	(GCG)6	(GCG)6	LOC_Os05g39400	CDS	expressed protein	CODON_CHANGE_PLUS_CODON_DELETION	AACACCAACGCCCAACTAAC	CTCCCTCCTCCTCGTACCC	261	P	A	A	NA
PMS3038	Os_Chr05	23128331	23128370	(AG)20	(AG)20	(AG)20	(AG)20	(AG)20	(AG)20	(AG)20	(AG)20	(AG)20	(AG)20	(AG)12	-	Introns/Intergenic	-	-	GGGAAGCGTCTTTGCGAG	GCCCATCTCTCCCTCTCC	237	A	A	A	NA
PMS3039	Os_Chr05	23167646	23167709	(AT)32	(AT)32	(AT)10	(AT)32	(AT)9	(AT)32	(AT)32	(AT)32	(AT)32	(AT)20	(AT)32	-	Introns/Intergenic	-	-	TCTCATTACAATTGTGCAATCATTT	GCATCGCTGGTTTAATGGAT	243	P	A	A	NA
PMS3040	Os_Chr05	23194912	23194929	(TGG)6	(TGG)6	(TGG)6	(TGG)6	(TGG)6	(TGG)5	(TGG)6	(TGG)6	(TGG)6	(TGG)6	(TGG)5	LOC_Os05g39520	URR	methyltransferase, putative, expressed	-	AGAGATCGAAGACGGTGGTG	GAAGGGGAAGAAGGCTCATC	235	P	A	A	NA
PMS3041	Os_Chr05	23230236	23230253	(CG)9	(CG)7	(CG)7	(CG)7	(CG)9	(CG)7	(CG)9	(CG)9	(CG)9	(CG)6	(CG)9	-	Introns/Intergenic	-	-	GTCAACAACGTCGTACAGGG	CAACTGCAGCACGGACTCTA	279	P	A	A	NA

Polymorphic SSR (PMS) markers	Chromosomes	Start physical positions (bp)	End physical positions (bp)	Nipponbare	Kasalath	Nagina22	IR64	Pokkali	Bala	Tainung67	Azucena	Moroberekan	*Oryza rufipogon*	*Oryza nivara*	MSU gene locus IDs	Structural annotation	Functional annotation	SSR functional effects	Forward primers (5'-3')	Reverse primers (5'-3')	Amplified product size (bp)	Markers exhibiting polymorphism within *indica* rice	Transcrption factor genes-derived markers	Known cloned genes-derived markers	PMS markers corresponding with RM markers
PMS3042	Os_Chr05	23278978	23279005	(AG)14	(AG)14	(AG)14	(AG)14	(AG)14	(AG)11	(AG)14	(AG)14	(AG)10	(AG)8	(AG)14	-	Introns/Intergenic	-	-	CTACTGAGCCATCG CATGAGTGC	TAGTTGTCTCCTGCC GTTGTTGG	134	P	A	A	RM1187
PMS3043	Os_Chr05	23343998	23344021	(CCG)8	(CCG)8	(CCG)5	(CCG)7	(CCG)8	(CCG)8	(CCG)8	(CCG)8	(CCG)8	(CCG)8	(CCG)8	-	Introns/Intergenic	-	-	AGGGCACGTGCATT CCTAGAGC	GGAGAAGAAGGGCA CATGCATCC	150	P	A	A	RM18862
PMS3044	Os_Chr05	23407905	23407944	(TG)20	(TG)20	(TG)20	(TG)20	(TG)20	(TG)20	(TG)20	(TG)20	(TG)20	(TG)7	(TG)20	-	Introns/Intergenic	-	-	CAAGGCAGAAGAAC AAACGTTCC	CGTATTGTGTGGCTG CTGCTAGG	143	A	A	A	RM5575
PMS3045	Os_Chr05	23419057	23419077	(CCG)7	(CCG)7	(CCG)7	(CCG)7	(CCG)7	(CCG)7	(CCG)7	(CCG)7	(CCG)7	(CCG)6	(CCG)7	LOC_Os05g39840	CDS	expressed protein	CODON_CHANGE_PLUS_CODON_DELETION	GCAGATCCTGAGCC AGAAGT	TACACAGCCTTGGTG CTGAA	222	A	A	A	NA
PMS3046	Os_Chr05	23467801	23467850	(TA)25	(TA)21	(TA)25	(TA)25	(TA)25	(TA)21	(TA)6	(TA)25	(TA)18	(TA)25	(TA)25	-	Introns/Intergenic	-	-	GCATGAGAAACACA TGGAACTGG	CATCCAATCAACTAC TCCCTCTATCC	384	P	A	A	RM18870
PMS3047	Os_Chr05	23497855	23497874	(GC)10	(GC)6	(GC)10	(GC)10	(GC)10	(GC)10	(GC)10	(GC)10	(GC)10	(GC)8	(GC)10	-	Introns/Intergenic	-	-	CCAGCTCTGTTGCG AGTGACC	AAGCAGTTGGTGGC TCTGTTACG	158	P	A	A	RM18872
PMS3048	Os_Chr05	23504816	23504833	(GA)9	(GA)9	(GA)9	(GA)9	(GA)9	(GA)9	(GA)9	(GA)9	(GA)9	(GA)9	(GA)10	-	Introns/Intergenic	-	-	CAGGGGAACATGA GAGAGGA	ATCTAATGTGGCGGC TGAGT	124	A	A	A	NA
PMS3049	Os_Chr05	23538888	23538905	(GGC)6	(GGC)5	(GGC)6	(GGC)6	(GGC)6	(GGC)5	(GGC)6	(GGC)6	(GGC)6	(GGC)5	(GGC)6	LOC_Os05g40070	CDS	WRKY84, expressed	CODON_DELETION	GAGCTGAGGGACAT GGAGAG	GGGTACACCTCAGC CAGCTA	263	P	P	A	NA
PMS3050	Os_Chr05	23642840	23642860	(CCA)7	(CCA)7	(CCA)7	(CCA)7	(CCA)7	(CCA)7	(CCA)7	(CCA)7	(CCA)7	(CCA)6	(CCA)7	LOC_Os05g40260	URR	acetyltransferase, GNAT family, putative, expressed	-	AATCGGCTACCACT GACCAC	AAAGAGGACGGTGT GACGAC	245	A	A	A	NA
PMS3051	Os_Chr05	23676700	23676723	(CAC)8	(CAC)8	(CAC)6	(CAC)8	(CAC)8	(CAC)8	(CAC)8	(CAC)8	(CAC)8	(CAC)8	(CAC)6	-	Introns/Intergenic	-	-	TCTGATGCTCCTGC AACAAC	TCTTTCTCTGAATTG CGGCT	151	P	A	A	NA
PMS3052	Os_Chr05	23677011	23677031	(GGC)7	(GGC)5	(GGC)7	(GGC)7	(GGC)7	(GGC)7	(GGC)7	(GGC)7	(GGC)7	(GGC)7	(GGC)7	LOC_Os05g40290	URR	KH domain-containing protein, putative, expressed	-	AAATCAAAACCCAA TCGTGG	TTCTTTGGTTTCCTC CTCCC	199	P	A	A	NA
PMS3053	Os_Chr05	23701053	23701076	(CGG)8	(CGG)8	(CGG)8	(CGG)8	(CGG)8	(CGG)8	(CGG)8	(CGG)6	(CGG)8	(CGG)8	(CGG)8	-	Introns/Intergenic	-	-	CTAGAGATCGCGCA ATGTGA	CGTCTTCTCAACTTC CCTCG	239	A	A	A	NA
PMS3054	Os_Chr05	23777928	23777949	(GA)11	(GA)11	(GA)11	(GA)11	(GA)11	(GA)7	(GA)11	(GA)11	(GA)11	(GA)7	(GA)11	-	Introns/Intergenic	-	-	AGAGCGGAGAAGA GGGAGAG	ATCCCGAATCCAAAT CCTTC	160	P	A	A	NA

Polymorphic SSR (PMS) markers	Chromosomes	Start physical positions (bp)	End physical positions (bp)	Nipponbare	Kasalath	Nagina22	IR64	Pokkali	Bala	Tainung67	Azucena	Moroberekan	*Oryza rufipogon*	*Oryza nivara*	MSU gene locus IDs	Structural annotation	Functional annotation	SSR functional effects	Forward primers (5'-3')	Reverse primers (5'-3')	Amplified product size (bp)	Markers exhibiting polymorphism within *indica* rice	Transcrption factor genes-derived markers	Known cloned genes-derived markers	PMS markers corresponding with RM markers
PMS3055	Os_Chr05	23871688	23871705	(CGG)6	(CGG)6	(CGG)6	(CGG)6	(CGG)6	(CGG)5	(CGG)6	(CGG)6	(CGG)6	(CGG)6	(CGG)6	LOC_Os05g40710	CDS	DELLA protein GAI, putative, expressed	CODON_CHANGE_PLUS_CODON_DELETION	AGCCTTTGTTGTCCCATCAC	AGTACACCTCCTTGCACGCT	156	P	P	A	NA
PMS3056	Os_Chr05	24008898	24008921	(CAT)8	(CAT)5	(CAT)8	(CAT)8	(CAT)8	(CAT)5	(CAT)8	(CAT)8	(CAT)8	(CAT)8	(CAT)7	LOC_Os05g40940	CDS	retrotransposon protein, putative, unclassified, expressed	CODON_DELETION	TTATCATGAGGATTTGGCCC	CAGGAGCAGCCTTTTGTCTT	138	P	A	A	NA
PMS3057	Os_Chr05	24027811	24027831	(CGC)7	(CGC)7	(CGC)7	(CGC)7	(CGC)7	(CGC)7	(CGC)7	(CGC)7	(CGC)7	(CGC)7	(CGC)6	LOC_Os05g40990	URR	NADH-cytochrome b5 reductase, putative, expressed	-	AGAAGCCAATCCACCTCCTC	ATACCCTTGGGCTTCTTGCT	237	A	A	A	NA
PMS3058	Os_Chr05	24126570	24126590	(CGG)7	(CGG)6	(CGG)7	(CGG)7	(CGG)7	(CGG)7	(CGG)7	(CGG)7	(CGG)7	(CGG)7	(CGG)7	LOC_Os05g41180	URR	peptidase, T1 family, putative, expressed	-	CGACGTACGGAAGAGGAAAC	AACTTAAACCGCACCACGAC	244	P	A	A	NA
PMS3059	Os_Chr05	24156981	24156996	(AC)8	(AC)8	(AC)8	(AC)8	(AC)8	(AC)8	(AC)7	(AC)8	(AC)8	(AC)8	(AC)8	-	Introns/Intergenic	-	-	TGCTGCATACCCGTATCAAA	CTGCTCATGGAAGGTGGAGT	132	A	A	A	NA
PMS3060	Os_Chr05	24227395	24227440	(TA)23	(TA)23	(TA)23	(TA)23	(TA)6	(TA)23	(TA)7	(TA)23	(TA)9	(TA)23	(TA)23	-	Introns/Intergenic	-	-	TCAATTTCAACAATGGTGCC	GCGGTAAGACCAATGGATGT	274	P	A	A	NA
PMS3061	Os_Chr05	24240894	24240911	(GGA)6	(GGA)6	(GGA)6	(GGA)6	(GGA)6	(GGA)6	(GGA)6	(GGA)6	(GGA)6	(GGA)6	(GGA)5	LOC_Os05g41390	URR	cyclin, putative, expressed	-	AAGAGGAGGAGGAGAGGACG	AAATCCGTAGCTTGGAACCC	262	A	A	A	NA
PMS3062	Os_Chr05	24292010	24292027	(GGA)6	(GGA)6	(GGA)6	(GGA)6	(GGA)6	(GGA)6	(GGA)6	(GGA)6	(GGA)6	(GGA)5	(GGA)6	LOC_Os05g41490	CDS	circadian clock coupling factor ZGT, putative, expressed	CODON_DELETION	CCTGAGCTGCGTAAACAGTG	GCCGTCTCGAGTACTTCGTC	279	A	A	A	NA
PMS3063	Os_Chr05	24358017	24358031	(CAA)5	(CAA)7	(CAA)5	(CAA)5	(CAA)5	(CAA)7	(CAA)5	(CAA)5	(CAA)5	(CAA)5	(CAA)5	LOC_Os05g41590	URR	glycerol-3-phosphate dehydrogenase, putative, expressed	-	TTTCTCTACCCAACAAGGCG	AAACGGATAAAATTCGTGCG	280	P	A	A	NA
PMS3064	Os_Chr05	24413004	24413023	(TA)10	(TA)10	(TA)10	(TA)10	(TA)10	(TA)8	(TA)10	(TA)10	(TA)10	(TA)10	(TA)8	-	Introns/Intergenic	-	-	ACCTTCAATTCGTACGTGCATCC	TAGTTGGCATCACATCAGGATCG	155	P	A	A	RM18921
PMS3065	Os_Chr05	24416897	24416914	(GGC)6	(GGC)6	(GGC)6	(GGC)6	(GGC)6	(GGC)6	(GGC)5	(GGC)6	(GGC)6	(GGC)6	(GGC)6	-	Introns/Intergenic	-	-	CTTGGTCGCCTTTGTCCTC	TCTCCTTTATGAACAGCCGC	209	A	A	A	NA
PMS3066	Os_Chr05	24488861	24488881	(TGG)7	(TGG)7	(TGG)5	(TGG)7	(TGG)7	(TGG)7	(TGG)7	(TGG)7	(TGG)7	(TGG)7	(TGG)7	-	Introns/Intergenic	-	-	ACAGCTTACGCGGATAGATGAGG	GAAGCGTGAGTTCTTGGTCTTCC	207	P	A	A	RM18927
PMS3067	Os_Chr05	24587068	24587103	(ACCA)9	(ACCA)9	(ACCA)5	(ACCA)9	(ACCA)9	(ACCA)9	(ACCA)9	(ACCA)9	(ACCA)9	(ACCA)9	(ACCA)9	-	Introns/Intergenic	-	-	CTTCCCGCACTACACTGCACTCC	CTGCAACTTGCTCATGGAGTTGG	101	P	A	A	RM7081

Polymorphic SSR (PMS) markers	Chromosomes	Start physical positions (bp)	End physical positions (bp)	Nipponbare	Kasalath	Nagina22	IR64	Pokkali	Bala	Tainung67	Azucena	Moroberekan	*Oryza rufipogon*	*Oryza nivara*	MSU gene locus IDs	Structural annotation	Functional annotation	SSR functional effects	Forward primers (5'-3')	Reverse primers (5'-3')	Amplified product size (bp)	Markers exhibiting polymorphism within *indica* rice	Transcrption factor genes-derived markers	Known cloned genes-derived markers	PMS markers corresponding with RM markers
PMS3068	Os_Chr05	24627011	24627028	(GTC)6	(GTC)6	(GTC)6	(GTC)6	(GTC)5	(GTC)6	(GTC)6	(GTC)6	(GTC)6	(GTC)6	(GTC)6	-	Introns/Intergenic	-	-	CATGTCAGCCTCTCCACAAG	AGAGGTGTGATTCCGTTTGG	219	P	A	A	NA
PMS3069	Os_Chr05	24817418	24817433	(CT)8	(CT)8	(CT)8	(CT)8	(CT)7	(CT)8	(CT)8	(CT)8	(CT)8	(CT)8	(CT)7	-	Introns/Intergenic	-	-	ATACGCAGGTGGTGTGATGA	GATACAGCCTTGTTCTCCGC	162	P	A	A	NA
PMS3070	Os_Chr05	24825939	24825952	(CT)7	(CT)7	(CT)7	(CT)7	(CT)7	(CT)6	(CT)7	(CT)7	(CT)7	(CT)7	(CT)7	-	Introns/Intergenic	-	-	AATTTCTCTCGGCCCATCTT	AGAAGGAGAGTGAGGGGAGG	272	P	A	A	NA
PMS3071	Os_Chr05	24916144	24916159	(CT)8	(CT)8	(CT)6	(CT)8	(CT)8	(CT)8	(CT)8	(CT)8	(CT)8	(CT)8	(CT)8	-	Introns/Intergenic	-	-	TCCAAATCCCTTCCAAATCA	AGCTTCTACGGGAGAGGGAG	236	P	A	A	NA
PMS3072	Os_Chr05	25003368	25003391	(GAG)8	(GAG)6	(GAG)8	(GAG)8	(GAG)8	(GAG)8	(GAG)8	(GAG)8	(GAG)8	(GAG)8	(GAG)6	LOC_Os05g43060	CDS	retrotransposon, putative, centromere-specific, expressed	CODON_DELETION	GTCTGGGGTCCTCCAGTTCT	AGCCGCATTGCTCATATACC	219	P	A	A	NA
PMS3073	Os_Chr05	25176730	25176750	(CCG)7	(CCG)7	(CCG)6	(CCG)7	(CCG)7	(CCG)7	(CCG)7	(CCG)7	(CCG)7	(CCG)7	(CCG)7	LOC_Os05g43280	URR	MATH domain containing protein, expressed	-	TTCCTTTCCTCCTCCTCCAT	CCTACCGATCTAGGTCAGCG	160	P	A	A	NA
PMS3074	Os_Chr05	25293487	25293500	(TA)7	(TA)7	(TA)7	(TA)7	(TA)7	(TA)7	(TA)7	(TA)6	(TA)6	(TA)7	(TA)7	-	Introns/Intergenic	-	-	TGCATTTTGTGGGTTTTTAT	TCTCTCTTCTCTACAGCACAT	300	A	A	A	NA
PMS3075	Os_Chr05	25392166	25392186	(GGC)7	(GGC)7	(GGC)7	(GGC)7	(GGC)7	(GGC)7	(GGC)7	(GGC)7	(GGC)7	(GGC)7	(GGC)6	LOC_Os05g43670	CDS	IQ calmodulin-binding motif family protein, putative, expressed	CODON_DELETION	ATACTGATGGGGTTTGGGCT	CCGACCAGCTACTACTACCCC	249	A	A	A	NA
PMS3076	Os_Chr05	25404518	25404531	(AG)7	(AG)6	(AG)7	(AG)7	(AG)7	(AG)7	(AG)7	(AG)7	(AG)7	(AG)7	(AG)7	-	Introns/Intergenic	-	-	CTGCTCTTGACGGAGAGAGG	GGGTTGTTTGGATAACGTGG	115	P	A	A	NA
PMS3077	Os_Chr05	25412491	25412506	(CT)8	(CT)8	(CT)8	(CT)8	(CT)8	(CT)8	(CT)8	(CT)8	(CT)8	(CT)7	(CT)8	-	Introns/Intergenic	-	-	AAATCCCTCCGCAAAAGTCT	GAGAGGGAGGACTCGGTTTG	229	A	A	A	NA
PMS3078	Os_Chr05	25438518	25438529	(TA)6	(TA)6	(TA)6	(TA)7	(TA)6	(TA)6	(TA)6	(TA)6	(TA)6	(TA)6	(TA)7	LOC_Os05g43760	DRR	TCP family transcription factor, putative, expressed	-	AAGCGCATTATTTGGTACGG	GTCGGATACATGCAGCTCAA	273	P	A	A	NA
PMS3079	Os_Chr05	25475176	25475203	(AG)14	(AG)15	(AG)14	(AG)14	(AG)14	(AG)14	(AG)14	(AG)14	(AG)14	(AG)14	(AG)14	-	Introns/Intergenic	-	-	CCGTTGTTCTTGCTGCTGTCC	ATCCTGCATAGCCAAGAAACAGC	170	P	A	A	RM18969
PMS3080	Os_Chr05	25503740	25503757	(GCC)6	(GCC)8	(GCC)6	(GCC)6	(GCC)6	(GCC)8	(GCC)6	(GCC)6	(GCC)5	(GCC)6	(GCC)6	LOC_Os05g43860	CDS	expressed protein	CODON_DELETION	CCTTGTCCTCCAATAGCAGC	AGACAGAAGCCACGACCAAT	188	P	A	A	NA

Polymorphic SSR (PMS) markers	Chromosomes	Start physical positions (bp)	End physical positions (bp)	Nipponbare	Kasalath	Nagina22	IR64	Pokkali	Bala	Tainung67	Azucena	Moroberekan	*Oryza rufipogon*	*Oryza nivara*	MSU gene locus IDs	Structural annotation	Functional annotation	SSR functional effects	Forward primers (5'-3')	Reverse primers (5'-3')	Amplified product size (bp)	Markers exhibiting polymorphism within *indica* rice	Transcrption factor genes-derived markers	Known cloned genes-derived markers	PMS markers corresponding with RM markers
PMS3081	Os_Chr05	25574022	25574087	(TA)33	(TA)33	(TA)8	(TA)33	(TA)33	(TA)33	(TA)33	(TA)33	(TA)33	(TA)33	(TA)33	-	Introns/Intergenic	-	-	GACCTTCCCTCCTTTGTCTCTAGG	GGGTTTAGTCCATAACGAAGAACC	479	P	A	A	RM18975
PMS3082	Os_Chr05	25617171	25617182	(AT)6	(AT)6	(AT)6	(AT)6	(AT)6	(AT)6	(AT)6	(AT)6	(AT)6	(AT)7	(AT)6	-	Introns/Intergenic	-	-	TGTCGAAACTTAACCCCTGC	TCGTCGTAGACATGCTGCTC	277	A	A	A	NA
PMS3083	Os_Chr05	25660376	25660401	(TA)13	(TA)13	(TA)13	(TA)13	(TA)13	(TA)13	(TA)13	(TA)12	(TA)13	(TA)13	(TA)13	-	Introns/Intergenic	-	-	CAACTCATACACTACTACTGCTACTGC	TGCGCGTATGCAAAGTTTAT	264	A	A	A	NA
PMS3084	Os_Chr05	25693870	25693961	(TA)46	(TA)46	(TA)46	(TA)10	(TA)10	(TA)10	(TA)46	(TA)7	(TA)7	(TA)10	(TA)10	-	Introns/Intergenic	-	-	TTTGCGGGTGTTAATCTGGT	ACCACCGTTGAAAATGATGC	163	P	A	A	NA
PMS3085	Os_Chr05	25763217	25763228	(TG)6	(TG)8	(TG)6	(TG)6	(TG)6	(TG)8	(TG)6	(TG)6	(TG)6	(TG)6	(TG)8	-	Introns/Intergenic	-	-	CGTTTTCGGTAGGGATAGCA	AAAGCTACACCACCGAGTGA	170	P	A	A	NA
PMS3086	Os_Chr05	25788379	25788396	(CCG)6	(CCG)6	(CCG)6	(CCG)5	(CCG)6	(CCG)6	(CCG)6	(CCG)6	(CCG)6	(CCG)6	(CCG)6	LOC_Os05g44310	CDS	OsSec18	CODON_DELETION	TACGAGATCGGCAAAAGGAT	GTCGTCCCCTTTCTCTGCTC	259	P	A	P	NA
PMS3087	Os_Chr05	25809697	25809714	(TGA)6	(TGA)6	(TGA)6	(TGA)7	(TGA)7	(TGA)6	(TGA)6	(TGA)6	(TGA)6	(TGA)6	(TGA)6	LOC_Os05g44350	CDS	conserved uncharacterized protein, putative, expressed	CODON_CHANGE_PLUS_CODON_INSERTION	GTGGAGCAAAATTGGCTGAT	GCATCAAATGAAACCGTGTG	252	P	A	A	NA
PMS3088	Os_Chr05	25850708	25850725	(CGA)6	(CGA)6	(CGA)6	(CGA)6	(CGA)6	(CGA)6	(CGA)6	(CGA)6	(CGA)6	(CGA)5	(CGA)6	-	Introns/Intergenic	-	-	AACGTTGGTTCTATGCAGCC	TCGATCTTAACACGGAACCC	207	A	A	A	NA
PMS3089	Os_Chr05	25857225	25857264	(TA)20	(TA)20	(TA)20	(TA)20	(TA)11	(TA)20	(TA)20	(TA)20	(TA)20	(TA)20	(TA)20	-	Introns/Intergenic	-	-	GGAGTGGTCAAATAAGTTGCTTGC	GGCTCTTACTCGTCAATGAACTCC	303	P	A	A	RM19000
PMS3090	Os_Chr05	25995138	25995157	(GA)10	(GA)12	(GA)10	(GA)10	(GA)10	(GA)10	(GA)10	(GA)10	(GA)10	(GA)10	(GA)10	-	Introns/Intergenic	-	-	ACGACTGCAAAAGGACGG	CTCTTCTTTCGCCAGCCTCT	261	P	A	A	NA
PMS3091	Os_Chr05	26162007	26162069	(AAC)21	(AAC)20	(AAC)21	(AAC)20	(AAC)21	(AAC)20	(AAC)21	(AAC)21	(AAC)21	(AAC)21	(AAC)18	-	Introns/Intergenic	-	-	AGGAGACGATCGAAAAGTTAC	ACATGTAAAGAGTTATATGCTGA	192	P	A	A	NA
PMS3092	Os_Chr05	26187547	26187620	(TA)37	(TA)37	(TA)37	(TA)37	(TA)37	(TA)37	(TA)7	(TA)7	(TA)6	(TA)37	(TA)37	-	Introns/Intergenic	-	-	CGTTGCACTGAAGCAGACAT	TTTCCTTTCATGTCCTTGCC	273	A	A	A	NA
PMS3093	Os_Chr05	26339972	26339993	(TA)11	(TA)7	(TA)11	(TA)11	(TA)11	(TA)10	(TA)9	(TA)11	(TA)11	(TA)11	(TA)11	-	Introns/Intergenic	-	-	TCATTAGCACATGTGGGTTACGG	CTATATATGCCGGGAACGACAGC	299	P	A	A	RM19019

Polymorphic SSR (PMS) markers	Chromosomes	Start physical positions (bp)	End physical positions (bp)	Nipponbare	Kasalath	Nagina22	IR64	Pokkali	Bala	Tainung67	Azucena	Moroberekan	*Oryza rufipogon*	*Oryza nivara*	MSU gene locus IDs	Structural annotation	Functional annotation	SSR functional effects	Forward primers (5'-3')	Reverse primers (5'-3')	Amplified product size (bp)	Markers exhibiting polymorphism within *indica* rice	Transcrption factor genes-derived markers	Known cloned genes-derived markers	PMS markers corresponding with RM markers
PMS3094	Os_Chr05	26397960	26397971	(GA)6	(GA)7	(GA)7	(GA)6	(GA)6	(GA)7	(GA)6	(GA)6	(GA)6	(GA)6	(GA)6	-	Introns/Intergenic	-	-	CCTGCACCGGATGTGTGT	AATCGAAAGTGGTTTTATCCT	142	P	A	A	NA
PMS3095	Os_Chr05	26552298	26552315	(CT)9	(CT)9	(CT)9	(CT)9	(CT)9	(CT)9	(CT)8	(CT)9	(CT)9	(CT)9	(CT)9	-	Introns/Intergenic	-	-	GGCAATGTTCCCTTTTCGTA	TCCTAAGTAAGGCCCAAGCA	253	A	A	A	NA
PMS3096	Os_Chr05	26681155	26681222	(TA)34	(TA)34	(TA)34	(TA)6	(TA)27	(TA)34	(TA)34	(TA)34	(TA)8	(TA)34	(TA)34	-	Introns/Intergenic	-	-	GGACAACCTCCTTTGTCGAGATTCC	AAAGTTCACCGGTCGGAACATGG	410	P	A	A	RM19031
PMS3097	Os_Chr05	26727688	26727705	(GA)9	(GA)9	(GA)9	(GA)9	(GA)9	(GA)12	(GA)9	(GA)6	(GA)6	(GA)9	(GA)9	-	Introns/Intergenic	-	-	AGCGGGGGAGAAGGGGAGAG	AAATCCTTCCCAAATCAAAT	138	P	A	A	NA
PMS3098	Os_Chr05	26880417	26880434	(CGA)6	(CGA)6	(CGA)6	(CGA)6	(CGA)6	(CGA)6	(CGA)6	(CGA)6	(CGA)6	(CGA)5	(CGA)6	LOC_Os05g46370	CDS	OsLF	CODON_DELETION	GGCGAGTCGAACACCTACAC	TCTCTGGTAGCTTCCGCTTC	103	A	P	P	NA
PMS3099	Os_Chr05	26989829	26989864	(AATT)9	(AATT)8	(AATT)8	(AATT)8	(AATT)8	(AATT)8	(AATT)9	(AATT)8	(AATT)8	(AATT)8	(AATT)8	-	Introns/Intergenic	-	-	ACCAAACAGGACCCAATTATGGTAGC	GCAAAGAGGATTCCACGAGAAGC	158	A	A	A	RM7452
PMS3100	Os_Chr05	27223159	27223173	(AGG)5	(AGG)6	(AGG)6	(AGG)6	(AGG)6	(AGG)6	(AGG)5	(AGG)6	(AGG)6	(AGG)6	(AGG)6	LOC_Os05g47520	CDS	expressed protein	CODON_INSERTION	TACCAAGGTTGAAGAACGGC	TCCATCACTGTTTCAGCGAC	187	A	A	A	NA
PMS3101	Os_Chr05	27227101	27227121	(CGC)7	(CGC)7	(CGC)7	(CGC)7	(CGC)7	(CGC)7	(CGC)7	(CGC)7	(CGC)7	(CGC)5	(CGC)7	LOC_Os05g47530	CDS	Divergent PAP2 family domain containing protein, expressed	CODON_DELETION	CGAGGAGGTTGAGGAAGTTG	GTACACACCACCAACGAACG	249	A	A	A	NA
PMS3102	Os_Chr05	27287213	27287233	(GCC)7	(GCC)7	(GCC)7	(GCC)7	(GCC)7	(GCC)7	(GCC)7	(GCC)7	(GCC)7	(GCC)6	(GCC)7	LOC_Os05g47630	CDS	peptidyl-tRNA hydrolase, mitochondrial precursor protein, putative, expressed	CODON_DELETION	CGAACCCTAGAAGCATGGC	GTTGTGACGAGTCCCCTTGT	191	A	A	A	NA
PMS3103	Os_Chr05	27360985	27361002	(CGT)6	(CGT)6	(CGT)6	(CGT)6	(CGT)6	(CGT)6	(CGT)6	(CGT)6	(CGT)6	(CGT)5	(CGT)5	LOC_Os05g47770	CDS	serine/threonine-protein kinase At1g18390 precursor, putative, expressed	CODON_CHANGE_PLUS_CODON_DELETION	CGCCACTACTCTTCCACCTC	ACTTGATCTCCACCTGCTCG	141	A	A	A	NA
PMS3104	Os_Chr05	27521691	27521712	(AT)11	(AT)10	(AT)11	(AT)11	(AT)11	(AT)10	(AT)10	(AT)11	(AT)11	(AT)11	(AT)11	LOC_Os05g48000	DRR	expressed protein	-	TCGCTTGTTAATTTGAATTGC	TCGATACGGGGATACGACAT	261	P	A	A	NA
PMS3105	Os_Chr05	27542161	27542182	(GA)11	(GA)15	(GA)11	(GA)11	(GA)11	(GA)15	(GA)11	(GA)11	(GA)15	(GA)11	(GA)11	LOC_Os05g48040	URR	OsMATE2	-	AAAGCCGTCTCTGAGAAGAAGAGAGG	GGGAATCGTTTCGAGAGCAGAGG	137	P	A	P	RM19080
PMS3106	Os_Chr05	27579552	27579569	(TTC)6	(TTC)6	(TTC)6	(TTC)6	(TTC)6	(TTC)6	(TTC)6	(TTC)6	(TTC)5	(TTC)6	(TTC)6	-	Introns/Intergenic	-	-	ATGCAGATGGTGTGAGCTTG	TGAAACCAGGTAGAACCCCA	238	A	A	A	NA

Polymorphic SSR (PMS) markers	Chromosomes	Start physical positions (bp)	End physical positions (bp)	Nipponbare	Kasalath	Nagina22	IR64	Pokkali	Bala	Tainung67	Azucena	Moroberekan	*Oryza rufipogon*	*Oryza nivara*	MSU gene locus IDs	Structural annotation	Functional annotation	SSR functional effects	Forward primers (5'-3')	Reverse primers (5'-3')	Amplified product size (bp)	Markers exhibiting polymorphism within *indica* rice	Transcrption factor genes-derived markers	Known cloned genes-derived markers	PMS markers corresponding with RM markers
PMS3107	Os_Chr05	27676954	27676971	(GCG)6	(GCG)6	(GCG)6	(GCG)6	(GCG)6	(GCG)6	(GCG)6	(GCG)6	(GCG)6	(GCG)5	(GCG)6	LOC_Os05g48280	CDS	expressed protein	CODON_CHANGE_PLUS_CODON_DELETION	GTCACCTCCTCCCTCCCC	AAGACGATGAGATGATGGGC	149	A	A	A	NA
PMS3108	Os_Chr05	27681011	27681028	(CT)9	(CT)9	(CT)9	(CT)9	(CT)9	(CT)9	(CT)8	(CT)9	(CT)9	(CT)9	(CT)9	-	Introns/Intergenic	-	-	ATGAAATTGGCGAGCAAAAA	GGACCACGGATCATAACCAC	240	A	A	A	NA
PMS3109	Os_Chr05	27718860	27718877	(TA)9	(TA)9	(TA)9	(TA)9	(TA)9	(TA)9	(TA)9	(TA)7	(TA)9	(TA)9	(TA)9	-	Introns/Intergenic	-	-	AGAAGAGGGAGGACACGGTT	CCACTCCAATAGTCCACGCT	213	A	A	A	NA
PMS3110	Os_Chr05	27747696	27747716	(CTG)7	(CTG)7	(CTG)7	(CTG)7	(CTG)7	(CTG)7	(CTG)6	(CTG)7	(CTG)7	(CTG)7	(CTG)7	LOC_Os05g48390	URR	LTN1|OsPHO2	-	AGAAAGCCGGATATTTCGAGACG	ATAGAAATCACCAGGACCCATCG	186	A	A	P	RM19094
PMS3111	Os_Chr05	27824481	27824570	(AATAT)18	(AATAT)18	(AATAT)5	(AATAT)5	(AATAT)18	(AATAT)18	(AATAT)5	(AATAT)5	(AATAT)5	(AATAT)18	(AATAT)18	-	Introns/Intergenic	-	-	CCAAGAGGTCTGGAGATTGC	TGGAGCTCCTGGAACAACAT	231	P	A	A	NA
PMS3112	Os_Chr05	27829996	27830015	(AT)10	(AT)10	(AT)10	(AT)10	(AT)10	(AT)10	(AT)10	(AT)10	(AT)10	(AT)7	(AT)10	-	Introns/Intergenic	-	-	TTTGGTGCAACGCAATGTAT	TCAATTTCAACAATGGTGCC	207	A	A	A	NA
PMS3113	Os_Chr05	27840169	27840186	(CTG)6	(CTG)6	(CTG)6	(CTG)6	(CTG)6	(CTG)6	(CTG)6	(CTG)5	(CTG)6	(CTG)6	(CTG)6	LOC_Os05g48580	CDS	expressed protein	CODON_DELETION	CTCCACTGCGTGAAGCTCT	CACGCCCAACAACAATGTC	249	A	A	A	NA
PMS3114	Os_Chr05	28016760	28016783	(CT)12	(CT)12	(CT)12	(CT)12	(CT)12	(CT)12	(CT)12	(CT)12	(CT)13	(CT)12	(CT)13	-	Introns/Intergenic	-	-	GCAGTGTCATTCTCATGAAACCTACC	CAGACTCCAAAGCACCCATAACC	189	A	A	A	RM3170
PMS3115	Os_Chr05	28125302	28125316	(ACC)5	(ACC)7	(ACC)5	(ACC)5	(ACC)5	(ACC)7	(ACC)5	(ACC)7	(ACC)7	(ACC)5	(ACC)7	LOC_Os05g49050	CDS	UreG|OsUreG	CODON_INSERTION	CGTTAAGGCCCAACCAAATA	GCAGTGAGCGAGATCATCAG	276	P	A	P	NA
PMS3116	Os_Chr05	28144991	28145023	(CTT)11	(CTT)10	(CTT)11	(CTT)11	(CTT)11	(CTT)11	(CTT)11	(CTT)11	(CTT)11	(CTT)9	(CTT)11	-	Introns/Intergenic	-	-	ACGTGGAATCCAAATTGACAGC	TTCGCTGCACTGTTTACTCTTGG	121	P	A	A	RM6360
PMS3117	Os_Chr05	28154990	28155004	(GCG)5	(GCG)5	(GCG)5	(GCG)5	(GCG)5	(GCG)5	(GCG)5	(GCG)5	(GCG)5	(GCG)7	(GCG)5	LOC_Os05g49100	CDS	WRKY49, expressed	CODON_INSERTION	GAGGTGAAGAGGGAGCTGGT	TTCTTGTCTTCTCCATCGCC	278	A	A	A	NA
PMS3118	Os_Chr05	28159755	28159766	(AC)6	(AC)9	(AC)9	(AC)6	(AC)6	(AC)6	(AC)6	(AC)6	(AC)6	(AC)6	(AC)6	-	Introns/Intergenic	-	-	TGCAGGAAGTGCATTTGTTT	CGAGTCTGCAAAGTAGCACG	253	P	A	A	NA
PMS3119	Os_Chr05	28172092	28172109	(TC)9	(TC)10	(TC)9	(TC)9	(TC)9	(TC)11	(TC)9	(TC)9	(TC)9	(TC)9	(TC)10	-	Introns/Intergenic	-	-	ATCCCGAATCCAATTCCTTC	AACGAGAGCGAAGAGGGAG	211	P	A	A	NA

**Polymorphic SSR (PMS) markers**	**Chromosomes**	**Start physical positions (bp)**	**End physical positions (bp)**	**Nipponbare**	**Kasalath**	**Nagina22**	**IR64**	**Pokkali**	**Bala**	**Tainung67**	**Azucena**	**Moroberekan**	***Oryza rufipogon***	***Oryza nivara***	**MSU gene locus IDs**	**Structural annotation**	**Functional annotation**	**SSR functional effects**	**Forward primers (5'-3')**	**Reverse primers (5'-3')**	**Amplified product size (bp)**	**Markers exhibiting polymorphism within *indica* rice**	**Transcrption factor genes-derived markers**	**Known cloned genes-derived markers**	**PMS markers corresponding with RM markers**
PMS3120	Os_Chr05	28194194	28194208	(TGC)5	(TGC)5	(TGC)6	(TGC)6	(TGC)6	(TGC)6	(TGC)5	(TGC)6	(TGC)6	(TGC)5	(TGC)6	-	Introns/Intergenic	-	-	CTTGCCCTTCCATGACAGTT	CCCAAATTTATTCGTCACGG	157	P	A	A	NA
PMS3121	Os_Chr05	28196334	28196361	(TA)14	(TA)14	(TA)6	(TA)8	(TA)14	(TA)14	(TA)14	(TA)14	(TA)14	(TA)14	(TA)14	-	Introns/Intergenic	-	-	GAGCAGTTGAGTGCCCTAGC	CGAGAATCGGGTCTAATGGA	240	P	A	A	NA
PMS3122	Os_Chr05	28197802	28197816	(AGC)5	(AGC)5	(AGC)5	(AGC)5	(AGC)5	(AGC)5	(AGC)5	(AGC)5	(AGC)5	(AGC)6	(AGC)5	-	Introns/Intergenic	-	-	ACTCATGGTGATGTGGACGA	GCCGCCAATTCTTTACTCTTT	257	A	A	A	NA
PMS3123	Os_Chr05	28281182	28281196	(GGA)5	(GGA)5	(GGA)5	(GGA)5	(GGA)5	(GGA)5	(GGA)5	(GGA)5	(GGA)6	(GGA)5	(GGA)6	LOC_Os05g49290	URR	3-ketoacyl-CoA synthase, putative, expressed	-	CAGCTGGAGAAGAGTGGGAG	CTTCTCACAAAGCCCCAAAC	140	A	A	A	NA
PMS3124	Os_Chr05	28307163	28307183	(GCC)7	(GCC)5	(GCC)7	(GCC)7	(GCC)7	(GCC)7	(GCC)7	(GCC)5	(GCC)7	(GCC)7	(GCC)7	LOC_Os05g49350	CDS	DUF1645 domain containing protein, putative, expressed	CODON_DELETION	AAAAACTCCACCACCACCAG	GGTGAAGTCCTCCTCGTCAG	126	P	A	A	NA
PMS3125	Os_Chr05	28323439	28323484	(AG)23	(AG)23	(AG)23	(AG)23	(AG)23	(AG)23	(AG)23	(AG)19	(AG)23	(AG)23	(AG)23	-	Introns/Intergenic	-	-	CTCCAGACAAAGAGAAATGTGGTAGG	CCTCTCCAGGTTGGTTCTTCTCC	321	A	A	A	RM19134
PMS3126	Os_Chr05	28340525	28340557	(CTT)11	(CTT)11	(CTT)11	(CTT)11	(CTT)11	(CTT)11	(CTT)11	(CTT)11	(CTT)6	(CTT)11	(CTT)11	-	Introns/Intergenic	-	-	ATCCAGATCCACTTTGACCG	AAACTCCCAAGACATGGCAC	248	A	A	A	NA
PMS3127	Os_Chr05	28404584	28404604	(GAC)7	(GAC)7	(GAC)7	(GAC)7	(GAC)7	(GAC)7	(GAC)6	(GAC)7	(GAC)7	(GAC)5	(GAC)7	LOC_Os05g49540	CDS	retrotransposon protein, putative, Ty1-copia subclass, expressed	CODON_DELETION	GGATATCCATGACCAACACAGTCC	CCTCTCTGTAGCAAAGGGTGTGG	252	A	A	A	RM19136
PMS3128	Os_Chr05	28436309	28436322	(CT)7	(CT)7	(CT)7	(CT)7	(CT)7	(CT)7	(CT)7	(CT)7	(CT)7	(CT)7	(CT)6	-	Introns/Intergenic	-	-	GTCTTCTTTTGGCTTTGGGC	TCCCCTCAATCAATTTCACC	240	A	A	A	NA
PMS3129	Os_Chr05	28472410	28472430	(GCC)7	(GCC)6	(GCC)7	(GCC)7	(GCC)7	(GCC)6	(GCC)7	(GCC)7	(GCC)6	(GCC)7	(GCC)7	-	Introns/Intergenic	-	-	CTGAGCTGGAGGAAGTACGG	CGGTGAGTGCACCTGAAGTA	205	P	A	A	NA
PMS3130	Os_Chr05	28479134	28479151	(CGT)6	(CGT)6	(CGT)6	(CGT)6	(CGT)6	(CGT)6	(CGT)6	(CGT)5	(CGT)6	(CGT)6	(CGT)6	LOC_Os05g49640	CDS	expressed protein	CODON_DELETION	GACTGCAGTTTCAGCTTCCC	AACAAGTGGAGCTCCTCAGC	205	A	A	A	NA
PMS3131	Os_Chr05	28547558	28547581	(TCT)8	(TCT)7	(TCT)8	(TCT)8	(TCT)8	(TCT)8	(TCT)8	(TCT)8	(TCT)8	(TCT)8	(TCT)8	LOC_Os05g49760	URR	dehydrogenase, putative, expressed	-	TTGATTGATGAACCAGCAGTGG	GGAGATTGGATTGGTAGTCTTGAGG	186	P	A	A	RM19140
PMS3132	Os_Chr05	28572675	28572692	(CGG)6	(CGG)5	(CGG)6	(CGG)6	(CGG)6	(CGG)6	(CGG)6	(CGG)6	(CGG)6	(CGG)6	(CGG)6	LOC_Os05g49780	CDS	OsHAP3C	CODON_CHANGE_PLUS_CODON_DELETION	CTCCTTCTCACCGACCTCTG	GCTGGTGATGAAGCTGATGA	234	P	A	P	NA

**Polymorphic SSR (PMS) markers**	**Chromosomes**	**Start physical positions (bp)**	**End physical positions (bp)**	**Nipponbare**	**Kasalath**	**Nagina22**	**IR64**	**Pokkali**	**Bala**	**Tainung67**	**Azucena**	**Moroberekan**	***Oryza rufipogon***	***Oryza nivara***	**MSU gene locus IDs**	**Structural annotation**	**Functional annotation**	**SSR functional effects**	**Forward primers (5'-3')**	**Reverse primers (5'-3')**	**Amplified product size (bp)**	**Markers exhibiting polymorphism within *indica* rice**	**Transcrption factor genes-derived markers**	**Known cloned genes-derived markers**	**PMS markers corresponding with RM markers**
PMS3133	Os_Chr05	28654637	28654668	(AAGA)8	(AAGA)8	(AAGA)8	(AAGA)8	(AAGA)6	(AAGA)8	(AAGA)8	(AAGA)8	(AAGA)8	(AAGA)8	(AAGA)8	-	Introns/Intergenic	-	-	TTTACAAGTTTCGCACGGAAGG	GGAGATAAGATCCTCGGCTAGGG	164	P	A	A	RM19144
PMS3134	Os_Chr05	28673431	28673470	(CT)20	(CT)20	(CT)20	(CT)20	(CT)20	(CT)20	(CT)20	(CT)20	(CT)19	(CT)20	(CT)20	-	Introns/Intergenic	-	-	CGCTCCTCCACTCTTCTCCTACC	CGTGCAGAAAGTCCATTACTCTCC	113	A	A	A	RM31
PMS3135	Os_Chr05	28730360	28730433	(TA)37	(TA)37	(TA)37	(TA)10	(TA)37	(TA)37	(TA)37	(TA)37	(TA)37	(TA)37	(TA)6	-	Introns/Intergenic	-	-	CCTCCGGTTCCATAATTTGA	TTCTTAACGTTTTAGACAATAATACGG	263	P	A	A	NA
PMS3136	Os_Chr05	28771722	28771733	(AG)6	(AG)7	(AG)7	(AG)7	(AG)7	(AG)6	(AG)6	(AG)6	(AG)6	(AG)6	(AG)6	-	Introns/Intergenic	-	-	TGGCTTGTATTGCACTCCTG	CCTCGAAGAGGCCCCTAATA	172	P	A	A	NA
PMS3137	Os_Chr05	28815577	28815597	(CGT)7	(CGT)6	(CGT)7	(CGT)7	(CGT)7	(CGT)6	(CGT)7	(CGT)7	(CGT)7	(CGT)7	(CGT)7	-	Introns/Intergenic	-	-	CCCTATACTCGCTCATCACAAGTGG	ATCAACAACACACCACACCATCG	149	P	A	A	RM19151
PMS3138	Os_Chr05	28836610	28836627	(CAG)6	(CAG)6	(CAG)6	(CAG)6	(CAG)6	(CAG)6	(CAG)5	(CAG)6	(CAG)6	(CAG)6	(CAG)6	LOC_Os05g50310	CDS	ZF-HD protein dimerisation region containing protein, expressed	CODON_DELETION	CAACAACAAGAGCAGCATCG	AGGTGGGGATTTACCAAACC	143	A	A	A	NA
PMS3139	Os_Chr05	28844785	28844814	(AG)15	(AG)15	(AG)15	(AG)15	(AG)15	(AG)14	(AG)15	(AG)15	(AG)15	(AG)15	(AG)15	-	Introns/Intergenic	-	-	GTACTGGGTGGGTCGGATAC	AACCGTAGCCGAGGACATAA	218	P	A	A	NA
PMS3140	Os_Chr05	28845136	28845151	(AT)8	(AT)8	(AT)7	(AT)8	(AT)7	(AT)8	(AT)8	(AT)8	(AT)8	(AT)8	(AT)8	-	Introns/Intergenic	-	-	TCTGCCAGCAATCCAGAATA	TCGATCTCTCAACCATGTGC	227	P	A	A	NA
PMS3141	Os_Chr05	28850055	28850072	(GGA)6	(GGA)6	(GGA)6	(GGA)6	(GGA)6	(GGA)6	(GGA)5	(GGA)6	(GGA)6	(GGA)6	(GGA)6	LOC_Os05g50350	CDS	MYB family transcription factor, putative, expressed	CODON_DELETION	GCGCGGTACTGATGAGAGAT	GCGGTGTACGACAAGGAGAC	231	A	A	A	NA
PMS3142	Os_Chr05	28871695	28871712	(CCG)6	(CCG)6	(CCG)6	(CCG)6	(CCG)6	(CCG)6	(CCG)7	(CCG)7	(CCG)7	(CCG)7	(CCG)6	-	Introns/Intergenic	-	-	GTTGAGGCCCGTCTAAATGA	CCATTTTCTGTTTCGCTGCT	206	A	A	A	NA
PMS3143	Os_Chr05	28997541	28997556	(AT)8	(AT)8	(AT)8	(AT)8	(AT)8	(AT)8	(AT)8	(AT)8	(AT)8	(AT)8	(AT)7	LOC_Os05g50570	DRR	OsSCP29 - Putative Serine Carboxypeptidase homologue, expressed	-	GAAGGCGTAAGGCAAAATGT	GCACAATACGGTGTGTACGG	238	A	A	A	NA
PMS3144	Os_Chr05	29035615	29035632	(GGC)6	(GGC)6	(GGC)5	(GGC)6	(GGC)5	(GGC)6	(GGC)5	(GGC)5	(GGC)6	(GGC)6	(GGC)6	LOC_Os05g50654	CDS	mitochondrial import receptor subunit TOM7-1, putative, expressed	CODON_DELETION	CTCTGAGAGCGAGGACGAAG	AAGAAAAGCGGAGGCAGAGT	277	P	A	A	NA
PMS3145	Os_Chr05	29103570	29103597	(AT)14	(AT)11	(AT)14	(AT)14	(AT)14	(AT)14	(AT)14	(AT)14	(AT)14	(AT)14	(AT)14	-	Introns/Intergenic	-	-	CAAACATTCCCCTTGACTCAT	AGAAAAGTCAGTTCTACCCTTCAA	278	P	A	A	NA

Polymorphic SSR (PMS) markers	Chromosomes	Start physical positions (bp)	End physical positions (bp)	Nipponbare	Kasalath	Nagina22	IR64	Pokkali	Bala	Tainung67	Azucena	Moroberekan	*Oryza rufipogon*	*Oryza nivara*	MSU gene locus IDs	Structural annotation	Functional annotation	SSR functional effects	Forward primers (5'-3')	Reverse primers (5'-3')	Amplified product size (bp)	Markers exhibiting polymorphism within *indica* rice	Transcrption factor genes-derived markers	Known cloned genes-derived markers	PMS markers corresponding with RM markers
PMS3146	Os_Chr05	29114167	29114190	(AT)12	(AT)12	(AT)12	(AT)12	(AT)12	(AT)11	(AT)12	(AT)12	(AT)12	(AT)12	(AT)12	-	Introns/Intergenic	-	-	CATGAAGCACCAAACAAAT	TTTAGAACAATGTGGAATCTGA	392	P	A	A	NA
PMS3147	Os_Chr05	29117706	29117731	(AG)13	(AG)9	(AG)13	(AG)13	(AG)13	(AG)13	(AG)13	(AG)13	(AG)13	(AG)13	(AG)13	-	Introns/Intergenic	-	-	AGACGAACGGTCAAACATGTGC	GTGTACCCGGCCTCAAATTAAGC	180	P	A	A	RM19177
PMS3148	Os_Chr05	29161571	29161594	(GCG)8	(GCG)8	(GCG)8	(GCG)8	(GCG)8	(GCG)8	(GCG)8	(GCG)8	(GCG)8	(GCG)6	(GCG)8	LOC_Os05g50840	URR	mitochondrial carrier protein, putative, expressed	-	TTCAACACGGAGACGATGAGACG	GTTAATTAGCAACCAGTCGCCTCTCC	434	A	A	A	RM19178
PMS3149	Os_Chr05	29312270	29312287	(CAC)6	(CAC)6	(CAC)6	(CAC)5	(CAC)5	(CAC)5	(CAC)6	(CAC)5	(CAC)6	(CAC)6	(CAC)6	LOC_Os05g51080	CDS	expressed protein	CODON_DELETION	TCGCCTACACTAGTGACCACC	GAAGTCCAGTGGCTGGCA	239	P	A	A	NA
PMS3150	Os_Chr05	29396275	29396292	(GCG)6	(GCG)6	(GCG)6	(GCG)6	(GCG)6	(GCG)6	(GCG)6	(GCG)6	(GCG)6	(GCG)6	(GCG)5	LOC_Os05g51250	CDS	transposon protein, putative, CACTA, En/Spm sub-class, expressed	CODON_DELETION	GTTCTCGTTCTCGTTCGTTA	TCCGATCTCGATCTCTCTC	335	A	A	A	NA
PMS3151	Os_Chr05	29446707	29446806	(TA)50	(TA)50	(TA)6	(TA)50	(TA)50	(TA)50	(TA)50	(TA)50	(TA)50	(TA)12	(TA)50	-	Introns/Intergenic	-	-	TCTTGCAACCACCATAAGAGA	GGATATTGGCCCTTTTGAGA	280	P	A	A	NA
PMS3152	Os_Chr05	29447116	29447147	(TA)16	(TA)16	(TA)16	(TA)16	(TA)16	(TA)16	(TA)16	(TA)16	(TA)16	(TA)13	(TA)12	-	Introns/Intergenic	-	-	GAAGCATCTCCCAATGGATTTACC	CCATAGCGGTACCTATGTGCTTACC	485	A	A	A	RM19196
PMS3153	Os_Chr05	29491281	29491296	(CT)8	(CT)8	(CT)8	(CT)8	(CT)8	(CT)8	(CT)8	(CT)7	(CT)8	(CT)8	(CT)8	-	Introns/Intergenic	-	-	AAAAATCGGTGCAAATCGAC	ATTGCCGTCTCTGAGCCTAA	268	A	A	A	NA
PMS3154	Os_Chr05	29543654	29543679	(AG)13	(AG)17	(AG)13	(AG)13	(AG)13	(AG)13	(AG)13	(AG)13	(AG)13	(AG)13	(AG)13	-	Introns/Intergenic	-	-	ACATCAGCACATAGATGGAAGGAAGG	GACGAGTGGTCAAACAATCCAAGC	280	P	A	A	RM3286
PMS3155	Os_Chr05	29589191	29589206	(AC)8	(AC)8	(AC)6	(AC)6	(AC)6	(AC)6	(AC)6	(AC)8	(AC)8	(AC)6	(AC)6	-	Introns/Intergenic	-	-	CTGCGGTCTGCCTGTTAGTA	GGCATTTGATAGTGTGGTCG	138	P	A	A	NA
PMS3156	Os_Chr05	29600885	29600902	(CGC)6	(CGC)6	(CGC)5	(CGC)6	(CGC)6	(CGC)6	(CGC)6	(CGC)6	(CGC)6	(CGC)6	(CGC)6	LOC_Os05g51620	CDS	expressed protein	CODON_DELETION	GAGAAGCTGACGGAGACCAC	AGGATGGTGTGGAAGAGCTG	110	P	A	A	NA
PMS3157	Os_Chr05	29711230	29711243	(GA)7	(GA)7	(GA)7	(GA)7	(GA)7	(GA)7	(GA)7	(GA)7	(GA)7	(GA)6	(GA)7	-	Introns/Intergenic	-	-	CTATTTGGCCGACGATGTG	GTCGTCCCCTACCTCTAGCC	233	A	A	A	NA
PMS3158	Os_Chr05	29719220	29719234	(TCG)5	(TCG)5	(TCG)5	(TCG)5	(TCG)5	(TCG)5	(TCG)5	(TCG)5	(TCG)5	(TCG)6	(TCG)5	LOC_Os05g51780	CDS	zinc finger, C3HC4 type domain containing protein, expressed	CODON_INSERTION	GAGGTGGAGATGGAGATGGA	GCTGTACGACGAGCGATG	263	A	A	A	NA

Polymorphic SSR (PMS) markers	Chromosomes	Start physical positions (bp)	End physical positions (bp)	Nipponbare	Kasalath	Nagina22	IR64	Pokkali	Bala	Tainung67	Azucena	Moroberekan	*Oryza rufipogon*	*Oryza nivara*	MSU gene locus IDs	Structural annotation	Functional annotation	SSR functional effects	Forward primers (5'-3')	Reverse primers (5'-3')	Amplified product size (bp)	Markers exhibiting polymorphism within *indica* rice	Transcrption factor genes-derived markers	Known cloned genes-derived markers	PMS markers corresponding with RM markers
PMS3159	Os_Chr05	29749553	29749588	(TAC)12	(TAC)11	(TAC)11	(TAC)11	(TAC)11	(TAC)11	(TAC)12	(TAC)11	(TAC)11	(TAC)11	(TAC)11	-	Introns/Intergenic	-	-	GCATCCACGCAGATAGACCT	CTTGTCTTGGCTTGGCTAGG	155	A	A	A	NA
PMS3160	Os_Chr05	29792799	29792864	(AT)33	(AT)33	(AT)33	(AT)6	(AT)11	(AT)33	(AT)33	(AT)33	(AT)33	(AT)24	(AT)33	-	Introns/Intergenic	-	-	ACCCTCAAAGCCTCCAAAAT	CACTTTTGGGGCCTATTTCC	266	P	A	A	NA
PMS3161	Os_Chr06	131434	131466	(CTC)11	(CTC)11	(CTC)11	(CTC)11	(CTC)11	(CTC)11	(CTC)5	(CTC)11	(CTC)11	(CTC)7	(CTC)6	LOC_Os06g01170	CDS	PHD-finger domain containing protein, expressed	CODON_DELETION	CTTCGCACTCCAGTCGCTCTCC	GTTGAGGAGGTGTATGGGCTTGG	241	A	P	A	RM6273
PMS3162	Os_Chr06	131830	131847	(TGC)6	(TGC)6	(TGC)6	(TGC)6	(TGC)6	(TGC)6	(TGC)6	(TGC)6	(TGC)6	(TGC)6	(TGC)5	LOC_Os06g01170	CDS	PHD-finger domain containing protein, expressed	CODON_DELETION	GCTCAATGTTTCTGCTGCTG	AGCATTGAGCTCCGTTGATT	157	A	P	A	NA
PMS3163	Os_Chr06	185928	185945	(CCT)6	(CCT)6	(CCT)6	(CCT)6	(CCT)6	(CCT)6	(CCT)6	(CCT)6	(CCT)6	(CCT)5	(CCT)6	LOC_Os06g01304	CDS	spotted leaf 11, putative, expressed	CODON_CHANGE_PLUS_CODON_DELETION	GATCGGAAAAAGGGGAAATC	ACCTGGTCGATCTTACTGCC	253	A	A	A	NA
PMS3164	Os_Chr06	210054	210077	(TCC)8	(TCC)8	(TCC)8	(TCC)8	(TCC)8	(TCC)5	(TCC)8	(TCC)8	(TCC)8	(TCC)8	(TCC)8	LOC_Os06g01340	CDS	OsCCT19	CODON_DELETION	AATTGATGCAGGTTCAGCAAGC	GGAAATGTGGTTGAGAGTTGAGAGC	170	P	A	P	RM6775
PMS3165	Os_Chr06	217356	217403	(ATAC)12	(ATAC)12	(ATAC)12	(ATAC)12	(ATAC)12	(ATAC)12	(ATAC)12	(ATAC)8	(ATAC)12	(ATAC)12	(ATAC)6	-	Introns/Intergenic	-	-	CATGGACTTGTTAGCAGCGTACC	ATTTCATCGATGGCCTCTACACC	168	A	A	A	RM7158
PMS3166	Os_Chr06	294003	294034	(AG)16	(AG)16	(AG)16	(AG)16	(AG)16	(AG)16	(AG)14	(AG)16	(AG)16	(AG)16	(AG)16	LOC_Os06g01480	URR	OsSWN7	-	GACTGGGAGGATGAAAGAGATGG	AGGTCAGTCGCTGTTCTCTTACG	157	A	P	P	RM19238
PMS3167	Os_Chr06	348371	348391	(CTT)7	(CTT)6	(CTT)7	(CTT)7	(CTT)6	(CTT)6	(CTT)7	(CTT)7	(CTT)7	(CTT)7	(CTT)7	LOC_Os06g01590	URR	lactate/malate dehydrogenase, putative, expressed	-	CGCCTTCTTCATCGATTTATCC	CCAACATTCGGTCTTCACTACTAGC	157	P	A	A	RM19239
PMS3168	Os_Chr06	468379	468402	(GGC)8	(GGC)8	(GGC)6	(GGC)8	(GGC)8	(GGC)8	(GGC)8	(GGC)8	(GGC)8	(GGC)8	(GGC)8	LOC_Os06g01810	CDS	expressed protein	CODON_DELETION	GGAACAACCAGGACTGAGAAGG	CTCGTCACTTGTCAGATCCAACG	111	P	A	A	RM19248
PMS3169	Os_Chr06	481131	481155	(GGATT)5	(GGATT)5	(GGATT)5	(GGATT)6	(GGATT)5	(GGATT)5	(GGATT)5	(GGATT)5	(GGATT)5	(GGATT)5	(GGATT)5	-	Introns/Intergenic	-	-	GTAGGGGATCTCACCTGCAA	ATCCAAGAAGCATGACGAGG	233	P	A	A	NA
PMS3170	Os_Chr06	536368	536383	(CT)8	(CT)8	(CT)8	(CT)8	(CT)8	(CT)8	(CT)8	(CT)8	(CT)8	(CT)6	(CT)8	-	Introns/Intergenic	-	-	TGGCCGATCTGTTCATTGTA	ACACCGCGAATGGTTAGTTC	228	A	A	A	NA
PMS3171	Os_Chr06	565233	565262	(CT)15	(CT)15	(CT)16	(CT)15	(CT)15	(CT)16	(CT)15	(CT)15	(CT)15	(CT)16	(CT)15	-	Introns/Intergenic	-	-	TTACGTGATCACACAGGCTCTCC	AAGCTGAACAAGCCCTGAAAGG	168	P	A	A	RM469

Polymorphic SSR (PMS) markers	Chromosomes	Start physical positions (bp)	End physical positions (bp)	Nipponbare	Kasalath	Nagina22	IR64	Pokkali	Bala	Tainung67	Azucena	Moroberekan	*Oryza rufipogon*	*Oryza nivara*	MSU gene locus IDs	Structural annotation	Functional annotation	SSR functional effects	Forward primers (5'-3')	Reverse primers (5'-3')	Amplified product size (bp)	Markers exhibiting polymorphism within *indica* rice	Transcrption factor genes-derived markers	Known cloned genes-derived markers	PMS markers corresponding with RM markers
PMS3172	Os_Chr06	579869	579892	(AT)12	(AT)12	(AT)12	(AT)12	(AT)12	(AT)12	(AT)12	(AT)12	(AT)12	(AT)12	(AT)8	-	Introns/Intergenic	-	-	CGCATGCCAATTGATCCTATCC	GCACGGTCTATTTCATCCAACTCG	542	A	A	A	RM19261
PMS3173	Os_Chr06	719053	719118	(AT)33	(AT)33	(AT)33	(AT)33	(AT)33	(AT)33	(AT)6	(AT)6	(AT)33	(AT)33	(AT)33	-	Introns/Intergenic	-	-	TGTGGTCGCTAGCTTCCTTT	GAACGGCCGGCTTAATTACT	272	A	A	A	NA
PMS3174	Os_Chr06	783146	783163	(TCT)6	(TCT)5	(TCT)5	(TCT)5	(TCT)6	(TCT)5	(TCT)6	(TCT)6	(TCT)6	(TCT)6	(TCT)5	LOC_Os06g02350	CDS	S-phase kinase-associated protein 1A, putative, expressed	CODON_DELETION	CTCGATGATCTTGGAGAGGG	AATCGGTTGCTAGCTTGCTT	211	P	A	A	NA
PMS3175	Os_Chr06	891174	891188	(CAG)5	(CAG)5	(CAG)5	(CAG)5	(CAG)5	(CAG)5	(CAG)5	(CAG)5	(CAG)5	(CAG)6	(CAG)5	LOC_Os06g02550	CDS	CPuORF21 - conserved peptide uORF-containing transcript, expressed	CODON_CHANGE_PLUS_CODON_INSERTION	ATGAATCAGCCTTGGTCCAG	TTATTAGTGCCCGAACACCC	169	A	A	A	NA
PMS3176	Os_Chr06	1010277	1010294	(GCG)6	(GCG)6	(GCG)6	(GCG)6	(GCG)6	(GCG)6	(GCG)6	(GCG)6	(GCG)5	(GCG)6	(GCG)6	LOC_Os06g02770	CDS	expressed protein	CODON_CHANGE_PLUS_CODON_DELETION	GTGGTGGCGGTAGCAAGG	GTGGACGTTGCCGATACTG	144	A	A	A	NA
PMS3177	Os_Chr06	1029540	1029611	(TTA)24	(TTA)24	(TTA)24	(TTA)24	(TTA)24	(TTA)15	(TTA)6	(TTA)8	(TTA)24	(TTA)24	(TTA)18	LOC_Os06g02810	DRR	expressed protein	-	GGCTGACGTACTAGGACATGATGG	TAGCAAATGCCAATGCACAAGC	488	P	A	A	RM19276
PMS3178	Os_Chr06	1087330	1087389	(TATG)15	(TATG)15	(TATG)16	(TATG)15	(TATG)15	(TATG)16	(TATG)15	(TATG)15	(TATG)15	(TATG)15	(TATG)15	-	Introns/Intergenic	-	-	TGATGTCCCACACTTTTACTGC	CAACTTATAATATGAAACGGATGGA	244	P	A	A	NA
PMS3179	Os_Chr06	1094677	1094712	(AG)18	(AG)18	(AG)18	(AG)18	(AG)18	(AG)18	(AG)18	(AG)19	(AG)18	(AG)18	(AG)18	-	Introns/Intergenic	-	-	TAATCAAGCAGCACAGCATCAGG	TCTGAACTTCCACTCGAGATTGC	243	A	A	A	RM19285
PMS3180	Os_Chr06	1174478	1174501	(GCG)8	(GCG)6	(GCG)8	(GCG)8	(GCG)8	(GCG)8	(GCG)8	(GCG)8	(GCG)8	(GCG)8	(GCG)8	LOC_Os06g03150	CDS	expressed protein	CODON_CHANGE_PLUS_CODON_DELETION	GCCAAGTGAAGCAATCCATC	CACCAGGTAGGCCATGTACC	266	P	A	A	NA
PMS3181	Os_Chr06	1219445	1219532	(TA)44	(TA)44	(TA)7	(TA)6	(TA)44	(TA)44	(TA)44	(TA)44	(TA)44	(TA)7	(TA)6	-	Introns/Intergenic	-	-	TAGCTTGCGGTTAGGGAGACG	GGAGGAGATTCAGCTCTCCATCG	609	P	A	A	RM19292
PMS3182	Os_Chr06	1228723	1228736	(CG)7	(CG)7	(CG)7	(CG)7	(CG)7	(CG)7	(CG)7	(CG)7	(CG)7	(CG)7	(CG)6	-	Introns/Intergenic	-	-	GCCCAAAATCCCTTCTCTCT	AGGAGATCCCGGAGATTGTT	230	A	A	A	NA
PMS3183	Os_Chr06	1263228	1263245	(CT)9	(CT)9	(CT)9	(CT)9	(CT)9	(CT)9	(CT)9	(CT)9	(CT)9	(CT)9	(CT)6	-	Introns/Intergenic	-	-	AAAAGAAGTCTGTTTCCCCC	GAGGAAGGCGATGACAGGT	176	A	A	A	NA
PMS3184	Os_Chr06	1270196	1270219	(TA)12	(TA)12	(TA)12	(TA)12	(TA)7	(TA)12	(TA)7	(TA)12	(TA)12	(TA)12	(TA)6	-	Introns/Intergenic	-	-	TTTACCCGTTACAACGCACA	CAATTTGAATCATGGAGCCC	106	P	A	A	NA

Polymorphic SSR (PMS) markers	Chromosomes	Start physical positions (bp)	End physical positions (bp)	Nipponbare	Kasalath	Nagina22	IR64	Pokkali	Bala	Tainung67	Azucena	Moroberekan	*Oryza rufipogon*	*Oryza nivara*	MSU gene locus IDs	Structural annotation	Functional annotation	SSR functional effects	Forward primers (5'-3')	Reverse primers (5'-3')	Amplified product size (bp)	Markers exhibiting polymorphism within *indica* rice	Transcrption factor genes-derived markers	Known cloned genes-derived markers	PMS markers corresponding with RM markers
PMS3185	Os_Chr06	1287044	1287059	(CG)8	(CG)8	(CG)7	(CG)8	(CG)8	(CG)8	(CG)8	(CG)8	(CG)8	(CG)8	(CG)8	-	Introns/Intergenic	-	-	CCCTGTTGACCAGTTGAGGT	GGTTAGTGGCTGCAAAAGGA	125	P	A	A	NA
PMS3186	Os_Chr06	1354334	1354354	(TCC)7	(TCC)7	(TCC)7	(TCC)7	(TCC)7	(TCC)7	(TCC)6	(TCC)7	(TCC)7	(TCC)7	(TCC)7	-	Introns/Intergenic	-	-	CCTACCGCGTCATTCACATGC	GACAAGATCGACAGCCGCTACG	178	A	A	A	RM19300
PMS3187	Os_Chr06	1383552	1383573	(AG)11	(AG)11	(AG)11	(AG)11	(AG)11	(AG)11	(AG)11	(AG)11	(AG)10	(AG)11	(AG)10	-	Introns/Intergenic	-	-	TCCCTTTCAGTCAAGACAACTACTGG	TCTCCTCTTCTCTGCTTCTCAAGG	157	A	A	A	RM19304
PMS3188	Os_Chr06	1389998	1390012	(TGC)5	(TGC)5	(TGC)6	(TGC)5	(TGC)5	(TGC)6	(TGC)5	(TGC)5	(TGC)5	(TGC)5	(TGC)5	LOC_Os06g03600	CDS	transcriptional corepressor SEUSS, putative, expressed	CODON_INSERTION	TACTTCTGCTGGGGAGGATG	CTCCATCAGTTGCTGCAAGA	250	P	A	A	NA
PMS3189	Os_Chr06	1435674	1435695	(CT)11	(CT)11	(CT)11	(CT)11	(CT)11	(CT)11	(CT)11	(CT)11	(CT)11	(CT)12	(CT)11	LOC_Os06g03670	DRR	dehydration-responsive element-binding protein, putative, expressed	-	AAAATGGCTGAGCTGGTAGG	ACCATAAGCAGAGCTGGCAT	168	A	A	A	NA
PMS3190	Os_Chr06	1448351	1448371	(CCT)7	(CCT)7	(CCT)7	(CCT)5	(CCT)7	(CCT)7	(CCT)7	(CCT)7	(CCT)7	(CCT)7	(CCT)7	-	Introns/Intergenic	-	-	GCTTCTTCGGCCACTGAATCTGG	TGGGTGGGTGCTCGATCTGC	343	P	A	A	RM19310
PMS3191	Os_Chr06	1464444	1464463	(AC)10	(AC)10	(AC)10	(AC)10	(AC)10	(AC)11	(AC)10	(AC)10	(AC)11	(AC)10	(AC)10	-	Introns/Intergenic	-	-	TGCGGTGCTGTTCACCTACTATCG	GCACTGAAGCTGGTGCAATCG	95	P	A	A	RM19311
PMS3192	Os_Chr06	1477904	1477949	(CT)23	(CT)23	(CT)23	(CT)23	(CT)23	(CT)23	(CT)23	(CT)23	(CT)22	(CT)22	(CT)23	-	Introns/Intergenic	-	-	TGCCATCTCATAAACCCACTAACC	CTGAGATACGCCAACGAGATACC	295	A	A	A	RM586
PMS3193	Os_Chr06	1490670	1490684	(TTA)5	(TTA)5	(TTA)5	(TTA)5	(TTA)5	(TTA)7	(TTA)5	(TTA)5	(TTA)5	(TTA)7	(TTA)5	-	Introns/Intergenic	-	-	CTCTGCTTTGCTTGCTGTTG	CTAAGTGCCATTGTTCGTGG	251	P	A	A	NA
PMS3194	Os_Chr06	1522221	1522232	(AG)6	(AG)6	(AG)6	(AG)7	(AG)7	(AG)6	(AG)6	(AG)6	(AG)7	(AG)6	(AG)6	-	Introns/Intergenic	-	-	GATTGGGAATCGATGCAACT	CTAGGCTGTACGATTGGGGA	251	P	A	A	NA
PMS3195	Os_Chr06	1558528	1558539	(GT)6	(GT)6	(GT)6	(GT)7	(GT)7	(GT)6	(GT)6	(GT)6	(GT)6	(GT)7	(GT)6	-	Introns/Intergenic	-	-	CAGTCCTCATTATTGGCCGT	GTTGCAACCAACGATGTCAC	255	P	A	A	NA
PMS3196	Os_Chr06	1614715	1614728	(AC)7	(AC)7	(AC)7	(AC)7	(AC)7	(AC)6	(AC)7	(AC)7	(AC)7	(AC)7	(AC)7	-	Introns/Intergenic	-	-	GCCTGCTTACCCTTCCTACC	AATGCCAAAAGGAGCACAAC	147	P	A	A	NA
PMS3197	Os_Chr06	1665729	1665748	(TA)10	(TA)10	(TA)10	(TA)10	(TA)10	(TA)8	(TA)10	(TA)10	(TA)10	(TA)10	(TA)10	-	Introns/Intergenic	-	-	TAGGGTAGAGAAATGGCTATGG	AAAGATGGTGGAGTCTAGTCG	187	P	A	A	RM19323

Polymorphic SSR (PMS) markers	Chromosomes	Start physical positions (bp)	End physical positions (bp)	Nipponbare	Kasalath	Nagina22	IR64	Pokkali	Bala	Tainung67	Azucena	Moroberekan	*Oryza rufipogon*	*Oryza nivara*	MSU gene locus IDs	Structural annotation	Functional annotation	SSR functional effects	Forward primers (5'-3')	Reverse primers (5'-3')	Amplified product size (bp)	Markers exhibiting polymorphism within *indica* rice	Transcrption factor genes-derived markers	Known cloned genes-derived markers	PMS markers corresponding with RM markers
PMS3198	Os_Chr06	1679336	1679359	(AT)12	(AT)12	(AT)12	(AT)6	(AT)12	(AT)12	(AT)12	(AT)12	(AT)12	(AT)12	(AT)12	-	Introns/Intergenic	-	-	CCACAAGTGAATATGGATACCG	GGAGTATTTCCTCGTCAATTCG	461	P	A	A	RM19326
PMS3199	Os_Chr06	1697983	1698028	(TA)23	(TA)20	(TA)23	(TA)17	(TA)11	(TA)23	(TA)23	(TA)14	(TA)23	(TA)23	(TA)8	-	Introns/Intergenic	-	-	CTGCTGCTGCTGGTTTCTGTCC	CAGCTTGGCCATTGCACTTCG	308	P	A	A	RM19328
PMS3200	Os_Chr06	1761373	1761388	(CT)8	(CT)8	(CT)8	(CT)8	(CT)7	(CT)8	(CT)8	(CT)8	(CT)8	(CT)8	(CT)8	-	Introns/Intergenic	-	-	ACAAAAACATCCTCGAACCG	CCCAGTTGCCCAAAGAAGAT	231	P	A	A	NA
PMS3201	Os_Chr06	1763214	1763225	(CT)6	(CT)6	(CT)6	(CT)6	(CT)7	(CT)6	(CT)6	(CT)6	(CT)6	(CT)6	(CT)6	-	Introns/Intergenic	-	-	TCGATCTGACGTGGTGCTTA	TCGTAGTCGACGTTGGACAG	263	P	A	A	NA
PMS3202	Os_Chr06	1777002	1777019	(CGG)6	(CGG)6	(CGG)6	(CGG)6	(CGG)6	(CGG)6	(CGG)5	(CGG)6	(CGG)6	(CGG)5	(CGG)6	LOC_Os06g04210	CDS	expressed protein	CODON_CHANGE_PLUS_CODON_DELETION	GATCATCGCCATGTCCGT	CGAGATGGTCTCGAAGCAGT	280	A	A	A	NA
PMS3203	Os_Chr06	1962659	1962676	(GCG)6	(GCG)5	(GCG)6	(GCG)6	(GCG)6	(GCG)5	(GCG)6	(GCG)6	(GCG)6	(GCG)6	(GCG)6	LOC_Os06g04540	CDS	DNA binding protein, putative, expressed	CODON_DELETION	CTGCTGGTGTGGTGGTAATG	GGCCTCACCGTCTACGTCT	222	P	P	A	NA
PMS3204	Os_Chr06	1963449	1963466	(GTG)6	(GTG)6	(GTG)6	(GTG)6	(GTG)6	(GTG)6	(GTG)6	(GTG)6	(GTG)6	(GTG)5	(GTG)6	LOC_Os06g04540	CDS	DNA binding protein, putative, expressed	CODON_DELETION	CCTCCTGAAACTGCTGGAAC	CCCTTCTCCTCTCCTCTCGT	256	A	P	A	NA
PMS3205	Os_Chr06	1968190	1968213	(AT)12	(AT)12	(AT)12	(AT)12	(AT)12	(AT)12	(AT)10	(AT)12	(AT)12	(AT)12	(AT)12	-	Introns/Intergenic	-	-	CCAGCTGCTTGTGATCAAGTCG	TGCTAGTGCATGCCAATACTACTGC	197	A	A	A	RM19350
PMS3206	Os_Chr06	2041118	2041144	(GGA)9	(GGA)9	(GGA)9	(GGA)9	(GGA)9	(GGA)9	(GGA)9	(GGA)9	(GGA)9	(GGA)9	(GGA)7	LOC_Os06g04680	CDS	expressed protein	CODON_DELETION	GTAGGACATGAAGGCGGCTAGG	ATCTCCTGCCACTGCACACC	384	A	A	A	RM6263
PMS3207	Os_Chr06	2175518	2175577	(TA)30	(TA)30	(TA)30	(TA)30	(TA)13	(TA)30	(TA)30	(TA)30	(TA)6	(TA)30	(TA)30	-	Introns/Intergenic	-	-	AATGCAGACTAAACCGCCAC	AAAATTCGATTCTAAGTTGCTGC	236	P	A	A	NA
PMS3208	Os_Chr06	2176087	2176107	(TAC)7	(TAC)6	(TAC)7	(TAC)7	(TAC)7	(TAC)7	(TAC)7	(TAC)7	(TAC)7	(TAC)7	(TAC)7	LOC_Os06g04940	DRR	early nodulin 93 ENOD93 protein, putative, expressed	-	TGTTGTAAACGGTTCGATCTCTGC	CCCACCCTGCCTTCTTCAGG	190	P	A	A	RM19357
PMS3209	Os_Chr06	2203942	2203959	(CT)9	(CT)9	(CT)9	(CT)8	(CT)8	(CT)8	(CT)9	(CT)9	(CT)9	(CT)9	(CT)8	-	Introns/Intergenic	-	-	TCAGATCACCATCCTCCTCC	CCCTTTCATTTTGCCGTCTA	162	P	A	A	NA
PMS3210	Os_Chr06	2216296	2216323	(TA)14	(TA)9	(TA)14	(TA)14	(TA)14	(TA)9	(TA)14	(TA)14	(TA)14	(TA)14	(TA)9	-	Introns/Intergenic	-	-	GTTGTATCCGTTCCTTTGACATGC	GTGTGGTTATCGCCGGGTAGC	525	P	A	A	RM19360

Polymorphic SSR (PMS) markers	Chromosomes	Start physical positions (bp)	End physical positions (bp)	Nipponbare	Kasalath	Nagina22	IR64	Pokkali	Bala	Tainung67	Azucena	Moroberekan	*Oryza rufipogon*	*Oryza nivara*	MSU gene locus IDs	Structural annotation	Functional annotation	SSR functional effects	Forward primers (5'-3')	Reverse primers (5'-3')	Amplified product size (bp)	Markers exhibiting polymorphism within *indica* rice	Transcrption factor genes-derived markers	Known cloned genes-derived markers	PMS markers corresponding with RM markers
PMS3211	Os_Chr06	2266907	2266922	(AG)8	(AG)8	(AG)8	(AG)8	(AG)8	(AG)6	(AG)8	(AG)8	(AG)8	(AG)8	(AG)8	-	Introns/Intergenic	-	-	CACACGAAACAGTGTTAACCAAA	ATGCTTGGAATTACACCCCA	253	P	A	A	NA
PMS3212	Os_Chr06	2289893	2289914	(GA)11	(GA)11	(GA)11	(GA)11	(GA)11	(GA)11	(GA)11	(GA)10	(GA)11	(GA)11	(GA)11	-	Introns/Intergenic	-	-	GGGATTAGGGCGACGAGAAGAGG	ACGGCCACGACTCCATCTCTCC	87	A	A	A	RM19363
PMS3213	Os_Chr06	2336080	2336097	(GCC)6	(GCC)5	(GCC)6	(GCC)6	(GCC)6	(GCC)5	(GCC)6	(GCC)6	(GCC)6	(GCC)5	(GCC)6	LOC_Os06g05200	CDS	zinc finger, C3HC4 type domain containing protein, expressed	CODON_DELETION	TACATTGTCGTCGTCGTCGT	AAGCTTACCTCCGCGACAT	200	P	A	A	NA
PMS3214	Os_Chr06	2365994	2366077	(TA)42	(TA)42	(TA)42	(TA)8	(TA)13	(TA)42	(TA)42	(TA)6	(TA)42	(TA)12	(TA)16	-	Introns/Intergenic	-	-	GGCGAAAGCAATTGTTCATT	GGCTGGATAACCCCTGATTT	165	P	A	A	NA
PMS3215	Os_Chr06	2381243	2381260	(CGG)6	(CGG)5	(CGG)6	(CGG)6	(CGG)6	(CGG)6	(CGG)6	(CGG)6	(CGG)6	(CGG)6	(CGG)6	LOC_Os06g05284	CDS	transferase family protein, putative, expressed	CODON_CHANGE_PLUS_CODON_DELETION	CAGTTTATGAGGGCGTGGTC	CGATTCTTCTGCGATGAGTG	227	P	A	A	NA
PMS3216	Os_Chr06	2390681	2390700	(GA)10	(GA)10	(GA)10	(GA)10	(GA)10	(GA)10	(GA)10	(GA)10	(GA)10	(GA)11	(GA)10	-	Introns/Intergenic	-	-	GGCGATTTGGTAGGGGTAGT	GCAACATGTTCCTCCGTCTT	270	A	A	A	NA
PMS3217	Os_Chr06	2392718	2392741	(AT)12	(AT)10	(AT)12	(AT)12	(AT)12	(AT)12	(AT)6	(AT)8	(AT)12	(AT)12	(AT)12	-	Introns/Intergenic	-	-	ACCCTAGAGCGTTGTGGCTA	TCAATTTCAACAATGGTGCC	210	P	A	A	NA
PMS3218	Os_Chr06	2393181	2393196	(TA)8	(TA)6	(TA)8	(TA)8	(TA)8	(TA)8	(TA)8	(TA)8	(TA)8	(TA)8	(TA)8	-	Introns/Intergenic	-	-	ATGGTGCCCCATATGAGTGT	TTGAGGTGAAGAATTCTACGGAA	168	P	A	A	NA
PMS3219	Os_Chr06	2483222	2483239	(GCG)6	(GCG)6	(GCG)6	(GCG)6	(GCG)6	(GCG)6	(GCG)6	(GCG)6	(GCG)6	(GCG)6	(GCG)5	LOC_Os06g05490	CDS	retrotransposon protein, putative, unclassified, expressed	CODON_CHANGE_PLUS_CODON_DELETION	GATGGGGGAGAGGTTAGGAG	TCCACCGTCTTTACCACCTC	244	A	A	A	NA
PMS3220	Os_Chr06	2489102	2489122	(GGC)7	(GGC)7	(GGC)7	(GGC)7	(GGC)7	(GGC)7	(GGC)5	(GGC)7	(GGC)7	(GGC)7	(GGC)7	-	Introns/Intergenic	-	-	CTCGTTCACGTGTTCGTTA	CTGATCTCGATCTCTCTCTCC	334	A	A	A	NA
PMS3221	Os_Chr06	2585949	2585966	(GCG)6	(GCG)6	(GCG)6	(GCG)6	(GCG)6	(GCG)6	(GCG)6	(GCG)6	(GCG)6	(GCG)5	(GCG)6	LOC_Os06g05720	CDS	expressed protein	CODON_DELETION	ATACCAAGGAGACGGTGTCG	AGGACATGCTCTTCTGCGTC	270	A	A	A	NA
PMS3222	Os_Chr06	2662162	2662182	(CGA)7	(CGA)6	(CGA)6	(CGA)7	(CGA)7	(CGA)6	(CGA)7	(CGA)7	(CGA)7	(CGA)7	(CGA)7	LOC_Os06g05820	DRR	OsLonP2 - Putative Lon protease homologue, expressed	-	CATCTTCCACGACGCCATCACC	CGATTACATGGAGAATGGCTCACTCG	94	P	A	A	RM19388
PMS3223	Os_Chr06	2683566	2683580	(CGC)5	(CGC)5	(CGC)5	(CGC)5	(CGC)5	(CGC)5	(CGC)5	(CGC)5	(CGC)5	(CGC)7	(CGC)7	LOC_Os06g05860	CDS	6-phosphofructokinase, putative, expressed	CODON_INSERTION	AATCCCCAGATCGATCCAA	GACCACCTGCACCACCTC	265	A	A	A	NA

Polymorphic SSR (PMS) markers	Chromosomes	Start physical positions (bp)	End physical positions (bp)	Nipponbare	Kasalath	Nagina22	IR64	Pokkali	Bala	Tainung67	Azucena	Moroberekan	*Oryza rufipogon*	*Oryza nivara*	MSU gene locus IDs	Structural annotation	Functional annotation	SSR functional effects	Forward primers (5'-3')	Reverse primers (5'-3')	Amplified product size (bp)	Markers exhibiting polymorphism within *indica* rice	Transcrption factor genes-derived markers	Known cloned genes-derived markers	PMS markers corresponding with RM markers
PMS3224	Os_Chr06	2827915	2827938	(CT)12	(CT)12	(CT)7	(CT)12	(CT)12	(CT)7	(CT)11	(CT)10	(CT)12	(CT)12	(CT)12	-	Introns/Intergenic	-	-	ATATTTTGGGACGGAGGAGG	GAGAGAGAGGAGGGGAGAGC	279	P	A	A	NA
PMS3225	Os_Chr06	2853319	2853332	(TA)7	(TA)6	(TA)7	(TA)7	(TA)7	(TA)7	(TA)7	(TA)7	(TA)7	(TA)7	(TA)6	-	Introns/Intergenic	-	-	TCATATACCAACCGATGCCA	TCCCTTCTGTCACAAATGCTT	247	P	A	A	NA
PMS3226	Os_Chr06	2859633	2859650	(TTA)6	(TTA)5	(TTA)6	(TTA)6	(TTA)6	(TTA)6	(TTA)6	(TTA)6	(TTA)6	(TTA)6	(TTA)6	-	Introns/Intergenic	-	-	TGAAGGGAAGGGTTTTGTTG	AAGACATGAAGGGATGGCAG	218	P	A	A	NA
PMS3227	Os_Chr06	2882807	2882840	(GA)17	(GA)14	(GA)17	(GA)17	(GA)17	(GA)14	(GA)17	(GA)17	(GA)17	(GA)17	(GA)14	-	Introns/Intergenic	-	-	GCAAGATTGGGAATTGGGTA	AAAATAGGGCAATTGTGCAAG	106	P	A	A	NA
PMS3228	Os_Chr06	2893996	2894013	(CGC)6	(CGC)5	(CGC)6	(CGC)6	(CGC)6	(CGC)5	(CGC)6	(CGC)6	(CGC)6	(CGC)6	(CGC)6	LOC_Os06g06250	URR	GDSL-like lipase/acylhydrolase, putative, expressed	-	ATCTTCAGCTTCGGCGACT	CCAAAACAGCAGTGAGCAAA	277	P	A	A	NA
PMS3229	Os_Chr06	2914899	2914997	(ATT)33	(ATT)33	(ATT)10	(ATT)23	(ATT)7	(ATT)33	(ATT)33	(ATT)33	(ATT)8	(ATT)33	(ATT)33	-	Introns/Intergenic	-	-	TGCTGATTGCTCACTACTTCATCC	GCGGGATACCATGGTCTAAAGG	175	P	A	A	RM19410
PMS3230	Os_Chr06	2929153	2929191	(CCT)13	(CCT)13	(CCT)13	(CCT)13	(CCT)13	(CCT)13	(CCT)13	(CCT)9	(CCT)13	(CCT)13	(CCT)7	-	Introns/Intergenic	-	-	TGGGTTGTCATAGGAAAGGG	GCCAAGCAGAATCTGAATCC	231	A	A	A	NA
PMS3231	Os_Chr06	2976186	2976205	(CT)10	(CT)10	(CT)10	(CT)10	(CT)9	(CT)10	(CT)10	(CT)10	(CT)10	(CT)10	(CT)10	-	Introns/Intergenic	-	-	CAGGTACACATGCCTTAGATAGTGG	CGAGTCTCAATTTCCTAGAGTTGG	230	P	A	A	RM19416
PMS3232	Os_Chr06	3011513	3011524	(AT)6	(AT)12	(AT)6	(AT)6	(AT)6	(AT)12	(AT)6	(AT)6	(AT)6	(AT)6	(AT)6	-	Introns/Intergenic	-	-	AAAATGCGCACTGGTTTTTC	CAATTTGACACGGATGTTCG	218	P	A	A	NA
PMS3233	Os_Chr06	3089100	3089113	(TC)7	(TC)6	(TC)7	(TC)7	(TC)7	(TC)7	(TC)7	(TC)7	(TC)7	(TC)7	(TC)7	-	Introns/Intergenic	-	-	ACGGTTTTTCACCGAGTCAC	CAGGACACACCGTACACGTC	164	P	A	A	NA
PMS3234	Os_Chr06	3145510	3145527	(GCC)6	(GCC)6	(GCC)6	(GCC)6	(GCC)5	(GCC)6	(GCC)6	(GCC)6	(GCC)6	(GCC)6	(GCC)6	LOC_Os06g06720	URR	flavonol synthase/flavanone 3-hydroxylase, putative, expressed	-	CTCAAGCTTGGACTGCCACT	ATGAGAGGTCGACGACGG	246	P	A	A	NA
PMS3235	Os_Chr06	3255932	3255949	(CT)9	(CT)9	(CT)9	(CT)9	(CT)9	(CT)9	(CT)8	(CT)9	(CT)9	(CT)9	(CT)9	-	Introns/Intergenic	-	-	ATCCCTCCTACCGATTCACC	TATCGCAGTCACCTGCTTTG	192	A	A	A	NA
PMS3236	Os_Chr06	3279372	3279389	(TA)9	(TA)9	(TA)9	(TA)9	(TA)9	(TA)9	(TA)9	(TA)9	(TA)6	(TA)9	(TA)9	-	Introns/Intergenic	-	-	GCTTGTACGGCGTAATGGTT	TAAATGTCACGGGCTAAGGC	275	A	A	A	NA

Polymorphic SSR (PMS) markers	Chromosomes	Start physical positions (bp)	End physical positions (bp)	Nipponbare	Kasalath	Nagina22	IR64	Pokkali	Bala	Tainung67	Azucena	Moroberekan	*Oryza rufipogon*	*Oryza nivara*	MSU gene locus IDs	Structural annotation	Functional annotation	SSR functional effects	Forward primers (5'-3')	Reverse primers (5'-3')	Amplified product size (bp)	Markers exhibiting polymorphism within *indica* rice	Transcrption factor genes-derived markers	Known cloned genes-derived markers	PMS markers corresponding with RM markers
PMS3237	Os_Chr06	3390223	3390237	(CCG)5	(CCG)6	(CCG)6	(CCG)5	(CCG)6	(CCG)6	(CCG)5	(CCG)6	(CCG)6	(CCG)6	(CCG)6	LOC_Os06g07100	CDS	RING-H2 finger protein, putative, expressed	CODON_INSERTION	TATCACCAGGACGAACACCA	GCAACTTGCAAAACAACCAA	222	P	A	A	NA
PMS3238	Os_Chr06	3440176	3440199	(CGC)8	(CGC)8	(CGC)8	(CGC)6	(CGC)7	(CGC)8	(CGC)8	(CGC)8	(CGC)8	(CGC)8	(CGC)5	LOC_Os06g07190	URR	RNA polymerase Rpb7, N-terminal domain containing protein, expressed	-	CTCAGCTCCTTCTCGCAGAGTCG	CGGCAGCAGCAGTAACCATAAGC	165	P	A	A	RM6490
PMS3239	Os_Chr06	3457174	3457187	(CG)7	(CG)7	(CG)7	(CG)7	(CG)7	(CG)7	(CG)7	(CG)7	(CG)7	(CG)6	(CG)7	-	Introns/Intergenic	-	-	TCTCGTCTCGTCATCTCGTG	TCTCGACCTCATCGTCTGTG	237	A	A	A	NA
PMS3240	Os_Chr06	3595798	3595818	(CCG)7	(CCG)7	(CCG)7	(CCG)7	(CCG)7	(CCG)7	(CCG)7	(CCG)7	(CCG)7	(CCG)5	(CCG)5	LOC_Os06g07474	URR	tetratricopeptide repeat domain containing protein, expressed	-	ACGGCAGCGAAAGGGACTAGC	GTGCTTTACACCCTCCTGTGACG	238	A	A	A	RM19438
PMS3241	Os_Chr06	3606687	3606724	(AT)19	(AT)19	(AT)8	(AT)19	(AT)9	(AT)10	(AT)19	(AT)19	(AT)19	(AT)10	(AT)19	-	Introns/Intergenic	-	-	GCGCATACATCTATCCATTCTCC	ATCTTTCCACGTACCGAGAGAGC	528	P	A	A	RM19440
PMS3242	Os_Chr06	3678814	3678831	(GGC)6	(GGC)5	(GGC)5	(GGC)6	(GGC)5	(GGC)5	(GGC)6	(GGC)6	(GGC)6	(GGC)5	(GGC)5	LOC_Os06g07600	URR	uncharacterized glycosyltransferase, putative, expressed	-	GGAGATCATGAGCAGCCTTC	CACACCACCACTCACCTCAC	216	P	A	A	NA
PMS3243	Os_Chr06	3701772	3701787	(AT)8	(AT)9	(AT)8	(AT)8	(AT)9	(AT)8	(AT)8	(AT)8	(AT)8	(AT)8	(AT)8	-	Introns/Intergenic	-	-	ACGCGTGTTCATCTTTCTCC	TGCCTCAACACAATATCCCA	243	P	A	A	NA
PMS3244	Os_Chr06	3776399	3776416	(GGA)6	(GGA)5	(GGA)6	(GGA)6	(GGA)6	(GGA)6	(GGA)6	(GGA)6	(GGA)6	(GGA)6	(GGA)6	LOC_Os06g07780	URR	vesicle-associated membrane protein, putative, expressed	-	GTAGGAGACGTGGCTGTCG	CTAATTCAACCTCCCCCTCG	241	P	A	A	NA
PMS3245	Os_Chr06	3823064	3823093	(CGC)10	(CGC)10	(CGC)10	(CGC)10	(CGC)9	(CGC)10	(CGC)9	(CGC)10	(CGC)10	(CGC)5	(CGC)10	LOC_Os06g07860	CDS	cystathionine gamma-synthase, putative, expressed	CODON_CHANGE_PLUS_CODON_DELETION	ATCGTTGCGCAAAAACAAAT	CTGAGCTGCCGCTAAGGTT	278	P	A	A	NA
PMS3246	Os_Chr06	3847922	3847939	(GGC)6	(GGC)6	(GGC)6	(GGC)6	(GGC)6	(GGC)6	(GGC)6	(GGC)5	(GGC)6	(GGC)6	(GGC)6	LOC_Os06g07914	DRR	hydroxylase, putative, expressed	-	CGACTTCCTCAAGGTCAAGG	AGTTTAAGCATGCAGGCCAC	132	A	A	A	NA
PMS3247	Os_Chr06	3885978	3885995	(GGC)6	(GGC)6	(GGC)6	(GGC)6	(GGC)6	(GGC)6	(GGC)6	(GGC)5	(GGC)6	(GGC)6	(GGC)6	-	Introns/Intergenic	-	-	CGACTTCCTCAAGGTCAAGG	AGTTTAAGCATGCAGGCCAC	132	A	A	A	NA
PMS3248	Os_Chr06	3946763	3946816	(AGA)18	(AGA)19	(AGA)18	(AGA)18	(AGA)18	(AGA)18	(AGA)18	(AGA)18	(AGA)18	(AGA)19	(AGA)18	-	Introns/Intergenic	-	-	AGCGTACCGATTCACGAAGTTGC	AGGACATGAGCTGCCTGAGATCC	390	P	A	A	RM19454
PMS3249	Os_Chr06	3960975	3961034	(AAT)20	(AAT)20	(AAT)20	(AAT)7	(AAT)8	(AAT)20	(AAT)20	(AAT)20	(AAT)20	(AAT)20	(AAT)20	LOC_Os06g08154	URR	receptor-like protein kinase 5 precursor, putative, expressed	-	GGGCTTGGACTTGGAGTCTTGG	GACCAGGACCGATCCATCTCC	174	P	A	A	RM6734

Polymorphic SSR (PMS) markers	Chromosomes	Start physical positions (bp)	End physical positions (bp)	Nipponbare	Kasalath	Nagina22	IR64	Pokkali	Bala	Tainung67	Azucena	Moroberekan	*Oryza rufipogon*	*Oryza nivara*	MSU gene locus IDs	Structural annotation	Functional annotation	SSR functional effects	Forward primers (5'-3')	Reverse primers (5'-3')	Amplified product size (bp)	Markers exhibiting polymorphism within *indica* rice	Transcrption factor genes-derived markers	Known cloned genes-derived markers	PMS markers corresponding with RM markers
PMS3250	Os_Chr06	3971473	3971538	(TA)33	(TA)33	(TA)33	(TA)33	(TA)33	(TA)33	(TA)9	(TA)33	(TA)6	(TA)33	(TA)33	-	Introns/Intergenic	-	-	CACGGCACGATACATAAACAACAGG	AGGCATGGCCCATAGGTAGTTGG	263	A	A	A	RM19457
PMS3251	Os_Chr06	4008699	4008719	(CGG)7	(CGG)7	(CGG)7	(CGG)7	(CGG)7	(CGG)7	(CGG)7	(CGG)7	(CGG)7	(CGG)7	(CGG)6	LOC_Os06g08280	CDS	protein kinase domain containing protein, expressed	CODON_CHANGE_PLUS_CODON_DELETION	GGGAAGGTGAAGCTGCTGTGC	TCGTACTCCTCCATCATGTTCTCG	284	A	A	A	RM19461
PMS3252	Os_Chr06	4083903	4083918	(GA)8	(GA)8	(GA)8	(GA)8	(GA)8	(GA)8	(GA)8	(GA)8	(GA)8	(GA)6	(GA)8	-	Introns/Intergenic	-	-	CGCTTCCTCTAGGGTTTCCT	TATAAATAGCCACGGGCACC	239	A	A	A	NA
PMS3253	Os_Chr06	4093652	4093675	(GGT)8	(GGT)8	(GGT)8	(GGT)8	(GGT)8	(GGT)8	(GGT)6	(GGT)8	(GGT)8	(GGT)8	(GGT)8	-	Introns/Intergenic	-	-	GTTTTCTCCGCGCAATTTTA	CAGCGTCGCATTAGAACGTA	207	A	A	A	NA
PMS3254	Os_Chr06	4123516	4123527	(GA)6	(GA)6	(GA)6	(GA)6	(GA)7	(GA)6	(GA)6	(GA)7	(GA)6	(GA)7	(GA)6	-	Introns/Intergenic	-	-	ATCTTTTTGGCCATGGATGA	CAAGGTCAATAGTGCAGCCA	236	P	A	A	NA
PMS3255	Os_Chr06	4134460	4134471	(GA)6	(GA)6	(GA)6	(GA)6	(GA)7	(GA)6	(GA)6	(GA)6	(GA)6	(GA)6	(GA)6	-	Introns/Intergenic	-	-	GTCTCCATCATCCCCACCT	ATGTCATACCTCCCCTTCCC	205	P	A	A	NA
PMS3256	Os_Chr06	4210998	4211011	(CG)7	(CG)7	(CG)7	(CG)7	(CG)7	(CG)7	(CG)7	(CG)7	(CG)7	(CG)7	(CG)6	-	Introns/Intergenic	-	-	AGTACGTACGTTGCAACCCC	CGATGATGAGTGGACGAGTG	253	A	A	A	NA
PMS3257	Os_Chr06	4235101	4235124	(AG)12	(AG)14	(AG)12	(AG)12	(AG)12	(AG)12	(AG)12	(AG)12	(AG)12	(AG)12	(AG)12	-	Introns/Intergenic	-	-	TTGTGTTTGGCTCGGGAATTGG	TGTGTTTCTTCGCTTGCCTTGG	114	P	A	A	RM19487
PMS3258	Os_Chr06	4265395	4265408	(GA)7	(GA)7	(GA)7	(GA)7	(GA)7	(GA)7	(GA)6	(GA)7	(GA)7	(GA)7	(GA)7	-	Introns/Intergenic	-	-	TCTGCTTAGCGTCGAAGGTT	CCGTGTCGAAACCTCAAAAT	144	A	A	A	NA
PMS3259	Os_Chr06	4272562	4272645	(TA)42	(TA)42	(TA)42	(TA)42	(TA)9	(TA)12	(TA)42	(TA)42	(TA)42	(TA)42	(TA)9	-	Introns/Intergenic	-	-	TAGGTGGACACCCATCCATTTATTGC	CCTGCCGCTCCTCTCTTCTATCG	448	P	A	A	RM19489
PMS3260	Os_Chr06	4304252	4304297	(AT)23	(AT)23	(AT)16	(AT)23	(AT)23	(AT)23	(AT)8	(AT)23	(AT)23	(AT)23	(AT)23	-	Introns/Intergenic	-	-	ACCCAATATGGTGCAATAGAGACC	CACCTCCACCAACTATTGACAGG	358	P	A	A	RM4608
PMS3261	Os_Chr06	4312673	4312690	(TC)9	(TC)9	(TC)9	(TC)9	(TC)7	(TC)9	(TC)9	(TC)9	(TC)9	(TC)9	(TC)9	-	Introns/Intergenic	-	-	CTTTGTTCCACGTGGTGTTG	GACCCTCTAGGATGAGCACG	280	P	A	A	NA
PMS3262	Os_Chr06	4350990	4351005	(AG)8	(AG)7	(AG)7	(AG)8	(AG)7	(AG)7	(AG)8	(AG)8	(AG)8	(AG)8	(AG)8	-	Introns/Intergenic	-	-	TGCACTCCAGGGTGACTAGA	ATCCCTATTCGTGGAACCAA	224	P	A	A	NA

Polymorphic SSR (PMS) markers	Chromosomes	Start physical positions (bp)	End physical positions (bp)	Nipponbare	Kasalath	Nagina22	IR64	Pokkali	Bala	Tainung67	Azucena	Moroberekan	*Oryza rufipogon*	*Oryza nivara*	MSU gene locus IDs	Structural annotation	Functional annotation	SSR functional effects	Forward primers (5'-3')	Reverse primers (5'-3')	Amplified product size (bp)	Markers exhibiting polymorphism within *indica* rice	Transcrption factor genes-derived markers	Known cloned genes-derived markers	PMS markers corresponding with RM markers
PMS3263	Os_Chr06	4353517	4353550	(TA)17	(TA)12	(TA)17	(TA)17	(TA)17	(TA)13	(TA)17	(TA)17	(TA)17	(TA)10	(TA)17	-	Introns/Intergenic	-	-	ATCTCGGAGAAAGTGGCTTG	AACTTTCTAGCATTGCCCACA	231	P	A	A	NA
PMS3264	Os_Chr06	4406034	4406057	(TCC)8	(TCC)8	(TCC)8	(TCC)8	(TCC)8	(TCC)8	(TCC)8	(TCC)8	(TCC)8	(TCC)8	(TCC)6	-	Introns/Intergenic	-	-	GCTGCTCCACCTTCACCTTCC	CGATGGTGTGTTGTTTGGTTGC	127	A	A	A	RM6773
PMS3265	Os_Chr06	4423675	4423692	(GCC)6	(GCC)5	(GCC)6	(GCC)6	(GCC)6	(GCC)6	(GCC)6	(GCC)6	(GCC)6	(GCC)6	(GCC)6	-	Introns/Intergenic	-	-	AGATGCCGCTGCAATGAG	GAGGGGTGGATGGGTAATTT	175	P	A	A	NA
PMS3266	Os_Chr06	4642266	4642292	(CCG)9	(CCG)9	(CCG)9	(CCG)9	(CCG)9	(CCG)9	(CCG)9	(CCG)9	(CCG)9	(CCG)5	(CCG)9	-	Introns/Intergenic	-	-	CTTCGTCTGGTGGAGGAACAGG	AGGTCGCAGTTCCAGCAAGAGG	252	A	A	A	RM19513
PMS3267	Os_Chr06	4700592	4700609	(TCC)6	(TCC)6	(TCC)6	(TCC)6	(TCC)6	(TCC)6	(TCC)6	(TCC)6	(TCC)6	(TCC)5	(TCC)6	LOC_Os06g09340	CDS	pectinesterase, putative, expressed	CODON_DELETION	CACGTCTGGTTGTGTTCGTC	ATGGACGTCTGTCACCCC	158	A	A	A	NA
PMS3268	Os_Chr06	4715352	4715378	(GGA)9	(GGA)9	(GGA)9	(GGA)9	(GGA)9	(GGA)5	(GGA)6	(GGA)9	(GGA)9	(GGA)8	(GGA)9	LOC_Os06g09370	URR	OsPTF1	-	AGAGTGGGTGTGGAGAACGAAGG	TCATCATCAGCAGCAGTCAGTCC	139	P	P	P	RM19521
PMS3269	Os_Chr06	4874678	4874695	(CGG)6	(CGG)6	(CGG)6	(CGG)6	(CGG)6	(CGG)6	(CGG)6	(CGG)6	(CGG)6	(CGG)6	(CGG)5	LOC_Os06g09570	URR	nucleolar GTP-binding protein 1, putative, expressed	-	CACCCACCCTTCCTTCCTTTCC	AGAAGACACGGAAGACTCACTCACG	179	A	A	A	RM19527
PMS3270	Os_Chr06	4883541	4883561	(GCG)7	(GCG)7	(GCG)7	(GCG)7	(GCG)5	(GCG)7	(GCG)7	(GCG)7	(GCG)7	(GCG)7	(GCG)5	-	Introns/Intergenic	-	-	AAGAGGACACGACGATGGCTAGG	GTTGCCTCCTCTTCTCTGCATCC	117	P	A	A	RM19528
PMS3271	Os_Chr06	4996264	4996277	(AT)7	(AT)7	(AT)7	(AT)7	(AT)8	(AT)7	(AT)7	(AT)7	(AT)7	(AT)7	(AT)6	-	Introns/Intergenic	-	-	GAAAACCTCCACACGGAAAA	ATCACATCCCCTCCTAGGCT	161	P	A	A	NA
PMS3272	Os_Chr06	5048100	5048123	(TA)12	(TA)12	(TA)12	(TA)12	(TA)12	(TA)12	(TA)12	(TA)11	(TA)12	(TA)12	(TA)7	-	Introns/Intergenic	-	-	TTACGGAAATGAATCCCGAG	CAAGAACAGATCATGTTGCCTCT	249	A	A	A	NA
PMS3273	Os_Chr06	5071418	5071432	(GGC)5	(GGC)5	(GGC)5	(GGC)5	(GGC)5	(GGC)5	(GGC)5	(GGC)5	(GGC)5	(GGC)6	(GGC)5	-	Introns/Intergenic	-	-	CCCACCAGCTGCTACTTTGTGC	CGCCAGGAAGTCGAGGATAGG	167	A	A	A	RM19551
PMS3274	Os_Chr06	5163459	5163476	(CCT)6	(CCT)6	(CCT)6	(CCT)5	(CCT)6	(CCT)6	(CCT)6	(CCT)6	(CCT)6	(CCT)6	(CCT)5	-	Introns/Intergenic	-	-	AAGCAAAGTGCGTGGAAACT	ACTTTTATCGGTGGTCGGTG	112	P	A	A	NA
PMS3275	Os_Chr06	5286012	5286029	(GGC)6	(GGC)6	(GGC)6	(GGC)6	(GGC)6	(GGC)6	(GGC)6	(GGC)5	(GGC)6	(GGC)6	(GGC)6	-	Introns/Intergenic	-	-	CTGTACCGCTTCAGCGTCTC	AAGTCGAAAGCGAAAGACCA	274	A	A	A	NA

Polymorphic SSR (PMS) markers	Chromosomes	Start physical positions (bp)	End physical positions (bp)	Nipponbare	Kasalath	Nagina22	IR64	Pokkali	Bala	Tainung67	Azucena	Moroberekan	*Oryza rufipogon*	*Oryza nivara*	MSU gene locus IDs	Structural annotation	Functional annotation	SSR functional effects	Forward primers (5'-3')	Reverse primers (5'-3')	Amplified product size (bp)	Markers exhibiting polymorphism within *indica* rice	Transcrption factor genes-derived markers	Known cloned genes-derived markers	PMS markers corresponding with RM markers
PMS3276	Os_Chr06	5300330	5300350	(CGC)7	(CGC)7	(CGC)7	(CGC)7	(CGC)7	(CGC)7	(CGC)7	(CGC)7	(CGC)7	(CGC)7	(CGC)5	LOC_Os06g10310	CDS	growth regulating factor protein, putative, expressed	CODON_DELETION	TTGTGGTTGTAGTGTGCTTGTGC	CGGAACGAGAGGACAATGTACG	277	A	P	A	RM19559
PMS3277	Os_Chr06	5350578	5350605	(TA)14	(TA)14	(TA)6	(TA)13	(TA)14	(TA)9	(TA)14	(TA)14	(TA)6	(TA)14	(TA)9	-	Introns/Intergenic	-	-	TCGACGAATGATCTGTTCTGA	ATTAGATACCATGGTGCCCG	213	P	A	A	NA
PMS3278	Os_Chr06	5369659	5369676	(CCG)6	(CCG)6	(CCG)6	(CCG)6	(CCG)6	(CCG)6	(CCG)6	(CCG)5	(CCG)6	(CCG)6	(CCG)6	LOC_Os06g10440	URR	gb protein, putative, expressed	-	ATCAAACCGGTTCAATTCCC	GCAGCTTCTCCTCGTACACC	181	A	A	A	NA
PMS3279	Os_Chr06	5433813	5433830	(GA)9	(GA)9	(GA)9	(GA)9	(GA)9	(GA)9	(GA)8	(GA)9	(GA)9	(GA)9	(GA)9	-	Introns/Intergenic	-	-	CCTACCAATTCCTCTCCCGT	GGATCACGTCGTGTCTCTCA	272	A	A	A	NA
PMS3280	Os_Chr06	5550858	5550873	(AG)8	(AG)8	(AG)8	(AG)8	(AG)8	(AG)8	(AG)8	(AG)8	(AG)8	(AG)8	(AG)10	-	Introns/Intergenic	-	-	CGATCGATCCCAAAAAGAAA	CTAGGATCTTTGGTCCCGGT	214	A	A	A	NA
PMS3281	Os_Chr06	5555445	5555460	(TC)8	(TC)8	(TC)8	(TC)8	(TC)8	(TC)8	(TC)8	(TC)8	(TC)9	(TC)8	(TC)8	-	Introns/Intergenic	-	-	TTCCTCAAGTCGCTCAACCT	CGTCAGAACCACACGAACAC	149	A	A	A	NA
PMS3282	Os_Chr06	5559545	5559582	(TA)19	(TA)19	(TA)19	(TA)19	(TA)19	(TA)19	(TA)6	(TA)19	(TA)19	(TA)19	(TA)19	-	Introns/Intergenic	-	-	ATGGCCCACAAGTCATTCTAAGG	CATGTCATGGAGACGAGGAAAGG	365	A	A	A	RM19586
PMS3283	Os_Chr06	5631304	5631321	(CGC)6	(CGC)6	(CGC)6	(CGC)6	(CGC)6	(CGC)6	(CGC)6	(CGC)6	(CGC)6	(CGC)5	(CGC)6	LOC_Os06g10780	CDS	AP2 domain containing protein, expressed	CODON_DELETION	CATGTCGCCCAAGTCCAT	GAGCAAGCTGTAGTCCACGTC	235	A	A	A	NA
PMS3284	Os_Chr06	5637657	5637671	(CCG)5	(CCG)5	(CCG)5	(CCG)5	(CCG)5	(CCG)5	(CCG)5	(CCG)6	(CCG)6	(CCG)6	(CCG)6	LOC_Os06g10800	CDS	zinc finger family protein, putative, expressed	CODON_INSERTION	AAGTACTCCCCCGGATCG	GAGGACCAGTTCTTCCAGCC	224	A	A	A	NA
PMS3285	Os_Chr06	5638344	5638361	(CGG)6	(CGG)6	(CGG)6	(CGG)6	(CGG)6	(CGG)6	(CGG)5	(CGG)6	(CGG)6	(CGG)6	(CGG)6	LOC_Os06g10810	URR	phosphate carrier protein, mitochondrial precursor, putative, expressed	-	AGCCGATATCTCCTTTGGCT	CGTCGAGTAGAGGAAGCTCG	215	A	A	A	NA
PMS3286	Os_Chr06	5767786	5767823	(TA)19	(TA)19	(TA)19	(TA)19	(TA)14	(TA)19	(TA)19	(TA)19	(TA)12	(TA)19	(TA)18	-	Introns/Intergenic	-	-	CTTGCTTTGCACCGGTATTT	CTGAATCGGGAGTTGAGGAG	204	P	A	A	NA
PMS3287	Os_Chr06	5770574	5770645	(AT)36	(AT)36	(AT)36	(AT)36	(AT)7	(AT)36	(AT)36	(AT)36	(AT)10	(AT)36	(AT)36	-	Introns/Intergenic	-	-	CGAGAAAGTTGTCATGGTACTGC	GAGAACGAGAGAACAGCCATACG	613	P	A	A	RM19599
PMS3288	Os_Chr06	5899348	5899374	(ATG)9	(ATG)6	(ATG)9	(ATG)9	(ATG)9	(ATG)9	(ATG)9	(ATG)9	(ATG)9	(ATG)9	(ATG)9	-	Introns/Intergenic	-	-	ACTTGAAACGAGGGAGGATAGGG	GTCGTTGGCTACACGGATTACCC	388	P	A	A	RM19606

Polymorphic SSR (PMS) markers	Chromosomes	Start physical positions (bp)	End physical positions (bp)	Nipponbare	Kasalath	Nagina22	IR64	Pokkali	Bala	Tainung67	Azucena	Moroberekan	*Oryza rufipogon*	*Oryza nivara*	MSU gene locus IDs	Structural annotation	Functional annotation	SSR functional effects	Forward primers (5'-3')	Reverse primers (5'-3')	Amplified product size (bp)	Markers exhibiting polymorphism within *indica* rice	Transcrption factor genes-derived markers	Known cloned genes-derived markers	PMS markers corresponding with RM markers
PMS3289	Os_Chr06	5929798	5929809	(AG)6	(AG)7	(AG)6	(AG)6	(AG)6	(AG)6	(AG)6	(AG)6	(AG)7	(AG)6	(AG)6	-	Introns/Intergenic	-	-	CTGCGATGGAACTGTTGTTG	GGAATTGGCCCAAGAGACTA	225	P	A	A	NA
PMS3290	Os_Chr06	5945721	5945738	(GGC)6	(GGC)6	(GGC)6	(GGC)6	(GGC)6	(GGC)6	(GGC)6	(GGC)6	(GGC)5	(GGC)6	(GGC)6	-	Introns/Intergenic	-	-	CTCTTCGTCTGTCTCTCTCAC	GCGTTGCGCACAGTCGAG	270	A	A	A	NA
PMS3291	Os_Chr06	5971659	5971692	(TA)17	(TA)17	(TA)6	(TA)17	(TA)10	(TA)17	(TA)17	(TA)14	(TA)17	(TA)17	(TA)17	-	Introns/Intergenic	-	-	TCAATTTCAACAATGGTGCC	TTACCCGTTGCAATACACGA	114	P	A	A	NA
PMS3292	Os_Chr06	6027909	6027926	(CGG)6	(CGG)6	(CGG)6	(CGG)6	(CGG)6	(CGG)6	(CGG)6	(CGG)6	(CGG)6	(CGG)5	(CGG)6	LOC_Os06g11420	CDS	SKIP/SNW domain containing protein, expressed	CODON_CHANGE_PLUS_CODON_DELETION	CATCTCCGGTCGTAGTGGAT	GAAGTCCTCCTTCCTCCGA	236	A	A	A	NA
PMS3293	Os_Chr06	6100996	6101009	(CG)7	(CG)6	(CG)7	(CG)7	(CG)7	(CG)6	(CG)7	(CG)7	(CG)6	(CG)7	(CG)7	-	Introns/Intergenic	-	-	TCCACCACCTCATCTCATCA	CATGGCTGGCAACTAACGTA	208	P	A	A	NA
PMS3294	Os_Chr06	6176279	6176293	(CGG)5	(CGG)6	(CGG)6	(CGG)5	(CGG)5	(CGG)6	(CGG)5	(CGG)5	(CGG)6	(CGG)5	(CGG)5	LOC_Os06g11640	URR	serine/threonine-protein phosphatase 2A activator 2, putative, expressed	-	GTGGTGGTGGGTTCGATTC	TGGGCTATCCAGTTTCAACC	214	P	A	A	NA
PMS3295	Os_Chr06	6204106	6204129	(AT)12	(AT)12	(AT)10	(AT)12	(AT)12	(AT)12	(AT)12	(AT)12	(AT)12	(AT)10	(AT)12	-	Introns/Intergenic	-	-	ACCTAAAGTCGGGAACTCATCC	TGTGTTCGACAGCCATATTTCC	431	P	A	A	RM19618
PMS3296	Os_Chr06	6207120	6207137	(GGC)6	(GGC)6	(GGC)6	(GGC)6	(GGC)6	(GGC)6	(GGC)5	(GGC)6	(GGC)6	(GGC)6	(GGC)6	LOC_Os06g11720	CDS	cytokinin-O-glucosyltransferase 2, putative, expressed	CODON_DELETION	CGACCAGTACATCAACTCGC	GTGTGTGCCATGTCGTCACT	274	A	A	A	NA
PMS3297	Os_Chr06	6213792	6213812	(GTG)7	(GTG)5	(GTG)7	(GTG)7	(GTG)7	(GTG)5	(GTG)6	(GTG)7	(GTG)5	(GTG)7	(GTG)7	-	Introns/Intergenic	-	-	GCGACGAGGAAGAAGATTAGTTCG	GCGGCACTTCGAGCAGTACG	173	P	A	A	RM19620
PMS3298	Os_Chr06	6240766	6240786	(CCA)7	(CCA)7	(CCA)7	(CCA)7	(CCA)7	(CCA)7	(CCA)6	(CCA)7	(CCA)7	(CCA)7	(CCA)7	LOC_Os06g11780	CDS	MYB family transcription factor, putative, expressed	CODON_CHANGE_PLUS_CODON_DELETION	CATTCTGCAGTGTCCATTGTGACC	GTGGTTGGTCTGCTCCAGTTCG	87	A	P	A	RM19623
PMS3299	Os_Chr06	6287082	6287097	(GC)8	(GC)8	(GC)8	(GC)8	(GC)8	(GC)8	(GC)8	(GC)8	(GC)8	(GC)8	(GC)7	-	Introns/Intergenic	-	-	AACCCAACTCCTCGACTGAA	GCAGCTGAGAGCACAGTGAC	160	A	A	A	NA
PMS3300	Os_Chr06	6310830	6310845	(AT)8	(AT)8	(AT)8	(AT)8	(AT)8	(AT)7	(AT)8	(AT)8	(AT)8	(AT)8	(AT)8	-	Introns/Intergenic	-	-	GACATCAAATTCACCGGCTT	GAACAATCATCGGTCGGTTC	221	P	A	A	NA
PMS3301	Os_Chr06	6340266	6340277	(AG)6	(AG)6	(AG)6	(AG)6	(AG)6	(AG)7	(AG)6	(AG)6	(AG)6	(AG)6	(AG)7	-	Introns/Intergenic	-	-	GTTCCATGCCCTCGTCTCT	TAGGTTACACGTGGCGTTGA	234	P	A	A	NA

Polymorphic SSR (PMS) markers	Chromosomes	Start physical positions (bp)	End physical positions (bp)	Nipponbare	Kasalath	Nagina22	IR64	Pokkali	Bala	Tainung67	Azucena	Moroberekan	*Oryza rufipogon*	*Oryza nivara*	MSU gene locus IDs	Structural annotation	Functional annotation	SSR functional effects	Forward primers (5'-3')	Reverse primers (5'-3')	Amplified product size (bp)	Markers exhibiting polymorphism within *indica* rice	Transcrption factor genes-derived markers	Known cloned genes-derived markers	PMS markers corresponding with RM markers
PMS3302	Os_Chr06	6376666	6376685	(CT)10	(CT)8	(CT)10	(CT)10	(CT)10	(CT)10	(CT)10	(CT)10	(CT)10	(CT)10	(CT)10	-	Introns/Intergenic	-	-	AGGCCCACCTGTCATCTCTTAGC	GTGAGGTCCACTTGCGTTGTAGG	134	P	A	A	RM19637
PMS3303	Os_Chr06	6376989	6377014	(TC)13	(TC)13	(TC)13	(TC)13	(TC)13	(TC)11	(TC)13	(TC)13	(TC)13	(TC)13	(TC)13	-	Introns/Intergenic	-	-	CCACACTGTACCGGTCGAAGACG	CTCTACAACTTGCAGCCCTGTCAGC	211	P	A	A	RM19638
PMS3304	Os_Chr06	6378387	6378424	(AT)19	(AT)19	(AT)19	(AT)19	(AT)19	(AT)18	(AT)19	(AT)19	(AT)19	(AT)19	(AT)9	-	Introns/Intergenic	-	-	GTTGGAGGGGGACTTTTAGC	TAGCGTTGCCCACATTCATA	219	P	A	A	NA
PMS3305	Os_Chr06	6428578	6428605	(TA)14	(TA)14	(TA)14	(TA)14	(TA)14	(TA)14	(TA)14	(TA)14	(TA)10	(TA)14	(TA)14	-	Introns/Intergenic	-	-	TCTCCGAGCACTTAAGAAAGTTGG	GAAGCGGTGATAGTGACACAAGG	263	A	A	A	RM19642
PMS3306	Os_Chr06	6436283	6436314	(CT)16	(CT)15	(CT)16	(CT)16	(CT)16	(CT)15	(CT)16	(CT)16	(CT)16	(CT)16	(CT)15	-	Introns/Intergenic	-	-	AAGGAGTTCGTCCTCGCTC	AAACTCGCGACCCATAACAC	229	P	A	A	NA
PMS3307	Os_Chr06	6447012	6447023	(AG)6	(AG)7	(AG)6	(AG)6	(AG)6	(AG)6	(AG)6	(AG)6	(AG)6	(AG)6	(AG)6	-	Introns/Intergenic	-	-	GCCATGGCTAACCAAGACAT	GAACTGTTTGGGGGCTTGTA	208	P	A	A	NA
PMS3308	Os_Chr06	6488150	6488176	(GGA)9	(GGA)9	(GGA)9	(GGA)9	(GGA)9	(GGA)9	(GGA)9	(GGA)5	(GGA)9	(GGA)6	(GGA)9	LOC_Os06g12120	URR	OsSERK3	-	GCCAGCGATTTCATCGAACAGG	ACGGCACAGCCTCTCACAAGG	248	A	A	P	RM6779
PMS3309	Os_Chr06	6493825	6493838	(GA)7	(GA)7	(GA)8	(GA)8	(GA)8	(GA)8	(GA)7	(GA)7	(GA)7	(GA)6	(GA)7	-	Introns/Intergenic	-	-	GATGGTGGTAGGAAGAGCCA	AGGCCCCACATGTCAGTAAG	275	P	A	A	NA
PMS3310	Os_Chr06	6563699	6563713	(GCG)5	(GCG)6	(GCG)6	(GCG)5	(GCG)6	(GCG)6	(GCG)5	(GCG)5	(GCG)5	(GCG)6	(GCG)6	-	Introns/Intergenic	-	-	AATGCTAGCACAACGCTCCT	AAGAGCGCTCATCTACCTGC	178	P	A	A	NA
PMS3311	Os_Chr06	6572417	6572437	(CGG)7	(CGG)7	(CGG)7	(CGG)7	(CGG)6	(CGG)7	(CGG)7	(CGG)7	(CGG)7	(CGG)7	(CGG)7	LOC_Os06g12230	CDS	OsTCP19	CODON_DELETION	AGTCAGCATCGTCAGCATCAGC	GACGGAACGGAAGCCCTACG	102	P	A	P	RM19653
PMS3312	Os_Chr06	6629426	6629443	(AT)9	(AT)7	(AT)7	(AT)9	(AT)7	(AT)7	(AT)9	(AT)9	(AT)9	(AT)6	(AT)9	-	Introns/Intergenic	-	-	GCAGGACAGGGCTATTTTGA	GGATTACGCATTTACGGGTG	270	P	A	A	NA
PMS3313	Os_Chr06	6699239	6699252	(AT)7	(AT)8	(AT)8	(AT)7	(AT)7	(AT)8	(AT)7	(AT)7	(AT)7	(AT)7	(AT)7	-	Introns/Intergenic	-	-	CTGGATCCGTCCCTATGAAA	AGTTGCAGTGGCCTGCTTAT	137	P	A	A	NA
PMS3314	Os_Chr06	6712419	6712432	(CT)7	(CT)7	(CT)7	(CT)7	(CT)6	(CT)7	(CT)7	(CT)7	(CT)7	(CT)7	(CT)6	-	Introns/Intergenic	-	-	ACCCTCAACTATGCAAACCG	TGATCTCAACGACAACCTCG	209	P	A	A	NA

Polymorphic SSR (PMS) markers	Chromosomes	Start physical positions (bp)	End physical positions (bp)	Nipponbare	Kasalath	Nagina22	IR64	Pokkali	Bala	Tainung67	Azucena	Moroberekan	*Oryza rufipogon*	*Oryza nivara*	MSU gene locus IDs	Structural annotation	Functional annotation	SSR functional effects	Forward primers (5'-3')	Reverse primers (5'-3')	Amplified product size (bp)	Markers exhibiting polymorphism within *indica* rice	Transcrption factor genes-derived markers	Known cloned genes-derived markers	PMS markers corresponding with RM markers
PMS3315	Os_Chr06	6774255	6774280	(TA)13	(TA)13	(TA)13	(TA)13	(TA)13	(TA)13	(TA)13	(TA)13	(TA)13	(TA)13	(TA)10	-	Introns/Intergenic	-	-	TTTTAAGATGGCTCTGTTTGGTT	GCCATTTACTAGCCTATGTCCAA	269	A	A	A	NA
PMS3316	Os_Chr06	6949842	6949879	(TC)19	(TC)19	(TC)19	(TC)19	(TC)19	(TC)13	(TC)19	(TC)19	(TC)19	(TC)19	(TC)19	-	Introns/Intergenic	-	-	CGTCAGCATCCTCTCTCGCTTTCC	CTGCGTGCTGCTCTTCCATCC	268	P	A	A	RM19666
PMS3317	Os_Chr06	6972234	6972277	(TCTT)11	(TCTT)11	(TCTT)11	(TCTT)11	(TCTT)11	(TCTT)11	(TCTT)11	(TCTT)7	(TCTT)11	(TCTT)11	(TCTT)11	-	Introns/Intergenic	-	-	GACAGATCTTGGGCATTTGTTAGC	TGTACACTTGCATGCTTCTCTACAGG	392	A	A	A	RM7023
PMS3318	Os_Chr06	6977595	6977621	(GCG)9	(GCG)9	(GCG)9	(GCG)5	(GCG)9	(GCG)9	(GCG)5	(GCG)9	(GCG)9	(GCG)5	(GCG)9	-	Introns/Intergenic	-	-	ATCCCTGAACCCAAATTCTGTCG	CTCTTTGATCTTCCGGTGATTTCG	192	P	A	A	RM549
PMS3319	Os_Chr06	6980261	6980278	(GCC)6	(GCC)6	(GCC)6	(GCC)6	(GCC)6	(GCC)6	(GCC)6	(GCC)6	(GCC)6	(GCC)6	(GCC)5	LOC_Os06g12780	CDS	OsDegp10 - Putative Deg protease homologue, expressed	CODON_DELETION	GCCATGGACAACCACGAG	AGAGGAGGGGTGGTGGTG	240	A	A	A	NA
PMS3320	Os_Chr06	7025810	7025827	(CGC)6	(CGC)6	(CGC)6	(CGC)6	(CGC)6	(CGC)6	(CGC)6	(CGC)6	(CGC)6	(CGC)7	(CGC)5	LOC_Os06g12840	CDS	expressed protein	CODON_DELETION	GAGTGGAGGCAGTCCGTCT	GGAGATGAGGAGCATCGTGT	195	A	A	A	NA
PMS3321	Os_Chr06	7031620	7031646	(CGC)9	(CGC)9	(CGC)9	(CGC)9	(CGC)9	(CGC)12	(CGC)9	(CGC)9	(CGC)9	(CGC)9	(CGC)9	LOC_Os06g12850	CDS	expressed protein	CODON_INSERTION	CAAGCAGAAGTGGAGGCAGTCG	GAGAAGGAGGTGGGAGGCATCG	231	P	A	A	RM19670
PMS3322	Os_Chr06	7106952	7106963	(GC)6	(GC)6	(GC)6	(GC)6	(GC)6	(GC)6	(GC)6	(GC)6	(GC)6	(GC)7	(GC)6	-	Introns/Intergenic	-	-	GAAGACATGATTGCGTCGTG	GCCGGGACAGACAAATAAAA	239	A	A	A	NA
PMS3323	Os_Chr06	7125652	7125665	(GA)7	(GA)7	(GA)6	(GA)7	(GA)6	(GA)7	(GA)7	(GA)7	(GA)7	(GA)6	(GA)7	-	Introns/Intergenic	-	-	CGCGTGTTGTGTGTAGAACC	AGACGCCGTCGTAGATGAG	280	P	A	A	NA
PMS3324	Os_Chr06	7157803	7157820	(CCG)6	(CCG)6	(CCG)5	(CCG)6	(CCG)6	(CCG)6	(CCG)6	(CCG)6	(CCG)6	(CCG)6	(CCG)6	-	Introns/Intergenic	-	-	TCCGTGTTATCTTCCTGCACT	CAACGAGACGGCGTTCTTC	220	P	A	A	NA
PMS3325	Os_Chr06	7186609	7186629	(CGC)7	(CGC)7	(CGC)7	(CGC)7	(CGC)7	(CGC)5	(CGC)7	(CGC)7	(CGC)7	(CGC)7	(CGC)7	LOC_Os06g13090	CDS	armadillo/beta-catenin repeat family protein, putative, expressed	CODON_DELETION	AACGACCAGACCAGTGTGTG	CTTCCTGAGGTTGGCGTTC	276	P	A	A	NA
PMS3326	Os_Chr06	7401329	7401349	(GGC)7	(GGC)7	(GGC)7	(GGC)7	(GGC)7	(GGC)6	(GGC)7	(GGC)7	(GGC)7	(GGC)7	(GGC)7	LOC_Os06g13450	CDS	cystathionine beta-lyase, putative, expressed	CODON_CHANGE_PLUS_CODON_DELETION	GGTCGCCGGACTTCCAAGACTACC	GTTGCCGTAACGGGCGTACTCG	298	P	A	A	RM19688
PMS3327	Os_Chr06	7497279	7497328	(AT)25	(AT)25	(AT)11	(AT)25	(AT)25	(AT)11	(AT)25	(AT)25	(AT)25	(AT)16	(AT)22	-	Introns/Intergenic	-	-	ATGGTGGATGAGAGCAGGAG	ATTAGATACCATGGTGCCCG	148	P	A	A	NA

Polymorphic SSR (PMS) markers	Chromosomes	Start physical positions (bp)	End physical positions (bp)	Nipponbare	Kasalath	Nagina22	IR64	Pokkali	Bala	Tainung67	Azucena	Moroberekan	*Oryza rufipogon*	*Oryza nivara*	MSU gene locus IDs	Structural annotation	Functional annotation	SSR functional effects	Forward primers (5'-3')	Reverse primers (5'-3')	Amplified product size (bp)	Markers exhibiting polymorphism within *indica* rice	Transcrption factor genes-derived markers	Known cloned genes-derived markers	PMS markers corresponding with RM markers
PMS3328	Os_Chr06	7688800	7688837	(TA)19	(TA)19	(TA)19	(TA)10	(TA)19	(TA)19	(TA)6	(TA)19	(TA)19	(TA)19	(TA)15	-	Introns/Intergenic	-	-	GCACTCGCATTAAATGTGCATACG	CTCCTTGTCCAGATGGAGGAACC	500	P	A	A	RM19707
PMS3329	Os_Chr06	7709056	7709067	(CA)6	(CA)6	(CA)6	(CA)6	(CA)6	(CA)6	(CA)6	(CA)7	(CA)6	(CA)6	(CA)6	LOC_Os06g13870	URR	U-box protein CMPG1, putative, expressed	-	TCAATCCACCCTTTCAAACC	CAGCAACGATATCTCTGGCA	258	A	A	A	NA
PMS3330	Os_Chr06	7726166	7726193	(TA)14	(TA)14	(TA)14	(TA)10	(TA)14	(TA)14	(TA)11	(TA)13	(TA)14	(TA)14	(TA)7	-	Introns/Intergenic	-	-	GGGTATGTGCTCAATCGAGAAGG	ACAAGGCGAACTCATTGTGATGG	358	P	A	A	RM19708
PMS3331	Os_Chr06	7769854	7769871	(GCG)6	(GCG)5	(GCG)6	(GCG)6	(GCG)6	(GCG)6	(GCG)6	(GCG)6	(GCG)6	(GCG)6	(GCG)6	LOC_Os06g13950	CDS	pentatricopeptide, putative, expressed	CODON_DELETION	CGGAGGAGGAGGAACAGAG	ACGAGTCCCCACTCCTTTCT	170	P	A	A	NA
PMS3332	Os_Chr06	7813943	7813999	(TAT)19	(TAT)19	(TAT)6	(TAT)19	(TAT)19	(TAT)19	(TAT)19	(TAT)19	(TAT)13	(TAT)19	(TAT)14	-	Introns/Intergenic	-	-	CTTATCAGCCACACATTTGGAACC	CATATGGGCTCAGGGATTATTGG	272	P	A	A	RM19713
PMS3333	Os_Chr06	7956134	7956151	(GCC)6	(GCC)6	(GCC)6	(GCC)6	(GCC)6	(GCC)6	(GCC)6	(GCC)6	(GCC)5	(GCC)6	(GCC)6	LOC_Os06g14260	CDS	lectin-like receptor kinase, putative, expressed	CODON_DELETION	GACAGCGACACGACCTTCACG	GCGGTCCATGGTGCTCAAGG	297	A	A	A	RM19718
PMS3334	Os_Chr06	8014520	8014537	(GAA)6	(GAA)7	(GAA)7	(GAA)6	(GAA)6	(GAA)7	(GAA)6	(GAA)6	(GAA)6	(GAA)6	(GAA)6	LOC_Os06g14370	CDS	caleosin related protein, putative, expressed	CODON_INSERTION	GGCGTTGTTGTTGTTGTCAC	TCTCTGATCTCGTCGTTCCC	257	P	A	A	NA
PMS3335	Os_Chr06	8085764	8085791	(TA)14	(TA)14	(TA)14	(TA)9	(TA)14	(TA)14	(TA)8	(TA)14	(TA)14	(TA)14	(TA)14	-	Introns/Intergenic	-	-	TCATGAGTGCCCTACCTCAATCC	ACAATGTGGTGGTTCTCTGTTGC	177	P	A	A	RM19723
PMS3336	Os_Chr06	8086026	8086039	(AT)7	(AT)7	(AT)7	(AT)7	(AT)7	(AT)7	(AT)7	(AT)7	(AT)6	(AT)7	(AT)7	-	Introns/Intergenic	-	-	TATTCCATCAATTCCAGCCC	CTCTGCCATGCACGTAATTG	177	A	A	A	NA
PMS3337	Os_Chr06	8105373	8105390	(GCG)6	(GCG)6	(GCG)6	(GCG)6	(GCG)6	(GCG)6	(GCG)6	(GCG)6	(GCG)6	(GCG)5	(GCG)6	-	Introns/Intergenic	-	-	CTCCTTCCTCTCTCCCTTCG	CTATGCCTTCTCCTCCACCA	239	A	A	A	NA
PMS3338	Os_Chr06	8136155	8136178	(AT)12	(AT)12	(AT)12	(AT)7	(AT)12	(AT)8	(AT)12	(AT)6	(AT)12	(AT)12	(AT)12	-	Introns/Intergenic	-	-	GACATTGGAACACCAACCAA	AGGGTTTTGAAAAATCGGCT	209	P	A	A	NA
PMS3339	Os_Chr06	8184525	8184542	(GGT)6	(GGT)6	(GGT)6	(GGT)6	(GGT)6	(GGT)6	(GGT)6	(GGT)6	(GGT)6	(GGT)6	(GGT)5	LOC_Os06g14540	URR	endoglucanase, putative, expressed	-	CCTCGAAGAACAGGATGCTC	GCTCAGGCTCAGCTCCTCTA	222	A	A	A	NA
PMS3340	Os_Chr06	8190754	8190804	(TTA)17	(TTA)17	(TTA)8	(TTA)17	(TTA)14	(TTA)17	(TTA)17	(TTA)17	(TTA)17	(TTA)17	(TTA)12	-	Introns/Intergenic	-	-	TGTCAATCTTTTGACCATTCG	CAACGCATGTGCAATATGCTA	271	P	A	A	NA

Polymorphic SSR (PMS) markers	Chromosomes	Start physical positions (bp)	End physical positions (bp)	Nipponbare	Kasalath	Nagina22	IR64	Pokkali	Bala	Tainung67	Azucena	Moroberekan	*Oryza rufipogon*	*Oryza nivara*	MSU gene locus IDs	Structural annotation	Functional annotation	SSR functional effects	Forward primers (5'-3')	Reverse primers (5'-3')	Amplified product size (bp)	Markers exhibiting polymorphism within *indica* rice	Transcrption factor genes-derived markers	Known cloned genes-derived markers	PMS markers corresponding with RM markers
PMS3341	Os_Chr06	8245105	8245122	(GGC)6	(GGC)6	(GGC)6	(GGC)6	(GGC)6	(GGC)6	(GGC)5	(GGC)6	(GGC)6	(GGC)6	(GGC)6	LOC_Os06g14650	CDS	zinc finger, C3HC4 type domain containing protein, expressed	CODON_DELETION	ATCGTAGTCGTGATCGGAGC	AGCCTGATGAGTCCCTAAGC	234	A	A	A	NA
PMS3342	Os_Chr06	8251830	8251853	(CGC)8	(CGC)8	(CGC)8	(CGC)8	(CGC)8	(CGC)8	(CGC)8	(CGC)6	(CGC)8	(CGC)8	(CGC)8	LOC_Os06g14670	CDS	ODORANT1, putative, expressed	CODON_CHANGE_PLUS_CODON_DELETION	ATCAAGAAGAAGCTCCGCAA	CAGGGGAAACAGAGCAGTTG	244	A	A	A	NA
PMS3343	Os_Chr06	8260683	8260696	(TC)7	(TC)7	(TC)7	(TC)7	(TC)7	(TC)7	(TC)6	(TC)7	(TC)7	(TC)7	(TC)7	-	Introns/Intergenic	-	-	CGTATCTCTGGGGTGAGAGC	GGGAGGCGGAGTCGTATT	144	A	A	A	NA
PMS3344	Os_Chr06	8385879	8385892	(AG)7	(AG)7	(AG)7	(AG)6	(AG)7	(AG)7	(AG)7	(AG)7	(AG)7	(AG)7	(AG)7	-	Introns/Intergenic	-	-	TCGGAGCTCGGATGAATTAG	GCTTTGAAGCCGAGTCGAG	211	P	A	A	NA
PMS3345	Os_Chr06	8430827	8430886	(TA)30	(TA)30	(TA)30	(TA)30	(TA)30	(TA)30	(TA)30	(TA)30	(TA)30	(TA)30	(TA)16	-	Introns/Intergenic	-	-	CTAGACCAGATTGTGGATGAACG	GAAGTGGAAGAGATCCGCTAGG	192	A	A	A	RM19744
PMS3346	Os_Chr06	8507676	8507717	(TAT)14	(TAT)14	(TAT)14	(TAT)14	(TAT)14	(TAT)14	(TAT)14	(TAT)13	(TAT)14	(TAT)14	(TAT)14	-	Introns/Intergenic	-	-	CCACCCATCCTGTAGATAGTACG	TTACAGAGGAGATTAGGAGTGAGG	400	A	A	A	RM19746
PMS3347	Os_Chr06	8580951	8580971	(GAC)7	(GAC)7	(GAC)7	(GAC)7	(GAC)7	(GAC)7	(GAC)7	(GAC)7	(GAC)7	(GAC)7	(GAC)5	-	Introns/Intergenic	-	-	CTTGGACGAAGGCGAGAC	CTCCGTCATCATCATCGCT	274	A	A	A	NA
PMS3348	Os_Chr06	8745984	8746019	(GA)18	(GA)18	(GA)18	(GA)18	(GA)18	(GA)18	(GA)18	(GA)13	(GA)18	(GA)18	(GA)18	LOC_Os06g15390	URR	acylphosphatase, putative, expressed	-	AACATTCTGGAAGACACGGG	AATCTACACGGGCACACACA	205	A	A	A	NA
PMS3349	Os_Chr06	8815672	8815698	(CTG)9	(CTG)9	(CTG)9	(CTG)9	(CTG)9	(CTG)9	(CTG)9	(CTG)9	(CTG)9	(CTG)9	(CTG)6	-	Introns/Intergenic	-	-	TCAAGTTACGGGAAATGTGTGG	CTGCCTAGCTTCCGTTTCTCC	81	A	A	A	RM5963
PMS3350	Os_Chr06	8920014	8920069	(AT)28	(AT)22	(AT)9	(AT)28	(AT)28	(AT)16	(AT)28	(AT)28	(AT)28	(AT)11	(AT)6	-	Introns/Intergenic	-	-	CCACATCGTCATCCGTAGTG	CCTATGACTGATCCAACGGC	256	P	A	A	NA
PMS3351	Os_Chr06	8973749	8973772	(GAA)8	(GAA)8	(GAA)8	(GAA)8	(GAA)8	(GAA)8	(GAA)8	(GAA)5	(GAA)8	(GAA)8	(GAA)8	-	Introns/Intergenic	-	-	TTCTCCGTCGAAGGATTCCAACC	ACTTCCACCTCCCACTCTCATCG	95	A	A	A	RM6302
PMS3352	Os_Chr06	9027483	9027500	(AGT)6	(AGT)5	(AGT)6	(AGT)6	(AGT)6	(AGT)6	(AGT)6	(AGT)6	(AGT)6	(AGT)6	(AGT)6	-	Introns/Intergenic	-	-	GGCAAGAGGAAGAATGCAAG	CACCAGCAGAAAATGAGCAA	254	P	A	A	NA
PMS3353	Os_Chr06	9042871	9042884	(TA)7	(TA)6	(TA)7	(TA)7	(TA)7	(TA)6	(TA)7	(TA)7	(TA)7	(TA)7	(TA)7	-	Introns/Intergenic	-	-	GCACTTCTCTCCCCTATCCC	CGTCTCACTCCTAAGCGACC	164	P	A	A	NA

**Polymorphic SSR (PMS) markers**	**Chromosomes**	**Start physical positions (bp)**	**End physical positions (bp)**	**Nipponbare**	**Kasalath**	**Nagina22**	**IR64**	**Pokkali**	**Bala**	**Tainung67**	**Azucena**	**Moroberekan**	***Oryza rufipogon***	***Oryza nivara***	**MSU gene locus IDs**	**Structural annotation**	**Functional annotation**	**SSR functional effects**	**Forward primers (5'-3')**	**Reverse primers (5'-3')**	**Amplified product size (bp)**	**Markers exhibiting polymorphism within *indica* rice**	**Transcrption factor genes-derived markers**	**Known cloned genes-derived markers**	**PMS markers corresponding with RM markers**
PMS3354	Os_Chr06	9142692	9142709	(CT)9	(CT)8	(CT)9	(CT)9	(CT)9	(CT)8	(CT)9	(CT)9	(CT)8	(CT)9	(CT)9	-	Introns/Intergenic	-	-	CCGGTGAGATCCATGTGTTA	CCCAAACTTGTCCTGGATGT	222	P	A	A	NA
PMS3355	Os_Chr06	9205401	9205421	(CGC)7	(CGC)7	(CGC)7	(CGC)7	(CGC)7	(CGC)7	(CGC)7	(CGC)5	(CGC)7	(CGC)7	(CGC)7	LOC_Os06g16160	CDS	expressed protein	CODON_DELETION	AGTTCCCGTCCTTGTTCCACTCC	CCAACGTCACCGACCTCACG	162	A	A	A	RM19775
PMS3356	Os_Chr06	9255144	9255167	(GCG)8	(GCG)5	(GCG)8	(GCG)8	(GCG)8	(GCG)8	(GCG)5	(GCG)5	(GCG)8	(GCG)8	(GCG)8	LOC_Os06g16250	CDS	expressed protein	CODON_DELETION	GCCATTGTCAAGCAGCCAGAGC	CGTCACGTCCATCAGCCTCAGC	324	P	A	A	RM19777
PMS3357	Os_Chr06	9269722	9269737	(GA)8	(GA)9	(GA)8	(GA)8	(GA)8	(GA)8	(GA)8	(GA)8	(GA)8	(GA)8	(GA)8	-	Introns/Intergenic	-	-	CCTACTGCGGTTGGGTAGAA	GAGACGTATGCAATCCAGCA	272	P	A	A	NA
PMS3358	Os_Chr06	9460183	9460198	(TA)8	(TA)7	(TA)8	(TA)8	(TA)8	(TA)8	(TA)8	(TA)8	(TA)8	(TA)8	(TA)8	-	Introns/Intergenic	-	-	TTTTCCGGTACGGAATCAAC	TAAAGGGTCCCATCACTTCG	225	P	A	A	NA
PMS3359	Os_Chr06	9561015	9561030	(TC)8	(TC)8	(TC)8	(TC)6	(TC)8	(TC)8	(TC)8	(TC)8	(TC)8	(TC)8	(TC)8	-	Introns/Intergenic	-	-	TCGATCTCTCTCCCCTCTCC	ACCGTAAGGTCATGGGTTCA	225	P	A	A	NA
PMS3360	Os_Chr06	9640056	9640070	(GAC)5	(GAC)7	(GAC)7	(GAC)7	(GAC)7	(GAC)7	(GAC)5	(GAC)7	(GAC)7	(GAC)5	(GAC)7	-	Introns/Intergenic	-	-	GCTAGCTACCACTCCGGCTT	CGGTCGCCTTATACGACACT	133	A	A	A	NA
PMS3361	Os_Chr06	9711299	9711342	(TA)22	(TA)22	(TA)22	(TA)22	(TA)22	(TA)22	(TA)22	(TA)22	(TA)22	(TA)22	(TA)13	-	Introns/Intergenic	-	-	AATGGGATGTGGATGTGAATGC	GGCTAGCCTCAGGCCTTTGC	178	A	A	A	RM19801
PMS3362	Os_Chr06	9908047	9908072	(TA)13	(TA)13	(TA)13	(TA)13	(TA)13	(TA)11	(TA)13	(TA)13	(TA)13	(TA)13	(TA)13	-	Introns/Intergenic	-	-	TTTCGTGTGCTGTTCACTAGG	AGGAGACGGGAGTAGGAAAGG	136	P	A	A	RM19808
PMS3363	Os_Chr06	9996203	9996224	(AT)11	(AT)11	(AT)11	(AT)11	(AT)11	(AT)11	(AT)11	(AT)11	(AT)7	(AT)11	(AT)11	-	Introns/Intergenic	-	-	CCATTGTGGGGGTAAAAACA	CGATTATACCATTGCGTTCG	275	A	A	A	NA
PMS3364	Os_Chr06	10101491	10101505	(GCG)5	(GCG)6	(GCG)5	(GCG)5	(GCG)5	(GCG)6	(GCG)5	(GCG)5	(GCG)6	(GCG)5	(GCG)5	LOC_Os06g17410	CDS	dof zinc finger domain containing protein, putative, expressed	CODON_INSERTION	TCTGGAATCAATCTCCGGTC	GGGCTGGGAGAGGTTGTAGT	186	P	A	A	NA
PMS3365	Os_Chr06	10130040	10130057	(AT)9	(AT)9	(AT)9	(AT)9	(AT)9	(AT)9	(AT)9	(AT)7	(AT)9	(AT)9	(AT)9	-	Introns/Intergenic	-	-	TCGGATTGTAAATCACTGCAC	ACTCCCTCCGTTTCACAATG	185	A	A	A	NA
PMS3366	Os_Chr06	10260977	10260990	(CG)7	(CG)7	(CG)7	(CG)7	(CG)6	(CG)7	(CG)7	(CG)7	(CG)7	(CG)7	(CG)7	-	Introns/Intergenic	-	-	CTTCTCTCTCTTCTCGGCCC	GGAGATCCCGGAGATCATTT	226	P	A	A	NA

Polymorphic SSR (PMS) markers	Chromosomes	Start physical positions (bp)	End physical positions (bp)	Nipponbare	Kasalath	Nagina22	IR64	Pokkali	Bala	Tainung67	Azucena	Moroberekan	*Oryza rufipogon*	*Oryza nivara*	MSU gene locus IDs	Structural annotation	Functional annotation	SSR functional effects	Forward primers (5'-3')	Reverse primers (5'-3')	Amplified product size (bp)	Markers exhibiting polymorphism within *indica* rice	Transcrption factor genes-derived markers	Known cloned genes-derived markers	PMS markers corresponding with RM markers
PMS3367	Os_Chr06	10600184	10600197	(GA)7	(GA)7	(GA)7	(GA)7	(GA)7	(GA)7	(GA)7	(GA)6	(GA)7	(GA)7	(GA)7	-	Introns/Intergenic	-	-	TCAACAGACGCCAACTTCAC	GCAGCTACGAGAACGACCTT	221	A	A	A	NA
PMS3368	Os_Chr06	10605442	10605459	(CT)9	(CT)9	(CT)9	(CT)10	(CT)9	(CT)9	(CT)9	(CT)9	(CT)9	(CT)9	(CT)10	-	Introns/Intergenic	-	-	CAAGCTCCCCAGTTTCCATA	ATGCGGCAGTGTTGATAGTG	214	P	A	A	NA
PMS3369	Os_Chr06	10605753	10605788	(TCTA)9	(TCTA)9	(TCTA)9	(TCTA)9	(TCTA)9	(TCTA)9	(TCTA)7	(TCTA)9	(TCTA)9	(TCTA)9	(TCTA)9	-	Introns/Intergenic	-	-	CTGCACCCATCTCTCTCTCC	CTATAGCCAGCGACGAGGAC	211	A	A	A	NA
PMS3370	Os_Chr06	10759704	10759721	(GCG)6	(GCG)6	(GCG)6	(GCG)6	(GCG)6	(GCG)6	(GCG)6	(GCG)6	(GCG)6	(GCG)6	(GCG)5	LOC_Os06g18930	CDS	cadmium tolerance factor, putative, expressed	CODON_CHANGE_PLUS_CODON_DELETION	GTACCACGCCTTCTTCCACT	GCGATGTACACCACGACG	243	A	A	A	NA
PMS3371	Os_Chr06	10779487	10779504	(AG)9	(AG)9	(AG)9	(AG)9	(AG)9	(AG)9	(AG)9	(AG)9	(AG)9	(AG)9	(AG)6	-	Introns/Intergenic	-	-	ATCCACACTGAGACGGGC	CCTCCTCTCCTCTCAGTCTTCTC	258	A	A	A	NA
PMS3372	Os_Chr06	10973835	10973858	(TA)12	(TA)12	(TA)12	(TA)12	(TA)12	(TA)12	(TA)13	(TA)12	(TA)12	(TA)12	(TA)12	-	Introns/Intergenic	-	-	TCTTAGCCGCGACTTGACTT	TCGCCTGTATTGTGTGATCC	129	A	A	A	NA
PMS3373	Os_Chr06	10999821	10999882	(TA)31	(TA)31	(TA)10	(TA)31	(TA)31	(TA)7	(TA)6	(TA)6	(TA)6	(TA)31	(TA)31	-	Introns/Intergenic	-	-	CTAGCCTGTTTGAGATGACTTCTGC	CCAGCCTTATCTCCTCCTGTGG	135	P	A	A	RM19841
PMS3374	Os_Chr06	11000422	11000442	(TTG)7	(TTG)15	(TTG)7	(TTG)7	(TTG)7	(TTG)15	(TTG)7	(TTG)7	(TTG)7	(TTG)7	(TTG)7	-	Introns/Intergenic	-	-	GCTAGGTACCACCTCCTCCATCC	CCCACATTCTCAAACCAAACACC	154	P	A	A	RM19842
PMS3375	Os_Chr06	11065782	11065805	(AT)12	(AT)12	(AT)12	(AT)12	(AT)6	(AT)12	(AT)12	(AT)12	(AT)12	(AT)12	(AT)12	-	Introns/Intergenic	-	-	TTGGAAGAAGTAGGGTGGATGAGC	ACGGTTGACGCAGAGGAGAGG	450	P	A	A	RM19845
PMS3376	Os_Chr06	11105215	11105230	(CT)8	(CT)8	(CT)8	(CT)6	(CT)8	(CT)8	(CT)8	(CT)8	(CT)6	(CT)7	(CT)6	-	Introns/Intergenic	-	-	CTTGCCGAATCATGAAGACA	TGTTTCACTGTGTTGCTCGG	158	P	A	A	NA
PMS3377	Os_Chr06	11140405	11140422	(GCG)6	(GCG)6	(GCG)6	(GCG)5	(GCG)6	(GCG)6	(GCG)6	(GCG)6	(GCG)5	(GCG)6	(GCG)5	-	Introns/Intergenic	-	-	GAAAGCAACGTCCTCTGCTC	ACGAGGATGGTGGAAAACCT	227	P	A	A	NA
PMS3378	Os_Chr06	11316540	11316565	(AT)13	(AT)13	(AT)9	(AT)13	(AT)8	(AT)6	(AT)13	(AT)13	(AT)12	(AT)13	(AT)13	-	Introns/Intergenic	-	-	TTTGTCACCTCTCACCATCATCC	TAGCTCCCTTAGGCCTTGATTGG	476	P	A	A	RM19854
PMS3379	Os_Chr06	11365780	11365857	(AT)39	(AT)39	(AT)17	(AT)10	(AT)11	(AT)39	(AT)39	(AT)39	(AT)6	(AT)15	(AT)39	-	Introns/Intergenic	-	-	GGTGGTTCAAGAACAACTCATGG	GGTGGAGAAAGCGTTTGTAGTCC	640	P	A	A	RM21206

Polymorphic SSR (PMS) markers	Chromosomes	Start physical positions (bp)	End physical positions (bp)	Nipponbare	Kasalath	Nagina22	IR64	Pokkali	Bala	Tainung67	Azucena	Moroberekan	*Oryza rufipogon*	*Oryza nivara*	MSU gene locus IDs	Structural annotation	Functional annotation	SSR functional effects	Forward primers (5'-3')	Reverse primers (5'-3')	Amplified product size (bp)	Markers exhibiting polymorphism within *indica* rice	Transcrption factor genes-derived markers	Known cloned genes-derived markers	PMS markers corresponding with RM markers
PMS3380	Os_Chr06	11463510	11463571	(TA)31	(TA)31	(TA)31	(TA)31	(TA)10	(TA)31	(TA)31	(TA)31	(TA)31	(TA)31	(TA)31	-	Introns/Intergenic	-	-	GGTAGCTGGTTTGTACTCTATCTAGC	CCAATTTGTGAGAAGGACTGC	484	P	A	A	RM19864
PMS3381	Os_Chr06	11511745	11511802	(TA)29	(TA)29	(TA)29	(TA)29	(TA)7	(TA)11	(TA)11	(TA)10	(TA)29	(TA)19	(TA)7	-	Introns/Intergenic	-	-	GCTGAAACATGCCTGGTGATGG	CGCCTCCAACCACCCTATCG	231	P	A	A	RM19866
PMS3382	Os_Chr06	11575761	11575772	(GA)6	(GA)9	(GA)6	(GA)6	(GA)9	(GA)6	(GA)6	(GA)6	(GA)6	(GA)6	(GA)6	-	Introns/Intergenic	-	-	GGACACCACAAAGACGTGAA	CCAGCTCCACTTGCCTACTC	239	P	A	A	NA
PMS3383	Os_Chr06	11657243	11657266	(TA)12	(TA)12	(TA)11	(TA)12	(TA)10	(TA)12	(TA)12	(TA)12	(TA)8	(TA)12	(TA)12	-	Introns/Intergenic	-	-	ATCTGTAAACTTCCCGGTGC	GGCGAGCTAGTTCAAGTTGG	272	P	A	A	NA
PMS3384	Os_Chr06	11768307	11768334	(GT)14	(GT)14	(GT)11	(GT)14	(GT)14	(GT)14	(GT)14	(GT)14	(GT)14	(GT)14	(GT)14	-	Introns/Intergenic	-	-	TTCCTGATACATCCCAAGCC	ATCCGAAATCGGTGTGTCAT	272	P	A	A	NA
PMS3385	Os_Chr06	11780185	11780234	(AT)25	(AT)25	(AT)13	(AT)25	(AT)13	(AT)25	(AT)7	(AT)25	(AT)25	(AT)11	(AT)9	-	Introns/Intergenic	-	-	TAGTTCTAGCCGCAGTGCAA	TATTTCATACCATGGTGCCC	206	P	A	A	NA
PMS3386	Os_Chr06	11950472	11950515	(TA)22	(TA)22	(TA)22	(TA)22	(TA)22	(TA)22	(TA)22	(TA)22	(TA)22	(TA)7	(TA)22	-	Introns/Intergenic	-	-	TTTAGCATGGGCATCGATTTGC	GCAAAGGTGGGATCAAGATTGG	249	A	A	A	RM19890
PMS3387	Os_Chr06	12312457	12312482	(TC)13	(TC)13	(TC)13	(TC)13	(TC)13	(TC)13	(TC)13	(TC)12	(TC)13	(TC)13	(TC)13	-	Introns/Intergenic	-	-	CTCCCTGCTTCTTGTCTCTCAGG	GCACAGAAAGCAGGTGACATATCG	149	A	A	A	RM19917
PMS3388	Os_Chr06	12398588	12398611	(TA)12	(TA)12	(TA)12	(TA)12	(TA)12	(TA)12	(TA)12	(TA)12	(TA)12	(TA)7	(TA)12	-	Introns/Intergenic	-	-	ACCAGGTGGTGTGAACCTATTCC	CACGATTAACCATCGTGAGCTACC	258	A	A	A	RM19922
PMS3389	Os_Chr06	12412562	12412585	(GCC)8	(GCC)6	(GCC)8	(GCC)8	(GCC)8	(GCC)6	(GCC)8	(GCC)8	(GCC)8	(GCC)8	(GCC)8	LOC_Os06g21480	URR	60S ribosomal protein L31, putative, expressed	-	TATAACGACGCGATCCAACA	CTCTCCTCCTCCTACGGCTT	114	P	A	A	NA
PMS3390	Os_Chr06	12622031	12622082	(AT)26	(AT)26	(AT)25	(AT)10	(AT)15	(AT)26	(AT)26	(AT)26	(AT)26	(AT)13	(AT)26	-	Introns/Intergenic	-	-	CTATCGGATGATCCACTGTCAGG	TAGAGGCCAGGGATGATGTCG	163	P	A	A	RM19930
PMS3391	Os_Chr06	12659109	12659132	(TA)12	(TA)12	(TA)12	(TA)12	(TA)12	(TA)12	(TA)12	(TA)12	(TA)12	(TA)8	(TA)12	-	Introns/Intergenic	-	-	AGCTCCAGTCCACAGGATGACC	GTCTGTCGAACAGCACCAATGC	129	A	A	A	RM19933
PMS3392	Os_Chr06	12801601	12801618	(CGC)6	(CGC)6	(CGC)6	(CGC)6	(CGC)6	(CGC)6	(CGC)6	(CGC)5	(CGC)6	(CGC)6	(CGC)6	LOC_Os06g22070	CDS	mitochondrial glycoprotein, putative, expressed	CODON_DELETION	CCGCAAAACCCTAGAAATTG	GGAGGTGATCCCAGAGGAC	231	A	A	A	NA

Polymorphic SSR (PMS) markers	Chromosomes	Start physical positions (bp)	End physical positions (bp)	Nipponbare	Kasalath	Nagina22	IR64	Pokkali	Bala	Tainung67	Azucena	Moroberekan	*Oryza rufipogon*	*Oryza nivara*	MSU gene locus IDs	Structural annotation	Functional annotation	SSR functional effects	Forward primers (5'-3')	Reverse primers (5'-3')	Amplified product size (bp)	Markers exhibiting polymorphism within *indica* rice	Transcrption factor genes-derived markers	Known cloned genes-derived markers	PMS markers corresponding with RM markers
PMS3393	Os_Chr06	12809883	12809897	(GGC)5	(GGC)5	(GGC)5	(GGC)5	(GGC)5	(GGC)6	(GGC)5	(GGC)5	(GGC)5	(GGC)5	(GGC)5	LOC_Os06g22080	CDS	diacylglycerol O-acyltransferase, putative, expressed	CODON_INSERTION	GGGAAGGAAAGGGAGAGAGA	AAGACGTTGAAGTGGATGCC	255	P	A	A	NA
PMS3394	Os_Chr06	12852755	12852778	(GGC)8	(GGC)8	(GGC)6	(GGC)8	(GGC)8	(GGC)8	(GGC)8	(GGC)8	(GGC)8	(GGC)8	(GGC)8	LOC_Os06g22140	URR	NTRA	-	GCTCCCGATGATGCACAC	TAGCCGCCAAGAAGAAGAAA	229	P	A	P	NA
PMS3395	Os_Chr06	12920451	12920468	(GGC)6	(GGC)6	(GGC)6	(GGC)6	(GGC)6	(GGC)6	(GGC)5	(GGC)6	(GGC)6	(GGC)6	(GGC)6	-	Introns/Intergenic	-	-	GAGGAAGGTGACGGAGGAGT	CTCTGTCCTCGACGCCCT	186	A	A	A	NA
PMS3396	Os_Chr06	13291652	13291671	(TA)10	(TA)10	(TA)10	(TA)10	(TA)9	(TA)10	(TA)10	(TA)10	(TA)10	(TA)10	(TA)10	-	Introns/Intergenic	-	-	TCAATTTCAACAATGGTGCC	CCGTAGGGAGGTAACGGATT	244	P	A	A	NA
PMS3397	Os_Chr06	13333834	13333909	(TA)38	(TA)38	(TA)38	(TA)38	(TA)12	(TA)14	(TA)38	(TA)38	(TA)38	(TA)12	(TA)38	-	Introns/Intergenic	-	-	CGCTCGCATCTTCTTAATTTG	AACGATTCAAAATTATGTGAAACTTAC	248	P	A	A	NA
PMS3398	Os_Chr06	13401073	13401092	(ATAG)5	(ATAG)7	(ATAG)7	(ATAG)7	(ATAG)5	(ATAG)7	(ATAG)5	(ATAG)5	(ATAG)5	(ATAG)5	(ATAG)7	-	Introns/Intergenic	-	-	GCCCAAATTGGAAGGAGATT	ATCCTTTGCCATCCTTACGA	228	P	A	A	NA
PMS3399	Os_Chr06	13618719	13618742	(AT)12	(AT)12	(AT)12	(AT)12	(AT)12	(AT)12	(AT)7	(AT)12	(AT)12	(AT)12	(AT)12	-	Introns/Intergenic	-	-	TGTTGAGATTTTTCATCGCA	CATCCCTCAGACGAATTACCA	261	A	A	A	NA
PMS3400	Os_Chr06	13906895	13906912	(GGT)6	(GGT)6	(GGT)6	(GGT)6	(GGT)6	(GGT)6	(GGT)5	(GGT)6	(GGT)6	(GGT)6	(GGT)6	-	Introns/Intergenic	-	-	AACAAGGAAGAGGGGAGGAG	CATTCCGATGTAGTGGGCTT	198	A	A	A	NA
PMS3401	Os_Chr06	13909543	13909574	(TA)16	(TA)9	(TA)16	(TA)16	(TA)9	(TA)16	(TA)16	(TA)6	(TA)16	(TA)16	(TA)16	-	Introns/Intergenic	-	-	ATTACGTATCCTGGGTGGTTTCC	CTACGTATCCTGGGTGGTTTCC	121	P	A	A	RM19986
PMS3402	Os_Chr06	13938454	13938469	(GC)8	(GC)8	(GC)8	(GC)8	(GC)6	(GC)8	(GC)8	(GC)8	(GC)8	(GC)8	(GC)8	-	Introns/Intergenic	-	-	GGTTAGTGGCTGCAAAAGGA	CCCTGTTGACCAGTTGAGGT	136	P	A	A	NA
PMS3403	Os_Chr06	14029976	14029989	(CT)7	(CT)6	(CT)7	(CT)7	(CT)7	(CT)7	(CT)7	(CT)7	(CT)7	(CT)7	(CT)7	-	Introns/Intergenic	-	-	CCACCATGCATGTAATCACC	GACGCCGTACCCTATTGAGA	258	P	A	A	NA
PMS3404	Os_Chr06	14305545	14305560	(AC)8	(AC)8	(AC)8	(AC)8	(AC)7	(AC)8	(AC)8	(AC)8	(AC)8	(AC)8	(AC)7	-	Introns/Intergenic	-	-	TTATCTCGGCGATGGTCTTC	TTGATATCGCGGTCTGAAAA	269	P	A	A	NA
PMS3405	Os_Chr06	14457299	14457313	(GCG)5	(GCG)6	(GCG)5	(GCG)5	(GCG)5	(GCG)5	(GCG)5	(GCG)5	(GCG)5	(GCG)6	(GCG)5	-	Introns/Intergenic	-	-	ATGGTGCTCACCAAAACGA	GTTGTCCTCTTTCGTTTGCC	264	P	A	A	NA

Polymorphic SSR (PMS) markers	Chromosomes	Start physical positions (bp)	End physical positions (bp)	Nipponbare	Kasalath	Nagina22	IR64	Pokkali	Bala	Tainung67	Azucena	Moroberekan	*Oryza rufipogon*	*Oryza nivara*	MSU gene locus IDs	Structural annotation	Functional annotation	SSR functional effects	Forward primers (5'-3')	Reverse primers (5'-3')	Amplified product size (bp)	Markers exhibiting polymorphism within *indica* rice	Transcrption factor genes-derived markers	Known cloned genes-derived markers	PMS markers corresponding with RM markers
PMS3406	Os_Chr06	14548994	14549011	(AG)9	(AG)8	(AG)8	(AG)9	(AG)9	(AG)8	(AG)9	(AG)9	(AG)9	(AG)9	(AG)9	-	Introns/Intergenic	-	-	CAAGCTCTTGCGTCTTTAGGA	AGGAGCGGAAAAAGATCCAT	280	P	A	A	NA
PMS3407	Os_Chr06	14581694	14581711	(CT)9	(CT)8	(CT)9	(CT)9	(CT)9	(CT)9	(CT)9	(CT)9	(CT)9	(CT)9	(CT)9	-	Introns/Intergenic	-	-	GTCCCAGATTATCCCCACCT	TTAGCTACGTACACACGCCG	261	P	A	A	NA
PMS3408	Os_Chr06	15089494	15089511	(CT)9	(CT)9	(CT)9	(CT)9	(CT)9	(CT)8	(CT)9	(CT)9	(CT)9	(CT)7	(CT)9	-	Introns/Intergenic	-	-	TAAAAAGTCCCCCTTCCCTC	CCCCACTTTATTGCACGC	268	P	A	A	NA
PMS3409	Os_Chr06	15133246	15133277	(TA)16	(TA)16	(TA)10	(TA)9	(TA)16	(TA)16	(TA)16	(TA)13	(TA)16	(TA)13	(TA)16	-	Introns/Intergenic	-	-	ATATGAATCAGTGCGTGTGCGTTGG	CACAAACAGGCACCATGTTGAGC	471	P	A	A	RM20012
PMS3410	Os_Chr06	15256406	15256431	(TA)13	(TA)13	(TA)13	(TA)13	(TA)13	(TA)13	(TA)13	(TA)13	(TA)7	(TA)13	(TA)7	-	Introns/Intergenic	-	-	GGTAAACCTGCATACTTCTCTCTCC	ACACAGGAAGCACAATTCTACGC	144	A	A	A	RM20013
PMS3411	Os_Chr06	15402555	15402572	(TCG)6	(TCG)6	(TCG)6	(TCG)6	(TCG)6	(TCG)6	(TCG)6	(TCG)6	(TCG)6	(TCG)6	(TCG)5	-	Introns/Intergenic	-	-	CGTTTCGTGCGATGACTG	AGATCGACTTGACGGAGGAA	256	A	A	A	NA
PMS3412	Os_Chr06	15828204	15828217	(TC)7	(TC)7	(TC)7	(TC)7	(TC)7	(TC)7	(TC)7	(TC)7	(TC)7	(TC)7	(TC)6	-	Introns/Intergenic	-	-	GTCGTCGGTGAGGGACTTT	TACCCTCGGGTAGATCGGAC	214	A	A	A	NA
PMS3413	Os_Chr06	15838270	15838349	(AT)40	(AT)40	(AT)40	(AT)40	(AT)40	(AT)8	(AT)6	(AT)40	(AT)40	(AT)8	(AT)40	-	Introns/Intergenic	-	-	CTGACCTGACGGCTGACATGACC	CAAGCAACCTTTCGGGATTTGC	135	P	A	A	RM20023
PMS3414	Os_Chr06	15855793	15855816	(CAT)8	(CAT)8	(CAT)8	(CAT)8	(CAT)5	(CAT)8	(CAT)8	(CAT)8	(CAT)8	(CAT)8	(CAT)8	-	Introns/Intergenic	-	-	ACTTTTGCGGGTTTTAGGCT	CGGCCGACTGGATCTAATAA	213	P	A	A	NA
PMS3415	Os_Chr06	15862895	15862919	(ATTTT)5	(ATTTT)5	(ATTTT)5	(ATTTT)5	(ATTTT)6	(ATTTT)5	(ATTTT)5	(ATTTT)5	(ATTTT)5	(ATTTT)6	(ATTTT)5	-	Introns/Intergenic	-	-	CACACAATACATCATTTGTTTTCG	TTTCGACAGGGCTTGAAAGT	176	P	A	A	NA
PMS3416	Os_Chr06	15988355	15988378	(TA)12	(TA)12	(TA)12	(TA)12	(TA)12	(TA)12	(TA)12	(TA)12	(TA)6	(TA)12	(TA)6	-	Introns/Intergenic	-	-	AAAATTTGAGTGATTTTGTGCATT	GTTTAGTTATTGCGCACCCC	188	A	A	A	NA
PMS3417	Os_Chr06	16036013	16036064	(AT)26	(AT)26	(AT)26	(AT)26	(AT)18	(AT)26	(AT)26	(AT)9	(AT)26	(AT)25	(AT)20	-	Introns/Intergenic	-	-	GCTTGCTGCCTGTTAATACACTATGC	GCTTGGTAGTTGGGCTGATGG	173	P	A	A	RM20039
PMS3418	Os_Chr06	16041171	16041236	(TA)33	(TA)33	(TA)33	(TA)33	(TA)33	(TA)33	(TA)33	(TA)33	(TA)24	(TA)33	(TA)33	-	Introns/Intergenic	-	-	TATGTTCCGCTTTTTACGGA	TGTTGAACTGCTCCATCGAC	275	A	A	A	NA

Polymorphic SSR (PMS) markers	Chromosomes	Start physical positions (bp)	End physical positions (bp)	Nipponbare	Kasalath	Nagina22	IR64	Pokkali	Bala	Tainung67	Azucena	Moroberekan	*Oryza rufipogon*	*Oryza nivara*	MSU gene locus IDs	Structural annotation	Functional annotation	SSR functional effects	Forward primers (5'-3')	Reverse primers (5'-3')	Amplified product size (bp)	Markers exhibiting polymorphism within *indica* rice	Transcrption factor genes-derived markers	Known cloned genes-derived markers	PMS markers corresponding with RM markers
PMS3419	Os_Chr06	16134819	16134840	(TA)11	(TA)11	(TA)11	(TA)11	(TA)8	(TA)11	(TA)11	(TA)11	(TA)11	(TA)11	(TA)11	-	Introns/Intergenic	-	-	TCGACGAACAGGTCCTTGATCC	CTGGCTGGCTGAGCTATCTTTAGTCC	392	P	A	A	RM20053
PMS3420	Os_Chr06	16346075	16346090	(CT)8	(CT)8	(CT)8	(CT)8	(CT)7	(CT)8	(CT)8	(CT)8	(CT)8	(CT)8	(CT)8	-	Introns/Intergenic	-	-	AGCAATCATCAGCCTTGCTT	GGAGCATGAAAATCAGGGAA	255	P	A	A	NA
PMS3421	Os_Chr06	16476030	16476092	(ATA)21	(ATA)21	(ATA)21	(ATA)21	(ATA)21	(ATA)21	(ATA)21	(ATA)21	(ATA)6	(ATA)21	(ATA)19	-	Introns/Intergenic	-	-	AAGAGATGCATGCGCTAAATGG	CTGGTATGATCTCCGACTCACTAAGC	349	A	A	A	RM20066
PMS3422	Os_Chr06	16509529	16509549	(CCA)7	(CCA)7	(CCA)7	(CCA)6	(CCA)7	(CCA)7	(CCA)5	(CCA)7	(CCA)7	(CCA)7	(CCA)7	LOC_Os06g28970	CDS	bolA, putative, expressed	CODON_CHANGE_PLUS_CODON_DELETION	ACCTCCTACTCCTCCTCCTGTGG	CGCCAAGATTAGAAGCAAGAAGC	255	P	A	A	RM20067
PMS3423	Os_Chr06	16645137	16645150	(AC)7	(AC)8	(AC)8	(AC)7	(AC)8	(AC)8	(AC)7	(AC)7	(AC)7	(AC)8	(AC)8	-	Introns/Intergenic	-	-	GGTCGTATTCAGCCCACTTG	TCCTGACCATGCCTACAAAA	272	P	A	A	NA
PMS3424	Os_Chr06	16890043	16890087	(TAT)15	(TAT)15	(TAT)15	(TAT)7	(TAT)15	(TAT)15	(TAT)15	(TAT)6	(TAT)15	(TAT)15	(TAT)15	-	Introns/Intergenic	-	-	TTTCTATGTGGGCCATTTCTC	GCACATGTAGAAATACAATTTAGAACC	247	P	A	A	NA
PMS3425	Os_Chr06	16963111	16963124	(TA)7	(TA)6	(TA)6	(TA)7	(TA)7	(TA)6	(TA)7	(TA)7	(TA)7	(TA)7	(TA)7	-	Introns/Intergenic	-	-	TCCTTTCTGCTCAAATGCCT	AAAGTTGCTGTTGGACTCCC	253	P	A	A	NA
PMS3426	Os_Chr06	17028177	17028192	(AG)8	(AG)7	(AG)8	(AG)8	(AG)8	(AG)8	(AG)8	(AG)8	(AG)8	(AG)8	(AG)8	-	Introns/Intergenic	-	-	ATGTGTCATTCGCACCACAG	GGCCCATGTAGATGCTTCCT	168	P	A	A	NA
PMS3427	Os_Chr06	17214414	17214447	(AT)17	(AT)17	(AT)10	(AT)6	(AT)17	(AT)9	(AT)17	(AT)17	(AT)8	(AT)17	(AT)17	-	Introns/Intergenic	-	-	CTCCCTCCGTTTCACAATCT	CCCCTCCATTTCACAATGTT	174	P	A	A	NA
PMS3428	Os_Chr06	17485738	17485751	(AT)7	(AT)10	(AT)7	(AT)7	(AT)7	(AT)7	(AT)7	(AT)7	(AT)7	(AT)7	(AT)7	-	Introns/Intergenic	-	-	ATTGCTCCACAAGGAATTGG	GAGGGAGTTCTGGCGTACCT	246	P	A	A	NA
PMS3429	Os_Chr06	17518710	17518729	(CT)10	(CT)10	(CT)10	(CT)10	(CT)10	(CT)10	(CT)9	(CT)8	(CT)10	(CT)10	(CT)10	-	Introns/Intergenic	-	-	GTAGTGGCAGCAGGGGATAA	GCTATGCCAGCTCTCCTGAC	134	A	A	A	NA
PMS3430	Os_Chr06	17527412	17527445	(AT)17	(AT)17	(AT)17	(AT)17	(AT)17	(AT)17	(AT)17	(AT)17	(AT)6	(AT)17	(AT)9	-	Introns/Intergenic	-	-	CCTACAAGCCGGTCAGACTGC	CATGCCAACCAGAGTCCAAAGC	383	A	A	A	RM20101
PMS3431	Os_Chr06	17631290	17631311	(TA)11	(TA)11	(TA)9	(TA)8	(TA)11	(TA)11	(TA)11	(TA)11	(TA)11	(TA)11	(TA)11	-	Introns/Intergenic	-	-	TTTGTCCTTGTAGGCTTCCCTAACC	CTTCATGCTGCTATCTTCATGTCACG	242	P	A	A	RM20102

Polymorphic SSR (PMS) markers	Chromosomes	Start physical positions (bp)	End physical positions (bp)	Nipponbare	Kasalath	Nagina22	IR64	Pokkali	Bala	Tainung67	Azucena	Moroberekan	*Oryza rufipogon*	*Oryza nivara*	MSU gene locus IDs	Structural annotation	Functional annotation	SSR functional effects	Forward primers (5'-3')	Reverse primers (5'-3')	Amplified product size (bp)	Markers exhibiting polymorphism within *indica* rice	Transcrption factor genes-derived markers	Known cloned genes-derived markers	PMS markers corresponding with RM markers
PMS3432	Os_Chr06	17653649	17653668	(TA)10	(TA)10	(TA)10	(TA)10	(TA)10	(TA)10	(TA)9	(TA)10	(TA)10	(TA)10	(TA)10	-	Introns/Intergenic	-	-	CCACTAACCTGATCCAATAAGC	GGTGCAGCAGAATTATAAGAGG	233	A	A	A	RM20103
PMS3433	Os_Chr06	17753695	17753708	(GA)7	(GA)10	(GA)7	(GA)7	(GA)7	(GA)7	(GA)7	(GA)7	(GA)7	(GA)7	(GA)7	-	Introns/Intergenic	-	-	GCCCGTCGGTGTTCTCAC	CCCACCTGTCATCTCCTCAT	192	P	A	A	NA
PMS3434	Os_Chr06	17753966	17753999	(AT)17	(AT)17	(AT)17	(AT)17	(AT)17	(AT)9	(AT)17	(AT)17	(AT)17	(AT)17	(AT)17	-	Introns/Intergenic	-	-	GGGGGTAATTTGTTCGGTTT	CTCTTGGTCCAGGTGGTTGT	194	P	A	A	NA
PMS3435	Os_Chr06	17761558	17761571	(AT)7	(AT)7	(AT)7	(AT)7	(AT)7	(AT)7	(AT)7	(AT)7	(AT)6	(AT)7	(AT)7	-	Introns/Intergenic	-	-	CCCTGTCGTCGAGACTGTTT	GGGAGATGATGCATGACACTT	262	A	A	A	NA
PMS3436	Os_Chr06	17795478	17795493	(TA)8	(TA)8	(TA)8	(TA)8	(TA)8	(TA)8	(TA)8	(TA)8	(TA)8	(TA)7	(TA)8	-	Introns/Intergenic	-	-	GATGGATGACCACACCACAC	ACTCCCTCCGTTTCACAATG	174	A	A	A	NA
PMS3437	Os_Chr06	17812189	17812203	(CCG)5	(CCG)7	(CCG)5	(CCG)5	(CCG)5	(CCG)5	(CCG)5	(CCG)5	(CCG)7	(CCG)5	(CCG)5	LOC_Os06g30710	URR	DGCR14, putative, expressed	-	GACCCAATCACAAACCAACC	CTTGGAGGAGGTGGTGGTAG	279	P	A	A	NA
PMS3438	Os_Chr06	17874312	17874325	(GA)7	(GA)6	(GA)7	(GA)7	(GA)7	(GA)7	(GA)6	(GA)7	(GA)7	(GA)7	(GA)7	-	Introns/Intergenic	-	-	AAGGGGGAAATATCAGAGGG	CTTGGATGGAGAAATGACCAA	215	P	A	A	NA
PMS3439	Os_Chr06	17937494	17937511	(TGG)6	(TGG)6	(TGG)5	(TGG)5	(TGG)5	(TGG)5	(TGG)6	(TGG)6	(TGG)6	(TGG)6	(TGG)5	LOC_Os06g30880	CDS	expressed protein	CODON_CHANGE_PLUS_CODON_DELETION	TAGGGGCCTAGGTGACAATG	CTCCATTGCCATATCACACG	236	P	A	A	NA
PMS3440	Os_Chr06	18050612	18050626	(TGG)5	(TGG)5	(TGG)5	(TGG)5	(TGG)5	(TGG)5	(TGG)5	(TGG)5	(TGG)5	(TGG)6	(TGG)5	LOC_Os06g31020	CDS	retrotransposon protein, putative, Ty3-gypsy subclass, expressed	CODON_INSERTION	GCATTCATCATGTTGGCTTG	CTGGTTCTGTGTGGGGTTTT	196	A	A	A	NA
PMS3441	Os_Chr06	18099174	18099193	(CT)10	(CT)10	(CT)10	(CT)10	(CT)10	(CT)10	(CT)10	(CT)9	(CT)10	(CT)10	(CT)10	-	Introns/Intergenic	-	-	TAACCTCCCGCCGCCTAGTTCC	GCCGGGAAAGCCTCCTATTTGG	246	A	A	A	RM20114
PMS3442	Os_Chr06	18110738	18110753	(CT)8	(CT)8	(CT)8	(CT)8	(CT)8	(CT)8	(CT)8	(CT)8	(CT)8	(CT)8	(CT)6	-	Introns/Intergenic	-	-	ACCTGTCGGTCGTCCCTAAC	GCCAGGAAAGCCTCCTATTT	255	A	A	A	NA
PMS3443	Os_Chr06	18121114	18121143	(CT)15	(CT)12	(CT)15	(CT)15	(CT)15	(CT)12	(CT)15	(CT)15	(CT)12	(CT)15	(CT)15	LOC_Os06g31190	URR	intracellular protein transport protein USO1 related, putative, expressed	-	CTGGCTGGCTTCTTCTTCTTCC	CGACAGACAGAACAGCACAAGG	154	P	A	A	RM20115
PMS3444	Os_Chr06	18157880	18157899	(GA)10	(GA)10	(GA)10	(GA)10	(GA)10	(GA)10	(GA)10	(GA)10	(GA)10	(GA)8	(GA)10	-	Introns/Intergenic	-	-	GCTCTGCTTCCGCCTCTG	TCCCCTCCTCTTCACCTTTT	209	A	A	A	NA

Polymorphic SSR (PMS) markers	Chromosomes	Start physical positions (bp)	End physical positions (bp)	Nipponbare	Kasalath	Nagina22	IR64	Pokkali	Bala	Tainung67	Azucena	Moroberekan	*Oryza rufipogon*	*Oryza nivara*	MSU gene locus IDs	Structural annotation	Functional annotation	SSR functional effects	Forward primers (5'-3')	Reverse primers (5'-3')	Amplified product size (bp)	Markers exhibiting polymorphism within *indica* rice	Transcrption factor genes-derived markers	Known cloned genes-derived markers	PMS markers corresponding with RM markers
PMS3445	Os_Chr06	18483030	18483057	(AT)14	(AT)8	(AT)14	(AT)14	(AT)14	(AT)14	(AT)14	(AT)14	(AT)9	(AT)14	(AT)9	-	Introns/Intergenic	-	-	GGCCGAGCTTGACTGTGTAT	TCTAAGCATGGCTAAGCAATCA	250	P	A	A	NA
PMS3446	Os_Chr06	18494719	18494739	(GCA)7	(GCA)7	(GCA)7	(GCA)7	(GCA)7	(GCA)5	(GCA)7	(GCA)7	(GCA)7	(GCA)7	(GCA)5	LOC_Os06g31840	CDS	retrotransposon protein, putative, Ty3-gypsy subclass, expressed	CODON_DELETION	ATTTCCAGCGTTTGGTTGAC	TTGCTGACTCTGCTGTTGCT	242	P	A	A	NA
PMS3447	Os_Chr06	18550968	18550985	(AT)9	(AT)6	(AT)9	(AT)9	(AT)9	(AT)9	(AT)9	(AT)9	(AT)7	(AT)9	(AT)9	-	Introns/Intergenic	-	-	TCAAGCTTCTACCGAGCCTG	CGAGCTCGGCCATAAGTAGT	271	P	A	A	NA
PMS3448	Os_Chr06	18597764	18597787	(AT)12	(AT)10	(AT)12	(AT)12	(AT)12	(AT)12	(AT)12	(AT)12	(AT)12	(AT)12	(AT)12	-	Introns/Intergenic	-	-	GGGCGAGCTTGACTGTGTAT	CGAGCTCGGCCATAAGTAGT	252	P	A	A	NA
PMS3449	Os_Chr06	18608692	18608705	(CG)7	(CG)7	(CG)7	(CG)6	(CG)7	(CG)7	(CG)7	(CG)7	(CG)7	(CG)7	(CG)7	-	Introns/Intergenic	-	-	GGCCCAAAACCTCTCTTCTC	ACGGAGATTGTTTCCCCTCT	228	P	A	A	NA
PMS3450	Os_Chr06	18719458	18719473	(AT)8	(AT)6	(AT)8	(AT)8	(AT)8	(AT)8	(AT)8	(AT)8	(AT)8	(AT)8	(AT)8	-	Introns/Intergenic	-	-	GCCGAGCTTGACTGTGAATA	GGTTACCGAGCTCAGCCATA	253	P	A	A	NA
PMS3451	Os_Chr06	18775724	18775739	(AG)8	(AG)8	(AG)8	(AG)8	(AG)8	(AG)8	(AG)8	(AG)8	(AG)7	(AG)8	(AG)7	-	Introns/Intergenic	-	-	GATCTAGTTGATGGCAGGGC	GATAAAATCGAGCCGAACCA	207	A	A	A	NA
PMS3452	Os_Chr06	18806004	18806015	(TA)6	(TA)6	(TA)6	(TA)6	(TA)6	(TA)7	(TA)6	(TA)6	(TA)6	(TA)6	(TA)6	-	Introns/Intergenic	-	-	TTGATGAACGGTGGATGAGA	GAACTCATTGTGGGCCTGAT	115	P	A	A	NA
PMS3453	Os_Chr06	18915937	18915956	(GA)10	(GA)10	(GA)10	(GA)10	(GA)10	(GA)10	(GA)8	(GA)10	(GA)10	(GA)10	(GA)7	-	Introns/Intergenic	-	-	GGGTTCCTCGGGACTATCAG	AGATCGGACGTCCTAGATGC	208	A	A	A	NA
PMS3454	Os_Chr06	18966123	18966136	(AT)7	(AT)7	(AT)7	(AT)7	(AT)7	(AT)7	(AT)6	(AT)7	(AT)7	(AT)7	(AT)7	-	Introns/Intergenic	-	-	TACATACACTTCGCGGATGC	TACATATCCCGCAGGAAAGC	216	A	A	A	NA
PMS3455	Os_Chr06	19087093	19087116	(TGC)8	(TGC)8	(TGC)8	(TGC)8	(TGC)8	(TGC)8	(TGC)8	(TGC)8	(TGC)8	(TGC)7	(TGC)8	LOC_Os06g32820	CDS	retrotransposon protein, putative, Ty3-gypsy subclass, expressed	CODON_DELETION	GGCATTTATCCGCAAAGTGT	TCCGCTACACCAATCCCTAC	208	A	A	A	NA
PMS3456	Os_Chr06	19321084	19321113	(AT)15	(AT)15	(AT)15	(AT)15	(AT)15	(AT)7	(AT)15	(AT)15	(AT)15	(AT)15	(AT)15	-	Introns/Intergenic	-	-	ATCGCTGAGGAAGATGAGAATCG	AGGAGACGACCAAATTGACAAGG	301	P	A	A	RM20142
PMS3457	Os_Chr06	19400051	19400062	(CT)6	(CT)6	(CT)7	(CT)7	(CT)7	(CT)7	(CT)6	(CT)6	(CT)6	(CT)6	(CT)6	-	Introns/Intergenic	-	-	CAGGTTCTCCTCCGTTACATCG	TCATATCATCCAGATCGCATCC	98	P	A	A	RM20144

Polymorphic SSR (PMS) markers	Chromosomes	Start physical positions (bp)	End physical positions (bp)	Nipponbare	Kasalath	Nagina22	IR64	Pokkali	Bala	Tainung67	Azucena	Moroberekan	*Oryza rufipogon*	*Oryza nivara*	MSU gene locus IDs	Structural annotation	Functional annotation	SSR functional effects	Forward primers (5'-3')	Reverse primers (5'-3')	Amplified product size (bp)	Markers exhibiting polymorphism within *indica* rice	Transcrption factor genes-derived markers	Known cloned genes-derived markers	PMS markers corresponding with RM markers
PMS3458	Os_Chr06	19444306	19444329	(AG)12	(AG)13	(AG)12	(AG)12	(AG)12	(AG)12	(AG)12	(AG)12	(AG)13	(AG)12	(AG)12	-	Introns/Intergenic	-	-	GCCATGAGCTTGGTGACGTTGC	CTGGCGACTCCTCCGGTAATCG	491	P	A	A	RM20145
PMS3459	Os_Chr06	19500374	19500399	(CT)13	(CT)14	(CT)13	(CT)13	(CT)13	(CT)14	(CT)13	(CT)13	(CT)14	(CT)10	(CT)13	-	Introns/Intergenic	-	-	ACACCCCCTAAATTTGAGCC	CAGATCTGGCAGAGGGGAG	267	P	A	A	NA
PMS3460	Os_Chr06	19542082	19542107	(GA)13	(GA)13	(GA)13	(GA)13	(GA)13	(GA)13	(GA)13	(GA)13	(GA)12	(GA)13	(GA)14	-	Introns/Intergenic	-	-	GAGAAAATGACGTCGTGTCG	ACCGACGAATCAATTTTCCA	135	A	A	A	NA
PMS3461	Os_Chr06	19641114	19641134	(TGC)7	(TGC)7	(TGC)7	(TGC)6	(TGC)7	(TGC)7	(TGC)7	(TGC)7	(TGC)7	(TGC)7	(TGC)7	LOC_Os06g33750	CDS	retrotransposon protein, putative, Ty3-gypsy subclass, expressed	CODON_DELETION	CTCGAAGCAGTTGAAGCACA	TCCGCTACACCAATCCCTAC	227	P	A	A	NA
PMS3462	Os_Chr06	19749626	19749653	(GA)14	(GA)12	(GA)14	(GA)14	(GA)14	(GA)12	(GA)14	(GA)14	(GA)14	(GA)14	(GA)14	-	Introns/Intergenic	-	-	CCATCGAGGAAACCAAAGTAAAGG	TTCTCACGCACCTTAGTCAATGG	182	P	A	A	RM20163
PMS3463	Os_Chr06	19843432	19843445	(AG)7	(AG)8	(AG)8	(AG)9	(AG)9	(AG)8	(AG)7	(AG)7	(AG)8	(AG)7	(AG)7	-	Introns/Intergenic	-	-	AGGAGCTAGCCAAAAGACCC	TTCCTTTGATTGGCTCAAAA	261	P	A	A	NA
PMS3464	Os_Chr06	19903340	19903363	(AT)12	(AT)12	(AT)12	(AT)12	(AT)12	(AT)12	(AT)6	(AT)12	(AT)6	(AT)12	(AT)12	-	Introns/Intergenic	-	-	CCCAAGATAATATGATGGCTCTGG	CAGCTAGTGGCTGCTCCTTACC	289	A	A	A	RM20172
PMS3465	Os_Chr06	19967558	19967575	(CA)9	(CA)9	(CA)9	(CA)9	(CA)9	(CA)9	(CA)9	(CA)9	(CA)6	(CA)9	(CA)7	-	Introns/Intergenic	-	-	CTAGCTCGCGAAGCCTAGAA	CGTCTTCACGGTGATAGCAA	201	A	A	A	NA
PMS3466	Os_Chr06	19973051	19973062	(AC)6	(AC)8	(AC)8	(AC)8	(AC)8	(AC)8	(AC)6	(AC)6	(AC)8	(AC)6	(AC)8	-	Introns/Intergenic	-	-	ATCTGTCACTCTTGGGACGC	CTCCACATGATTTTGCATCG	180	A	A	A	NA
PMS3467	Os_Chr06	19981411	19981424	(TC)7	(TC)7	(TC)7	(TC)7	(TC)7	(TC)7	(TC)7	(TC)7	(TC)6	(TC)7	(TC)7	-	Introns/Intergenic	-	-	ATGACCACAGGACCATCTCC	CATGGTACGCAAACGACAAC	215	A	A	A	NA
PMS3468	Os_Chr06	20132214	20132245	(AT)16	(AT)16	(AT)16	(AT)16	(AT)16	(AT)16	(AT)6	(AT)16	(AT)7	(AT)16	(AT)16	-	Introns/Intergenic	-	-	CACCGGTGTAGCTAGTTCTGTGC	AGAAGACTGCAATCGGAGTGACG	364	A	A	A	RM20180
PMS3469	Os_Chr06	20138271	20138288	(GCC)6	(GCC)6	(GCC)6	(GCC)6	(GCC)6	(GCC)6	(GCC)6	(GCC)5	(GCC)6	(GCC)6	(GCC)6	LOC_Os06g34640	CDS	zinc finger, C3HC4 type domain containing protein, expressed	CODON_DELETION	GGCCATGACATCTCCAGTTC	GCCACCATCTCCACCACT	210	A	A	A	NA
PMS3470	Os_Chr06	20143981	20143998	(CAT)6	(CAT)5	(CAT)5	(CAT)5	(CAT)5	(CAT)5	(CAT)6	(CAT)6	(CAT)5	(CAT)6	(CAT)6	LOC_Os06g34650	URR	zinc finger, C3HC4 type domain containing protein, expressed	-	AAATTTGGCTCCTCCAGCTT	GAGGTTGTCGGGTGACATCT	144	A	A	A	NA

Polymorphic SSR (PMS) markers	Chromosomes	Start physical positions (bp)	End physical positions (bp)	Nipponbare	Kasalath	Nagina22	IR64	Pokkali	Bala	Tainung67	Azucena	Moroberekan	*Oryza rufipogon*	*Oryza nivara*	MSU gene locus IDs	Structural annotation	Functional annotation	SSR functional effects	Forward primers (5'-3')	Reverse primers (5'-3')	Amplified product size (bp)	Markers exhibiting polymorphism within *indica* rice	Transcrption factor genes-derived markers	Known cloned genes-derived markers	PMS markers corresponding with RM markers
PMS3471	Os_Chr06	20147941	20147958	(GAG)6	(GAG)6	(GAG)6	(GAG)6	(GAG)6	(GAG)6	(GAG)6	(GAG)6	(GAG)6	(GAG)5	(GAG)6	LOC_Os06g34660	CDS	X8 domain containing protein, expressed	CODON_DELETION	GTCGTCCGCGTTGTTCTT	AGCTCGCTCGCTCTCACTAC	199	A	A	A	NA
PMS3472	Os_Chr06	20298220	20298247	(AT)14	(AT)14	(AT)14	(AT)14	(AT)14	(AT)13	(AT)14	(AT)14	(AT)14	(AT)14	(AT)14	-	Introns/Intergenic	-	-	AATAACTAGCCAACGTGAGTCC	TAATGGCCTTCCATCTATCC	187	P	A	A	RM20184
PMS3473	Os_Chr06	20319255	20319276	(TA)11	(TA)10	(TA)11	(TA)11	(TA)11	(TA)11	(TA)11	(TA)11	(TA)11	(TA)8	(TA)11	-	Introns/Intergenic	-	-	AATACGTAGTGGTGGGTGGG	ATTTCATACCATGTTGCCCG	168	P	A	A	NA
PMS3474	Os_Chr06	20319676	20319769	(AT)47	(AT)47	(AT)47	(AT)47	(AT)47	(AT)47	(AT)9	(AT)47	(AT)47	(AT)37	(AT)47	-	Introns/Intergenic	-	-	TCAATTTCAACAATGGTGCC	GACCTTTGACTGAGGTGGGA	242	A	A	A	NA
PMS3475	Os_Chr06	20322134	20322209	(TA)38	(TA)38	(TA)38	(TA)38	(TA)38	(TA)38	(TA)8	(TA)12	(TA)38	(TA)12	(TA)38	-	Introns/Intergenic	-	-	AGAAAAAGGGAGAGGAGGGG	GTGCATGAGTGCACCAAGAT	255	A	A	A	NA
PMS3476	Os_Chr06	20374047	20374061	(GCG)5	(GCG)6	(GCG)5	(GCG)5	(GCG)5	(GCG)5	(GCG)5	(GCG)5	(GCG)6	(GCG)5	(GCG)5	LOC_Os06g35030	CDS	RNA recognition motif containing protein, putative, expressed	CODON_INSERTION	AGCTCCGTTCTCTCCTCTCC	CGTCGCTCTTGTACTCCCTC	268	P	A	A	NA
PMS3477	Os_Chr06	20395290	20395307	(CT)9	(CT)9	(CT)9	(CT)9	(CT)9	(CT)9	(CT)9	(CT)9	(CT)7	(CT)9	(CT)9	-	Introns/Intergenic	-	-	GCTCGGCCCATCTCTTCC	CCTTCCGCGTCCACGAAC	271	A	A	A	NA
PMS3478	Os_Chr06	20415290	20415391	(AT)51	(AT)51	(AT)51	(AT)51	(AT)51	(AT)51	(AT)51	(AT)51	(AT)8	(AT)51	(AT)9	-	Introns/Intergenic	-	-	GTGGACCCACTACACAACATGG	TGTTCTACACTACGCCATTAGGC	480	A	A	A	RM20196
PMS3479	Os_Chr06	20756753	20756766	(GA)7	(GA)7	(GA)7	(GA)7	(GA)7	(GA)7	(GA)7	(GA)7	(GA)7	(GA)7	(GA)6	LOC_Os06g35570	CDS	expressed protein	FRAME_SHIFT	CCATCACTGGCCACCTAGTC	GGGACCCACCTCTCTACCTC	100	A	A	A	NA
PMS3480	Os_Chr06	20787893	20787904	(AG)6	(AG)6	(AG)6	(AG)6	(AG)6	(AG)6	(AG)6	(AG)7	(AG)6	(AG)6	(AG)6	-	Introns/Intergenic	-	-	ACATGAACCTCCACTGCCAC	TTAGTTTTGGGACCCGACAG	210	A	A	A	NA
PMS3481	Os_Chr06	20789561	20789584	(TC)12	(TC)12	(TC)12	(TC)12	(TC)12	(TC)12	(TC)12	(TC)10	(TC)12	(TC)6	(TC)12	-	Introns/Intergenic	-	-	ATGATAATGTGTGCGGGTCC	ATACCAAATTAGCCCCTCCG	212	A	A	A	NA
PMS3482	Os_Chr06	20869300	20869332	(CTC)11	(CTC)11	(CTC)11	(CTC)11	(CTC)11	(CTC)11	(CTC)11	(CTC)11	(CTC)11	(CTC)11	(CTC)10	LOC_Os06g35770	CDS	expressed protein	CODON_DELETION	AGAAGGTGAACCCGAGGTCTTGC	CCCTCTTCTCCTGCTGCTGTTGC	412	A	A	A	RM20215
PMS3483	Os_Chr06	20926821	20926844	(GA)12	(GA)12	(GA)9	(GA)12	(GA)12	(GA)12	(GA)12	(GA)12	(GA)12	(GA)12	(GA)12	LOC_Os06g35860	CDS	expressed protein	CODON_DELETION	GCGGCTCTACGAGCTCCTCTACTGG	GAGCTTTGTCAGCGGATTTGTTCG	369	P	P	A	RM3187

Polymorphic SSR (PMS) markers	Chromosomes	Start physical positions (bp)	End physical positions (bp)	Nipponbare	Kasalath	Nagina22	IR64	Pokkali	Bala	Tainung67	Azucena	Moroberekan	*Oryza rufipogon*	*Oryza nivara*	MSU gene locus IDs	Structural annotation	Functional annotation	SSR functional effects	Forward primers (5'-3')	Reverse primers (5'-3')	Amplified product size (bp)	Markers exhibiting polymorphism within *indica* rice	Transcrption factor genes-derived markers	Known cloned genes-derived markers	PMS markers corresponding with RM markers
PMS3484	Os_Chr06	20927089	20927112	(GGGA)6	(GGGA)6	(GGGA)6	(GGGA)6	(GGGA)6	(GGGA)6	(GGGA)5	(GGGA)6	(GGGA)6	(GGGA)6	(GGGA)6	LOC_Os06g35860	CDS	expressed protein	FRAME_SHIFT	GCCCTTCTCATTGT CTTCACAGC	GCTCACAGTGGATGT CCCAACC	258	A	P	A	RM20219
PMS3485	Os_Chr06	20954807	20954818	(CT)6	(CT)6	(CT)6	(CT)6	(CT)6	(CT)6	(CT)6	(CT)6	(CT)9	(CT)6	(CT)7	-	Introns/Intergenic	-	-	TGGAGTGTTTTCCC CAAGAG	CTCCAACCCGCTCAG TAAAA	265	A	A	A	NA
PMS3486	Os_Chr06	21208369	21208380	(GA)6	(GA)7	(GA)6	(GA)6	(GA)6	(GA)7	(GA)6	(GA)6	(GA)6	(GA)6	(GA)6	LOC_Os06g36210	URR	amino acid transporter, putative, expressed	-	CTCCTATTCCCTCT CCCGAC	TAGACGTCCATAGCT GACGC	269	P	A	A	NA
PMS3487	Os_Chr06	21352788	21352801	(CT)7	(CT)7	(CT)7	(CT)7	(CT)7	(CT)7	(CT)7	(CT)7	(CT)7	(CT)6	(CT)7	-	Introns/Intergenic	-	-	GGGTGGGATTGTTG TTTTTG	GGAGAGGAGAGGTG GGAGAG	185	A	A	A	NA
PMS3488	Os_Chr06	21420710	21420725	(AG)8	(AG)6	(AG)8	(AG)8	(AG)8	(AG)6	(AG)8	(AG)8	(AG)8	(AG)8	(AG)8	-	Introns/Intergenic	-	-	ACTTTGGAAGATGT GCAGGG	AGCTTGTGCAAGAAG GGAAA	218	P	A	A	NA
PMS3489	Os_Chr06	21424722	21424735	(CT)7	(CT)7	(CT)7	(CT)7	(CT)7	(CT)7	(CT)7	(CT)7	(CT)7	(CT)6	(CT)7	-	Introns/Intergenic	-	-	CTAGCAAAGTACCC GTGCGT	GGAGCAGCAGCTAG CATAGG	167	A	A	A	NA
PMS3490	Os_Chr06	21425017	21425030	(TC)7	(TC)6	(TC)7	(TC)7	(TC)7	(TC)6	(TC)7	(TC)7	(TC)6	(TC)7	(TC)6	-	Introns/Intergenic	-	-	TCGGATATTTTGAA CCGGAA	TCAACTGGATTAATT TTGCCTTT	105	P	A	A	NA
PMS3491	Os_Chr06	21455306	21455323	(GGC)6	(GGC)6	(GGC)6	(GGC)6	(GGC)6	(GGC)6	(GGC)6	(GGC)6	(GGC)5	(GGC)6	(GGC)6	-	Introns/Intergenic	-	-	ATCCCCTCTCTCGA AGCAAC	GTCGGATCTAGGTC GGGAG	273	A	A	A	NA
PMS3492	Os_Chr06	21539958	21539978	(CGA)7	(CGA)6	(CGA)6	(CGA)7	(CGA)6	(CGA)6	(CGA)7	(CGA)7	(CGA)6	(CGA)6	(CGA)7	LOC_Os06g36650	CDS	ABC transporter family protein, putative, expressed	CODON_CHANGE_PLUS_CODON_DELETION	AAACAGGACGACGT GACGAGAGC	GAAGCTTCCATCCTG GACATGG	90	P	A	A	RM20252
PMS3493	Os_Chr06	21565241	21565255	(CGC)5	(CGC)6	(CGC)5	(CGC)5	(CGC)5	(CGC)6	(CGC)5	(CGC)5	(CGC)6	(CGC)5	(CGC)5	LOC_Os06g36680	CDS	homeobox domain containing protein, expressed	CODON_INSERTION	CAGTGCTTCTGGAT GAACGA	GTACAGGAGGGTGA GGAGGC	135	P	P	A	NA
PMS3494	Os_Chr06	21589448	21589503	(TA)28	(TA)18	(TA)28	(TA)6	(TA)28	(TA)28	(TA)28	(TA)28	(TA)28	(TA)10	(TA)28	-	Introns/Intergenic	-	-	CTACGTCGTACTGA GACGAGAGC	TGGAATGGAATGACT TCACG	244	P	A	A	RM20256
PMS3495	Os_Chr06	21649387	21649404	(GCG)6	(GCG)6	(GCG)6	(GCG)6	(GCG)6	(GCG)6	(GCG)6	(GCG)5	(GCG)6	(GCG)6	(GCG)6	LOC_Os06g36750	CDS	expressed protein	CODON_DELETION	GGACTACACCATGG ACCAGC	CTCCCTTCCCCATCT CTCTC	144	A	A	A	NA
PMS3496	Os_Chr06	21802255	21802280	(AG)13	(AG)12	(AG)13	(AG)13	(AG)13	(AG)13	(AG)13	(AG)13	(AG)13	(AG)13	(AG)13	-	Introns/Intergenic	-	-	ATTGGAAATCAAGT GCTGCC	TGCTATTAAGCAATA ATGTGAGCC	187	P	A	A	NA

Polymorphic SSR (PMS) markers	Chromosomes	Start physical positions (bp)	End physical positions (bp)	Nipponbare	Kasalath	Nagina22	IR64	Pokkali	Bala	Tainung67	Azucena	Moroberekan	*Oryza rufipogon*	*Oryza nivara*	MSU gene locus IDs	Structural annotation	Functional annotation	SSR functional effects	Forward primers (5'-3')	Reverse primers (5'-3')	Amplified product size (bp)	Markers exhibiting polymorphism within *indica* rice	Transcrption factor genes-derived markers	Known cloned genes-derived markers	PMS markers corresponding with RM markers
PMS3497	Os_Chr06	21908743	21908756	(CG)7	(CG)6	(CG)7	(CG)7	(CG)7	(CG)6	(CG)7	(CG)7	(CG)7	(CG)7	(CG)7	LOC_Os06g37090	CDS	transposon protein, putative, unclassified, expressed	FRAME_SHIFT	GTACTGGCGAGTTACCAGGC	CTCGAAGCCGTTGACGAT	135	P	A	A	NA
PMS3498	Os_Chr06	21992531	21992574	(TA)22	(TA)22	(TA)8	(TA)22	(TA)22	(TA)22	(TA)6	(TA)22	(TA)22	(TA)22	(TA)13	LOC_Os06g37224	URR	OsKOS2|OsKOL5	-	ACAGGTTCACTGCGAGTTTGC	CCACACACTTGCTCCTTCTTCC	156	P	A	P	RM20270
PMS3499	Os_Chr06	22067368	22067385	(GAA)6	(GAA)5	(GAA)6	(GAA)6	(GAA)6	(GAA)6	(GAA)6	(GAA)6	(GAA)6	(GAA)6	(GAA)6	-	Introns/Intergenic	-	-	GAGGCCGAGAGCATCTACTG	AAGAATCTCTCCTCCCCCAA	157	P	A	A	NA
PMS3500	Os_Chr06	22179719	22179740	(AT)11	(AT)10	(AT)11	(AT)11	(AT)11	(AT)11	(AT)11	(AT)11	(AT)11	(AT)11	(AT)11	-	Introns/Intergenic	-	-	GGGAGTAGATGGCTTTGATAGTTGC	TACCATCCCATGCTCTCACTATAAGG	474	P	A	A	RM20275
PMS3501	Os_Chr06	22291013	22291027	(CAT)5	(CAT)5	(CAT)5	(CAT)5	(CAT)5	(CAT)5	(CAT)5	(CAT)6	(CAT)6	(CAT)6	(CAT)5	-	Introns/Intergenic	-	-	ATGTGCCGGTTTTCTCAATC	TGCGATTGTGTTTGGATGAT	185	A	A	A	NA
PMS3502	Os_Chr06	22300982	22300999	(GCT)6	(GCT)5	(GCT)6	(GCT)6	(GCT)6	(GCT)6	(GCT)6	(GCT)6	(GCT)6	(GCT)6	(GCT)6	LOC_Os06g37640	CDS	expressed protein	CODON_DELETION	AGAAGCCCTTCTCGACGAT	CCTAATCCTTCCCTTCCTCG	244	P	A	A	NA
PMS3503	Os_Chr06	22380461	22380524	(TA)32	(TA)32	(TA)8	(TA)32	(TA)32	(TA)32	(TA)32	(TA)32	(TA)32	(TA)32	(TA)32	-	Introns/Intergenic	-	-	AAGCATGCAATTCATCATTTT	TAGTTATGGTCCGTTTGCGA	165	P	A	A	NA
PMS3504	Os_Chr06	22425592	22425603	(GA)6	(GA)6	(GA)6	(GA)6	(GA)6	(GA)8	(GA)6	(GA)6	(GA)6	(GA)6	(GA)6	LOC_Os06g37870	CDS	expressed protein	FRAME_SHIFT	GGTGAAAATCGAACCACGAG	TCCCTTTTTCCCTTCTAGGC	157	P	A	A	NA
PMS3505	Os_Chr06	22459280	22459300	(GTC)7	(GTC)7	(GTC)7	(GTC)7	(GTC)7	(GTC)7	(GTC)7	(GTC)7	(GTC)7	(GTC)5	(GTC)7	-	Introns/Intergenic	-	-	GTGTTCCACGTCATCCTCGT	CTTGGACGAAGGCGAGAC	250	A	A	A	NA
PMS3506	Os_Chr06	22482594	22482613	(TC)10	(TC)10	(TC)10	(TC)10	(TC)10	(TC)10	(TC)10	(TC)10	(TC)10	(TC)10	(TC)8	-	Introns/Intergenic	-	-	CTAGTGTCCGTGCCATCTCA	GAGCTCGCTAGGGATGGAG	243	A	A	A	NA
PMS3507	Os_Chr06	22658873	22658902	(AT)15	(AT)15	(AT)7	(AT)7	(AT)7	(AT)7	(AT)8	(AT)15	(AT)6	(AT)7	(AT)6	-	Introns/Intergenic	-	-	AACTTGTTTGGTGGCGTACC	TTACCACGTCTCGTAAATGGC	204	P	A	A	NA
PMS3508	Os_Chr06	22912772	22912785	(CT)7	(CT)7	(CT)7	(CT)7	(CT)7	(CT)7	(CT)6	(CT)7	(CT)7	(CT)7	(CT)7	-	Introns/Intergenic	-	-	AGTGGAATTTTCAGAGCACAAA	GAGGAAGGCGATGACAGGT	251	A	A	A	NA
PMS3509	Os_Chr06	22967565	22967626	(TA)31	(TA)31	(TA)10	(TA)11	(TA)9	(TA)31	(TA)31	(TA)31	(TA)22	(TA)31	(TA)9	-	Introns/Intergenic	-	-	ATGGTGCCCCATATGAGTGT	TAGCTCTGAGCCGCTGATGT	214	P	A	A	NA

Polymorphic SSR (PMS) markers	Chromosomes	Start physical positions (bp)	End physical positions (bp)	Nipponbare	Kasalath	Nagina22	IR64	Pokkali	Bala	Tainung67	Azucena	Moroberekan	*Oryza rufipogon*	*Oryza nivara*	MSU gene locus IDs	Structural annotation	Functional annotation	SSR functional effects	Forward primers (5'-3')	Reverse primers (5'-3')	Amplified product size (bp)	Markers exhibiting polymorphism within *indica* rice	Transcrption factor genes-derived markers	Known cloned genes-derived markers	PMS markers corresponding with RM markers
PMS3510	Os_Chr06	23136727	23136747	(GCG)7	(GCG)7	(GCG)5	(GCG)7	(GCG)7	(GCG)7	(GCG)7	(GCG)7	(GCG)7	(GCG)7	(GCG)7	LOC_Os06g38970	DRR	expressed protein	-	ATAATTCATCCGGCCACCAACACC	CTCCGGGACAAGGTTGCTGAGG	158	P	A	A	RM20311
PMS3511	Os_Chr06	23163023	23163074	(AT)26	(AT)26	(AT)26	(AT)26	(AT)26	(AT)26	(AT)26	(AT)26	(AT)7	(AT)26	(AT)26	-	Introns/Intergenic	-	-	CTCGATCCCTTGTGTTTGTGTGC	TGGACTTGGATTGATTCCTACCG	148	A	A	A	RM20313
PMS3512	Os_Chr06	23202638	23202655	(CCG)6	(CCG)5	(CCG)6	(CCG)5	(CCG)6	(CCG)6	(CCG)5	(CCG)6	(CCG)6	(CCG)6	(CCG)6	-	Introns/Intergenic	-	-	TGCTTCAGCTCCAGTCCAAT	CACATGCCATCTTCTCCCTT	147	P	A	A	NA
PMS3513	Os_Chr06	23333943	23333957	(CGC)5	(CGC)5	(CGC)5	(CGC)5	(CGC)5	(CGC)5	(CGC)5	(CGC)5	(CGC)5	(CGC)6	(CGC)5	LOC_Os06g39330	CDS	UDP-glucoronosyl and UDP-glucosyl transferase domain containing protein, expressed	CODON_INSERTION	GCACCACTCCACCACCAC	GCCAAGTACATGGAGTGGCT	237	A	A	A	NA
PMS3514	Os_Chr06	23362310	23362333	(GAG)8	(GAG)8	(GAG)8	(GAG)8	(GAG)5	(GAG)7	(GAG)8	(GAG)8	(GAG)8	(GAG)8	(GAG)8	-	Introns/Intergenic	-	-	ACTGAAGCCACGCAAGAAGAGG	TATCAGCCCAAGAAAGCAAGTCC	275	P	A	A	RM20319
PMS3515	Os_Chr06	23381359	23381382	(GCT)8	(GCT)8	(GCT)8	(GCT)8	(GCT)6	(GCT)8	(GCT)8	(GCT)8	(GCT)8	(GCT)8	(GCT)8	LOC_Os06g39390	URR	OsAt10	-	ACCTTGGTGACGGCGAAGACG	CCCACCACACGCACACAGTTAGC	105	P	A	P	RM6446
PMS3516	Os_Chr06	23404856	23404870	(TTC)5	(TTC)5	(TTC)5	(TTC)6	(TTC)5	(TTC)5	(TTC)6	(TTC)5	(TTC)5	(TTC)5	(TTC)5	-	Introns/Intergenic	-	-	GCTGGTGGATTTCATCGTTT	CGAAGTGGTGGCATTATCCT	170	P	A	A	NA
PMS3517	Os_Chr06	23502178	23502198	(CTC)7	(CTC)7	(CTC)7	(CTC)7	(CTC)7	(CTC)7	(CTC)7	(CTC)7	(CTC)7	(CTC)5	(CTC)7	LOC_Os06g39590	CDS	OsIAA23	CODON_DELETION	ACCGCGCGCATCTTTATAGGC	AGAAACCCGCGGGAACGTAGG	398	A	A	P	RM20325
PMS3518	Os_Chr06	23556542	23556556	(CCG)5	(CCG)5	(CCG)5	(CCG)5	(CCG)5	(CCG)5	(CCG)6	(CCG)5	(CCG)5	(CCG)5	(CCG)5	LOC_Os06g39680	URR	transposon protein, putative, unclassified, expressed	-	TCCTTCGTTTCACTCCATCC	AGGGTTAACGAACGAACGTG	251	A	P	A	NA
PMS3519	Os_Chr06	23608824	23608841	(AG)9	(AG)9	(AG)9	(AG)9	(AG)9	(AG)9	(AG)7	(AG)9	(AG)9	(AG)9	(AG)9	LOC_Os06g39750	URR	WSL1	-	CGAATTTTCTCCCCCTCTTC	GAAGGGATCTTTCTCCGGTC	192	A	A	P	NA
PMS3520	Os_Chr06	23628505	23628520	(GT)8	(GT)8	(GT)8	(GT)8	(GT)7	(GT)8	(GT)8	(GT)7	(GT)7	(GT)8	(GT)8	-	Introns/Intergenic	-	-	CTAGCCGATCCACCATCATT	AGCTTCCACAAGGTGTGCTT	189	P	A	A	NA
PMS3521	Os_Chr06	23644971	23644994	(TAT)8	(TAT)8	(TAT)8	(TAT)8	(TAT)8	(TAT)6	(TAT)8	(TAT)8	(TAT)8	(TAT)8	(TAT)8	-	Introns/Intergenic	-	-	CGGCCATCTGTACTTCCTTTCC	GGTGTTGGAAATCTAGGGACTAATCG	321	P	A	A	RM20336
PMS3522	Os_Chr06	23649670	23649687	(GCG)6	(GCG)6	(GCG)6	(GCG)6	(GCG)6	(GCG)6	(GCG)6	(GCG)6	(GCG)6	(GCG)6	(GCG)5	LOC_Os06g39800	CDS	expressed protein	CODON_DELETION	CGGTAATTTCATAGACCGCC	CGTTCCTCCTGTGGCTACTC	133	A	A	A	NA

Polymorphic SSR (PMS) markers	Chromosomes	Start physical positions (bp)	End physical positions (bp)	Nipponbare	Kasalath	Nagina22	IR64	Pokkali	Bala	Tainung67	Azucena	Moroberekan	*Oryza rufipogon*	*Oryza nivara*	MSU gene locus IDs	Structural annotation	Functional annotation	SSR functional effects	Forward primers (5'-3')	Reverse primers (5'-3')	Amplified product size (bp)	Markers exhibiting polymorphism within *indica* rice	Transcrption factor genes-derived markers	Known cloned genes-derived markers	PMS markers corresponding with RM markers
PMS3523	Os_Chr06	23731602	23731622	(CGC)7	(CGC)7	(CGC)7	(CGC)7	(CGC)7	(CGC)7	(CGC)7	(CGC)7	(CGC)7	(CGC)5	(CGC)7	-	Introns/Intergenic	-	-	GAGCTCCTCGCCTTCCTC	ATACGTGGTCTTTGTTGGGC	155	A	A	A	NA
PMS3524	Os_Chr06	23737217	23737240	(GAA)8	(GAA)7	(GAA)8	(GAA)8	(GAA)8	(GAA)7	(GAA)8	(GAA)8	(GAA)8	(GAA)8	(GAA)8	LOC_Os06g39912	CDS	expressed protein	CODON_CHANGE_PLUS_CODON_DELETION	GAAGACGACGAAGCAGCAGAGG	GGTTGTCGTCCTCCTTCTGACC	77	P	A	A	RM6298
PMS3525	Os_Chr06	23921811	23921824	(TG)7	(TG)7	(TG)7	(TG)6	(TG)7	(TG)7	(TG)6	(TG)7	(TG)7	(TG)7	(TG)6	-	Introns/Intergenic	-	-	GTACGATCGAACGTGCTTCA	ACTTGACAAGGCTGGGAAGA	220	P	A	A	NA
PMS3526	Os_Chr06	23958501	23958521	(GCC)7	(GCC)6	(GCC)7	(GCC)7	(GCC)7	(GCC)6	(GCC)7	(GCC)7	(GCC)7	(GCC)7	(GCC)7	LOC_Os06g40220	CDS	OsFBX199 - F-box domain containing protein, expressed	CODON_DELETION	CAGTCGAGGATGTGGTGCTTGC	CTCGCCATCGATCATCGTCACC	384	P	A	A	RM20351
PMS3527	Os_Chr06	23965278	23965291	(AT)7	(AT)7	(AT)7	(AT)7	(AT)7	(AT)7	(AT)7	(AT)7	(AT)8	(AT)7	(AT)7	-	Introns/Intergenic	-	-	CATACATGCGTGCCTCAACT	AACGTGCCGATTATTTCCTG	229	A	A	A	NA
PMS3528	Os_Chr06	24037840	24037859	(AG)10	(AG)13	(AG)10	(AG)10	(AG)13	(AG)10	(AG)10	(AG)10	(AG)13	(AG)13	(AG)13	-	Introns/Intergenic	-	-	TTACCAGGCTTCCTCTCTTGACC	CCACGTCACCCAGAAACTAATCC	211	P	A	A	RM20356
PMS3529	Os_Chr06	24197477	24197526	(GA)25	(GA)25	(GA)9	(GA)25	(GA)25	(GA)25	(GA)25	(GA)21	(GA)25	(GA)25	(GA)10	-	Introns/Intergenic	-	-	CAGGTAAAGCGATGAGCAATTCG	AAGGAGTTGGCAACAGCGAAGG	266	P	A	A	RM20366
PMS3530	Os_Chr06	24434423	24434442	(TC)10	(TC)9	(TC)10	(TC)10	(TC)10	(TC)11	(TC)10	(TC)10	(TC)12	(TC)10	(TC)10	-	Introns/Intergenic	-	-	TCAGATCACTGTGCTCCAACTCC	TCTATCTATCATGCCGACCTTGC	192	P	A	A	RM20384
PMS3531	Os_Chr06	24463367	24463380	(CT)7	(CT)7	(CT)7	(CT)7	(CT)7	(CT)7	(CT)6	(CT)7	(CT)7	(CT)7	(CT)7	-	Introns/Intergenic	-	-	CCAATTCAGAGCGGTTTTGT	CCTGCACCACCCAGTGAC	151	A	A	A	NA
PMS3532	Os_Chr06	24507473	24507493	(CGC)7	(CGC)7	(CGC)7	(CGC)7	(CGC)7	(CGC)5	(CGC)5	(CGC)7	(CGC)7	(CGC)7	(CGC)7	LOC_Os06g41030	CDS	DUF1680 domain containing protein, putative, expressed	CODON_DELETION	CCCTGCGTGTCGTTGAACAGC	GGAATTAGGATGCGGCAATTTCACC	359	P	A	A	RM20386
PMS3533	Os_Chr06	24690340	24690395	(AT)28	(AT)28	(AT)28	(AT)9	(AT)19	(AT)28	(AT)28	(AT)28	(AT)28	(AT)28	(AT)10	-	Introns/Intergenic	-	-	AATTCAAGGATCTTCGGGAGTGG	AAATATCCGCCTGCGAAGATAGG	331	P	A	A	RM20399
PMS3534	Os_Chr06	24714899	24714938	(AT)20	(AT)20	(AT)20	(AT)14	(AT)20	(AT)20	(AT)20	(AT)20	(AT)20	(AT)20	(AT)11	-	Introns/Intergenic	-	-	CAACAACCCAAGTAACGATCTGC	GGCAAGAGAGTTAATCAGGAGAAAGC	264	P	A	A	RM20402
PMS3535	Os_Chr06	24727942	24727957	(CA)8	(CA)7	(CA)8	(CA)7	(CA)7	(CA)8	(CA)7	(CA)8	(CA)8	(CA)7	(CA)7	-	Introns/Intergenic	-	-	CGGCTTACGCATCAATTAGG	CGTGTCTCTGGCAGCACTAA	192	P	A	A	NA

Polymorphic SSR (PMS) markers	Chromosomes	Start physical positions (bp)	End physical positions (bp)	Nipponbare	Kasalath	Nagina22	IR64	Pokkali	Bala	Tainung67	Azucena	Moroberekan	*Oryza rufipogon*	*Oryza nivara*	MSU gene locus IDs	Structural annotation	Functional annotation	SSR functional effects	Forward primers (5'-3')	Reverse primers (5'-3')	Amplified product size (bp)	Markers exhibiting polymorphism within *indica* rice	Transcrption factor genes-derived markers	Known cloned genes-derived markers	PMS markers corresponding with RM markers
PMS3536	Os_Chr06	24770502	24770515	(GA)7	(GA)7	(GA)7	(GA)6	(GA)7	(GA)6	(GA)7	(GA)7	(GA)7	(GA)7	(GA)7	-	Introns/Intergenic	-	-	CGCCGAGGAGAGGAGTTC	CCAAATCCCTCCAACTCAAA	232	P	A	A	NA
PMS3537	Os_Chr06	24773549	24773594	(AT)23	(AT)23	(AT)23	(AT)7	(AT)7	(AT)7	(AT)15	(AT)23	(AT)23	(AT)23	(AT)6	-	Introns/Intergenic	-	-	GCATCACAGAGACAGAGACAATGG	CAAACCACACCATCACTCACTAGC	452	P	A	A	RM20403
PMS3538	Os_Chr06	24811889	24811904	(CT)8	(CT)10	(CT)8	(CT)8	(CT)8	(CT)10	(CT)8	(CT)8	(CT)8	(CT)8	(CT)8	LOC_Os06g41384	URR	zinc finger C-x8-C-x5-C-x3-H type family protein, expressed	-	GAAACCACGAAGAGGCTCAC	CCCGACCCGATTTAACCTAA	256	P	P	A	NA
PMS3539	Os_Chr06	24843886	24843909	(TC)12	(TC)9	(TC)12	(TC)12	(TC)12	(TC)12	(TC)12	(TC)12	(TC)12	(TC)12	(TC)12	LOC_Os06g41450	DRR	VQ domain containing protein, putative, expressed	-	TAACCCTTGCATACACATCACC	GTTCATCGAGGATGGATTTCG	250	P	A	A	RM5314
PMS3540	Os_Chr06	24920905	24920919	(GGC)5	(GGC)5	(GGC)5	(GGC)5	(GGC)5	(GGC)5	(GGC)5	(GGC)5	(GGC)5	(GGC)6	(GGC)5	LOC_Os06g41600	CDS	expressed protein	CODON_INSERTION	CACAGCAGCCAAGAAGAAGA	CCACAGCACGTAGAACACCA	261	A	A	A	NA
PMS3541	Os_Chr06	24924491	24924511	(CGG)7	(CGG)7	(CGG)7	(CGG)7	(CGG)7	(CGG)7	(CGG)7	(CGG)7	(CGG)6	(CGG)7	(CGG)7	LOC_Os06g41610	CDS	retrotransposon protein, putative, unclassified, expressed	CODON_DELETION	CAACAGCTCCAACAACAAGAATCG	TCCTGGACAGGGATTTAATACGC	194	A	A	A	RM20406
PMS3542	Os_Chr06	24938654	24938679	(TC)13	(TC)12	(TC)13	(TC)9	(TC)13	(TC)13	(TC)13	(TC)13	(TC)13	(TC)13	(TC)13	-	Introns/Intergenic	-	-	GTTCAGGGACCTTCGGTGTA	TGATGATTTACGTCAGCGAAA	171	P	A	A	NA
PMS3543	Os_Chr06	24945478	24945515	(AT)19	(AT)19	(AT)19	(AT)19	(AT)19	(AT)19	(AT)19	(AT)7	(AT)19	(AT)19	(AT)19	-	Introns/Intergenic	-	-	GGAGGTATTGGGAGTTTACAGAGA	TGGACACGATCTCCAAAACA	276	A	A	A	NA
PMS3544	Os_Chr06	24990605	24990620	(CA)8	(CA)8	(CA)7	(CA)8	(CA)8	(CA)7	(CA)8	(CA)8	(CA)8	(CA)6	(CA)8	-	Introns/Intergenic	-	-	TCATCCGAGGATTCAGGAAC	CTTGATGAGATGTAGCCGCA	248	P	A	A	NA
PMS3545	Os_Chr06	25007142	25007157	(GA)8	(GA)8	(GA)8	(GA)8	(GA)8	(GA)8	(GA)8	(GA)8	(GA)8	(GA)6	(GA)8	-	Introns/Intergenic	-	-	GGAGGTGCTAGGCTTGAGTG	GCCCCAACTTATTCCCAATC	276	A	A	A	NA
PMS3546	Os_Chr06	25020626	25020649	(CCT)8	(CCT)8	(CCT)8	(CCT)8	(CCT)8	(CCT)8	(CCT)7	(CCT)8	(CCT)8	(CCT)8	(CCT)8	LOC_Os06g41740	CDS	transposon protein, putative, CACTA, En/Spm sub-class, expressed	CODON_DELETION	TGAATTGACGAACGCCATTAGC	GTGGCAGAGGCGATGTAAGTGG	81	A	A	A	RM6071
PMS3547	Os_Chr06	25034568	25034585	(GCG)6	(GCG)6	(GCG)6	(GCG)6	(GCG)6	(GCG)6	(GCG)6	(GCG)5	(GCG)6	(GCG)6	(GCG)6	-	Introns/Intergenic	-	-	GATCTGAACCGCGCTTACTC	TCTCCTCCTCCTCGATTCAA	256	A	A	A	NA
PMS3548	Os_Chr06	25070983	25070997	(GCG)5	(GCG)5	(GCG)5	(GCG)5	(GCG)5	(GCG)5	(GCG)5	(GCG)5	(GCG)5	(GCG)7	(GCG)5	LOC_Os06g41800	URR	dihydroflavonol-4-reductase, putative, expressed	-	GATTCGATTCGATTGGTCGT	AGGGGACGAGGAGAGAAAAC	223	A	A	A	NA

Polymorphic SSR (PMS) markers	Chromosomes	Start physical positions (bp)	End physical positions (bp)	Nipponbare	Kasalath	Nagina22	IR64	Pokkali	Bala	Tainung67	Azucena	Moroberekan	*Oryza rufipogon*	*Oryza nivara*	MSU gene locus IDs	Structural annotation	Functional annotation	SSR functional effects	Forward primers (5'-3')	Reverse primers (5'-3')	Amplified product size (bp)	Markers exhibiting polymorphism within *indica* rice	Transcrption factor genes-derived markers	Known cloned genes-derived markers	PMS markers corresponding with RM markers
PMS3549	Os_Chr06	25072377	25072458	(TA)41	(TA)40	(TA)15	(TA)41	(TA)41	(TA)41	(TA)41	(TA)21	(TA)9	(TA)41	(TA)9	-	Introns/Intergenic	-	-	TGCCGGTAATTTAGTACGTCATCC	CACTGTGGGAGTTCGCTTGG	303	P	A	A	RM20412
PMS3550	Os_Chr06	25104792	25104803	(GT)6	(GT)6	(GT)6	(GT)6	(GT)6	(GT)6	(GT)6	(GT)6	(GT)6	(GT)8	(GT)6	-	Introns/Intergenic	-	-	TGCTTGTGTACTTGGCCTTG	CTCACCTCATGCTCAGTCCA	120	A	A	A	NA
PMS3551	Os_Chr06	25169215	25169236	(AT)11	(AT)12	(AT)11	(AT)11	(AT)11	(AT)11	(AT)11	(AT)11	(AT)11	(AT)11	(AT)11	-	Introns/Intergenic	-	-	GTCGTCGATCTGTTGTCTCTCG	GTCACAAACAATCAGCTGCAAGG	640	P	A	A	RM20418
PMS3552	Os_Chr06	25363683	25363700	(CGG)6	(CGG)5	(CGG)6	(CGG)6	(CGG)6	(CGG)5	(CGG)5	(CGG)6	(CGG)5	(CGG)6	(CGG)6	-	Introns/Intergenic	-	-	AGGGAAGACAGCAAGCTACG	GACTACCCACGAGGCAAATG	199	P	A	A	NA
PMS3553	Os_Chr06	25488563	25488588	(TA)13	(TA)13	(TA)13	(TA)11	(TA)13	(TA)13	(TA)13	(TA)13	(TA)12	(TA)13	(TA)13	-	Introns/Intergenic	-	-	GGACCTGTGTGCATAGGATACGC	ATTGAATTGGTGGCGGACAGG	113	P	A	A	RM20432
PMS3554	Os_Chr06	25571313	25571332	(ATAG)5	(ATAG)9	(ATAG)5	(ATAG)9	(ATAG)9	(ATAG)5	(ATAG)9	(ATAG)5	(ATAG)9	(ATAG)9	(ATAG)9	-	Introns/Intergenic	-	-	CACAACGAATGTGGTGTGTCC	CGACATAGATGAGGCCCTATTCC	127	P	A	A	RM20438
PMS3555	Os_Chr06	25671946	25671959	(GA)7	(GA)7	(GA)7	(GA)7	(GA)7	(GA)7	(GA)6	(GA)7	(GA)7	(GA)7	(GA)7	-	Introns/Intergenic	-	-	GCTTTGCTGCCATTACCAGT	CGGGAAATTGGACTTTGAGA	224	A	A	A	NA
PMS3556	Os_Chr06	25677399	25677422	(GCG)8	(GCG)8	(GCG)8	(GCG)8	(GCG)8	(GCG)8	(GCG)8	(GCG)8	(GCG)8	(GCG)5	(GCG)8	LOC_Os06g42690	CDS	bZIP transcription factor domain containing protein, expressed	CODON_CHANGE_PLUS_CODON_DELETION	GACGAGCTGTCGTCGCAC	TTTTAATCTTAATCCATTGGCG	254	A	A	A	NA
PMS3557	Os_Chr06	25681760	25681780	(CCA)7	(CCA)7	(CCA)7	(CCA)7	(CCA)7	(CCA)7	(CCA)7	(CCA)7	(CCA)7	(CCA)5	(CCA)6	LOC_Os06g42700	CDS	zinc finger, C3HC4 type domain containing protein, expressed	CODON_DELETION	GATCTCCCTCTCGTCGAGGAACC	GACCACGACGAAACCCTAGCC	318	A	A	A	RM20453
PMS3558	Os_Chr06	25736782	25736795	(AG)7	(AG)7	(AG)7	(AG)7	(AG)7	(AG)7	(AG)7	(AG)7	(AG)7	(AG)6	(AG)7	-	Introns/Intergenic	-	-	GCCACAGCAAAGAAAACCAT	CGTGTTGCTTTCCAAGATGA	164	A	A	A	NA
PMS3559	Os_Chr06	25900279	25900302	(GGT)8	(GGT)8	(GGT)8	(GGT)8	(GGT)8	(GGT)8	(GGT)8	(GGT)8	(GGT)8	(GGT)7	(GGT)8	LOC_Os06g43090	CDS	MYB transcription factor TaMYB1, putative, expressed	CODON_DELETION	GAGTTCTTGGAAGTGGCTGTGG	ACTGGAACTGCTCCCTCAAGC	371	A	P	A	RM20464
PMS3560	Os_Chr06	25904560	25904571	(AT)6	(AT)6	(AT)7	(AT)6	(AT)6	(AT)7	(AT)6	(AT)6	(AT)6	(AT)6	(AT)6	-	Introns/Intergenic	-	-	CTAATGAGCCGGTGGTTTGT	TCCGAGCTAGTTATGTCCTCA	231	P	A	A	NA
PMS3561	Os_Chr06	25970623	25970634	(AC)6	(AC)6	(AC)6	(AC)6	(AC)6	(AC)6	(AC)6	(AC)7	(AC)7	(AC)6	(AC)6	-	Introns/Intergenic	-	-	AAATGGGTCAAAAATGCTGC	TCCATCGATTTTTCCTCTGG	136	A	A	A	NA

Polymorphic SSR (PMS) markers	Chromosomes	Start physical positions (bp)	End physical positions (bp)	Nipponbare	Kasalath	Nagina22	IR64	Pokkali	Bala	Tainung67	Azucena	Moroberekan	*Oryza rufipogon*	*Oryza nivara*	MSU gene locus IDs	Structural annotation	Functional annotation	SSR functional effects	Forward primers (5'-3')	Reverse primers (5'-3')	Amplified product size (bp)	Markers exhibiting polymorphism within *indica* rice	Transcrption factor genes-derived markers	Known cloned genes-derived markers	PMS markers corresponding with RM markers
PMS3562	Os_Chr06	26156248	26156268	(GTC)7	(GTC)7	(GTC)7	(GTC)7	(GTC)7	(GTC)7	(GTC)7	(GTC)7	(GTC)7	(GTC)6	(GTC)7	LOC_Os06g43510	CDS	cytochrome P450 71D6, putative, expressed	CODON_DELETION	TTTGCAGGCGGTCAAATTAGG	CGAGGCGGAGGTACATGACG	347	A	A	A	RM20478
PMS3563	Os_Chr06	26198971	26198988	(TAT)6	(TAT)6	(TAT)6	(TAT)6	(TAT)6	(TAT)6	(TAT)6	(TAT)5	(TAT)5	(TAT)6	(TAT)6	-	Introns/Intergenic	-	-	ATGAAATACGGATCCAAGCG	CCCTGTTAGTGCTTCCTTCG	145	A	A	A	NA
PMS3564	Os_Chr06	26233608	26233621	(AG)7	(AG)7	(AG)7	(AG)7	(AG)7	(AG)7	(AG)7	(AG)7	(AG)7	(AG)6	(AG)7	-	Introns/Intergenic	-	-	GTTCTCTCGCGTACCACCTG	TTTATGACCAGCCTTCGCTT	216	A	A	A	NA
PMS3565	Os_Chr06	26314380	26314394	(CGC)5	(CGC)5	(CGC)5	(CGC)6	(CGC)5	(CGC)5	(CGC)5	(CGC)5	(CGC)5	(CGC)5	(CGC)5	LOC_Os06g43700	CDS	transmembrane amino acid transporter protein, putative, expressed	CODON_INSERTION	ATGACCAGGATGGAGACGAG	GCCAAGATCTCCAAGCAAGA	242	P	A	A	NA
PMS3566	Os_Chr06	26329829	26329842	(GC)7	(GC)7	(GC)7	(GC)7	(GC)7	(GC)7	(GC)6	(GC)7	(GC)7	(GC)7	(GC)7	-	Introns/Intergenic	-	-	GGTAAACCCGTTTTCCCTTC	AGCGGTGGATTATCCGTTTT	265	A	A	A	NA
PMS3567	Os_Chr06	26332983	26333003	(CCG)7	(CCG)8	(CCG)8	(CCG)7	(CCG)7	(CCG)8	(CCG)7	(CCG)7	(CCG)7	(CCG)7	(CCG)7	LOC_Os06g43760	URR	tRNA synthetase class I, putative, expressed	-	CACGTGAACTTCCAGCTCTAAACC	GCCCGTCGTAGAAGATGAACTCC	330	P	A	A	RM20482
PMS3568	Os_Chr06	26381229	26381244	(TA)8	(TA)8	(TA)8	(TA)8	(TA)8	(TA)8	(TA)6	(TA)8	(TA)8	(TA)8	(TA)8	-	Introns/Intergenic	-	-	TCAATTTCAACAATGGTGCC	CGTCCCATGAGAACAAGGAT	239	A	A	A	NA
PMS3569	Os_Chr06	26399537	26399554	(GAG)6	(GAG)6	(GAG)5	(GAG)6	(GAG)6	(GAG)6	(GAG)6	(GAG)6	(GAG)6	(GAG)6	(GAG)6	LOC_Os06g43840	URR	protein kinase domain containing protein, expressed	-	CTAACCTCGCAAGCAGCAG	ACAGCAGCTTCATCCTCTCC	279	P	A	A	NA
PMS3570	Os_Chr06	26462997	26463032	(TC)18	(TC)17	(TC)17	(TC)18	(TC)17	(TC)17	(TC)18	(TC)18	(TC)18	(TC)18	(TC)18	-	Introns/Intergenic	-	-	GAAGCTTGCACATGTGGATTACC	CTTTGCAGCTGATTTGGATTGG	208	P	A	A	RM20487
PMS3571	Os_Chr06	26479995	26480012	(GGC)6	(GGC)5	(GGC)5	(GGC)5	(GGC)5	(GGC)5	(GGC)6	(GGC)6	(GGC)6	(GGC)5	(GGC)6	-	Introns/Intergenic	-	-	CGTCCAACTGATACACGCAC	AGTTGATTTGCTGGAATGGC	157	A	A	A	NA
PMS3572	Os_Chr06	26483082	26483103	(GA)11	(GA)11	(GA)11	(GA)11	(GA)11	(GA)11	(GA)11	(GA)11	(GA)11	(GA)9	(GA)11	LOC_Os06g43960	URR	expressed protein	-	TGAACACAAGTGAGTTGCCA	TCAGTTGCGCTTTTGATTTG	271	A	A	A	NA
PMS3573	Os_Chr06	26526285	26526305	(GCG)7	(GCG)7	(GCG)5	(GCG)5	(GCG)7	(GCG)7	(GCG)7	(GCG)7	(GCG)7	(GCG)5	(GCG)7	-	Introns/Intergenic	-	-	AGATCAGATGCTGCTGGTCATGG	GTTTGATGGGCCTCATGTTTGG	309	P	A	A	RM20494
PMS3574	Os_Chr06	26526451	26526465	(GCG)5	(GCG)8	(GCG)5	(GCG)5	(GCG)5	(GCG)5	(GCG)5	(GCG)5	(GCG)5	(GCG)5	(GCG)5	-	Introns/Intergenic	-	-	GCCTCCCGGTAAATTCGG	GAACTGGGCCGTTGAAATTA	226	P	A	A	NA

Polymorphic SSR (PMS) markers	Chromosomes	Start physical positions (bp)	End physical positions (bp)	Nipponbare	Kasalath	Nagina22	IR64	Pokkali	Bala	Tainung67	Azucena	Moroberekan	*Oryza rufipogon*	*Oryza nivara*	MSU gene locus IDs	Structural annotation	Functional annotation	SSR functional effects	Forward primers (5'-3')	Reverse primers (5'-3')	Amplified product size (bp)	Markers exhibiting polymorphism within *indica* rice	Transcrption factor genes-derived markers	Known cloned genes-derived markers	PMS markers corresponding with RM markers
PMS3575	Os_Chr06	26571587	26571610	(CCG)8	(CCG)8	(CCG)8	(CCG)8	(CCG)8	(CCG)8	(CCG)8	(CCG)5	(CCG)8	(CCG)8	(CCG)8	LOC_Os06g44050	CDS	methyladenine glycosylase, putative, expressed	CODON_DELETION	CTCACCACTGGTCGGAGTCACC	ACCTCAACCTGTCGCTCAACG	253	A	A	A	RM20497
PMS3576	Os_Chr06	26721130	26721189	(TA)30	(TA)30	(TA)30	(TA)30	(TA)30	(TA)30	(TA)30	(TA)12	(TA)30	(TA)30	(TA)30	-	Introns/Intergenic	-	-	GCCTTTTACATGGTGCGATT	TGGAATGGTTGAATGGTTGA	209	A	A	A	NA
PMS3577	Os_Chr06	26796141	26796158	(CTC)6	(CTC)6	(CTC)6	(CTC)6	(CTC)6	(CTC)7	(CTC)6	(CTC)6	(CTC)6	(CTC)5	(CTC)6	-	Introns/Intergenic	-	-	TGACAATCTGCAAGACCCAA	ACATATCGCGAGATCGAACC	225	P	A	A	NA
PMS3578	Os_Chr06	26937529	26937546	(GGC)6	(GGC)6	(GGC)5	(GGC)6	(GGC)6	(GGC)6	(GGC)6	(GGC)6	(GGC)6	(GGC)6	(GGC)6	LOC_Os06g44610	CDS	membrane associated DUF588 domain containing protein, putative, expressed	CODON_CHANGE_PLUS_CODON_DELETION	TCCATCTCATCAAGCGATCA	CCGAGGAGAGAGATGTCGAG	266	P	A	A	NA
PMS3579	Os_Chr06	27107155	27107228	(TA)37	(TA)37	(TA)10	(TA)37	(TA)37	(TA)9	(TA)12	(TA)8	(TA)37	(TA)15	(TA)37	-	Introns/Intergenic	-	-	GACACGGTTTCCGATATACTGC	TACGTATAGTACGTGCCACATTGC	397	P	A	A	RM20514
PMS3580	Os_Chr06	27110744	27110757	(CT)7	(CT)7	(CT)7	(CT)7	(CT)7	(CT)7	(CT)7	(CT)7	(CT)7	(CT)6	(CT)7	-	Introns/Intergenic	-	-	GGCATGCAGACATTCCTGTT	CGCTGGAGGACAAGGTAGAG	196	A	A	A	NA
PMS3581	Os_Chr06	27162777	27162794	(GCC)6	(GCC)6	(GCC)6	(GCC)6	(GCC)6	(GCC)5	(GCC)6	(GCC)6	(GCC)6	(GCC)6	(GCC)6	LOC_Os06g44930	CDS	expressed protein	CODON_DELETION	ATCGACGCGAAGGTGATCTA	GGGAAGAGGATGACGAAGC	242	P	A	A	NA
PMS3582	Os_Chr06	27168409	27168434	(AT)13	(AT)13	(AT)13	(AT)13	(AT)13	(AT)13	(AT)6	(AT)13	(AT)7	(AT)13	(AT)13	-	Introns/Intergenic	-	-	ATCGTACGTGGAACCTAGACAACG	ACGACGACGATGTCCATTCTTACC	128	A	A	A	RM20536
PMS3583	Os_Chr06	27168558	27168569	(AC)6	(AC)6	(AC)6	(AC)7	(AC)7	(AC)6	(AC)6	(AC)6	(AC)6	(AC)6	(AC)6	-	Introns/Intergenic	-	-	AACGGAGCCATGAGCATATC	GCATGTTGTGTGGTCCTCTG	235	P	A	A	NA
PMS3584	Os_Chr06	27355057	27355070	(CG)7	(CG)6	(CG)7	(CG)7	(CG)7	(CG)7	(CG)7	(CG)7	(CG)7	(CG)7	(CG)7	LOC_Os06g45260	CDS	transposon protein, putative, unclassified, expressed	FRAME_SHIFT	GTGCTAGCGAGTTACCAGGC	AAAAGTTAAGATTGGCGGGG	273	P	A	A	NA
PMS3585	Os_Chr06	27403621	27403656	(GGC)12	(GGC)12	(GGC)12	(GGC)7	(GGC)5	(GGC)12	(GGC)12	(GGC)12	(GGC)12	(GGC)12	(GGC)12	LOC_Os06g45340	CDS	peptidyl-prolyl cis-trans isomerase, FKBP-type, putative, expressed	CODON_DELETION	GAAGTTTCACGCTGGGGTT	GTGTTTCTCTGGTTTGGGGA	173	P	A	A	NA
PMS3586	Os_Chr06	27449823	27449840	(AG)9	(AG)9	(AG)9	(AG)9	(AG)9	(AG)8	(AG)9	(AG)9	(AG)9	(AG)9	(AG)9	-	Introns/Intergenic	-	-	AGCGAGAGCCACCTAATCTTGG	CTGCTCTTCTCCACCACGAACC	261	P	A	A	RM3430
PMS3587	Os_Chr06	27487956	27487973	(GCG)6	(GCG)6	(GCG)6	(GCG)6	(GCG)6	(GCG)6	(GCG)6	(GCG)5	(GCG)6	(GCG)6	(GCG)6	-	Introns/Intergenic	-	-	AGAGAGACCACGGATGGCTA	TTTTCACTATTTCGCCGAGC	255	A	A	A	NA

**Polymorphic SSR (PMS) markers**	**Chromosomes**	**Start physical positions (bp)**	**End physical positions (bp)**	**Nipponbare**	**Kasalath**	**Nagina22**	**IR64**	**Pokkali**	**Bala**	**Tainung67**	**Azucena**	**Moroberekan**	***Oryza rufipogon***	***Oryza nivara***	**MSU gene locus IDs**	**Structural annotation**	**Functional annotation**	**SSR functional effects**	**Forward primers (5'-3')**	**Reverse primers (5'-3')**	**Amplified product size (bp)**	**Markers exhibiting polymorphism within *indica* rice**	**Transcrption factor genes-derived markers**	**Known cloned genes-derived markers**	**PMS markers corresponding with RM markers**
PMS3588	Os_Chr06	27530175	27530192	(CGC)6	(CGC)5	(CGC)5	(CGC)6	(CGC)6	(CGC)5	(CGC)6	(CGC)6	(CGC)6	(CGC)5	(CGC)6	LOC_Os06g45510	CDS	thioredoxin, putative, expressed	CODON_DELETION	CACAGTATGGCTCTGGCTCA	GAGTACCTGAGGAGGAGGGG	190	P	A	A	NA
PMS3589	Os_Chr06	27620349	27620366	(CGG)6	(CGG)6	(CGG)6	(CGG)6	(CGG)5	(CGG)6	(CGG)6	(CGG)6	(CGG)6	(CGG)6	(CGG)6	LOC_Os06g45620	CDS	expressed protein	CODON_DELETION	GGCGTAGGGGTTTCTCTTCT	CAACATCATCCTCAACACCG	277	P	A	A	NA
PMS3590	Os_Chr06	27661107	27661124	(GGC)6	(GGC)6	(GGC)6	(GGC)6	(GGC)6	(GGC)6	(GGC)6	(GGC)6	(GGC)6	(GGC)6	(GGC)5	LOC_Os06g45670	CDS	glycine cleavage system H protein, putative, expressed	CODON_DELETION	GGAGATCCGAAGGTAGGAGG	CCCAGTTTAGCCCAATTCAA	220	A	A	A	NA
PMS3591	Os_Chr06	27663426	27663445	(AG)10	(AG)9	(AG)10	(AG)10	(AG)10	(AG)10	(AG)10	(AG)10	(AG)10	(AG)10	(AG)10	-	Introns/Intergenic	-	-	GTCCTCCTCTCCCGCCTC	CCAAACGATGAGGGCATATC	133	P	A	A	NA
PMS3592	Os_Chr06	27733590	27733607	(CCG)6	(CCG)6	(CCG)5	(CCG)6	(CCG)6	(CCG)6	(CCG)6	(CCG)6	(CCG)6	(CCG)6	(CCG)6	LOC_Os06g45830	URR	expressed protein	-	CGTCCACCACCACCATTT	AAGAGAACACGCCATTTTGG	261	P	A	A	NA
PMS3593	Os_Chr06	27820087	27820104	(TC)9	(TC)9	(TC)6	(TC)9	(TC)9	(TC)9	(TC)9	(TC)9	(TC)9	(TC)9	(TC)9	-	Introns/Intergenic	-	-	AAACCAACCATGGAATGGAA	CGCTGGAATTCTTTCTCCTG	262	P	A	A	NA
PMS3594	Os_Chr06	27921828	27921881	(TA)27	(TA)23	(TA)27	(TA)6	(TA)27	(TA)27	(TA)27	(TA)18	(TA)6	(TA)27	(TA)27	-	Introns/Intergenic	-	-	AATAACCTGCACCTGCTCAGTCC	CTGGACATCGGCACTAGTTACCC	395	P	A	A	RM20581
PMS3595	Os_Chr06	27927344	27927355	(GA)6	(GA)6	(GA)6	(GA)6	(GA)6	(GA)7	(GA)6	(GA)6	(GA)6	(GA)6	(GA)6	-	Introns/Intergenic	-	-	TACGTCGCCGTTTGCTTTAT	TCGTCTCCCTCTTCTTCTCG	257	P	A	A	NA
PMS3596	Os_Chr06	27972257	27972306	(TA)25	(TA)25	(TA)25	(TA)25	(TA)25	(TA)25	(TA)7	(TA)8	(TA)25	(TA)25	(TA)25	-	Introns/Intergenic	-	-	TACGTTTACAACTCCCTCCGATCC	GTTCAAACGCTCGATGTGACAGG	485	A	A	A	RM20584
PMS3597	Os_Chr06	28007077	28007120	(AC)22	(AC)22	(AC)22	(AC)22	(AC)22	(AC)19	(AC)22	(AC)22	(AC)22	(AC)22	(AC)22	-	Introns/Intergenic	-	-	CATGTATTTGTGTGCACGTACCG	ACCTTTCTTGGGCCTTTCTTGG	262	P	A	A	RM20589
PMS3598	Os_Chr06	28021617	28021634	(AT)9	(AT)9	(AT)9	(AT)9	(AT)9	(AT)9	(AT)9	(AT)9	(AT)8	(AT)9	(AT)8	-	Introns/Intergenic	-	-	CAAGACGATGAACAGTGGGA	ATTTCCTCACATGATTGGGC	226	A	A	A	NA
PMS3599	Os_Chr06	28024712	28024725	(GT)7	(GT)6	(GT)6	(GT)7	(GT)7	(GT)6	(GT)7	(GT)7	(GT)7	(GT)6	(GT)6	-	Introns/Intergenic	-	-	ATGGCTTTTGGTTTTCGTTG	ATCCAAATAGCAGCACGGAC	212	P	A	A	NA
PMS3600	Os_Chr06	28051066	28051081	(AG)8	(AG)8	(AG)8	(AG)8	(AG)8	(AG)8	(AG)8	(AG)7	(AG)8	(AG)8	(AG)8	-	Introns/Intergenic	-	-	TGGAAAGAGGAAAAGGTGGA	ATCCCCACTCCTCCCACTC	185	A	A	A	NA

Polymorphic SSR (PMS) markers	Chromosomes	Start physical positions (bp)	End physical positions (bp)	Nipponbare	Kasalath	Nagina22	IR64	Pokkali	Bala	Tainung67	Azucena	Moroberekan	*Oryza rufipogon*	*Oryza nivara*	MSU gene locus IDs	Structural annotation	Functional annotation	SSR functional effects	Forward primers (5'-3')	Reverse primers (5'-3')	Amplified product size (bp)	Markers exhibiting polymorphism within *indica* rice	Transcrption factor genes-derived markers	Known cloned genes-derived markers	PMS markers corresponding with RM markers
PMS3601	Os_Chr06	28115929	28115964	(TA)18	(TA)18	(TA)18	(TA)7	(TA)8	(TA)18	(TA)18	(TA)18	(TA)9	(TA)18	(TA)18	-	Introns/Intergenic	-	-	TCAATTTCAACAATGGTGCC	AAGATGGTTTGTGTGGGACA	164	P	A	A	NA
PMS3602	Os_Chr06	28130731	28130756	(TA)13	(TA)13	(TA)13	(TA)13	(TA)13	(TA)13	(TA)13	(TA)13	(TA)7	(TA)13	(TA)13	-	Introns/Intergenic	-	-	GGAGTGAAACTGAGGCTCCTATCG	TCGTTCTCCCTGCAAGTTAATGG	403	A	A	A	RM20601
PMS3603	Os_Chr06	28164602	28164635	(CT)17	(CT)11	(CT)17	(CT)17	(CT)17	(CT)17	(CT)17	(CT)17	(CT)17	(CT)17	(CT)17	-	Introns/Intergenic	-	-	TGGTTTGGTTTGGTTTGGTT	AGTGTAGCAACTGAAGGGCG	236	P	A	A	NA
PMS3604	Os_Chr06	28204153	28204170	(CTT)6	(CTT)7	(CTT)6	(CTT)6	(CTT)7	(CTT)6	(CTT)6	(CTT)5	(CTT)6	(CTT)6	(CTT)6	LOC_Os06g46460	CDS	expressed protein	CODON_CHANGE_PLUS_CODON_DELETION	GGAGGAAGATCACTCCAGCA	CGCCTCTGCTCTCAAAATTC	205	P	A	A	NA
PMS3605	Os_Chr06	28345661	28345678	(GCG)6	(GCG)6	(GCG)5	(GCG)6	(GCG)6	(GCG)6	(GCG)6	(GCG)6	(GCG)6	(GCG)6	(GCG)6	-	Introns/Intergenic	-	-	CGATCCAGGTCTAGAATCGC	CCAATCACCGAGTCGTAGGT	261	P	A	A	NA
PMS3606	Os_Chr06	28414591	28414608	(CTC)6	(CTC)6	(CTC)6	(CTC)6	(CTC)6	(CTC)6	(CTC)6	(CTC)6	(CTC)6	(CTC)5	(CTC)6	-	Introns/Intergenic	-	-	CCATCTCCGTCATCGTAAGC	CTGGAAGGAGGAGAGGAGGT	218	A	A	A	NA
PMS3607	Os_Chr06	28432669	28432857	(ATA)63	(ATA)63	(ATA)63	(ATA)8	(ATA)6	(ATA)63	(ATA)63	(ATA)63	(ATA)11	(ATA)63	(ATA)63	-	Introns/Intergenic	-	-	TTACACCAGGCTACCCAAACTCG	TTGCTGAGTTCCCTCGTCTATCC	272	P	A	A	RM400
PMS3608	Os_Chr06	28456660	28456674	(GGC)5	(GGC)6	(GGC)5	(GGC)6	(GGC)6	(GGC)6	(GGC)5	(GGC)5	(GGC)5	(GGC)6	(GGC)5	LOC_Os06g46880	URR	surp module family protein, putative, expressed	-	CTGAGGGTGGAAGCCGTACT	TCTCGCTCTCTCTCGGAGTC	209	P	A	A	NA
PMS3609	Os_Chr06	28470115	28470146	(TG)16	(TG)14	(TG)16	(TG)16	(TG)16	(TG)14	(TG)16	(TG)16	(TG)16	(TG)16	(TG)16	-	Introns/Intergenic	-	-	AAATGCGTGTGTTTATCAGTGG	AGCCAAGTTGCTGTCTGGTT	288	P	A	A	RM3138
PMS3610	Os_Chr06	28471173	28471188	(GT)8	(GT)7	(GT)7	(GT)7	(GT)7	(GT)7	(GT)8	(GT)8	(GT)8	(GT)7	(GT)7	-	Introns/Intergenic	-	-	TCATCAGCTTCGACAGGATG	GTCTTCACGTCGTCCATCAG	258	A	A	A	NA
PMS3611	Os_Chr06	28501034	28501054	(ATA)7	(ATA)7	(ATA)7	(ATA)7	(ATA)7	(ATA)5	(ATA)7	(ATA)7	(ATA)7	(ATA)7	(ATA)7	-	Introns/Intergenic	-	-	CCTGACTTGATGCTTTCTTCTTCC	CCAACCCACCCACTGATATAATCC	370	P	A	A	RM20622
PMS3612	Os_Chr06	28524268	28524285	(GT)9	(GT)9	(GT)9	(GT)9	(GT)9	(GT)7	(GT)9	(GT)9	(GT)9	(GT)9	(GT)9	-	Introns/Intergenic	-	-	AAGCGGCCTGGAATTACTTT	CGCACACCACTCCTCTAACA	215	P	A	A	NA
PMS3613	Os_Chr06	28528963	28528998	(TA)18	(TA)18	(TA)18	(TA)18	(TA)18	(TA)18	(TA)7	(TA)13	(TA)18	(TA)18	(TA)18	-	Introns/Intergenic	-	-	GCTCGTACGGTGGGTGAATCC	GATGCGTCCTTCTTAGGTTGAAAGC	253	A	A	A	RM20623

Polymorphic SSR (PMS) markers	Chromosomes	Start physical positions (bp)	End physical positions (bp)	Nipponbare	Kasalath	Nagina22	IR64	Pokkali	Bala	Tainung67	Azucena	Moroberekan	*Oryza rufipogon*	*Oryza nivara*	MSU gene locus IDs	Structural annotation	Functional annotation	SSR functional effects	Forward primers (5'-3')	Reverse primers (5'-3')	Amplified product size (bp)	Markers exhibiting polymorphism within *indica* rice	Transcrption factor genes-derived markers	Known cloned genes-derived markers	PMS markers corresponding with RM markers
PMS3614	Os_Chr06	28530074	28530085	(AT)6	(AT)6	(AT)6	(AT)6	(AT)10	(AT)6	(AT)6	(AT)6	(AT)6	(AT)6	(AT)6	-	Introns/Intergenic	-	-	TGATGGTCCAATACCGATTT	TCACTCTTTACGAACATTAGCTCTACA	144	P	A	A	NA
PMS3615	Os_Chr06	28533532	28533587	(TC)28	(TC)28	(TC)28	(TC)28	(TC)28	(TC)28	(TC)28	(TC)28	(TC)28	(TC)24	(TC)28	-	Introns/Intergenic	-	-	GGAGGGAGGAATGGGTACACG	TTGAGAGTGAAACGAGAACCAACC	163	A	A	A	RM1370
PMS3616	Os_Chr06	28557853	28557872	(CT)10	(CT)10	(CT)10	(CT)10	(CT)7	(CT)10	(CT)10	(CT)10	(CT)10	(CT)10	(CT)10	LOC_Os06g47120	URR	expressed protein	-	CACTCGTCGGAGTTGGAATCG	CTTCTCGGCGCTTCTCTTCTTCC	175	P	A	A	RM20628
PMS3617	Os_Chr06	28585810	28585827	(AGC)6	(AGC)6	(AGC)6	(AGC)6	(AGC)6	(AGC)6	(AGC)5	(AGC)6	(AGC)6	(AGC)6	(AGC)6	-	Introns/Intergenic	-	-	GCAACCTTCACATGCGAGTA	TTTAGGAGGAGGATGATGCG	219	A	A	A	NA
PMS3618	Os_Chr06	28600213	28600242	(TTC)10	(TTC)7	(TTC)10	(TTC)10	(TTC)10	(TTC)7	(TTC)10	(TTC)10	(TTC)10	(TTC)10	(TTC)10	-	Introns/Intergenic	-	-	GGGTAAATGGACAATCCTATGG	ACCCTATTCTGGAGTTCATCTGG	139	P	A	A	RM340
PMS3619	Os_Chr06	28617548	28617565	(GA)9	(GA)9	(GA)8	(GA)9	(GA)9	(GA)9	(GA)9	(GA)9	(GA)9	(GA)9	(GA)9	LOC_Os06g47200	URR	LTPL85 - Protease inhibitor/seed storage/LTP family protein precursor, expressed	-	AAGCTTCACAGAGTCCAGGG	GTGTCCGTCCCGTTCATGT	267	P	A	A	NA
PMS3620	Os_Chr06	28652992	28653009	(GAG)6	(GAG)6	(GAG)6	(GAG)6	(GAG)6	(GAG)6	(GAG)6	(GAG)6	(GAG)6	(GAG)6	(GAG)8	LOC_Os06g47270	CDS	zinc finger, C3HC4 type domain containing protein, expressed	CODON_INSERTION	AGACGGTTCCGAAGGAGG	TACTCGACCTCCTCCACGTC	187	A	A	A	NA
PMS3621	Os_Chr06	28686289	28686302	(CA)7	(CA)7	(CA)7	(CA)6	(CA)7	(CA)7	(CA)7	(CA)7	(CA)7	(CA)7	(CA)7	-	Introns/Intergenic	-	-	ACATCCACGGATCACCAGAT	AAGACTGTTTTGCGCGATTT	169	P	A	A	NA
PMS3622	Os_Chr06	28797137	28797154	(TGG)6	(TGG)6	(TGG)6	(TGG)6	(TGG)6	(TGG)6	(TGG)5	(TGG)6	(TGG)6	(TGG)6	(TGG)6	LOC_Os06g47550	CDS	cadmium/zinc-transporting ATPase, putative, expressed	CODON_DELETION	GAGATGCAGCAGAGTTGCAG	CTAGGTCCCCTCTCCTCCTC	232	A	A	A	NA
PMS3623	Os_Chr06	28818582	28818599	(CGG)6	(CGG)6	(CGG)6	(CGG)6	(CGG)6	(CGG)6	(CGG)5	(CGG)6	(CGG)6	(CGG)6	(CGG)6	LOC_Os06g47590	CDS	AP2 domain containing protein, expressed	CODON_CHANGE_PLUS_CODON_DELETION	CGATCTGGCTGGGTACCTT	TAGCTGTGGCAATCCTCCTC	280	A	A	A	NA
PMS3624	Os_Chr06	28832121	28832138	(GGA)6	(GGA)6	(GGA)6	(GGA)6	(GGA)6	(GGA)6	(GGA)6	(GGA)6	(GGA)6	(GGA)6	(GGA)5	LOC_Os06g47620	CDS	peptidase, putative, expressed	CODON_DELETION	GCTGATCGACTGACTTTGCC	CCGCCGGATTCTACTACATC	248	A	A	A	NA
PMS3625	Os_Chr06	29163837	29163863	(GCC)9	(GCC)8	(GCC)6	(GCC)9	(GCC)9	(GCC)9	(GCC)8	(GCC)9	(GCC)9	(GCC)9	(GCC)7	LOC_Os06g48210	CDS	DEAD-box ATP-dependent RNA helicase, putative, expressed	CODON_DELETION	GAATCGCCTCCCTCCAACTCG	CTCATCGCTGTCCGGTGTTCC	102	P	A	A	RM6224
PMS3626	Os_Chr06	29170160	29170171	(GA)6	(GA)6	(GA)6	(GA)6	(GA)6	(GA)6	(GA)7	(GA)6	(GA)6	(GA)6	(GA)6	-	Introns/Intergenic	-	-	TGCACGTTTTCAGTGCTACC	TGTCCGTGCCCATAATTCTT	220	A	A	A	NA

Polymorphic SSR (PMS) markers	Chromosomes	Start physical positions (bp)	End physical positions (bp)	Nipponbare	Kasalath	Nagina22	IR64	Pokkali	Bala	Tainung67	Azucena	Moroberekan	*Oryza rufipogon*	*Oryza nivara*	MSU gene locus IDs	Structural annotation	Functional annotation	SSR functional effects	Forward primers (5'-3')	Reverse primers (5'-3')	Amplified product size (bp)	Markers exhibiting polymorphism within *indica* rice	Transcrption factor genes-derived markers	Known cloned genes-derived markers	PMS markers corresponding with RM markers
PMS3627	Os_Chr06	29180706	29180720	(CTC)5	(CTC)6	(CTC)5	(CTC)5	(CTC)5	(CTC)5	(CTC)5	(CTC)5	(CTC)5	(CTC)5	(CTC)5	LOC_Os06g48240	CDS	AAA ATPase, putative, expressed	CODON_INSERTION	GATGCACCTCCACAAGGC	GAGAACGAAGGGGATGTACG	271	P	A	A	NA
PMS3628	Os_Chr06	29187663	29187683	(CCT)7	(CCT)7	(CCT)7	(CCT)7	(CCT)7	(CCT)7	(CCT)7	(CCT)7	(CCT)7	(CCT)7	(CCT)5	-	Introns/Intergenic	-	-	GCTGTTGTCGTGTGGTCTGAGC	CACAACAAGGGAATGTCACACG	119	A	A	A	RM20652
PMS3629	Os_Chr06	29381283	29381297	(GCG)5	(GCG)6	(GCG)5	(GCG)5	(GCG)5	(GCG)5	(GCG)5	(GCG)5	(GCG)6	(GCG)6	(GCG)6	LOC_Os06g48560	CDS	transferase family protein, putative, expressed	CODON_INSERTION	CAACGTTCCCAGCCTAGAAG	GCCGGCTAAGGGGTAGTAGT	260	P	A	A	NA
PMS3630	Os_Chr06	29447478	29447498	(CGA)7	(CGA)7	(CGA)7	(CGA)5	(CGA)7	(CGA)7	(CGA)7	(CGA)7	(CGA)7	(CGA)8	(CGA)7	LOC_Os06g48680	URR	expressed protein	-	CGCTAGAGCTAGCTATGGAGATCG	CCCTCCTCTACAGTTTCCACTCC	109	P	A	A	RM20660
PMS3631	Os_Chr06	29480853	29480870	(GGA)6	(GGA)6	(GGA)6	(GGA)6	(GGA)6	(GGA)6	(GGA)5	(GGA)6	(GGA)6	(GGA)6	(GGA)6	LOC_Os06g48720	URR	OsHMA2|OsHMA2v	-	CCCAACTCCCAAGAAGAACA	TGCTTGTCTTCGTCATCGTC	237	A	A	P	NA
PMS3632	Os_Chr06	29523770	29523787	(AAC)6	(AAC)5	(AAC)6	(AAC)6	(AAC)6	(AAC)6	(AAC)6	(AAC)6	(AAC)6	(AAC)6	(AAC)6	-	Introns/Intergenic	-	-	CGAATCTAAATCAGGGGCAA	CTTCGTTCTTGGTCGTCCTC	210	P	A	A	NA
PMS3633	Os_Chr06	29568638	29568719	(AT)41	(AT)12	(AT)41	(AT)41	(AT)41	(AT)35	(AT)41	(AT)41	(AT)41	(AT)41	(AT)41	-	Introns/Intergenic	-	-	TTTTGATGGACGATGGAAGC	CCACCTACTAGTCCGAAACCTC	179	P	A	A	NA
PMS3634	Os_Chr06	29570810	29570857	(AATA)12	(AATA)12	(AATA)12	(AATA)12	(AATA)12	(AATA)6	(AATA)6	(AATA)5	(AATA)12	(AATA)12	(AATA)12	-	Introns/Intergenic	-	-	AGTATAACCGTCTGGTTTCACTGACG	GCCTGCTCGAGAGAATCAGG	187	P	A	A	RM7555
PMS3635	Os_Chr06	29581107	29581121	(ACG)5	(ACG)6	(ACG)6	(ACG)6	(ACG)6	(ACG)6	(ACG)5	(ACG)5	(ACG)6	(ACG)6	(ACG)6	-	Introns/Intergenic	-	-	CAAGATCAAACACCACACCG	TCATACGAGTTTGGTTGCCA	270	A	A	A	NA
PMS3636	Os_Chr06	29625974	29626012	(TAT)13	(TAT)13	(TAT)13	(TAT)8	(TAT)8	(TAT)13	(TAT)13	(TAT)13	(TAT)13	(TAT)13	(TAT)12	-	Introns/Intergenic	-	-	CTGGGTCTAATCTCGTCCTAAATTGC	CGCCTCTCATAACAGTCCACTCC	380	P	A	A	RM439
PMS3637	Os_Chr06	29761617	29761634	(AGA)6	(AGA)5	(AGA)6	(AGA)5	(AGA)5	(AGA)6	(AGA)6	(AGA)6	(AGA)6	(AGA)6	(AGA)5	-	Introns/Intergenic	-	-	ATTGAATTGAGCTTGACGGG	CTAGTCTGCTAAGGTGGGCG	223	P	A	A	NA
PMS3638	Os_Chr06	29782137	29782157	(CGC)7	(CGC)7	(CGC)7	(CGC)5	(CGC)7	(CGC)7	(CGC)7	(CGC)7	(CGC)7	(CGC)7	(CGC)7	LOC_Os06g49140	URR	ribosomal RNA large subunit methyltransferase J, putative, expressed	-	CCAACCCAACATCTGCTCTT	AGAAGCAGCGAAGTAAAGCG	258	P	A	A	NA
PMS3639	Os_Chr06	29786304	29786318	(CCG)5	(CCG)5	(CCG)5	(CCG)5	(CCG)5	(CCG)6	(CCG)5	(CCG)6	(CCG)6	(CCG)5	(CCG)5	LOC_Os06g49150	URR	exostosin, putative, expressed	-	GCGAATAAAGCACTTGGAGC	GCTCGGAGTCAATACGCTTC	241	P	A	A	NA

Polymorphic SSR (PMS) markers	Chromosomes	Start physical positions (bp)	End physical positions (bp)	Nipponbare	Kasalath	Nagina22	IR64	Pokkali	Bala	Tainung67	Azucena	Moroberekan	*Oryza rufipogon*	*Oryza nivara*	MSU gene locus IDs	Structural annotation	Functional annotation	SSR functional effects	Forward primers (5'-3')	Reverse primers (5'-3')	Amplified product size (bp)	Markers exhibiting polymorphism within *indica* rice	Transcrption factor genes-derived markers	Known cloned genes-derived markers	PMS markers corresponding with RM markers
PMS3640	Os_Chr06	29889880	29889895	(TC)8	(TC)7	(TC)7	(TC)8	(TC)8	(TC)7	(TC)8	(TC)8	(TC)7	(TC)7	(TC)8	-	Introns/Intergenic	-	-	CATTTGACGTGGCGTTTATG	AGCTATTGCTCCAAGGTCCA	250	P	A	A	NA
PMS3641	Os_Chr06	29996469	29996486	(CGG)6	(CGG)6	(CGG)6	(CGG)6	(CGG)6	(CGG)6	(CGG)5	(CGG)6	(CGG)6	(CGG)6	(CGG)6	LOC_Os06g49530	CDS	OsFBX206 - F-box domain containing protein, expressed	CODON_DELETION	GGTCTGGACGATGACCTTGT	ATCACAACCAGATTCGCACA	225	A	A	A	NA
PMS3642	Os_Chr06	30045083	30045096	(GA)7	(GA)8	(GA)7	(GA)7	(GA)7	(GA)7	(GA)7	(GA)7	(GA)7	(GA)7	(GA)7	-	Introns/Intergenic	-	-	CTTCCTCCCCTCTTCCCTCT	TCTCTCTTTTTCTCTCTGCCG	243	P	A	A	NA
PMS3643	Os_Chr06	30084426	30084439	(CT)7	(CT)7	(CT)7	(CT)7	(CT)7	(CT)7	(CT)7	(CT)6	(CT)7	(CT)7	(CT)7	-	Introns/Intergenic	-	-	ATTACCCCCTCGATCCAATC	GCCACTAGCGAGAGAGAGGA	150	A	A	A	NA
PMS3644	Os_Chr06	30092246	30092263	(GCG)6	(GCG)6	(GCG)6	(GCG)5	(GCG)6	(GCG)6	(GCG)6	(GCG)6	(GCG)6	(GCG)6	(GCG)6	LOC_Os06g49750	CDS	kelch motif family protein, expressed	CODON_DELETION	TGTACCGAATCGCATATCCA	GGAAGGTGTTGATCGTGGAC	208	P	A	A	NA
PMS3645	Os_Chr06	30176296	30176351	(TA)28	(TA)28	(TA)28	(TA)28	(TA)28	(TA)28	(TA)9	(TA)21	(TA)28	(TA)28	(TA)28	-	Introns/Intergenic	-	-	ATGCACACATAGTCAACAGCTTCC	GTGATCACCACACAGACTGAAACC	272	A	A	A	RM20686
PMS3646	Os_Chr06	30184452	30184469	(CGG)6	(CGG)6	(CGG)6	(CGG)6	(CGG)6	(CGG)6	(CGG)6	(CGG)6	(CGG)5	(CGG)6	(CGG)6	LOC_Os06g49860	CDS	methyltransferase, putative, expressed	CODON_DELETION	CCCACCAACACAAAGCCTAAACC	CTTCCTCTCGGGCCTACTGACC	378	A	A	A	RM20688
PMS3647	Os_Chr06	30234714	30234734	(GCC)7	(GCC)7	(GCC)7	(GCC)7	(GCC)7	(GCC)7	(GCC)7	(GCC)7	(GCC)7	(GCC)5	(GCC)7	-	Introns/Intergenic	-	-	AGGAAGGAGAAGGCTCGTCTAGG	GTGTGATCTTGATTGGTGCTTGC	171	A	A	A	RM20690
PMS3648	Os_Chr06	30317556	30317645	(TA)45	(TA)45	(TA)45	(TA)9	(TA)7	(TA)45	(TA)45	(TA)10	(TA)45	(TA)18	(TA)45	-	Introns/Intergenic	-	-	ACGGTCGTAGCAATAACTAGC	GGTCATAGGTCATAACTAGTCTGC	414	P	A	A	RM20698
PMS3649	Os_Chr06	30343075	30343092	(GA)9	(GA)14	(GA)9	(GA)9	(GA)9	(GA)14	(GA)9	(GA)9	(GA)9	(GA)14	(GA)9	-	Introns/Intergenic	-	-	CCACGGCACTGGAGATTATT	AGGCCAACCAGAGATAACCA	279	P	A	A	NA
PMS3650	Os_Chr06	30349822	30349847	(AG)13	(AG)13	(AG)13	(AG)13	(AG)13	(AG)13	(AG)13	(AG)13	(AG)6	(AG)13	(AG)13	LOC_Os06g50100	URR	tyrosine protein kinase domain containing protein, putative, expressed	-	CCCAGTGAGCAGTGACAAGA	GGATTCCAACCACATGATCC	264	A	A	A	NA
PMS3651	Os_Chr06	30418483	30418500	(CGG)6	(CGG)6	(CGG)6	(CGG)6	(CGG)6	(CGG)7	(CGG)5	(CGG)6	(CGG)6	(CGG)6	(CGG)6	LOC_Os06g50230	DRR	expressed protein	-	GGGACCCTCAAGTATTCGGT	GGATTGCAGGAGAAGAACCA	158	P	A	A	NA
PMS3652	Os_Chr06	30474423	30474446	(GGA)8	(GGA)6	(GGA)8	(GGA)6	(GGA)5	(GGA)6	(GGA)8	(GGA)6	(GGA)6	(GGA)6	(GGA)8	LOC_Os06g50340	CDS	FON1	CODON_DELETION	GGTGAGGTTGATGGCGAC	TCACCATCGCATTTCACAAT	249	P	A	P	NA

Polymorphic SSR (PMS) markers	Chromosomes	Start physical positions (bp)	End physical positions (bp)	Nipponbare	Kasalath	Nagina22	IR64	Pokkali	Bala	Tainung67	Azucena	Moroberekan	*Oryza rufipogon*	*Oryza nivara*	MSU gene locus IDs	Structural annotation	Functional annotation	SSR functional effects	Forward primers (5'-3')	Reverse primers (5'-3')	Amplified product size (bp)	Markers exhibiting polymorphism within *indica* rice	Transcrption factor genes-derived markers	Known cloned genes-derived markers	PMS markers corresponding with RM markers
PMS3653	Os_Chr06	30563991	30564014	(AG)12	(AG)12	(AG)12	(AG)12	(AG)12	(AG)12	(AG)12	(AG)12	(AG)12	(AG)10	(AG)12	-	Introns/Intergenic	-	-	CACGCATAATGACACTGTTGG	GATCCTTAATTCGATCGCCA	212	A	A	A	NA
PMS3654	Os_Chr06	30575618	30575649	(TA)16	(TA)16	(TA)16	(TA)16	(TA)10	(TA)16	(TA)9	(TA)16	(TA)16	(TA)15	(TA)16	-	Introns/Intergenic	-	-	CCTCTAGTGTGTTGCACGGA	TAAATTTGGGAGGGGTAGGG	241	P	A	A	NA
PMS3655	Os_Chr06	30595740	30595761	(TA)11	(TA)11	(TA)10	(TA)11	(TA)10	(TA)9	(TA)7	(TA)11	(TA)11	(TA)11	(TA)11	-	Introns/Intergenic	-	-	TAAATTTGGGAGGGGTAGGG	CCTCTAGTGTGTTGCACGGA	233	P	A	A	NA
PMS3656	Os_Chr06	30599484	30599517	(TA)17	(TA)17	(TA)16	(TA)17	(TA)13	(TA)17	(TA)17	(TA)17	(TA)17	(TA)14	(TA)11	-	Introns/Intergenic	-	-	ATGCACACGCTGTCAATTTC	ATTCAGGCCCATGTAACTGC	224	P	A	A	NA
PMS3657	Os_Chr06	30622743	30622758	(AT)8	(AT)6	(AT)7	(AT)8	(AT)8	(AT)6	(AT)8	(AT)8	(AT)8	(AT)8	(AT)8	-	Introns/Intergenic	-	-	ATGCGTACAAGGGATGGAAA	AAGGGCCCATACCAGAGAGT	225	P	A	A	NA
PMS3658	Os_Chr06	30666113	30666162	(TA)25	(TA)19	(TA)25	(TA)25	(TA)25	(TA)12	(TA)25	(TA)25	(TA)25	(TA)14	(TA)24	-	Introns/Intergenic	-	-	CTATAGATGTGAGGGGGAGCA	GTAACCGCCACATTCCAGAT	103	P	A	A	NA
PMS3659	Os_Chr06	30750878	30750915	(TA)19	(TA)25	(TA)19	(TA)19	(TA)19	(TA)12	(TA)19	(TA)19	(TA)19	(TA)25	(TA)24	-	Introns/Intergenic	-	-	GCTCTGTGGGTTGTGTTTAAATGG	GCGCAGTCCAATTTCATTTCC	547	P	A	A	RM20727
PMS3660	Os_Chr06	30756700	30756715	(AT)8	(AT)6	(AT)7	(AT)8	(AT)8	(AT)6	(AT)8	(AT)8	(AT)8	(AT)8	(AT)8	-	Introns/Intergenic	-	-	ATGCGTACAAGGGATGGAAA	AAGGGCCCATACCAGAGAGT	225	P	A	A	NA
PMS3661	Os_Chr06	30787734	30787785	(AT)26	(AT)26	(AT)26	(AT)26	(AT)9	(AT)26	(AT)8	(AT)26	(AT)26	(AT)26	(AT)21	-	Introns/Intergenic	-	-	GCATTGATTCAAAGGGCAAC	ATGTGGCATGCATTTCAAGA	213	P	A	A	NA
PMS3662	Os_Chr06	30844065	30844130	(TTA)22	(TTA)22	(TTA)22	(TTA)22	(TTA)22	(TTA)22	(TTA)22	(TTA)7	(TTA)22	(TTA)22	(TTA)22	-	Introns/Intergenic	-	-	CCCGCTGCTGTTACTCTTAATCG	GCTCGATGGAATAATCGAGATCC	693	A	A	A	RM8242
PMS3663	Os_Chr06	30846337	30846351	(GCA)5	(GCA)5	(GCA)5	(GCA)5	(GCA)5	(GCA)5	(GCA)5	(GCA)6	(GCA)6	(GCA)5	(GCA)5	-	Introns/Intergenic	-	-	CGAGTGACTCTCACCATCCA	CGACAAGGTGGAGAAGAAGC	229	A	A	A	NA
PMS3664	Os_Chr06	30918769	30918788	(CT)10	(CT)10	(CT)10	(CT)9	(CT)10	(CT)9	(CT)10	(CT)10	(CT)10	(CT)9	(CT)10	LOC_Os06g51110	URR	cyclin, putative, expressed	-	ACCAAGAACGCATCAATCTACCG	CTCTGCTGCTTCTTCTTCTTCTGG	147	P	A	A	RM20743
PMS3665	Os_Chr06	31011437	31011448	(TC)6	(TC)6	(TC)6	(TC)6	(TC)7	(TC)7	(TC)6	(TC)6	(TC)6	(TC)6	(TC)6	-	Introns/Intergenic	-	-	CACAGGTGATTAAGCTGCCA	AATGCTGGTTTCAGGTTTGG	158	P	A	A	NA

Polymorphic SSR (PMS) markers	Chromosomes	Start physical positions (bp)	End physical positions (bp)	Nipponbare	Kasalath	Nagina22	IR64	Pokkali	Bala	Tainung67	Azucena	Moroberekan	*Oryza rufipogon*	*Oryza nivara*	MSU gene locus IDs	Structural annotation	Functional annotation	SSR functional effects	Forward primers (5'-3')	Reverse primers (5'-3')	Amplified product size (bp)	Markers exhibiting polymorphism within *indica* rice	Transcrption factor genes-derived markers	Known cloned genes-derived markers	PMS markers corresponding with RM markers
PMS3666	Os_Chr06	31098525	31098538	(GA)7	(GA)7	(GA)7	(GA)7	(GA)7	(GA)6	(GA)7	(GA)7	(GA)7	(GA)7	(GA)7	-	Introns/Intergenic	-	-	GAAACAGAGAGGG AGCGAGA	TTTTGGATGGACCGA TTTTC	147	P	A	A	NA
PMS3667	Os_Chr06	31127364	31127385	(AG)11	(AG)11	(AG)8	(AG)11	(AG)11	(AG)11	(AG)11	(AG)11	(AG)11	(AG)11	(AG)11	-	Introns/Intergenic	-	-	CAGGGAATAAACAG GAGGAAGAGG	CCAACCTCAACCTAG TTAACCTCACC	174	P	A	A	RM20768
PMS3668	Os_Chr06	31132918	31132935	(CCG)6	(CCG)6	(CCG)6	(CCG)6	(CCG)6	(CCG)6	(CCG)6	(CCG)6	(CCG)6	(CCG)5	(CCG)6	LOC_Os06g51400	URR	expressed protein	-	GTTTTACGGTTCAG GGACCA	GATGGGGAGAGATG GAGACA	251	A	A	A	NA
PMS3669	Os_Chr06	31169330	31169353	(CCG)8	(CCG)5	(CCG)5	(CCG)5	(CCG)5	(CCG)5	(CCG)8	(CCG)8	(CCG)8	(CCG)5	(CCG)5	LOC_Os06g51450	CDS	PHD-finger domain containing protein, expressed	CODON_DELETION	CAGATGCGGAGGAT GAAGAG	GCAATTTCCCTTCCC AATTT	219	A	A	A	NA
PMS3670	Os_Chr06	31180014	31180027	(AT)7	(AT)7	(AT)7	(AT)7	(AT)7	(AT)6	(AT)7	(AT)7	(AT)7	(AT)7	(AT)7	-	Introns/Intergenic	-	-	CCTCACTCACATTT GGGCTT	CTCGCATACTAACTG GACATGA	224	P	A	A	NA
PMS3671	Os_Chr06	31212913	31212940	(CTCC)7	(CTCC)8	(CTCC)7	(CTCC)8	(CTCC)8	(CTCC)7	(CTCC)5	(CTCC)7	(CTCC)7	(CTCC)7	(CTCC)7	-	Introns/Intergenic	-	-	TGTTCACTCCAACT CCGACATGTACC	AAACCAGACCCAGA CTGAGCAAGG	149	P	A	A	RM20774
PMS3672	Os_Chr07	112229	112268	(GA)20	(GA)21	(GA)20	(GA)20	(GA)20	(GA)20	(GA)20	(GA)20	(GA)20	(GA)20	(GA)20	-	Introns/Intergenic	-	-	GCTAGTAAGAGTGT GCAGTCATGG	CATGTGCCTCTTCTG AGTACTGG	244	P	A	A	RM20782
PMS3673	Os_Chr07	134003	134022	(TG)10	(TG)10	(TG)9	(TG)11	(TG)11	(TG)11	(TG)10	(TG)10	(TG)10	(TG)10	(TG)11	-	Introns/Intergenic	-	-	GCTGCAGAACCTGT ATCTTGAACG	AGGCTGCTTACCGTG ATCTCTCC	96	P	A	A	RM20785
PMS3674	Os_Chr07	143518	143532	(GAG)5	(GAG)5	(GAG)5	(GAG)5	(GAG)5	(GAG)5	(GAG)5	(GAG)5	(GAG)5	(GAG)6	(GAG)5	LOC_Os07g01240	URR	SRL1	-	GGAAAGGAAACAGA GGAGGG	CGGGTGTACGTAGC GAATTT	278	A	A	P	NA
PMS3675	Os_Chr07	183539	183550	(AT)6	(AT)6	(AT)6	(AT)6	(AT)6	(AT)7	(AT)6	(AT)6	(AT)6	(AT)6	(AT)6	-	Introns/Intergenic	-	-	AGGGCATGTTTCAT ACTCGG	CATGCCAATTCAGCT GGTTA	173	P	A	A	NA
PMS3676	Os_Chr07	209037	209054	(CCT)6	(CCT)6	(CCT)6	(CCT)6	(CCT)6	(CCT)6	(CCT)6	(CCT)5	(CCT)6	(CCT)5	(CCT)6	-	Introns/Intergenic	-	-	CAAATCCGTGCCGT TAAACT	CTGACTGGGAGCGA CTTGAC	229	A	A	A	NA
PMS3677	Os_Chr07	239657	239678	(AG)11	(AG)10	(AG)11	(AG)11	(AG)11	(AG)11	(AG)11	(AG)11	(AG)11	(AG)11	(AG)11	-	Introns/Intergenic	-	-	TTCAACCTCCAACC TCATCC	AGGACATTGAATCGA GACGG	137	P	A	A	NA
PMS3678	Os_Chr07	239784	239797	(GA)7	(GA)7	(GA)7	(GA)7	(GA)7	(GA)7	(GA)7	(GA)6	(GA)7	(GA)7	(GA)7	-	Introns/Intergenic	-	-	CCGTCTCGATTCAA TGTCCT	CTCCTCTCTCTCAGG CGTGT	280	A	A	A	NA

Polymorphic SSR (PMS) markers	Chromosomes	Start physical positions (bp)	End physical positions (bp)	Nipponbare	Kasalath	Nagina22	IR64	Pokkali	Bala	Tainung67	Azucena	Moroberekan	*Oryza rufipogon*	*Oryza nivara*	MSU gene locus IDs	Structural annotation	Functional annotation	SSR functional effects	Forward primers (5'-3')	Reverse primers (5'-3')	Amplified product size (bp)	Markers exhibiting polymorphism within *indica* rice	Transcrption factor genes-derived markers	Known cloned genes-derived markers	PMS markers corresponding with RM markers
PMS3679	Os_Chr07	252749	252764	(CA)8	(CA)8	(CA)8	(CA)8	(CA)8	(CA)8	(CA)6	(CA)7	(CA)7	(CA)8	(CA)8	-	Introns/Intergenic	-	-	CGTCCAGCCCGTTTACTTAC	GTCGAGGAGATTCTTGCTGC	176	A	A	A	NA
PMS3680	Os_Chr07	259152	259214	(TAA)21	(TAA)21	(TAA)21	(TAA)21	(TAA)21	(TAA)21	(TAA)8	(TAA)21	(TAA)21	(TAA)21	(TAA)7	-	Introns/Intergenic	-	-	TTGTTTCTGTGTGGTCGGAG	AAATGATAGAGCTCTCGCGG	154	A	A	A	NA
PMS3681	Os_Chr07	264500	264517	(GGT)6	(GGT)6	(GGT)6	(GGT)6	(GGT)6	(GGT)6	(GGT)6	(GGT)6	(GGT)6	(GGT)5	(GGT)5	-	Introns/Intergenic	-	-	GGCAGGGATGATGAGTAGGA	GACGAAGCAGTCGTGGAAGT	226	A	A	A	NA
PMS3682	Os_Chr07	264959	264982	(AT)12	(AT)12	(AT)12	(AT)8	(AT)12	(AT)12	(AT)12	(AT)12	(AT)12	(AT)12	(AT)12	-	Introns/Intergenic	-	-	AGCTAACCTACCTTGCTGCATCG	GTTCTTGACGGCGAAGTATTGG	183	P	A	A	RM20804
PMS3683	Os_Chr07	300575	300594	(TA)10	(TA)10	(TA)10	(TA)10	(TA)10	(TA)7	(TA)10	(TA)10	(TA)10	(TA)10	(TA)10	-	Introns/Intergenic	-	-	ACAACGTCTTTGCACCCTTC	AACCTCTATTCCCCAATCCG	270	P	A	A	NA
PMS3684	Os_Chr07	306947	306970	(AT)12	(AT)10	(AT)12	(AT)12	(AT)12	(AT)6	(AT)12	(AT)12	(AT)12	(AT)12	(AT)12	LOC_Os07g01480	DRR	oxygen evolving enhancer protein 3 domain containing protein, expressed	-	CTATGCCGACACCAAGATCATCC	AGAAGTGCCCTGTGTTGTGTTGG	292	P	A	A	RM20811
PMS3685	Os_Chr07	312052	312067	(AG)8	(AG)8	(AG)6	(AG)8	(AG)8	(AG)8	(AG)8	(AG)8	(AG)8	(AG)8	(AG)8	-	Introns/Intergenic	-	-	TTTTGGATTTGGATTTGGGA	ACAAGCACAAGCACAAGCAC	215	P	A	A	NA
PMS3686	Os_Chr07	329532	329587	(TA)28	(TA)28	(TA)17	(TA)10	(TA)15	(TA)8	(TA)28	(TA)28	(TA)28	(TA)28	(TA)28	-	Introns/Intergenic	-	-	GAAATGATGGAGTGCGTGTG	ATGTGCATGCAAGGCATAAA	166	P	A	A	NA
PMS3687	Os_Chr07	334505	334519	(TCT)5	(TCT)6	(TCT)6	(TCT)5	(TCT)5	(TCT)6	(TCT)5	(TCT)5	(TCT)5	(TCT)5	(TCT)5	LOC_Os07g01530	CDS	expressed protein	CODON_INSERTION	GATGATGAGTCGCCGAGAAT	GGAGCTCTTCAGCTTCCTCA	273	P	A	A	NA
PMS3688	Os_Chr07	372345	372360	(AT)8	(AT)8	(AT)8	(AT)7	(AT)8	(AT)8	(AT)8	(AT)8	(AT)8	(AT)8	(AT)8	-	Introns/Intergenic	-	-	CTTGCCCCAAGAATTTTTCA	GCGTGCCCAATGATAAGAGT	253	P	A	A	NA
PMS3689	Os_Chr07	412965	412982	(GCG)6	(GCG)6	(GCG)6	(GCG)6	(GCG)5	(GCG)5	(GCG)6	(GCG)6	(GCG)6	(GCG)5	(GCG)6	LOC_Os07g01680	CDS	dirigent, putative, expressed	CODON_DELETION	CTCATGTAACGACGACGGTG	GCTACGTGCTGTGGAGGAC	153	P	A	A	NA
PMS3690	Os_Chr07	463681	463702	(TA)11	(TA)11	(TA)11	(TA)11	(TA)11	(TA)11	(TA)11	(TA)11	(TA)11	(TA)10	(TA)8	-	Introns/Intergenic	-	-	GGATTCTATTAGAGCCCATACTCC	CCTGCCTAATCAATTTGACC	145	A	A	A	RM20820
PMS3691	Os_Chr07	477609	477624	(GA)8	(GA)9	(GA)8	(GA)8	(GA)8	(GA)8	(GA)8	(GA)8	(GA)8	(GA)8	(GA)8	LOC_Os07g01820	URR	OsMADS15|DEP	-	CGATCTCGATCCGACCTAAC	CAATCACAGCTCGACAGCAT	168	P	P	P	NA

**Polymorphic SSR (PMS) markers**	**Chromosomes**	**Start physical positions (bp)**	**End physical positions (bp)**	**Nipponbare**	**Kasalath**	**Nagina22**	**IR64**	**Pokkali**	**Bala**	**Tainung67**	**Azucena**	**Moroberekan**	***Oryza rufipogon***	***Oryza nivara***	**MSU gene locus IDs**	**Structural annotation**	**Functional annotation**	**SSR functional effects**	**Forward primers (5'-3')**	**Reverse primers (5'-3')**	**Amplified product size (bp)**	**Markers exhibiting polymorphism within *indica* rice**	**Transcrption factor genes-derived markers**	**Known cloned genes-derived markers**	**PMS markers corresponding with RM markers**
PMS3692	Os_Chr07	502229	502308	(TA)40	(TA)40	(TA)40	(TA)40	(TA)40	(TA)21	(TA)40	(TA)40	(TA)40	(TA)40	(TA)14	-	Introns/Intergenic	-	-	CGTCAGCTTCTTGATCATCG	TTTTATGATGAGATGCCTCAAA	220	P	A	A	NA
PMS3693	Os_Chr07	516940	516951	(AG)6	(AG)10	(AG)10	(AG)6	(AG)10	(AG)10	(AG)6	(AG)6	(AG)6	(AG)6	(AG)10	-	Introns/Intergenic	-	-	AAATAGCTGGGAGTTGGTCG	AGCCACATTTCTCGGAAAGA	172	P	A	A	NA
PMS3694	Os_Chr07	547533	547552	(CT)10	(CT)9	(CT)10	(CT)10	(CT)7	(CT)10	(CT)10	(CT)10	(CT)10	(CT)10	(CT)10	-	Introns/Intergenic	-	-	GATACGACGATCCGTGAATAGGG	CTCTTCCTTCCGTCATCGATCC	238	P	A	A	RM20829
PMS3695	Os_Chr07	581773	581796	(GTG)8	(GTG)8	(GTG)8	(GTG)8	(GTG)8	(GTG)8	(GTG)8	(GTG)8	(GTG)8	(GTG)7	(GTG)8	LOC_Os07g01940	CDS	sister chromatid cohesion 2, putative, expressed	CODON_CHANGE_PLUS_CODON_DELETION	AAGCTCGTCGAGAGTAAGAAGACC	ACCAGTCCAATCTTCGTCATCG	100	A	A	A	RM6652
PMS3696	Os_Chr07	634015	634032	(GCG)6	(GCG)6	(GCG)6	(GCG)6	(GCG)6	(GCG)6	(GCG)6	(GCG)5	(GCG)6	(GCG)6	(GCG)6	LOC_Os07g02060	CDS	WRKY29, expressed	CODON_DELETION	GTACTTACGCTGCCTCGGTG	ACTCGTCGGCCATATTCG	240	A	A	A	NA
PMS3697	Os_Chr07	668650	668664	(GGC)5	(GGC)6	(GGC)5	(GGC)5	(GGC)5	(GGC)5	(GGC)5	(GGC)5	(GGC)5	(GGC)5	(GGC)5	LOC_Os07g02110	CDS	expressed protein	CODON_INSERTION	CAGATCTGGCCTGAGGGG	AGATCTGGGAGGGAGGGAG	179	P	A	A	NA
PMS3698	Os_Chr07	690591	690608	(GGC)6	(GGC)6	(GGC)6	(GGC)6	(GGC)6	(GGC)6	(GGC)5	(GGC)6	(GGC)6	(GGC)6	(GGC)6	-	Introns/Intergenic	-	-	GAGCTAGAACACCAGGCACC	GAGTACGTGGTGCGCGTC	258	A	A	A	NA
PMS3699	Os_Chr07	746673	746686	(AT)7	(AT)6	(AT)7	(AT)7	(AT)7	(AT)7	(AT)7	(AT)7	(AT)7	(AT)7	(AT)7	-	Introns/Intergenic	-	-	ATTTTTCCTGACCGCATTTG	TGTTGAATGTGCCCACTGAT	250	P	A	A	NA
PMS3700	Os_Chr07	903070	903102	(TCT)11	(TCT)11	(TCT)11	(TCT)11	(TCT)11	(TCT)11	(TCT)11	(TCT)6	(TCT)11	(TCT)11	(TCT)11	-	Introns/Intergenic	-	-	TAGCTCCATGCCAGTTTGTG	CCCACCACAACCTTCTTGAT	150	A	A	A	NA
PMS3701	Os_Chr07	983111	983124	(AT)7	(AT)8	(AT)7	(AT)7	(AT)7	(AT)7	(AT)7	(AT)7	(AT)7	(AT)7	(AT)7	-	Introns/Intergenic	-	-	CAACGGGTGAATGCAATTTT	TCGGTTTCTAAGTCCGTTCG	265	P	A	A	NA
PMS3702	Os_Chr07	987502	987519	(AT)9	(AT)9	(AT)8	(AT)9	(AT)9	(AT)9	(AT)9	(AT)9	(AT)9	(AT)9	(AT)9	-	Introns/Intergenic	-	-	GAAAACCCATTCCACAACCTT	GGGTGGTGTTTGGATGAGAG	258	P	A	A	NA
PMS3703	Os_Chr07	1011985	1012002	(CGG)6	(CGG)6	(CGG)6	(CGG)6	(CGG)6	(CGG)6	(CGG)6	(CGG)6	(CGG)6	(CGG)8	(CGG)6	LOC_Os07g02770	CDS	OsFBL32 - F-box domain and LRR containing protein, expressed	CODON_INSERTION	ACCCAAGTCGATGGAGGAC	GGAGGAGGTACCAGAGGGAG	212	A	A	A	NA
PMS3704	Os_Chr07	1069574	1069591	(CCT)6	(CCT)5	(CCT)6	(CCT)6	(CCT)6	(CCT)6	(CCT)6	(CCT)6	(CCT)6	(CCT)8	(CCT)6	LOC_Os07g02860	CDS	expressed protein	CODON_INSERTION	CCCCCTCTAGGTCTTTCTGG	CGAACGATGAGGAAGAGGAG	189	P	A	A	NA

Polymorphic SSR (PMS) markers	Chromosomes	Start physical positions (bp)	End physical positions (bp)	Nipponbare	Kasalath	Nagina22	IR64	Pokkali	Bala	Tainung67	Azucena	Moroberekan	*Oryza rufipogon*	*Oryza nivara*	MSU gene locus IDs	Structural annotation	Functional annotation	SSR functional effects	Forward primers (5'-3')	Reverse primers (5'-3')	Amplified product size (bp)	Markers exhibiting polymorphism within *indica* rice	Transcrption factor genes-derived markers	Known cloned genes-derived markers	PMS markers corresponding with RM markers
PMS3705	Os_Chr07	1103352	1103379	(TGTA)7	(TGTA)7	(TGTA)7	(TGTA)7	(TGTA)7	(TGTA)7	(TGTA)7	(TGTA)7	(TGTA)7	(TGTA)9	(TGTA)7	LOC_Os07g02960	URR	expressed protein	-	CCTCCTTTCTCACTCACCTTCTATCC	TTGGCCATTTGATCGTGATAGG	546	A	A	A	RM7454
PMS3706	Os_Chr07	1134860	1134874	(TCA)5	(TCA)5	(TCA)5	(TCA)5	(TCA)5	(TCA)5	(TCA)5	(TCA)5	(TCA)5	(TCA)6	(TCA)5	LOC_Os07g03020	CDS	expressed protein	CODON_INSERTION	GAGGACCCACCATTCAGAGA	AGCAGGGGTGTCCTCCTATC	195	A	A	A	NA
PMS3707	Os_Chr07	1194970	1194990	(CGG)7	(CGG)7	(CGG)7	(CGG)7	(CGG)7	(CGG)7	(CGG)7	(CGG)7	(CGG)7	(CGG)7	(CGG)5	LOC_Os07g03120	CDS	expressed protein	CODON_CHANGE_PLUS_CODON_DELETION	ACGCGTCTGATTACTGCAAGAGC	AGTTCAATTGTGTCGTCGTCTCG	176	A	A	A	RM20868
PMS3708	Os_Chr07	1245744	1245770	(CCG)9	(CCG)9	(CCG)7	(CCG)9	(CCG)9	(CCG)9	(CCG)9	(CCG)9	(CCG)9	(CCG)9	(CCG)9	LOC_Os07g03180	CDS	GCRP3 - Glycine and cysteine rich family protein precursor, expressed	CODON_DELETION	TAGAGAGGGCCATCGATTCTTCG	CTGCTACAAGAAGCGGCTCACC	184	P	A	A	RM6223
PMS3709	Os_Chr07	1255678	1255695	(CTT)6	(CTT)6	(CTT)6	(CTT)6	(CTT)6	(CTT)6	(CTT)6	(CTT)6	(CTT)6	(CTT)5	(CTT)6	-	Introns/Intergenic	-	-	ACGAGATGAGGTCGAGGAGA	GCTCCTCAAACGAGACCAAG	220	A	A	A	NA
PMS3710	Os_Chr07	1257660	1257680	(AAG)7	(AAG)5	(AAG)7	(AAG)7	(AAG)5	(AAG)5	(AAG)7	(AAG)7	(AAG)7	(AAG)7	(AAG)5	-	Introns/Intergenic	-	-	GAGGTGCTCTCCCTTTCTCC	GCATGTGGCACCCTCTCTAT	161	P	A	A	NA
PMS3711	Os_Chr07	1281481	1281498	(GGC)6	(GGC)6	(GGC)5	(GGC)6	(GGC)6	(GGC)6	(GGC)6	(GGC)6	(GGC)6	(GGC)6	(GGC)6	LOC_Os07g03240	CDS	RNA recognition motif containing protein, putative, expressed	CODON_DELETION	GGTGATGTGTTGTTGTACTGTGACG	GGTTCGAGATCTTCCTCCTCAGC	103	P	A	A	RM20874
PMS3712	Os_Chr07	1307408	1307425	(GCC)6	(GCC)6	(GCC)6	(GCC)6	(GCC)6	(GCC)6	(GCC)6	(GCC)6	(GCC)6	(GCC)5	(GCC)6	LOC_Os07g03260	CDS	CSLC10 - cellulose synthase-like family C, expressed	CODON_DELETION	GGGCAATTTGTGAGAGGTGT	GTGCTCTTCCTCGTCCAGAG	236	A	A	A	NA
PMS3713	Os_Chr07	1341916	1341929	(CT)7	(CT)7	(CT)7	(CT)7	(CT)7	(CT)7	(CT)7	(CT)7	(CT)7	(CT)7	(CT)6	-	Introns/Intergenic	-	-	ATCTTGATCGGCCTCTCCTT	CGATCGAAGTCGGTCGTC	225	A	A	A	NA
PMS3714	Os_Chr07	1375069	1375082	(CT)7	(CT)7	(CT)7	(CT)7	(CT)7	(CT)7	(CT)7	(CT)7	(CT)7	(CT)7	(CT)6	-	Introns/Intergenic	-	-	ATCTTGATCGGCCTCTCCTT	CGATCGAAGTCGGTCGTC	225	A	A	A	NA
PMS3715	Os_Chr07	1431048	1431065	(GA)9	(GA)9	(GA)9	(GA)7	(GA)9	(GA)9	(GA)9	(GA)9	(GA)9	(GA)9	(GA)9	-	Introns/Intergenic	-	-	AGGGAGCAGAGAGGGAAGAC	CAAAAGTCCATTTTGCCTCC	280	P	A	A	NA
PMS3716	Os_Chr07	1466960	1466979	(CT)10	(CT)10	(CT)10	(CT)10	(CT)10	(CT)10	(CT)10	(CT)10	(CT)10	(CT)10	(CT)9	-	Introns/Intergenic	-	-	CTATCGTCGAGTTCAATTCC	AGAAACGCGCCGGGGAAAG	159	A	A	A	NA
PMS3717	Os_Chr07	1476083	1476097	(GAC)5	(GAC)6	(GAC)5	(GAC)5	(GAC)5	(GAC)5	(GAC)5	(GAC)5	(GAC)5	(GAC)5	(GAC)5	-	Introns/Intergenic	-	-	GAGAACGGCTTGACGGAG	GTCTTCACCGTTCATCCTCG	208	P	A	A	NA

Polymorphic SSR (PMS) markers	Chromosomes	Start physical positions (bp)	End physical positions (bp)	Nipponbare	Kasalath	Nagina22	IR64	Pokkali	Bala	Tainung67	Azucena	Moroberekan	*Oryza rufipogon*	*Oryza nivara*	MSU gene locus IDs	Structural annotation	Functional annotation	SSR functional effects	Forward primers (5'-3')	Reverse primers (5'-3')	Amplified product size (bp)	Markers exhibiting polymorphism within *indica* rice	Transcrption factor genes-derived markers	Known cloned genes-derived markers	PMS markers corresponding with RM markers
PMS3718	Os_Chr07	1507635	1507646	(CT)6	(CT)8	(CT)6	(CT)6	(CT)6	(CT)6	(CT)6	(CT)6	(CT)6	(CT)6	(CT)6	-	Introns/Intergenic	-	-	CCCTCCTCACCACTTCAAAA	GCGCAAAGGAGAGAAAGAGA	146	P	A	A	NA
PMS3719	Os_Chr07	1524432	1524455	(TA)12	(TA)12	(TA)12	(TA)8	(TA)12	(TA)12	(TA)12	(TA)12	(TA)12	(TA)12	(TA)12	LOC_Os07g03730	DRR	SCP-like extracellular protein, expressed	-	GTGGATGTACGTATAAGCATGTGTGC	GTACGGCTGACGGCTTTATTCC	108	P	A	A	RM20891
PMS3720	Os_Chr07	1530516	1530547	(GA)16	(GA)16	(GA)16	(GA)16	(GA)14	(GA)16	(GA)16	(GA)16	(GA)16	(GA)16	(GA)16	-	Introns/Intergenic	-	-	GCGAGAGAGAAACAGAGGGA	TCTATTTTGCCTCCTCCGTG	173	P	A	A	NA
PMS3721	Os_Chr07	1545616	1545633	(TC)9	(TC)9	(TC)9	(TC)9	(TC)9	(TC)10	(TC)9	(TC)9	(TC)9	(TC)9	(TC)9	-	Introns/Intergenic	-	-	TTAGAGTTGACAGGGGGTGG	ATCACGAGAGGCTAGCTGGA	187	P	A	A	NA
PMS3722	Os_Chr07	1550729	1550754	(CT)13	(CT)13	(CT)13	(CT)13	(CT)6	(CT)13	(CT)13	(CT)13	(CT)13	(CT)13	(CT)13	-	Introns/Intergenic	-	-	CTTGCTTCACAGAATCATCACTGC	AACTGTCCGAGGGAAGAAAGAGC	141	P	A	A	RM20894
PMS3723	Os_Chr07	1721864	1721885	(TA)11	(TA)11	(TA)8	(TA)11	(TA)11	(TA)8	(TA)11	(TA)11	(TA)11	(TA)10	(TA)11	-	Introns/Intergenic	-	-	CCACCCTGCAAAGATCCTAA	TTGTCTATCCTGGATTCGCC	178	P	A	A	NA
PMS3724	Os_Chr07	1758045	1758062	(GAC)6	(GAC)6	(GAC)6	(GAC)6	(GAC)6	(GAC)6	(GAC)6	(GAC)6	(GAC)6	(GAC)6	(GAC)5	-	Introns/Intergenic	-	-	AAGAATTGGTGACGGACTCG	AAGAAACGAACCTGGTGGTG	207	A	A	A	NA
PMS3725	Os_Chr07	1799739	1799758	(AT)10	(AT)9	(AT)10	(AT)10	(AT)10	(AT)9	(AT)10	(AT)10	(AT)10	(AT)10	(AT)10	-	Introns/Intergenic	-	-	CACGTGTTCAAGAGCGAAAA	AGGTGAAGAAAGGCAGGACA	193	P	A	A	NA
PMS3726	Os_Chr07	1832642	1832701	(TA)30	(TA)30	(TA)8	(TA)30	(TA)30	(TA)6	(TA)30	(TA)30	(TA)30	(TA)11	(TA)30	-	Introns/Intergenic	-	-	ATGGTGCCCCATATGAGTGT	GGGTTGAATCATTTCCTTTCC	126	P	A	A	NA
PMS3727	Os_Chr07	1863767	1863780	(CA)7	(CA)6	(CA)7	(CA)7	(CA)6	(CA)7	(CA)7	(CA)7	(CA)7	(CA)7	(CA)7	-	Introns/Intergenic	-	-	GAAGAGGAAGCTACAGTGCCA	AATCTGGTGCAAAGCGAAAC	141	P	A	A	NA
PMS3728	Os_Chr07	1877642	1877657	(AG)8	(AG)8	(AG)8	(AG)8	(AG)8	(AG)8	(AG)8	(AG)8	(AG)8	(AG)7	(AG)8	-	Introns/Intergenic	-	-	ATACTAGGCCGGAGCCAGTT	CTAGGGTTTTGGGTTGGGTT	246	A	A	A	NA
PMS3729	Os_Chr07	1905646	1905671	(TC)13	(TC)13	(TC)13	(TC)13	(TC)13	(TC)13	(TC)13	(TC)13	(TC)14	(TC)14	(TC)13	-	Introns/Intergenic	-	-	CATGACCTTCCTCGCAAGAG	CACACAAGGCTCTCAACCAA	182	A	A	A	NA
PMS3730	Os_Chr07	1915501	1915576	(AT)38	(AT)38	(AT)38	(AT)9	(AT)29	(AT)38	(AT)38	(AT)38	(AT)38	(AT)38	(AT)38	-	Introns/Intergenic	-	-	GGTGACATTTGTTAGTTGGTGTGC	AACGACTGTCGGTTAAACGTTGC	435	P	A	A	RM20925

Polymorphic SSR (PMS) markers	Chromosomes	Start physical positions (bp)	End physical positions (bp)	Nipponbare	Kasalath	Nagina22	IR64	Pokkali	Bala	Tainung67	Azucena	Moroberekan	*Oryza rufipogon*	*Oryza nivara*	MSU gene locus IDs	Structural annotation	Functional annotation	SSR functional effects	Forward primers (5'-3')	Reverse primers (5'-3')	Amplified product size (bp)	Markers exhibiting polymorphism within *indica* rice	Transcrption factor genes-derived markers	Known cloned genes-derived markers	PMS markers corresponding with RM markers
PMS3731	Os_Chr07	2063386	2063412	(CTC)9	(CTC)9	(CTC)9	(CTC)9	(CTC)9	(CTC)9	(CTC)9	(CTC)9	(CTC)9	(CTC)9	(CTC)8	LOC_Os07g04650	CDS	zinc finger C-x8-C-x5-C x3-H type family protein, expressed	CODON_DELETION	GTCGACGAGTAGGT CGAGGT	GGAGGATAGTGCCG ACCAC	223	A	P	A	NA
PMS3732	Os_Chr07	2152497	2152517	(GGC)7	(GGC)7	(GGC)7	(GGC)7	(GGC)7	(GGC)7	(GGC)7	(GGC)7	(GGC)7	(GGC)7	(GGC)5	-	Introns/Intergenic	-	-	CGCTCGCTTTACAG GATCAATGG	GGATCCGGGAGCTC AAATCG	395	A	A	A	RM20935
PMS3733	Os_Chr07	2197848	2197871	(CGG)8	(CGG)8	(CGG)5	(CGG)8	(CGG)8	(CGG)8	(CGG)8	(CGG)8	(CGG)8	(CGG)8	(CGG)8	LOC_Os07g05000	CDS	oxidoreductase, aldo/keto reductase family protein, putative, expressed	CODON_CHANGE_P LUS_CODON_DELE TION	GCAACAGTGAGGA GGTAGCTTGC	ACTCGTTCCAGTAGG TGGTGTCG	263	P	A	A	RM20937
PMS3734	Os_Chr07	2217632	2217701	(TA)35	(TA)35	(TA)35	(TA)35	(TA)35	(TA)16	(TA)6	(TA)35	(TA)35	(TA)17	(TA)35	-	Introns/Intergenic	-	-	GGAATAAAGTGGAG AGGGGC	TTGCATGTCAATTTG ATGGG	250	P	A	A	NA
PMS3735	Os_Chr07	2238921	2238936	(AG)8	(AG)8	(AG)8	(AG)8	(AG)8	(AG)12	(AG)8	(AG)8	(AG)8	(AG)8	(AG)8	-	Introns/Intergenic	-	-	CGCCGTCTAAAGGA GAAGAG	AACCGGATATTCTTC ACCCC	182	P	A	A	NA
PMS3736	Os_Chr07	2255306	2255321	(TA)8	(TA)8	(TA)8	(TA)8	(TA)8	(TA)8	(TA)8	(TA)8	(TA)8	(TA)6	(TA)8	-	Introns/Intergenic	-	-	GGTCGATCCCTATG GCTGTA	GACAAACGATCAAAC ATGTGC	206	A	A	A	NA
PMS3737	Os_Chr07	2257404	2257421	(GGC)6	(GGC)5	(GGC)6	(GGC)6	(GGC)6	(GGC)6	(GGC)6	(GGC)6	(GGC)6	(GGC)6	(GGC)6	LOC_Os07g05110	CDS	PLA IIIA/PLP7, putative, expressed	CODON_DELETION	CTCTCCCGCCTTTT TCTTCT	ACCCTGTTCCCTCTC GACAC	226	P	A	A	NA
PMS3738	Os_Chr07	2404680	2404693	(AG)7	(AG)7	(AG)7	(AG)7	(AG)7	(AG)6	(AG)7	(AG)7	(AG)7	(AG)7	(AG)7	-	Introns/Intergenic	-	-	CGTTCGAGCTGAGA CCGT	AGCGGTTTTGTCCTA TGTGG	237	P	A	A	NA
PMS3739	Os_Chr07	2419737	2419788	(AT)26	(AT)25	(AT)26	(AT)26	(AT)26	(AT)10	(AT)26	(AT)9	(AT)26	(AT)9	(AT)10	-	Introns/Intergenic	-	-	TGAGATCCTGTGAT TGACGC	TTAGGCCTCAGGGG TTACCT	259	P	A	A	NA
PMS3740	Os_Chr07	2436280	2436293	(AT)7	(AT)7	(AT)7	(AT)7	(AT)6	(AT)7	(AT)7	(AT)7	(AT)7	(AT)7	(AT)7	-	Introns/Intergenic	-	-	TGAGTGACTGCTCT GTTGCC	AAAAGTCAACGGCGT CAAAT	142	P	A	A	NA
PMS3741	Os_Chr07	2619885	2619956	(AT)36	(AT)36	(AT)6	(AT)36	(AT)36	(AT)36	(AT)36	(AT)36	(AT)36	(AT)14	(AT)7	-	Introns/Intergenic	-	-	GCTTGTACTGCCTC CATTTCAGG	CTCCACGCGTTATAA GTGTCTGC	464	P	A	A	RM20961
PMS3742	Os_Chr07	2833740	2833759	(TA)10	(TA)10	(TA)6	(TA)10	(TA)8	(TA)6	(TA)10	(TA)10	(TA)10	(TA)8	(TA)10	-	Introns/Intergenic	-	-	GCACTCCATTACAA GATCGTTTGC	AGAAAGGAGCGACG GGAAGG	549	P	A	A	RM20980
PMS3743	Os_Chr07	2848025	2848050	(TA)13	(TA)13	(TA)7	(TA)13	(TA)13	(TA)13	(TA)13	(TA)13	(TA)13	(TA)13	(TA)13	-	Introns/Intergenic	-	-	ATGGTGCCCCATAT GAGTGT	ACCCGAAACCTTGAT GTGAG	106	P	A	A	NA

Polymorphic SSR (PMS) markers	Chromosomes	Start physical positions (bp)	End physical positions (bp)	Nipponbare	Kasalath	Nagina22	IR64	Pokkali	Bala	Tainung67	Azucena	Moroberekan	*Oryza rufipogon*	*Oryza nivara*	MSU gene locus IDs	Structural annotation	Functional annotation	SSR functional effects	Forward primers (5'-3')	Reverse primers (5'-3')	Amplified product size (bp)	Markers exhibiting polymorphism within *indica* rice	Transcrption factor genes-derived markers	Known cloned genes-derived markers	PMS markers corresponding with RM markers
PMS3744	Os_Chr07	2860660	2860675	(TG)8	(TG)8	(TG)8	(TG)8	(TG)8	(TG)8	(TG)8	(TG)8	(TG)8	(TG)7	(TG)8	-	Introns/Intergenic	-	-	GGTTAGTTTGTCGGGCTCAA	CAACCGTATCAAATGCGATG	135	A	A	A	NA
PMS3745	Os_Chr07	2866958	2866972	(CAG)5	(CAG)5	(CAG)5	(CAG)5	(CAG)5	(CAG)5	(CAG)5	(CAG)5	(CAG)6	(CAG)5	(CAG)5	-	Introns/Intergenic	-	-	AAGAAATGAAAATGTGCCGC	CCATGCACGCGTAGATTAAC	248	A	A	A	NA
PMS3746	Os_Chr07	2880556	2880597	(TA)21	(TA)21	(TA)21	(TA)21	(TA)21	(TA)21	(TA)8	(TA)21	(TA)6	(TA)21	(TA)9	-	Introns/Intergenic	-	-	TGCATGAGTTGATGGATAAAAGA	CTCGAGGCTACCTGCTGAGT	225	A	A	A	NA
PMS3747	Os_Chr07	2942739	2942759	(CGC)7	(CGC)7	(CGC)7	(CGC)7	(CGC)7	(CGC)7	(CGC)7	(CGC)7	(CGC)7	(CGC)7	(CGC)5	LOC_Os07g06060	URR	expressed protein	-	CCGAGATAGCAAAAACCCAA	AACACACACAGCCCATTTCA	202	A	A	A	NA
PMS3748	Os_Chr07	3002510	3002525	(CT)8	(CT)8	(CT)8	(CT)8	(CT)7	(CT)7	(CT)8	(CT)8	(CT)8	(CT)8	(CT)8	LOC_Os07g06166	CDS	expressed protein	FRAME_SHIFT	GTCATTCGGTGGTTTTGACC	GGTTTGCCCAGAGAAGAGAA	170	P	A	A	NA
PMS3749	Os_Chr07	3036432	3036447	(CT)8	(CT)8	(CT)8	(CT)8	(CT)7	(CT)7	(CT)8	(CT)8	(CT)8	(CT)8	(CT)8	LOC_Os07g06250	CDS	expressed protein	FRAME_SHIFT	GTCATTCGGTGGTTTTGACC	GGTTTGCCCAGAGAAGAGAA	170	P	A	A	NA
PMS3750	Os_Chr07	3081269	3081284	(CT)8	(CT)8	(CT)8	(CT)8	(CT)7	(CT)7	(CT)8	(CT)8	(CT)8	(CT)8	(CT)8	LOC_Os07g06345	CDS	expressed protein	FRAME_SHIFT	GTCATTCGGTGGTTTTGACC	GGTTTGCCCAGAGAAGAGAA	170	P	A	A	NA
PMS3751	Os_Chr07	3180630	3180647	(CGA)6	(CGA)5	(CGA)6	(CGA)6	(CGA)6	(CGA)5	(CGA)5	(CGA)5	(CGA)5	(CGA)5	(CGA)5	LOC_Os07g06540	CDS	zinc finger, C3HC4 type domain containing protein, expressed	CODON_CHANGE_PLUS_CODON_DELETION	ATCACGCAGCTCTGCAAAC	CACGTGAAGATGCAGTCCC	269	P	A	A	NA
PMS3752	Os_Chr07	3189016	3189030	(GCC)5	(GCC)5	(GCC)5	(GCC)5	(GCC)5	(GCC)6	(GCC)5	(GCC)5	(GCC)5	(GCC)5	(GCC)5	LOC_Os07g06560	CDS	zinc finger, C3HC4 type domain containing protein, expressed	CODON_INSERTION	CGCTCGCCATCTACTTCTG	GTAGTTCTCCAGGCACACGG	230	P	A	A	NA
PMS3753	Os_Chr07	3263134	3263179	(AT)23	(AT)23	(AT)23	(AT)9	(AT)8	(AT)23	(AT)23	(AT)23	(AT)15	(AT)23	(AT)23	-	Introns/Intergenic	-	-	TCCGTCGAAGAATTTGAACA	GGAACGGAACCTTCAGAGTG	242	P	A	A	NA
PMS3754	Os_Chr07	3283150	3283187	(TA)19	(TA)19	(TA)7	(TA)19	(TA)19	(TA)11	(TA)19	(TA)19	(TA)19	(TA)19	(TA)10	-	Introns/Intergenic	-	-	TGCAAAATTGCAACTCGATG	GTGTGACCTTTTTGGGGTCT	261	P	A	A	NA
PMS3755	Os_Chr07	3298586	3298612	(TTC)9	(TTC)6	(TTC)6	(TTC)6	(TTC)6	(TTC)6	(TTC)9	(TTC)9	(TTC)9	(TTC)9	(TTC)6	-	Introns/Intergenic	-	-	GCTCCATAGCTTATTGTCACACACC	GGCGTTAAGTGTCCGGTATTGC	247	A	A	A	RM21019
PMS3756	Os_Chr07	3304067	3304086	(TA)10	(TA)10	(TA)10	(TA)10	(TA)7	(TA)10	(TA)10	(TA)10	(TA)10	(TA)10	(TA)6	-	Introns/Intergenic	-	-	TAGTCCCGGTTCGTGTTACC	CACGTGCCATTTAATTCGTG	183	P	A	A	NA

Polymorphic SSR (PMS) markers	Chromosomes	Start physical positions (bp)	End physical positions (bp)	Nipponbare	Kasalath	Nagina22	IR64	Pokkali	Bala	Tainung67	Azucena	Moroberekan	*Oryza rufipogon*	*Oryza nivara*	MSU gene locus IDs	Structural annotation	Functional annotation	SSR functional effects	Forward primers (5'-3')	Reverse primers (5'-3')	Amplified product size (bp)	Markers exhibiting polymorphism within *indica* rice	Transcrption factor genes-derived markers	Known cloned genes-derived markers	PMS markers corresponding with RM markers
PMS3757	Os_Chr07	3312296	3312323	(AT)14	(AT)14	(AT)14	(AT)14	(AT)14	(AT)14	(AT)14	(AT)14	(AT)14	(AT)14	(AT)6	-	Introns/Intergenic	-	-	ACCTTTTAGGCACCTCCGAT	CGGTTGGTGGAGATCGTACT	264	A	A	A	NA
PMS3758	Os_Chr07	3389262	3389279	(GT)9	(GT)9	(GT)9	(GT)9	(GT)9	(GT)9	(GT)9	(GT)9	(GT)9	(GT)8	(GT)8	-	Introns/Intergenic	-	-	CATGATTTGGTCCAGTTCCA	GGCAAACAAAGCAAGTAGCA	190	A	A	A	NA
PMS3759	Os_Chr07	3458618	3458632	(TAA)5	(TAA)6	(TAA)5	(TAA)5	(TAA)5	(TAA)5	(TAA)5	(TAA)5	(TAA)5	(TAA)6	(TAA)5	-	Introns/Intergenic	-	-	AAAGATTCAGGAGTTGGCGA	TACCACCCCATCACTCCATT	243	P	A	A	NA
PMS3760	Os_Chr07	3505574	3505594	(CCG)7	(CCG)7	(CCG)7	(CCG)7	(CCG)7	(CCG)7	(CCG)7	(CCG)5	(CCG)7	(CCG)7	(CCG)7	LOC_Os07g07094	CDS	expressed protein	CODON_DELETION	TACGTCCTTTTTGCTCTGGC	AGGAGGGTGCGATTATTGGT	241	A	A	A	NA
PMS3761	Os_Chr07	3538397	3538416	(TA)10	(TA)10	(TA)10	(TA)10	(TA)10	(TA)6	(TA)10	(TA)10	(TA)10	(TA)10	(TA)10	-	Introns/Intergenic	-	-	GTGTGGTCCAATCACATAGAAGG	GAAACGATCGAACTCCTAACTGC	300	P	A	A	RM21033
PMS3762	Os_Chr07	3555717	3555746	(TC)15	(TC)10	(TC)15	(TC)15	(TC)15	(TC)15	(TC)15	(TC)15	(TC)15	(TC)15	(TC)15	-	Introns/Intergenic	-	-	CACCTCAACCGATAGAGTGAACC	TCTATTTCGGGAGGGATTATGACC	144	P	A	A	RM1243
PMS3763	Os_Chr07	3593803	3593826	(CT)12	(CT)12	(CT)12	(CT)12	(CT)12	(CT)12	(CT)12	(CT)6	(CT)12	(CT)12	(CT)12	LOC_Os07g07240	URR	ubiquitin-conjugating enzyme, putative, expressed	-	AGCTAGGGTTTCGATCTCCC	ACACCCAACTTTTCTCACGC	144	A	A	A	NA
PMS3764	Os_Chr07	3623155	3623168	(AG)7	(AG)7	(AG)7	(AG)7	(AG)7	(AG)7	(AG)7	(AG)6	(AG)7	(AG)7	(AG)7	-	Introns/Intergenic	-	-	AGGGAGGGAAGAACGAACAG	ATACCGGACACTTAACCCCC	171	A	A	A	NA
PMS3765	Os_Chr07	3668931	3668951	(GCT)7	(GCT)7	(GCT)7	(GCT)7	(GCT)7	(GCT)7	(GCT)6	(GCT)7	(GCT)7	(GCT)7	(GCT)7	LOC_Os07g07340	URR	glucan endo-1,3-beta-glucosidase precursor, putative, expressed	-	AAGAACCTGACGCCAGCTAA	TATCGAGCAAACACCAAGCA	263	A	A	A	NA
PMS3766	Os_Chr07	3678046	3678063	(CAA)6	(CAA)5	(CAA)6	(CAA)6	(CAA)6	(CAA)6	(CAA)6	(CAA)6	(CAA)6	(CAA)5	(CAA)6	-	Introns/Intergenic	-	-	AGGTCAACCTCCGAACACAC	CCCCTCTCCTCGTTCATCTT	256	P	A	A	NA
PMS3767	Os_Chr07	3698381	3698412	(TA)16	(TA)16	(TA)6	(TA)16	(TA)9	(TA)16	(TA)16	(TA)16	(TA)16	(TA)16	(TA)16	-	Introns/Intergenic	-	-	ACATGCGCACAGTTTCAATG	TTGAGGGCTCAAGACGAACT	219	P	A	A	NA
PMS3768	Os_Chr07	3707660	3707680	(GCC)7	(GCC)7	(GCC)7	(GCC)7	(GCC)7	(GCC)7	(GCC)7	(GCC)7	(GCC)7	(GCC)7	(GCC)6	LOC_Os07g07430	CDS	CAF1 family ribonuclease containing protein, putative, expressed	CODON_DELETION	AACAGCGACTCGATCAGGTT	GCTGTTCTCCTACTCACCGC	246	A	A	A	NA
PMS3769	Os_Chr07	3710793	3710813	(GCG)7	(GCG)7	(GCG)7	(GCG)7	(GCG)7	(GCG)6	(GCG)5	(GCG)7	(GCG)7	(GCG)7	(GCG)7	-	Introns/Intergenic	-	-	TCTTCCTCAACGGCACTTCT	CTAGATCTGGGAGGGGAGGG	200	P	A	A	NA

Polymorphic SSR (PMS) markers	Chromosomes	Start physical positions (bp)	End physical positions (bp)	Nipponbare	Kasalath	Nagina22	IR64	Pokkali	Bala	Tainung67	Azucena	Moroberekan	*Oryza rufipogon*	*Oryza nivara*	MSU gene locus IDs	Structural annotation	Functional annotation	SSR functional effects	Forward primers (5'-3')	Reverse primers (5'-3')	Amplified product size (bp)	Markers exhibiting polymorphism within *indica* rice	Transcrption factor genes-derived markers	Known cloned genes-derived markers	PMS markers corresponding with RM markers
PMS3770	Os_Chr07	3711012	3711029	(GGC)6	(GGC)5	(GGC)6	(GGC)6	(GGC)6	(GGC)6	(GGC)6	(GGC)6	(GGC)6	(GGC)6	(GGC)6	-	Introns/Intergenic	-	-	CTCCCAGATCTAGCCGGAG	GACCACATTCCTCGCTGC	249	P	A	A	NA
PMS3771	Os_Chr07	3738969	3738992	(CCT)8	(CCT)6	(CCT)8	(CCT)8	(CCT)8	(CCT)8	(CCT)8	(CCT)8	(CCT)8	(CCT)8	(CCT)5	-	Introns/Intergenic	-	-	GGAGGAGATAACCAGCCACA	CCTCTCCCCTATCGGATCTC	196	P	A	A	NA
PMS3772	Os_Chr07	3741140	3741155	(AG)8	(AG)8	(AG)8	(AG)8	(AG)8	(AG)8	(AG)8	(AG)6	(AG)8	(AG)8	(AG)8	-	Introns/Intergenic	-	-	CATGTTTGACCGTTCGTTTT	TTCTTGGAGAATCCTCACCG	249	A	A	A	NA
PMS3773	Os_Chr07	3745618	3745629	(CT)6	(CT)6	(CT)6	(CT)6	(CT)6	(CT)6	(CT)6	(CT)6	(CT)6	(CT)7	(CT)7	-	Introns/Intergenic	-	-	GAGGAGGAAGAGGAGGAGGA	CGTGCAGGCTGTAACGAGAG	218	A	A	A	NA
PMS3774	Os_Chr07	3760269	3760295	(CGT)9	(CGT)9	(CGT)9	(CGT)9	(CGT)9	(CGT)9	(CGT)8	(CGT)9	(CGT)9	(CGT)9	(CGT)9	LOC_Os07g07540	CDS	SHOOT1 protein, putative, expressed	CODON_DELETION	CACTTCTTGGCCGGGACT	TACGGCTTCAGGATCTCCAC	193	A	A	A	NA
PMS3775	Os_Chr07	3800267	3800284	(TTG)6	(TTG)6	(TTG)6	(TTG)6	(TTG)6	(TTG)6	(TTG)6	(TTG)6	(TTG)6	(TTG)6	(TTG)5	-	Introns/Intergenic	-	-	TGAATGTCGCCCATTGTTTA	CGCTATCATCGTCCACATTG	248	A	A	A	NA
PMS3776	Os_Chr07	3823325	3823362	(TA)19	(TA)9	(TA)19	(TA)19	(TA)19	(TA)19	(TA)19	(TA)19	(TA)19	(TA)19	(TA)19	-	Introns/Intergenic	-	-	AGCCGGTTTTTAAGTTCGGT	TGCAACGGGTGGAAACTATT	186	P	A	A	NA
PMS3777	Os_Chr07	3823650	3823669	(AT)10	(AT)10	(AT)10	(AT)10	(AT)10	(AT)7	(AT)10	(AT)10	(AT)7	(AT)10	(AT)10	-	Introns/Intergenic	-	-	ACTCCATCCGTTTCACAATG	CCTGCAAAAACTTCGTCACA	209	P	A	A	NA
PMS3778	Os_Chr07	3913390	3913403	(CT)7	(CT)6	(CT)7	(CT)7	(CT)7	(CT)7	(CT)7	(CT)7	(CT)7	(CT)7	(CT)7	-	Introns/Intergenic	-	-	TAGGTCCTTCGGTGGTTTTG	TGAGAGAGAAAGGGGGAAGG	148	P	A	A	NA
PMS3779	Os_Chr07	3928290	3928303	(GA)7	(GA)8	(GA)7	(GA)7	(GA)7	(GA)8	(GA)7	(GA)7	(GA)8	(GA)7	(GA)7	-	Introns/Intergenic	-	-	AAAACCATCAAAAGCCATGC	GGAGGTGCGTTCGTAGGTTA	220	P	A	A	NA
PMS3780	Os_Chr07	3981669	3981692	(CGG)8	(CGG)8	(CGG)8	(CGG)8	(CGG)8	(CGG)8	(CGG)8	(CGG)8	(CGG)8	(CGG)7	(CGG)8	-	Introns/Intergenic	-	-	AGATCGAGCTGACGAAGACAACG	TGTGTGAGAGAGTCGTGGAGAGG	167	A	A	A	RM21065
PMS3781	Os_Chr07	3984390	3984409	(TA)10	(TA)10	(TA)9	(TA)10	(TA)10	(TA)10	(TA)10	(TA)10	(TA)10	(TA)10	(TA)10	-	Introns/Intergenic	-	-	GTTTTTATGAGACGGCCGAA	ATTTGATACCATGGTGCCCG	149	P	A	A	NA
PMS3782	Os_Chr07	3985520	3985537	(AG)9	(AG)9	(AG)9	(AG)9	(AG)8	(AG)9	(AG)9	(AG)9	(AG)9	(AG)9	(AG)9	-	Introns/Intergenic	-	-	GGGTTGGTTCATCTTGTCGT	AGGAACGAAGTAATGGGGCT	266	P	A	A	NA

Polymorphic SSR (PMS) markers	Chromosomes	Start physical positions (bp)	End physical positions (bp)	Nipponbare	Kasalath	Nagina22	IR64	Pokkali	Bala	Tainung67	Azucena	Moroberekan	*Oryza rufipogon*	*Oryza nivara*	MSU gene locus IDs	Structural annotation	Functional annotation	SSR functional effects	Forward primers (5'-3')	Reverse primers (5'-3')	Amplified product size (bp)	Markers exhibiting polymorphism within *indica* rice	Transcrption factor genes-derived markers	Known cloned genes-derived markers	PMS markers corresponding with RM markers
PMS3783	Os_Chr07	4062295	4062338	(TA)22	(TA)22	(TA)22	(TA)22	(TA)9	(TA)22	(TA)22	(TA)22	(TA)22	(TA)22	(TA)22	-	Introns/Intergenic	-	-	CTTTACCCGTAGCGAAGCAC	CAAGAAAACAGCGTGCAAAA	252	P	A	A	NA
PMS3784	Os_Chr07	4091992	4092015	(GCG)8	(GCG)8	(GCG)8	(GCG)8	(GCG)8	(GCG)8	(GCG)8	(GCG)8	(GCG)8	(GCG)5	(GCG)8	LOC_Os07g08070	URR	transmembrane BAX inhibitor motif-containing protein, putative, expressed	-	CCTCTCCTCTCTCCCAACTCACC	TATCTCACACGGGAAGTCGTTCG	150	A	A	A	RM6111
PMS3785	Os_Chr07	4155868	4155891	(CGC)8	(CGC)8	(CGC)8	(CGC)8	(CGC)8	(CGC)8	(CGC)8	(CGC)8	(CGC)8	(CGC)5	(CGC)8	LOC_Os07g08170	URR	ruvB-like, putative, expressed	-	CGATCCTCTGCTTCTTCGTC	CCCCTCCCTCCTCTTCTTCT	266	A	A	A	NA
PMS3786	Os_Chr07	4221368	4221385	(AGA)6	(AGA)6	(AGA)6	(AGA)6	(AGA)6	(AGA)5	(AGA)6	(AGA)6	(AGA)6	(AGA)6	(AGA)5	LOC_Os07g08270	CDS	expressed protein	CODON_DELETION	CCCATTCCTCGAAACCATAA	GGGTGGTTCACCAAGAGGTA	213	P	A	A	NA
PMS3787	Os_Chr07	4419972	4420015	(CT)22	(CT)22	(CT)8	(CT)22	(CT)22	(CT)22	(CT)22	(CT)22	(CT)22	(CT)22	(CT)22	-	Introns/Intergenic	-	-	CTCTATGAGCAATCGACGCC	ATGGAAGCTGGTGGAGAGG	190	P	A	A	NA
PMS3788	Os_Chr07	4427029	4427076	(AT)24	(AT)24	(AT)24	(AT)24	(AT)24	(AT)16	(AT)24	(AT)24	(AT)24	(AT)24	(AT)24	-	Introns/Intergenic	-	-	GAGGAGGAGGAATTCGAACC	CTTGCATGTTTGTGGAGTGA	230	P	A	A	NA
PMS3789	Os_Chr07	4476499	4476552	(TA)27	(TA)27	(TA)27	(TA)7	(TA)10	(TA)27	(TA)27	(TA)13	(TA)7	(TA)27	(TA)27	-	Introns/Intergenic	-	-	GCATGTGAGGCTTTAACCCA	TCTTTGTCGTCGAAAGGTCC	278	P	A	A	NA
PMS3790	Os_Chr07	4655943	4655968	(TC)13	(TC)13	(TC)13	(TC)13	(TC)13	(TC)13	(TC)13	(TC)7	(TC)13	(TC)13	(TC)13	-	Introns/Intergenic	-	-	TTTTGCTGTCCCTTTGTTCC	AAGTCTTAGGGGATGGGTGG	188	A	A	A	NA
PMS3791	Os_Chr07	4744812	4744827	(AT)8	(AT)8	(AT)8	(AT)6	(AT)6	(AT)8	(AT)8	(AT)8	(AT)8	(AT)8	(AT)8	-	Introns/Intergenic	-	-	CCCATTGGGTCCTGAAATAA	ATTTGGACGACCACGAAAAC	268	P	A	A	NA
PMS3792	Os_Chr07	4754539	4754560	(AG)11	(AG)10	(AG)11	(AG)11	(AG)11	(AG)10	(AG)11	(AG)11	(AG)11	(AG)11	(AG)10	-	Introns/Intergenic	-	-	TGGTGTGCGTCGAAGTCTCTTCC	ACTATCGCGCTCCCAGGTTCG	139	P	A	A	RM21113
PMS3793	Os_Chr07	4779243	4779380	(TA)69	(TA)69	(TA)69	(TA)8	(TA)69	(TA)69	(TA)69	(TA)69	(TA)69	(TA)69	(TA)69	-	Introns/Intergenic	-	-	CACCTTAGAGCATTATTGAATGT	CACCGTTGTGTTCGTAATAAT	446	P	A	A	NA
PMS3794	Os_Chr07	4857346	4857361	(GA)8	(GA)8	(GA)8	(GA)8	(GA)9	(GA)8	(GA)8	(GA)8	(GA)8	(GA)9	(GA)10	-	Introns/Intergenic	-	-	AAAACATGCATGCAGTCCAA	CATGCATGCTCACCTGCTAT	159	P	A	A	NA
PMS3795	Os_Chr07	4895562	4895581	(TA)10	(TA)10	(TA)10	(TA)10	(TA)10	(TA)10	(TA)9	(TA)10	(TA)10	(TA)10	(TA)10	-	Introns/Intergenic	-	-	ATGTCGTCCCCATACGAAAA	CAGATGGGCTAGGGTTTCAA	101	A	A	A	NA

Polymorphic SSR (PMS) markers	Chromosomes	Start physical positions (bp)	End physical positions (bp)	Nipponbare	Kasalath	Nagina22	IR64	Pokkali	Bala	Tainung67	Azucena	Moroberekan	*Oryza rufipogon*	*Oryza nivara*	MSU gene locus IDs	Structural annotation	Functional annotation	SSR functional effects	Forward primers (5'-3')	Reverse primers (5'-3')	Amplified product size (bp)	Markers exhibiting polymorphism within *indica* rice	Transcrption factor genes-derived markers	Known cloned genes-derived markers	PMS markers corresponding with RM markers
PMS3796	Os_Chr07	4914660	4914683	(GCA)8	(GCA)8	(GCA)8	(GCA)8	(GCA)8	(GCA)8	(GCA)5	(GCA)8	(GCA)8	(GCA)8	(GCA)8	LOC_Os07g09340	URR	plasma membrane ATPase, putative, expressed	-	ATATAGGGCAGCAAAGGCTGTGG	CCTCCAGATTGTCACCCTTCTCG	186	A	A	A	RM21131
PMS3797	Os_Chr07	4933571	4933588	(GGC)6	(GGC)6	(GGC)6	(GGC)6	(GGC)5	(GGC)6	(GGC)6	(GGC)6	(GGC)6	(GGC)6	(GGC)6	LOC_Os07g09360	CDS	hypothetical protein	CODON_DELETION	CGACGAAGGAAAGTCTCTGG	GCTTGGGACACTTGTTGGTT	214	P	A	A	NA
PMS3798	Os_Chr07	5000460	5000498	(GAA)13	(GAA)13	(GAA)13	(GAA)13	(GAA)13	(GAA)13	(GAA)13	(GAA)13	(GAA)13	(GAA)12	(GAA)13	LOC_Os07g09470	CDS	ATPase, putative, expressed	CODON_DELETION	AGGAGAAGGCGAAGGAGAAG	TCGGTGAATTGTCCTGTATCA	202	A	A	A	NA
PMS3799	Os_Chr07	5040473	5040490	(AT)9	(AT)9	(AT)9	(AT)7	(AT)8	(AT)9	(AT)9	(AT)9	(AT)9	(AT)7	(AT)7	-	Introns/Intergenic	-	-	CTACCGGGGACAGGTGTCTA	GTCCGTTCTGTGAATCCGTT	237	P	A	A	NA
PMS3800	Os_Chr07	5040658	5040739	(AT)41	(AT)41	(AT)41	(AT)41	(AT)41	(AT)10	(AT)41	(AT)41	(AT)41	(AT)41	(AT)8	-	Introns/Intergenic	-	-	ATCCCTTTTCCCAGCTCATT	TGCATATCGGTAGTTCCACG	216	P	A	A	NA
PMS3801	Os_Chr07	5230637	5230651	(CGC)5	(CGC)6	(CGC)5	(CGC)5	(CGC)5	(CGC)5	(CGC)5	(CGC)5	(CGC)5	(CGC)5	(CGC)5	LOC_Os07g09830	CDS	no apical meristem protein, expressed	CODON_INSERTION	GGTTGTCGCTCTGAGATTCC	ATGGTGAGAGGCTGGTCGT	240	P	P	A	NA
PMS3802	Os_Chr07	5248505	5248522	(CGG)6	(CGG)5	(CGG)6	(CGG)6	(CGG)6	(CGG)6	(CGG)6	(CGG)6	(CGG)6	(CGG)6	(CGG)6	LOC_Os07g09870	CDS	OsFBA2 - F-box and FBA domain containing protein, expressed	CODON_CHANGE_PLUS_CODON_DELETION	AGCAGAGACACATCCCGAGT	CAGGACGTCGAGCAGCAG	272	P	A	A	NA
PMS3803	Os_Chr07	5426175	5426195	(CCT)7	(CCT)7	(CCT)7	(CCT)7	(CCT)7	(CCT)5	(CCT)7	(CCT)7	(CCT)7	(CCT)7	(CCT)7	LOC_Os07g10110	CDS	expressed protein	CODON_DELETION	AAGATCTTGAGCTTGTCGGC	GTGGTGTTGACGGGGGAG	196	P	A	A	NA
PMS3804	Os_Chr07	5440438	5440458	(CGG)7	(CGG)5	(CGG)7	(CGG)7	(CGG)7	(CGG)7	(CGG)7	(CGG)7	(CGG)7	(CGG)7	(CGG)7	LOC_Os07g10130	URR	squalene synthetase, putative, expressed	-	TCGAAGCCGCTAGAAGAAGAAGC	AGTAGGCGAAGGCCCAGTGC	202	P	A	A	RM21154
PMS3805	Os_Chr07	5447338	5447355	(TA)9	(TA)9	(TA)9	(TA)9	(TA)9	(TA)9	(TA)9	(TA)7	(TA)9	(TA)9	(TA)9	-	Introns/Intergenic	-	-	TATGTTTCCATGAACCGCTG	TCCCAATACACATCCCTCGT	276	A	A	A	NA
PMS3806	Os_Chr07	5449230	5449255	(TA)13	(TA)13	(TA)13	(TA)9	(TA)13	(TA)8	(TA)13	(TA)13	(TA)9	(TA)13	(TA)8	-	Introns/Intergenic	-	-	GGGTCATCTATCACCGGAGAGTCC	ATCATCGTCGCCGCCTAATCC	551	P	A	A	RM21155
PMS3807	Os_Chr07	5464093	5464107	(CCT)5	(CCT)5	(CCT)5	(CCT)5	(CCT)5	(CCT)5	(CCT)5	(CCT)5	(CCT)5	(CCT)6	(CCT)5	LOC_Os07g10160	CDS	glucosyltransferase, putative, expressed	CODON_INSERTION	GATTCTGGGAGATGGAGCAC	TCCATGCTAACATCGTCGTC	187	A	A	A	NA
PMS3808	Os_Chr07	5477209	5477230	(AT)11	(AT)11	(AT)11	(AT)11	(AT)11	(AT)11	(AT)11	(AT)6	(AT)11	(AT)11	(AT)11	-	Introns/Intergenic	-	-	CGTACATGCAATGGTCTTGG	CGGCAAAGTACGGGGAATTA	126	A	A	A	NA

Polymorphic SSR (PMS) markers	Chromosomes	Start physical positions (bp)	End physical positions (bp)	Nipponbare	Kasalath	Nagina22	IR64	Pokkali	Bala	Tainung67	Azucena	Moroberekan	*Oryza rufipogon*	*Oryza nivara*	MSU gene locus IDs	Structural annotation	Functional annotation	SSR functional effects	Forward primers (5'-3')	Reverse primers (5'-3')	Amplified product size (bp)	Markers exhibiting polymorphism within *indica* rice	Transcrption factor genes-derived markers	Known cloned genes-derived markers	PMS markers corresponding with RM markers
PMS3809	Os_Chr07	5659669	5659692	(TTC)8	(TTC)8	(TTC)8	(TTC)7	(TTC)8	(TTC)8	(TTC)6	(TTC)8	(TTC)8	(TTC)8	(TTC)8	LOC_Os07g10520	URR	nucleotide binding protein, putative, expressed	-	GTTCTTCCTCCCTTTGCCTCTGC	GGTGCACCTTCTTCTCCATCTCC	189	P	A	A	RM6338
PMS3810	Os_Chr07	5736303	5736332	(TTA)10	(TTA)10	(TTA)10	(TTA)10	(TTA)10	(TTA)10	(TTA)10	(TTA)10	(TTA)10	(TTA)10	(TTA)8	-	Introns/Intergenic	-	-	CCTTCTCCTTCTTTCAGCTTCTGC	CAACTTGCTCTACTTGTGGTGAGG	190	A	A	A	RM180
PMS3811	Os_Chr07	5769837	5769898	(AT)31	(AT)31	(AT)31	(AT)10	(AT)13	(AT)31	(AT)31	(AT)31	(AT)31	(AT)31	(AT)31	-	Introns/Intergenic	-	-	CTCCTCGATCTCAAGCTCAAACC	CATGAGATTTACAGCCGCATCG	305	P	A	A	RM21182
PMS3812	Os_Chr07	5880144	5880159	(CT)8	(CT)8	(CT)8	(CT)9	(CT)9	(CT)8	(CT)8	(CT)8	(CT)8	(CT)8	(CT)8	LOC_Os07g10820	URR	transporter-related, putative, expressed	-	TCAGCTCATCACTCCGTCAC	GCATATGCACCTTGTGGTTG	266	P	A	A	NA
PMS3813	Os_Chr07	5996460	5996480	(CGT)7	(CGT)7	(CGT)7	(CGT)7	(CGT)7	(CGT)7	(CGT)7	(CGT)7	(CGT)7	(CGT)7	(CGT)6	LOC_Os07g10940	CDS	exo70 exocyst complex subunit family protein, expressed	CODON_DELETION	CGGGATACTCAGCAAGACGA	AGCTGGTGACCACGTGTATG	116	A	A	A	NA
PMS3814	Os_Chr07	6035920	6035941	(TC)11	(TC)6	(TC)11	(TC)11	(TC)11	(TC)11	(TC)11	(TC)11	(TC)11	(TC)11	(TC)8	-	Introns/Intergenic	-	-	TATGGCTACACGCCTACACG	GAAGCGTGGGATGTTTGTTT	202	P	A	A	NA
PMS3815	Os_Chr07	6049493	6049520	(TA)14	(TA)14	(TA)14	(TA)14	(TA)14	(TA)14	(TA)14	(TA)14	(TA)14	(TA)14	(TA)10	-	Introns/Intergenic	-	-	GGGAGAGGGAGTACATACATCTGG	CTACATCGGTTGTGTCAATAGCC	149	A	A	A	RM21196
PMS3816	Os_Chr07	6153136	6153153	(AT)9	(AT)9	(AT)9	(AT)9	(AT)9	(AT)9	(AT)9	(AT)9	(AT)6	(AT)9	(AT)9	-	Introns/Intergenic	-	-	ACACGTGTTTTTCGTTGTCG	CTTCCGAATTTCATCCCGTA	247	A	A	A	NA
PMS3817	Os_Chr07	6254082	6254107	(AT)13	(AT)13	(AT)13	(AT)8	(AT)13	(AT)13	(AT)13	(AT)13	(AT)13	(AT)13	(AT)11	-	Introns/Intergenic	-	-	GGGTCAAAGTTAAATTGCTTCAA	CAAAGCACGGCCATTTTACT	276	P	A	A	NA
PMS3818	Os_Chr07	6297558	6297585	(TA)14	(TA)14	(TA)7	(TA)14	(TA)14	(TA)14	(TA)14	(TA)14	(TA)14	(TA)14	(TA)14	-	Introns/Intergenic	-	-	CCGGGCACCATAATATGAAA	CCCATGAAGCCCCAGTTAAT	173	P	A	A	NA
PMS3819	Os_Chr07	6313031	6313054	(TA)12	(TA)12	(TA)12	(TA)12	(TA)12	(TA)12	(TA)6	(TA)12	(TA)12	(TA)12	(TA)12	-	Introns/Intergenic	-	-	ACTCCCTCCGGTTAATTATGACG	GCGGCTAGTGTAACTAATGCATGG	463	A	A	A	RM21209
PMS3820	Os_Chr07	6384541	6384552	(GC)6	(GC)7	(GC)6	(GC)6	(GC)6	(GC)6	(GC)6	(GC)6	(GC)7	(GC)6	(GC)6	-	Introns/Intergenic	-	-	AGTAGACTTCTCTCGGGCGG	CGGACTCTCTCTCCTCCCTC	128	P	A	A	NA
PMS3821	Os_Chr07	6492373	6492390	(TA)9	(TA)9	(TA)9	(TA)9	(TA)9	(TA)9	(TA)7	(TA)9	(TA)9	(TA)9	(TA)9	-	Introns/Intergenic	-	-	AGCAATGGCACAGAATAGCA	CTGCTCTGTGTCACTCCACC	253	A	A	A	NA

Polymorphic SSR (PMS) markers	Chromosomes	Start physical positions (bp)	End physical positions (bp)	Nipponbare	Kasalath	Nagina22	IR64	Pokkali	Bala	Tainung67	Azucena	Moroberekan	*Oryza rufipogon*	*Oryza nivara*	MSU gene locus IDs	Structural annotation	Functional annotation	SSR functional effects	Forward primers (5'-3')	Reverse primers (5'-3')	Amplified product size (bp)	Markers exhibiting polymorphism within *indica* rice	Transcrption factor genes-derived markers	Known cloned genes-derived markers	PMS markers corresponding with RM markers
PMS3822	Os_Chr07	6598676	6598731	(TA)28	(TA)28	(TA)28	(TA)28	(TA)28	(TA)28	(TA)8	(TA)28	(TA)7	(TA)28	(TA)28	-	Introns/Intergenic	-	-	ATGCTAGTACTCTGCTTGCATACC	CCTTCTGTGCAGTTCTGATCG	169	A	A	A	RM21224
PMS3823	Os_Chr07	6730308	6730339	(TA)16	(TA)16	(TA)16	(TA)16	(TA)8	(TA)16	(TA)6	(TA)16	(TA)16	(TA)10	(TA)16	-	Introns/Intergenic	-	-	CCAGGCCTGATACATAAACAGTAGGG	GCGACGTCGGTGGTATAGATGG	454	P	A	A	RM21234
PMS3824	Os_Chr07	6756134	6756155	(CT)11	(CT)11	(CT)11	(CT)11	(CT)11	(CT)10	(CT)11	(CT)11	(CT)11	(CT)11	(CT)11	-	Introns/Intergenic	-	-	TCGAGTTCAATTCCGAATCC	GAGCGGAGAGGGAGGACT	274	P	A	A	NA
PMS3825	Os_Chr07	6760235	6760272	(TA)19	(TA)19	(TA)19	(TA)9	(TA)19	(TA)7	(TA)19	(TA)19	(TA)19	(TA)19	(TA)19	-	Introns/Intergenic	-	-	ATGGCGTTAAACATTTTGGG	CTGCATATCCACTGTCCCCT	275	P	A	A	NA
PMS3826	Os_Chr07	6848202	6848247	(GA)23	(GA)23	(GA)23	(GA)23	(GA)23	(GA)23	(GA)23	(GA)23	(GA)23	(GA)17	(GA)23	-	Introns/Intergenic	-	-	GTCCTCCTCGCTCCACCT	ACCTACCCTTCCAGCGACTT	263	A	A	A	NA
PMS3827	Os_Chr07	6903428	6903441	(TC)7	(TC)7	(TC)7	(TC)7	(TC)7	(TC)7	(TC)7	(TC)7	(TC)7	(TC)7	(TC)6	-	Introns/Intergenic	-	-	ATTTACCGAGCAGGCATGAG	TCCTGCCCGAATCTAGAAGA	199	A	A	A	NA
PMS3828	Os_Chr07	7056960	7056977	(TGG)6	(TGG)6	(TGG)5	(TGG)6	(TGG)6	(TGG)6	(TGG)6	(TGG)6	(TGG)6	(TGG)6	(TGG)6	-	Introns/Intergenic	-	-	GTCGTCGGTGGAGAAGAGAG	AGAAGGACCGCTTGCATAGA	150	P	A	A	NA
PMS3829	Os_Chr07	7314335	7314352	(CGC)6	(CGC)6	(CGC)6	(CGC)5	(CGC)6	(CGC)6	(CGC)6	(CGC)6	(CGC)6	(CGC)6	(CGC)6	LOC_Os07g12780	CDS	cyclin, putative, expressed	CODON_DELETION	GGGAGAAGAAGGGGAGGAC	AATGGTGAACCTAATTGGCG	104	P	A	A	NA
PMS3830	Os_Chr07	7413366	7413385	(GA)10	(GA)10	(GA)10	(GA)10	(GA)10	(GA)11	(GA)10	(GA)10	(GA)10	(GA)10	(GA)10	-	Introns/Intergenic	-	-	CAGACGATGACGATGATGACTGC	ACAGCCTGCTTCCCTCTCTCC	177	P	A	A	RM21264
PMS3831	Os_Chr07	7604621	7604638	(CCG)6	(CCG)6	(CCG)6	(CCG)6	(CCG)6	(CCG)6	(CCG)5	(CCG)6	(CCG)6	(CCG)6	(CCG)6	LOC_Os07g13260	CDS	dof zinc finger domain containing protein, putative, expressed	CODON_DELETION	ACCCATTCTGCATATGCCTC	CGATTGAGTTCCTCGGTCTC	200	A	P	A	NA
PMS3832	Os_Chr07	7711346	7711371	(AT)13	(AT)13	(AT)13	(AT)7	(AT)7	(AT)9	(AT)13	(AT)13	(AT)6	(AT)13	(AT)13	-	Introns/Intergenic	-	-	AGTGTGCGTTCTAGGGGTGT	TACCGCACATCTCATCAGGA	192	P	A	A	NA
PMS3833	Os_Chr07	7721407	7721424	(GCC)6	(GCC)6	(GCC)6	(GCC)6	(GCC)6	(GCC)6	(GCC)5	(GCC)6	(GCC)6	(GCC)6	(GCC)6	-	Introns/Intergenic	-	-	AGCCTGGTCCCTAGGTGAAT	ATCAAATAGCAACAGGGGCA	177	A	A	A	NA
PMS3834	Os_Chr07	7881204	7881221	(GTC)6	(GTC)6	(GTC)6	(GTC)6	(GTC)6	(GTC)6	(GTC)6	(GTC)6	(GTC)6	(GTC)6	(GTC)5	-	Introns/Intergenic	-	-	CATCCTCGTCTTCGTGCAG	GAGGCTTGATGACGCAGAG	208	A	A	A	NA

Polymorphic SSR (PMS) markers	Chromosomes	Start physical positions (bp)	End physical positions (bp)	Nipponbare	Kasalath	Nagina22	IR64	Pokkali	Bala	Tainung67	Azucena	Moroberekan	*Oryza rufipogon*	*Oryza nivara*	MSU gene locus IDs	Structural annotation	Functional annotation	SSR functional effects	Forward primers (5'-3')	Reverse primers (5'-3')	Amplified product size (bp)	Markers exhibiting polymorphism within *indica* rice	Transcrption factor genes-derived markers	Known cloned genes-derived markers	PMS markers corresponding with RM markers
PMS3835	Os_Chr07	7999984	8000001	(GGC)6	(GGC)6	(GGC)6	(GGC)6	(GGC)6	(GGC)6	(GGC)5	(GGC)6	(GGC)6	(GGC)6	(GGC)6	-	Introns/Intergenic	-	-	TACCATGCATTTTTCCCCAT	AGAGAGGAGGACATGGACGA	256	A	A	A	NA
PMS3836	Os_Chr07	8045041	8045058	(CGG)6	(CGG)6	(CGG)6	(CGG)6	(CGG)6	(CGG)6	(CGG)6	(CGG)5	(CGG)6	(CGG)6	(CGG)6	LOC_Os07g14100	CDS	pentatricopeptide, putative, expressed	CODON_DELETION	GTGGTGGTTTGGTGGGTTT	GCAGGGGATAAGCAAAAACA	271	A	A	A	NA
PMS3837	Os_Chr07	8055707	8055724	(ATT)6	(ATT)5	(ATT)6	(ATT)6	(ATT)6	(ATT)5	(ATT)6	(ATT)6	(ATT)6	(ATT)6	(ATT)6	-	Introns/Intergenic	-	-	CAGGTGGATCAACTGAGGCT	ATAAAGGCTCCCACACCACA	223	P	A	A	NA
PMS3838	Os_Chr07	8233222	8233249	(AT)14	(AT)13	(AT)6	(AT)14	(AT)12	(AT)13	(AT)14	(AT)14	(AT)14	(AT)14	(AT)14	-	Introns/Intergenic	-	-	AGTAGTTGTGGAAGGCGTGC	ACGTACGCTTCTTCGCTTGT	266	P	A	A	NA
PMS3839	Os_Chr07	8271431	8271448	(GAG)6	(GAG)6	(GAG)6	(GAG)6	(GAG)6	(GAG)6	(GAG)6	(GAG)6	(GAG)6	(GAG)8	(GAG)6	-	Introns/Intergenic	-	-	GGGCAAAGCTAGCTGAGAGA	TGGAGACGAGAGGAGAGGAA	264	A	A	A	NA
PMS3840	Os_Chr07	8331657	8331671	(CGG)5	(CGG)5	(CGG)5	(CGG)6	(CGG)5	(CGG)6	(CGG)5	(CGG)5	(CGG)5	(CGG)5	(CGG)5	-	Introns/Intergenic	-	-	GAGGTGGGCGAACAGGAC	CGATGTCACAACCAAACCAG	213	P	A	A	NA
PMS3841	Os_Chr07	8349270	8349290	(GCG)7	(GCG)7	(GCG)7	(GCG)7	(GCG)7	(GCG)7	(GCG)7	(GCG)7	(GCG)7	(GCG)5	(GCG)7	LOC_Os07g14630	CDS	glucosyltransferase-11, putative, expressed	CODON_DELETION	GACGGCGAACGGCAGTAT	GTCACCTTCCTCCACACCC	269	A	A	A	NA
PMS3842	Os_Chr07	8374162	8374179	(AT)9	(AT)9	(AT)9	(AT)6	(AT)9	(AT)9	(AT)9	(AT)9	(AT)9	(AT)9	(AT)9	-	Introns/Intergenic	-	-	AAACATGGGTTTCATAGCGG	GGCATCGAGCAGTTAACACA	198	P	A	A	NA
PMS3843	Os_Chr07	8413867	8413896	(AT)15	(AT)14	(AT)15	(AT)15	(AT)15	(AT)15	(AT)15	(AT)15	(AT)15	(AT)9	(AT)15	-	Introns/Intergenic	-	-	TGGAGTAGTCATTTGTCTTCCA	TCAAAATCAATGAAAGTTTTGTGAA	260	P	A	A	NA
PMS3844	Os_Chr07	8457696	8457743	(AT)24	(AT)16	(AT)11	(AT)24	(AT)24	(AT)24	(AT)24	(AT)24	(AT)24	(AT)24	(AT)11	-	Introns/Intergenic	-	-	CCACAATAGCCGGGACTAAA	TCAAGGGGAGGGAGATCAAT	277	P	A	A	NA
PMS3845	Os_Chr07	8464219	8464246	(TATG)7	(TATG)7	(TATG)7	(TATG)7	(TATG)7	(TATG)7	(TATG)7	(TATG)7	(TATG)7	(TATG)5	(TATG)7	-	Introns/Intergenic	-	-	CCTAGGTGATCCTAGTGCAAAGTCC	GGTAACACCAACCAGGAATCAGG	171	A	A	A	RM7531
PMS3846	Os_Chr07	8555457	8555474	(TA)9	(TA)9	(TA)9	(TA)6	(TA)9	(TA)9	(TA)9	(TA)9	(TA)9	(TA)9	(TA)9	-	Introns/Intergenic	-	-	GCCAAGTGGCACCCTATAAA	CGGACGAAGGAAACATCTTG	195	P	A	A	NA
PMS3847	Os_Chr07	8614810	8614823	(TA)7	(TA)7	(TA)7	(TA)6	(TA)7	(TA)7	(TA)7	(TA)7	(TA)7	(TA)7	(TA)7	-	Introns/Intergenic	-	-	ATGGTGCCCCATATGAGTGT	TTGTACTTTTCGGTTTCGGC	212	P	A	A	NA

Polymorphic SSR (PMS) markers	Chromosomes	Start physical positions (bp)	End physical positions (bp)	Nipponbare	Kasalath	Nagina22	IR64	Pokkali	Bala	Tainung67	Azucena	Moroberekan	*Oryza rufipogon*	*Oryza nivara*	MSU gene locus IDs	Structural annotation	Functional annotation	SSR functional effects	Forward primers (5'-3')	Reverse primers (5'-3')	Amplified product size (bp)	Markers exhibiting polymorphism within *indica* rice	Transcrption factor genes-derived markers	Known cloned genes-derived markers	PMS markers corresponding with RM markers
PMS3848	Os_Chr07	8700573	8700620	(AT)24	(AT)15	(AT)24	(AT)24	(AT)24	(AT)24	(AT)24	(AT)24	(AT)6	(AT)24	(AT)24	-	Introns/Intergenic	-	-	AGATCATCTTTAGCCCCGGT	GATCAGAGGAGAAGGGGAGG	253	P	A	A	NA
PMS3849	Os_Chr07	8768169	8768186	(CTG)6	(CTG)6	(CTG)6	(CTG)6	(CTG)6	(CTG)6	(CTG)6	(CTG)6	(CTG)6	(CTG)5	(CTG)5	LOC_Os07g15210	CDS	retrotransposon protein, putative, Ty3-gypsy subclass, expressed	CODON_DELETION	GAGCAACCTTCTCTTGGTCG	GATGCAACAGATGCAGCAAT	224	A	A	A	NA
PMS3850	Os_Chr07	8807500	8807517	(TCC)6	(TCC)6	(TCC)6	(TCC)6	(TCC)6	(TCC)6	(TCC)6	(TCC)6	(TCC)6	(TCC)5	(TCC)6	LOC_Os07g15270	CDS	RNA-binding region RNP-1, putative, expressed	CODON_CHANGE_PLUS_CODON_DELETION	GATGATGCAACCATCACAGC	GAGAGGAGGAATCAACGCAG	140	A	A	A	NA
PMS3851	Os_Chr07	8837872	8837895	(AT)12	(AT)12	(AT)12	(AT)12	(AT)12	(AT)9	(AT)12	(AT)12	(AT)12	(AT)12	(AT)12	-	Introns/Intergenic	-	-	TCATTGCGATGTTTGGATGT	CAATGAACATGCTTGTGCTG	242	P	A	A	NA
PMS3852	Os_Chr07	9014756	9014773	(CGG)6	(CGG)9	(CGG)6	(CGG)6	(CGG)6	(CGG)6	(CGG)6	(CGG)6	(CGG)6	(CGG)6	(CGG)6	LOC_Os07g15550	CDS	retrotransposon protein, putative, unclassified, expressed	CODON_INSERTION	GTCGTCCTCCACCAACATCT	TGGTTGACCCATGTAGCAGA	210	P	A	A	NA
PMS3853	Os_Chr07	9404220	9404237	(TC)9	(TC)9	(TC)9	(TC)8	(TC)8	(TC)9	(TC)9	(TC)9	(TC)9	(TC)9	(TC)8	-	Introns/Intergenic	-	-	ACGACATGGTGATGATAGCG	CGCTGCTCAGTAGTCACTCG	211	P	A	A	NA
PMS3854	Os_Chr07	9454443	9454462	(CT)10	(CT)10	(CT)10	(CT)10	(CT)10	(CT)10	(CT)10	(CT)10	(CT)10	(CT)8	(CT)10	-	Introns/Intergenic	-	-	CTCTTTCTTTCCTCCCTCCC	CGCTGTCGTCGGTAAGCC	127	A	A	A	NA
PMS3855	Os_Chr07	9638521	9638532	(GA)6	(GA)7	(GA)6	(GA)6	(GA)6	(GA)7	(GA)6	(GA)6	(GA)6	(GA)6	(GA)6	-	Introns/Intergenic	-	-	AGGAAGGGGATGACGGGT	TGGAATTTTCAGAGCACCAA	250	P	A	A	NA
PMS3856	Os_Chr07	10241230	10241275	(AT)23	(AT)23	(AT)8	(AT)12	(AT)17	(AT)23	(AT)16	(AT)23	(AT)23	(AT)11	(AT)23	-	Introns/Intergenic	-	-	GCTCCAATTCCTTCTAGGGC	CGTGAATGAGAACAAGCGAA	268	P	A	A	NA
PMS3857	Os_Chr07	10262237	10262258	(GA)11	(GA)11	(GA)11	(GA)11	(GA)11	(GA)11	(GA)11	(GA)11	(GA)11	(GA)10	(GA)11	-	Introns/Intergenic	-	-	CACCCACCCTGGTAAGAACA	CCACTTCACTCGCAGAATCA	229	A	A	A	NA
PMS3858	Os_Chr07	10338983	10339018	(AT)18	(AT)13	(AT)18	(AT)18	(AT)18	(AT)18	(AT)18	(AT)18	(AT)18	(AT)18	(AT)18	-	Introns/Intergenic	-	-	AGGTTACTGACACTGCAGACTTCC	GATGGACGGTACAGATTGATCC	313	P	A	A	RM21370
PMS3859	Os_Chr07	10623201	10623234	(TA)17	(TA)17	(TA)17	(TA)17	(TA)17	(TA)17	(TA)17	(TA)17	(TA)17	(TA)11	(TA)17	-	Introns/Intergenic	-	-	TAGGGCTAGCTCTTCCACACAGG	CGAACGAACAAGGACGAACG	144	A	A	A	RM21379
PMS3860	Os_Chr07	10690429	10690442	(CT)7	(CT)7	(CT)6	(CT)7	(CT)7	(CT)7	(CT)7	(CT)7	(CT)7	(CT)7	(CT)7	-	Introns/Intergenic	-	-	TGATGAAGTGAGCGAACCAG	AGCCCCTCCCCTATGAAAT	122	P	A	A	NA

Polymorphic SSR (PMS) markers	Chromosomes	Start physical positions (bp)	End physical positions (bp)	Nipponbare	Kasalath	Nagina22	IR64	Pokkali	Bala	Tainung67	Azucena	Moroberekan	*Oryza rufipogon*	*Oryza nivara*	MSU gene locus IDs	Structural annotation	Functional annotation	SSR functional effects	Forward primers (5'-3')	Reverse primers (5'-3')	Amplified product size (bp)	Markers exhibiting polymorphism within *indica* rice	Transcrption factor genes-derived markers	Known cloned genes-derived markers	PMS markers corresponding with RM markers
PMS3861	Os_Chr07	10760974	10760993	(TA)10	(TA)10	(TA)6	(TA)8	(TA)8	(TA)10	(TA)10	(TA)10	(TA)10	(TA)10	(TA)10	-	Introns/Intergenic	-	-	TGCTGTGTACAGGTTTGATGACC	GGTGTGTGAGGTGGGTTTGC	137	P	A	A	RM21386
PMS3862	Os_Chr07	10873793	10873816	(TTG)8	(TTG)5	(TTG)8	(TTG)8	(TTG)8	(TTG)8	(TTG)8	(TTG)8	(TTG)8	(TTG)8	(TTG)8	LOC_Os07g18350	CDS	retrotransposon protein, putative, Ty3-gypsy subclass, expressed	CODON_DELETION	TATTTCCCTGGTTCTGCTGG	TTTCCCCGGTTTCTCTTCTT	193	P	A	A	NA
PMS3863	Os_Chr07	10934131	10934146	(AG)8	(AG)6	(AG)8	(AG)8	(AG)8	(AG)8	(AG)8	(AG)8	(AG)8	(AG)8	(AG)8	-	Introns/Intergenic	-	-	CGCAGCCTTGCTCTCTTACT	ACGCTGGCAACTCACTTCTT	196	P	A	A	NA
PMS3864	Os_Chr07	11311560	11311577	(GA)9	(GA)9	(GA)9	(GA)9	(GA)9	(GA)9	(GA)9	(GA)6	(GA)9	(GA)9	(GA)9	-	Introns/Intergenic	-	-	GAGAGACAGTGGAAGGGAAGACG	GTTCCCTCCCTCCTCCTAGTTCG	90	A	A	A	RM3635
PMS3865	Os_Chr07	11622428	11622455	(AT)14	(AT)14	(AT)14	(AT)14	(AT)14	(AT)14	(AT)6	(AT)14	(AT)14	(AT)14	(AT)14	-	Introns/Intergenic	-	-	CTAATGTGAAGCTCAACTCCATCG	GAAGCTGGAAATTCTGAGAGAAGC	361	A	A	A	RM21407
PMS3866	Os_Chr07	11629691	11629710	(GA)10	(GA)10	(GA)10	(GA)10	(GA)10	(GA)9	(GA)10	(GA)10	(GA)10	(GA)10	(GA)10	LOC_Os07g20135	CDS	expressed protein	FRAME_SHIFT	AGCGAGCTCCGCTTCTTT	GGACGATGGTGTTGATGATG	259	P	A	A	NA
PMS3867	Os_Chr07	12002132	12002205	(TA)37	(TA)37	(TA)37	(TA)37	(TA)37	(TA)37	(TA)37	(TA)37	(TA)17	(TA)37	(TA)35	-	Introns/Intergenic	-	-	TATTTCATACCATGGTGCCC	AGTGGTGGGTAGTTTGACCG	254	A	A	A	NA
PMS3868	Os_Chr07	12758297	12758317	(TGG)7	(TGG)7	(TGG)7	(TGG)7	(TGG)7	(TGG)7	(TGG)5	(TGG)7	(TGG)7	(TGG)5	(TGG)7	-	Introns/Intergenic	-	-	TTGCCGTACTTTCTGCACAC	CACGGGCAATATGCAAGTTA	187	A	A	A	NA
PMS3869	Os_Chr07	13025273	13025296	(TTC)8	(TTC)7	(TTC)8	(TTC)8	(TTC)8	(TTC)7	(TTC)8	(TTC)8	(TTC)8	(TTC)8	(TTC)7	LOC_Os07g23100	CDS	expressed protein	CODON_DELETION	GCAACTACAAGCCATAGCTCCTTTCC	TCATCCCATCATGGTCGAGTGC	185	P	A	A	RM8245
PMS3870	Os_Chr07	13033563	13033576	(TC)7	(TC)6	(TC)7	(TC)7	(TC)7	(TC)7	(TC)7	(TC)7	(TC)7	(TC)7	(TC)6	-	Introns/Intergenic	-	-	ATAATGGTGACCCTGCTTGC	TTTTCATTCCCAACTCGTCC	154	P	A	A	NA
PMS3871	Os_Chr07	13067862	13067879	(GGC)6	(GGC)6	(GGC)6	(GGC)6	(GGC)6	(GGC)6	(GGC)5	(GGC)6	(GGC)6	(GGC)6	(GGC)6	LOC_Os07g23169	CDS	methyltransferase, putative, expressed	CODON_DELETION	AGCGGAGATTGAAACCTTGA	AGGAGTACCACTGCCACGAC	203	A	A	A	NA
PMS3872	Os_Chr07	13070093	13070110	(CCA)6	(CCA)6	(CCA)6	(CCA)6	(CCA)6	(CCA)6	(CCA)6	(CCA)5	(CCA)6	(CCA)6	(CCA)6	-	Introns/Intergenic	-	-	AGAAGGACCGCTTGCATAGA	TGGAGAAGAGACGGAGGAGA	151	A	A	A	NA
PMS3873	Os_Chr07	13206706	13206757	(AT)26	(AT)26	(AT)26	(AT)26	(AT)26	(AT)6	(AT)26	(AT)26	(AT)26	(AT)26	(AT)6	-	Introns/Intergenic	-	-	AGCGCACATTTCCTTCAAAT	AAGGGAAGGGACAGGAGAAA	264	P	A	A	NA

Polymorphic SSR (PMS) markers	Chromosomes	Start physical positions (bp)	End physical positions (bp)	Nipponbare	Kasalath	Nagina22	IR64	Pokkali	Bala	Tainung67	Azucena	Moroberekan	*Oryza rufipogon*	*Oryza nivara*	MSU gene locus IDs	Structural annotation	Functional annotation	SSR functional effects	Forward primers (5'-3')	Reverse primers (5'-3')	Amplified product size (bp)	Markers exhibiting polymorphism within *indica* rice	Transcrption factor genes-derived markers	Known cloned genes-derived markers	PMS markers corresponding with RM markers
PMS3874	Os_Chr07	13313875	13313892	(GGC)6	(GGC)5	(GGC)6	(GGC)6	(GGC)6	(GGC)5	(GGC)6	(GGC)6	(GGC)6	(GGC)6	(GGC)6	-	Introns/Intergenic	-	-	ATTACTCTGCGTCGAGGAGC	GTTCAATACCGAGGCCACAT	141	P	A	A	NA
PMS3875	Os_Chr07	13439961	13439988	(GA)14	(GA)14	(GA)14	(GA)14	(GA)14	(GA)14	(GA)14	(GA)14	(GA)14	(GA)14	(GA)11	LOC_Os07g23790	URR	expressed protein	-	CGCAGATCAAGAAGCAGGACACC	CGATCTGGAGTCGCGTCAAACC	110	A	A	A	RM3670
PMS3876	Os_Chr07	13531156	13531169	(TA)7	(TA)7	(TA)6	(TA)7	(TA)7	(TA)7	(TA)6	(TA)7	(TA)7	(TA)7	(TA)7	-	Introns/Intergenic	-	-	TCCAAAGACAGCACATGAGG	TTTATTTGCATGGCCCTGTT	243	P	A	A	NA
PMS3877	Os_Chr07	13644380	13644397	(GGC)6	(GGC)6	(GGC)6	(GGC)6	(GGC)6	(GGC)6	(GGC)6	(GGC)6	(GGC)6	(GGC)6	(GGC)5	LOC_Os07g24060	CDS	retrotransposon protein, putative, unclassified, expressed	CODON_DELETION	CAACTTCTGCGTGTACGAGG	GACGGCACCTTCTTGACGTA	279	A	A	A	NA
PMS3878	Os_Chr07	13655766	13655786	(GCG)7	(GCG)7	(GCG)7	(GCG)7	(GCG)7	(GCG)7	(GCG)7	(GCG)6	(GCG)7	(GCG)7	(GCG)5	LOC_Os07g24090	URR	mTERF domain containing protein, expressed	-	ACCTTGAGGTGGTGGTGGTA	GACGGAAAGTTGAGGGATGA	257	A	A	A	NA
PMS3879	Os_Chr07	13849918	13849952	(GCCTG)7	(GCCTG)7	(GCCTG)7	(GCCTG)7	(GCCTG)7	(GCCTG)7	(GCCTG)7	(GCCTG)7	(GCCTG)7	(GCCTG)7	(GCCTG)5	-	Introns/Intergenic	-	-	AGCCACTTGAAGCATGTGTG	GCAGCAAAACAAAACTGCAA	214	A	A	A	NA
PMS3880	Os_Chr07	13857235	13857248	(TG)7	(TG)7	(TG)7	(TG)7	(TG)7	(TG)7	(TG)7	(TG)6	(TG)7	(TG)7	(TG)7	-	Introns/Intergenic	-	-	AAGGCACGTCTCTTGCTTGT	TCCGTCTCTCCCCTATCTCA	234	A	A	A	NA
PMS3881	Os_Chr07	13888017	13888034	(GAT)6	(GAT)5	(GAT)5	(GAT)6	(GAT)5	(GAT)5	(GAT)6	(GAT)6	(GAT)6	(GAT)6	(GAT)6	-	Introns/Intergenic	-	-	GGTTTGCAGAAGCCAAGAAG	TCAGCCTTGGTGATGTCAAA	194	P	A	A	NA
PMS3882	Os_Chr07	13889789	13889802	(CT)7	(CT)7	(CT)7	(CT)7	(CT)7	(CT)7	(CT)7	(CT)6	(CT)7	(CT)7	(CT)7	-	Introns/Intergenic	-	-	AGCAAACCGACATCTCCAAC	GTGAGGGAGATCGACAGTGG	244	A	A	A	NA
PMS3883	Os_Chr07	14145143	14145170	(TC)14	(TC)14	(TC)14	(TC)14	(TC)14	(TC)14	(TC)14	(TC)13	(TC)14	(TC)14	(TC)14	-	Introns/Intergenic	-	-	CACGATGAACATCGCGTTAC	GGTGGGAGGGAGGGTATAGA	256	A	A	A	NA
PMS3884	Os_Chr07	14356232	14356252	(CAA)7	(CAA)5	(CAA)7	(CAA)5	(CAA)5	(CAA)5	(CAA)7	(CAA)7	(CAA)5	(CAA)7	(CAA)7	-	Introns/Intergenic	-	-	ATGGTGAATACTCGCAGCACTGG	GCAAGCGTGGGATTCTCTCC	74	P	A	A	RM21460
PMS3885	Os_Chr07	14452141	14452167	(TGT)9	(TGT)9	(TGT)9	(TGT)9	(TGT)7	(TGT)9	(TGT)9	(TGT)9	(TGT)9	(TGT)9	(TGT)9	LOC_Os07g25300	CDS	retrotransposon, putative, centromere-specific	CODON_DELETION	CTGCATCAACATCCAACACC	TTAGCAACCCCTCTCTTCCA	224	P	A	A	NA
PMS3886	Os_Chr07	14552681	14552695	(GGC)5	(GGC)5	(GGC)5	(GGC)5	(GGC)5	(GGC)5	(GGC)5	(GGC)5	(GGC)5	(GGC)6	(GGC)5	-	Introns/Intergenic	-	-	GGGGTGGAATGGTAATGTTG	CTCCCATGGACCACCTACTC	233	A	A	A	NA

Polymorphic SSR (PMS) markers	Chromosomes	Start physical positions (bp)	End physical positions (bp)	Nipponbare	Kasalath	Nagina22	IR64	Pokkali	Bala	Tainung67	Azucena	Moroberekan	*Oryza rufipogon*	*Oryza nivara*	MSU gene locus IDs	Structural annotation	Functional annotation	SSR functional effects	Forward primers (5'-3')	Reverse primers (5'-3')	Amplified product size (bp)	Markers exhibiting polymorphism within *indica* rice	Transcrption factor genes-derived markers	Known cloned genes-derived markers	PMS markers corresponding with RM markers
PMS3887	Os_Chr07	14606482	14606502	(CCG)7	(CCG)7	(CCG)7	(CCG)7	(CCG)7	(CCG)7	(CCG)7	(CCG)7	(CCG)7	(CCG)5	(CCG)7	LOC_Os07g25500	CDS	pentatricopeptide, putative, expressed	CODON_CHANGE_PLUS_CODON_DELETION	CGTTCTTCAACTCCCTCCTCTCC	CTTCAGAGCGAAGCAATGAAGC	500	A	A	A	RM21469
PMS3888	Os_Chr07	14668282	14668296	(GGC)5	(GGC)6	(GGC)5	(GGC)5	(GGC)5	(GGC)5	(GGC)5	(GGC)5	(GGC)5	(GGC)5	(GGC)5	-	Introns/Intergenic	-	-	CTCGCGAATTCCTTTTCTTG	AGAGTTGCAAATGCTTGCCT	238	P	A	A	NA
PMS3889	Os_Chr07	14723239	14723268	(TCC)10	(TCC)6	(TCC)10	(TCC)10	(TCC)10	(TCC)6	(TCC)10	(TCC)10	(TCC)10	(TCC)10	(TCC)10	LOC_Os07g25680	URR	protein kinase domain containing protein, expressed	-	ACGATCTCCATCTCCACCAG	GTGGAGGTCAGAGGCAAGAG	123	P	A	A	NA
PMS3890	Os_Chr07	14796335	14796368	(AT)17	(AT)17	(AT)6	(AT)17	(AT)10	(AT)6	(AT)17	(AT)17	(AT)17	(AT)17	(AT)17	-	Introns/Intergenic	-	-	CTTGCCCATTTGGTTATGCT	ATTTGATACCATGGTGCCCG	149	P	A	A	NA
PMS3891	Os_Chr07	14796765	14796820	(AT)28	(AT)28	(AT)14	(AT)28	(AT)28	(AT)28	(AT)28	(AT)19	(AT)28	(AT)9	(AT)17	-	Introns/Intergenic	-	-	TCAATTTCAACAATGGTGCC	TAGATAGCCAGACATCCGGG	155	P	A	A	NA
PMS3892	Os_Chr07	14912170	14912203	(AT)17	(AT)17	(AT)17	(AT)17	(AT)17	(AT)17	(AT)17	(AT)12	(AT)17	(AT)17	(AT)17	-	Introns/Intergenic	-	-	AAAAGTAACTTAGAACAAAACGAAGG	TCCGTTGTAGAGATAAAAACATGG	249	A	A	A	NA
PMS3893	Os_Chr07	15144198	15144211	(CT)7	(CT)7	(CT)7	(CT)7	(CT)7	(CT)6	(CT)7	(CT)7	(CT)7	(CT)7	(CT)7	-	Introns/Intergenic	-	-	CACACCGTCCTCTTTCGTTT	GGAGATGGAATCACCTTGGA	104	P	A	A	NA
PMS3894	Os_Chr07	15339295	15339312	(CGG)6	(CGG)5	(CGG)6	(CGG)6	(CGG)6	(CGG)6	(CGG)6	(CGG)5	(CGG)6	(CGG)6	(CGG)6	-	Introns/Intergenic	-	-	GAATTGAATCCAAAGCCTCG	GGATATCATCGACCCCTCAG	219	P	A	A	NA
PMS3895	Os_Chr07	15448158	15448175	(AGC)6	(AGC)5	(AGC)6	(AGC)6	(AGC)6	(AGC)6	(AGC)6	(AGC)6	(AGC)6	(AGC)6	(AGC)6	-	Introns/Intergenic	-	-	ACATGGTTGCAGTCTTGCAG	CGCCACATCATGTTAGATGC	212	P	A	A	NA
PMS3896	Os_Chr07	15645000	15645023	(TA)12	(TA)9	(TA)12	(TA)12	(TA)12	(TA)12	(TA)12	(TA)12	(TA)12	(TA)12	(TA)12	-	Introns/Intergenic	-	-	AATGCCAGAAATCCTCCGAGTAGTGG	GTTCGAGTATTGCGGCCAGACG	188	P	A	A	RM21511
PMS3897	Os_Chr07	15692632	15692657	(AT)13	(AT)13	(AT)13	(AT)13	(AT)13	(AT)13	(AT)13	(AT)6	(AT)6	(AT)12	(AT)13	-	Introns/Intergenic	-	-	CCTGTTGTAGTGGGCACAGTTGG	GATGCTTCAGGCAACAGCAAGG	320	A	A	A	RM21513
PMS3898	Os_Chr07	15860953	15860966	(GA)7	(GA)7	(GA)7	(GA)7	(GA)6	(GA)7	(GA)7	(GA)7	(GA)7	(GA)7	(GA)7	-	Introns/Intergenic	-	-	GTGATTTATAGGGCACGGGA	TCGGTTGTCCCTAACCTCC	280	P	A	A	NA
PMS3899	Os_Chr07	15911661	15911687	(TCT)9	(TCT)10	(TCT)10	(TCT)9	(TCT)9	(TCT)10	(TCT)9	(TCT)9	(TCT)9	(TCT)9	(TCT)9	-	Introns/Intergenic	-	-	GCACACACAGGAGGATGCTA	TAATGTGCACGAATCTCGGA	270	P	A	A	NA

Polymorphic SSR (PMS) markers	Chromosomes	Start physical positions (bp)	End physical positions (bp)	Nipponbare	Kasalath	Nagina22	IR64	Pokkali	Bala	Tainung67	Azucena	Moroberekan	*Oryza rufipogon*	*Oryza nivara*	MSU gene locus IDs	Structural annotation	Functional annotation	SSR functional effects	Forward primers (5'-3')	Reverse primers (5'-3')	Amplified product size (bp)	Markers exhibiting polymorphism within *indica* rice	Transcrption factor genes-derived markers	Known cloned genes-derived markers	PMS markers corresponding with RM markers
PMS3900	Os_Chr07	15953109	15953128	(TAGA)5	(TAGA)7	(TAGA)7	(TAGA)7	(TAGA)7	(TAGA)7	(TAGA)5	(TAGA)5	(TAGA)5	(TAGA)5	(TAGA)7	-	Introns/Intergenic	-	-	GAATGACGGAGACAGAGGGA	TTTTGTCAATGTCCCTAGGTTG	189	A	A	A	NA
PMS3901	Os_Chr07	15963307	15963328	(AT)11	(AT)11	(AT)11	(AT)8	(AT)11	(AT)11	(AT)11	(AT)11	(AT)11	(AT)11	(AT)11	-	Introns/Intergenic	-	-	CCACCATGTCAAACATGCCAAGC	ATGTTCGGTCCCATGCTGTACCC	375	P	A	A	RM21520
PMS3902	Os_Chr07	15981913	15981924	(GA)6	(GA)7	(GA)6	(GA)6	(GA)6	(GA)7	(GA)6	(GA)6	(GA)6	(GA)7	(GA)7	-	Introns/Intergenic	-	-	TGCTAATTTCATATTTGTTGGGA	GGGAGCTTCTGACAACTGGA	176	P	A	A	NA
PMS3903	Os_Chr07	16022455	16022475	(GGT)7	(GGT)5	(GGT)7	(GGT)7	(GGT)7	(GGT)7	(GGT)7	(GGT)7	(GGT)7	(GGT)7	(GGT)5	LOC_Os07g27480	CDS	ELMO/CED-12 family protein, putative, expressed	CODON_DELETION	TGGGATTCGATTACCTCCTG	TCTCCTCCCTAAAACCCCAC	258	P	A	A	NA
PMS3904	Os_Chr07	16022634	16022655	(AG)11	(AG)11	(AG)11	(AG)11	(AG)11	(AG)11	(AG)11	(AG)11	(AG)11	(AG)11	(AG)7	-	Introns/Intergenic	-	-	GTGGGGTTTTAGGGAGGAGA	GCAACTGAGCAGCTAGCCAT	196	A	A	A	NA
PMS3905	Os_Chr07	16332731	16332748	(AT)9	(AT)10	(AT)9	(AT)9	(AT)9	(AT)10	(AT)9	(AT)9	(AT)9	(AT)9	(AT)9	-	Introns/Intergenic	-	-	GGAGCTCAAAATGGCAAACT	AAACATACATCCCTCGGACG	195	P	A	A	NA
PMS3906	Os_Chr07	16394367	16394380	(CG)7	(CG)7	(CG)7	(CG)7	(CG)7	(CG)7	(CG)7	(CG)6	(CG)7	(CG)7	(CG)7	-	Introns/Intergenic	-	-	CCCTGTTGACCAGTTGAGGT	GGTTAGTGGCTGCAAAAGGA	133	A	A	A	NA
PMS3907	Os_Chr07	16438264	16438281	(TC)9	(TC)8	(TC)9	(TC)9	(TC)8	(TC)9	(TC)9	(TC)9	(TC)9	(TC)9	(TC)7	-	Introns/Intergenic	-	-	CGAAAGCCTTCCTGTTCAAG	ATTTAAAGGGGGAGGGTGTG	160	P	A	A	NA
PMS3908	Os_Chr07	16602403	16602427	(TCTCT)5	(TCTCT)6	(TCTCT)5	(TCTCT)5	(TCTCT)5	(TCTCT)5	(TCTCT)5	(TCTCT)5	(TCTCT)5	(TCTCT)5	(TCTCT)5	-	Introns/Intergenic	-	-	ACCCACCCGTCACTTCTCT	AATTTCTGGGCGTGACAGAC	199	P	A	A	NA
PMS3909	Os_Chr07	16632065	16632084	(TA)10	(TA)10	(TA)10	(TA)9	(TA)10	(TA)10	(TA)10	(TA)10	(TA)10	(TA)10	(TA)10	-	Introns/Intergenic	-	-	TCGTCAAATTTGTGTGGGAA	AGCCGTTTGATTTGATTTGG	140	P	A	A	NA
PMS3910	Os_Chr07	16632550	16632567	(ATT)6	(ATT)6	(ATT)6	(ATT)6	(ATT)6	(ATT)6	(ATT)6	(ATT)6	(ATT)6	(ATT)8	(ATT)6	-	Introns/Intergenic	-	-	TTGTGTTAGGTAGTATCCCTCCG	CGCATCAAAACATACATTCCC	238	A	A	A	NA
PMS3911	Os_Chr07	16844000	16844013	(AT)7	(AT)7	(AT)7	(AT)7	(AT)7	(AT)6	(AT)7	(AT)7	(AT)7	(AT)7	(AT)7	-	Introns/Intergenic	-	-	CCGTAATATTGAAACTCTGCT	ATACGATTGTTAGACTTAGCAT	287	P	A	A	NA
PMS3912	Os_Chr07	16921796	16921813	(TA)9	(TA)9	(TA)9	(TA)9	(TA)9	(TA)9	(TA)9	(TA)9	(TA)9	(TA)6	(TA)9	-	Introns/Intergenic	-	-	TGTTCATTTTTGGAAACGCA	CATTACAACGGGTGAAGGCT	201	A	A	A	NA

Polymorphic SSR (PMS) markers	Chromosomes	Start physical positions (bp)	End physical positions (bp)	Nipponbare	Kasalath	Nagina22	IR64	Pokkali	Bala	Tainung67	Azucena	Moroberekan	*Oryza rufipogon*	*Oryza nivara*	MSU gene locus IDs	Structural annotation	Functional annotation	SSR functional effects	Forward primers (5'-3')	Reverse primers (5'-3')	Amplified product size (bp)	Markers exhibiting polymorphism within *indica* rice	Transcrption factor genes-derived markers	Known cloned genes-derived markers	PMS markers corresponding with RM markers
PMS3913	Os_Chr07	16929270	16929281	(CT)6	(CT)6	(CT)6	(CT)6	(CT)6	(CT)6	(CT)6	(CT)6	(CT)6	(CT)6	(CT)7	-	Introns/Intergenic	-	-	AAAAATGGGTGGAGGTAGCA	TACAATTTGTTGCGCCTGAG	249	A	A	A	NA
PMS3914	Os_Chr07	16932314	16932331	(TCC)6	(TCC)5	(TCC)6	(TCC)6	(TCC)6	(TCC)6	(TCC)6	(TCC)6	(TCC)6	(TCC)6	(TCC)6	LOC_Os07g28880	CDS	expressed protein	CODON_DELETION	CCTTCTCGATCCTCCTCCTT	GGTGGTGGTGGTGTTGGT	270	P	A	A	NA
PMS3915	Os_Chr07	16933036	16933059	(AG)12	(AG)12	(AG)11	(AG)12	(AG)12	(AG)12	(AG)12	(AG)12	(AG)12	(AG)12	(AG)12	-	Introns/Intergenic	-	-	GTATAGCCAACCAAGCAAGATAGC	GATGCCTAGACACACATGTAAGC	155	P	A	A	RM1135
PMS3916	Os_Chr07	16933927	16933944	(CGG)6	(CGG)6	(CGG)6	(CGG)6	(CGG)6	(CGG)6	(CGG)6	(CGG)6	(CGG)6	(CGG)5	(CGG)6	LOC_Os07g28890	CDS	ethylene-responsive protein related, putative, expressed	CODON_CHANGE_PLUS_CODON_DELETION	GAGCTGCTCAACTCGAGGAC	GAGCTCAGGTGCTTCTCCAG	164	A	A	A	NA
PMS3917	Os_Chr07	17114392	17114405	(TA)7	(TA)9	(TA)6	(TA)7	(TA)7	(TA)7	(TA)7	(TA)7	(TA)7	(TA)7	(TA)7	-	Introns/Intergenic	-	-	GGGTATGCATGAACTCACCA	CAGGCTCTCAAGCTCCTCC	234	P	A	A	NA
PMS3918	Os_Chr07	17190544	17190576	(TCT)11	(TCT)9	(TCT)11	(TCT)11	(TCT)11	(TCT)9	(TCT)11	(TCT)11	(TCT)11	(TCT)9	(TCT)11	-	Introns/Intergenic	-	-	CATTGCTTAGGGCCATTTGT	AGTTGGAGCTTGGAAGCAAA	130	P	A	A	NA
PMS3919	Os_Chr07	17269080	17269097	(GGC)6	(GGC)6	(GGC)6	(GGC)6	(GGC)6	(GGC)6	(GGC)5	(GGC)6	(GGC)6	(GGC)6	(GGC)6	LOC_Os07g29410	CDS	CDSP32	CODON_DELETION	AGACGGCGAGGTTCTTGAG	GTAGATGCGGCTGCTGTTC	239	A	A	P	NA
PMS3920	Os_Chr07	17489423	17489434	(TC)6	(TC)10	(TC)6	(TC)9	(TC)6	(TC)9	(TC)6	(TC)6	(TC)9	(TC)6	(TC)6	-	Introns/Intergenic	-	-	GATCACAAGGCTCACAAGCA	CATGCATGCACTATCGCTCT	255	P	A	A	NA
PMS3921	Os_Chr07	17754977	17754994	(CCT)6	(CCT)6	(CCT)6	(CCT)5	(CCT)6	(CCT)6	(CCT)6	(CCT)6	(CCT)6	(CCT)6	(CCT)6	LOC_Os07g30100	CDS	RIN3, putative, expressed	CODON_DELETION	GAGGATGAGGACGAGGAACA	CGGTGAACAGCCTCTACGTC	215	P	A	A	NA
PMS3922	Os_Chr07	17796772	17796833	(TA)31	(TA)31	(TA)31	(TA)31	(TA)31	(TA)31	(TA)31	(TA)31	(TA)13	(TA)31	(TA)6	-	Introns/Intergenic	-	-	TGCCACCTCAGTTTCTCAAA	TTGCATTGGGTGAACATCAT	206	A	A	A	NA
PMS3923	Os_Chr07	17799072	17799089	(CT)9	(CT)9	(CT)9	(CT)9	(CT)9	(CT)8	(CT)9	(CT)9	(CT)9	(CT)9	(CT)9	-	Introns/Intergenic	-	-	AGAGGCCAAACCTCCTGTTT	TGATTTTCATTTGTCGATTCCA	226	P	A	A	NA
PMS3924	Os_Chr07	17800227	17800260	(AT)17	(AT)17	(AT)17	(AT)17	(AT)17	(AT)7	(AT)17	(AT)11	(AT)8	(AT)17	(AT)17	-	Introns/Intergenic	-	-	CACGCGTGCTCAAATTAAAC	CATCGTGATGATGAAGGTGG	150	P	A	A	NA
PMS3925	Os_Chr07	17816175	17816186	(TC)6	(TC)6	(TC)6	(TC)6	(TC)6	(TC)6	(TC)6	(TC)6	(TC)6	(TC)7	(TC)6	-	Introns/Intergenic	-	-	CCATTCCGACGTCTCATTCT	ACTGGCTGAGCTTTCGTGTT	257	A	A	A	NA

Polymorphic SSR (PMS) markers	Chromosomes	Start physical positions (bp)	End physical positions (bp)	Nipponbare	Kasalath	Nagina22	IR64	Pokkali	Bala	Tainung67	Azucena	Moroberekan	*Oryza rufipogon*	*Oryza nivara*	MSU gene locus IDs	Structural annotation	Functional annotation	SSR functional effects	Forward primers (5'-3')	Reverse primers (5'-3')	Amplified product size (bp)	Markers exhibiting polymorphism within *indica* rice	Transcrption factor genes-derived markers	Known cloned genes-derived markers	PMS markers corresponding with RM markers
PMS3926	Os_Chr07	17825403	17825422	(AG)10	(AG)10	(AG)10	(AG)10	(AG)10	(AG)11	(AG)10	(AG)10	(AG)11	(AG)10	(AG)10	-	Introns/Intergenic	-	-	GACGCCTCCTTGAGTATTTCACC	CTCCGTGCTCATTGTGAGAAACC	166	P	A	A	RM21605
PMS3927	Os_Chr07	17870395	17870412	(AG)9	(AG)9	(AG)9	(AG)9	(AG)9	(AG)9	(AG)8	(AG)9	(AG)9	(AG)9	(AG)9	-	Introns/Intergenic	-	-	GAGAGAGGAGGGTTTCCTCG	GGAGTGGGCTGTGAGACTGT	189	A	A	A	NA
PMS3928	Os_Chr07	17898663	17898698	(TA)18	(TA)18	(TA)8	(TA)18	(TA)18	(TA)12	(TA)18	(TA)9	(TA)18	(TA)18	(TA)18	-	Introns/Intergenic	-	-	AGGCAGATAAGTGAGGCATACGG	CCCTCTTTGTCTATCCTGGATTCG	265	P	A	A	RM20908
PMS3929	Os_Chr07	17944632	17944669	(GA)19	(GA)19	(GA)19	(GA)19	(GA)19	(GA)19	(GA)19	(GA)19	(GA)19	(GA)19	(GA)18	-	Introns/Intergenic	-	-	GCGTGCGCGGGAAGTAAC	CTCTCTCTCCCGGGCGTCT	294	A	A	A	NA
PMS3930	Os_Chr07	18248069	18248084	(AT)8	(AT)10	(AT)8	(AT)8	(AT)8	(AT)8	(AT)8	(AT)8	(AT)8	(AT)8	(AT)8	-	Introns/Intergenic	-	-	TTGATCATCGAAGAAGATGATTG	AGGGAAAGCTATCAAAACACTG	270	P	A	A	NA
PMS3931	Os_Chr07	18250965	18250986	(TC)11	(TC)11	(TC)11	(TC)11	(TC)11	(TC)11	(TC)11	(TC)11	(TC)11	(TC)11	(TC)6	LOC_Os07g30830	URR	SNF7 family protein, putative, expressed	-	CCATCCCCATATGCTTTTTC	GGAGAGAAGATTCTTGGGGG	271	A	A	A	NA
PMS3932	Os_Chr07	18280626	18280646	(TGC)7	(TGC)7	(TGC)7	(TGC)7	(TGC)7	(TGC)7	(TGC)7	(TGC)7	(TGC)7	(TGC)7	(TGC)5	LOC_Os07g30880	CDS	retrotransposon protein, putative, Ty3-gypsy subclass, expressed	CODON_DELETION	TTGCTGATTCTGCTGCTGTT	TCCGCTACACCAATCCCTAC	131	A	A	A	NA
PMS3933	Os_Chr07	18310119	18310136	(CTC)6	(CTC)6	(CTC)6	(CTC)6	(CTC)6	(CTC)6	(CTC)6	(CTC)6	(CTC)6	(CTC)6	(CTC)5	LOC_Os07g30930	CDS	pentatricopeptide repeat domain containing protein, putative, expressed	CODON_DELETION	GTGAGCTGCTACGTCTCGC	GAGGCTGCGGATCAGAGA	189	A	A	A	NA
PMS3934	Os_Chr07	18477143	18477158	(AT)8	(AT)8	(AT)8	(AT)8	(AT)8	(AT)8	(AT)8	(AT)8	(AT)8	(AT)8	(AT)6	-	Introns/Intergenic	-	-	GGTCCCTGAGTTCTTCCACA	GGCCAGTCAACTCAAAGCAT	140	A	A	A	NA
PMS3935	Os_Chr07	18621985	18621999	(CGG)5	(CGG)8	(CGG)5	(CGG)5	(CGG)5	(CGG)8	(CGG)5	(CGG)5	(CGG)5	(CGG)5	(CGG)5	-	Introns/Intergenic	-	-	GTCCGACGGCTACAAGATGT	CGGGCCTGAAATCATACATT	272	P	A	A	NA
PMS3936	Os_Chr07	18639613	18639630	(GTG)6	(GTG)5	(GTG)6	(GTG)6	(GTG)6	(GTG)6	(GTG)5	(GTG)6	(GTG)6	(GTG)6	(GTG)5	-	Introns/Intergenic	-	-	CTCTGGCTGGCCTCCTCT	CTCACTCAAACCTTCCCACC	171	P	A	A	NA
PMS3937	Os_Chr07	18694458	18694493	(TA)18	(TA)18	(TA)7	(TA)18	(TA)18	(TA)7	(TA)18	(TA)18	(TA)18	(TA)6	(TA)12	-	Introns/Intergenic	-	-	TACAGCAGGGTGGAATATCGACAAGC	TCGTTGTAACGCGTGGATATTTGG	496	P	A	A	RM320
PMS3938	Os_Chr07	18698614	18698627	(GA)7	(GA)7	(GA)7	(GA)7	(GA)7	(GA)7	(GA)7	(GA)7	(GA)8	(GA)7	(GA)7	-	Introns/Intergenic	-	-	ACCTACGCGACAACCTTCAC	AGCTCCCTCCGCTCTCTC	192	A	A	A	NA

Polymorphic SSR (PMS) markers	Chromosomes	Start physical positions (bp)	End physical positions (bp)	Nipponbare	Kasalath	Nagina22	IR64	Pokkali	Bala	Tainung67	Azucena	Moroberekan	*Oryza rufipogon*	*Oryza nivara*	MSU gene locus IDs	Structural annotation	Functional annotation	SSR functional effects	Forward primers (5'-3')	Reverse primers (5'-3')	Amplified product size (bp)	Markers exhibiting polymorphism within *indica* rice	Transcrption factor genes-derived markers	Known cloned genes-derived markers	PMS markers corresponding with RM markers
PMS3939	Os_Chr07	18767863	18767886	(AG)12	(AG)12	(AG)12	(AG)12	(AG)12	(AG)12	(AG)12	(AG)6	(AG)12	(AG)12	(AG)12	-	Introns/Intergenic	-	-	CTTCTCTTGCCCTTGTCTCTCTCC	CTAGGTGCCGTTGTTGGTCTAACG	70	A	A	A	RM5322
PMS3940	Os_Chr07	18811128	18811148	(CCG)7	(CCG)7	(CCG)7	(CCG)7	(CCG)7	(CCG)7	(CCG)7	(CCG)7	(CCG)6	(CCG)7	(CCG)7	-	Introns/Intergenic	-	-	TCTTGAGCACGCTCTTTCTTATGC	CGTTCACGTGCTGCCTCTACC	387	A	A	A	RM21644
PMS3941	Os_Chr07	18855158	18855169	(TC)6	(TC)6	(TC)6	(TC)6	(TC)6	(TC)8	(TC)6	(TC)6	(TC)6	(TC)6	(TC)6	-	Introns/Intergenic	-	-	TTAATATGGCAAGGCCCATC	CAAGCCCCATCAAGGTAAAA	219	P	A	A	NA
PMS3942	Os_Chr07	18876301	18876324	(AG)12	(AG)12	(AG)12	(AG)12	(AG)12	(AG)10	(AG)12	(AG)12	(AG)12	(AG)12	(AG)12	-	Introns/Intergenic	-	-	GGAAGGACTGTTGACAAGGATTAACG	CAGGAAATTACGAGCTGAGGAACG	88	P	A	A	RM3211
PMS3943	Os_Chr07	18965859	18965876	(TA)9	(TA)9	(TA)9	(TA)9	(TA)9	(TA)7	(TA)9	(TA)9	(TA)9	(TA)9	(TA)9	-	Introns/Intergenic	-	-	ATGCAAATGCATATGTGTG	GCGACTACTACTACGTCGAAA	164	P	A	A	NA
PMS3944	Os_Chr07	18971556	18971569	(GA)7	(GA)7	(GA)7	(GA)7	(GA)7	(GA)7	(GA)7	(GA)7	(GA)7	(GA)6	(GA)7	-	Introns/Intergenic	-	-	GTCGTGTCGTGACGAGGTC	GCCTCCCTCTCGATCTCTCT	108	A	A	A	NA
PMS3945	Os_Chr07	19100100	19100115	(AG)8	(AG)8	(AG)8	(AG)8	(AG)8	(AG)9	(AG)8	(AG)8	(AG)8	(AG)9	(AG)7	LOC_Os07g32170	DRR	OsSPL13 - SBP-box gene family member, expressed	-	TACATAGGCACACTGCAGCC	TGCTGCTGCTGATTAACTGG	253	P	P	A	NA
PMS3946	Os_Chr07	19103259	19103279	(GGA)7	(GGA)6	(GGA)7	(GGA)7	(GGA)7	(GGA)6	(GGA)7	(GGA)7	(GGA)6	(GGA)7	(GGA)7	LOC_Os07g32170	URR	OsSPL13 - SBP-box gene family member, expressed	-	CTCGAGCTCATGAGTGGAAGTGG	CCGCCTTTATATCCCTTTCAACC	111	P	P	A	RM21662
PMS3947	Os_Chr07	19236460	19236474	(GGC)5	(GGC)6	(GGC)5	(GGC)5	(GGC)5	(GGC)5	(GGC)5	(GGC)5	(GGC)5	(GGC)5	(GGC)6	-	Introns/Intergenic	-	-	TAACGAGGGAAATGTTTGGC	GTGCTCCATGAGATCCACCT	273	P	A	A	NA
PMS3948	Os_Chr07	19358073	19358100	(TC)14	(TC)13	(TC)14	(TC)14	(TC)14	(TC)13	(TC)14	(TC)13	(TC)13	(TC)13	(TC)13	-	Introns/Intergenic	-	-	TCCGTCCTGATCTCAACACA	GGAAGTCTTGTTGCGCGTAT	129	P	A	A	NA
PMS3949	Os_Chr07	19376489	19376504	(CT)8	(CT)8	(CT)8	(CT)7	(CT)8	(CT)7	(CT)8	(CT)8	(CT)8	(CT)8	(CT)8	-	Introns/Intergenic	-	-	ATTTGCTGGGATGGAACAAG	GTGCATCATATCATGGCGTC	248	P	A	A	NA
PMS3950	Os_Chr07	19386085	19386102	(GCC)6	(GCC)6	(GCC)6	(GCC)6	(GCC)6	(GCC)6	(GCC)6	(GCC)5	(GCC)6	(GCC)6	(GCC)6	LOC_Os07g32530	CDS	potassium transporter, putative, expressed	CODON_DELETION	CGATCTCGTCCTCCTCGTAG	CTGCAGTTTCCGTTTCATCA	272	A	A	A	NA
PMS3951	Os_Chr07	19444102	19444131	(TA)15	(TA)15	(TA)15	(TA)9	(TA)15	(TA)15	(TA)15	(TA)13	(TA)15	(TA)14	(TA)14	-	Introns/Intergenic	-	-	GAGCCCATATTAGAGGATCTCAAACG	TGACTGCACGATTTGGTGTGC	420	P	A	A	RM21686

Polymorphic SSR (PMS) markers	Chromosomes	Start physical positions (bp)	End physical positions (bp)	Nipponbare	Kasalath	Nagina22	IR64	Pokkali	Bala	Tainung67	Azucena	Moroberekan	*Oryza rufipogon*	*Oryza nivara*	MSU gene locus IDs	Structural annotation	Functional annotation	SSR functional effects	Forward primers (5'-3')	Reverse primers (5'-3')	Amplified product size (bp)	Markers exhibiting polymorphism within *indica* rice	Transcrption factor genes-derived markers	Known cloned genes-derived markers	PMS markers corresponding with RM markers
PMS3952	Os_Chr07	19502221	19502247	(TTA)9	(TTA)9	(TTA)10	(TTA)10	(TTA)9	(TTA)9	(TTA)9	(TTA)9	(TTA)9	(TTA)9	(TTA)9	-	Introns/Intergenic	-	-	GGCCTTTCCTTCAGTTTCAGTGG	CCCTTCTCTTTCAGTAATTGGTCTCC	361	P	A	A	RM21691
PMS3953	Os_Chr07	19511705	19511792	(AT)44	(AT)44	(AT)6	(AT)12	(AT)8	(AT)44	(AT)44	(AT)44	(AT)7	(AT)11	(AT)6	-	Introns/Intergenic	-	-	GCGTGGGCATGCTATTTATT	TCGTGATTGGCGAGTGTAGA	257	P	A	A	NA
PMS3954	Os_Chr07	19588962	19588979	(AGG)6	(AGG)8	(AGG)6	(AGG)6	(AGG)6	(AGG)8	(AGG)6	(AGG)6	(AGG)8	(AGG)6	(AGG)6	-	Introns/Intergenic	-	-	CAAGCTCGTCGTGAAGCTCT	CTTGACAGCGAAGCTAAGGG	278	P	A	A	NA
PMS3955	Os_Chr07	19604696	19604765	(AT)35	(AT)8	(AT)7	(AT)8	(AT)35	(AT)11	(AT)8	(AT)35	(AT)35	(AT)35	(AT)6	-	Introns/Intergenic	-	-	TAGCTCCAATTATCAGGGGC	TTTGAAAAATTTGAGTGATTTTGTG	270	P	A	A	NA
PMS3956	Os_Chr07	19605118	19605147	(TA)15	(TA)15	(TA)6	(TA)15	(TA)7	(TA)15	(TA)15	(TA)15	(TA)15	(TA)10	(TA)15	-	Introns/Intergenic	-	-	TCAATTTCAACAATGGTGCC	ATGTGTCCTCCCCCTTCTTT	147	P	A	A	NA
PMS3957	Os_Chr07	19605297	19605318	(TA)11	(TA)8	(TA)11	(TA)11	(TA)11	(TA)11	(TA)11	(TA)11	(TA)11	(TA)11	(TA)11	-	Introns/Intergenic	-	-	CTTTGCTTGATGGGCTGTTT	TATCTTCCGAGCTTGAAGGG	244	P	A	A	NA
PMS3958	Os_Chr07	19631178	19631195	(TCG)6	(TCG)6	(TCG)6	(TCG)6	(TCG)6	(TCG)6	(TCG)6	(TCG)6	(TCG)6	(TCG)5	(TCG)6	-	Introns/Intergenic	-	-	TGCATGCATTTCCAAAAAGA	GGAGGGAGATGAGGAAGGTC	198	A	A	A	NA
PMS3959	Os_Chr07	19637446	19637459	(GA)7	(GA)7	(GA)7	(GA)7	(GA)7	(GA)7	(GA)7	(GA)7	(GA)7	(GA)6	(GA)6	-	Introns/Intergenic	-	-	GCCGATGTAGTCGTGAGAGA	TAATCGACGGTCACGATTGA	241	A	A	A	NA
PMS3960	Os_Chr07	19668413	19668430	(CCG)6	(CCG)6	(CCG)6	(CCG)6	(CCG)6	(CCG)6	(CCG)5	(CCG)6	(CCG)6	(CCG)5	(CCG)6	LOC_Os07g32900	URR	PPR repeat containing protein, expressed	-	AATTTACGTGCACCTTTGGG	GTGAGGCTAGAGCAGGCAGA	278	A	A	A	NA
PMS3961	Os_Chr07	19806043	19806056	(CT)7	(CT)7	(CT)7	(CT)6	(CT)7	(CT)7	(CT)7	(CT)7	(CT)7	(CT)7	(CT)7	-	Introns/Intergenic	-	-	GGGAATTATTTGAGCAAGGGA	GGTTGAAGAAACCTCCGACA	225	P	A	A	NA
PMS3962	Os_Chr07	19809196	19809210	(GCG)5	(GCG)6	(GCG)5	(GCG)5	(GCG)5	(GCG)5	(GCG)5	(GCG)5	(GCG)6	(GCG)5	(GCG)5	-	Introns/Intergenic	-	-	CCGAGGATCATATCCCCTCT	GGCCCAAAACCTCTCTTCTC	217	P	A	A	NA
PMS3963	Os_Chr07	19882679	19882690	(TA)6	(TA)6	(TA)6	(TA)8	(TA)8	(TA)6	(TA)6	(TA)6	(TA)6	(TA)6	(TA)6	-	Introns/Intergenic	-	-	CCTATCTCGTGTGGCTCCAT	CCGATCGTTTCGCTTATTCT	252	P	A	A	NA
PMS3964	Os_Chr07	19917080	19917097	(CGG)6	(CGG)6	(CGG)6	(CGG)6	(CGG)5	(CGG)6	(CGG)6	(CGG)6	(CGG)6	(CGG)6	(CGG)6	LOC_Os07g33320	CDS	expressed protein	CODON_DELETION	GATCACGATGATGATGCTGC	CTTGTGGATCAGGTACTGCG	130	P	A	A	NA

Polymorphic SSR (PMS) markers	Chromosomes	Start physical positions (bp)	End physical positions (bp)	Nipponbare	Kasalath	Nagina22	IR64	Pokkali	Bala	Tainung67	Azucena	Moroberekan	*Oryza rufipogon*	*Oryza nivara*	MSU gene locus IDs	Structural annotation	Functional annotation	SSR functional effects	Forward primers (5'-3')	Reverse primers (5'-3')	Amplified product size (bp)	Markers exhibiting polymorphism within *indica* rice	Transcrption factor genes-derived markers	Known cloned genes-derived markers	PMS markers corresponding with RM markers
PMS3965	Os_Chr07	19931964	19931978	(GGC)5	(GGC)5	(GGC)5	(GGC)6	(GGC)6	(GGC)6	(GGC)5	(GGC)5	(GGC)6	(GGC)5	(GGC)6	LOC_Os07g33340	URR	DEAD-box ATP-dependent RNA helicase, putative, expressed	-	AGGAGGAGGACTTGGAGAGG	AGAGCCACCAGAAAATCCCT	227	P	A	A	NA
PMS3966	Os_Chr07	19936126	19936140	(CGG)5	(CGG)5	(CGG)5	(CGG)5	(CGG)5	(CGG)7	(CGG)5	(CGG)5	(CGG)5	(CGG)5	(CGG)5	LOC_Os07g33350	CDS	hsp20/alpha crystallin family protein, putative, expressed	CODON_CHANGE_PLUS_CODON_INSERTION	GTGCTCAGGGTAACCGTCC	CCCCAATCCTAAGCTCACAG	190	P	A	A	NA
PMS3967	Os_Chr07	19938625	19938642	(GGC)6	(GGC)6	(GGC)6	(GGC)6	(GGC)6	(GGC)6	(GGC)5	(GGC)6	(GGC)6	(GGC)6	(GGC)6	LOC_Os07g33360	CDS	expressed protein	CODON_DELETION	AGGTTTTGGTGGAAAGGAGG	AAAAGAAAACCCCACCTTCG	246	A	A	A	NA
PMS3968	Os_Chr07	19957328	19957351	(CGG)8	(CGG)7	(CGG)8	(CGG)8	(CGG)8	(CGG)7	(CGG)8	(CGG)8	(CGG)8	(CGG)8	(CGG)8	LOC_Os07g33380	CDS	expressed protein	CODON_CHANGE_PLUS_CODON_DELETION	GTCGGATGGACTCCTCCTC	AGGAGGTTGCTGTCCTCGTA	248	P	A	A	NA
PMS3969	Os_Chr07	19963145	19963162	(CGA)6	(CGA)7	(CGA)6	(CGA)6	(CGA)6	(CGA)7	(CGA)6	(CGA)7	(CGA)7	(CGA)6	(CGA)5	LOC_Os07g33390	CDS	BCS1 protein, putative, expressed	CODON_CHANGE_PLUS_CODON_DELETION	CTCTCCAGAGGCTGCTCATT	CGATGTGTTCTTGGTGGATG	210	P	A	A	NA
PMS3970	Os_Chr07	19975560	19975573	(AT)7	(AT)7	(AT)7	(AT)6	(AT)6	(AT)6	(AT)7	(AT)7	(AT)7	(AT)6	(AT)6	LOC_Os07g33420	DRR	hydroxylase, putative, expressed	-	AAGGAAGGTTGGGGTACACA	TGGCCCATAGGTTTGAACTC	207	P	A	A	NA
PMS3971	Os_Chr07	20066835	20066848	(CA)7	(CA)6	(CA)7	(CA)7	(CA)7	(CA)7	(CA)7	(CA)7	(CA)7	(CA)7	(CA)7	-	Introns/Intergenic	-	-	AAGGCTACAATGATGTTGGG	TAGGAAGGGGCCAAGTAGGT	112	P	A	A	NA
PMS3972	Os_Chr07	20093335	20093348	(GA)7	(GA)7	(GA)7	(GA)7	(GA)7	(GA)7	(GA)7	(GA)8	(GA)8	(GA)7	(GA)7	LOC_Os07g33630	URR	HEAT repeat family protein, putative, expressed	-	GGATCGATCGGAGGGTTT	CTCCTCATCGTCTCGACTCC	183	A	A	A	NA
PMS3973	Os_Chr07	20187925	20187939	(CAG)5	(CAG)5	(CAG)5	(CAG)5	(CAG)5	(CAG)5	(CAG)5	(CAG)6	(CAG)6	(CAG)5	(CAG)5	-	Introns/Intergenic	-	-	ATATAACCGACGATGCGGAC	CTGCCTTGAAGTGACGATGA	144	A	A	A	NA
PMS3974	Os_Chr07	20202984	20202999	(GA)8	(GA)8	(GA)8	(GA)8	(GA)8	(GA)10	(GA)8	(GA)7	(GA)8	(GA)8	(GA)8	-	Introns/Intergenic	-	-	TCTCCCGGCTAGGAGAGTTT	ATGCGGACCTCTTGAGAGAA	200	P	A	A	NA
PMS3975	Os_Chr07	20236518	20236533	(TC)8	(TC)10	(TC)8	(TC)8	(TC)8	(TC)8	(TC)8	(TC)8	(TC)8	(TC)8	(TC)8	-	Introns/Intergenic	-	-	ATAAAACGGACGGTCAAACG	TCATGTCCAGACAAAACGGA	205	P	A	A	NA
PMS3976	Os_Chr07	20267838	20267861	(CCT)8	(CCT)7	(CCT)7	(CCT)7	(CCT)7	(CCT)7	(CCT)7	(CCT)5	(CCT)7	(CCT)7	(CCT)7	-	Introns/Intergenic	-	-	TCTCTCTCCTTCTCCTCCCC	AACACCATGAGAAAGACGGC	199	A	A	A	NA
PMS3977	Os_Chr07	20286058	20286078	(CCT)7	(CCT)8	(CCT)7	(CCT)8	(CCT)7	(CCT)8	(CCT)8	(CCT)5	(CCT)8	(CCT)8	(CCT)8	-	Introns/Intergenic	-	-	TCTCTCTCCTTCTCCTCCCC	AACACCATGAGAAAGACGGC	196	P	A	A	NA

Polymorphic SSR (PMS) markers	Chromosomes	Start physical positions (bp)	End physical positions (bp)	Nipponbare	Kasalath	Nagina22	IR64	Pokkali	Bala	Tainung67	Azucena	Moroberekan	*Oryza rufipogon*	*Oryza nivara*	MSU gene locus IDs	Structural annotation	Functional annotation	SSR functional effects	Forward primers (5'-3')	Reverse primers (5'-3')	Amplified product size (bp)	Markers exhibiting polymorphism within *indica* rice	Transcrption factor genes-derived markers	Known cloned genes-derived markers	PMS markers corresponding with RM markers
PMS3978	Os_Chr07	20297795	20297815	(CGG)7	(CGG)7	(CGG)7	(CGG)7	(CGG)7	(CGG)7	(CGG)7	(CGG)7	(CGG)7	(CGG)7	(CGG)5	LOC_Os07g33954	CDS	transporter family protein, putative, expressed	CODON_DELETION	CGAAGTAGACCCCGATCTTG	ATTATTCAAGCCCCTCCACC	251	A	A	A	NA
PMS3979	Os_Chr07	20304277	20304297	(CCT)7	(CCT)8	(CCT)7	(CCT)8	(CCT)7	(CCT)8	(CCT)8	(CCT)5	(CCT)8	(CCT)8	(CCT)8	-	Introns/Intergenic	-	-	TCTCTCTCCTTCTCCTCCCC	AACACCATGAGAAAGACGGC	196	P	A	A	NA
PMS3980	Os_Chr07	20316013	20316033	(CGG)7	(CGG)7	(CGG)7	(CGG)7	(CGG)7	(CGG)7	(CGG)7	(CGG)7	(CGG)7	(CGG)7	(CGG)5	LOC_Os07g34006	CDS	transporter family protein, putative, expressed	CODON_DELETION	CGAAGTAGACCCCGATCTTG	ATTATTCAAGCCCCTCCACC	251	A	A	A	NA
PMS3981	Os_Chr07	20351804	20351843	(AT)20	(AT)12	(AT)20	(AT)20	(AT)20	(AT)20	(AT)20	(AT)10	(AT)20	(AT)10	(AT)9	-	Introns/Intergenic	-	-	TGCTCTTGCTTTGAGCAATTT	TCAATTTCAACAATGGTGCC	182	P	A	A	NA
PMS3982	Os_Chr07	20492486	20492503	(GA)9	(GA)9	(GA)9	(GA)9	(GA)9	(GA)10	(GA)9	(GA)9	(GA)9	(GA)9	(GA)10	-	Introns/Intergenic	-	-	GAGCGAGGGGAAAGGGAG	CCGCAAAAGTCTAGTTTGCC	276	P	A	A	NA
PMS3983	Os_Chr07	20582174	20582197	(TA)12	(TA)12	(TA)12	(TA)12	(TA)12	(TA)15	(TA)12	(TA)12	(TA)12	(TA)12	(TA)12	-	Introns/Intergenic	-	-	TTCGTATACCGCTGATGCAA	CATATGCATCACCATCACGC	173	P	A	A	NA
PMS3984	Os_Chr07	20592012	20592041	(AT)15	(AT)15	(AT)15	(AT)15	(AT)15	(AT)15	(AT)6	(AT)15	(AT)15	(AT)15	(AT)15	-	Introns/Intergenic	-	-	TGTACTGGTTTCTGAGAGTTGAGG	CATTCAATCGACCAGACTAACG	291	A	A	A	RM21755
PMS3985	Os_Chr07	20600594	20600607	(TA)7	(TA)7	(TA)7	(TA)8	(TA)7	(TA)8	(TA)7	(TA)7	(TA)9	(TA)7	(TA)7	-	Introns/Intergenic	-	-	TTCAAAGCAACATGCCTACG	TTGGATTTATGCGTATATAAAATTGG	257	P	A	A	NA
PMS3986	Os_Chr07	20673384	20673399	(GA)8	(GA)8	(GA)8	(GA)8	(GA)8	(GA)7	(GA)8	(GA)8	(GA)8	(GA)8	(GA)8	-	Introns/Intergenic	-	-	ATTGAATCAAGCGGATTTGG	CTCCCCCTCCTCTCCTCCT	236	P	A	A	NA
PMS3987	Os_Chr07	20711010	20711053	(TA)22	(TA)22	(TA)22	(TA)22	(TA)9	(TA)22	(TA)22	(TA)22	(TA)12	(TA)8	(TA)22	-	Introns/Intergenic	-	-	GCACGGTCTCCGAAAAGTAG	GTCGTTTCGGACAAGGGTAA	210	P	A	A	NA
PMS3988	Os_Chr07	20885696	20885713	(CGG)6	(CGG)6	(CGG)6	(CGG)6	(CGG)6	(CGG)6	(CGG)6	(CGG)6	(CGG)6	(CGG)5	(CGG)6	LOC_Os07g34850	CDS	aspartic proteinase nepenthesin, putative, expressed	CODON_DELETION	ACGGCTACGAGATGGTGAAG	TCAACTCCGGCGAAGTAAGT	193	A	A	A	NA
PMS3989	Os_Chr07	20899763	20899810	(ATAG)12	(ATAG)11	(ATAG)11	(ATAG)12	(ATAG)12	(ATAG)12	(ATAG)12	(ATAG)11	(ATAG)12	(ATAG)12	(ATAG)10	-	Introns/Intergenic	-	-	ATGTGGGAATTTCTAGCCCC	AAAATAGGAAGGAAACCCGC	138	P	A	A	NA
PMS3990	Os_Chr07	20901525	20901545	(TGG)7	(TGG)7	(TGG)7	(TGG)7	(TGG)7	(TGG)7	(TGG)5	(TGG)7	(TGG)7	(TGG)6	(TGG)7	LOC_Os07g34870	CDS	aspartic proteinase nepenthesin precursor, putative, expressed	CODON_DELETION	CGAGCAGTGTCTGGAAGCTCACC	CTCGCGTTCCTCTCCAACAACG	97	A	A	A	RM21764

Polymorphic SSR (PMS) markers	Chromosomes	Start physical positions (bp)	End physical positions (bp)	Nipponbare	Kasalath	Nagina22	IR64	Pokkali	Bala	Tainung67	Azucena	Moroberekan	*Oryza rufipogon*	*Oryza nivara*	MSU gene locus IDs	Structural annotation	Functional annotation	SSR functional effects	Forward primers (5'-3')	Reverse primers (5'-3')	Amplified product size (bp)	Markers exhibiting polymorphism within *indica* rice	Transcrption factor genes-derived markers	Known cloned genes-derived markers	PMS markers corresponding with RM markers
PMS3991	Os_Chr07	21010622	21010633	(GA)6	(GA)6	(GA)6	(GA)6	(GA)6	(GA)6	(GA)6	(GA)6	(GA)6	(GA)7	(GA)6	-	Introns/Intergenic	-	-	GGGTTACCGGTTTTCAGACA	GGTTCGAAGCCTCACTCCTT	271	A	A	A	NA
PMS3992	Os_Chr07	21097156	21097200	(TTA)15	(TTA)10	(TTA)10	(TTA)15	(TTA)6	(TTA)15	(TTA)9	(TTA)15	(TTA)15	(TTA)15	(TTA)15	-	Introns/Intergenic	-	-	GGGTATGAACACATGATTGAGTGG	AAACAACGAAGAGCAGGAAGG	338	P	A	A	RM21769
PMS3993	Os_Chr07	21266691	21266711	(ACG)7	(ACG)7	(ACG)7	(ACG)7	(ACG)7	(ACG)7	(ACG)7	(ACG)7	(ACG)7	(ACG)7	(ACG)6	LOC_Os07g35540	CDS	TKL_IRAK_DUF26-lc.19 - DUF26 kinases have homology to DUF26 containing loci, expressed	CODON_DELETION	TGCTCCTCGTCCTAAACAGTTGG	CCGTGGCGAAATACTTGATGG	186	A	A	A	RM21772
PMS3994	Os_Chr07	21322823	21322842	(AG)10	(AG)6	(AG)6	(AG)10	(AG)10	(AG)10	(AG)10	(AG)10	(AG)10	(AG)10	(AG)10	-	Introns/Intergenic	-	-	AAGCTCGGAGAAAGATGCAA	CCATGGATGCAATGCAATAG	220	P	A	A	NA
PMS3995	Os_Chr07	21382484	21382495	(AT)6	(AT)7	(AT)6	(AT)6	(AT)6	(AT)6	(AT)6	(AT)6	(AT)6	(AT)6	(AT)6	-	Introns/Intergenic	-	-	CACTTAAATTGGTGGACCCG	CCGTTAACCTCCTTCCACAA	274	P	A	A	NA
PMS3996	Os_Chr07	21505468	21505479	(AT)6	(AT)7	(AT)6	(AT)6	(AT)6	(AT)6	(AT)6	(AT)6	(AT)6	(AT)6	(AT)6	-	Introns/Intergenic	-	-	ATGCTAAAGATCTCGGCGAA	GGAGATCTGCCAGCAAACAT	169	P	A	A	NA
PMS3997	Os_Chr07	21629077	21629094	(TAA)6	(TAA)6	(TAA)6	(TAA)6	(TAA)6	(TAA)6	(TAA)6	(TAA)6	(TAA)6	(TAA)6	(TAA)5	-	Introns/Intergenic	-	-	ACAAGGCATACCGCATGATT	TTAGCCCCTGTGGAATTTTG	253	A	A	A	NA
PMS3998	Os_Chr07	21646563	21646574	(AT)6	(AT)7	(AT)7	(AT)6	(AT)6	(AT)6	(AT)6	(AT)6	(AT)6	(AT)6	(AT)6	-	Introns/Intergenic	-	-	TGCCATGATGACTCGATGTT	GAAATGGCTGTTCTGTGGCT	244	P	A	A	NA
PMS3999	Os_Chr07	21699479	21699492	(TA)7	(TA)7	(TA)7	(TA)6	(TA)7	(TA)7	(TA)7	(TA)7	(TA)7	(TA)7	(TA)7	-	Introns/Intergenic	-	-	CTCGTTGGTGGGATTTTGTC	TTGAAGATGCGACTTTGCAC	280	P	A	A	NA
PMS4000	Os_Chr07	21816534	21816551	(TC)9	(TC)10	(TC)9	(TC)10	(TC)9	(TC)10	(TC)9	(TC)9	(TC)9	(TC)9	(TC)10	-	Introns/Intergenic	-	-	GACGAGGCGAAGAACAAAAC	ACGGGGAAGAGAGGAGAAGA	228	P	A	A	NA
PMS4001	Os_Chr07	21846753	21846770	(CCG)6	(CCG)6	(CCG)6	(CCG)6	(CCG)6	(CCG)6	(CCG)6	(CCG)6	(CCG)6	(CCG)7	(CCG)6	LOC_Os07g36544	DRR	serine/threonine-protein kinase receptor precursor, putative, expressed	-	ACCTCCGACCTGCTGTACC	CACGTAAGCAGCGATCAAGA	182	A	P	A	NA
PMS4002	Os_Chr07	21850288	21850305	(CCG)6	(CCG)5	(CCG)6	(CCG)6	(CCG)6	(CCG)6	(CCG)6	(CCG)6	(CCG)6	(CCG)6	(CCG)5	LOC_Os07g36544	CDS	serine/threonine-protein kinase receptor precursor, putative, expressed	CODON_DELETION	ACTGGTTCAGCACGAACCTC	GGGTTCATCTGGTACAACGG	205	P	P	A	NA
PMS4003	Os_Chr07	21872237	21872290	(GAA)18	(GAA)17	(GAA)18	(GAA)18	(GAA)18	(GAA)18	(GAA)18	(GAA)18	(GAA)18	(GAA)18	(GAA)17	LOC_Os07g36580	CDS	expressed protein	CODON_CHANGE_PLUS_CODON_DELETION	GTATCTTACAGAGAAACGGCATCG	GGTTTGTTTCAGGTTCGTCTATCC	156	P	A	A	RM336

Polymorphic SSR (PMS) markers	Chromosomes	Start physical positions (bp)	End physical positions (bp)	Nipponbare	Kasalath	Nagina22	IR64	Pokkali	Bala	Tainung67	Azucena	Moroberekan	*Oryza rufipogon*	*Oryza nivara*	MSU gene locus IDs	Structural annotation	Functional annotation	SSR functional effects	Forward primers (5'-3')	Reverse primers (5'-3')	Amplified product size (bp)	Markers exhibiting polymorphism within *indica* rice	Transcrption factor genes-derived markers	Known cloned genes-derived markers	PMS markers corresponding with RM markers
PMS4004	Os_Chr07	21922226	21922237	(CT)6	(CT)6	(CT)6	(CT)6	(CT)6	(CT)7	(CT)6	(CT)6	(CT)6	(CT)6	(CT)6	-	Introns/Intergenic	-	-	GACTCTTGTTGAAGTCGGGG	TGTAGCCCGCTGATTTTCTC	280	P	A	A	NA
PMS4005	Os_Chr07	22019046	22019063	(CGC)6	(CGC)5	(CGC)6	(CGC)6	(CGC)6	(CGC)6	(CGC)6	(CGC)6	(CGC)5	(CGC)5	(CGC)6	LOC_Os07g36750	CDS	CSLF3 - cellulose synthase-like family F; beta1,3;1,4 glucan synthase, expressed	CODON_DELETION	TGTACGACCTGAACAGCAGC	GGCTTAAGCTTGCCAGTGAG	279	P	A	A	NA
PMS4006	Os_Chr07	22051613	22051633	(TCG)7	(TCG)7	(TCG)7	(TCG)7	(TCG)7	(TCG)7	(TCG)7	(TCG)7	(TCG)7	(TCG)5	(TCG)7	LOC_Os07g36810	CDS	expressed protein	CODON_DELETION	CAAGAAGTACTCTTCGGCGG	ACAGCGACGACAAGGAGC	182	A	A	A	NA
PMS4007	Os_Chr07	22059663	22059682	(CT)10	(CT)10	(CT)10	(CT)10	(CT)10	(CT)10	(CT)10	(CT)7	(CT)10	(CT)10	(CT)10	LOC_Os07g36820	URR	uncharacterized Cys-rich domain containing protein, putative, expressed	-	CACAGCATGTCGAAGTCCAG	GAGGCAAATACGAAACGGAA	183	A	A	A	NA
PMS4008	Os_Chr07	22107716	22107733	(CCG)6	(CCG)6	(CCG)5	(CCG)6	(CCG)6	(CCG)6	(CCG)6	(CCG)6	(CCG)6	(CCG)6	(CCG)6	LOC_Os07g36920	CDS	OsFBX255 - F-box domain containing protein, expressed	CODON_CHANGE_PLUS_CODON_DELETION	CCCACACACACCAATCTGTC	GACGAAGATCTCCCTCAGGA	208	P	A	A	NA
PMS4009	Os_Chr07	22114864	22114875	(TC)6	(TC)6	(TC)6	(TC)6	(TC)6	(TC)6	(TC)6	(TC)6	(TC)7	(TC)6	(TC)6	-	Introns/Intergenic	-	-	CAGTAGTAGAAGGACAAGTCCCAGA	ACGGACGAAGAAATTGATGG	195	A	A	A	NA
PMS4010	Os_Chr07	22188794	22188814	(CCT)7	(CCT)7	(CCT)7	(CCT)7	(CCT)7	(CCT)7	(CCT)5	(CCT)7	(CCT)7	(CCT)7	(CCT)7	LOC_Os07g37040	CDS	PB1 domain containing protein, expressed	CODON_DELETION	CGATCTCCTCGATGAGGTTGTCC	CCGCCTACGACTGCCAAATCC	456	A	A	A	RM21809
PMS4011	Os_Chr07	22299765	22299776	(AT)6	(AT)10	(AT)6	(AT)6	(AT)6	(AT)6	(AT)6	(AT)6	(AT)6	(AT)6	(AT)6	-	Introns/Intergenic	-	-	TCGTTTAAGACAGCGACACG	TGACCTAAAAATATGGGGAGTCA	279	P	A	A	NA
PMS4012	Os_Chr07	22313445	22313462	(CGG)6	(CGG)6	(CGG)6	(CGG)6	(CGG)6	(CGG)6	(CGG)6	(CGG)5	(CGG)6	(CGG)6	(CGG)6	LOC_Os07g37230	CDS	inositol-1-monophosphatase, putative, expressed	CODON_DELETION	ACGGTTCACCTTACGCAGAC	ATCACGTGTTGTATTCGCCA	220	A	A	A	NA
PMS4013	Os_Chr07	22386687	22386710	(GGA)8	(GGA)8	(GGA)8	(GGA)8	(GGA)8	(GGA)8	(GGA)7	(GGA)8	(GGA)8	(GGA)8	(GGA)8	-	Introns/Intergenic	-	-	AACTCCGTCTCCACGAACAC	CTCGCCTTCCTCTATCTCCC	266	A	A	A	NA
PMS4014	Os_Chr07	22426878	22426891	(AG)7	(AG)8	(AG)7	(AG)8	(AG)8	(AG)8	(AG)7	(AG)7	(AG)7	(AG)7	(AG)7	-	Introns/Intergenic	-	-	AGAAATTCACGTGTTTGCCC	GATGTAGGTCTCGATGGCGT	246	P	A	A	NA
PMS4015	Os_Chr07	22580544	22580564	(CCT)7	(CCT)7	(CCT)7	(CCT)7	(CCT)7	(CCT)7	(CCT)5	(CCT)7	(CCT)7	(CCT)7	(CCT)7	-	Introns/Intergenic	-	-	ACAGCACGAAGATGGAGGAGACG	GGTAGAGCAGGTAGGCGAAGTGG	251	A	A	A	RM21834
PMS4016	Os_Chr07	22619513	22619530	(GGC)6	(GGC)6	(GGC)6	(GGC)6	(GGC)6	(GGC)6	(GGC)5	(GGC)6	(GGC)6	(GGC)5	(GGC)6	LOC_Os07g37730	CDS	NADH-ubiquinone oxidoreductase, mitochondrial precursor, putative, expressed	CODON_DELETION	GATCAGCACGACGTCACCT	ACGTCCTTGAGGAACCTGC	192	A	A	A	NA

Polymorphic SSR (PMS) markers	Chromosomes	Start physical positions (bp)	End physical positions (bp)	Nipponbare	Kasalath	Nagina22	IR64	Pokkali	Bala	Tainung67	Azucena	Moroberekan	*Oryza rufipogon*	*Oryza nivara*	MSU gene locus IDs	Structural annotation	Functional annotation	SSR functional effects	Forward primers (5'-3')	Reverse primers (5'-3')	Amplified product size (bp)	Markers exhibiting polymorphism within *indica* rice	Transcrption factor genes-derived markers	Known cloned genes-derived markers	PMS markers corresponding with RM markers
PMS4017	Os_Chr07	22680336	22680353	(GTG)6	(GTG)6	(GTG)5	(GTG)5	(GTG)6	(GTG)6	(GTG)5	(GTG)5	(GTG)6	(GTG)5	(GTG)5	-	Introns/Intergenic	-	-	GCCACCACAAAAAGGAGAAA	GCAACTGATGAAAAGCCGTT	245	P	A	A	NA
PMS4018	Os_Chr07	22717762	22717776	(TAA)5	(TAA)5	(TAA)5	(TAA)5	(TAA)5	(TAA)5	(TAA)5	(TAA)5	(TAA)5	(TAA)7	(TAA)5	-	Introns/Intergenic	-	-	AGTTGTGGGTTTCCGTGTTC	TCCAACGTTCGACTGTTCAT	171	A	A	A	NA
PMS4019	Os_Chr07	22959066	22959083	(CT)9	(CT)9	(CT)9	(CT)9	(CT)9	(CT)9	(CT)6	(CT)9	(CT)9	(CT)9	(CT)9	-	Introns/Intergenic	-	-	AGTCGAGGCGACGACGAG	GTGTCAAACAGCGTAGCCAA	227	A	A	A	NA
PMS4020	Os_Chr07	23092307	23092327	(GCG)7	(GCG)7	(GCG)7	(GCG)7	(GCG)7	(GCG)7	(GCG)6	(GCG)7	(GCG)7	(GCG)7	(GCG)7	LOC_Os07g38430	CDS	WD domain, G-beta repeat domain containing protein, expressed	CODON_CHANGE_PLUS_CODON_DELETION	CGGACTCGTTCTACCTCTGC	AAGTTGGCGCTAAAGACGAA	190	A	A	A	NA
PMS4021	Os_Chr07	23156865	23156882	(GCG)6	(GCG)6	(GCG)6	(GCG)6	(GCG)6	(GCG)6	(GCG)5	(GCG)6	(GCG)6	(GCG)6	(GCG)6	LOC_Os07g38550	CDS	expressed protein	CODON_CHANGE_PLUS_CODON_DELETION	AGGAGATCATGGCGAGGAG	AAGTTGAGCGCGTAGCTCTT	100	A	A	A	NA
PMS4022	Os_Chr07	23160759	23160788	(GAG)10	(GAG)10	(GAG)10	(GAG)10	(GAG)10	(GAG)10	(GAG)10	(GAG)10	(GAG)10	(GAG)10	(GAG)6	LOC_Os07g38560	CDS	adenylyl-sulfate kinase, putative, expressed	CODON_DELETION	AGGATAACCGCTGCAGTCAC	GCATTGCATCAACTCATCGT	245	A	A	A	NA
PMS4023	Os_Chr07	23166162	23166176	(TCC)5	(TCC)5	(TCC)5	(TCC)5	(TCC)5	(TCC)5	(TCC)5	(TCC)5	(TCC)5	(TCC)6	(TCC)5	LOC_Os07g38570	CDS	expressed protein	CODON_INSERTION	CTTCGTGTTCTTCTGCCTCC	TTCGATGCGTCCTCCTAATC	240	A	A	A	NA
PMS4024	Os_Chr07	23184225	23184240	(CT)8	(CT)8	(CT)8	(CT)8	(CT)8	(CT)7	(CT)8	(CT)8	(CT)8	(CT)8	(CT)8	-	Introns/Intergenic	-	-	GCTGTGACAGTGGTGAGCAT	GAAATGGGCGAAACAAAAGA	251	P	A	A	NA
PMS4025	Os_Chr07	23229642	23229713	(TATC)18	(TATC)18	(TATC)18	(TATC)18	(TATC)18	(TATC)18	(TATC)18	(TATC)13	(TATC)18	(TATC)18	(TATC)18	-	Introns/Intergenic	-	-	GTTTCGGTTTTGCACCATTT	CGAACGTGATGCCTCTTTTC	190	A	A	A	NA
PMS4026	Os_Chr07	23305294	23305309	(TA)8	(TA)6	(TA)8	(TA)6	(TA)6	(TA)6	(TA)8	(TA)6	(TA)8	(TA)8	(TA)6	-	Introns/Intergenic	-	-	AATCCCCATGTTTATGCTGC	TGAGAAGAAAACCACGGACC	136	P	A	A	NA
PMS4027	Os_Chr07	23423749	23423766	(TGG)6	(TGG)6	(TGG)6	(TGG)6	(TGG)6	(TGG)6	(TGG)5	(TGG)6	(TGG)6	(TGG)6	(TGG)6	LOC_Os07g39080	CDS	expressed protein	CODON_DELETION	CCGTCCTTGCATTTCTTGAT	AAACAGGAGGAGCGGCTT	251	A	A	A	NA
PMS4028	Os_Chr07	23450289	23450322	(GA)17	(GA)17	(GA)17	(GA)17	(GA)17	(GA)17	(GA)17	(GA)17	(GA)16	(GA)10	(GA)17	-	Introns/Intergenic	-	-	AGAGCGAGAGCGAGAGAATG	GATCTCGAATCTCCGATCCA	270	A	A	A	NA
PMS4029	Os_Chr07	23462207	23462220	(GA)7	(GA)7	(GA)7	(GA)7	(GA)7	(GA)7	(GA)7	(GA)6	(GA)7	(GA)7	(GA)7	-	Introns/Intergenic	-	-	CGGAGGAGGAGGAGCACT	TCGAGTTCAAATCCGAATCC	257	A	A	A	NA

Polymorphic SSR (PMS) markers	Chromosomes	Start physical positions (bp)	End physical positions (bp)	Nipponbare	Kasalath	Nagina22	IR64	Pokkali	Bala	Tainung67	Azucena	Moroberekan	*Oryza rufipogon*	*Oryza nivara*	MSU gene locus IDs	Structural annotation	Functional annotation	SSR functional effects	Forward primers (5'-3')	Reverse primers (5'-3')	Amplified product size (bp)	Markers exhibiting polymorphism within *indica* rice	Transcrption factor genes-derived markers	Known cloned genes-derived markers	PMS markers corresponding with RM markers
PMS4030	Os_Chr07	23524220	23524251	(CT)16	(CT)16	(CT)16	(CT)15	(CT)15	(CT)16	(CT)16	(CT)16	(CT)16	(CT)16	(CT)15	-	Introns/Intergenic	-	-	TGAACTGATCGTGATTGTGATG	GAAAACCACATCTGAAAGCCA	229	P	A	A	NA
PMS4031	Os_Chr07	23527269	23527290	(CT)11	(CT)11	(CT)11	(CT)11	(CT)11	(CT)8	(CT)11	(CT)11	(CT)11	(CT)11	(CT)11	LOC_Os07g39300	URR	expressed protein	-	GTGACTGGTGAGGGGGTCT	TGGAGTGGTAGTGGGAGAGG	127	P	A	A	NA
PMS4032	Os_Chr07	23624282	23624299	(TAA)6	(TAA)5	(TAA)5	(TAA)5	(TAA)5	(TAA)5	(TAA)6	(TAA)6	(TAA)6	(TAA)6	(TAA)5	-	Introns/Intergenic	-	-	TGACCTCACTGCGTCTGTGT	GCCTTGGTCTCTACTGAGCG	150	A	A	A	NA
PMS4033	Os_Chr07	23687737	23687754	(GTC)6	(GTC)6	(GTC)6	(GTC)6	(GTC)6	(GTC)6	(GTC)6	(GTC)6	(GTC)6	(GTC)5	(GTC)6	LOC_Os07g39530	CDS	BTBN16 - Bric-a-Brac, Tramtrack, Broad Complex BTB domain with non-phototropic hypocotyl 3 NPH3 domain, expressed	CODON_DELETION	GAGTAGGATCCTGCGCATCT	CTGCGTGCTTCGTTCTACAG	152	A	A	A	NA
PMS4034	Os_Chr07	23797428	23797445	(CGG)6	(CGG)5	(CGG)6	(CGG)6	(CGG)6	(CGG)6	(CGG)6	(CGG)6	(CGG)6	(CGG)6	(CGG)6	LOC_Os07g39700	CDS	Sdr4	CODON_DELETION	GGTGAACGACGCGTACAAG	CGAAGACGTAGTCCCTCGAC	266	P	A	P	NA
PMS4035	Os_Chr07	23849153	23849180	(TC)14	(TC)13	(TC)14	(TC)14	(TC)14	(TC)14	(TC)14	(TC)14	(TC)14	(TC)14	(TC)14	-	Introns/Intergenic	-	-	CCCGGCCGTTAATGTTAGTA	GCAAAGGCACCCAACTATGT	246	P	A	A	NA
PMS4036	Os_Chr07	23887240	23887261	(TA)11	(TA)11	(TA)11	(TA)11	(TA)11	(TA)8	(TA)11	(TA)11	(TA)11	(TA)11	(TA)11	-	Introns/Intergenic	-	-	GCATGTTTGATCGTTCGTCTT	TATTAGCTTTACGGCCGCTG	273	P	A	A	NA
PMS4037	Os_Chr07	23920865	23920880	(TG)8	(TG)8	(TG)7	(TG)8	(TG)8	(TG)8	(TG)8	(TG)8	(TG)8	(TG)8	(TG)8	-	Introns/Intergenic	-	-	GATGCACTGATGCTGATGCT	CGAGGTGAAGAACAGGAAGC	149	P	A	A	NA
PMS4038	Os_Chr07	23926005	23926031	(CCG)9	(CCG)9	(CCG)9	(CCG)9	(CCG)9	(CCG)9	(CCG)9	(CCG)9	(CCG)9	(CCG)8	(CCG)9	-	Introns/Intergenic	-	-	GAGAGGAAACAGCTTCCGTG	GAGGGATGGAGGAAGGAAAG	201	A	A	A	NA
PMS4039	Os_Chr07	23985517	23985540	(CT)12	(CT)13	(CT)12	(CT)12	(CT)12	(CT)12	(CT)12	(CT)12	(CT)12	(CT)12	(CT)12	-	Introns/Intergenic	-	-	TCAAGGTCGACATGTTAGGTATGC	AACCCTATCACCTGAGAAACATCC	116	P	A	A	RM1132
PMS4040	Os_Chr07	23997082	23997099	(CGA)6	(CGA)6	(CGA)6	(CGA)6	(CGA)6	(CGA)6	(CGA)6	(CGA)5	(CGA)6	(CGA)6	(CGA)6	LOC_Os07g40000	CDS	DUF260 domain containing protein, putative, expressed	CODON_CHANGE_PLUS_CODON_DELETION	GGTGCTCCACGTTCTCGAC	ATCCCTCGGAGTTCATCGAC	210	A	P	A	NA
PMS4041	Os_Chr07	24035406	24035420	(GCT)5	(GCT)6	(GCT)5	(GCT)5	(GCT)5	(GCT)6	(GCT)5	(GCT)5	(GCT)5	(GCT)5	(GCT)5	LOC_Os07g40040	CDS	expressed protein	CODON_INSERTION	TCTTGGAATCTGTGGAGCGT	GGAGCGAGTCGTCCAAGAG	261	P	A	A	NA
PMS4042	Os_Chr07	24064613	24064630	(TA)9	(TA)9	(TA)9	(TA)9	(TA)9	(TA)9	(TA)9	(TA)9	(TA)9	(TA)9	(TA)6	-	Introns/Intergenic	-	-	TTTTTCCATTGGAGGAGTCG	CTTCCGTTGTCCGTGAAAAT	165	A	A	A	NA

Polymorphic SSR (PMS) markers	Chromosomes	Start physical positions (bp)	End physical positions (bp)	Nipponbare	Kasalath	Nagina22	IR64	Pokkali	Bala	Tainung67	Azucena	Moroberekan	*Oryza rufipogon*	*Oryza nivara*	MSU gene locus IDs	Structural annotation	Functional annotation	SSR functional effects	Forward primers (5'-3')	Reverse primers (5'-3')	Amplified product size (bp)	Markers exhibiting polymorphism within *indica* rice	Transcrption factor genes-derived markers	Known cloned genes-derived markers	PMS markers corresponding with RM markers
PMS4043	Os_Chr07	24289013	24289028	(TA)8	(TA)8	(TA)8	(TA)8	(TA)8	(TA)8	(TA)8	(TA)8	(TA)8	(TA)8	(TA)6	-	Introns/Intergenic	-	-	AGCACTCGGCTTTGGAGTAA	AAACCAGGACTTTAGGCCGT	226	A	A	A	NA
PMS4044	Os_Chr07	24298329	24298346	(CAC)6	(CAC)6	(CAC)6	(CAC)6	(CAC)6	(CAC)6	(CAC)6	(CAC)6	(CAC)6	(CAC)5	(CAC)6	LOC_Os07g40550	URR	IBS1, putative, expressed	-	GTCCCTCTTCTTCTCCGACC	ACGGAGACCTGAGTCCAATG	251	A	A	A	NA
PMS4045	Os_Chr07	24432605	24432628	(GGC)8	(GGC)8	(GGC)8	(GGC)8	(GGC)8	(GGC)8	(GGC)8	(GGC)7	(GGC)8	(GGC)8	(GGC)8	-	Introns/Intergenic	-	-	GACCGCGAGTTCAGTTCC	CTGCTTTACTCCGTAGCGGC	275	A	A	A	NA
PMS4046	Os_Chr07	24450791	24450814	(AT)12	(AT)12	(AT)9	(AT)12	(AT)12	(AT)12	(AT)12	(AT)12	(AT)12	(AT)12	(AT)7	-	Introns/Intergenic	-	-	ATTAGATACCATGGTGCCCG	CCTGTTGAGTGCAGGAACAA	191	P	A	A	NA
PMS4047	Os_Chr07	24477915	24477932	(CCG)6	(CCG)6	(CCG)6	(CCG)6	(CCG)6	(CCG)5	(CCG)6	(CCG)6	(CCG)6	(CCG)6	(CCG)6	LOC_Os07g40870	CDS	igA FC receptor precursor, putative, expressed	CODON_DELETION	TTCAGGCTTTGGTATCTCCG	AGCCATCCATTCTCCTCCTT	161	P	A	A	NA
PMS4048	Os_Chr07	24517680	24517707	(TC)14	(TC)14	(TC)14	(TC)14	(TC)10	(TC)12	(TC)14	(TC)14	(TC)14	(TC)14	(TC)14	LOC_Os07g40974	URR	NAD dependent epimerase/dehydratase family protein, putative, expressed	-	AAGAGCTTCTTGACGAGGTAGGC	GGAATGGATGGATATGAACAGTGC	184	P	A	A	RM21925
PMS4049	Os_Chr07	24539137	24539170	(CT)17	(CT)18	(CT)17	(CT)18	(CT)17	(CT)17	(CT)17	(CT)15	(CT)17	(CT)17	(CT)17	-	Introns/Intergenic	-	-	CTCTTCTTTCGCCAGCCTCT	ACGACTGCAAAAGGACGG	273	P	A	A	NA
PMS4050	Os_Chr07	24549525	24549550	(CT)13	(CT)14	(CT)13	(CT)10	(CT)13	(CT)13	(CT)13	(CT)10	(CT)13	(CT)13	(CT)13	-	Introns/Intergenic	-	-	CTCTTCTTTCGCCAGCCTCT	ACGACTGCAAAAGGACGG	265	P	A	A	NA
PMS4051	Os_Chr07	24629738	24629755	(TCG)6	(TCG)6	(TCG)6	(TCG)6	(TCG)6	(TCG)6	(TCG)6	(TCG)5	(TCG)6	(TCG)6	(TCG)6	-	Introns/Intergenic	-	-	ACGCAACACACGTTAGGACA	GTGGATTCCGTAGTCGTCGT	255	A	A	A	NA
PMS4052	Os_Chr07	24635626	24635643	(CGC)6	(CGC)6	(CGC)6	(CGC)6	(CGC)6	(CGC)6	(CGC)5	(CGC)6	(CGC)6	(CGC)6	(CGC)6	LOC_Os07g41160	CDS	protein of unknown function DUF1675 domain containing protein, expressed	CODON_DELETION	CTTCAGGCTCTGCATCTCG	GGCGAGGGAGTTCAAGAAG	205	A	A	A	NA
PMS4053	Os_Chr07	24680376	24680387	(TA)6	(TA)6	(TA)6	(TA)6	(TA)6	(TA)7	(TA)6	(TA)6	(TA)6	(TA)6	(TA)6	-	Introns/Intergenic	-	-	GCTAAAGAGCAAGGGTCTCG	TTTTTCTGTTTCCCGTTGCT	188	P	A	A	NA
PMS4054	Os_Chr07	24691262	24691277	(CT)8	(CT)10	(CT)8	(CT)10	(CT)8	(CT)8	(CT)8	(CT)8	(CT)8	(CT)6	(CT)8	-	Introns/Intergenic	-	-	ATTTCCTCCTCAGCCCAACT	CAACGAGAGCAGAGAGGGAG	137	P	A	A	NA
PMS4055	Os_Chr07	24777629	24777694	(TA)33	(TA)33	(TA)33	(TA)11	(TA)16	(TA)33	(TA)8	(TA)33	(TA)33	(TA)17	(TA)33	-	Introns/Intergenic	-	-	TCAATTTCAACAATGGTGCC	GGGCGTAATCTCTCAATTTCA	250	P	A	A	NA

Polymorphic SSR (PMS) markers	Chromosomes	Start physical positions (bp)	End physical positions (bp)	Nipponbare	Kasalath	Nagina22	IR64	Pokkali	Bala	Tainung67	Azucena	Moroberekan	*Oryza rufipogon*	*Oryza nivara*	MSU gene locus IDs	Structural annotation	Functional annotation	SSR functional effects	Forward primers (5'-3')	Reverse primers (5'-3')	Amplified product size (bp)	Markers exhibiting polymorphism within *indica* rice	Transcrption factor genes-derived markers	Known cloned genes-derived markers	PMS markers corresponding with RM markers
PMS4056	Os_Chr07	24788539	24788556	(CGC)6	(CGC)5	(CGC)6	(CGC)6	(CGC)6	(CGC)5	(CGC)6	(CGC)6	(CGC)6	(CGC)6	(CGC)6	LOC_Os07g41370	URR	OsMADS18	-	GAAATCGACAACGAGGCACT	TATCTTGTTCTCGATCCGCC	230	P	P	P	NA
PMS4057	Os_Chr07	24844109	24844126	(CT)9	(CT)9	(CT)9	(CT)9	(CT)9	(CT)9	(CT)9	(CT)9	(CT)8	(CT)9	(CT)9	-	Introns/Intergenic	-	-	GATCCTCCAAAATGGCAAAA	TTTGGTGGGAAAAGATTTGG	240	A	A	A	NA
PMS4058	Os_Chr07	25147875	25147888	(CT)7	(CT)7	(CT)7	(CT)7	(CT)6	(CT)7	(CT)7	(CT)7	(CT)7	(CT)7	(CT)7	-	Introns/Intergenic	-	-	CTCGTCATCTCCTCTCCAGG	TTGTCCCCAAAATTCATCGT	142	P	A	A	NA
PMS4059	Os_Chr07	25363873	25363893	(GCG)7	(GCG)6	(GCG)7	(GCG)7	(GCG)7	(GCG)6	(GCG)7	(GCG)7	(GCG)7	(GCG)6	(GCG)6	LOC_Os07g42390	CDS	DUF581 domain containing protein, expressed	CODON_DELETION	GTCGGCGCAGATGAAGAAGATGC	TGGAGAGAAGGCGTTCTGCAAGG	196	P	A	A	RM21965
PMS4060	Os_Chr07	25372440	25372451	(TG)6	(TG)7	(TG)7	(TG)7	(TG)7	(TG)7	(TG)6	(TG)7	(TG)7	(TG)6	(TG)7	-	Introns/Intergenic	-	-	GATGGAGCAGGAGCTGGA	AACACGACAAATGGAAAGCC	280	A	A	A	NA
PMS4061	Os_Chr07	25402047	25402110	(TA)32	(TA)32	(TA)32	(TA)7	(TA)10	(TA)32	(TA)32	(TA)6	(TA)7	(TA)11	(TA)8	-	Introns/Intergenic	-	-	TGACAGTGACGACGTGGTTT	TTGGTGCTCCATGGTGTCTA	277	P	A	A	NA
PMS4062	Os_Chr07	25403625	25403639	(TGC)5	(TGC)6	(TGC)6	(TGC)6	(TGC)6	(TGC)6	(TGC)5	(TGC)5	(TGC)5	(TGC)5	(TGC)6	-	Introns/Intergenic	-	-	GCATCTATGCAGCTTCACCA	GTCAATTTTTGGCCACTTCG	224	A	A	A	NA
PMS4063	Os_Chr07	25423309	25423329	(CGC)7	(CGC)7	(CGC)7	(CGC)7	(CGC)7	(CGC)7	(CGC)7	(CGC)7	(CGC)5	(CGC)7	(CGC)7	LOC_Os07g42470	CDS	expressed protein	CODON_CHANGE_PLUS_CODON_DELETION	GTCATCGACATCCAGCTTCAAGG	TCGACTCCATTATCGGATGTGC	200	A	A	A	RM21968
PMS4064	Os_Chr07	25472048	25472068	(TCT)7	(TCT)7	(TCT)7	(TCT)7	(TCT)7	(TCT)7	(TCT)7	(TCT)7	(TCT)7	(TCT)7	(TCT)6	-	Introns/Intergenic	-	-	GGGAAGGAGAAAGCCAAAAG	TGCCATTTGGCATATTCAGA	280	A	A	A	NA
PMS4065	Os_Chr07	25489139	25489159	(CGG)7	(CGG)6	(CGG)7	(CGG)7	(CGG)7	(CGG)6	(CGG)7	(CGG)7	(CGG)7	(CGG)7	(CGG)7	LOC_Os07g42590	CDS	OsFBX258 - F-box domain containing protein, expressed	CODON_DELETION	ACCTCTCCATGACGGTGTTC	CCGGAGTTGCTCAGGAAGTA	274	P	A	A	NA
PMS4066	Os_Chr07	25531094	25531111	(GCC)6	(GCC)5	(GCC)6	(GCC)6	(GCC)6	(GCC)5	(GCC)6	(GCC)6	(GCC)6	(GCC)5	(GCC)6	LOC_Os07g42640	URR	FYVE zinc finger family protein, expressed	-	CTCAGATCGGGGAGGAATC	GAGCGAGGGAGAGGGATAAG	242	P	A	A	NA
PMS4067	Os_Chr07	25742002	25742033	(TA)16	(TA)16	(TA)16	(TA)16	(TA)16	(TA)16	(TA)16	(TA)16	(TA)16	(TA)16	(TA)11	-	Introns/Intergenic	-	-	TCCACCACATTGAGCATAGC	GACAGCGACACGGTTTCTAA	280	A	A	A	NA
PMS4068	Os_Chr07	25858339	25858414	(TA)38	(TA)38	(TA)38	(TA)8	(TA)14	(TA)10	(TA)38	(TA)6	(TA)38	(TA)38	(TA)38	-	Introns/Intergenic	-	-	ATGGGTGAGGCTTTAAACACAGG	TAAGCCAGTCCATTGGCAAACC	270	P	A	A	RM21992

Polymorphic SSR (PMS) markers	Chromosomes	Start physical positions (bp)	End physical positions (bp)	Nipponbare	Kasalath	Nagina22	IR64	Pokkali	Bala	Tainung67	Azucena	Moroberekan	*Oryza rufipogon*	*Oryza nivara*	MSU gene locus IDs	Structural annotation	Functional annotation	SSR functional effects	Forward primers (5'-3')	Reverse primers (5'-3')	Amplified product size (bp)	Markers exhibiting polymorphism within *indica* rice	Transcrption factor genes-derived markers	Known cloned genes-derived markers	PMS markers corresponding with RM markers
PMS4069	Os_Chr07	25859988	25860003	(CG)8	(CG)7	(CG)8	(CG)8	(CG)8	(CG)8	(CG)8	(CG)8	(CG)8	(CG)8	(CG)8	-	Introns/Intergenic	-	-	CGATCGATGGAATGTGTGTC	GAGAGCCCAAGCTATACCCC	210	P	A	A	NA
PMS4070	Os_Chr07	25874374	25874401	(TAAT)7	(TAAT)7	(TAAT)7	(TAAT)7	(TAAT)7	(TAAT)7	(TAAT)7	(TAAT)6	(TAAT)7	(TAAT)7	(TAAT)7	-	Introns/Intergenic	-	-	TGTTTTAGCAGCAGTGCAGG	CCCCCGAAGAAAGTATAGCC	210	A	A	A	NA
PMS4071	Os_Chr07	25884657	25884674	(CGG)6	(CGG)6	(CGG)6	(CGG)6	(CGG)6	(CGG)6	(CGG)5	(CGG)6	(CGG)6	(CGG)6	(CGG)6	LOC_Os07g43180	CDS	SKP1-like protein 1B, putative, expressed	CODON_DELETION	GATCCACCTCAAGAGCAAGG	GAGGGTATCGTGGTCGATGT	154	A	A	A	NA
PMS4072	Os_Chr07	25904795	25904816	(GA)11	(GA)11	(GA)11	(GA)11	(GA)11	(GA)7	(GA)11	(GA)11	(GA)11	(GA)11	(GA)11	LOC_Os07g43240	CDS	SKP1-like protein 1B, putative, expressed	FRAME_SHIFT	CGCTAAGGTAATCAGTTTCCGAGAGC	GTCGCCATCACTTGCGAAACC	70	P	A	A	RM21997
PMS4073	Os_Chr07	25967507	25967521	(CCG)5	(CCG)6	(CCG)5	(CCG)5	(CCG)5	(CCG)6	(CCG)5	(CCG)5	(CCG)5	(CCG)5	(CCG)5	LOC_Os07g43370	CDS	amine oxidase family protein, putative, expressed	CODON_INSERTION	CGACAATGACGGCCTTATTAC	ACTCGTCTTCTCCCTCCCTC	223	P	A	A	NA
PMS4074	Os_Chr07	25970126	25970161	(TA)18	(TA)18	(TA)18	(TA)18	(TA)8	(TA)11	(TA)7	(TA)18	(TA)18	(TA)18	(TA)18	-	Introns/Intergenic	-	-	TCGAACATGATCTCACATACCA	TTGGATCTTTACCTCGCACC	200	P	A	A	NA
PMS4075	Os_Chr07	26067960	26067995	(AT)18	(AT)18	(AT)18	(AT)18	(AT)10	(AT)12	(AT)7	(AT)18	(AT)6	(AT)15	(AT)18	-	Introns/Intergenic	-	-	GCGATGCAATGAATCACAAA	TCCTCTTCTCCTCCCCTCTC	265	P	A	A	NA
PMS4076	Os_Chr07	26071191	26071208	(GAG)6	(GAG)6	(GAG)6	(GAG)5	(GAG)5	(GAG)6	(GAG)6	(GAG)6	(GAG)6	(GAG)6	(GAG)6	-	Introns/Intergenic	-	-	ATTTTCAGTACCTGCACGGC	CGTGTATCCTAGTCGCCCAT	161	P	A	A	NA
PMS4077	Os_Chr07	26076023	26076040	(CGG)6	(CGG)6	(CGG)6	(CGG)6	(CGG)6	(CGG)6	(CGG)6	(CGG)6	(CGG)6	(CGG)5	(CGG)6	LOC_Os07g43580	CDS	MYB family transcription factor, putative, expressed	CODON_DELETION	TGGTGGTGGTGTGGATTAGA	AGTACGAGACAAAGCCGACC	167	A	P	A	NA
PMS4078	Os_Chr07	26111773	26111852	(TA)40	(TA)40	(TA)6	(TA)40	(TA)8	(TA)40	(TA)7	(TA)12	(TA)6	(TA)12	(TA)40	-	Introns/Intergenic	-	-	ATGGTGCCCCATATGAGTGT	TCGGGCTCCATAGAAACAAC	266	P	A	A	NA
PMS4079	Os_Chr07	26130979	26130990	(AG)6	(AG)6	(AG)6	(AG)6	(AG)6	(AG)6	(AG)6	(AG)6	(AG)6	(AG)6	(AG)8	-	Introns/Intergenic	-	-	CTGAAGAGGAAGACGGCAAC	AACACACGCACACAGACACA	203	A	A	A	NA
PMS4080	Os_Chr07	26135697	26135720	(TGG)8	(TGG)8	(TGG)8	(TGG)8	(TGG)8	(TGG)8	(TGG)8	(TGG)8	(TGG)8	(TGG)8	(TGG)7	-	Introns/Intergenic	-	-	GTCCCTCCTCCCATCGTC	GGAGCAAGTGGAGCTTCTCA	208	A	A	A	NA
PMS4081	Os_Chr07	26224855	26224875	(GGC)7	(GGC)7	(GGC)7	(GGC)5	(GGC)7	(GGC)7	(GGC)6	(GGC)7	(GGC)7	(GGC)7	(GGC)7	LOC_Os07g43850	CDS	nucleic acid binding protein, putative, expressed	CODON_DELETION	CATTGTTGGTCAGGAGGATCACG	CTTCCAGAACAACCGCCTCTGC	390	P	A	A	RM22013

Polymorphic SSR (PMS) markers	Chromosomes	Start physical positions (bp)	End physical positions (bp)	Nipponbare	Kasalath	Nagina22	IR64	Pokkali	Bala	Tainung67	Azucena	Moroberekan	*Oryza rufipogon*	*Oryza nivara*	MSU gene locus IDs	Structural annotation	Functional annotation	SSR functional effects	Forward primers (5'-3')	Reverse primers (5'-3')	Amplified product size (bp)	Markers exhibiting polymorphism within *indica* rice	Transcrption factor genes-derived markers	Known cloned genes-derived markers	PMS markers corresponding with RM markers
PMS4082	Os_Chr07	26256558	26256578	(GCC)7	(GCC)7	(GCC)7	(GCC)7	(GCC)5	(GCC)7	(GCC)5	(GCC)7	(GCC)5	(GCC)7	(GCC)7	LOC_Os07g43925	CDS	expressed protein	CODON_DELETION	GAACGTGAGGTCACAATTCATCG	AACAACGGCGAGCACTAACACC	254	P	A	A	RM22016
PMS4083	Os_Chr07	26287893	26287912	(TC)10	(TC)10	(TC)10	(TC)10	(TC)7	(TC)10	(TC)10	(TC)10	(TC)10	(TC)10	(TC)10	-	Introns/Intergenic	-	-	CCAGTACTACGCTTTTCCGC	TTCAACGCTCACTCACCTTG	274	P	A	A	NA
PMS4084	Os_Chr07	26326563	26326580	(GGC)6	(GGC)6	(GGC)6	(GGC)6	(GGC)6	(GGC)6	(GGC)6	(GGC)5	(GGC)6	(GGC)6	(GGC)6	LOC_Os07g44050	URR	expressed protein	-	TCATCATCTCCCTCTCCCAC	CATTTTCCTCCACCCACTTC	273	A	A	A	NA
PMS4085	Os_Chr07	26367604	26367621	(TA)9	(TA)9	(TA)9	(TA)7	(TA)9	(TA)9	(TA)9	(TA)9	(TA)9	(TA)9	(TA)9	-	Introns/Intergenic	-	-	CCCTCTACGGTATGCCATGT	AAGCCCTTTGCCAAGTAGGT	278	P	A	A	NA
PMS4086	Os_Chr07	26387656	26387671	(AT)8	(AT)8	(AT)8	(AT)8	(AT)7	(AT)8	(AT)8	(AT)8	(AT)8	(AT)8	(AT)8	-	Introns/Intergenic	-	-	TCTCCGTGACTTCACCGATAAGG	TCACGTCTGCCAAGCAAATGG	187	P	A	A	RM22030
PMS4087	Os_Chr07	26396950	26396967	(AT)9	(AT)9	(AT)8	(AT)9	(AT)8	(AT)9	(AT)9	(AT)8	(AT)9	(AT)8	(AT)9	-	Introns/Intergenic	-	-	TGTCGTGTGTCTAACGTGCAAAGG	GTTGTTAGGCTGCGTTCGTTCTCC	244	P	A	A	RM22032
PMS4088	Os_Chr07	26461965	26462000	(TC)18	(TC)18	(TC)18	(TC)18	(TC)18	(TC)18	(TC)18	(TC)18	(TC)18	(TC)7	(TC)18	-	Introns/Intergenic	-	-	TAATACTATCGCGGTCGGCT	TCGCTCGCGTCTTGTATATG	267	A	A	A	NA
PMS4089	Os_Chr07	26503180	26503195	(TC)8	(TC)8	(TC)8	(TC)8	(TC)8	(TC)8	(TC)8	(TC)8	(TC)8	(TC)7	(TC)8	-	Introns/Intergenic	-	-	GAAGGCTAGCCAAATGGTGA	GAGGGCTGCACTTATTTTGG	109	A	A	A	NA
PMS4090	Os_Chr07	26518229	26518254	(AT)13	(AT)13	(AT)13	(AT)6	(AT)13	(AT)13	(AT)13	(AT)8	(AT)6	(AT)13	(AT)13	-	Introns/Intergenic	-	-	CTGCCTCCGTCTCAAAATAA	AAAATTTGGGCGAATTTGTG	138	P	A	A	NA
PMS4091	Os_Chr07	26518649	26518670	(TA)11	(TA)11	(TA)8	(TA)11	(TA)7	(TA)11	(TA)11	(TA)11	(TA)11	(TA)7	(TA)11	-	Introns/Intergenic	-	-	AAATTTCAATCATGGTGCCC	AGCCACCCTCCCACTTTTAT	140	P	A	A	NA
PMS4092	Os_Chr07	26606724	26606744	(CCG)7	(CCG)7	(CCG)7	(CCG)7	(CCG)7	(CCG)7	(CCG)7	(CCG)7	(CCG)6	(CCG)7	(CCG)7	LOC_Os07g44560	CDS	AMP-binding domain containing protein, expressed	CODON_DELETION	TCCTCGTTGTTCAGGTAGCC	ATCTGCAGCACAACGACAAC	277	A	A	A	NA
PMS4093	Os_Chr07	26673498	26673517	(GA)10	(GA)10	(GA)7	(GA)10	(GA)10	(GA)10	(GA)10	(GA)10	(GA)10	(GA)10	(GA)10	-	Introns/Intergenic	-	-	ACCATAGCACTTTGAACCGC	GAGAGTGGTGGTGGTGTGTG	132	P	A	A	NA
PMS4094	Os_Chr07	26788063	26788080	(CCG)6	(CCG)6	(CCG)6	(CCG)6	(CCG)6	(CCG)6	(CCG)5	(CCG)6	(CCG)6	(CCG)6	(CCG)6	-	Introns/Intergenic	-	-	CGACCATGCAGAGTACCAGA	AAGGGAGAGGGGAGAGGC	246	A	A	A	NA

Polymorphic SSR (PMS) markers	Chromosomes	Start physical positions (bp)	End physical positions (bp)	Nipponbare	Kasalath	Nagina22	IR64	Pokkali	Bala	Tainung67	Azucena	Moroberekan	*Oryza rufipogon*	*Oryza nivara*	MSU gene locus IDs	Structural annotation	Functional annotation	SSR functional effects	Forward primers (5'-3')	Reverse primers (5'-3')	Amplified product size (bp)	Markers exhibiting polymorphism within *indica* rice	Transcrption factor genes-derived markers	Known cloned genes-derived markers	PMS markers corresponding with RM markers
PMS4095	Os_Chr07	26790531	26790546	(CT)8	(CT)8	(CT)8	(CT)8	(CT)8	(CT)8	(CT)8	(CT)8	(CT)7	(CT)8	(CT)8	-	Introns/Intergenic	-	-	AACTTTGCACCGAAAACACA	CATGATGCTGACGGAGACAT	144	A	A	A	NA
PMS4096	Os_Chr07	26822086	26822103	(AT)9	(AT)9	(AT)9	(AT)9	(AT)9	(AT)8	(AT)9	(AT)9	(AT)9	(AT)9	(AT)9	-	Introns/Intergenic	-	-	GAAGCTCCACAGTTTCAGGG	AGATGGAGCTGAGGCACAGT	247	P	A	A	NA
PMS4097	Os_Chr07	27001486	27001503	(GCC)6	(GCC)6	(GCC)6	(GCC)5	(GCC)6	(GCC)6	(GCC)6	(GCC)5	(GCC)6	(GCC)6	(GCC)6	-	Introns/Intergenic	-	-	TGCTGCTTCGCAAGATTAGA	AGCAAGCAAGGGAGATTCAA	240	P	A	A	NA
PMS4098	Os_Chr07	27096644	27096655	(TA)6	(TA)6	(TA)6	(TA)6	(TA)6	(TA)9	(TA)6	(TA)6	(TA)6	(TA)6	(TA)6	-	Introns/Intergenic	-	-	ATTCCATCCAGAAGAGGGCT	ACCTCACGGAAGCTTTACGA	101	P	A	A	NA
PMS4099	Os_Chr07	27127304	27127319	(CT)8	(CT)8	(CT)8	(CT)8	(CT)8	(CT)8	(CT)8	(CT)7	(CT)8	(CT)8	(CT)6	-	Introns/Intergenic	-	-	AGCTCCATGGGGAAATCTCT	TACTCGTCGTTCGCTGACTG	235	A	A	A	NA
PMS4100	Os_Chr07	27227254	27227267	(CG)7	(CG)7	(CG)7	(CG)7	(CG)6	(CG)7	(CG)7	(CG)7	(CG)7	(CG)7	(CG)7	-	Introns/Intergenic	-	-	CAAAACCGCTCTCTCTCTCC	AGATCCCGAGGATCATTTCC	202	P	A	A	NA
PMS4101	Os_Chr07	27497503	27497520	(GCG)6	(GCG)6	(GCG)6	(GCG)6	(GCG)6	(GCG)5	(GCG)6	(GCG)6	(GCG)6	(GCG)6	(GCG)6	LOC_Os07g46070	CDS	expressed protein	CODON_CHANGE_PLUS_CODON_DELETION	GTCTAAAGACCCTCCAGCCC	GTGTGGATGCACAGATTTGG	243	P	A	A	NA
PMS4102	Os_Chr07	27653998	27654018	(CTC)7	(CTC)7	(CTC)5	(CTC)7	(CTC)7	(CTC)7	(CTC)7	(CTC)7	(CTC)7	(CTC)7	(CTC)7	LOC_Os07g46360	CDS	glyoxalase family protein, putative, expressed	CODON_DELETION	GCACGTCCTCGTACAGCTTC	AATAGCAGGTCTCGGCTCTG	183	P	A	A	NA
PMS4103	Os_Chr07	27700273	27700290	(GCG)6	(GCG)6	(GCG)6	(GCG)6	(GCG)6	(GCG)6	(GCG)6	(GCG)5	(GCG)6	(GCG)6	(GCG)6	LOC_Os07g46420	CDS	expressed protein	CODON_DELETION	TTCCTCTCCTCCCTAATCCC	CAGTAGTGCCCGTACCGC	236	A	A	A	NA
PMS4104	Os_Chr07	27746419	27746436	(CGG)6	(CGG)6	(CGG)6	(CGG)6	(CGG)6	(CGG)6	(CGG)6	(CGG)6	(CGG)6	(CGG)5	(CGG)6	LOC_Os07g46480	CDS	eukaryotic aspartyl protease domain containing protein, expressed	CODON_DELETION	TGCTGTGGTGAGAGAGATCG	GAGTCGAGGTACAGCAGCCT	271	A	A	A	NA
PMS4105	Os_Chr07	27845328	27845348	(GCG)7	(GCG)7	(GCG)7	(GCG)5	(GCG)7	(GCG)7	(GCG)7	(GCG)7	(GCG)7	(GCG)7	(GCG)7	LOC_Os07g46600	URR	RNA pseudouridine synthase, putative, expressed	-	GAGAAGAGCAGCTTGGTCTGAGG	TTATCTTCTGAACCCGCCAAACC	359	P	A	A	RM22090
PMS4106	Os_Chr07	27877643	27877656	(AT)7	(AT)6	(AT)6	(AT)6	(AT)7	(AT)6	(AT)7	(AT)6	(AT)6	(AT)7	(AT)7	-	Introns/Intergenic	-	-	TAAATCTATTGCAGCGCACG	AGACTTTCTAGCATCGCCCA	165	P	A	A	NA
PMS4107	Os_Chr07	27893050	27893085	(TA)18	(TA)18	(TA)18	(TA)18	(TA)9	(TA)18	(TA)18	(TA)18	(TA)18	(TA)18	(TA)13	-	Introns/Intergenic	-	-	CAGTGGGATGACTAGAAGGTACGC	GGTTGGGCAGATTCAACTAAGTAGG	328	P	A	A	RM22094

Polymorphic SSR (PMS) markers	Chromosomes	Start physical positions (bp)	End physical positions (bp)	Nipponbare	Kasalath	Nagina22	IR64	Pokkali	Bala	Tainung67	Azucena	Moroberekan	*Oryza rufipogon*	*Oryza nivara*	MSU gene locus IDs	Structural annotation	Functional annotation	SSR functional effects	Forward primers (5'-3')	Reverse primers (5'-3')	Amplified product size (bp)	Markers exhibiting polymorphism within *indica* rice	Transcrption factor genes-derived markers	Known cloned genes-derived markers	PMS markers corresponding with RM markers
PMS4108	Os_Chr07	27907286	27907299	(TA)7	(TA)8	(TA)7	(TA)7	(TA)7	(TA)8	(TA)7	(TA)7	(TA)7	(TA)7	(TA)7	-	Introns/Intergenic	-	-	TTCTAAAGTCCATGCTCGCC	ACTTCCTCCGCTTCACAATG	266	P	A	A	NA
PMS4109	Os_Chr07	27975174	27975237	(TA)32	(TA)32	(TA)12	(TA)32	(TA)32	(TA)32	(TA)32	(TA)32	(TA)32	(TA)32	(TA)32	-	Introns/Intergenic	-	-	GTTGGAGCGATGCTAAATACTGC	TACCGAAGACTCGAAGAATACCG	516	P	A	A	RM22098
PMS4110	Os_Chr07	28024221	28024242	(TA)11	(TA)11	(TA)11	(TA)8	(TA)11	(TA)11	(TA)11	(TA)11	(TA)11	(TA)11	(TA)11	-	Introns/Intergenic	-	-	AGCTCAGTTCGATTCAGCTCTGC	GATACTCCCGGTCCCATATCACC	196	P	A	A	RM22100
PMS4111	Os_Chr07	28173434	28173451	(AGG)6	(AGG)6	(AGG)6	(AGG)7	(AGG)7	(AGG)6	(AGG)6	(AGG)6	(AGG)6	(AGG)6	(AGG)6	LOC_Os07g47110	URR	OsFBT11 - F-box and tubby domain containing protein, expressed	-	ACGAATCCCTTCTTAGCGGT	GGATCACGCTCCTGAAAGAC	226	P	A	A	NA
PMS4112	Os_Chr07	28278295	28278318	(GAC)8	(GAC)8	(GAC)8	(GAC)8	(GAC)8	(GAC)8	(GAC)8	(GAC)8	(GAC)8	(GAC)5	(GAC)8	LOC_Os07g47300	CDS	OsObgC1	CODON_DELETION	GAGGAGTTCGTCGCTACTGC	CTCCTTCGTCTCCTCCTCCT	198	A	A	P	NA
PMS4113	Os_Chr07	28300468	28300494	(GTG)9	(GTG)5	(GTG)9	(GTG)9	(GTG)9	(GTG)9	(GTG)9	(GTG)9	(GTG)9	(GTG)9	(GTG)8	LOC_Os07g47330	CDS	FZP|BFL1	CODON_DELETION	GATCAACCGGGACCATCAC	AACTTCGTCTACACCCACGC	247	P	P	P	NA
PMS4114	Os_Chr07	28647325	28647392	(AT)34	(AT)34	(AT)10	(AT)9	(AT)8	(AT)10	(AT)34	(AT)34	(AT)34	(AT)34	(AT)34	-	Introns/Intergenic	-	-	TGCATGTTGTACGTGTAGTGAACACC	CGCAGAGGACACCGTACACTAGC	237	P	A	A	RM2715
PMS4115	Os_Chr07	28669237	28669258	(TA)11	(TA)11	(TA)6	(TA)6	(TA)11	(TA)11	(TA)11	(TA)11	(TA)11	(TA)11	(TA)11	-	Introns/Intergenic	-	-	GCAGCCGACATATCGAATACTGC	CTGTAATGCACGATAGACGGATTAGC	86	P	A	A	RM22130
PMS4116	Os_Chr07	28693658	28693673	(CA)8	(CA)8	(CA)6	(CA)6	(CA)8	(CA)8	(CA)8	(CA)8	(CA)8	(CA)6	(CA)6	-	Introns/Intergenic	-	-	TGACCCCTGTTTTGGCTAAG	AGTGAGAATGACAGGTGGGG	180	P	A	A	NA
PMS4117	Os_Chr07	28783250	28783273	(TA)12	(TA)12	(TA)9	(TA)6	(TA)12	(TA)12	(TA)12	(TA)12	(TA)8	(TA)12	(TA)12	-	Introns/Intergenic	-	-	TCGTACAAGCTGCCTTTCAC	TTGCTTTTGATGACCAACCA	239	P	A	A	NA
PMS4118	Os_Chr07	28882368	28882382	(CGC)5	(CGC)5	(CGC)5	(CGC)5	(CGC)5	(CGC)5	(CGC)5	(CGC)5	(CGC)5	(CGC)6	(CGC)5	LOC_Os07g48330	CDS	CYP714B1	CODON_INSERTION	CTGAGAAGACAAGGGATCGG	CTTCTTGTCGTCCTTCTCCG	121	A	A	P	NA
PMS4119	Os_Chr07	28889302	28889322	(AGC)7	(AGC)7	(AGC)7	(AGC)7	(AGC)7	(AGC)7	(AGC)7	(AGC)5	(AGC)7	(AGC)7	(AGC)7	LOC_Os07g48340	CDS	OsCML24 - Calmodulin related calcium sensor protein, expressed	CODON_DELETION	GCGCATCTTCCGAAATAGAG	AGCTCGTCCACGGTGATCT	228	A	P	A	NA
PMS4120	Os_Chr07	28889639	28889659	(CGG)7	(CGG)7	(CGG)5	(CGG)7	(CGG)7	(CGG)7	(CGG)7	(CGG)7	(CGG)7	(CGG)7	(CGG)7	LOC_Os07g48340	CDS	OsCML24 - Calmodulin related calcium sensor protein, expressed	CODON_DELETION	AGGACTTCGAGTCCCTCCAC	TTGAACGCCTCCTTCATCTC	115	P	P	A	NA

Polymorphic SSR (PMS) markers	Chromosomes	Start physical positions (bp)	End physical positions (bp)	Nipponbare	Kasalath	Nagina22	IR64	Pokkali	Bala	Tainung67	Azucena	Moroberekan	*Oryza rufipogon*	*Oryza nivara*	MSU gene locus IDs	Structural annotation	Functional annotation	SSR functional effects	Forward primers (5'-3')	Reverse primers (5'-3')	Amplified product size (bp)	Markers exhibiting polymorphism within *indica* rice	Transcrption factor genes-derived markers	Known cloned genes-derived markers	PMS markers corresponding with RM markers
PMS4121	Os_Chr07	28909264	28909281	(GCC)6	(GCC)5	(GCC)6	(GCC)7	(GCC)6	(GCC)6	(GCC)6	(GCC)6	(GCC)6	(GCC)6	(GCC)6	LOC_Os07g48370	CDS	glycosyl transferase, family 8, putative, expressed	CODON_INSERTION	CCATCCATCGATCAGTTGTG	GATTGAGAGCGGTCGAAGAG	273	P	A	A	NA
PMS4122	Os_Chr07	28931512	28931529	(CTG)6	(CTG)6	(CTG)6	(CTG)6	(CTG)6	(CTG)6	(CTG)6	(CTG)6	(CTG)6	(CTG)6	(CTG)5	LOC_Os07g48410	CDS	RNA-binding zinc finger protein, putative, expressed	CODON_DELETION	AGCTCGGAGGTGGAAAAAGT	GGAGCAGCAGTGTCAGGACT	191	A	A	A	NA
PMS4123	Os_Chr07	28977092	28977103	(AC)6	(AC)6	(AC)6	(AC)6	(AC)6	(AC)7	(AC)6	(AC)7	(AC)7	(AC)6	(AC)6	-	Introns/Intergenic	-	-	AGGTTGTTCCTCGTTGATGC	TGAAACAAGTCACGGAGGTG	174	P	A	A	NA
PMS4124	Os_Chr07	29007116	29007135	(GA)10	(GA)10	(GA)10	(GA)10	(GA)10	(GA)10	(GA)10	(GA)9	(GA)10	(GA)10	(GA)10	-	Introns/Intergenic	-	-	ACATGTCCCTCCTCCTTCCT	GTTGCATCCGGTAAGGAAAA	254	A	A	A	NA
PMS4125	Os_Chr07	29008768	29008795	(AG)14	(AG)14	(AG)9	(AG)8	(AG)8	(AG)14	(AG)14	(AG)14	(AG)14	(AG)7	(AG)8	-	Introns/Intergenic	-	-	ACCATGCAGTGCACGAATAATGC	GTTCGTTCGATCGTTGATGTGC	171	P	A	A	RM22150
PMS4126	Os_Chr07	29145354	29145368	(CGG)5	(CGG)5	(CGG)5	(CGG)5	(CGG)7	(CGG)5	(CGG)5	(CGG)5	(CGG)5	(CGG)5	(CGG)5	LOC_Os07g48660	CDS	bZIP transcription factor domain containing protein, expressed	CODON_CHANGE_PLUS_CODON_INSERTION	TAGGGTGAGTGTGTGTGCGT	AGGAGGTCGTCCAGGTTCAT	178	P	P	A	NA
PMS4127	Os_Chr07	29145694	29145711	(CCG)6	(CCG)5	(CCG)6	(CCG)6	(CCG)6	(CCG)5	(CCG)6	(CCG)6	(CCG)6	(CCG)6	(CCG)6	LOC_Os07g48660	CDS	bZIP transcription factor domain containing protein, expressed	CODON_DELETION	CTCGAGGACTTCCTCTCGC	TATACACGCCGTCCACCAC	160	P	P	A	NA
PMS4128	Os_Chr07	29331792	29331803	(TC)6	(TC)7	(TC)6	(TC)6	(TC)6	(TC)6	(TC)6	(TC)6	(TC)6	(TC)6	(TC)6	-	Introns/Intergenic	-	-	CCAAGAATCAATGTAAGCGAGAGC	CAGCAGAACGTCGTCTCTAATCC	182	P	A	A	RM22161
PMS4129	Os_Chr07	29334248	29334267	(AGGC)5	(AGGC)6	(AGGC)6	(AGGC)5	(AGGC)5	(AGGC)6	(AGGC)5	(AGGC)6	(AGGC)5	(AGGC)6	(AGGC)6	-	Introns/Intergenic	-	-	GCTTGTTGTTGCTCCTCCTC	AGCAAACATTGGTGTGGTGA	257	P	A	A	NA
PMS4130	Os_Chr07	29341037	29341066	(TC)15	(TC)10	(TC)15	(TC)15	(TC)15	(TC)15	(TC)15	(TC)15	(TC)15	(TC)15	(TC)15	LOC_Os07g49000	DRR	DNAJ heat shock N-terminal domain-containing protein, putative, expressed	-	AGAGAGCAAGTTTGAAGCGAAGC	ACCAAGAGGGTAGCCTAGCATGG	275	P	A	A	RM248
PMS4131	Os_Chr07	29458565	29458582	(GGA)6	(GGA)6	(GGA)6	(GGA)6	(GGA)6	(GGA)6	(GGA)6	(GGA)6	(GGA)6	(GGA)5	(GGA)6	LOC_Os07g49200	CDS	membrane associated DUF588 domain containing protein, putative, expressed	CODON_DELETION	GGCCTATTAAGTTGGAGCCC	ACCATCCGAATGAGATCAGC	266	A	A	A	NA
PMS4132	Os_Chr07	29488580	29488601	(TC)11	(TC)9	(TC)11	(TC)11	(TC)11	(TC)9	(TC)11	(TC)11	(TC)11	(TC)11	(TC)11	-	Introns/Intergenic	-	-	TTCTCCTTCCCTTCTTCCTTATGC	GTGGAGAAGAGGCAACCAAAGG	85	P	A	A	RM22170
PMS4133	Os_Chr07	29520973	29520990	(CTT)6	(CTT)6	(CTT)6	(CTT)5	(CTT)6	(CTT)6	(CTT)6	(CTT)6	(CTT)6	(CTT)6	(CTT)6	LOC_Os07g49290	CDS	PHD finger family protein, putative, expressed	CODON_DELETION	ACACGTAGTCGTCGTCCTCC	GGCTGCATCAACAACAACAA	280	P	A	A	NA

Polymorphic SSR (PMS) markers	Chromosomes	Start physical positions (bp)	End physical positions (bp)	Nipponbare	Kasalath	Nagina22	IR64	Pokkali	Bala	Tainung67	Azucena	Moroberekan	*Oryza rufipogon*	*Oryza nivara*	MSU gene locus IDs	Structural annotation	Functional annotation	SSR functional effects	Forward primers (5'-3')	Reverse primers (5'-3')	Amplified product size (bp)	Markers exhibiting polymorphism within *indica* rice	Transcrption factor genes-derived markers	Known cloned genes-derived markers	PMS markers corresponding with RM markers
PMS4134	Os_Chr07	29548723	29548752	(TCT)10	(TCT)10	(TCT)10	(TCT)10	(TCT)10	(TCT)10	(TCT)10	(TCT)10	(TCT)10	(TCT)9	(TCT)10	LOC_Os07g49330	URR	phospholipase C, putative, expressed	-	CCTTCCCAAATCAGTTCACAACC	TGTTGTTGGCTTGATGATGAGC	148	A	A	A	RM22175
PMS4135	Os_Chr07	29563870	29563887	(CT)9	(CT)9	(CT)9	(CT)9	(CT)9	(CT)7	(CT)9	(CT)9	(CT)9	(CT)9	(CT)9	-	Introns/Intergenic	-	-	TATCAAATCGTTTCTCCGCC	GCCTCTGTCTGTGGGGTG	271	P	A	A	NA
PMS4136	Os_Chr07	29593568	29593585	(GGA)6	(GGA)6	(GGA)6	(GGA)5	(GGA)6	(GGA)6	(GGA)6	(GGA)6	(GGA)6	(GGA)6	(GGA)6	LOC_Os07g49410	CDS	uncharacterized ACR, YagE family COG1723 containing protein, expressed	CODON_DELETION	GTGGTAGTAGGCGTCGTCGT	ATGCCCCTTGTAGAACCCTC	224	P	A	A	NA
PMS4137	Os_Chr08	78131	78174	(AT)22	(AT)22	(AT)8	(AT)22	(AT)6	(AT)22	(AT)22	(AT)14	(AT)22	(AT)22	(AT)22	-	Introns/Intergenic	-	-	AGTCAACGGCGTCAAACATT	CGCCTTTTCCCTTCTCTCTT	278	P	A	A	NA
PMS4138	Os_Chr08	95846	95897	(CATA)13	(CATA)11	(CATA)13	(CATA)13	(CATA)13	(CATA)8	(CATA)13	(CATA)13	(CATA)13	(CATA)13	(CATA)12	LOC_Os08g01140	DRR	cytochrome b561, putative, expressed	-	GCAGACGGCAAAAACAATTT	ATTCCTCAATTCCATCGTGC	211	P	A	A	NA
PMS4139	Os_Chr08	204915	204930	(TC)8	(TC)7	(TC)8	(TC)8	(TC)7	(TC)8	(TC)8	(TC)8	(TC)8	(TC)8	(TC)7	-	Introns/Intergenic	-	-	GCCCTTGAGGGAAACAAAAC	GGGCTCATTTGCTTTGTAGG	244	P	A	A	NA
PMS4140	Os_Chr08	234872	234891	(AT)10	(AT)10	(AT)10	(AT)10	(AT)10	(AT)10	(AT)10	(AT)10	(AT)6	(AT)10	(AT)10	-	Introns/Intergenic	-	-	ACTCCTGCACCGACCTCAATCG	CAACGGATCTGGATGGACAAGC	495	A	A	A	RM22221
PMS4141	Os_Chr08	239514	239533	(GA)10	(GA)10	(GA)10	(GA)7	(GA)7	(GA)10	(GA)10	(GA)10	(GA)10	(GA)10	(GA)10	-	Introns/Intergenic	-	-	GGAGAGGGAGGGAGAGGC	TTGCTTGTTGTCAGTCTCCG	249	P	A	A	NA
PMS4142	Os_Chr08	262963	262980	(GAA)6	(GAA)5	(GAA)6	(GAA)6	(GAA)6	(GAA)6	(GAA)6	(GAA)6	(GAA)6	(GAA)6	(GAA)6	-	Introns/Intergenic	-	-	CCTCAATCTGCGTTTCAACA	TTTTTGTCCTCCCTCCTCCT	172	P	A	A	NA
PMS4143	Os_Chr08	272079	272096	(TC)9	(TC)6	(TC)9	(TC)9	(TC)9	(TC)9	(TC)9	(TC)9	(TC)9	(TC)9	(TC)9	-	Introns/Intergenic	-	-	GATTCCCTCCCTCTCTTTGC	GTGTGAGCACATGGAACCAG	202	P	A	A	NA
PMS4144	Os_Chr08	379141	379156	(TA)8	(TA)6	(TA)8	(TA)8	(TA)8	(TA)6	(TA)8	(TA)8	(TA)8	(TA)8	(TA)6	-	Introns/Intergenic	-	-	TCGCAAAAATTTTACCCTGAA	GCGAAGATTTTGTTCGCTCT	250	P	A	A	NA
PMS4145	Os_Chr08	505517	505530	(TA)7	(TA)7	(TA)7	(TA)8	(TA)7	(TA)7	(TA)7	(TA)7	(TA)7	(TA)7	(TA)7	-	Introns/Intergenic	-	-	CACAAAACCCATCCGTATCC	GGGTAGATCATTTACGATTGCAT	280	P	A	A	NA
PMS4146	Os_Chr08	510388	510419	(TA)16	(TA)16	(TA)16	(TA)16	(TA)16	(TA)7	(TA)16	(TA)16	(TA)7	(TA)9	(TA)16	-	Introns/Intergenic	-	-	TGGCTGTGTACATTCTAGTTACGC	CCACATTAGCAGGAGCATAGAGG	178	P	A	A	RM22238

Polymorphic SSR (PMS) markers	Chromosomes	Start physical positions (bp)	End physical positions (bp)	Nipponbare	Kasalath	Nagina22	IR64	Pokkali	Bala	Tainung67	Azucena	Moroberekan	*Oryza rufipogon*	*Oryza nivara*	MSU gene locus IDs	Structural annotation	Functional annotation	SSR functional effects	Forward primers (5'-3')	Reverse primers (5'-3')	Amplified product size (bp)	Markers exhibiting polymorphism within *indica* rice	Transcrption factor genes-derived markers	Known cloned genes-derived markers	PMS markers corresponding with RM markers
PMS4147	Os_Chr08	533465	533556	(AT)46	(AT)46	(AT)6	(AT)10	(AT)15	(AT)34	(AT)46	(AT)46	(AT)46	(AT)46	(AT)46	-	Introns/Intergenic	-	-	TGGGTCACTCAGTTAATTCCC	AAATTTCAATCATGGTGCCC	214	P	A	A	NA
PMS4148	Os_Chr08	617393	617408	(TA)8	(TA)8	(TA)8	(TA)8	(TA)8	(TA)8	(TA)7	(TA)7	(TA)8	(TA)8	(TA)8	-	Introns/Intergenic	-	-	TATGCAAGCATGCAACATCA	CCCTCACTTTGGTTATATGGATG	152	A	A	A	NA
PMS4149	Os_Chr08	620239	620259	(GAC)7	(GAC)5	(GAC)7	(GAC)7	(GAC)7	(GAC)5	(GAC)7	(GAC)7	(GAC)7	(GAC)7	(GAC)7	LOC_Os08g01970	CDS	retrotransposon protein, putative, unclassified, expressed	CODON_DELETION	TCTGGATGTAGTCGATGCTGAGG	CAAGAGAGCAGCGTGACATGG	189	P	A	A	RM22245
PMS4150	Os_Chr08	620760	620775	(GA)8	(GA)8	(GA)8	(GA)8	(GA)8	(GA)8	(GA)8	(GA)8	(GA)8	(GA)8	(GA)9	-	Introns/Intergenic	-	-	TTCGTGAGATGATGTGGCAT	CAAATGGGCTCCAACTCACT	273	A	A	A	NA
PMS4151	Os_Chr08	621128	621181	(AT)27	(AT)27	(AT)27	(AT)27	(AT)27	(AT)27	(AT)8	(AT)27	(AT)27	(AT)27	(AT)27	-	Introns/Intergenic	-	-	ACATCAGCACACGAAGTGAGTTGG	AGGAACCAACAATGGGAACAATGG	401	A	A	A	RM22246
PMS4152	Os_Chr08	684049	684078	(CGC)10	(CGC)10	(CGC)10	(CGC)10	(CGC)10	(CGC)10	(CGC)10	(CGC)10	(CGC)10	(CGC)10	(CGC)5	LOC_Os08g02080	URR	rickettsia 17 kDa surface antigen family protein, expressed	-	AAGGAGAAGTTCTTCGCCCAGTGC	GCCCATTAGTGACTGCTCCTAGTCG	197	A	A	A	RM152,OSR34
PMS4153	Os_Chr08	692020	692045	(TA)13	(TA)13	(TA)13	(TA)9	(TA)13	(TA)13	(TA)13	(TA)13	(TA)13	(TA)6	(TA)13	-	Introns/Intergenic	-	-	ATGGGGATCTGCTTACCAAA	TCCATGTGTTGGAGTGTGTG	254	P	A	A	NA
PMS4154	Os_Chr08	701360	701380	(GCG)7	(GCG)7	(GCG)7	(GCG)7	(GCG)5	(GCG)7	(GCG)7	(GCG)7	(GCG)7	(GCG)7	(GCG)7	LOC_Os08g02120	CDS	OsFKII	CODON_DELETION	AATTCTAGCAATGGCTCCTCTCG	GACGAAGTCGATCAGCATCTCC	97	P	A	P	RM22255
PMS4155	Os_Chr08	725958	725981	(TA)12	(TA)12	(TA)12	(TA)8	(TA)12	(TA)12	(TA)12	(TA)12	(TA)6	(TA)12	(TA)12	-	Introns/Intergenic	-	-	CTCGATCGTATGTATAAGGAGAGC	CACGTGTATCCTGATCGAAGG	519	P	A	A	RM22260
PMS4156	Os_Chr08	731491	731522	(AT)16	(AT)16	(AT)16	(AT)16	(AT)16	(AT)16	(AT)16	(AT)16	(AT)6	(AT)12	(AT)16	-	Introns/Intergenic	-	-	GGCCGACGTGAAGAATATTGACC	GTAGCATTAGCCAGCCAGCAAGC	123	A	A	A	RM22261
PMS4157	Os_Chr08	776143	776200	(TA)29	(TA)29	(TA)8	(TA)29	(TA)8	(TA)29	(TA)29	(TA)29	(TA)29	(TA)6	(TA)29	-	Introns/Intergenic	-	-	GACAAAGGGACGTGTCTTGTTGC	CAAGCACGGATAACGAATCACG	322	P	A	A	RM22268
PMS4158	Os_Chr08	795767	795790	(CCG)8	(CCG)8	(CCG)8	(CCG)8	(CCG)8	(CCG)8	(CCG)8	(CCG)5	(CCG)8	(CCG)8	(CCG)8	LOC_Os08g02250	CDS	IQ calmodulin-binding motif domain containing protein, expressed	CODON_DELETION	TGCTCCGACGAGAACCTC	GGTGCCGAACTACATGGC	240	A	A	A	NA
PMS4159	Os_Chr08	859988	860001	(AG)7	(AG)7	(AG)7	(AG)6	(AG)6	(AG)6	(AG)7	(AG)6	(AG)6	(AG)7	(AG)6	-	Introns/Intergenic	-	-	GCCTTTTGGTCCCCAATAAT	GCCAGTAGAGACGCAAGACC	237	P	A	A	NA

Polymorphic SSR (PMS) markers	Chromosomes	Start physical positions (bp)	End physical positions (bp)	Nipponbare	Kasalath	Nagina22	IR64	Pokkali	Bala	Tainung67	Azucena	Moroberekan	*Oryza rufipogon*	*Oryza nivara*	MSU gene locus IDs	Structural annotation	Functional annotation	SSR functional effects	Forward primers (5'-3')	Reverse primers (5'-3')	Amplified product size (bp)	Markers exhibiting polymorphism within *indica* rice	Transcrption factor genes-derived markers	Known cloned genes-derived markers	PMS markers corresponding with RM markers
PMS4160	Os_Chr08	970784	970795	(CT)6	(CT)6	(CT)6	(CT)6	(CT)6	(CT)6	(CT)6	(CT)6	(CT)6	(CT)6	(CT)8	-	Introns/Intergenic	-	-	TCGCTGACATGGCACTTTAC	CTGCAGAAAAGGCAAAGAGG	214	A	A	A	NA
PMS4161	Os_Chr08	983289	983306	(CGC)6	(CGC)6	(CGC)6	(CGC)6	(CGC)6	(CGC)6	(CGC)6	(CGC)6	(CGC)6	(CGC)5	(CGC)6	LOC_Os08g02450	CDS	ATCHX, putative, expressed	CODON_CHANGE_PLUS_CODON_DELETION	CCTCAACTACTCCCCCTGCT	GAGGAAGTAGAGCGCGACG	118	A	A	A	NA
PMS4162	Os_Chr08	1035009	1035028	(TA)10	(TA)10	(TA)10	(TA)10	(TA)10	(TA)10	(TA)10	(TA)6	(TA)10	(TA)10	(TA)10	-	Introns/Intergenic	-	-	GCTTATGCTGTGTTTGGTTTCTGG	CTAGAACTGATGGAGTGCGTTCG	498	A	A	A	RM22288
PMS4163	Os_Chr08	1062616	1062633	(TCG)6	(TCG)6	(TCG)6	(TCG)6	(TCG)6	(TCG)6	(TCG)6	(TCG)6	(TCG)6	(TCG)5	(TCG)6	LOC_Os08g02570	CDS	DUF1517 domain containing protein, putative, expressed	CODON_DELETION	GGGCCCACTGATCTCTCTCT	ATAGGGCAGCTGGTGGTATG	260	A	A	A	NA
PMS4164	Os_Chr08	1135307	1135318	(CA)6	(CA)6	(CA)6	(CA)6	(CA)6	(CA)6	(CA)6	(CA)7	(CA)7	(CA)6	(CA)6	-	Introns/Intergenic	-	-	ATGTTGGGAACCAGGTATGC	GCAAAAACACATGCATGCTAC	108	A	A	A	NA
PMS4165	Os_Chr08	1200006	1200033	(CT)14	(CT)14	(CT)10	(CT)14	(CT)14	(CT)14	(CT)14	(CT)14	(CT)14	(CT)14	(CT)14	LOC_Os08g02850	URR	zinc finger family protein, putative, expressed	-	GCCTACTCAGCTTCCTCTCCTTCG	CGCCATTTACGGCAGCAACC	236	P	A	A	RM3309
PMS4166	Os_Chr08	1433827	1433850	(GCA)8	(GCA)8	(GCA)8	(GCA)8	(GCA)8	(GCA)8	(GCA)8	(GCA)8	(GCA)8	(GCA)8	(GCA)7	LOC_Os08g03130	CDS	retrotransposon protein, putative, Ty3-gypsy subclass, expressed	CODON_CHANGE_PLUS_CODON_DELETION	TCCGCTACACCAATCCCTAC	ATTTATCCGCAAAGTGTCCG	252	A	A	A	NA
PMS4167	Os_Chr08	1462297	1462322	(AT)13	(AT)13	(AT)13	(AT)13	(AT)13	(AT)13	(AT)13	(AT)13	(AT)6	(AT)13	(AT)13	-	Introns/Intergenic	-	-	CAATTTGAATCATGGAGCCC	GCTGTCCAAGTCTCCAAAGC	260	A	A	A	NA
PMS4168	Os_Chr08	1645939	1645956	(CGC)6	(CGC)6	(CGC)6	(CGC)6	(CGC)6	(CGC)6	(CGC)6	(CGC)6	(CGC)6	(CGC)5	(CGC)6	LOC_Os08g03520	URR	OsCSP2	-	GAGGATTGGTGGACGAAGAG	CCACACCGATCTAGGGTTTC	184	A	A	P	NA
PMS4169	Os_Chr08	1648981	1649007	(GCC)9	(GCC)8	(GCC)9	(GCC)9	(GCC)9	(GCC)8	(GCC)9	(GCC)9	(GCC)7	(GCC)9	(GCC)9	LOC_Os08g03540	CDS	expressed protein	CODON_DELETION	CGTCTCAAAGACGAACAAGATGC	CATGAGAGCAACACAGTAGTTTGACC	162	P	A	A	RM22312
PMS4170	Os_Chr08	1652798	1652812	(GCC)5	(GCC)7	(GCC)7	(GCC)7	(GCC)7	(GCC)7	(GCC)5	(GCC)7	(GCC)7	(GCC)5	(GCC)7	-	Introns/Intergenic	-	-	GGCCCAATTGACCAAGTCTA	GGCCAACGGTTTGTGTAGAT	213	A	A	A	NA
PMS4171	Os_Chr08	1700830	1700905	(TA)38	(TA)38	(TA)8	(TA)19	(TA)38	(TA)13	(TA)7	(TA)16	(TA)7	(TA)12	(TA)38	-	Introns/Intergenic	-	-	TGCATCATATCGAGAGCAGTG	TCTTCATCGACGTGTGCATT	225	P	A	A	NA
PMS4172	Os_Chr08	1729771	1729782	(GA)6	(GA)7	(GA)7	(GA)6	(GA)6	(GA)6	(GA)6	(GA)6	(GA)6	(GA)7	(GA)6	LOC_Os08g03650	URR	BTBN17 - Bric-a-Brac, Tramtrack, Broad Complex BTB domain with non-phototropic hypocotyl 3 NPH3 and coiled-coil domains, expressed	-	CAACCCAAGAAACCAGCAAT	ATGGGAAACTGTGATGAGGC	201	P	A	A	NA

Polymorphic SSR (PMS) markers	Chromosomes	Start physical positions (bp)	End physical positions (bp)	Nipponbare	Kasalath	Nagina22	IR64	Pokkali	Bala	Tainung67	Azucena	Moroberekan	*Oryza rufipogon*	*Oryza nivara*	MSU gene locus IDs	Structural annotation	Functional annotation	SSR functional effects	Forward primers (5'-3')	Reverse primers (5'-3')	Amplified product size (bp)	Markers exhibiting polymorphism within *indica* rice	Transcrption factor genes-derived markers	Known cloned genes-derived markers	PMS markers corresponding with RM markers
PMS4173	Os_Chr08	2016882	2016909	(TC)14	(TC)14	(TC)14	(TC)14	(TC)14	(TC)11	(TC)14	(TC)14	(TC)14	(TC)6	(TC)14	-	Introns/Intergenic	-	-	TTCGGGACAGCAGCAAGTTACC	GCCAAACACTGCCTTAGCATCC	389	P	A	A	RM22328
PMS4174	Os_Chr08	2140420	2140434	(GGC)5	(GGC)6	(GGC)5	(GGC)5	(GGC)5	(GGC)5	(GGC)5	(GGC)5	(GGC)5	(GGC)6	(GGC)5	LOC_Os08g04370	CDS	plastocyanin-like domain containing protein, putative, expressed	CODON_INSERTION	CAAGCTCCATCGATCTCCTC	CGAGTACCTGAACACGAGCC	235	P	A	A	NA
PMS4175	Os_Chr08	2169999	2170040	(AT)21	(AT)16	(AT)9	(AT)9	(AT)9	(AT)21	(AT)21	(AT)21	(AT)21	(AT)9	(AT)21	-	Introns/Intergenic	-	-	AATTAGCTTGCAGCGGAATCACATGC	GACGGAGGGAGTAGCGATTTGTCC	165	P	A	A	RM8018
PMS4176	Os_Chr08	2213190	2213201	(AC)6	(AC)6	(AC)8	(AC)8	(AC)8	(AC)8	(AC)6	(AC)6	(AC)6	(AC)6	(AC)6	-	Introns/Intergenic	-	-	TTTGACCAAACTCAAAACGACTT	CGGGTTCATGAGGAAGAAGA	207	P	A	A	NA
PMS4177	Os_Chr08	2228708	2228729	(GA)11	(GA)6	(GA)11	(GA)11	(GA)11	(GA)11	(GA)11	(GA)11	(GA)11	(GA)11	(GA)11	-	Introns/Intergenic	-	-	GAAAGGAGGGCTTGGCTC	TCGAGTTCAATTCCGAATCC	280	P	A	A	NA
PMS4178	Os_Chr08	2324940	2324957	(CT)9	(CT)9	(CT)9	(CT)9	(CT)9	(CT)9	(CT)9	(CT)9	(CT)9	(CT)9	(CT)6	-	Introns/Intergenic	-	-	AATCCAATTCCTTCCAAATC	AAAACGCGCCGGGGAAAG	153	A	A	A	NA
PMS4179	Os_Chr08	2363378	2363389	(CG)6	(CG)7	(CG)6	(CG)6	(CG)6	(CG)6	(CG)6	(CG)6	(CG)6	(CG)6	(CG)6	-	Introns/Intergenic	-	-	GAAAAGGAAGGAGGGAGGAA	GTCCACCGGCTCTCTCTCT	146	P	A	A	NA
PMS4180	Os_Chr08	2442584	2442597	(GA)7	(GA)7	(GA)7	(GA)7	(GA)7	(GA)7	(GA)6	(GA)7	(GA)7	(GA)7	(GA)7	-	Introns/Intergenic	-	-	TACAACGACCATTGGCAGAA	TCTACAGCTAGCGGTTAGCAA	267	A	A	A	NA
PMS4181	Os_Chr08	2449743	2449763	(GGC)7	(GGC)7	(GGC)7	(GGC)7	(GGC)7	(GGC)6	(GGC)7	(GGC)7	(GGC)7	(GGC)7	(GGC)6	LOC_Os08g04850	CDS	transposon protein, putative, CACTA, En/Spm sub-class, expressed	CODON_DELETION	GTTCTCGTTCTCGTTCGTTA	TCCGATCTCGATCTCTCTC	356	P	A	A	NA
PMS4182	Os_Chr08	2488001	2488030	(AT)15	(AT)15	(AT)15	(AT)8	(AT)15	(AT)15	(AT)15	(AT)15	(AT)15	(AT)15	(AT)15	-	Introns/Intergenic	-	-	GCTCCAATTCCTTCTAGGGC	CGTGAATGAGAACAAGCGAA	216	P	A	A	NA
PMS4183	Os_Chr08	2488307	2488324	(GGC)6	(GGC)6	(GGC)6	(GGC)6	(GGC)6	(GGC)6	(GGC)5	(GGC)6	(GGC)6	(GGC)6	(GGC)6	LOC_Os08g04870	CDS	transposon protein, putative, CACTA, En/Spm sub-class, expressed	CODON_DELETION	AGAGAGATCGATATCGGAGAC	CACTGCCTAGGGCAAATC	163	A	A	A	NA
PMS4184	Os_Chr08	2642477	2642494	(GGC)6	(GGC)5	(GGC)6	(GGC)6	(GGC)6	(GGC)6	(GGC)6	(GGC)6	(GGC)6	(GGC)6	(GGC)6	-	Introns/Intergenic	-	-	CTCCACCAGCTTCTCAGCTC	ATCTAGGAGGGAAGGGGAGG	229	P	A	A	NA
PMS4185	Os_Chr08	2892904	2892936	(AAG)11	(AAG)11	(AAG)11	(AAG)11	(AAG)11	(AAG)10	(AAG)11	(AAG)11	(AAG)11	(AAG)11	(AAG)11	-	Introns/Intergenic	-	-	GTTCCATCCAGACATTGTAGAAGC	TAAACTTGGTCGTGGACAGTGC	182	P	A	A	RM5647

Polymorphic SSR (PMS) markers	Chromosomes	Start physical positions (bp)	End physical positions (bp)	Nipponbare	Kasalath	Nagina22	IR64	Pokkali	Bala	Tainung67	Azucena	Moroberekan	*Oryza rufipogon*	*Oryza nivara*	MSU gene locus IDs	Structural annotation	Functional annotation	SSR functional effects	Forward primers (5'-3')	Reverse primers (5'-3')	Amplified product size (bp)	Markers exhibiting polymorphism within *indica* rice	Transcrption factor genes-derived markers	Known cloned genes-derived markers	PMS markers corresponding with RM markers
PMS4186	Os_Chr08	2924331	2924357	(TCC)9	(TCC)9	(TCC)7	(TCC)9	(TCC)9	(TCC)9	(TCC)9	(TCC)9	(TCC)9	(TCC)9	(TCC)9	LOC_Os08g05490	CDS	tRNA synthetase, putative, expressed	CODON_DELETION	ACTTATCAGCCGCCTCCTCTCG	ATGAGCTCGTCCTCCTGGATGC	246	P	A	A	RM22383
PMS4187	Os_Chr08	3016139	3016178	(GA)20	(GA)18	(GA)20	(GA)20	(GA)20	(GA)20	(GA)20	(GA)20	(GA)20	(GA)20	(GA)20	-	Introns/Intergenic	-	-	GAACAGAGGCGAGCGTTGTGG	AAGGCAACACCAAGAAAGCATCC	84	P	A	A	RM3819
PMS4188	Os_Chr08	3091419	3091472	(TA)27	(TA)24	(TA)27	(TA)6	(TA)27	(TA)27	(TA)27	(TA)21	(TA)27	(TA)18	(TA)27	-	Introns/Intergenic	-	-	TCATTAAAAACATGTACTCCCTCTG	GGCACGGTCTCCAAAACATA	271	P	A	A	NA
PMS4189	Os_Chr08	3145745	3145759	(GAG)5	(GAG)9	(GAG)5	(GAG)5	(GAG)5	(GAG)5	(GAG)5	(GAG)5	(GAG)5	(GAG)5	(GAG)5	-	Introns/Intergenic	-	-	AAGAGGTGGTTTACGGGGAC	GAAGGGGTATGAGAGGGGAG	179	P	A	A	NA
PMS4190	Os_Chr08	3164527	3164544	(TC)9	(TC)10	(TC)9	(TC)9	(TC)9	(TC)9	(TC)9	(TC)9	(TC)9	(TC)9	(TC)9	-	Introns/Intergenic	-	-	AGAAGCTGAGGTTGGAACGA	TGAAGCTTACTCGACTGCGA	162	P	A	A	NA
PMS4191	Os_Chr08	3180655	3180724	(AT)35	(AT)8	(AT)35	(AT)8	(AT)8	(AT)11	(AT)35	(AT)35	(AT)35	(AT)8	(AT)9	-	Introns/Intergenic	-	-	TGTGGCCAGTTGAACATCATACC	GTCGAAACAAAGGAGGTGGTTCC	328	P	A	A	RM2819
PMS4192	Os_Chr08	3211268	3211287	(AT)10	(AT)10	(AT)10	(AT)10	(AT)10	(AT)10	(AT)10	(AT)10	(AT)10	(AT)9	(AT)10	-	Introns/Intergenic	-	-	GCCTTTAGATTCTCTGTGATGC	CAATGCTACTAGATGGTCAGTCC	442	A	A	A	RM22411
PMS4193	Os_Chr08	3268954	3268983	(AT)15	(AT)15	(AT)15	(AT)11	(AT)15	(AT)15	(AT)15	(AT)15	(AT)11	(AT)15	(AT)15	-	Introns/Intergenic	-	-	CTTTCTAAGATTACCTACATTCA	TTCTTATTTGTTGAACACCAG	143	P	A	A	NA
PMS4194	Os_Chr08	3318580	3318597	(GCA)6	(GCA)6	(GCA)6	(GCA)6	(GCA)6	(GCA)6	(GCA)5	(GCA)6	(GCA)6	(GCA)5	(GCA)6	-	Introns/Intergenic	-	-	CATCCTAGGGTTTCCCCAAA	CGAGCTTCCTCTCCTCCTCT	187	A	A	A	NA
PMS4195	Os_Chr08	3379017	3379042	(GT)13	(GT)6	(GT)13	(GT)13	(GT)13	(GT)12	(GT)13	(GT)13	(GT)13	(GT)13	(GT)13	-	Introns/Intergenic	-	-	ATTTCGTCGTCGTGTCTGTCC	TTAAAGCAGCCTGATCGAAAGC	461	P	A	A	RM22426
PMS4196	Os_Chr08	3386783	3386806	(GAG)8	(GAG)8	(GAG)8	(GAG)8	(GAG)8	(GAG)8	(GAG)8	(GAG)8	(GAG)8	(GAG)8	(GAG)6	-	Introns/Intergenic	-	-	GGCATGAGAGACAATCAAGAAGAGG	CCCTCTCCAAGATGGTAACAACC	76	A	A	A	RM22427
PMS4197	Os_Chr08	3405062	3405073	(AT)6	(AT)9	(AT)6	(AT)6	(AT)6	(AT)6	(AT)6	(AT)6	(AT)6	(AT)6	(AT)6	-	Introns/Intergenic	-	-	TCTTATCCCTTCCCTTCGCT	GGCCCAATCCCTACATCAGT	232	P	A	A	NA
PMS4198	Os_Chr08	3429506	3429521	(AT)8	(AT)8	(AT)7	(AT)8	(AT)8	(AT)8	(AT)8	(AT)8	(AT)8	(AT)8	(AT)8	-	Introns/Intergenic	-	-	TCCGAGATTCGTCCATGTAA	TCGAGGTATGCATAAGAAACGA	213	P	A	A	NA

Polymorphic SSR (PMS) markers	Chromosomes	Start physical positions (bp)	End physical positions (bp)	Nipponbare	Kasalath	Nagina22	IR64	Pokkali	Bala	Tainung67	Azucena	Moroberekan	*Oryza rufipogon*	*Oryza nivara*	MSU gene locus IDs	Structural annotation	Functional annotation	SSR functional effects	Forward primers (5'-3')	Reverse primers (5'-3')	Amplified product size (bp)	Markers exhibiting polymorphism within *indica* rice	Transcrption factor genes-derived markers	Known cloned genes-derived markers	PMS markers corresponding with RM markers
PMS4199	Os_Chr08	3500888	3500971	(TA)42	(TA)42	(TA)42	(TA)13	(TA)10	(TA)31	(TA)42	(TA)42	(TA)8	(TA)38	(TA)42	-	Introns/Intergenic	-	-	CGCGTGTGGAATGTGTATTG	TGATTTTGTGCATTTGAAACC	209	P	A	A	NA
PMS4200	Os_Chr08	3601629	3601662	(AG)17	(AG)17	(AG)16	(AG)17	(AG)16	(AG)16	(AG)17	(AG)17	(AG)17	(AG)17	(AG)17	LOC_Os08g06420	URR	abhydrolase domain-containing protein FAM108C1, putative, expressed	-	CAGCTAGACCTCACACTCTCACAGC	GCACTGCACGCTCTCTCACG	173	P	A	A	RM22446
PMS4201	Os_Chr08	3662813	3662826	(CT)7	(CT)7	(CT)7	(CT)7	(CT)7	(CT)7	(CT)7	(CT)6	(CT)7	(CT)7	(CT)7	-	Introns/Intergenic	-	-	CACAATCGTAGGTCCCTCGT	TGTCACACTGGGGGATGTTA	169	A	A	A	NA
PMS4202	Os_Chr08	3782193	3782224	(TA)16	(TA)16	(TA)16	(TA)12	(TA)12	(TA)16	(TA)16	(TA)16	(TA)16	(TA)16	(TA)11	-	Introns/Intergenic	-	-	TTTTACGCACGTATACAAAGTT	CACATTATCATCCTCTCTTTTT	208	P	A	A	NA
PMS4203	Os_Chr08	3839229	3839252	(CT)12	(CT)12	(CT)10	(CT)8	(CT)8	(CT)12	(CT)12	(CT)12	(CT)12	(CT)12	(CT)12	LOC_Os08g06880	CDS	expressed protein	FRAME_SHIFT	AGTTTTGTTGGAGCCTTGGA	CAGGTAGGAGCATGAGAGCC	175	P	A	A	NA
PMS4204	Os_Chr08	4086267	4086282	(TA)8	(TA)8	(TA)8	(TA)8	(TA)8	(TA)8	(TA)8	(TA)6	(TA)8	(TA)8	(TA)8	-	Introns/Intergenic	-	-	TATTTATTTTGGCTTCGCGG	TTTGGAAATAAGCACAGCCC	197	A	A	A	NA
PMS4205	Os_Chr08	4178261	4178281	(CCT)7	(CCT)7	(CCT)7	(CCT)7	(CCT)7	(CCT)7	(CCT)5	(CCT)7	(CCT)7	(CCT)7	(CCT)7	LOC_Os08g07440	CDS	AP2 domain containing protein, expressed	CODON_DELETION	TCCAAAAATTCCAAACCCAA	ATGATGGGAAAGCGGCTG	204	A	A	A	NA
PMS4206	Os_Chr08	4180036	4180056	(CGC)7	(CGC)7	(CGC)7	(CGC)7	(CGC)7	(CGC)7	(CGC)5	(CGC)7	(CGC)7	(CGC)5	(CGC)7	LOC_Os08g07450	CDS	glutaredoxin protein, putative, expressed	CODON_DELETION	GACGTCGAGGGCTTCTTCATCG	CATGTCTCCGTCGACTTCGACTTCC	429	A	A	A	RM22468
PMS4207	Os_Chr08	4248999	4249024	(AT)13	(AT)13	(AT)13	(AT)13	(AT)13	(AT)8	(AT)13	(AT)13	(AT)10	(AT)13	(AT)13	-	Introns/Intergenic	-	-	CCACGCTCCGTCACACATA	AGAAGCAACTTCAGGACGGA	242	P	A	A	NA
PMS4208	Os_Chr08	4259686	4259719	(AT)17	(AT)17	(AT)7	(AT)17	(AT)17	(AT)17	(AT)17	(AT)17	(AT)17	(AT)17	(AT)17	-	Introns/Intergenic	-	-	ATTGGGTGAGACTGGGACTG	GGAGAAATTCATGTTACTATGCAGC	242	P	A	A	NA
PMS4209	Os_Chr08	4333911	4333928	(CGC)6	(CGC)6	(CGC)6	(CGC)6	(CGC)6	(CGC)6	(CGC)6	(CGC)6	(CGC)6	(CGC)8	(CGC)6	LOC_Os08g07740	CDS	Hd5|DTH8|Ghd8|OsHAP3H|LHD1|EF8	CODON_INSERTION	AGTCTAAACCATTGCACGCC	AGGGTGATGGAGTTCGAGG	110	A	A	P	NA
PMS4210	Os_Chr08	4450307	4450332	(TA)13	(TA)13	(TA)13	(TA)13	(TA)13	(TA)13	(TA)13	(TA)10	(TA)13	(TA)13	(TA)13	-	Introns/Intergenic	-	-	ACTAGCATGGTGGCTCACG	TTGAAAGACGAGTCTAGAATCCATA	277	A	A	A	NA
PMS4211	Os_Chr08	4473240	4473261	(AG)11	(AG)7	(AG)11	(AG)11	(AG)11	(AG)11	(AG)11	(AG)11	(AG)11	(AG)11	(AG)11	-	Introns/Intergenic	-	-	GGTGAAGAGGAAGAGACAGACG	CTTTCAGCCCATCTATTGTAGCC	134	P	A	A	RM22490

Polymorphic SSR (PMS) markers	Chromosomes	Start physical positions (bp)	End physical positions (bp)	Nipponbare	Kasalath	Nagina22	IR64	Pokkali	Bala	Tainung67	Azucena	Moroberekan	*Oryza rufipogon*	*Oryza nivara*	MSU gene locus IDs	Structural annotation	Functional annotation	SSR functional effects	Forward primers (5'-3')	Reverse primers (5'-3')	Amplified product size (bp)	Markers exhibiting polymorphism within *indica* rice	Transcrption factor genes-derived markers	Known cloned genes-derived markers	PMS markers corresponding with RM markers
PMS4212	Os_Chr08	4506492	4506509	(CGG)6	(CGG)7	(CGG)6	(CGG)6	(CGG)6	(CGG)7	(CGG)6	(CGG)6	(CGG)6	(CGG)6	(CGG)6	LOC_Os08g07970	CDS	transcription factor, putative, expressed	CODON_INSERTION	GGAGATGGGAGGTAGGTGGT	CATGGACAAGCTGAAGCAGA	245	P	P	A	NA
PMS4213	Os_Chr08	4523010	4523053	(AT)22	(AT)13	(AT)22	(AT)22	(AT)8	(AT)22	(AT)22	(AT)22	(AT)10	(AT)22	(AT)22	-	Introns/Intergenic	-	-	GCTTCCGTTCTGTTTGCTTT	TTGCACAAACTTAGGTGACCA	175	P	A	A	NA
PMS4214	Os_Chr08	4586220	4586237	(TA)9	(TA)9	(TA)9	(TA)9	(TA)7	(TA)9	(TA)9	(TA)8	(TA)9	(TA)9	(TA)9	-	Introns/Intergenic	-	-	ACCGCCCCAAAATAATCTCT	TTCATTGAAAATTTGTAGTATGTGTTT	276	P	A	A	NA
PMS4215	Os_Chr08	4594517	4594534	(TA)9	(TA)9	(TA)9	(TA)9	(TA)9	(TA)9	(TA)6	(TA)9	(TA)9	(TA)9	(TA)9	-	Introns/Intergenic	-	-	AAATGAGAATGGTGGAACGG	TCTCTTCGTTTCATAATGTAAGTCA	222	A	A	A	NA
PMS4216	Os_Chr08	4680800	4680817	(TTC)6	(TTC)6	(TTC)6	(TTC)6	(TTC)6	(TTC)5	(TTC)6	(TTC)6	(TTC)6	(TTC)6	(TTC)6	-	Introns/Intergenic	-	-	TCCCAGTTTCCCACTAGCAA	CTCCTCCTGTTGGTGTGGAT	251	P	A	A	NA
PMS4217	Os_Chr08	4773794	4773817	(CT)12	(CT)12	(CT)12	(CT)12	(CT)12	(CT)12	(CT)12	(CT)10	(CT)12	(CT)12	(CT)10	-	Introns/Intergenic	-	-	CCTATACCAAACCGAACCGAACC	CGCTGCCAATATCACCAAAGG	209	A	A	A	RM1111
PMS4218	Os_Chr08	4809448	4809461	(GA)7	(GA)7	(GA)7	(GA)6	(GA)7	(GA)7	(GA)7	(GA)7	(GA)7	(GA)7	(GA)7	-	Introns/Intergenic	-	-	ACTCCCTTCCCTCCTCCTCT	CTCTCTCTCGGCTTCTCCCT	241	P	A	A	NA
PMS4219	Os_Chr08	4893887	4893910	(TA)12	(TA)12	(TA)12	(TA)12	(TA)9	(TA)12	(TA)12	(TA)12	(TA)12	(TA)12	(TA)12	-	Introns/Intergenic	-	-	CTACTAGCACCGACGGTTTGTGG	TGCTCATGTGGCGTAATACAACC	308	P	A	A	RM22514
PMS4220	Os_Chr08	5049606	5049620	(CCG)5	(CCG)6	(CCG)5	(CCG)5	(CCG)5	(CCG)5	(CCG)5	(CCG)5	(CCG)6	(CCG)5	(CCG)5	-	Introns/Intergenic	-	-	AATAGGAAATGTTGCCGGTG	AGATTCGATGCAAGAGGCAG	274	P	A	A	NA
PMS4221	Os_Chr08	5102760	5102827	(TA)34	(TA)34	(TA)34	(TA)6	(TA)34	(TA)34	(TA)9	(TA)6	(TA)34	(TA)34	(TA)34	-	Introns/Intergenic	-	-	TCAATTTCAACAATGGTGCC	AACGACGGTGACAACAATCA	220	P	A	A	NA
PMS4222	Os_Chr08	5112954	5112965	(GT)6	(GT)6	(GT)7	(GT)6	(GT)6	(GT)7	(GT)6	(GT)7	(GT)7	(GT)6	(GT)6	-	Introns/Intergenic	-	-	TCCCTCTGGCCTCTACTCCT	CCTCTCGTGCTGGTTCTGA	250	P	A	A	NA
PMS4223	Os_Chr08	5130578	5130593	(AT)8	(AT)8	(AT)8	(AT)8	(AT)8	(AT)8	(AT)8	(AT)8	(AT)7	(AT)9	(AT)8	-	Introns/Intergenic	-	-	CGCTTTAAACAATGACACGC	CGTTTTGGACAAGGTTGAGA	221	A	A	A	NA
PMS4224	Os_Chr08	5136726	5136737	(TA)6	(TA)6	(TA)9	(TA)6	(TA)6	(TA)9	(TA)6	(TA)6	(TA)6	(TA)6	(TA)6	-	Introns/Intergenic	-	-	CATTGACGCCGTTGATTTTT	CCGCTACTCGTCGAAAAGAC	237	P	A	A	NA

Polymorphic SSR (PMS) markers	Chromosomes	Start physical positions (bp)	End physical positions (bp)	Nipponbare	Kasalath	Nagina22	IR64	Pokkali	Bala	Tainung67	Azucena	Moroberekan	*Oryza rufipogon*	*Oryza nivara*	MSU gene locus IDs	Structural annotation	Functional annotation	SSR functional effects	Forward primers (5'-3')	Reverse primers (5'-3')	Amplified product size (bp)	Markers exhibiting polymorphism within *indica* rice	Transcrption factor genes-derived markers	Known cloned genes-derived markers	PMS markers corresponding with RM markers
PMS4225	Os_Chr08	5173175	5173190	(AG)8	(AG)8	(AG)8	(AG)8	(AG)8	(AG)8	(AG)8	(AG)8	(AG)9	(AG)8	(AG)8	-	Introns/Intergenic	-	-	AGCGACGGTAAAAC CATGAC	TCATGTGCTCCTCTA ACCCC	247	A	A	A	NA
PMS4226	Os_Chr08	5192176	5192205	(AT)15	(AT)14	(AT)15	(AT)15	(AT)15	(AT)14	(AT)15	(AT)15	(AT)15	(AT)15	(AT)15	-	Introns/Intergenic	-	-	CTCCCTCCTCTTTG GGTTGTGG	CTCCACCGGCACGG TATAATGG	205	P	A	A	RM22531
PMS4227	Os_Chr08	5226900	5226921	(TA)11	(TA)11	(TA)11	(TA)11	(TA)9	(TA)6	(TA)11	(TA)11	(TA)11	(TA)11	(TA)9	-	Introns/Intergenic	-	-	GTCGGTTGATTAAC TCGCAAGC	ACATTGATTGGTCGG TCATTGC	428	P	A	A	RM22539
PMS4228	Os_Chr08	5229010	5229021	(TA)6	(TA)6	(TA)6	(TA)6	(TA)6	(TA)7	(TA)6	(TA)6	(TA)6	(TA)6	(TA)6	-	Introns/Intergenic	-	-	GGCCATTGCTTTTG ATCCTA	TCTTTTAGTGCCATG ACGCA	195	P	A	A	NA
PMS4229	Os_Chr08	5452741	5452761	(TCC)7	(TCC)7	(TCC)7	(TCC)7	(TCC)7	(TCC)5	(TCC)7	(TCC)7	(TCC)7	(TCC)7	(TCC)6	LOC_Os08g09410	CDS	OsFBX265 - F-box domain containing protein, expressed	CODON_DELETION	CTTCGATCTCCTCG TCCTCTTCC	GAGCATGAGATGAT GCATGACG	136	P	A	A	RM22551
PMS4230	Os_Chr08	5456939	5456958	(TA)10	(TA)10	(TA)10	(TA)10	(TA)10	(TA)10	(TA)10	(TA)9	(TA)10	(TA)10	(TA)9	-	Introns/Intergenic	-	-	CAGCAAATCACCGA AGACACTCG	GGGAACAGGATTGC AACAGAGG	435	A	A	A	RM22552
PMS4231	Os_Chr08	5604513	5604554	(AT)21	(AT)21	(AT)21	(AT)21	(AT)21	(AT)20	(AT)21	(AT)7	(AT)12	(AT)21	(AT)21	-	Introns/Intergenic	-	-	TTGGTTGCAAGATG CTCTACACC	AGATGGATGAACGG ATTGAAGC	691	P	A	A	RM22556
PMS4232	Os_Chr08	5657558	5657579	(CT)11	(CT)6	(CT)11	(CT)11	(CT)11	(CT)11	(CT)11	(CT)11	(CT)11	(CT)11	(CT)11	-	Introns/Intergenic	-	-	TGCTACTTGTCTTG CCGTTG	CATTGCGTGCTGTTC ATCTT	255	P	A	A	NA
PMS4233	Os_Chr08	5690511	5690528	(CT)9	(CT)9	(CT)9	(CT)9	(CT)9	(CT)9	(CT)8	(CT)9	(CT)9	(CT)9	(CT)9	-	Introns/Intergenic	-	-	GTCCACCTCCTCCT CCCTC	CCACGAAAATGGCTT GAGTT	107	A	A	A	NA
PMS4234	Os_Chr08	5702746	5702778	(CAG)11	(CAG)6	(CAG)11	(CAG)11	(CAG)11	(CAG)11	(CAG)11	(CAG)6	(CAG)11	(CAG)5	(CAG)10	LOC_Os08g09880	CDS	transposon protein, putative, unclassified, expressed	CODON_DELETION	CAGTTCGTGCAATA TTCCCTTGG	CAAACGTCTGGGTCA AGTGATAGC	262	P	A	A	RM22559
PMS4235	Os_Chr08	5719139	5719156	(GGC)6	(GGC)6	(GGC)5	(GGC)6	(GGC)6	(GGC)6	(GGC)6	(GGC)6	(GGC)6	(GGC)6	(GGC)6	LOC_Os08g09900	URR	WRKY118, expressed	-	GAGCTTGGAAGATG CTGACC	CATAGTGGCCTCGAA TGCTT	233	P	P	A	NA
PMS4236	Os_Chr08	5789076	5789099	(CGG)8	(CGG)5	(CGG)8	(CGG)8	(CGG)8	(CGG)8	(CGG)8	(CGG)8	(CGG)8	(CGG)8	(CGG)8	LOC_Os08g10010	URR	acyl-desaturase, chloroplast precursor, putative, expressed	-	GATCGGAGGAGGG AGGAAGACG	GTCGCATCCACTCAT ATTCCAAGC	87	P	A	A	RM6208
PMS4237	Os_Chr08	5804339	5804356	(TC)9	(TC)9	(TC)9	(TC)9	(TC)9	(TC)12	(TC)9	(TC)9	(TC)9	(TC)9	(TC)7	-	Introns/Intergenic	-	-	TGCTGCACAAGGAT AGAGGA	TGGTTTACACAAACC CGTGA	154	P	A	A	NA

Polymorphic SSR (PMS) markers	Chromosomes	Start physical positions (bp)	End physical positions (bp)	Nipponbare	Kasalath	Nagina22	IR64	Pokkali	Bala	Tainung67	Azucena	Moroberekan	*Oryza rufipogon*	*Oryza nivara*	MSU gene locus IDs	Structural annotation	Functional annotation	SSR functional effects	Forward primers (5'-3')	Reverse primers (5'-3')	Amplified product size (bp)	Markers exhibiting polymorphism within *indica* rice	Transcrption factor genes-derived markers	Known cloned genes-derived markers	PMS markers corresponding with RM markers
PMS4238	Os_Chr08	5840198	5840219	(AG)11	(AG)10	(AG)11	(AG)11	(AG)11	(AG)12	(AG)11	(AG)11	(AG)11	(AG)11	(AG)11	-	Introns/Intergenic	-	-	GAACTACGGATTACAACTGCTGATGG	GCGGATGCCAAGAAATAGAAACC	391	P	A	A	RM22569
PMS4239	Os_Chr08	5847159	5847194	(ATCAAT)6	(ATCAAT)6	(ATCAAT)6	(ATCAAT)5	(ATCAAT)6	(ATCAAT)6	(ATCAAT)6	(ATCAAT)6	(ATCAAT)5	(ATCAAT)6	(ATCAAT)6	LOC_Os08g10080	DRR	OMTN6	-	TGCACAGCTACTACTCCAGCA	CAGAGAAGAAGGCCATCAGG	271	P	P	P	NA
PMS4240	Os_Chr08	5874032	5874071	(TA)20	(TA)20	(TA)20	(TA)20	(TA)20	(TA)20	(TA)20	(TA)20	(TA)6	(TA)20	(TA)15	-	Introns/Intergenic	-	-	CAGAGGAGGAGAAACTCAAAGTGC	TATTAACGAGTGCGTCGGTTACG	186	A	A	A	RM22574
PMS4241	Os_Chr08	5973698	5973763	(ATA)22	(ATA)22	(ATA)22	(ATA)22	(ATA)17	(ATA)22	(ATA)22	(ATA)22	(ATA)5	(ATA)22	(ATA)13	-	Introns/Intergenic	-	-	GCACACATGTAACGGAGGTG	AACTGAAGGGAGTCTCTTTTCCT	200	P	A	A	NA
PMS4242	Os_Chr08	5989486	5989499	(TG)7	(TG)7	(TG)7	(TG)7	(TG)7	(TG)6	(TG)7	(TG)7	(TG)7	(TG)7	(TG)7	-	Introns/Intergenic	-	-	CCCAACCCTCTGATGGAATA	TGAAATTCTGCAGCGAAATG	249	P	A	A	NA
PMS4243	Os_Chr08	6027245	6027320	(AT)38	(AT)38	(AT)38	(AT)38	(AT)6	(AT)9	(AT)38	(AT)38	(AT)9	(AT)38	(AT)6	-	Introns/Intergenic	-	-	ATTAGATACCATGGTGCCCG	TCTCCAAGCCAAATGGAAAG	224	P	A	A	NA
PMS4244	Os_Chr08	6202915	6202940	(TA)13	(TA)13	(TA)13	(TA)13	(TA)13	(TA)13	(TA)13	(TA)13	(TA)13	(TA)11	(TA)13	-	Introns/Intergenic	-	-	TCGGATCTACAACCCGATTA	TCCATTGTGGAGGAAAAACA	257	A	A	A	NA
PMS4245	Os_Chr08	6205970	6205997	(CT)14	(CT)14	(CT)14	(CT)14	(CT)14	(CT)14	(CT)14	(CT)14	(CT)14	(CT)15	(CT)14	LOC_Os08g10550	URR	potassium transporter, putative, expressed	-	TGCTCCTCCACCTACTACCATCC	GCAGAAATCTTGACAGAAGAGAGTGG	177	A	A	A	RM3644
PMS4246	Os_Chr08	6289517	6289530	(GA)7	(GA)7	(GA)7	(GA)7	(GA)6	(GA)7	(GA)7	(GA)7	(GA)7	(GA)7	(GA)7	-	Introns/Intergenic	-	-	CAGTGGGATCCACATGTCAC	CCTCAACTTGTCTCGGGTGT	261	P	A	A	NA
PMS4247	Os_Chr08	6316716	6316735	(TA)10	(TA)10	(TA)10	(TA)10	(TA)10	(TA)10	(TA)10	(TA)9	(TA)10	(TA)10	(TA)10	-	Introns/Intergenic	-	-	GATCAGCCAAGCTGTAACAAACC	TTGTTCTGTACACTTCCCGTTGC	264	A	A	A	RM22595
PMS4248	Os_Chr08	6324603	6324680	(TA)39	(TA)39	(TA)39	(TA)39	(TA)23	(TA)39	(TA)8	(TA)39	(TA)39	(TA)39	(TA)39	-	Introns/Intergenic	-	-	GCCTAAAGGTCATTAATGCGATGG	AGGGCCTGTGTGCATAGGATACG	174	P	A	A	RM22598
PMS4249	Os_Chr08	6325065	6325118	(AT)27	(AT)27	(AT)25	(AT)25	(AT)22	(AT)27	(AT)7	(AT)27	(AT)27	(AT)27	(AT)27	-	Introns/Intergenic	-	-	GCTTGTGTGCATAGGATATGTTGC	CATTTAGGGCCTCAGTCAGTTCG	110	P	A	A	RM22599
PMS4250	Os_Chr08	6472880	6472897	(GTC)6	(GTC)6	(GTC)6	(GTC)6	(GTC)6	(GTC)6	(GTC)6	(GTC)6	(GTC)6	(GTC)6	(GTC)5	-	Introns/Intergenic	-	-	GTCGTTGTCTCCGTTCGTTC	TGCTGCACACTTGACGAAAC	265	A	A	A	NA

Polymorphic SSR (PMS) markers	Chromosomes	Start physical positions (bp)	End physical positions (bp)	Nipponbare	Kasalath	Nagina22	IR64	Pokkali	Bala	Tainung67	Azucena	Moroberekan	*Oryza rufipogon*	*Oryza nivara*	MSU gene locus IDs	Structural annotation	Functional annotation	SSR functional effects	Forward primers (5'-3')	Reverse primers (5'-3')	Amplified product size (bp)	Markers exhibiting polymorphism within *indica* rice	Transcrption factor genes-derived markers	Known cloned genes-derived markers	PMS markers corresponding with RM markers
PMS4251	Os_Chr08	6667976	6668015	(AT)20	(AT)20	(AT)10	(AT)20	(AT)9	(AT)20	(AT)20	(AT)20	(AT)20	(AT)20	(AT)20	-	Introns/Intergenic	-	-	ACTGTGTCAGGTGCCGTTTAAGG	AATGGCTGGGATTGGGATGG	376	P	A	A	RM22608
PMS4252	Os_Chr08	6681669	6681686	(CGC)6	(CGC)6	(CGC)6	(CGC)6	(CGC)6	(CGC)6	(CGC)5	(CGC)6	(CGC)6	(CGC)6	(CGC)6	-	Introns/Intergenic	-	-	CTCCCCCTCTCCCTCTTG	GAGGGAATCACGGGGAATA	276	A	A	A	NA
PMS4253	Os_Chr08	6690420	6690431	(GC)6	(GC)6	(GC)6	(GC)6	(GC)6	(GC)6	(GC)6	(GC)6	(GC)7	(GC)6	(GC)6	-	Introns/Intergenic	-	-	TATAGACTTTTCCTCGGGCG	ATCCGTTCCTCCGCAGAC	272	A	A	A	NA
PMS4254	Os_Chr08	6712230	6712243	(AG)7	(AG)6	(AG)7	(AG)7	(AG)7	(AG)7	(AG)7	(AG)7	(AG)7	(AG)7	(AG)7	-	Introns/Intergenic	-	-	CTGGGGGAGGAAGACGAC	TCCCTCCTCCCTCTCTTTTT	232	P	A	A	NA
PMS4255	Os_Chr08	6722350	6722431	(TA)41	(TA)41	(TA)41	(TA)17	(TA)37	(TA)7	(TA)41	(TA)41	(TA)41	(TA)41	(TA)41	-	Introns/Intergenic	-	-	ATGGTGCCCCATATGAGTGT	TTGTTTGATGGGCTGTTTGA	169	P	A	A	NA
PMS4256	Os_Chr08	6843856	6843877	(TA)11	(TA)11	(TA)11	(TA)11	(TA)11	(TA)11	(TA)11	(TA)7	(TA)11	(TA)11	(TA)11	LOC_Os08g11669	URR	expressed protein	-	GCGAGCACCTAAGCATCTAGC	TTCCCAACCAGGAGAGTAATAGG	189	A	A	A	RM22621
PMS4257	Os_Chr08	7047515	7047528	(CG)7	(CG)7	(CG)7	(CG)7	(CG)7	(CG)7	(CG)7	(CG)6	(CG)7	(CG)7	(CG)7	-	Introns/Intergenic	-	-	AAAACCGCCTCCTCTCTCTC	TAGCCGAAGATTCCAAAGGA	267	A	A	A	NA
PMS4258	Os_Chr08	7126597	7126608	(GA)6	(GA)8	(GA)6	(GA)6	(GA)6	(GA)8	(GA)6	(GA)6	(GA)6	(GA)6	(GA)6	-	Introns/Intergenic	-	-	GAGGAGGAGAGGGAGAGGAA	CCTTGGTCTAAAAGGAGCCC	211	P	A	A	NA
PMS4259	Os_Chr08	7145146	7145166	(GAT)7	(GAT)7	(GAT)7	(GAT)7	(GAT)7	(GAT)5	(GAT)7	(GAT)7	(GAT)7	(GAT)7	(GAT)7	LOC_Os08g12130	CDS	retrotransposon protein, putative, Ty3-gypsy subclass, expressed	CODON_DELETION	GGATATGATTGCTCCGATTAGGG	TCTCACCCATTCGGCTACTGC	208	P	A	A	RM22624
PMS4260	Os_Chr08	7327066	7327089	(TA)12	(TA)12	(TA)12	(TA)6	(TA)8	(TA)12	(TA)12	(TA)7	(TA)12	(TA)12	(TA)12	-	Introns/Intergenic	-	-	TCGGGCGGTATAAAAATCTG	AAAAACAAGAATCGGGGGAC	264	P	A	A	NA
PMS4261	Os_Chr08	7399853	7399870	(AT)9	(AT)9	(AT)9	(AT)9	(AT)9	(AT)9	(AT)9	(AT)9	(AT)9	(AT)9	(AT)7	-	Introns/Intergenic	-	-	CTCAAGGTTCCTCTGTTCCG	GCTCCAATTCCTTCTAGGGC	278	A	A	A	NA
PMS4262	Os_Chr08	7488658	7488678	(CGG)7	(CGG)7	(CGG)7	(CGG)7	(CGG)7	(CGG)7	(CGG)7	(CGG)7	(CGG)5	(CGG)7	(CGG)7	LOC_Os08g12680	CDS	zinc finger domain, LSD1 subclass family protein, expressed	CODON_DELETION	CCCATCTAGAATCCCAGATTTCC	AAGCTGAGGATGAGGCTGTGG	297	A	A	A	RM22638
PMS4263	Os_Chr08	7605862	7605881	(ATTT)5	(ATTT)6	(ATTT)5	(ATTT)5	(ATTT)6	(ATTT)5	(ATTT)5	(ATTT)5	(ATTT)5	(ATTT)5	(ATTT)5	-	Introns/Intergenic	-	-	ATTGCTCGAACGAGGGAGAATCC	AAGGCGCGAGATTCAATGAGAGG	94	P	A	A	RM22646

Polymorphic SSR (PMS) markers	Chromosomes	Start physical positions (bp)	End physical positions (bp)	Nipponbare	Kasalath	Nagina22	IR64	Pokkali	Bala	Tainung67	Azucena	Moroberekan	*Oryza rufipogon*	*Oryza nivara*	MSU gene locus IDs	Structural annotation	Functional annotation	SSR functional effects	Forward primers (5'-3')	Reverse primers (5'-3')	Amplified product size (bp)	Markers exhibiting polymorphism within *indica* rice	Transcrption factor genes-derived markers	Known cloned genes-derived markers	PMS markers corresponding with RM markers
PMS4264	Os_Chr08	7614580	7614599	(GA)10	(GA)10	(GA)10	(GA)6	(GA)10	(GA)10	(GA)10	(GA)10	(GA)10	(GA)10	(GA)10	-	Introns/Intergenic	-	-	CCTCAGTTAATGGCGAACACAGG	CATCGACTCTCTTTGCAGTTTGG	359	P	A	A	RM22647
PMS4265	Os_Chr08	7637366	7637427	(AT)31	(AT)31	(AT)9	(AT)31	(AT)31	(AT)31	(AT)31	(AT)7	(AT)31	(AT)31	(AT)31	-	Introns/Intergenic	-	-	CATGCCTGAGCTCAAACTGA	TCAATTTCAACAATGGTGCC	230	P	A	A	NA
PMS4266	Os_Chr08	7762160	7762180	(CGG)7	(CGG)7	(CGG)7	(CGG)5	(CGG)7	(CGG)7	(CGG)7	(CGG)7	(CGG)7	(CGG)7	(CGG)7	LOC_Os08g13070	URR	MBTB23 - Bric-a-Brac, Tramtrack, Broad Complex BTB domain with Meprin and TRAF Homology MATH domain, expressed	-	GAAGGAAGGGCGGGTTAGTACGG	CACCGCGGATATTACCACCTTCC	190	P	A	A	RM22654
PMS4267	Os_Chr08	7939721	7939741	(CTG)7	(CTG)7	(CTG)7	(CTG)7	(CTG)7	(CTG)7	(CTG)7	(CTG)7	(CTG)7	(CTG)7	(CTG)5	LOC_Os08g13350	URR	expressed protein	-	CACTGCTGCCAACATCCATTCC	AAGGAAGAAGTGAAGTGGCATACGG	202	A	A	A	RM22664
PMS4268	Os_Chr08	7951410	7951424	(CGC)5	(CGC)5	(CGC)5	(CGC)5	(CGC)5	(CGC)5	(CGC)5	(CGC)5	(CGC)6	(CGC)5	(CGC)5	LOC_Os08g13360	CDS	kelch repeat protein, putative, expressed	CODON_INSERTION	GACCTCGGCGTAGATCCTC	ACCAGGTGCTCTCCATCCT	212	A	A	A	NA
PMS4269	Os_Chr08	7973938	7973953	(AG)8	(AG)8	(AG)8	(AG)8	(AG)8	(AG)8	(AG)8	(AG)8	(AG)8	(AG)8	(AG)9	-	Introns/Intergenic	-	-	TAGCGGAACTACCCACTTGC	ACCAAGATCAGCAGAGCAGG	222	A	A	A	NA
PMS4270	Os_Chr08	7975402	7975419	(ACG)6	(ACG)6	(ACG)5	(ACG)6	(ACG)6	(ACG)6	(ACG)6	(ACG)6	(ACG)6	(ACG)6	(ACG)6	-	Introns/Intergenic	-	-	ACGACGACCATGGGAAGAC	CTGGAGCAGTTCGTTTGTGA	279	P	A	A	NA
PMS4271	Os_Chr08	8016904	8016919	(AT)8	(AT)7	(AT)8	(AT)8	(AT)6	(AT)8	(AT)8	(AT)8	(AT)7	(AT)8	(AT)8	-	Introns/Intergenic	-	-	AACGAAGGTAACTTTGGCATGT	ATTTGATACCATGGTGCCCG	243	P	A	A	NA
PMS4272	Os_Chr08	8017312	8017329	(TA)9	(TA)9	(TA)9	(TA)7	(TA)9	(TA)6	(TA)9	(TA)9	(TA)7	(TA)9	(TA)9	-	Introns/Intergenic	-	-	CCATGGAGCACCAAACAAAT	CACCGTTGTGTTCGTAATAATTAAA	280	P	A	A	NA
PMS4273	Os_Chr08	8042666	8042683	(TC)9	(TC)9	(TC)9	(TC)9	(TC)9	(TC)9	(TC)9	(TC)9	(TC)9	(TC)9	(TC)6	-	Introns/Intergenic	-	-	GCTCTCATCGTCTCGCTTTT	AACTAGAGGAGGGGAGGACG	273	A	A	A	NA
PMS4274	Os_Chr08	8135554	8135565	(AT)6	(AT)6	(AT)6	(AT)7	(AT)6	(AT)6	(AT)6	(AT)6	(AT)6	(AT)6	(AT)6	-	Introns/Intergenic	-	-	GCATTTCAAAATCCCACACA	TTCCTTTTTGAGGCATTGCT	266	P	A	A	NA
PMS4275	Os_Chr08	8137203	8137232	(AT)15	(AT)15	(AT)15	(AT)15	(AT)15	(AT)15	(AT)6	(AT)15	(AT)13	(AT)15	(AT)15	-	Introns/Intergenic	-	-	TGCAGGATTGTTGATGAACTCG	CGTCGAATCAAGTTGTGTTGC	334	A	A	A	RM22665
PMS4276	Os_Chr08	8207070	8207087	(CT)9	(CT)9	(CT)9	(CT)9	(CT)9	(CT)9	(CT)9	(CT)9	(CT)9	(CT)10	(CT)9	-	Introns/Intergenic	-	-	ACGACGGTTTTGATCCTACG	TTGAAGGCTGATGCTGTACG	280	A	A	A	NA

**Polymorphic SSR (PMS) markers**	**Chromosomes**	**Start physical positions (bp)**	**End physical positions (bp)**	**Nipponbare**	**Kasalath**	**Nagina22**	**IR64**	**Pokkali**	**Bala**	**Tainung67**	**Azucena**	**Moroberekan**	***Oryza rufipogon***	***Oryza nivara***	**MSU gene locus IDs**	**Structural annotation**	**Functional annotation**	**SSR functional effects**	**Forward primers (5'-3')**	**Reverse primers (5'-3')**	**Amplified product size (bp)**	**Markers exhibiting polymorphism within *indica* rice**	**Transcrption factor genes-derived markers**	**Known cloned genes-derived markers**	**PMS markers corresponding with RM markers**
PMS4277	Os_Chr08	8451186	8451219	(TG)17	(TG)17	(TG)17	(TG)17	(TG)17	(TG)17	(TG)17	(TG)17	(TG)17	(TG)9	(TG)17	-	Introns/Intergenic	-	-	GTCAGGGTGGCTCTTACAGAAGTCC	TATGCCCTAGCACCGACGATCC	449	A	A	A	RM22671
PMS4278	Os_Chr08	8464719	8464732	(AT)7	(AT)7	(AT)7	(AT)7	(AT)7	(AT)7	(AT)6	(AT)7	(AT)7	(AT)7	(AT)7	-	Introns/Intergenic	-	-	GGGCCTAGCCCAATTATTCT	CGATGGAGTGAGCACGTAGA	255	A	A	A	NA
PMS4279	Os_Chr08	8497626	8497669	(AC)22	(AC)17	(AC)22	(AC)22	(AC)22	(AC)22	(AC)22	(AC)22	(AC)22	(AC)22	(AC)22	-	Introns/Intergenic	-	-	TCAATCAATGGCATGTCAGACC	GTGTTACAGTCTGCATTAACCACACC	121	P	A	A	RM22674
PMS4280	Os_Chr08	8594384	8594403	(CT)10	(CT)6	(CT)10	(CT)10	(CT)10	(CT)10	(CT)10	(CT)10	(CT)10	(CT)10	(CT)10	LOC_Os08g14330	URR	aldose 1-epimerase, putative, expressed	-	CCCTAACCCTAACCCTAGCC	GAAAAGATCCTCAAAGCCCC	193	P	A	A	NA
PMS4281	Os_Chr08	8652543	8652620	(TA)39	(TA)39	(TA)13	(TA)39	(TA)39	(TA)39	(TA)39	(TA)15	(TA)39	(TA)39	(TA)39	-	Introns/Intergenic	-	-	TCAATTTCAACAATGGTGCC	CAGCGAAAACCTAGCTCCAC	277	P	A	A	NA
PMS4282	Os_Chr08	8678468	8678482	(GAC)5	(GAC)5	(GAC)5	(GAC)6	(GAC)5	(GAC)5	(GAC)5	(GAC)5	(GAC)5	(GAC)5	(GAC)5	LOC_Os08g14450	CDS	OsSIG6	CODON_INSERTION	TTTCGTTCTCGACAGTGGTG	AGATGGCTCGTCAGCAAGAT	246	P	A	P	NA
PMS4283	Os_Chr08	8722853	8722868	(TA)8	(TA)8	(TA)8	(TA)7	(TA)8	(TA)8	(TA)8	(TA)8	(TA)8	(TA)8	(TA)8	-	Introns/Intergenic	-	-	CGGTGCATGGTCGTATGTAG	AATGACTCGAGAGCGCTTGT	102	P	A	A	NA
PMS4284	Os_Chr08	8875843	8875858	(GT)8	(GT)8	(GT)8	(GT)8	(GT)8	(GT)7	(GT)8	(GT)8	(GT)8	(GT)8	(GT)8	-	Introns/Intergenic	-	-	TCCAATTTCAACCAAAATCCA	TGCCATCATGTTTGTGTGTG	228	P	A	A	NA
PMS4285	Os_Chr08	8936459	8936480	(TG)11	(TG)11	(TG)11	(TG)11	(TG)8	(TG)11	(TG)11	(TG)11	(TG)11	(TG)11	(TG)11	-	Introns/Intergenic	-	-	TCTACCTTAGCTGTCCTGAATTGG	CACATACCTGTTCCGTTTGATCC	148	P	A	A	RM8243
PMS4286	Os_Chr08	8950126	8950137	(TG)6	(TG)6	(TG)6	(TG)6	(TG)6	(TG)7	(TG)6	(TG)6	(TG)6	(TG)6	(TG)6	-	Introns/Intergenic	-	-	TCCATAGTGACATTGGTGGG	TCTTAGCGCGGAATTAATGG	226	P	A	A	NA
PMS4287	Os_Chr08	9111541	9111564	(CGG)8	(CGG)8	(CGG)8	(CGG)8	(CGG)8	(CGG)8	(CGG)8	(CGG)5	(CGG)8	(CGG)8	(CGG)8	LOC_Os08g15070	CDS	GDU1, putative, expressed	CODON_DELETION	AAGAACGTGGGCATCCTCTCC	TGCTCCTACTGGAAGCTCAACAGC	194	A	A	A	RM6027
PMS4288	Os_Chr08	9123441	9123468	(CT)14	(CT)14	(CT)14	(CT)14	(CT)14	(CT)9	(CT)14	(CT)6	(CT)9	(CT)14	(CT)14	-	Introns/Intergenic	-	-	GGCCTGAAATCAGCTGAAAG	AGAGAGATTGGGTTTTGGGG	152	P	A	A	NA
PMS4289	Os_Chr08	9182603	9182632	(AT)15	(AT)15	(AT)15	(AT)15	(AT)15	(AT)15	(AT)15	(AT)15	(AT)6	(AT)15	(AT)9	-	Introns/Intergenic	-	-	CTTCAAACAGCTTCTCGGACTGG	TATGCCACGGTCGAAAGAATAGG	368	A	A	A	RM22702

Polymorphic SSR (PMS) markers	Chromosomes	Start physical positions (bp)	End physical positions (bp)	Nipponbare	Kasalath	Nagina22	IR64	Pokkali	Bala	Tainung67	Azucena	Moroberekan	*Oryza rufipogon*	*Oryza nivara*	MSU gene locus IDs	Structural annotation	Functional annotation	SSR functional effects	Forward primers (5'-3')	Reverse primers (5'-3')	Amplified product size (bp)	Markers exhibiting polymorphism within *indica* rice	Transcrption factor genes-derived markers	Known cloned genes-derived markers	PMS markers corresponding with RM markers
PMS4290	Os_Chr08	9188936	9188949	(AG)7	(AG)7	(AG)7	(AG)7	(AG)7	(AG)6	(AG)7	(AG)7	(AG)7	(AG)7	(AG)7	-	Introns/Intergenic	-	-	CAACCGTATACCGG CAAGAT	ACGTTGTCCTGTTCT CCACC	235	P	A	A	NA
PMS4291	Os_Chr08	9378328	9378343	(CT)8	(CT)8	(CT)8	(CT)8	(CT)8	(CT)8	(CT)8	(CT)8	(CT)8	(CT)7	(CT)7	-	Introns/Intergenic	-	-	TGGAAAATCGGTTT CCGTAG	CTGTAGAGGTTCGCT ACCGC	250	A	A	A	NA
PMS4292	Os_Chr08	9416363	9416390	(GA)14	(GA)14	(GA)14	(GA)14	(GA)9	(GA)14	(GA)14	(GA)9	(GA)14	(GA)9	(GA)14	-	Introns/Intergenic	-	-	AAAGAGACACGGGA TCGACTGC	ATAGTGTGCACCTGT CCCATACTCC	221	P	A	A	RM22716
PMS4293	Os_Chr08	9484813	9484830	(CGG)6	(CGG)6	(CGG)6	(CGG)6	(CGG)5	(CGG)6	(CGG)6	(CGG)6	(CGG)6	(CGG)6	(CGG)6	LOC_Os08g15610	CDS	expressed protein	CODON_DELETION	CACTGACCAACGCA TGTACC	ATGCATTTTACTGCA GCCCT	112	P	A	A	NA
PMS4294	Os_Chr08	9535640	9535679	(TA)20	(TA)20	(TA)20	(TA)20	(TA)7	(TA)20	(TA)20	(TA)20	(TA)6	(TA)20	(TA)20	-	Introns/Intergenic	-	-	ACTGCGTTGCGTAG TTTAGAGC	AAACAGCTGTAGCGA GAGATAGC	331	P	A	A	RM22720
PMS4295	Os_Chr08	9835362	9835379	(GCG)6	(GCG)6	(GCG)6	(GCG)6	(GCG)5	(GCG)6	(GCG)6	(GCG)6	(GCG)6	(GCG)6	(GCG)6	-	Introns/Intergenic	-	-	GGTTTTGTTCTACG TGGCGT	AAAGTGAGCTCCTCA CGTCG	172	P	A	A	NA
PMS4296	Os_Chr08	9958810	9958833	(ATAG)6	(ATAG)6	(ATAG)6	(ATAG)6	(ATAG)6	(ATAG)6	(ATAG)6	(ATAG)5	(ATAG)6	(ATAG)6	(ATAG)6	-	Introns/Intergenic	-	-	ACGTTCGGCTGAAA GCATTCTCG	GGCATATGCATTCCG TAGCTTTGC	181	A	A	A	RM22741
PMS4297	Os_Chr08	9960768	9960791	(AT)12	(AT)12	(AT)7	(AT)7	(AT)12	(AT)12	(AT)12	(AT)12	(AT)6	(AT)12	(AT)12	-	Introns/Intergenic	-	-	TGCAAACCGGAACC AAGAATCC	TTTGACCGTGGTTTG ACTGACG	278	P	A	A	RM22742
PMS4298	Os_Chr08	9962282	9962307	(TA)13	(TA)13	(TA)13	(TA)8	(TA)13	(TA)13	(TA)13	(TA)13	(TA)13	(TA)13	(TA)13	-	Introns/Intergenic	-	-	AGAGAGAGATCGG GAGGAGG	GGGGGACCTGGGAG TATAAA	129	P	A	A	NA
PMS4299	Os_Chr08	9984156	9984179	(ATAG)6	(ATAG)6	(ATAG)6	(ATAG)6	(ATAG)5	(ATAG)6	(ATAG)6	(ATAG)6	(ATAG)6	(ATAG)6	(ATAG)5	-	Introns/Intergenic	-	-	GGCAGCTTGAGAG CGTTTAT	AAAGATGGGCAGGA TGTACG	170	P	A	A	NA
PMS4300	Os_Chr08	10044597	10044622	(TA)13	(TA)13	(TA)13	(TA)13	(TA)10	(TA)13	(TA)13	(TA)13	(TA)13	(TA)13	(TA)13	-	Introns/Intergenic	-	-	CGAGTACGCGTTAT GGTAATTGG	AAACTTCAAGACATG TGGCGTACC	165	P	A	A	RM22753
PMS4301	Os_Chr08	10065042	10065053	(AC)6	(AC)6	(AC)6	(AC)6	(AC)8	(AC)6	(AC)6	(AC)6	(AC)6	(AC)6	(AC)6	-	Introns/Intergenic	-	-	TGATCGAGAATTGC ATTTGG	TGTGGACAATGCTAG AAAGTCTTA	183	P	A	A	NA
PMS4302	Os_Chr08	10097707	10097752	(AT)23	(AT)23	(AT)23	(AT)23	(AT)23	(AT)23	(AT)23	(AT)23	(AT)6	(AT)23	(AT)23	-	Introns/Intergenic	-	-	CCACATCGCAGACT ATGAATGC	AGATACGGATAGGC GGATACAGG	115	A	A	A	RM22755

Polymorphic SSR (PMS) markers	Chromosomes	Start physical positions (bp)	End physical positions (bp)	Nipponbare	Kasalath	Nagina22	IR64	Pokkali	Bala	Tainung67	Azucena	Moroberekan	*Oryza rufipogon*	*Oryza nivara*	MSU gene locus IDs	Structural annotation	Functional annotation	SSR functional effects	Forward primers (5'-3')	Reverse primers (5'-3')	Amplified product size (bp)	Markers exhibiting polymorphism within *indica* rice	Transcrption factor genes-derived markers	Known cloned genes-derived markers	PMS markers corresponding with RM markers
PMS4303	Os_Chr08	10187857	10187888	(ATAG)8	(ATAG)8	(ATAG)8	(ATAG)8	(ATAG)8	(ATAG)8	(ATAG)8	(ATAG)7	(ATAG)8	(ATAG)8	(ATAG)8	-	Introns/Intergenic	-	-	GGCAGCTTGAGAGCGTTTAT	TGTGCTCTATAGCTGGGGCT	231	A	A	A	NA
PMS4304	Os_Chr08	10287964	10287981	(AT)9	(AT)9	(AT)9	(AT)9	(AT)9	(AT)9	(AT)6	(AT)9	(AT)9	(AT)9	(AT)9	-	Introns/Intergenic	-	-	CGTGTGTGGAGGCTCTTCTT	AGCTACTCCCTCCGTTTCAT	208	A	A	A	NA
PMS4305	Os_Chr08	10302306	10302350	(TAT)15	(TAT)15	(TAT)12	(TAT)9	(TAT)15	(TAT)15	(TAT)15	(TAT)8	(TAT)6	(TAT)15	(TAT)15	-	Introns/Intergenic	-	-	GAGGAGTTCGGCCGTATACTAGAGC	GCCACATTAACGCTATGTAGGATGC	659	P	A	A	RM22761
PMS4306	Os_Chr08	10363799	10363819	(GAG)7	(GAG)7	(GAG)7	(GAG)7	(GAG)7	(GAG)5	(GAG)7	(GAG)7	(GAG)7	(GAG)7	(GAG)7	LOC_Os08g16914	CDS	expressed protein	CODON_DELETION	AACCGGCGCAAGGTTTAAAGG	ATGTCTTCTCCTTCTCCTCAAGATGG	269	P	A	A	RM22765
PMS4307	Os_Chr08	10367061	10367072	(CT)6	(CT)6	(CT)6	(CT)6	(CT)6	(CT)6	(CT)6	(CT)6	(CT)6	(CT)6	(CT)8	-	Introns/Intergenic	-	-	TCCGCTACCACCGCTACTAC	TGGTCATGGATGGTGCTAGA	138	A	A	A	NA
PMS4308	Os_Chr08	10394079	10394093	(TTA)5	(TTA)5	(TTA)6	(TTA)5	(TTA)5	(TTA)6	(TTA)5	(TTA)5	(TTA)5	(TTA)5	(TTA)5	-	Introns/Intergenic	-	-	GTGTAGCGATGGAGCATTTG	CACGAGCTTAAACTGTAGGCG	214	P	A	A	NA
PMS4309	Os_Chr08	10394290	10394316	(TTA)9	(TTA)8	(TTA)9	(TTA)9	(TTA)9	(TTA)7	(TTA)9	(TTA)9	(TTA)8	(TTA)9	(TTA)8	-	Introns/Intergenic	-	-	GTTTAAACTCGAGCTGCGATGG	CAAGATCTCAAGTGCCTAAACTGTGC	198	P	A	A	RM5663
PMS4310	Os_Chr08	10412871	10412904	(TA)17	(TA)16	(TA)7	(TA)17	(TA)17	(TA)17	(TA)17	(TA)8	(TA)17	(TA)17	(TA)17	-	Introns/Intergenic	-	-	CGCACTAGATGCGTCAGTTTGC	CCCTATTCTCCCTCTCTATCCTTTCC	290	P	A	A	RM22771
PMS4311	Os_Chr08	10534134	10534145	(AG)6	(AG)7	(AG)6	(AG)6	(AG)6	(AG)6	(AG)6	(AG)6	(AG)6	(AG)6	(AG)6	-	Introns/Intergenic	-	-	TTTGTCACCCATGTACCAGC	TGTTTCCCAGAAGAAATGGG	153	P	A	A	NA
PMS4312	Os_Chr08	10537337	10537350	(TG)7	(TG)7	(TG)7	(TG)7	(TG)7	(TG)7	(TG)7	(TG)7	(TG)7	(TG)6	(TG)6	-	Introns/Intergenic	-	-	GGACTAGCCTTGCATTCTGG	TTGGACAGACTTAGACGCCC	198	A	A	A	NA
PMS4313	Os_Chr08	10574202	10574227	(AT)13	(AT)13	(AT)13	(AT)13	(AT)13	(AT)13	(AT)6	(AT)13	(AT)13	(AT)13	(AT)13	-	Introns/Intergenic	-	-	GGAGGCAGTGGTAAGAGAAGAGG	GGTTAGAACTGCTTGTCATGAACC	676	A	A	A	RM22775
PMS4314	Os_Chr08	11062331	11062357	(CGG)9	(CGG)9	(CGG)9	(CGG)9	(CGG)9	(CGG)9	(CGG)9	(CGG)7	(CGG)9	(CGG)9	(CGG)7	LOC_Os08g18044	URR	tetraspanin family protein, putative, expressed	-	CAAAGAGGATCGAGATGCTCACC	CCTATGATCTGACGCCCTTCTCC	179	A	A	A	RM22786
PMS4315	Os_Chr08	11150420	11150437	(CCG)6	(CCG)6	(CCG)6	(CCG)6	(CCG)6	(CCG)5	(CCG)6	(CCG)5	(CCG)6	(CCG)6	(CCG)6	-	Introns/Intergenic	-	-	CTCCGATCTCGCATTCTCTC	GAGGTGGAGGTGGAGGTAGA	262	P	A	A	NA

Polymorphic SSR (PMS) markers	Chromosomes	Start physical positions (bp)	End physical positions (bp)	Nipponbare	Kasalath	Nagina22	IR64	Pokkali	Bala	Tainung67	Azucena	Moroberekan	*Oryza rufipogon*	*Oryza nivara*	MSU gene locus IDs	Structural annotation	Functional annotation	SSR functional effects	Forward primers (5'-3')	Reverse primers (5'-3')	Amplified product size (bp)	Markers exhibiting polymorphism within *indica* rice	Transcrption factor genes-derived markers	Known cloned genes-derived markers	PMS markers corresponding with RM markers
PMS4316	Os_Chr08	11314886	11314909	(CT)12	(CT)14	(CT)12	(CT)12	(CT)12	(CT)13	(CT)12	(CT)12	(CT)14	(CT)12	(CT)14	-	Introns/Intergenic	-	-	GGACCAACCTAAGCAGTGACTCG	CTGAAGAGCGATCATCAAATGTGG	199	P	A	A	RM22804
PMS4317	Os_Chr08	11320818	11320835	(TTA)6	(TTA)5	(TTA)6	(TTA)6	(TTA)6	(TTA)6	(TTA)6	(TTA)5	(TTA)5	(TTA)6	(TTA)6	-	Introns/Intergenic	-	-	AAGCTAGCACCGAGTCCTCA	GGTTGATTTATGCTCCCATCA	218	P	A	A	NA
PMS4318	Os_Chr08	11327973	11327984	(TA)6	(TA)7	(TA)6	(TA)6	(TA)6	(TA)6	(TA)6	(TA)6	(TA)6	(TA)6	(TA)6	-	Introns/Intergenic	-	-	GTTTCCCTGCCATCTGACAT	TATGTCCTTCATGTCCGGGT	222	P	A	A	NA
PMS4319	Os_Chr08	11369617	11369634	(CT)9	(CT)9	(CT)7	(CT)7	(CT)7	(CT)7	(CT)9	(CT)9	(CT)9	(CT)9	(CT)9	-	Introns/Intergenic	-	-	TCCTTATCCATTCCCTTCCC	TTAGATGGAGAGGACGGCAC	268	P	A	A	NA
PMS4320	Os_Chr08	11376018	11376033	(CT)8	(CT)8	(CT)9	(CT)9	(CT)9	(CT)9	(CT)8	(CT)10	(CT)10	(CT)8	(CT)8	-	Introns/Intergenic	-	-	GCTGTCTCCACCACACACAC	ACGCGTTTGCTCTTGACAAT	170	P	A	A	NA
PMS4321	Os_Chr08	11411191	11411244	(AT)27	(AT)27	(AT)27	(AT)27	(AT)9	(AT)24	(AT)27	(AT)27	(AT)27	(AT)8	(AT)10	-	Introns/Intergenic	-	-	CAATGGCGTTCCAAATTTCT	GGGTATATACTCAATTTCAACAATGG	260	P	A	A	NA
PMS4322	Os_Chr08	11411611	11411700	(TA)45	(TA)45	(TA)7	(TA)45	(TA)45	(TA)22	(TA)45	(TA)20	(TA)45	(TA)45	(TA)45	-	Introns/Intergenic	-	-	ATGGTGCCCCATATGAGTGT	ATGGTGGATGGTCTACAGCC	229	P	A	A	NA
PMS4323	Os_Chr08	11501662	11501675	(TA)7	(TA)7	(TA)7	(TA)7	(TA)7	(TA)7	(TA)7	(TA)7	(TA)6	(TA)7	(TA)7	-	Introns/Intergenic	-	-	CGTCGGTTCTTCCTCTTCTG	TGTTTGCTGCATGGTTGATT	249	A	A	A	NA
PMS4324	Os_Chr08	11623804	11623825	(TA)11	(TA)11	(TA)11	(TA)11	(TA)11	(TA)11	(TA)11	(TA)7	(TA)11	(TA)11	(TA)10	-	Introns/Intergenic	-	-	ATTCCTGAGATGGATTCTCTGC	AAAGGAAGACGAGGAAGTATGC	171	A	A	A	RM22819
PMS4325	Os_Chr08	11636833	11636852	(CT)10	(CT)10	(CT)10	(CT)10	(CT)10	(CT)10	(CT)10	(CT)10	(CT)10	(CT)9	(CT)10	-	Introns/Intergenic	-	-	TTTCTCTATTGGGCCGTCAGC	GGTAGGAGGCACGGGAAAGC	442	A	A	A	RM22820
PMS4326	Os_Chr08	11665353	11665370	(GA)9	(GA)9	(GA)9	(GA)9	(GA)9	(GA)9	(GA)9	(GA)6	(GA)6	(GA)6	(GA)6	-	Introns/Intergenic	-	-	CTGAGAGAGAGGAGGGAGGG	TTAAAATCCCCCTTTTTGGC	125	A	A	A	NA
PMS4327	Os_Chr08	11671337	11671376	(TA)20	(TA)20	(TA)20	(TA)20	(TA)20	(TA)20	(TA)20	(TA)20	(TA)20	(TA)6	(TA)7	-	Introns/Intergenic	-	-	TGAAACGGAGGAAGTATGCAC	AGGAAAGCTGTCTGCAAAGG	240	A	A	A	NA
PMS4328	Os_Chr08	11755172	11755193	(TC)11	(TC)11	(TC)11	(TC)11	(TC)11	(TC)12	(TC)11	(TC)11	(TC)11	(TC)11	(TC)11	-	Introns/Intergenic	-	-	ATTGTCACTCGCGCCTAATTCTACC	AATCCGTGACGTTGAGCTCTTGG	385	P	A	A	RM22824

Polymorphic SSR (PMS) markers	Chromosomes	Start physical positions (bp)	End physical positions (bp)	Nipponbare	Kasalath	Nagina22	IR64	Pokkali	Bala	Tainung67	Azucena	Moroberekan	*Oryza rufipogon*	*Oryza nivara*	MSU gene locus IDs	Structural annotation	Functional annotation	SSR functional effects	Forward primers (5'-3')	Reverse primers (5'-3')	Amplified product size (bp)	Markers exhibiting polymorphism within *indica* rice	Transcrption factor genes-derived markers	Known cloned genes-derived markers	PMS markers corresponding with RM markers
PMS4329	Os_Chr08	12295372	12295401	(CTT)10	(CTT)10	(CTT)10	(CTT)10	(CTT)10	(CTT)5	(CTT)10	(CTT)10	(CTT)10	(CTT)10	(CTT)10	LOC_Os08g20486	CDS	transposon protein, putative, unclassified, expressed	CODON_DELETION	ATGTTGCACTCCTTCAATGTCC	CATGAGACAATGCCAGAAAGC	627	P	P	A	RM331
PMS4330	Os_Chr08	12381619	12381645	(GCC)9	(GCC)9	(GCC)6	(GCC)7	(GCC)6	(GCC)9	(GCC)9	(GCC)9	(GCC)9	(GCC)9	(GCC)6	-	Introns/Intergenic	-	-	CCCATTTGTTGCCATTGACTAGC	ACTCCATGTCCGGGAACATACG	359	P	A	A	RM6471
PMS4331	Os_Chr08	12489697	12489717	(TGC)7	(TGC)7	(TGC)7	(TGC)7	(TGC)7	(TGC)7	(TGC)7	(TGC)7	(TGC)7	(TGC)7	(TGC)6	LOC_Os08g20800	CDS	retrotransposon protein, putative, Ty3-gypsy subclass, expressed	CODON_DELETION	ACGCTGTTGACTTTGAGCATCC	AGGAATTGGAGTCGCACAAGC	272	A	A	A	RM22840
PMS4332	Os_Chr08	12509352	12509375	(TGC)8	(TGC)7	(TGC)8	(TGC)8	(TGC)8	(TGC)8	(TGC)8	(TGC)8	(TGC)8	(TGC)8	(TGC)8	LOC_Os08g20849	CDS	retrotransposon protein, putative, Ty3-gypsy subclass, expressed	CODON_DELETION	TTGAACAGGCTGCACAGTTGG	ATCAACAGGGTGCTAACCAGAGG	200	P	A	A	RM22833
PMS4333	Os_Chr08	12635260	12635277	(TA)9	(TA)9	(TA)9	(TA)9	(TA)9	(TA)9	(TA)9	(TA)8	(TA)9	(TA)9	(TA)9	-	Introns/Intergenic	-	-	ATTATCCAACGCGGCATTAC	ACCATTCAACCTACCAACGC	222	A	A	A	NA
PMS4334	Os_Chr08	12635885	12635908	(AT)12	(AT)12	(AT)12	(AT)7	(AT)12	(AT)12	(AT)12	(AT)12	(AT)12	(AT)12	(AT)12	-	Introns/Intergenic	-	-	GTGAATCCTGAAACACATCTACGG	ACCACACGAGAGCTTGATGTACG	160	P	A	A	RM22844
PMS4335	Os_Chr08	12640105	12640122	(AT)9	(AT)9	(AT)6	(AT)9	(AT)9	(AT)9	(AT)9	(AT)9	(AT)9	(AT)9	(AT)9	-	Introns/Intergenic	-	-	AGCTACCACTTCGGCTTGAG	GACGTGCACGAACGAGATAA	180	P	A	A	NA
PMS4336	Os_Chr08	12646290	12646334	(GAA)15	(GAA)15	(GAA)15	(GAA)15	(GAA)15	(GAA)15	(GAA)15	(GAA)11	(GAA)15	(GAA)15	(GAA)15	LOC_Os08g21170	CDS	transposon protein, putative, CACTA, En/Spm sub-class, expressed	CODON_DELETION	TCCAAACCAGGCTACAGTACACG	CAACTGTACTGTGCTGACCATTGC	172	A	A	A	RM22845
PMS4337	Os_Chr08	12896598	12896612	(GAC)5	(GAC)5	(GAC)5	(GAC)7	(GAC)5	(GAC)7	(GAC)5	(GAC)5	(GAC)5	(GAC)5	(GAC)5	-	Introns/Intergenic	-	-	CCGACCATTTGTTCCTCTTC	GTCGTCGTCTTCGTCATCG	232	P	A	A	NA
PMS4338	Os_Chr08	13209522	13209543	(CT)11	(CT)11	(CT)11	(CT)11	(CT)11	(CT)10	(CT)11	(CT)11	(CT)11	(CT)9	(CT)11	-	Introns/Intergenic	-	-	GTCCACCCCTCTCCCATC	CACCTAGCGAACACGATGAA	161	P	A	A	NA
PMS4339	Os_Chr08	13311480	13311497	(CT)9	(CT)9	(CT)9	(CT)9	(CT)9	(CT)8	(CT)9	(CT)9	(CT)9	(CT)9	(CT)9	-	Introns/Intergenic	-	-	GAACCCACTCCGCACTGT	CCACTTAGCGAACACGATGA	126	P	A	A	NA
PMS4340	Os_Chr08	13348224	13348243	(CT)10	(CT)10	(CT)10	(CT)10	(CT)10	(CT)10	(CT)10	(CT)10	(CT)8	(CT)10	(CT)10	-	Introns/Intergenic	-	-	GTCCTCGCCCTCTTCCAT	ATCCTTAGTCCCTGCGATGA	113	A	A	A	NA
PMS4341	Os_Chr08	13764963	13765000	(TA)19	(TA)19	(TA)14	(TA)14	(TA)19	(TA)19	(TA)19	(TA)12	(TA)19	(TA)19	(TA)19	-	Introns/Intergenic	-	-	TGCTTGTTCTTCCTCCCAAGC	CTTGCCCGTATTGTGGTCTCG	183	P	A	A	RM22866

Polymorphic SSR (PMS) markers	Chromosomes	Start physical positions (bp)	End physical positions (bp)	Nipponbare	Kasalath	Nagina22	IR64	Pokkali	Bala	Tainung67	Azucena	Moroberekan	*Oryza rufipogon*	*Oryza nivara*	MSU gene locus IDs	Structural annotation	Functional annotation	SSR functional effects	Forward primers (5'-3')	Reverse primers (5'-3')	Amplified product size (bp)	Markers exhibiting polymorphism within *indica* rice	Transcrption factor genes-derived markers	Known cloned genes-derived markers	PMS markers corresponding with RM markers
PMS4342	Os_Chr08	13787499	13787519	(GTC)7	(GTC)7	(GTC)7	(GTC)7	(GTC)7	(GTC)7	(GTC)7	(GTC)7	(GTC)7	(GTC)7	(GTC)6	-	Introns/Intergenic	-	-	GTCATCATCCCGTTCCCTCT	CGAGGACGAACACGAGGAC	273	A	A	A	NA
PMS4343	Os_Chr08	13794501	13794521	(GTC)7	(GTC)7	(GTC)7	(GTC)7	(GTC)7	(GTC)7	(GTC)7	(GTC)7	(GTC)7	(GTC)7	(GTC)6	-	Introns/Intergenic	-	-	GTCATCATCCCGTTCCCTCT	CGAGGACGAACACGAGGAC	273	A	A	A	NA
PMS4344	Os_Chr08	13953014	13953033	(TA)10	(TA)11	(TA)10	(TA)10	(TA)10	(TA)6	(TA)10	(TA)8	(TA)10	(TA)10	(TA)10	-	Introns/Intergenic	-	-	GTGGCGGAATAGAAAGAGAGTGG	GGGTGATGATGTGACATGTTTGC	330	P	A	A	RM22871
PMS4345	Os_Chr08	13966504	13966517	(CT)7	(CT)6	(CT)7	(CT)7	(CT)7	(CT)7	(CT)7	(CT)7	(CT)7	(CT)7	(CT)7	-	Introns/Intergenic	-	-	CTCCAGCGCAAATCTAGCTC	TAGCTTGCCTGGAGGAGAGA	185	P	A	A	NA
PMS4346	Os_Chr08	14335402	14335437	(GGT)12	(GGT)13	(GGT)12	(GGT)12	(GGT)12	(GGT)12	(GGT)12	(GGT)10	(GGT)12	(GGT)12	(GGT)12	-	Introns/Intergenic	-	-	CTAACGCTTTGCCTGCTTCTGC	GTTGGGCCCATTACCACTCAGG	159	P	A	A	RM6637
PMS4347	Os_Chr08	14357037	14357060	(CCG)8	(CCG)8	(CCG)8	(CCG)8	(CCG)5	(CCG)8	(CCG)8	(CCG)8	(CCG)8	(CCG)8	(CCG)8	LOC_Os08g23730	URR	class I glutamine amidotransferase, putative, expressed	-	CCAGGATCCCGTGGGTAACTCC	GGGTGATGAGGTGGGTGTACTGC	194	P	A	A	RM5999
PMS4348	Os_Chr08	14449118	14449132	(CCT)5	(CCT)5	(CCT)5	(CCT)6	(CCT)6	(CCT)6	(CCT)5	(CCT)5	(CCT)5	(CCT)5	(CCT)5	-	Introns/Intergenic	-	-	CACTGACCAAGACCAAACCC	AAAAGAATGGATGCCGGTAG	263	P	A	A	NA
PMS4349	Os_Chr08	14492223	14492236	(TA)7	(TA)6	(TA)6	(TA)6	(TA)6	(TA)6	(TA)6	(TA)6	(TA)6	(TA)7	(TA)7	-	Introns/Intergenic	-	-	GGACATCCATGTCGTCATTCT	TGAATTGCTGCACTGTCTCA	270	A	A	A	NA
PMS4350	Os_Chr08	14593367	14593381	(CGA)5	(CGA)5	(CGA)5	(CGA)5	(CGA)5	(CGA)5	(CGA)5	(CGA)6	(CGA)6	(CGA)5	(CGA)5	LOC_Os08g24170	CDS	expressed protein	CODON_INSERTION	TGTATTTGTACTGCGGCGAG	TCGTGGTACCTGAAACCGAT	142	A	A	A	NA
PMS4351	Os_Chr08	14662868	14662899	(AT)16	(AT)16	(AT)16	(AT)16	(AT)16	(AT)16	(AT)7	(AT)16	(AT)16	(AT)16	(AT)9	-	Introns/Intergenic	-	-	AATTCAAGGATCTTCGGGAGTGG	CTTCCGCCTGCGAAGATAGG	300	A	A	A	RM22891
PMS4352	Os_Chr08	14692799	14692814	(CT)8	(CT)8	(CT)8	(CT)8	(CT)8	(CT)8	(CT)8	(CT)8	(CT)8	(CT)7	(CT)8	-	Introns/Intergenic	-	-	GCCTTGATTCTGTTGCCTGT	TCGGAAGGAGCTGACTCTGT	278	A	A	A	NA
PMS4353	Os_Chr08	14717863	14717878	(TG)8	(TG)9	(TG)9	(TG)9	(TG)9	(TG)9	(TG)9	(TG)9	(TG)9	(TG)9	(TG)9	-	Introns/Intergenic	-	-	ACGCCAGTACCTGAAAATGG	CAAACCAGGTTGTCAGGGTT	229	A	A	A	NA
PMS4354	Os_Chr08	14764330	14764345	(TA)8	(TA)8	(TA)8	(TA)8	(TA)8	(TA)8	(TA)8	(TA)8	(TA)8	(TA)9	(TA)8	-	Introns/Intergenic	-	-	TTAGGAATCCTTCAAGGCCA	TTTATAAGCCGTTGCCAAAA	227	A	A	A	NA

Polymorphic SSR (PMS) markers	Chromosomes	Start physical positions (bp)	End physical positions (bp)	Nipponbare	Kasalath	Nagina22	IR64	Pokkali	Bala	Tainung67	Azucena	Moroberekan	*Oryza rufipogon*	*Oryza nivara*	MSU gene locus IDs	Structural annotation	Functional annotation	SSR functional effects	Forward primers (5'-3')	Reverse primers (5'-3')	Amplified product size (bp)	Markers exhibiting polymorphism within *indica* rice	Transcrption factor genes-derived markers	Known cloned genes-derived markers	PMS markers corresponding with RM markers
PMS4355	Os_Chr08	14992270	14992287	(GCG)6	(GCG)6	(GCG)6	(GCG)6	(GCG)5	(GCG)6	(GCG)6	(GCG)6	(GCG)6	(GCG)6	(GCG)6	LOC_Os08g24760	URR	ATP-dependent RNA helicase, putative, expressed	-	CGATGGGAAAGGTGGAGAT	TGGCCCAACCAAATAGAAAG	206	P	A	A	NA
PMS4356	Os_Chr08	15027954	15027995	(AT)21	(AT)20	(AT)21	(AT)21	(AT)21	(AT)21	(AT)21	(AT)21	(AT)6	(AT)21	(AT)6	-	Introns/Intergenic	-	-	GGATCTTCTGGATTGTCTAACG	AGGAGCTCATATCTCTTCAACC	497	P	A	A	RM22903
PMS4357	Os_Chr08	15134638	15134651	(CT)7	(CT)7	(CT)7	(CT)7	(CT)7	(CT)7	(CT)7	(CT)7	(CT)7	(CT)6	(CT)7	-	Introns/Intergenic	-	-	TGGTCTAAAAGGAGCCCTCA	GAGGAGGGAGATGACAGGTG	280	A	A	A	NA
PMS4358	Os_Chr08	15227644	15227658	(GGC)5	(GGC)6	(GGC)5	(GGC)5	(GGC)5	(GGC)6	(GGC)6	(GGC)5	(GGC)5	(GGC)5	(GGC)5	LOC_Os08g25080	CDS	expressed protein	CODON_CHANGE_PLUS_CODON_INSERTION	GGGAACAATCTCGTGCTCTT	CACAAGCAAGCAAAGATCCA	246	P	A	A	NA
PMS4359	Os_Chr08	15330698	15330711	(CT)7	(CT)7	(CT)6	(CT)6	(CT)6	(CT)6	(CT)7	(CT)7	(CT)7	(CT)7	(CT)6	-	Introns/Intergenic	-	-	AGGATGCCACGTCATCTCTC	AGCCAGGAAAGTCCACGTC	130	P	A	A	NA
PMS4360	Os_Chr08	15342781	15342802	(TA)11	(TA)11	(TA)11	(TA)11	(TA)11	(TA)11	(TA)11	(TA)11	(TA)11	(TA)8	(TA)11	-	Introns/Intergenic	-	-	TGAACAAACCGAGAACTGTCTCC	CCAGATCGCATGGAATATATCG	342	A	A	A	RM22911
PMS4361	Os_Chr08	15359282	15359299	(TG)9	(TG)10	(TG)9	(TG)9	(TG)9	(TG)10	(TG)9	(TG)6	(TG)9	(TG)9	(TG)9	-	Introns/Intergenic	-	-	GAAAAAGACCAAATGGGCAA	GTTTCCTTTGTCCTCCCCTC	232	P	A	A	NA
PMS4362	Os_Chr08	15369249	15369275	(CCA)9	(CCA)9	(CCA)9	(CCA)7	(CCA)9	(CCA)9	(CCA)9	(CCA)9	(CCA)8	(CCA)9	(CCA)8	-	Introns/Intergenic	-	-	AGAAGGACCGCTTGCATAGATGG	GAGAGTTGCGAGGGAGAGAAGG	144	P	A	A	RM22912
PMS4363	Os_Chr08	15391503	15391516	(TC)7	(TC)6	(TC)7	(TC)7	(TC)7	(TC)7	(TC)7	(TC)7	(TC)7	(TC)7	(TC)7	-	Introns/Intergenic	-	-	GTAGCCCAAGAAAACCCACA	AGAGCCGAAGAGGAGGAAAC	172	P	A	A	NA
PMS4364	Os_Chr08	15511359	15511376	(GCC)6	(GCC)5	(GCC)6	(GCC)6	(GCC)6	(GCC)5	(GCC)6	(GCC)5	(GCC)5	(GCC)5	(GCC)6	LOC_Os08g25490	CDS	berberine and berberine like domain containing protein, expressed	CODON_DELETION	GTAAAACTCGTCGGGCAGAG	AGAACGTGTTCTGGGCGAT	194	P	A	A	NA
PMS4365	Os_Chr08	15630889	15630906	(GGA)6	(GGA)6	(GGA)6	(GGA)6	(GGA)6	(GGA)5	(GGA)6	(GGA)5	(GGA)6	(GGA)6	(GGA)6	LOC_Os08g25690	CDS	expressed protein	CODON_DELETION	GGAGGTCGATCACGGATG	CACACAGCACTAGCAGCGAC	280	P	A	A	NA
PMS4366	Os_Chr08	15645129	15645152	(CCG)8	(CCG)8	(CCG)8	(CCG)8	(CCG)8	(CCG)7	(CCG)5	(CCG)8	(CCG)8	(CCG)8	(CCG)8	LOC_Os08g25710	CDS	CSLD3 - cellulose synthase-like family D, expressed	CODON_DELETION	ATGCGCCACGTGAGGTAGAAGAGG	TTCGAGAGCTCCGGCACCTACG	257	P	A	A	RM22919
PMS4367	Os_Chr08	15773075	15773092	(GCG)6	(GCG)6	(GCG)6	(GCG)6	(GCG)6	(GCG)6	(GCG)6	(GCG)6	(GCG)6	(GCG)5	(GCG)5	LOC_Os08g25900	CDS	PsbP, putative, expressed	CODON_DELETION	GAATGAGGCCTTGGAAGGAG	CGAAACTCGAAAACCGACAG	280	A	A	A	NA

Polymorphic SSR (PMS) markers	Chromosomes	Start physical positions (bp)	End physical positions (bp)	Nipponbare	Kasalath	Nagina22	IR64	Pokkali	Bala	Tainung67	Azucena	Moroberekan	*Oryza rufipogon*	*Oryza nivara*	MSU gene locus IDs	Structural annotation	Functional annotation	SSR functional effects	Forward primers (5'-3')	Reverse primers (5'-3')	Amplified product size (bp)	Markers exhibiting polymorphism within *indica* rice	Transcrption factor genes-derived markers	Known cloned genes-derived markers	PMS markers corresponding with RM markers
PMS4368	Os_Chr08	15951245	15951258	(AG)7	(AG)8	(AG)8	(AG)8	(AG)8	(AG)8	(AG)7	(AG)8	(AG)8	(AG)7	(AG)8	-	Introns/Intergenic	-	-	CGCCTCCATCTCTTCTTTCA	GGGGCATAGAAGGGATTAGC	275	A	A	A	NA
PMS4369	Os_Chr08	15955333	15955348	(TA)8	(TA)8	(TA)8	(TA)8	(TA)8	(TA)8	(TA)8	(TA)8	(TA)8	(TA)7	(TA)8	-	Introns/Intergenic	-	-	TCAGCTTTCATATGGGTGTTTTT	AGGTGTGGGGTGTCATGAGT	217	A	A	A	NA
PMS4370	Os_Chr08	16037085	16037102	(GA)9	(GA)9	(GA)9	(GA)9	(GA)9	(GA)7	(GA)9	(GA)9	(GA)9	(GA)9	(GA)9	-	Introns/Intergenic	-	-	TCGCTTGATGATGCTATCCA	AAGCCATACTTCCTCCATGC	273	P	A	A	NA
PMS4371	Os_Chr08	16192670	16192749	(AT)40	(AT)33	(AT)40	(AT)40	(AT)40	(AT)40	(AT)40	(AT)40	(AT)40	(AT)6	(AT)40	-	Introns/Intergenic	-	-	AACACAGCCCAAATGTGTAACTCG	AGATCCCTGCGAAGAATGTCC	588	P	A	A	RM22942
PMS4372	Os_Chr08	16379955	16379974	(TATG)5	(TATG)6	(TATG)5	(TATG)5	(TATG)5	(TATG)5	(TATG)5	(TATG)5	(TATG)5	(TATG)6	(TATG)6	-	Introns/Intergenic	-	-	GGATCCAAGCCCTAATCCAT	CATATCCATCAATGCGTGAGA	174	P	A	A	NA
PMS4373	Os_Chr08	16506601	16506618	(AGC)6	(AGC)6	(AGC)6	(AGC)6	(AGC)6	(AGC)6	(AGC)5	(AGC)6	(AGC)6	(AGC)6	(AGC)6	-	Introns/Intergenic	-	-	GCGCGTTCTTTAATTCTTGC	TGCAATCCCAACTTACGACA	173	A	A	A	NA
PMS4374	Os_Chr08	16545269	16545306	(AT)19	(AT)19	(AT)19	(AT)7	(AT)8	(AT)19	(AT)19	(AT)19	(AT)19	(AT)19	(AT)19	-	Introns/Intergenic	-	-	ATACCTTGTTTGACGGTTGAGG	TAAACGGAGCCTTGGTGTAGC	178	P	A	A	RM22957
PMS4375	Os_Chr08	16582079	16582099	(GCA)7	(GCA)7	(GCA)7	(GCA)7	(GCA)7	(GCA)7	(GCA)7	(GCA)7	(GCA)7	(GCA)5	(GCA)7	LOC_Os08g27140	CDS	expressed protein	CODON_DELETION	TACACCACCTGGTTCACCAATCC	CACAAGTGCTGAAGAATCTCACTGG	192	A	A	A	RM22959
PMS4376	Os_Chr08	16765089	16765170	(AT)41	(AT)41	(AT)9	(AT)41	(AT)41	(AT)41	(AT)9	(AT)13	(AT)41	(AT)14	(AT)41	-	Introns/Intergenic	-	-	TGGTGCAGTAGAGTACAAAGACG	CTACAAGAGTGTGTGAGATGATCG	261	P	A	A	RM22970
PMS4377	Os_Chr08	16772609	16772620	(AG)6	(AG)6	(AG)6	(AG)7	(AG)7	(AG)6	(AG)6	(AG)6	(AG)7	(AG)6	(AG)6	-	Introns/Intergenic	-	-	TATTAATGGATGCCTTGGGC	GGTCTACGTGGCAGTCCAGT	257	P	A	A	NA
PMS4378	Os_Chr08	16783987	16784014	(AAAT)7	(AAAT)6	(AAAT)7	(AAAT)7	(AAAT)7	(AAAT)6	(AAAT)7	(AAAT)7	(AAAT)6	(AAAT)7	(AAAT)6	-	Introns/Intergenic	-	-	AGCGCTATTGCTGGGAATTA	TGGAGAGACACCCAACTGTG	248	P	A	A	NA
PMS4379	Os_Chr08	16855292	16855309	(TA)9	(TA)9	(TA)9	(TA)8	(TA)8	(TA)9	(TA)9	(TA)9	(TA)9	(TA)8	(TA)9	-	Introns/Intergenic	-	-	TTATCCAATGGAGCAAAGGG	TTTTTGACGGACGATGGAAG	184	P	A	A	NA
PMS4380	Os_Chr08	17265908	17265927	(ATGT)5	(ATGT)6	(ATGT)6	(ATGT)5	(ATGT)5	(ATGT)5	(ATGT)5	(ATGT)5	(ATGT)5	(ATGT)5	(ATGT)6	-	Introns/Intergenic	-	-	ATCGAATTAGACTCGGGCAACG	ATCGTGCTGGGAGACTATCAAGC	271	P	A	A	RM22980

Polymorphic SSR (PMS) markers	Chromosomes	Start physical positions (bp)	End physical positions (bp)	Nipponbare	Kasalath	Nagina22	IR64	Pokkali	Bala	Tainung67	Azucena	Moroberekan	*Oryza rufipogon*	*Oryza nivara*	MSU gene locus IDs	Structural annotation	Functional annotation	SSR functional effects	Forward primers (5'-3')	Reverse primers (5'-3')	Amplified product size (bp)	Markers exhibiting polymorphism within *indica* rice	Transcrption factor genes-derived markers	Known cloned genes-derived markers	PMS markers corresponding with RM markers
PMS4381	Os_Chr08	17338290	17338303	(CG)7	(CG)7	(CG)7	(CG)7	(CG)7	(CG)7	(CG)7	(CG)7	(CG)7	(CG)6	(CG)7	-	Introns/Intergenic	-	-	GGGCGTAGGATGATCAGATG	CCTGCTCTGTGATTGGTTCC	238	A	A	A	NA
PMS4382	Os_Chr08	17440803	17440822	(TA)10	(TA)10	(TA)6	(TA)10	(TA)10	(TA)10	(TA)10	(TA)10	(TA)10	(TA)9	(TA)10	-	Introns/Intergenic	-	-	TGAAGAGTAAGCTAGGGAGTCG	TAATTAGTAGTGCAGCCACACC	377	P	A	A	RM22983
PMS4383	Os_Chr08	17458210	17458229	(CT)10	(CT)7	(CT)10	(CT)10	(CT)10	(CT)9	(CT)10	(CT)10	(CT)10	(CT)10	(CT)10	LOC_Os08g28580	CDS	hypothetical protein	FRAME_SHIFT	TTGCCCATCCAAACAAACTGG	ACCATGGTCCTCCTTCCTGTCC	190	P	A	A	RM22984
PMS4384	Os_Chr08	17476624	17476655	(AT)16	(AT)16	(AT)8	(AT)16	(AT)16	(AT)16	(AT)8	(AT)16	(AT)16	(AT)16	(AT)16	-	Introns/Intergenic	-	-	GGTCCTAAAGACATCACAATGC	GTGCTGGTCACTTCTCAAATACC	290	P	A	A	RM22985
PMS4385	Os_Chr08	17557103	17557142	(AT)20	(AT)20	(AT)20	(AT)20	(AT)20	(AT)20	(AT)9	(AT)20	(AT)20	(AT)20	(AT)20	-	Introns/Intergenic	-	-	CTGGTAGCTCTTCGACCACTGC	GGAACGAATGAGTAGCATCTTAGGG	374	A	A	A	RM22990
PMS4386	Os_Chr08	17584522	17584589	(AT)34	(AT)34	(AT)10	(AT)34	(AT)34	(AT)34	(AT)8	(AT)11	(AT)34	(AT)34	(AT)34	-	Introns/Intergenic	-	-	GCTTACCGCACAAACACTCTTGG	GGGACTTAAGGGAAGGGAGTGC	448	P	A	A	RM22992
PMS4387	Os_Chr08	17783075	17783118	(TA)22	(TA)22	(TA)22	(TA)22	(TA)22	(TA)22	(TA)22	(TA)22	(TA)22	(TA)12	(TA)22	-	Introns/Intergenic	-	-	TGTCAGTTGAAGGAGTTACCAAGG	AGATTCGCCACATTGTGAGAGG	447	A	A	A	RM22999
PMS4388	Os_Chr08	17904302	17904319	(CCT)6	(CCT)5	(CCT)6	(CCT)6	(CCT)6	(CCT)6	(CCT)6	(CCT)6	(CCT)6	(CCT)6	(CCT)6	LOC_Os08g29200	CDS	BURP domain containing protein, expressed	CODON_DELETION	TCCTCTTCCAAATCCCCTCT	GTTCCAGTACCGGATGAACG	181	P	A	A	NA
PMS4389	Os_Chr08	17965524	17965535	(GA)6	(GA)6	(GA)6	(GA)6	(GA)6	(GA)7	(GA)6	(GA)6	(GA)6	(GA)6	(GA)6	-	Introns/Intergenic	-	-	GGGAGAGAAGGAGAGGGAGA	CCTTGGTCTAAAAGGAGCCC	228	P	A	A	NA
PMS4390	Os_Chr08	18024567	18024584	(CT)9	(CT)10	(CT)9	(CT)9	(CT)9	(CT)9	(CT)9	(CT)9	(CT)9	(CT)9	(CT)9	-	Introns/Intergenic	-	-	ATGTGTGGCTATATTCCGGC	CTGATGGCTCACTCCTCACA	116	P	A	A	NA
PMS4391	Os_Chr08	18080318	18080338	(GCC)7	(GCC)7	(GCC)7	(GCC)5	(GCC)7	(GCC)7	(GCC)7	(GCC)7	(GCC)5	(GCC)7	(GCC)7	-	Introns/Intergenic	-	-	TAAATAGCATCCCTTTGGCG	CCTCCGTGCGCATCTAAC	188	P	A	A	NA
PMS4392	Os_Chr08	18087109	18087148	(AT)20	(AT)20	(AT)8	(AT)20	(AT)10	(AT)9	(AT)20	(AT)20	(AT)14	(AT)20	(AT)7	-	Introns/Intergenic	-	-	CAAAGTTTGTATACGTGCATATTTTT	GCCTGTTCCTAATACGCCAC	200	P	A	A	NA
PMS4393	Os_Chr08	18252286	18252309	(CCT)8	(CCT)8	(CCT)8	(CCT)8	(CCT)8	(CCT)8	(CCT)8	(CCT)6	(CCT)8	(CCT)8	(CCT)8	LOC_Os08g29720	CDS	mitochondrial carrier protein, putative, expressed	CODON_CHANGE_PLUS_CODON_DELETION	ATGGTGGGCTGTTCCAGA	GAGAGGGTTGACGATGATGG	273	A	A	A	NA

Polymorphic SSR (PMS) markers	Chromosomes	Start physical positions (bp)	End physical positions (bp)	Nipponbare	Kasalath	Nagina22	IR64	Pokkali	Bala	Tainung67	Azucena	Moroberekan	*Oryza rufipogon*	*Oryza nivara*	MSU gene locus IDs	Structural annotation	Functional annotation	SSR functional effects	Forward primers (5'-3')	Reverse primers (5'-3')	Amplified product size (bp)	Markers exhibiting polymorphism within *indica* rice	Transcrption factor genes-derived markers	Known cloned genes-derived markers	PMS markers corresponding with RM markers
PMS4394	Os_Chr08	18265053	18265082	(TAT)10	(TAT)10	(TAT)10	(TAT)10	(TAT)10	(TAT)10	(TAT)10	(TAT)7	(TAT)10	(TAT)10	(TAT)10	-	Introns/Intergenic	-	-	TGATTGGCAGGCCATATTTT	CCGTTCTGCATACATTGTGG	233	A	A	A	NA
PMS4395	Os_Chr08	18292818	18292835	(CCG)6	(CCG)6	(CCG)6	(CCG)6	(CCG)6	(CCG)6	(CCG)6	(CCG)6	(CCG)6	(CCG)6	(CCG)5	LOC_Os08g29760	CDS	membrane protein, putative, expressed	CODON_CHANGE_PLUS_CODON_DELETION	AAAATGTTTGGGTTTCGCAG	CCATTAATTACCGGCCACAC	253	A	A	A	NA
PMS4396	Os_Chr08	18363592	18363609	(GAC)6	(GAC)6	(GAC)6	(GAC)6	(GAC)6	(GAC)6	(GAC)6	(GAC)5	(GAC)5	(GAC)5	(GAC)6	-	Introns/Intergenic	-	-	GAGATCCACTTGAGGTCCGA	CTTTGCTGAAATCCTGGCTC	273	A	A	A	NA
PMS4397	Os_Chr08	18365962	18366019	(TA)29	(TA)29	(TA)8	(TA)15	(TA)29	(TA)29	(TA)29	(TA)29	(TA)29	(TA)13	(TA)7	-	Introns/Intergenic	-	-	ACGGGACCAAACCTTATAGTGC	AAGGCCTCCTCACATCATAAGC	321	P	A	A	RM23022
PMS4398	Os_Chr08	18474345	18474374	(CT)15	(CT)15	(CT)15	(CT)15	(CT)15	(CT)15	(CT)15	(CT)15	(CT)13	(CT)15	(CT)15	-	Introns/Intergenic	-	-	CCATCTCCTCCTCACTCTGC	CCAAAAATTTTCCCTTTCCC	273	A	A	A	NA
PMS4399	Os_Chr08	18475526	18475546	(AGC)7	(AGC)7	(AGC)7	(AGC)7	(AGC)7	(AGC)7	(AGC)5	(AGC)7	(AGC)7	(AGC)7	(AGC)7	-	Introns/Intergenic	-	-	CTCTGACCTTGAGAAAGGAAATCG	GCATGATCTAATACCGGTGATGC	197	A	A	A	RM23026
PMS4400	Os_Chr08	18622981	18622994	(CT)7	(CT)7	(CT)7	(CT)7	(CT)7	(CT)7	(CT)7	(CT)6	(CT)6	(CT)7	(CT)7	-	Introns/Intergenic	-	-	TCCAAGAACGGATTGTTTCC	TTTCTTTTTCAATGCCACCC	132	A	A	A	NA
PMS4401	Os_Chr08	18763625	18763684	(AGAT)15	(AGAT)15	(AGAT)15	(AGAT)15	(AGAT)15	(AGAT)15	(AGAT)15	(AGAT)15	(AGAT)15	(AGAT)12	(AGAT)15	-	Introns/Intergenic	-	-	CGTTTATATGAGTGCCACATGACG	ACTTGAGGTTCTGGGCAAGACG	238	A	A	A	RM7285
PMS4402	Os_Chr08	18821646	18821672	(AGA)9	(AGA)9	(AGA)9	(AGA)9	(AGA)9	(AGA)5	(AGA)9	(AGA)9	(AGA)9	(AGA)9	(AGA)9	LOC_Os08g30554	CDS	expressed protein	CODON_CHANGE_PLUS_CODON_DELETION	GTCCGACTCACCTCCCTCTTGC	CCTCATCCTCTCCACGCTCTCC	190	P	A	A	RM5767
PMS4403	Os_Chr08	18935344	18935359	(CT)8	(CT)8	(CT)8	(CT)8	(CT)8	(CT)9	(CT)7	(CT)8	(CT)8	(CT)8	(CT)8	-	Introns/Intergenic	-	-	TGATATTGTGGGCAGACAGC	CGCGCGTGTTTAATTCAGTA	188	P	A	A	NA
PMS4404	Os_Chr08	19043555	19043578	(TC)12	(TC)11	(TC)12	(TC)12	(TC)12	(TC)11	(TC)12	(TC)11	(TC)12	(TC)12	(TC)12	-	Introns/Intergenic	-	-	CGAGTCAATCCTTCAGCCTC	TACGGACTCACCTCCTCGTC	235	P	A	A	NA
PMS4405	Os_Chr08	19043803	19043826	(GTG)8	(GTG)8	(GTG)8	(GTG)8	(GTG)8	(GTG)8	(GTG)8	(GTG)8	(GTG)8	(GTG)8	(GTG)5	LOC_Os08g30850	CDS	YDG/SRA domain containing protein, expressed	CODON_DELETION	TTCGTCGTGTTGTGATGTGA	GGGACAGCGAGACGGAAG	256	A	A	A	NA
PMS4406	Os_Chr08	19050952	19050973	(CT)11	(CT)11	(CT)11	(CT)10	(CT)10	(CT)10	(CT)11	(CT)10	(CT)10	(CT)11	(CT)10	-	Introns/Intergenic	-	-	CATCACCCATCCTTCTCTTCTCG	CGAACACGAAGAACAACACAGAGG	199	P	A	A	RM23052

Polymorphic SSR (PMS) markers	Chromosomes	Start physical positions (bp)	End physical positions (bp)	Nipponbare	Kasalath	Nagina22	IR64	Pokkali	Bala	Tainung67	Azucena	Moroberekan	*Oryza rufipogon*	*Oryza nivara*	MSU gene locus IDs	Structural annotation	Functional annotation	SSR functional effects	Forward primers (5'-3')	Reverse primers (5'-3')	Amplified product size (bp)	Markers exhibiting polymorphism within *indica* rice	Transcrption factor genes-derived markers	Known cloned genes-derived markers	PMS markers corresponding with RM markers
PMS4407	Os_Chr08	19106604	19106618	(GAT)5	(GAT)6	(GAT)5	(GAT)5	(GAT)5	(GAT)6	(GAT)5	(GAT)5	(GAT)6	(GAT)5	(GAT)6	LOC_Os08g30940	CDS	retrotransposon protein, putative, Ty3-gypsy subclass, expressed	CODON_INSERTION	TGCAGAAGCCAGGAAGAACT	ATTGTATGGCCAGCAGGAAG	252	P	A	A	NA
PMS4408	Os_Chr08	19290985	19290996	(GA)6	(GA)6	(GA)6	(GA)6	(GA)6	(GA)6	(GA)6	(GA)6	(GA)6	(GA)7	(GA)6	-	Introns/Intergenic	-	-	CCACCGTCGTTTACAGTGAG	TAATCCGTGGTGGTTTTGGT	187	A	A	A	NA
PMS4409	Os_Chr08	19323579	19323604	(AT)13	(AT)9	(AT)8	(AT)8	(AT)12	(AT)13	(AT)13	(AT)13	(AT)13	(AT)13	(AT)13	-	Introns/Intergenic	-	-	GGTCACATCACATATACCTTGG	CCTCTCCTACACAGATCTTACCC	397	P	A	A	RM23062
PMS4410	Os_Chr08	19357286	19357303	(GGC)6	(GGC)6	(GGC)6	(GGC)6	(GGC)6	(GGC)6	(GGC)6	(GGC)6	(GGC)6	(GGC)5	(GGC)6	-	Introns/Intergenic	-	-	ACGGCTAGACAATGGTGGAC	GAAAGTTGGCCCAAATAGCA	268	A	A	A	NA
PMS4411	Os_Chr08	19447719	19447738	(AT)10	(AT)10	(AT)8	(AT)10	(AT)10	(AT)10	(AT)10	(AT)10	(AT)10	(AT)10	(AT)10	-	Introns/Intergenic	-	-	CGTAAGCCAAATGAGCCAAGC	GCAAGCCTTCATGTTGAAACACC	300	P	A	A	RM23067
PMS4412	Os_Chr08	19596889	19596908	(AG)10	(AG)10	(AG)10	(AG)10	(AG)10	(AG)10	(AG)10	(AG)8	(AG)10	(AG)10	(AG)10	-	Introns/Intergenic	-	-	CTCTACCTGCCCGTGTGAC	AATTACCCCTCGACCCAATC	275	A	A	A	NA
PMS4413	Os_Chr08	19661312	19661327	(GA)8	(GA)7	(GA)8	(GA)8	(GA)8	(GA)8	(GA)8	(GA)8	(GA)8	(GA)7	(GA)8	-	Introns/Intergenic	-	-	CACCACCACAAACTTTGCAC	TCGGTTTGTTTTCTCCCCTA	139	P	A	A	NA
PMS4414	Os_Chr08	19726350	19726361	(CT)6	(CT)6	(CT)6	(CT)6	(CT)6	(CT)6	(CT)6	(CT)6	(CT)6	(CT)6	(CT)7	-	Introns/Intergenic	-	-	ATAGGTCCTCAGGCGGTTTT	CTAGTCCTGCTCCCCCTTCT	175	A	A	A	NA
PMS4415	Os_Chr08	19744333	19744366	(GA)17	(GA)17	(GA)17	(GA)17	(GA)17	(GA)17	(GA)17	(GA)7	(GA)17	(GA)17	(GA)17	-	Introns/Intergenic	-	-	CGGTGAGGAATTGTATTCGG	GTCGTCTCGCGTCTCTTCTC	278	A	A	A	NA
PMS4416	Os_Chr08	19770145	19770164	(GA)10	(GA)8	(GA)10	(GA)10	(GA)10	(GA)10	(GA)10	(GA)10	(GA)10	(GA)9	(GA)10	-	Introns/Intergenic	-	-	CGACGGAGATGACTCAGTGACG	CACCTAAGAGCATCACCAATCAGG	513	P	A	A	RM23088
PMS4417	Os_Chr08	19778706	19778735	(AAG)10	(AAG)10	(AAG)10	(AAG)10	(AAG)10	(AAG)8	(AAG)10	(AAG)10	(AAG)10	(AAG)10	(AAG)10	-	Introns/Intergenic	-	-	GCATCCTGAATGAACGGAAACC	ATCGGTGGAGGTGTGCTTCC	186	P	A	A	RM23089
PMS4418	Os_Chr08	19796527	19796553	(CGC)9	(CGC)9	(CGC)9	(CGC)9	(CGC)9	(CGC)9	(CGC)9	(CGC)6	(CGC)9	(CGC)9	(CGC)9	LOC_Os08g31930	URR	BRCA1 C Terminus domain containing protein, expressed	-	AGCCCACCTCCCATCAATCTTACG	GAGAGGCAGGCCAATCAGTGACG	245	A	A	A	RM23090
PMS4419	Os_Chr08	19836003	19836032	(GA)15	(GA)15	(GA)15	(GA)15	(GA)15	(GA)15	(GA)15	(GA)13	(GA)15	(GA)15	(GA)15	LOC_Os08g31980	URR	trehalose-6-phosphate synthase, putative, expressed	-	TTCTACGGAATTTCTCCCTCTGG	CTAATCAATCTCTCGCGTTCTTGG	162	A	A	A	RM8264

Polymorphic SSR (PMS) markers	Chromosomes	Start physical positions (bp)	End physical positions (bp)	Nipponbare	Kasalath	Nagina22	IR64	Pokkali	Bala	Tainung67	Azucena	Moroberekan	*Oryza rufipogon*	*Oryza nivara*	MSU gene locus IDs	Structural annotation	Functional annotation	SSR functional effects	Forward primers (5'-3')	Reverse primers (5'-3')	Amplified product size (bp)	Markers exhibiting polymorphism within *indica* rice	Transcrption factor genes-derived markers	Known cloned genes-derived markers	PMS markers corresponding with RM markers
PMS4420	Os_Chr08	20020974	20020997	(AAAT)6	(AAAT)5	(AAAT)6	(AAAT)6	(AAAT)6	(AAAT)5	(AAAT)6	(AAAT)6	(AAAT)6	(AAAT)6	(AAAT)6	-	Introns/Intergenic	-	-	CGGCAAAGGAGAATGTACACAGC	GGAGGAAATGGTTTGGTTTGTGC	190	P	A	A	RM23102
PMS4421	Os_Chr08	20025329	20025350	(TA)11	(TA)12	(TA)11	(TA)11	(TA)11	(TA)11	(TA)11	(TA)11	(TA)11	(TA)11	(TA)11	-	Introns/Intergenic	-	-	CGGAGTGAGGACTAGGGAGAGG	TCATTTGAGCAGCTTGAGAGTGC	270	P	A	A	RM23103
PMS4422	Os_Chr08	20037021	20037042	(TC)11	(TC)11	(TC)11	(TC)11	(TC)11	(TC)9	(TC)11	(TC)11	(TC)11	(TC)11	(TC)11	-	Introns/Intergenic	-	-	GCCATCGAAGACAAAGGTATGC	GTGGCAGGTTAAGCAAATTACGC	288	P	A	A	RM23104
PMS4423	Os_Chr08	20134566	20134589	(CCG)8	(CCG)8	(CCG)8	(CCG)8	(CCG)8	(CCG)8	(CCG)8	(CCG)8	(CCG)8	(CCG)8	(CCG)6	-	Introns/Intergenic	-	-	CGGCCTCGAGTCACAGAAACC	GCCCGTGAAATGCGTAAAGC	229	A	A	A	RM6182
PMS4424	Os_Chr08	20142557	20142574	(TC)9	(TC)9	(TC)9	(TC)9	(TC)9	(TC)9	(TC)9	(TC)9	(TC)9	(TC)8	(TC)9	-	Introns/Intergenic	-	-	AAAACAAGTGGGGGAAAAGAA	GGGTCCCATCATCAGGTTTA	144	A	A	A	NA
PMS4425	Os_Chr08	20166964	20166975	(AG)6	(AG)6	(AG)6	(AG)6	(AG)6	(AG)7	(AG)6	(AG)6	(AG)7	(AG)7	(AG)6	-	Introns/Intergenic	-	-	GTAGTCCACGCCTCACTCGT	GGGTCCCACCTTTCTCTTCT	102	P	A	A	NA
PMS4426	Os_Chr08	20183045	20183078	(TA)17	(TA)17	(TA)17	(TA)17	(TA)17	(TA)17	(TA)7	(TA)17	(TA)17	(TA)17	(TA)17	-	Introns/Intergenic	-	-	CGAAGACACCATTCAGAGCA	ATGCGAGGCAATGTAGGATT	233	A	A	A	NA
PMS4427	Os_Chr08	20194524	20194550	(TCT)9	(TCT)8	(TCT)9	(TCT)9	(TCT)9	(TCT)9	(TCT)9	(TCT)9	(TCT)9	(TCT)9	(TCT)9	-	Introns/Intergenic	-	-	GTTCATTTCGCGTTCTGATTCC	TTGCCGTTTGTTAATGCAGAGG	199	P	A	A	RM23116
PMS4428	Os_Chr08	20209257	20209271	(GCG)5	(GCG)6	(GCG)6	(GCG)6	(GCG)6	(GCG)6	(GCG)5	(GCG)5	(GCG)6	(GCG)5	(GCG)5	-	Introns/Intergenic	-	-	CGCGTTTGCACTTTTTCTTT	GCTCATAAACAGCAAAGCCC	185	A	A	A	NA
PMS4429	Os_Chr08	20317126	20317203	(TA)39	(TA)39	(TA)39	(TA)39	(TA)39	(TA)39	(TA)39	(TA)39	(TA)39	(TA)39	(TA)7	-	Introns/Intergenic	-	-	AGATGATCGGACTTCTAACTA	AATTCATTGTGGGGGTTC	500	A	A	A	NA
PMS4430	Os_Chr08	20382111	20382124	(AT)7	(AT)6	(AT)7	(AT)7	(AT)7	(AT)7	(AT)7	(AT)7	(AT)7	(AT)7	(AT)7	-	Introns/Intergenic	-	-	GCAATTTTGATGTGCCCTCT	TGAGCTGCATTAACACGGAG	120	P	A	A	NA
PMS4431	Os_Chr08	20460119	20460139	(CGG)7	(CGG)7	(CGG)7	(CGG)7	(CGG)7	(CGG)7	(CGG)5	(CGG)7	(CGG)7	(CGG)7	(CGG)7	LOC_Os08g32970	CDS	annexin, putative, expressed	CODON_DELETION	GAGGTGGTGTGCTCCAGG	AGAAGCAGCCTCTGGTGGT	140	A	A	A	NA
PMS4432	Os_Chr08	20486092	20486115	(AG)12	(AG)11	(AG)12	(AG)9	(AG)12	(AG)9	(AG)12	(AG)12	(AG)12	(AG)12	(AG)12	-	Introns/Intergenic	-	-	TCGGCTGATCTGACCGTACATCC	GTGCATGCAACCACCTCTTGG	179	P	A	A	RM8265,RM1109B,RM1109

**Polymorphic SSR (PMS) markers**	**Chromosomes**	**Start physical positions (bp)**	**End physical positions (bp)**	**Nipponbare**	**Kasalath**	**Nagina22**	**IR64**	**Pokkali**	**Bala**	**Tainung67**	**Azucena**	**Moroberekan**	***Oryza rufipogon***	***Oryza nivara***	**MSU gene locus IDs**	**Structural annotation**	**Functional annotation**	**SSR functional effects**	**Forward primers (5'-3')**	**Reverse primers (5'-3')**	**Amplified product size (bp)**	**Markers exhibiting polymorphism within *indica* rice**	**Transcrption factor genes-derived markers**	**Known cloned genes-derived markers**	**PMS markers corresponding with RM markers**
PMS4433	Os_Chr08	20486315	20486330	(AG)8	(AG)8	(AG)7	(AG)8	(AG)8	(AG)7	(AG)8	(AG)8	(AG)8	(AG)8	(AG)8	-	Introns/Intergenic	-	-	GGTCCCAACCCCTCTGTACT	AGTGATGTCGGTCCATACCC	229	P	A	A	NA
PMS4434	Os_Chr08	20540107	20540154	(TA)24	(TA)24	(TA)24	(TA)10	(TA)24	(TA)17	(TA)24	(TA)24	(TA)6	(TA)13	(TA)24	-	Introns/Intergenic	-	-	TCAATTTCAACAATGGTGCC	AAGTAGCAACCACCGACCAC	123	P	A	A	NA
PMS4435	Os_Chr08	20616542	20616557	(AT)8	(AT)8	(AT)8	(AT)8	(AT)8	(AT)7	(AT)8	(AT)8	(AT)8	(AT)8	(AT)8	-	Introns/Intergenic	-	-	TAACACCCCACACGATCTGA	TGTGGTTTGGACGTTTGGTA	270	P	A	A	NA
PMS4436	Os_Chr08	20769223	20769274	(AT)26	(AT)26	(AT)26	(AT)10	(AT)26	(AT)26	(AT)8	(AT)9	(AT)7	(AT)20	(AT)10	-	Introns/Intergenic	-	-	CACAGAGCTAGTTGTATTTGGACC	TTGATTTTAAGATGGCTCTATTTGG	165	P	A	A	NA
PMS4437	Os_Chr08	20930043	20930058	(AC)8	(AC)7	(AC)7	(AC)8	(AC)8	(AC)8	(AC)8	(AC)8	(AC)8	(AC)8	(AC)8	-	Introns/Intergenic	-	-	AATAAAGAGGCCAAGCAGCA	TGGTGCTCAAGCTCAAGAGA	206	P	A	A	NA
PMS4438	Os_Chr08	20947850	20947891	(CT)21	(CT)21	(CT)13	(CT)17	(CT)21	(CT)21	(CT)21	(CT)21	(CT)21	(CT)21	(CT)21	LOC_Os08g33540	URR	ATP-dependent Clp protease adaptor protein ClpS containing protein, expressed	-	GTGGTGGTGGTGGTTGGAGAGC	CCCAACTGGGCCACAAATACTCC	236	P	A	A	RM23161
PMS4439	Os_Chr08	20970400	20970427	(AT)14	(AT)14	(AT)14	(AT)6	(AT)14	(AT)6	(AT)14	(AT)14	(AT)14	(AT)14	(AT)8	-	Introns/Intergenic	-	-	CGAATGGTCAAACAGTGCAA	ATGCAGCATTCGCATCATTA	213	P	A	A	NA
PMS4440	Os_Chr08	20974150	20974231	(AT)41	(AT)41	(AT)41	(AT)41	(AT)41	(AT)41	(AT)41	(AT)6	(AT)41	(AT)41	(AT)41	-	Introns/Intergenic	-	-	CCTCCAATTCCACTTCCATTGTATCC	GGTGTTATGGTGTGTGCATGAGG	280	A	A	A	RM1578
PMS4441	Os_Chr08	20996759	20996774	(TC)8	(TC)8	(TC)8	(TC)8	(TC)8	(TC)8	(TC)8	(TC)6	(TC)8	(TC)8	(TC)8	-	Introns/Intergenic	-	-	ATAGGTCCCTCGGCAGTTTT	CTGCCCAGAGCCATAGAGAG	168	A	A	A	NA
PMS4442	Os_Chr08	21014495	21014515	(CGC)7	(CGC)7	(CGC)7	(CGC)7	(CGC)7	(CGC)7	(CGC)7	(CGC)6	(CGC)5	(CGC)5	(CGC)7	LOC_Os08g33650	CDS	SET domain-containing protein, putative, expressed	CODON_CHANGE_PLUS_CODON_DELETION	TTTAGTCCAAAACCCGCATC	CAACCTGCTGGGTTGGAG	177	A	A	A	NA
PMS4443	Os_Chr08	21078853	21078874	(TA)11	(TA)11	(TA)11	(TA)11	(TA)9	(TA)11	(TA)11	(TA)11	(TA)11	(TA)11	(TA)11	-	Introns/Intergenic	-	-	GGTGATTAATGGGTGGATGAAGG	GCCTAGAGTAAGTAGCCTTCACATGC	283	P	A	A	RM23173
PMS4444	Os_Chr08	21090265	21090296	(TA)16	(TA)16	(TA)16	(TA)16	(TA)16	(TA)16	(TA)7	(TA)16	(TA)16	(TA)16	(TA)16	LOC_Os08g33750	DRR	myb-like DNA-binding domain containing protein, expressed	-	CGTGAAACACAAGAACCAGATGC	AGCAATGTCTGCCTGCTACTGC	127	A	P	A	RM23175
PMS4445	Os_Chr08	21145155	21145178	(AG)12	(AG)11	(AG)12	(AG)12	(AG)12	(AG)12	(AG)12	(AG)12	(AG)12	(AG)12	(AG)12	-	Introns/Intergenic	-	-	ACTGCATGATCCTCCTCAGATCC	CCCTCTGATCTCTGATACTCCATCC	87	P	A	A	RM284

Polymorphic SSR (PMS) markers	Chromosomes	Start physical positions (bp)	End physical positions (bp)	Nipponbare	Kasalath	Nagina22	IR64	Pokkali	Bala	Tainung67	Azucena	Moroberekan	*Oryza rufipogon*	*Oryza nivara*	MSU gene locus IDs	Structural annotation	Functional annotation	SSR functional effects	Forward primers (5'-3')	Reverse primers (5'-3')	Amplified product size (bp)	Markers exhibiting polymorphism within *indica* rice	Transcrption factor genes-derived markers	Known cloned genes-derived markers	PMS markers corresponding with RM markers
PMS4446	Os_Chr08	21159955	21159982	(TA)14	(TA)14	(TA)14	(TA)14	(TA)8	(TA)14	(TA)14	(TA)11	(TA)14	(TA)14	(TA)14	-	Introns/Intergenic	-	-	TGTAGCCAGAAGAAATGTGTCG	ATATTCTCTCTGCAGCACAACG	358	P	A	A	RM23183
PMS4447	Os_Chr08	21213994	21214011	(TA)9	(TA)10	(TA)9	(TA)9	(TA)9	(TA)8	(TA)9	(TA)9	(TA)9	(TA)9	(TA)9	-	Introns/Intergenic	-	-	ATTGCTCATCGGCAGACAGT	AAAGTTTGTTTAGGGCAGGGA	223	P	A	A	NA
PMS4448	Os_Chr08	21235865	21235896	(TTCT)8	(TTCT)7	(TTCT)7	(TTCT)8	(TTCT)8	(TTCT)8	(TTCT)7	(TTCT)8	(TTCT)8	(TTCT)7	(TTCT)7	-	Introns/Intergenic	-	-	GATGACGAGAGCGATCAACAGC	GGGTACGTACGTGTGTGAATGTGG	152	P	A	A	RM23190
PMS4449	Os_Chr08	21240732	21240770	(TCT)13	(TCT)13	(TCT)13	(TCT)13	(TCT)13	(TCT)13	(TCT)13	(TCT)13	(TCT)9	(TCT)13	(TCT)13	LOC_Os08g33940	URR	MYB family transcription factor, putative, expressed	-	TTGCTTCCCTACCTTCTCCTTCC	GCACCACCTCCTCCAACTCC	173	A	P	A	RM5637
PMS4450	Os_Chr08	21265243	21265257	(GGC)5	(GGC)6	(GGC)5	(GGC)5	(GGC)5	(GGC)5	(GGC)5	(GGC)5	(GGC)5	(GGC)5	(GGC)5	LOC_Os08g33960	CDS	expressed protein	CODON_INSERTION	GGAGCCAATGGCGATGAG	CGTGCACACCTGCATCTAAC	213	P	A	A	NA
PMS4451	Os_Chr08	21282780	21282803	(TAGA)6	(TAGA)11	(TAGA)6	(TAGA)11	(TAGA)11	(TAGA)11	(TAGA)6	(TAGA)11	(TAGA)11	(TAGA)5	(TAGA)11	-	Introns/Intergenic	-	-	ATGCAATTCATGGCGCTGTTCG	CTCTCTCGGCCTCGATCTGTCG	381	P	A	A	RM7356
PMS4452	Os_Chr08	21329920	21329935	(GA)8	(GA)8	(GA)8	(GA)8	(GA)8	(GA)7	(GA)8	(GA)8	(GA)8	(GA)8	(GA)8	-	Introns/Intergenic	-	-	GCCATTTGCAGTAATGCGT	GCTCGTGGTACGTTGCTCAC	198	P	A	A	NA
PMS4453	Os_Chr08	21474381	21474392	(CT)6	(CT)8	(CT)6	(CT)6	(CT)6	(CT)6	(CT)6	(CT)6	(CT)6	(CT)6	(CT)6	-	Introns/Intergenic	-	-	TCTCAGACATGCAGTTTGGC	ACGACGACTTCTGGCTCATC	227	P	A	A	NA
PMS4454	Os_Chr08	21487383	21487394	(CT)6	(CT)8	(CT)6	(CT)6	(CT)6	(CT)6	(CT)6	(CT)6	(CT)6	(CT)6	(CT)6	-	Introns/Intergenic	-	-	TCTCAGACATGCAGTTTGGC	ACGACGACTTCTGGCTCATC	227	P	A	A	NA
PMS4455	Os_Chr08	21523361	21523378	(CA)9	(CA)8	(CA)8	(CA)9	(CA)9	(CA)9	(CA)9	(CA)9	(CA)9	(CA)9	(CA)9	-	Introns/Intergenic	-	-	CCGGAGTTAATTGGATGGTG	TGATTCAGATGTGTGTGCGA	163	P	A	A	NA
PMS4456	Os_Chr08	21564041	21564054	(AG)7	(AG)9	(AG)7	(AG)7	(AG)9	(AG)9	(AG)7	(AG)7	(AG)7	(AG)7	(AG)7	-	Introns/Intergenic	-	-	CGGATAGTTTGTGGCTGGTT	GCTTTAATATGCTCGCTCGC	259	P	A	A	NA
PMS4457	Os_Chr08	21564419	21564436	(GCG)6	(GCG)6	(GCG)6	(GCG)6	(GCG)6	(GCG)7	(GCG)6	(GCG)6	(GCG)6	(GCG)7	(GCG)6	LOC_Os08g34360	URR	AP2 domain containing protein, expressed	-	AGCAGCGACGACTCTAGCTC	AAAATCCAAACCACAAACGC	223	P	P	A	NA
PMS4458	Os_Chr08	21587927	21587944	(GCT)6	(GCT)6	(GCT)6	(GCT)6	(GCT)6	(GCT)6	(GCT)6	(GCT)5	(GCT)6	(GCT)6	(GCT)6	-	Introns/Intergenic	-	-	CGTTTGCTGGGAAAGAGAAG	AATGCAAGGGAAGAGAACGA	244	A	A	A	NA

Polymorphic SSR (PMS) markers	Chromosomes	Start physical positions (bp)	End physical positions (bp)	Nipponbare	Kasalath	Nagina22	IR64	Pokkali	Bala	Tainung67	Azucena	Moroberekan	*Oryza rufipogon*	*Oryza nivara*	MSU gene locus IDs	Structural annotation	Functional annotation	SSR functional effects	Forward primers (5'-3')	Reverse primers (5'-3')	Amplified product size (bp)	Markers exhibiting polymorphism within *indica* rice	Transcrption factor genes-derived markers	Known cloned genes-derived markers	PMS markers corresponding with RM markers
PMS4459	Os_Chr08	21901668	21901721	(TA)27	(TA)27	(TA)6	(TA)27	(TA)27	(TA)27	(TA)27	(TA)27	(TA)27	(TA)27	(TA)12	-	Introns/Intergenic	-	-	GTTATTCCGTTGCAACACACG	CCACGGGAGTGTAATATAGGAATAGG	426	P	A	A	RM23220
PMS4460	Os_Chr08	21919769	21919792	(GCC)8	(GCC)8	(GCC)8	(GCC)8	(GCC)8	(GCC)8	(GCC)8	(GCC)8	(GCC)8	(GCC)8	(GCC)6	-	Introns/Intergenic	-	-	AGACGTCGGCAACAACAACTACC	AGTGAGTCTCCCACCCTGTGC	362	A	A	A	RM15148
PMS4461	Os_Chr08	22000306	22000325	(TA)10	(TA)10	(TA)10	(TA)10	(TA)7	(TA)10	(TA)10	(TA)10	(TA)10	(TA)10	(TA)10	-	Introns/Intergenic	-	-	ACCTATTACCCGTAAGATGCAAGG	GAGCATATTTGATGCCTTCTGG	555	P	A	A	RM23224
PMS4462	Os_Chr08	22093067	22093087	(GGC)7	(GGC)6	(GGC)6	(GGC)6	(GGC)6	(GGC)6	(GGC)5	(GGC)7	(GGC)7	(GGC)7	(GGC)6	-	Introns/Intergenic	-	-	AATCGACTATCACAACACCGAACC	CCAGCTCTACCCTCTCCTCTTCC	123	A	A	A	RM23227
PMS4463	Os_Chr08	22150709	22150722	(CT)7	(CT)7	(CT)7	(CT)6	(CT)6	(CT)6	(CT)7	(CT)7	(CT)7	(CT)7	(CT)7	-	Introns/Intergenic	-	-	ATGAAAGAAATTTGCGTGGG	CGATCTGTATGGGGCAGAGT	184	P	A	A	NA
PMS4464	Os_Chr08	22164944	22164959	(CT)8	(CT)8	(CT)8	(CT)8	(CT)7	(CT)7	(CT)8	(CT)8	(CT)8	(CT)8	(CT)8	-	Introns/Intergenic	-	-	TATTTGAAAATAACGCCCGC	TTTGGTGTGGTTGCTACCCT	100	P	A	A	NA
PMS4465	Os_Chr08	22173346	22173375	(GGT)10	(GGT)10	(GGT)10	(GGT)10	(GGT)10	(GGT)10	(GGT)7	(GGT)10	(GGT)10	(GGT)10	(GGT)10	-	Introns/Intergenic	-	-	CAAATACTTCTCCATCTGACCGTACC	GTTGCTGTCGATTCGGATTAGG	178	A	A	A	RM6635
PMS4466	Os_Chr08	22188780	22188795	(AT)8	(AT)8	(AT)8	(AT)6	(AT)8	(AT)8	(AT)8	(AT)8	(AT)8	(AT)8	(AT)8	-	Introns/Intergenic	-	-	TGAAACGGAGGAAGTAACACC	TTACATTGGTACGTGCGGAA	200	P	A	A	NA
PMS4467	Os_Chr08	22263328	22263353	(GA)13	(GA)13	(GA)13	(GA)13	(GA)13	(GA)13	(GA)13	(GA)12	(GA)13	(GA)13	(GA)13	-	Introns/Intergenic	-	-	CACGACAGAGCGAGGAGAG	CCGCAAAAGTCTAGTTTGCC	260	A	A	A	NA
PMS4468	Os_Chr08	22381275	22381300	(AG)13	(AG)13	(AG)13	(AG)13	(AG)13	(AG)10	(AG)13	(AG)13	(AG)13	(AG)13	(AG)13	-	Introns/Intergenic	-	-	CTGGAGATGCAGATCCTCAACC	TAGTACAACATGGGAGCCTGTCG	296	P	A	A	RM3262
PMS4469	Os_Chr08	22414618	22414638	(CGG)7	(CGG)6	(CGG)7	(CGG)7	(CGG)7	(CGG)6	(CGG)5	(CGG)5	(CGG)7	(CGG)7	(CGG)7	LOC_Os08g35590	CDS	expressed protein	CODON_DELETION	CTGTCCTACCTCGATCGATCACC	GAAGACCTTGGTGAGCCAGACC	391	P	A	A	RM23245
PMS4470	Os_Chr08	22415395	22415409	(AAG)5	(AAG)7	(AAG)7	(AAG)7	(AAG)7	(AAG)7	(AAG)5	(AAG)5	(AAG)5	(AAG)5	(AAG)7	-	Introns/Intergenic	-	-	GCCAAAAGATAGAGCCGATG	CCCAGAATCGATGGTTGACT	230	A	A	A	NA
PMS4471	Os_Chr08	22468181	22468194	(AT)7	(AT)6	(AT)7	(AT)7	(AT)7	(AT)6	(AT)7	(AT)7	(AT)7	(AT)7	(AT)6	-	Introns/Intergenic	-	-	CATCATTATGGCAACACCAAA	GACTGTCATGTTGCACGCTT	178	P	A	A	NA

Polymorphic SSR (PMS) markers	Chromosomes	Start physical positions (bp)	End physical positions (bp)	Nipponbare	Kasalath	Nagina22	IR64	Pokkali	Bala	Tainung67	Azucena	Moroberekan	*Oryza rufipogon*	*Oryza nivara*	MSU gene locus IDs	Structural annotation	Functional annotation	SSR functional effects	Forward primers (5'-3')	Reverse primers (5'-3')	Amplified product size (bp)	Markers exhibiting polymorphism within *indica* rice	Transcrption factor genes-derived markers	Known cloned genes-derived markers	PMS markers corresponding with RM markers
PMS4472	Os_Chr08	22474646	22474691	(GA)23	(GA)22	(GA)23	(GA)23	(GA)23	(GA)23	(GA)23	(GA)23	(GA)23	(GA)23	(GA)23	-	Introns/Intergenic	-	-	GACAACTCCATATCAACGCAAAGC	GAGGATGGTTGTTCACTTGTTTGG	186	P	A	A	RM210
PMS4473	Os_Chr08	22558242	22558256	(ACG)5	(ACG)6	(ACG)6	(ACG)6	(ACG)6	(ACG)6	(ACG)5	(ACG)5	(ACG)5	(ACG)6	(ACG)6	LOC_Os08g35760	DRR	OsGLP1	-	TTCCCATGACAGAAGTGCAT	GGTGAAGAAGCTCAAGTCCG	155	A	A	P	NA
PMS4474	Os_Chr08	22619623	22619634	(TC)6	(TC)9	(TC)6	(TC)6	(TC)6	(TC)9	(TC)6	(TC)6	(TC)6	(TC)6	(TC)9	LOC_Os08g35860	URR	cytokinin dehydrogenase precursor, putative, expressed	-	CATGTACGCGAGCATCATCT	GTGATGGCTGTTTTGGAGGT	226	P	A	A	NA
PMS4475	Os_Chr08	22688446	22688485	(TA)20	(TA)20	(TA)9	(TA)6	(TA)20	(TA)20	(TA)7	(TA)7	(TA)20	(TA)20	(TA)20	-	Introns/Intergenic	-	-	GCAGGTCTAATGACCTCAAGTAATGC	CCTCGACTTCAATGCACTTATGC	373	P	A	A	RM23260
PMS4476	Os_Chr08	22790327	22790342	(CT)8	(CT)8	(CT)8	(CT)8	(CT)8	(CT)8	(CT)8	(CT)8	(CT)8	(CT)6	(CT)8	-	Introns/Intergenic	-	-	GGTTATCTCCCACCTCCAGC	CCCTATTTGGCCGGAGAC	245	A	A	A	NA
PMS4477	Os_Chr08	22830445	22830465	(CGC)7	(CGC)7	(CGC)7	(CGC)7	(CGC)7	(CGC)7	(CGC)7	(CGC)7	(CGC)7	(CGC)5	(CGC)7	LOC_Os08g36220	CDS	homeobox associated leucine zipper, putative, expressed	CODON_DELETION	CCATGCATTCTTACGGGATTCAGC	ATCCTTCCCGCTGGACCTCTCC	219	A	P	A	RM23266
PMS4478	Os_Chr08	22865496	22865530	(CCTGC)7	(CCTGC)7	(CCTGC)7	(CCTGC)6	(CCTGC)7	(CCTGC)7	(CCTGC)7	(CCTGC)7	(CCTGC)7	(CCTGC)7	(CCTGC)7	-	Introns/Intergenic	-	-	GCAGCAAAACAAAACTGCAA	AGTCGCTTGAAGCATGTGTG	204	P	A	A	NA
PMS4479	Os_Chr08	22994529	22994546	(TGG)6	(TGG)7	(TGG)7	(TGG)7	(TGG)7	(TGG)7	(TGG)6	(TGG)6	(TGG)6	(TGG)6	(TGG)7	LOC_Os08g36420	CDS	metal cation transporter, putative, expressed	CODON_INSERTION	GTGATAACTCGGCCTGGAAG	CCACGATGATCACCACCAC	275	A	A	A	NA
PMS4480	Os_Chr08	23059860	23059886	(TGA)9	(TGA)5	(TGA)9	(TGA)9	(TGA)9	(TGA)9	(TGA)9	(TGA)9	(TGA)9	(TGA)9	(TGA)9	LOC_Os08g36510	CDS	retrotransposon protein, putative, unclassified, expressed	CODON_DELETION	CCTCTCACTCCTTCCGTGAG	ATCAATCCTCGTCTTCGTCG	255	P	A	A	NA
PMS4481	Os_Chr08	23120736	23120753	(AC)9	(AC)9	(AC)9	(AC)9	(AC)9	(AC)7	(AC)9	(AC)9	(AC)9	(AC)9	(AC)9	-	Introns/Intergenic	-	-	GGTGGGTAATAACCCTCATCG	GGCCGGAAGTCAATCACTAA	173	P	A	A	NA
PMS4482	Os_Chr08	23156439	23156454	(TC)8	(TC)8	(TC)8	(TC)8	(TC)8	(TC)8	(TC)8	(TC)8	(TC)10	(TC)8	(TC)8	-	Introns/Intergenic	-	-	TGTCAAGGTGTCCACGTGAT	GCTGCTGAAGCACCACCTAT	179	A	A	A	NA
PMS4483	Os_Chr08	23187857	23187870	(CG)7	(CG)8	(CG)7	(CG)8	(CG)6	(CG)8	(CG)7	(CG)7	(CG)7	(CG)8	(CG)8	-	Introns/Intergenic	-	-	GTCACGCAAAGGATCGAAAT	GTACGCATGGGCTAGCTGTT	226	P	A	A	NA
PMS4484	Os_Chr08	23213404	23213430	(GGC)9	(GGC)9	(GGC)9	(GGC)9	(GGC)5	(GGC)9	(GGC)9	(GGC)9	(GGC)9	(GGC)9	(GGC)9	LOC_Os08g36760	CDS	remorin C-terminal domain containing protein, putative, expressed	CODON_DELETION	ATTCTGCTCGGTAGAGGAGATGC	AACCGAGTCGCAGAGAGACG	177	P	A	A	RM23289

Polymorphic SSR (PMS) markers	Chromosomes	Start physical positions (bp)	End physical positions (bp)	Nipponbare	Kasalath	Nagina22	IR64	Pokkali	Bala	Tainung67	Azucena	Moroberekan	*Oryza rufipogon*	*Oryza nivara*	MSU gene locus IDs	Structural annotation	Functional annotation	SSR functional effects	Forward primers (5'-3')	Reverse primers (5'-3')	Amplified product size (bp)	Markers exhibiting polymorphism within *indica* rice	Transcrption factor genes-derived markers	Known cloned genes-derived markers	PMS markers corresponding with RM markers
PMS4485	Os_Chr08	23234557	23234577	(CGG)7	(CGG)5	(CGG)7	(CGG)7	(CGG)7	(CGG)5	(CGG)7	(CGG)7	(CGG)7	(CGG)7	(CGG)7	LOC_Os08g36790	CDS	TRAB1|OsbZIP66	CODON_CHANGE_PLUS_CODON_DELETION	CACTGACGTTCGACGAGTTC	GACGCCTCTCGCTCTAAGTC	256	P	P	P	NA
PMS4486	Os_Chr08	23292691	23292717	(CGC)9	(CGC)9	(CGC)9	(CGC)9	(CGC)9	(CGC)9	(CGC)9	(CGC)6	(CGC)9	(CGC)9	(CGC)5	LOC_Os08g36860	CDS	OsABA8ox2	CODON_DELETION	GGTACTTGAGGATCCAGGTGAGG	TCGTGGAGAGAGGCTACAACTGC	379	A	A	P	RM23296
PMS4487	Os_Chr08	23302213	23302242	(TA)15	(TA)15	(TA)15	(TA)11	(TA)15	(TA)15	(TA)7	(TA)15	(TA)15	(TA)15	(TA)8	-	Introns/Intergenic	-	-	TGCACATGATGTTGACAGAAGA	ATTAGATACCATGGTGCCCG	215	P	A	A	NA
PMS4488	Os_Chr08	23381875	23381901	(TCG)9	(TCG)9	(TCG)9	(TCG)8	(TCG)9	(TCG)9	(TCG)9	(TCG)9	(TCG)9	(TCG)9	(TCG)9	LOC_Os08g36960	CDS	OsFBX296 - F-box domain containing protein, expressed	CODON_DELETION	CGGCGTCTCTGTGATATCTTGG	GAACTCTGGGCTGGCTATCAGG	295	P	A	A	RM23301
PMS4489	Os_Chr08	23430890	23430904	(GTG)5	(GTG)6	(GTG)6	(GTG)6	(GTG)6	(GTG)6	(GTG)5	(GTG)5	(GTG)5	(GTG)6	(GTG)6	-	Introns/Intergenic	-	-	CAAGATTGCAAGAAGCAAATCA	AAGACAGTGATGATGGCAGC	169	A	A	A	NA
PMS4490	Os_Chr08	23473682	23473695	(CT)7	(CT)7	(CT)7	(CT)7	(CT)7	(CT)7	(CT)7	(CT)7	(CT)7	(CT)8	(CT)7	-	Introns/Intergenic	-	-	CCCTTGTCGACCAGAACAAT	ATTGATGGTGGAGGTGTGGT	210	A	A	A	NA
PMS4491	Os_Chr08	23512660	23512707	(GA)24	(GA)24	(GA)24	(GA)24	(GA)24	(GA)24	(GA)24	(GA)19	(GA)24	(GA)24	(GA)24	-	Introns/Intergenic	-	-	AAGAACGGAGGGAGACACTACCG	CGTCGAGATCAATTCCGAATCC	471	A	A	A	RM23307
PMS4492	Os_Chr08	23558619	23558636	(CGC)6	(CGC)5	(CGC)6	(CGC)6	(CGC)6	(CGC)5	(CGC)6	(CGC)6	(CGC)6	(CGC)6	(CGC)6	LOC_Os08g37270	CDS	heat shock protein DnaJ, putative, expressed	CODON_DELETION	ATCCAATGGGACCCTACTCC	CGATCACCACATGCTTGTTC	216	P	A	A	NA
PMS4493	Os_Chr08	23560780	23560794	(ACA)5	(ACA)6	(ACA)5	(ACA)5	(ACA)5	(ACA)5	(ACA)5	(ACA)5	(ACA)5	(ACA)5	(ACA)5	-	Introns/Intergenic	-	-	CGGATCTAGCGTCTTCGTTC	TCCATGAGCCACCATCACTA	221	P	A	A	NA
PMS4494	Os_Chr08	23578912	23578926	(GGA)5	(GGA)6	(GGA)6	(GGA)6	(GGA)5	(GGA)6	(GGA)5	(GGA)5	(GGA)5	(GGA)5	(GGA)6	LOC_Os08g37310	CDS	uncharacterized protein, YigZ family, putative, expressed	CODON_INSERTION	TTAAGACTTGCCCGATTTGG	CTCCTCCTGTGGCGGTAG	275	P	A	A	NA
PMS4495	Os_Chr08	23582095	23582112	(CGC)6	(CGC)7	(CGC)7	(CGC)7	(CGC)7	(CGC)7	(CGC)6	(CGC)6	(CGC)6	(CGC)7	(CGC)7	LOC_Os08g37320	URR	expressed protein	-	CCTCGACCTCTCTCTCTCCC	GGCCCGTAGTGGGTAGAAAT	215	A	A	A	NA
PMS4496	Os_Chr08	23644536	23644559	(TA)12	(TA)12	(TA)12	(TA)12	(TA)12	(TA)12	(TA)12	(TA)12	(TA)12	(TA)10	(TA)12	-	Introns/Intergenic	-	-	ACACGATATCCCGAGCAAAG	TGATTTTGTGCATTTGAAACC	197	A	A	A	NA
PMS4497	Os_Chr08	23916956	23916967	(CT)6	(CT)6	(CT)6	(CT)6	(CT)6	(CT)6	(CT)6	(CT)6	(CT)6	(CT)7	(CT)6	-	Introns/Intergenic	-	-	TACATGCTTCTTGTGGCAGG	GGTTCAACAAAGGCTGCACT	266	A	A	A	NA

Polymorphic SSR (PMS) markers	Chromosomes	Start physical positions (bp)	End physical positions (bp)	Nipponbare	Kasalath	Nagina22	IR64	Pokkali	Bala	Tainung67	Azucena	Moroberekan	*Oryza rufipogon*	*Oryza nivara*	MSU gene locus IDs	Structural annotation	Functional annotation	SSR functional effects	Forward primers (5'-3')	Reverse primers (5'-3')	Amplified product size (bp)	Markers exhibiting polymorphism within *indica* rice	Transcrption factor genes-derived markers	Known cloned genes-derived markers	PMS markers corresponding with RM markers
PMS4498	Os_Chr08	23970054	23970071	(GGC)6	(GGC)6	(GGC)6	(GGC)6	(GGC)6	(GGC)6	(GGC)6	(GGC)5	(GGC)6	(GGC)6	(GGC)6	LOC_Os08g37840	CDS	phosphate-induced protein 1 conserved region domain containing protein, expressed	CODON_DELETION	CCGCAACTGTTTGATGTCACC	ATCGTGGGCCAGATGTTAGG	221	A	A	A	RM23336
PMS4499	Os_Chr08	24014417	24014430	(GA)7	(GA)7	(GA)7	(GA)7	(GA)7	(GA)7	(GA)7	(GA)6	(GA)7	(GA)7	(GA)7	-	Introns/Intergenic	-	-	GGGGAGCCTAGGACAGTGAG	GATGCCACCTCATCACCTCT	249	A	A	A	NA
PMS4500	Os_Chr08	24015041	24015060	(CT)10	(CT)10	(CT)10	(CT)10	(CT)10	(CT)10	(CT)10	(CT)9	(CT)10	(CT)10	(CT)10	LOC_Os08g37920	URR	ZOS8-09 - C2H2 zinc finger protein, expressed	-	ACAGCCTATAGCTCACACCAAACC	GAACACCTCCGTCTCCATTGC	224	A	P	A	RM23339
PMS4501	Os_Chr08	24069951	24069972	(CT)11	(CT)11	(CT)11	(CT)11	(CT)11	(CT)11	(CT)11	(CT)8	(CT)11	(CT)11	(CT)11	-	Introns/Intergenic	-	-	AGATTGTGTAGGGCTGGTCTGC	GATTTGATGAGGCCTCTCTTTGC	88	A	A	A	RM23343
PMS4502	Os_Chr08	24083063	24083116	(TA)27	(TA)27	(TA)6	(TA)27	(TA)27	(TA)27	(TA)27	(TA)27	(TA)9	(TA)27	(TA)27	LOC_Os08g38020	DRR	bZIP transcription factor domain containing protein, expressed	-	GAGATCCTGCACATCTTTGAGACC	TGTGCCACGAAACAAATCTAGGC	206	P	P	A	RM23345
PMS4503	Os_Chr08	24084486	24084506	(GTG)7	(GTG)7	(GTG)7	(GTG)7	(GTG)7	(GTG)7	(GTG)5	(GTG)7	(GTG)7	(GTG)7	(GTG)7	LOC_Os08g38020	CDS	bZIP transcription factor domain containing protein, expressed	CODON_DELETION	CAGCAGTGGCTTCTTGGAAC	CTCCAAACCCTCCCTTCTTC	135	A	P	A	NA
PMS4504	Os_Chr08	24202673	24202692	(AT)10	(AT)10	(AT)10	(AT)10	(AT)10	(AT)10	(AT)10	(AT)8	(AT)10	(AT)10	(AT)10	-	Introns/Intergenic	-	-	GACGAATCCCATTTCGTCAT	ACACTATCTCCGCCACTGCT	183	A	A	A	NA
PMS4505	Os_Chr08	24223545	24223565	(CCG)7	(CCG)7	(CCG)7	(CCG)7	(CCG)6	(CCG)7	(CCG)7	(CCG)7	(CCG)7	(CCG)7	(CCG)7	LOC_Os08g38210	CDS	transcription factor BIM2, putative, expressed	CODON_DELETION	CGATCAGATCTCCGCAAGTTGG	GGCGTGGTTGATGGTGAACG	361	P	P	A	RM23352
PMS4506	Os_Chr08	24273393	24273410	(CT)9	(CT)10	(CT)10	(CT)9	(CT)9	(CT)10	(CT)9	(CT)9	(CT)9	(CT)9	(CT)9	-	Introns/Intergenic	-	-	GAGATCAACCCACCACCAAC	AAGAGATGATGTTTGCGTGC	268	P	A	A	NA
PMS4507	Os_Chr08	24384890	24384921	(TTTA)8	(TTTA)7	(TTTA)8	(TTTA)8	(TTTA)8	(TTTA)8	(TTTA)8	(TTTA)8	(TTTA)8	(TTTA)6	(TTTA)8	-	Introns/Intergenic	-	-	CAGAGAGGTGAGATTGTGACG	ACAGTAACAGAGTGCTGAAACG	380	P	A	A	RM23358
PMS4508	Os_Chr08	24396326	24396340	(GCT)5	(GCT)5	(GCT)5	(GCT)5	(GCT)5	(GCT)5	(GCT)5	(GCT)5	(GCT)5	(GCT)6	(GCT)5	-	Introns/Intergenic	-	-	CCCAACACAAACGATGCTAA	AGCAGACGGAGATGCAGATT	259	A	A	A	NA
PMS4509	Os_Chr08	24481450	24481506	(CTT)19	(CTT)19	(CTT)19	(CTT)19	(CTT)15	(CTT)19	(CTT)19	(CTT)18	(CTT)19	(CTT)10	(CTT)19	-	Introns/Intergenic	-	-	CTCATCTCCGCCCTTGATTCC	GCCCATCAACCTCGTCTTCACC	191	P	A	A	RM80
PMS4510	Os_Chr08	24533846	24533860	(GGC)5	(GGC)5	(GGC)5	(GGC)5	(GGC)5	(GGC)5	(GGC)5	(GGC)5	(GGC)5	(GGC)6	(GGC)5	LOC_Os08g38810	CDS	RAFTIN1	CODON_INSERTION	GGATCTGATTCTCCGACGAC	TGTAGTCGTACCCGAAAGGC	253	A	A	P	NA

Polymorphic SSR (PMS) markers	Chromosomes	Start physical positions (bp)	End physical positions (bp)	Nipponbare	Kasalath	Nagina22	IR64	Pokkali	Bala	Tainung67	Azucena	Moroberekan	*Oryza rufipogon*	*Oryza nivara*	MSU gene locus IDs	Structural annotation	Functional annotation	SSR functional effects	Forward primers (5'-3')	Reverse primers (5'-3')	Amplified product size (bp)	Markers exhibiting polymorphism within *indica* rice	Transcrption factor genes-derived markers	Known cloned genes-derived markers	PMS markers corresponding with RM markers
PMS4511	Os_Chr08	24558416	24558429	(CT)7	(CT)7	(CT)7	(CT)8	(CT)8	(CT)7	(CT)7	(CT)7	(CT)7	(CT)7	(CT)8	-	Introns/Intergenic	-	-	CCCCTCACCTACTTCTGCAA	CACCACTTTCCAAATCTCGG	209	P	A	A	NA
PMS4512	Os_Chr08	24592610	24592627	(GAG)6	(GAG)6	(GAG)6	(GAG)6	(GAG)6	(GAG)6	(GAG)6	(GAG)6	(GAG)6	(GAG)6	(GAG)5	-	Introns/Intergenic	-	-	CGCGTCACCGAAGAAGTT	CCCATCTCTGCCATTACACA	251	A	A	A	NA
PMS4513	Os_Chr08	24621729	24621743	(GCG)5	(GCG)5	(GCG)5	(GCG)5	(GCG)5	(GCG)5	(GCG)5	(GCG)7	(GCG)5	(GCG)5	(GCG)5	LOC_Os08g38960	CDS	expressed protein	CODON_INSERTION	CAGGATCTTCTCCAAGACGC	AAGTTCGGGACGACCTCAC	183	A	A	A	NA
PMS4514	Os_Chr08	24627501	24627521	(CAT)7	(CAT)7	(CAT)7	(CAT)7	(CAT)7	(CAT)7	(CAT)7	(CAT)7	(CAT)7	(CAT)7	(CAT)6	-	Introns/Intergenic	-	-	GAAGCAAAGCTGTTGTCAGTTGG	GGGTGCAAAGAGCAGTGTAAGG	100	A	A	A	RM23371
PMS4515	Os_Chr08	24666716	24666733	(GGC)6	(GGC)6	(GGC)6	(GGC)6	(GGC)6	(GGC)6	(GGC)6	(GGC)6	(GGC)6	(GGC)5	(GGC)6	LOC_Os08g39030	CDS	expressed protein	CODON_CHANGE_PLUS_CODON_DELETION	TGTTCCACCTGTCGATGAAG	ACACCCACACCCTCTCTGTC	124	A	A	A	NA
PMS4516	Os_Chr08	24673188	24673208	(GCC)7	(GCC)7	(GCC)7	(GCC)7	(GCC)7	(GCC)7	(GCC)7	(GCC)7	(GCC)7	(GCC)5	(GCC)7	LOC_Os08g39050	URR	pentatricopeptide, putative, expressed	-	CTCTCCTCCTCACCTACGCATCC	GAAGTCCGGCTGGGAGTAGTGC	289	A	A	A	RM23372
PMS4517	Os_Chr08	24724155	24724174	(TA)10	(TA)10	(TA)10	(TA)10	(TA)10	(TA)10	(TA)8	(TA)10	(TA)10	(TA)7	(TA)9	-	Introns/Intergenic	-	-	GGAAGCCTTTCCTCGTAACACG	GAACCTAGGCCGTGTTCTTTGC	233	A	A	A	RM149,OSR7
PMS4518	Os_Chr08	24863050	24863085	(AT)18	(AT)10	(AT)18	(AT)18	(AT)18	(AT)18	(AT)18	(AT)18	(AT)18	(AT)18	(AT)18	-	Introns/Intergenic	-	-	GGTGGTTGCATCCCTTTAGACC	CTGGTTAGCGCTATGTTTCTTGC	199	P	A	A	RM23390
PMS4519	Os_Chr08	24905611	24905631	(CCG)7	(CCG)7	(CCG)7	(CCG)7	(CCG)5	(CCG)7	(CCG)7	(CCG)7	(CCG)7	(CCG)7	(CCG)7	LOC_Os08g39390	CDS	ZOS8-10 - C2H2 zinc finger protein, expressed	CODON_CHANGE_PLUS_CODON_DELETION	GAAGTGAAGTGAAGCAGCCAAGC	GTAGGATACAGGGCAGCGTAAGG	178	P	P	A	RM23391
PMS4520	Os_Chr08	24924656	24924721	(TA)33	(TA)33	(TA)33	(TA)11	(TA)33	(TA)33	(TA)33	(TA)33	(TA)33	(TA)12	(TA)33	-	Introns/Intergenic	-	-	TCAATTTCAACAATGGTGCC	CTCCATTTTCCGTTTGTTCG	229	P	A	A	NA
PMS4521	Os_Chr08	24927241	24927256	(AT)8	(AT)8	(AT)8	(AT)6	(AT)8	(AT)8	(AT)8	(AT)8	(AT)8	(AT)8	(AT)8	-	Introns/Intergenic	-	-	TGTCCATACCATCTAAAACACCA	ATGAATCGATTGAAATGGGG	133	P	A	A	NA
PMS4522	Os_Chr08	24935856	24935873	(CGG)6	(CGG)6	(CGG)6	(CGG)6	(CGG)6	(CGG)6	(CGG)5	(CGG)6	(CGG)6	(CGG)5	(CGG)6	LOC_Os08g39430	CDS	thylakoid lumenal 19 kDa protein, chloroplast precursor, putative, expressed	CODON_CHANGE_PLUS_CODON_DELETION	CTCTCCCCTTCCCCTCTTC	ACCTCGTACACGTACTCCGC	266	A	A	A	NA
PMS4523	Os_Chr08	24938769	24938798	(CGC)10	(CGC)10	(CGC)6	(CGC)10	(CGC)10	(CGC)10	(CGC)8	(CGC)10	(CGC)10	(CGC)10	(CGC)10	LOC_Os08g39440	URR	RNA recognition motif containing protein, putative, expressed	-	CAATAAATCTCGCCCTCGTTGC	GTGAGGTCGGCCTTGAAGTACC	333	P	A	A	RM23397

Polymorphic SSR (PMS) markers	Chromosomes	Start physical positions (bp)	End physical positions (bp)	Nipponbare	Kasalath	Nagina22	IR64	Pokkali	Bala	Tainung67	Azucena	Moroberekan	*Oryza rufipogon*	*Oryza nivara*	MSU gene locus IDs	Structural annotation	Functional annotation	SSR functional effects	Forward primers (5'-3')	Reverse primers (5'-3')	Amplified product size (bp)	Markers exhibiting polymorphism within *indica* rice	Transcrption factor genes-derived markers	Known cloned genes-derived markers	PMS markers corresponding with RM markers
PMS4524	Os_Chr08	24964988	24964999	(TA)6	(TA)6	(TA)6	(TA)6	(TA)6	(TA)6	(TA)6	(TA)6	(TA)6	(TA)6	(TA)8	-	Introns/Intergenic	-	-	TCATCTGATGGGAGCTTGAA	CTAGGCTCAAGAAGAACGCC	280	A	A	A	NA
PMS4525	Os_Chr08	25082435	25082528	(TA)47	(TA)47	(TA)47	(TA)47	(TA)47	(TA)7	(TA)47	(TA)47	(TA)7	(TA)47	(TA)13	-	Introns/Intergenic	-	-	CCATCTCAACTCCTTCGTTTACTGC	TCGACTGTTTGCTTGGAATAGGC	275	P	A	A	RM23408
PMS4526	Os_Chr08	25106749	25106766	(AG)9	(AG)15	(AG)9	(AG)9	(AG)9	(AG)9	(AG)9	(AG)9	(AG)9	(AG)9	(AG)9	-	Introns/Intergenic	-	-	CTCTGTGTATCGGCCCATCT	TCACGATTCACACAAGCACA	223	P	A	A	NA
PMS4527	Os_Chr08	25130373	25130420	(ATT)16	(ATT)16	(ATT)16	(ATT)16	(ATT)16	(ATT)16	(ATT)16	(ATT)16	(ATT)8	(ATT)16	(ATT)16	-	Introns/Intergenic	-	-	GGCCCCACCATTTTTAGATT	GTACTACCCGTCCCCCAACT	258	A	A	A	NA
PMS4528	Os_Chr08	25283150	25283167	(GA)9	(GA)7	(GA)7	(GA)9	(GA)9	(GA)7	(GA)9	(GA)9	(GA)9	(GA)9	(GA)9	-	Introns/Intergenic	-	-	CAGCGAGAGAGGAGAGGAGA	AATTTCTTCAGAGAGCCCCC	228	P	A	A	NA
PMS4529	Os_Chr08	25353695	25353715	(GCC)7	(GCC)7	(GCC)7	(GCC)7	(GCC)6	(GCC)7	(GCC)7	(GCC)7	(GCC)7	(GCC)7	(GCC)7	LOC_Os08g40030	CDS	CUP-SHAPED COTYLEDON3, putative, expressed	CODON_DELETION	GCGAGGTAGAAGGTGACGAG	CTTGCAGTGCAGAGATCGAG	232	P	P	A	NA
PMS4530	Os_Chr08	25451013	25451027	(CGG)5	(CGG)5	(CGG)5	(CGG)5	(CGG)5	(CGG)7	(CGG)5	(CGG)5	(CGG)5	(CGG)5	(CGG)7	LOC_Os08g40240	CDS	calvin cycle protein CP12, putative, expressed	CODON_INSERTION	TACGCACGCTCACTTCTCAC	CCCCTTGTACCTCCACTTCA	199	P	A	A	NA
PMS4531	Os_Chr08	25552117	25552176	(AT)30	(AT)30	(AT)24	(AT)17	(AT)8	(AT)30	(AT)7	(AT)30	(AT)30	(AT)30	(AT)30	-	Introns/Intergenic	-	-	TGCAAATAGTGCTTCACTGACC	CAGCATGCATAGAGAAGAGTTGG	288	P	A	A	RM23439
PMS4532	Os_Chr08	25596698	25596718	(CGC)7	(CGC)7	(CGC)7	(CGC)7	(CGC)7	(CGC)7	(CGC)5	(CGC)7	(CGC)7	(CGC)5	(CGC)7	LOC_Os08g40450	CDS	uvrB/uvrC motif family protein, expressed	CODON_DELETION	AGATCGGAGAACAGGTCCTCATCC	TTCCCACTCCACCCTCTTCTCG	295	A	A	A	RM23441
PMS4533	Os_Chr08	25774845	25774908	(TA)32	(TA)32	(TA)32	(TA)8	(TA)32	(TA)32	(TA)32	(TA)32	(TA)32	(TA)10	(TA)32	-	Introns/Intergenic	-	-	AATCATCTCAGAGCATTATGAA	CGTTTTACAATCCGATTACAC	384	P	A	A	NA
PMS4534	Os_Chr08	25829016	25829039	(TC)12	(TC)12	(TC)12	(TC)12	(TC)12	(TC)12	(TC)12	(TC)7	(TC)12	(TC)12	(TC)12	-	Introns/Intergenic	-	-	TTCCTTCCCAGTGATTTCTCTCC	CCCTTTCTCCTCCTTTGGTATGC	193	A	A	A	RM419
PMS4535	Os_Chr08	25888514	25888534	(TCC)7	(TCC)7	(TCC)7	(TCC)7	(TCC)7	(TCC)7	(TCC)7	(TCC)7	(TCC)8	(TCC)7	(TCC)7	LOC_Os08g40910	CDS	expressed protein	CODON_INSERTION	CCCAATTTCACCACCACATCACC	GGCGCCGAAGAAGAAGAAGAGG	322	A	A	A	RM23458
PMS4536	Os_Chr08	25979846	25979863	(GTC)6	(GTC)6	(GTC)5	(GTC)6	(GTC)5	(GTC)5	(GTC)6	(GTC)6	(GTC)6	(GTC)6	(GTC)5	-	Introns/Intergenic	-	-	CTTAGTTTGAGCTCGGTCGG	TTCTTATCCGGATGGACCTG	231	P	A	A	NA

Polymorphic SSR (PMS) markers	Chromosomes	Start physical positions (bp)	End physical positions (bp)	Nipponbare	Kasalath	Nagina22	IR64	Pokkali	Bala	Tainung67	Azucena	Moroberekan	*Oryza rufipogon*	*Oryza nivara*	MSU gene locus IDs	Structural annotation	Functional annotation	SSR functional effects	Forward primers (5'-3')	Reverse primers (5'-3')	Amplified product size (bp)	Markers exhibiting polymorphism within *indica* rice	Transcrption factor genes-derived markers	Known cloned genes-derived markers	PMS markers corresponding with RM markers
PMS4537	Os_Chr08	25984545	25984562	(CGA)6	(CGA)6	(CGA)6	(CGA)6	(CGA)6	(CGA)6	(CGA)6	(CGA)6	(CGA)6	(CGA)5	(CGA)6	LOC_Os08g41120	CDS	MBTB29 - Bric-a-Brac, Tramtrack, Broad Complex BTB domain with Meprin and TRAF Homology MATH domain, expressed	CODON_DELETION	TGTGCACACATCAG CTTCAA	GGCCACGTTCAAAG CTCTAC	159	A	A	A	NA
PMS4538	Os_Chr08	26062474	26062491	(CGA)6	(CGA)5	(CGA)5	(CGA)5	(CGA)5	(CGA)5	(CGA)6	(CGA)6	(CGA)6	(CGA)6	(CGA)5	LOC_Os08g41270	URR	endonuclease/exonucl ease/phosphatase family domain containing protein, expressed	-	ACCCATTTAAACCC CACCTC	CGAGTCCATCCTAAA TCCGA	165	A	A	A	NA
PMS4539	Os_Chr08	26088157	26088196	(AT)20	(AT)18	(AT)20	(AT)18	(AT)20	(AT)20	(AT)20	(AT)20	(AT)20	(AT)20	(AT)20	-	Introns/Intergenic	-	-	GTTGGGAGGGAGT GTAAATGC	ACTACCTCCGTTTCA GGTTATAGG	248	P	A	A	RM4487
PMS4540	Os_Chr08	26148230	26148277	(AG)24	(AG)24	(AG)24	(AG)24	(AG)24	(AG)24	(AG)24	(AG)18	(AG)24	(AG)24	(AG)24	-	Introns/Intergenic	-	-	GCGGTAACAAACCA ACCAACC	AAAGCAGGACACAG TCACACAGG	170	A	A	A	RM5493
PMS4541	Os_Chr08	26211804	26211821	(AGC)6	(AGC)6	(AGC)6	(AGC)6	(AGC)6	(AGC)6	(AGC)6	(AGC)6	(AGC)6	(AGC)5	(AGC)6	-	Introns/Intergenic	-	-	TCCAGTCATCAGTC GTCGTC	GATCCATTCACTCCG TTGCT	211	A	A	A	NA
PMS4542	Os_Chr08	26244319	26244342	(ACC)8	(ACC)8	(ACC)8	(ACC)8	(ACC)8	(ACC)8	(ACC)8	(ACC)5	(ACC)8	(ACC)8	(ACC)8	-	Introns/Intergenic	-	-	CGTACTCAGGCTAG GGCAAACC	CGTCTTCCCGGTGA GATTTCC	258	A	A	A	RM23473
PMS4543	Os_Chr08	26250157	26250174	(CTG)6	(CTG)6	(CTG)7	(CTG)6	(CTG)6	(CTG)6	(CTG)6	(CTG)6	(CTG)6	(CTG)6	(CTG)7	-	Introns/Intergenic	-	-	CTTCTCCTCTTCCAT CACGC	CCGTACAATTCATGG AACCC	217	P	A	A	NA
PMS4544	Os_Chr08	26250891	26250914	(GA)12	(GA)12	(GA)12	(GA)12	(GA)7	(GA)12	(GA)12	(GA)12	(GA)12	(GA)12	(GA)12	LOC_Os08g41550	URR	ubiquitin carboxyl-terminal hydrolase, putative, expressed	-	ATTTGGGGAAGAAA TGGAGG	TTACGGCCCATTTAC CAGTC	275	P	A	A	NA
PMS4545	Os_Chr08	26313668	26313682	(CAG)5	(CAG)5	(CAG)5	(CAG)5	(CAG)5	(CAG)5	(CAG)5	(CAG)5	(CAG)5	(CAG)6	(CAG)5	LOC_Os08g41680	URR	expressed protein	-	AAGAGGATCTCGAA CTCGGC	ATCCATCCTGGTGAG CACAT	270	A	A	A	NA
PMS4546	Os_Chr08	26322440	26322463	(GAG)8	(GAG)8	(GAG)8	(GAG)5	(GAG)8	(GAG)8	(GAG)8	(GAG)8	(GAG)8	(GAG)8	(GAG)8	LOC_Os08g41700	URR	expressed protein	-	CTCCCAGTCACCCT GCTACTCC	TGTGTCACGTTCGTT GCTACTCC	138	P	A	A	RM6070
PMS4547	Os_Chr08	26342313	26342333	(AAG)7	(AAG)7	(AAG)5	(AAG)7	(AAG)7	(AAG)6	(AAG)7	(AAG)7	(AAG)7	(AAG)5	(AAG)7	LOC_Os08g41730	URR	peptidase, T1 family, putative, expressed	-	ACCGAGCCTCTCTT AGCTTCTTCC	GCTCCCTCACCAACC TCAAACC	271	P	A	A	RM23481
PMS4548	Os_Chr08	26384370	26384390	(CGC)7	(CGC)7	(CGC)7	(CGC)7	(CGC)7	(CGC)7	(CGC)7	(CGC)7	(CGC)6	(CGC)7	(CGC)5	LOC_Os08g41780	CDS	triacylglycerol lipase precursor, putative, expressed	CODON_DELETION	GATTGATTAACCTC GTGCTCTTCG	CTGATCAACCACGCT GAACTCC	131	A	A	A	RM23486
PMS4549	Os_Chr08	26423945	26423962	(CGC)6	(CGC)6	(CGC)6	(CGC)6	(CGC)6	(CGC)6	(CGC)6	(CGC)6	(CGC)6	(CGC)6	(CGC)5	LOC_Os08g41840	CDS	expressed protein	CODON_DELETION	GCAGGATGTACTCC ACCGAC	ATCTGCTGATAGACC GTGGG	264	A	A	A	NA

Polymorphic SSR (PMS) markers	Chromosomes	Start physical positions (bp)	End physical positions (bp)	Nipponbare	Kasalath	Nagina22	IR64	Pokkali	Bala	Tainung67	Azucena	Moroberekan	*Oryza rufipogon*	*Oryza nivara*	MSU gene locus IDs	Structural annotation	Functional annotation	SSR functional effects	Forward primers (5'-3')	Reverse primers (5'-3')	Amplified product size (bp)	Markers exhibiting polymorphism within *indica* rice	Transcrption factor genes-derived markers	Known cloned genes-derived markers	PMS markers corresponding with RM markers
PMS4550	Os_Chr08	26439903	26439924	(AG)11	(AG)11	(AG)10	(AG)10	(AG)11	(AG)10	(AG)11	(AG)11	(AG)10	(AG)11	(AG)11	-	Introns/Intergenic	-	-	GGAAGCAGGAGAG GTAGTTAGATTGC	CGTGATCTGAGTGCA AGAATTTCG	397	P	A	A	RM23491
PMS4551	Os_Chr08	26442292	26442313	(TC)11	(TC)11	(TC)11	(TC)11	(TC)11	(TC)9	(TC)11	(TC)11	(TC)11	(TC)11	(TC)11	-	Introns/Intergenic	-	-	TACGCACGCACGCA TATTTA	GCTAATCTAGTTGGC GTGGC	131	P	A	A	NA
PMS4552	Os_Chr08	26515020	26515037	(AG)9	(AG)9	(AG)9	(AG)9	(AG)8	(AG)9	(AG)9	(AG)9	(AG)9	(AG)9	(AG)9	-	Introns/Intergenic	-	-	CGACAGTGATAGAG CGGTGA	TCTCCGTTCTGACGA GGAGT	234	P	A	A	NA
PMS4553	Os_Chr08	26519035	26519055	(CAG)7	(CAG)7	(CAG)7	(CAG)7	(CAG)7	(CAG)7	(CAG)7	(CAG)7	(CAG)7	(CAG)7	(CAG)6	LOC_Os08g41960	URR	OsMADS37 - MADS-box family gene with MIKC* type-box, expressed	-	ATGTACAATCCGAG TGCTCCTAGTGG	GGGCACCATCTCCAT CTCTCC	179	A	A	A	RM23497
PMS4554	Os_Chr08	26648568	26648587	(GA)10	(GA)10	(GA)10	(GA)10	(GA)10	(GA)10	(GA)10	(GA)10	(GA)10	(GA)7	(GA)10	-	Introns/Intergenic	-	-	AGGAGATGTCGAAG ATCGAGTGG	ATTGTGGGCCCTACT GACATGC	275	A	A	A	RM23507
PMS4555	Os_Chr08	26728501	26728524	(GAT)8	(GAT)5	(GAT)8	(GAT)8	(GAT)8	(GAT)6	(GAT)8	(GAT)8	(GAT)8	(GAT)8	(GAT)8	-	Introns/Intergenic	-	-	CCCTGCTGCACTGT GTGAACTGC	CCTCTCCGGTGTTGC AGGTAAAGC	286	P	A	A	RM23511
PMS4556	Os_Chr08	26748033	26748054	(GA)11	(GA)11	(GA)11	(GA)11	(GA)11	(GA)17	(GA)11	(GA)11	(GA)11	(GA)11	(GA)11	-	Introns/Intergenic	-	-	TCAGATAGCACTCC CATCTCACC	AGCCTAGGGTTTACC AGGTTTGG	164	P	A	A	RM23513
PMS4557	Os_Chr08	26767245	26767274	(TCG)10	(TCG)11	(TCG)10	(TCG)10	(TCG)10	(TCG)10	(TCG)10	(TCG)10	(TCG)10	(TCG)10	(TCG)10	LOC_Os08g42400	URR	no apical meristem protein, putative, expressed	-	CTTCTTGGCTGTGT GACTGAGTAGC	GCAACACCACTCCTC CATGC	146	P	P	A	RM23515
PMS4558	Os_Chr08	26767798	26767815	(CGG)6	(CGG)6	(CGG)6	(CGG)6	(CGG)6	(CGG)6	(CGG)6	(CGG)6	(CGG)6	(CGG)5	(CGG)6	LOC_Os08g42400	CDS	no apical meristem protein, putative, expressed	CODON_CHANGE_P LUS_CODON_DELE TION	GTCAAGAAGGGCAT GGTGTT	TCTCACCTTCTTGTG CATCG	132	A	P	A	NA
PMS4559	Os_Chr08	26848525	26848552	(TAGC)7	(TAGC)6	(TAGC)6	(TAGC)6	(TAGC)6	(TAGC)6	(TAGC)7	(TAGC)7	(TAGC)6	(TAGC)7	(TAGC)6	-	Introns/Intergenic	-	-	TCTAGCTCTCCGTG ACCGAT	GTAGAGACGACTGA ACCGGG	244	A	A	A	NA
PMS4560	Os_Chr08	26909868	26909885	(TGC)6	(TGC)6	(TGC)6	(TGC)6	(TGC)6	(TGC)6	(TGC)6	(TGC)6	(TGC)6	(TGC)5	(TGC)6	-	Introns/Intergenic	-	-	GTGGAATCGTGTAC ATCCCC	AAATTTGAACCGAAT TCCCC	110	A	A	A	NA
PMS4561	Os_Chr08	27109527	27109562	(CT)18	(CT)15	(CT)18	(CT)18	(CT)18	(CT)14	(CT)18	(CT)14	(CT)18	(CT)18	(CT)18	-	Introns/Intergenic	-	-	TGTGGAGTGTGGAG GCGAGAGC	AGGCACCCACCCAC TGACTTCC	199	P	A	A	RM3761
PMS4562	Os_Chr08	27232208	27232219	(AC)6	(AC)7	(AC)7	(AC)8	(AC)6	(AC)7	(AC)6	(AC)6	(AC)6	(AC)6	(AC)6	-	Introns/Intergenic	-	-	CGCTATTCTCATCA ATACACGG	ATTTGCGAAGATGAA TTGGC	277	P	A	A	NA

Polymorphic SSR (PMS) markers	Chromosomes	Start physical positions (bp)	End physical positions (bp)	Nipponbare	Kasalath	Nagina22	IR64	Pokkali	Bala	Tainung67	Azucena	Moroberekan	*Oryza rufipogon*	*Oryza nivara*	MSU gene locus IDs	Structural annotation	Functional annotation	SSR functional effects	Forward primers (5'-3')	Reverse primers (5'-3')	Amplified product size (bp)	Markers exhibiting polymorphism within *indica* rice	Transcrption factor genes-derived markers	Known cloned genes-derived markers	PMS markers corresponding with RM markers
PMS4563	Os_Chr08	27233855	27233886	(TA)16	(TA)16	(TA)16	(TA)16	(TA)12	(TA)16	(TA)16	(TA)16	(TA)10	(TA)16	(TA)16	-	Introns/Intergenic	-	-	TGAACAAGCCCGTAACTTCA	GGCACCATAGAGCACCAAAC	170	P	A	A	NA
PMS4564	Os_Chr08	27254023	27254040	(AT)9	(AT)8	(AT)9	(AT)9	(AT)9	(AT)9	(AT)9	(AT)9	(AT)9	(AT)8	(AT)8	-	Introns/Intergenic	-	-	TCAAGAAAACACGGGTGACA	GGCAAGAAAGTCAGCTGAAAC	231	P	A	A	NA
PMS4565	Os_Chr08	27327733	27327746	(AT)7	(AT)7	(AT)6	(AT)7	(AT)7	(AT)7	(AT)7	(AT)7	(AT)7	(AT)7	(AT)7	-	Introns/Intergenic	-	-	AGGCTCCGATTGAATCACAC	CATTTCCGATCGTTGGATCT	171	P	A	A	NA
PMS4566	Os_Chr08	27341060	27341077	(TGC)6	(TGC)6	(TGC)6	(TGC)6	(TGC)6	(TGC)6	(TGC)6	(TGC)6	(TGC)6	(TGC)5	(TGC)6	-	Introns/Intergenic	-	-	CCCAACCAAAATCCAATCTG	ATGTAAGAAACGGGTGGACG	138	A	A	A	NA
PMS4567	Os_Chr08	27355166	27355189	(ACT)8	(ACT)8	(ACT)8	(ACT)8	(ACT)6	(ACT)8	(ACT)8	(ACT)8	(ACT)8	(ACT)8	(ACT)6	-	Introns/Intergenic	-	-	CATGCTACAAATCTTGGCTTGC	CTGACAGATCTTCTGCCATCAGC	97	P	A	A	RM23564
PMS4568	Os_Chr08	27363482	27363507	(GA)13	(GA)13	(GA)13	(GA)13	(GA)12	(GA)13	(GA)13	(GA)13	(GA)13	(GA)13	(GA)12	-	Introns/Intergenic	-	-	ACGAGATCGAGTAGGGGGAC	CCCGCATATCATCCTCTCTC	245	P	A	A	NA
PMS4569	Os_Chr08	27378366	27378385	(TC)10	(TC)10	(TC)10	(TC)10	(TC)10	(TC)9	(TC)10	(TC)10	(TC)10	(TC)10	(TC)10	-	Introns/Intergenic	-	-	AAAGATCCAAGTAGTCGCCATCG	TTTCCGCTCCAGGTGTCTAGG	75	P	A	A	RM23569
PMS4570	Os_Chr08	27423440	27423454	(GCC)5	(GCC)5	(GCC)5	(GCC)5	(GCC)5	(GCC)5	(GCC)5	(GCC)5	(GCC)5	(GCC)6	(GCC)5	-	Introns/Intergenic	-	-	TCTTCTCCCGCGATATTTGT	CCATCTGAAATCGACAGCAA	273	A	A	A	NA
PMS4571	Os_Chr08	27574702	27574715	(TA)7	(TA)7	(TA)7	(TA)6	(TA)7	(TA)7	(TA)7	(TA)7	(TA)7	(TA)9	(TA)7	-	Introns/Intergenic	-	-	TTAACACTCCCTCCATTCCG	TCAATGTTATTGGGGTTGGC	231	P	A	A	NA
PMS4572	Os_Chr08	27739536	27739550	(GAC)5	(GAC)5	(GAC)5	(GAC)6	(GAC)6	(GAC)5	(GAC)5	(GAC)5	(GAC)5	(GAC)5	(GAC)5	-	Introns/Intergenic	-	-	CTCCTCGTCTTCTTGCTGCT	ATCTCCCTCCTCTCCTCTCG	240	P	A	A	NA
PMS4573	Os_Chr08	27780874	27780885	(TA)6	(TA)8	(TA)6	(TA)6	(TA)6	(TA)6	(TA)6	(TA)6	(TA)6	(TA)6	(TA)6	-	Introns/Intergenic	-	-	CTGATCCGAAGGTTAAAGCG	TCTCATGCTGCATCGGTTAG	260	P	A	A	NA
PMS4574	Os_Chr08	27849003	27849020	(GGA)6	(GGA)6	(GGA)6	(GGA)6	(GGA)6	(GGA)6	(GGA)6	(GGA)6	(GGA)6	(GGA)5	(GGA)6	-	Introns/Intergenic	-	-	GAAGAGAGGATCCAACGTGC	CACCTCCACCTCCATCCC	243	A	A	A	NA
PMS4575	Os_Chr08	27926558	27926603	(CT)23	(CT)19	(CT)23	(CT)23	(CT)23	(CT)23	(CT)23	(CT)23	(CT)23	(CT)23	(CT)23	LOC_Os08g44370	URR	inosine-uridine preferring nucleoside hydrolase family protein, putative, expressed	-	GAGGAAGAGGAAGGGACTCACC	TTGCGTCCTACTGCTACTTCTTCC	197	P	A	A	RM3840

Polymorphic SSR (PMS) markers	Chromosomes	Start physical positions (bp)	End physical positions (bp)	Nipponbare	Kasalath	Nagina22	IR64	Pokkali	Bala	Tainung67	Azucena	Moroberekan	*Oryza rufipogon*	*Oryza nivara*	MSU gene locus IDs	Structural annotation	Functional annotation	SSR functional effects	Forward primers (5'-3')	Reverse primers (5'-3')	Amplified product size (bp)	Markers exhibiting polymorphism within *indica* rice	Transcrption factor genes-derived markers	Known cloned genes-derived markers	PMS markers corresponding with RM markers
PMS4576	Os_Chr08	27958698	27958715	(GAA)6	(GAA)6	(GAA)6	(GAA)6	(GAA)6	(GAA)6	(GAA)6	(GAA)6	(GAA)6	(GAA)6	(GAA)5	-	Introns/Intergenic	-	-	GGCATCAGAGTCGGAGGTTA	CAGAGCCCTCTCTCTCCCTT	169	A	A	A	NA
PMS4577	Os_Chr08	28005011	28005028	(GCC)6	(GCC)6	(GCC)6	(GCC)6	(GCC)6	(GCC)6	(GCC)6	(GCC)5	(GCC)6	(GCC)6	(GCC)6	LOC_Os08g44530	CDS	dihydroxy-acid dehydratase, putative, expressed	CODON_CHANGE_PLUS_CODON_DELETION	TAAACCTCCCTCCCATTTCC	TCGTACCAGACGGAGGAGAC	277	A	A	A	NA
PMS4578	Os_Chr08	28070247	28070258	(GA)6	(GA)9	(GA)6	(GA)6	(GA)6	(GA)9	(GA)6	(GA)6	(GA)6	(GA)6	(GA)9	-	Introns/Intergenic	-	-	CGACCTCCTCTTCGTCGTAA	CTGCGGTAGAGTTGGGAGAG	279	P	A	A	NA
PMS4579	Os_Chr08	28111381	28111400	(CT)10	(CT)9	(CT)9	(CT)9	(CT)9	(CT)9	(CT)10	(CT)10	(CT)10	(CT)10	(CT)9	-	Introns/Intergenic	-	-	TATGCTCGATAGCGGCCCAAACC	GCGAGAAACGGGCTCACATGG	73	A	A	A	RM23620
PMS4580	Os_Chr08	28166746	28166763	(TA)9	(TA)9	(TA)9	(TA)9	(TA)9	(TA)10	(TA)9	(TA)9	(TA)9	(TA)9	(TA)9	-	Introns/Intergenic	-	-	CATGCGTACCCAGTACATGC	GCAATCACTCCATACGTCCA	280	P	A	A	NA
PMS4581	Os_Chr08	28274073	28274086	(AT)7	(AT)7	(AT)7	(AT)7	(AT)7	(AT)7	(AT)7	(AT)7	(AT)7	(AT)8	(AT)7	-	Introns/Intergenic	-	-	CAGCGTGCAAACTACGAGAA	ACTGCCTCCACTGCGAGTAT	182	A	A	A	NA
PMS4582	Os_Chr08	28283759	28283776	(TC)9	(TC)7	(TC)7	(TC)9	(TC)9	(TC)7	(TC)9	(TC)9	(TC)9	(TC)7	(TC)9	-	Introns/Intergenic	-	-	TAGAAAACCGAGTTGAGCCG	CGAGAAGTGAGAGAGCCGAG	155	P	A	A	NA
PMS4583	Os_Chr09	83005	83022	(CT)9	(CT)7	(CT)9	(CT)9	(CT)9	(CT)9	(CT)9	(CT)9	(CT)9	(CT)9	(CT)9	-	Introns/Intergenic	-	-	AAAAAGTCCTCTTTTCCCCC	GAGGAGGGAGATGACAGGTG	171	P	A	A	NA
PMS4584	Os_Chr09	175433	175446	(AG)7	(AG)6	(AG)7	(AG)7	(AG)7	(AG)6	(AG)6	(AG)7	(AG)6	(AG)7	(AG)7	-	Introns/Intergenic	-	-	GAGGAAGGCGATGACAGGT	GCCCTCCAATTTTAGTGGAA	265	P	A	A	NA
PMS4585	Os_Chr09	182617	182628	(AG)6	(AG)7	(AG)7	(AG)6	(AG)7	(AG)6	(AG)7	(AG)6	(AG)7	(AG)6	(AG)6	-	Introns/Intergenic	-	-	GAGGAAGGCGATGACAGGT	CCTTGGTCTAAAAGGAGCCC	279	P	A	A	NA
PMS4586	Os_Chr09	216688	216701	(CT)7	(CT)6	(CT)6	(CT)6	(CT)6	(CT)6	(CT)7	(CT)6	(CT)6	(CT)6	(CT)6	-	Introns/Intergenic	-	-	AGTGGAATTTTCAGAGCACAAA	GAGGAAGGCGATGACAGGT	250	A	A	A	NA
PMS4587	Os_Chr09	225617	225628	(CT)6	(CT)7	(CT)6	(CT)7	(CT)6	(CT)7	(CT)7	(CT)7	(CT)7	(CT)7	(CT)7	-	Introns/Intergenic	-	-	TCCTAAAAGCCTAAAATGCG	GAGGAAGGCGATGACAGGT	280	P	A	A	NA
PMS4588	Os_Chr09	267202	267219	(TCC)6	(TCC)10	(TCC)10	(TCC)10	(TCC)10	(TCC)10	(TCC)6	(TCC)6	(TCC)6	(TCC)6	(TCC)10	LOC_Os09g01320	CDS	expressed protein	CODON_INSERTION	TCCACTTCCAGTCACAACCA	GCAGCAATATAGGCAGGAGC	184	A	A	A	NA

Polymorphic SSR (PMS) markers	Chromosomes	Start physical positions (bp)	End physical positions (bp)	Nipponbare	Kasalath	Nagina22	IR64	Pokkali	Bala	Tainung67	Azucena	Moroberekan	*Oryza rufipogon*	*Oryza nivara*	MSU gene locus IDs	Structural annotation	Functional annotation	SSR functional effects	Forward primers (5'-3')	Reverse primers (5'-3')	Amplified product size (bp)	Markers exhibiting polymorphism within *indica* rice	Transcrption factor genes-derived markers	Known cloned genes-derived markers	PMS markers corresponding with RM markers
PMS4589	Os_Chr09	272096	272111	(AG)8	(AG)8	(AG)8	(AG)8	(AG)8	(AG)7	(AG)8	(AG)8	(AG)8	(AG)8	(AG)8	-	Introns/Intergenic	-	-	CTCCTTCACCACCTCCCTC	CCCCAGGTATTTTCTTGACG	272	P	A	A	NA
PMS4590	Os_Chr09	674378	674405	(AT)14	(AT)14	(AT)14	(AT)10	(AT)14	(AT)7	(AT)14	(AT)14	(AT)6	(AT)14	(AT)14	-	Introns/Intergenic	-	-	GATAAAGGTTTGTGGCTCCTTTGG	GACGCCGCTACTACCAGATCC	438	P	A	A	RM23669
PMS4591	Os_Chr09	699832	699843	(TG)6	(TG)6	(TG)6	(TG)7	(TG)6	(TG)6	(TG)6	(TG)6	(TG)6	(TG)7	(TG)7	-	Introns/Intergenic	-	-	CACCACCTAAAGCACATGGA	AGTGACCTTTACGCCGTGAC	277	P	A	A	NA
PMS4592	Os_Chr09	869884	869923	(AGAA)10	(AGAA)10	(AGAA)10	(AGAA)10	(AGAA)10	(AGAA)10	(AGAA)10	(AGAA)10	(AGAA)10	(AGAA)8	(AGAA)10	-	Introns/Intergenic	-	-	TCACAGCTTAGTGCATGTTGAGC	GATTCACCTGGCAATGAGAACG	171	A	A	A	RM23679
PMS4593	Os_Chr09	940429	940442	(AT)7	(AT)7	(AT)7	(AT)7	(AT)7	(AT)7	(AT)7	(AT)7	(AT)7	(AT)8	(AT)7	-	Introns/Intergenic	-	-	TTCGTCTCGCGAATTAGTCC	AAAAGACCACATGGAATGGC	224	A	A	A	NA
PMS4594	Os_Chr09	970697	970714	(CGC)6	(CGC)6	(CGC)6	(CGC)6	(CGC)6	(CGC)6	(CGC)5	(CGC)6	(CGC)6	(CGC)6	(CGC)6	LOC_Os09g02360	CDS	cyclin, putative, expressed	CODON_DELETION	CTCACCGCACACATTTTCAT	TCTACGACAAAGCCACCACA	274	A	A	A	NA
PMS4595	Os_Chr09	982675	982688	(CT)7	(CT)6	(CT)7	(CT)7	(CT)7	(CT)6	(CT)7	(CT)7	(CT)6	(CT)7	(CT)7	-	Introns/Intergenic	-	-	AATACCGGACGAATTACCCC	GTCGTCGACTGACTGGTCC	224	P	A	A	NA
PMS4596	Os_Chr09	1173589	1173626	(TA)19	(TA)19	(TA)19	(TA)8	(TA)19	(TA)19	(TA)7	(TA)7	(TA)10	(TA)19	(TA)19	-	Introns/Intergenic	-	-	CACCTTATCGGCTGGTGTAAAGG	GTGGCAACATGATGCAATACAAGG	386	P	A	A	RM4413
PMS4597	Os_Chr09	1184911	1184926	(TG)8	(TG)8	(TG)8	(TG)8	(TG)8	(TG)8	(TG)8	(TG)8	(TG)8	(TG)8	(TG)7	-	Introns/Intergenic	-	-	TGTGGGCACATTTGTTTGTT	TTCCATTGCACCAAAATTGA	164	A	A	A	NA
PMS4598	Os_Chr09	1255022	1255067	(AT)23	(AT)23	(AT)23	(AT)7	(AT)23	(AT)23	(AT)23	(AT)23	(AT)23	(AT)23	(AT)23	-	Introns/Intergenic	-	-	CTCGTAGTTTAAAGGCGGAACC	TACATTGCACCAGGTGATTACG	359	P	A	A	RM23702
PMS4599	Os_Chr09	1680830	1680847	(GA)9	(GA)9	(GA)9	(GA)9	(GA)9	(GA)9	(GA)9	(GA)9	(GA)9	(GA)9	(GA)6	-	Introns/Intergenic	-	-	GAGGACAGGGATGACAGGTG	TGGTCTAAAAGGAGCCCTCA	279	A	A	A	NA
PMS4600	Os_Chr09	1716836	1716886	(TTA)17	(TTA)17	(TTA)8	(TTA)17	(TTA)16	(TTA)17	(TTA)17	(TTA)17	(TTA)14	(TTA)17	(TTA)17	-	Introns/Intergenic	-	-	GGTGATGATGAGTGTTTGATGC	TGACAGTAGTAGTTCAGCAGTGTCC	137	P	A	A	RM5688
PMS4601	Os_Chr09	1730589	1730660	(TA)36	(TA)36	(TA)36	(TA)36	(TA)36	(TA)36	(TA)36	(TA)12	(TA)36	(TA)36	(TA)36	-	Introns/Intergenic	-	-	GCGACATGTTCACATACTTCTTCG	AGTGTTTCATTCCACACCAAGG	340	A	A	A	RM23715

Polymorphic SSR (PMS) markers	Chromosomes	Start physical positions (bp)	End physical positions (bp)	Nipponbare	Kasalath	Nagina22	IR64	Pokkali	Bala	Tainung67	Azucena	Moroberekan	*Oryza rufipogon*	*Oryza nivara*	MSU gene locus IDs	Structural annotation	Functional annotation	SSR functional effects	Forward primers (5'-3')	Reverse primers (5'-3')	Amplified product size (bp)	Markers exhibiting polymorphism within *indica* rice	Transcrption factor genes-derived markers	Known cloned genes-derived markers	PMS markers corresponding with RM markers
PMS4602	Os_Chr09	1965871	1965914	(TA)22	(TA)22	(TA)22	(TA)22	(TA)22	(TA)22	(TA)22	(TA)18	(TA)6	(TA)22	(TA)22	-	Introns/Intergenic	-	-	CACAACCCACCATTGACAAG	TCTATTCCTTGATACCTCGGG	266	A	A	A	NA
PMS4603	Os_Chr09	2006826	2006849	(CT)12	(CT)12	(CT)12	(CT)12	(CT)12	(CT)12	(CT)12	(CT)12	(CT)12	(CT)12	(CT)10	-	Introns/Intergenic	-	-	GTTGGTAACGGGACGAAAGA	AAGAGTGGAGGGGAGGAAGA	263	A	A	A	NA
PMS4604	Os_Chr09	2043917	2043934	(GA)9	(GA)9	(GA)9	(GA)9	(GA)9	(GA)9	(GA)9	(GA)9	(GA)10	(GA)9	(GA)9	LOC_Os09g03980	CDS	retrotransposon protein, putative, unclassified, expressed	FRAME_SHIFT	TTATTGGGGCTCGAGAGAGA	GTCAGCCCTTTCCCTAACCT	273	A	A	A	NA
PMS4605	Os_Chr09	2068489	2068512	(TA)12	(TA)12	(TA)12	(TA)12	(TA)12	(TA)12	(TA)6	(TA)12	(TA)12	(TA)11	(TA)12	-	Introns/Intergenic	-	-	CTCACAACTGAGAAATAGCCTACTGG	TTCTCACGTGTACAAATCCTCTCC	614	A	A	A	RM23720
PMS4606	Os_Chr09	2117781	2117810	(TA)15	(TA)15	(TA)15	(TA)15	(TA)8	(TA)15	(TA)15	(TA)15	(TA)15	(TA)14	(TA)15	-	Introns/Intergenic	-	-	GAGAAGCTGATGCAAGAAGACAGG	GTATTGCAAAGCACTACCGATGG	489	P	A	A	RM23722
PMS4607	Os_Chr09	2283808	2283879	(AT)36	(AT)36	(AT)36	(AT)36	(AT)36	(AT)36	(AT)36	(AT)36	(AT)12	(AT)36	(AT)6	-	Introns/Intergenic	-	-	TTCCACGTCGGAAGAAAAAC	GCCTATACTCCATGTAGCACCA	265	A	A	A	NA
PMS4608	Os_Chr09	2292802	2292815	(GT)7	(GT)7	(GT)7	(GT)7	(GT)7	(GT)6	(GT)7	(GT)7	(GT)7	(GT)7	(GT)7	-	Introns/Intergenic	-	-	TTTTTGGGTGTCCCGTAGC	CCCGACACACACTTGCTAGA	165	P	A	A	NA
PMS4609	Os_Chr09	2532417	2532470	(TA)27	(TA)27	(TA)27	(TA)27	(TA)13	(TA)14	(TA)6	(TA)27	(TA)27	(TA)11	(TA)27	-	Introns/Intergenic	-	-	GGTATGTAATCATGGATTGTGAAA	TCAATTTCAACAATGGTGCC	215	P	A	A	NA
PMS4610	Os_Chr09	2742150	2742167	(AGG)6	(AGG)5	(AGG)6	(AGG)6	(AGG)6	(AGG)5	(AGG)6	(AGG)6	(AGG)6	(AGG)6	(AGG)6	LOC_Os09g05100	CDS	retrotransposon protein, putative, Ty3-gypsy subclass, expressed	CODON_DELETION	CCGATGGAGTTCGATCACTT	CGGCAGCAACAACACTTAAA	163	P	A	A	NA
PMS4611	Os_Chr09	3184280	3184294	(CCG)5	(CCG)6	(CCG)5	(CCG)5	(CCG)5	(CCG)5	(CCG)5	(CCG)5	(CCG)5	(CCG)5	(CCG)5	-	Introns/Intergenic	-	-	ATCTAGCTGGAGGGGAGGC	AATTAGATCGAAGGGGGTCG	279	P	A	A	NA
PMS4612	Os_Chr09	3287222	3287242	(GAC)7	(GAC)7	(GAC)7	(GAC)7	(GAC)7	(GAC)7	(GAC)7	(GAC)7	(GAC)7	(GAC)5	(GAC)7	-	Introns/Intergenic	-	-	CTTGGACGAAGGCGAGAC	CTCCGTCATCATCATCGCT	274	A	A	A	NA
PMS4613	Os_Chr09	3293481	3293501	(GAC)7	(GAC)7	(GAC)7	(GAC)7	(GAC)7	(GAC)7	(GAC)7	(GAC)7	(GAC)7	(GAC)5	(GAC)7	-	Introns/Intergenic	-	-	CTTGGACGAAGGCGAGAC	CTCCGTCATCATCATCGCT	274	A	A	A	NA
PMS4614	Os_Chr09	3350582	3350599	(AT)9	(AT)10	(AT)9	(AT)9	(AT)9	(AT)9	(AT)9	(AT)9	(AT)9	(AT)9	(AT)9	-	Introns/Intergenic	-	-	TTAGAGCCCTCAAAGCCTCC	CGAACACCTAGGGGCTAATG	274	P	A	A	NA

Polymorphic SSR (PMS) markers	Chromosomes	Start physical positions (bp)	End physical positions (bp)	Nipponbare	Kasalath	Nagina22	IR64	Pokkali	Bala	Tainung67	Azucena	Moroberekan	*Oryza rufipogon*	*Oryza nivara*	MSU gene locus IDs	Structural annotation	Functional annotation	SSR functional effects	Forward primers (5'-3')	Reverse primers (5'-3')	Amplified product size (bp)	Markers exhibiting polymorphism within *indica* rice	Transcrption factor genes-derived markers	Known cloned genes-derived markers	PMS markers corresponding with RM markers
PMS4615	Os_Chr09	3749955	3749972	(CT)9	(CT)8	(CT)8	(CT)8	(CT)9	(CT)8	(CT)9	(CT)9	(CT)9	(CT)9	(CT)8	-	Introns/Intergenic	-	-	ACACCATTCCGTCGCTATTC	GTTAGCTGCTGGTGGTGGTC	198	P	A	A	NA
PMS4616	Os_Chr09	3803783	3803809	(TGC)9	(TGC)8	(TGC)8	(TGC)7	(TGC)9	(TGC)8	(TGC)9	(TGC)9	(TGC)9	(TGC)9	(TGC)7	-	Introns/Intergenic	-	-	CTTGCACAAGAGGCAACACTCC	GTTTGGTAGGTCGCATTGTTTGG	143	P	A	A	RM5799
PMS4617	Os_Chr09	3851325	3851340	(AT)8	(AT)8	(AT)8	(AT)8	(AT)8	(AT)8	(AT)8	(AT)8	(AT)8	(AT)8	(AT)7	-	Introns/Intergenic	-	-	GATGTGAGGGGAGCGTTTTA	CGTGAACAACAAAGTGGTGG	230	A	A	A	NA
PMS4618	Os_Chr09	3900356	3900373	(GGA)6	(GGA)6	(GGA)6	(GGA)6	(GGA)6	(GGA)6	(GGA)6	(GGA)6	(GGA)6	(GGA)5	(GGA)5	LOC_Os09g07730	CDS	expressed protein	CODON_DELETION	GAAGGACGGGAAAGAGAAGG	ACCCATCTGCCAGTCTGTTC	254	A	A	A	NA
PMS4619	Os_Chr09	3953321	3953334	(AT)7	(AT)7	(AT)7	(AT)6	(AT)7	(AT)7	(AT)7	(AT)7	(AT)7	(AT)7	(AT)7	-	Introns/Intergenic	-	-	GTTAGGGATTCAGTTGGATACG	GATAGCCCTCTATTCAGATTCG	416	P	A	A	RM23777
PMS4620	Os_Chr09	4354753	4354809	(ATT)19	(ATT)19	(ATT)11	(ATT)19	(ATT)19	(ATT)19	(ATT)19	(ATT)19	(ATT)19	(ATT)19	(ATT)19	-	Introns/Intergenic	-	-	GCATCTCAGCAAACAAGAACAACC	GAGGCCATCAAGCAGTTCTCC	167	P	A	A	RM23793
PMS4621	Os_Chr09	4454295	4454328	(AT)17	(AT)17	(AT)17	(AT)8	(AT)17	(AT)17	(AT)17	(AT)17	(AT)17	(AT)16	(AT)17	-	Introns/Intergenic	-	-	TGCGCCGGCTATAGATAGAATAGG	GCGGAAGCTAGATGGAGGAAGC	179	P	A	A	RM23796
PMS4622	Os_Chr09	4600539	4600562	(AT)12	(AT)12	(AT)12	(AT)8	(AT)12	(AT)12	(AT)12	(AT)12	(AT)12	(AT)8	(AT)12	-	Introns/Intergenic	-	-	TTGGAGCCCATACTCCATGT	ACATATAGACTCACGCGGGG	213	P	A	A	NA
PMS4623	Os_Chr09	4616243	4616258	(GA)8	(GA)8	(GA)8	(GA)6	(GA)8	(GA)8	(GA)8	(GA)8	(GA)8	(GA)8	(GA)8	-	Introns/Intergenic	-	-	GCTCGGGTAGGAGGGAGA	CTCCCGGCTCCTTTCTCT	111	P	A	A	NA
PMS4624	Os_Chr09	4820778	4820795	(CGG)6	(CGG)6	(CGG)6	(CGG)6	(CGG)6	(CGG)6	(CGG)6	(CGG)6	(CGG)6	(CGG)6	(CGG)5	-	Introns/Intergenic	-	-	AGAGGATCCCGGGGAATAAT	TCACCTCTCCCCTCTAATGG	274	A	A	A	NA
PMS4625	Os_Chr09	4829691	4829708	(CGG)6	(CGG)6	(CGG)6	(CGG)6	(CGG)6	(CGG)6	(CGG)6	(CGG)6	(CGG)6	(CGG)5	(CGG)6	-	Introns/Intergenic	-	-	AGAGGATCCCGGGGAATAAT	TCACCTCTCCCCTCTAATGG	274	A	A	A	NA
PMS4626	Os_Chr09	5009002	5009017	(TG)8	(TG)7	(TG)8	(TG)8	(TG)8	(TG)8	(TG)8	(TG)8	(TG)8	(TG)8	(TG)8	-	Introns/Intergenic	-	-	GCTTAAGGAAATCGGCTGTG	ACCTGGACAGGCAATCATTC	279	P	A	A	NA
PMS4627	Os_Chr09	5154529	5154542	(AG)7	(AG)7	(AG)7	(AG)7	(AG)7	(AG)7	(AG)7	(AG)8	(AG)8	(AG)7	(AG)8	-	Introns/Intergenic	-	-	TGCCAAGTGTCAAAGTGTGTG	CTGTGCGTATGGGGATCTTT	268	A	A	A	NA

Polymorphic SSR (PMS) markers	Chromosomes	Start physical positions (bp)	End physical positions (bp)	Nipponbare	Kasalath	Nagina22	IR64	Pokkali	Bala	Tainung67	Azucena	Moroberekan	*Oryza rufipogon*	*Oryza nivara*	MSU gene locus IDs	Structural annotation	Functional annotation	SSR functional effects	Forward primers (5'-3')	Reverse primers (5'-3')	Amplified product size (bp)	Markers exhibiting polymorphism within *indica* rice	Transcrption factor genes-derived markers	Known cloned genes-derived markers	PMS markers corresponding with RM markers
PMS4628	Os_Chr09	5308691	5308714	(GGC)8	(GGC)8	(GGC)8	(GGC)8	(GGC)8	(GGC)8	(GGC)8	(GGC)8	(GGC)8	(GGC)5	(GGC)6	LOC_Os09g09790	CDS	Fe-S metabolism associated domain containing protein, expressed	CODON_DELETION	GAGGAGCTGCTTGTACCGTGTGC	GAAGCCACCCTCTCCTCCATCC	309	A	A	A	RM6021
PMS4629	Os_Chr09	5391897	5391926	(CTC)10	(CTC)10	(CTC)10	(CTC)7	(CTC)7	(CTC)10	(CTC)10	(CTC)10	(CTC)10	(CTC)10	(CTC)10	LOC_Os09g09920	CDS	expressed protein	CODON_DELETION	AGATAGGGTTGGACGTCTTGAGG	GGAGGAGATGATCGAAGGAGAGG	392	P	A	A	RM23830
PMS4630	Os_Chr09	5414846	5414863	(AT)9	(AT)7	(AT)9	(AT)9	(AT)9	(AT)7	(AT)9	(AT)9	(AT)9	(AT)9	(AT)7	-	Introns/Intergenic	-	-	ACATACCGTGACCTTGCACA	ATGGTTTGGCCATGTTCATT	260	P	A	A	NA
PMS4631	Os_Chr09	5450121	5450144	(CGG)8	(CGG)8	(CGG)8	(CGG)5	(CGG)8	(CGG)8	(CGG)8	(CGG)8	(CGG)8	(CGG)6	(CGG)8	LOC_Os09g10000	URR	expressed protein	-	GGGCACGGACTACCAAAGGAAGC	ATCAGGCCCAGCACCCAAACC	178	P	A	A	RM6196
PMS4632	Os_Chr09	5649196	5649209	(CT)7	(CT)6	(CT)7	(CT)7	(CT)7	(CT)7	(CT)7	(CT)7	(CT)7	(CT)7	(CT)7	-	Introns/Intergenic	-	-	ATCCTCTCTTCCACCATCCC	CCGAACCTGTATACATCGCC	258	P	A	A	NA
PMS4633	Os_Chr09	5873079	5873096	(TGG)6	(TGG)6	(TGG)6	(TGG)6	(TGG)6	(TGG)6	(TGG)5	(TGG)6	(TGG)6	(TGG)5	(TGG)5	LOC_Os09g10760	CDS	RNA recognition motif containing protein, putative, expressed	CODON_DELETION	GAGGGGTTATGAGGGAGGAG	ATATCGGCCTGCATAGCATC	177	A	A	A	NA
PMS4634	Os_Chr09	5920547	5920578	(TC)16	(TC)11	(TC)16	(TC)16	(TC)16	(TC)15	(TC)16	(TC)16	(TC)16	(TC)16	(TC)16	-	Introns/Intergenic	-	-	AATCCACCTGGCCCTAATCTTCC	CACTGCTGCTTCCTTCCTTCTGC	245	P	A	A	RM8206
PMS4635	Os_Chr09	5926314	5926337	(TA)12	(TA)12	(TA)12	(TA)12	(TA)12	(TA)12	(TA)7	(TA)12	(TA)12	(TA)12	(TA)12	-	Introns/Intergenic	-	-	TGCATCTTTCACCGTAGTCCTAGC	CTTGCTGGAGCTCGTAGATGC	546	A	A	A	RM444
PMS4636	Os_Chr09	5959236	5959261	(AT)13	(AT)13	(AT)13	(AT)13	(AT)9	(AT)13	(AT)13	(AT)13	(AT)13	(AT)13	(AT)13	-	Introns/Intergenic	-	-	TCTAGCATCTCGGAGACATTGC	ATGCACGGATGTTTGAAGAAGG	516	P	A	A	RM23855
PMS4637	Os_Chr09	6022338	6022355	(AGA)6	(AGA)5	(AGA)6	(AGA)6	(AGA)6	(AGA)6	(AGA)6	(AGA)6	(AGA)6	(AGA)6	(AGA)6	LOC_Os09g10940	URR	RGP1	-	AATCGATTGCTGTTCGGTTC	CCACCTCCTCCTCTTCTCCT	118	P	A	P	NA
PMS4638	Os_Chr09	6466414	6466431	(CA)9	(CA)10	(CA)9	(CA)9	(CA)9	(CA)9	(CA)9	(CA)9	(CA)9	(CA)9	(CA)9	-	Introns/Intergenic	-	-	TGCCACATGTTGAGAGTGATGC	TACGCAAGCCATGACAATTCG	328	P	A	A	RM23878
PMS4639	Os_Chr09	6594987	6595010	(TATC)6	(TATC)6	(TATC)6	(TATC)6	(TATC)6	(TATC)5	(TATC)6	(TATC)6	(TATC)6	(TATC)6	(TATC)6	-	Introns/Intergenic	-	-	CAGTGTTGCTGCAAAAAGGA	AACATTGGTCGTGCTCAACA	220	P	A	A	NA
PMS4640	Os_Chr09	6755914	6755933	(ATAG)5	(ATAG)5	(ATAG)5	(ATAG)5	(ATAG)7	(ATAG)5	(ATAG)5	(ATAG)5	(ATAG)5	(ATAG)5	(ATAG)7	-	Introns/Intergenic	-	-	GGCGGCTTAAGAGTGTTTGTAGG	AGAGGATTGCTTTGCTGATGAGG	177	P	A	A	RM23893

Polymorphic SSR (PMS) markers	Chromosomes	Start physical positions (bp)	End physical positions (bp)	Nipponbare	Kasalath	Nagina22	IR64	Pokkali	Bala	Tainung67	Azucena	Moroberekan	*Oryza rufipogon*	*Oryza nivara*	MSU gene locus IDs	Structural annotation	Functional annotation	SSR functional effects	Forward primers (5'-3')	Reverse primers (5'-3')	Amplified product size (bp)	Markers exhibiting polymorphism within *indica* rice	Transcrption factor genes-derived markers	Known cloned genes-derived markers	PMS markers corresponding with RM markers
PMS4641	Os_Chr09	6840249	6840262	(GA)7	(GA)7	(GA)7	(GA)7	(GA)7	(GA)7	(GA)6	(GA)7	(GA)7	(GA)6	(GA)7	-	Introns/Intergenic	-	-	GGCAATGAGCTATAGGCAGC	ATCGATCTCGGCTTCTCCTT	196	A	A	A	NA
PMS4642	Os_Chr09	6845010	6845037	(AT)14	(AT)14	(AT)14	(AT)14	(AT)7	(AT)14	(AT)14	(AT)9	(AT)14	(AT)14	(AT)14	-	Introns/Intergenic	-	-	GCATGACCTCCCCTCTTTTT	TAGTCCCGGTTGGTGTTACC	134	P	A	A	NA
PMS4643	Os_Chr09	7066203	7066217	(GTC)5	(GTC)5	(GTC)5	(GTC)5	(GTC)5	(GTC)6	(GTC)5	(GTC)5	(GTC)5	(GTC)5	(GTC)5	-	Introns/Intergenic	-	-	GTCGTCTTCACCGTTCATCC	ACGAACGAGAACGGCTTG	218	P	A	A	NA
PMS4644	Os_Chr09	7092697	7092788	(AT)46	(AT)46	(AT)46	(AT)46	(AT)46	(AT)46	(AT)46	(AT)46	(AT)7	(AT)46	(AT)46	-	Introns/Intergenic	-	-	CACATATCGAGGGTGAAGAACC	AACCGGTGGACTCATTATTTCC	387	A	A	A	RM23907
PMS4645	Os_Chr09	7495748	7495765	(ATA)6	(ATA)6	(ATA)5	(ATA)5	(ATA)5	(ATA)5	(ATA)6	(ATA)6	(ATA)6	(ATA)5	(ATA)6	-	Introns/Intergenic	-	-	CTCACGTGGTAGTCCAGGCT	TATGGCCAAGAAACGACCAT	159	P	A	A	NA
PMS4646	Os_Chr09	7584968	7584983	(AT)8	(AT)8	(AT)8	(AT)8	(AT)8	(AT)8	(AT)8	(AT)6	(AT)8	(AT)8	(AT)8	-	Introns/Intergenic	-	-	TAACTTCCCACCATTCGAGC	ACCGTGCAAACTGTGTGGTA	268	A	A	A	NA
PMS4647	Os_Chr09	7700449	7700536	(AT)44	(AT)44	(AT)44	(AT)44	(AT)44	(AT)44	(AT)8	(AT)44	(AT)44	(AT)44	(AT)44	-	Introns/Intergenic	-	-	AACGATGAACATTGCCCATT	GGGCATCATAAAGCACCAAA	266	A	A	A	NA
PMS4648	Os_Chr09	7700926	7700945	(AT)10	(AT)10	(AT)10	(AT)10	(AT)10	(AT)10	(AT)9	(AT)10	(AT)10	(AT)10	(AT)11	-	Introns/Intergenic	-	-	ATTTGATACCATGATGCCCG	TATATTTGCGTGCGTCCGTA	246	A	A	A	NA
PMS4649	Os_Chr09	7721094	7721149	(TA)28	(TA)28	(TA)28	(TA)28	(TA)28	(TA)28	(TA)28	(TA)28	(TA)8	(TA)28	(TA)21	-	Introns/Intergenic	-	-	GGGCCTGTGTGCATAGGATA	AACCAACAAAGGCAATCCAG	207	A	A	A	NA
PMS4650	Os_Chr09	7723507	7723578	(TA)36	(TA)36	(TA)36	(TA)36	(TA)36	(TA)36	(TA)36	(TA)36	(TA)36	(TA)36	(TA)35	-	Introns/Intergenic	-	-	CTGTTTTTGCGTGCAGATGT	TGGGTTGTCTGAAATCTTGAAA	263	A	A	A	NA
PMS4651	Os_Chr09	7774454	7774465	(TA)6	(TA)6	(TA)6	(TA)7	(TA)6	(TA)6	(TA)6	(TA)6	(TA)6	(TA)6	(TA)6	-	Introns/Intergenic	-	-	CAGCATCTGCAACAAGGAAA	ATGCAGATGGAAAGAGTGGC	172	P	A	A	NA
PMS4652	Os_Chr09	7785306	7785319	(TA)7	(TA)7	(TA)6	(TA)7	(TA)7	(TA)7	(TA)7	(TA)7	(TA)7	(TA)7	(TA)7	-	Introns/Intergenic	-	-	GCAGCAGGGCCAGTTATTAG	GGATATGGCTATCATCCCGA	252	P	A	A	NA
PMS4653	Os_Chr09	7809547	7809588	(AT)21	(AT)21	(AT)21	(AT)10	(AT)8	(AT)21	(AT)6	(AT)21	(AT)21	(AT)21	(AT)21	-	Introns/Intergenic	-	-	AAGTGGATAAATCGTAATAAGCAAA	TCATGCATGTGGCATTTACA	277	P	A	A	NA

Polymorphic SSR (PMS) markers	Chromosomes	Start physical positions (bp)	End physical positions (bp)	Nipponbare	Kasalath	Nagina22	IR64	Pokkali	Bala	Tainung67	Azucena	Moroberekan	*Oryza rufipogon*	*Oryza nivara*	MSU gene locus IDs	Structural annotation	Functional annotation	SSR functional effects	Forward primers (5'-3')	Reverse primers (5'-3')	Amplified product size (bp)	Markers exhibiting polymorphism within *indica* rice	Transcrption factor genes-derived markers	Known cloned genes-derived markers	PMS markers corresponding with RM markers
PMS4654	Os_Chr09	7888535	7888574	(CT)20	(CT)20	(CT)17	(CT)20	(CT)20	(CT)17	(CT)20	(CT)19	(CT)20	(CT)20	(CT)20	-	Introns/Intergenic	-	-	CAATGCGAATCAAGGTTCAGAGC	GTGAGGTGGTGGGTGGTTATGG	194	P	A	A	RM23946
PMS4655	Os_Chr09	7966806	7966823	(AT)9	(AT)9	(AT)9	(AT)9	(AT)9	(AT)9	(AT)9	(AT)10	(AT)9	(AT)9	(AT)9	-	Introns/Intergenic	-	-	AACATACTCGGGAGAAGCCATCACG	CACACACTTGATGGGTGCCAAGG	285	A	A	A	RM23949
PMS4656	Os_Chr09	7971329	7971368	(AT)20	(AT)20	(AT)20	(AT)10	(AT)20	(AT)20	(AT)20	(AT)20	(AT)11	(AT)11	(AT)9	-	Introns/Intergenic	-	-	CCTTTCAGTGACTTGCGTTGC	CACCAGTGATCCTCTAGCAAATTACC	324	P	A	A	RM23950
PMS4657	Os_Chr09	7974259	7974272	(TG)7	(TG)7	(TG)7	(TG)7	(TG)6	(TG)7	(TG)7	(TG)7	(TG)7	(TG)7	(TG)6	-	Introns/Intergenic	-	-	TCTCTGCCAATGTCAGCTTG	GAATCGATGATCAGAAACCGA	249	P	A	A	NA
PMS4658	Os_Chr09	8031923	8031958	(TA)18	(TA)18	(TA)18	(TA)18	(TA)18	(TA)18	(TA)18	(TA)8	(TA)9	(TA)18	(TA)18	-	Introns/Intergenic	-	-	TGCCAATCATTGCTTTGGAAGG	GGCTATCCACAAACATCGGAAGC	251	A	A	A	RM23962
PMS4659	Os_Chr09	8066540	8066551	(AC)6	(AC)6	(AC)6	(AC)6	(AC)6	(AC)6	(AC)6	(AC)6	(AC)6	(AC)7	(AC)6	-	Introns/Intergenic	-	-	TAACACCGAACATCGCAATC	ATTGGCAAATCATCGAGAGC	190	A	A	A	NA
PMS4660	Os_Chr09	8085893	8085906	(AT)7	(AT)7	(AT)7	(AT)7	(AT)12	(AT)7	(AT)7	(AT)7	(AT)7	(AT)7	(AT)7	-	Introns/Intergenic	-	-	CAGCTTTGCTGCCAAATTCT	TGACAGACGATCGAACTTGC	100	P	A	A	NA
PMS4661	Os_Chr09	8162801	8162816	(CT)8	(CT)8	(CT)8	(CT)8	(CT)8	(CT)8	(CT)8	(CT)6	(CT)8	(CT)8	(CT)8	-	Introns/Intergenic	-	-	CAATACGTGTTTCCCATCGTTGC	CGAGTTAAACCGTCGAAAGATCG	244	A	A	A	RM23966
PMS4662	Os_Chr09	8188025	8188076	(AT)26	(AT)26	(AT)10	(AT)10	(AT)26	(AT)26	(AT)26	(AT)26	(AT)7	(AT)26	(AT)7	-	Introns/Intergenic	-	-	TGACCTCCCCTCCTCTTTTT	ATATTTGACACCAACCGGGA	155	P	A	A	NA
PMS4663	Os_Chr09	8217625	8217638	(GA)7	(GA)7	(GA)7	(GA)7	(GA)7	(GA)6	(GA)7	(GA)7	(GA)7	(GA)7	(GA)7	-	Introns/Intergenic	-	-	AAGTAGCCTCCCTCTCGCTC	ATTGCTTTTGAGAGCAGCGT	205	P	A	A	NA
PMS4664	Os_Chr09	8317635	8317684	(TA)25	(TA)25	(TA)25	(TA)8	(TA)25	(TA)25	(TA)25	(TA)25	(TA)6	(TA)12	(TA)25	-	Introns/Intergenic	-	-	ACTACTACCTCCGCAATTTAGC	TGTATGTACTCCTGGTCTCATCC	442	P	A	A	RM23973
PMS4665	Os_Chr09	8423125	8423162	(TA)19	(TA)19	(TA)19	(TA)13	(TA)19	(TA)19	(TA)19	(TA)19	(TA)19	(TA)19	(TA)19	-	Introns/Intergenic	-	-	ACAGGGCCTTAGTCTCGGAT	CGCTCCAGGGGTGTAGAATA	182	P	A	A	NA
PMS4666	Os_Chr09	8505974	8505991	(GGC)6	(GGC)6	(GGC)6	(GGC)6	(GGC)6	(GGC)6	(GGC)5	(GGC)6	(GGC)6	(GGC)6	(GGC)6	LOC_Os09g14400	CDS	expressed protein	CODON_DELETION	TTGGCTCCTCCGTATAGCTC	ATCTGGAACGGGAAGGTTG	242	A	A	A	NA

Polymorphic SSR (PMS) markers	Chromosomes	Start physical positions (bp)	End physical positions (bp)	Nipponbare	Kasalath	Nagina22	IR64	Pokkali	Bala	Tainung67	Azucena	Moroberekan	*Oryza rufipogon*	*Oryza nivara*	MSU gene locus IDs	Structural annotation	Functional annotation	SSR functional effects	Forward primers (5'-3')	Reverse primers (5'-3')	Amplified product size (bp)	Markers exhibiting polymorphism within *indica* rice	Transcrption factor genes-derived markers	Known cloned genes-derived markers	PMS markers corresponding with RM markers
PMS4667	Os_Chr09	8533146	8533225	(AT)40	(AT)40	(AT)11	(AT)9	(AT)10	(AT)10	(AT)40	(AT)40	(AT)11	(AT)40	(AT)40	-	Introns/Intergenic	-	-	GCTAGCTAGGACATCATAACGGA	CGGGACTAGAGATGGCTCAG	280	P	A	A	NA
PMS4668	Os_Chr09	8544988	8545033	(TA)23	(TA)23	(TA)23	(TA)7	(TA)23	(TA)23	(TA)23	(TA)10	(TA)23	(TA)23	(TA)23	-	Introns/Intergenic	-	-	CAAGCAGGAGCTCTTCTATTCC	GCAGAAGCGTGATGTTAATCG	166	P	A	A	RM23982
PMS4669	Os_Chr09	8549742	8549765	(TCA)8	(TCA)8	(TCA)8	(TCA)8	(TCA)7	(TCA)8	(TCA)8	(TCA)8	(TCA)8	(TCA)6	(TCA)8	LOC_Os09g14460	CDS	retrotransposon protein, putative, unclassified	CODON_DELETION	GAGACTCATCGGACTGTGTAGAGG	GATCCATTTGAGGCTTATGAGG	190	P	A	A	RM23983
PMS4670	Os_Chr09	8615143	8615156	(CT)7	(CT)7	(CT)7	(CT)7	(CT)7	(CT)9	(CT)7	(CT)7	(CT)7	(CT)7	(CT)7	-	Introns/Intergenic	-	-	CGTTTCCACTTTTGGTTCGT	TGAGAAAAAGTCAATGGCGTC	181	P	A	A	NA
PMS4671	Os_Chr09	8641978	8641991	(GA)7	(GA)7	(GA)7	(GA)7	(GA)7	(GA)7	(GA)7	(GA)7	(GA)6	(GA)7	(GA)7	-	Introns/Intergenic	-	-	CACTGTGCAGCGATGAAACT	TACTTCACAGTTGGCTCCCC	189	A	A	A	NA
PMS4672	Os_Chr09	8960199	8960218	(TA)10	(TA)10	(TA)10	(TA)8	(TA)8	(TA)10	(TA)10	(TA)10	(TA)10	(TA)10	(TA)10	-	Introns/Intergenic	-	-	GTGGAGTCAAGAGGGGAGTG	TTTCATACCATGATGCCCAG	154	P	A	A	NA
PMS4673	Os_Chr09	8967335	8967352	(AT)9	(AT)9	(AT)9	(AT)9	(AT)9	(AT)9	(AT)7	(AT)9	(AT)9	(AT)9	(AT)9	-	Introns/Intergenic	-	-	AGCGTGAATGCTCTTCATGG	GCATGGTGGCTTGATTTTCT	255	A	A	A	NA
PMS4674	Os_Chr09	9024739	9024759	(TCC)7	(TCC)7	(TCC)7	(TCC)7	(TCC)7	(TCC)7	(TCC)7	(TCC)7	(TCC)7	(TCC)6	(TCC)5	-	Introns/Intergenic	-	-	CTTCTTCCTCCGGGCTTGTCC	CTCTTGCCCTTCCAGTGTCTGC	289	A	A	A	RM23999
PMS4675	Os_Chr09	9206700	9206713	(TC)7	(TC)11	(TC)7	(TC)7	(TC)7	(TC)11	(TC)7	(TC)7	(TC)7	(TC)7	(TC)7	LOC_Os09g15170	URR	nucleobase-ascorbate transporter, putative, expressed	-	TCATCAACTAGGGCGGTAGG	AGGAGAGAGAGAGGGCAACC	154	P	A	A	NA
PMS4676	Os_Chr09	9301852	9301871	(AT)10	(AT)10	(AT)10	(AT)10	(AT)10	(AT)10	(AT)7	(AT)10	(AT)10	(AT)10	(AT)10	-	Introns/Intergenic	-	-	TGTGATAAACGAAGGCACTGA	GCCAAAGTTGCCAATGATTT	236	A	A	A	NA
PMS4677	Os_Chr09	9414769	9414812	(ATAG)11	(ATAG)11	(ATAG)11	(ATAG)11	(ATAG)11	(ATAG)10	(ATAG)11	(ATAG)11	(ATAG)11	(ATAG)11	(ATAG)11	-	Introns/Intergenic	-	-	CGGTCGGATCTTTCTGATTAACG	AGTAGGTCGAACCTAACCGATGC	141	P	A	A	RM24012
PMS4678	Os_Chr09	9508206	9508223	(AG)9	(AG)9	(AG)8	(AG)9	(AG)9	(AG)9	(AG)9	(AG)9	(AG)9	(AG)8	(AG)9	-	Introns/Intergenic	-	-	ATTGACGCAGAGCTCGAGAA	CAGCAAAAAGACTCCTTGGG	238	P	A	A	NA
PMS4679	Os_Chr09	9605336	9605353	(GTG)6	(GTG)6	(GTG)6	(GTG)5	(GTG)6	(GTG)6	(GTG)6	(GTG)6	(GTG)6	(GTG)6	(GTG)6	-	Introns/Intergenic	-	-	GCTAGGTACTCCAAGCAGCG	AGGAGCAGCCACTCCTGTAG	275	P	A	A	NA

Polymorphic SSR (PMS) markers	Chromosomes	Start physical positions (bp)	End physical positions (bp)	Nipponbare	Kasalath	Nagina22	IR64	Pokkali	Bala	Tainung67	Azucena	Moroberekan	*Oryza rufipogon*	*Oryza nivara*	MSU gene locus IDs	Structural annotation	Functional annotation	SSR functional effects	Forward primers (5'-3')	Reverse primers (5'-3')	Amplified product size (bp)	Markers exhibiting polymorphism within *indica* rice	Transcrption factor genes-derived markers	Known cloned genes-derived markers	PMS markers corresponding with RM markers
PMS4680	Os_Chr09	9887412	9887425	(GA)7	(GA)7	(GA)7	(GA)7	(GA)7	(GA)6	(GA)7	(GA)7	(GA)7	(GA)7	(GA)6	-	Introns/Intergenic	-	-	GTCGCCGCTCTAGTGCTT	CTCTCAGCTCCCACCAGTTC	188	P	A	A	NA
PMS4681	Os_Chr09	9906139	9906202	(TA)32	(TA)14	(TA)32	(TA)32	(TA)32	(TA)32	(TA)32	(TA)32	(TA)32	(TA)32	(TA)32	-	Introns/Intergenic	-	-	AAGGTGGTAGCTGCTTCATTCG	GGAGATCATATGGAGGGAAAGG	447	P	A	A	RM24031
PMS4682	Os_Chr09	9986483	9986512	(CT)15	(CT)15	(CT)15	(CT)13	(CT)15	(CT)15	(CT)15	(CT)15	(CT)15	(CT)15	(CT)15	-	Introns/Intergenic	-	-	AAATCCCTCCGCAAAAGTCT	GAAAGGAGGGCTTGGCTC	254	P	A	A	NA
PMS4683	Os_Chr09	10050872	10050887	(GA)8	(GA)8	(GA)8	(GA)8	(GA)8	(GA)8	(GA)8	(GA)8	(GA)8	(GA)8	(GA)6	-	Introns/Intergenic	-	-	GCTCGGGTGAGGGTTTTTAT	TTCGATTTCGAATCCCACC	119	A	A	A	NA
PMS4684	Os_Chr09	10067629	10067682	(AT)27	(AT)27	(AT)27	(AT)27	(AT)27	(AT)27	(AT)27	(AT)16	(AT)27	(AT)27	(AT)27	-	Introns/Intergenic	-	-	TCGTGCGCCTCAACAAAGATAGC	ACACAATGGTTGGCTTGCCTATGG	386	A	A	A	RM24040
PMS4685	Os_Chr09	10121467	10121492	(CT)13	(CT)12	(CT)13	(CT)13	(CT)12	(CT)13	(CT)13	(CT)13	(CT)13	(CT)13	(CT)13	-	Introns/Intergenic	-	-	CCTGCTGCCTAGGACGTTGTCG	TTGCCGCTAAGGCATCTAAGTTCAGC	372	P	A	A	RM24042
PMS4686	Os_Chr09	10128416	10128431	(AT)8	(AT)8	(AT)7	(AT)8	(AT)8	(AT)8	(AT)8	(AT)8	(AT)8	(AT)8	(AT)8	-	Introns/Intergenic	-	-	ACCATCTCCGAAACATCCAG	AGCGTCAGCTTGGACACTTT	260	P	A	A	NA
PMS4687	Os_Chr09	10183556	10183587	(TA)16	(TA)16	(TA)16	(TA)6	(TA)16	(TA)16	(TA)16	(TA)16	(TA)16	(TA)16	(TA)13	-	Introns/Intergenic	-	-	TCCTCCCTCTCCTTCTCTCC	TTGAATCGGATATATAAACTTTAGGTG	267	P	A	A	NA
PMS4688	Os_Chr09	10321465	10321492	(AT)14	(AT)14	(AT)14	(AT)10	(AT)14	(AT)14	(AT)14	(AT)14	(AT)14	(AT)14	(AT)14	-	Introns/Intergenic	-	-	ATACACATATGCACATACATACA	GGGACTAAAGATCGGTGTACT	379	P	A	A	NA
PMS4689	Os_Chr09	10336186	10336197	(GA)6	(GA)6	(GA)7	(GA)6	(GA)6	(GA)6	(GA)6	(GA)6	(GA)6	(GA)6	(GA)6	-	Introns/Intergenic	-	-	TGTGATTTTGCGAACCACAT	TTAACGGCATCTCTTTTGGG	205	P	A	A	NA
PMS4690	Os_Chr09	10361054	10361067	(GA)7	(GA)6	(GA)6	(GA)7	(GA)7	(GA)7	(GA)7	(GA)7	(GA)7	(GA)7	(GA)7	-	Introns/Intergenic	-	-	TTTCACTTCGCGATCTCCTT	AGCAACCAATTCTGGGACAC	275	P	A	A	NA
PMS4691	Os_Chr09	10432599	10432624	(TA)13	(TA)13	(TA)13	(TA)13	(TA)13	(TA)13	(TA)13	(TA)13	(TA)13	(TA)8	(TA)13	-	Introns/Intergenic	-	-	GGCCGGCTAATCCATCTTTAGTCC	TCCTCTTCTGACGTAACGCAAGC	394	A	A	A	RM24067
PMS4692	Os_Chr09	10453120	10453137	(CGG)6	(CGG)7	(CGG)6	(CGG)6	(CGG)6	(CGG)6	(CGG)6	(CGG)5	(CGG)5	(CGG)6	(CGG)6	-	Introns/Intergenic	-	-	CTCTTGGAGTGTTCGCTTCC	TAACCCCTCGTTTCCATCTG	226	P	A	A	NA

Polymorphic SSR (PMS) markers	Chromosomes	Start physical positions (bp)	End physical positions (bp)	Nipponbare	Kasalath	Nagina22	IR64	Pokkali	Bala	Tainung67	Azucena	Moroberekan	*Oryza rufipogon*	*Oryza nivara*	MSU gene locus IDs	Structural annotation	Functional annotation	SSR functional effects	Forward primers (5'-3')	Reverse primers (5'-3')	Amplified product size (bp)	Markers exhibiting polymorphism within *indica* rice	Transcrption factor genes-derived markers	Known cloned genes-derived markers	PMS markers corresponding with RM markers
PMS4693	Os_Chr09	10466355	10466447	(TTA)31	(TTA)31	(TTA)31	(TTA)31	(TTA)31	(TTA)27	(TTA)14	(TTA)12	(TTA)31	(TTA)31	(TTA)31	-	Introns/Intergenic	-	-	GCGAATTAGCATTCTCGTTCTCTGG	TGGGCGATACCAAGGACTATTCAGG	743	P	A	A	RM24069
PMS4694	Os_Chr09	10487528	10487545	(GGC)6	(GGC)5	(GGC)6	(GGC)6	(GGC)6	(GGC)5	(GGC)6	(GGC)6	(GGC)6	(GGC)6	(GGC)6	-	Introns/Intergenic	-	-	TCTCTCGTCTCGCCTCTCTC	CTAGATCTGGCGGAGGGAA	239	P	A	A	NA
PMS4695	Os_Chr09	10499067	10499087	(AAT)7	(AAT)7	(AAT)7	(AAT)7	(AAT)6	(AAT)7	(AAT)7	(AAT)7	(AAT)7	(AAT)7	(AAT)7	-	Introns/Intergenic	-	-	GGACACTAGCACGCAAACAC	AGGTAGGATGCTAGGAGGGG	266	P	A	A	NA
PMS4696	Os_Chr09	10559802	10559825	(GGA)8	(GGA)8	(GGA)8	(GGA)8	(GGA)8	(GGA)8	(GGA)8	(GGA)8	(GGA)8	(GGA)7	(GGA)8	-	Introns/Intergenic	-	-	GTGTCTCTGGCTTGACTTTGATGAGG	GCTGATGCGACAATGGTACACG	328	A	A	A	RM24076
PMS4697	Os_Chr09	10609231	10609252	(AT)11	(AT)6	(AT)11	(AT)11	(AT)11	(AT)11	(AT)11	(AT)11	(AT)11	(AT)11	(AT)11	-	Introns/Intergenic	-	-	ACGGAATTGACCAAAAATGA	CCGATTACACCATTGTGTTTG	241	P	A	A	NA
PMS4698	Os_Chr09	10751725	10751748	(CGA)8	(CGA)5	(CGA)8	(CGA)8	(CGA)8	(CGA)8	(CGA)6	(CGA)8	(CGA)8	(CGA)8	(CGA)8	LOC_Os09g17580	CDS	expressed protein	CODON_CHANGE_PLUS_CODON_DELETION	TGGTTACTTCTCGAAACCGC	CCGAGTCTTCCACGCATC	272	P	A	A	NA
PMS4699	Os_Chr09	10868998	10869079	(TA)41	(TA)41	(TA)41	(TA)6	(TA)7	(TA)15	(TA)41	(TA)41	(TA)41	(TA)41	(TA)16	-	Introns/Intergenic	-	-	TCAATTTCAACAATGGTGCC	CATGGATCCCATGTTTCTCC	272	P	A	A	NA
PMS4700	Os_Chr09	10890579	10890600	(AG)11	(AG)11	(AG)11	(AG)11	(AG)11	(AG)11	(AG)11	(AG)11	(AG)11	(AG)13	(AG)11	-	Introns/Intergenic	-	-	AATTTCAGCTGCTCCAGCCCTAACC	TAACCTCGCCGCCAGATCTACTCG	161	A	A	A	RM24092
PMS4701	Os_Chr09	10939760	10939801	(GA)21	(GA)21	(GA)15	(GA)21	(GA)14	(GA)21	(GA)21	(GA)21	(GA)21	(GA)16	(GA)21	-	Introns/Intergenic	-	-	GTCCACCAACCTCTTAGACTCAACC	AGTGATGAAGGCAGATCTCAAGC	166	P	A	A	RM24093
PMS4702	Os_Chr09	10957730	10957785	(TA)28	(TA)26	(TA)28	(TA)28	(TA)28	(TA)28	(TA)28	(TA)28	(TA)28	(TA)28	(TA)28	-	Introns/Intergenic	-	-	GGAGTAATAGCAAGTTTGCACTGG	TCCACCAAATACATGTGGACTAGC	409	P	A	A	RM24094
PMS4703	Os_Chr09	10975107	10975122	(TA)8	(TA)8	(TA)8	(TA)8	(TA)8	(TA)7	(TA)8	(TA)8	(TA)8	(TA)8	(TA)8	-	Introns/Intergenic	-	-	ACATCGAGCATCACCTGACA	AAATTTCAATCATGGTGCCC	130	P	A	A	NA
PMS4704	Os_Chr09	11020536	11020556	(CGG)7	(CGG)7	(CGG)7	(CGG)7	(CGG)7	(CGG)6	(CGG)7	(CGG)7	(CGG)7	(CGG)7	(CGG)6	LOC_Os09g18000	CDS	senescence-induced receptor-like serine/threonine-protein kinase precursor, putative, expressed	CODON_DELETION	GTCCTTCGTCCACGGCAACTACG	TACGCCGAGGTACAGGTCGAAGC	109	P	A	A	RM24097
PMS4705	Os_Chr09	11175987	11175998	(AT)6	(AT)7	(AT)6	(AT)6	(AT)6	(AT)6	(AT)6	(AT)6	(AT)6	(AT)6	(AT)6	-	Introns/Intergenic	-	-	AAAAGTAAATCTGTTGTGGGGA	CGAATCTACAACCCAATTATACCA	214	P	A	A	NA

Polymorphic SSR (PMS) markers	Chromosomes	Start physical positions (bp)	End physical positions (bp)	Nipponbare	Kasalath	Nagina22	IR64	Pokkali	Bala	Tainung67	Azucena	Moroberekan	*Oryza rufipogon*	*Oryza nivara*	MSU gene locus IDs	Structural annotation	Functional annotation	SSR functional effects	Forward primers (5'-3')	Reverse primers (5'-3')	Amplified product size (bp)	Markers exhibiting polymorphism within *indica* rice	Transcrption factor genes-derived markers	Known cloned genes-derived markers	PMS markers corresponding with RM markers
PMS4706	Os_Chr09	11255694	11255713	(AC)10	(AC)6	(AC)10	(AC)10	(AC)10	(AC)10	(AC)10	(AC)10	(AC)10	(AC)10	(AC)10	-	Introns/Intergenic	-	-	GCACCTGAACCTAGACTCGACACG	TAGGGAGAGGGCTCTCACATGC	174	P	A	A	RM24102
PMS4707	Os_Chr09	11292958	11292978	(GCT)7	(GCT)7	(GCT)7	(GCT)7	(GCT)7	(GCT)7	(GCT)5	(GCT)7	(GCT)7	(GCT)7	(GCT)7	LOC_Os09g18400	CDS	expressed protein	CODON_DELETION	GTGGCACAGTGTCCTGCAGAGG	CCCTCGCTGGTGATGTATCTAAAGG	187	A	A	A	RM24107
PMS4708	Os_Chr09	11302179	11302196	(AG)9	(AG)7	(AG)7	(AG)7	(AG)7	(AG)7	(AG)9	(AG)9	(AG)9	(AG)9	(AG)9	-	Introns/Intergenic	-	-	TGTATGACAGGTGGGACCAG	CTGCATTGTGAATGGCCTTA	166	A	A	A	NA
PMS4709	Os_Chr09	11474418	11474493	(TA)38	(TA)38	(TA)38	(TA)38	(TA)38	(TA)38	(TA)38	(TA)12	(TA)38	(TA)38	(TA)38	-	Introns/Intergenic	-	-	AACGTCACCGTTTCCCGTATAGC	AGGTGCTGTGATAGTGCTTGTCG	357	A	A	A	RM24119
PMS4710	Os_Chr09	11492996	11493069	(TA)37	(TA)37	(TA)37	(TA)37	(TA)37	(TA)37	(TA)37	(TA)37	(TA)6	(TA)37	(TA)37	-	Introns/Intergenic	-	-	TGGAGTCGTGAGTACATCCTTTGG	CACTCTCATTGCACATAGCACTCC	350	A	A	A	RM24121
PMS4711	Os_Chr09	11611519	11611556	(TA)19	(TA)19	(TA)8	(TA)19	(TA)11	(TA)11	(TA)19	(TA)19	(TA)19	(TA)19	(TA)19	-	Introns/Intergenic	-	-	GAGATGATTGATGTGACGGAAGG	CCTGTTGACGTTGCATTATAGACC	272	P	A	A	RM24127
PMS4712	Os_Chr09	11768459	11768478	(AT)10	(AT)10	(AT)10	(AT)10	(AT)10	(AT)10	(AT)7	(AT)10	(AT)10	(AT)10	(AT)10	-	Introns/Intergenic	-	-	CAAGTCGTTCTCTCGCCTACTGC	CATTGGAATGGAGGATGATAGTGG	214	A	A	A	RM1896
PMS4713	Os_Chr09	11815858	11815875	(CGG)6	(CGG)5	(CGG)6	(CGG)6	(CGG)6	(CGG)6	(CGG)6	(CGG)6	(CGG)6	(CGG)6	(CGG)6	-	Introns/Intergenic	-	-	GCTTCTGGACTCTGGAGGTG	CGAAACCAACGATCCAGTTT	148	P	A	A	NA
PMS4714	Os_Chr09	11880838	11880861	(GCA)8	(GCA)8	(GCA)8	(GCA)8	(GCA)8	(GCA)8	(GCA)8	(GCA)5	(GCA)8	(GCA)8	(GCA)8	LOC_Os09g19850	CDS	chloroplastic group IIA intron splicing facilitator CRS1, chloroplast precursor, putative, expressed	CODON_DELETION	GTAAGAAGAGGAGGTCCCTGAAGC	ACCATCCGAGGAATCGTAGTCG	230	A	A	A	RM24141
PMS4715	Os_Chr09	11956471	11956491	(GGA)7	(GGA)7	(GGA)7	(GGA)7	(GGA)7	(GGA)7	(GGA)5	(GGA)7	(GGA)7	(GGA)7	(GGA)7	LOC_Os09g19970	CDS	expressed protein	CODON_CHANGE_PLUS_CODON_DELETION	AGCTTCTACGCGTTCCACC	GACCTCCTCACCTCCTCCTC	278	A	A	A	NA
PMS4716	Os_Chr09	12088983	12089040	(TA)29	(TA)29	(TA)29	(TA)29	(TA)29	(TA)11	(TA)7	(TA)29	(TA)29	(TA)29	(TA)7	-	Introns/Intergenic	-	-	CTGGCAGGTGAATAACAGCAAGC	CCCACCACTCACCTCTTCTCAGC	457	P	A	A	RM24149
PMS4717	Os_Chr09	12139570	12139601	(AT)16	(AT)16	(AT)11	(AT)16	(AT)16	(AT)16	(AT)16	(AT)16	(AT)8	(AT)16	(AT)16	-	Introns/Intergenic	-	-	TAGTTGGCAAGACGACCGAAAGG	GTGAACCGACAAAGGCGTAATCG	431	P	A	A	RM24154
PMS4718	Os_Chr09	12208437	12208454	(GGT)6	(GGT)6	(GGT)6	(GGT)6	(GGT)6	(GGT)6	(GGT)5	(GGT)6	(GGT)6	(GGT)6	(GGT)6	LOC_Os09g20340	DRR	DUF630/DUF632 domains containing protein, putative, expressed	-	TGAAGAACTCAACCGAGGCT	CCTAGTCAGGAGCCACCAAC	232	A	A	A	NA

Polymorphic SSR (PMS) markers	Chromosomes	Start physical positions (bp)	End physical positions (bp)	Nipponbare	Kasalath	Nagina22	IR64	Pokkali	Bala	Tainung67	Azucena	Moroberekan	*Oryza rufipogon*	*Oryza nivara*	MSU gene locus IDs	Structural annotation	Functional annotation	SSR functional effects	Forward primers (5'-3')	Reverse primers (5'-3')	Amplified product size (bp)	Markers exhibiting polymorphism within *indica* rice	Transcrption factor genes-derived markers	Known cloned genes-derived markers	PMS markers corresponding with RM markers
PMS4719	Os_Chr09	12257249	12257272	(TA)12	(TA)12	(TA)12	(TA)12	(TA)12	(TA)12	(TA)12	(TA)10	(TA)12	(TA)12	(TA)12	-	Introns/Intergenic	-	-	TGGGCCTAATAGAGAAGGAATGG	GGCGGGCCTAGTAGTGATTAAGG	168	A	A	A	RM24158
PMS4720	Os_Chr09	12268366	12268379	(TA)7	(TA)7	(TA)7	(TA)7	(TA)7	(TA)7	(TA)7	(TA)7	(TA)7	(TA)7	(TA)6	-	Introns/Intergenic	-	-	CAGGCTGTTAGCTACACTTGGT	GCCCTACATGTACAGCTCGC	102	A	A	A	NA
PMS4721	Os_Chr09	12284570	12284584	(GGA)5	(GGA)5	(GGA)5	(GGA)5	(GGA)5	(GGA)5	(GGA)5	(GGA)5	(GGA)5	(GGA)6	(GGA)5	LOC_Os09g20420	CDS	expressed protein	CODON_INSERTION	CCAAGAAGCAGAGGAAGGTG	GCTCGATCTCCTCCACCAC	105	A	P	A	NA
PMS4722	Os_Chr09	12284783	12284800	(GAC)6	(GAC)6	(GAC)6	(GAC)6	(GAC)6	(GAC)6	(GAC)5	(GAC)6	(GAC)6	(GAC)6	(GAC)6	LOC_Os09g20420	CDS	expressed protein	CODON_DELETION	CACGAGCAGAGGAAGAAGGT	CATGAGCCACTTCGAGAACA	255	A	P	A	NA
PMS4723	Os_Chr09	12319985	12320008	(CAG)8	(CAG)5	(CAG)8	(CAG)8	(CAG)8	(CAG)5	(CAG)8	(CAG)8	(CAG)8	(CAG)8	(CAG)8	LOC_Os09g20460	CDS	VQ domain containing protein, putative, expressed	CODON_DELETION	ACGTCGTTGCAGCATCAG	GGAAGTTGGAGGTGTCGGT	280	P	A	A	NA
PMS4724	Os_Chr09	12349350	12349367	(TGA)6	(TGA)6	(TGA)5	(TGA)6	(TGA)5	(TGA)5	(TGA)6	(TGA)6	(TGA)6	(TGA)6	(TGA)6	LOC_Os09g20480	CDS	transporter, putative, expressed	CODON_CHANGE_PLUS_CODON_DELETION	ATCGGGCAGGAATAGGAAGT	GGTGCAAACTAGGACCTGGA	280	P	A	A	NA
PMS4725	Os_Chr09	12352925	12352976	(ATAG)13	(ATAG)13	(ATAG)13	(ATAG)5	(ATAG)7	(ATAG)13	(ATAG)13	(ATAG)13	(ATAG)13	(ATAG)6	(ATAG)13	-	Introns/Intergenic	-	-	TTGGGAGCGTTTGTAGGAAG	CTGCTAATTTCTTGCCCTGC	209	P	A	A	NA
PMS4726	Os_Chr09	12438973	12438999	(ACA)9	(ACA)9	(ACA)6	(ACA)9	(ACA)9	(ACA)6	(ACA)9	(ACA)9	(ACA)9	(ACA)8	(ACA)9	-	Introns/Intergenic	-	-	CTCCGGTGACCTAGAACCAAACACC	GATTCGGCGGCCTCTCCTATCC	247	P	A	A	RM24170
PMS4727	Os_Chr09	12453055	12453076	(AT)11	(AT)11	(AT)11	(AT)9	(AT)11	(AT)11	(AT)11	(AT)11	(AT)11	(AT)11	(AT)11	-	Introns/Intergenic	-	-	TCATTGAGATACCGATGGCA	CCCTTACTACCCTAGCTATACGGA	267	P	A	A	NA
PMS4728	Os_Chr09	12556133	12556147	(CGG)5	(CGG)6	(CGG)5	(CGG)5	(CGG)5	(CGG)5	(CGG)5	(CGG)5	(CGG)5	(CGG)5	(CGG)5	LOC_Os09g20850	CDS	expressed protein	CODON_CHANGE_PLUS_CODON_INSERTION	ACGGTTTCGGTGTTCTGGT	AGGTAGAGCCGGTTGTGGT	107	P	A	A	NA
PMS4729	Os_Chr09	12596508	12596545	(CT)19	(CT)18	(CT)19	(CT)13	(CT)13	(CT)19	(CT)19	(CT)19	(CT)19	(CT)19	(CT)13	-	Introns/Intergenic	-	-	CAATTTGAGCCAGCCAGTTTACC	CTTAGGGCTTGGCTCTTCATGC	369	P	A	A	RM24183
PMS4730	Os_Chr09	12607412	12607429	(TCG)6	(TCG)6	(TCG)6	(TCG)6	(TCG)6	(TCG)5	(TCG)6	(TCG)6	(TCG)6	(TCG)6	(TCG)6	LOC_Os09g20940	CDS	expressed protein	CODON_DELETION	CCTGAGGAGCTCCACCCT	TCGGTTAATTATGAAGGCGG	223	P	A	A	NA
PMS4731	Os_Chr09	12620573	12620590	(TA)9	(TA)9	(TA)6	(TA)9	(TA)9	(TA)9	(TA)9	(TA)9	(TA)9	(TA)9	(TA)9	-	Introns/Intergenic	-	-	TTGATATTCCGAATGTACACAAA	CCCTACCTAAGCTTCCCGAC	248	P	A	A	NA

Polymorphic SSR (PMS) markers	Chromosomes	Start physical positions (bp)	End physical positions (bp)	Nipponbare	Kasalath	Nagina22	IR64	Pokkali	Bala	Tainung67	Azucena	Moroberekan	*Oryza rufipogon*	*Oryza nivara*	MSU gene locus IDs	Structural annotation	Functional annotation	SSR functional effects	Forward primers (5'-3')	Reverse primers (5'-3')	Amplified product size (bp)	Markers exhibiting polymorphism within *indica* rice	Transcrption factor genes-derived markers	Known cloned genes-derived markers	PMS markers corresponding with RM markers
PMS4732	Os_Chr09	12695127	12695162	(AT)18	(AT)18	(AT)18	(AT)18	(AT)18	(AT)18	(AT)18	(AT)14	(AT)9	(AT)18	(AT)18	-	Introns/Intergenic	-	-	CGAGATAGTTGGGTTGGGAA	TGGTGCATTTTATTCAAACTCAG	269	A	A	A	NA
PMS4733	Os_Chr09	12746602	12746619	(CGC)6	(CGC)6	(CGC)6	(CGC)6	(CGC)6	(CGC)6	(CGC)6	(CGC)6	(CGC)6	(CGC)6	(CGC)5	LOC_Os09g21120	CDS	armadillo/beta-catenin repeat family protein, putative, expressed	CODON_DELETION	GCTGTCCTCTCATCGTCCTC	GCCGGAAGAGACAGTAGCC	250	A	A	A	NA
PMS4734	Os_Chr09	12831215	12831232	(AT)9	(AT)9	(AT)9	(AT)9	(AT)6	(AT)9	(AT)9	(AT)9	(AT)9	(AT)6	(AT)9	-	Introns/Intergenic	-	-	CTTTCGATCCCTATGTCCCA	CATGGATCTTGGATCAGCCT	242	P	A	A	NA
PMS4735	Os_Chr09	12833979	12834000	(TC)11	(TC)7	(TC)11	(TC)11	(TC)11	(TC)11	(TC)11	(TC)11	(TC)11	(TC)11	(TC)11	-	Introns/Intergenic	-	-	TGTCTGAACCGGTGAGCTATTGG	AAAGAGGGAGAGACGGCGAAGG	157	P	A	A	RM24201
PMS4736	Os_Chr09	12839933	12839959	(GCC)9	(GCC)9	(GCC)6	(GCC)9	(GCC)9	(GCC)9	(GCC)9	(GCC)9	(GCC)9	(GCC)9	(GCC)7	-	Introns/Intergenic	-	-	TCTCGTTCTCTTCTCTTTCGCTGACC	GGAGGGAGAGGTGGGATTAAGAAGC	164	P	A	A	RM6475
PMS4737	Os_Chr09	12840111	12840140	(GGAGGT)5	(GGAGGT)7	(GGAGGT)5	(GGAGGT)5	(GGAGGT)5	(GGAGGT)5	(GGAGGT)5	(GGAGGT)5	(GGAGGT)5	(GGAGGT)7	(GGAGGT)5	-	Introns/Intergenic	-	-	CTTCTCTTTCGCTGACCTGG	ACAACCTCAACTCTCCACCG	255	P	A	A	NA
PMS4738	Os_Chr09	12925372	12925393	(AT)11	(AT)11	(AT)11	(AT)8	(AT)7	(AT)11	(AT)11	(AT)11	(AT)11	(AT)11	(AT)11	-	Introns/Intergenic	-	-	ATCATAGCCCAGACCAAGAATGC	AGATGAAGAGCAGGAACCGTAGG	148	P	A	A	RM524
PMS4739	Os_Chr09	12944322	12944335	(TC)7	(TC)8	(TC)7	(TC)7	(TC)7	(TC)7	(TC)7	(TC)7	(TC)7	(TC)7	(TC)7	-	Introns/Intergenic	-	-	AGAACCCACGTCATCACTCC	ACCGCCTCCTTCTGTAAGGT	202	P	A	A	NA
PMS4740	Os_Chr09	12981804	12981829	(TA)13	(TA)13	(TA)6	(TA)13	(TA)8	(TA)13	(TA)13	(TA)13	(TA)13	(TA)6	(TA)6	-	Introns/Intergenic	-	-	AACGTTTATCTCCACCTCATGC	CATTCCGTGAAGAACATCAAGC	405	P	A	A	RM24212
PMS4741	Os_Chr09	13010117	13010140	(CCG)8	(CCG)8	(CCG)8	(CCG)8	(CCG)8	(CCG)8	(CCG)8	(CCG)5	(CCG)8	(CCG)8	(CCG)8	-	Introns/Intergenic	-	-	ACCACTCCACTCCCGCATGTCC	AGGCACGAGCTGATCCGTACCC	365	A	A	A	RM24213
PMS4742	Os_Chr09	13054376	13054395	(AT)10	(AT)10	(AT)7	(AT)10	(AT)10	(AT)10	(AT)10	(AT)10	(AT)10	(AT)10	(AT)10	-	Introns/Intergenic	-	-	ACACACATGCACTCGGTCAGTCG	TTAGTCGCGCCACGGTATTTGG	460	P	A	A	RM24216
PMS4743	Os_Chr09	13095336	13095429	(TA)47	(TA)47	(TA)10	(TA)6	(TA)47	(TA)8	(TA)7	(TA)47	(TA)47	(TA)47	(TA)9	-	Introns/Intergenic	-	-	CAGAATCCAATAGGCTCCACACG	GTCAACGGCCACTTCAAGCTACC	458	P	A	A	RM24217
PMS4744	Os_Chr09	13164501	13164521	(GGT)7	(GGT)5	(GGT)7	(GGT)7	(GGT)7	(GGT)7	(GGT)7	(GGT)7	(GGT)7	(GGT)7	(GGT)7	LOC_Os09g21740	DRR	armadillo/beta-catenin repeat family protein, putative, expressed	-	TCGGTGGAATCAATCAATCA	GACATGGCGAGGAAGACG	236	P	A	A	NA

Polymorphic SSR (PMS) markers	Chromosomes	Start physical positions (bp)	End physical positions (bp)	Nipponbare	Kasalath	Nagina22	IR64	Pokkali	Bala	Tainung67	Azucena	Moroberekan	*Oryza rufipogon*	*Oryza nivara*	MSU gene locus IDs	Structural annotation	Functional annotation	SSR functional effects	Forward primers (5'-3')	Reverse primers (5'-3')	Amplified product size (bp)	Markers exhibiting polymorphism within *indica* rice	Transcrption factor genes-derived markers	Known cloned genes-derived markers	PMS markers corresponding with RM markers
PMS4745	Os_Chr09	13379516	13379530	(ATA)5	(ATA)5	(ATA)5	(ATA)5	(ATA)5	(ATA)5	(ATA)5	(ATA)5	(ATA)5	(ATA)6	(ATA)5	LOC_Os09g22090	URR	mannose-6-phosphate isomerase, putative, expressed	-	GGGCAGACGCGTTTAATAGA	AGGAGCTGTGTTAGGGGAGC	236	A	A	A	NA
PMS4746	Os_Chr09	13436047	13436084	(TA)19	(TA)19	(TA)9	(TA)6	(TA)19	(TA)10	(TA)19	(TA)19	(TA)19	(TA)19	(TA)9	-	Introns/Intergenic	-	-	AACGGGTGGTGTGAACCTATTCC	CGATTGACCATCGTGAGCTACC	263	P	A	A	RM24229
PMS4747	Os_Chr09	13658039	13658080	(TA)21	(TA)21	(TA)6	(TA)9	(TA)21	(TA)6	(TA)21	(TA)21	(TA)6	(TA)12	(TA)21	-	Introns/Intergenic	-	-	CAAATCCGTACTCCCTCCAG	GAATGGTCAAACGATGCAAA	267	P	A	A	NA
PMS4748	Os_Chr09	13713195	13713215	(GAC)7	(GAC)5	(GAC)7	(GAC)7	(GAC)7	(GAC)6	(GAC)7	(GAC)7	(GAC)7	(GAC)7	(GAC)6	-	Introns/Intergenic	-	-	GACCGGATCGATCACGTACAGG	CGTTTGACTGCTCATTCGTCTCC	370	P	A	A	RM24239
PMS4749	Os_Chr09	13740139	13740152	(AT)7	(AT)8	(AT)7	(AT)7	(AT)7	(AT)8	(AT)7	(AT)7	(AT)7	(AT)7	(AT)8	-	Introns/Intergenic	-	-	TAGACAAACCAAGCTGGCCT	AGAGACCAGTAACACCATCCAA	260	P	A	A	NA
PMS4750	Os_Chr09	13786499	13786513	(GCG)5	(GCG)5	(GCG)5	(GCG)5	(GCG)5	(GCG)5	(GCG)5	(GCG)5	(GCG)5	(GCG)7	(GCG)5	-	Introns/Intergenic	-	-	GCGATGAGGAGACCAGTAGC	GTTCTCGCTATTCCTGCCAC	245	A	A	A	NA
PMS4751	Os_Chr09	13804851	13804866	(GA)8	(GA)8	(GA)8	(GA)8	(GA)8	(GA)8	(GA)8	(GA)8	(GA)8	(GA)6	(GA)7	-	Introns/Intergenic	-	-	AGCTCGCCAGGGATGGAG	AATCCTTCCCAAATCAATTC	152	A	A	A	NA
PMS4752	Os_Chr09	13826943	13826958	(TA)8	(TA)8	(TA)8	(TA)8	(TA)8	(TA)8	(TA)8	(TA)8	(TA)8	(TA)8	(TA)6	-	Introns/Intergenic	-	-	CAAGCAAAAACTCAAAATCCA	CCAGGTTAATTGGATCCTCG	143	A	A	A	NA
PMS4753	Os_Chr09	13875519	13875536	(CTT)6	(CTT)6	(CTT)6	(CTT)6	(CTT)6	(CTT)6	(CTT)6	(CTT)6	(CTT)6	(CTT)7	(CTT)6	LOC_Os09g23350	URR	trehalose synthase, putative, expressed	-	TTTGGTTTGATTTTGGGAGC	GCAGCAACAACAAAGAACCA	114	A	A	A	NA
PMS4754	Os_Chr09	13891885	13891905	(GCG)7	(GCG)7	(GCG)7	(GCG)7	(GCG)7	(GCG)7	(GCG)7	(GCG)7	(GCG)7	(GCG)6	(GCG)7	LOC_Os09g23380	URR	metallo-beta-lactamase, putative, expressed	-	GGCTCACACTGATATGGGCT	CGAAGGGTACCTAGCACGAG	240	A	A	A	NA
PMS4755	Os_Chr09	13958709	13958726	(GGC)6	(GGC)6	(GGC)6	(GGC)6	(GGC)6	(GGC)6	(GGC)6	(GGC)6	(GGC)6	(GGC)5	(GGC)6	LOC_Os09g23480	CDS	transposon protein, putative, CACTA, En/Spm sub-class, expressed	CODON_DELETION	ACAACGGAGAGAGATCGAG	CGTATCTCACCGTCGTCT	145	A	A	A	NA
PMS4756	Os_Chr09	13980274	13980301	(TA)14	(TA)14	(TA)14	(TA)14	(TA)14	(TA)6	(TA)14	(TA)14	(TA)6	(TA)14	(TA)6	-	Introns/Intergenic	-	-	GGGCCCACAGCTTTGTACT	AACACGCTATACCTAGTTCGACG	226	P	A	A	NA
PMS4757	Os_Chr09	14030823	14030836	(GA)7	(GA)7	(GA)7	(GA)7	(GA)7	(GA)7	(GA)7	(GA)7	(GA)7	(GA)7	(GA)6	LOC_Os09g23620	DRR	MYB family transcription factor, putative, expressed	-	GCGCTAGCTGCACTTAGTGAT	TTAAAGAGGGCTAGCTCATGC	157	A	P	A	NA

Polymorphic SSR (PMS) markers	Chromosomes	Start physical positions (bp)	End physical positions (bp)	Nipponbare	Kasalath	Nagina22	IR64	Pokkali	Bala	Tainung67	Azucena	Moroberekan	*Oryza rufipogon*	*Oryza nivara*	MSU gene locus IDs	Structural annotation	Functional annotation	SSR functional effects	Forward primers (5'-3')	Reverse primers (5'-3')	Amplified product size (bp)	Markers exhibiting polymorphism within *indica* rice	Transcrption factor genes-derived markers	Known cloned genes-derived markers	PMS markers corresponding with RM markers
PMS4758	Os_Chr09	14096951	14096965	(CTC)5	(CTC)5	(CTC)5	(CTC)5	(CTC)5	(CTC)5	(CTC)5	(CTC)5	(CTC)5	(CTC)6	(CTC)5	-	Introns/Intergenic	-	-	TGAGCAATTGCCAAACGTAA	GTCTACTACTCCGCCCCGAT	222	A	A	A	NA
PMS4759	Os_Chr09	14106816	14106836	(GCG)7	(GCG)7	(GCG)7	(GCG)7	(GCG)7	(GCG)6	(GCG)7	(GCG)7	(GCG)7	(GCG)7	(GCG)7	LOC_Os09g23730	CDS	PF1	CODON_DELETION	CTGTTCGACCTTGGCCTTCTTGG	CCGGAGGTAAGAACCGCTTTCC	507	P	A	P	RM24257
PMS4760	Os_Chr09	14118440	14118460	(ACC)7	(ACC)7	(ACC)7	(ACC)7	(ACC)7	(ACC)7	(ACC)6	(ACC)5	(ACC)5	(ACC)7	(ACC)7	-	Introns/Intergenic	-	-	GAATTCCTTCCACTCAAAGTCAGC	GTCCACTCCTCCTCATCCAACC	279	A	A	A	RM24259
PMS4761	Os_Chr09	14184199	14184224	(TA)13	(TA)12	(TA)11	(TA)6	(TA)13	(TA)9	(TA)13	(TA)13	(TA)7	(TA)8	(TA)11	-	Introns/Intergenic	-	-	ATCTGTAAACTTCCCGGTGC	GGTTCAGACCCTCTCAACGA	249	P	A	A	NA
PMS4762	Os_Chr09	14363396	14363413	(GTA)6	(GTA)5	(GTA)6	(GTA)6	(GTA)6	(GTA)6	(GTA)6	(GTA)6	(GTA)6	(GTA)6	(GTA)6	-	Introns/Intergenic	-	-	GTTTCTACGGCTACGCAAGG	TGCTCAGCTGGGTAAATGTG	273	P	A	A	NA
PMS4763	Os_Chr09	14565081	14565098	(AT)9	(AT)9	(AT)9	(AT)9	(AT)9	(AT)9	(AT)9	(AT)9	(AT)9	(AT)9	(AT)11	-	Introns/Intergenic	-	-	CAGTGCGTCGTAGGAAGAAA	TTTTCCACTTTGGCCTCATC	280	A	A	A	NA
PMS4764	Os_Chr09	14665638	14665651	(AG)7	(AG)7	(AG)7	(AG)7	(AG)7	(AG)7	(AG)7	(AG)7	(AG)7	(AG)7	(AG)6	-	Introns/Intergenic	-	-	GAGGAAGTCATGGGCTTGAA	AAGGGTAGAACCTGGGAGGA	108	A	A	A	NA
PMS4765	Os_Chr09	14697141	14697158	(TGA)6	(TGA)5	(TGA)5	(TGA)5	(TGA)5	(TGA)5	(TGA)6	(TGA)6	(TGA)6	(TGA)5	(TGA)5	LOC_Os09g24700	CDS	expressed protein	CODON_DELETION	GTCAGAGATTGCTTCTGGGC	GAAGACGAGCACAAGGGAAA	267	A	A	A	NA
PMS4766	Os_Chr09	14705827	14705856	(AG)15	(AG)12	(AG)15	(AG)15	(AG)15	(AG)15	(AG)15	(AG)15	(AG)15	(AG)15	(AG)15	-	Introns/Intergenic	-	-	AATATGGTGGCGCGTACATCC	TGATCGAGCCAACAACAACTGG	175	P	A	A	RM566
PMS4767	Os_Chr09	14812148	14812177	(AAT)10	(AAT)5	(AAT)10	(AAT)10	(AAT)10	(AAT)10	(AAT)10	(AAT)10	(AAT)10	(AAT)10	(AAT)10	LOC_Os09g24820	DRR	ZF-HD protein dimerisation region containing protein, expressed	-	GCAAAGAGCAAAGCGAAAGTACAAGC	ATGGAAGCAGCCTCACCTCTTGC	126	P	P	A	RM24312
PMS4768	Os_Chr09	14829727	14829746	(TA)10	(TA)9	(TA)10	(TA)10	(TA)10	(TA)10	(TA)10	(TA)10	(TA)10	(TA)10	(TA)10	-	Introns/Intergenic	-	-	CACCTTTCACCTGTCCTCTGTGG	ACCTAGCTGACCCACAACAATGC	250	P	A	A	RM24314
PMS4769	Os_Chr09	14917562	14917585	(GGA)8	(GGA)8	(GGA)8	(GGA)8	(GGA)8	(GGA)8	(GGA)8	(GGA)5	(GGA)8	(GGA)8	(GGA)8	LOC_Os09g24970	CDS	expressed protein	CODON_DELETION	CGTCGGATTACGGGTGCTACG	GGACGTTGACGAAGGTCTTGTGG	408	A	A	A	RM24323
PMS4770	Os_Chr09	14939672	14939689	(GCG)6	(GCG)6	(GCG)6	(GCG)6	(GCG)6	(GCG)6	(GCG)5	(GCG)6	(GCG)6	(GCG)6	(GCG)6	LOC_Os09g25000	CDS	spotted leaf 11, putative, expressed	CODON_CHANGE_PLUS_CODON_DELETION	CTCGAGCTCACCCTCGTC	ATGTTGCTCTGGAGGCTCTG	242	A	A	A	NA

**Polymorphic SSR (PMS) markers**	**Chromosomes**	**Start physical positions (bp)**	**End physical positions (bp)**	**Nipponbare**	**Kasalath**	**Nagina22**	**IR64**	**Pokkali**	**Bala**	**Tainung67**	**Azucena**	**Moroberekan**	***Oryza rufipogon***	***Oryza nivara***	**MSU gene locus IDs**	**Structural annotation**	**Functional annotation**	**SSR functional effects**	**Forward primers (5'-3')**	**Reverse primers (5'-3')**	**Amplified product size (bp)**	**Markers exhibiting polymorphism within *indica* rice**	**Transcrption factor genes-derived markers**	**Known cloned genes-derived markers**	**PMS markers corresponding with RM markers**
PMS4771	Os_Chr09	14944459	14944484	(AT)13	(AT)13	(AT)13	(AT)13	(AT)13	(AT)7	(AT)6	(AT)13	(AT)13	(AT)13	(AT)13	-	Introns/Intergenic	-	-	AGGTGTGTGTATAC TGGCCTTGC	CCCTATCCTTTGGCT TATTCACG	378	P	A	A	RM24327
PMS4772	Os_Chr09	14962340	14962357	(CGC)6	(CGC)6	(CGC)6	(CGC)6	(CGC)6	(CGC)6	(CGC)5	(CGC)6	(CGC)6	(CGC)6	(CGC)6	LOC_Os09g25040	CDS	joka8, putative, expressed	CODON_DELETION	ATGAATCAGTACGG CGGAAT	ACATGATGCTCCAGG GCTT	164	A	P	A	NA
PMS4773	Os_Chr09	15065367	15065384	(CT)9	(CT)9	(CT)9	(CT)9	(CT)9	(CT)9	(CT)9	(CT)9	(CT)9	(CT)9	(CT)8	-	Introns/Intergenic	-	-	GAACGGTTTGAGGA AGAAGAAGACG	ATCCATCCACGACAC ACCATCC	188	A	A	A	RM24334
PMS4774	Os_Chr09	15073240	15073253	(GA)7	(GA)7	(GA)7	(GA)7	(GA)7	(GA)7	(GA)6	(GA)7	(GA)7	(GA)7	(GA)7	-	Introns/Intergenic	-	-	TCTCGGCCCAAACT TAGAAA	CTCTCGCGGGTAGA GACAAG	228	A	A	A	NA
PMS4775	Os_Chr09	15118703	15118716	(GA)7	(GA)7	(GA)7	(GA)7	(GA)7	(GA)7	(GA)7	(GA)7	(GA)7	(GA)6	(GA)7	-	Introns/Intergenic	-	-	TCATTCACGTTTGTT CGCTC	CCACCTCCCTCTCGA TCTCT	239	A	A	A	NA
PMS4776	Os_Chr09	15160127	15160164	(AT)19	(AT)19	(AT)19	(AT)19	(AT)19	(AT)19	(AT)6	(AT)19	(AT)19	(AT)19	(AT)7	-	Introns/Intergenic	-	-	CCATTCTACTCTCT GCCTACCAACC	CCTCCATCCCAATAT ACCTATCATCC	443	A	A	A	RM24337
PMS4777	Os_Chr09	15171983	15172000	(GT)9	(GT)9	(GT)8	(GT)9	(GT)8	(GT)9	(GT)9	(GT)9	(GT)9	(GT)9	(GT)9	-	Introns/Intergenic	-	-	TAACTTTGTGGGTA AGGCGG	TAGGACTGGAGGGA GGGAGT	253	P	A	A	NA
PMS4778	Os_Chr09	15241720	15241740	(CGG)7	(CGG)7	(CGG)7	(CGG)7	(CGG)7	(CGG)7	(CGG)6	(CGG)7	(CGG)7	(CGG)7	(CGG)7	LOC_Os09g25430	CDS	ZOS9-07 - C2H2 zinc finger protein, expressed	CODON_DELETION	CCGGTGTAGCCTGC TTGATATGC	AGACTCGCTGGTGG TGGTGAGG	490	A	A	A	RM24341
PMS4779	Os_Chr09	15256269	15256280	(TG)6	(TG)6	(TG)7	(TG)7	(TG)7	(TG)7	(TG)6	(TG)6	(TG)6	(TG)6	(TG)7	LOC_Os09g25460	URR	transferase family protein, putative, expressed	-	CACCACCTCCACCT TCATCT	CTCCCTTCTCTGCAC ACACA	107	P	A	A	NA
PMS4780	Os_Chr09	15268165	15268178	(CA)7	(CA)7	(CA)7	(CA)7	(CA)7	(CA)8	(CA)6	(CA)7	(CA)7	(CA)7	(CA)8	-	Introns/Intergenic	-	-	TACACCGAAGGTCC CTCAAC	GCCTGCCCAGTCTA GAGAGA	219	P	A	A	NA
PMS4781	Os_Chr09	15269710	15269723	(GA)7	(GA)7	(GA)7	(GA)6	(GA)7	(GA)7	(GA)7	(GA)7	(GA)7	(GA)7	(GA)7	-	Introns/Intergenic	-	-	CCTTCGGCTTCCAC TTCTG	TTCTGACCCTGGTTT TGTCC	247	P	A	A	NA
PMS4782	Os_Chr09	15345471	15345491	(GGC)7	(GGC)7	(GGC)6	(GGC)7	(GGC)6	(GGC)7	(GGC)7	(GGC)7	(GGC)7	(GGC)6	(GGC)6	LOC_Os09g25570	CDS	aspartic proteinase, putative, expressed	CODON_DELETION	GACTGATCGATGAT GCTTTGAGG	CACGTAGTTCAGCGT CCGTAGC	285	P	A	A	RM24345
PMS4783	Os_Chr09	15400894	15400919	(CT)13	(CT)9	(CT)8	(CT)13	(CT)13	(CT)13	(CT)13	(CT)13	(CT)13	(CT)13	(CT)13	-	Introns/Intergenic	-	-	GTACTACTAGCTCG GCTGCTCTGC	GTAGTGGAGAGCGT GGACAGC	177	P	A	A	RM24349

Polymorphic SSR (PMS) markers	Chromosomes	Start physical positions (bp)	End physical positions (bp)	Nipponbare	Kasalath	Nagina22	IR64	Pokkali	Bala	Tainung67	Azucena	Moroberekan	*Oryza rufipogon*	*Oryza nivara*	MSU gene locus IDs	Structural annotation	Functional annotation	SSR functional effects	Forward primers (5'-3')	Reverse primers (5'-3')	Amplified product size (bp)	Markers exhibiting polymorphism within *indica* rice	Transcrption factor genes-derived markers	Known cloned genes-derived markers	PMS markers corresponding with RM markers
PMS4784	Os_Chr09	15456795	15456815	(ATA)7	(ATA)7	(ATA)7	(ATA)7	(ATA)7	(ATA)6	(ATA)7	(ATA)7	(ATA)7	(ATA)7	(ATA)7	-	Introns/Intergenic	-	-	ATATCCTCTTCCCTGCTCATGC	GAGACCTGATTAACAAGCACAAGG	286	P	A	A	RM24351
PMS4785	Os_Chr09	15476627	15476668	(TA)21	(TA)21	(TA)21	(TA)6	(TA)6	(TA)6	(TA)21	(TA)21	(TA)21	(TA)21	(TA)6	-	Introns/Intergenic	-	-	TCTGCACGCTATTTAGAGAAGTGG	TTCCTCCTCCTCTTCTTCTCTTCC	417	P	A	A	RM24352
PMS4786	Os_Chr09	15511478	15511493	(GC)8	(GC)8	(GC)8	(GC)8	(GC)8	(GC)8	(GC)8	(GC)8	(GC)8	(GC)8	(GC)6	-	Introns/Intergenic	-	-	GACCAAGCTGTGATCTGCAA	GTTCTTAAATCAGTGCCGCC	267	A	A	A	NA
PMS4787	Os_Chr09	15710511	15710524	(AT)7	(AT)7	(AT)7	(AT)6	(AT)7	(AT)7	(AT)7	(AT)7	(AT)7	(AT)7	(AT)7	-	Introns/Intergenic	-	-	CAGCACCCTATCCAACTGGT	GGAACACGCATAGGGAAAAA	209	P	A	A	NA
PMS4788	Os_Chr09	15752985	15753004	(CT)10	(CT)10	(CT)10	(CT)10	(CT)10	(CT)10	(CT)10	(CT)10	(CT)10	(CT)10	(CT)6	LOC_Os09g26144	URR	glutamate receptor, putative, expressed	-	AAATGGGCAAGTGTACTCACACG	GAGGAGGAGGAAGAGAGGATTCG	144	A	A	A	RM24371
PMS4789	Os_Chr09	15868547	15868570	(CTC)8	(CTC)8	(CTC)8	(CTC)8	(CTC)8	(CTC)8	(CTC)8	(CTC)8	(CTC)8	(CTC)8	(CTC)7	LOC_Os09g26260	CDS	AAA-type ATPase family protein, putative, expressed	CODON_DELETION	CACTCACTCACTCAGCTGTTTCTTGG	CTGATCAAGAACCGCAGGAACG	243	A	A	A	RM24383
PMS4790	Os_Chr09	15972274	15972288	(ATG)5	(ATG)7	(ATG)5	(ATG)5	(ATG)7	(ATG)5	(ATG)5	(ATG)5	(ATG)5	(ATG)6	(ATG)5	-	Introns/Intergenic	-	-	TGTCCAACTTTGATCGTCCA	CCCTCTGTCACATTGTAAGCG	188	P	A	A	NA
PMS4791	Os_Chr09	16075324	16075349	(GA)13	(GA)8	(GA)13	(GA)13	(GA)13	(GA)13	(GA)13	(GA)13	(GA)13	(GA)13	(GA)13	-	Introns/Intergenic	-	-	GCGAATGGAGTCGCTTGTGACC	ACCAGGCACGCCACTCAAATCC	168	P	A	A	RM24400
PMS4792	Os_Chr09	16137159	16137176	(TCT)6	(TCT)7	(TCT)7	(TCT)7	(TCT)7	(TCT)7	(TCT)6	(TCT)6	(TCT)6	(TCT)6	(TCT)7	-	Introns/Intergenic	-	-	TGCATTCATTGTTCGTTCGT	AGCTCAAAAGCCCTCCTCTC	251	A	A	A	NA
PMS4793	Os_Chr09	16146206	16146255	(AT)25	(AT)25	(AT)12	(AT)25	(AT)25	(AT)25	(AT)25	(AT)25	(AT)25	(AT)25	(AT)8	-	Introns/Intergenic	-	-	GGTTGTGATTGGTTGAGAGGA	TAATTCTGTTGGCCCCATTG	272	P	A	A	NA
PMS4794	Os_Chr09	16188261	16188281	(GCA)7	(GCA)6	(GCA)7	(GCA)7	(GCA)7	(GCA)7	(GCA)7	(GCA)7	(GCA)7	(GCA)7	(GCA)7	LOC_Os09g26660	CDS	OsrbohB|Osrboh7	CODON_DELETION	GTCGTCTTCCTCGTTTGTGTCC	GTACTGCACGAACTTCCACACG	372	P	A	P	RM24415
PMS4795	Os_Chr09	16198583	16198632	(TA)25	(TA)25	(TA)25	(TA)25	(TA)25	(TA)10	(TA)25	(TA)25	(TA)25	(TA)10	(TA)25	-	Introns/Intergenic	-	-	TATTGTATGCCACGGTCGAA	TTTTTCCGTCCCTCCTTTTT	181	P	A	A	NA
PMS4796	Os_Chr09	16201311	16201331	(CGT)7	(CGT)7	(CGT)7	(CGT)7	(CGT)7	(CGT)7	(CGT)7	(CGT)7	(CGT)7	(CGT)6	(CGT)6	LOC_Os09g26670	CDS	expressed protein	CODON_DELETION	GCCATACTGCTAGGGTATGACAAGC	GTGGACAGCGTCAGGAGATGC	425	A	A	A	RM24417

Polymorphic SSR (PMS) markers	Chromosomes	Start physical positions (bp)	End physical positions (bp)	Nipponbare	Kasalath	Nagina22	IR64	Pokkali	Bala	Tainung67	Azucena	Moroberekan	*Oryza rufipogon*	*Oryza nivara*	MSU gene locus IDs	Structural annotation	Functional annotation	SSR functional effects	Forward primers (5'-3')	Reverse primers (5'-3')	Amplified product size (bp)	Markers exhibiting polymorphism within *indica* rice	Transcrption factor genes-derived markers	Known cloned genes-derived markers	PMS markers corresponding with RM markers
PMS4797	Os_Chr09	16230761	16230778	(CCG)6	(CCG)7	(CCG)6	(CCG)6	(CCG)6	(CCG)6	(CCG)6	(CCG)6	(CCG)6	(CCG)6	(CCG)6	LOC_Os09g26710	CDS	major ampullate spidroin 2, putative, expressed	CODON_INSERTION	AACTCTCCTTCCTCGGGGT	CAGGCAGTACGATGCCTACA	230	P	A	A	NA
PMS4798	Os_Chr09	16272177	16272204	(TC)14	(TC)10	(TC)14	(TC)14	(TC)14	(TC)14	(TC)14	(TC)14	(TC)14	(TC)14	(TC)14	-	Introns/Intergenic	-	-	ATTTAACCGCGTTTGGTGCAATGG	GCGGGCAACCGGAGATTATGG	89	P	A	A	RM1189
PMS4799	Os_Chr09	16338358	16338373	(AG)8	(AG)8	(AG)8	(AG)8	(AG)7	(AG)8	(AG)8	(AG)8	(AG)8	(AG)8	(AG)8	LOC_Os09g26880	URR	OsALDH7|OsALDH7B6|ALDH7B7	-	GGAACTGGTGCTCCTTCCTC	AGTATCCCTTCCTTACCGCC	175	P	A	P	NA
PMS4800	Os_Chr09	16347159	16347173	(CCG)5	(CCG)6	(CCG)6	(CCG)6	(CCG)5	(CCG)6	(CCG)5	(CCG)5	(CCG)5	(CCG)6	(CCG)5	LOC_Os09g26900	URR	ctr copper transporter family protein, putative, expressed	-	AAAATCCCACCGGTCTCTTT	CCTTCCCCCAGTAGAAGGTC	188	P	A	A	NA
PMS4801	Os_Chr09	16495537	16495584	(CT)24	(CT)24	(CT)24	(CT)24	(CT)24	(CT)24	(CT)24	(CT)18	(CT)24	(CT)24	(CT)24	-	Introns/Intergenic	-	-	ACATCTCTCTCGCTCCCTTTCC	GGCTTTAAAGGGTGGATTGAACC	401	A	A	A	RM3492
PMS4802	Os_Chr09	16510239	16510259	(GCG)7	(GCG)7	(GCG)7	(GCG)7	(GCG)7	(GCG)7	(GCG)7	(GCG)7	(GCG)7	(GCG)6	(GCG)7	LOC_Os09g27150	CDS	protein kinase domain containing protein, expressed	CODON_DELETION	CGTCTGCTCGTGGAGGTAG	GTGACGGAGCTGCTGACC	180	A	A	A	NA
PMS4803	Os_Chr09	16520316	16520331	(GC)8	(GC)8	(GC)8	(GC)8	(GC)8	(GC)8	(GC)8	(GC)8	(GC)8	(GC)7	(GC)8	-	Introns/Intergenic	-	-	GGTTAGTGGCTGCAAAAGGA	CCCTGTTGACCAGTTGAGGT	158	A	A	A	NA
PMS4804	Os_Chr09	16701026	16701111	(TA)43	(TA)43	(TA)43	(TA)12	(TA)8	(TA)43	(TA)43	(TA)6	(TA)16	(TA)43	(TA)14	-	Introns/Intergenic	-	-	TTCACCAGAGGTCCCTCAAC	ATGTCATGTCAGCGAAACCA	218	P	A	A	NA
PMS4805	Os_Chr09	16774555	16774581	(GCT)9	(GCT)9	(GCT)9	(GCT)9	(GCT)7	(GCT)7	(GCT)9	(GCT)9	(GCT)9	(GCT)9	(GCT)9	-	Introns/Intergenic	-	-	AGGCGATCACTGCTAACGAC	GCTAGGGCCACTGCTAATTG	255	P	A	A	NA
PMS4806	Os_Chr09	16810130	16810193	(AT)32	(AT)32	(AT)32	(AT)7	(AT)32	(AT)32	(AT)32	(AT)32	(AT)32	(AT)32	(AT)32	-	Introns/Intergenic	-	-	AGCGGATAGCATCATTATATTT	TTGGAAGAAGAGCATAGAGTC	355	P	A	A	NA
PMS4807	Os_Chr09	16822256	16822277	(CT)11	(CT)11	(CT)11	(CT)11	(CT)11	(CT)11	(CT)10	(CT)11	(CT)11	(CT)11	(CT)11	LOC_Os09g27650	URR	ZOS9-14 - C2H2 zinc finger protein, expressed	-	AAACCTAAAGCCGGTGGAAAGC	TGGGCTGCTACAGAGACAACTCC	79	A	P	A	RM24458
PMS4808	Os_Chr09	16822908	16822925	(GGC)6	(GGC)5	(GGC)6	(GGC)6	(GGC)6	(GGC)6	(GGC)6	(GGC)5	(GGC)5	(GGC)6	(GGC)6	LOC_Os09g27650	URR	ZOS9-14 - C2H2 zinc finger protein, expressed	-	GCAATGGCCATGGTAAAAAT	ATCTGCAGGTTCTGGTCTCG	219	P	P	A	NA
PMS4809	Os_Chr09	16865352	16865367	(CG)8	(CG)8	(CG)8	(CG)8	(CG)8	(CG)8	(CG)8	(CG)8	(CG)8	(CG)8	(CG)6	-	Introns/Intergenic	-	-	TTAGCTAGCTCCAGCCTCCA	AAGCAGCTGTTTCTTGAGGG	238	A	A	A	NA

Polymorphic SSR (PMS) markers	Chromosomes	Start physical positions (bp)	End physical positions (bp)	Nipponbare	Kasalath	Nagina22	IR64	Pokkali	Bala	Tainung67	Azucena	Moroberekan	*Oryza rufipogon*	*Oryza nivara*	MSU gene locus IDs	Structural annotation	Functional annotation	SSR functional effects	Forward primers (5'-3')	Reverse primers (5'-3')	Amplified product size (bp)	Markers exhibiting polymorphism within *indica* rice	Transcrption factor genes-derived markers	Known cloned genes-derived markers	PMS markers corresponding with RM markers
PMS4810	Os_Chr09	16865705	16865734	(GGTTG)6	(GGTTG)6	(GGTTG)6	(GGTTG)6	(GGTTG)6	(GGTTG)6	(GGTTG)6	(GGTTG)6	(GGTTG)6	(GGTTG)5	(GGTTG)6	-	Introns/Intergenic	-	-	ACGCATACCGAATGTGACAG	GTTGGCACTCCCGATTAAAA	250	A	A	A	NA
PMS4811	Os_Chr09	17014148	17014165	(GCT)6	(GCT)6	(GCT)6	(GCT)6	(GCT)6	(GCT)6	(GCT)6	(GCT)6	(GCT)6	(GCT)6	(GCT)5	LOC_Os09g28019	CDS	nucleoporin p58/p45, putative, expressed	CODON_DELETION	CTCCTCCCACTTCGTGTTGT	CTTCGCCGTTTCAGTTCAA	158	A	A	A	NA
PMS4812	Os_Chr09	17099231	17099263	(TCC)11	(TCC)11	(TCC)11	(TCC)11	(TCC)11	(TCC)10	(TCC)11	(TCC)11	(TCC)11	(TCC)11	(TCC)11	-	Introns/Intergenic	-	-	CTCCTTCTTCCTCCTGGCTC	GTTTGGGACGAGTCGGGT	279	P	A	A	NA
PMS4813	Os_Chr09	17153454	17153467	(CT)7	(CT)7	(CT)7	(CT)7	(CT)7	(CT)6	(CT)7	(CT)7	(CT)7	(CT)7	(CT)7	-	Introns/Intergenic	-	-	ATCAATCCCCTCCGAAAAAG	GAAAGAGCGAAGGCGGTG	259	P	A	A	NA
PMS4814	Os_Chr09	17247719	17247730	(TA)6	(TA)6	(TA)6	(TA)6	(TA)6	(TA)6	(TA)6	(TA)6	(TA)6	(TA)7	(TA)6	-	Introns/Intergenic	-	-	GGCACCGCATTACTTTCCTA	TGAATCGGGTATGTAAACTTTTGA	271	A	A	A	NA
PMS4815	Os_Chr09	17335049	17335070	(GA)11	(GA)11	(GA)11	(GA)11	(GA)11	(GA)11	(GA)11	(GA)10	(GA)11	(GA)11	(GA)11	-	Introns/Intergenic	-	-	GGCTTGCTAGTAGAGCCTTCATGC	CCACTCTCGTCAAATTTCTTACGC	189	A	A	A	RM24483
PMS4816	Os_Chr09	17345945	17345982	(AG)19	(AG)19	(AG)19	(AG)19	(AG)19	(AG)17	(AG)19	(AG)19	(AG)19	(AG)19	(AG)19	-	Introns/Intergenic	-	-	GAAGGTCGAGCACGTTATAATTCC	GGATCGTACTGTGTGCTCTCTCC	100	P	A	A	RM24484
PMS4817	Os_Chr09	17357840	17357893	(TA)27	(TA)27	(TA)27	(TA)7	(TA)10	(TA)27	(TA)6	(TA)27	(TA)8	(TA)27	(TA)11	-	Introns/Intergenic	-	-	CAAGCAAGGGGATTCTATGC	TCTTGTTGTGTAATGAGGCCA	278	P	A	A	NA
PMS4818	Os_Chr09	17373516	17373549	(TA)17	(TA)17	(TA)11	(TA)7	(TA)7	(TA)17	(TA)17	(TA)17	(TA)17	(TA)17	(TA)8	-	Introns/Intergenic	-	-	CTAGATGGTTATCTCTCACGTTGC	CTAGGCCTAATTGACACTTTCAGG	109	P	A	A	RM24489
PMS4819	Os_Chr09	17406402	17406435	(AG)17	(AG)17	(AG)17	(AG)17	(AG)17	(AG)17	(AG)17	(AG)16	(AG)17	(AG)17	(AG)17	-	Introns/Intergenic	-	-	AATTGGCAGGCGAATAGTTAGG	AAAGTGGATGCCAGTTACTACCG	312	A	A	A	RM24495
PMS4820	Os_Chr09	17426771	17426798	(AT)14	(AT)13	(AT)14	(AT)14	(AT)14	(AT)13	(AT)14	(AT)14	(AT)14	(AT)14	(AT)14	-	Introns/Intergenic	-	-	AGCCGAACATGCCCAAAGAAGC	TGGTATGGCCCAAAGAATAGGAACG	182	P	A	A	RM24496
PMS4821	Os_Chr09	17660615	17660642	(TGAG)7	(TGAG)6	(TGAG)6	(TGAG)7	(TGAG)7	(TGAG)7	(TGAG)7	(TGAG)7	(TGAG)7	(TGAG)6	(TGAG)7	-	Introns/Intergenic	-	-	GTGTGAGAGAACTGGCACTACCG	ATCTGCGCTTGTTACGATCTGG	284	P	A	A	RM24506
PMS4822	Os_Chr09	17710067	17710118	(TA)26	(TA)26	(TA)26	(TA)26	(TA)26	(TA)26	(TA)26	(TA)26	(TA)11	(TA)26	(TA)26	-	Introns/Intergenic	-	-	AAAAAGTCAAAATCCGGACG	GCAAAGCACTACAATTTCAGCA	147	A	A	A	NA

**Polymorphic SSR (PMS) markers**	**Chromosomes**	**Start physical positions (bp)**	**End physical positions (bp)**	**Nipponbare**	**Kasalath**	**Nagina22**	**IR64**	**Pokkali**	**Bala**	**Tainung67**	**Azucena**	**Moroberekan**	***Oryza rufipogon***	***Oryza nivara***	**MSU gene locus IDs**	**Structural annotation**	**Functional annotation**	**SSR functional effects**	**Forward primers (5'-3')**	**Reverse primers (5'-3')**	**Amplified product size (bp)**	**Markers exhibiting polymorphism within *indica* rice**	**Transcrption factor genes-derived markers**	**Known cloned genes-derived markers**	**PMS markers corresponding with RM markers**
PMS4823	Os_Chr09	17712097	17712111	(CGA)5	(CGA)5	(CGA)5	(CGA)5	(CGA)5	(CGA)5	(CGA)5	(CGA)5	(CGA)6	(CGA)5	(CGA)5	-	Introns/Intergenic	-	-	TCATCTCTCTCTCACGGTCG	GAGAAGCTCGCCTCGGAT	267	A	A	A	NA
PMS4824	Os_Chr09	17791339	17791362	(CCG)8	(CCG)8	(CCG)8	(CCG)8	(CCG)8	(CCG)8	(CCG)5	(CCG)8	(CCG)8	(CCG)7	(CCG)8	LOC_Os09g29310	CDS	zinc finger, C3HC4 type domain containing protein, expressed	CODON_DELETION	TGATCCTCTCTTCCTCGGTCTCC	GTCTTCCACCAGGACTGCATCG	250	A	A	A	RM24517
PMS4825	Os_Chr09	17817422	17817441	(TA)10	(TA)10	(TA)10	(TA)10	(TA)6	(TA)10	(TA)10	(TA)7	(TA)10	(TA)10	(TA)10	-	Introns/Intergenic	-	-	GGATTATCCGCTAGGAAGAAGG	CAATTCCAGCACAAGAAAGACC	387	P	A	A	RM4405
PMS4826	Os_Chr09	17834917	17834934	(GCG)6	(GCG)7	(GCG)7	(GCG)7	(GCG)7	(GCG)7	(GCG)6	(GCG)6	(GCG)6	(GCG)6	(GCG)6	-	Introns/Intergenic	-	-	GGAACACCTAGCGACGATCC	AATCCACTCCACGTCTCCAC	128	A	A	A	NA
PMS4827	Os_Chr09	17903191	17903210	(AG)10	(AG)10	(AG)10	(AG)10	(AG)10	(AG)10	(AG)10	(AG)10	(AG)10	(AG)15	(AG)10	LOC_Os09g29460	URR	Oshox4	-	TACTGGACTCAACGCAAAGAACG	GGGATGAGAGTGTGGAGTGTGG	297	A	P	P	RM24526
PMS4828	Os_Chr09	17918819	17918839	(CCA)7	(CCA)7	(CCA)7	(CCA)7	(CCA)7	(CCA)7	(CCA)6	(CCA)7	(CCA)7	(CCA)7	(CCA)7	LOC_Os09g29470	URR	AGAP005770-PA, putative, expressed	-	CCTCTCTTTCTCGCCCGTTTCC	AGCGCCTTGAACTCCCTGTACG	172	A	A	A	RM24527
PMS4829	Os_Chr09	18056393	18056407	(GCG)5	(GCG)5	(GCG)5	(GCG)5	(GCG)5	(GCG)5	(GCG)5	(GCG)5	(GCG)5	(GCG)6	(GCG)5	-	Introns/Intergenic	-	-	ACCGTCACTGGTGACCTCTT	CATTACCATCTCCACCCGTC	227	A	A	A	NA
PMS4830	Os_Chr09	18057841	18057854	(CG)7	(CG)7	(CG)7	(CG)7	(CG)6	(CG)7	(CG)7	(CG)7	(CG)7	(CG)7	(CG)7	-	Introns/Intergenic	-	-	ATCGGCCTTCTTGGACTTTT	TTACCAACTGCAAACTGGGG	211	P	A	A	NA
PMS4831	Os_Chr09	18109746	18109766	(GGC)7	(GGC)7	(GGC)7	(GGC)7	(GGC)7	(GGC)7	(GGC)7	(GGC)7	(GGC)7	(GGC)7	(GGC)5	-	Introns/Intergenic	-	-	AGGGAGATGTCGTCCGTAGAGC	CTAATCGAGCCAACACGAACTCC	249	A	A	A	RM24536
PMS4832	Os_Chr09	18111644	18111658	(AGG)5	(AGG)6	(AGG)6	(AGG)6	(AGG)6	(AGG)6	(AGG)5	(AGG)5	(AGG)5	(AGG)5	(AGG)6	-	Introns/Intergenic	-	-	TATACGCGGTCACACTGAGC	TGAGGCAGCGTTCATACAAG	232	A	A	A	NA
PMS4833	Os_Chr09	18167175	18167240	(TA)33	(TA)33	(TA)33	(TA)33	(TA)10	(TA)33	(TA)33	(TA)9	(TA)7	(TA)33	(TA)9	-	Introns/Intergenic	-	-	ACTCTATTCCTCGATACCTTGG	GGAGCCCATACTCCATATAGC	173	P	A	A	RM24540
PMS4834	Os_Chr09	18222029	18222043	(TTA)5	(TTA)5	(TTA)5	(TTA)5	(TTA)5	(TTA)5	(TTA)5	(TTA)5	(TTA)5	(TTA)6	(TTA)5	-	Introns/Intergenic	-	-	AACAAGCTTCCACGACAAGG	AAATTCGTGGACACCAGGAC	208	A	A	A	NA
PMS4835	Os_Chr09	18313693	18313749	(ATA)19	(ATA)19	(ATA)19	(ATA)19	(ATA)19	(ATA)19	(ATA)19	(ATA)19	(ATA)5	(ATA)19	(ATA)18	-	Introns/Intergenic	-	-	AATGGAAGTAAGAGTGTGGTGAGG	GATACTGCTGGTGCTCTACTACAGG	443	A	A	A	RM24547

Polymorphic SSR (PMS) markers	Chromosomes	Start physical positions (bp)	End physical positions (bp)	Nipponbare	Kasalath	Nagina22	IR64	Pokkali	Bala	Tainung67	Azucena	Moroberekan	*Oryza rufipogon*	*Oryza nivara*	MSU gene locus IDs	Structural annotation	Functional annotation	SSR functional effects	Forward primers (5'-3')	Reverse primers (5'-3')	Amplified product size (bp)	Markers exhibiting polymorphism within *indica* rice	Transcrption factor genes-derived markers	Known cloned genes-derived markers	PMS markers corresponding with RM markers
PMS4836	Os_Chr09	18325568	18325605	(TA)19	(TA)19	(TA)8	(TA)6	(TA)19	(TA)19	(TA)19	(TA)10	(TA)19	(TA)19	(TA)19	-	Introns/Intergenic	-	-	CAAAACGAGATTGGATGGAA	TGCAGTCTTGTCCACTCCTG	246	P	A	A	NA
PMS4837	Os_Chr09	18441172	18441192	(GCG)7	(GCG)7	(GCG)7	(GCG)7	(GCG)7	(GCG)7	(GCG)7	(GCG)6	(GCG)7	(GCG)7	(GCG)7	LOC_Os09g30280	URR	glycosyl transferase 8 domain containing protein, putative, expressed	-	CACACAACACATCTCGCCTC	ACGAGAGGAGGAGCAGCAG	247	A	A	A	NA
PMS4838	Os_Chr09	18451486	18451581	(ATA)32	(ATA)32	(ATA)32	(ATA)32	(ATA)24	(ATA)32	(ATA)32	(ATA)32	(ATA)32	(ATA)32	(ATA)32	-	Introns/Intergenic	-	-	AGTTGAGTGGCAAACCACAGAGC	CTTCACTTGGGTTTGGGTGATGG	196	P	A	A	RM24559
PMS4839	Os_Chr09	18472880	18472894	(CAT)5	(CAT)5	(CAT)5	(CAT)10	(CAT)10	(CAT)10	(CAT)5	(CAT)5	(CAT)5	(CAT)5	(CAT)5	LOC_Os09g30350	CDS	expressed protein	CODON_INSERTION	CACTGCTGCCCTTTACCAAT	ATGGCGTTCACAAGGTTCTC	162	P	A	A	NA
PMS4840	Os_Chr09	18476120	18476157	(AG)19	(AG)19	(AG)19	(AG)19	(AG)19	(AG)19	(AG)19	(AG)19	(AG)19	(AG)19	(AG)18	-	Introns/Intergenic	-	-	CAAACACATCTCCTCGGAATCG	GGAGGTAGCCTGCACAGTAAGACC	267	A	A	A	RM24562
PMS4841	Os_Chr09	18520791	18520828	(TA)19	(TA)19	(TA)19	(TA)8	(TA)6	(TA)7	(TA)19	(TA)7	(TA)6	(TA)9	(TA)15	-	Introns/Intergenic	-	-	CAATTTGTGACACAGGTCGG	GAACCTGAATGCGAAATGCT	199	P	A	A	NA
PMS4842	Os_Chr09	18532250	18532287	(TA)19	(TA)19	(TA)19	(TA)8	(TA)6	(TA)7	(TA)19	(TA)7	(TA)6	(TA)9	(TA)15	-	Introns/Intergenic	-	-	CTATGCCCATTTCGATCGTAGCC	TGAATTCTTGCCCATTGGAACC	154	P	A	A	RM24566
PMS4843	Os_Chr09	18551306	18551323	(GCC)6	(GCC)6	(GCC)6	(GCC)6	(GCC)6	(GCC)6	(GCC)6	(GCC)6	(GCC)6	(GCC)6	(GCC)5	LOC_Os09g30454	URR	OsWAK87 - OsWAK receptor-like protein kinase, expressed	-	GCCAGGCTTCCATCTACCTA	ATCCGATTGGTTAGACGACG	122	A	A	A	NA
PMS4844	Os_Chr09	18573768	18573785	(GAG)6	(GAG)6	(GAG)6	(GAG)6	(GAG)6	(GAG)6	(GAG)6	(GAG)5	(GAG)6	(GAG)6	(GAG)6	LOC_Os09g30474	CDS	BPI/LBP family protein At3g20270 precursor, putative, expressed	CODON_DELETION	GACACGAGGAGGTCCTTGAC	CCCAATCCCTAGCCTCTCTC	186	A	A	A	NA
PMS4845	Os_Chr09	18611478	18611499	(CT)11	(CT)11	(CT)11	(CT)11	(CT)11	(CT)11	(CT)11	(CT)8	(CT)11	(CT)11	(CT)10	LOC_Os09g30486	DRR	fasciclin domain containing protein, expressed	-	TTCGATCCTACAAACATTGCC	ATTGGCTGGTAGCTTGATGC	180	A	A	A	NA
PMS4846	Os_Chr09	18622931	18622948	(CT)9	(CT)9	(CT)9	(CT)9	(CT)9	(CT)9	(CT)9	(CT)9	(CT)9	(CT)9	(CT)8	-	Introns/Intergenic	-	-	CTCTTCTTTCGCCAGCCTCT	CGATGACTGCAAAAGGACG	257	A	A	A	NA
PMS4847	Os_Chr09	18669334	18669351	(CCA)6	(CCA)6	(CCA)6	(CCA)6	(CCA)6	(CCA)6	(CCA)5	(CCA)6	(CCA)6	(CCA)5	(CCA)6	LOC_Os09g31031	URR	UbL404	-	TGGGATATTTCGCTTTCGTG	GATCTTGGCCTTGACGTTGT	239	A	A	P	NA
PMS4848	Os_Chr09	18772967	18772980	(CT)7	(CT)7	(CT)7	(CT)7	(CT)7	(CT)7	(CT)7	(CT)7	(CT)7	(CT)8	(CT)7	-	Introns/Intergenic	-	-	CAAAACATTAGCCCCTCCAA	GGATCCATGGGCTAATTCAA	145	A	A	A	NA

Polymorphic SSR (PMS) markers	Chromosomes	Start physical positions (bp)	End physical positions (bp)	Nipponbare	Kasalath	Nagina22	IR64	Pokkali	Bala	Tainung67	Azucena	Moroberekan	*Oryza rufipogon*	*Oryza nivara*	MSU gene locus IDs	Structural annotation	Functional annotation	SSR functional effects	Forward primers (5'-3')	Reverse primers (5'-3')	Amplified product size (bp)	Markers exhibiting polymorphism within *indica* rice	Transcrption factor genes-derived markers	Known cloned genes-derived markers	PMS markers corresponding with RM markers
PMS4849	Os_Chr09	18796008	18796059	(AT)26	(AT)26	(AT)6	(AT)15	(AT)15	(AT)26	(AT)26	(AT)26	(AT)26	(AT)26	(AT)16	-	Introns/Intergenic	-	-	CATATGCTTCCCAAGCTCCTAACC	GGGATACAAGCGAATGGATTACC	412	P	A	A	RM24570
PMS4850	Os_Chr09	18808951	18808965	(GTG)5	(GTG)5	(GTG)6	(GTG)5	(GTG)5	(GTG)5	(GTG)5	(GTG)5	(GTG)5	(GTG)5	(GTG)7	-	Introns/Intergenic	-	-	TTTGGTCAAGGCTGTGAGGT	CCCCCGTACTGCATAGATGT	256	P	A	A	NA
PMS4851	Os_Chr09	18815551	18815575	(TGGCG)5	(TGGCG)5	(TGGCG)5	(TGGCG)5	(TGGCG)5	(TGGCG)5	(TGGCG)5	(TGGCG)5	(TGGCG)5	(TGGCG)6	(TGGCG)5	-	Introns/Intergenic	-	-	CCATCGAGAAGTTCCTCCAG	TCAGGAATCGAAGGAAATGG	209	A	A	A	NA
PMS4852	Os_Chr09	18835127	18835146	(TA)10	(TA)10	(TA)10	(TA)10	(TA)10	(TA)6	(TA)10	(TA)10	(TA)10	(TA)10	(TA)6	-	Introns/Intergenic	-	-	ACGTCTTCCTTTACGAGTACGG	GAAGACCGTGCTAAGTTTGACC	285	P	A	A	RM24573
PMS4853	Os_Chr09	18850222	18850242	(CGG)7	(CGG)7	(CGG)7	(CGG)7	(CGG)7	(CGG)5	(CGG)5	(CGG)5	(CGG)7	(CGG)7	(CGG)7	LOC_Os09g31360	CDS	expressed protein	CODON_CHANGE_PLUS_CODON_DELETION	AGGACGCGAGGAGGAAGATGG	TACCTTGCAACAAGAGGCACTCG	471	P	A	A	RM24574
PMS4854	Os_Chr09	18899111	18899128	(AT)9	(AT)9	(AT)8	(AT)6	(AT)6	(AT)9	(AT)9	(AT)9	(AT)9	(AT)9	(AT)6	-	Introns/Intergenic	-	-	CTCATCCCTCTAAACTCTCAATCC	TCGTACTGTCGCAATAGTTTGG	218	P	A	A	RM24576
PMS4855	Os_Chr09	19095801	19095834	(TA)17	(TA)17	(TA)6	(TA)17	(TA)17	(TA)17	(TA)17	(TA)17	(TA)7	(TA)17	(TA)17	-	Introns/Intergenic	-	-	TATAGAACCGGAGGGAGTACTGG	GACGGCTTAATTAGTTCCAACG	187	P	A	A	RM24579
PMS4856	Os_Chr09	19159099	19159122	(CGC)8	(CGC)7	(CGC)8	(CGC)8	(CGC)8	(CGC)7	(CGC)8	(CGC)8	(CGC)8	(CGC)8	(CGC)8	LOC_Os09g32095	CDS	expressed protein	CODON_DELETION	CGCGAGTTAGTTGATGGCATACG	CTCCCTTCCCGAATCACACTAGC	399	P	A	A	RM24582
PMS4857	Os_Chr09	19172079	19172092	(GA)7	(GA)7	(GA)7	(GA)7	(GA)7	(GA)7	(GA)7	(GA)6	(GA)7	(GA)7	(GA)7	-	Introns/Intergenic	-	-	GCCACAGAAGGAGAAGGTGA	GGGCCTCCTAATCTACCCAC	182	A	A	A	NA
PMS4858	Os_Chr09	19216051	19216068	(GGA)6	(GGA)6	(GGA)6	(GGA)6	(GGA)6	(GGA)6	(GGA)6	(GGA)5	(GGA)6	(GGA)6	(GGA)6	LOC_Os09g32200	CDS	mitochondrial carrier protein, putative, expressed	CODON_DELETION	CTCCCTCCCAAATCCAAAG	CACGAGCAGCTCCTTGTAAC	231	A	A	A	NA
PMS4859	Os_Chr09	19320405	19320426	(TC)11	(TC)12	(TC)11	(TC)11	(TC)11	(TC)12	(TC)11	(TC)11	(TC)11	(TC)13	(TC)12	-	Introns/Intergenic	-	-	AACTCAGCCTCTCTGTCCTCTTCC	CCAGGGTGCAATTGTAGTGAGC	155	P	A	A	RM24597
PMS4860	Os_Chr09	19430934	19430954	(GCG)7	(GCG)7	(GCG)7	(GCG)7	(GCG)7	(GCG)7	(GCG)7	(GCG)5	(GCG)7	(GCG)7	(GCG)7	-	Introns/Intergenic	-	-	CCAATCCAAGGAGAAGCACACC	CGGTGGGCTTTACTCCAAACC	443	A	A	A	RM24603
PMS4861	Os_Chr09	19441785	19441798	(GA)7	(GA)7	(GA)7	(GA)7	(GA)7	(GA)7	(GA)7	(GA)7	(GA)7	(GA)12	(GA)7	-	Introns/Intergenic	-	-	CACACCATCTCGTTCCCTTT	TTAATGTTGCACCAACGGTC	279	A	A	A	NA

Polymorphic SSR (PMS) markers	Chromosomes	Start physical positions (bp)	End physical positions (bp)	Nipponbare	Kasalath	Nagina22	IR64	Pokkali	Bala	Tainung67	Azucena	Moroberekan	*Oryza rufipogon*	*Oryza nivara*	MSU gene locus IDs	Structural annotation	Functional annotation	SSR functional effects	Forward primers (5'-3')	Reverse primers (5'-3')	Amplified product size (bp)	Markers exhibiting polymorphism within *indica* rice	Transcrption factor genes-derived markers	Known cloned genes-derived markers	PMS markers corresponding with RM markers
PMS4862	Os_Chr09	19519913	19519933	(CGT)7	(CGT)7	(CGT)7	(CGT)7	(CGT)7	(CGT)7	(CGT)7	(CGT)7	(CGT)7	(CGT)7	(CGT)6	LOC_Os09g32730	CDS	zinc finger, C3HC4 type domain containing protein, expressed	CODON_DELETION	GATGACGTCGAGCAGCGT	CCTTCTCCAGGTGAGTGAGG	247	A	A	A	NA
PMS4863	Os_Chr09	19581152	19581227	(TA)38	(TA)38	(TA)6	(TA)38	(TA)6	(TA)38	(TA)7	(TA)38	(TA)6	(TA)38	(TA)8	-	Introns/Intergenic	-	-	CACCTTGGCCAACTAACTAATCG	GGGCAAGAGGAATTCACAACC	225	P	A	A	RM24616
PMS4864	Os_Chr09	19620661	19620680	(CT)10	(CT)10	(CT)10	(CT)10	(CT)10	(CT)9	(CT)10	(CT)10	(CT)10	(CT)10	(CT)9	LOC_Os09g32930	URR	PAIR2	-	CAAATCTCGGAAGCAGGAAACC	GAACCCACCATTTCGTCTCTACG	355	P	A	P	RM24620
PMS4865	Os_Chr09	19650220	19650235	(AT)8	(AT)7	(AT)8	(AT)8	(AT)8	(AT)7	(AT)8	(AT)8	(AT)8	(AT)8	(AT)8	-	Introns/Intergenic	-	-	ACATCGAACGGACGAATACC	CACGAGATATCTGCAGGCAA	209	P	A	A	NA
PMS4866	Os_Chr09	19709842	19709895	(TTA)18	(TTA)18	(TTA)10	(TTA)11	(TTA)18	(TTA)18	(TTA)6	(TTA)18	(TTA)18	(TTA)18	(TTA)18	-	Introns/Intergenic	-	-	ACCACCTATACACGAGCGGT	AAATGGTGCTGATGACTTCTGA	280	P	A	A	NA
PMS4867	Os_Chr09	19736067	19736080	(AT)7	(AT)7	(AT)7	(AT)7	(AT)7	(AT)7	(AT)7	(AT)7	(AT)7	(AT)6	(AT)7	-	Introns/Intergenic	-	-	GTCACTTCGGTGTCTCCTCC	TTAAACTGCTAAACGGTGCG	189	A	A	A	NA
PMS4868	Os_Chr09	19752747	19752764	(CT)9	(CT)8	(CT)7	(CT)14	(CT)9	(CT)8	(CT)9	(CT)9	(CT)9	(CT)14	(CT)9	LOC_Os09g33500	URR	transketolase, putative, expressed	-	AATCTCGCACAGAGGCGTAT	CCTCGCGAATGGGATAGAG	101	P	A	A	NA
PMS4869	Os_Chr09	19780588	19780599	(CG)6	(CG)6	(CG)6	(CG)6	(CG)7	(CG)6	(CG)6	(CG)6	(CG)6	(CG)6	(CG)6	LOC_Os09g33530	URR	expressed protein	-	GCCTCTGGTCTTCTCCTTGA	TGCACTAGGCCGGTTTACTT	121	P	A	A	NA
PMS4870	Os_Chr09	19782050	19782067	(GAG)6	(GAG)6	(GAG)6	(GAG)6	(GAG)6	(GAG)9	(GAG)6	(GAG)6	(GAG)6	(GAG)9	(GAG)6	-	Introns/Intergenic	-	-	CTTCAAGAGGGAGGAGGAGG	GGGAGGCATGGTACAAGGTA	263	P	A	A	NA
PMS4871	Os_Chr09	19795922	19795943	(AT)11	(AT)11	(AT)11	(AT)11	(AT)11	(AT)10	(AT)11	(AT)8	(AT)11	(AT)11	(AT)8	-	Introns/Intergenic	-	-	TCGGTGAAGCAACGGCTACGG	GGCAGCGAGAAAGGAATAAGTTCACC	478	P	A	A	RM24632
PMS4872	Os_Chr09	19831090	19831107	(CCA)6	(CCA)6	(CCA)6	(CCA)5	(CCA)6	(CCA)6	(CCA)6	(CCA)6	(CCA)6	(CCA)6	(CCA)6	LOC_Os09g33600	URR	ARF GTPase-activating domain-containing protein, putative, expressed	-	CCCTACCACCACAACCTCAT	CTGGAGGAGCTGCTCTGACT	152	P	A	A	NA
PMS4873	Os_Chr09	19878504	19878527	(CGG)8	(CGG)8	(CGG)5	(CGG)7	(CGG)8	(CGG)8	(CGG)8	(CGG)8	(CGG)8	(CGG)8	(CGG)8	LOC_Os09g33670	CDS	zinc finger, C3HC4 type domain containing protein, expressed	CODON_CHANGE_PLUS_CODON_DELETION	TCCTCCTTAACCCAACCAAA	GCTGACCTAGGTGGAACTCG	268	P	A	A	NA
PMS4874	Os_Chr09	19905481	19905500	(TA)10	(TA)10	(TA)10	(TA)6	(TA)10	(TA)10	(TA)10	(TA)10	(TA)10	(TA)10	(TA)10	-	Introns/Intergenic	-	-	TCATTGGGCGTGACTACACAGC	CAACCGTGTGAACAGTGGAGTGG	603	P	A	A	RM24641

Polymorphic SSR (PMS) markers	Chromosomes	Start physical positions (bp)	End physical positions (bp)	Nipponbare	Kasalath	Nagina22	IR64	Pokkali	Bala	Tainung67	Azucena	Moroberekan	*Oryza rufipogon*	*Oryza nivara*	MSU gene locus IDs	Structural annotation	Functional annotation	SSR functional effects	Forward primers (5'-3')	Reverse primers (5'-3')	Amplified product size (bp)	Markers exhibiting polymorphism within *indica* rice	Transcrption factor genes-derived markers	Known cloned genes-derived markers	PMS markers corresponding with RM markers
PMS4875	Os_Chr09	19938363	19938378	(CG)8	(CG)8	(CG)8	(CG)6	(CG)8	(CG)8	(CG)8	(CG)6	(CG)8	(CG)8	(CG)8	-	Introns/Intergenic	-	-	CCCTGTTGACTGGTTGAGGT	TGCAAAAGAGTAGCGACGTG	138	P	A	A	NA
PMS4876	Os_Chr09	20073592	20073609	(CGC)6	(CGC)6	(CGC)6	(CGC)6	(CGC)6	(CGC)6	(CGC)6	(CGC)6	(CGC)6	(CGC)6	(CGC)5	LOC_Os09g33992	CDS	expressed protein	CODON_DELETION	CGGAGCATCTCCTCGTACTC	ACGGTCCATCTTGCTCTGAC	200	A	A	A	NA
PMS4877	Os_Chr09	20142011	20142028	(ACG)6	(ACG)5	(ACG)5	(ACG)6	(ACG)6	(ACG)5	(ACG)6	(ACG)6	(ACG)6	(ACG)6	(ACG)6	-	Introns/Intergenic	-	-	AAGCACTCACGAAGTCACGA	TCCTTTGTTTGGTCGCTTTC	193	P	A	A	NA
PMS4878	Os_Chr09	20143892	20143919	(TA)14	(TA)14	(TA)14	(TA)14	(TA)14	(TA)14	(TA)10	(TA)14	(TA)14	(TA)14	(TA)14	-	Introns/Intergenic	-	-	GAATCGTTTTCACGGCTCAT	CGTCATCTGCAAAACACGAC	229	A	A	A	NA
PMS4879	Os_Chr09	20174851	20174870	(CT)10	(CT)10	(CT)10	(CT)10	(CT)10	(CT)10	(CT)10	(CT)9	(CT)10	(CT)10	(CT)10	-	Introns/Intergenic	-	-	GTACTCTCGCCGTTCACAACTCC	TTAGTGACCGGGATGACACAGC	408	A	A	A	RM201
PMS4880	Os_Chr09	20196773	20196787	(CGG)5	(CGG)5	(CGG)5	(CGG)5	(CGG)5	(CGG)6	(CGG)5	(CGG)5	(CGG)5	(CGG)5	(CGG)5	LOC_Os09g34214	CDS	UDP-glucoronosyl and UDP-glucosyl transferase domain containing protein, expressed	CODON_CHANGE_PLUS_CODON_INSERTION	TGCGCAGTTCAGACTTTCAG	GAGGATGTAGCGGGTGACAG	201	P	A	A	NA
PMS4881	Os_Chr09	20198169	20198286	(TA)59	(TA)59	(TA)59	(TA)11	(TA)9	(TA)59	(TA)10	(TA)59	(TA)59	(TA)59	(TA)59	-	Introns/Intergenic	-	-	TTGCTAGGTTAGCATCCGGTACG	TGGTCTTTGCGAATCTGAATCC	599	P	A	A	RM24654
PMS4882	Os_Chr09	20215715	20215732	(GCG)6	(GCG)5	(GCG)6	(GCG)6	(GCG)6	(GCG)5	(GCG)6	(GCG)6	(GCG)6	(GCG)6	(GCG)6	LOC_Os09g34230	CDS	UDP-glucoronosyl/UDP-glucosyl transferase, putative, expressed	CODON_CHANGE_PLUS_CODON_DELETION	AGGTACATCGAGGACGTGTG	CCTCATGAACTCCTTGCTCC	144	P	A	A	NA
PMS4883	Os_Chr09	20236946	20236963	(TG)9	(TG)8	(TG)9	(TG)9	(TG)9	(TG)9	(TG)9	(TG)9	(TG)9	(TG)9	(TG)9	-	Introns/Intergenic	-	-	ATACTCGTTGCTTGTTGGGG	CAAGACGGGAGATTTTGGAA	246	P	A	A	NA
PMS4884	Os_Chr09	20260912	20260935	(ATCC)6	(ATCC)5	(ATCC)6	(ATCC)6	(ATCC)6	(ATCC)6	(ATCC)6	(ATCC)6	(ATCC)6	(ATCC)5	(ATCC)5	-	Introns/Intergenic	-	-	CACAAACCGGCAATCTCTCTCC	GTGATCATGGCAAATCACTGACC	349	P	A	A	RM24661
PMS4885	Os_Chr09	20278199	20278216	(TA)9	(TA)9	(TA)9	(TA)9	(TA)9	(TA)9	(TA)7	(TA)9	(TA)9	(TA)9	(TA)9	-	Introns/Intergenic	-	-	CAAACAGGCCCACCTAAAGT	TCCCGCTATACGCTTCTCTC	262	A	A	A	NA
PMS4886	Os_Chr09	20283403	20283414	(TA)6	(TA)8	(TA)6	(TA)6	(TA)6	(TA)6	(TA)6	(TA)6	(TA)6	(TA)8	(TA)8	-	Introns/Intergenic	-	-	CCAATACCAAGGTCTCGCAT	GTTTGTAACACTCCCGCGTT	216	P	A	A	NA
PMS4887	Os_Chr09	20285200	20285213	(CA)7	(CA)7	(CA)7	(CA)6	(CA)6	(CA)7	(CA)7	(CA)7	(CA)7	(CA)7	(CA)7	-	Introns/Intergenic	-	-	CAGACAGGAGTGGCAAAATG	TGGAAATCATGGAAGCACAA	202	P	A	A	NA

Polymorphic SSR (PMS) markers	Chromosomes	Start physical positions (bp)	End physical positions (bp)	Nipponbare	Kasalath	Nagina22	IR64	Pokkali	Bala	Tainung67	Azucena	Moroberekan	*Oryza rufipogon*	*Oryza nivara*	MSU gene locus IDs	Structural annotation	Functional annotation	SSR functional effects	Forward primers (5'-3')	Reverse primers (5'-3')	Amplified product size (bp)	Markers exhibiting polymorphism within *indica* rice	Transcrption factor genes-derived markers	Known cloned genes-derived markers	PMS markers corresponding with RM markers
PMS4888	Os_Chr09	20318635	20318712	(TA)39	(TA)39	(TA)9	(TA)9	(TA)9	(TA)9	(TA)7	(TA)13	(TA)17	(TA)8	(TA)9	-	Introns/Intergenic	-	-	GTGATTGCCATTGATCAGCTTGG	GCAACCGATTCCCGTATAACAATACC	321	P	A	A	RM24663
PMS4889	Os_Chr09	20367443	20367474	(GA)16	(GA)14	(GA)16	(GA)14	(GA)16	(GA)14	(GA)16	(GA)16	(GA)16	(GA)16	(GA)16	LOC_Os09g34960	URR	hydroxymethylglutaryl-CoA synthase, putative, expressed	-	CTAGTGCACACGGGCGACAAGC	CTCCAGATGAGACGAGACCACACG	158	P	A	A	RM24666
PMS4890	Os_Chr09	20447276	20447309	(TA)17	(TA)17	(TA)17	(TA)17	(TA)17	(TA)17	(TA)9	(TA)17	(TA)17	(TA)8	(TA)17	-	Introns/Intergenic	-	-	GAGACGAGTTGAGTTGAATCGTGACC	TTGTTGTCCTGCATATCACTGCATCC	271	A	A	A	RM24675
PMS4891	Os_Chr09	20528449	20528496	(TA)24	(TA)24	(TA)6	(TA)24	(TA)8	(TA)13	(TA)24	(TA)24	(TA)24	(TA)24	(TA)8	LOC_Os09g35690	DRR	zinc RING finger protein, putative, expressed	-	GTGAAACCCTTACAGACACACC	GTGTGTTACTACCTCCATTTCAGG	173	P	A	A	RM24681
PMS4892	Os_Chr09	20532664	20532739	(AT)38	(AT)38	(AT)8	(AT)8	(AT)38	(AT)8	(AT)38	(AT)38	(AT)7	(AT)8	(AT)38	-	Introns/Intergenic	-	-	ACAGAACAGGATAGCAACCAGAGC	AGAGGGATGGCAATTTGAAGG	405	P	A	A	RM24682
PMS4893	Os_Chr09	20727777	20727794	(GCG)6	(GCG)6	(GCG)6	(GCG)6	(GCG)6	(GCG)6	(GCG)5	(GCG)6	(GCG)6	(GCG)6	(GCG)6	LOC_Os09g35970	CDS	6-phosphogluconolactonase, putative, expressed	CODON_DELETION	TGTACTTCGCCAGGGACAC	CTCCCGTACCCTCGTCCT	239	A	A	A	NA
PMS4894	Os_Chr09	20794748	20794771	(TCT)8	(TCT)8	(TCT)8	(TCT)8	(TCT)8	(TCT)5	(TCT)8	(TCT)8	(TCT)8	(TCT)8	(TCT)5	-	Introns/Intergenic	-	-	TCTTACGCTGTTTTGGTCCC	AAACCACGCCACATCTAAGC	214	P	A	A	NA
PMS4895	Os_Chr09	20839891	20839923	(GAA)11	(GAA)11	(GAA)11	(GAA)11	(GAA)11	(GAA)10	(GAA)11	(GAA)11	(GAA)11	(GAA)11	(GAA)11	LOC_Os09g36160	URR	LRP1, putative, expressed	-	GTTGTCTCGGCTGTGGCT	TTCGGTTTCTTGATCGGTTC	172	P	P	A	NA
PMS4896	Os_Chr09	20880955	20880972	(CGC)6	(CGC)6	(CGC)6	(CGC)6	(CGC)6	(CGC)5	(CGC)6	(CGC)6	(CGC)6	(CGC)6	(CGC)6	LOC_Os09g36210	CDS	harpin-induced protein 1 domain containing protein, expressed	CODON_DELETION	TTCCACCACAAAATCGATCA	ATGGGCAGGGTCAAGTACAG	216	P	A	A	NA
PMS4897	Os_Chr09	20902767	20902780	(TC)7	(TC)7	(TC)7	(TC)7	(TC)7	(TC)7	(TC)7	(TC)7	(TC)7	(TC)7	(TC)6	-	Introns/Intergenic	-	-	TACTGCAACATGCGGATAGC	CATGTGGAGCATGACCTGAC	269	A	A	A	NA
PMS4898	Os_Chr09	20942557	20942574	(CCT)6	(CCT)6	(CCT)6	(CCT)6	(CCT)6	(CCT)6	(CCT)6	(CCT)6	(CCT)6	(CCT)5	(CCT)6	LOC_Os09g36290	CDS	Ser/Thr protein phosphatase family protein, putative, expressed	CODON_DELETION	TAAACAAAACCGCATCCACA	CTTCTCCTTCCTCCCTGCTC	181	A	A	A	NA
PMS4899	Os_Chr09	21003974	21003999	(AT)13	(AT)13	(AT)13	(AT)9	(AT)13	(AT)13	(AT)13	(AT)13	(AT)13	(AT)13	(AT)6	-	Introns/Intergenic	-	-	TTATTGTCCATGCGGTACAACG	CAACCACCTCATCACCAACTCC	199	P	A	A	RM1553
PMS4900	Os_Chr09	21045327	21045356	(TA)15	(TA)15	(TA)6	(TA)15	(TA)15	(TA)14	(TA)15	(TA)15	(TA)15	(TA)15	(TA)11	-	Introns/Intergenic	-	-	CCATGGAGCACCAAACAAAT	TGCTCCACCAATTAAGTCCA	105	P	A	A	NA

Polymorphic SSR (PMS) markers	Chromosomes	Start physical positions (bp)	End physical positions (bp)	Nipponbare	Kasalath	Nagina22	IR64	Pokkali	Bala	Tainung67	Azucena	Moroberekan	*Oryza rufipogon*	*Oryza nivara*	MSU gene locus IDs	Structural annotation	Functional annotation	SSR functional effects	Forward primers (5'-3')	Reverse primers (5'-3')	Amplified product size (bp)	Markers exhibiting polymorphism within *indica* rice	Transcrption factor genes-derived markers	Known cloned genes-derived markers	PMS markers corresponding with RM markers
PMS4901	Os_Chr09	21086981	21087002	(CT)11	(CT)11	(CT)11	(CT)11	(CT)11	(CT)10	(CT)11	(CT)11	(CT)11	(CT)11	(CT)11	-	Introns/Intergenic	-	-	ACACATTTGGCAGATGAACAGG	CTAACCGCTAAATGCCTTAACTCG	242	P	A	A	RM24708
PMS4902	Os_Chr09	21093449	21093476	(GA)14	(GA)14	(GA)14	(GA)14	(GA)14	(GA)14	(GA)14	(GA)8	(GA)14	(GA)14	(GA)14	-	Introns/Intergenic	-	-	TGAAAAGTTAGCAATGTAAAGAACACA	AAGGCTACGACTACTCGCCA	256	A	A	A	NA
PMS4903	Os_Chr09	21095357	21095380	(GCA)8	(GCA)8	(GCA)8	(GCA)8	(GCA)8	(GCA)8	(GCA)5	(GCA)8	(GCA)8	(GCA)8	(GCA)8	-	Introns/Intergenic	-	-	GCGATAGAGAAGATGATGAAGACG	ACAGAACGACGAAGAACAATCG	182	A	A	A	RM24711
PMS4904	Os_Chr09	21123411	21123470	(TA)30	(TA)30	(TA)30	(TA)30	(TA)7	(TA)30	(TA)7	(TA)30	(TA)12	(TA)30	(TA)7	-	Introns/Intergenic	-	-	TGCTTTTAAGAGAGATGAGGCA	GACTGTTTCCAAGGATTCGG	258	P	A	A	NA
PMS4905	Os_Chr09	21189620	21189651	(TC)16	(TC)16	(TC)16	(TC)17	(TC)16	(TC)14	(TC)16	(TC)17	(TC)17	(TC)16	(TC)16	LOC_Os09g36730	DRR	MYB family transcription factor, putative, expressed	-	GAGCAGCAAGAGCAGCAGAGG	CATGCTCGACTTCAGAAGCTTGG	174	P	P	A	RM215
PMS4906	Os_Chr09	21190712	21190732	(TGC)7	(TGC)5	(TGC)7	(TGC)7	(TGC)6	(TGC)7	(TGC)7	(TGC)7	(TGC)7	(TGC)6	(TGC)7	LOC_Os09g36730	URR	MYB family transcription factor, putative, expressed	-	CTCGGTGGGTTGTTTCAGTGG	AAGCTGGTAATCCAGTGATGATGC	196	P	P	A	RM24716
PMS4907	Os_Chr09	21267450	21267473	(CCG)8	(CCG)8	(CCG)8	(CCG)8	(CCG)8	(CCG)8	(CCG)8	(CCG)8	(CCG)8	(CCG)8	(CCG)6	LOC_Os09g36870	CDS	plant protein of unknown function domain containing protein, expressed	CODON_DELETION	ACGAGGAGGAGGTCGTAGTAGACG	CGCAGAACAAGAGCCTGTACG	382	A	A	A	RM24722
PMS4908	Os_Chr09	21291982	21292002	(ACT)7	(ACT)6	(ACT)7	(ACT)7	(ACT)7	(ACT)7	(ACT)7	(ACT)7	(ACT)7	(ACT)7	(ACT)5	LOC_Os09g36910	URR	bZIP transcription factor domain containing protein, expressed	-	ACCAGTGAGCAACCCAGTGT	CCTCTTTTGAGCAAGCAAGC	207	P	P	A	NA
PMS4909	Os_Chr09	21292112	21292132	(GCG)7	(GCG)6	(GCG)5	(GCG)7	(GCG)6	(GCG)7	(GCG)7	(GCG)7	(GCG)7	(GCG)7	(GCG)6	LOC_Os09g36910	URR	bZIP transcription factor domain containing protein, expressed	-	GCTTGCTTGCTCAAAAGAGG	CACATGTCCTCGTCGTCCT	117	P	P	A	NA
PMS4910	Os_Chr09	21313259	21313276	(GGC)6	(GGC)6	(GGC)6	(GGC)6	(GGC)6	(GGC)6	(GGC)6	(GGC)6	(GGC)6	(GGC)5	(GGC)6	LOC_Os09g36940	CDS	plastocyanin-like domain containing protein, putative, expressed	CODON_DELETION	CAAGAAACCTGCACCATCAA	ACTTCATCTGCAACGTCAGC	211	A	P	A	NA
PMS4911	Os_Chr09	21313746	21313763	(CCG)6	(CCG)6	(CCG)6	(CCG)6	(CCG)6	(CCG)6	(CCG)6	(CCG)6	(CCG)6	(CCG)5	(CCG)6	LOC_Os09g36940	CDS	plastocyanin-like domain containing protein, putative, expressed	CODON_DELETION	ACTGACCTAGAGTGTCGCCG	TTCCCAAATGCAAGAACACA	259	A	P	A	NA
PMS4912	Os_Chr09	21401470	21401483	(AT)7	(AT)7	(AT)7	(AT)8	(AT)7	(AT)7	(AT)7	(AT)7	(AT)7	(AT)7	(AT)7	-	Introns/Intergenic	-	-	AGCAGAACACGATCCCTGAC	TGACCGTTCGTCTTATTCAAAA	269	P	A	A	NA
PMS4913	Os_Chr09	21419324	21419365	(AT)21	(AT)21	(AT)11	(AT)21	(AT)8	(AT)6	(AT)8	(AT)21	(AT)21	(AT)8	(AT)8	-	Introns/Intergenic	-	-	TCACGCATCATAAGGGCATA	CATCATGGAGCACCAAACAA	244	P	A	A	NA

Polymorphic SSR (PMS) markers	Chromosomes	Start physical positions (bp)	End physical positions (bp)	Nipponbare	Kasalath	Nagina22	IR64	Pokkali	Bala	Tainung67	Azucena	Moroberekan	*Oryza rufipogon*	*Oryza nivara*	MSU gene locus IDs	Structural annotation	Functional annotation	SSR functional effects	Forward primers (5'-3')	Reverse primers (5'-3')	Amplified product size (bp)	Markers exhibiting polymorphism within *indica* rice	Transcrption factor genes-derived markers	Known cloned genes-derived markers	PMS markers corresponding with RM markers
PMS4914	Os_Chr09	21426528	21426539	(TC)6	(TC)12	(TC)6	(TC)6	(TC)6	(TC)6	(TC)6	(TC)6	(TC)6	(TC)6	(TC)6	-	Introns/Intergenic	-	-	TGCTCTCTCCTTCTTCCTCG	AAAAAGTGAGGGAGGACGGT	215	P	A	A	NA
PMS4915	Os_Chr09	21630832	21630849	(CCT)6	(CCT)6	(CCT)6	(CCT)6	(CCT)6	(CCT)5	(CCT)6	(CCT)6	(CCT)6	(CCT)5	(CCT)5	LOC_Os09g37520	URR	DUF630/DUF632 domains containing protein, putative, expressed	-	AAAACCAGCCTCCTCACCTC	CTGCTTCAGCTTCTTCCACC	222	P	A	A	NA
PMS4916	Os_Chr09	21677486	21677501	(AC)8	(AC)8	(AC)8	(AC)8	(AC)8	(AC)8	(AC)7	(AC)8	(AC)8	(AC)8	(AC)8	LOC_Os09g37600	URR	lysM domain-containing GPI-anchored protein precursor, putative, expressed	-	CCCACCTCTCCTCTACCTCC	GCCATTGCTGCTGTCATTAG	140	A	A	A	NA
PMS4917	Os_Chr09	21758042	21758097	(TA)28	(TA)28	(TA)28	(TA)28	(TA)28	(TA)28	(TA)28	(TA)28	(TA)6	(TA)28	(TA)28	-	Introns/Intergenic	-	-	AGATGTGCTACTAGCCTACTACTGC	TATTGGAATCAGTCGCTACCC	428	A	A	A	RM24758
PMS4918	Os_Chr09	21768619	21768632	(CG)7	(CG)6	(CG)7	(CG)7	(CG)7	(CG)6	(CG)6	(CG)7	(CG)7	(CG)6	(CG)7	-	Introns/Intergenic	-	-	AAATACAAGCCAAGGGGGAG	CATGGCAGTAGAGTGGCAGA	251	P	A	A	NA
PMS4919	Os_Chr09	21770595	21770615	(TCA)7	(TCA)7	(TCA)5	(TCA)7	(TCA)7	(TCA)7	(TCA)7	(TCA)7	(TCA)7	(TCA)7	(TCA)7	-	Introns/Intergenic	-	-	GACCGAAGAACAGTTTGTGAAGG	TCTTCGGCTCTTCATTGATTCG	80	P	A	A	RM24759
PMS4920	Os_Chr09	21938216	21938233	(CGG)6	(CGG)5	(CGG)6	(CGG)6	(CGG)6	(CGG)5	(CGG)5	(CGG)6	(CGG)6	(CGG)6	(CGG)6	LOC_Os09g38070	CDS	expressed protein	CODON_DELETION	TTTCACTCGTTGAGAGCGAAGC	GGCGATGAGGAGGAGTATGAGG	249	P	P	A	RM24770
PMS4921	Os_Chr09	21938523	21938536	(AG)7	(AG)7	(AG)7	(AG)7	(AG)7	(AG)7	(AG)7	(AG)7	(AG)7	(AG)6	(AG)7	LOC_Os09g38070	URR	expressed protein	-	GAGAAAAGCGAAGCGAGAGA	GCATCCAGATCACAACCAGA	223	A	P	A	NA
PMS4922	Os_Chr09	22034799	22034813	(GAG)5	(GAG)6	(GAG)5	(GAG)5	(GAG)5	(GAG)5	(GAG)5	(GAG)5	(GAG)5	(GAG)5	(GAG)5	LOC_Os09g38300	CDS	OsFBK20 - F-box domain and kelch repeat containing protein, expressed	CODON_INSERTION	CGGAGGAGAAGGATGACG	TCAAGTCGTCAGGGAGAACC	183	P	A	A	NA
PMS4923	Os_Chr09	22045018	22045035	(CGG)6	(CGG)6	(CGG)6	(CGG)6	(CGG)6	(CGG)6	(CGG)6	(CGG)6	(CGG)6	(CGG)5	(CGG)6	LOC_Os09g38320	CDS	OsPSY|OsPSY3	CODON_DELETION	CTCCACGGCTCTGCTACCT	AAGAAGAAGAAACCAGCGGC	252	A	A	P	NA
PMS4924	Os_Chr09	22053008	22053023	(AT)8	(AT)8	(AT)8	(AT)8	(AT)8	(AT)8	(AT)8	(AT)8	(AT)8	(AT)9	(AT)9	-	Introns/Intergenic	-	-	CCCTGTAACTCGACGGAAAA	CCTGCTTGCAACGTAAGCTC	185	A	A	A	NA
PMS4925	Os_Chr09	22085764	22085841	(TA)39	(TA)39	(TA)39	(TA)8	(TA)39	(TA)6	(TA)39	(TA)39	(TA)39	(TA)39	(TA)13	-	Introns/Intergenic	-	-	AAAGGTCAAATGAGCGAGAGA	CCCCGGATAAACAATTTCCT	267	P	A	A	NA
PMS4926	Os_Chr09	22113507	22113524	(ACC)6	(ACC)6	(ACC)6	(ACC)5	(ACC)6	(ACC)6	(ACC)6	(ACC)6	(ACC)6	(ACC)6	(ACC)6	-	Introns/Intergenic	-	-	CCTCCTCTACCACCTTCGCT	GGCTCTGCTCCTTCTCCC	200	P	A	A	NA

Polymorphic SSR (PMS) markers	Chromosomes	Start physical positions (bp)	End physical positions (bp)	Nipponbare	Kasalath	Nagina22	IR64	Pokkali	Bala	Tainung67	Azucena	Moroberekan	*Oryza rufipogon*	*Oryza nivara*	MSU gene locus IDs	Structural annotation	Functional annotation	SSR functional effects	Forward primers (5'-3')	Reverse primers (5'-3')	Amplified product size (bp)	Markers exhibiting polymorphism within *indica* rice	Transcrption factor genes-derived markers	Known cloned genes-derived markers	PMS markers corresponding with RM markers
PMS4927	Os_Chr09	22181691	22181705	(GAG)5	(GAG)6	(GAG)6	(GAG)6	(GAG)6	(GAG)6	(GAG)5	(GAG)5	(GAG)5	(GAG)5	(GAG)5	LOC_Os09g38560	URR	OsRCI2-11 - Hydrophobic protein OSR8, expressed	-	AATTAAGGCTAGGCGACGTG	GATCTCGAGGAACGTGCAG	252	A	A	A	NA
PMS4928	Os_Chr09	22185770	22185787	(CTG)6	(CTG)5	(CTG)6	(CTG)6	(CTG)6	(CTG)5	(CTG)6	(CTG)6	(CTG)6	(CTG)6	(CTG)6	-	Introns/Intergenic	-	-	CAGTGAATGGGAAACGTGTG	GAGACGTCCAGGTCAAGCTC	140	P	A	A	NA
PMS4929	Os_Chr09	22208578	22208604	(TCT)9	(TCT)9	(TCT)9	(TCT)9	(TCT)9	(TCT)9	(TCT)5	(TCT)9	(TCT)9	(TCT)9	(TCT)9	-	Introns/Intergenic	-	-	TCGCTCTCCATTGATAGCTAGACC	AGTGGCGCGATACTGTAAGAACC	214	A	A	A	RM6816
PMS4930	Os_Chr09	22212526	22212543	(AT)9	(AT)11	(AT)8	(AT)8	(AT)9	(AT)9	(AT)9	(AT)8	(AT)9	(AT)9	(AT)9	LOC_Os09g38620	DRR	NADPH reductase, putative, expressed	-	TAGTGCAGCTAGGTTGCTACTCC	GCTAGTGACGATGAAATGATGC	279	P	A	A	RM2144
PMS4931	Os_Chr09	22312982	22313015	(TA)17	(TA)17	(TA)8	(TA)17	(TA)17	(TA)17	(TA)8	(TA)15	(TA)17	(TA)7	(TA)17	-	Introns/Intergenic	-	-	GCCCGTGGTAACTACAGCATTCC	GCACCACTACCAATTCCAACTCC	565	P	A	A	RM24795
PMS4932	Os_Chr09	22315806	22315823	(GCT)6	(GCT)6	(GCT)6	(GCT)6	(GCT)6	(GCT)6	(GCT)6	(GCT)6	(GCT)6	(GCT)5	(GCT)6	LOC_Os09g38850	CDS	OsDEES1	CODON_CHANGE_PLUS_CODON_DELETION	CCAAATTGCCCATACCTCAG	GTACGGCACGCTCACGTC	250	A	A	P	NA
PMS4933	Os_Chr09	22326117	22326137	(TCC)7	(TCC)7	(TCC)7	(TCC)7	(TCC)7	(TCC)7	(TCC)7	(TCC)7	(TCC)7	(TCC)6	(TCC)7	LOC_Os09g38870	CDS	expressed protein	CODON_DELETION	TTCCTCTCCTCCAGATCAATTCC	GGTAAGTCGTCCTCGGAGTCG	141	A	A	A	RM24796
PMS4934	Os_Chr09	22329330	22329343	(TC)7	(TC)8	(TC)7	(TC)7	(TC)7	(TC)7	(TC)7	(TC)7	(TC)7	(TC)7	(TC)7	-	Introns/Intergenic	-	-	ACGAGACGATGCGAAGTTCT	CAGGAACCAAACGGAGAAGA	248	P	A	A	NA
PMS4935	Os_Chr09	22356995	22357063	(TCT)23	(TCT)23	(TCT)23	(TCT)23	(TCT)23	(TCT)22	(TCT)23	(TCT)23	(TCT)23	(TCT)23	(TCT)23	LOC_Os09g38930	CDS	expressed protein	FRAME_SHIFT	AACCTCGGGATGGTGTACTTGC	CATGGACTCATCAAAGGTTCCTAGC	480	P	A	A	RM24799
PMS4936	Os_Chr09	22358140	22358211	(AAG)24	(AAG)24	(AAG)24	(AAG)24	(AAG)24	(AAG)24	(AAG)24	(AAG)24	(AAG)24	(AAG)24	(AAG)23	-	Introns/Intergenic	-	-	GCTTTCTGATTACCATTTCATGC	ATGACCACAAACACGCAAAG	194	A	A	A	NA
PMS4937	Os_Chr09	22418248	22418331	(TA)42	(TA)42	(TA)6	(TA)6	(TA)42	(TA)42	(TA)10	(TA)42	(TA)9	(TA)42	(TA)42	-	Introns/Intergenic	-	-	AAAGTCAAGGGTGGTGCTAATGG	TCAGCCAGCTTAATTTCACAGG	491	P	A	A	RM24807
PMS4938	Os_Chr09	22445008	22445029	(CT)11	(CT)11	(CT)11	(CT)11	(CT)11	(CT)9	(CT)11	(CT)11	(CT)11	(CT)11	(CT)9	-	Introns/Intergenic	-	-	TTGGTTCGCTGGAAAATACC	CAATTAGGAAATAAATGAAATTCGG	279	P	A	A	NA
PMS4939	Os_Chr09	22458453	22458464	(TC)6	(TC)8	(TC)6	(TC)6	(TC)6	(TC)6	(TC)6	(TC)6	(TC)6	(TC)6	(TC)6	-	Introns/Intergenic	-	-	CCACACCCCCTAATCTCAAA	TGTAGGAGGCAAGACCAACC	202	P	A	A	NA

Polymorphic SSR (PMS) markers	Chromosomes	Start physical positions (bp)	End physical positions (bp)	Nipponbare	Kasalath	Nagina22	IR64	Pokkali	Bala	Tainung67	Azucena	Moroberekan	*Oryza rufipogon*	*Oryza nivara*	MSU gene locus IDs	Structural annotation	Functional annotation	SSR functional effects	Forward primers (5'-3')	Reverse primers (5'-3')	Amplified product size (bp)	Markers exhibiting polymorphism within *indica* rice	Transcrption factor genes-derived markers	Known cloned genes-derived markers	PMS markers corresponding with RM markers
PMS4940	Os_Chr09	22463083	22463157	(TAA)25	(TAA)25	(TAA)25	(TAA)25	(TAA)25	(TAA)25	(TAA)25	(TAA)25	(TAA)10	(TAA)25	(TAA)25	-	Introns/Intergenic	-	-	TTTTGGTGTGTCGGATTTGA	TTGCTCGCATTGTGTTTCTC	265	A	A	A	NA
PMS4941	Os_Chr09	22670962	22670979	(CAC)6	(CAC)6	(CAC)6	(CAC)7	(CAC)7	(CAC)6	(CAC)6	(CAC)6	(CAC)6	(CAC)5	(CAC)5	LOC_Os09g39420	CDS	WD domain, G-beta repeat domain containing protein, expressed	CODON_DELETION	CCAACGGCTGTTAAACGAAT	TGGGCAATTCTCAAAACACA	248	P	A	A	NA
PMS4942	Os_Chr09	22692734	22692751	(GCA)6	(GCA)6	(GCA)6	(GCA)6	(GCA)5	(GCA)6	(GCA)6	(GCA)6	(GCA)6	(GCA)6	(GCA)6	LOC_Os09g39500	URR	ubiquitin fusion protein, putative, expressed	-	GCATATAAGTAGCCGCAGCC	CCTTGACATTGTCGATGGTG	174	P	A	A	NA
PMS4943	Os_Chr09	22713217	22713230	(CT)7	(CT)6	(CT)7	(CT)7	(CT)7	(CT)6	(CT)7	(CT)7	(CT)7	(CT)7	(CT)7	-	Introns/Intergenic	-	-	TTGCACATGAGACCGAAAAG	GAAGAGCGCAGGAGAAGAAA	267	P	A	A	NA
PMS4944	Os_Chr09	22727441	22727464	(CGG)8	(CGG)8	(CGG)8	(CGG)8	(CGG)8	(CGG)8	(CGG)8	(CGG)8	(CGG)8	(CGG)7	(CGG)8	LOC_Os09g39590	URR	metallo-beta-lactamase, putative, expressed	-	AGTCGAGGAGGAAGCGGAAGC	CCTCGAGTAGGAAAGTGAAGGAAACC	289	A	A	A	RM24832
PMS4945	Os_Chr09	22773634	22773648	(TTG)5	(TTG)6	(TTG)6	(TTG)6	(TTG)6	(TTG)6	(TTG)5	(TTG)5	(TTG)5	(TTG)5	(TTG)6	LOC_Os09g39690	URR	protein binding protein, putative, expressed	-	CAGAAAGAAGTGAAAGGGCG	CTGACACTGCTGGAGACGAC	219	A	A	A	NA
PMS4946	Os_Chr09	22811324	22811349	(AC)13	(AC)14	(AC)14	(AC)14	(AC)14	(AC)13	(AC)13	(AC)13	(AC)13	(AC)12	(AC)12	LOC_Os09g39790	DRR	lipase, putative, expressed	-	CAGCTGCAATGTCTTTCACTGC	ATGGGTAAAGGCGAAAGTCTGC	172	P	A	A	RM1013
PMS4947	Os_Chr09	22819741	22819796	(AT)28	(AT)28	(AT)21	(AT)28	(AT)28	(AT)28	(AT)28	(AT)6	(AT)28	(AT)7	(AT)28	-	Introns/Intergenic	-	-	GATAGGAGGTTGTTGTTACGTTGTCG	CTAGCTGGTACCGTTGGAAGTGG	341	P	A	A	RM24837
PMS4948	Os_Chr09	22842659	22842684	(TA)13	(TA)13	(TA)13	(TA)13	(TA)13	(TA)13	(TA)12	(TA)13	(TA)13	(TA)13	(TA)7	-	Introns/Intergenic	-	-	GGCCATGGTTGTGAATTAAGGAAGC	GACGGTTAAGTCTGGTGTGACTGTGG	290	A	A	A	RM24841
PMS4949	Os_Chr09	22943788	22943801	(GA)7	(GA)7	(GA)7	(GA)7	(GA)7	(GA)7	(GA)7	(GA)7	(GA)6	(GA)7	(GA)7	-	Introns/Intergenic	-	-	CTCCCTCTTCCCCTTCTCC	TCTTTTCTTTCTCTCCTCCCG	280	A	A	A	NA
PMS4950	Os_Chr09	22956048	22956061	(GA)7	(GA)7	(GA)7	(GA)7	(GA)7	(GA)7	(GA)7	(GA)7	(GA)6	(GA)7	(GA)7	-	Introns/Intergenic	-	-	CTCCCTCTTCCCCTTCTCC	TCTTTTCTTTCTCTCCTCCCG	280	A	A	A	NA
PMS4951	Os_Chr09	22969720	22969735	(GA)8	(GA)8	(GA)8	(GA)8	(GA)8	(GA)8	(GA)8	(GA)8	(GA)8	(GA)8	(GA)6	-	Introns/Intergenic	-	-	TTCTCCCTCTTCCCCTTCTC	CTTTCTCTCCTCCCGGCTC	278	A	A	A	NA
PMS4952	Os_Chr10	52842	52907	(TA)33	(TA)33	(TA)13	(TA)33	(TA)9	(TA)33	(TA)14	(TA)33	(TA)33	(TA)18	(TA)33	-	Introns/Intergenic	-	-	ATCGCTCGACAGATTTTTCC	ACTATGCGAATGGCTTCTGC	204	P	A	A	NA

Polymorphic SSR (PMS) markers	Chromosomes	Start physical positions (bp)	End physical positions (bp)	Nipponbare	Kasalath	Nagina22	IR64	Pokkali	Bala	Tainung67	Azucena	Moroberekan	*Oryza rufipogon*	*Oryza nivara*	MSU gene locus IDs	Structural annotation	Functional annotation	SSR functional effects	Forward primers (5'-3')	Reverse primers (5'-3')	Amplified product size (bp)	Markers exhibiting polymorphism within *indica* rice	Transcrption factor genes-derived markers	Known cloned genes-derived markers	PMS markers corresponding with RM markers
PMS4953	Os_Chr10	74286	74317	(TA)16	(TA)16	(TA)16	(TA)16	(TA)16	(TA)16	(TA)16	(TA)10	(TA)10	(TA)16	(TA)16	-	Introns/Intergenic	-	-	ACAACCCCAAAACAAATCCA	GCAAATATCAAAGCATTTCATGG	246	A	A	A	NA
PMS4954	Os_Chr10	194580	194600	(GTC)7	(GTC)7	(GTC)7	(GTC)7	(GTC)7	(GTC)7	(GTC)6	(GTC)7	(GTC)7	(GTC)6	(GTC)7	-	Introns/Intergenic	-	-	GTTCGTCGTCATCGTTCCTT	AGCTCGCTTGACGAACCAC	245	A	A	A	NA
PMS4955	Os_Chr10	278369	278408	(AT)20	(AT)10	(AT)20	(AT)20	(AT)20	(AT)20	(AT)20	(AT)20	(AT)20	(AT)12	(AT)20	-	Introns/Intergenic	-	-	GCTGAAAAGCTTCTTCGTGA	TCCGGATGTGGTTAAAAAGG	225	P	A	A	NA
PMS4956	Os_Chr10	286829	286846	(CT)9	(CT)9	(CT)9	(CT)9	(CT)9	(CT)9	(CT)9	(CT)9	(CT)9	(CT)6	(CT)9	LOC_Os10g01470	URR	homeobox associated leucine zipper, putative, expressed	-	GACCCACGTCCTCATCATCT	GTGATGCCTTGCTTCCTCTC	223	A	A	A	NA
PMS4957	Os_Chr10	338639	338652	(AT)7	(AT)6	(AT)7	(AT)7	(AT)7	(AT)7	(AT)7	(AT)7	(AT)7	(AT)7	(AT)7	-	Introns/Intergenic	-	-	AGCTGCCCATCATATCAACC	CGAAATAAGGATCGGAGAAGC	135	P	A	A	NA
PMS4958	Os_Chr10	345411	345452	(TCT)14	(TCT)14	(TCT)14	(TCT)14	(TCT)14	(TCT)14	(TCT)14	(TCT)8	(TCT)14	(TCT)14	(TCT)14	-	Introns/Intergenic	-	-	TCCTCCCTTGGTTCTTTCCT	AGTTTGTGCAATCCTGGACC	225	A	A	A	NA
PMS4959	Os_Chr10	348651	348688	(AT)19	(AT)19	(AT)19	(AT)19	(AT)19	(AT)19	(AT)19	(AT)19	(AT)7	(AT)7	(AT)19	-	Introns/Intergenic	-	-	AGCTAGCTAACCAGTGGAGTAACC	GACTGAAGTTTGAAGCCAATGC	196	A	A	A	RM24883
PMS4960	Os_Chr10	384016	384031	(AC)8	(AC)8	(AC)8	(AC)8	(AC)8	(AC)8	(AC)8	(AC)8	(AC)8	(AC)10	(AC)8	-	Introns/Intergenic	-	-	TGCAATCAAGCACATTGTCA	TCTTTGAGGGGACTGACCAC	274	A	A	A	NA
PMS4961	Os_Chr10	446582	446613	(AT)16	(AT)16	(AT)7	(AT)16	(AT)11	(AT)16	(AT)16	(AT)16	(AT)16	(AT)16	(AT)16	-	Introns/Intergenic	-	-	CCTGGCAATGAAATTCAACA	GTGCGATGTGACGGAAAAAT	280	P	A	A	NA
PMS4962	Os_Chr10	801788	801815	(TA)14	(TA)14	(TA)14	(TA)14	(TA)6	(TA)8	(TA)14	(TA)14	(TA)14	(TA)14	(TA)14	-	Introns/Intergenic	-	-	CCGGATCTAAACACATCCTAAGC	TATTTCCCGCCATCTCACTAGC	589	P	A	A	RM24902
PMS4963	Os_Chr10	805652	805687	(TTC)12	(TTC)12	(TTC)12	(TTC)12	(TTC)12	(TTC)12	(TTC)12	(TTC)12	(TTC)12	(TTC)12	(TTC)9	LOC_Os10g02260	DRR	peptide transporter PTR2, putative, expressed	-	GAGATCTGACAAGGAAGGAAGG	CCCTTGCAAGATTCTACTACTGG	350	A	A	A	RM24903
PMS4964	Os_Chr10	1404091	1404102	(CG)6	(CG)6	(CG)6	(CG)6	(CG)6	(CG)6	(CG)6	(CG)6	(CG)7	(CG)6	(CG)6	-	Introns/Intergenic	-	-	CCCTGTTGACTGGTTGAGGT	TGCAAAAGAATAGCGACGTG	133	A	A	A	NA
PMS4965	Os_Chr10	1433094	1433109	(AT)8	(AT)8	(AT)8	(AT)8	(AT)8	(AT)8	(AT)8	(AT)7	(AT)8	(AT)8	(AT)8	-	Introns/Intergenic	-	-	AAGGCACGGGTGGTCTATC	GGCACACAAGGAATAGGGAG	182	A	A	A	NA

Polymorphic SSR (PMS) markers	Chromosomes	Start physical positions (bp)	End physical positions (bp)	Nipponbare	Kasalath	Nagina22	IR64	Pokkali	Bala	Tainung67	Azucena	Moroberekan	*Oryza rufipogon*	*Oryza nivara*	MSU gene locus IDs	Structural annotation	Functional annotation	SSR functional effects	Forward primers (5'-3')	Reverse primers (5'-3')	Amplified product size (bp)	Markers exhibiting polymorphism within *indica* rice	Transcrption factor genes-derived markers	Known cloned genes-derived markers	PMS markers corresponding with RM markers
PMS4966	Os_Chr10	1502101	1502118	(AGC)6	(AGC)6	(AGC)6	(AGC)6	(AGC)6	(AGC)6	(AGC)6	(AGC)6	(AGC)6	(AGC)6	(AGC)5	-	Introns/Intergenic	-	-	AGATGGAGCCTAGCCGTAGC	CCTCAACACTTTGCGCAGTA	180	A	A	A	NA
PMS4967	Os_Chr10	1510184	1510231	(TA)24	(TA)21	(TA)24	(TA)24	(TA)24	(TA)22	(TA)7	(TA)24	(TA)24	(TA)10	(TA)24	-	Introns/Intergenic	-	-	GACAAGAGAGAAGGAAGGAGAGG	GTGCAAAGATTGAAGGACAAGC	424	P	A	A	RM24917
PMS4968	Os_Chr10	1516456	1516485	(TTC)10	(TTC)6	(TTC)10	(TTC)10	(TTC)10	(TTC)10	(TTC)10	(TTC)10	(TTC)10	(TTC)10	(TTC)10	LOC_Os10g03510	CDS	hypothetical protein	CODON_DELETION	AACAGGCGGTGTTGTTTGCTTCC	GCCGTACAGGAGCACTGGATCG	126	P	A	A	RM24918
PMS4969	Os_Chr10	1533006	1533023	(CGG)6	(CGG)6	(CGG)6	(CGG)6	(CGG)6	(CGG)6	(CGG)5	(CGG)6	(CGG)6	(CGG)6	(CGG)6	LOC_Os10g03550	CDS	expressed protein	CODON_CHANGE_PLUS_CODON_DELETION	GAGAGAGAGGCCGGAGAGAT	AACGAACCTTATCGATCCCC	162	A	A	A	NA
PMS4970	Os_Chr10	1556846	1556869	(CGG)8	(CGG)8	(CGG)8	(CGG)8	(CGG)8	(CGG)8	(CGG)8	(CGG)5	(CGG)8	(CGG)8	(CGG)5	LOC_Os10g03600	CDS	OsFBX343 - F-box domain containing protein, expressed	CODON_DELETION	ACAATGCTCCTTCGATCTGG	GAGTTGAGAGGGGAACCTCC	104	A	A	A	NA
PMS4971	Os_Chr10	1576287	1576300	(TA)7	(TA)7	(TA)7	(TA)7	(TA)6	(TA)7	(TA)7	(TA)7	(TA)7	(TA)7	(TA)7	-	Introns/Intergenic	-	-	GCGCACTCCTACCTATTTGG	GGGTCAAGCAATTGATGATG	235	P	A	A	NA
PMS4972	Os_Chr10	1783178	1783192	(CTC)5	(CTC)5	(CTC)5	(CTC)5	(CTC)5	(CTC)5	(CTC)5	(CTC)5	(CTC)5	(CTC)5	(CTC)6	-	Introns/Intergenic	-	-	CCACTCCCGTCACATTTTCT	GAAACCACTGCAAGAAGGGA	241	A	A	A	NA
PMS4973	Os_Chr10	1940822	1940851	(AT)15	(AT)15	(AT)15	(AT)15	(AT)15	(AT)15	(AT)15	(AT)14	(AT)15	(AT)15	(AT)15	-	Introns/Intergenic	-	-	AATAGCGGTCGGCATTCTAAACG	GCCTGTGTGCATAGGATATGTTGC	194	A	A	A	RM24942
PMS4974	Os_Chr10	2138561	2138575	(CGC)5	(CGC)6	(CGC)6	(CGC)5	(CGC)5	(CGC)6	(CGC)6	(CGC)5	(CGC)6	(CGC)5	(CGC)5	-	Introns/Intergenic	-	-	AAAGAGAAGGGGAAAACGGA	CTGAGAGCGTGAAATGTCCA	184	P	A	A	NA
PMS4975	Os_Chr10	2194760	2194783	(TA)12	(TA)12	(TA)12	(TA)12	(TA)10	(TA)12	(TA)12	(TA)12	(TA)7	(TA)12	(TA)12	-	Introns/Intergenic	-	-	CTGACATACTCTGTCCCAAAGC	ACATATCTTGGTCTTCCCTTGG	456	P	A	A	RM24960
PMS4976	Os_Chr10	2370579	2370594	(AG)8	(AG)8	(AG)8	(AG)8	(AG)8	(AG)8	(AG)8	(AG)8	(AG)8	(AG)9	(AG)8	-	Introns/Intergenic	-	-	CCATTGCTCGTTCTAGCGTT	AGAGAGATGGGGAGAGGAGC	206	A	A	A	NA
PMS4977	Os_Chr10	2405988	2406005	(CTG)6	(CTG)6	(CTG)6	(CTG)6	(CTG)6	(CTG)6	(CTG)6	(CTG)7	(CTG)7	(CTG)6	(CTG)6	-	Introns/Intergenic	-	-	CTCATGTGGAGGCACTTTTG	ATGGCCGAGATCAATGTTGT	266	A	A	A	NA
PMS4978	Os_Chr10	2507584	2507601	(GCG)6	(GCG)6	(GCG)6	(GCG)6	(GCG)6	(GCG)6	(GCG)6	(GCG)6	(GCG)6	(GCG)6	(GCG)5	LOC_Os10g05130	CDS	expressed protein	CODON_DELETION	CGCTGTACTCCTCGCTCTCT	CGGCTTCTTAATCCTCCTCC	161	A	A	A	NA

Polymorphic SSR (PMS) markers	Chromosomes	Start physical positions (bp)	End physical positions (bp)	Nipponbare	Kasalath	Nagina22	IR64	Pokkali	Bala	Tainung67	Azucena	Moroberekan	*Oryza rufipogon*	*Oryza nivara*	MSU gene locus IDs	Structural annotation	Functional annotation	SSR functional effects	Forward primers (5'-3')	Reverse primers (5'-3')	Amplified product size (bp)	Markers exhibiting polymorphism within *indica* rice	Transcrption factor genes-derived markers	Known cloned genes-derived markers	PMS markers corresponding with RM markers
PMS4979	Os_Chr10	2554038	2554058	(CCG)7	(CCG)7	(CCG)7	(CCG)5	(CCG)7	(CCG)7	(CCG)6	(CCG)7	(CCG)7	(CCG)7	(CCG)7	LOC_Os10g05210	URR	OsFBX368 - F-box domain containing protein, expressed	-	TTGCCTTGTTGGGCTATTTC	TCCGCGAGAGTAGAGTAGGG	232	P	A	A	NA
PMS4980	Os_Chr10	2567568	2567585	(CCG)6	(CCG)5	(CCG)5	(CCG)5	(CCG)5	(CCG)5	(CCG)5	(CCG)5	(CCG)5	(CCG)5	(CCG)5	LOC_Os10g05230	URR	zinc finger, C3HC4 type domain containing protein, expressed	-	GAGGGGAAAGTGCACAAAAC	GCGAGACGAACTCCAAAGAC	242	A	A	A	NA
PMS4981	Os_Chr10	2587353	2587376	(CGC)8	(CGC)8	(CGC)5	(CGC)8	(CGC)8	(CGC)8	(CGC)8	(CGC)8	(CGC)8	(CGC)8	(CGC)8	LOC_Os10g05240	CDS	OsFBX369 - F-box domain containing protein, expressed	CODON_DELETION	TCCAACGGTCAAGATTAGCC	CGGCATCTCGTCCATCTC	112	P	A	A	NA
PMS4982	Os_Chr10	2614874	2614891	(CGC)6	(CGC)6	(CGC)6	(CGC)6	(CGC)5	(CGC)6	(CGC)6	(CGC)6	(CGC)6	(CGC)6	(CGC)6	LOC_Os10g05290	URR	tRNA/rRNA methyltransferase, putative, expressed	-	CTATAGCTGAGCCTCCCACG	GAGATGAAAGGGGAGAAGGG	246	P	A	A	NA
PMS4983	Os_Chr10	2729145	2729160	(AC)8	(AC)8	(AC)8	(AC)8	(AC)8	(AC)8	(AC)8	(AC)8	(AC)8	(AC)6	(AC)8	-	Introns/Intergenic	-	-	GAAAACCGTGTTCAGGTCGT	TTGGAAATGCTTGGCTATGT	168	A	A	A	NA
PMS4984	Os_Chr10	2789374	2789391	(TA)9	(TA)9	(TA)9	(TA)9	(TA)7	(TA)8	(TA)9	(TA)9	(TA)9	(TA)9	(TA)9	-	Introns/Intergenic	-	-	TGGACGGAGAGAGAGTACCTTG	TCAATTTCAACAATGGTGCC	252	P	A	A	NA
PMS4985	Os_Chr10	2870319	2870333	(AGA)5	(AGA)5	(AGA)5	(AGA)6	(AGA)5	(AGA)5	(AGA)5	(AGA)5	(AGA)5	(AGA)5	(AGA)5	-	Introns/Intergenic	-	-	CAGACTGCAGACTACGCCAC	TGTCGTCAAACTGTGGTGGT	264	P	A	A	NA
PMS4986	Os_Chr10	2889196	2889219	(CCA)8	(CCA)8	(CCA)8	(CCA)8	(CCA)8	(CCA)8	(CCA)8	(CCA)8	(CCA)8	(CCA)8	(CCA)6	LOC_Os10g05730	CDS	armadillo/beta-catenin repeat family protein, putative, expressed	CODON_DELETION	GGAACAGTATGGGCTCGTTC	GCTCCTCAAGGCCTCCTC	275	A	A	A	NA
PMS4987	Os_Chr10	2913519	2913533	(GCC)5	(GCC)7	(GCC)5	(GCC)5	(GCC)5	(GCC)7	(GCC)5	(GCC)5	(GCC)7	(GCC)7	(GCC)5	LOC_Os10g05780	CDS	POT domain containing peptide transporter, putative, expressed	CODON_INSERTION	GTAGGTGAAGCTGACGAGGC	AACCACAAGAGGAGGTGGTG	239	P	P	A	NA
PMS4988	Os_Chr10	2914013	2914030	(CGC)6	(CGC)6	(CGC)6	(CGC)6	(CGC)6	(CGC)6	(CGC)6	(CGC)5	(CGC)5	(CGC)5	(CGC)5	LOC_Os10g05780	CDS	POT domain containing peptide transporter, putative, expressed	CODON_DELETION	ACGTAGCTGAGCAAGGTGGT	GTTCGAGAGAGAGGGTGACG	138	A	P	A	NA
PMS4989	Os_Chr10	3012947	3012958	(AC)6	(AC)6	(AC)6	(AC)7	(AC)6	(AC)6	(AC)6	(AC)6	(AC)6	(AC)6	(AC)6	LOC_Os10g05970	DRR	POEI12 - Pollen Ole e I allergen and extensin family protein precursor, expressed	-	ACGATTTGCCAGGATCAGAC	GGCGAATTAAGCAATGGAAG	274	P	A	A	NA
PMS4990	Os_Chr10	3059260	3059277	(CTC)6	(CTC)5	(CTC)6	(CTC)6	(CTC)6	(CTC)5	(CTC)6	(CTC)6	(CTC)5	(CTC)6	(CTC)5	-	Introns/Intergenic	-	-	GCCACCGAACGACTTTATCT	TGGTAATGAGGAGGTAGGCG	161	P	A	A	NA
PMS4991	Os_Chr10	3275138	3275155	(AT)9	(AT)9	(AT)7	(AT)9	(AT)9	(AT)9	(AT)9	(AT)9	(AT)9	(AT)9	(AT)9	-	Introns/Intergenic	-	-	CGGGGTATTAGCCGATACAT	CGATCGGCTGTAGACATCAA	202	P	A	A	NA

Polymorphic SSR (PMS) markers	Chromosomes	Start physical positions (bp)	End physical positions (bp)	Nipponbare	Kasalath	Nagina22	IR64	Pokkali	Bala	Tainung67	Azucena	Moroberekan	*Oryza rufipogon*	*Oryza nivara*	MSU gene locus IDs	Structural annotation	Functional annotation	SSR functional effects	Forward primers (5'-3')	Reverse primers (5'-3')	Amplified product size (bp)	Markers exhibiting polymorphism within *indica* rice	Transcrption factor genes-derived markers	Known cloned genes-derived markers	PMS markers corresponding with RM markers
PMS4992	Os_Chr10	3381721	3381734	(TA)7	(TA)7	(TA)7	(TA)7	(TA)7	(TA)7	(TA)7	(TA)7	(TA)6	(TA)7	(TA)7	-	Introns/Intergenic	-	-	CGTTTATAGCTGTGCTGCCA	AGAATTGGGGTGCGTTAAAA	265	A	A	A	NA
PMS4993	Os_Chr10	3388125	3388174	(TA)25	(TA)25	(TA)25	(TA)14	(TA)25	(TA)25	(TA)7	(TA)25	(TA)10	(TA)25	(TA)25	-	Introns/Intergenic	-	-	TCAATTTCAACAATGGTGCC	ACGTGGAGCAAGAACCTGAG	203	P	A	A	NA
PMS4994	Os_Chr10	3463827	3463844	(CTG)6	(CTG)6	(CTG)6	(CTG)6	(CTG)6	(CTG)6	(CTG)6	(CTG)6	(CTG)6	(CTG)6	(CTG)5	LOC_Os10g06680	CDS	expressed protein	CODON_CHANGE_PLUS_CODON_DELETION	ATTGCCAGGTGACATGGGT	CTGTCGTACGGGCTGTTGT	231	A	A	A	NA
PMS4995	Os_Chr10	3472187	3472207	(CCT)7	(CCT)7	(CCT)7	(CCT)7	(CCT)7	(CCT)7	(CCT)5	(CCT)7	(CCT)7	(CCT)7	(CCT)7	LOC_Os10g06710	CDS	amidase, putative, expressed	CODON_DELETION	GCAACAAAGCTCAGATGCAA	ACTGTGGCCTCCTCGAGAT	117	A	A	A	NA
PMS4996	Os_Chr10	3514663	3514689	(CCA)9	(CCA)9	(CCA)9	(CCA)9	(CCA)9	(CCA)9	(CCA)9	(CCA)9	(CCA)9	(CCA)6	(CCA)9	-	Introns/Intergenic	-	-	TTTCCTAAAGCCGAAAAGCA	TCATGGAGGAGGAGGAGATG	247	A	A	A	NA
PMS4997	Os_Chr10	3548239	3548262	(TA)12	(TA)12	(TA)12	(TA)12	(TA)12	(TA)12	(TA)12	(TA)12	(TA)12	(TA)12	(TA)6	-	Introns/Intergenic	-	-	AGCTCAAGCGGTTCAGTGTC	CTTAGAAGGGAGGGGAGGTG	254	A	A	A	NA
PMS4998	Os_Chr10	3554356	3554397	(CT)21	(CT)23	(CT)21	(CT)21	(CT)21	(CT)21	(CT)21	(CT)21	(CT)21	(CT)21	(CT)21	-	Introns/Intergenic	-	-	GAGTTCGATGGGCCGAAT	ACCGGTTTGGAGTGGAGC	280	P	A	A	NA
PMS4999	Os_Chr10	3607785	3607832	(TA)24	(TA)24	(TA)6	(TA)24	(TA)24	(TA)18	(TA)24	(TA)24	(TA)8	(TA)24	(TA)24	-	Introns/Intergenic	-	-	CTTAAAAGGGAGGGGAGGTG	CTTTGACTGGGGTGTGAGGT	254	P	A	A	NA
PMS5000	Os_Chr10	3609633	3609674	(TA)21	(TA)21	(TA)21	(TA)13	(TA)21	(TA)21	(TA)7	(TA)21	(TA)21	(TA)21	(TA)21	-	Introns/Intergenic	-	-	GCTGAGAAGAGTGGAGGTGG	GAGCACGCACAGGAGACATA	268	P	A	A	NA
PMS5001	Os_Chr10	3611419	3611446	(TA)14	(TA)14	(TA)14	(TA)6	(TA)14	(TA)14	(TA)12	(TA)14	(TA)14	(TA)8	(TA)14	-	Introns/Intergenic	-	-	TTTACAGAGGTGGGAGGTGG	ATTAGATACCATGGTGCCCG	189	P	A	A	NA
PMS5002	Os_Chr10	3644734	3644753	(AT)10	(AT)9	(AT)10	(AT)10	(AT)10	(AT)10	(AT)10	(AT)10	(AT)10	(AT)10	(AT)10	-	Introns/Intergenic	-	-	TCCACCACATATTTTCCCTCA	TGCAATAAAACAACTCACACCA	181	P	A	A	NA
PMS5003	Os_Chr10	3661969	3662006	(AT)19	(AT)19	(AT)19	(AT)19	(AT)9	(AT)19	(AT)19	(AT)19	(AT)19	(AT)11	(AT)19	-	Introns/Intergenic	-	-	CATTGGAAACTTTTGGCACA	TGCAACCACGTAATTCCAAA	208	P	A	A	NA
PMS5004	Os_Chr10	3695091	3695158	(TA)34	(TA)34	(TA)34	(TA)34	(TA)34	(TA)7	(TA)34	(TA)34	(TA)34	(TA)34	(TA)7	-	Introns/Intergenic	-	-	TGACAAAGTTACTTCCGTTTT	CAATTAAATCTTCACAACAAGG	292	P	A	A	NA

Polymorphic SSR (PMS) markers	Chromosomes	Start physical positions (bp)	End physical positions (bp)	Nipponbare	Kasalath	Nagina22	IR64	Pokkali	Bala	Tainung67	Azucena	Moroberekan	*Oryza rufipogon*	*Oryza nivara*	MSU gene locus IDs	Structural annotation	Functional annotation	SSR functional effects	Forward primers (5'-3')	Reverse primers (5'-3')	Amplified product size (bp)	Markers exhibiting polymorphism within *indica* rice	Transcrption factor genes-derived markers	Known cloned genes-derived markers	PMS markers corresponding with RM markers
PMS5005	Os_Chr10	3848073	3848088	(TA)8	(TA)7	(TA)8	(TA)8	(TA)8	(TA)8	(TA)8	(TA)8	(TA)8	(TA)6	(TA)8	-	Introns/Intergenic	-	-	TGGGATGAAGAGATAGGTGAAAA	TCCCTCCGTTTCATTCTAACA	188	P	A	A	NA
PMS5006	Os_Chr10	4008032	4008083	(AT)26	(AT)26	(AT)8	(AT)26	(AT)9	(AT)26	(AT)26	(AT)26	(AT)26	(AT)26	(AT)26	-	Introns/Intergenic	-	-	AATCAGCTTTGATGCACACG	AACCAAGTCCAACCCAAAAA	176	P	A	A	NA
PMS5007	Os_Chr10	4043751	4043795	(AAG)15	(AAG)16	(AAG)14	(AAG)15	(AAG)15	(AAG)15	(AAG)15	(AAG)15	(AAG)16	(AAG)7	(AAG)15	-	Introns/Intergenic	-	-	AACCGTCCACCTTGTAGCC	GGTATCCTGGTAGTGCGGTG	180	P	A	A	NA
PMS5008	Os_Chr10	4257907	4257942	(TA)18	(TA)17	(TA)10	(TA)18	(TA)10	(TA)17	(TA)18	(TA)18	(TA)7	(TA)18	(TA)18	-	Introns/Intergenic	-	-	GCCTTAGCTCGGATGACTTTT	AAAAATGGGGGAAGAAGTGG	267	P	A	A	NA
PMS5009	Os_Chr10	4388135	4388148	(CG)7	(CG)7	(CG)7	(CG)7	(CG)7	(CG)7	(CG)7	(CG)7	(CG)7	(CG)6	(CG)7	-	Introns/Intergenic	-	-	AAGGAAAGGATCGACCGAAT	CCAAAACCGCTCTCTCTCTC	183	A	A	A	NA
PMS5010	Os_Chr10	4446772	4446785	(TC)7	(TC)7	(TC)7	(TC)8	(TC)7	(TC)7	(TC)7	(TC)8	(TC)8	(TC)7	(TC)8	-	Introns/Intergenic	-	-	CCGTTTTGTCCTAAAAGGCA	CACCTACCAGTCCCCTTCCT	257	P	A	A	NA
PMS5011	Os_Chr10	4503737	4503752	(TA)8	(TA)8	(TA)6	(TA)8	(TA)7	(TA)8	(TA)8	(TA)8	(TA)8	(TA)8	(TA)8	-	Introns/Intergenic	-	-	CTAGGCCTGACGCCTGATAG	TAGTTGGGTTGGGCTAGGTG	120	P	A	A	NA
PMS5012	Os_Chr10	4529166	4529243	(AT)39	(AT)39	(AT)8	(AT)7	(AT)8	(AT)39	(AT)39	(AT)7	(AT)39	(AT)13	(AT)39	-	Introns/Intergenic	-	-	CGCAGGCAAGTGATGTAAAG	TCCTGAAACAAAAATCAGGATG	274	P	A	A	NA
PMS5013	Os_Chr10	4584703	4584716	(GA)7	(GA)6	(GA)6	(GA)7	(GA)6	(GA)6	(GA)7	(GA)7	(GA)7	(GA)7	(GA)7	-	Introns/Intergenic	-	-	CATAACACCGCCACTTAGCA	GATTTTAAAAAGAGCGGGGC	132	P	A	A	NA
PMS5014	Os_Chr10	4627326	4627369	(ATAG)11	(ATAG)11	(ATAG)11	(ATAG)11	(ATAG)6	(ATAG)11	(ATAG)11	(ATAG)11	(ATAG)11	(ATAG)11	(ATAG)11	-	Introns/Intergenic	-	-	AGGATGACACGTGGCGACTTAGG	CAACGGACGGGATTTCAGTACC	212	P	A	A	RM7217
PMS5015	Os_Chr10	4858134	4858147	(AG)7	(AG)7	(AG)7	(AG)6	(AG)7	(AG)7	(AG)7	(AG)7	(AG)7	(AG)7	(AG)6	-	Introns/Intergenic	-	-	CTTTCTCAGTTGCTCCCCTG	CACGTCAGCCTCCTCTCTCT	107	P	A	A	NA
PMS5016	Os_Chr10	4868897	4868978	(TA)41	(TA)40	(TA)41	(TA)41	(TA)41	(TA)41	(TA)41	(TA)41	(TA)41	(TA)41	(TA)28	-	Introns/Intergenic	-	-	CTATAGACCTGGTGGGAGCG	GGCAAACCCGTTATGATGAG	280	P	A	A	NA
PMS5017	Os_Chr10	4872531	4872566	(AT)18	(AT)18	(AT)18	(AT)18	(AT)18	(AT)18	(AT)7	(AT)10	(AT)18	(AT)12	(AT)18	-	Introns/Intergenic	-	-	TCAATTTCAACAATGGTGCC	CCAAAGCTGTCTCCTCGAAC	279	A	A	A	NA

Polymorphic SSR (PMS) markers	Chromosomes	Start physical positions (bp)	End physical positions (bp)	Nipponbare	Kasalath	Nagina22	IR64	Pokkali	Bala	Tainung67	Azucena	Moroberekan	*Oryza rufipogon*	*Oryza nivara*	MSU gene locus IDs	Structural annotation	Functional annotation	SSR functional effects	Forward primers (5'-3')	Reverse primers (5'-3')	Amplified product size (bp)	Markers exhibiting polymorphism within *indica* rice	Transcrption factor genes-derived markers	Known cloned genes-derived markers	PMS markers corresponding with RM markers
PMS5018	Os_Chr10	4938370	4938385	(TG)8	(TG)8	(TG)8	(TG)8	(TG)9	(TG)8	(TG)8	(TG)8	(TG)8	(TG)8	(TG)8	-	Introns/Intergenic	-	-	CCGATTAAGCTGCTTGTGGT	CCATGCATGTCTTCACCAAC	218	P	A	A	NA
PMS5019	Os_Chr10	4966466	4966485	(CT)10	(CT)9	(CT)10	(CT)10	(CT)10	(CT)10	(CT)10	(CT)10	(CT)10	(CT)10	(CT)10	-	Introns/Intergenic	-	-	ACCTTGTTCTCCAAACCCTAGCC	AGGTGAAGGAGATGGTGATGAGC	138	P	A	A	RM6565
PMS5020	Os_Chr10	5044238	5044269	(AT)16	(AT)6	(AT)16	(AT)16	(AT)16	(AT)16	(AT)16	(AT)16	(AT)16	(AT)6	(AT)7	-	Introns/Intergenic	-	-	TTGGGAGTGGAACACTGCTATGC	GCCAACGACTGTGAAGAAGATCG	389	P	A	A	RM25083
PMS5021	Os_Chr10	5173007	5173022	(GA)8	(GA)8	(GA)8	(GA)8	(GA)8	(GA)8	(GA)7	(GA)8	(GA)8	(GA)8	(GA)8	-	Introns/Intergenic	-	-	CAGGGCCAGCGTGACATC	CTCCCTCTCTCTCTTGACCT	168	A	A	A	NA
PMS5022	Os_Chr10	5181639	5181656	(GTC)6	(GTC)6	(GTC)6	(GTC)6	(GTC)6	(GTC)6	(GTC)6	(GTC)6	(GTC)6	(GTC)6	(GTC)5	-	Introns/Intergenic	-	-	ATCATCGTAACGGCGTGTTC	GAGGACAAAGACGATGACGG	214	A	A	A	NA
PMS5023	Os_Chr10	5272303	5272336	(AT)17	(AT)17	(AT)17	(AT)17	(AT)8	(AT)7	(AT)17	(AT)17	(AT)17	(AT)17	(AT)17	-	Introns/Intergenic	-	-	CAACACGAGATTCGGAGTGA	TTTCTCTCGTCTCCTCAGATG	134	P	A	A	NA
PMS5024	Os_Chr10	5486579	5486599	(GGC)7	(GGC)7	(GGC)7	(GGC)7	(GGC)7	(GGC)5	(GGC)7	(GGC)7	(GGC)7	(GGC)7	(GGC)7	-	Introns/Intergenic	-	-	GACACGTACGATCGCCGAGACG	CAAACCCTAACCCACCGTTCACC	375	P	A	A	RM25095
PMS5025	Os_Chr10	5495952	5495963	(TC)6	(TC)7	(TC)7	(TC)6	(TC)7	(TC)7	(TC)6	(TC)6	(TC)7	(TC)6	(TC)6	-	Introns/Intergenic	-	-	TGAGCTCTTTGTAGTTGAAAGTGG	TAGGGTGTGACAAACATGCC	128	P	A	A	NA
PMS5026	Os_Chr10	5576689	5576702	(TA)7	(TA)7	(TA)7	(TA)7	(TA)7	(TA)7	(TA)6	(TA)7	(TA)7	(TA)7	(TA)7	-	Introns/Intergenic	-	-	TGTAATGGGGCCATCAATTT	TGCTTCCTTTCTTTTCCTTTTT	243	A	A	A	NA
PMS5027	Os_Chr10	5723153	5723234	(TA)41	(TA)41	(TA)41	(TA)7	(TA)41	(TA)41	(TA)41	(TA)16	(TA)8	(TA)41	(TA)41	-	Introns/Intergenic	-	-	GTCATTTGAAGTAAATCCGTTGT	GTTTTATACCTTGGAGCCCG	280	P	A	A	NA
PMS5028	Os_Chr10	5763011	5763028	(TC)9	(TC)9	(TC)9	(TC)9	(TC)7	(TC)9	(TC)9	(TC)9	(TC)9	(TC)9	(TC)7	-	Introns/Intergenic	-	-	GCTGCTTTCCTCCTGTCTGA	TGCAGGTATGAGGTTTGCAT	248	P	A	A	NA
PMS5029	Os_Chr10	5779017	5779036	(AT)10	(AT)10	(AT)10	(AT)10	(AT)8	(AT)10	(AT)10	(AT)10	(AT)10	(AT)10	(AT)7	-	Introns/Intergenic	-	-	GAGCCCATAATGGAATCTGATGG	AGTAACCCAGATACGGCGATACG	163	P	A	A	RM25102
PMS5030	Os_Chr10	5795946	5795957	(AG)6	(AG)6	(AG)6	(AG)7	(AG)6	(AG)6	(AG)6	(AG)6	(AG)6	(AG)7	(AG)6	-	Introns/Intergenic	-	-	CTAGCCACCATCGTCGTCTT	CGCATTTAATTTGTCCCGTT	207	P	A	A	NA

Polymorphic SSR (PMS) markers	Chromosomes	Start physical positions (bp)	End physical positions (bp)	Nipponbare	Kasalath	Nagina22	IR64	Pokkali	Bala	Tainung67	Azucena	Moroberekan	*Oryza rufipogon*	*Oryza nivara*	MSU gene locus IDs	Structural annotation	Functional annotation	SSR functional effects	Forward primers (5'-3')	Reverse primers (5'-3')	Amplified product size (bp)	Markers exhibiting polymorphism within *indica* rice	Transcrption factor genes-derived markers	Known cloned genes-derived markers	PMS markers corresponding with RM markers
PMS5031	Os_Chr10	5897421	5897436	(GA)8	(GA)8	(GA)8	(GA)8	(GA)7	(GA)8	(GA)8	(GA)8	(GA)8	(GA)8	(GA)7	-	Introns/Intergenic	-	-	AGCCGCTCGTGGACGACT	ATCCAGCTCCACCGCCAAC	287	P	A	A	NA
PMS5032	Os_Chr10	6079410	6079449	(ATAG)10	(ATAG)10	(ATAG)10	(ATAG)10	(ATAG)10	(ATAG)10	(ATAG)10	(ATAG)10	(ATAG)5	(ATAG)10	(ATAG)8	-	Introns/Intergenic	-	-	TCTCCACAGCGAATATGGTG	CCGCTCAACAACAATCCTTT	249	A	A	A	NA
PMS5033	Os_Chr10	6287883	6287897	(CGC)5	(CGC)5	(CGC)5	(CGC)5	(CGC)5	(CGC)5	(CGC)5	(CGC)5	(CGC)5	(CGC)8	(CGC)5	LOC_Os10g11340	CDS	nitroreductase family protein, putative, expressed	CODON_INSERTION	ACCAAGCACAGCCTCACC	GAGAGGGAGTCGACGGAGA	209	A	A	A	NA
PMS5034	Os_Chr10	6318512	6318525	(GC)7	(GC)7	(GC)7	(GC)7	(GC)7	(GC)7	(GC)6	(GC)7	(GC)7	(GC)7	(GC)7	-	Introns/Intergenic	-	-	GGAGATCCCGGAGATCATTT	CTTCTCTCTCTTCTCGGCCC	226	A	A	A	NA
PMS5035	Os_Chr10	6351318	6351353	(TTC)12	(TTC)8	(TTC)12	(TTC)12	(TTC)12	(TTC)8	(TTC)12	(TTC)12	(TTC)12	(TTC)12	(TTC)8	-	Introns/Intergenic	-	-	TCCCAAATCGACATAGCCTC	GTGACCCGAGCAGAAGAAAG	193	P	A	A	NA
PMS5036	Os_Chr10	6371707	6371718	(AT)6	(AT)7	(AT)6	(AT)6	(AT)6	(AT)6	(AT)6	(AT)6	(AT)6	(AT)6	(AT)6	-	Introns/Intergenic	-	-	TTTTGTTACCGTTTCCCTCG	TGTCGCAGCTTTGGTTTATG	214	P	A	A	NA
PMS5037	Os_Chr10	6421362	6421377	(GA)8	(GA)8	(GA)8	(GA)8	(GA)8	(GA)8	(GA)8	(GA)8	(GA)8	(GA)7	(GA)8	-	Introns/Intergenic	-	-	ACGATGAGGCGAGAGAGAGA	CTCACCAATAAAAGCGCCTC	227	A	A	A	NA
PMS5038	Os_Chr10	6659171	6659228	(TA)29	(TA)20	(TA)29	(TA)29	(TA)29	(TA)29	(TA)9	(TA)29	(TA)29	(TA)29	(TA)29	-	Introns/Intergenic	-	-	TGCAGGGAATCAACATCAAA	TGTGAGCAAGGCAAGTCATC	175	P	A	A	NA
PMS5039	Os_Chr10	6674366	6674405	(TA)20	(TA)29	(TA)20	(TA)20	(TA)20	(TA)20	(TA)9	(TA)20	(TA)20	(TA)29	(TA)20	-	Introns/Intergenic	-	-	CTGGATCCAAACACCACCATAGC	AGTGAAGACCAGGGTCAGCAAGG	294	P	A	A	RM25126
PMS5040	Os_Chr10	6799988	6800031	(TA)22	(TA)22	(TA)22	(TA)10	(TA)22	(TA)22	(TA)22	(TA)22	(TA)22	(TA)9	(TA)22	-	Introns/Intergenic	-	-	CCCCTCTAGTCTGATCTCTTATCC	TTTTATACGTACGCACACAAACTT	236	P	A	A	NA
PMS5041	Os_Chr10	6801129	6801176	(TA)24	(TA)24	(TA)24	(TA)6	(TA)24	(TA)24	(TA)24	(TA)24	(TA)12	(TA)24	(TA)8	-	Introns/Intergenic	-	-	ATGTTTCAGACGGGCAAAAC	CGAGAACTATTTATGCGAAAACTT	248	P	A	A	NA
PMS5042	Os_Chr10	7252588	7252647	(TA)30	(TA)30	(TA)9	(TA)30	(TA)30	(TA)14	(TA)30	(TA)8	(TA)6	(TA)30	(TA)30	-	Introns/Intergenic	-	-	TATAGGGCAGGCTTCACTCAAGG	TTGAATCTTGGAGCCCATACTCC	305	P	A	A	RM25138
PMS5043	Os_Chr10	7259060	7259077	(AT)9	(AT)9	(AT)9	(AT)9	(AT)9	(AT)9	(AT)9	(AT)9	(AT)9	(AT)9	(AT)7	-	Introns/Intergenic	-	-	GCGATTTAATATGAGATTAGTAG	GGATGTATTTGCTAACCGTAA	472	A	A	A	NA

Polymorphic SSR (PMS) markers	Chromosomes	Start physical positions (bp)	End physical positions (bp)	Nipponbare	Kasalath	Nagina22	IR64	Pokkali	Bala	Tainung67	Azucena	Moroberekan	*Oryza rufipogon*	*Oryza nivara*	MSU gene locus IDs	Structural annotation	Functional annotation	SSR functional effects	Forward primers (5'-3')	Reverse primers (5'-3')	Amplified product size (bp)	Markers exhibiting polymorphism within *indica* rice	Transcrption factor genes-derived markers	Known cloned genes-derived markers	PMS markers corresponding with RM markers
PMS5044	Os_Chr10	7331672	7331692	(TAA)7	(TAA)6	(TAA)7	(TAA)7	(TAA)7	(TAA)7	(TAA)7	(TAA)7	(TAA)7	(TAA)7	(TAA)7	-	Introns/Intergenic	-	-	TCTTGTGGGTACAGTGGTACATCC	ACCTCCAAACCAGGAATTAACC	196	P	A	A	RM25144
PMS5045	Os_Chr10	7419165	7419182	(GCG)6	(GCG)6	(GCG)6	(GCG)6	(GCG)6	(GCG)6	(GCG)6	(GCG)6	(GCG)6	(GCG)5	(GCG)6	LOC_Os10g13670	CDS	Zinc finger, C3HC4 type domain containing protein, expressed	CODON_DELETION	GTAGTACTGCGGCAGGTTGG	TTGAAGATACTTCCGGCCAC	252	A	A	A	NA
PMS5046	Os_Chr10	7507008	7507058	(TAT)17	(TAT)17	(TAT)11	(TAT)17	(TAT)11	(TAT)17	(TAT)17	(TAT)17	(TAT)17	(TAT)17	(TAT)10	-	Introns/Intergenic	-	-	CTGTCATGGAGCCATCCTTT	TTCACACCGTGAATTTTACCC	279	P	A	A	NA
PMS5047	Os_Chr10	7566991	7567056	(AT)33	(AT)33	(AT)9	(AT)33	(AT)33	(AT)33	(AT)33	(AT)15	(AT)33	(AT)33	(AT)6	-	Introns/Intergenic	-	-	TGCCTCATACCTTTGTTCGATAGC	CTGCAGCTCCTGCTACATTATGC	264	P	A	A	RM8015
PMS5048	Os_Chr10	7619274	7619289	(TA)8	(TA)8	(TA)8	(TA)8	(TA)6	(TA)8	(TA)8	(TA)8	(TA)8	(TA)8	(TA)8	-	Introns/Intergenic	-	-	GGGCTTTGACCAGACTTTGA	TAGCGTGGAAGCACTCTTGA	252	P	A	A	NA
PMS5049	Os_Chr10	7701033	7701048	(AT)8	(AT)8	(AT)8	(AT)7	(AT)7	(AT)8	(AT)8	(AT)8	(AT)8	(AT)7	(AT)8	-	Introns/Intergenic	-	-	TCTCCGTTTCACAATGTAAGTCA	CGTCATGGAAGGCGTACTTT	259	P	A	A	NA
PMS5050	Os_Chr10	7747334	7747349	(AG)8	(AG)8	(AG)8	(AG)8	(AG)8	(AG)8	(AG)8	(AG)8	(AG)7	(AG)8	(AG)8	-	Introns/Intergenic	-	-	GTAGACTGATCGGCAGGAGC	AGAAAGGAGCAATGGCGTTA	206	A	A	A	NA
PMS5051	Os_Chr10	7781870	7781884	(CAG)5	(CAG)7	(CAG)7	(CAG)5	(CAG)5	(CAG)7	(CAG)5	(CAG)6	(CAG)5	(CAG)5	(CAG)5	-	Introns/Intergenic	-	-	CGTTGGTGTTGATGTTCCTG	TGAGTAACTGCACGCGTAGG	220	P	A	A	NA
PMS5052	Os_Chr10	7861324	7861377	(TA)27	(TA)24	(TA)27	(TA)27	(TA)27	(TA)27	(TA)27	(TA)27	(TA)10	(TA)8	(TA)27	-	Introns/Intergenic	-	-	CACCGCAAGGTGTTTATGTG	TCAATTTCAATCATGGTGCC	240	P	A	A	NA
PMS5053	Os_Chr10	7942783	7942816	(TA)17	(TA)17	(TA)9	(TA)10	(TA)17	(TA)17	(TA)17	(TA)17	(TA)17	(TA)17	(TA)17	-	Introns/Intergenic	-	-	TTCCCGTGAATTGTGGATTGAGC	ACCCAGCCTCAGCCATATGATCG	356	P	A	A	RM25160
PMS5054	Os_Chr10	8174170	8174196	(ATC)9	(ATC)9	(ATC)9	(ATC)9	(ATC)9	(ATC)9	(ATC)9	(ATC)9	(ATC)9	(ATC)6	(ATC)9	LOC_Os10g16390	CDS	retrotransposon protein, putative, Ty3-gypsy subclass, expressed	CODON_DELETION	CCTTCTTGCTTCTAACCCATTCG	TCTTGCACAGAAGTTGACCTTGC	164	A	A	A	RM25162
PMS5055	Os_Chr10	8351885	8351908	(TC)12	(TC)12	(TC)9	(TC)12	(TC)12	(TC)12	(TC)12	(TC)12	(TC)12	(TC)12	(TC)12	-	Introns/Intergenic	-	-	CATCTCTGGGGTGAGAGCC	CGCTTGGACGTTACGTTGT	200	P	A	A	NA
PMS5056	Os_Chr10	8405030	8405047	(AT)9	(AT)9	(AT)9	(AT)6	(AT)6	(AT)9	(AT)9	(AT)9	(AT)9	(AT)9	(AT)9	-	Introns/Intergenic	-	-	ATGCCATTTGCAGGTACACA	ATCACCGGGTTAAATGTGGT	250	P	A	A	NA

Polymorphic SSR (PMS) markers	Chromosomes	Start physical positions (bp)	End physical positions (bp)	Nipponbare	Kasalath	Nagina22	IR64	Pokkali	Bala	Tainung67	Azucena	Moroberekan	*Oryza rufipogon*	*Oryza nivara*	MSU gene locus IDs	Structural annotation	Functional annotation	SSR functional effects	Forward primers (5'-3')	Reverse primers (5'-3')	Amplified product size (bp)	Markers exhibiting polymorphism within *indica* rice	Transcrption factor genes-derived markers	Known cloned genes-derived markers	PMS markers corresponding with RM markers
PMS5057	Os_Chr10	8405844	8405909	(AT)33	(AT)33	(AT)21	(AT)33	(AT)33	(AT)33	(AT)12	(AT)33	(AT)15	(AT)20	(AT)33	-	Introns/Intergenic	-	-	ACACTTCAGCGGTATAGATTCG	TTTCCTCAAACTGTAGGTCTGC	298	P	A	A	RM25172
PMS5058	Os_Chr10	8418239	8418260	(AT)11	(AT)10	(AT)11	(AT)11	(AT)11	(AT)10	(AT)11	(AT)11	(AT)11	(AT)10	(AT)10	-	Introns/Intergenic	-	-	ATTTGATACCATGTTGCCCG	AATCGCTCCCTCGACAGTTA	213	P	A	A	NA
PMS5059	Os_Chr10	8848647	8848704	(TA)29	(TA)29	(TA)19	(TA)29	(TA)29	(TA)29	(TA)6	(TA)16	(TA)29	(TA)29	(TA)29	-	Introns/Intergenic	-	-	CGAGCATGTTTCCCAAAGTT	ATCATCGAGCAGCATGACAG	279	P	A	A	NA
PMS5060	Os_Chr10	8920463	8920528	(TTC)22	(TTC)22	(TTC)22	(TTC)23	(TTC)22	(TTC)22	(TTC)22	(TTC)22	(TTC)22	(TTC)22	(TTC)22	-	Introns/Intergenic	-	-	GATGGCTATGTCGCAAGGAT	TTTTCATTTGCCGTGTACCA	279	P	A	A	NA
PMS5061	Os_Chr10	8927134	8927173	(TA)20	(TA)20	(TA)20	(TA)20	(TA)10	(TA)20	(TA)20	(TA)20	(TA)8	(TA)20	(TA)20	-	Introns/Intergenic	-	-	AATCACCTCAGAGCATTATGA	GTAAATTCAGTAAAGGCTAAAAG	205	P	A	A	NA
PMS5062	Os_Chr10	9069962	9069975	(TA)7	(TA)7	(TA)7	(TA)6	(TA)7	(TA)7	(TA)7	(TA)7	(TA)7	(TA)7	(TA)6	-	Introns/Intergenic	-	-	TCCGTCCCTAAATATCTGACG	TGACGCCGCTGACTTTTT	280	P	A	A	NA
PMS5063	Os_Chr10	9228292	9228315	(ATA)8	(ATA)7	(ATA)8	(ATA)8	(ATA)8	(ATA)8	(ATA)8	(ATA)8	(ATA)8	(ATA)8	(ATA)8	-	Introns/Intergenic	-	-	GACTATTCCAGCTCTGGTATGATTGG	TTGCTAGTGGAAGATGATGTGACC	244	P	A	A	RM25192
PMS5064	Os_Chr10	9352153	9352174	(TA)11	(TA)12	(TA)9	(TA)10	(TA)11	(TA)11	(TA)11	(TA)11	(TA)11	(TA)8	(TA)11	-	Introns/Intergenic	-	-	GCAATGTTTCTCATGACCTTCG	TGTGGAATATCACGTAGCCAAGC	376	P	A	A	RM25200
PMS5065	Os_Chr10	9395598	9395611	(TA)7	(TA)7	(TA)7	(TA)7	(TA)7	(TA)7	(TA)7	(TA)7	(TA)7	(TA)6	(TA)7	-	Introns/Intergenic	-	-	GGTGAGGAGCCTTGTCACAT	TTGCTTCTCCAAACAAGGCT	211	A	A	A	NA
PMS5066	Os_Chr10	9560190	9560237	(TA)24	(TA)24	(TA)13	(TA)24	(TA)24	(TA)24	(TA)6	(TA)24	(TA)24	(TA)24	(TA)24	-	Introns/Intergenic	-	-	ACGTACGGTGTTCCAATTGTGG	GATTTGTATAGGGTGGGAAACACTCG	330	P	A	A	RM25212
PMS5067	Os_Chr10	9763043	9763054	(CT)6	(CT)6	(CT)6	(CT)6	(CT)6	(CT)6	(CT)6	(CT)6	(CT)6	(CT)7	(CT)6	-	Introns/Intergenic	-	-	GGTCTCCCTCCCTTCCTCT	TAGATCAGACGGCTGTAGCG	197	A	A	A	NA
PMS5068	Os_Chr10	9770809	9770832	(TC)12	(TC)12	(TC)12	(TC)12	(TC)12	(TC)12	(TC)12	(TC)12	(TC)12	(TC)6	(TC)12	LOC_Os10g19180	URR	zinc finger family protein, putative, expressed	-	TATTCTACTCGCAAGTCGCAAGC	GTGGATCACAATGCGAACTTGG	247	A	A	A	RM1126
PMS5069	Os_Chr10	9831066	9831086	(GGA)7	(GGA)7	(GGA)7	(GGA)7	(GGA)7	(GGA)7	(GGA)5	(GGA)7	(GGA)7	(GGA)7	(GGA)7	-	Introns/Intergenic	-	-	ATCACCGTCGTCTTCCTTTG	GGACCACGAATCGAAGAAAA	195	A	A	A	NA

Polymorphic SSR (PMS) markers	Chromosomes	Start physical positions (bp)	End physical positions (bp)	Nipponbare	Kasalath	Nagina22	IR64	Pokkali	Bala	Tainung67	Azucena	Moroberekan	*Oryza rufipogon*	*Oryza nivara*	MSU gene locus IDs	Structural annotation	Functional annotation	SSR functional effects	Forward primers (5'-3')	Reverse primers (5'-3')	Amplified product size (bp)	Markers exhibiting polymorphism within *indica* rice	Transcrption factor genes-derived markers	Known cloned genes-derived markers	PMS markers corresponding with RM markers
PMS5070	Os_Chr10	9900683	9900696	(GA)7	(GA)8	(GA)7	(GA)7	(GA)7	(GA)7	(GA)7	(GA)7	(GA)7	(GA)7	(GA)7	-	Introns/Intergenic	-	-	GCGGATGGTTCTAC TTCTGC	GGTGGAAATCCAATC AGCAT	259	P	A	A	NA
PMS5071	Os_Chr10	9919002	9919015	(TC)7	(TC)6	(TC)7	(TC)7	(TC)7	(TC)6	(TC)7	(TC)6	(TC)7	(TC)7	(TC)6	-	Introns/Intergenic	-	-	GAGAGCTTCGCCGT GTAATC	CGTAAAACACACATG CCCAG	270	P	A	A	NA
PMS5072	Os_Chr10	10023520	10023545	(AT)13	(AT)12	(AT)13	(AT)13	(AT)13	(AT)13	(AT)13	(AT)13	(AT)13	(AT)13	(AT)13	-	Introns/Intergenic	-	-	ATCTGAGAGCGATT GGGATTCG	TGAGGCTGTCTAAAG TAGGACTCAGC	370	P	A	A	RM25228
PMS5073	Os_Chr10	10028391	10028412	(TA)11	(TA)11	(TA)11	(TA)11	(TA)10	(TA)11	(TA)10	(TA)11	(TA)8	(TA)11	(TA)11	-	Introns/Intergenic	-	-	CCACCCTGCAAAAG ATCCTA	TGGTGCTACAATGCT TCGAG	235	P	A	A	NA
PMS5074	Os_Chr10	10040701	10040714	(CT)7	(CT)7	(CT)7	(CT)6	(CT)7	(CT)7	(CT)7	(CT)7	(CT)7	(CT)7	(CT)7	-	Introns/Intergenic	-	-	ATGTCGGAGCCTGT CTTGTT	TCCTGGTTGTCACCA TCTGA	176	P	A	A	NA
PMS5075	Os_Chr10	10254071	10254094	(TTAA)6	(TTAA)6	(TTAA)6	(TTAA)6	(TTAA)5	(TTAA)6	(TTAA)6	(TTAA)5	(TTAA)6	(TTAA)6	(TTAA)6	-	Introns/Intergenic	-	-	GAGGCTGAAGACTG AAGAGAGTGG	GTCCAAACACATTCC GAGTTCC	96	P	A	A	RM25234
PMS5076	Os_Chr10	10391465	10391482	(TA)9	(TA)9	(TA)7	(TA)9	(TA)9	(TA)9	(TA)6	(TA)9	(TA)9	(TA)9	(TA)9	-	Introns/Intergenic	-	-	GGCCCATCCTCTAC AATGAA	AAATACCCACCATTG TTTGAAAT	267	P	A	A	NA
PMS5077	Os_Chr10	10484872	10484889	(CGG)6	(CGG)6	(CGG)6	(CGG)6	(CGG)6	(CGG)6	(CGG)6	(CGG)6	(CGG)6	(CGG)5	(CGG)6	LOC_Os10g20710	CDS	formin-like protein 6 precursor, putative, expressed	CODON_DELETION	AGTGCAGTGGCTTC AGCTTT	TTCAACAACACAACC CCAAA	241	A	A	A	NA
PMS5078	Os_Chr10	10552478	10552495	(AT)9	(AT)10	(AT)9	(AT)9	(AT)9	(AT)9	(AT)9	(AT)9	(AT)9	(AT)9	(AT)9	-	Introns/Intergenic	-	-	AAAATGTGAGAACG AACGGG	ACGACGACGACGTC TTTTCT	188	P	A	A	NA
PMS5079	Os_Chr10	10645059	10645078	(AT)10	(AT)6	(AT)10	(AT)10	(AT)10	(AT)10	(AT)10	(AT)10	(AT)10	(AT)10	(AT)10	-	Introns/Intergenic	-	-	AGCCCTCGAGTGAA TGATTG	GAGATTAGCCTTCTC CGCCT	275	P	A	A	NA
PMS5080	Os_Chr10	10717052	10717073	(TA)11	(TA)11	(TA)11	(TA)8	(TA)11	(TA)11	(TA)11	(TA)11	(TA)11	(TA)11	(TA)11	-	Introns/Intergenic	-	-	CATTGGAAGAGCAT ATCTAGGACACG	GATTCTTTGACAACC TCGTCAGG	373	P	A	A	RM25252
PMS5081	Os_Chr10	10725109	10725124	(GA)8	(GA)6	(GA)6	(GA)8	(GA)8	(GA)6	(GA)8	(GA)8	(GA)8	(GA)8	(GA)8	-	Introns/Intergenic	-	-	GCGAGAAGGCTAGT TAGCGA	CCAACCAAACAGAAA CCAAAA	169	P	A	A	NA
PMS5082	Os_Chr10	10725434	10725457	(AT)12	(AT)12	(AT)12	(AT)9	(AT)7	(AT)12	(AT)12	(AT)12	(AT)12	(AT)12	(AT)12	-	Introns/Intergenic	-	-	TGCAAATTTTGTATA CGTTGTGT	TGTTGATAGGTATAG GTATGAATCGAA	279	P	A	A	NA

Polymorphic SSR (PMS) markers	Chromosomes	Start physical positions (bp)	End physical positions (bp)	Nipponbare	Kasalath	Nagina22	IR64	Pokkali	Bala	Tainung67	Azucena	Moroberekan	*Oryza rufipogon*	*Oryza nivara*	MSU gene locus IDs	Structural annotation	Functional annotation	SSR functional effects	Forward primers (5'-3')	Reverse primers (5'-3')	Amplified product size (bp)	Markers exhibiting polymorphism within *indica* rice	Transcrption factor genes-derived markers	Known cloned genes-derived markers	PMS markers corresponding with RM markers
PMS5083	Os_Chr10	10725821	10725872	(AT)26	(AT)26	(AT)18	(AT)26	(AT)26	(AT)26	(AT)14	(AT)26	(AT)9	(AT)26	(AT)9	-	Introns/Intergenic	-	-	TCAATTTCAACAATGGTGCC	GCCAATACTCCATGTAGCACG	135	P	A	A	NA
PMS5084	Os_Chr10	10753795	10753815	(GCG)7	(GCG)7	(GCG)7	(GCG)7	(GCG)7	(GCG)7	(GCG)7	(GCG)7	(GCG)7	(GCG)6	(GCG)7	-	Introns/Intergenic	-	-	GTCGAGCGTGAGAGAGCAC	TTTAATTCCTTAAGCGTCGCA	278	A	A	A	NA
PMS5085	Os_Chr10	10755719	10755740	(TA)11	(TA)11	(TA)11	(TA)11	(TA)11	(TA)11	(TA)8	(TA)11	(TA)11	(TA)11	(TA)9	-	Introns/Intergenic	-	-	ATTAGCCGATGGATACCGAA	GGTAGCTTCAGCATCCCAAA	265	A	A	A	NA
PMS5086	Os_Chr10	10780425	10780438	(AG)7	(AG)7	(AG)7	(AG)7	(AG)7	(AG)7	(AG)7	(AG)7	(AG)7	(AG)7	(AG)11	-	Introns/Intergenic	-	-	TTGCTATAGTCGCCATCGTG	CGGGAGCTCCTCTTCCTATC	274	A	A	A	NA
PMS5087	Os_Chr10	10983152	10983211	(TA)30	(TA)19	(TA)9	(TA)9	(TA)9	(TA)9	(TA)30	(TA)9	(TA)9	(TA)9	(TA)9	-	Introns/Intergenic	-	-	ACGTCCATCCCAAGCATTAAAGG	CCCAGGACAGTATAGGCACATGG	432	P	A	A	RM25263
PMS5088	Os_Chr10	11006019	11006056	(AT)19	(AT)19	(AT)19	(AT)19	(AT)11	(AT)19	(AT)9	(AT)19	(AT)19	(AT)19	(AT)19	-	Introns/Intergenic	-	-	ACGGTCTCTATTTTGTGGCG	CGTTCCAATTCGACTGGATT	152	P	A	A	NA
PMS5089	Os_Chr10	11058074	11058095	(TA)11	(TA)11	(TA)11	(TA)11	(TA)11	(TA)11	(TA)11	(TA)11	(TA)8	(TA)11	(TA)11	-	Introns/Intergenic	-	-	CCTAAACTGCATGGAACGCT	AACGTGGAACTAAACACACCC	261	A	A	A	NA
PMS5090	Os_Chr10	11149495	11149515	(CCG)7	(CCG)6	(CCG)7	(CCG)7	(CCG)7	(CCG)7	(CCG)7	(CCG)7	(CCG)7	(CCG)7	(CCG)7	LOC_Os10g21720	CDS	expressed protein	CODON_DELETION	CTACAAACCACCCAATCCAAGC	CTCTCAATGTCTCCCTTTCTCTCC	261	P	A	A	RM25270
PMS5091	Os_Chr10	11272865	11272878	(GA)7	(GA)7	(GA)7	(GA)7	(GA)7	(GA)7	(GA)6	(GA)7	(GA)7	(GA)7	(GA)7	-	Introns/Intergenic	-	-	TCTCTCCTCTCCTCACGCAT	GCACACAGCTTCCTTCCTTC	243	A	A	A	NA
PMS5092	Os_Chr10	11296817	11296840	(AT)12	(AT)12	(AT)12	(AT)12	(AT)12	(AT)12	(AT)12	(AT)12	(AT)12	(AT)7	(AT)12	-	Introns/Intergenic	-	-	ACGACGACGACCTGCTTAAT	TGAATGTGGGCAATGATAGAA	221	A	A	A	NA
PMS5093	Os_Chr10	11433622	11433635	(GA)7	(GA)7	(GA)6	(GA)7	(GA)7	(GA)7	(GA)7	(GA)7	(GA)7	(GA)7	(GA)7	-	Introns/Intergenic	-	-	GAGGAGGAGCTGACAAGTGG	AATCCCCCTTCCTCTCTTTG	217	P	A	A	NA
PMS5094	Os_Chr10	11546016	11546029	(CT)7	(CT)7	(CT)7	(CT)7	(CT)6	(CT)7	(CT)7	(CT)7	(CT)7	(CT)7	(CT)7	LOC_Os10g22330	URR	clathrin adaptor complex small chain domain containing protein, expressed	-	CTCGATCTCCTCCTCCTCCT	GCATGATTGGGGACCTTAGA	237	P	A	A	NA
PMS5095	Os_Chr10	11586313	11586332	(TC)10	(TC)8	(TC)10	(TC)10	(TC)10	(TC)8	(TC)10	(TC)10	(TC)10	(TC)10	(TC)10	-	Introns/Intergenic	-	-	CATGTTGGCAAGGTGACATATCC	GGCACTTTACGGACTACCACTCG	146	P	A	A	RM25286

Polymorphic SSR (PMS) markers	Chromosomes	Start physical positions (bp)	End physical positions (bp)	Nipponbare	Kasalath	Nagina22	IR64	Pokkali	Bala	Tainung67	Azucena	Moroberekan	*Oryza rufipogon*	*Oryza nivara*	MSU gene locus IDs	Structural annotation	Functional annotation	SSR functional effects	Forward primers (5'-3')	Reverse primers (5'-3')	Amplified product size (bp)	Markers exhibiting polymorphism within *indica* rice	Transcrption factor genes-derived markers	Known cloned genes-derived markers	PMS markers corresponding with RM markers
PMS5096	Os_Chr10	11617660	11617677	(CT)9	(CT)9	(CT)9	(CT)9	(CT)9	(CT)6	(CT)9	(CT)9	(CT)9	(CT)6	(CT)9	-	Introns/Intergenic	-	-	CCGAGCTGGCCGAGATTC	CGGTAAGAGAACGGAAAAG	159	P	A	A	NA
PMS5097	Os_Chr10	11624449	11624466	(GGA)6	(GGA)6	(GGA)6	(GGA)6	(GGA)6	(GGA)6	(GGA)6	(GGA)6	(GGA)6	(GGA)5	(GGA)6	LOC_Os10g22450	URR	OsINO1-2	-	ATCTCCGCAAATTCGTCATC	TCGGTACTCCGACTCGATCT	273	A	A	P	NA
PMS5098	Os_Chr10	11737240	11737279	(TA)20	(TA)20	(TA)20	(TA)7	(TA)20	(TA)20	(TA)6	(TA)20	(TA)20	(TA)20	(TA)20	-	Introns/Intergenic	-	-	TGCTCCGGAGATGTAGACTATGTCG	TTCGTGGGTGATGGATATCTGC	624	P	A	A	RM4455
PMS5099	Os_Chr10	11818232	11818251	(AT)10	(AT)10	(AT)10	(AT)10	(AT)10	(AT)10	(AT)10	(AT)10	(AT)6	(AT)10	(AT)10	-	Introns/Intergenic	-	-	TGCAACGAGGCAGAAATATCAGG	CGGTGAACTTATTCCTCTAGCTTTCC	386	A	A	A	RM25293
PMS5100	Os_Chr10	11819046	11819063	(GCC)6	(GCC)6	(GCC)6	(GCC)6	(GCC)6	(GCC)6	(GCC)6	(GCC)6	(GCC)6	(GCC)6	(GCC)5	-	Introns/Intergenic	-	-	CACACACCTCACGTCCTCC	ACGTGATCGCCTCAACCTAC	206	A	A	A	NA
PMS5101	Os_Chr10	11833342	11833363	(TC)11	(TC)8	(TC)11	(TC)11	(TC)11	(TC)8	(TC)11	(TC)11	(TC)11	(TC)11	(TC)11	-	Introns/Intergenic	-	-	CCCACTTGTCGGTGTCTCTT	GCGCACTTGGAGAAAGAAAG	134	P	A	A	NA
PMS5102	Os_Chr10	11867364	11867377	(AT)7	(AT)7	(AT)7	(AT)7	(AT)7	(AT)7	(AT)7	(AT)7	(AT)7	(AT)9	(AT)7	-	Introns/Intergenic	-	-	AACATGGAAATGCTAAGCGG	TATGGTGGATTCGAAGTTTGT	197	A	A	A	NA
PMS5103	Os_Chr10	11915896	11915907	(GA)6	(GA)7	(GA)7	(GA)6	(GA)7	(GA)7	(GA)6	(GA)6	(GA)6	(GA)6	(GA)6	-	Introns/Intergenic	-	-	GGGGAGAGAGGAAGCAGAGA	TCTTTTTGTGGGCCAAATTC	153	P	A	A	NA
PMS5104	Os_Chr10	12029872	12029889	(TTC)6	(TTC)6	(TTC)6	(TTC)6	(TTC)6	(TTC)6	(TTC)6	(TTC)6	(TTC)6	(TTC)6	(TTC)5	-	Introns/Intergenic	-	-	CCTCCTCACCTTCTCGTCTG	CTCGATACCCATCGCAGTTT	248	A	A	A	NA
PMS5105	Os_Chr10	12033059	12033074	(TC)8	(TC)8	(TC)8	(TC)8	(TC)8	(TC)8	(TC)8	(TC)8	(TC)8	(TC)8	(TC)9	-	Introns/Intergenic	-	-	TTGAGTGCCCATTACAGCAG	GCATCTGATGGACGATGATG	127	A	A	A	NA
PMS5106	Os_Chr10	12039400	12039413	(AT)7	(AT)7	(AT)7	(AT)7	(AT)7	(AT)7	(AT)7	(AT)7	(AT)7	(AT)16	(AT)7	-	Introns/Intergenic	-	-	AACGTCGACCGAAAATATGG	TTTAAACGGCTACCTGAGGG	227	A	A	A	NA
PMS5107	Os_Chr10	12119796	12119816	(CCT)7	(CCT)7	(CCT)7	(CCT)7	(CCT)7	(CCT)7	(CCT)5	(CCT)7	(CCT)7	(CCT)7	(CCT)7	LOC_Os10g23230	CDS	AT hook-containing MAR binding 1, putative, expressed	CODON_DELETION	GTGAAGCCCTTCTCGCCAATCC	CCACGTCGAGGAAGAAGATGTGC	154	A	A	A	RM25306
PMS5108	Os_Chr10	12193771	12193784	(GA)7	(GA)7	(GA)7	(GA)7	(GA)8	(GA)8	(GA)8	(GA)8	(GA)7	(GA)8	(GA)8	-	Introns/Intergenic	-	-	CTGCCTCTCTTTCTTGCGTT	AGCCAATGAGTCGGAGATTG	208	P	A	A	NA

**Polymorphic SSR (PMS) markers**	**Chromosomes**	**Start physical positions (bp)**	**End physical positions (bp)**	**Nipponbare**	**Kasalath**	**Nagina22**	**IR64**	**Pokkali**	**Bala**	**Tainung67**	**Azucena**	**Moroberekan**	***Oryza rufipogon***	***Oryza nivara***	**MSU gene locus IDs**	**Structural annotation**	**Functional annotation**	**SSR functional effects**	**Forward primers (5'-3')**	**Reverse primers (5'-3')**	**Amplified product size (bp)**	**Markers exhibiting polymorphism within *indica* rice**	**Transcrption factor genes-derived markers**	**Known cloned genes-derived markers**	**PMS markers corresponding with RM markers**
PMS5109	Os_Chr10	12196424	12196439	(GA)8	(GA)7	(GA)8	(GA)8	(GA)8	(GA)8	(GA)7	(GA)8	(GA)7	(GA)8	(GA)7	-	Introns/Intergenic	-	-	CTGCCTCTCTTTCTTGCGTT	TCTAGAGCTGTGTCGGTCCC	276	P	A	A	NA
PMS5110	Os_Chr10	12233852	12233869	(CT)9	(CT)9	(CT)9	(CT)9	(CT)9	(CT)9	(CT)9	(CT)9	(CT)9	(CT)9	(CT)8	-	Introns/Intergenic	-	-	CCCCTTCTTCCACCTCTCAC	GGGGATGGTTTATATGGGCT	250	A	A	A	NA
PMS5111	Os_Chr10	12305151	12305228	(TA)39	(TA)39	(TA)39	(TA)39	(TA)39	(TA)39	(TA)39	(TA)39	(TA)39	(TA)39	(TA)10	-	Introns/Intergenic	-	-	ACGTCATCCGTGTATGTGTTGG	AGTTGTTCGTTCATCGACTTGG	509	A	A	A	RM25311
PMS5112	Os_Chr10	12320943	12320957	(CGA)5	(CGA)6	(CGA)6	(CGA)6	(CGA)5	(CGA)6	(CGA)5	(CGA)6	(CGA)5	(CGA)6	(CGA)5	LOC_Os10g24050	CDS	ribosome inactivating protein, putative, expressed	CODON_CHANGE_PLUS_CODON_INSERTION	CCTCTGCTGATCTCCACTCC	AACAGGTAGGCTTGCTCGAA	134	P	A	A	NA
PMS5113	Os_Chr10	12383518	12383543	(GA)13	(GA)13	(GA)6	(GA)13	(GA)13	(GA)13	(GA)13	(GA)13	(GA)13	(GA)13	(GA)13	-	Introns/Intergenic	-	-	AGCGGAATCAGATTGTGACGAACTCC	CTCCCTGCCGATGAGCAAGTATCG	188	P	A	A	RM3283
PMS5114	Os_Chr10	12449267	12449280	(GA)7	(GA)7	(GA)7	(GA)6	(GA)7	(GA)7	(GA)7	(GA)7	(GA)7	(GA)7	(GA)6	-	Introns/Intergenic	-	-	CCCAAGAGAAGGAGGGAAAG	TCCTTGGTCTAAAAAGAGCCC	239	P	A	A	NA
PMS5115	Os_Chr10	12653577	12653590	(CT)7	(CT)7	(CT)7	(CT)6	(CT)7	(CT)7	(CT)7	(CT)7	(CT)7	(CT)7	(CT)6	-	Introns/Intergenic	-	-	ACCCTAAAAGTCCCCCTTCC	GACGGTATGTGGCGGATTT	247	P	A	A	NA
PMS5116	Os_Chr10	12760407	12760458	(AT)26	(AT)26	(AT)26	(AT)26	(AT)26	(AT)26	(AT)26	(AT)11	(AT)26	(AT)26	(AT)26	-	Introns/Intergenic	-	-	TGTAGAGCACAATGATGCAAAT	CGATTATATCGTTGTGTTCGTTGT	259	A	A	A	NA
PMS5117	Os_Chr10	12761300	12761319	(GA)10	(GA)10	(GA)9	(GA)10	(GA)10	(GA)9	(GA)10	(GA)10	(GA)10	(GA)10	(GA)10	-	Introns/Intergenic	-	-	ATAGATGCCATTGAGGTCGC	ATCTCCAAGTCTGGTGACGC	203	P	A	A	NA
PMS5118	Os_Chr10	12784352	12784369	(GCG)6	(GCG)6	(GCG)6	(GCG)5	(GCG)6	(GCG)6	(GCG)6	(GCG)6	(GCG)6	(GCG)6	(GCG)6	LOC_Os10g24850	CDS	expressed protein	CODON_CHANGE_PLUS_CODON_DELETION	ACGTCATCTTCGTGGTTTCC	TAGTGATGCCGTACTCGCTG	273	P	A	A	NA
PMS5119	Os_Chr10	12873262	12873288	(CGC)9	(CGC)9	(CGC)9	(CGC)9	(CGC)9	(CGC)9	(CGC)9	(CGC)9	(CGC)9	(CGC)9	(CGC)5	LOC_Os10g25010	CDS	OsCML8 - Calmodulin-related calcium sensor protein, expressed	CODON_DELETION	CCTGCTTCTCCCTCCTGTACCC	GCGAGCAAATACAGAGGCTACTACC	100	A	A	A	RM6142
PMS5120	Os_Chr10	12895189	12895206	(AT)9	(AT)9	(AT)9	(AT)8	(AT)9	(AT)9	(AT)9	(AT)9	(AT)9	(AT)9	(AT)9	-	Introns/Intergenic	-	-	ATCATGGTGGGTCCGTCTTA	GGTAACGTTTGTTGTGTGGG	239	P	A	A	NA
PMS5121	Os_Chr10	12992346	12992369	(CCG)8	(CCG)8	(CCG)8	(CCG)8	(CCG)8	(CCG)8	(CCG)5	(CCG)8	(CCG)8	(CCG)8	(CCG)8	LOC_Os10g25170	CDS	EBL1	CODON_DELETION	ACACCAAAAAGAACAAGCGG	TGCAAACCAATTCTTGATCG	244	A	A	P	NA

Polymorphic SSR (PMS) markers	Chromosomes	Start physical positions (bp)	End physical positions (bp)	Nipponbare	Kasalath	Nagina22	IR64	Pokkali	Bala	Tainung67	Azucena	Moroberekan	*Oryza rufipogon*	*Oryza nivara*	MSU gene locus IDs	Structural annotation	Functional annotation	SSR functional effects	Forward primers (5'-3')	Reverse primers (5'-3')	Amplified product size (bp)	Markers exhibiting polymorphism within *indica* rice	Transcrption factor genes-derived markers	Known cloned genes-derived markers	PMS markers corresponding with RM markers
PMS5122	Os_Chr10	13019702	13019722	(GCT)7	(GCT)7	(GCT)7	(GCT)7	(GCT)7	(GCT)7	(GCT)5	(GCT)7	(GCT)7	(GCT)7	(GCT)7	LOC_Os10g25220	CDS	SAP domain containing protein, expressed	CODON_DELETION	TGATGTTTGGGATACTGCTGTGG	TCATTAACCATCAGGCTCCTTCC	145	A	P	A	RM25335
PMS5123	Os_Chr10	13026374	13026387	(CT)7	(CT)11	(CT)7	(CT)7	(CT)7	(CT)11	(CT)7	(CT)7	(CT)7	(CT)7	(CT)7	-	Introns/Intergenic	-	-	ATCTCCATACCTGACGACGG	TGCCTGATGCTCTCTACCAA	207	P	A	A	NA
PMS5124	Os_Chr10	13065830	13065913	(TA)42	(TA)42	(TA)42	(TA)42	(TA)42	(TA)42	(TA)6	(TA)42	(TA)42	(TA)19	(TA)42	-	Introns/Intergenic	-	-	ATGGTGCCCCATATGAGTGT	TACCACCTCGTTTTGCATGA	187	A	A	A	NA
PMS5125	Os_Chr10	13071162	13071175	(CT)7	(CT)8	(CT)7	(CT)7	(CT)7	(CT)8	(CT)7	(CT)7	(CT)7	(CT)7	(CT)8	-	Introns/Intergenic	-	-	AGGGCGAGGGAGGAGTAGTA	GTGCCCTAGCAATCCTATGC	275	P	A	A	NA
PMS5126	Os_Chr10	13118674	13118741	(AT)34	(AT)34	(AT)34	(AT)34	(AT)34	(AT)34	(AT)34	(AT)6	(AT)34	(AT)34	(AT)34	-	Introns/Intergenic	-	-	GCACATCTTGTGATCGGATTAACG	CTCACGGACGAAGTCAAGTTTGG	394	A	A	A	RM25344
PMS5127	Os_Chr10	13134943	13134956	(TG)7	(TG)6	(TG)7	(TG)7	(TG)7	(TG)7	(TG)7	(TG)7	(TG)7	(TG)7	(TG)7	-	Introns/Intergenic	-	-	TTTTTGGCAGTGTTACGACAA	AACGGTGTACATTGCTCGAT	232	P	A	A	NA
PMS5128	Os_Chr10	13292367	13292404	(TC)19	(TC)14	(TC)19	(TC)19	(TC)19	(TC)19	(TC)19	(TC)19	(TC)19	(TC)19	(TC)19	-	Introns/Intergenic	-	-	TTTTCTCTCCTTTGGACCCC	AAATCGCATGAACTCCCAAC	254	P	A	A	NA
PMS5129	Os_Chr10	13386518	13386538	(CTC)7	(CTC)5	(CTC)7	(CTC)7	(CTC)7	(CTC)5	(CTC)7	(CTC)7	(CTC)5	(CTC)7	(CTC)7	-	Introns/Intergenic	-	-	GTTCCCAAATTTCCAGTAGTGACG	CCGAGGTCGATCAGTGAAAGG	545	P	A	A	RM25352
PMS5130	Os_Chr10	13436788	13436805	(ACG)6	(ACG)5	(ACG)6	(ACG)6	(ACG)6	(ACG)5	(ACG)6	(ACG)6	(ACG)5	(ACG)5	(ACG)6	-	Introns/Intergenic	-	-	TCCCTTCTCCTCTCCTCTCC	TCGTTGTTGTCATCGTCCTC	278	P	A	A	NA
PMS5131	Os_Chr10	13437748	13437763	(CT)8	(CT)8	(CT)8	(CT)8	(CT)8	(CT)6	(CT)8	(CT)8	(CT)8	(CT)8	(CT)8	-	Introns/Intergenic	-	-	TTCAAGTTTTGGGTTGGAGG	TTAATGCAAGGGATTAGCGG	187	P	A	A	NA
PMS5132	Os_Chr10	13534107	13534132	(AT)13	(AT)13	(AT)13	(AT)13	(AT)13	(AT)13	(AT)13	(AT)13	(AT)13	(AT)13	(AT)7	-	Introns/Intergenic	-	-	ACCTTTTATTCCGGCGAGTT	AAGCCGATTCGACTCTAAACA	230	A	A	A	NA
PMS5133	Os_Chr10	13554809	13554859	(TTA)17	(TTA)17	(TTA)17	(TTA)17	(TTA)17	(TTA)17	(TTA)17	(TTA)17	(TTA)5	(TTA)17	(TTA)17	-	Introns/Intergenic	-	-	CTGTAGTAGGTCACACCGACG	GAGCCTAACACACTACACCGC	200	A	A	A	NA
PMS5134	Os_Chr10	13621847	13621884	(TA)19	(TA)19	(TA)9	(TA)19	(TA)9	(TA)9	(TA)19	(TA)19	(TA)19	(TA)19	(TA)19	-	Introns/Intergenic	-	-	ACTCAAACAACCCACACACG	TGTGCTATCTTTGCTGGTTGA	255	P	A	A	NA

Polymorphic SSR (PMS) markers	Chromosomes	Start physical positions (bp)	End physical positions (bp)	Nipponbare	Kasalath	Nagina22	IR64	Pokkali	Bala	Tainung67	Azucena	Moroberekan	*Oryza rufipogon*	*Oryza nivara*	MSU gene locus IDs	Structural annotation	Functional annotation	SSR functional effects	Forward primers (5'-3')	Reverse primers (5'-3')	Amplified product size (bp)	Markers exhibiting polymorphism within *indica* rice	Transcrption factor genes-derived markers	Known cloned genes-derived markers	PMS markers corresponding with RM markers
PMS5135	Os_Chr10	13658415	13658444	(ACATT)6	(ACATT)6	(ACATT)6	(ACATT)6	(ACATT)6	(ACATT)6	(ACATT)6	(ACATT)6	(ACATT)6	(ACATT)5	(ACATT)6	-	Introns/Intergenic	-	-	CATCTCACAAGCTCACACGG	GGCTTGATTTGTGTGACGAA	201	A	A	A	NA
PMS5136	Os_Chr10	13695919	13695932	(AG)7	(AG)9	(AG)7	(AG)9	(AG)9	(AG)9	(AG)7	(AG)9	(AG)7	(AG)7	(AG)9	-	Introns/Intergenic	-	-	ATGCATGTGTCCACGTCTCT	TTGATGCAGCTTTGAACTCG	278	P	A	A	NA
PMS5137	Os_Chr10	13737852	13737867	(TA)8	(TA)8	(TA)8	(TA)7	(TA)8	(TA)8	(TA)8	(TA)8	(TA)6	(TA)8	(TA)8	-	Introns/Intergenic	-	-	GGTCCTTCCTCTTCTGACATAACG	TTACCAACCGGGACTAAAGATCG	258	P	A	A	RM25374
PMS5138	Os_Chr10	13773673	13773696	(TA)12	(TA)12	(TA)12	(TA)12	(TA)12	(TA)12	(TA)9	(TA)7	(TA)12	(TA)12	(TA)12	-	Introns/Intergenic	-	-	TTTCCTAGCTTGGCCTACGA	TAGAATGCGATGCGAGACAG	156	A	A	A	NA
PMS5139	Os_Chr10	13799854	13799909	(CT)28	(CT)28	(CT)28	(CT)28	(CT)28	(CT)28	(CT)28	(CT)9	(CT)28	(CT)28	(CT)28	-	Introns/Intergenic	-	-	AGCAATCTCTGCACCTCCAT	GGAGGAATGAGCTGGCTATG	254	A	A	A	NA
PMS5140	Os_Chr10	13805906	13805926	(CGC)7	(CGC)7	(CGC)7	(CGC)7	(CGC)7	(CGC)7	(CGC)7	(CGC)7	(CGC)7	(CGC)7	(CGC)6	LOC_Os10g26500	CDS	homeobox associated leucine zipper, putative, expressed	CODON_DELETION	CGAGAACAGCTCCGAGATCAACC	ATGGAGGCGTAGAACGGGATGC	111	A	A	A	RM25380
PMS5141	Os_Chr10	13824165	13824185	(GCG)7	(GCG)7	(GCG)7	(GCG)7	(GCG)7	(GCG)7	(GCG)5	(GCG)7	(GCG)7	(GCG)7	(GCG)7	LOC_Os10g26520	CDS	protein kinase domain containing protein, expressed	CODON_DELETION	GACGCTCGGTAGATCGGTAG	GAAGAAGCCAAGTCTGTGCC	188	A	A	A	NA
PMS5142	Os_Chr10	13878009	13878029	(CGC)7	(CGC)7	(CGC)7	(CGC)7	(CGC)7	(CGC)7	(CGC)5	(CGC)7	(CGC)7	(CGC)7	(CGC)7	-	Introns/Intergenic	-	-	GGACAACCTGGAGCATTGAT	GCTGAATACCCCACCTCTGA	262	A	A	A	NA
PMS5143	Os_Chr10	13919998	13920015	(CGC)6	(CGC)5	(CGC)5	(CGC)6	(CGC)6	(CGC)5	(CGC)6	(CGC)6	(CGC)6	(CGC)6	(CGC)6	LOC_Os10g26660	URR	expressed protein	-	GTGCTTTCGGACTTGGAGAG	ACGGGTCAGAGAGAAGCAGA	188	P	A	A	NA
PMS5144	Os_Chr10	14125175	14125190	(AT)8	(AT)8	(AT)6	(AT)8	(AT)8	(AT)8	(AT)8	(AT)8	(AT)8	(AT)8	(AT)8	-	Introns/Intergenic	-	-	TGCCCAAAGATACAACACCA	TAGCTTGCCGAGGATCTCAT	278	P	A	A	NA
PMS5145	Os_Chr10	14245558	14245569	(AG)6	(AG)7	(AG)6	(AG)6	(AG)6	(AG)6	(AG)6	(AG)6	(AG)6	(AG)6	(AG)6	-	Introns/Intergenic	-	-	CATCAGAAGACTGATCGGCA	AGAGCAATGGCGTTACAACC	206	P	A	A	NA
PMS5146	Os_Chr10	14361998	14362015	(CAC)6	(CAC)6	(CAC)6	(CAC)6	(CAC)6	(CAC)6	(CAC)6	(CAC)6	(CAC)6	(CAC)5	(CAC)6	LOC_Os10g27230	URR	endonuclease/exonuclease/phosphatase family domain containing protein, expressed	-	CTCTCCTCCTCTTCTCCCCA	GTGCGTGTGTGATTCATTCC	190	A	A	A	NA
PMS5147	Os_Chr10	14400799	14400818	(TG)10	(TG)10	(TG)10	(TG)10	(TG)10	(TG)8	(TG)10	(TG)10	(TG)10	(TG)10	(TG)10	-	Introns/Intergenic	-	-	CGGAGAGCAAGGATAAGCAC	CCTGCTACCCCAAATCATGT	254	P	A	A	NA

Polymorphic SSR (PMS) markers	Chromosomes	Start physical positions (bp)	End physical positions (bp)	Nipponbare	Kasalath	Nagina22	IR64	Pokkali	Bala	Tainung67	Azucena	Moroberekan	*Oryza rufipogon*	*Oryza nivara*	MSU gene locus IDs	Structural annotation	Functional annotation	SSR functional effects	Forward primers (5'-3')	Reverse primers (5'-3')	Amplified product size (bp)	Markers exhibiting polymorphism within *indica* rice	Transcrption factor genes-derived markers	Known cloned genes-derived markers	PMS markers corresponding with RM markers
PMS5148	Os_Chr10	14438491	14438508	(GAC)6	(GAC)6	(GAC)6	(GAC)6	(GAC)6	(GAC)6	(GAC)6	(GAC)6	(GAC)6	(GAC)7	(GAC)6	LOC_Os10g27360	CDS	no apical meristem protein, putative, expressed	CODON_INSERTION	AGGAGGGCTACCTCATCGTC	AATTCCAATGCTCAAATCGG	237	A	P	A	NA
PMS5149	Os_Chr10	14447733	14447750	(GCG)6	(GCG)6	(GCG)6	(GCG)6	(GCG)6	(GCG)6	(GCG)6	(GCG)6	(GCG)6	(GCG)6	(GCG)5	LOC_Os10g27390	CDS	no apical meristem protein, putative, expressed	CODON_DELETION	CAAAGTCAAGCTCATCGCAA	ATGGACATCCTCCTTACCCC	244	A	P	A	NA
PMS5150	Os_Chr10	14644679	14644714	(ATCT)9	(ATCT)9	(ATCT)9	(ATCT)5	(ATCT)5	(ATCT)9	(ATCT)9	(ATCT)9	(ATCT)9	(ATCT)9	(ATCT)5	-	Introns/Intergenic	-	-	GGCCTTAAGTGTTATTCCCTTTGC	GCGGCTTAGGAGCGTTTATAGG	312	P	A	A	RM25416
PMS5151	Os_Chr10	14658942	14658959	(TCG)6	(TCG)6	(TCG)6	(TCG)6	(TCG)6	(TCG)6	(TCG)6	(TCG)6	(TCG)6	(TCG)5	(TCG)5	LOC_Os10g28210	CDS	plant-specific domain TIGR01615 family protein, expressed	CODON_DELETION	ACCCCACCTACCTTGATCGT	GGGAGAAGGATGAGGTGGA	223	A	A	A	NA
PMS5152	Os_Chr10	14708560	14708573	(TA)7	(TA)7	(TA)7	(TA)6	(TA)7	(TA)7	(TA)7	(TA)7	(TA)7	(TA)7	(TA)7	-	Introns/Intergenic	-	-	TGGAACGGAGGGATTAAGTG	ACGGAATGTCAAACACACCA	247	P	A	A	NA
PMS5153	Os_Chr10	14713440	14713453	(GA)7	(GA)9	(GA)7	(GA)7	(GA)7	(GA)7	(GA)7	(GA)7	(GA)7	(GA)7	(GA)7	-	Introns/Intergenic	-	-	GCCGTCAGTTTTCGATCAAG	CGTTCTATCGAATGGACGGT	143	P	A	A	NA
PMS5154	Os_Chr10	14723172	14723209	(GA)19	(GA)19	(GA)19	(GA)18	(GA)19	(GA)19	(GA)19	(GA)19	(GA)19	(GA)19	(GA)19	-	Introns/Intergenic	-	-	CTTTTTGTGCTGCCATGAGA	CGTGCATTAGCAAGTCCAAA	271	P	A	A	NA
PMS5155	Os_Chr10	14740496	14740513	(TGT)6	(TGT)7	(TGT)7	(TGT)6	(TGT)6	(TGT)7	(TGT)6	(TGT)6	(TGT)6	(TGT)7	(TGT)5	LOC_Os10g28330	CDS	Ehd2|RID1	CODON_DELETION	TGTTGTTGCAGGAACAGGAG	AACAACAGCAGCAGCATCAC	203	P	P	P	NA
PMS5156	Os_Chr10	14743250	14743263	(TC)7	(TC)9	(TC)7	(TC)7	(TC)7	(TC)9	(TC)7	(TC)7	(TC)7	(TC)7	(TC)9	-	Introns/Intergenic	-	-	GAAAGCTAGCTACGCCATGC	TGGAACTGATCAATGCCTGA	161	P	A	A	NA
PMS5157	Os_Chr10	14872133	14872150	(GGC)6	(GGC)6	(GGC)6	(GGC)6	(GGC)6	(GGC)6	(GGC)5	(GGC)6	(GGC)6	(GGC)6	(GGC)6	LOC_Os10g28570	URR	expressed protein	-	ACTGGAGAAGCTGACCTGGA	TCTTGATTGTATCCCTGGGC	232	A	A	A	NA
PMS5158	Os_Chr10	14992656	14992673	(GA)9	(GA)7	(GA)9	(GA)9	(GA)9	(GA)9	(GA)9	(GA)9	(GA)9	(GA)9	(GA)9	-	Introns/Intergenic	-	-	GAGGGAGCGGAGAGGGAG	AAATCCCTCCGCAAAAGTCT	180	P	A	A	NA
PMS5159	Os_Chr10	15002643	15002660	(GA)9	(GA)7	(GA)9	(GA)9	(GA)9	(GA)9	(GA)9	(GA)9	(GA)9	(GA)9	(GA)9	-	Introns/Intergenic	-	-	GAGGGAGCGGAGAGGGAG	AAATCCCTCCGCAAAAGTCT	182	P	A	A	NA
PMS5160	Os_Chr10	15020264	15020278	(GTC)5	(GTC)6	(GTC)6	(GTC)5	(GTC)5	(GTC)6	(GTC)5	(GTC)5	(GTC)5	(GTC)5	(GTC)5	LOC_Os10g28810	CDS	MBTB37 - Bric-a-Brac, Tramtrack, Broad Complex BTB domain with Meprin and TRAF Homology MATH domain, expressed	CODON_INSERTION	GCAGATCAGCTTCAGCCTCT	CCTTCATGTACACCGACACG	170	P	A	A	NA

Polymorphic SSR (PMS) markers	Chromosomes	Start physical positions (bp)	End physical positions (bp)	Nipponbare	Kasalath	Nagina22	IR64	Pokkali	Bala	Tainung67	Azucena	Moroberekan	*Oryza rufipogon*	*Oryza nivara*	MSU gene locus IDs	Structural annotation	Functional annotation	SSR functional effects	Forward primers (5'-3')	Reverse primers (5'-3')	Amplified product size (bp)	Markers exhibiting polymorphism within *indica* rice	Transcrption factor genes-derived markers	Known cloned genes-derived markers	PMS markers corresponding with RM markers
PMS5161	Os_Chr10	15204443	15204464	(TA)11	(TA)11	(TA)11	(TA)11	(TA)11	(TA)11	(TA)11	(TA)11	(TA)11	(TA)10	(TA)11	-	Introns/Intergenic	-	-	TGAGTTTGACCCATGGGAGAGG	CCTGTCAAAGGGACATTGACTATCG	349	A	A	A	RM25429
PMS5162	Os_Chr10	15336553	15336578	(TA)13	(TA)13	(TA)7	(TA)8	(TA)9	(TA)13	(TA)13	(TA)11	(TA)13	(TA)13	(TA)13	-	Introns/Intergenic	-	-	TATTTACGGAGCCACCATAACG	TATATTTGGCTCGCAAGTCTGC	266	P	A	A	RM25434
PMS5163	Os_Chr10	15357354	15357374	(GAG)7	(GAG)7	(GAG)7	(GAG)7	(GAG)7	(GAG)7	(GAG)7	(GAG)7	(GAG)7	(GAG)8	(GAG)7	LOC_Os10g29549	CDS	expressed protein	CODON_CHANGE_PLUS_CODON_INSERTION	AGCGGTGATGGTGGGAATGAGG	CACCAGCAAGAAGCCCGAATCC	100	A	A	A	RM25435
PMS5164	Os_Chr10	15530599	15530622	(TA)12	(TA)12	(TA)12	(TA)12	(TA)12	(TA)12	(TA)12	(TA)8	(TA)12	(TA)12	(TA)12	-	Introns/Intergenic	-	-	CCCGTACCTGATACTTCCTTCG	GCCGTAAAGGCACTTTGTTATCG	510	A	A	A	RM25444
PMS5165	Os_Chr10	15537730	15537775	(TA)23	(TA)23	(TA)23	(TA)23	(TA)23	(TA)23	(TA)23	(TA)23	(TA)23	(TA)7	(TA)10	-	Introns/Intergenic	-	-	TTGATTGTGTCAGGTGCCAT	TCAAGCGCTTCCTCTACTCC	217	A	A	A	NA
PMS5166	Os_Chr10	15627422	15627445	(TA)12	(TA)10	(TA)12	(TA)12	(TA)6	(TA)12	(TA)12	(TA)12	(TA)12	(TA)7	(TA)12	-	Introns/Intergenic	-	-	CAACTTTCTCAACTCTCCTCACTCG	CTCTCTTGGTATACTAGGCGTGTGC	426	P	A	A	RM25451
PMS5167	Os_Chr10	15708543	15708566	(AT)12	(AT)12	(AT)8	(AT)7	(AT)12	(AT)8	(AT)7	(AT)12	(AT)12	(AT)12	(AT)12	-	Introns/Intergenic	-	-	GGATGGATTGATCGGTGCTTACC	GAGTGAGGGAGGTGGGTTTGACC	178	P	A	A	RM25459
PMS5168	Os_Chr10	15878028	15878045	(CGG)6	(CGG)5	(CGG)6	(CGG)6	(CGG)6	(CGG)5	(CGG)6	(CGG)6	(CGG)6	(CGG)5	(CGG)6	LOC_Os10g30540	CDS	lectin-like receptor kinase, putative, expressed	CODON_CHANGE_PLUS_CODON_DELETION	AACGACACGGACATGAACAA	ACATCCCGAGATACTGCTGC	223	P	A	A	NA
PMS5169	Os_Chr10	15916820	15916877	(TA)29	(TA)18	(TA)7	(TA)9	(TA)10	(TA)29	(TA)29	(TA)9	(TA)29	(TA)29	(TA)14	-	Introns/Intergenic	-	-	TGAATCCAAAACGCCAAACT	TTTTGAAAAATTTGGGCGAA	234	P	A	A	NA
PMS5170	Os_Chr10	15917271	15917316	(TA)23	(TA)21	(TA)10	(TA)23	(TA)11	(TA)19	(TA)23	(TA)23	(TA)23	(TA)10	(TA)23	-	Introns/Intergenic	-	-	CCATGGAGCACCAAACAAAT	AATGTGACACGCCAAAACCT	270	P	A	A	NA
PMS5171	Os_Chr10	15929074	15929097	(CGC)8	(CGC)8	(CGC)8	(CGC)8	(CGC)7	(CGC)8	(CGC)8	(CGC)8	(CGC)8	(CGC)8	(CGC)8	-	Introns/Intergenic	-	-	GCATGGCCAACGTGTAAGTA	ATTACCTTGCGTCTCCTCCA	202	P	A	A	NA
PMS5172	Os_Chr10	15929781	15929801	(GCG)7	(GCG)7	(GCG)7	(GCG)7	(GCG)7	(GCG)7	(GCG)6	(GCG)7	(GCG)7	(GCG)7	(GCG)7	-	Introns/Intergenic	-	-	AAATGAACGGCACAGATCAA	ACTCGTGTTGGGGATTCAAG	262	A	A	A	NA
PMS5173	Os_Chr10	16029442	16029474	(AAG)11	(AAG)11	(AAG)11	(AAG)6	(AAG)11	(AAG)11	(AAG)11	(AAG)11	(AAG)11	(AAG)11	(AAG)11	-	Introns/Intergenic	-	-	GAGGAGGTGTCCAAGGAGAACG	CAACCAATGAGCTCTGTTGAACC	114	P	A	A	RM25470

Polymorphic SSR (PMS) markers	Chromosomes	Start physical positions (bp)	End physical positions (bp)	Nipponbare	Kasalath	Nagina22	IR64	Pokkali	Bala	Tainung67	Azucena	Moroberekan	*Oryza rufipogon*	*Oryza nivara*	MSU gene locus IDs	Structural annotation	Functional annotation	SSR functional effects	Forward primers (5'-3')	Reverse primers (5'-3')	Amplified product size (bp)	Markers exhibiting polymorphism within *indica* rice	Transcrption factor genes-derived markers	Known cloned genes-derived markers	PMS markers corresponding with RM markers
PMS5174	Os_Chr10	16110393	16110410	(GTC)6	(GTC)6	(GTC)6	(GTC)6	(GTC)6	(GTC)6	(GTC)6	(GTC)6	(GTC)6	(GTC)5	(GTC)6	LOC_Os10g30890	CDS	expressed protein	CODON_DELETION	TTCTTCCTAGCGTCACCGTT	CGGAGATTGCTGATCCTCAT	256	A	A	A	NA
PMS5175	Os_Chr10	16226520	16226539	(TC)10	(TC)10	(TC)10	(TC)11	(TC)11	(TC)11	(TC)10	(TC)10	(TC)10	(TC)11	(TC)10	-	Introns/Intergenic	-	-	CTGAGAGCGCCGAGAGAGAAGG	GAAGCCAATATTCCTCTCCAAGTGC	326	P	A	A	RM25484
PMS5176	Os_Chr10	16234764	16234817	(TA)27	(TA)27	(TA)27	(TA)9	(TA)27	(TA)16	(TA)27	(TA)27	(TA)27	(TA)27	(TA)27	-	Introns/Intergenic	-	-	ACCAGACACTTGCTGGCTTC	TGTTCTGTTCGGTTCTGCAC	236	P	A	A	NA
PMS5177	Os_Chr10	16333829	16333842	(TA)7	(TA)7	(TA)7	(TA)7	(TA)7	(TA)7	(TA)7	(TA)8	(TA)8	(TA)7	(TA)7	-	Introns/Intergenic	-	-	AGAACAACAAAGTGGTGGGC	TTCTTGCGCATTACCATTCA	238	A	A	A	NA
PMS5178	Os_Chr10	16361881	16361898	(GCT)6	(GCT)5	(GCT)5	(GCT)6	(GCT)6	(GCT)5	(GCT)6	(GCT)6	(GCT)6	(GCT)6	(GCT)6	-	Introns/Intergenic	-	-	TCAGCTGATGCATTACCCAG	CAGGGATTTAACGCGTTGTT	276	P	A	A	NA
PMS5179	Os_Chr10	16376556	16376569	(CG)7	(CG)7	(CG)6	(CG)7	(CG)7	(CG)7	(CG)7	(CG)7	(CG)7	(CG)7	(CG)7	-	Introns/Intergenic	-	-	CCCTGTTGACCAGTTGAGGT	GGTTAGTGGCTGCAAAAGGA	158	P	A	A	NA
PMS5180	Os_Chr10	16382641	16382656	(TA)8	(TA)8	(TA)8	(TA)8	(TA)8	(TA)8	(TA)8	(TA)7	(TA)8	(TA)8	(TA)8	-	Introns/Intergenic	-	-	TTATACACCAAACTTTCTAATTGCAT	TTCACTCCCATTCTTTTCCG	227	A	A	A	NA
PMS5181	Os_Chr10	16401949	16401966	(AT)9	(AT)9	(AT)9	(AT)9	(AT)9	(AT)9	(AT)9	(AT)9	(AT)9	(AT)9	(AT)7	-	Introns/Intergenic	-	-	GCACACATGGTCTGTTTCCA	AAGACGAATGGTCAAACGTG	141	A	A	A	NA
PMS5182	Os_Chr10	16417891	16417914	(TC)12	(TC)12	(TC)12	(TC)12	(TC)12	(TC)12	(TC)12	(TC)11	(TC)12	(TC)12	(TC)12	-	Introns/Intergenic	-	-	TTTGCCTCCCTCTGTTTTTG	GATAGCAGGAAGAGGCGTTG	218	A	A	A	NA
PMS5183	Os_Chr10	16503867	16503906	(AT)20	(AT)17	(AT)20	(AT)20	(AT)20	(AT)20	(AT)20	(AT)20	(AT)20	(AT)20	(AT)20	-	Introns/Intergenic	-	-	TCAGTTAGGACCATCGTAAAAACA	TTGATGGTATGTTTGTTCAGTGTAAA	201	P	A	A	NA
PMS5184	Os_Chr10	16596547	16596564	(CTC)6	(CTC)6	(CTC)6	(CTC)6	(CTC)6	(CTC)6	(CTC)6	(CTC)6	(CTC)6	(CTC)6	(CTC)5	-	Introns/Intergenic	-	-	CCGCCCCTTATCTCTTCTCT	GTCCTATGCGCATGCCTC	207	A	A	A	NA
PMS5185	Os_Chr10	16750804	16750824	(CGC)7	(CGC)7	(CGC)7	(CGC)7	(CGC)6	(CGC)7	(CGC)7	(CGC)7	(CGC)7	(CGC)7	(CGC)7	LOC_Os10g31900	URR	RNA recognition motif containing protein, putative, expressed	-	TGGGCTGTACCCATCCTCTTACCC	TAGGTGGTGGCGTCCTGGTTCC	251	P	A	A	RM25514
PMS5186	Os_Chr10	16760612	16760626	(CCG)5	(CCG)5	(CCG)5	(CCG)5	(CCG)5	(CCG)5	(CCG)6	(CCG)5	(CCG)6	(CCG)5	(CCG)5	LOC_Os10g31910	CDS	pectate lyase precursor, putative, expressed	CODON_INSERTION	CAGTGGTCGATGGGGTTC	GTTTCTTGGCTAATGGCAGC	115	A	A	A	NA

Polymorphic SSR (PMS) markers	Chromosomes	Start physical positions (bp)	End physical positions (bp)	Nipponbare	Kasalath	Nagina22	IR64	Pokkali	Bala	Tainung67	Azucena	Moroberekan	*Oryza rufipogon*	*Oryza nivara*	MSU gene locus IDs	Structural annotation	Functional annotation	SSR functional effects	Forward primers (5'-3')	Reverse primers (5'-3')	Amplified product size (bp)	Markers exhibiting polymorphism within *indica* rice	Transcrption factor genes-derived markers	Known cloned genes-derived markers	PMS markers corresponding with RM markers
PMS5187	Os_Chr10	16763053	16763076	(CT)12	(CT)7	(CT)7	(CT)12	(CT)12	(CT)7	(CT)12	(CT)12	(CT)12	(CT)12	(CT)12	-	Introns/Intergenic	-	-	TTGCTGCTGTAATCATCCCA	CGGAAGGCAAAAGATGATGT	215	P	A	A	NA
PMS5188	Os_Chr10	16937145	16937194	(AT)25	(AT)25	(AT)25	(AT)8	(AT)7	(AT)6	(AT)25	(AT)25	(AT)7	(AT)25	(AT)25	-	Introns/Intergenic	-	-	TGATGATTAAACCCAAAAGGAAA	GCGAGCGAAAAATGAGAGAG	254	P	A	A	NA
PMS5189	Os_Chr10	16943740	16943760	(TCT)7	(TCT)7	(TCT)7	(TCT)7	(TCT)7	(TCT)6	(TCT)7	(TCT)7	(TCT)7	(TCT)7	(TCT)6	-	Introns/Intergenic	-	-	TATCCGCACTACACTGCGTC	TGTGCGTACCGTGAGAGCTA	214	P	A	A	NA
PMS5190	Os_Chr10	17006313	17006327	(GCG)5	(GCG)5	(GCG)6	(GCG)5	(GCG)5	(GCG)5	(GCG)5	(GCG)5	(GCG)5	(GCG)5	(GCG)5	LOC_Os10g32444	CDS	expressed protein	CODON_INSERTION	GTGTTCTGGCAGTTCTGCTG	GAGGAGGGAGGGGACGAG	197	P	A	A	NA
PMS5191	Os_Chr10	17032877	17032898	(AT)11	(AT)11	(AT)11	(AT)11	(AT)11	(AT)11	(AT)11	(AT)11	(AT)11	(AT)6	(AT)10	-	Introns/Intergenic	-	-	TCAATTTCAACAATGGTGCC	CGCATGCTAATTTGAGACGA	245	A	A	A	NA
PMS5192	Os_Chr10	17082413	17082426	(TA)7	(TA)7	(TA)6	(TA)7	(TA)7	(TA)6	(TA)7	(TA)7	(TA)7	(TA)7	(TA)7	-	Introns/Intergenic	-	-	ATGAATGTTCCTCCTCCAGC	CTACCTGATCCCCAAACCCT	248	P	A	A	NA
PMS5193	Os_Chr10	17140996	17141010	(CGG)5	(CGG)7	(CGG)5	(CGG)5	(CGG)5	(CGG)5	(CGG)5	(CGG)5	(CGG)5	(CGG)5	(CGG)5	LOC_Os10g32740	CDS	zinc finger family protein, putative, expressed	CODON_INSERTION	ATTAGCTACTGCATTCGGCG	TACCTACCAGACGCCCTTCA	234	P	A	A	NA
PMS5194	Os_Chr10	17215966	17215983	(TC)9	(TC)9	(TC)11	(TC)9	(TC)9	(TC)11	(TC)9	(TC)9	(TC)9	(TC)9	(TC)6	-	Introns/Intergenic	-	-	CTCCGCTCGGTACATACCAT	TGCTGATTAGGCAGGTGTCA	104	P	A	A	NA
PMS5195	Os_Chr10	17229334	17229351	(GCG)6	(GCG)6	(GCG)6	(GCG)6	(GCG)6	(GCG)6	(GCG)6	(GCG)5	(GCG)6	(GCG)6	(GCG)6	LOC_Os10g32920	URR	HLL	-	CACGTACCTCGCTTCGACAT	CATTAGCAGTAAATGGGCCG	165	A	A	P	NA
PMS5196	Os_Chr10	17261476	17261493	(GT)9	(GT)8	(GT)9	(GT)9	(GT)9	(GT)8	(GT)9	(GT)9	(GT)9	(GT)9	(GT)9	LOC_Os10g32970	DRR	p28	-	TGCTTTTCTCACTGCACACC	AGATGAAATCCTGCGATGCT	246	P	A	P	NA
PMS5197	Os_Chr10	17267510	17267527	(CGC)6	(CGC)6	(CGC)6	(CGC)6	(CGC)6	(CGC)7	(CGC)6	(CGC)7	(CGC)7	(CGC)6	(CGC)5	LOC_Os10g32990	CDS	receptor-like protein kinase 2 precursor, putative, expressed	CODON_DELETION	CTCTGATCCACCACGTCCTT	TACATGAGGGTGACGACCAA	218	P	P	A	NA
PMS5198	Os_Chr10	17270456	17270476	(GCC)7	(GCC)7	(GCC)7	(GCC)7	(GCC)7	(GCC)7	(GCC)7	(GCC)7	(GCC)7	(GCC)5	(GCC)5	LOC_Os10g32990	CDS	receptor-like protein kinase 2 precursor, putative, expressed	CODON_DELETION	AGCAGTCGTGGTGGAGGTAG	GAGAGGAGCTTCGAGAACGA	247	A	P	A	NA
PMS5199	Os_Chr10	17353725	17353742	(GCG)6	(GCG)6	(GCG)6	(GCG)6	(GCG)6	(GCG)6	(GCG)6	(GCG)6	(GCG)6	(GCG)5	(GCG)6	-	Introns/Intergenic	-	-	GTCCCACCTTTCTCTCCTCC	GGGCTTTTGTGATTTTCCAA	265	A	A	A	NA

Polymorphic SSR (PMS) markers	Chromosomes	Start physical positions (bp)	End physical positions (bp)	Nipponbare	Kasalath	Nagina22	IR64	Pokkali	Bala	Tainung67	Azucena	Moroberekan	*Oryza rufipogon*	*Oryza nivara*	MSU gene locus IDs	Structural annotation	Functional annotation	SSR functional effects	Forward primers (5'-3')	Reverse primers (5'-3')	Amplified product size (bp)	Markers exhibiting polymorphism within *indica* rice	Transcrption factor genes-derived markers	Known cloned genes-derived markers	PMS markers corresponding with RM markers
PMS5200	Os_Chr10	17374975	17375002	(TC)14	(TC)14	(TC)14	(TC)7	(TC)14	(TC)6	(TC)14	(TC)7	(TC)7	(TC)14	(TC)14	-	Introns/Intergenic	-	-	GTACGAGCTGTTCGAGAGGATGG	AGGCAGAAGAGAACAGGGTATGTAGC	283	P	A	A	RM5392
PMS5201	Os_Chr10	17456387	17456476	(TTA)30	(TTA)28	(TTA)8	(TTA)30	(TTA)30	(TTA)8	(TTA)30	(TTA)18	(TTA)30	(TTA)30	(TTA)8	-	Introns/Intergenic	-	-	TCAGTTCGGGAAGTCAGATTCCTACC	CCAGTCTTACGGAGGTGCTCATAGG	195	P	A	A	RM25544
PMS5202	Os_Chr10	17458393	17458408	(AT)8	(AT)8	(AT)8	(AT)8	(AT)8	(AT)8	(AT)8	(AT)8	(AT)7	(AT)8	(AT)8	-	Introns/Intergenic	-	-	TCCCGCACTTAACAATCACA	GCCACGAGTCAGCATATGAA	273	A	A	A	NA
PMS5203	Os_Chr10	17556321	17556334	(AG)7	(AG)6	(AG)6	(AG)7	(AG)6	(AG)6	(AG)7	(AG)7	(AG)7	(AG)7	(AG)6	-	Introns/Intergenic	-	-	CAAATCACGTCGTCCACATC	GGCATCTTGACTCCTCCTTG	213	P	A	A	NA
PMS5204	Os_Chr10	17593185	17593256	(TTA)24	(TTA)24	(TTA)5	(TTA)5	(TTA)15	(TTA)24	(TTA)24	(TTA)24	(TTA)24	(TTA)24	(TTA)15	-	Introns/Intergenic	-	-	CGATTTATTGCGTGGCCGTACC	CTTCACCCTTCAGCACAGATTCG	447	P	A	A	RM25553
PMS5205	Os_Chr10	17692738	17692789	(AT)26	(AT)12	(AT)26	(AT)26	(AT)26	(AT)26	(AT)26	(AT)26	(AT)10	(AT)26	(AT)26	-	Introns/Intergenic	-	-	AGGGCACTAAGATCGGAACAACC	TGCATGTCTTACCCTTGTTCACC	548	P	A	A	RM25560
PMS5206	Os_Chr10	17706219	17706232	(CG)7	(CG)6	(CG)7	(CG)7	(CG)7	(CG)7	(CG)7	(CG)7	(CG)7	(CG)7	(CG)7	-	Introns/Intergenic	-	-	CCCTGTTGACTGGTTGAGGT	TGCAAAAGAATAGCGACGTG	112	P	A	A	NA
PMS5207	Os_Chr10	17752042	17752062	(CCG)7	(CCG)7	(CCG)7	(CCG)7	(CCG)7	(CCG)7	(CCG)5	(CCG)7	(CCG)7	(CCG)7	(CCG)7	LOC_Os10g33670	CDS	expressed protein	CODON_DELETION	GAGGATGTGGCAGTGTCAATGG	TGCACCGATCTCCTCTTTCTATCC	258	A	A	A	RM25564
PMS5208	Os_Chr10	17837792	17837809	(CA)9	(CA)9	(CA)9	(CA)9	(CA)9	(CA)9	(CA)9	(CA)9	(CA)9	(CA)9	(CA)8	-	Introns/Intergenic	-	-	TAAGCCAAAATGCACCATTG	TCCTGTCCAATGATCACACG	224	A	A	A	NA
PMS5209	Os_Chr10	17842785	17842808	(CT)12	(CT)9	(CT)9	(CT)9	(CT)9	(CT)9	(CT)12	(CT)12	(CT)12	(CT)12	(CT)9	-	Introns/Intergenic	-	-	AATGAGAGTGGGAAGAACACATCC	GGGACACACTCAGAAGTGAGAGC	235	A	A	A	RM1125
PMS5210	Os_Chr10	17941157	17941232	(AT)38	(AT)35	(AT)38	(AT)38	(AT)7	(AT)38	(AT)38	(AT)16	(AT)38	(AT)38	(AT)31	-	Introns/Intergenic	-	-	CGACGCCTAAACACAGAGAAACG	TATCCCTTGATGATCGGATCTGG	347	P	A	A	RM25587
PMS5211	Os_Chr10	17972992	17973005	(CT)7	(CT)7	(CT)7	(CT)7	(CT)7	(CT)7	(CT)7	(CT)7	(CT)7	(CT)6	(CT)7	-	Introns/Intergenic	-	-	GATGGTTTCTCAACCACTCCA	CATCGGGTGGATGAGTTTTT	235	A	A	A	NA
PMS5212	Os_Chr10	17980165	17980185	(CCG)7	(CCG)7	(CCG)5	(CCG)7	(CCG)7	(CCG)7	(CCG)7	(CCG)6	(CCG)7	(CCG)7	(CCG)7	-	Introns/Intergenic	-	-	CAATATGGGCCTCCAAAGAATCACC	CCGCCGAGGGATAATACGAGAGC	379	P	A	A	RM25591

Polymorphic SSR (PMS) markers	Chromosomes	Start physical positions (bp)	End physical positions (bp)	Nipponbare	Kasalath	Nagina22	IR64	Pokkali	Bala	Tainung67	Azucena	Moroberekan	*Oryza rufipogon*	*Oryza nivara*	MSU gene locus IDs	Structural annotation	Functional annotation	SSR functional effects	Forward primers (5'-3')	Reverse primers (5'-3')	Amplified product size (bp)	Markers exhibiting polymorphism within *indica* rice	Transcrption factor genes-derived markers	Known cloned genes-derived markers	PMS markers corresponding with RM markers
PMS5213	Os_Chr10	18005569	18005631	(TAG)21	(TAG)21	(TAG)21	(TAG)21	(TAG)21	(TAG)21	(TAG)21	(TAG)21	(TAG)21	(TAG)21	(TAG)19	LOC_Os10g33900	DRR	possible lysine decarboxylase domain containing protein, expressed	-	TCAGGGGCAGCTTGAATACT	CACACATTGCATTACGAGGG	241	A	A	A	NA
PMS5214	Os_Chr10	18216253	18216270	(AAG)6	(AAG)5	(AAG)6	(AAG)6	(AAG)6	(AAG)5	(AAG)6	(AAG)6	(AAG)5	(AAG)6	(AAG)6	-	Introns/Intergenic	-	-	GCAGCAATGAGTTTTGCGTA	GCAACCAGCTTTCGTTTGA	126	P	A	A	NA
PMS5215	Os_Chr10	18262369	18262438	(TC)35	(TC)34	(TC)35	(TC)35	(TC)35	(TC)34	(TC)35	(TC)18	(TC)35	(TC)35	(TC)34	LOC_Os10g34220	DRR	TKL_IRAK_DUF26-lf.1 DUF26 kinases have homology to DUF26 containing loci, expressed	-	TCCTCCTCCAGTTCATCTTCTGC	GATCCGAGCATACAAGGAGTGC	350	P	A	A	RM25610
PMS5216	Os_Chr10	18262976	18263003	(TA)14	(TA)14	(TA)14	(TA)14	(TA)7	(TA)14	(TA)7	(TA)14	(TA)14	(TA)14	(TA)14	-	Introns/Intergenic	-	-	GATCACCTGCCATCATACTAAACC	ATAGAGAGCAAACGGTCAATGG	164	P	A	A	RM25611
PMS5217	Os_Chr10	18271237	18271286	(CT)25	(CT)28	(CT)25	(CT)25	(CT)25	(CT)25	(CT)25	(CT)25	(CT)25	(CT)25	(CT)25	-	Introns/Intergenic	-	-	TCAGTGGGCAGACATTCAAG	CAGGTCACAATCTCACCCCT	268	P	A	A	NA
PMS5218	Os_Chr10	18285516	18285533	(CCT)6	(CCT)6	(CCT)5	(CCT)6	(CCT)6	(CCT)6	(CCT)6	(CCT)5	(CCT)6	(CCT)6	(CCT)5	LOC_Os10g34270	URR	expressed protein	-	CACCGAGGCTCTACTTTTGC	GGAGACGAAGAAAGTGTGGC	213	P	A	A	NA
PMS5219	Os_Chr10	18314116	18314133	(GGC)6	(GGC)6	(GGC)6	(GGC)6	(GGC)5	(GGC)6	(GGC)6	(GGC)6	(GGC)6	(GGC)6	(GGC)6	LOC_Os10g34350	CDS	aminotransferase, classes I and II, domain containing protein, expressed	CODON_DELETION	CCTGTACTGGTTGAGGTCGG	TGTATCTTATCCAGAACGCCG	231	P	A	A	NA
PMS5220	Os_Chr10	18376038	18376064	(CGC)9	(CGC)9	(CGC)9	(CGC)9	(CGC)9	(CGC)9	(CGC)7	(CGC)9	(CGC)9	(CGC)5	(CGC)9	LOC_Os10g34440	CDS	expressed protein	CODON_DELETION	GTACAGGCACGAGCAGAGGAAGG	CGTGCAATCCTTGTCGTCATCG	391	A	A	A	RM6150
PMS5221	Os_Chr10	18393370	18393390	(CCG)7	(CCG)7	(CCG)7	(CCG)7	(CCG)7	(CCG)7	(CCG)7	(CCG)8	(CCG)7	(CCG)7	(CCG)7	LOC_Os10g34480	CDS	cytochrome P450, putative, expressed	CODON_INSERTION	GCGGAGGAACTTGTCGGAGTAGG	AGGTTCGCCTACGACGTCATCC	153	A	A	A	RM25616
PMS5222	Os_Chr10	18404283	18404303	(GCC)7	(GCC)6	(GCC)7	(GCC)7	(GCC)6	(GCC)6	(GCC)7	(GCC)6	(GCC)7	(GCC)5	(GCC)7	LOC_Os10g34500	CDS	ZOS10-05 - C2H2 zinc finger protein, expressed	CODON_DELETION	ATCACACTTAGGCTGCTCCG	AACGTGAAGTGCATGACGC	256	P	A	A	NA
PMS5223	Os_Chr10	18428503	18428564	(AT)31	(AT)31	(AT)31	(AT)8	(AT)31	(AT)6	(AT)31	(AT)9	(AT)31	(AT)19	(AT)10	-	Introns/Intergenic	-	-	CGATTACACCATTGTGTTCGTT	TTCCGTTTTGTGTTAAGTTACTTTT	275	P	A	A	NA
PMS5224	Os_Chr10	18428859	18428928	(AT)35	(AT)35	(AT)35	(AT)7	(AT)7	(AT)35	(AT)35	(AT)35	(AT)35	(AT)35	(AT)9	-	Introns/Intergenic	-	-	GGATTTACTTTAAATAACATGCCAAA	TTGCAACAAGATCTCACATGATT	262	P	A	A	NA
PMS5225	Os_Chr10	18429537	18429557	(CGC)7	(CGC)9	(CGC)9	(CGC)7	(CGC)7	(CGC)9	(CGC)7	(CGC)7	(CGC)7	(CGC)7	(CGC)7	LOC_Os10g34560	CDS	expressed protein	CODON_INSERTION	CACGATGCTATTGCTACCGA	GACGTGTCCGTGTCGTACC	263	P	A	A	NA

Polymorphic SSR (PMS) markers	Chromosomes	Start physical positions (bp)	End physical positions (bp)	Nipponbare	Kasalath	Nagina22	IR64	Pokkali	Bala	Tainung67	Azucena	Moroberekan	*Oryza rufipogon*	*Oryza nivara*	MSU gene locus IDs	Structural annotation	Functional annotation	SSR functional effects	Forward primers (5'-3')	Reverse primers (5'-3')	Amplified product size (bp)	Markers exhibiting polymorphism within *indica* rice	Transcrption factor genes-derived markers	Known cloned genes-derived markers	PMS markers corresponding with RM markers
PMS5226	Os_Chr10	18509750	18509761	(AC)6	(AC)6	(AC)6	(AC)6	(AC)6	(AC)6	(AC)6	(AC)6	(AC)6	(AC)7	(AC)6	-	Introns/Intergenic	-	-	GAAAAATCCAAACATGTCCTAATTC	CGAAAGGTGAAAAGGAGAAGC	256	A	A	A	NA
PMS5227	Os_Chr10	18575740	18575757	(CGC)6	(CGC)6	(CGC)6	(CGC)5	(CGC)6	(CGC)6	(CGC)6	(CGC)6	(CGC)6	(CGC)6	(CGC)5	LOC_Os10g34790	CDS	terpene synthase, putative, expressed	CODON_DELETION	CTTCTCTCATCAGCCGGAAC	GCGGCGTCAGAGGAGTAGTA	203	P	A	A	NA
PMS5228	Os_Chr10	18580697	18580710	(GA)7	(GA)7	(GA)7	(GA)7	(GA)7	(GA)7	(GA)7	(GA)7	(GA)7	(GA)7	(GA)6	LOC_Os10g34795	URR	expressed protein	-	GATGGGAGATCGGGGAAG	TTTCTCAGCCTCTCCTTTTCC	151	A	A	A	NA
PMS5229	Os_Chr10	18595509	18595522	(GA)7	(GA)7	(GA)7	(GA)7	(GA)7	(GA)7	(GA)7	(GA)7	(GA)7	(GA)6	(GA)7	LOC_Os10g34820	DRR	CDT1B - Putative DNA replication initiation protein, expressed	-	CATGAGCGAAGTGAGGGAAT	GAGATGTTCAAAGGGTGAAGC	171	A	A	A	NA
PMS5230	Os_Chr10	18600640	18600653	(TC)7	(TC)6	(TC)7	(TC)7	(TC)7	(TC)6	(TC)7	(TC)7	(TC)7	(TC)7	(TC)7	-	Introns/Intergenic	-	-	AACAAGTTCCGTTTCCTCCA	TACGTAGGAGATTCCGCACC	189	P	A	A	NA
PMS5231	Os_Chr10	18635301	18635370	(TA)35	(TA)35	(TA)35	(TA)35	(TA)35	(TA)35	(TA)7	(TA)35	(TA)35	(TA)8	(TA)8	LOC_Os10g34910	DRR	secretory protein, putative, expressed	-	GGTCAAGGCTCAGCCATACTTGG	AAGTTGAAGGACGGGTACAATGTCG	386	A	A	A	RM25628
PMS5232	Os_Chr10	18727055	18727114	(TA)30	(TA)30	(TA)10	(TA)11	(TA)10	(TA)11	(TA)6	(TA)10	(TA)9	(TA)30	(TA)22	-	Introns/Intergenic	-	-	TCAAACCGGCACATATAAGACC	CGTTGTAGTGTCAGCAAGATAGGG	182	P	A	A	RM304
PMS5233	Os_Chr10	18785229	18785252	(CGG)8	(CGG)8	(CGG)8	(CGG)8	(CGG)8	(CGG)8	(CGG)8	(CGG)8	(CGG)8	(CGG)8	(CGG)5	LOC_Os10g35180	CDS	white-brown complex homolog protein 11, putative, expressed	CODON_CHANGE_PLUS_CODON_DELETION	GACCTCACCGTCACCGTC	AGGAGAGGTTGGCCTTGC	235	A	A	A	NA
PMS5234	Os_Chr10	18809916	18809937	(CT)11	(CT)11	(CT)11	(CT)11	(CT)11	(CT)11	(CT)11	(CT)11	(CT)11	(CT)11	(CT)12	-	Introns/Intergenic	-	-	CCCAGTTCTCCGAGTAGCAA	TGCTATCGTCAGCTCCCTTT	276	A	A	A	NA
PMS5235	Os_Chr10	18826203	18826214	(GA)6	(GA)6	(GA)6	(GA)6	(GA)7	(GA)6	(GA)6	(GA)6	(GA)6	(GA)7	(GA)7	-	Introns/Intergenic	-	-	TCTTCATCATCCCCTCCTCC	ACGATTTTGTCCTACGTGGC	277	P	A	A	NA
PMS5236	Os_Chr10	18848497	18848517	(GCC)7	(GCC)7	(GCC)7	(GCC)7	(GCC)7	(GCC)7	(GCC)7	(GCC)7	(GCC)7	(GCC)7	(GCC)6	LOC_Os10g35260	CDS	Rf1, mitochondrial precursor, putative, expressed	CODON_DELETION	GCGGTTGAAGCTGTAGATCG	TCTCCATTTCTTCAGCTCCG	239	A	A	A	NA
PMS5237	Os_Chr10	18877064	18877085	(TA)11	(TA)11	(TA)11	(TA)11	(TA)11	(TA)11	(TA)6	(TA)11	(TA)11	(TA)11	(TA)11	-	Introns/Intergenic	-	-	CGAGGATAGAGGAACAGGCA	TAGTAGCTACCCGCCGATCA	248	A	A	A	NA
PMS5238	Os_Chr10	18903596	18903610	(TCT)5	(TCT)6	(TCT)6	(TCT)6	(TCT)6	(TCT)6	(TCT)5	(TCT)6	(TCT)5	(TCT)6	(TCT)6	LOC_Os10g35370	DRR	OsPORB|FGL	-	GGAGAAGGCCAAGAAGGTCT	TGAGAAACGCGTCCTACAAA	179	A	A	P	NA

Polymorphic SSR (PMS) markers	Chromosomes	Start physical positions (bp)	End physical positions (bp)	Nipponbare	Kasalath	Nagina22	IR64	Pokkali	Bala	Tainung67	Azucena	Moroberekan	*Oryza rufipogon*	*Oryza nivara*	MSU gene locus IDs	Structural annotation	Functional annotation	SSR functional effects	Forward primers (5'-3')	Reverse primers (5'-3')	Amplified product size (bp)	Markers exhibiting polymorphism within *indica* rice	Transcrption factor genes-derived markers	Known cloned genes-derived markers	PMS markers corresponding with RM markers
PMS5239	Os_Chr10	18959447	18959468	(AT)11	(AT)11	(AT)11	(AT)11	(AT)11	(AT)7	(AT)11	(AT)11	(AT)11	(AT)11	(AT)11	-	Introns/Intergenic	-	-	ACTTGAGACGATCAGACGCC	AACTCGCCCTCCTGCTAACT	235	P	A	A	NA
PMS5240	Os_Chr10	19002046	19002119	(TA)37	(TA)37	(TA)37	(TA)37	(TA)13	(TA)37	(TA)37	(TA)37	(TA)37	(TA)37	(TA)8	-	Introns/Intergenic	-	-	AAGGGTGAGGTCAAACTTTAGAA	GATGCGTCATCTCTACGGGT	268	P	A	A	NA
PMS5241	Os_Chr10	19049452	19049463	(CG)6	(CG)7	(CG)6	(CG)6	(CG)6	(CG)6	(CG)6	(CG)7	(CG)6	(CG)7	(CG)6	-	Introns/Intergenic	-	-	GGAAGGAGTTTCCTCTTCGG	CCTTGCTCTTATGAGACCGC	239	P	A	A	NA
PMS5242	Os_Chr10	19053265	19053288	(TA)12	(TA)12	(TA)12	(TA)12	(TA)12	(TA)12	(TA)7	(TA)12	(TA)12	(TA)12	(TA)11	-	Introns/Intergenic	-	-	CCCATCCCATTTATTCCAAA	GCCCTCCACTTCGAGATATG	219	A	A	A	NA
PMS5243	Os_Chr10	19066826	19066843	(CCG)6	(CCG)6	(CCG)6	(CCG)5	(CCG)6	(CCG)6	(CCG)6	(CCG)6	(CCG)6	(CCG)6	(CCG)6	LOC_Os10g35650	URR	expressed protein	-	AATTGCCATCCAAATTGCTC	AGGAAGGGAGGAGCTAACCA	158	P	A	A	NA
PMS5244	Os_Chr10	19135776	19135791	(AT)8	(AT)8	(AT)8	(AT)8	(AT)8	(AT)8	(AT)8	(AT)8	(AT)7	(AT)8	(AT)8	-	Introns/Intergenic	-	-	AAAACTGAGGGTGTGTTCGG	TGACTTACCTTATGAAACGGAGAA	184	A	A	A	NA
PMS5245	Os_Chr10	19170916	19170927	(AG)6	(AG)6	(AG)6	(AG)6	(AG)6	(AG)7	(AG)6	(AG)6	(AG)6	(AG)6	(AG)6	-	Introns/Intergenic	-	-	AGTCGGTCTCCTTGCCATC	AGCCACATCATCACCCTCTC	113	P	A	A	NA
PMS5246	Os_Chr10	19194303	19194323	(GCG)7	(GCG)7	(GCG)7	(GCG)7	(GCG)7	(GCG)7	(GCG)7	(GCG)5	(GCG)7	(GCG)7	(GCG)7	LOC_Os10g35920	URR	OsFBX389 - F-box domain containing protein, expressed	-	GATCTCTCCGGGAGGAAGAGTGC	CTCCTCGTCTCGTCTCGTCTCG	241	A	A	A	RM25668
PMS5247	Os_Chr10	19203052	19203087	(CA)18	(CA)16	(CA)18	(CA)18	(CA)18	(CA)18	(CA)18	(CA)18	(CA)18	(CA)18	(CA)18	-	Introns/Intergenic	-	-	TGCAAGGGAAATGAATTTGA	TGGCACCAATTCTAGGAAGG	259	P	A	A	NA
PMS5248	Os_Chr10	19260722	19260735	(CG)7	(CG)7	(CG)7	(CG)6	(CG)7	(CG)7	(CG)7	(CG)6	(CG)7	(CG)7	(CG)6	-	Introns/Intergenic	-	-	TCGAGTTCGAGGAAACCTTC	TACAGTTGTGCACGCTCGTT	266	P	A	A	NA
PMS5249	Os_Chr10	19335774	19335793	(AT)10	(AT)7	(AT)10	(AT)10	(AT)10	(AT)7	(AT)10	(AT)7	(AT)10	(AT)7	(AT)10	-	Introns/Intergenic	-	-	TGGGTATTTATAGACAGGAGGTGTCG	ACCCAGACATCACCACTAACATCG	206	P	A	A	RM25677
PMS5250	Os_Chr10	19416187	19416402	(AAT)72	(AAT)72	(AAT)5	(AAT)24	(AAT)18	(AAT)72	(AAT)72	(AAT)16	(AAT)11	(AAT)12	(AAT)72	-	Introns/Intergenic	-	-	CGTAAGCAAAGTGCATAACGA	TTCTCTAGGTTATCCATAGACGACAA	280	P	A	A	NA
PMS5251	Os_Chr10	19466825	19466856	(TA)16	(TA)16	(TA)6	(TA)6	(TA)6	(TA)16	(TA)16	(TA)6	(TA)16	(TA)12	(TA)16	-	Introns/Intergenic	-	-	AAGTGGGATGCATACGGTTTAGGG	CTCGCGATCTAGACTCCCATCG	278	P	A	A	RM25684

Polymorphic SSR (PMS) markers	Chromosomes	Start physical positions (bp)	End physical positions (bp)	Nipponbare	Kasalath	Nagina22	IR64	Pokkali	Bala	Tainung67	Azucena	Moroberekan	*Oryza rufipogon*	*Oryza nivara*	MSU gene locus IDs	Structural annotation	Functional annotation	SSR functional effects	Forward primers (5'-3')	Reverse primers (5'-3')	Amplified product size (bp)	Markers exhibiting polymorphism within *indica* rice	Transcrption factor genes-derived markers	Known cloned genes-derived markers	PMS markers corresponding with RM markers
PMS5252	Os_Chr10	19473010	19473057	(AG)24	(AG)24	(AG)24	(AG)24	(AG)24	(AG)15	(AG)19	(AG)24	(AG)24	(AG)24	(AG)24	-	Introns/Intergenic	-	-	GGAGAGAAACATGGCAACACTGC	GCCAAGAACGTCAGTACTCTCTCG	169	P	A	A	RM25685
PMS5253	Os_Chr10	19483190	19483223	(AG)17	(AG)19	(AG)19	(AG)17	(AG)17	(AG)19	(AG)17	(AG)14	(AG)17	(AG)15	(AG)17	-	Introns/Intergenic	-	-	TATCGGATCTCTAACTCGTTGC	AATACACCAGTCTCTCACTCTCACC	226	P	A	A	RM25689
PMS5254	Os_Chr10	19483425	19483446	(CT)11	(CT)11	(CT)11	(CT)11	(CT)11	(CT)11	(CT)10	(CT)11	(CT)11	(CT)9	(CT)11	-	Introns/Intergenic	-	-	TTCACTCTCTCTCCATGCCC	TGCGAAGCTGTATTCGTAGG	255	A	A	A	NA
PMS5255	Os_Chr10	19525312	19525329	(GGC)6	(GGC)6	(GGC)6	(GGC)6	(GGC)5	(GGC)6	(GGC)6	(GGC)6	(GGC)6	(GGC)6	(GGC)6	LOC_Os10g36490	URR	HEAT repeat family protein, putative, expressed	-	GATCTCCCACTCCTGGATGA	GTCTCCCGCACACACTCTCT	240	P	A	A	NA
PMS5256	Os_Chr10	19560808	19560828	(GCC)7	(GCC)7	(GCC)7	(GCC)7	(GCC)7	(GCC)7	(GCC)7	(GCC)7	(GCC)7	(GCC)5	(GCC)7	-	Introns/Intergenic	-	-	TTGATGCTGCCCTTCTTTTT	CTGCATCCACTACCAACCCT	161	A	A	A	NA
PMS5257	Os_Chr10	19561455	19561468	(AT)7	(AT)9	(AT)7	(AT)7	(AT)7	(AT)7	(AT)7	(AT)7	(AT)7	(AT)7	(AT)7	-	Introns/Intergenic	-	-	GCCGATTGCTGACTAGGATT	TTTTGCCCGGTTTTACTGAC	120	P	A	A	NA
PMS5258	Os_Chr10	19581251	19581276	(CT)13	(CT)12	(CT)13	(CT)13	(CT)13	(CT)13	(CT)13	(CT)13	(CT)13	(CT)13	(CT)13	-	Introns/Intergenic	-	-	CCTCCACACGGACTGAAAAT	GCGATAAGTGAGAGAGCCGA	232	P	A	A	NA
PMS5259	Os_Chr10	19583275	19583298	(CGG)8	(CGG)7	(CGG)8	(CGG)6	(CGG)6	(CGG)7	(CGG)8	(CGG)8	(CGG)8	(CGG)8	(CGG)8	-	Introns/Intergenic	-	-	CCATCGGAACAAAGAAGGAA	CAGGCCGTCGAGTACCAG	227	P	A	A	NA
PMS5260	Os_Chr10	19688233	19688256	(TC)12	(TC)11	(TC)12	(TC)13	(TC)12	(TC)11	(TC)12	(TC)12	(TC)12	(TC)12	(TC)12	-	Introns/Intergenic	-	-	TCTCCCTATTCCCGTGTAAATCG	CCCGATGATCGATTGTACCTAGC	184	P	A	A	RM1146
PMS5261	Os_Chr10	19703969	19703983	(TCC)5	(TCC)5	(TCC)5	(TCC)5	(TCC)5	(TCC)5	(TCC)5	(TCC)5	(TCC)5	(TCC)6	(TCC)5	LOC_Os10g36810	CDS	plus-3 domain containing protein, putative, expressed	CODON_CHANGE_PLUS_CODON_INSERTION	TAGGGTTTTGGGTTGTCGAG	GAGCTGGGGAGTGGATGG	199	A	A	A	NA
PMS5262	Os_Chr10	19713950	19713963	(TA)7	(TA)7	(TA)7	(TA)7	(TA)7	(TA)7	(TA)7	(TA)7	(TA)7	(TA)7	(TA)8	-	Introns/Intergenic	-	-	GGAACTAATCCTCCACGCAA	TTTGTTTGGGTTGAAAGCAC	228	A	A	A	NA
PMS5263	Os_Chr10	19734003	19734020	(CGG)6	(CGG)8	(CGG)6	(CGG)6	(CGG)6	(CGG)8	(CGG)6	(CGG)6	(CGG)6	(CGG)6	(CGG)6	LOC_Os10g36848	CDS	cytochrome P450, putative, expressed	CODON_INSERTION	ACGTACCATGATGATGGCCT	CGACAGGTTCATCGACAAGA	226	P	A	A	NA
PMS5264	Os_Chr10	19825436	19825453	(CCT)6	(CCT)5	(CCT)6	(CCT)6	(CCT)6	(CCT)6	(CCT)6	(CCT)6	(CCT)6	(CCT)6	(CCT)6	-	Introns/Intergenic	-	-	CTGCGCTCGCCTCCTCCTCT	CTCACTCTCTTCTCACTCACG	311	P	A	A	NA

Polymorphic SSR (PMS) markers	Chromosomes	Start physical positions (bp)	End physical positions (bp)	Nipponbare	Kasalath	Nagina22	IR64	Pokkali	Bala	Tainung67	Azucena	Moroberekan	*Oryza rufipogon*	*Oryza nivara*	MSU gene locus IDs	Structural annotation	Functional annotation	SSR functional effects	Forward primers (5'-3')	Reverse primers (5'-3')	Amplified product size (bp)	Markers exhibiting polymorphism within *indica* rice	Transcrption factor genes-derived markers	Known cloned genes-derived markers	PMS markers corresponding with RM markers
PMS5265	Os_Chr10	19859847	19859867	(CGG)7	(CGG)7	(CGG)6	(CGG)7	(CGG)7	(CGG)7	(CGG)7	(CGG)7	(CGG)7	(CGG)7	(CGG)7	LOC_Os10g37110	CDS	transposon protein, putative, unclassified, expressed	CODON_DELETION	GACGACGAGATGGTCAACCT	CATGTCGTGGATGTCCTCCT	201	P	A	A	NA
PMS5266	Os_Chr10	19933859	19933896	(AG)19	(AG)18	(AG)19	(AG)19	(AG)19	(AG)18	(AG)19	(AG)19	(AG)19	(AG)19	(AG)19	-	Introns/Intergenic	-	-	GAGGGAGATGATGTGGGAGA	CCCCTCCTTTCTTCTTCACC	181	P	A	A	NA
PMS5267	Os_Chr10	19937529	19937546	(AT)9	(AT)9	(AT)9	(AT)6	(AT)7	(AT)6	(AT)9	(AT)8	(AT)9	(AT)9	(AT)9	-	Introns/Intergenic	-	-	GGGCACCATAGAGTCCCATA	GTGCCCCTCTTTTCTTTCAA	243	P	A	A	NA
PMS5268	Os_Chr10	19979449	19979466	(CT)9	(CT)9	(CT)9	(CT)9	(CT)9	(CT)9	(CT)8	(CT)9	(CT)9	(CT)9	(CT)9	-	Introns/Intergenic	-	-	CATCTTGTGATGTGGTAGACTGG	CAGTTTCAGCGGTAGCAACA	184	A	A	A	NA
PMS5269	Os_Chr10	20023246	20023263	(CCG)6	(CCG)8	(CCG)6	(CCG)8	(CCG)6	(CCG)8	(CCG)6	(CCG)8	(CCG)6	(CCG)6	(CCG)6	-	Introns/Intergenic	-	-	GCTCCATCGACTGCTTGAA	AGCTCAACGACCACTCACCT	280	P	A	A	NA
PMS5270	Os_Chr10	20060981	20060998	(CT)9	(CT)9	(CT)9	(CT)9	(CT)9	(CT)9	(CT)8	(CT)9	(CT)9	(CT)9	(CT)9	LOC_Os10g37480	URR	STE_PAK_Ste20_STLK.6 - STE kinases include homologs to sterile 7, sterile 11 and sterile 20 from yeast, expressed	-	CGACCTCGTTTCTTAAAGCG	CCGGAGCTTCTACCTCCTCT	201	A	A	A	NA
PMS5271	Os_Chr10	20083744	20083759	(GA)8	(GA)9	(GA)8	(GA)8	(GA)8	(GA)9	(GA)8	(GA)8	(GA)8	(GA)8	(GA)8	-	Introns/Intergenic	-	-	GTTGTGCGTTTTGATTCGGT	AGAAATCCTTTTGTCCGGCT	240	P	A	A	NA
PMS5272	Os_Chr10	20126233	20126250	(TC)9	(TC)9	(TC)9	(TC)6	(TC)9	(TC)9	(TC)9	(TC)9	(TC)9	(TC)9	(TC)9	-	Introns/Intergenic	-	-	TCTCTCCTCTGCCTCAGCTC	GTAACACTACGCCACTGCGA	110	P	A	A	NA
PMS5273	Os_Chr10	20134110	20134139	(GT)15	(GT)6	(GT)15	(GT)15	(GT)15	(GT)6	(GT)15	(GT)15	(GT)15	(GT)15	(GT)15	-	Introns/Intergenic	-	-	GCACTGTCATGACCGACAATGG	GCCCAAACGATTTAAGGCAAGC	142	P	A	A	RM25737
PMS5274	Os_Chr10	20139621	20139641	(CGG)7	(CGG)5	(CGG)7	(CGG)7	(CGG)5	(CGG)7	(CGG)7	(CGG)7	(CGG)7	(CGG)7	(CGG)7	LOC_Os10g37630	URR	UBX domain-containing protein, putative, expressed	-	AGCTAGGGTTTGGGGATTTG	ACGCGTATATTTTCCGTTGC	206	P	A	A	NA
PMS5275	Os_Chr10	20144688	20144711	(GGA)8	(GGA)8	(GGA)8	(GGA)8	(GGA)8	(GGA)8	(GGA)6	(GGA)8	(GGA)8	(GGA)8	(GGA)8	LOC_Os10g37640	CDS	HIT zinc finger domain containing protein, expressed	CODON_DELETION	AGCTCACACGATCACGATTAGGC	GGAGAGGGAAGTCGTGGTCTCC	174	A	A	A	RM25740
PMS5276	Os_Chr10	20190616	20190629	(TA)7	(TA)9	(TA)7	(TA)7	(TA)7	(TA)7	(TA)7	(TA)7	(TA)7	(TA)7	(TA)7	-	Introns/Intergenic	-	-	GGCACTCCATTTTCAAGGAA	TCCATCCACAAATACTCGCTC	254	P	A	A	NA
PMS5277	Os_Chr10	20201145	20201168	(CTC)8	(CTC)8	(CTC)8	(CTC)8	(CTC)8	(CTC)5	(CTC)7	(CTC)8	(CTC)8	(CTC)6	(CTC)8	LOC_Os10g37740	URR	CGMC_GSK.9 - CGMC includes CDA, MAPK, GSK3, and CLKC kinases, expressed	-	GCATCATATGCATCCTCCTTCTCG	CGAGAAATCTAGTGGTGCAGCAAGC	370	P	A	A	RM25743

Polymorphic SSR (PMS) markers	Chromosomes	Start physical positions (bp)	End physical positions (bp)	Nipponbare	Kasalath	Nagina22	IR64	Pokkali	Bala	Tainung67	Azucena	Moroberekan	*Oryza rufipogon*	*Oryza nivara*	MSU gene locus IDs	Structural annotation	Functional annotation	SSR functional effects	Forward primers (5'-3')	Reverse primers (5'-3')	Amplified product size (bp)	Markers exhibiting polymorphism within *indica* rice	Transcrption factor genes-derived markers	Known cloned genes-derived markers	PMS markers corresponding with RM markers
PMS5278	Os_Chr10	20263492	20263512	(GCA)7	(GCA)7	(GCA)7	(GCA)7	(GCA)7	(GCA)8	(GCA)7	(GCA)7	(GCA)7	(GCA)7	(GCA)7	-	Introns/Intergenic	-	-	TGGTGATTAGCGTCTCGTTG	GGGAAGAAAAGGCAAGGTCT	185	P	A	A	NA
PMS5279	Os_Chr10	20295405	20295422	(CGG)6	(CGG)6	(CGG)6	(CGG)6	(CGG)6	(CGG)6	(CGG)6	(CGG)5	(CGG)6	(CGG)6	(CGG)6	LOC_Os10g37899	CDS	transposon protein, putative, unclassified, expressed	CODON_CHANGE_PLUS_CODON_DELETION	ACCCTAGATGGGCCAAGTTT	GGATTGGGTACCTTTCGGAT	237	A	A	A	NA
PMS5280	Os_Chr10	20307055	20307069	(CGC)5	(CGC)5	(CGC)5	(CGC)5	(CGC)6	(CGC)5	(CGC)5	(CGC)5	(CGC)5	(CGC)5	(CGC)5	-	Introns/Intergenic	-	-	TCCTTCCATCCAGACACCTC	TAGGGTCATTCGATGCAACA	224	P	A	A	NA
PMS5281	Os_Chr10	20345875	20345892	(CCG)6	(CCG)6	(CCG)6	(CCG)6	(CCG)6	(CCG)6	(CCG)5	(CCG)6	(CCG)6	(CCG)6	(CCG)6	LOC_Os10g37980	CDS	prephenate dehydratase domain containing protein, expressed	CODON_DELETION	CTCTCGATCTCCTTGATGGC	CTGACCAAGCTGGAGGTCAT	200	A	A	A	NA
PMS5282	Os_Chr10	20422643	20422654	(TC)6	(TC)6	(TC)6	(TC)6	(TC)7	(TC)6	(TC)6	(TC)6	(TC)6	(TC)6	(TC)6	-	Introns/Intergenic	-	-	TCTCTCGCTCTCTCGTCCTC	CACAGCCGAAAATCGAATG	124	P	A	A	NA
PMS5283	Os_Chr10	20443802	20443819	(AG)9	(AG)9	(AG)9	(AG)9	(AG)9	(AG)9	(AG)9	(AG)7	(AG)9	(AG)9	(AG)9	-	Introns/Intergenic	-	-	GCAGAGTGGAGAAGAGGGAG	ATCCCGAATCCAAATCCTTC	152	A	A	A	NA
PMS5284	Os_Chr10	20529750	20529765	(TC)8	(TC)8	(TC)8	(TC)8	(TC)8	(TC)8	(TC)8	(TC)7	(TC)8	(TC)8	(TC)8	-	Introns/Intergenic	-	-	CTCAGGTTCTGTGGACCGAT	ATAGCGGAGAGGGAGGGAG	144	A	A	A	NA
PMS5285	Os_Chr10	20536396	20536421	(TA)13	(TA)13	(TA)13	(TA)13	(TA)13	(TA)13	(TA)6	(TA)13	(TA)13	(TA)13	(TA)13	-	Introns/Intergenic	-	-	CCGTGACTTGCTGAGTACGA	AACACCAACAACAGCAGCAG	246	A	A	A	NA
PMS5286	Os_Chr10	20696204	20696224	(GGT)7	(GGT)7	(GGT)7	(GGT)7	(GGT)7	(GGT)7	(GGT)7	(GGT)7	(GGT)7	(GGT)7	(GGT)6	LOC_Os10g38860	URR	hydrolase, alpha/beta fold family domain containing protein, expressed	-	AATAGCACCTCCACCACGTC	CCTCGCAGCTGCTTCTACTT	212	A	A	A	NA
PMS5287	Os_Chr10	20755597	20755638	(AT)21	(AT)21	(AT)7	(AT)21	(AT)21	(AT)10	(AT)21	(AT)13	(AT)7	(AT)21	(AT)21	-	Introns/Intergenic	-	-	TAGGCCGGGATGTTACAGTT	CATCGACCTGGTGGAGATTT	185	P	A	A	NA
PMS5288	Os_Chr10	20792287	20792307	(GGC)7	(GGC)7	(GGC)7	(GGC)7	(GGC)7	(GGC)7	(GGC)6	(GGC)7	(GGC)7	(GGC)7	(GGC)7	LOC_Os10g39010	DRR	TKL_IRAK_CrRLK1L-1.16 - The CrRLK1L-1 subfamily has homology to the CrRLK1L homolog, expressed	-	GTTGGAGGCGGTATCTGAAG	TGGCCCATAAAAACTTGAGG	248	A	A	A	NA
PMS5289	Os_Chr10	20878262	20878275	(AG)7	(AG)7	(AG)7	(AG)7	(AG)7	(AG)7	(AG)7	(AG)7	(AG)7	(AG)6	(AG)7	-	Introns/Intergenic	-	-	GAGGGAGCCTTTGTGCCT	CAGGCGGAACCTTATGTGAT	175	A	A	A	NA
PMS5290	Os_Chr10	20922967	20922998	(TA)16	(TA)16	(TA)8	(TA)16	(TA)16	(TA)16	(TA)16	(TA)7	(TA)16	(TA)16	(TA)16	-	Introns/Intergenic	-	-	GGCGCCGTGAATTAAAAA	CACGTGAGGGAAAAATGTCA	223	P	A	A	NA

Polymorphic SSR (PMS) markers	Chromosomes	Start physical positions (bp)	End physical positions (bp)	Nipponbare	Kasalath	Nagina22	IR64	Pokkali	Bala	Tainung67	Azucena	Moroberekan	*Oryza rufipogon*	*Oryza nivara*	MSU gene locus IDs	Structural annotation	Functional annotation	SSR functional effects	Forward primers (5'-3')	Reverse primers (5'-3')	Amplified product size (bp)	Markers exhibiting polymorphism within *indica* rice	Transcrption factor genes-derived markers	Known cloned genes-derived markers	PMS markers corresponding with RM markers
PMS5291	Os_Chr10	20949985	20950002	(TC)9	(TC)9	(TC)9	(TC)9	(TC)9	(TC)9	(TC)9	(TC)9	(TC)9	(TC)9	(TC)7	-	Introns/Intergenic	-	-	CAAATCCTTCCAAATTAATCC	GTGCTCGCCAGGGATGGAG	200	A	A	A	NA
PMS5292	Os_Chr10	20999920	20999939	(TC)10	(TC)10	(TC)10	(TC)10	(TC)10	(TC)10	(TC)10	(TC)10	(TC)10	(TC)10	(TC)12	-	Introns/Intergenic	-	-	TTGCTTGTTGTCAGTCTCCG	AGAGAGAACGAGAGAGGGGG	276	A	A	A	NA
PMS5293	Os_Chr10	21043776	21043813	(TA)19	(TA)19	(TA)10	(TA)19	(TA)8	(TA)19	(TA)19	(TA)19	(TA)19	(TA)19	(TA)19	-	Introns/Intergenic	-	-	AATCCCTCAAACGCACACAC	GCAAAAGCAGTCCACCTCTC	239	P	A	A	NA
PMS5294	Os_Chr10	21055502	21055522	(GGA)7	(GGA)7	(GGA)7	(GGA)7	(GGA)7	(GGA)7	(GGA)7	(GGA)7	(GGA)7	(GGA)6	(GGA)6	LOC_Os10g39450	CDS	RING-H2 finger protein, putative, expressed	CODON_DELETION	GTGGAAAGGAGCAGCTCAAC	AAGTGCGAGGCCATAGTCC	196	A	A	A	NA
PMS5295	Os_Chr10	21151171	21151192	(TA)11	(TA)11	(TA)8	(TA)11	(TA)11	(TA)11	(TA)11	(TA)11	(TA)11	(TA)11	(TA)11	-	Introns/Intergenic	-	-	CCGTGCATCTAGGAATGGAG	GTGGACGGTTAGCTAGGCAG	266	P	A	A	NA
PMS5296	Os_Chr10	21219591	21219611	(GAC)7	(GAC)7	(GAC)7	(GAC)7	(GAC)7	(GAC)7	(GAC)6	(GAC)7	(GAC)7	(GAC)7	(GAC)7	LOC_Os10g39700	CDS	CHIT15 - Chitinase family protein precursor, putative, expressed	CODON_DELETION	GACAACTCATACGACAGCGATCC	CAAGCTGCAAGCTGAACAAACC	319	A	A	A	RM25802
PMS5297	Os_Chr10	21271770	21271787	(TTG)6	(TTG)5	(TTG)5	(TTG)6	(TTG)6	(TTG)5	(TTG)6	(TTG)6	(TTG)6	(TTG)6	(TTG)5	LOC_Os10g39770	URR	zinc finger, C3HC4 type, domain containing protein, expressed	-	CTCATTCAGGCCAGTGGATT	CGTCCATTGGTTGCAGACT	247	P	A	A	NA
PMS5298	Os_Chr10	21381544	21381558	(CGC)5	(CGC)6	(CGC)5	(CGC)5	(CGC)5	(CGC)6	(CGC)6	(CGC)6	(CGC)5	(CGC)6	(CGC)6	-	Introns/Intergenic	-	-	GATGTCACCCCCACACCTTA	AATCAACCAAACCCTAGCCC	198	P	A	A	NA
PMS5299	Os_Chr10	21547967	21548042	(TA)38	(TA)38	(TA)38	(TA)38	(TA)8	(TA)38	(TA)6	(TA)38	(TA)38	(TA)38	(TA)13	-	Introns/Intergenic	-	-	TGCAAATGATGTGAGTGTGG	ATCTCAACATGCAACCATGC	210	P	A	A	NA
PMS5300	Os_Chr10	21592669	21592690	(GA)11	(GA)11	(GA)11	(GA)11	(GA)11	(GA)11	(GA)11	(GA)11	(GA)11	(GA)11	(GA)7	-	Introns/Intergenic	-	-	GCGGGATCATACAAAGGCTA	CCTAGGGTTTCGTGGGTTG	221	A	A	A	NA
PMS5301	Os_Chr10	21599059	21599076	(AGA)6	(AGA)6	(AGA)6	(AGA)7	(AGA)6	(AGA)6	(AGA)6	(AGA)6	(AGA)6	(AGA)6	(AGA)7	LOC_Os10g40290	CDS	pectinacetylesterase domain containing protein, expressed	CODON_INSERTION	GGAATTTTCCAATTGCATGG	CAGGCGAGGAATTCTTCTTG	176	P	A	A	NA
PMS5302	Os_Chr10	21623166	21623177	(GA)6	(GA)6	(GA)6	(GA)6	(GA)6	(GA)6	(GA)6	(GA)6	(GA)6	(GA)7	(GA)6	-	Introns/Intergenic	-	-	CGGAGCGAAAAATGAGAGAG	ACCCGGTTTTATCTGACGTG	116	A	A	A	NA
PMS5303	Os_Chr10	21665808	21665857	(AT)25	(AT)23	(AT)10	(AT)25	(AT)11	(AT)25	(AT)25	(AT)25	(AT)8	(AT)25	(AT)25	-	Introns/Intergenic	-	-	GATGGGAATTGAAGCTGGTATCC	TCAGACCAGTCGGCAATTAGTCC	171	P	A	A	RM25818

Polymorphic SSR (PMS) markers	Chromosomes	Start physical positions (bp)	End physical positions (bp)	Nipponbare	Kasalath	Nagina22	IR64	Pokkali	Bala	Tainung67	Azucena	Moroberekan	*Oryza rufipogon*	*Oryza nivara*	MSU gene locus IDs	Structural annotation	Functional annotation	SSR functional effects	Forward primers (5'-3')	Reverse primers (5'-3')	Amplified product size (bp)	Markers exhibiting polymorphism within *indica* rice	Transcrption factor genes-derived markers	Known cloned genes-derived markers	PMS markers corresponding with RM markers
PMS5304	Os_Chr10	21714946	21714973	(TC)14	(TC)11	(TC)14	(TC)14	(TC)14	(TC)14	(TC)14	(TC)14	(TC)14	(TC)14	(TC)14	-	Introns/Intergenic	-	-	TTGTCGGTGCAATTTAGCTG	CCTTCCTACATCCGGTCAAA	276	P	A	A	NA
PMS5305	Os_Chr10	21798744	21798763	(TC)10	(TC)10	(TC)10	(TC)10	(TC)10	(TC)10	(TC)10	(TC)10	(TC)10	(TC)10	(TC)9	-	Introns/Intergenic	-	-	ACTTCGCCTTCGTCTTTCCT	GCGGCGTTTCCCTTATTTAT	236	A	A	A	NA
PMS5306	Os_Chr10	21810352	21810372	(CCA)7	(CCA)7	(CCA)7	(CCA)7	(CCA)7	(CCA)7	(CCA)5	(CCA)7	(CCA)7	(CCA)7	(CCA)7	LOC_Os10g40660	CDS	ZOS10-07 - C2H2 zinc finger protein, expressed	CODON_DELETION	GACAGCATCAAGAGCGACATGG	CCAAGAGGAGCTACAAGGTGAGC	268	A	A	A	RM25833
PMS5307	Os_Chr10	21978640	21978657	(TA)9	(TA)9	(TA)8	(TA)9	(TA)9	(TA)9	(TA)9	(TA)9	(TA)9	(TA)7	(TA)9	-	Introns/Intergenic	-	-	ACCAGTCAGGTCAGTGGGAG	CGGGGCTAAGTTCCCTATTT	236	P	A	A	NA
PMS5308	Os_Chr10	22042969	22042989	(CGG)7	(CGG)7	(CGG)7	(CGG)7	(CGG)6	(CGG)7	(CGG)7	(CGG)7	(CGG)7	(CGG)7	(CGG)7	LOC_Os10g41040	CDS	ternary complex factor MIP1, putative, expressed	CODON_DELETION	CTCACATCATTCTCCAGCGA	CTTGAGCCAGTGCTTCTTCC	250	P	A	A	NA
PMS5309	Os_Chr10	22099438	22099455	(TA)9	(TA)9	(TA)9	(TA)8	(TA)7	(TA)9	(TA)8	(TA)9	(TA)9	(TA)9	(TA)9	-	Introns/Intergenic	-	-	GGGAACTCAGTACAGAACATGGA	TTTCTTTCCTCCTTTTCCCTTT	236	P	A	A	NA
PMS5310	Os_Chr10	22193151	22193171	(GGC)7	(GGC)7	(GGC)7	(GGC)7	(GGC)6	(GGC)7	(GGC)7	(GGC)7	(GGC)7	(GGC)7	(GGC)7	LOC_Os10g41300	CDS	expressed protein	CODON_DELETION	TCAAAATGTGAATGCCCAGA	TGGATCTCGTCTCCAACCTC	270	P	P	A	NA
PMS5311	Os_Chr10	22194062	22194082	(CCA)7	(CCA)7	(CCA)7	(CCA)6	(CCA)6	(CCA)7	(CCA)7	(CCA)7	(CCA)7	(CCA)6	(CCA)6	LOC_Os10g41300	URR	expressed protein	-	AAGATTCCTGGGGCTTGTCT	TGACCATCTCCATCTCCCTC	244	P	P	A	NA
PMS5312	Os_Chr10	22209580	22209601	(TA)11	(TA)11	(TA)11	(TA)6	(TA)11	(TA)11	(TA)6	(TA)11	(TA)11	(TA)11	(TA)11	-	Introns/Intergenic	-	-	CATGTGAGAAAAAGTCAACGG	GGTCCAACGTAAACATCCCA	259	P	A	A	NA
PMS5313	Os_Chr10	22261007	22261030	(AT)12	(AT)12	(AT)12	(AT)12	(AT)12	(AT)12	(AT)9	(AT)12	(AT)12	(AT)12	(AT)12	-	Introns/Intergenic	-	-	GTCGGATTATTGAAACGGATGG	CTCTTTGTGCCGGTACACTTGC	270	A	A	A	RM25862
PMS5314	Os_Chr10	22285411	22285428	(CAC)6	(CAC)6	(CAC)6	(CAC)5	(CAC)5	(CAC)6	(CAC)6	(CAC)6	(CAC)6	(CAC)5	(CAC)5	LOC_Os10g41460	CDS	transcription factor like protein, putative, expressed	CODON_DELETION	AGTCTGAGATGAAGCGCACC	ATCACCCTCCCAAACACAAA	278	P	A	A	NA
PMS5315	Os_Chr10	22364898	22364918	(CCG)7	(CCG)7	(CCG)7	(CCG)7	(CCG)7	(CCG)7	(CCG)6	(CCG)7	(CCG)7	(CCG)7	(CCG)7	-	Introns/Intergenic	-	-	ACCTCTCCACACGCTGATCT	CAGTGCCCTCGATTGATTCT	252	A	A	A	NA
PMS5316	Os_Chr10	22444844	22444933	(TA)45	(TA)45	(TA)11	(TA)45	(TA)45	(TA)7	(TA)45	(TA)45	(TA)10	(TA)45	(TA)45	-	Introns/Intergenic	-	-	CGCCACGTCATCTGTTAAACTAGC	TTAAATTCTGTGGGTGGCGTAGC	189	P	A	A	RM25877

Polymorphic SSR (PMS) markers	Chromosomes	Start physical positions (bp)	End physical positions (bp)	Nipponbare	Kasalath	Nagina22	IR64	Pokkali	Bala	Tainung67	Azucena	Moroberekan	*Oryza rufipogon*	*Oryza nivara*	MSU gene locus IDs	Structural annotation	Functional annotation	SSR functional effects	Forward primers (5'-3')	Reverse primers (5'-3')	Amplified product size (bp)	Markers exhibiting polymorphism within *indica* rice	Transcrption factor genes-derived markers	Known cloned genes-derived markers	PMS markers corresponding with RM markers
PMS5317	Os_Chr10	22501756	22501803	(TC)24	(TC)24	(TC)24	(TC)24	(TC)24	(TC)24	(TC)24	(TC)24	(TC)24	(TC)24	(TC)23	-	Introns/Intergenic	-	-	CAGGGTCACACAGATCATCG	CATGCGATCCTGTGCTAAAA	239	A	A	A	NA
PMS5318	Os_Chr10	22537474	22537511	(AT)19	(AT)19	(AT)7	(AT)19	(AT)7	(AT)19	(AT)19	(AT)19	(AT)19	(AT)19	(AT)19	-	Introns/Intergenic	-	-	TCATCGCAATGCAAGAGTTC	CATGTAAGTGCACTGTAATTTTGA	222	P	A	A	NA
PMS5319	Os_Chr10	22597328	22597348	(AGC)7	(AGC)7	(AGC)7	(AGC)7	(AGC)7	(AGC)7	(AGC)7	(AGC)7	(AGC)7	(AGC)7	(AGC)6	-	Introns/Intergenic	-	-	ACATGGTTGCAGTCTTGCAG	CGCCACATCATGTTAGATGC	215	A	A	A	NA
PMS5320	Os_Chr10	22649904	22649919	(AG)8	(AG)8	(AG)8	(AG)7	(AG)8	(AG)8	(AG)8	(AG)8	(AG)8	(AG)8	(AG)8	-	Introns/Intergenic	-	-	ACTCCACTTGGTTGGAGCAA	CGCACAGAAGCAGATGATGT	170	P	A	A	NA
PMS5321	Os_Chr10	22658232	22658252	(GTC)7	(GTC)6	(GTC)7	(GTC)7	(GTC)7	(GTC)6	(GTC)6	(GTC)6	(GTC)6	(GTC)5	(GTC)6	-	Introns/Intergenic	-	-	CGTCCACTCCAGATTCCATT	TAGCAAGGTAGCGGTCGAAT	162	P	A	A	NA
PMS5322	Os_Chr10	22659915	22659940	(CT)13	(CT)13	(CT)13	(CT)13	(CT)13	(CT)11	(CT)13	(CT)13	(CT)13	(CT)13	(CT)13	-	Introns/Intergenic	-	-	GCCCATCTCTCCCTCTCC	GGGAAGCGTCTTTGCGAG	223	P	A	A	NA
PMS5323	Os_Chr10	22711557	22711612	(AT)28	(AT)10	(AT)28	(AT)28	(AT)28	(AT)14	(AT)28	(AT)10	(AT)28	(AT)28	(AT)28	-	Introns/Intergenic	-	-	GCAGCCAATCAGAAATCATAGC	AAATATGTCCGTGTCCCAATGC	177	P	A	A	RM25898
PMS5324	Os_Chr10	22814222	22814245	(GA)12	(GA)12	(GA)12	(GA)12	(GA)12	(GA)12	(GA)12	(GA)12	(GA)12	(GA)12	(GA)10	-	Introns/Intergenic	-	-	GAGCTCGCTAGGGATGGAG	ATCCCGAATCCAAATCCTTC	242	A	A	A	NA
PMS5325	Os_Chr10	22832893	22832916	(AGAT)6	(AGAT)6	(AGAT)6	(AGAT)6	(AGAT)6	(AGAT)6	(AGAT)6	(AGAT)6	(AGAT)6	(AGAT)6	(AGAT)5	-	Introns/Intergenic	-	-	CTCTCTTTGCCTAATTGCCTTGTTCG	TTTGTAGACCGGCAGCATTATTGG	165	A	A	A	RM7093
PMS5326	Os_Chr10	22836526	22836543	(ATG)6	(ATG)6	(ATG)6	(ATG)6	(ATG)6	(ATG)6	(ATG)6	(ATG)6	(ATG)5	(ATG)6	(ATG)6	-	Introns/Intergenic	-	-	ACTTTGCTCAGGTCGTCGTT	CCGCGTAGCTTCTATCGTTC	221	A	A	A	NA
PMS5327	Os_Chr10	22880767	22880782	(CT)8	(CT)8	(CT)8	(CT)8	(CT)8	(CT)7	(CT)8	(CT)8	(CT)8	(CT)8	(CT)8	-	Introns/Intergenic	-	-	GATGGGCTGAATTTCTCTC	AGGCGCGGCGGATTTGAG	276	P	A	A	NA
PMS5328	Os_Chr10	22965401	22965415	(CTG)5	(CTG)6	(CTG)6	(CTG)6	(CTG)6	(CTG)6	(CTG)6	(CTG)5	(CTG)5	(CTG)6	(CTG)5	LOC_Os10g42610	CDS	expressed protein	CODON_INSERTION	GTCCTCCTCCAGGATGTCG	TCAAGCTCAAGCTCAGCCTC	279	A	A	A	NA
PMS5329	Os_Chr10	22994691	22994711	(CAT)7	(CAT)7	(CAT)7	(CAT)7	(CAT)7	(CAT)7	(CAT)7	(CAT)7	(CAT)7	(CAT)9	(CAT)7	-	Introns/Intergenic	-	-	GATTTCAGCGAGGCGGATGAGG	CGGACCACCACCCATGTATCTGC	144	A	A	A	RM25921

Polymorphic SSR (PMS) markers	Chromosomes	Start physical positions (bp)	End physical positions (bp)	Nipponbare	Kasalath	Nagina22	IR64	Pokkali	Bala	Tainung67	Azucena	Moroberekan	*Oryza rufipogon*	*Oryza nivara*	MSU gene locus IDs	Structural annotation	Functional annotation	SSR functional effects	Forward primers (5'-3')	Reverse primers (5'-3')	Amplified product size (bp)	Markers exhibiting polymorphism within *indica* rice	Transcrption factor genes-derived markers	Known cloned genes-derived markers	PMS markers corresponding with RM markers
PMS5330	Os_Chr10	23011851	23011870	(CT)10	(CT)9	(CT)10	(CT)10	(CT)10	(CT)9	(CT)10	(CT)10	(CT)10	(CT)10	(CT)10	-	Introns/Intergenic	-	-	ACTTTTCGCAAGTCGTTCGT	TTGCCCATAAATTTCCAAGC	136	P	A	A	NA
PMS5331	Os_Chr10	23024023	23024043	(GAA)7	(GAA)7	(GAA)7	(GAA)7	(GAA)7	(GAA)7	(GAA)7	(GAA)7	(GAA)7	(GAA)9	(GAA)7	-	Introns/Intergenic	-	-	GCTAGGCTAAAGGTCAGGCA	ATCTCACACTTCCGTTCCGT	140	A	A	A	NA
PMS5332	Os_Chr10	23071742	23071756	(CGG)5	(CGG)6	(CGG)6	(CGG)6	(CGG)5	(CGG)6	(CGG)5	(CGG)5	(CGG)5	(CGG)5	(CGG)6	-	Introns/Intergenic	-	-	GAGATTACAAGGGGATCGCA	CTATGTTGGTTTGCTGGGCT	168	P	A	A	NA
PMS5333	Os_Chr10	23107375	23107392	(GTC)6	(GTC)5	(GTC)6	(GTC)6	(GTC)6	(GTC)5	(GTC)6	(GTC)6	(GTC)6	(GTC)6	(GTC)6	-	Introns/Intergenic	-	-	CCTTCGTCTTCTTTCCGTTG	CTGCCAAAACAAGCTGTCAC	237	P	A	A	NA
PMS5334	Os_Chr10	23114770	23114799	(AAG)10	(AAG)10	(AAG)10	(AAG)10	(AAG)10	(AAG)10	(AAG)10	(AAG)10	(AAG)10	(AAG)7	(AAG)10	LOC_Os10g42870	URR	peptide transporter PTR2, putative, expressed	-	TCTTGTTCGCCACCATTACA	ACTGCCGATCATATGGAAGC	239	A	A	A	NA
PMS5335	Os_Chr10	23183507	23183578	(AT)36	(AT)36	(AT)36	(AT)36	(AT)36	(AT)8	(AT)6	(AT)36	(AT)36	(AT)36	(AT)11	-	Introns/Intergenic	-	-	TTTCGTCGAAGATTGTCGTTT	CTCCAACCACAGCACGTAGA	238	P	A	A	NA
PMS5336	Os_Chr10	23185910	23185921	(AG)6	(AG)6	(AG)6	(AG)6	(AG)6	(AG)6	(AG)6	(AG)6	(AG)6	(AG)8	(AG)6	-	Introns/Intergenic	-	-	ATTCATTCTTGGCGTGTGTG	TTTAATGGATTGGAAAGCGG	249	A	A	A	NA
PMS5337	Os_Chr10	23186504	23186530	(CGG)9	(CGG)9	(CGG)9	(CGG)9	(CGG)9	(CGG)9	(CGG)9	(CGG)9	(CGG)9	(CGG)9	(CGG)7	LOC_Os10g43040	CDS	ankyrin repeat domain-containing protein 28, putative, expressed	CODON_CHANGE_PLUS_CODON_DELETION	TCCCATCCTTCTTCCTCTCTGATCC	CCTTGTGCAGATGCAAGTCATCC	148	A	A	A	RM25938
PMS5338	Os_Chr10	23195007	23195024	(TGG)6	(TGG)6	(TGG)6	(TGG)6	(TGG)6	(TGG)6	(TGG)5	(TGG)6	(TGG)6	(TGG)6	(TGG)6	LOC_Os10g43060	URR	expressed protein	-	GGAGAGCACAGCACAAATGA	TTCAGCGTGTGAAAATGGAG	240	A	A	A	NA
PMS5339	Os_Chr11	84523	84540	(TCG)6	(TCG)6	(TCG)6	(TCG)6	(TCG)6	(TCG)6	(TCG)6	(TCG)6	(TCG)6	(TCG)6	(TCG)5	-	Introns/Intergenic	-	-	CGTGGAGGAGAGGAGAAGAA	TGCCCTTACTCATTTGACCC	207	A	A	A	NA
PMS5340	Os_Chr11	125805	125856	(TA)26	(TA)26	(TA)13	(TA)26	(TA)26	(TA)26	(TA)7	(TA)8	(TA)26	(TA)26	(TA)26	-	Introns/Intergenic	-	-	AGGAAGGAAACAAAGCTACTACCC	CACATCACGTACTACCTCTGTTAGGG	500	P	A	A	RM25951
PMS5341	Os_Chr11	247570	247591	(AG)11	(AG)11	(AG)11	(AG)11	(AG)11	(AG)11	(AG)11	(AG)11	(AG)11	(AG)11	(AG)10	-	Introns/Intergenic	-	-	AGGTGGACCTCGTTCGTG	AGAGGCAACTGTGGCGAG	153	A	A	A	NA
PMS5342	Os_Chr11	448513	448533	(CCT)7	(CCT)7	(CCT)7	(CCT)7	(CCT)7	(CCT)7	(CCT)7	(CCT)7	(CCT)7	(CCT)6	(CCT)7	LOC_Os11g01842	CDS	SAG20, putative, expressed	CODON_DELETION	ATGCAGCCTTCTCTCTCCAAAGG	GACGTGCTGTACGCCAAAGG	80	A	A	A	RM25975

**Polymorphic SSR (PMS) markers**	**Chromosomes**	**Start physical positions (bp)**	**End physical positions (bp)**	**Nipponbare**	**Kasalath**	**Nagina22**	**IR64**	**Pokkali**	**Bala**	**Tainung67**	**Azucena**	**Moroberekan**	***Oryza rufipogon***	***Oryza nivara***	**MSU gene locus IDs**	**Structural annotation**	**Functional annotation**	**SSR functional effects**	**Forward primers (5'-3')**	**Reverse primers (5'-3')**	**Amplified product size (bp)**	**Markers exhibiting polymorphism within *indica* rice**	**Transcrption factor genes-derived markers**	**Known cloned genes-derived markers**	**PMS markers corresponding with RM markers**
PMS5343	Os_Chr11	669338	669349	(AC)6	(AC)9	(AC)6	(AC)6	(AC)6	(AC)6	(AC)6	(AC)6	(AC)6	(AC)6	(AC)6	-	Introns/Intergenic	-	-	GATCCAAATGGAAAGACCTGTGC	TTATTGCTTGCGCCTTTAGTCC	179	P	A	A	RM5568
PMS5344	Os_Chr11	755942	755973	(AT)16	(AT)16	(AT)7	(AT)16	(AT)16	(AT)16	(AT)16	(AT)16	(AT)16	(AT)16	(AT)12	-	Introns/Intergenic	-	-	ATTTTGTCGTGAGACCAGGC	ACCTCCCCCAAACTCGATAA	188	P	A	A	NA
PMS5345	Os_Chr11	851637	851657	(CCG)7	(CCG)7	(CCG)7	(CCG)7	(CCG)7	(CCG)7	(CCG)7	(CCG)7	(CCG)7	(CCG)7	(CCG)6	LOC_Os11g02660	URR	hydrolase, alpha/beta fold family domain containing protein, expressed	-	GTCTCCTTGCTGTGATTTGTTTGG	GCAGAGGGAGGGATAGCATGG	151	A	A	A	RM25998
PMS5346	Os_Chr11	957887	957900	(TG)7	(TG)7	(TG)7	(TG)7	(TG)7	(TG)7	(TG)8	(TG)7	(TG)8	(TG)7	(TG)7	-	Introns/Intergenic	-	-	AAGCGAATCAGATCACCGAC	CCGTCGCTATCTTGCATCTT	236	A	A	A	NA
PMS5347	Os_Chr11	1005383	1005448	(TA)33	(TA)33	(TA)7	(TA)7	(TA)7	(TA)33	(TA)33	(TA)33	(TA)11	(TA)33	(TA)33	-	Introns/Intergenic	-	-	GTGCGATGGTGGAGGTAAGT	ATTTGATACCATGGTGCCCG	223	P	A	A	NA
PMS5348	Os_Chr11	1149175	1149192	(CT)9	(CT)8	(CT)9	(CT)9	(CT)9	(CT)8	(CT)9	(CT)9	(CT)9	(CT)9	(CT)9	-	Introns/Intergenic	-	-	TTCCAAAGCAAAGAGGCAGT	AGGGAGGAAACCGACTTAGG	233	P	A	A	NA
PMS5349	Os_Chr11	1156495	1156512	(TC)9	(TC)9	(TC)9	(TC)9	(TC)9	(TC)9	(TC)9	(TC)10	(TC)9	(TC)9	(TC)9	-	Introns/Intergenic	-	-	AGGATGCCACATCAGTAGGC	GCCTGCCCAGAGTGTAGAGA	103	A	A	A	NA
PMS5350	Os_Chr11	1233278	1233295	(AT)9	(AT)9	(AT)7	(AT)9	(AT)9	(AT)9	(AT)6	(AT)9	(AT)9	(AT)9	(AT)9	-	Introns/Intergenic	-	-	AACGAACGTGGTGACTCCTT	GGTAATAAGATGAGAGCCACACAA	146	P	A	A	NA
PMS5351	Os_Chr11	1242166	1242183	(GCA)6	(GCA)6	(GCA)6	(GCA)6	(GCA)6	(GCA)6	(GCA)6	(GCA)6	(GCA)6	(GCA)6	(GCA)5	LOC_Os11g03310	CDS	no apical meristem protein, putative, expressed	CODON_DELETION	CTACCGCCAGTAGCTTCCAG	CGTACCATAATCCGGGACAG	200	A	P	A	NA
PMS5352	Os_Chr11	1339333	1339384	(TA)26	(TA)15	(TA)7	(TA)26	(TA)26	(TA)26	(TA)26	(TA)26	(TA)6	(TA)26	(TA)7	-	Introns/Intergenic	-	-	TGGCACACACGTGGTAATCT	GGACGAATTATCCCCTGACA	237	P	A	A	NA
PMS5353	Os_Chr11	1602351	1602364	(GA)7	(GA)7	(GA)7	(GA)7	(GA)7	(GA)7	(GA)7	(GA)7	(GA)7	(GA)7	(GA)9	-	Introns/Intergenic	-	-	CAATCCGAGAACGAGGAGAG	TCCCCATCACCCACTATTTC	167	A	A	A	NA
PMS5354	Os_Chr11	1633136	1633153	(GA)9	(GA)9	(GA)9	(GA)9	(GA)9	(GA)9	(GA)9	(GA)6	(GA)9	(GA)9	(GA)9	-	Introns/Intergenic	-	-	GGAGATTGGTGAAGGCAAAA	ATTGTGGCTGCTTAGGCTGT	154	A	A	A	NA
PMS5355	Os_Chr11	1642590	1642601	(AT)6	(AT)8	(AT)6	(AT)8	(AT)8	(AT)6	(AT)6	(AT)6	(AT)6	(AT)6	(AT)6	-	Introns/Intergenic	-	-	GGCACCTGAAACGAAGAGAC	CTGCTGCAGGTATATTGCCA	154	P	A	A	NA

Polymorphic SSR (PMS) markers	Chromosomes	Start physical positions (bp)	End physical positions (bp)	Nipponbare	Kasalath	Nagina22	IR64	Pokkali	Bala	Tainung67	Azucena	Moroberekan	*Oryza rufipogon*	*Oryza nivara*	MSU gene locus IDs	Structural annotation	Functional annotation	SSR functional effects	Forward primers (5'-3')	Reverse primers (5'-3')	Amplified product size (bp)	Markers exhibiting polymorphism within *indica* rice	Transcrption factor genes-derived markers	Known cloned genes-derived markers	PMS markers corresponding with RM markers
PMS5356	Os_Chr11	1940197	1940216	(CT)10	(CT)10	(CT)10	(CT)6	(CT)7	(CT)10	(CT)10	(CT)9	(CT)10	(CT)10	(CT)10	-	Introns/Intergenic	-	-	TCCTCTCTCTCTTCCCCCTC	AAGAAAACGGTCGCAAAATG	183	P	A	A	NA
PMS5357	Os_Chr11	2144103	2144116	(AC)7	(AC)7	(AC)7	(AC)7	(AC)7	(AC)7	(AC)7	(AC)7	(AC)7	(AC)7	(AC)8	-	Introns/Intergenic	-	-	CAGCCAACCACTCTCTCTCC	TGGCTTGTTTTGCTTGTGAG	260	A	A	A	NA
PMS5358	Os_Chr11	2159439	2159452	(TG)7	(TG)6	(TG)6	(TG)7	(TG)6	(TG)6	(TG)7	(TG)7	(TG)7	(TG)7	(TG)7	-	Introns/Intergenic	-	-	ACATCCCGAGGATTGCATAC	CACGTACATGCTCACTACTCCC	271	P	A	A	NA
PMS5359	Os_Chr11	2175360	2175381	(CT)11	(CT)11	(CT)11	(CT)11	(CT)11	(CT)11	(CT)11	(CT)11	(CT)11	(CT)11	(CT)16	-	Introns/Intergenic	-	-	ATAATGGCGGCTTTAAGCGTAGG	GGCAACAGATTGTTCAGGTTAAGG	113	A	A	A	RM26058
PMS5360	Os_Chr11	2291370	2291390	(CTC)7	(CTC)7	(CTC)8	(CTC)7	(CTC)7	(CTC)8	(CTC)7	(CTC)7	(CTC)7	(CTC)7	(CTC)7	LOC_Os11g05190	URR	phytosulfokines precursor, putative, expressed	-	CAAAGCAGCAATTGCAAGAAGC	AAACTATGGTGCGTTGTCGAAGG	95	P	A	A	RM26065
PMS5361	Os_Chr11	2344710	2344737	(GAGT)7	(GAGT)7	(GAGT)7	(GAGT)7	(GAGT)7	(GAGT)7	(GAGT)7	(GAGT)7	(GAGT)7	(GAGT)7	(GAGT)5	LOC_Os11g05290	URR	stress responsive A/B Barrel domain containing protein, expressed	-	CCTCCAGGTGAAGTGCCTTTGC	CTTCATCTTCCCTGCTGCCTTTGC	251	A	A	A	RM7557
PMS5362	Os_Chr11	2404826	2404839	(CT)7	(CT)7	(CT)8	(CT)7	(CT)7	(CT)7	(CT)7	(CT)7	(CT)7	(CT)7	(CT)7	-	Introns/Intergenic	-	-	GTTTTGCCCTGCTGTTTGAT	AAATTTCGGCATCAATTTCG	169	P	A	A	NA
PMS5363	Os_Chr11	2432501	2432516	(CT)8	(CT)8	(CT)8	(CT)8	(CT)9	(CT)8	(CT)8	(CT)8	(CT)8	(CT)8	(CT)8	-	Introns/Intergenic	-	-	CGTGTCGATCAACTGAGCAT	CACTCTGTTCCAGCCTCTCC	137	P	A	A	NA
PMS5364	Os_Chr11	2481608	2481623	(TC)8	(TC)8	(TC)8	(TC)8	(TC)8	(TC)8	(TC)8	(TC)8	(TC)8	(TC)6	(TC)8	LOC_Os11g05494	URR	expressed protein	-	GCAATTGAAAGGAACCAAGC	CAAAACGAGAGAGAGGACCG	119	A	A	A	NA
PMS5365	Os_Chr11	2640645	2640659	(TTC)5	(TTC)6	(TTC)6	(TTC)6	(TTC)6	(TTC)6	(TTC)5	(TTC)5	(TTC)5	(TTC)5	(TTC)6	LOC_Os11g05760	DRR	OsProCP5 - Putative Lysosomal Pro-x Carboxypeptidase homologue, expressed	-	ACGATTACGTTTCACCCAGC	TGGATTGGTTTTATTCCATATTGTT	222	A	A	A	NA
PMS5366	Os_Chr11	2688418	2688447	(CCG)10	(CCG)10	(CCG)10	(CCG)10	(CCG)10	(CCG)10	(CCG)10	(CCG)10	(CCG)10	(CCG)10	(CCG)8	LOC_Os11g05810	CDS	expressed protein	CODON_DELETION	GTCAGTCACCGTCCGAAGAGC	GCCGCCGGAGATAGAGATCC	357	A	A	A	RM26091
PMS5367	Os_Chr11	2705366	2705383	(AT)9	(AT)9	(AT)9	(AT)9	(AT)9	(AT)9	(AT)6	(AT)9	(AT)9	(AT)9	(AT)9	-	Introns/Intergenic	-	-	GCTTTGTCCAATCCAACGAT	CTTGCAAAGTAGGAGGCTGG	187	A	A	A	NA
PMS5368	Os_Chr11	2711030	2711069	(AT)20	(AT)20	(AT)20	(AT)9	(AT)20	(AT)10	(AT)20	(AT)20	(AT)20	(AT)20	(AT)20	-	Introns/Intergenic	-	-	CACTACTCCCACCACCAACTCC	ATTTGGGTCCAAGTGATGAACC	381	P	A	A	RM26093

Polymorphic SSR (PMS) markers	Chromosomes	Start physical positions (bp)	End physical positions (bp)	Nipponbare	Kasalath	Nagina22	IR64	Pokkali	Bala	Tainung67	Azucena	Moroberekan	*Oryza rufipogon*	*Oryza nivara*	MSU gene locus IDs	Structural annotation	Functional annotation	SSR functional effects	Forward primers (5'-3')	Reverse primers (5'-3')	Amplified product size (bp)	Markers exhibiting polymorphism within *indica* rice	Transcrption factor genes-derived markers	Known cloned genes-derived markers	PMS markers corresponding with RM markers
PMS5369	Os_Chr11	2759507	2759536	(TA)15	(TA)15	(TA)8	(TA)15	(TA)15	(TA)15	(TA)7	(TA)15	(TA)7	(TA)15	(TA)7	-	Introns/Intergenic	-	-	TTGTGCTCGGGAAATAAAATG	AAACCGTGTCGATCTGTTCC	240	P	A	A	NA
PMS5370	Os_Chr11	2812219	2812296	(TA)39	(TA)39	(TA)9	(TA)39	(TA)39	(TA)7	(TA)12	(TA)39	(TA)6	(TA)39	(TA)39	LOC_Os11g05970	DRR	pyridine nucleotide-disulphide oxidoreductase family protein, putative, expressed	-	GCGCTATGATCAATCCATGTTTCG	AGTTTGTGCTAGACGAGGCAGTCC	149	P	A	A	RM26101
PMS5371	Os_Chr11	2869811	2869885	(ATT)25	(ATT)25	(ATT)25	(ATT)9	(ATT)25	(ATT)25	(ATT)25	(ATT)25	(ATT)22	(ATT)25	(ATT)25	-	Introns/Intergenic	-	-	TTGAAAATGTGCCACGAAAA	TAGCGAGTCTTTTGCCACCT	228	P	A	A	NA
PMS5372	Os_Chr11	2917884	2917907	(AAG)8	(AAG)8	(AAG)8	(AAG)8	(AAG)8	(AAG)8	(AAG)7	(AAG)8	(AAG)8	(AAG)8	(AAG)8	-	Introns/Intergenic	-	-	GGGAACACAAGCGAGATTTGC	GGAGAGAGATTGACAGGGATCTGC	172	A	A	A	RM26113
PMS5373	Os_Chr11	2940032	2940046	(CTT)5	(CTT)6	(CTT)5	(CTT)6	(CTT)5	(CTT)5	(CTT)5	(CTT)5	(CTT)5	(CTT)6	(CTT)6	-	Introns/Intergenic	-	-	TTGAAGTTGCTGGCCTATTG	CACCTTCCTCTCCAGCTCAC	181	P	A	A	NA
PMS5374	Os_Chr11	2965880	2965911	(TA)16	(TA)16	(TA)16	(TA)16	(TA)16	(TA)10	(TA)6	(TA)13	(TA)16	(TA)16	(TA)16	-	Introns/Intergenic	-	-	CGCACTGAACGGTTTATATGATGTGC	ACAGCAGAAACCAGAAACTCCATGC	647	P	A	A	RM26116
PMS5375	Os_Chr11	2979142	2979173	(TC)16	(TC)15	(TC)16	(TC)16	(TC)16	(TC)16	(TC)16	(TC)16	(TC)16	(TC)16	(TC)16	-	Introns/Intergenic	-	-	CAATTGACGGACCTCATACTGC	ACACCACTGTCAGAACTCAGAAGC	344	P	A	A	RM3729
PMS5376	Os_Chr11	3036359	3036379	(CCG)7	(CCG)7	(CCG)7	(CCG)7	(CCG)5	(CCG)7	(CCG)7	(CCG)7	(CCG)7	(CCG)7	(CCG)7	LOC_Os11g06330	CDS	expressed protein	CODON_DELETION	CAGCAGAACAAGGCGTTGAAGG	ATGTTCGGCTTGGTGTCTCTGG	200	P	A	A	RM26119
PMS5377	Os_Chr11	3065835	3065852	(CCG)6	(CCG)6	(CCG)6	(CCG)6	(CCG)6	(CCG)6	(CCG)6	(CCG)6	(CCG)6	(CCG)6	(CCG)5	LOC_Os11g06370	CDS	expressed protein	CODON_DELETION	GGGGATCATCGTCACATACC	GTGCTGTGCACATGGAAATC	135	A	A	A	NA
PMS5378	Os_Chr11	3160840	3160857	(GA)9	(GA)11	(GA)9	(GA)9	(GA)9	(GA)9	(GA)9	(GA)9	(GA)9	(GA)9	(GA)9	-	Introns/Intergenic	-	-	CTTGGCCTGTGACCCTATGT	AACCCTATCCACTCCCCTTG	156	P	A	A	NA
PMS5379	Os_Chr11	3246283	3246297	(CGG)5	(CGG)5	(CGG)5	(CGG)5	(CGG)5	(CGG)6	(CGG)5	(CGG)5	(CGG)5	(CGG)5	(CGG)5	-	Introns/Intergenic	-	-	GTGATCCTGCTCGCTGTCAT	GTCGTCATCCAACAAGCTCC	105	P	A	A	NA
PMS5380	Os_Chr11	3266806	3266829	(CGG)8	(CGG)8	(CGG)8	(CGG)8	(CGG)8	(CGG)8	(CGG)7	(CGG)8	(CGG)8	(CGG)8	(CGG)8	LOC_Os11g06700	CDS	exo70 exocyst complex subunit, putative, expressed	CODON_CHANGE_PLUS_CODON_DELETION	GTCGAGGACGAGTTCCACACG	CCTTCTCGTACCCGAGGTTCTGC	331	A	A	A	RM26139
PMS5381	Os_Chr11	3308258	3308278	(GCC)7	(GCC)7	(GCC)7	(GCC)7	(GCC)7	(GCC)6	(GCC)7	(GCC)7	(GCC)7	(GCC)7	(GCC)7	LOC_Os11g06770	CDS	ethylene-responsive transcription factor ERF110, putative, expressed	CODON_DELETION	CTGCAAGAACCTGGCGTACTCG	GCCTTCAGTACTCTGTTTCGGTTGG	459	P	A	A	RM26140

Polymorphic SSR (PMS) markers	Chromosomes	Start physical positions (bp)	End physical positions (bp)	Nipponbare	Kasalath	Nagina22	IR64	Pokkali	Bala	Tainung67	Azucena	Moroberekan	*Oryza rufipogon*	*Oryza nivara*	MSU gene locus IDs	Structural annotation	Functional annotation	SSR functional effects	Forward primers (5'-3')	Reverse primers (5'-3')	Amplified product size (bp)	Markers exhibiting polymorphism within *indica* rice	Transcrption factor genes-derived markers	Known cloned genes-derived markers	PMS markers corresponding with RM markers
PMS5382	Os_Chr11	3357697	3357717	(GCG)7	(GCG)5	(GCG)7	(GCG)6	(GCG)7	(GCG)5	(GCG)7	(GCG)7	(GCG)7	(GCG)7	(GCG)7	LOC_Os11g06840	CDS	ZOS11-01 - C2H2 zinc finger protein, expressed	CODON_CHANGE_PLUS_CODON_DELETION	TGGCAAGAAGGAGAAGAGGA	GCCGTCATCACAGAGTTGAA	249	P	P	A	NA
PMS5383	Os_Chr11	3428400	3428417	(GCC)6	(GCC)6	(GCC)6	(GCC)6	(GCC)6	(GCC)6	(GCC)5	(GCC)6	(GCC)6	(GCC)6	(GCC)6	-	Introns/Intergenic	-	-	GTGTGTGACCTCGCCTGTC	ACCGAGGATAGGGAGAGGAA	200	A	A	A	NA
PMS5384	Os_Chr11	3494707	3494727	(GCG)7	(GCG)7	(GCG)7	(GCG)7	(GCG)7	(GCG)7	(GCG)7	(GCG)7	(GCG)7	(GCG)5	(GCG)7	LOC_Os11g07030	URR	integrator complex subunit 3, putative, expressed	-	AGAGGGAGGTGAAGGGAGG	AGGGAATCCATCCAAAATCC	190	A	A	A	NA
PMS5385	Os_Chr11	3633361	3633381	(GGC)7	(GGC)7	(GGC)7	(GGC)7	(GGC)7	(GGC)5	(GGC)7	(GGC)7	(GGC)5	(GGC)5	(GGC)7	LOC_Os11g07230	URR	receptor kinase, putative, expressed	-	CCGTCCCTCGGTAACTGTAAGC	AGCCCTGAAACCATGGAACG	162	P	A	A	RM26153
PMS5386	Os_Chr11	3655660	3655689	(TA)15	(TA)11	(TA)15	(TA)15	(TA)15	(TA)15	(TA)15	(TA)15	(TA)10	(TA)15	(TA)15	-	Introns/Intergenic	-	-	ACACTATATGCTCTTCTTCCAA	TGAGTTTAGGGTCCACATAGA	299	P	A	A	NA
PMS5387	Os_Chr11	3699012	3699023	(TC)6	(TC)10	(TC)6	(TC)6	(TC)6	(TC)6	(TC)6	(TC)6	(TC)6	(TC)6	(TC)6	-	Introns/Intergenic	-	-	ATCCCAATCCTTCCCAAATC	GGAGAGGGAGAGCGAGGG	212	P	A	A	NA
PMS5388	Os_Chr11	3733914	3733937	(TCG)8	(TCG)8	(TCG)8	(TCG)8	(TCG)8	(TCG)8	(TCG)8	(TCG)8	(TCG)8	(TCG)5	(TCG)7	-	Introns/Intergenic	-	-	CTAGTGCCTTGCTCTTCGTGTCC	TCCACAGTTACCCATCGTGAGC	155	A	A	A	RM26155
PMS5389	Os_Chr11	3763359	3763372	(AG)7	(AG)7	(AG)7	(AG)7	(AG)7	(AG)7	(AG)7	(AG)7	(AG)7	(AG)6	(AG)7	-	Introns/Intergenic	-	-	AACAGCACCAACCAGGTCTC	TTGGAAATGGAGAGGACAGG	145	A	A	A	NA
PMS5390	Os_Chr11	3776084	3776117	(TA)17	(TA)17	(TA)17	(TA)6	(TA)17	(TA)17	(TA)17	(TA)8	(TA)17	(TA)17	(TA)8	-	Introns/Intergenic	-	-	TGGTCTACTGCTACCTGGCTCTGTCC	GGTGGTAAAGGTGCGGGTTGG	217	P	A	A	RM26159
PMS5391	Os_Chr11	3779079	3779094	(TA)8	(TA)8	(TA)8	(TA)8	(TA)8	(TA)8	(TA)9	(TA)8	(TA)8	(TA)8	(TA)8	-	Introns/Intergenic	-	-	AAATGGCTAATATGTGCGGG	GCCAGTATGGGTTGCACTTT	231	A	A	A	NA
PMS5392	Os_Chr11	3819542	3819557	(AT)8	(AT)8	(AT)8	(AT)8	(AT)8	(AT)8	(AT)8	(AT)8	(AT)9	(AT)8	(AT)8	-	Introns/Intergenic	-	-	AACGTCCGTTAGTGGGTGAT	CATTTTCGTCGATCTGAGCA	279	A	A	A	NA
PMS5393	Os_Chr11	3828536	3828568	(TGT)11	(TGT)13	(TGT)11	(TGT)11	(TGT)6	(TGT)11	(TGT)11	(TGT)6	(TGT)11	(TGT)11	(TGT)11	-	Introns/Intergenic	-	-	GTCAGCGTGGTGAATGTGTTGC	ATCCACCCAACAACACAAAGAGG	153	P	A	A	RM5599
PMS5394	Os_Chr11	3835380	3835401	(AT)11	(AT)11	(AT)11	(AT)10	(AT)10	(AT)11	(AT)11	(AT)11	(AT)11	(AT)11	(AT)6	-	Introns/Intergenic	-	-	TACTCTCACCGTCCCATAATACAAGG	TTAGTTTGCTTTGCACGAGAGG	678	P	A	A	RM26164

Polymorphic SSR (PMS) markers	Chromosomes	Start physical positions (bp)	End physical positions (bp)	Nipponbare	Kasalath	Nagina22	IR64	Pokkali	Bala	Tainung67	Azucena	Moroberekan	*Oryza rufipogon*	*Oryza nivara*	MSU gene locus IDs	Structural annotation	Functional annotation	SSR functional effects	Forward primers (5'-3')	Reverse primers (5'-3')	Amplified product size (bp)	Markers exhibiting polymorphism within *indica* rice	Transcrption factor genes-derived markers	Known cloned genes-derived markers	PMS markers corresponding with RM markers
PMS5395	Os_Chr11	3848601	3848615	(CGG)5	(CGG)6	(CGG)5	(CGG)5	(CGG)5	(CGG)5	(CGG)5	(CGG)5	(CGG)5	(CGG)5	(CGG)5	LOC_Os11g07590	CDS	expressed protein	CODON_INSERTION	ATCGACGACCTCTTCCCC	TACAGGTCGAGGAGAGAGCC	122	P	A	A	NA
PMS5396	Os_Chr11	3857799	3857825	(AGC)9	(AGC)8	(AGC)9	(AGC)9	(AGC)9	(AGC)8	(AGC)9	(AGC)8	(AGC)9	(AGC)9	(AGC)5	-	Introns/Intergenic	-	-	ACACCGTGAATGCTCTGCTTCG	ATGCACCCTTCGTCACAGAAGATCC	169	P	A	A	RM6544
PMS5397	Os_Chr11	3871264	3871277	(AC)7	(AC)7	(AC)7	(AC)7	(AC)7	(AC)7	(AC)7	(AC)7	(AC)7	(AC)6	(AC)6	-	Introns/Intergenic	-	-	GATTATCGGTGAAACGGTGG	TGCATGCATCCAATAGAATTAG	242	A	A	A	NA
PMS5398	Os_Chr11	3920527	3920544	(CCG)6	(CCG)6	(CCG)6	(CCG)6	(CCG)6	(CCG)6	(CCG)6	(CCG)6	(CCG)6	(CCG)6	(CCG)5	LOC_Os11g07680	CDS	dirigent, putative, expressed	CODON_DELETION	GTTCATGTCCATCAGGAGGC	TCAAGGTGTACTGGCACGAC	230	A	A	A	NA
PMS5399	Os_Chr11	4015373	4015386	(AC)7	(AC)7	(AC)7	(AC)7	(AC)7	(AC)7	(AC)7	(AC)7	(AC)7	(AC)6	(AC)7	-	Introns/Intergenic	-	-	AGGGAGAGTGGTCATGATGC	CACCCCTACTTGAACTGGGA	189	A	A	A	NA
PMS5400	Os_Chr11	4037742	4037777	(AT)18	(AT)6	(AT)18	(AT)18	(AT)18	(AT)18	(AT)18	(AT)18	(AT)18	(AT)18	(AT)18	-	Introns/Intergenic	-	-	TATCCAATGCGAATGGTACG	ATTTTTGATGGATTCGGTGC	266	P	A	A	NA
PMS5401	Os_Chr11	4091497	4091514	(CGG)6	(CGG)7	(CGG)7	(CGG)6	(CGG)6	(CGG)7	(CGG)6	(CGG)6	(CGG)6	(CGG)6	(CGG)6	LOC_Os11g07916	CDS	nifU, putative, expressed	CODON_CHANGE_PLUS_CODON_INSERTION	TAGGCCCAGGTCATCTTTTG	TTGGCAGAACCAAGGAAATC	182	P	A	A	NA
PMS5402	Os_Chr11	4265615	4265630	(CT)8	(CT)8	(CT)8	(CT)8	(CT)8	(CT)8	(CT)8	(CT)8	(CT)8	(CT)8	(CT)7	-	Introns/Intergenic	-	-	TCGAGTTCAATTCCGAATCC	AGGGGAGCGGAGAGAGAG	241	A	A	A	NA
PMS5403	Os_Chr11	4287846	4287871	(TC)13	(TC)13	(TC)13	(TC)7	(TC)13	(TC)13	(TC)13	(TC)13	(TC)13	(TC)13	(TC)13	-	Introns/Intergenic	-	-	CGGAGGAAGTAATTGGGTTGAGC	CTACCCAGAAGGGTAGACGTTGC	275	P	A	A	RM26186
PMS5404	Os_Chr11	4299175	4299228	(TTA)18	(TTA)18	(TTA)18	(TTA)18	(TTA)18	(TTA)18	(TTA)5	(TTA)12	(TTA)5	(TTA)18	(TTA)18	LOC_Os11g08210	DRR	OsNAC5	-	TGGTGAGAGGGCTAGAGAGATCG	AATGTAAGGGTGATTGCGACATGG	211	A	P	P	RM26187
PMS5405	Os_Chr11	4343377	4343428	(ATAG)13	(ATAG)13	(ATAG)9	(ATAG)9	(ATAG)5	(ATAG)5	(ATAG)13	(ATAG)13	(ATAG)13	(ATAG)5	(ATAG)9	-	Introns/Intergenic	-	-	TGACTGTGCTGATACCGTTAGCC	GCGTCAACACCTGCTTCTTAGG	226	P	A	A	RM26190
PMS5406	Os_Chr11	4343871	4343916	(TA)23	(TA)23	(TA)23	(TA)23	(TA)23	(TA)23	(TA)23	(TA)23	(TA)11	(TA)23	(TA)11	-	Introns/Intergenic	-	-	TTAGGGTTGCTAGAACTCAACC	GCTGCTATCCCAATGTTTAGG	204	A	A	A	RM26191
PMS5407	Os_Chr11	4364109	4364128	(AAAT)5	(AAAT)5	(AAAT)6	(AAAT)6	(AAAT)6	(AAAT)5	(AAAT)5	(AAAT)5	(AAAT)5	(AAAT)5	(AAAT)5	-	Introns/Intergenic	-	-	CGTACGATCAAGGGATGTAGAGAGC	CGTCAGTGGTCCTAGCTTGATCG	103	P	A	A	RM26193

**Polymorphic SSR (PMS) markers**	**Chromosomes**	**Start physical positions (bp)**	**End physical positions (bp)**	**Nipponbare**	**Kasalath**	**Nagina22**	**IR64**	**Pokkali**	**Bala**	**Tainung67**	**Azucena**	**Moroberekan**	***Oryza rufipogon***	***Oryza nivara***	**MSU gene locus IDs**	**Structural annotation**	**Functional annotation**	**SSR functional effects**	**Forward primers (5'-3')**	**Reverse primers (5'-3')**	**Amplified product size (bp)**	**Markers exhibiting polymorphism within *indica* rice**	**Transcrption factor genes-derived markers**	**Known cloned genes-derived markers**	**PMS markers corresponding with RM markers**
PMS5408	Os_Chr11	4427454	4427477	(AC)12	(AC)12	(AC)12	(AC)12	(AC)14	(AC)12	(AC)12	(AC)12	(AC)12	(AC)12	(AC)12	-	Introns/Intergenic	-	-	TTCAGGACCTTTGAACTGGG	GGCACACAGTTTATGAGCAAA	253	P	A	A	NA
PMS5409	Os_Chr11	4464155	4464175	(TAA)7	(TAA)5	(TAA)7	(TAA)7	(TAA)7	(TAA)7	(TAA)6	(TAA)7	(TAA)7	(TAA)7	(TAA)7	-	Introns/Intergenic	-	-	CTTAATCCCAGCCCTGGAAGC	GATTGATGTCAGCCGTAACATGG	169	P	A	A	RM26200
PMS5410	Os_Chr11	4658780	4658793	(AG)7	(AG)7	(AG)6	(AG)6	(AG)7	(AG)6	(AG)7	(AG)7	(AG)7	(AG)7	(AG)7	-	Introns/Intergenic	-	-	GGAGGGTCCAAACACACCTA	CATGCATCGCTGGCTAGTAA	154	P	A	A	NA
PMS5411	Os_Chr11	4802322	4802349	(TA)14	(TA)14	(TA)7	(TA)7	(TA)6	(TA)14	(TA)14	(TA)14	(TA)14	(TA)14	(TA)8	-	Introns/Intergenic	-	-	TTCTTCCAATCTCCTGGTGC	TCAGAATAACCTCTGCAAGCAT	256	P	A	A	NA
PMS5412	Os_Chr11	4820551	4820586	(AT)18	(AT)18	(AT)18	(AT)18	(AT)18	(AT)8	(AT)18	(AT)18	(AT)18	(AT)18	(AT)18	-	Introns/Intergenic	-	-	CCAAAGGCACAATGGGCTATCG	CCATAAAGCACTAGGCCCGAAGG	412	P	A	A	RM26217
PMS5413	Os_Chr11	4864436	4864449	(CT)7	(CT)7	(CT)7	(CT)7	(CT)7	(CT)7	(CT)6	(CT)8	(CT)8	(CT)7	(CT)7	-	Introns/Intergenic	-	-	GTCACAAAAGGTGCTTCGGT	GGAGGAGCTGATGGCTTTC	270	A	A	A	NA
PMS5414	Os_Chr11	4925575	4925598	(ACCA)6	(ACCA)6	(ACCA)6	(ACCA)6	(ACCA)6	(ACCA)6	(ACCA)6	(ACCA)6	(ACCA)5	(ACCA)6	(ACCA)6	LOC_Os11g09180	URR	expressed protein	-	GTTCTTGGAGTTTGGAGATTGG	CAAATCAAAGACCTGGCATAGC	93	A	A	A	RM26221
PMS5415	Os_Chr11	5086591	5086650	(TA)30	(TA)30	(TA)30	(TA)7	(TA)7	(TA)30	(TA)8	(TA)30	(TA)8	(TA)30	(TA)30	-	Introns/Intergenic	-	-	CCCTAAGATTCGTGCAAGCTA	CGATCTTCAGGCATCCTGTT	217	P	A	A	NA
PMS5416	Os_Chr11	5139804	5139815	(TC)6	(TC)7	(TC)7	(TC)7	(TC)7	(TC)7	(TC)6	(TC)6	(TC)6	(TC)6	(TC)6	-	Introns/Intergenic	-	-	GCCAATGGGGTGATTCTTTT	TGCGAATGTACGTTAAGGGTC	270	A	A	A	NA
PMS5417	Os_Chr11	5265069	5265084	(AT)8	(AT)8	(AT)8	(AT)8	(AT)8	(AT)8	(AT)8	(AT)6	(AT)8	(AT)8	(AT)8	-	Introns/Intergenic	-	-	GCAACTATCTGCTTGCCTCC	GCGGAAAACGAGACTCAATC	265	A	A	A	NA
PMS5418	Os_Chr11	5383260	5383274	(ATC)5	(ATC)5	(ATC)5	(ATC)5	(ATC)5	(ATC)5	(ATC)5	(ATC)5	(ATC)5	(ATC)6	(ATC)5	LOC_Os11g10050	CDS	G-protein alpha subunit, putative, expressed	CODON_INSERTION	CATCTCCTCGAACTCCTTGC	TCGGTACTGCAGAATGAGGA	189	A	A	A	NA
PMS5419	Os_Chr11	5400576	5400590	(GCA)5	(GCA)6	(GCA)6	(GCA)6	(GCA)6	(GCA)6	(GCA)5	(GCA)6	(GCA)6	(GCA)6	(GCA)5	LOC_Os11g10060	CDS	transcriptional corepressor SEUSS, putative, expressed	CODON_CHANGE_PLUS_CODON_INSERTION	ATACCCTCCAGCAGCAGAGA	TTCACTGTAGCCTGTGGCTG	280	A	A	A	NA
PMS5420	Os_Chr11	5476123	5476174	(TA)26	(TA)26	(TA)22	(TA)7	(TA)18	(TA)26	(TA)26	(TA)13	(TA)26	(TA)26	(TA)26	-	Introns/Intergenic	-	-	TTTGTAGTCTCTTCGTGCATCC	GGCCATGCTACTAATTTCAGC	370	P	A	A	RM26238

Polymorphic SSR (PMS) markers	Chromosomes	Start physical positions (bp)	End physical positions (bp)	Nipponbare	Kasalath	Nagina22	IR64	Pokkali	Bala	Tainung67	Azucena	Moroberekan	*Oryza rufipogon*	*Oryza nivara*	MSU gene locus IDs	Structural annotation	Functional annotation	SSR functional effects	Forward primers (5'-3')	Reverse primers (5'-3')	Amplified product size (bp)	Markers exhibiting polymorphism within *indica* rice	Transcrption factor genes-derived markers	Known cloned genes-derived markers	PMS markers corresponding with RM markers
PMS5421	Os_Chr11	5481471	5481530	(ATT)20	(ATT)20	(ATT)20	(ATT)20	(ATT)20	(ATT)20	(ATT)9	(ATT)20	(ATT)20	(ATT)20	(ATT)20	-	Introns/Intergenic	-	-	TTGATCACCTACACGTACACATGC	AAGGTCCTTTGAGCATTCAGTCG	351	A	A	A	RM5704
PMS5422	Os_Chr11	5553466	5553483	(ATC)6	(ATC)6	(ATC)6	(ATC)6	(ATC)6	(ATC)6	(ATC)6	(ATC)6	(ATC)6	(ATC)6	(ATC)5	LOC_Os11g10230	CDS	retrotransposon protein, putative, Ty1-copia subclass, expressed	CODON_DELETION	TCTAGCTCAACTGTGGTGTCAGC	CTCGTTGGAGGTTTAGCATATTGG	368	A	A	A	RM26242
PMS5423	Os_Chr11	5561017	5561040	(CTG)8	(CTG)8	(CTG)8	(CTG)8	(CTG)8	(CTG)8	(CTG)8	(CTG)7	(CTG)8	(CTG)8	(CTG)8	-	Introns/Intergenic	-	-	TCCTATTTCTCTCGGAAACCATGC	TTGTCCTGTGCTGTCACGAACC	167	A	A	A	RM26243
PMS5424	Os_Chr11	5595296	5595343	(ATCT)12	(ATCT)12	(ATCT)12	(ATCT)13	(ATCT)12	(ATCT)7	(ATCT)12	(ATCT)12	(ATCT)12	(ATCT)12	(ATCT)12	-	Introns/Intergenic	-	-	TCAAACGTTGGTCCAAAAGTC	ACGTGGCAATCTAAGAGCGT	240	P	A	A	NA
PMS5425	Os_Chr11	5760554	5760571	(CTT)6	(CTT)6	(CTT)6	(CTT)6	(CTT)6	(CTT)6	(CTT)6	(CTT)6	(CTT)6	(CTT)6	(CTT)5	-	Introns/Intergenic	-	-	TGAGAGGTACCAGGAGGCAC	TTCGACATGACTCGGAGAGA	230	A	A	A	NA
PMS5426	Os_Chr11	5845298	5845315	(TCC)6	(TCC)6	(TCC)5	(TCC)6	(TCC)6	(TCC)6	(TCC)6	(TCC)6	(TCC)6	(TCC)6	(TCC)6	-	Introns/Intergenic	-	-	TAAACAGCCTCATCCATCCC	CCTTGCCTACCTCACGGTC	197	P	A	A	NA
PMS5427	Os_Chr11	5936686	5936703	(CCA)6	(CCA)6	(CCA)6	(CCA)6	(CCA)6	(CCA)6	(CCA)6	(CCA)6	(CCA)6	(CCA)7	(CCA)6	LOC_Os11g10790	CDS	expressed protein	CODON_CHANGE_PLUS_CODON_INSERTION	CTTCCCTATGCTCCCTCCTC	CGATGTGGCATCATTGTTGT	218	A	A	A	NA
PMS5428	Os_Chr11	5956930	5956981	(AT)26	(AT)26	(AT)26	(AT)26	(AT)26	(AT)26	(AT)26	(AT)26	(AT)26	(AT)7	(AT)26	-	Introns/Intergenic	-	-	GGAGGTTGTTGGGCATACTCTTATCC	TGCGTCGTGATTCTAGGAGAGAGC	394	A	A	A	RM26256
PMS5429	Os_Chr11	5978493	5978510	(CCA)6	(CCA)6	(CCA)6	(CCA)6	(CCA)6	(CCA)6	(CCA)6	(CCA)6	(CCA)6	(CCA)5	(CCA)6	LOC_Os11g10850	CDS	dirigent, putative, expressed	CODON_DELETION	GCGCTAGCACTCGCTATCTT	CGTCGTGCATGTAGAAGTGG	226	A	A	A	NA
PMS5430	Os_Chr11	6006932	6006975	(TA)22	(TA)10	(TA)22	(TA)22	(TA)8	(TA)15	(TA)6	(TA)6	(TA)22	(TA)22	(TA)7	-	Introns/Intergenic	-	-	GGGTTTCCATATCCAACTTTGAGC	AAGGAGAAATGGGCCGATGG	445	P	A	A	RM26260
PMS5431	Os_Chr11	6085183	6085200	(GA)9	(GA)9	(GA)9	(GA)9	(GA)9	(GA)6	(GA)9	(GA)9	(GA)9	(GA)9	(GA)9	-	Introns/Intergenic	-	-	TCAAGATTTGCACCAACCAA	GAGTGGCTCTCATCCACCAT	195	P	A	A	NA
PMS5432	Os_Chr11	6085497	6085530	(TC)17	(TC)15	(TC)17	(TC)17	(TC)17	(TC)17	(TC)17	(TC)17	(TC)17	(TC)17	(TC)17	-	Introns/Intergenic	-	-	AAGGGAGTAGCCTCTCCATCTCC	GTGCTGACTCCTCTCCCTGTCC	183	P	A	A	RM441
PMS5433	Os_Chr11	6137373	6137410	(TA)19	(TA)19	(TA)19	(TA)7	(TA)19	(TA)19	(TA)19	(TA)19	(TA)19	(TA)19	(TA)19	-	Introns/Intergenic	-	-	TCATAACTCGCCTAGAAACTGC	TCTAGCATGCATGGAGTAGTGC	385	P	A	A	RM26265

Polymorphic SSR (PMS) markers	Chromosomes	Start physical positions (bp)	End physical positions (bp)	Nipponbare	Kasalath	Nagina22	IR64	Pokkali	Bala	Tainung67	Azucena	Moroberekan	*Oryza rufipogon*	*Oryza nivara*	MSU gene locus IDs	Structural annotation	Functional annotation	SSR functional effects	Forward primers (5'-3')	Reverse primers (5'-3')	Amplified product size (bp)	Markers exhibiting polymorphism within *indica* rice	Transcrption factor genes-derived markers	Known cloned genes-derived markers	PMS markers corresponding with RM markers
PMS5434	Os_Chr11	6333970	6333989	(AT)10	(AT)10	(AT)10	(AT)6	(AT)9	(AT)10	(AT)10	(AT)9	(AT)10	(AT)9	(AT)10	-	Introns/Intergenic	-	-	GTTGGTGAGTAGGCTGGGAA	TAGATACCATGGTGCCTGGG	202	P	A	A	NA
PMS5435	Os_Chr11	6358534	6358549	(TA)8	(TA)8	(TA)8	(TA)8	(TA)8	(TA)8	(TA)6	(TA)8	(TA)8	(TA)8	(TA)8	-	Introns/Intergenic	-	-	TAAGTGCTGAAATGGCATCG	CGACAAAGTTGGGGAAGAAA	238	A	A	A	NA
PMS5436	Os_Chr11	6383394	6383423	(CGC)10	(CGC)10	(CGC)10	(CGC)10	(CGC)10	(CGC)9	(CGC)10	(CGC)10	(CGC)10	(CGC)10	(CGC)10	LOC_Os11g11490	CDS	TKL_IRAK_CR4L.7 - The CR4L subfamily has homology with Crinkly4, expressed	CODON_DELETION	AGCTGTACACGTCGCTCTTGTCC	GGCCTCTCCTCCATCCTAGATCC	169	P	A	A	RM26280
PMS5437	Os_Chr11	6405312	6405329	(CGC)6	(CGC)6	(CGC)6	(CGC)6	(CGC)6	(CGC)5	(CGC)6	(CGC)6	(CGC)6	(CGC)5	(CGC)6	LOC_Os11g11520	CDS	expressed protein	CODON_DELETION	GAGTTGGACCGGTTCTTGAG	CTTCTGCCTTCCACTCGC	188	P	A	A	NA
PMS5438	Os_Chr11	6408782	6408799	(CT)9	(CT)9	(CT)9	(CT)9	(CT)9	(CT)9	(CT)9	(CT)6	(CT)9	(CT)9	(CT)9	-	Introns/Intergenic	-	-	TTTCCCATCCAAATCTGAGC	ATCTCAATCCGAGGGAGAGA	222	A	A	A	NA
PMS5439	Os_Chr11	6452278	6452295	(GCG)6	(GCG)5	(GCG)6	(GCG)6	(GCG)6	(GCG)5	(GCG)6	(GCG)6	(GCG)6	(GCG)6	(GCG)6	-	Introns/Intergenic	-	-	GCCCACGTGTAAGTGACAAA	CCTGTTCCTCCTGAGCCTG	215	P	A	A	NA
PMS5440	Os_Chr11	6508389	6508402	(CT)7	(CT)7	(CT)7	(CT)8	(CT)7	(CT)7	(CT)7	(CT)7	(CT)7	(CT)7	(CT)7	-	Introns/Intergenic	-	-	TGCACTCATCTTTGGTTGGA	TATAGAGGGAGGGTGGAGGG	160	P	A	A	NA
PMS5441	Os_Chr11	6772088	6772101	(GA)7	(GA)7	(GA)7	(GA)7	(GA)7	(GA)7	(GA)7	(GA)7	(GA)7	(GA)6	(GA)7	-	Introns/Intergenic	-	-	TCTCCACAGGAGAGAGGAGC	GGGAATTATTTGAGCAAGGGA	188	A	A	A	NA
PMS5442	Os_Chr11	6775678	6775691	(CT)7	(CT)7	(CT)7	(CT)7	(CT)6	(CT)7	(CT)7	(CT)7	(CT)7	(CT)6	(CT)7	-	Introns/Intergenic	-	-	TATCCACAGCCATGTACCGA	CAACGGGAAAAGAAAGAACG	148	P	A	A	NA
PMS5443	Os_Chr11	6850430	6850456	(ATA)9	(ATA)8	(ATA)9	(ATA)9	(ATA)9	(ATA)9	(ATA)9	(ATA)9	(ATA)9	(ATA)9	(ATA)9	-	Introns/Intergenic	-	-	CGGCTTAGGAGCGTTTGTAG	AAGCACCTTCACAGCGATCT	157	P	A	A	NA
PMS5444	Os_Chr11	6858086	6858159	(TA)37	(TA)37	(TA)37	(TA)37	(TA)37	(TA)8	(TA)6	(TA)37	(TA)37	(TA)37	(TA)37	-	Introns/Intergenic	-	-	AGGTGTGCAGGCATTCTTCTTATTGG	GTGGTGGTGCGTCTGTGTCAGG	182	P	A	A	RM26296
PMS5445	Os_Chr11	6859651	6859690	(TA)20	(TA)20	(TA)20	(TA)20	(TA)20	(TA)20	(TA)11	(TA)20	(TA)20	(TA)7	(TA)20	-	Introns/Intergenic	-	-	GACTATGGGTGGCGCTGTGTACC	CCGCGCGGTAACTTCTTATTTCC	251	A	A	A	RM26297
PMS5446	Os_Chr11	6905486	6905503	(GGC)6	(GGC)5	(GGC)6	(GGC)6	(GGC)6	(GGC)6	(GGC)6	(GGC)6	(GGC)6	(GGC)6	(GGC)6	-	Introns/Intergenic	-	-	AGAGATCGAGATGCATGAAG	CACTGCCTAGGGCAAATC	155	P	A	A	NA

Polymorphic SSR (PMS) markers	Chromosomes	Start physical positions (bp)	End physical positions (bp)	Nipponbare	Kasalath	Nagina22	IR64	Pokkali	Bala	Tainung67	Azucena	Moroberekan	*Oryza rufipogon*	*Oryza nivara*	MSU gene locus IDs	Structural annotation	Functional annotation	SSR functional effects	Forward primers (5'-3')	Reverse primers (5'-3')	Amplified product size (bp)	Markers exhibiting polymorphism within *indica* rice	Transcrption factor genes-derived markers	Known cloned genes-derived markers	PMS markers corresponding with RM markers
PMS5447	Os_Chr11	6919609	6919628	(AT)10	(AT)10	(AT)10	(AT)10	(AT)10	(AT)7	(AT)10	(AT)10	(AT)10	(AT)10	(AT)10	-	Introns/Intergenic	-	-	GGTTTATCCACCGACATAGAACAAGC	CCTTTACTTACCGCGCACTCTAGC	194	P	A	A	RM26300
PMS5448	Os_Chr11	6961516	6961565	(AT)25	(AT)25	(AT)8	(AT)6	(AT)7	(AT)6	(AT)25	(AT)25	(AT)6	(AT)6	(AT)6	-	Introns/Intergenic	-	-	TTTTTCTACTCCATCAGTTTCA	TGTATTATTAGTACCCCCATT	314	P	A	A	NA
PMS5449	Os_Chr11	7102522	7102537	(CT)8	(CT)9	(CT)8	(CT)8	(CT)8	(CT)8	(CT)8	(CT)9	(CT)8	(CT)8	(CT)8	-	Introns/Intergenic	-	-	CGCTTTAGTTTTGCCACCTC	TTGCATGTTCGTGGAGTGAT	187	P	A	A	NA
PMS5450	Os_Chr11	7151512	7151531	(TC)10	(TC)8	(TC)10	(TC)10	(TC)10	(TC)10	(TC)10	(TC)10	(TC)10	(TC)10	(TC)8	-	Introns/Intergenic	-	-	CCGTTTCTTCTCGGTCCATTTCC	GTGAAGGCGGTGGTGGTTCG	180	P	A	A	RM26306
PMS5451	Os_Chr11	7194363	7194380	(CGC)6	(CGC)5	(CGC)6	(CGC)6	(CGC)6	(CGC)6	(CGC)6	(CGC)6	(CGC)5	(CGC)6	(CGC)6	LOC_Os11g12740	CDS	SP1	CODON_DELETION	ATGAGGACGAGGTAGGGGAT	TCGTCTTCAACACCATCCTG	148	P	A	P	NA
PMS5452	Os_Chr11	7250405	7250424	(AG)10	(AG)13	(AG)10	(AG)10	(AG)13	(AG)10	(AG)10	(AG)10	(AG)16	(AG)10	(AG)7	-	Introns/Intergenic	-	-	CATACCCAACAAGTAGGCATTGAAGC	GATCTTGGCCGTGGAGCTTTACC	257	P	A	A	RM26315
PMS5453	Os_Chr11	7254387	7254407	(GGA)7	(GGA)7	(GGA)7	(GGA)7	(GGA)7	(GGA)7	(GGA)7	(GGA)5	(GGA)5	(GGA)7	(GGA)7	LOC_Os11g12800	URR	glycerophosphoryl diester phosphodiesterase family protein, putative, expressed	-	TAGGGATTCAGGAATGGGAGAGG	TGACGAGAGAGTAGCAATGAGAAGG	286	A	A	A	RM26316
PMS5454	Os_Chr11	7317183	7317218	(TA)18	(TA)18	(TA)18	(TA)18	(TA)18	(TA)18	(TA)18	(TA)9	(TA)9	(TA)18	(TA)18	-	Introns/Intergenic	-	-	CCACATACGGTGGTCTGACTGG	TAGATCGTGTTTAATGGACGGTCAGG	271	A	A	A	RM26319
PMS5455	Os_Chr11	7459934	7459954	(GCG)7	(GCG)7	(GCG)7	(GCG)7	(GCG)7	(GCG)7	(GCG)7	(GCG)7	(GCG)7	(GCG)7	(GCG)6	-	Introns/Intergenic	-	-	ACACGCCTTGACGAGGTGTCTCC	CGCCGTGTTCACGTTACAGAGG	148	A	A	A	RM26323
PMS5456	Os_Chr11	7751529	7751542	(AT)7	(AT)7	(AT)7	(AT)7	(AT)7	(AT)6	(AT)7	(AT)7	(AT)7	(AT)7	(AT)7	-	Introns/Intergenic	-	-	CCAATGTAGGCCCAACAGAT	GACGTCGGACTCCTAACTGC	237	P	A	A	NA
PMS5457	Os_Chr11	7978674	7978694	(CGT)7	(CGT)7	(CGT)7	(CGT)7	(CGT)7	(CGT)8	(CGT)7	(CGT)7	(CGT)7	(CGT)7	(CGT)7	LOC_Os11g14250	CDS	expressed protein	CODON_CHANGE_PLUS_CODON_INSERTION	CTTTCCCGCCATATAAGGGT	CCTCTTCTCCGCCTTGTCT	275	P	A	A	NA
PMS5458	Os_Chr11	8085215	8085229	(CCG)5	(CCG)7	(CCG)5	(CCG)5	(CCG)5	(CCG)7	(CCG)5	(CCG)5	(CCG)5	(CCG)5	(CCG)5	LOC_Os11g14400	DRR	polygalacturonase, putative, expressed	-	GGTTAGACAGCTCGGTGGAT	TCTCTGCTGTGTGTTCAGGG	261	P	A	A	NA
PMS5459	Os_Chr11	8105338	8105367	(GA)15	(GA)15	(GA)15	(GA)15	(GA)8	(GA)12	(GA)15	(GA)15	(GA)15	(GA)15	(GA)15	LOC_Os11g14430	URR	RBS1	-	GAAAGAGGAGGAAGAGCTAGAGG	CCATATGTACGGAGTGTGTTTACC	454	P	A	P	RM3701

Polymorphic SSR (PMS) markers	Chromosomes	Start physical positions (bp)	End physical positions (bp)	Nipponbare	Kasalath	Nagina22	IR64	Pokkali	Bala	Tainung67	Azucena	Moroberekan	*Oryza rufipogon*	*Oryza nivara*	MSU gene locus IDs	Structural annotation	Functional annotation	SSR functional effects	Forward primers (5'-3')	Reverse primers (5'-3')	Amplified product size (bp)	Markers exhibiting polymorphism within *indica* rice	Transcrption factor genes-derived markers	Known cloned genes-derived markers	PMS markers corresponding with RM markers
PMS5460	Os_Chr11	8210080	8210097	(TC)9	(TC)9	(TC)9	(TC)9	(TC)9	(TC)9	(TC)7	(TC)9	(TC)9	(TC)9	(TC)9	-	Introns/Intergenic	-	-	CCACTATCCCCACTCCTCCT	CGGTGGAAAGAGGAAAAGGT	197	A	A	A	NA
PMS5461	Os_Chr11	8237571	8237620	(TA)25	(TA)25	(TA)10	(TA)25	(TA)25	(TA)25	(TA)25	(TA)6	(TA)25	(TA)25	(TA)25	-	Introns/Intergenic	-	-	TTTCTGGGTTGATGTGTTGC	TGTTGCTGTCCATTTCCTACC	269	P	A	A	NA
PMS5462	Os_Chr11	8258846	8258859	(CT)7	(CT)6	(CT)7	(CT)7	(CT)7	(CT)7	(CT)7	(CT)7	(CT)7	(CT)7	(CT)7	-	Introns/Intergenic	-	-	AAAATCCCCTTTTGGCTTTG	GATCGTTTCCCCTCAATCAA	250	P	A	A	NA
PMS5463	Os_Chr11	8270568	8270627	(TA)30	(TA)30	(TA)30	(TA)30	(TA)30	(TA)9	(TA)13	(TA)9	(TA)10	(TA)30	(TA)12	-	Introns/Intergenic	-	-	GTACATCAGCTTGGGCGATT	GAGCCACGCATTGAGTATGA	280	P	A	A	NA
PMS5464	Os_Chr11	8293074	8293087	(TA)7	(TA)7	(TA)7	(TA)9	(TA)9	(TA)7	(TA)7	(TA)7	(TA)7	(TA)7	(TA)7	-	Introns/Intergenic	-	-	CGGTTGGAAAACTGGGACTA	TGTGTTTTATACCAAGCCCG	220	P	A	A	NA
PMS5465	Os_Chr11	8301323	8301342	(TA)10	(TA)10	(TA)10	(TA)10	(TA)10	(TA)10	(TA)6	(TA)10	(TA)10	(TA)10	(TA)10	-	Introns/Intergenic	-	-	CCCGAGTTTTCGATAACGGT	TATGTGAGTGCGGTTTAGCG	219	A	A	A	NA
PMS5466	Os_Chr11	8367549	8367566	(TGG)6	(TGG)6	(TGG)6	(TGG)6	(TGG)6	(TGG)6	(TGG)6	(TGG)6	(TGG)6	(TGG)6	(TGG)5	-	Introns/Intergenic	-	-	CTGCCGTGGTGAGATTTCTT	CGTTAACCCTGCATCGAGTT	216	A	A	A	NA
PMS5467	Os_Chr11	8370788	8370803	(GA)8	(GA)8	(GA)8	(GA)8	(GA)8	(GA)8	(GA)6	(GA)8	(GA)8	(GA)8	(GA)8	-	Introns/Intergenic	-	-	AAGGGAGGGAGAAGAGGACA	TGTTTTTAAGAACCGGCACC	154	A	A	A	NA
PMS5468	Os_Chr11	8574327	8574350	(AT)12	(AT)12	(AT)12	(AT)12	(AT)12	(AT)10	(AT)12	(AT)12	(AT)12	(AT)12	(AT)12	-	Introns/Intergenic	-	-	TACTTCCGAGGCAGAGACAAAGC	CCAAAGATCAAGGCAAAGATGAGG	504	P	A	A	RM26385
PMS5469	Os_Chr11	8835514	8835527	(GA)7	(GA)7	(GA)7	(GA)6	(GA)7	(GA)6	(GA)7	(GA)7	(GA)7	(GA)6	(GA)6	-	Introns/Intergenic	-	-	CTCCTCTGTTGCCGCTACTC	CCCACATGTCATATCCCCTC	238	P	A	A	NA
PMS5470	Os_Chr11	9059344	9059365	(AG)11	(AG)11	(AG)11	(AG)11	(AG)11	(AG)11	(AG)11	(AG)11	(AG)11	(AG)11	(AG)12	LOC_Os11g16390	URR	expressed protein	-	AAGAGGAGGAGGAGAGAGAAGC	CAGAGAGTTGTACACATTCTGAGC	199	A	A	A	RM26406
PMS5471	Os_Chr11	9252743	9252762	(AT)10	(AT)10	(AT)10	(AT)10	(AT)10	(AT)10	(AT)10	(AT)10	(AT)6	(AT)10	(AT)6	-	Introns/Intergenic	-	-	AGATGCCACATCATCATCCA	AAAAAGGAGAAAGATAATTTGAACCA	267	A	A	A	NA
PMS5472	Os_Chr11	9380072	9380089	(TA)9	(TA)9	(TA)9	(TA)9	(TA)6	(TA)9	(TA)9	(TA)9	(TA)9	(TA)9	(TA)9	-	Introns/Intergenic	-	-	CGACGGAAATTTCTTTCATTTT	CAAAAGTCAAAAGGGCCAAA	201	P	A	A	NA

Polymorphic SSR (PMS) markers	Chromosomes	Start physical positions (bp)	End physical positions (bp)	Nipponbare	Kasalath	Nagina22	IR64	Pokkali	Bala	Tainung67	Azucena	Moroberekan	*Oryza rufipogon*	*Oryza nivara*	MSU gene locus IDs	Structural annotation	Functional annotation	SSR functional effects	Forward primers (5'-3')	Reverse primers (5'-3')	Amplified product size (bp)	Markers exhibiting polymorphism within *indica* rice	Transcrption factor genes-derived markers	Known cloned genes-derived markers	PMS markers corresponding with RM markers
PMS5473	Os_Chr11	9521671	9521718	(TA)24	(TA)24	(TA)24	(TA)24	(TA)24	(TA)20	(TA)6	(TA)24	(TA)24	(TA)24	(TA)24	-	Introns/Intergenic	-	-	ATTCCCTTGTTATG GCCAGTTTGC	CACGACTGACCATCG TGAGAAACC	257	P	A	A	RM26429
PMS5474	Os_Chr11	9567707	9567718	(AT)6	(AT)6	(AT)6	(AT)6	(AT)6	(AT)6	(AT)6	(AT)6	(AT)6	(AT)8	(AT)6	-	Introns/Intergenic	-	-	TTGCTGCATGGTTG ATTAT	CTATAGCTACCGAGC AAATGA	355	A	A	A	NA
PMS5475	Os_Chr11	9708144	9708185	(AT)21	(AT)21	(AT)21	(AT)12	(AT)21	(AT)21	(AT)21	(AT)21	(AT)8	(AT)21	(AT)21	-	Introns/Intergenic	-	-	CCATTGAAACGTGA ACGAAA	ACGACGTCTTTTCTG CAACA	193	P	A	A	NA
PMS5476	Os_Chr11	9715521	9715544	(AT)12	(AT)12	(AT)12	(AT)12	(AT)12	(AT)8	(AT)12	(AT)12	(AT)12	(AT)12	(AT)12	-	Introns/Intergenic	-	-	GCATGAGGGATAAC TCATACACAAGG	GGGTACATACTGCGT GACTCTGC	484	P	A	A	RM26437
PMS5477	Os_Chr11	9735306	9735329	(CTC)8	(CTC)8	(CTC)8	(CTC)8	(CTC)8	(CTC)8	(CTC)8	(CTC)8	(CTC)8	(CTC)7	(CTC)8	-	Introns/Intergenic	-	-	AAAATTTCAACAACT CGCCG	AAGTGGATGGTGGC CAAG	179	A	A	A	NA
PMS5478	Os_Chr11	9748403	9748420	(CGG)6	(CGG)6	(CGG)6	(CGG)6	(CGG)6	(CGG)5	(CGG)6	(CGG)6	(CGG)6	(CGG)6	(CGG)6	LOC_Os11g17480	URR	retrotransposon protein, putative, Ty1-copia subclass, expressed	-	GTCTTCGTTGCTGC TCTGCT	ATAGATGTCGGAGG CCACG	238	P	A	A	NA
PMS5479	Os_Chr11	9795821	9795836	(AT)8	(AT)6	(AT)6	(AT)6	(AT)6	(AT)8	(AT)8	(AT)8	(AT)8	(AT)8	(AT)8	-	Introns/Intergenic	-	-	TGCACATTCCTCCT GGTAAA	CTCCGGAGGTTATCA AACCC	272	P	A	A	NA
PMS5480	Os_Chr11	9961819	9961842	(TTG)8	(TTG)8	(TTG)6	(TTG)8	(TTG)8	(TTG)8	(TTG)8	(TTG)8	(TTG)8	(TTG)8	(TTG)8	-	Introns/Intergenic	-	-	CTCCACCCTAAGCT TCTCCTGTGG	GCGCATCCATCCCTC TTCACC	184	P	A	A	RM5731
PMS5481	Os_Chr11	9991955	9991972	(CT)9	(CT)9	(CT)9	(CT)9	(CT)9	(CT)8	(CT)9	(CT)9	(CT)9	(CT)9	(CT)9	-	Introns/Intergenic	-	-	ACTTCTTGGGTGAG AGCCG	ACAGTGTGGCGTCA GTGG	264	P	A	A	NA
PMS5482	Os_Chr11	10051796	10051813	(CT)9	(CT)8	(CT)9	(CT)9	(CT)9	(CT)8	(CT)9	(CT)9	(CT)9	(CT)9	(CT)9	-	Introns/Intergenic	-	-	TTTGACTTGAGCCT GCACAC	AGCTGCCTACTCGAG TGCTT	164	P	A	A	NA
PMS5483	Os_Chr11	10052653	10052664	(GA)6	(GA)7	(GA)7	(GA)7	(GA)9	(GA)10	(GA)6	(GA)6	(GA)6	(GA)6	(GA)6	LOC_Os11g17910	CDS	expressed protein	FRAME_SHIFT	CAGCCACTCGAGCT CACTC	CATCATGGCAATCCA GTCAG	198	P	A	A	NA
PMS5484	Os_Chr11	10057104	10057125	(AT)11	(AT)9	(AT)11	(AT)11	(AT)11	(AT)9	(AT)11	(AT)11	(AT)11	(AT)11	(AT)9	-	Introns/Intergenic	-	-	TACTCCCTCCGGTT AACAATGC	CTGACGTGTCAGACT GTTCTCG	283	P	A	A	RM26456
PMS5485	Os_Chr11	10087120	10087159	(TA)20	(TA)18	(TA)20	(TA)6	(TA)20	(TA)12	(TA)7	(TA)6	(TA)6	(TA)20	(TA)20	-	Introns/Intergenic	-	-	CTTAGGGTCGGGCC TGAGTAGC	CATGGTATCTGTCCG TTCATCACC	396	P	A	A	RM26457

Polymorphic SSR (PMS) markers	Chromosomes	Start physical positions (bp)	End physical positions (bp)	Nipponbare	Kasalath	Nagina22	IR64	Pokkali	Bala	Tainung67	Azucena	Moroberekan	*Oryza rufipogon*	*Oryza nivara*	MSU gene locus IDs	Structural annotation	Functional annotation	SSR functional effects	Forward primers (5'-3')	Reverse primers (5'-3')	Amplified product size (bp)	Markers exhibiting polymorphism within *indica* rice	Transcrption factor genes-derived markers	Known cloned genes-derived markers	PMS markers corresponding with RM markers
PMS5486	Os_Chr11	10094786	10094799	(TA)7	(TA)6	(TA)7	(TA)7	(TA)7	(TA)7	(TA)7	(TA)7	(TA)7	(TA)7	(TA)7	-	Introns/Intergenic	-	-	CACGGGCGAAAAATTACCTA	GCTGACCGGAAATTTGGATA	219	P	A	A	NA
PMS5487	Os_Chr11	10133543	10133560	(AG)9	(AG)6	(AG)6	(AG)6	(AG)6	(AG)6	(AG)9	(AG)9	(AG)9	(AG)9	(AG)6	LOC_Os11g18044	URR	peptide transporter PTR2, putative, expressed	-	TTGGCTGCAACTTCAACAAC	GGATGCTCTGACACCTCCAT	163	A	A	A	NA
PMS5488	Os_Chr11	10151208	10151225	(CGG)6	(CGG)7	(CGG)6	(CGG)6	(CGG)6	(CGG)6	(CGG)6	(CGG)6	(CGG)6	(CGG)6	(CGG)6	-	Introns/Intergenic	-	-	GATCTCGACGACAGCCACTC	TCAGATCCAACACCTCCTCC	146	P	A	A	NA
PMS5489	Os_Chr11	10178558	10178643	(TA)43	(TA)43	(TA)43	(TA)43	(TA)43	(TA)43	(TA)7	(TA)43	(TA)7	(TA)43	(TA)43	-	Introns/Intergenic	-	-	TAACCTATATCCCGCAGCAAAGC	TATGGGATCTGTACCCGTCATGC	124	A	A	A	RM26459
PMS5490	Os_Chr11	10373183	10373212	(TA)15	(TA)15	(TA)15	(TA)15	(TA)15	(TA)15	(TA)15	(TA)15	(TA)15	(TA)14	(TA)15	-	Introns/Intergenic	-	-	TCCGCAAGAAACATGAAAAC	CAATTTGAATCATGGAGCCC	164	A	A	A	NA
PMS5491	Os_Chr11	10381158	10381175	(AG)9	(AG)9	(AG)9	(AG)9	(AG)10	(AG)9	(AG)9	(AG)9	(AG)9	(AG)9	(AG)9	-	Introns/Intergenic	-	-	CTATTGGCATGTAATGGGGG	CTGGTTCTTCGCCAGCTATC	226	P	A	A	NA
PMS5492	Os_Chr11	10714399	10714498	(AT)50	(AT)50	(AT)50	(AT)9	(AT)12	(AT)50	(AT)50	(AT)50	(AT)50	(AT)9	(AT)50	-	Introns/Intergenic	-	-	CCATGGAGCACCAAACAAAT	TACCCGCTTGAGAAAAGGAA	205	P	A	A	NA
PMS5493	Os_Chr11	10774417	10774434	(TAC)6	(TAC)6	(TAC)6	(TAC)6	(TAC)6	(TAC)6	(TAC)6	(TAC)6	(TAC)6	(TAC)6	(TAC)5	-	Introns/Intergenic	-	-	GTGGAAATGCAGGTAGGCAT	GCCTCTCTCTTTGGTCGTTG	256	A	A	A	NA
PMS5494	Os_Chr11	10813059	10813076	(GGC)6	(GGC)5	(GGC)6	(GGC)6	(GGC)6	(GGC)5	(GGC)6	(GGC)6	(GGC)6	(GGC)6	(GGC)6	LOC_Os11g18970	CDS	expressed protein	CODON_DELETION	TCTTCCCTTCTTCCTCCTCC	AATAATCCAGTGCGCCTGTC	187	P	A	A	NA
PMS5495	Os_Chr11	10818399	10818425	(AAG)9	(AAG)8	(AAG)9	(AAG)9	(AAG)9	(AAG)8	(AAG)9	(AAG)9	(AAG)9	(AAG)9	(AAG)9	LOC_Os11g18980	CDS	expressed protein	CODON_DELETION	AAGGCGTAGCCAGGTGAAGTTGG	GGAACCTGGAATTCTGCATCAGC	152	P	A	A	RM26490
PMS5496	Os_Chr11	10825035	10825048	(GA)7	(GA)7	(GA)7	(GA)7	(GA)7	(GA)7	(GA)6	(GA)7	(GA)7	(GA)7	(GA)7	-	Introns/Intergenic	-	-	ATTGATTCGCTTGCCGTTAC	TAAAATCTCAAGGGCCCACA	263	A	A	A	NA
PMS5497	Os_Chr11	10863203	10863218	(CT)8	(CT)8	(CT)8	(CT)8	(CT)8	(CT)8	(CT)8	(CT)8	(CT)8	(CT)9	(CT)8	-	Introns/Intergenic	-	-	CCAATACAAATGCAGGGCTT	CATCTGGGGAAGAGTTGCTG	183	A	A	A	NA
PMS5498	Os_Chr11	11052322	11052335	(GA)7	(GA)7	(GA)7	(GA)7	(GA)7	(GA)12	(GA)7	(GA)7	(GA)7	(GA)7	(GA)7	-	Introns/Intergenic	-	-	GTTGGCCACGTAAGCAACTT	CTCATCTCATCTCCACCCGT	100	P	A	A	NA

Polymorphic SSR (PMS) markers	Chromosomes	Start physical positions (bp)	End physical positions (bp)	Nipponbare	Kasalath	Nagina22	IR64	Pokkali	Bala	Tainung67	Azucena	Moroberekan	*Oryza rufipogon*	*Oryza nivara*	MSU gene locus IDs	Structural annotation	Functional annotation	SSR functional effects	Forward primers (5'-3')	Reverse primers (5'-3')	Amplified product size (bp)	Markers exhibiting polymorphism within *indica* rice	Transcrption factor genes-derived markers	Known cloned genes-derived markers	PMS markers corresponding with RM markers
PMS5499	Os_Chr11	11101545	11101562	(GCT)6	(GCT)6	(GCT)6	(GCT)6	(GCT)6	(GCT)6	(GCT)6	(GCT)6	(GCT)6	(GCT)5	(GCT)6	-	Introns/Intergenic	-	-	TTCAGCTATCGGGGTAGTGG	TTGGCCACGGTAGTTTTAGG	238	A	A	A	NA
PMS5500	Os_Chr11	11109029	11109066	(GA)19	(GA)19	(GA)19	(GA)19	(GA)19	(GA)19	(GA)18	(GA)19	(GA)19	(GA)19	(GA)19	-	Introns/Intergenic	-	-	GAGCAAGAGGATGAGGGTGATTGG	CTGTCTCATGTGCCTCCACTCG	151	A	A	A	RM26501
PMS5501	Os_Chr11	11264944	11264985	(TA)21	(TA)21	(TA)21	(TA)21	(TA)21	(TA)20	(TA)21	(TA)18	(TA)21	(TA)21	(TA)21	-	Introns/Intergenic	-	-	TCAATTTCAACAATGGTGCC	GAACAAGTCCTTGATCCCGA	264	P	A	A	NA
PMS5502	Os_Chr11	11306449	11306472	(TGA)8	(TGA)8	(TGA)8	(TGA)8	(TGA)8	(TGA)7	(TGA)8	(TGA)8	(TGA)8	(TGA)8	(TGA)8	LOC_Os11g19610	CDS	retrotransposon protein, putative, unclassified, expressed	CODON_DELETION	GTTGCCGGTGAAGGTCTATCC	TCGGTGTCACCCTCATCACC	95	P	A	A	RM26510
PMS5503	Os_Chr11	11544341	11544410	(TA)35	(TA)35	(TA)9	(TA)35	(TA)35	(TA)35	(TA)35	(TA)35	(TA)35	(TA)18	(TA)35	-	Introns/Intergenic	-	-	TTAGTAGGCGGGTTTGACAGG	TGTAGGGATAGTCCCATATCACAGG	478	P	A	A	RM26514
PMS5504	Os_Chr11	11587352	11587437	(TA)43	(TA)43	(TA)43	(TA)43	(TA)43	(TA)11	(TA)43	(TA)43	(TA)43	(TA)43	(TA)43	-	Introns/Intergenic	-	-	ATCATTGGTCTGTGTGCCAA	CCATCAAAAGTAGATGGAGGGA	195	P	A	A	NA
PMS5505	Os_Chr11	11632052	11632065	(CT)7	(CT)7	(CT)7	(CT)7	(CT)7	(CT)6	(CT)7	(CT)7	(CT)7	(CT)6	(CT)6	-	Introns/Intergenic	-	-	TAGCCCAAAGTCTTCCTCCA	CTGGGCAGAGAGGAAGGAG	121	P	A	A	NA
PMS5506	Os_Chr11	11658008	11658025	(GCG)6	(GCG)5	(GCG)6	(GCG)6	(GCG)6	(GCG)5	(GCG)6	(GCG)6	(GCG)6	(GCG)6	(GCG)5	-	Introns/Intergenic	-	-	GGCACTGTAGCATGGTGATG	ACACGCAAGGCCTACTGATT	231	P	A	A	NA
PMS5507	Os_Chr11	11763598	11763667	(AT)35	(AT)35	(AT)15	(AT)35	(AT)35	(AT)35	(AT)35	(AT)35	(AT)35	(AT)10	(AT)35	-	Introns/Intergenic	-	-	ACAAGACGAGAACAGCAAAGTGG	TTGAATCTTGGAGCCCATACTCC	276	P	A	A	RM26526
PMS5508	Os_Chr11	11869871	11869904	(TA)17	(TA)17	(TA)17	(TA)17	(TA)17	(TA)17	(TA)17	(TA)17	(TA)17	(TA)13	(TA)17	-	Introns/Intergenic	-	-	TCAATTTCAACAATGGTGCC	ATGTGCTTGTTGTCGTCGAG	215	A	A	A	NA
PMS5509	Os_Chr11	12344093	12344120	(AT)14	(AT)14	(AT)14	(AT)14	(AT)8	(AT)14	(AT)6	(AT)14	(AT)14	(AT)14	(AT)14	-	Introns/Intergenic	-	-	GCAAACATTATACCCGCACC	AAATCAAGAGTCCGGAAGCC	239	P	A	A	NA
PMS5510	Os_Chr11	12464762	12464779	(CCG)6	(CCG)6	(CCG)6	(CCG)6	(CCG)6	(CCG)6	(CCG)6	(CCG)6	(CCG)6	(CCG)6	(CCG)5	-	Introns/Intergenic	-	-	CTTAAGCTAGCTGCTGCACG	CACAACATGGTGTACGGGAG	198	A	A	A	NA
PMS5511	Os_Chr11	12593320	12593333	(GC)7	(GC)6	(GC)7	(GC)7	(GC)7	(GC)7	(GC)7	(GC)7	(GC)7	(GC)7	(GC)7	-	Introns/Intergenic	-	-	TAGCTTCCCATTGTTCGAGG	GCACTCCCCCTCCTCTATTC	252	P	A	A	NA

Polymorphic SSR (PMS) markers	Chromosomes	Start physical positions (bp)	End physical positions (bp)	Nipponbare	Kasalath	Nagina22	IR64	Pokkali	Bala	Tainung67	Azucena	Moroberekan	*Oryza rufipogon*	*Oryza nivara*	MSU gene locus IDs	Structural annotation	Functional annotation	SSR functional effects	Forward primers (5'-3')	Reverse primers (5'-3')	Amplified product size (bp)	Markers exhibiting polymorphism within *indica* rice	Transcrption factor genes-derived markers	Known cloned genes-derived markers	PMS markers corresponding with RM markers
PMS5512	Os_Chr11	12623803	12623817	(TGC)5	(TGC)6	(TGC)6	(TGC)6	(TGC)6	(TGC)6	(TGC)5	(TGC)5	(TGC)5	(TGC)6	(TGC)5	-	Introns/Intergenic	-	-	CTCGAAGCAATTGAAGCACA	TCCGCTACACCAATCCCTAC	224	A	A	A	NA
PMS5513	Os_Chr11	12624561	12624578	(TGG)6	(TGG)6	(TGG)7	(TGG)6	(TGG)6	(TGG)7	(TGG)6	(TGG)6	(TGG)6	(TGG)7	(TGG)6	LOC_Os11g21980	CDS	retrotransposon protein, putative, Ty3-gypsy subclass, expressed	CODON_INSERTION	ATGTGATGGAGGAAAGCAGC	TCTCTCTGTTTGCCGGTTCT	223	P	A	A	NA
PMS5514	Os_Chr11	12862127	12862168	(AT)21	(AT)21	(AT)21	(AT)14	(AT)21	(AT)8	(AT)21	(AT)12	(AT)21	(AT)21	(AT)21	-	Introns/Intergenic	-	-	CTACGATCCGATTACACTGTT	ATCACCTCAAAGCATTATGAA	369	P	A	A	NA
PMS5515	Os_Chr11	13034961	13034974	(TC)7	(TC)7	(TC)7	(TC)6	(TC)6	(TC)7	(TC)7	(TC)7	(TC)7	(TC)7	(TC)7	-	Introns/Intergenic	-	-	AATCCTCTTTTGGCTTTGGG	GAGGACGACGAGTCCGATAG	149	P	A	A	NA
PMS5516	Os_Chr11	13165478	13165489	(TC)6	(TC)7	(TC)6	(TC)6	(TC)6	(TC)6	(TC)6	(TC)6	(TC)6	(TC)6	(TC)7	LOC_Os11g22890	CDS	expressed protein	FRAME_SHIFT	CCAAGCTCTCATCTGCCCTA	AGCTGACGATGGTGACCG	260	P	A	A	NA
PMS5517	Os_Chr11	13243897	13243920	(TA)12	(TA)12	(TA)12	(TA)12	(TA)12	(TA)8	(TA)6	(TA)12	(TA)8	(TA)12	(TA)9	-	Introns/Intergenic	-	-	TTACTCGGACTATGGGAGTGAAGG	TGGAATGCTCTCTGTAGGTTTCG	434	P	A	A	RM26563
PMS5518	Os_Chr11	13262042	13262057	(AG)8	(AG)8	(AG)8	(AG)8	(AG)8	(AG)8	(AG)8	(AG)8	(AG)8	(AG)6	(AG)6	-	Introns/Intergenic	-	-	CCGTAATCCCAAACCCTAGC	TTAAATGTGAAATGGCACGG	122	A	A	A	NA
PMS5519	Os_Chr11	13869069	13869101	(AGG)11	(AGG)11	(AGG)11	(AGG)11	(AGG)11	(AGG)11	(AGG)11	(AGG)10	(AGG)11	(AGG)11	(AGG)11	LOC_Os11g24340	CDS	OsSCP53 - Putative Serine Carboxypeptidase homologue, expressed	CODON_DELETION	GCGGACACACCAGAGAATAAGC	GTGCTGTCCTGTCCTTGAATCC	82	A	A	A	RM6091
PMS5520	Os_Chr11	13983070	13983105	(AT)18	(AT)18	(AT)6	(AT)9	(AT)18	(AT)18	(AT)18	(AT)18	(AT)18	(AT)18	(AT)6	-	Introns/Intergenic	-	-	ATCGGTTGGAGCTCTTGTTGG	TTAGGGTTCATGCTGGAAATGC	62	P	A	A	RM26584
PMS5521	Os_Chr11	14136895	14136906	(AG)6	(AG)7	(AG)6	(AG)7	(AG)7	(AG)6	(AG)6	(AG)6	(AG)6	(AG)6	(AG)6	-	Introns/Intergenic	-	-	CCACCGCCACTTACTAGCC	TGATCCCGGTTTTGGTCTAC	183	P	A	A	NA
PMS5522	Os_Chr11	14181137	14181174	(TA)19	(TA)19	(TA)19	(TA)19	(TA)9	(TA)19	(TA)19	(TA)19	(TA)6	(TA)19	(TA)19	-	Introns/Intergenic	-	-	GCTAGCTAGGACATATAATGGATTG	CCATGGAGCACCAAACAAAT	265	P	A	A	NA
PMS5523	Os_Chr11	14241301	14241320	(AT)10	(AT)10	(AT)10	(AT)6	(AT)10	(AT)10	(AT)10	(AT)10	(AT)10	(AT)10	(AT)8	-	Introns/Intergenic	-	-	GCTCCAATTCCTTCTAGGGC	AATTTTAACAATGGTGCCCG	180	P	A	A	NA
PMS5524	Os_Chr11	14354350	14354365	(GA)8	(GA)8	(GA)8	(GA)8	(GA)8	(GA)8	(GA)8	(GA)8	(GA)8	(GA)8	(GA)7	-	Introns/Intergenic	-	-	AGGCGAAGTCGTGTTTGG	TGATCGATCTCTCTCGGCTT	259	A	A	A	NA

Polymorphic SSR (PMS) markers	Chromosomes	Start physical positions (bp)	End physical positions (bp)	Nipponbare	Kasalath	Nagina22	IR64	Pokkali	Bala	Tainung67	Azucena	Moroberekan	*Oryza rufipogon*	*Oryza nivara*	MSU gene locus IDs	Structural annotation	Functional annotation	SSR functional effects	Forward primers (5'-3')	Reverse primers (5'-3')	Amplified product size (bp)	Markers exhibiting polymorphism within *indica* rice	Transcrption factor genes-derived markers	Known cloned genes-derived markers	PMS markers corresponding with RM markers
PMS5525	Os_Chr11	14574915	14574934	(CT)10	(CT)10	(CT)10	(CT)10	(CT)10	(CT)10	(CT)10	(CT)10	(CT)10	(CT)10	(CT)7	-	Introns/Intergenic	-	-	TGACCTTTGAAGCCAAACGACTGC	CAGCACATGGACGTTGGAGAGG	461	A	A	A	RM26615
PMS5526	Os_Chr11	14605382	14605393	(CT)6	(CT)6	(CT)6	(CT)7	(CT)7	(CT)6	(CT)6	(CT)6	(CT)6	(CT)7	(CT)6	-	Introns/Intergenic	-	-	AAGGAAAAATATGTCCCACCA	TATATATAGCGGCGTGCGTG	201	P	A	A	NA
PMS5527	Os_Chr11	14675400	14675489	(AT)45	(AT)45	(AT)45	(AT)7	(AT)45	(AT)45	(AT)8	(AT)45	(AT)45	(AT)45	(AT)45	-	Introns/Intergenic	-	-	GTGGAGGACGGTGATGGAGAAGC	ATTTCTTGACCGCCGCTGATTCC	228	P	A	A	RM26616
PMS5528	Os_Chr11	14972423	14972452	(TA)15	(TA)14	(TA)15	(TA)15	(TA)15	(TA)14	(TA)15	(TA)15	(TA)12	(TA)15	(TA)15	-	Introns/Intergenic	-	-	GTATTGTACTTGCAACGGTGTTGG	CGTTTAGCGGTTTGGGAAGC	416	P	A	A	RM26624
PMS5529	Os_Chr11	15102415	15102435	(GGC)7	(GGC)7	(GGC)7	(GGC)7	(GGC)7	(GGC)7	(GGC)7	(GGC)7	(GGC)7	(GGC)7	(GGC)5	-	Introns/Intergenic	-	-	GACGTGTTCGTTCTCGTTCA	CTCGATCTCTCTCTCCGTCG	273	A	A	A	NA
PMS5530	Os_Chr11	15166360	15166387	(ATCT)7	(ATCT)7	(ATCT)5	(ATCT)7	(ATCT)7	(ATCT)5	(ATCT)7	(ATCT)6	(ATCT)7	(ATCT)7	(ATCT)7	-	Introns/Intergenic	-	-	TCAATCCGTCCATAAATCCA	GGTGGGAAAAGTGAATGGTG	182	P	A	A	NA
PMS5531	Os_Chr11	15180429	15180446	(GA)9	(GA)9	(GA)9	(GA)9	(GA)9	(GA)9	(GA)9	(GA)9	(GA)9	(GA)6	(GA)9	-	Introns/Intergenic	-	-	ACATGTCGGTGGCTAGAGGA	TAAGTTGTTGGGCCGTTTTT	131	A	A	A	NA
PMS5532	Os_Chr11	15183386	15183403	(GCG)6	(GCG)6	(GCG)6	(GCG)6	(GCG)6	(GCG)5	(GCG)6	(GCG)6	(GCG)6	(GCG)6	(GCG)6	-	Introns/Intergenic	-	-	GAAACAATGGATGGCTAGGG	TTTTTCATTTTGCAATCGCC	266	P	A	A	NA
PMS5533	Os_Chr11	15235804	15235849	(TA)23	(TA)23	(TA)23	(TA)23	(TA)7	(TA)23	(TA)23	(TA)23	(TA)6	(TA)23	(TA)16	-	Introns/Intergenic	-	-	CGGGTGCAAATAATCCAATTCC	CGGAAACCCTGATACCATATTCG	285	P	A	A	RM26635
PMS5534	Os_Chr11	15256593	15256608	(GA)8	(GA)8	(GA)8	(GA)8	(GA)8	(GA)8	(GA)8	(GA)8	(GA)8	(GA)8	(GA)9	-	Introns/Intergenic	-	-	GTGCGGTGGAGAAGAGAAAG	GAGGAGCGGATAAGGGAGAG	116	A	A	A	NA
PMS5535	Os_Chr11	15413590	15413607	(GA)9	(GA)9	(GA)9	(GA)9	(GA)9	(GA)6	(GA)9	(GA)9	(GA)9	(GA)9	(GA)9	-	Introns/Intergenic	-	-	CCGACTCGCGAGAGAGAGTA	AACCCGAAAATCAGACATCG	226	P	A	A	NA
PMS5536	Os_Chr11	15423307	15423320	(AG)7	(AG)8	(AG)7	(AG)8	(AG)7	(AG)8	(AG)7	(AG)7	(AG)7	(AG)7	(AG)7	-	Introns/Intergenic	-	-	CTCCATTCGATGCGAAAAAT	TCTACACCAACAAGCTCCCC	142	P	A	A	NA
PMS5537	Os_Chr11	15445917	15445934	(TG)9	(TG)10	(TG)9	(TG)9	(TG)9	(TG)10	(TG)9	(TG)9	(TG)9	(TG)10	(TG)10	LOC_Os11g26880	URR	RALFL14 - Rapid ALkalinization Factor RALF family protein precursor, expressed	-	GGCTCTGGAGTCTGGATCTG	GAAAGGACACAATGGCAGGT	166	P	A	A	NA

Polymorphic SSR (PMS) markers	Chromosomes	Start physical positions (bp)	End physical positions (bp)	Nipponbare	Kasalath	Nagina22	IR64	Pokkali	Bala	Tainung67	Azucena	Moroberekan	*Oryza rufipogon*	*Oryza nivara*	MSU gene locus IDs	Structural annotation	Functional annotation	SSR functional effects	Forward primers (5'-3')	Reverse primers (5'-3')	Amplified product size (bp)	Markers exhibiting polymorphism within *indica* rice	Transcrption factor genes-derived markers	Known cloned genes-derived markers	PMS markers corresponding with RM markers
PMS5538	Os_Chr11	15835115	15835160	(AT)23	(AT)23	(AT)9	(AT)10	(AT)12	(AT)9	(AT)23	(AT)23	(AT)6	(AT)11	(AT)6	-	Introns/Intergenic	-	-	GGATGAAGTTGCTAACCTTGTAGGC	AATTCCACAGTAGTCCCGATTGC	379	P	A	A	RM26661
PMS5539	Os_Chr11	15854061	15854074	(GT)7	(GT)7	(GT)7	(GT)7	(GT)7	(GT)7	(GT)7	(GT)7	(GT)7	(GT)7	(GT)6	-	Introns/Intergenic	-	-	TTTCAAGCAAGCCCTACGTT	TTTCTGCCACCTTGTAGCCT	240	A	A	A	NA
PMS5540	Os_Chr11	15899624	15899669	(TA)23	(TA)23	(TA)23	(TA)23	(TA)13	(TA)7	(TA)23	(TA)7	(TA)23	(TA)23	(TA)23	-	Introns/Intergenic	-	-	AAAATAGCCTTCACCCGTTG	ACGGAGATGCGAAATCAATC	274	P	A	A	NA
PMS5541	Os_Chr11	15905434	15905455	(AT)11	(AT)10	(AT)6	(AT)6	(AT)11	(AT)7	(AT)11	(AT)11	(AT)11	(AT)11	(AT)11	-	Introns/Intergenic	-	-	CACTGCATGAGCTTAAACTGTAGG	AAATTGGTTCTACCCGTTGC	476	P	A	A	RM26665
PMS5542	Os_Chr11	15936053	15936066	(AG)7	(AG)7	(AG)7	(AG)7	(AG)7	(AG)8	(AG)7	(AG)7	(AG)7	(AG)7	(AG)7	-	Introns/Intergenic	-	-	CAACACGCACGACAACAGAT	ACTCCTCTCGGGTGTTTCCT	197	P	A	A	NA
PMS5543	Os_Chr11	16087034	16087051	(CGG)6	(CGG)5	(CGG)6	(CGG)6	(CGG)6	(CGG)6	(CGG)6	(CGG)6	(CGG)6	(CGG)6	(CGG)5	-	Introns/Intergenic	-	-	TTCATGGGAGGAAGAGGATG	CAGTTCATGTACCTCGGCAA	229	P	A	A	NA
PMS5544	Os_Chr11	16414487	16414504	(AT)9	(AT)9	(AT)9	(AT)9	(AT)9	(AT)7	(AT)9	(AT)9	(AT)9	(AT)9	(AT)9	-	Introns/Intergenic	-	-	TACGCAATCCCTTCTATGCC	AATCTGTCATAAATAATGTCTTGCAT	235	P	A	A	NA
PMS5545	Os_Chr11	16496187	16496200	(TA)7	(TA)7	(TA)6	(TA)7	(TA)7	(TA)7	(TA)7	(TA)7	(TA)7	(TA)7	(TA)7	-	Introns/Intergenic	-	-	CAGAGGTGGGAAAATGCCTA	AACGTCGACAGGGTGGATAG	265	P	A	A	NA
PMS5546	Os_Chr11	16556749	16556796	(AT)24	(AT)24	(AT)24	(AT)24	(AT)24	(AT)16	(AT)24	(AT)24	(AT)24	(AT)24	(AT)24	-	Introns/Intergenic	-	-	AAGAAGAAGCGTACGCATGAA	AATACTATGGGCCCCACCTC	208	P	A	A	NA
PMS5547	Os_Chr11	16783071	16783091	(TTG)7	(TTG)7	(TTG)7	(TTG)7	(TTG)7	(TTG)7	(TTG)7	(TTG)7	(TTG)7	(TTG)7	(TTG)6	LOC_Os11g29000	CDS	pollen signalling protein with adenylyl cyclase activity, putative, expressed	CODON_DELETION	ACCAGAAAGCCGATGATAGGG	ATCAGCAACTGCAGCAACAGC	94	A	A	A	RM26699
PMS5548	Os_Chr11	16834381	16834402	(AT)11	(AT)11	(AT)11	(AT)11	(AT)11	(AT)12	(AT)11	(AT)11	(AT)11	(AT)11	(AT)11	-	Introns/Intergenic	-	-	CGCTTGTACTAACAATCCCAAACC	AAGTCACCGGACCGATTATGG	300	P	A	A	RM26700
PMS5549	Os_Chr11	16963215	16963228	(AG)7	(AG)7	(AG)7	(AG)7	(AG)7	(AG)8	(AG)7	(AG)7	(AG)7	(AG)7	(AG)7	-	Introns/Intergenic	-	-	CTGTCCTTGTCAAAGCAGGC	GAAGGAACACATGGGCACTT	185	P	A	A	NA
PMS5550	Os_Chr11	16965019	16965062	(AT)22	(AT)22	(AT)22	(AT)9	(AT)22	(AT)22	(AT)22	(AT)22	(AT)22	(AT)22	(AT)7	-	Introns/Intergenic	-	-	CGTCACCGATCACTACACATGC	TGTAGGCTCTCTGACCCTGATGG	310	P	A	A	RM26702

Polymorphic SSR (PMS) markers	Chromosomes	Start physical positions (bp)	End physical positions (bp)	Nipponbare	Kasalath	Nagina22	IR64	Pokkali	Bala	Tainung67	Azucena	Moroberekan	*Oryza rufipogon*	*Oryza nivara*	MSU gene locus IDs	Structural annotation	Functional annotation	SSR functional effects	Forward primers (5'-3')	Reverse primers (5'-3')	Amplified product size (bp)	Markers exhibiting polymorphism within *indica* rice	Transcrption factor genes-derived markers	Known cloned genes-derived markers	PMS markers corresponding with RM markers
PMS5551	Os_Chr11	16988307	16988322	(AG)8	(AG)8	(AG)8	(AG)8	(AG)8	(AG)7	(AG)8	(AG)8	(AG)8	(AG)8	(AG)8	-	Introns/Intergenic	-	-	AAGAGCATGTGGGGATTTTG	TGTGGCAACACATGGAATTT	247	P	A	A	NA
PMS5552	Os_Chr11	17027863	17027894	(AT)16	(AT)16	(AT)11	(AT)16	(AT)6	(AT)6	(AT)16	(AT)16	(AT)16	(AT)16	(AT)16	-	Introns/Intergenic	-	-	ACGTCTGTCGTCTGAGCAAAGG	TTCGTGTTTGAATGCTGAGAGC	424	P	A	A	RM26718
PMS5553	Os_Chr11	17061797	17061848	(AT)26	(AT)26	(AT)26	(AT)26	(AT)26	(AT)26	(AT)6	(AT)26	(AT)11	(AT)13	(AT)26	-	Introns/Intergenic	-	-	AGGCTGCAAGTGAAGGCAAGC	CTTTCTTTCCGTTGGGAATCAACC	517	A	A	A	RM26724
PMS5554	Os_Chr11	17114144	17114179	(AT)18	(AT)18	(AT)18	(AT)18	(AT)6	(AT)18	(AT)18	(AT)6	(AT)6	(AT)8	(AT)18	-	Introns/Intergenic	-	-	ATGTAGGCCCAAACGAGCTCTGACC	ATGCCATAGTAGCGCTTGCGTATCC	313	P	A	A	RM26725
PMS5555	Os_Chr11	17180907	17180924	(CGC)6	(CGC)6	(CGC)6	(CGC)6	(CGC)6	(CGC)6	(CGC)6	(CGC)6	(CGC)5	(CGC)6	(CGC)6	LOC_Os11g29630	CDS	expressed protein	CODON_DELETION	ACACCCAGTGCAGCGAGTA	CCCAGCTCAGCCAGTACTTC	135	A	A	A	NA
PMS5556	Os_Chr11	17188888	17188917	(AT)15	(AT)15	(AT)15	(AT)15	(AT)9	(AT)15	(AT)15	(AT)15	(AT)15	(AT)15	(AT)12	-	Introns/Intergenic	-	-	GCTCCAATTCCTTCTAGGGC	CGTGAATGAGAACAAGCGAA	233	P	A	A	NA
PMS5557	Os_Chr11	17196892	17196905	(GT)7	(GT)7	(GT)7	(GT)6	(GT)7	(GT)7	(GT)7	(GT)7	(GT)7	(GT)7	(GT)7	-	Introns/Intergenic	-	-	CGAATGGTCAAACGTTGGTT	TAGGCGCACTGTAATGCAAG	227	P	A	A	NA
PMS5558	Os_Chr11	17233629	17233658	(CT)15	(CT)15	(CT)15	(CT)16	(CT)16	(CT)15	(CT)15	(CT)15	(CT)15	(CT)15	(CT)15	-	Introns/Intergenic	-	-	CTGTTTGGCCAAGGAAACAC	CTACACGCGAGAACCAGACC	276	P	A	A	NA
PMS5559	Os_Chr11	17299192	17299206	(AGG)5	(AGG)5	(AGG)5	(AGG)5	(AGG)5	(AGG)6	(AGG)5	(AGG)5	(AGG)5	(AGG)5	(AGG)5	LOC_Os11g29810	CDS	expressed protein	CODON_CHANGE_PLUS_CODON_INSERTION	GTTCGAGGACCACCTCTCC	CGAGAATCGATTTTGGTGGT	215	P	P	A	NA
PMS5560	Os_Chr11	17299342	17299359	(CCG)6	(CCG)6	(CCG)6	(CCG)6	(CCG)6	(CCG)6	(CCG)5	(CCG)6	(CCG)6	(CCG)6	(CCG)6	LOC_Os11g29810	CDS	expressed protein	CODON_DELETION	AGGAGGAGGAGGAGGTCGT	CGAGAATCGATTTTGGTGGT	192	A	P	A	NA
PMS5561	Os_Chr11	17347037	17347052	(AG)8	(AG)8	(AG)8	(AG)8	(AG)8	(AG)8	(AG)8	(AG)8	(AG)8	(AG)8	(AG)7	-	Introns/Intergenic	-	-	TTGGTTTGTGTGTCTTTAGGTCA	CGAGTTGTCTCTCTGCACCA	254	A	A	A	NA
PMS5562	Os_Chr11	17377663	17377674	(GA)6	(GA)6	(GA)6	(GA)6	(GA)6	(GA)9	(GA)6	(GA)6	(GA)6	(GA)6	(GA)6	-	Introns/Intergenic	-	-	GCGGAGAGGATAAGGATGGT	TTTATTTTCGTTGGCGGAAC	180	P	A	A	NA
PMS5563	Os_Chr11	17428480	17428493	(AT)7	(AT)6	(AT)7	(AT)7	(AT)7	(AT)7	(AT)7	(AT)7	(AT)7	(AT)7	(AT)7	-	Introns/Intergenic	-	-	TCCATTAAATTTCCAACCTCC	AAAAACTACTCCCTCCGTTTCA	262	P	A	A	NA

Polymorphic SSR (PMS) markers	Chromosomes	Start physical positions (bp)	End physical positions (bp)	Nipponbare	Kasalath	Nagina22	IR64	Pokkali	Bala	Tainung67	Azucena	Moroberekan	*Oryza rufipogon*	*Oryza nivara*	MSU gene locus IDs	Structural annotation	Functional annotation	SSR functional effects	Forward primers (5'-3')	Reverse primers (5'-3')	Amplified product size (bp)	Markers exhibiting polymorphism within *indica* rice	Transcrption factor genes-derived markers	Known cloned genes-derived markers	PMS markers corresponding with RM markers
PMS5564	Os_Chr11	17429621	17429632	(GA)6	(GA)10	(GA)6	(GA)6	(GA)6	(GA)6	(GA)6	(GA)6	(GA)6	(GA)6	(GA)6	-	Introns/Intergenic	-	-	GCATGCTGGGATCCACTAAG	CCAATGACCCAAATGAAACC	260	P	A	A	NA
PMS5565	Os_Chr11	17465510	17465530	(GGC)7	(GGC)5	(GGC)7	(GGC)7	(GGC)7	(GGC)6	(GGC)7	(GGC)7	(GGC)7	(GGC)7	(GGC)7	-	Introns/Intergenic	-	-	AATATGGCTGGAGGAGGGAAGG	GAGGAGAGGATCGCCTGTAGCC	361	P	A	A	RM26747
PMS5566	Os_Chr11	17473796	17473831	(ATCT)9	(ATCT)5	(ATCT)9	(ATCT)9	(ATCT)9	(ATCT)7	(ATCT)8	(ATCT)9	(ATCT)9	(ATCT)9	(ATCT)8	-	Introns/Intergenic	-	-	TGACCGGAACATTGAACAGA	GTGGCGGATTGAGAGTGTTT	216	P	A	A	NA
PMS5567	Os_Chr11	17483326	17483361	(GGGAAT)6	(GGGAAT)6	(GGGAAT)6	(GGGAAT)6	(GGGAAT)6	(GGGAAT)6	(GGGAAT)6	(GGGAAT)6	(GGGAAT)6	(GGGAAT)6	(GGGAAT)5	-	Introns/Intergenic	-	-	TGCAAGCTGAGAGAAAAGCA	GACGCTCACTTCGATCACAC	266	A	A	A	NA
PMS5568	Os_Chr11	17625814	17625837	(AG)12	(AG)11	(AG)12	(AG)12	(AG)11	(AG)11	(AG)12	(AG)12	(AG)12	(AG)12	(AG)12	LOC_Os11g30360	URR	expressed protein	-	CATCTGCATATCTTCCCTGCAACC	AACAGCACCAGCCCTATGAACACC	184	P	A	A	RM5312
PMS5569	Os_Chr11	17649882	17649899	(AT)9	(AT)9	(AT)9	(AT)9	(AT)9	(AT)6	(AT)9	(AT)9	(AT)9	(AT)9	(AT)9	-	Introns/Intergenic	-	-	CTGTCTCACCTTGCTAGGGC	CCAACATCGATCACGATCAC	249	P	A	A	NA
PMS5570	Os_Chr11	17687239	17687286	(TC)24	(TC)24	(TC)24	(TC)24	(TC)24	(TC)24	(TC)24	(TC)22	(TC)24	(TC)24	(TC)24	-	Introns/Intergenic	-	-	ATGTTACTGTGTGCGGGTCC	AGCCAGAGAACCCTAGCTCC	204	A	A	A	NA
PMS5571	Os_Chr11	17833195	17833208	(CT)7	(CT)7	(CT)7	(CT)7	(CT)6	(CT)6	(CT)7	(CT)7	(CT)7	(CT)7	(CT)7	-	Introns/Intergenic	-	-	GGTCCTCTTAATTGACCGCA	AGCAGAGGAGAGGGAAGGAC	157	P	A	A	NA
PMS5572	Os_Chr11	18074282	18074299	(TA)9	(TA)9	(TA)9	(TA)9	(TA)9	(TA)9	(TA)9	(TA)6	(TA)9	(TA)9	(TA)9	-	Introns/Intergenic	-	-	TGAATGTACTCCCTCCGGACT	CTTGTCGTTTTGGACAAGGG	256	A	A	A	NA
PMS5573	Os_Chr11	18076352	18076369	(CGC)6	(CGC)6	(CGC)6	(CGC)6	(CGC)6	(CGC)6	(CGC)6	(CGC)5	(CGC)6	(CGC)6	(CGC)6	LOC_Os11g31070	CDS	hypothetical protein	CODON_DELETION	GCATCATGAGCGTGTCATCT	GACACCCACCTCGTTCTGAT	272	A	A	A	NA
PMS5574	Os_Chr11	18082292	18082331	(ATAG)10	(ATAG)10	(ATAG)5	(ATAG)10	(ATAG)10	(ATAG)6	(ATAG)10	(ATAG)10	(ATAG)10	(ATAG)8	(ATAG)10	-	Introns/Intergenic	-	-	GGATACAGAAGGAAGGAGAGC	ATGGACGCTTACTCTGTTATGG	436	P	A	A	RM26782
PMS5575	Os_Chr11	18102365	18102382	(CCG)6	(CCG)5	(CCG)6	(CCG)6	(CCG)6	(CCG)5	(CCG)6	(CCG)5	(CCG)6	(CCG)5	(CCG)6	LOC_Os11g31100	CDS	gibberellin response modulator protein, putative, expressed	CODON_DELETION	ACGTAGCTCTGGTGGTGGAG	GGGATTACATGGTGACCTCG	190	P	P	A	NA
PMS5576	Os_Chr11	18149534	18149555	(TA)11	(TA)11	(TA)11	(TA)11	(TA)11	(TA)11	(TA)11	(TA)11	(TA)11	(TA)6	(TA)11	-	Introns/Intergenic	-	-	ACGAGAACAAGGTGAATGAACG	GGTGACGTATATTATAGGACCCTAGC	493	A	A	A	RM26786

Polymorphic SSR (PMS) markers	Chromosomes	Start physical positions (bp)	End physical positions (bp)	Nipponbare	Kasalath	Nagina22	IR64	Pokkali	Bala	Tainung67	Azucena	Moroberekan	*Oryza rufipogon*	*Oryza nivara*	MSU gene locus IDs	Structural annotation	Functional annotation	SSR functional effects	Forward primers (5'-3')	Reverse primers (5'-3')	Amplified product size (bp)	Markers exhibiting polymorphism within *indica* rice	Transcrption factor genes-derived markers	Known cloned genes-derived markers	PMS markers corresponding with RM markers
PMS5577	Os_Chr11	18399523	18399540	(GAA)6	(GAA)6	(GAA)6	(GAA)6	(GAA)6	(GAA)7	(GAA)6	(GAA)6	(GAA)6	(GAA)6	(GAA)6	-	Introns/Intergenic	-	-	AGATCTGATCGGGGCAGAG	CCACCTGTCAATCTCCCATC	151	P	A	A	NA
PMS5578	Os_Chr11	18502811	18502891	(TTA)27	(TTA)27	(TTA)27	(TTA)27	(TTA)27	(TTA)27	(TTA)17	(TTA)27	(TTA)13	(TTA)27	(TTA)27	-	Introns/Intergenic	-	-	TATGGCGTGTAAGAGGATTAGAGG	TCTTGGTGGTCGTGTAAGATTAGC	418	A	A	A	RM26799
PMS5579	Os_Chr11	18673793	18673837	(TAA)15	(TAA)15	(TAA)15	(TAA)15	(TAA)15	(TAA)13	(TAA)7	(TAA)15	(TAA)15	(TAA)15	(TAA)15	-	Introns/Intergenic	-	-	TAAATTGGGTGGGGACGTAA	GTCTCTCAGTTTCTTGGCGG	221	P	A	A	NA
PMS5580	Os_Chr11	18702585	18702646	(AT)31	(AT)16	(AT)31	(AT)31	(AT)31	(AT)31	(AT)31	(AT)31	(AT)31	(AT)6	(AT)31	-	Introns/Intergenic	-	-	GCGTAGTTGCGTACATACGTT	CACCATGGAACACCAAACAA	278	P	A	A	NA
PMS5581	Os_Chr11	18717892	18717903	(GT)6	(GT)7	(GT)6	(GT)8	(GT)6	(GT)6	(GT)6	(GT)6	(GT)6	(GT)7	(GT)7	-	Introns/Intergenic	-	-	GAAATTGGCCTACCGATTCA	TGGCGCAAAGTATGAACTCTT	279	P	A	A	NA
PMS5582	Os_Chr11	18729032	18729049	(CCG)6	(CCG)6	(CCG)6	(CCG)5	(CCG)6	(CCG)6	(CCG)6	(CCG)6	(CCG)6	(CCG)6	(CCG)6	LOC_Os11g31880	URR	phosphoglycerate mutase, putative, expressed	-	ATCGATCCAGCACCAATCTC	AGGAGTGGGAGGAGTGGG	157	P	A	A	NA
PMS5583	Os_Chr11	18753225	18753245	(GAG)7	(GAG)7	(GAG)7	(GAG)7	(GAG)7	(GAG)7	(GAG)7	(GAG)7	(GAG)7	(GAG)6	(GAG)7	LOC_Os11g31900	URR	acyl carrier protein, putative, expressed	-	CTTCACCTTCCAGCTACCGA	AGCTTCCATTTGCCCCTACT	207	A	A	A	NA
PMS5584	Os_Chr11	18773575	18773588	(AT)7	(AT)7	(AT)7	(AT)6	(AT)7	(AT)7	(AT)7	(AT)7	(AT)7	(AT)7	(AT)7	-	Introns/Intergenic	-	-	ACCGGATATATCGACCTCCC	AGAGAGGATGACATGTGGGC	241	P	A	A	NA
PMS5585	Os_Chr11	18820593	18820612	(AT)10	(AT)10	(AT)10	(AT)10	(AT)10	(AT)10	(AT)10	(AT)10	(AT)10	(AT)10	(AT)6	-	Introns/Intergenic	-	-	ACTAGTGGCCACCCACTCTATGG	GCACGTATATGTGAGAATAGGCTTGG	98	A	A	A	RM26813
PMS5586	Os_Chr11	18868747	18868796	(TA)25	(TA)25	(TA)11	(TA)22	(TA)25	(TA)25	(TA)25	(TA)25	(TA)25	(TA)13	(TA)25	-	Introns/Intergenic	-	-	TCCAACCGGGACTAAAGATAGAGC	AACACAATTCACCGGTCCTTAGC	281	P	A	A	RM10645
PMS5587	Os_Chr11	18873082	18873111	(AT)15	(AT)15	(AT)15	(AT)15	(AT)15	(AT)15	(AT)15	(AT)15	(AT)15	(AT)15	(AT)8	-	Introns/Intergenic	-	-	TGATAGCAATCGGGGACATT	CAACTTCAGATGAAACGATTGG	254	A	A	A	NA
PMS5588	Os_Chr11	18874111	18874146	(TC)18	(TC)18	(TC)18	(TC)18	(TC)18	(TC)17	(TC)18	(TC)18	(TC)18	(TC)12	(TC)18	LOC_Os11g32030	URR	sex determination protein tasselseed-2, putative, expressed	-	TATTTAAGCAGCCCCCACAC	TCGCAGGTTCTTGTGAAATG	180	P	A	A	NA
PMS5589	Os_Chr11	18922701	18922718	(CGG)6	(CGG)6	(CGG)6	(CGG)6	(CGG)6	(CGG)6	(CGG)6	(CGG)6	(CGG)6	(CGG)6	(CGG)5	LOC_Os11g32100	CDS	OsICE1	CODON_DELETION	GTCCCAAATGCCGGAGTTC	CTTCTTGTCGTCGTCGTCCT	271	A	P	P	NA

Polymorphic SSR (PMS) markers	Chromosomes	Start physical positions (bp)	End physical positions (bp)	Nipponbare	Kasalath	Nagina22	IR64	Pokkali	Bala	Tainung67	Azucena	Moroberekan	*Oryza rufipogon*	*Oryza nivara*	MSU gene locus IDs	Structural annotation	Functional annotation	SSR functional effects	Forward primers (5'-3')	Reverse primers (5'-3')	Amplified product size (bp)	Markers exhibiting polymorphism within *indica* rice	Transcrption factor genes-derived markers	Known cloned genes-derived markers	PMS markers corresponding with RM markers
PMS5590	Os_Chr11	19247020	19247105	(TA)43	(TA)43	(TA)43	(TA)43	(TA)43	(TA)43	(TA)43	(TA)43	(TA)43	(TA)7	(TA)43	-	Introns/Intergenic	-	-	GAGAAGGCCTGATGAGTACAAGG	GATTATTGTGCAGGTGAGAAGTGG	383	A	A	A	RM26829
PMS5591	Os_Chr11	19253234	19253299	(TA)33	(TA)33	(TA)33	(TA)6	(TA)33	(TA)12	(TA)33	(TA)33	(TA)33	(TA)33	(TA)33	-	Introns/Intergenic	-	-	TTGAGAGCATACGATTGAGGTTGG	GCTGCCATAGCTTTCTTCGTTAGG	160	P	A	A	RM26830
PMS5592	Os_Chr11	19276469	19276510	(TA)21	(TA)21	(TA)21	(TA)21	(TA)6	(TA)10	(TA)7	(TA)21	(TA)10	(TA)21	(TA)21	-	Introns/Intergenic	-	-	ATGGCCCAGCTTATTAGCTTACG	TGCTTCGGGTATCAATGAATCG	236	P	A	A	RM26835
PMS5593	Os_Chr11	19286290	19286309	(AT)10	(AT)10	(AT)10	(AT)10	(AT)6	(AT)10	(AT)10	(AT)7	(AT)10	(AT)10	(AT)10	-	Introns/Intergenic	-	-	ATCCCGCACTTTGATTATACGG	TTGGACAAGGGTACGGTCTCC	408	P	A	A	RM26836
PMS5594	Os_Chr11	19295065	19295090	(TA)13	(TA)13	(TA)8	(TA)13	(TA)13	(TA)13	(TA)6	(TA)13	(TA)13	(TA)13	(TA)13	-	Introns/Intergenic	-	-	CTGTTGCTGCATGGTTTAT	CATATGTTTCTATATGCATGTGT	250	P	A	A	NA
PMS5595	Os_Chr11	19361203	19361272	(AT)35	(AT)17	(AT)35	(AT)35	(AT)35	(AT)35	(AT)35	(AT)35	(AT)35	(AT)35	(AT)35	-	Introns/Intergenic	-	-	CAACTTAGAGCATTATGAATGTA	GTGTTCGTTGCAATTAAATCT	233	P	A	A	NA
PMS5596	Os_Chr11	19532570	19532581	(CA)6	(CA)6	(CA)7	(CA)6	(CA)6	(CA)6	(CA)6	(CA)6	(CA)6	(CA)6	(CA)6	-	Introns/Intergenic	-	-	GGCCCTAGATCCAATTGTCA	TCTAATGGCAGTAGCCGGTC	215	P	A	A	NA
PMS5597	Os_Chr11	19534583	19534609	(CTC)9	(CTC)9	(CTC)9	(CTC)9	(CTC)9	(CTC)9	(CTC)9	(CTC)9	(CTC)9	(CTC)9	(CTC)7	LOC_Os11g33050	CDS	expressed protein	CODON_DELETION	CTGTACGTCCTCACCTTCCC	CACACCACACGCCATTATCT	272	A	A	A	NA
PMS5598	Os_Chr11	19541142	19541153	(TC)6	(TC)6	(TC)6	(TC)7	(TC)6	(TC)6	(TC)6	(TC)6	(TC)6	(TC)6	(TC)6	-	Introns/Intergenic	-	-	GCTGCCGAGTAATACCCAGA	TTCGGTATGGAGGGAATGAG	109	P	A	A	NA
PMS5599	Os_Chr11	19593365	19593382	(TA)9	(TA)9	(TA)9	(TA)9	(TA)9	(TA)8	(TA)9	(TA)9	(TA)9	(TA)9	(TA)9	-	Introns/Intergenic	-	-	ATGATGCTCAAAGCATTCCC	CAAGCCACAGGGTTTCAAGT	259	P	A	A	NA
PMS5600	Os_Chr11	19645040	19645060	(CCG)7	(CCG)7	(CCG)7	(CCG)7	(CCG)7	(CCG)7	(CCG)7	(CCG)7	(CCG)7	(CCG)5	(CCG)7	LOC_Os11g33220	CDS	OsFBX423 - F-box domain containing protein, expressed	CODON_CHANGE_PLUS_CODON_DELETION	CCGTCGTCGAAGCGTACT	TCGATGATCCCCTGTAGGTC	238	A	A	A	NA
PMS5601	Os_Chr11	19658320	19658340	(CCG)7	(CCG)7	(CCG)7	(CCG)7	(CCG)7	(CCG)5	(CCG)7	(CCG)5	(CCG)7	(CCG)7	(CCG)7	LOC_Os11g33250	CDS	expressed protein	CODON_DELETION	ATCTCCTGCGAGCGAGACAGC	GAGTTCTTCAAGCAGGCGAAGG	175	P	A	A	RM26857
PMS5602	Os_Chr11	19683608	19683627	(AG)10	(AG)7	(AG)10	(AG)10	(AG)10	(AG)10	(AG)10	(AG)10	(AG)10	(AG)10	(AG)10	-	Introns/Intergenic	-	-	GCAGAGCTGTGGAAGAGGAG	ACGAGTACTGAGTGGCCCAG	223	P	A	A	NA

Polymorphic SSR (PMS) markers	Chromosomes	Start physical positions (bp)	End physical positions (bp)	Nipponbare	Kasalath	Nagina22	IR64	Pokkali	Bala	Tainung67	Azucena	Moroberekan	*Oryza rufipogon*	*Oryza nivara*	MSU gene locus IDs	Structural annotation	Functional annotation	SSR functional effects	Forward primers (5'-3')	Reverse primers (5'-3')	Amplified product size (bp)	Markers exhibiting polymorphism within *indica* rice	Transcrption factor genes-derived markers	Known cloned genes-derived markers	PMS markers corresponding with RM markers
PMS5603	Os_Chr11	19758532	19758559	(GA)14	(GA)14	(GA)14	(GA)14	(GA)14	(GA)18	(GA)14	(GA)14	(GA)14	(GA)14	(GA)14	-	Introns/Intergenic	-	-	CCTCTTCCTCCTCACTGGCTGTACC	GGCTTTCTATCTCTCGCTCTCAGTCG	289	P	A	A	RM26861
PMS5604	Os_Chr11	19887201	19887212	(CT)6	(CT)6	(CT)6	(CT)8	(CT)6	(CT)7	(CT)6	(CT)6	(CT)6	(CT)6	(CT)6	-	Introns/Intergenic	-	-	ATTTCGATAAAGCAGTGCCG	TCTGGAAACTGGATCAGCGT	129	P	A	A	NA
PMS5605	Os_Chr11	19889239	19889256	(TC)9	(TC)9	(TC)9	(TC)9	(TC)9	(TC)9	(TC)9	(TC)7	(TC)9	(TC)9	(TC)9	-	Introns/Intergenic	-	-	AGAAAAATTCGATGGAGCCC	GACGGCAATGTGGTCCTACT	164	A	A	A	NA
PMS5606	Os_Chr11	19916607	19916621	(TGT)5	(TGT)6	(TGT)6	(TGT)6	(TGT)5	(TGT)6	(TGT)5	(TGT)5	(TGT)5	(TGT)6	(TGT)6	-	Introns/Intergenic	-	-	CACCCTCCGTCTCAAAAAGA	GACAAGTGAACGTGGGGAAT	205	P	A	A	NA
PMS5607	Os_Chr11	20034573	20034599	(GGC)9	(GGC)9	(GGC)9	(GGC)9	(GGC)9	(GGC)9	(GGC)8	(GGC)6	(GGC)9	(GGC)9	(GGC)9	LOC_Os11g34210	URR	aspartyl/glutamyl-tRNA amidotransferase subunit B, putative, expressed	-	GAGGAGGCTGCGTCTACACTCC	GACTGGTGGACCCTCTCATATCC	160	A	A	A	RM26881
PMS5608	Os_Chr11	20063237	20063264	(AT)14	(AT)14	(AT)9	(AT)9	(AT)14	(AT)9	(AT)14	(AT)14	(AT)14	(AT)14	(AT)12	-	Introns/Intergenic	-	-	AATCATGGCGCTATTAGGCTT	GCACTCACGTCCCTCTCAAG	249	P	A	A	NA
PMS5609	Os_Chr11	20115517	20115534	(GCG)6	(GCG)6	(GCG)6	(GCG)5	(GCG)6	(GCG)6	(GCG)6	(GCG)6	(GCG)6	(GCG)6	(GCG)6	-	Introns/Intergenic	-	-	CTAGCTGCTCCCAGTCGTTC	CTCCTCGGTAGCCGACCT	179	P	A	A	NA
PMS5610	Os_Chr11	20152480	20152494	(CGC)5	(CGC)7	(CGC)5	(CGC)5	(CGC)5	(CGC)6	(CGC)5	(CGC)5	(CGC)6	(CGC)5	(CGC)5	-	Introns/Intergenic	-	-	GAGAACGAAAGAGCTAGGCG	GCGAGCCGAACAAGATCTAC	229	P	A	A	NA
PMS5611	Os_Chr11	20176173	20176190	(CGG)6	(CGG)6	(CGG)6	(CGG)6	(CGG)6	(CGG)6	(CGG)5	(CGG)5	(CGG)6	(CGG)5	(CGG)5	LOC_Os11g34440	CDS	phospholipase A2, putative, expressed	CODON_DELETION	AACGATCGACCAACGAAATC	GCGAAGCAAACGTACCAAAT	233	A	A	A	NA
PMS5612	Os_Chr11	20203475	20203500	(GA)13	(GA)13	(GA)13	(GA)13	(GA)13	(GA)13	(GA)13	(GA)13	(GA)13	(GA)6	(GA)13	-	Introns/Intergenic	-	-	GGAGTGGCAGAGGCTTAC	TCCTCCGCAAAAGTCTAAT	416	A	A	A	NA
PMS5613	Os_Chr11	20237992	20238007	(AG)8	(AG)8	(AG)8	(AG)8	(AG)8	(AG)6	(AG)8	(AG)8	(AG)8	(AG)8	(AG)8	-	Introns/Intergenic	-	-	GATGACGACGGCGATGAC	TCCTCCGCAAAAGTCTAAT	475	P	A	A	NA
PMS5614	Os_Chr11	20256619	20256694	(TA)38	(TA)38	(TA)38	(TA)38	(TA)38	(TA)38	(TA)38	(TA)38	(TA)8	(TA)38	(TA)6	-	Introns/Intergenic	-	-	CATGTAACTCCTCTTGCCTCTGC	ACGTACTTAGCTTGTTGCGATCC	519	A	A	A	RM26889
PMS5615	Os_Chr11	20260683	20260720	(TA)19	(TA)19	(TA)7	(TA)19	(TA)7	(TA)19	(TA)19	(TA)19	(TA)19	(TA)19	(TA)19	-	Introns/Intergenic	-	-	AATTGGAGAAGGGTCACTCAACG	GATTTAGAATTGTCCCGCTGTTCC	129	P	A	A	RM26890

Polymorphic SSR (PMS) markers	Chromosomes	Start physical positions (bp)	End physical positions (bp)	Nipponbare	Kasalath	Nagina22	IR64	Pokkali	Bala	Tainung67	Azucena	Moroberekan	*Oryza rufipogon*	*Oryza nivara*	MSU gene locus IDs	Structural annotation	Functional annotation	SSR functional effects	Forward primers (5'-3')	Reverse primers (5'-3')	Amplified product size (bp)	Markers exhibiting polymorphism within *indica* rice	Transcrption factor genes-derived markers	Known cloned genes-derived markers	PMS markers corresponding with RM markers
PMS5616	Os_Chr11	20316103	20316126	(AAT)8	(AAT)8	(AAT)8	(AAT)8	(AAT)8	(AAT)8	(AAT)8	(AAT)7	(AAT)8	(AAT)8	(AAT)8	-	Introns/Intergenic	-	-	AGGCTTCTGGAGGTAGCACA	CACTGCGCTGCTACTCTCTG	242	A	A	A	NA
PMS5617	Os_Chr11	20360107	20360122	(TG)8	(TG)8	(TG)8	(TG)8	(TG)8	(TG)8	(TG)8	(TG)8	(TG)10	(TG)8	(TG)8	-	Introns/Intergenic	-	-	CCCCAAATTTGAGAGACGCT	AGCGCAACCATACAAACCTC	211	A	A	A	NA
PMS5618	Os_Chr11	20529064	20529081	(CCA)6	(CCA)6	(CCA)6	(CCA)6	(CCA)6	(CCA)6	(CCA)6	(CCA)6	(CCA)6	(CCA)6	(CCA)5	LOC_Os11g35040	CDS	OsNAAT5	CODON_DELETION	CGCAGGAGGAGTAGGAGTTG	GTGCTGGGCAGGGTCAAG	193	A	A	P	NA
PMS5619	Os_Chr11	20549746	20549787	(TA)21	(TA)21	(TA)21	(TA)7	(TA)12	(TA)7	(TA)21	(TA)21	(TA)21	(TA)7	(TA)21	-	Introns/Intergenic	-	-	CATTGTGAAACGGAGGGAGT	GCCGATAATGAAAATGGCTG	183	P	A	A	NA
PMS5620	Os_Chr11	20554058	20554072	(CTT)5	(CTT)5	(CTT)5	(CTT)5	(CTT)5	(CTT)6	(CTT)5	(CTT)6	(CTT)6	(CTT)5	(CTT)5	-	Introns/Intergenic	-	-	GTTGGTCGCACGCTTAAACT	ACCCTCGAATAATTGTCGCA	239	P	A	A	NA
PMS5621	Os_Chr11	20671349	20671362	(TC)7	(TC)7	(TC)7	(TC)7	(TC)7	(TC)7	(TC)7	(TC)6	(TC)7	(TC)7	(TC)7	-	Introns/Intergenic	-	-	ATGAGTCCCACTCCCACAAG	GGTCTGCTGTCGTTCTACCG	149	A	A	A	NA
PMS5622	Os_Chr11	20690230	20690241	(GA)6	(GA)6	(GA)7	(GA)6	(GA)6	(GA)6	(GA)6	(GA)6	(GA)6	(GA)6	(GA)6	-	Introns/Intergenic	-	-	CATCTCCTCTCTCAGCCGTT	GTTTTGGGGAGGGAGAGAAG	263	P	A	A	NA
PMS5623	Os_Chr11	20770142	20770165	(CGC)8	(CGC)8	(CGC)5	(CGC)8	(CGC)8	(CGC)8	(CGC)8	(CGC)8	(CGC)8	(CGC)8	(CGC)8	LOC_Os11g35450	CDS	leucine-rich repeat receptor protein kinase EXS precursor, putative, expressed	CODON_DELETION	AAGCATCCATCAAGCCACTT	GGTTTGCAGTAGTTGCCGAT	223	P	A	A	NA
PMS5624	Os_Chr11	20962393	20962492	(AT)50	(AT)50	(AT)50	(AT)50	(AT)50	(AT)47	(AT)8	(AT)50	(AT)50	(AT)9	(AT)15	-	Introns/Intergenic	-	-	GGTTGAACTTAGGCAAGGCA	TTGGTCTCCGAACCATTAAA	226	P	A	A	NA
PMS5625	Os_Chr11	20970627	20970676	(TA)25	(TA)25	(TA)9	(TA)25	(TA)8	(TA)17	(TA)25	(TA)25	(TA)10	(TA)25	(TA)25	-	Introns/Intergenic	-	-	ACACATCTGAAGTTTGGAGACC	GTATATACTCCTCCCTCCTCTCC	694	P	A	A	RM26920
PMS5626	Os_Chr11	21107688	21107725	(AT)19	(AT)19	(AT)19	(AT)19	(AT)19	(AT)19	(AT)19	(AT)19	(AT)6	(AT)19	(AT)19	-	Introns/Intergenic	-	-	GAACGAACGAGAGCGAGAAC	GCTCCAATTCCTTCTAGGGC	251	A	A	A	NA
PMS5627	Os_Chr11	21217950	21217964	(AAT)5	(AAT)5	(AAT)5	(AAT)6	(AAT)6	(AAT)5	(AAT)5	(AAT)5	(AAT)5	(AAT)5	(AAT)5	-	Introns/Intergenic	-	-	GCAGTGTTATCGCCACTCAA	GGCAAAGAGGGAGGAATCTT	206	P	A	A	NA
PMS5628	Os_Chr11	21246729	21246746	(AGG)6	(AGG)6	(AGG)6	(AGG)6	(AGG)6	(AGG)5	(AGG)6	(AGG)6	(AGG)6	(AGG)5	(AGG)7	LOC_Os11g36140	CDS	receptor-like protein kinase 2 precursor, putative, expressed	CODON_INSERTION	GGACTTAATGGAGAGCAGCG	TCTGTTCCCACTCTCTGGCT	116	P	A	A	NA

Polymorphic SSR (PMS) markers	Chromosomes	Start physical positions (bp)	End physical positions (bp)	Nipponbare	Kasalath	Nagina22	IR64	Pokkali	Bala	Tainung67	Azucena	Moroberekan	*Oryza rufipogon*	*Oryza nivara*	MSU gene locus IDs	Structural annotation	Functional annotation	SSR functional effects	Forward primers (5'-3')	Reverse primers (5'-3')	Amplified product size (bp)	Markers exhibiting polymorphism within *indica* rice	Transcrption factor genes-derived markers	Known cloned genes-derived markers	PMS markers corresponding with RM markers
PMS5629	Os_Chr11	21277345	21277365	(AGC)7	(AGC)6	(AGC)7	(AGC)7	(AGC)7	(AGC)6	(AGC)7	(AGC)6	(AGC)7	(AGC)7	(AGC)6	LOC_Os11g36180	CDS	receptor kinase, putative, expressed	CODON_DELETION	TTCCAAGATGCCAGCGACAAGC	AACGTCGGTCATGATCTCTTCTTTGC	183	P	A	A	RM26930
PMS5630	Os_Chr11	21355927	21355960	(AT)17	(AT)17	(AT)7	(AT)9	(AT)8	(AT)17	(AT)17	(AT)13	(AT)17	(AT)17	(AT)17	-	Introns/Intergenic	-	-	GACAGCCAAATTAAAGCCTGTCC	CAATTCATAACGTCGGTCCTTCC	347	P	A	A	RM26935
PMS5631	Os_Chr11	21394018	21394065	(AT)24	(AT)24	(AT)24	(AT)24	(AT)24	(AT)24	(AT)24	(AT)10	(AT)7	(AT)24	(AT)11	-	Introns/Intergenic	-	-	AGAGGATGAACTACAGGGCAAGC	GTGAGGTGGGAACTAATCCATCG	250	A	A	A	RM26937
PMS5632	Os_Chr11	21463892	21463912	(CGC)7	(CGC)8	(CGC)7	(CGC)7	(CGC)7	(CGC)7	(CGC)7	(CGC)7	(CGC)7	(CGC)7	(CGC)7	LOC_Os11g36400	CDS	expressed protein	CODON_INSERTION	CTCGTCGACTCTGAGAACCC	TCGTCTTCGGCATCCTTTAC	279	P	A	A	NA
PMS5633	Os_Chr11	21516318	21516344	(GCG)9	(GCG)8	(GCG)9	(GCG)5	(GCG)9	(GCG)9	(GCG)9	(GCG)5	(GCG)9	(GCG)9	(GCG)5	-	Introns/Intergenic	-	-	GGAGGCGTCATCATAGTCATGG	AAACTCCCAAACTCCTCCATTCC	187	P	A	A	RM6534
PMS5634	Os_Chr11	21524136	21524153	(GCG)6	(GCG)6	(GCG)6	(GCG)6	(GCG)6	(GCG)6	(GCG)5	(GCG)6	(GCG)6	(GCG)6	(GCG)6	-	Introns/Intergenic	-	-	ACCTCAGCCTCGAGATCGT	GCTGCTTCCTCTTCTCCTTG	198	A	A	A	NA
PMS5635	Os_Chr11	21646391	21646406	(AG)8	(AG)8	(AG)8	(AG)8	(AG)8	(AG)7	(AG)8	(AG)8	(AG)8	(AG)8	(AG)8	-	Introns/Intergenic	-	-	GGTGCTATGTCGCTTGATGA	TACAACGCCTCGCTTTTCTT	125	P	A	A	NA
PMS5636	Os_Chr11	21714586	21714599	(CT)7	(CT)7	(CT)7	(CT)7	(CT)7	(CT)6	(CT)7	(CT)7	(CT)7	(CT)7	(CT)7	-	Introns/Intergenic	-	-	CCCATCTCCTCCTTTTCCTC	GGATCGGGATCGATCTTCTC	237	P	A	A	NA
PMS5637	Os_Chr11	21761447	21761458	(TG)6	(TG)6	(TG)7	(TG)6	(TG)6	(TG)6	(TG)6	(TG)6	(TG)6	(TG)6	(TG)6	-	Introns/Intergenic	-	-	CCTTGCGTACCGTTCATCTT	AGCAACTTGTGAATCCGACC	174	P	A	A	NA
PMS5638	Os_Chr11	21849801	21849821	(TGG)7	(TGG)6	(TGG)7	(TGG)7	(TGG)7	(TGG)6	(TGG)7	(TGG)7	(TGG)6	(TGG)7	(TGG)6	-	Introns/Intergenic	-	-	GTGTGCCTGTATGGTGGTTGG	TGCATACGGATTCGATAGTACACG	386	P	A	A	RM26962
PMS5639	Os_Chr11	21871600	21871681	(AT)41	(AT)22	(AT)41	(AT)41	(AT)41	(AT)41	(AT)41	(AT)41	(AT)41	(AT)41	(AT)7	-	Introns/Intergenic	-	-	CTAGCCGAATACCTGTGCTGTGC	GTGAACTGGGTGATGTGTTCTTCG	484	P	A	A	RM26963
PMS5640	Os_Chr11	21906439	21906456	(GA)9	(GA)9	(GA)9	(GA)7	(GA)9	(GA)9	(GA)9	(GA)9	(GA)9	(GA)9	(GA)9	LOC_Os11g37100	URR	expressed protein	-	AGACCAGCCGTAAAACCCTT	TCACAATCTCCACCGAATCA	206	P	A	A	NA
PMS5641	Os_Chr11	21925518	21925533	(GA)8	(GA)8	(GA)8	(GA)8	(GA)8	(GA)8	(GA)8	(GA)8	(GA)6	(GA)8	(GA)8	-	Introns/Intergenic	-	-	AGAGAGGGAGGGACGACCTA	TCATTACCCCTTTTCCCCTC	203	A	A	A	NA

Polymorphic SSR (PMS) markers	Chromosomes	Start physical positions (bp)	End physical positions (bp)	Nipponbare	Kasalath	Nagina22	IR64	Pokkali	Bala	Tainung67	Azucena	Moroberekan	*Oryza rufipogon*	*Oryza nivara*	MSU gene locus IDs	Structural annotation	Functional annotation	SSR functional effects	Forward primers (5'-3')	Reverse primers (5'-3')	Amplified product size (bp)	Markers exhibiting polymorphism within *indica* rice	Transcrption factor genes-derived markers	Known cloned genes-derived markers	PMS markers corresponding with RM markers
PMS5642	Os_Chr11	21957899	21957912	(GA)7	(GA)7	(GA)7	(GA)7	(GA)7	(GA)7	(GA)6	(GA)7	(GA)7	(GA)7	(GA)7	-	Introns/Intergenic	-	-	GGCCACAAAAGACAGGTAGC	CCCCCGTTTTACAAACACAA	221	A	A	A	NA
PMS5643	Os_Chr11	21965772	21965833	(TA)31	(TA)31	(TA)10	(TA)7	(TA)31	(TA)17	(TA)6	(TA)31	(TA)31	(TA)8	(TA)7	-	Introns/Intergenic	-	-	ACGCACCAGCCATTCACTGAGC	CCCATAGTAGCAGGGTCTGCATGG	380	P	A	A	RM26968
PMS5644	Os_Chr11	22003385	22003418	(CT)17	(CT)17	(CT)17	(CT)17	(CT)17	(CT)17	(CT)17	(CT)15	(CT)17	(CT)17	(CT)17	LOC_Os11g37260	URR	SEY1, putative, expressed	-	GAACCCTCTCACGTGTGGAT	GAGGAGGGAAGCCGACAG	236	A	A	A	NA
PMS5645	Os_Chr11	22019223	22019312	(TA)45	(TA)13	(TA)7	(TA)9	(TA)15	(TA)45	(TA)45	(TA)45	(TA)15	(TA)45	(TA)45	-	Introns/Intergenic	-	-	ATTAACAGGGCGGAGCTAGAACG	CCCACAGCCATTGTCTCTACCC	330	P	A	A	RM26972
PMS5646	Os_Chr11	22046853	22046867	(GCT)5	(GCT)6	(GCT)5	(GCT)5	(GCT)5	(GCT)5	(GCT)5	(GCT)5	(GCT)5	(GCT)5	(GCT)5	-	Introns/Intergenic	-	-	TCAACTTTGATCCATTGCCA	ATACTGTTCCTGGGCTTCCA	111	P	A	A	NA
PMS5647	Os_Chr11	22099114	22099147	(AT)17	(AT)17	(AT)17	(AT)17	(AT)17	(AT)17	(AT)17	(AT)17	(AT)6	(AT)17	(AT)17	-	Introns/Intergenic	-	-	GCTCCAATTCCTTCTAGGGC	CGTGAATGAGAACAAGCGAA	238	A	A	A	NA
PMS5648	Os_Chr11	22131696	22131721	(GA)13	(GA)13	(GA)10	(GA)13	(GA)13	(GA)13	(GA)13	(GA)13	(GA)13	(GA)13	(GA)7	-	Introns/Intergenic	-	-	GCTCGATTTGTACCAGGTGTCTCC	GCTCTCCCTTCCTCTCCTTTCC	183	P	A	A	RM26976
PMS5649	Os_Chr11	22135068	22135091	(CTG)8	(CTG)8	(CTG)8	(CTG)8	(CTG)8	(CTG)7	(CTG)8	(CTG)8	(CTG)8	(CTG)8	(CTG)8	LOC_Os11g37490	URR	expressed protein	-	AGCCTCCGCTGAGTCTTC	GCCTCCTTCTCCTCTCATCC	211	P	A	A	NA
PMS5650	Os_Chr11	22150093	22150118	(CT)13	(CT)13	(CT)11	(CT)13	(CT)13	(CT)12	(CT)13	(CT)13	(CT)13	(CT)13	(CT)13	-	Introns/Intergenic	-	-	ACAACTTCCTCTCTGTTTCACTACCC	CTGCTCTTTGTGCGCTTTATCC	137	P	A	A	RM26979
PMS5651	Os_Chr11	22215985	22216002	(GAG)6	(GAG)6	(GAG)6	(GAG)5	(GAG)6	(GAG)6	(GAG)6	(GAG)6	(GAG)5	(GAG)6	(GAG)6	LOC_Os11g37640	URR	ADP-ribosylation factor, putative, expressed	-	ACCCAATTCCCTTCTCACCT	CGATCTTGTACTCCTTCGCC	268	P	A	A	NA
PMS5652	Os_Chr11	22253371	22253388	(TC)9	(TC)9	(TC)9	(TC)8	(TC)9	(TC)8	(TC)9	(TC)9	(TC)9	(TC)8	(TC)9	-	Introns/Intergenic	-	-	GATACTGTGGCATGCAGTGG	CAAACAAAACCAACCAACCC	258	P	A	A	NA
PMS5653	Os_Chr11	22311014	22311034	(CCA)7	(CCA)6	(CCA)7	(CCA)6	(CCA)6	(CCA)6	(CCA)7	(CCA)7	(CCA)7	(CCA)7	(CCA)6	LOC_Os11g37740	URR	stripe rust resistance protein Yr10, putative, expressed	-	CCCTCATTTGATGCCTCACT	GCCAAGCTCTTGTTCTTGCT	142	P	A	A	NA
PMS5654	Os_Chr11	22362907	22362934	(GA)14	(GA)14	(GA)14	(GA)14	(GA)14	(GA)14	(GA)13	(GA)14	(GA)14	(GA)14	(GA)14	-	Introns/Intergenic	-	-	GAGAGGGAGGACTCGGCT	AAATCCCTCCGCAAAAGTCT	245	A	A	A	NA

Polymorphic SSR (PMS) markers	Chromosomes	Start physical positions (bp)	End physical positions (bp)	Nipponbare	Kasalath	Nagina22	IR64	Pokkali	Bala	Tainung67	Azucena	Moroberekan	*Oryza rufipogon*	*Oryza nivara*	MSU gene locus IDs	Structural annotation	Functional annotation	SSR functional effects	Forward primers (5'-3')	Reverse primers (5'-3')	Amplified product size (bp)	Markers exhibiting polymorphism within *indica* rice	Transcrption factor genes-derived markers	Known cloned genes-derived markers	PMS markers corresponding with RM markers
PMS5655	Os_Chr11	22554311	22554325	(GCA)5	(GCA)5	(GCA)6	(GCA)6	(GCA)6	(GCA)5	(GCA)5	(GCA)5	(GCA)5	(GCA)6	(GCA)5	LOC_Os11g38010	CDS	targeting protein for Xklp2, putative, expressed	CODON_CHANGE_PLUS_CODON_INSERTION	AAAGCTGGGGAGGAGGAAG	GAGAAGCAGCTAGTGGACGG	262	P	A	A	NA
PMS5656	Os_Chr11	22609466	22609483	(GGC)6	(GGC)6	(GGC)6	(GGC)6	(GGC)6	(GGC)6	(GGC)6	(GGC)5	(GGC)6	(GGC)6	(GGC)6	-	Introns/Intergenic	-	-	GGATCTGGCGAGGCGAAG	CCTGAAGCATTTTCATCTCTA	204	A	A	A	NA
PMS5657	Os_Chr11	22747979	22747993	(GCA)5	(GCA)5	(GCA)5	(GCA)5	(GCA)5	(GCA)5	(GCA)5	(GCA)5	(GCA)6	(GCA)5	(GCA)5	-	Introns/Intergenic	-	-	CCGATTAGTATCGCAGCACA	CTAAGCTTTGTCGCTCCCAC	189	A	A	A	NA
PMS5658	Os_Chr11	22790900	22790953	(AT)27	(AT)11	(AT)27	(AT)27	(AT)27	(AT)27	(AT)27	(AT)27	(AT)27	(AT)27	(AT)27	-	Introns/Intergenic	-	-	GGGAATTTCTTGTCTCCACAACG	TTTCCCTCTTTCGTTGGTATGC	242	P	A	A	RM27014
PMS5659	Os_Chr11	22794329	22794416	(AT)44	(AT)34	(AT)44	(AT)44	(AT)44	(AT)44	(AT)44	(AT)14	(AT)44	(AT)44	(AT)15	-	Introns/Intergenic	-	-	TGATATCCCGCACTTTGATT	TCATGCAGCATCATCTAATTCA	232	P	A	A	NA
PMS5660	Os_Chr11	22844340	22844360	(GGC)7	(GGC)6	(GGC)7	(GGC)7	(GGC)7	(GGC)7	(GGC)7	(GGC)7	(GGC)7	(GGC)7	(GGC)7	LOC_Os11g38550	CDS	transposon protein, putative, CACTA, En/Spm sub-class	CODON_DELETION	GTTCTCGTTCTCGTTCGTTA	TCCGATCTCGATCTCTCTC	434	P	A	A	NA
PMS5661	Os_Chr11	22864004	22864021	(GAG)6	(GAG)7	(GAG)6	(GAG)6	(GAG)6	(GAG)6	(GAG)6	(GAG)7	(GAG)6	(GAG)7	(GAG)6	LOC_Os11g38580	CDS	NBS-LRR type disease resistance protein, putative, expressed	CODON_INSERTION	AGCAGGAGCACAAGAAGGAA	GATGAATCGGGTCGTCATCT	213	P	A	A	NA
PMS5662	Os_Chr11	22910631	22910654	(CGC)8	(CGC)8	(CGC)8	(CGC)8	(CGC)8	(CGC)8	(CGC)8	(CGC)5	(CGC)8	(CGC)8	(CGC)8	-	Introns/Intergenic	-	-	AAATACCCTCCTCTCGCCTC	CCTCCTCGCGGTACATCC	239	A	A	A	NA
PMS5663	Os_Chr11	22924135	22924148	(CA)7	(CA)7	(CA)7	(CA)7	(CA)7	(CA)6	(CA)7	(CA)7	(CA)7	(CA)7	(CA)7	-	Introns/Intergenic	-	-	ACGTTGATAGGCGACCATTC	CTTGTGTTGACGACATTGGG	172	P	A	A	NA
PMS5664	Os_Chr11	23066177	23066197	(CCG)7	(CCG)7	(CCG)7	(CCG)7	(CCG)7	(CCG)7	(CCG)7	(CCG)7	(CCG)7	(CCG)5	(CCG)6	LOC_Os11g38800	URR	zinc finger family protein, putative, expressed	-	GAGTCGAGACGACCAAAAGC	CCACACACATCAACGGACTC	236	A	A	A	NA
PMS5665	Os_Chr11	23077640	23077654	(CCA)5	(CCA)5	(CCA)5	(CCA)5	(CCA)5	(CCA)6	(CCA)5	(CCA)5	(CCA)5	(CCA)5	(CCA)6	LOC_Os11g38810	CDS	mannose-6-phosphate isomerase, putative, expressed	CODON_INSERTION	GACGACGACGATCGAAATG	GTACGGCCTCTCATCACCAT	274	P	A	A	NA
PMS5666	Os_Chr11	23106025	23106046	(TA)11	(TA)11	(TA)11	(TA)11	(TA)11	(TA)11	(TA)11	(TA)6	(TA)11	(TA)11	(TA)11	-	Introns/Intergenic	-	-	CCTGACTGCACAGAATTGGA	CACCAACTGTCTGGCTATGC	205	A	A	A	NA
PMS5667	Os_Chr11	23155759	23155792	(TA)17	(TA)17	(TA)17	(TA)17	(TA)17	(TA)17	(TA)17	(TA)17	(TA)8	(TA)17	(TA)17	-	Introns/Intergenic	-	-	AATGGCTATGAACTCTCGTGTGC	AGGGCCTGTGTGCATAGAATACG	121	A	A	A	RM27033

Polymorphic SSR (PMS) markers	Chromosomes	Start physical positions (bp)	End physical positions (bp)	Nipponbare	Kasalath	Nagina22	IR64	Pokkali	Bala	Tainung67	Azucena	Moroberekan	*Oryza rufipogon*	*Oryza nivara*	MSU gene locus IDs	Structural annotation	Functional annotation	SSR functional effects	Forward primers (5'-3')	Reverse primers (5'-3')	Amplified product size (bp)	Markers exhibiting polymorphism within *indica* rice	Transcrption factor genes-derived markers	Known cloned genes-derived markers	PMS markers corresponding with RM markers
PMS5668	Os_Chr11	23205907	23205926	(TA)10	(TA)10	(TA)10	(TA)8	(TA)10	(TA)10	(TA)10	(TA)10	(TA)10	(TA)10	(TA)10	-	Introns/Intergenic	-	-	CCGTGTAACAATGGTAATGCACTGG	TTCATCGACCTTCTGCCTTCATCC	418	P	A	A	RM27036
PMS5669	Os_Chr11	23230354	23230371	(CCA)6	(CCA)6	(CCA)6	(CCA)6	(CCA)6	(CCA)5	(CCA)6	(CCA)6	(CCA)5	(CCA)6	(CCA)6	LOC_Os11g39020	CDS	ABC transporter, ATP-binding protein, putative, expressed	CODON_DELETION	CTCCTCTCCTCCAAGCTCCT	CTGCTATTCGACCGCTTCTT	236	P	A	A	NA
PMS5670	Os_Chr11	23443407	23443420	(TC)7	(TC)6	(TC)7	(TC)7	(TC)7	(TC)7	(TC)7	(TC)7	(TC)6	(TC)6	(TC)7	-	Introns/Intergenic	-	-	CCTTGACCAAACCCTATCCA	GCTGTTTCCTCCGCTCCT	225	P	A	A	NA
PMS5671	Os_Chr11	23472491	23472580	(AAT)30	(AAT)30	(AAT)30	(AAT)30	(AAT)12	(AAT)30	(AAT)30	(AAT)30	(AAT)30	(AAT)30	(AAT)30	-	Introns/Intergenic	-	-	TTTCCAACTGGCACCGATTAGG	AATACCGACGGGTAGGATCTAGAGG	554	P	A	A	RM5716
PMS5672	Os_Chr11	23574265	23574296	(TA)16	(TA)6	(TA)16	(TA)16	(TA)16	(TA)16	(TA)16	(TA)16	(TA)10	(TA)16	(TA)16	-	Introns/Intergenic	-	-	CCCCGTCAAGTAGTTTTCCA	ATTGACGGGATGGTGAGAAG	161	P	A	A	NA
PMS5673	Os_Chr11	23594047	23594078	(TA)16	(TA)6	(TA)16	(TA)16	(TA)16	(TA)16	(TA)16	(TA)16	(TA)10	(TA)16	(TA)16	-	Introns/Intergenic	-	-	GGCTAATCCTTCCCTATGTATGTACG	TTCTTCGACAACCTCGTCAGG	200	P	A	A	RM27049
PMS5674	Os_Chr11	23611515	23611532	(CGA)6	(CGA)6	(CGA)6	(CGA)6	(CGA)6	(CGA)6	(CGA)6	(CGA)6	(CGA)6	(CGA)6	(CGA)5	LOC_Os11g39640	CDS	zinc finger, C3HC4 type domain containing protein, expressed	CODON_DELETION	GCTGGTGTGTCGGCTTATG	GTACCACACGTGGTGAACCC	214	A	P	A	NA
PMS5675	Os_Chr11	23611656	23611679	(CGG)8	(CGG)8	(CGG)8	(CGG)8	(CGG)8	(CGG)8	(CGG)6	(CGG)8	(CGG)8	(CGG)8	(CGG)8	LOC_Os11g39640	CDS	zinc finger, C3HC4 type domain containing protein, expressed	CODON_CHANGE_PLUS_CODON_DELETION	TGTACGACGCCAAGAAGACC	GGACTTGTCGCTGATCTGG	227	A	P	A	NA
PMS5676	Os_Chr11	23787597	23787610	(AT)7	(AT)7	(AT)7	(AT)6	(AT)6	(AT)6	(AT)7	(AT)7	(AT)6	(AT)7	(AT)6	-	Introns/Intergenic	-	-	CCGATACATGTGTGTCAAGATTT	ACATCGCTGCGAATTCATTA	266	P	A	A	NA
PMS5677	Os_Chr11	23840538	23840585	(TA)24	(TA)24	(TA)24	(TA)24	(TA)24	(TA)24	(TA)7	(TA)9	(TA)24	(TA)24	(TA)24	-	Introns/Intergenic	-	-	GTCCATGTGGCACAATACTCAGG	TTAGCCATAAGGAGGTGACATTGG	404	A	A	A	RM27061
PMS5678	Os_Chr11	23947200	23947217	(CGG)6	(CGG)6	(CGG)6	(CGG)6	(CGG)6	(CGG)7	(CGG)6	(CGG)6	(CGG)6	(CGG)6	(CGG)6	LOC_Os11g40160	URR	expressed protein	-	GAGAAGGAGAGGCGAAGGAT	AGATTGCTGCCAGATTGTCC	267	P	A	A	NA
PMS5679	Os_Chr11	24178750	24178764	(GAT)5	(GAT)5	(GAT)5	(GAT)5	(GAT)5	(GAT)6	(GAT)5	(GAT)5	(GAT)5	(GAT)7	(GAT)5	-	Introns/Intergenic	-	-	GTGACGTCGCCGCTATAAAT	CCAACAACGACATTGACGAC	121	P	A	A	NA
PMS5680	Os_Chr11	24184552	24184565	(TC)7	(TC)7	(TC)7	(TC)6	(TC)6	(TC)6	(TC)7	(TC)7	(TC)7	(TC)7	(TC)7	-	Introns/Intergenic	-	-	TATATTTTGCGACGGAGGGA	ATGGAAGCTGCTGATTTGCT	151	P	A	A	NA

Polymorphic SSR (PMS) markers	Chromosomes	Start physical positions (bp)	End physical positions (bp)	Nipponbare	Kasalath	Nagina22	IR64	Pokkali	Bala	Tainung67	Azucena	Moroberekan	*Oryza rufipogon*	*Oryza nivara*	MSU gene locus IDs	Structural annotation	Functional annotation	SSR functional effects	Forward primers (5'-3')	Reverse primers (5'-3')	Amplified product size (bp)	Markers exhibiting polymorphism within *indica* rice	Transcrption factor genes-derived markers	Known cloned genes-derived markers	PMS markers corresponding with RM markers
PMS5681	Os_Chr11	24199990	24200007	(AT)9	(AT)9	(AT)9	(AT)9	(AT)9	(AT)9	(AT)9	(AT)9	(AT)9	(AT)8	(AT)9	-	Introns/Intergenic	-	-	GCGTTGTAACGGGTGAAGTA	CCGAAGGGAGCCCTATTATC	143	A	A	A	NA
PMS5682	Os_Chr11	24200201	24200236	(TA)18	(TA)18	(TA)8	(TA)18	(TA)18	(TA)17	(TA)18	(TA)18	(TA)9	(TA)18	(TA)18	-	Introns/Intergenic	-	-	GTGGATGGGAGAGGGAGTACG	TCCGTTGCCACAATTCATCC	299	P	A	A	RM11878
PMS5683	Os_Chr11	24214077	24214094	(CGG)6	(CGG)6	(CGG)6	(CGG)6	(CGG)6	(CGG)6	(CGG)6	(CGG)6	(CGG)6	(CGG)6	(CGG)5	-	Introns/Intergenic	-	-	TCGTCACGACACCAAGAAAC	TCTCGTCCCCATCATAAAGC	154	A	A	A	NA
PMS5684	Os_Chr11	24227572	24227586	(GCC)5	(GCC)5	(GCC)5	(GCC)5	(GCC)5	(GCC)5	(GCC)5	(GCC)5	(GCC)5	(GCC)6	(GCC)5	LOC_Os11g40600	CDS	expressed protein	CODON_INSERTION	ACAAGCATGGTCATGGTCAA	CCATAAGCAAGAAGAAGCCG	260	A	A	A	NA
PMS5685	Os_Chr11	24233551	24233571	(CTG)7	(CTG)7	(CTG)7	(CTG)7	(CTG)7	(CTG)7	(CTG)7	(CTG)7	(CTG)7	(CTG)7	(CTG)6	-	Introns/Intergenic	-	-	TGCAAAGTGCAAAGCGTAAAGC	TAGCAGCAGCAGTAGGAACAAGAAGG	169	A	A	A	RM27088
PMS5686	Os_Chr11	24268616	24268630	(CCT)5	(CCT)5	(CCT)5	(CCT)5	(CCT)5	(CCT)5	(CCT)5	(CCT)5	(CCT)6	(CCT)5	(CCT)5	-	Introns/Intergenic	-	-	CCTCTCCATGCCATACCTGT	CGAGGAGACAATCCATGTCC	187	A	A	A	NA
PMS5687	Os_Chr11	24274440	24274453	(TA)7	(TA)8	(TA)7	(TA)7	(TA)7	(TA)7	(TA)7	(TA)7	(TA)7	(TA)7	(TA)7	-	Introns/Intergenic	-	-	CGATTTTTCTTGAATGGGGA	GAACGGACGGAAGAAACAGA	198	P	A	A	NA
PMS5688	Os_Chr11	24276424	24276457	(AT)17	(AT)17	(AT)17	(AT)17	(AT)17	(AT)17	(AT)7	(AT)17	(AT)17	(AT)17	(AT)17	-	Introns/Intergenic	-	-	AAAGTCTGTTCTAGGTTCCTCAGC	GGAAGATAACAGCTTGGTGTGC	177	A	A	A	RM27092
PMS5689	Os_Chr11	24332567	24332676	(TA)55	(TA)55	(TA)7	(TA)14	(TA)14	(TA)14	(TA)55	(TA)12	(TA)55	(TA)55	(TA)55	-	Introns/Intergenic	-	-	AGTTAGGATCGCTTCCAGGTTCC	TCCAACTGGAATATCGTCTTGTAGGC	198	P	A	A	RM27096
PMS5690	Os_Chr11	24541248	24541265	(GA)9	(GA)9	(GA)9	(GA)9	(GA)9	(GA)9	(GA)9	(GA)9	(GA)9	(GA)9	(GA)8	-	Introns/Intergenic	-	-	CTCTCCCTCTTCCCCTTCTC	CGGCTACTTCCTCCTTTCCT	276	A	A	A	NA
PMS5691	Os_Chr11	24585198	24585271	(AT)37	(AT)37	(AT)37	(AT)37	(AT)37	(AT)37	(AT)37	(AT)14	(AT)34	(AT)37	(AT)37	-	Introns/Intergenic	-	-	GCTATAGCTACCGAGTAAATGAATTG	CTGTTTGTTGCATGGTTGATT	280	A	A	A	NA
PMS5692	Os_Chr11	24595548	24595567	(TA)10	(TA)10	(TA)10	(TA)10	(TA)10	(TA)11	(TA)10	(TA)10	(TA)10	(TA)10	(TA)10	-	Introns/Intergenic	-	-	AAGGTGATCAGGACTGTGCATGG	AAGGGCCGCAGAACACATAAACC	185	P	A	A	RM27114
PMS5693	Os_Chr11	24601698	24601733	(TATG)9	(TATG)9	(TATG)9	(TATG)9	(TATG)9	(TATG)5	(TATG)9	(TATG)9	(TATG)9	(TATG)9	(TATG)9	-	Introns/Intergenic	-	-	CTCCCTCCACCGGCTTCTTAGC	GGTAGTTGGACGGCAGGGATGG	255	P	A	A	RM27115

Polymorphic SSR (PMS) markers	Chromosomes	Start physical positions (bp)	End physical positions (bp)	Nipponbare	Kasalath	Nagina22	IR64	Pokkali	Bala	Tainung67	Azucena	Moroberekan	*Oryza rufipogon*	*Oryza nivara*	MSU gene locus IDs	Structural annotation	Functional annotation	SSR functional effects	Forward primers (5'-3')	Reverse primers (5'-3')	Amplified product size (bp)	Markers exhibiting polymorphism within *indica* rice	Transcrption factor genes-derived markers	Known cloned genes-derived markers	PMS markers corresponding with RM markers
PMS5694	Os_Chr11	24639297	24639311	(CCG)5	(CCG)5	(CCG)5	(CCG)5	(CCG)5	(CCG)5	(CCG)5	(CCG)5	(CCG)5	(CCG)6	(CCG)5	-	Introns/Intergenic	-	-	CATCAAGGACATCCACATCG	CTATCCCTCGGCTTCTCCTC	276	A	A	A	NA
PMS5695	Os_Chr11	24779482	24779557	(TA)38	(TA)38	(TA)30	(TA)38	(TA)7	(TA)38	(TA)6	(TA)38	(TA)38	(TA)38	(TA)11	-	Introns/Intergenic	-	-	ATGATTTGTTTTGCGACGTG	TCAATTTCAACAATGGTGCC	207	P	A	A	NA
PMS5696	Os_Chr11	24779951	24779972	(TA)11	(TA)11	(TA)7	(TA)11	(TA)7	(TA)7	(TA)11	(TA)11	(TA)11	(TA)11	(TA)11	-	Introns/Intergenic	-	-	ATGGTGCCCCATATGAGTGT	AGCAAATGTGGTGTGCGTAG	174	P	A	A	NA
PMS5697	Os_Chr11	24810800	24810814	(CGG)5	(CGG)6	(CGG)6	(CGG)5	(CGG)5	(CGG)5	(CGG)5	(CGG)5	(CGG)6	(CGG)5	(CGG)5	LOC_Os11g41350	CDS	MBTB67 - Bric-a-Brac, Tramtrack, Broad Complex BTB domain with Meprin and TRAF Homology MATH domain, expressed	CODON_INSERTION	TAGCACACCCTCTCGACCTT	CGGACGTACGTATCCAATCC	162	P	A	A	NA
PMS5698	Os_Chr11	25041022	25041036	(CTT)5	(CTT)5	(CTT)5	(CTT)5	(CTT)5	(CTT)5	(CTT)5	(CTT)5	(CTT)6	(CTT)5	(CTT)5	LOC_Os11g41710	CDS	cytochrome P450, putative, expressed	CODON_INSERTION	CAAGAGCAAACGAAATGCAA	GAGAGCCCATGGAGATGGT	235	A	A	A	NA
PMS5699	Os_Chr11	25103810	25103842	(TTC)11	(TTC)14	(TTC)11	(TTC)11	(TTC)11	(TTC)14	(TTC)11	(TTC)11	(TTC)11	(TTC)11	(TTC)16	LOC_Os11g41780	CDS	transposon protein, putative, CACTA, En/Spm sub-class, expressed	CODON_INSERTION	TGTGGCCCTTCATCTTCTTC	TTATTGACCTTGGGGCTGAC	208	P	A	A	NA
PMS5700	Os_Chr11	25271517	25271586	(AT)35	(AT)35	(AT)7	(AT)35	(AT)35	(AT)9	(AT)35	(AT)35	(AT)35	(AT)11	(AT)35	-	Introns/Intergenic	-	-	GAAGAAGCGTACGCATGAAT	AAGTCCTCCTCCTCCCTAGAT	280	P	A	A	NA
PMS5701	Os_Chr11	25321694	25321708	(CTC)5	(CTC)5	(CTC)5	(CTC)5	(CTC)5	(CTC)7	(CTC)5	(CTC)5	(CTC)5	(CTC)5	(CTC)5	-	Introns/Intergenic	-	-	CGGCATAGTTGAGCACTTGA	GCTATACACCTCCAGCTCCG	217	P	A	A	NA
PMS5702	Os_Chr11	25356938	25356988	(GAT)17	(GAT)17	(GAT)17	(GAT)17	(GAT)17	(GAT)17	(GAT)17	(GAT)17	(GAT)9	(GAT)17	(GAT)17	-	Introns/Intergenic	-	-	GATAGTGATTGCTGGTCTATCG	ATATTGTCTCAGTTGGGTCTGC	209	A	A	A	RM6440
PMS5703	Os_Chr11	25439279	25439300	(AT)11	(AT)11	(AT)8	(AT)11	(AT)11	(AT)11	(AT)11	(AT)11	(AT)8	(AT)11	(AT)11	-	Introns/Intergenic	-	-	CCTTGTGATGCTAACCGAATAGG	TATTTAGACGGACGATGGAAGC	300	P	A	A	RM27165
PMS5704	Os_Chr11	25442117	25442137	(GCG)7	(GCG)7	(GCG)7	(GCG)7	(GCG)7	(GCG)7	(GCG)5	(GCG)7	(GCG)8	(GCG)7	(GCG)7	LOC_Os11g42240	CDS	OsFBX431 - F-box domain containing protein, expressed	CODON_INSERTION	GGTGGTTGTAGCTCTGGTTGTCG	GAGGAGCCCAGTACCGACTTAGG	298	A	A	A	RM27166
PMS5705	Os_Chr11	25492284	25492301	(CCA)6	(CCA)6	(CCA)5	(CCA)6	(CCA)6	(CCA)6	(CCA)6	(CCA)6	(CCA)6	(CCA)6	(CCA)6	LOC_Os11g42350	CDS	glutathione synthetase, chloroplast precursor, putative, expressed	CODON_CHANGE_PLUS_CODON_DELETION	AATTCATCGCCAATGTCCTC	CTCCTCCGCCATCTCGTC	261	P	A	A	NA
PMS5706	Os_Chr11	25551835	25551852	(CCA)6	(CCA)6	(CCA)6	(CCA)6	(CCA)6	(CCA)6	(CCA)6	(CCA)6	(CCA)6	(CCA)5	(CCA)6	-	Introns/Intergenic	-	-	AGCTCCACTGAGAGAGCTGC	GCTTGGAGGGAGAAGGCTAT	270	A	A	A	NA

Polymorphic SSR (PMS) markers	Chromosomes	Start physical positions (bp)	End physical positions (bp)	Nipponbare	Kasalath	Nagina22	IR64	Pokkali	Bala	Tainung67	Azucena	Moroberekan	*Oryza rufipogon*	*Oryza nivara*	MSU gene locus IDs	Structural annotation	Functional annotation	SSR functional effects	Forward primers (5'-3')	Reverse primers (5'-3')	Amplified product size (bp)	Markers exhibiting polymorphism within *indica* rice	Transcrption factor genes-derived markers	Known cloned genes-derived markers	PMS markers corresponding with RM markers
PMS5707	Os_Chr11	25772788	25772844	(TTA)19	(TTA)19	(TTA)7	(TTA)19	(TTA)7	(TTA)19	(TTA)9	(TTA)19	(TTA)19	(TTA)8	(TTA)19	-	Introns/Intergenic	-	-	AAGAAGAGAAGGGATGGGATCTGG	TCTAAACAGGGCCTCAAACTGTATCC	327	P	A	A	RM27180
PMS5708	Os_Chr11	25779864	25779883	(AG)10	(AG)10	(AG)10	(AG)10	(AG)10	(AG)10	(AG)10	(AG)13	(AG)13	(AG)10	(AG)10	-	Introns/Intergenic	-	-	CAATTCAGAGGAGCAAGGTGTCC	TTCTTAACCTGGACTTGCCATGC	173	A	A	A	RM27181
PMS5709	Os_Chr11	25780581	25780592	(GA)6	(GA)7	(GA)6	(GA)6	(GA)7	(GA)7	(GA)6	(GA)6	(GA)6	(GA)6	(GA)6	-	Introns/Intergenic	-	-	TGCCATCACCAATGAAATGT	TTCATGGTGGGAATAGATTCAA	212	P	A	A	NA
PMS5710	Os_Chr11	25831478	25831513	(CT)18	(CT)18	(CT)18	(CT)18	(CT)18	(CT)17	(CT)18	(CT)18	(CT)18	(CT)18	(CT)18	-	Introns/Intergenic	-	-	GATCAGGAAGCCCAGACACTTCG	ACACCAAGTGCAGTTCCAAGTTCC	418	P	A	A	RM27187
PMS5711	Os_Chr11	25841142	25841155	(AG)7	(AG)7	(AG)6	(AG)7	(AG)6	(AG)6	(AG)7	(AG)7	(AG)7	(AG)7	(AG)6	-	Introns/Intergenic	-	-	GCGAGTTTGAAGAGTCGGTC	CCTCTGCAGCTTGCACACTA	274	P	A	A	NA
PMS5712	Os_Chr11	25847134	25847163	(TTTTC)6	(TTTTC)6	(TTTTC)6	(TTTTC)5	(TTTTC)6	(TTTTC)6	(TTTTC)6	(TTTTC)6	(TTTTC)6	(TTTTC)5	(TTTTC)6	-	Introns/Intergenic	-	-	CAAGTGCAAAAAGGAGGAGG	TGTTTAAGTGATTTGGGCTGAA	125	P	A	A	NA
PMS5713	Os_Chr11	25867781	25867802	(TA)11	(TA)11	(TA)11	(TA)11	(TA)6	(TA)11	(TA)11	(TA)11	(TA)11	(TA)11	(TA)11	-	Introns/Intergenic	-	-	AAGGGTGGTTCGTTCAGAAATCC	TGACACATGAGCGAAACTTTAGGG	374	P	A	A	RM27190
PMS5714	Os_Chr11	25896693	25896716	(ACG)8	(ACG)8	(ACG)8	(ACG)8	(ACG)8	(ACG)8	(ACG)8	(ACG)8	(ACG)8	(ACG)7	(ACG)8	LOC_Os11g42970	URR	membrane associated DUF588 domain containing protein, putative, expressed	-	CGGGAAGTCCTTGTAGGTGATGG	TCTGGATCATCATGTCACCATGC	373	A	A	A	RM27191
PMS5715	Os_Chr11	25904478	25904507	(AT)15	(AT)15	(AT)15	(AT)12	(AT)15	(AT)15	(AT)11	(AT)15	(AT)6	(AT)15	(AT)15	-	Introns/Intergenic	-	-	TTTTGAAGTATGGATGGGGC	TTTTCCTGGGGTCGATACTG	124	P	A	A	NA
PMS5716	Os_Chr11	25934575	25934606	(AT)16	(AT)16	(AT)11	(AT)16	(AT)9	(AT)16	(AT)13	(AT)16	(AT)16	(AT)16	(AT)16	-	Introns/Intergenic	-	-	CATGCGTCTCACCAAGCTAAACC	CAATTCATAACGTCGGTCCTTCC	284	P	A	A	RM27199
PMS5717	Os_Chr11	25950259	25950272	(TA)7	(TA)7	(TA)7	(TA)7	(TA)7	(TA)7	(TA)7	(TA)7	(TA)7	(TA)7	(TA)9	-	Introns/Intergenic	-	-	TGTACATGGCAGCATGTGTG	TTGCAAATCAAGTGCAAACAG	186	A	A	A	NA
PMS5718	Os_Chr11	25999467	25999480	(CG)7	(CG)7	(CG)7	(CG)7	(CG)7	(CG)6	(CG)7	(CG)7	(CG)7	(CG)7	(CG)7	-	Introns/Intergenic	-	-	TCCCTCCCTCTCTCTCTTCC	GAGAGGAGGGCGATGACAG	123	P	A	A	NA
PMS5719	Os_Chr11	26058205	26058222	(AAT)6	(AAT)6	(AAT)6	(AAT)6	(AAT)6	(AAT)6	(AAT)6	(AAT)5	(AAT)5	(AAT)6	(AAT)6	-	Introns/Intergenic	-	-	CAAGTGAATTCCCACGATGA	GGAGGCGTCAAGACTGACAT	255	A	A	A	NA

Polymorphic SSR (PMS) markers	Chromosomes	Start physical positions (bp)	End physical positions (bp)	Nipponbare	Kasalath	Nagina22	IR64	Pokkali	Bala	Tainung67	Azucena	Moroberekan	*Oryza rufipogon*	*Oryza nivara*	MSU gene locus IDs	Structural annotation	Functional annotation	SSR functional effects	Forward primers (5'-3')	Reverse primers (5'-3')	Amplified product size (bp)	Markers exhibiting polymorphism within *indica* rice	Transcrption factor genes-derived markers	Known cloned genes-derived markers	PMS markers corresponding with RM markers
PMS5720	Os_Chr11	26084840	26084889	(TA)25	(TA)10	(TA)10	(TA)25	(TA)25	(TA)25	(TA)25	(TA)11	(TA)8	(TA)25	(TA)25	-	Introns/Intergenic	-	-	GTGGATGGCCGGTTATGATTGG	GACACCGGCTGCTTCTTCTTTCC	237	P	A	A	RM27207
PMS5721	Os_Chr11	26127861	26127888	(TGTC)7	(TGTC)7	(TGTC)7	(TGTC)7	(TGTC)7	(TGTC)7	(TGTC)7	(TGTC)6	(TGTC)7	(TGTC)7	(TGTC)7	-	Introns/Intergenic	-	-	CTGTGTGTGAGTGGTGGGTC	AGCCTCCATAGCAAACATGA	274	A	A	A	NA
PMS5722	Os_Chr11	26155114	26155155	(AT)21	(AT)21	(AT)7	(AT)21	(AT)16	(AT)21	(AT)21	(AT)9	(AT)6	(AT)16	(AT)21	-	Introns/Intergenic	-	-	CGACAACCTTGAAGCACTCA	ATTAGATACCATGGTGCCCG	127	P	A	A	NA
PMS5723	Os_Chr11	26276230	26276243	(GA)7	(GA)7	(GA)7	(GA)7	(GA)7	(GA)8	(GA)7	(GA)7	(GA)7	(GA)7	(GA)7	LOC_Os11g43530	DRR	OsGrx_C13 - glutaredoxin subgroup III, expressed	-	TGCACTGTGGCTTTGAGATT	CGTGTACAATCGCAGTGAGAA	262	P	A	A	NA
PMS5724	Os_Chr11	26276390	26276415	(TA)13	(TA)13	(TA)13	(TA)13	(TA)13	(TA)13	(TA)13	(TA)12	(TA)13	(TA)13	(TA)13	-	Introns/Intergenic	-	-	TTATCACTTCTCACTGCGATT	ACACATACACATACACATACACA	223	A	A	A	NA
PMS5725	Os_Chr11	26336803	26336846	(TA)22	(TA)22	(TA)22	(TA)22	(TA)22	(TA)17	(TA)7	(TA)22	(TA)22	(TA)19	(TA)6	-	Introns/Intergenic	-	-	GGTCTATGAAGTGTGGACTGTGAACC	GCTGGCTAATTTATAGCCCATACAGG	604	P	A	A	RM27223
PMS5726	Os_Chr11	26389670	26389701	(TA)16	(TA)16	(TA)16	(TA)7	(TA)16	(TA)16	(TA)6	(TA)16	(TA)16	(TA)8	(TA)7	-	Introns/Intergenic	-	-	TTGGCGATCGGTACATAAATCG	GTATGCAAGGTTGGTGTCCTTGG	428	P	A	A	RM27227
PMS5727	Os_Chr11	26414176	26414193	(TCG)6	(TCG)6	(TCG)6	(TCG)6	(TCG)6	(TCG)6	(TCG)6	(TCG)6	(TCG)6	(TCG)7	(TCG)6	-	Introns/Intergenic	-	-	CTTCTCCTTCTCCCTCTCCC	CCAATCGATAATCCCAGGTG	263	A	A	A	NA
PMS5728	Os_Chr11	26450265	26450284	(ATAG)5	(ATAG)7	(ATAG)5	(ATAG)8	(ATAG)5	(ATAG)5	(ATAG)5	(ATAG)5	(ATAG)5	(ATAG)8	(ATAG)5	-	Introns/Intergenic	-	-	TGGGAATTTCTAGCCTCCATAACG	CAAGATCAAGCGAGGAAAGAACC	195	P	A	A	RM27229
PMS5729	Os_Chr11	26453625	26453642	(GGA)6	(GGA)6	(GGA)6	(GGA)6	(GGA)6	(GGA)6	(GGA)6	(GGA)6	(GGA)6	(GGA)5	(GGA)6	LOC_Os11g43790	CDS	DUF581 domain containing protein, expressed	CODON_CHANGE_PLUS_CODON_DELETION	CGATCTCGTCTCTCCATCGT	GCGACCAACTTAGTACGCAA	247	A	A	A	NA
PMS5730	Os_Chr11	26518197	26518216	(TA)10	(TA)10	(TA)10	(TA)9	(TA)10	(TA)10	(TA)10	(TA)10	(TA)10	(TA)10	(TA)10	-	Introns/Intergenic	-	-	GATTATTCCCCTCGGAAAGG	AGAGGCTGTGCTGTCCTGTT	249	P	A	A	NA
PMS5731	Os_Chr11	26562718	26562741	(CTG)8	(CTG)8	(CTG)8	(CTG)8	(CTG)8	(CTG)8	(CTG)8	(CTG)6	(CTG)8	(CTG)8	(CTG)8	LOC_Os11g43970	CDS	AAA-type ATPase family protein, putative, expressed	CODON_DELETION	CCCATGTACCTGTGAGGACTGC	GTTAGGGTTCTGATGCTTTGTTGC	166	A	A	A	RM27233
PMS5732	Os_Chr11	26580852	26580863	(TA)6	(TA)6	(TA)6	(TA)6	(TA)6	(TA)6	(TA)7	(TA)6	(TA)6	(TA)6	(TA)7	-	Introns/Intergenic	-	-	CGAAATTGGACACGGAAACT	CCCTCTCCTTGCTCACAGTC	158	A	A	A	NA

Polymorphic SSR (PMS) markers	Chromosomes	Start physical positions (bp)	End physical positions (bp)	Nipponbare	Kasalath	Nagina22	IR64	Pokkali	Bala	Tainung67	Azucena	Moroberekan	*Oryza rufipogon*	*Oryza nivara*	MSU gene locus IDs	Structural annotation	Functional annotation	SSR functional effects	Forward primers (5'-3')	Reverse primers (5'-3')	Amplified product size (bp)	Markers exhibiting polymorphism within *indica* rice	Transcrption factor genes-derived markers	Known cloned genes-derived markers	PMS markers corresponding with RM markers
PMS5733	Os_Chr11	26585404	26585419	(TG)8	(TG)8	(TG)8	(TG)8	(TG)8	(TG)8	(TG)8	(TG)8	(TG)8	(TG)8	(TG)7	-	Introns/Intergenic	-	-	TCCACAGACATGAAAGACCAA	CCAACCGAGTCGCAAATAAT	252	A	A	A	NA
PMS5734	Os_Chr11	26635006	26635026	(CGC)7	(CGC)6	(CGC)7	(CGC)7	(CGC)7	(CGC)7	(CGC)7	(CGC)7	(CGC)7	(CGC)7	(CGC)7	-	Introns/Intergenic	-	-	AGATCTCCCTTTATGCATCTC	TGTTCGTTCTCGTTCGTTA	381	P	A	A	NA
PMS5735	Os_Chr11	26878770	26878787	(CGA)6	(CGA)6	(CGA)5	(CGA)6	(CGA)6	(CGA)5	(CGA)6	(CGA)6	(CGA)6	(CGA)5	(CGA)6	LOC_Os11g44430	CDS	protein kinase, putative, expressed	CODON_DELETION	GCTTGATGTCACGGTGGATA	CTGTGATGTGTTGGTCGAGG	263	P	A	A	NA
PMS5736	Os_Chr11	26900173	26900196	(GCC)8	(GCC)8	(GCC)8	(GCC)8	(GCC)8	(GCC)8	(GCC)8	(GCC)8	(GCC)5	(GCC)8	(GCC)8	-	Introns/Intergenic	-	-	GCCTGTATGCCATCGATTTT	TCTCCCTCCCTCCTAGATCC	214	A	A	A	NA
PMS5737	Os_Chr11	26922254	26922265	(CT)6	(CT)7	(CT)7	(CT)6	(CT)6	(CT)6	(CT)6	(CT)6	(CT)6	(CT)7	(CT)6	-	Introns/Intergenic	-	-	CTTTGTTTCTCGCCACCATT	TTTCATCGGGAGAACGAATC	205	P	A	A	NA
PMS5738	Os_Chr11	26997868	26997881	(GA)7	(GA)7	(GA)7	(GA)6	(GA)7	(GA)7	(GA)7	(GA)7	(GA)7	(GA)7	(GA)7	-	Introns/Intergenic	-	-	AGAGAGAGGTGGGGAGAAGC	TTTTTCCCTTTCCTTTTTCTCTC	138	P	A	A	NA
PMS5739	Os_Chr11	27029776	27029799	(ATCC)6	(ATCC)6	(ATCC)6	(ATCC)6	(ATCC)6	(ATCC)6	(ATCC)6	(ATCC)5	(ATCC)6	(ATCC)6	(ATCC)6	-	Introns/Intergenic	-	-	AAGCGACGACGAAGCTACCTACC	GGTGATCAGACAACGAGCTTCC	394	A	A	A	RM7240
PMS5740	Os_Chr11	27278630	27278647	(GA)9	(GA)9	(GA)9	(GA)9	(GA)9	(GA)9	(GA)9	(GA)9	(GA)9	(GA)8	(GA)9	-	Introns/Intergenic	-	-	GCAGAACGCAGAGCAGAGA	CGGCTGAGTTAGCGTAGGAC	133	A	A	A	NA
PMS5741	Os_Chr11	27310094	27310107	(AG)7	(AG)7	(AG)7	(AG)7	(AG)7	(AG)7	(AG)7	(AG)7	(AG)8	(AG)7	(AG)7	-	Introns/Intergenic	-	-	GAGAGGGAGGAGGAGGGAGT	AAGTCTGGCCCAAAGTCCTC	142	A	A	A	NA
PMS5742	Os_Chr11	27355939	27355982	(TC)22	(TC)21	(TC)22	(TC)22	(TC)13	(TC)22	(TC)22	(TC)22	(TC)22	(TC)22	(TC)22	-	Introns/Intergenic	-	-	AGTTCCAAGCCAACCTTCCT	TCTTCGTCAAGGAAAAGCGT	203	P	A	A	NA
PMS5743	Os_Chr11	27361167	27361204	(TC)19	(TC)18	(TC)19	(TC)19	(TC)7	(TC)19	(TC)19	(TC)19	(TC)19	(TC)19	(TC)19	-	Introns/Intergenic	-	-	AGTTCCAAGCCAACCTTCCT	TCTTCGTCAAGGAAAAGCGT	197	P	A	A	NA
PMS5744	Os_Chr11	27425897	27425910	(AT)7	(AT)7	(AT)6	(AT)7	(AT)7	(AT)9	(AT)7	(AT)7	(AT)7	(AT)7	(AT)7	-	Introns/Intergenic	-	-	ATGCAAGCATGCAACATCAT	TTGTAGATGAAATATAATATCCCGCA	148	P	A	A	NA
PMS5745	Os_Chr11	27487741	27487824	(TA)42	(TA)10	(TA)42	(TA)42	(TA)42	(TA)8	(TA)7	(TA)42	(TA)42	(TA)42	(TA)42	-	Introns/Intergenic	-	-	TTGATGTTGTGGGCTTCTCTACC	TCCAATCACGCGTATTACATGC	281	P	A	A	RM2064

Polymorphic SSR (PMS) markers	Chromosomes	Start physical positions (bp)	End physical positions (bp)	Nipponbare	Kasalath	Nagina22	IR64	Pokkali	Bala	Tainung67	Azucena	Moroberekan	*Oryza rufipogon*	*Oryza nivara*	MSU gene locus IDs	Structural annotation	Functional annotation	SSR functional effects	Forward primers (5'-3')	Reverse primers (5'-3')	Amplified product size (bp)	Markers exhibiting polymorphism within *indica* rice	Transcrption factor genes-derived markers	Known cloned genes-derived markers	PMS markers corresponding with RM markers
PMS5746	Os_Chr11	27497722	27497736	(GCG)5	(GCG)6	(GCG)5	(GCG)5	(GCG)5	(GCG)5	(GCG)5	(GCG)5	(GCG)5	(GCG)5	(GCG)5	-	Introns/Intergenic	-	-	GTACCAGGTGGATGGGGTC	CCTCCTCGCCTTCTACTTCC	100	P	A	A	NA
PMS5747	Os_Chr11	27560101	27560122	(GA)11	(GA)11	(GA)11	(GA)11	(GA)11	(GA)10	(GA)11	(GA)11	(GA)11	(GA)11	(GA)11	-	Introns/Intergenic	-	-	CTGCCTCCTTTCCTTGGAAGC	GATGGGCCGAATTCCTCTCG	355	P	A	A	RM27283
PMS5748	Os_Chr11	27577216	27577239	(CATG)6	(CATG)6	(CATG)6	(CATG)6	(CATG)5	(CATG)6	(CATG)6	(CATG)6	(CATG)6	(CATG)6	(CATG)6	-	Introns/Intergenic	-	-	TAAGCCCATTAATCACCTCACC	GAGCAAGCTAGCTATGTCAGTCG	120	P	A	A	RM27284
PMS5749	Os_Chr11	27711046	27711057	(TA)6	(TA)6	(TA)6	(TA)7	(TA)6	(TA)6	(TA)6	(TA)6	(TA)6	(TA)6	(TA)6	-	Introns/Intergenic	-	-	CTGTAAACCGCAGGACGAAT	GTTTGGTATGTGAAACCGGG	262	P	A	A	NA
PMS5750	Os_Chr11	27728433	27728450	(CCT)6	(CCT)5	(CCT)6	(CCT)6	(CCT)6	(CCT)6	(CCT)6	(CCT)6	(CCT)6	(CCT)6	(CCT)6	-	Introns/Intergenic	-	-	GCCTCCTCTTCCTCCTTCC	CATCATTCAATCGGTTGTGC	256	P	A	A	NA
PMS5751	Os_Chr11	27757786	27757803	(AG)9	(AG)9	(AG)9	(AG)9	(AG)9	(AG)9	(AG)8	(AG)9	(AG)9	(AG)9	(AG)9	-	Introns/Intergenic	-	-	GAGAGTGAGGAAGGGTGGG	AACGGTCTAGATCGATCCCC	219	A	A	A	NA
PMS5752	Os_Chr11	27775236	27775253	(CT)9	(CT)9	(CT)9	(CT)9	(CT)9	(CT)9	(CT)9	(CT)9	(CT)9	(CT)9	(CT)11	-	Introns/Intergenic	-	-	CTGTCACTTCCCCTCTCCTG	CATCTCGAACCGTGTCTCCT	215	A	A	A	NA
PMS5753	Os_Chr11	27901810	27901825	(GC)8	(GC)6	(GC)8	(GC)8	(GC)8	(GC)8	(GC)8	(GC)8	(GC)8	(GC)8	(GC)8	-	Introns/Intergenic	-	-	GGTTAGTGGCTGCAAAAGGA	CCCTGTTGACCAGTTGAGGT	153	P	A	A	NA
PMS5754	Os_Chr11	27991082	27991102	(CCG)7	(CCG)7	(CCG)7	(CCG)7	(CCG)7	(CCG)8	(CCG)7	(CCG)7	(CCG)7	(CCG)7	(CCG)7	LOC_Os11g46220	CDS	hypothetical protein	CODON_INSERTION	CAACCGAGAGGGTGAAGGAAGC	CGATTCCACAGCTGCCAACG	176	P	A	A	RM27299
PMS5755	Os_Chr11	28140468	28140491	(ATAG)6	(ATAG)6	(ATAG)6	(ATAG)6	(ATAG)6	(ATAG)6	(ATAG)6	(ATAG)5	(ATAG)6	(ATAG)6	(ATAG)6	-	Introns/Intergenic	-	-	GAGCGTTTGTAGGAAGTTTCATGG	GCACCACATTGTCAAATCATGG	248	A	A	A	RM27319
PMS5756	Os_Chr11	28175706	28175797	(AT)46	(AT)46	(AT)46	(AT)46	(AT)41	(AT)46	(AT)46	(AT)46	(AT)46	(AT)12	(AT)31	-	Introns/Intergenic	-	-	AGAGCCCATGTAGCTACGCCTTCG	AATCATGCCGGCTGAAATTGTACC	266	P	A	A	RM27322
PMS5757	Os_Chr11	28240742	28240759	(GCT)6	(GCT)6	(GCT)6	(GCT)6	(GCT)6	(GCT)6	(GCT)6	(GCT)6	(GCT)6	(GCT)6	(GCT)5	-	Introns/Intergenic	-	-	ATCATCTTGGCAATGCATCA	GGCAAGGTGAAGGATGTTGT	219	A	A	A	NA
PMS5758	Os_Chr11	28241919	28241938	(CT)10	(CT)8	(CT)10	(CT)10	(CT)10	(CT)10	(CT)10	(CT)10	(CT)10	(CT)10	(CT)10	-	Introns/Intergenic	-	-	AAATCCTTCCCAAATCAAAT	AAGCGGCAGCGAGGGAGGA	193	P	A	A	NA

Polymorphic SSR (PMS) markers	Chromosomes	Start physical positions (bp)	End physical positions (bp)	Nipponbare	Kasalath	Nagina22	IR64	Pokkali	Bala	Tainung67	Azucena	Moroberekan	*Oryza rufipogon*	*Oryza nivara*	MSU gene locus IDs	Structural annotation	Functional annotation	SSR functional effects	Forward primers (5'-3')	Reverse primers (5'-3')	Amplified product size (bp)	Markers exhibiting polymorphism within *indica* rice	Transcrption factor genes-derived markers	Known cloned genes-derived markers	PMS markers corresponding with RM markers
PMS5759	Os_Chr11	28262093	28262106	(AG)7	(AG)8	(AG)7	(AG)7	(AG)7	(AG)7	(AG)7	(AG)7	(AG)7	(AG)7	(AG)7	-	Introns/Intergenic	-	-	AGAGGTCGCGTGAGAGAGAG	TCCCGTGCGGATCTATTAAG	182	P	A	A	NA
PMS5760	Os_Chr11	28283583	28283622	(TA)20	(TA)20	(TA)20	(TA)20	(TA)20	(TA)9	(TA)20	(TA)20	(TA)20	(TA)20	(TA)20	-	Introns/Intergenic	-	-	CAAGCAGGTACTGAGGCGTAGC	TTATGCGGAACTCCTCCTATATCC	247	P	A	A	RM27328
PMS5761	Os_Chr11	28322471	28322498	(TA)14	(TA)14	(TA)13	(TA)14	(TA)14	(TA)14	(TA)14	(TA)14	(TA)14	(TA)11	(TA)14	-	Introns/Intergenic	-	-	CATAGCCGATAAAAACTGGTA	TAGTAAATTTGTTTGGTGCTC	270	P	A	A	NA
PMS5762	Os_Chr11	28330906	28330961	(ATAG)14	(ATAG)14	(ATAG)14	(ATAG)14	(ATAG)14	(ATAG)16	(ATAG)14	(ATAG)14	(ATAG)14	(ATAG)14	(ATAG)12	-	Introns/Intergenic	-	-	CTCGTATTGGGTTCTTGTATGG	AGACTGCTGTTCAGGTGTAATCC	590	P	A	A	RM7228
PMS5763	Os_Chr11	28390743	28390756	(CG)7	(CG)6	(CG)7	(CG)7	(CG)7	(CG)7	(CG)7	(CG)7	(CG)7	(CG)7	(CG)7	LOC_Os11g47200	CDS	transposon protein, putative, unclassified, expressed	FRAME_SHIFT	AAAAGTTAAGATTGGCGGGG	GTGCTAGCGAGTTACCAGGC	273	P	A	A	NA
PMS5764	Os_Chr11	28395422	28395439	(AG)9	(AG)7	(AG)7	(AG)7	(AG)9	(AG)7	(AG)7	(AG)7	(AG)7	(AG)8	(AG)7	-	Introns/Intergenic	-	-	GACGGGTGATCTTTCCACAT	CTTGTGGGACCCAACTGACT	172	P	A	A	NA
PMS5765	Os_Chr11	28398868	28398899	(AGAT)8	(AGAT)8	(AGAT)6	(AGAT)8	(AGAT)7	(AGAT)8	(AGAT)8	(AGAT)8	(AGAT)8	(AGAT)8	(AGAT)8	-	Introns/Intergenic	-	-	CCACGTAGCGTCGTAGGAGT	GTGGTTAGAACGGCAACGAT	196	P	A	A	NA
PMS5766	Os_Chr11	28427015	28427038	(TC)12	(TC)12	(TC)12	(TC)12	(TC)12	(TC)12	(TC)12	(TC)12	(TC)12	(TC)7	(TC)12	-	Introns/Intergenic	-	-	AGCTTGAGCAGTGCCTTGAT	CCGATCCCATTCACAGATTC	139	A	A	A	NA
PMS5767	Os_Chr11	28479314	28479357	(TA)22	(TA)22	(TA)11	(TA)6	(TA)22	(TA)22	(TA)22	(TA)22	(TA)7	(TA)12	(TA)8	-	Introns/Intergenic	-	-	CCCTGCAGTTTTGTTCCAAT	TGAACCGTACTTGCTTGCAC	150	P	A	A	NA
PMS5768	Os_Chr11	28481192	28481213	(TA)11	(TA)11	(TA)11	(TA)7	(TA)11	(TA)11	(TA)11	(TA)11	(TA)11	(TA)11	(TA)11	-	Introns/Intergenic	-	-	ACATGCATGCTCAAGGTTATGG	TTGCTTACCTTCTGGAACTTGG	343	P	A	A	RM27344
PMS5769	Os_Chr11	28537931	28537948	(AT)9	(AT)9	(AT)8	(AT)8	(AT)9	(AT)9	(AT)9	(AT)8	(AT)8	(AT)9	(AT)9	-	Introns/Intergenic	-	-	CCTTTAATTCACAAGATTTAGGGC	TTCCACTTCCTTTCATCAAAAC	156	P	A	A	NA
PMS5770	Os_Chr11	28541464	28541501	(AT)19	(AT)19	(AT)19	(AT)19	(AT)19	(AT)19	(AT)19	(AT)8	(AT)19	(AT)19	(AT)19	-	Introns/Intergenic	-	-	ATGCGCTAAAAAGTCAACGG	AGACCCCTAGTCACTGCCCT	257	A	A	A	NA
PMS5771	Os_Chr11	28553885	28553905	(CGG)7	(CGG)7	(CGG)7	(CGG)7	(CGG)5	(CGG)7	(CGG)7	(CGG)7	(CGG)7	(CGG)7	(CGG)7	-	Introns/Intergenic	-	-	CTGAGCGGCTTGTCACATAA	ACGGTAGGAGATCCATGTCG	254	P	A	A	NA

Polymorphic SSR (PMS) markers	Chromosomes	Start physical positions (bp)	End physical positions (bp)	Nipponbare	Kasalath	Nagina22	IR64	Pokkali	Bala	Tainung67	Azucena	Moroberekan	*Oryza rufipogon*	*Oryza nivara*	MSU gene locus IDs	Structural annotation	Functional annotation	SSR functional effects	Forward primers (5'-3')	Reverse primers (5'-3')	Amplified product size (bp)	Markers exhibiting polymorphism within *indica* rice	Transcrption factor genes-derived markers	Known cloned genes-derived markers	PMS markers corresponding with RM markers
PMS5772	Os_Chr11	28582118	28582133	(AT)8	(AT)9	(AT)9	(AT)8	(AT)8	(AT)8	(AT)8	(AT)8	(AT)9	(AT)8	(AT)8	-	Introns/Intergenic	-	-	CCTTTAATTCACAAGATTTAGGGC	TTCCACTTCCTTTCATCAAAAC	154	P	A	A	NA
PMS5773	Os_Chr11	28598112	28598132	(CGG)7	(CGG)7	(CGG)7	(CGG)7	(CGG)5	(CGG)7	(CGG)7	(CGG)7	(CGG)7	(CGG)7	(CGG)7	-	Introns/Intergenic	-	-	GGAGGCAATGGTAGAACTTCAGG	GGACGGTAGGAGATCCATGTCG	188	P	A	A	RM27352
PMS5774	Os_Chr11	28625770	28625839	(AT)35	(AT)30	(AT)35	(AT)35	(AT)35	(AT)35	(AT)9	(AT)35	(AT)14	(AT)35	(AT)35	-	Introns/Intergenic	-	-	TCGATGTTTTGGATAAGTTTGAGA	ACTCCCTCCGGTTTCATTTT	273	P	A	A	NA
PMS5775	Os_Chr11	28692776	28692797	(TC)11	(TC)11	(TC)13	(TC)11	(TC)11	(TC)11	(TC)11	(TC)11	(TC)11	(TC)11	(TC)11	-	Introns/Intergenic	-	-	TTCACATCAGAGGAACTCTCC	CTGGTTAAGTTTGACCATGC	387	P	A	A	RM27357
PMS5776	Os_Chr11	28736975	28736998	(AT)12	(AT)12	(AT)12	(AT)12	(AT)12	(AT)11	(AT)12	(AT)12	(AT)12	(AT)12	(AT)12	LOC_Os11g47550	DRR	glycosyl hydrolase, putative, expressed	-	ACGACAAGAAGACCGACTACTCC	ATGCGTTTGAACAGCTATCAGG	285	P	A	A	RM27358
PMS5777	Os_Chr11	28805009	28805029	(TAT)7	(TAT)7	(TAT)7	(TAT)7	(TAT)7	(TAT)6	(TAT)7	(TAT)5	(TAT)7	(TAT)7	(TAT)7	-	Introns/Intergenic	-	-	CATGTTGTGCTTGTCCTACTGC	AGCTAGAGGAGATCAGATGGTAGTGC	245	P	A	A	RM144
PMS5778	Os_Chr11	28816419	28816460	(AT)21	(AT)21	(AT)11	(AT)16	(AT)19	(AT)21	(AT)21	(AT)21	(AT)21	(AT)21	(AT)21	-	Introns/Intergenic	-	-	AATTTGGTCGGCCTTTCTTT	GCATATGCACACCTTGGAGA	279	P	A	A	NA
PMS5779	Os_Chr11	28835662	28835679	(TA)9	(TA)9	(TA)9	(TA)9	(TA)9	(TA)9	(TA)8	(TA)9	(TA)9	(TA)9	(TA)9	-	Introns/Intergenic	-	-	TGTGCAGACGCCTATTTGAG	CCATTGATGTCTTTGAGCCC	129	A	A	A	NA
PMS5780	Os_Chr11	28852845	28852865	(CGC)7	(CGC)7	(CGC)7	(CGC)7	(CGC)7	(CGC)7	(CGC)7	(CGC)7	(CGC)7	(CGC)5	(CGC)7	LOC_Os11g47830	URR	RNA recognition motif containing protein, expressed	-	GCTGGGCAATGAGAAATTAGGG	CATCTGTCGTCTCCTCCTCTTCC	175	A	A	A	RM27371
PMS5781	Os_Chr11	28927954	28927969	(TC)8	(TC)8	(TC)8	(TC)8	(TC)8	(TC)8	(TC)8	(TC)8	(TC)8	(TC)8	(TC)7	-	Introns/Intergenic	-	-	TCGAGTTCAAATCCGAATCC	GGGGGAAGAGAGAGAGAAAAA	147	A	A	A	NA
PMS5782	Os_Chr11	28932540	28932578	(CCT)13	(CCT)13	(CCT)13	(CCT)12	(CCT)12	(CCT)12	(CCT)13	(CCT)13	(CCT)13	(CCT)13	(CCT)13	LOC_Os11g47960	CDS	expressed protein	CODON_DELETION	TGCTTGATCTGTGTTCGTCC	CAGCACCAGCATGAAAGAAA	179	P	A	A	NA
PMS5783	Os_Chr11	28937951	28937972	(AT)11	(AT)11	(AT)11	(AT)8	(AT)11	(AT)11	(AT)11	(AT)11	(AT)11	(AT)11	(AT)11	-	Introns/Intergenic	-	-	TAGGGTGCGAAGTACAAGGAAGC	TGCGTCTACAACTGAACTCAATCC	138	P	A	A	RM27381
PMS5784	Os_Chr11	28956350	28956371	(AC)11	(AC)12	(AC)11	(AC)11	(AC)11	(AC)11	(AC)11	(AC)11	(AC)11	(AC)11	(AC)11	-	Introns/Intergenic	-	-	TCCCATCCCATAGTCACTGAAGC	GAGCCTTCTGGTTTCTGCTATGC	194	P	A	A	RM27384

Polymorphic SSR (PMS) markers	Chromosomes	Start physical positions (bp)	End physical positions (bp)	Nipponbare	Kasalath	Nagina22	IR64	Pokkali	Bala	Tainung67	Azucena	Moroberekan	*Oryza rufipogon*	*Oryza nivara*	MSU gene locus IDs	Structural annotation	Functional annotation	SSR functional effects	Forward primers (5'-3')	Reverse primers (5'-3')	Amplified product size (bp)	Markers exhibiting polymorphism within *indica* rice	Transcrption factor genes-derived markers	Known cloned genes-derived markers	PMS markers corresponding with RM markers
PMS5785	Os_Chr11	28957317	28957336	(CTCC)5	(CTCC)6	(CTCC)5	(CTCC)5	(CTCC)5	(CTCC)5	(CTCC)5	(CTCC)5	(CTCC)5	(CTCC)5	(CTCC)5	-	Introns/Intergenic	-	-	TGCTTTGCTTGTCTTGTCTTGC	TGCTACTACTACGTGGAGAGCAGAGC	178	P	A	A	RM27386
PMS5786	Os_Chr12	77085	77118	(TA)17	(TA)17	(TA)17	(TA)17	(TA)17	(TA)17	(TA)9	(TA)8	(TA)17	(TA)17	(TA)6	-	Introns/Intergenic	-	-	CGTACGCACAGAGAATCCAA	GTGTGGCCTGCATGTGTACT	272	A	A	A	NA
PMS5787	Os_Chr12	323581	323595	(GCG)5	(GCG)6	(GCG)6	(GCG)6	(GCG)5	(GCG)6	(GCG)5	(GCG)5	(GCG)5	(GCG)5	(GCG)5	LOC_Os12g01530	CDS	OsFER2	CODON_INSERTION	AAGAGACTGGTCCTTGGCCT	CTCACTTAATCCGCCATGCT	261	P	A	P	NA
PMS5788	Os_Chr12	396888	396909	(AT)11	(AT)11	(AT)7	(AT)11	(AT)11	(AT)11	(AT)11	(AT)8	(AT)11	(AT)11	(AT)11	-	Introns/Intergenic	-	-	GCATCAACGGTTCACATTCTAGG	ATTACACACCAACCCGCTAATGG	188	P	A	A	RM27414
PMS5789	Os_Chr12	508987	509020	(AT)17	(AT)17	(AT)17	(AT)17	(AT)13	(AT)17	(AT)17	(AT)17	(AT)10	(AT)17	(AT)17	-	Introns/Intergenic	-	-	GAACGAGAGCGAGAACGAAC	CGACAACACGTCTTTTCTGC	179	P	A	A	NA
PMS5790	Os_Chr12	550810	550824	(TGC)5	(TGC)6	(TGC)6	(TGC)5	(TGC)5	(TGC)6	(TGC)5	(TGC)5	(TGC)5	(TGC)5	(TGC)5	-	Introns/Intergenic	-	-	GTGGGAGGGGTTTAGGGTTA	GAAAAGGGGGCAAGAAGAAG	263	P	A	A	NA
PMS5791	Os_Chr12	586558	586571	(GA)7	(GA)7	(GA)7	(GA)7	(GA)7	(GA)7	(GA)7	(GA)7	(GA)7	(GA)7	(GA)6	-	Introns/Intergenic	-	-	TGGTTGTTTCCTCGCTCTCT	CAAGCTAATGATGCCAGCAA	232	A	A	A	NA
PMS5792	Os_Chr12	599151	599206	(AT)28	(AT)28	(AT)28	(AT)28	(AT)28	(AT)28	(AT)28	(AT)28	(AT)28	(AT)14	(AT)28	-	Introns/Intergenic	-	-	GCAGAGTGATTTGCAACACG	TCCACTCTGAGCAATGGAGTT	273	A	A	A	NA
PMS5793	Os_Chr12	621466	621487	(TA)11	(TA)7	(TA)11	(TA)11	(TA)11	(TA)11	(TA)11	(TA)11	(TA)11	(TA)11	(TA)11	-	Introns/Intergenic	-	-	TTTCTCATGCATGTTTGCGT	AAGTGCATGCGTACTGATCG	170	P	A	A	NA
PMS5794	Os_Chr12	725996	726023	(CATC)7	(CATC)7	(CATC)7	(CATC)7	(CATC)7	(CATC)7	(CATC)5	(CATC)7	(CATC)7	(CATC)7	(CATC)7	-	Introns/Intergenic	-	-	TCACAGATTCCAGCAAGCAG	GTTGGATGGATGTGTGTTGC	225	A	A	A	NA
PMS5795	Os_Chr12	748522	748561	(TA)20	(TA)20	(TA)9	(TA)20	(TA)20	(TA)20	(TA)20	(TA)20	(TA)20	(TA)20	(TA)20	-	Introns/Intergenic	-	-	GCACACCAAAATGGAGATCA	CGATGACGTGGTGTCAACAT	255	P	A	A	NA
PMS5796	Os_Chr12	852543	852556	(AG)7	(AG)7	(AG)7	(AG)7	(AG)7	(AG)6	(AG)7	(AG)7	(AG)7	(AG)6	(AG)7	-	Introns/Intergenic	-	-	GCAATGGCATCATCATCAAC	CTAGCGTGAACCCCACGTAT	163	P	A	A	NA
PMS5797	Os_Chr12	930122	930141	(TC)10	(TC)10	(TC)10	(TC)10	(TC)10	(TC)10	(TC)10	(TC)10	(TC)10	(TC)10	(TC)8	-	Introns/Intergenic	-	-	GACGGCGCGGCAGTATCATAGG	CGCTAGGCAACCATTTGCTCACG	307	A	A	A	RM27452

Polymorphic SSR (PMS) markers	Chromosomes	Start physical positions (bp)	End physical positions (bp)	Nipponbare	Kasalath	Nagina22	IR64	Pokkali	Bala	Tainung67	Azucena	Moroberekan	*Oryza rufipogon*	*Oryza nivara*	MSU gene locus IDs	Structural annotation	Functional annotation	SSR functional effects	Forward primers (5'-3')	Reverse primers (5'-3')	Amplified product size (bp)	Markers exhibiting polymorphism within *indica* rice	Transcrption factor genes-derived markers	Known cloned genes-derived markers	PMS markers corresponding with RM markers
PMS5798	Os_Chr12	959408	959435	(AT)14	(AT)14	(AT)12	(AT)6	(AT)9	(AT)14	(AT)14	(AT)14	(AT)14	(AT)14	(AT)14	-	Introns/Intergenic	-	-	TGGTCACTAGCATCTGTCGC	CGAGTTTCGGTTTTTGGGTA	127	P	A	A	NA
PMS5799	Os_Chr12	983509	983528	(CT)10	(CT)10	(CT)10	(CT)9	(CT)8	(CT)9	(CT)10	(CT)9	(CT)10	(CT)8	(CT)10	-	Introns/Intergenic	-	-	CAATTGGCAGTGCAGTGGTAAGG	GCTCTGTTCACGAGTCTGTCTCTTCC	331	P	A	A	RM27457
PMS5800	Os_Chr12	1032073	1032102	(TA)15	(TA)13	(TA)15	(TA)15	(TA)15	(TA)15	(TA)7	(TA)13	(TA)15	(TA)15	(TA)15	-	Introns/Intergenic	-	-	GCTCGCCTGCCTTTAATACTCC	GAGAAGTAAACACGCCACCAACC	438	P	A	A	RM27458
PMS5801	Os_Chr12	1055731	1055754	(CTG)8	(CTG)6	(CTG)8	(CTG)8	(CTG)8	(CTG)6	(CTG)8	(CTG)8	(CTG)8	(CTG)6	(CTG)6	-	Introns/Intergenic	-	-	CTGATGGGAAAGCCAACATT	ATCAAGATATGGGCGACAGC	254	P	A	A	NA
PMS5802	Os_Chr12	1078673	1078686	(TA)7	(TA)7	(TA)7	(TA)7	(TA)7	(TA)6	(TA)7	(TA)7	(TA)7	(TA)7	(TA)7	-	Introns/Intergenic	-	-	GAAACCAATATCGGCTCAGG	CAGCTGCTTGTTTGCTTCAG	170	P	A	A	NA
PMS5803	Os_Chr12	1144916	1144931	(AC)8	(AC)6	(AC)8	(AC)8	(AC)8	(AC)8	(AC)8	(AC)8	(AC)8	(AC)8	(AC)8	-	Introns/Intergenic	-	-	GATCTGACTCGGCGCTCTAA	GGACGGGTGGTGTACTGTCT	118	P	A	A	NA
PMS5804	Os_Chr12	1256758	1256783	(AT)13	(AT)13	(AT)13	(AT)13	(AT)13	(AT)7	(AT)13	(AT)13	(AT)13	(AT)13	(AT)13	-	Introns/Intergenic	-	-	CTTGCAAGCGGCGTACAGAGC	TTCCTGGATCCTGAAGGTACGTAGG	380	P	A	A	RM27469
PMS5805	Os_Chr12	1272893	1272910	(GA)9	(GA)9	(GA)9	(GA)9	(GA)9	(GA)9	(GA)9	(GA)9	(GA)9	(GA)9	(GA)8	-	Introns/Intergenic	-	-	ATCCATGCAGAGCATACACG	TGAGAAGGAGAGGTCGGAGA	254	A	A	A	NA
PMS5806	Os_Chr12	1368959	1368978	(CT)10	(CT)10	(CT)10	(CT)10	(CT)10	(CT)10	(CT)10	(CT)10	(CT)10	(CT)7	(CT)10	LOC_Os12g03480	URR	alpha-N-arabinofuranosidase, putative, expressed	-	CGTCTTCAACCTCCAGCTCATGG	GAGGAGGCAGGAGGGAATCG	62	A	A	A	RM27472
PMS5807	Os_Chr12	1511844	1511861	(GAG)6	(GAG)6	(GAG)6	(GAG)5	(GAG)5	(GAG)6	(GAG)6	(GAG)6	(GAG)6	(GAG)6	(GAG)5	-	Introns/Intergenic	-	-	CGGAAGTGCTTCTTGGAGTC	GGCAACTGCAGAGGAGAATC	253	P	A	A	NA
PMS5808	Os_Chr12	1712737	1712751	(CGG)5	(CGG)5	(CGG)5	(CGG)5	(CGG)6	(CGG)5	(CGG)5	(CGG)5	(CGG)5	(CGG)5	(CGG)5	LOC_Os12g04070	CDS	expressed protein	CODON_INSERTION	GACCAGGCTCAGGAGGAGAT	CAGGTGTACTGTGTGGGACG	162	P	A	A	NA
PMS5809	Os_Chr12	1807692	1807713	(AT)11	(AT)11	(AT)11	(AT)11	(AT)11	(AT)7	(AT)11	(AT)6	(AT)11	(AT)11	(AT)11	LOC_Os12g04270	DRR	cytochrome c-type biogenesis protein dbsD-like, putative, expressed	-	TAATGCATCCACCGAAATGA	CAGAGGCAAGAGCAGAACAG	137	P	A	A	NA
PMS5810	Os_Chr12	1839193	1839206	(CT)7	(CT)7	(CT)7	(CT)7	(CT)7	(CT)7	(CT)7	(CT)8	(CT)8	(CT)7	(CT)7	-	Introns/Intergenic	-	-	GAGACACAAATCGGGAAGTCA	AAGCTAACGCTGAGCACGAT	149	A	A	A	NA

Polymorphic SSR (PMS) markers	Chromosomes	Start physical positions (bp)	End physical positions (bp)	Nipponbare	Kasalath	Nagina22	IR64	Pokkali	Bala	Tainung67	Azucena	Moroberekan	*Oryza rufipogon*	*Oryza nivara*	MSU gene locus IDs	Structural annotation	Functional annotation	SSR functional effects	Forward primers (5'-3')	Reverse primers (5'-3')	Amplified product size (bp)	Markers exhibiting polymorphism within *indica* rice	Transcrption factor genes-derived markers	Known cloned genes-derived markers	PMS markers corresponding with RM markers
PMS5811	Os_Chr12	1861131	1861148	(CGG)6	(CGG)6	(CGG)6	(CGG)6	(CGG)6	(CGG)6	(CGG)6	(CGG)5	(CGG)6	(CGG)6	(CGG)6	LOC_Os12g04380	CDS	GRAS family transcription factor containing protein, expressed	CODON_DELETION	AGGGGCTGAGCTCAAAGAAC	GGCCTAGCTCTGTTGGAGTG	271	A	P	A	NA
PMS5812	Os_Chr12	1874988	1875003	(AT)8	(AT)8	(AT)8	(AT)8	(AT)8	(AT)8	(AT)8	(AT)8	(AT)8	(AT)7	(AT)8	-	Introns/Intergenic	-	-	CCTTACCCATAACCCTGACA	CAGATGAGAAAAGCCATCTGAA	238	A	A	A	NA
PMS5813	Os_Chr12	1882944	1882961	(GGC)6	(GGC)6	(GGC)6	(GGC)6	(GGC)6	(GGC)6	(GGC)6	(GGC)6	(GGC)6	(GGC)6	(GGC)5	LOC_Os12g04424	URR	strictosidine synthase, putative, expressed	-	TTCGTCTCACTCACCCTCCT	CTCTGCCTCACCACCAAATC	199	A	A	A	NA
PMS5814	Os_Chr12	2007660	2007716	(ATT)19	(ATT)19	(ATT)14	(ATT)19	(ATT)19	(ATT)19	(ATT)8	(ATT)19	(ATT)8	(ATT)8	(ATT)19	-	Introns/Intergenic	-	-	CAGCCTCAACAACCTATTGTGACAGC	ATTATTTGGCCACGGCCTGACG	234	P	A	A	RM27502
PMS5815	Os_Chr12	2090578	2090595	(TGG)6	(TGG)6	(TGG)6	(TGG)5	(TGG)5	(TGG)6	(TGG)5	(TGG)6	(TGG)6	(TGG)5	(TGG)6	LOC_Os12g04860	URR	DTW domain containing protein, putative, expressed	-	AACCCACTGTTCCTGTACGC	ATGGTGTGACGGGGTTATGT	174	P	A	A	NA
PMS5816	Os_Chr12	2182499	2182516	(TAC)6	(TAC)6	(TAC)6	(TAC)5	(TAC)5	(TAC)6	(TAC)6	(TAC)6	(TAC)6	(TAC)6	(TAC)5	-	Introns/Intergenic	-	-	GCGATCGTACGTGTTTGAGA	CAAACAGCCCCTAACTCCTG	235	P	A	A	NA
PMS5817	Os_Chr12	2261019	2261030	(TA)6	(TA)7	(TA)6	(TA)6	(TA)6	(TA)7	(TA)6	(TA)6	(TA)6	(TA)6	(TA)6	-	Introns/Intergenic	-	-	CAAACACGTAACCGCTCAGA	CACAGCAAGCAAAACGAGAA	172	P	A	A	NA
PMS5818	Os_Chr12	2305433	2305466	(TC)17	(TC)17	(TC)17	(TC)17	(TC)17	(TC)17	(TC)17	(TC)15	(TC)17	(TC)18	(TC)17	LOC_Os12g05200	URR	DNA binding protein, putative, expressed	-	CTTGGTTTGGTTGTGTCCAAGC	GTTCTGATGAACAGGCCGTAGC	176	A	A	A	RM3747
PMS5819	Os_Chr12	2315612	2315629	(TGC)6	(TGC)5	(TGC)5	(TGC)5	(TGC)5	(TGC)5	(TGC)6	(TGC)6	(TGC)6	(TGC)6	(TGC)5	-	Introns/Intergenic	-	-	TGAAACTGAACTGCTGGTGC	TTTTTGGTGGCTTCGAGACT	202	A	A	A	NA
PMS5820	Os_Chr12	2433280	2433306	(ATC)9	(ATC)9	(ATC)9	(ATC)9	(ATC)9	(ATC)6	(ATC)9	(ATC)9	(ATC)9	(ATC)9	(ATC)6	-	Introns/Intergenic	-	-	CCAAAAACAGCAGCAGATGA	CTCAAGATGGACGCCAAGAT	217	P	A	A	NA
PMS5821	Os_Chr12	2445466	2445486	(CCG)7	(CCG)7	(CCG)7	(CCG)7	(CCG)7	(CCG)7	(CCG)7	(CCG)7	(CCG)7	(CCG)7	(CCG)6	LOC_Os12g05394	CDS	protein kinase, putative, expressed	CODON_CHANGE_PLUS_CODON_DELETION	AAGGACCTCGTCACGTCG	GCTTGGCTTTGCTTGTCTCT	258	A	A	A	NA
PMS5822	Os_Chr12	2473717	2473740	(TA)12	(TA)12	(TA)12	(TA)12	(TA)6	(TA)8	(TA)12	(TA)12	(TA)12	(TA)12	(TA)12	-	Introns/Intergenic	-	-	GAGCATGGAGGAGAATTTGG	GCAGTGCACTTACATATCATATCAAC	271	P	A	A	NA
PMS5823	Os_Chr12	2483185	2483198	(AT)7	(AT)7	(AT)7	(AT)7	(AT)7	(AT)6	(AT)7	(AT)7	(AT)7	(AT)7	(AT)7	-	Introns/Intergenic	-	-	ATTGCATGAAGCCAGGCATA	CACTCTCTCCGTTTCACAATG	234	P	A	A	NA

Polymorphic SSR (PMS) markers	Chromosomes	Start physical positions (bp)	End physical positions (bp)	Nipponbare	Kasalath	Nagina22	IR64	Pokkali	Bala	Tainung67	Azucena	Moroberekan	*Oryza rufipogon*	*Oryza nivara*	MSU gene locus IDs	Structural annotation	Functional annotation	SSR functional effects	Forward primers (5'-3')	Reverse primers (5'-3')	Amplified product size (bp)	Markers exhibiting polymorphism within *indica* rice	Transcrption factor genes-derived markers	Known cloned genes-derived markers	PMS markers corresponding with RM markers
PMS5824	Os_Chr12	2546558	2546575	(CGT)6	(CGT)5	(CGT)6	(CGT)6	(CGT)5	(CGT)6	(CGT)6	(CGT)6	(CGT)6	(CGT)6	(CGT)6	LOC_Os12g05550	CDS	sialyltransferase family domain containing protein, expressed	CODON_CHANGE_PLUS_CODON_DELETION	CCTCCTCTCAATCCTCTCCC	GGAGGTAGAGCAGCCGGT	233	P	A	A	NA
PMS5825	Os_Chr12	2552780	2552817	(TA)19	(TA)19	(TA)10	(TA)9	(TA)10	(TA)10	(TA)19	(TA)19	(TA)19	(TA)19	(TA)19	-	Introns/Intergenic	-	-	CAACGGTGTCAAACATTTCG	TAGAAATGGTGGGTTCGCTC	148	P	A	A	NA
PMS5826	Os_Chr12	2563224	2563241	(CGA)6	(CGA)6	(CGA)5	(CGA)6	(CGA)6	(CGA)5	(CGA)6	(CGA)6	(CGA)6	(CGA)6	(CGA)6	LOC_Os12g05580	CDS	expressed protein	CODON_DELETION	ACGACGACGACGAAGAAGAC	GAAGAACCTCCTCCACCTCC	206	P	A	A	NA
PMS5827	Os_Chr12	2586671	2586684	(CT)7	(CT)7	(CT)7	(CT)7	(CT)7	(CT)7	(CT)7	(CT)7	(CT)7	(CT)6	(CT)7	-	Introns/Intergenic	-	-	CGGAGAGGTAGAAGACGACG	TTGCCTTCTGTCTCGGCTAT	128	A	A	A	NA
PMS5828	Os_Chr12	2611698	2611733	(AG)18	(AG)18	(AG)18	(AG)18	(AG)18	(AG)13	(AG)18	(AG)18	(AG)18	(AG)18	(AG)18	-	Introns/Intergenic	-	-	GAACGTGAAAGAGAAGCTCATGG	TCTCCTTCTCTCCCAACATCTCG	175	P	A	A	RM1302
PMS5829	Os_Chr12	2762488	2762505	(GCG)6	(GCG)6	(GCG)6	(GCG)5	(GCG)6	(GCG)6	(GCG)6	(GCG)6	(GCG)6	(GCG)6	(GCG)6	LOC_Os12g05990	CDS	No apical meristem protein, putative, expressed	CODON_CHANGE_PLUS_CODON_DELETION	CCAATTCATTCATTTCACGC	CTCCTCCGTCGGGTAGAATC	254	P	P	A	NA
PMS5830	Os_Chr12	2776150	2776167	(CCG)6	(CCG)6	(CCG)6	(CCG)6	(CCG)6	(CCG)6	(CCG)6	(CCG)5	(CCG)6	(CCG)6	(CCG)6	LOC_Os12g06010	CDS	osZIP-2b	CODON_DELETION	CAGCATCATCCAGTCCTCCT	GATAGGCGAGCGATGTCATT	159	A	P	P	NA
PMS5831	Os_Chr12	2821652	2821663	(AT)6	(AT)6	(AT)7	(AT)6	(AT)6	(AT)6	(AT)6	(AT)6	(AT)6	(AT)6	(AT)6	-	Introns/Intergenic	-	-	AGTCAAGACAGCGACACTGC	TCAGTTCACAAGCATATAAAATCAAA	247	P	A	A	NA
PMS5832	Os_Chr12	2947913	2947939	(CGC)9	(CGC)8	(CGC)9	(CGC)5	(CGC)5	(CGC)7	(CGC)9	(CGC)9	(CGC)9	(CGC)9	(CGC)9	LOC_Os12g06210	CDS	harpin-induced protein 1 domain containing protein, expressed	CODON_DELETION	CGTGCAAACAAACGTCAATC	GATAAGAGCCAGGCCGAAG	275	P	A	A	NA
PMS5833	Os_Chr12	2992849	2992863	(CGA)5	(CGA)6	(CGA)6	(CGA)6	(CGA)5	(CGA)6	(CGA)5	(CGA)5	(CGA)5	(CGA)6	(CGA)5	-	Introns/Intergenic	-	-	TCCAAACCGCTTTAATTTCG	ATCCTCGGGTCCTTCGAC	170	P	A	A	NA
PMS5834	Os_Chr12	3009825	3009854	(CGG)10	(CGG)8	(CGG)10	(CGG)10	(CGG)6	(CGG)9	(CGG)10	(CGG)10	(CGG)10	(CGG)10	(CGG)5	LOC_Os12g06290	URR	expressed protein	-	CTGTGCAGGTCGTCCATCT	AAATTGCCATTTCTACCCCC	220	P	A	A	NA
PMS5835	Os_Chr12	3021416	3021433	(AT)9	(AT)9	(AT)9	(AT)6	(AT)9	(AT)9	(AT)9	(AT)9	(AT)9	(AT)9	(AT)9	-	Introns/Intergenic	-	-	AGGCTCTGCCATCTGATTGT	GGTTATGCCCTGATGGTTTG	209	P	A	A	NA
PMS5836	Os_Chr12	3024680	3024700	(CTG)7	(CTG)7	(CTG)7	(CTG)7	(CTG)7	(CTG)5	(CTG)7	(CTG)7	(CTG)7	(CTG)7	(CTG)7	-	Introns/Intergenic	-	-	TCCCGCTAGATTTCGTTTTG	CTGCTCCCCCAAAATCACTA	219	P	A	A	NA

Polymorphic SSR (PMS) markers	Chromosomes	Start physical positions (bp)	End physical positions (bp)	Nipponbare	Kasalath	Nagina22	IR64	Pokkali	Bala	Tainung67	Azucena	Moroberekan	*Oryza rufipogon*	*Oryza nivara*	MSU gene locus IDs	Structural annotation	Functional annotation	SSR functional effects	Forward primers (5'-3')	Reverse primers (5'-3')	Amplified product size (bp)	Markers exhibiting polymorphism within *indica* rice	Transcrption factor genes-derived markers	Known cloned genes-derived markers	PMS markers corresponding with RM markers
PMS5837	Os_Chr12	3025679	3025702	(TTG)8	(TTG)8	(TTG)8	(TTG)8	(TTG)8	(TTG)8	(TTG)8	(TTG)8	(TTG)8	(TTG)8	(TTG)6	-	Introns/Intergenic	-	-	CTAGTTGCAGAAGCACACAAATGG	GATTGTTCATCTCAGCAGCAACC	273	A	A	A	RM27555
PMS5838	Os_Chr12	3029832	3029855	(TAAT)6	(TAAT)6	(TAAT)5	(TAAT)6	(TAAT)5	(TAAT)6	(TAAT)6	(TAAT)6	(TAAT)6	(TAAT)6	(TAAT)6	LOC_Os12g06340	DRR	OsBLH1	-	CTTCTCTCAAGTTACCAACTGC	CTCAGCTCATGAGAGACTTCG	336	P	P	P	RM27556
PMS5839	Os_Chr12	3030160	3030177	(CGT)6	(CGT)6	(CGT)6	(CGT)6	(CGT)6	(CGT)6	(CGT)6	(CGT)6	(CGT)6	(CGT)5	(CGT)6	LOC_Os12g06340	DRR	OsBLH1	-	GAACTGATGCCCGTACTGC	AATGAAGTTCACTGAGGCCG	133	A	P	P	NA
PMS5840	Os_Chr12	3033336	3033359	(CGG)8	(CGG)8	(CGG)8	(CGG)8	(CGG)8	(CGG)8	(CGG)8	(CGG)6	(CGG)8	(CGG)8	(CGG)8	LOC_Os12g06340	CDS	OsBLH1	CODON_DELETION	CATCACCACCTTCTCCTGGT	CTCTCCAGTCGGGGTACAAC	233	A	P	P	NA
PMS5841	Os_Chr12	3035478	3035497	(CT)10	(CT)10	(CT)10	(CT)8	(CT)10	(CT)10	(CT)10	(CT)10	(CT)10	(CT)10	(CT)8	-	Introns/Intergenic	-	-	GGATATAACCTGGAGTCGAATGG	GGTGGAACTAAATGAAGGAAGG	273	P	A	A	RM27559
PMS5842	Os_Chr12	3105928	3105941	(AT)7	(AT)8	(AT)7	(AT)7	(AT)7	(AT)7	(AT)7	(AT)7	(AT)7	(AT)7	(AT)7	-	Introns/Intergenic	-	-	TGTTAAGCCACATCACCCAA	CGTTCCCCAAACCATAAAAA	213	P	A	A	NA
PMS5843	Os_Chr12	3110265	3110282	(CGG)6	(CGG)6	(CGG)6	(CGG)6	(CGG)6	(CGG)6	(CGG)5	(CGG)6	(CGG)6	(CGG)6	(CGG)6	LOC_Os12g06464	URR	expressed protein	-	CACACCAACAGCAGCAATCT	TCTCCTCGCTTCTTCGTCTC	164	A	A	A	NA
PMS5844	Os_Chr12	3153481	3153501	(GGA)7	(GGA)7	(GGA)7	(GGA)7	(GGA)5	(GGA)7	(GGA)7	(GGA)7	(GGA)7	(GGA)7	(GGA)7	LOC_Os12g06520	CDS	RSG	CODON_DELETION	CTTCTTCGAGGGTTTCTCGTTGC	CTCCTCTTATGCCCACCACTTCC	93	P	A	P	RM27564
PMS5845	Os_Chr12	3186494	3186549	(AG)28	(AG)28	(AG)28	(AG)28	(AG)28	(AG)28	(AG)28	(AG)25	(AG)28	(AG)28	(AG)28	-	Introns/Intergenic	-	-	AAGGCGAACTGTCCTAGTGAAGC	CAGGATGTTCTTGCCAAGTTGC	181	A	A	A	RM247
PMS5846	Os_Chr12	3293625	3293648	(GGT)8	(GGT)8	(GGT)8	(GGT)8	(GGT)8	(GGT)9	(GGT)8	(GGT)8	(GGT)8	(GGT)8	(GGT)8	LOC_Os12g06780	CDS	expressed protein	CODON_INSERTION	TTTCCTTTCTTGCGCCATTTCC	GAACTCCTCCTGGCACAGAATAAACG	376	P	A	A	RM27575
PMS5847	Os_Chr12	3302563	3302577	(CGG)5	(CGG)5	(CGG)5	(CGG)5	(CGG)5	(CGG)5	(CGG)5	(CGG)5	(CGG)5	(CGG)7	(CGG)5	LOC_Os12g06800	URR	serine esterase family protein, putative, expressed	-	AACCCCCTACTCCAGACGAC	GATCTGCGAATTTTGCCAAC	127	A	A	A	NA
PMS5848	Os_Chr12	3318687	3318707	(TCG)7	(TCG)7	(TCG)7	(TCG)7	(TCG)7	(TCG)7	(TCG)7	(TCG)7	(TCG)7	(TCG)7	(TCG)5	LOC_Os12g06820	CDS	PE repeat family protein, putative, expressed	CODON_DELETION	CTCGTACTCCAACTCGAGGC	GAGTTGCTCGTCGTCATCAT	246	A	A	A	NA
PMS5849	Os_Chr12	3332928	3332975	(TA)24	(TA)17	(TA)24	(TA)24	(TA)24	(TA)24	(TA)24	(TA)24	(TA)24	(TA)24	(TA)24	-	Introns/Intergenic	-	-	ATGGTGTTGAAGGGCTTGTTGG	ATGCCCTGATAGGTGAACAGTCG	539	P	A	A	RM27583

Polymorphic SSR (PMS) markers	Chromosomes	Start physical positions (bp)	End physical positions (bp)	Nipponbare	Kasalath	Nagina22	IR64	Pokkali	Bala	Tainung67	Azucena	Moroberekan	*Oryza rufipogon*	*Oryza nivara*	MSU gene locus IDs	Structural annotation	Functional annotation	SSR functional effects	Forward primers (5'-3')	Reverse primers (5'-3')	Amplified product size (bp)	Markers exhibiting polymorphism within *indica* rice	Transcrption factor genes-derived markers	Known cloned genes-derived markers	PMS markers corresponding with RM markers
PMS5850	Os_Chr12	3338274	3338291	(AGG)6	(AGG)5	(AGG)6	(AGG)6	(AGG)6	(AGG)6	(AGG)6	(AGG)6	(AGG)6	(AGG)6	(AGG)6	LOC_Os12g06870	URR	OsNucAP2 - Putative Nucleoporin Autopeptidase homologue, expressed	-	TCTTCAGATCCTAATCGCCG	AGAGAAGAGGAGAGGAGGCG	216	P	A	A	NA
PMS5851	Os_Chr12	3375376	3375399	(TA)12	(TA)12	(TA)12	(TA)12	(TA)12	(TA)12	(TA)6	(TA)12	(TA)12	(TA)12	(TA)12	-	Introns/Intergenic	-	-	CTTCTCTGTGCCGATAAGGG	ATTTGCATGCTTCTCAACCC	158	A	A	A	NA
PMS5852	Os_Chr12	3401924	3401941	(CGG)6	(CGG)6	(CGG)6	(CGG)6	(CGG)6	(CGG)6	(CGG)6	(CGG)5	(CGG)6	(CGG)6	(CGG)6	LOC_Os12g06970	CDS	ECAGL3 - ECA1 gametogenesis related family protein precursor, putative, expressed	CODON_DELETION	TTTCACAGACGTGTTCGTGC	GTGCTCTTCTTCCTCAACGG	244	A	A	A	NA
PMS5853	Os_Chr12	3418571	3418594	(TA)12	(TA)12	(TA)9	(TA)10	(TA)12	(TA)12	(TA)12	(TA)12	(TA)12	(TA)12	(TA)12	-	Introns/Intergenic	-	-	CCTTTTCCCCCGATAATGTT	GCCACTGGAGGACTTTTCTCT	271	P	A	A	NA
PMS5854	Os_Chr12	3448790	3448805	(AT)8	(AT)8	(AT)8	(AT)6	(AT)8	(AT)8	(AT)8	(AT)8	(AT)8	(AT)8	(AT)8	-	Introns/Intergenic	-	-	ACGCAAAAACTTGTCAGCCT	GTCTCGCAATTTACACGCAA	109	P	A	A	NA
PMS5855	Os_Chr12	3490867	3490884	(AG)9	(AG)9	(AG)9	(AG)9	(AG)9	(AG)9	(AG)9	(AG)9	(AG)9	(AG)7	(AG)7	-	Introns/Intergenic	-	-	TTTTTGCATCACTCGAGACG	ACTGTTCTGCTCCCGTGTCT	145	A	A	A	NA
PMS5856	Os_Chr12	3521191	3521232	(CT)21	(CT)18	(CT)21	(CT)18	(CT)21	(CT)10	(CT)21	(CT)21	(CT)21	(CT)21	(CT)21	-	Introns/Intergenic	-	-	CTACACGGAGAAGACGGGAA	CCAGCTCAGCTCAAGAGGAT	174	P	A	A	NA
PMS5857	Os_Chr12	3564615	3564664	(AT)25	(AT)25	(AT)15	(AT)25	(AT)12	(AT)25	(AT)25	(AT)25	(AT)8	(AT)25	(AT)25	-	Introns/Intergenic	-	-	TCCCATCTAATTCTCATCCCA	ACCCTCAAAGCCTCCAAAAT	225	P	A	A	NA
PMS5858	Os_Chr12	3589308	3589319	(TC)6	(TC)6	(TC)6	(TC)6	(TC)6	(TC)6	(TC)6	(TC)6	(TC)7	(TC)6	(TC)6	-	Introns/Intergenic	-	-	CCATAGCGTCTTGCAAATCA	CAACTCAGCAGGAGCATTCA	207	A	A	A	NA
PMS5859	Os_Chr12	3665004	3665085	(TA)41	(TA)41	(TA)41	(TA)41	(TA)41	(TA)41	(TA)8	(TA)41	(TA)41	(TA)18	(TA)41	-	Introns/Intergenic	-	-	GGGTTAGGGTTTCAACATGG	GCCGAATGTCTTCCACAACT	219	A	A	A	NA
PMS5860	Os_Chr12	3697694	3697715	(TA)11	(TA)11	(TA)11	(TA)9	(TA)11	(TA)11	(TA)11	(TA)11	(TA)11	(TA)11	(TA)11	-	Introns/Intergenic	-	-	AGGTATGAGCTCGTTTTGCG	GTGCCATAGGAAAAGCCAAG	275	P	A	A	NA
PMS5861	Os_Chr12	3711568	3711581	(TC)7	(TC)8	(TC)8	(TC)8	(TC)7	(TC)8	(TC)7	(TC)7	(TC)7	(TC)7	(TC)7	LOC_Os12g07480	DRR	TCP family transcription factor, putative, expressed	-	TTGAGACGGGTGTTTGATGA	CATAAACACAGCCACAGGCA	238	P	P	A	NA
PMS5862	Os_Chr12	3767238	3767261	(TTA)8	(TTA)8	(TTA)8	(TTA)8	(TTA)8	(TTA)8	(TTA)8	(TTA)8	(TTA)8	(TTA)5	(TTA)8	-	Introns/Intergenic	-	-	TGATATTGGGCCTTCAGTTGTGG	TGACCAACGTTTGACCATTCG	333	A	A	A	RM27616

Polymorphic SSR (PMS) markers	Chromosomes	Start physical positions (bp)	End physical positions (bp)	Nipponbare	Kasalath	Nagina22	IR64	Pokkali	Bala	Tainung67	Azucena	Moroberekan	*Oryza rufipogon*	*Oryza nivara*	MSU gene locus IDs	Structural annotation	Functional annotation	SSR functional effects	Forward primers (5'-3')	Reverse primers (5'-3')	Amplified product size (bp)	Markers exhibiting polymorphism within *indica* rice	Transcrption factor genes-derived markers	Known cloned genes-derived markers	PMS markers corresponding with RM markers
PMS5863	Os_Chr12	3782747	3782764	(GCC)6	(GCC)6	(GCC)6	(GCC)6	(GCC)6	(GCC)5	(GCC)6	(GCC)6	(GCC)5	(GCC)6	(GCC)6	-	Introns/Intergenic	-	-	CAGATCTGGGAGGGAGGG	AGGGTATGCAGACACGGC	179	P	A	A	NA
PMS5864	Os_Chr12	3797265	3797282	(GAC)6	(GAC)6	(GAC)6	(GAC)6	(GAC)6	(GAC)6	(GAC)6	(GAC)6	(GAC)6	(GAC)5	(GAC)6	LOC_Os12g07590	CDS	Protein-tyrosine phosphatase domain containing protein, expressed	CODON_DELETION	CTGCATAGACCGACCTGCTC	CCGTGTCTTAATACCCCTCG	276	A	A	A	NA
PMS5865	Os_Chr12	3804528	3804579	(TA)26	(TA)26	(TA)10	(TA)26	(TA)13	(TA)6	(TA)26	(TA)26	(TA)6	(TA)26	(TA)12	-	Introns/Intergenic	-	-	TGCATGGTCAACCATATGAAGC	TTTCACATCCCAGACGAGAGG	238	P	A	A	RM27618
PMS5866	Os_Chr12	3824949	3824962	(CT)7	(CT)7	(CT)7	(CT)7	(CT)7	(CT)7	(CT)7	(CT)6	(CT)7	(CT)7	(CT)7	-	Introns/Intergenic	-	-	TCGGCCTAACAAAACAAAGG	TGGGATCAAGGGAAAAAGATT	267	A	A	A	NA
PMS5867	Os_Chr12	3842141	3842158	(ATC)6	(ATC)9	(ATC)6	(ATC)6	(ATC)6	(ATC)6	(ATC)6	(ATC)6	(ATC)6	(ATC)6	(ATC)6	LOC_Os12g07640	DRR	Osmyb2	-	CCAAGCTTGAGCTAGGCATC	AGCATGGAATTCAACCAACC	194	P	P	P	NA
PMS5868	Os_Chr12	3866779	3866792	(AT)7	(AT)9	(AT)9	(AT)7	(AT)7	(AT)9	(AT)7	(AT)7	(AT)7	(AT)7	(AT)7	-	Introns/Intergenic	-	-	TTCAACTTTGTAAAGCATATCCCTC	TGATTGCATAGATTGAAGGCA	266	P	A	A	NA
PMS5869	Os_Chr12	3879367	3879390	(CCG)8	(CCG)7	(CCG)7	(CCG)8	(CCG)8	(CCG)7	(CCG)8	(CCG)8	(CCG)8	(CCG)8	(CCG)8	LOC_Os12g07730	URR	SWIRM domain containing protein, expressed	-	AAAGCCGAAAAAGTGGGAAG	ACAGGGGAGGTAGGGTTTTG	275	P	A	A	NA
PMS5870	Os_Chr12	3890384	3890399	(TA)8	(TA)8	(TA)8	(TA)8	(TA)8	(TA)8	(TA)8	(TA)8	(TA)8	(TA)7	(TA)8	-	Introns/Intergenic	-	-	CGGGTATATAAACTTTGGGTACG	GCCCTTTTCTCACGTGATTT	239	A	A	A	NA
PMS5871	Os_Chr12	3922688	3922731	(TA)22	(TA)22	(TA)22	(TA)22	(TA)22	(TA)22	(TA)22	(TA)14	(TA)22	(TA)14	(TA)22	-	Introns/Intergenic	-	-	TCAATTTCAACAATGGTGCC	TTCAAAGAAGGTCTTCATGGGT	150	A	A	A	NA
PMS5872	Os_Chr12	3937674	3937694	(CCG)7	(CCG)7	(CCG)7	(CCG)7	(CCG)7	(CCG)7	(CCG)7	(CCG)7	(CCG)7	(CCG)5	(CCG)7	LOC_Os12g07790	CDS	no apical meristem protein, putative, expressed	CODON_DELETION	CATGACGTCTTCTTCGTGGA	CAAGTGGTTCTTCCTTTGCC	198	A	P	A	NA
PMS5873	Os_Chr12	4041892	4041913	(AT)11	(AT)11	(AT)11	(AT)11	(AT)11	(AT)11	(AT)11	(AT)10	(AT)11	(AT)11	(AT)9	-	Introns/Intergenic	-	-	GTCCTAGGCCGTCTTCTTTGTCC	CTAATGTCGTGTTGGGTTGTTCG	247	A	A	A	RM27639
PMS5874	Os_Chr12	4063080	4063109	(GAG)10	(GAG)8	(GAG)10	(GAG)10	(GAG)10	(GAG)6	(GAG)10	(GAG)10	(GAG)10	(GAG)10	(GAG)10	LOC_Os12g08000	CDS	5-nucleotidase domain-containing protein, putative, expressed	CODON_DELETION	CGGGGCCTTACACTGAAATA	CCCTTCACAGGCTCAACTTC	265	P	A	A	NA
PMS5875	Os_Chr12	4110947	4110964	(TCG)6	(TCG)5	(TCG)6	(TCG)6	(TCG)6	(TCG)6	(TCG)6	(TCG)6	(TCG)5	(TCG)6	(TCG)6	LOC_Os12g08080	CDS	transposon protein, putative, unclassified, expressed	CODON_DELETION	CACAAGTTGTATTGCGTGGG	GAAGAACTTGGTGGCTCGTC	209	P	A	A	NA

Polymorphic SSR (PMS) markers	Chromosomes	Start physical positions (bp)	End physical positions (bp)	Nipponbare	Kasalath	Nagina22	IR64	Pokkali	Bala	Tainung67	Azucena	Moroberekan	*Oryza rufipogon*	*Oryza nivara*	MSU gene locus IDs	Structural annotation	Functional annotation	SSR functional effects	Forward primers (5'-3')	Reverse primers (5'-3')	Amplified product size (bp)	Markers exhibiting polymorphism within *indica* rice	Transcrption factor genes-derived markers	Known cloned genes-derived markers	PMS markers corresponding with RM markers
PMS5876	Os_Chr12	4156955	4157042	(TA)44	(TA)29	(TA)44	(TA)44	(TA)44	(TA)44	(TA)44	(TA)14	(TA)44	(TA)44	(TA)11	-	Introns/Intergenic	-	-	GGAAATTTGAAGAAAAAGTTTGGA	TGCTACCTCCGTTTCAGGTT	277	P	A	A	NA
PMS5877	Os_Chr12	4187294	4187323	(TA)15	(TA)14	(TA)13	(TA)15	(TA)15	(TA)14	(TA)15	(TA)15	(TA)15	(TA)15	(TA)15	-	Introns/Intergenic	-	-	CCAGTTGGATGGATTTGTCTG	TCATGTCCTACTTCCTCTGTTTCA	276	P	A	A	NA
PMS5878	Os_Chr12	4228137	4228150	(CT)7	(CT)7	(CT)7	(CT)7	(CT)7	(CT)7	(CT)7	(CT)6	(CT)7	(CT)7	(CT)7	-	Introns/Intergenic	-	-	AACTATTTTCGCATGCGGTC	GAGAGGGGGAGGAGAAAAGA	243	A	A	A	NA
PMS5879	Os_Chr12	4235461	4235478	(TC)9	(TC)8	(TC)9	(TC)9	(TC)6	(TC)9	(TC)9	(TC)9	(TC)9	(TC)9	(TC)9	-	Introns/Intergenic	-	-	CCTTGGACTAGAGACGTGCC	AGAATATGTCGTCGCGTTCC	136	P	A	A	NA
PMS5880	Os_Chr12	4414776	4414803	(AT)14	(AT)14	(AT)14	(AT)11	(AT)14	(AT)11	(AT)6	(AT)14	(AT)14	(AT)14	(AT)14	-	Introns/Intergenic	-	-	ACCGTGTCGACGTCATGTAA	CCCTGGAATCGACTAACACC	223	P	A	A	NA
PMS5881	Os_Chr12	4434927	4434944	(TC)9	(TC)9	(TC)9	(TC)9	(TC)6	(TC)9	(TC)9	(TC)9	(TC)9	(TC)9	(TC)9	-	Introns/Intergenic	-	-	CCTTGACCAAACCCTATCCA	CTCTCCCTCCACAAGCCAT	146	P	A	A	NA
PMS5882	Os_Chr12	4468259	4468272	(CA)7	(CA)7	(CA)7	(CA)7	(CA)6	(CA)7	(CA)7	(CA)7	(CA)7	(CA)7	(CA)7	-	Introns/Intergenic	-	-	GGGGTTGACGTGGTGTTTAG	AAGGCCGAAGGGTAGAGAAG	180	P	A	A	NA
PMS5883	Os_Chr12	4500324	4500339	(CT)8	(CT)7	(CT)8	(CT)8	(CT)8	(CT)8	(CT)8	(CT)8	(CT)8	(CT)8	(CT)7	-	Introns/Intergenic	-	-	TTGCTGACACTAGCCGTTTC	ACAAACATGTCCCAGCTGAA	279	P	A	A	NA
PMS5884	Os_Chr12	4572052	4572069	(CCG)6	(CCG)6	(CCG)6	(CCG)6	(CCG)6	(CCG)6	(CCG)6	(CCG)6	(CCG)6	(CCG)5	(CCG)6	LOC_Os12g08850	CDS	expressed protein	CODON_DELETION	CCGTAGAAGGGAAGGACCTC	CCATGTCGTCGGAGAAGAAG	205	A	A	A	NA
PMS5885	Os_Chr12	4604667	4604681	(TCG)5	(TCG)5	(TCG)5	(TCG)5	(TCG)5	(TCG)5	(TCG)5	(TCG)5	(TCG)5	(TCG)6	(TCG)5	-	Introns/Intergenic	-	-	CAATATCACAACCGTCGGC	CTCCTTCTCCCTCTCCCAAA	235	A	A	A	NA
PMS5886	Os_Chr12	4624100	4624123	(AT)12	(AT)12	(AT)12	(AT)12	(AT)8	(AT)12	(AT)6	(AT)12	(AT)12	(AT)12	(AT)12	-	Introns/Intergenic	-	-	CTGCAATGGCTCAACCATAA	TACTTCCTGGGAGCATGGAG	203	P	A	A	NA
PMS5887	Os_Chr12	4746206	4746241	(TTG)12	(TTG)12	(TTG)12	(TTG)12	(TTG)12	(TTG)12	(TTG)12	(TTG)12	(TTG)12	(TTG)12	(TTG)11	-	Introns/Intergenic	-	-	GGATTAAAGCACACGGGAAA	GAGAGACGCCGTAGGATCTG	261	A	A	A	NA
PMS5888	Os_Chr12	4746647	4746670	(GGC)8	(GGC)8	(GGC)8	(GGC)8	(GGC)8	(GGC)8	(GGC)6	(GGC)8	(GGC)8	(GGC)8	(GGC)8	LOC_Os12g09080	CDS	OsBRXL6	CODON_DELETION	ATTTCAAAACCACCTCGTCG	GAAGTCGCTGATGACGTTGA	154	A	A	P	NA

Polymorphic SSR (PMS) markers	Chromosomes	Start physical positions (bp)	End physical positions (bp)	Nipponbare	Kasalath	Nagina22	IR64	Pokkali	Bala	Tainung67	Azucena	Moroberekan	*Oryza rufipogon*	*Oryza nivara*	MSU gene locus IDs	Structural annotation	Functional annotation	SSR functional effects	Forward primers (5'-3')	Reverse primers (5'-3')	Amplified product size (bp)	Markers exhibiting polymorphism within *indica* rice	Transcrption factor genes-derived markers	Known cloned genes-derived markers	PMS markers corresponding with RM markers
PMS5889	Os_Chr12	4775774	4775794	(GAG)7	(GAG)7	(GAG)6	(GAG)7	(GAG)7	(GAG)7	(GAG)7	(GAG)7	(GAG)7	(GAG)7	(GAG)7	-	Introns/Intergenic	-	-	TAATTCCTCCCCCACAACTG	TCCCTTATGGACTCGGATGA	197	P	A	A	NA
PMS5890	Os_Chr12	4829877	4829928	(TGTA)13	(TGTA)12	(TGTA)13	(TGTA)13	(TGTA)13	(TGTA)11	(TGTA)13	(TGTA)13	(TGTA)11	(TGTA)13	(TGTA)13	-	Introns/Intergenic	-	-	TCTTGGTATGTATTGGCAGCGAAAGC	AGGATGTGAATGAAGGCGAATGG	161	P	A	A	RM7619
PMS5891	Os_Chr12	4965276	4965357	(TA)41	(TA)41	(TA)41	(TA)41	(TA)18	(TA)41	(TA)8	(TA)10	(TA)41	(TA)14	(TA)41	-	Introns/Intergenic	-	-	GAAAGAAATACGCCGTTTATGA	CGTCCATACCACTTCACACG	236	P	A	A	NA
PMS5892	Os_Chr12	4967805	4967818	(TC)7	(TC)7	(TC)6	(TC)7	(TC)7	(TC)7	(TC)7	(TC)7	(TC)7	(TC)7	(TC)7	-	Introns/Intergenic	-	-	CTCCGCCTGGAAAAATATGA	GGGGTGGAGAGGAGAAGAGA	179	P	A	A	NA
PMS5893	Os_Chr12	4983635	4983648	(GA)7	(GA)7	(GA)6	(GA)7	(GA)7	(GA)7	(GA)7	(GA)7	(GA)7	(GA)7	(GA)7	-	Introns/Intergenic	-	-	CAGTCGTCGTTTGACCTCCT	AACTTTATGTGGTCCGCCTG	180	P	A	A	NA
PMS5894	Os_Chr12	5002208	5002265	(TA)29	(TA)29	(TA)29	(TA)29	(TA)12	(TA)29	(TA)9	(TA)29	(TA)29	(TA)29	(TA)29	-	Introns/Intergenic	-	-	ATCACGGTGTTGCGGTTTAT	TCAATTGGCTAAAAGGACGG	277	P	A	A	NA
PMS5895	Os_Chr12	5040855	5040878	(GA)12	(GA)11	(GA)12	(GA)12	(GA)12	(GA)12	(GA)12	(GA)12	(GA)12	(GA)11	(GA)12	LOC_Os12g09580	URR	OsSGS3	-	TTGTGTGTGTTGGGGTTTTC	GTGAGGACCAAACGAGCATT	155	P	A	P	NA
PMS5896	Os_Chr12	5076589	5076612	(CGG)8	(CGG)5	(CGG)8	(CGG)8	(CGG)8	(CGG)8	(CGG)8	(CGG)8	(CGG)7	(CGG)8	(CGG)8	LOC_Os12g09640	CDS	protein phosphatase 2C, putative, expressed	CODON_DELETION	CGGCTCTATACCGGCAAGAATGG	CTCTGGTGCAAGGTGTCCAACC	176	P	A	A	RM27709
PMS5897	Os_Chr12	5079014	5079043	(TA)15	(TA)15	(TA)10	(TA)7	(TA)15	(TA)6	(TA)15	(TA)15	(TA)15	(TA)15	(TA)7	-	Introns/Intergenic	-	-	GACCTGGTAAAATGCGTCGT	CTTCCCTTGATAAGGACATTCA	239	P	A	A	NA
PMS5898	Os_Chr12	5188941	5189004	(TA)32	(TA)11	(TA)8	(TA)32	(TA)32	(TA)32	(TA)32	(TA)16	(TA)32	(TA)32	(TA)32	-	Introns/Intergenic	-	-	TTTTTCAGATTACGTGGCCC	TAGGTGGGATGGAGAGATCG	278	P	A	A	NA
PMS5899	Os_Chr12	5274023	5274046	(AT)12	(AT)12	(AT)10	(AT)12	(AT)12	(AT)10	(AT)12	(AT)12	(AT)12	(AT)12	(AT)12	-	Introns/Intergenic	-	-	TCTAGCTGCACGTGCGTAAT	GGGAGGGGAGGTGAGTTTAG	184	P	A	A	NA
PMS5900	Os_Chr12	5276376	5276409	(TA)17	(TA)17	(TA)17	(TA)17	(TA)17	(TA)17	(TA)17	(TA)8	(TA)6	(TA)17	(TA)17	-	Introns/Intergenic	-	-	GGGAGGGAGGGGTACAGAAT	TGCATGGCTGTTCTTCTTTG	267	A	A	A	NA
PMS5901	Os_Chr12	5511208	5511249	(TA)21	(TA)21	(TA)21	(TA)21	(TA)8	(TA)21	(TA)8	(TA)7	(TA)6	(TA)21	(TA)21	-	Introns/Intergenic	-	-	TCCTATGTGCACGTCCTTCA	GTCCCTTCACCAAACTCCAA	161	P	A	A	NA

Polymorphic SSR (PMS) markers	Chromosomes	Start physical positions (bp)	End physical positions (bp)	Nipponbare	Kasalath	Nagina22	IR64	Pokkali	Bala	Tainung67	Azucena	Moroberekan	*Oryza rufipogon*	*Oryza nivara*	MSU gene locus IDs	Structural annotation	Functional annotation	SSR functional effects	Forward primers (5'-3')	Reverse primers (5'-3')	Amplified product size (bp)	Markers exhibiting polymorphism within *indica* rice	Transcrption factor genes-derived markers	Known cloned genes-derived markers	PMS markers corresponding with RM markers
PMS5902	Os_Chr12	5583025	5583036	(TC)6	(TC)6	(TC)6	(TC)7	(TC)6	(TC)6	(TC)6	(TC)6	(TC)6	(TC)6	(TC)6	-	Introns/Intergenic	-	-	ATCGACACTGACCACCCATT	CAATTCCCCATTGTCTTGCT	113	P	A	A	NA
PMS5903	Os_Chr12	5717642	5717659	(TGC)6	(TGC)6	(TGC)6	(TGC)5	(TGC)6	(TGC)6	(TGC)6	(TGC)6	(TGC)6	(TGC)6	(TGC)6	LOC_Os12g10680	CDS	retrotransposon protein, putative, unclassified, expressed	CODON_DELETION	CATTCTGACGGACATCATCG	CAGAGAAACTTACCGCCGAG	203	P	P	A	NA
PMS5904	Os_Chr12	5718523	5718546	(TGC)8	(TGC)6	(TGC)8	(TGC)8	(TGC)8	(TGC)8	(TGC)8	(TGC)8	(TGC)8	(TGC)8	(TGC)8	LOC_Os12g10680	CDS	retrotransposon protein, putative, unclassified, expressed	CODON_DELETION	AGGCTCTCATTCTGACGGAC	CAGAGAAACTTACCGCCGAG	216	P	P	A	NA
PMS5905	Os_Chr12	6005690	6005707	(GAC)6	(GAC)6	(GAC)6	(GAC)6	(GAC)6	(GAC)6	(GAC)6	(GAC)6	(GAC)6	(GAC)6	(GAC)5	-	Introns/Intergenic	-	-	GAGAACGGCTTGACGGAG	ATCATCGTTTCCTCGTCACC	248	A	A	A	NA
PMS5906	Os_Chr12	6047119	6047146	(AT)14	(AT)14	(AT)9	(AT)14	(AT)14	(AT)7	(AT)14	(AT)14	(AT)14	(AT)14	(AT)14	-	Introns/Intergenic	-	-	GCTAGCGAGCAAATGAATTGT	CTGTTTCCTGCATGGTTGATT	274	P	A	A	NA
PMS5907	Os_Chr12	6214543	6214558	(AT)8	(AT)9	(AT)8	(AT)8	(AT)9	(AT)8	(AT)8	(AT)8	(AT)8	(AT)8	(AT)8	-	Introns/Intergenic	-	-	CCCTGGATCTAAACACCACC	AAGGGGGAAACATGTTGTGA	150	P	A	A	NA
PMS5908	Os_Chr12	6266722	6266747	(AT)13	(AT)13	(AT)13	(AT)13	(AT)13	(AT)13	(AT)10	(AT)13	(AT)7	(AT)13	(AT)13	-	Introns/Intergenic	-	-	AGAGCTCAGCGCCTAAGTTATTGG	ACGTATGGGTGTCTCTCGTCAAGG	685	A	A	A	RM27749
PMS5909	Os_Chr12	6361145	6361160	(GC)8	(GC)8	(GC)7	(GC)8	(GC)8	(GC)8	(GC)8	(GC)8	(GC)8	(GC)8	(GC)8	-	Introns/Intergenic	-	-	CAGGAGGAGAAAGAACGTGG	CCCTGTTGACCAGTTGAGGT	185	P	A	A	NA
PMS5910	Os_Chr12	6393287	6393302	(AG)8	(AG)6	(AG)8	(AG)8	(AG)8	(AG)8	(AG)8	(AG)8	(AG)8	(AG)8	(AG)6	-	Introns/Intergenic	-	-	GCAGAGTGCAAACAGACAGG	AACCGGATATTCTTCACCCC	243	P	A	A	NA
PMS5911	Os_Chr12	6456767	6456794	(TA)14	(TA)14	(TA)14	(TA)14	(TA)14	(TA)14	(TA)14	(TA)14	(TA)6	(TA)14	(TA)14	-	Introns/Intergenic	-	-	TTTTTCACCGGCTGATTTTC	CCAGGAAGCACCAAACAAAT	189	A	A	A	NA
PMS5912	Os_Chr12	6459049	6459068	(TA)10	(TA)10	(TA)10	(TA)10	(TA)10	(TA)10	(TA)10	(TA)10	(TA)10	(TA)9	(TA)10	-	Introns/Intergenic	-	-	TAATTTCGGTGTGATTGGCA	GACGTAGCAGTGTGGACGAA	137	A	A	A	NA
PMS5913	Os_Chr12	6466724	6466755	(AT)16	(AT)14	(AT)16	(AT)16	(AT)16	(AT)13	(AT)11	(AT)16	(AT)16	(AT)16	(AT)12	-	Introns/Intergenic	-	-	AAAAATTGAGTGATTTTGTGCAT	TTGAATCAACCGGAGAAAATCT	219	P	A	A	NA
PMS5914	Os_Chr12	6483828	6483841	(TC)7	(TC)7	(TC)6	(TC)7	(TC)7	(TC)7	(TC)7	(TC)7	(TC)7	(TC)7	(TC)7	-	Introns/Intergenic	-	-	CTGTGACTCTGTGGGCTTGA	TTCCTCTATATTGCCGCCAC	206	P	A	A	NA

Polymorphic SSR (PMS) markers	Chromosomes	Start physical positions (bp)	End physical positions (bp)	Nipponbare	Kasalath	Nagina22	IR64	Pokkali	Bala	Tainung67	Azucena	Moroberekan	*Oryza rufipogon*	*Oryza nivara*	MSU gene locus IDs	Structural annotation	Functional annotation	SSR functional effects	Forward primers (5'-3')	Reverse primers (5'-3')	Amplified product size (bp)	Markers exhibiting polymorphism within *indica* rice	Transcrption factor genes-derived markers	Known cloned genes-derived markers	PMS markers corresponding with RM markers
PMS5915	Os_Chr12	6511832	6511851	(TA)10	(TA)10	(TA)10	(TA)10	(TA)8	(TA)10	(TA)10	(TA)10	(TA)10	(TA)10	(TA)10	-	Introns/Intergenic	-	-	CTAATGGGCGGGTGAAGTTA	TTCAAAAGTTTACATACCCGATTT	187	P	A	A	NA
PMS5916	Os_Chr12	6590588	6590608	(CAG)7	(CAG)7	(CAG)6	(CAG)7	(CAG)7	(CAG)6	(CAG)7	(CAG)6	(CAG)6	(CAG)7	(CAG)7	LOC_Os12g12020	CDS	retrotransposon protein, putative, Ty1-copia subclass	CODON_DELETION	ACAACGGGAACAAGAACAGG	GAGATCTGCACCGAGTAGGC	276	P	A	A	NA
PMS5917	Os_Chr12	6712856	6712903	(ATCT)12	(ATCT)5	(ATCT)11	(ATCT)13	(ATCT)11	(ATCT)9	(ATCT)12	(ATCT)12	(ATCT)11	(ATCT)6	(ATCT)8	-	Introns/Intergenic	-	-	AAAACCCAACCATTCATCCA	GAAGTGTCACATCCGTCCAA	132	P	A	A	NA
PMS5918	Os_Chr12	6734504	6734542	(ATT)13	(ATT)14	(ATT)13	(ATT)13	(ATT)13	(ATT)13	(ATT)12	(ATT)13	(ATT)13	(ATT)13	(ATT)13	-	Introns/Intergenic	-	-	GGCGTTTTAAAAAGGAAGGG	CGACTAAATTGAACTTTTGGTGAA	246	P	A	A	NA
PMS5919	Os_Chr12	6979287	6979300	(AC)7	(AC)7	(AC)7	(AC)7	(AC)7	(AC)7	(AC)6	(AC)7	(AC)7	(AC)7	(AC)7	-	Introns/Intergenic	-	-	TGGAGGGTGTTTTTACTCCG	TGGGTTTGGGGAGATTGTTA	255	A	A	A	NA
PMS5920	Os_Chr12	7168241	7168261	(CGG)7	(CGG)7	(CGG)7	(CGG)7	(CGG)7	(CGG)7	(CGG)6	(CGG)7	(CGG)7	(CGG)7	(CGG)5	LOC_Os12g12934	CDS	peptide transporter PTR3-A, putative, expressed	CODON_CHANGE_PLUS_CODON_DELETION	GCTGGGCCTGTACGTGAT	AAGGCGAACCAGTTGAAGAA	165	A	A	A	NA
PMS5921	Os_Chr12	7186875	7186892	(CTG)6	(CTG)6	(CTG)6	(CTG)6	(CTG)5	(CTG)6	(CTG)6	(CTG)6	(CTG)6	(CTG)5	(CTG)5	LOC_Os12g12970	CDS	RWP-RK domain-containing protein, putative, expressed	CODON_DELETION	TCACCACTGCCATTGTTGTT	ACGACGCACATAGAAGAGCA	181	P	P	A	NA
PMS5922	Os_Chr12	7264354	7264368	(CCG)5	(CCG)5	(CCG)5	(CCG)5	(CCG)5	(CCG)5	(CCG)5	(CCG)5	(CCG)5	(CCG)7	(CCG)5	-	Introns/Intergenic	-	-	CTTTGTCGCCGACTCCAC	CGTTCTCGTTCAAACGTTCC	245	A	A	A	NA
PMS5923	Os_Chr12	7293414	7293437	(CT)12	(CT)12	(CT)12	(CT)12	(CT)12	(CT)12	(CT)12	(CT)11	(CT)12	(CT)12	(CT)11	LOC_Os12g13120	URR	histidine triad family protein, putative, expressed	-	CTTCCGGATAAAACGCAGAG	CGTGTGCTTTGGAGTTCGT	102	A	A	A	NA
PMS5924	Os_Chr12	7334291	7334311	(CAG)7	(CAG)7	(CAG)7	(CAG)6	(CAG)6	(CAG)7	(CAG)7	(CAG)7	(CAG)7	(CAG)7	(CAG)6	LOC_Os12g13170	CDS	OsZIP-1a|OsbZIP86	CODON_DELETION	CTGATGCCCCAGAGGTTTTA	TGGCAATGCTGATCCTACTG	255	P	P	P	NA
PMS5925	Os_Chr12	7360327	7360342	(AC)8	(AC)8	(AC)8	(AC)8	(AC)9	(AC)8	(AC)8	(AC)8	(AC)8	(AC)8	(AC)9	-	Introns/Intergenic	-	-	ACCGCCATTACAACCATCAT	CGGAGCAGAGTGTACCAACA	265	P	A	A	NA
PMS5926	Os_Chr12	7383302	7383331	(AT)15	(AT)15	(AT)15	(AT)7	(AT)15	(AT)15	(AT)15	(AT)15	(AT)9	(AT)15	(AT)15	-	Introns/Intergenic	-	-	AGGTAGAGCATGGGGGATTT	ATCCGGTATGCATGGATGTT	226	P	A	A	NA
PMS5927	Os_Chr12	7430572	7430589	(CCG)6	(CCG)6	(CCG)6	(CCG)6	(CCG)5	(CCG)5	(CCG)5	(CCG)6	(CCG)6	(CCG)5	(CCG)6	LOC_Os12g13320	URR	argininosuccinate synthase, chloroplast precursor, putative, expressed	-	TAGCCCACCACCTTTTCAAC	AAGAAGATCTAGCTCACCGGC	245	P	A	A	NA

Polymorphic SSR (PMS) markers	Chromosomes	Start physical positions (bp)	End physical positions (bp)	Nipponbare	Kasalath	Nagina22	IR64	Pokkali	Bala	Tainung67	Azucena	Moroberekan	*Oryza rufipogon*	*Oryza nivara*	MSU gene locus IDs	Structural annotation	Functional annotation	SSR functional effects	Forward primers (5'-3')	Reverse primers (5'-3')	Amplified product size (bp)	Markers exhibiting polymorphism within *indica* rice	Transcrption factor genes-derived markers	Known cloned genes-derived markers	PMS markers corresponding with RM markers
PMS5928	Os_Chr12	7430724	7430744	(TCT)7	(TCT)7	(TCT)7	(TCT)7	(TCT)7	(TCT)7	(TCT)7	(TCT)6	(TCT)7	(TCT)7	(TCT)7	-	Introns/Intergenic	-	-	AATACTCCCCCACCAACCTC	CCGCAAAAACCAATCAGAAC	256	A	A	A	NA
PMS5929	Os_Chr12	7464201	7464306	(TA)53	(TA)53	(TA)8	(TA)53	(TA)9	(TA)8	(TA)53	(TA)10	(TA)53	(TA)53	(TA)8	-	Introns/Intergenic	-	-	CTGGAGTGGAGAAGAGAGAACAGG	TCTCCGCTCGGTTTCATCTAGG	317	P	A	A	RM3103
PMS5930	Os_Chr12	7476698	7476717	(CT)10	(CT)9	(CT)10	(CT)10	(CT)10	(CT)10	(CT)10	(CT)10	(CT)10	(CT)10	(CT)10	-	Introns/Intergenic	-	-	TAGTTGTTGCTGCGGAGATG	AGCAAGCCACAGCAATCTTT	252	P	A	A	NA
PMS5931	Os_Chr12	7546811	7546828	(CCG)6	(CCG)6	(CCG)6	(CCG)6	(CCG)6	(CCG)6	(CCG)6	(CCG)5	(CCG)6	(CCG)6	(CCG)6	LOC_Os12g13460	CDS	SET domain-containing protein, putative, expressed	CODON_CHANGE_PLUS_CODON_DELETION	ACACCCTCCTCCCCTCCT	GAAATCCCCGCTTGCTAAC	223	A	A	A	NA
PMS5932	Os_Chr12	7582147	7582168	(CT)11	(CT)11	(CT)11	(CT)11	(CT)11	(CT)11	(CT)11	(CT)11	(CT)11	(CT)11	(CT)6	-	Introns/Intergenic	-	-	CCACATATCAATGGCCTATCTTCC	AGCAGCAACTTCCTCATGTTGG	163	A	A	A	RM27819
PMS5933	Os_Chr12	7671181	7671212	(ATCT)8	(ATCT)8	(ATCT)8	(ATCT)7	(ATCT)8	(ATCT)8	(ATCT)8	(ATCT)8	(ATCT)8	(ATCT)8	(ATCT)8	-	Introns/Intergenic	-	-	GACACGCTTTAAGAACCTTCC	TGGATTAGGAGCGTTTGTAGG	213	P	A	A	RM27823
PMS5934	Os_Chr12	7685779	7685796	(CCT)6	(CCT)5	(CCT)6	(CCT)6	(CCT)6	(CCT)5	(CCT)6	(CCT)6	(CCT)6	(CCT)5	(CCT)6	LOC_Os12g13660	URR	expressed protein	-	GGAGAGAGCTCAGAGAGGCA	GAAACCCTAGCCAACCCATC	205	P	A	A	NA
PMS5935	Os_Chr12	7770857	7770904	(AGAT)12	(AGAT)12	(AGAT)11	(AGAT)12	(AGAT)12	(AGAT)10	(AGAT)12	(AGAT)12	(AGAT)12	(AGAT)12	(AGAT)11	-	Introns/Intergenic	-	-	GCTTGAGAGCGTTTTGAGGA	TCTTTAAAGGCACCGTTTGATT	232	P	A	A	NA
PMS5936	Os_Chr12	7813593	7813614	(GA)11	(GA)11	(GA)6	(GA)9	(GA)7	(GA)11	(GA)11	(GA)11	(GA)11	(GA)6	(GA)11	-	Introns/Intergenic	-	-	TCACAACCCTCTACCAAGGC	TTATTAGTCGCTCGGTCGCT	139	P	A	A	NA
PMS5937	Os_Chr12	7841244	7841287	(ATAG)11	(ATAG)11	(ATAG)11	(ATAG)11	(ATAG)6	(ATAG)11	(ATAG)11	(ATAG)11	(ATAG)11	(ATAG)11	(ATAG)11	-	Introns/Intergenic	-	-	ACGTGACAGCTTGAGAGCGT	TCACTGAGCTCGTCTCCTGA	147	P	A	A	NA
PMS5938	Os_Chr12	7848366	7848383	(CCA)6	(CCA)5	(CCA)6	(CCA)5	(CCA)6	(CCA)6	(CCA)6	(CCA)6	(CCA)6	(CCA)6	(CCA)6	LOC_Os12g13860	CDS	retrotransposon protein, putative, unclassified, expressed	CODON_DELETION	CCTCTAGCATGAGCTCCACC	TCCGGGTATGGTTCTGGATA	116	P	A	A	NA
PMS5939	Os_Chr12	7881623	7881694	(ATAG)18	(ATAG)18	(ATAG)18	(ATAG)18	(ATAG)11	(ATAG)18	(ATAG)18	(ATAG)18	(ATAG)18	(ATAG)17	(ATAG)18	-	Introns/Intergenic	-	-	GCTTGAGAGCGTTTTGAGGA	AGTCAGCTCCGATCAAAGGA	279	P	A	A	NA
PMS5940	Os_Chr12	7889729	7889756	(AT)14	(AT)14	(AT)14	(AT)14	(AT)14	(AT)14	(AT)7	(AT)14	(AT)7	(AT)14	(AT)14	-	Introns/Intergenic	-	-	GTTGCAACGGGTAAAGGCTA	CGTCATCCGTGTATGAGTCG	267	A	A	A	NA

Polymorphic SSR (PMS) markers	Chromosomes	Start physical positions (bp)	End physical positions (bp)	Nipponbare	Kasalath	Nagina22	IR64	Pokkali	Bala	Tainung67	Azucena	Moroberekan	*Oryza rufipogon*	*Oryza nivara*	MSU gene locus IDs	Structural annotation	Functional annotation	SSR functional effects	Forward primers (5'-3')	Reverse primers (5'-3')	Amplified product size (bp)	Markers exhibiting polymorphism within *indica* rice	Transcrption factor genes-derived markers	Known cloned genes-derived markers	PMS markers corresponding with RM markers
PMS5941	Os_Chr12	8033963	8033974	(CT)6	(CT)6	(CT)6	(CT)7	(CT)6	(CT)6	(CT)6	(CT)6	(CT)6	(CT)6	(CT)6	-	Introns/Intergenic	-	-	TTAATGGTGGATCCTAGCGG	CTGCATCGAACACAGCCTAA	222	P	A	A	NA
PMS5942	Os_Chr12	8145398	8145419	(TC)11	(TC)10	(TC)11	(TC)11	(TC)11	(TC)11	(TC)11	(TC)11	(TC)11	(TC)11	(TC)11	-	Introns/Intergenic	-	-	CCTCGGCATTATCTCTTTCTCTCC	CTCCTCCATCCATCAGAGAATGC	270	P	A	A	RM27847
PMS5943	Os_Chr12	8249731	8249744	(GA)7	(GA)6	(GA)7	(GA)7	(GA)7	(GA)7	(GA)7	(GA)7	(GA)7	(GA)7	(GA)7	-	Introns/Intergenic	-	-	AGGCGGCGGTAGAGATTAAA	GTCCTTCCCTCACCTCCAG	256	P	A	A	NA
PMS5944	Os_Chr12	8330051	8330066	(TA)8	(TA)8	(TA)8	(TA)8	(TA)8	(TA)8	(TA)8	(TA)6	(TA)6	(TA)8	(TA)8	-	Introns/Intergenic	-	-	TTCCATTCATTTGTGCGGTA	TTCGGCTTTTTATTCATGCC	248	A	A	A	NA
PMS5945	Os_Chr12	8380056	8380081	(AT)13	(AT)11	(AT)13	(AT)13	(AT)13	(AT)13	(AT)13	(AT)13	(AT)13	(AT)13	(AT)13	-	Introns/Intergenic	-	-	AGCCACGGAAATGAGTTTGT	GGTTTGGTGCCAACACCTTA	239	P	A	A	NA
PMS5946	Os_Chr12	8490922	8490997	(TA)38	(TA)38	(TA)38	(TA)38	(TA)38	(TA)11	(TA)38	(TA)30	(TA)15	(TA)38	(TA)38	-	Introns/Intergenic	-	-	ACCCAGAGCCTGAGTGTGAC	TCCCAACTATTCCCAACCAG	248	P	A	A	NA
PMS5947	Os_Chr12	8707234	8707285	(AT)26	(AT)8	(AT)26	(AT)26	(AT)26	(AT)26	(AT)26	(AT)12	(AT)12	(AT)7	(AT)8	-	Introns/Intergenic	-	-	GGAGGTGAACTAAACACGGC	GGGGCTGTATCAGGTGTCAT	266	P	A	A	NA
PMS5948	Os_Chr12	8711527	8711540	(GA)7	(GA)7	(GA)7	(GA)7	(GA)7	(GA)7	(GA)7	(GA)7	(GA)7	(GA)7	(GA)6	-	Introns/Intergenic	-	-	GTGTCCCTTGCCACTGCT	AATATGGGACCCACCTGTCA	234	A	A	A	NA
PMS5949	Os_Chr12	8959133	8959148	(TA)8	(TA)8	(TA)8	(TA)8	(TA)8	(TA)8	(TA)8	(TA)8	(TA)8	(TA)11	(TA)8	-	Introns/Intergenic	-	-	GGCCCATGTGTGAAATCTG	GCACATAACATTTCCATGCG	249	A	A	A	NA
PMS5950	Os_Chr12	9209825	9209842	(CGG)6	(CGG)6	(CGG)6	(CGG)6	(CGG)6	(CGG)6	(CGG)6	(CGG)5	(CGG)6	(CGG)6	(CGG)6	LOC_Os12g16130	CDS	transposon protein, putative, unclassified, expressed	CODON_DELETION	GCAGACGAATCCACGACC	TGGTGACAGCATTGTCGATT	250	A	A	A	NA
PMS5951	Os_Chr12	9374348	9374361	(TA)7	(TA)7	(TA)7	(TA)6	(TA)6	(TA)7	(TA)7	(TA)7	(TA)7	(TA)7	(TA)7	-	Introns/Intergenic	-	-	GCACCATGGAGTTCCGTATG	GATCAAGATTGGTCGTCGGT	190	P	A	A	NA
PMS5952	Os_Chr12	9491020	9491034	(CGC)5	(CGC)5	(CGC)5	(CGC)5	(CGC)5	(CGC)6	(CGC)5	(CGC)5	(CGC)5	(CGC)5	(CGC)5	-	Introns/Intergenic	-	-	AGTCCAAGCTTGCTCATCGT	CACACAGCCTCACGACTCAC	257	P	A	A	NA
PMS5953	Os_Chr12	9524141	9524166	(AT)13	(AT)13	(AT)13	(AT)9	(AT)8	(AT)13	(AT)13	(AT)13	(AT)13	(AT)13	(AT)13	-	Introns/Intergenic	-	-	TCCTTTTGCAGGATGAATTTG	TCAATTTCAACAATGGTGCC	206	P	A	A	NA

Polymorphic SSR (PMS) markers	Chromosomes	Start physical positions (bp)	End physical positions (bp)	Nipponbare	Kasalath	Nagina22	IR64	Pokkali	Bala	Tainung67	Azucena	Moroberekan	*Oryza rufipogon*	*Oryza nivara*	MSU gene locus IDs	Structural annotation	Functional annotation	SSR functional effects	Forward primers (5'-3')	Reverse primers (5'-3')	Amplified product size (bp)	Markers exhibiting polymorphism within *indica* rice	Transcrption factor genes-derived markers	Known cloned genes-derived markers	PMS markers corresponding with RM markers
PMS5954	Os_Chr12	9578109	9578176	(TA)34	(TA)34	(TA)15	(TA)34	(TA)34	(TA)34	(TA)34	(TA)34	(TA)7	(TA)34	(TA)34	-	Introns/Intergenic	-	-	CTATGAACACCAGCCAGCAA	TAGTTTTGGACGGAGCGAAG	234	P	A	A	NA
PMS5955	Os_Chr12	9597528	9597548	(GCC)7	(GCC)5	(GCC)7	(GCC)7	(GCC)7	(GCC)7	(GCC)7	(GCC)7	(GCC)7	(GCC)5	(GCC)7	-	Introns/Intergenic	-	-	CGCTTGTGTGCATGAGAGAT	GACGTTGACATTTGCAGTGG	253	P	A	A	NA
PMS5956	Os_Chr12	9836885	9836900	(GA)8	(GA)8	(GA)7	(GA)8	(GA)8	(GA)8	(GA)8	(GA)8	(GA)8	(GA)8	(GA)8	-	Introns/Intergenic	-	-	ACTTTATTGCACGTGGGGAC	TAAAAAGTCCCCCTTCCCTC	263	P	A	A	NA
PMS5957	Os_Chr12	9864852	9864866	(CTC)5	(CTC)5	(CTC)5	(CTC)5	(CTC)5	(CTC)5	(CTC)5	(CTC)5	(CTC)5	(CTC)7	(CTC)7	LOC_Os12g17230	CDS	expressed protein	CODON_INSERTION	AACAGGAGGAGCCATCGAG	GACCGTTGCAGCACCAAG	181	A	A	A	NA
PMS5958	Os_Chr12	9896629	9896656	(ATAG)7	(ATAG)7	(ATAG)7	(ATAG)7	(ATAG)7	(ATAG)5	(ATAG)7	(ATAG)7	(ATAG)7	(ATAG)7	(ATAG)7	-	Introns/Intergenic	-	-	ACGCGTGAGAGCTCCTAAAA	TCCTTGTGTAACTACCGCCC	139	P	A	A	NA
PMS5959	Os_Chr12	10033999	10034026	(CT)14	(CT)14	(CT)14	(CT)7	(CT)14	(CT)14	(CT)14	(CT)12	(CT)14	(CT)14	(CT)14	-	Introns/Intergenic	-	-	TCGAGTTCAATTCCGAATCC	GAGGAGAGATGGGAGAGCG	189	P	A	A	NA
PMS5960	Os_Chr12	10049039	10049056	(CGC)6	(CGC)6	(CGC)6	(CGC)5	(CGC)6	(CGC)6	(CGC)6	(CGC)6	(CGC)6	(CGC)6	(CGC)6	LOC_Os12g17530	CDS	expressed protein	CODON_DELETION	TTCGAAAAGGCAATCCAATC	AACCCCACCATTAGAAACCC	269	P	A	A	NA
PMS5961	Os_Chr12	10065943	10065960	(GCG)6	(GCG)5	(GCG)6	(GCG)6	(GCG)6	(GCG)5	(GCG)6	(GCG)5	(GCG)6	(GCG)6	(GCG)6	-	Introns/Intergenic	-	-	AGGCTCGAGCTTGATTTCAG	TCGTTTCCTTCATTAACCCG	202	P	A	A	NA
PMS5962	Os_Chr12	10066745	10066764	(TA)10	(TA)10	(TA)10	(TA)10	(TA)10	(TA)10	(TA)6	(TA)10	(TA)10	(TA)10	(TA)10	-	Introns/Intergenic	-	-	GGTAAGGTTTACTCACCTCAAACG	CCGACCTTATCTCCTCCTATGG	118	A	A	A	RM27917
PMS5963	Os_Chr12	10070614	10070631	(CCG)6	(CCG)6	(CCG)6	(CCG)6	(CCG)6	(CCG)6	(CCG)6	(CCG)6	(CCG)6	(CCG)6	(CCG)5	LOC_Os12g17570	CDS	GDSL-like lipase/acylhydrolase, putative, expressed	CODON_DELETION	TGTTGATCTCCTCGTTGCAC	CCGGCATTAGCATCTCTCTC	135	A	A	A	NA
PMS5964	Os_Chr12	10081167	10081205	(GAA)13	(GAA)13	(GAA)13	(GAA)13	(GAA)13	(GAA)14	(GAA)13	(GAA)13	(GAA)13	(GAA)13	(GAA)13	-	Introns/Intergenic	-	-	CGGCCACACCTAAATAAACG	CTTGGTGAGCTGCAGAGATG	276	P	A	A	NA
PMS5965	Os_Chr12	10196047	10196070	(AG)12	(AG)12	(AG)12	(AG)11	(AG)12	(AG)12	(AG)12	(AG)12	(AG)12	(AG)12	(AG)7	LOC_Os12g17804	CDS	expressed protein	FRAME_SHIFT	AAATGTTACTGTGCAGGGGC	CTCTTCTACTTGCCGTTCCG	272	P	A	A	NA
PMS5966	Os_Chr12	10306163	10306184	(GA)11	(GA)11	(GA)10	(GA)11	(GA)11	(GA)7	(GA)11	(GA)11	(GA)11	(GA)11	(GA)11	-	Introns/Intergenic	-	-	CCTCCAGGTTCTTGCATGTT	ATGCTTTCCCGTCTCAAATG	168	P	A	A	NA

Polymorphic SSR (PMS) markers	Chromosomes	Start physical positions (bp)	End physical positions (bp)	Nipponbare	Kasalath	Nagina22	IR64	Pokkali	Bala	Tainung67	Azucena	Moroberekan	*Oryza rufipogon*	*Oryza nivara*	MSU gene locus IDs	Structural annotation	Functional annotation	SSR functional effects	Forward primers (5'-3')	Reverse primers (5'-3')	Amplified product size (bp)	Markers exhibiting polymorphism within *indica* rice	Transcrption factor genes-derived markers	Known cloned genes-derived markers	PMS markers corresponding with RM markers
PMS5967	Os_Chr12	10409391	10409404	(CT)7	(CT)8	(CT)7	(CT)7	(CT)7	(CT)7	(CT)7	(CT)7	(CT)7	(CT)7	(CT)7	-	Introns/Intergenic	-	-	CTAAGCCAAGCCAACTCGAC	TGGGATGGAGAGAGGATGAG	139	P	A	A	NA
PMS5968	Os_Chr12	10478323	10478343	(GCT)7	(GCT)7	(GCT)7	(GCT)7	(GCT)7	(GCT)7	(GCT)7	(GCT)7	(GCT)7	(GCT)6	(GCT)7	LOC_Os12g18150	URR	ZOS12-04 - C2H2 zinc finger protein, expressed	-	GAGGAGGAGGAGTCGCATTT	AAGGGGCAAATAACGGATTC	269	A	P	A	NA
PMS5969	Os_Chr12	10515641	10515658	(GGC)6	(GGC)5	(GGC)6	(GGC)6	(GGC)6	(GGC)6	(GGC)6	(GGC)5	(GGC)6	(GGC)6	(GGC)6	LOC_Os12g18200	CDS	endonuclease/exonuclease/phosphatase family domain containing protein, expressed	CODON_DELETION	AGGGAGTCGGAGTCGGAG	TTTAACTACGCGGCCTAACG	223	P	A	A	NA
PMS5970	Os_Chr12	10602777	10602794	(CT)9	(CT)9	(CT)9	(CT)9	(CT)9	(CT)9	(CT)6	(CT)9	(CT)9	(CT)9	(CT)9	-	Introns/Intergenic	-	-	CCACTCCCCCTCTCTCTCTC	GAGCGGTGGAAAAAGAAAAA	209	A	A	A	NA
PMS5971	Os_Chr12	10653315	10653328	(AT)7	(AT)7	(AT)6	(AT)6	(AT)7	(AT)7	(AT)7	(AT)6	(AT)7	(AT)6	(AT)6	LOC_Os12g18410	DRR	MIR	-	ATGTAATTGGGAGTGTGCCA	GCAGCAGCTCCATACATCAA	218	P	A	P	NA
PMS5972	Os_Chr12	10732577	10732594	(CCG)6	(CCG)6	(CCG)6	(CCG)6	(CCG)6	(CCG)5	(CCG)6	(CCG)6	(CCG)6	(CCG)6	(CCG)6	LOC_Os12g18560	CDS	invertase/pectin methylesterase inhibitor family protein, putative, expressed	CODON_DELETION	GGCACAAGGAGAGAAACACC	GTAGGAGGTGGAGTTGCAGG	243	P	A	A	NA
PMS5973	Os_Chr12	10868417	10868428	(AT)6	(AT)6	(AT)6	(AT)6	(AT)6	(AT)7	(AT)6	(AT)6	(AT)6	(AT)6	(AT)6	-	Introns/Intergenic	-	-	TCACGTGAATGCCCATAGAA	TAGCAGTGGGCTTATTCGCT	180	P	A	A	NA
PMS5974	Os_Chr12	10875526	10875557	(AT)16	(AT)16	(AT)16	(AT)16	(AT)16	(AT)16	(AT)7	(AT)16	(AT)16	(AT)16	(AT)16	-	Introns/Intergenic	-	-	TCACGTGAATGCCCATAGAA	AGCAGTGGGCTTATTCGCTA	197	A	A	A	NA
PMS5975	Os_Chr12	10942699	10942718	(AT)10	(AT)10	(AT)10	(AT)10	(AT)10	(AT)8	(AT)10	(AT)10	(AT)10	(AT)10	(AT)10	-	Introns/Intergenic	-	-	TCTATGCTCGTGAGGTGTGG	TGTTCCGTTGTTTCTATGGTG	273	P	A	A	NA
PMS5976	Os_Chr12	10957122	10957163	(GTGCGT)7	(GTGCGT)7	(GTGCGT)7	(GTGCGT)7	(GTGCGT)7	(GTGCGT)7	(GTGCGT)7	(GTGCGT)7	(GTGCGT)7	(GTGCGT)8	(GTGCGT)5	-	Introns/Intergenic	-	-	TGATTAACAATCGCACCGTC	CAACCCCACCACCTCTCTC	243	A	A	A	NA
PMS5977	Os_Chr12	10974999	10975024	(TA)13	(TA)13	(TA)13	(TA)13	(TA)13	(TA)12	(TA)13	(TA)13	(TA)13	(TA)13	(TA)13	-	Introns/Intergenic	-	-	GGTGTGGGTAGTGTTTGAATCTCC	ATACAGCTGGGAGACCGATACG	445	P	A	A	RM27948
PMS5978	Os_Chr12	11005369	11005382	(TA)7	(TA)7	(TA)7	(TA)6	(TA)7	(TA)7	(TA)7	(TA)7	(TA)7	(TA)7	(TA)7	-	Introns/Intergenic	-	-	CGTGTATTTTTGGGGTTTTATACG	AGGGGAGGGGTGTATAATAGAG	278	P	A	A	NA
PMS5979	Os_Chr12	11007902	11007925	(AT)12	(AT)12	(AT)12	(AT)12	(AT)12	(AT)12	(AT)12	(AT)12	(AT)7	(AT)12	(AT)12	-	Introns/Intergenic	-	-	TTCTAGGGGAGGGGTGTAGAA	GTAAGGAACTTGGCAGGCAG	253	A	A	A	NA

Polymorphic SSR (PMS) markers	Chromosomes	Start physical positions (bp)	End physical positions (bp)	Nipponbare	Kasalath	Nagina22	IR64	Pokkali	Bala	Tainung67	Azucena	Moroberekan	*Oryza rufipogon*	*Oryza nivara*	MSU gene locus IDs	Structural annotation	Functional annotation	SSR functional effects	Forward primers (5'-3')	Reverse primers (5'-3')	Amplified product size (bp)	Markers exhibiting polymorphism within *indica* rice	Transcrption factor genes-derived markers	Known cloned genes-derived markers	PMS markers corresponding with RM markers
PMS5980	Os_Chr12	11047712	11047723	(TA)6	(TA)6	(TA)6	(TA)6	(TA)6	(TA)6	(TA)6	(TA)6	(TA)6	(TA)8	(TA)6	-	Introns/Intergenic	-	-	CTAGGACAGCATTGCGTTCA	AAAAGCCATACCTTCCCGAG	121	A	A	A	NA
PMS5981	Os_Chr12	11399913	11399926	(AT)7	(AT)7	(AT)7	(AT)6	(AT)7	(AT)7	(AT)7	(AT)7	(AT)7	(AT)7	(AT)6	-	Introns/Intergenic	-	-	CACAAGGATGAAATGCATGG	TGGCTCAAACAAAGCATTAGTC	132	P	A	A	NA
PMS5982	Os_Chr12	11914591	11914604	(CA)7	(CA)6	(CA)7	(CA)7	(CA)7	(CA)7	(CA)7	(CA)7	(CA)7	(CA)7	(CA)7	-	Introns/Intergenic	-	-	CCAGGTTTCTCCCTCACAAA	TAGAAGGAGGGGTGATGTGG	202	P	A	A	NA
PMS5983	Os_Chr12	11936055	11936096	(AG)21	(AG)21	(AG)21	(AG)21	(AG)21	(AG)21	(AG)21	(AG)21	(AG)20	(AG)21	(AG)21	-	Introns/Intergenic	-	-	ACTAGCGGGAGGTTGGAAGT	GATAGTGGCCCGAACCTGTA	195	A	A	A	NA
PMS5984	Os_Chr12	12136990	12137001	(AT)6	(AT)6	(AT)6	(AT)6	(AT)7	(AT)6	(AT)6	(AT)6	(AT)6	(AT)6	(AT)6	-	Introns/Intergenic	-	-	ACTGCCACTGATGAGCACAA	GTGGGTTTTAGCGTATGGGA	240	P	A	A	NA
PMS5985	Os_Chr12	12204471	12204500	(CCA)10	(CCA)10	(CCA)8	(CCA)10	(CCA)7	(CCA)10	(CCA)9	(CCA)10	(CCA)10	(CCA)5	(CCA)10	LOC_Os12g21710	URR	nnrU, putative, expressed	-	TCCACCACTCTGACGTCTACTAACC	CTGCGGGAAGTGTAGGAGAAGC	141	P	A	A	RM27970
PMS5986	Os_Chr12	12206219	12206236	(TG)9	(TG)9	(TG)9	(TG)9	(TG)9	(TG)8	(TG)9	(TG)9	(TG)9	(TG)9	(TG)9	-	Introns/Intergenic	-	-	TCTTCATAATCCCTTCCGTTG	GCACACACGATGTCAACCAT	247	P	A	A	NA
PMS5987	Os_Chr12	12239761	12239824	(TA)32	(TA)32	(TA)32	(TA)32	(TA)32	(TA)32	(TA)32	(TA)32	(TA)8	(TA)11	(TA)32	-	Introns/Intergenic	-	-	GGACGAGGGTTTAACGCATA	CGATTGGACTCCTAACAGCAG	205	A	A	A	NA
PMS5988	Os_Chr12	12269331	12269351	(GCT)7	(GCT)7	(GCT)7	(GCT)7	(GCT)6	(GCT)7	(GCT)7	(GCT)7	(GCT)7	(GCT)6	(GCT)6	-	Introns/Intergenic	-	-	CCACACTGCCCAGGATTTAAGC	CTGTTCCCATCATCCAAATGACC	282	P	A	A	RM27973
PMS5989	Os_Chr12	12305033	12305124	(AT)46	(AT)46	(AT)32	(AT)17	(AT)36	(AT)46	(AT)46	(AT)25	(AT)46	(AT)46	(AT)7	-	Introns/Intergenic	-	-	TTTGTGTGTTTTATACCTTGGA	TCTGCATAGTTTCCTTTCTTTT	446	P	A	A	NA
PMS5990	Os_Chr12	12444307	12444340	(AT)17	(AT)17	(AT)17	(AT)17	(AT)17	(AT)17	(AT)17	(AT)17	(AT)17	(AT)17	(AT)10	-	Introns/Intergenic	-	-	TGCAATGCTATTAGCGGCTT	GTTTAGTTATTGCGCACCCC	218	A	A	A	NA
PMS5991	Os_Chr12	12447507	12447590	(TA)42	(TA)42	(TA)29	(TA)12	(TA)9	(TA)41	(TA)42	(TA)42	(TA)42	(TA)39	(TA)42	-	Introns/Intergenic	-	-	TATACATTGGAGCCCAGGCA	GTGAGGGAGAGAGGAGCGAT	245	P	A	A	NA
PMS5992	Os_Chr12	12464620	12464633	(AG)7	(AG)7	(AG)7	(AG)6	(AG)6	(AG)6	(AG)7	(AG)7	(AG)7	(AG)7	(AG)6	-	Introns/Intergenic	-	-	ACACCAGCTCTGCTTGACCT	GGAGACCTCCTCCTAATCGG	173	P	A	A	NA

Polymorphic SSR (PMS) markers	Chromosomes	Start physical positions (bp)	End physical positions (bp)	Nipponbare	Kasalath	Nagina22	IR64	Pokkali	Bala	Tainung67	Azucena	Moroberekan	*Oryza rufipogon*	*Oryza nivara*	MSU gene locus IDs	Structural annotation	Functional annotation	SSR functional effects	Forward primers (5'-3')	Reverse primers (5'-3')	Amplified product size (bp)	Markers exhibiting polymorphism within *indica* rice	Transcrption factor genes-derived markers	Known cloned genes-derived markers	PMS markers corresponding with RM markers
PMS5993	Os_Chr12	12618208	12618221	(GC)7	(GC)7	(GC)6	(GC)7	(GC)7	(GC)7	(GC)7	(GC)7	(GC)7	(GC)7	(GC)7	-	Introns/Intergenic	-	-	AAGCGCTATTGTTCATTGCC	AATGGCTAGGGTTTTCAGGC	265	P	A	A	NA
PMS5994	Os_Chr12	12709343	12709363	(GAT)7	(GAT)7	(GAT)7	(GAT)7	(GAT)7	(GAT)7	(GAT)7	(GAT)7	(GAT)7	(GAT)7	(GAT)5	LOC_Os12g22510	CDS	retrotransposon protein, putative, Ty3-gypsy subclass	CODON_DELETION	TGCAGAAGCCAGGAAGAACT	TTGTCTCTAACCCATTCGGC	117	A	A	A	NA
PMS5995	Os_Chr12	12760711	12760742	(TA)16	(TA)16	(TA)16	(TA)16	(TA)16	(TA)16	(TA)16	(TA)16	(TA)16	(TA)7	(TA)16	-	Introns/Intergenic	-	-	GGGACGGAGGGAGTATAGTCTT	TTGTCTTTGATTGATCCATACATTTT	190	A	A	A	NA
PMS5996	Os_Chr12	12842468	12842497	(CCT)10	(CCT)10	(CCT)5	(CCT)10	(CCT)10	(CCT)10	(CCT)10	(CCT)10	(CCT)10	(CCT)10	(CCT)10	-	Introns/Intergenic	-	-	AAGGGGAAAACAGATGGGAT	GACGTTGTCGCTTCTCCCT	263	P	A	A	NA
PMS5997	Os_Chr12	12869102	12869155	(AT)27	(AT)27	(AT)12	(AT)7	(AT)11	(AT)27	(AT)27	(AT)27	(AT)27	(AT)27	(AT)7	-	Introns/Intergenic	-	-	TGTTTGTGGGTTTCTGTCCA	ATTCAGCCCATGTACTTCCG	249	P	A	A	NA
PMS5998	Os_Chr12	12931478	12931489	(TA)6	(TA)8	(TA)6	(TA)8	(TA)8	(TA)8	(TA)8	(TA)6	(TA)6	(TA)8	(TA)8	-	Introns/Intergenic	-	-	TCATGCATGTGGCATTTACA	AAGTGGATAAATCGTAATAAGCAAA	247	P	A	A	NA
PMS5999	Os_Chr12	12942919	12942956	(TA)19	(TA)19	(TA)9	(TA)19	(TA)6	(TA)19	(TA)6	(TA)19	(TA)19	(TA)14	(TA)12	-	Introns/Intergenic	-	-	GGTGTACGTGAGAGGAGCGT	ATTAGATACCATGGTGCCCG	146	P	A	A	NA
PMS6000	Os_Chr12	12951007	12951050	(AT)22	(AT)22	(AT)22	(AT)6	(AT)22	(AT)22	(AT)6	(AT)22	(AT)12	(AT)22	(AT)22	-	Introns/Intergenic	-	-	TGTACACGTGAGGGCCTGTA	CCCACATCCATATTGAAGCC	174	P	A	A	NA
PMS6001	Os_Chr12	13046835	13046848	(CT)7	(CT)9	(CT)7	(CT)7	(CT)7	(CT)7	(CT)7	(CT)7	(CT)7	(CT)7	(CT)7	-	Introns/Intergenic	-	-	CGGTTTCTCTCCTCCTTCCT	CCTCCTCCACTTGCTCGG	114	P	A	A	NA
PMS6002	Os_Chr12	13096568	13096585	(CGC)6	(CGC)6	(CGC)6	(CGC)6	(CGC)6	(CGC)6	(CGC)6	(CGC)6	(CGC)6	(CGC)6	(CGC)5	LOC_Os12g23150	CDS	4-nitrophenylphosphatase-like, putative, expressed	CODON_DELETION	CAACAAAAAGAACGCGACAA	CGGTGCTTCCTCTCCATTAC	269	A	A	A	NA
PMS6003	Os_Chr12	13147458	13147473	(AT)8	(AT)8	(AT)8	(AT)8	(AT)8	(AT)8	(AT)7	(AT)8	(AT)8	(AT)8	(AT)8	-	Introns/Intergenic	-	-	AAGTGGATAAATCGTAATAAGCAAA	TCATGCATGTGGCATTTACA	251	A	A	A	NA
PMS6004	Os_Chr12	13299118	13299135	(GAC)6	(GAC)6	(GAC)6	(GAC)6	(GAC)6	(GAC)5	(GAC)6	(GAC)5	(GAC)6	(GAC)6	(GAC)6	-	Introns/Intergenic	-	-	CATCCGTGTGTTTCGTCAAC	TAACCTGACGACCATGAGCA	156	P	A	A	NA
PMS6005	Os_Chr12	13431979	13431996	(GAC)6	(GAC)5	(GAC)5	(GAC)5	(GAC)6	(GAC)5	(GAC)6	(GAC)5	(GAC)5	(GAC)6	(GAC)6	LOC_Os12g23670	CDS	expressed protein	CODON_DELETION	CACTGGAACTGGATGTGGTG	TAACCTGACGACCATGAGCA	242	P	A	A	NA

Polymorphic SSR (PMS) markers	Chromosomes	Start physical positions (bp)	End physical positions (bp)	Nipponbare	Kasalath	Nagina22	IR64	Pokkali	Bala	Tainung67	Azucena	Moroberekan	*Oryza rufipogon*	*Oryza nivara*	MSU gene locus IDs	Structural annotation	Functional annotation	SSR functional effects	Forward primers (5'-3')	Reverse primers (5'-3')	Amplified product size (bp)	Markers exhibiting polymorphism within *indica* rice	Transcrption factor genes-derived markers	Known cloned genes-derived markers	PMS markers corresponding with RM markers
PMS6006	Os_Chr12	13510416	13510465	(TA)25	(TA)25	(TA)8	(TA)8	(TA)25	(TA)12	(TA)25	(TA)10	(TA)25	(TA)25	(TA)25	-	Introns/Intergenic	-	-	ACAGTGCGCTTCATGTATGG	TTGCGAATGCTAAGTTCCAA	254	P	A	A	NA
PMS6007	Os_Chr12	13520797	13520814	(TA)9	(TA)9	(TA)9	(TA)9	(TA)9	(TA)9	(TA)6	(TA)9	(TA)9	(TA)9	(TA)9	-	Introns/Intergenic	-	-	CCCGATTGAGATGTGGGTAT	ACTCATTTGGTCGTTCCAGT	230	A	A	A	NA
PMS6008	Os_Chr12	13715405	13715419	(CCA)5	(CCA)6	(CCA)5	(CCA)5	(CCA)5	(CCA)5	(CCA)5	(CCA)5	(CCA)6	(CCA)6	(CCA)5	LOC_Os12g24110	CDS	retrotransposon protein, putative, Ty3-gypsy subclass, expressed	CODON_INSERTION	TGCTGGTTCTGTGTGGGATA	ATTTTGGATGTGGTGGAGGA	221	P	A	A	NA
PMS6009	Os_Chr12	13735230	13735244	(TGC)5	(TGC)7	(TGC)5	(TGC)5	(TGC)5	(TGC)5	(TGC)5	(TGC)5	(TGC)5	(TGC)5	(TGC)5	-	Introns/Intergenic	-	-	TGATTGCTTGCTGCTGATTC	GAATTTGAGAACAAACCCGC	189	P	A	A	NA
PMS6010	Os_Chr12	13744051	13744066	(TA)8	(TA)9	(TA)8	(TA)8	(TA)8	(TA)8	(TA)8	(TA)8	(TA)8	(TA)8	(TA)8	-	Introns/Intergenic	-	-	TCAATTATAACCTTCCCCCAA	TTTGCTACCGTGGCACAAT	250	P	A	A	NA
PMS6011	Os_Chr12	13811791	13811811	(CGG)7	(CGG)6	(CGG)7	(CGG)7	(CGG)7	(CGG)6	(CGG)7	(CGG)6	(CGG)6	(CGG)5	(CGG)7	-	Introns/Intergenic	-	-	GCCTCCCCTTCCTCCTCT	TCCATCAGCTGTATTGCACG	273	P	A	A	NA
PMS6012	Os_Chr12	13871695	13871744	(AT)25	(AT)25	(AT)25	(AT)25	(AT)25	(AT)25	(AT)25	(AT)25	(AT)6	(AT)16	(AT)8	-	Introns/Intergenic	-	-	TCCCTCTGTCCTAAAAACCA	TGCGACATGGCTTGTTTTAC	262	A	A	A	NA
PMS6013	Os_Chr12	14109150	14109173	(CGC)8	(CGC)8	(CGC)8	(CGC)8	(CGC)8	(CGC)8	(CGC)8	(CGC)8	(CGC)8	(CGC)5	(CGC)8	-	Introns/Intergenic	-	-	CTACACAGGCCAAGGTCGTT	TTCCATTCGAGCAACCCTAC	272	A	A	A	NA
PMS6014	Os_Chr12	14127545	14127558	(TA)7	(TA)7	(TA)7	(TA)7	(TA)7	(TA)7	(TA)7	(TA)7	(TA)7	(TA)7	(TA)6	-	Introns/Intergenic	-	-	AAGGGAAGATGGTTTGGGAT	AAAAACCCACAAACAGACATCA	183	A	A	A	NA
PMS6015	Os_Chr12	14411812	14411825	(TA)7	(TA)7	(TA)7	(TA)7	(TA)7	(TA)6	(TA)7	(TA)7	(TA)7	(TA)7	(TA)7	-	Introns/Intergenic	-	-	GATAAGACTTGGGTGGACATCACG	CTTCTATGGTCGCAATTCAGATGC	205	P	A	A	RM28050
PMS6016	Os_Chr12	14424132	14424146	(GGC)5	(GGC)5	(GGC)5	(GGC)5	(GGC)6	(GGC)5	(GGC)5	(GGC)5	(GGC)5	(GGC)6	(GGC)5	LOC_Os12g25120	DRR	core histone H2A/H2B/H3/H4, putative, expressed	-	ATCAGGCCACCAAAACAAAC	ACGACGACGACAACTGAGC	187	P	A	A	NA
PMS6017	Os_Chr12	14621482	14621501	(TA)10	(TA)10	(TA)10	(TA)10	(TA)10	(TA)10	(TA)7	(TA)10	(TA)10	(TA)8	(TA)10	-	Introns/Intergenic	-	-	TTTTTATACATACGCACACAAACTT	GAGAAGAAGGGGAGGAGGG	280	A	A	A	NA
PMS6018	Os_Chr12	14743899	14743918	(TA)10	(TA)10	(TA)8	(TA)8	(TA)10	(TA)7	(TA)10	(TA)6	(TA)10	(TA)10	(TA)10	-	Introns/Intergenic	-	-	AGACCATTTCCTTTGGTCCC	ACCAACAACAACAGCAGCAG	231	P	A	A	NA

Polymorphic SSR (PMS) markers	Chromosomes	Start physical positions (bp)	End physical positions (bp)	Nipponbare	Kasalath	Nagina22	IR64	Pokkali	Bala	Tainung67	Azucena	Moroberekan	*Oryza rufipogon*	*Oryza nivara*	MSU gene locus IDs	Structural annotation	Functional annotation	SSR functional effects	Forward primers (5'-3')	Reverse primers (5'-3')	Amplified product size (bp)	Markers exhibiting polymorphism within *indica* rice	Transcrption factor genes-derived markers	Known cloned genes-derived markers	PMS markers corresponding with RM markers
PMS6019	Os_Chr12	14757960	14757971	(AG)6	(AG)9	(AG)6	(AG)6	(AG)6	(AG)6	(AG)6	(AG)6	(AG)6	(AG)6	(AG)6	LOC_Os12g25494	URR	transposon protein, putative, unclassified, expressed	-	TGGAGGATTAGAGACGGTGG	GCTAGCCGAAACCCTAGCTT	200	P	A	A	NA
PMS6020	Os_Chr12	14933086	14933129	(TA)22	(TA)22	(TA)7	(TA)22	(TA)10	(TA)20	(TA)7	(TA)12	(TA)11	(TA)22	(TA)8	-	Introns/Intergenic	-	-	AAGGCACCAGGAATATGACAAGC	GGGATGTGGGATTTGGAGAGG	176	P	A	A	RM28070
PMS6021	Os_Chr12	14959146	14959161	(CT)8	(CT)7	(CT)8	(CT)8	(CT)8	(CT)8	(CT)8	(CT)8	(CT)8	(CT)8	(CT)8	-	Introns/Intergenic	-	-	GAAGACCGACACCCTACCAA	TCGTCAGATCTGGCAGAGG	227	P	A	A	NA
PMS6022	Os_Chr12	15010389	15010408	(CT)10	(CT)6	(CT)10	(CT)10	(CT)10	(CT)10	(CT)10	(CT)10	(CT)10	(CT)10	(CT)10	-	Introns/Intergenic	-	-	AAAAATCCAGCCATTCTCCA	ACCCATCTTTAGTCCCCAGC	188	P	A	A	NA
PMS6023	Os_Chr12	15088639	15088656	(TCG)6	(TCG)6	(TCG)6	(TCG)6	(TCG)6	(TCG)5	(TCG)6	(TCG)6	(TCG)6	(TCG)6	(TCG)6	-	Introns/Intergenic	-	-	CTCCTCGTCGATGTCTCCTC	ACTACAACCGGGAACGATGA	177	P	A	A	NA
PMS6024	Os_Chr12	15147098	15147147	(AT)25	(AT)25	(AT)25	(AT)25	(AT)25	(AT)25	(AT)25	(AT)25	(AT)9	(AT)11	(AT)25	-	Introns/Intergenic	-	-	GCTCCAATTCCTTCTAGGGC	AACGAGCTAGGGAACGTGAA	219	A	A	A	NA
PMS6025	Os_Chr12	15163764	15163805	(AT)21	(AT)21	(AT)21	(AT)9	(AT)21	(AT)21	(AT)21	(AT)21	(AT)21	(AT)21	(AT)13	-	Introns/Intergenic	-	-	ACAAAGTCCGAAGGAATTGTAA	TTTGTTGTGTTGCTTGCTGA	273	P	A	A	NA
PMS6026	Os_Chr12	15325376	15325397	(AT)11	(AT)11	(AT)11	(AT)11	(AT)8	(AT)6	(AT)11	(AT)11	(AT)11	(AT)11	(AT)7	-	Introns/Intergenic	-	-	TGCAGGTCGTGAAAATGAAG	TAAACGGCCTCAAAATGGAA	173	P	A	A	NA
PMS6027	Os_Chr12	15414489	15414524	(ATC)12	(ATC)11	(ATC)12	(ATC)12	(ATC)12	(ATC)12	(ATC)12	(ATC)12	(ATC)11	(ATC)5	(ATC)12	-	Introns/Intergenic	-	-	GGGAGGACACCTGTGTAAGTAGG	GGTTCAAATGAGACCCAATTCC	247	P	A	A	RM28089
PMS6028	Os_Chr12	15459145	15459165	(GCA)7	(GCA)7	(GCA)7	(GCA)7	(GCA)7	(GCA)7	(GCA)7	(GCA)6	(GCA)7	(GCA)7	(GCA)7	-	Introns/Intergenic	-	-	CGAAAATGAATCCTCTTGCC	TACAATGCTCACCATGGACC	264	A	A	A	NA
PMS6029	Os_Chr12	15517658	15517678	(ATA)7	(ATA)6	(ATA)7	(ATA)7	(ATA)7	(ATA)7	(ATA)7	(ATA)6	(ATA)7	(ATA)7	(ATA)6	-	Introns/Intergenic	-	-	GGTCCGTACGTCCCAATGTA	TCCAATTTAACTATGCACTCCA	254	P	A	A	NA
PMS6030	Os_Chr12	15517905	15517934	(AT)15	(AT)15	(AT)15	(AT)15	(AT)7	(AT)15	(AT)15	(AT)15	(AT)15	(AT)15	(AT)15	-	Introns/Intergenic	-	-	AAAAGTTTACTCACTCCATACCGTG	GCTCATCCAATGAGGTAGCC	220	P	A	A	NA
PMS6031	Os_Chr12	15592550	15592585	(AT)18	(AT)18	(AT)10	(AT)17	(AT)14	(AT)18	(AT)6	(AT)18	(AT)18	(AT)18	(AT)14	-	Introns/Intergenic	-	-	GCTCCAATTCCTTCTAGGGC	CGTGAATGAGAACAAGCGAA	258	P	A	A	NA

Polymorphic SSR (PMS) markers	Chromosomes	Start physical positions (bp)	End physical positions (bp)	Nipponbare	Kasalath	Nagina22	IR64	Pokkali	Bala	Tainung67	Azucena	Moroberekan	*Oryza rufipogon*	*Oryza nivara*	MSU gene locus IDs	Structural annotation	Functional annotation	SSR functional effects	Forward primers (5'-3')	Reverse primers (5'-3')	Amplified product size (bp)	Markers exhibiting polymorphism within *indica* rice	Transcrption factor genes-derived markers	Known cloned genes-derived markers	PMS markers corresponding with RM markers
PMS6032	Os_Chr12	15646706	15646723	(AT)9	(AT)9	(AT)8	(AT)9	(AT)9	(AT)9	(AT)9	(AT)9	(AT)9	(AT)9	(AT)9	-	Introns/Intergenic	-	-	CACAGATCCCAAACAACCCT	TGATTCACCCCACTAATGAGC	266	P	A	A	NA
PMS6033	Os_Chr12	15704032	15704049	(CGG)6	(CGG)5	(CGG)6	(CGG)6	(CGG)6	(CGG)6	(CGG)6	(CGG)6	(CGG)6	(CGG)6	(CGG)6	-	Introns/Intergenic	-	-	TTTTCTTCTTCCTCCTCCCG	ACACACACATCCCCCATCTT	258	P	A	A	NA
PMS6034	Os_Chr12	15858220	15858243	(CGG)8	(CGG)8	(CGG)8	(CGG)8	(CGG)8	(CGG)8	(CGG)5	(CGG)8	(CGG)8	(CGG)8	(CGG)8	LOC_Os12g27090	CDS	expressed protein	CODON_DELETION	GATCCCCGAAACCCTAACC	GAACACGGAGGGCAGGAG	265	A	A	A	NA
PMS6035	Os_Chr12	15876791	15876868	(AT)39	(AT)39	(AT)39	(AT)9	(AT)8	(AT)39	(AT)39	(AT)8	(AT)39	(AT)39	(AT)10	-	Introns/Intergenic	-	-	GAGCATTATGAATGTTTAAGAAG	ATTCTGCCAAGAAAAATATGC	218	P	A	A	NA
PMS6036	Os_Chr12	16065493	16065512	(TA)10	(TA)9	(TA)10	(TA)10	(TA)10	(TA)10	(TA)10	(TA)10	(TA)10	(TA)10	(TA)10	-	Introns/Intergenic	-	-	GGGAGCCAATTTGATAAGCA	GCGGGCTACCTTCCTAGTTT	228	P	A	A	NA
PMS6037	Os_Chr12	16277594	16277647	(TA)27	(TA)27	(TA)7	(TA)27	(TA)27	(TA)6	(TA)8	(TA)27	(TA)6	(TA)27	(TA)7	-	Introns/Intergenic	-	-	CGAATTCACCACTTTGCTCA	AACCAACTTGCCAACCAAAG	271	P	A	A	NA
PMS6038	Os_Chr12	16486447	16486470	(CGG)8	(CGG)8	(CGG)5	(CGG)6	(CGG)8	(CGG)8	(CGG)8	(CGG)8	(CGG)8	(CGG)8	(CGG)8	LOC_Os12g27960	CDS	expressed protein	CODON_DELETION	GCACCATGCATGTTTTTACG	GACGAGGTAGCTCTCCCGAT	280	P	A	A	NA
PMS6039	Os_Chr12	16490762	16490775	(AT)7	(AT)6	(AT)7	(AT)7	(AT)7	(AT)7	(AT)7	(AT)7	(AT)6	(AT)6	(AT)7	-	Introns/Intergenic	-	-	GTGTGGCAGCTTGATTTTCA	GGGGCAGACATGGAAAAATA	228	P	A	A	NA
PMS6040	Os_Chr12	16498642	16498659	(TCG)6	(TCG)5	(TCG)6	(TCG)6	(TCG)6	(TCG)6	(TCG)6	(TCG)6	(TCG)6	(TCG)6	(TCG)6	LOC_Os12g27980	CDS	transposon protein, putative, unclassified, expressed	CODON_DELETION	CCTTCGGCTCGTACTCGTAG	TTGTCGAGGAATCCAACTCC	189	P	P	A	NA
PMS6041	Os_Chr12	16498846	16498863	(TCG)6	(TCG)6	(TCG)6	(TCG)6	(TCG)6	(TCG)6	(TCG)6	(TCG)6	(TCG)6	(TCG)9	(TCG)6	LOC_Os12g27980	CDS	transposon protein, putative, unclassified, expressed	CODON_INSERTION	GGAGTTGGATTCCTCGACAA	ACCTCCTGTGGAGCGTCTT	272	A	P	A	NA
PMS6042	Os_Chr12	16565546	16565560	(GCA)5	(GCA)5	(GCA)5	(GCA)5	(GCA)5	(GCA)5	(GCA)5	(GCA)6	(GCA)5	(GCA)5	(GCA)5	LOC_Os12g28065	CDS	expressed protein	CODON_INSERTION	GGCTCACACATCCTCCTCTC	CGATGGTCAGCTCCTTCTTC	253	A	A	A	NA
PMS6043	Os_Chr12	16658379	16658398	(CT)10	(CT)9	(CT)10	(CT)10	(CT)10	(CT)10	(CT)10	(CT)10	(CT)10	(CT)10	(CT)10	-	Introns/Intergenic	-	-	TCCTTCCATTTTCATAGCCG	AGTAATGATCGCACGCACAC	197	P	A	A	NA
PMS6044	Os_Chr12	16706346	16706366	(GAG)7	(GAG)7	(GAG)7	(GAG)7	(GAG)7	(GAG)7	(GAG)7	(GAG)7	(GAG)7	(GAG)7	(GAG)6	LOC_Os12g28260	CDS	cyclic nucleotide-gated ion channel, putative, expressed	CODON_DELETION	CAGCAGACGTTCCGGTTCTACTCG	AGGACGGTGGTGGTGATCTGG	173	A	A	A	RM28130

Polymorphic SSR (PMS) markers	Chromosomes	Start physical positions (bp)	End physical positions (bp)	Nipponbare	Kasalath	Nagina22	IR64	Pokkali	Bala	Tainung67	Azucena	Moroberekan	*Oryza rufipogon*	*Oryza nivara*	MSU gene locus IDs	Structural annotation	Functional annotation	SSR functional effects	Forward primers (5'-3')	Reverse primers (5'-3')	Amplified product size (bp)	Markers exhibiting polymorphism within *indica* rice	Transcrption factor genes-derived markers	Known cloned genes-derived markers	PMS markers corresponding with RM markers
PMS6045	Os_Chr12	16756693	16756728	(CAT)12	(CAT)12	(CAT)12	(CAT)12	(CAT)12	(CAT)12	(CAT)12	(CAT)7	(CAT)12	(CAT)7	(CAT)12	-	Introns/Intergenic	-	-	ACTTTTGCGGGTTTTAGGCT	GGCCGACCGGATCTAATAAT	216	A	A	A	NA
PMS6046	Os_Chr12	16804431	16804451	(GTC)7	(GTC)7	(GTC)7	(GTC)7	(GTC)7	(GTC)7	(GTC)7	(GTC)7	(GTC)7	(GTC)7	(GTC)5	-	Introns/Intergenic	-	-	ATCGTCGTCTCCGATCGTT	GCGGCTCACTTGACGAAC	267	A	A	A	NA
PMS6047	Os_Chr12	16810206	16810226	(GTC)7	(GTC)7	(GTC)7	(GTC)7	(GTC)7	(GTC)7	(GTC)7	(GTC)7	(GTC)7	(GTC)7	(GTC)5	-	Introns/Intergenic	-	-	ATCGTCGTCTCCGATCGTT	GCGGCTCACTTGACGAAC	267	A	A	A	NA
PMS6048	Os_Chr12	16845180	16845200	(TTA)7	(TTA)5	(TTA)7	(TTA)7	(TTA)7	(TTA)7	(TTA)7	(TTA)7	(TTA)7	(TTA)7	(TTA)7	-	Introns/Intergenic	-	-	GCCGATGAACAGTGGTTTTT	GCGACCATATTTGGATCAAT	257	P	A	A	NA
PMS6049	Os_Chr12	16879724	16879739	(TA)8	(TA)6	(TA)6	(TA)6	(TA)6	(TA)6	(TA)6	(TA)6	(TA)6	(TA)6	(TA)6	-	Introns/Intergenic	-	-	TCATGCATGTGGCATTTACA	AAGTGGATAAATCGTAATAAGCAAA	251	A	A	A	NA
PMS6050	Os_Chr12	16901130	16901153	(ATCT)6	(ATCT)6	(ATCT)6	(ATCT)6	(ATCT)6	(ATCT)6	(ATCT)6	(ATCT)5	(ATCT)6	(ATCT)6	(ATCT)6	-	Introns/Intergenic	-	-	CTGGTCTAGTGGCCAGGTGT	GGGTCCACCGATTTAAGTGA	265	A	A	A	NA
PMS6051	Os_Chr12	16989380	16989393	(TC)7	(TC)9	(TC)7	(TC)7	(TC)7	(TC)7	(TC)7	(TC)7	(TC)7	(TC)7	(TC)7	-	Introns/Intergenic	-	-	AATTCTCGAAATACCGCACG	AATGCAAATTCCAGAAACCG	131	P	A	A	NA
PMS6052	Os_Chr12	16997118	16997201	(ATA)28	(ATA)28	(ATA)20	(ATA)28	(ATA)28	(ATA)5	(ATA)6	(ATA)28	(ATA)28	(ATA)28	(ATA)21	-	Introns/Intergenic	-	-	TTTAACGTACTCCCTTCCCTTGG	CCACTTCTGCCTTCCTACCTACG	565	P	A	A	RM28148
PMS6053	Os_Chr12	17059019	17059062	(TA)22	(TA)16	(TA)22	(TA)9	(TA)8	(TA)22	(TA)22	(TA)7	(TA)13	(TA)22	(TA)17	-	Introns/Intergenic	-	-	ATTATGACGCGTGGCACATA	TGATTTATGCATGTTGAGTACAAA	268	P	A	A	NA
PMS6054	Os_Chr12	17062630	17062643	(CT)7	(CT)7	(CT)7	(CT)7	(CT)7	(CT)6	(CT)7	(CT)7	(CT)7	(CT)7	(CT)6	-	Introns/Intergenic	-	-	CAACGTGGCAATCCATACAG	CGAGTTCGCTCTACCTCCAC	196	P	A	A	NA
PMS6055	Os_Chr12	17149347	17149370	(TGC)8	(TGC)7	(TGC)8	(TGC)8	(TGC)8	(TGC)8	(TGC)8	(TGC)8	(TGC)8	(TGC)8	(TGC)8	LOC_Os12g28990	CDS	retrotransposon protein, putative, Ty3-gypsy subclass, expressed	CODON_DELETION	GCACGAGTCCCTTGTTGATT	TCCGCTACACCAATCCCTAC	142	P	A	A	NA
PMS6056	Os_Chr12	17301143	17301182	(AT)20	(AT)20	(AT)20	(AT)20	(AT)20	(AT)15	(AT)20	(AT)6	(AT)20	(AT)20	(AT)20	-	Introns/Intergenic	-	-	AACCGCTGTCTAAACTGACTTGG	CATGTACGTACCTCCACACATAGTGC	678	P	A	A	RM28154
PMS6057	Os_Chr12	17396462	17396569	(TTA)36	(TTA)35	(TTA)36	(TTA)12	(TTA)32	(TTA)36	(TTA)36	(TTA)36	(TTA)13	(TTA)35	(TTA)6	-	Introns/Intergenic	-	-	GCTTAATTTCTGACAGACCAGTGC	GATCTAAACACAGCCTTCCTTGG	510	P	A	A	RM28157

**Polymorphic SSR (PMS) markers**	**Chromosomes**	**Start physical positions (bp)**	**End physical positions (bp)**	**Nipponbare**	**Kasalath**	**Nagina22**	**IR64**	**Pokkali**	**Bala**	**Tainung67**	**Azucena**	**Moroberekan**	***Oryza rufipogon***	***Oryza nivara***	**MSU gene locus IDs**	**Structural annotation**	**Functional annotation**	**SSR functional effects**	**Forward primers (5'-3')**	**Reverse primers (5'-3')**	**Amplified product size (bp)**	**Markers exhibiting polymorphism within *indica* rice**	**Transcrption factor genes-derived markers**	**Known cloned genes-derived markers**	**PMS markers corresponding with RM markers**
PMS6058	Os_Chr12	17446100	17446113	(AG)7	(AG)7	(AG)7	(AG)8	(AG)8	(AG)8	(AG)7	(AG)8	(AG)7	(AG)7	(AG)8	-	Introns/Intergenic	-	-	CATCACGGACAACAACTTGG	TCTCAGGATCACGTCATTGC	177	P	A	A	NA
PMS6059	Os_Chr12	17543720	17543735	(TA)8	(TA)8	(TA)8	(TA)8	(TA)8	(TA)8	(TA)8	(TA)6	(TA)8	(TA)8	(TA)6	-	Introns/Intergenic	-	-	GAAGGTGATTGGCTGCTTTC	GGATTTATCCCGTCCGATTT	235	A	A	A	NA
PMS6060	Os_Chr12	17565924	17565941	(CGC)6	(CGC)6	(CGC)5	(CGC)6	(CGC)6	(CGC)6	(CGC)6	(CGC)6	(CGC)6	(CGC)6	(CGC)6	-	Introns/Intergenic	-	-	CACGGGTAGATTGGGGTAGA	GGTTATACAAGGGCTTGCCA	240	P	A	A	NA
PMS6061	Os_Chr12	17596178	17596191	(AC)7	(AC)7	(AC)7	(AC)7	(AC)7	(AC)7	(AC)7	(AC)6	(AC)7	(AC)6	(AC)7	-	Introns/Intergenic	-	-	AAAGCACACCAGAGTGACCC	CATGTCAAATGCTGCTCCTG	136	A	A	A	NA
PMS6062	Os_Chr12	17604607	17604618	(AT)6	(AT)6	(AT)6	(AT)6	(AT)6	(AT)8	(AT)6	(AT)6	(AT)6	(AT)6	(AT)6	-	Introns/Intergenic	-	-	TGAATGCATGCGTACTTTGG	AGGTTCAGTATGCCACCACC	223	P	A	A	NA
PMS6063	Os_Chr12	17716011	17716026	(GA)8	(GA)8	(GA)8	(GA)8	(GA)8	(GA)8	(GA)8	(GA)8	(GA)8	(GA)8	(GA)6	-	Introns/Intergenic	-	-	CTACTTCACCTTTGCCTCCG	TTATGGGACCCACCTGTCAT	210	A	A	A	NA
PMS6064	Os_Chr12	17744060	17744077	(GA)9	(GA)9	(GA)9	(GA)9	(GA)9	(GA)9	(GA)9	(GA)9	(GA)7	(GA)9	(GA)8	-	Introns/Intergenic	-	-	AGTTCTGCTACGCCGTCTGT	TTATGGGACCCACCTGTCAT	240	A	A	A	NA
PMS6065	Os_Chr12	17766582	17766599	(GA)9	(GA)9	(GA)9	(GA)9	(GA)9	(GA)9	(GA)9	(GA)9	(GA)7	(GA)9	(GA)8	-	Introns/Intergenic	-	-	AGTTCTGCTACGCCGTCTGT	TTATGGGACCCACCTGTCAT	240	A	A	A	NA
PMS6066	Os_Chr12	17779603	17779641	(TCC)13	(TCC)7	(TCC)12	(TCC)13	(TCC)13	(TCC)7	(TCC)13	(TCC)7	(TCC)7	(TCC)13	(TCC)13	LOC_Os12g29740	CDS	expressed protein	CODON_DELETION	GGTTCGAGGTGATCACTGCTTCC	GTGGTCACCGAGGTGGTTGAGG	170	P	A	A	RM28179
PMS6067	Os_Chr12	17911273	17911290	(CGG)6	(CGG)6	(CGG)6	(CGG)6	(CGG)5	(CGG)6	(CGG)6	(CGG)6	(CGG)6	(CGG)6	(CGG)6	-	Introns/Intergenic	-	-	GTTTCCGGCGAATACGAATA	ACACTAGGGGGATTGAAGGC	278	P	A	A	NA
PMS6068	Os_Chr12	17936433	17936450	(AGC)6	(AGC)6	(AGC)6	(AGC)6	(AGC)5	(AGC)6	(AGC)6	(AGC)6	(AGC)6	(AGC)6	(AGC)6	-	Introns/Intergenic	-	-	CAACCACCTAGCCTCCTCTG	GACAGGATGTGAGGCACCTT	186	P	A	A	NA
PMS6069	Os_Chr12	18012784	18012801	(CTG)6	(CTG)6	(CTG)6	(CTG)6	(CTG)6	(CTG)5	(CTG)6	(CTG)6	(CTG)6	(CTG)5	(CTG)6	-	Introns/Intergenic	-	-	GGAGAGAGGGAAGGAGGAGA	CTGAGACCGCACAACAAAAA	226	P	A	A	NA
PMS6070	Os_Chr12	18102768	18102785	(AG)9	(AG)7	(AG)9	(AG)9	(AG)9	(AG)9	(AG)9	(AG)9	(AG)9	(AG)9	(AG)9	-	Introns/Intergenic	-	-	AACAAGGAAGATAGCCCACG	GCTGACCGGTGGTCTCTATC	176	P	A	A	NA

Polymorphic SSR (PMS) markers	Chromosomes	Start physical positions (bp)	End physical positions (bp)	Nipponbare	Kasalath	Nagina22	IR64	Pokkali	Bala	Tainung67	Azucena	Moroberekan	*Oryza rufipogon*	*Oryza nivara*	MSU gene locus IDs	Structural annotation	Functional annotation	SSR functional effects	Forward primers (5'-3')	Reverse primers (5'-3')	Amplified product size (bp)	Markers exhibiting polymorphism within *indica* rice	Transcrption factor genes-derived markers	Known cloned genes-derived markers	PMS markers corresponding with RM markers
PMS6071	Os_Chr12	18111232	18111253	(TC)11	(TC)11	(TC)8	(TC)11	(TC)11	(TC)9	(TC)11	(TC)11	(TC)12	(TC)11	(TC)11	LOC_Os12g30170	URR	expressed protein	-	ACTCGGTAGTACTC CCCAAGTG	GCAGAAGAGAAGTT GTGTGTCG	282	P	A	A	RM28195
PMS6072	Os_Chr12	18204158	18204171	(TC)7	(TC)7	(TC)7	(TC)6	(TC)7	(TC)7	(TC)7	(TC)7	(TC)7	(TC)7	(TC)7	-	Introns/Intergenic	-	-	TTTAGAGGAAAAAC CGGCCT	GTAAAGGGAAATTGG CGAGA	202	P	A	A	NA
PMS6073	Os_Chr12	18254304	18254315	(TA)6	(TA)8	(TA)6	(TA)8	(TA)8	(TA)8	(TA)8	(TA)6	(TA)6	(TA)8	(TA)8	-	Introns/Intergenic	-	-	TCATGCATGTGGCA TTTACA	AAGTGGATAAATCGT AATAAGCAAA	247	P	A	A	NA
PMS6074	Os_Chr12	18335350	18335376	(GTT)9	(GTT)9	(GTT)9	(GTT)9	(GTT)9	(GTT)9	(GTT)5	(GTT)5	(GTT)5	(GTT)9	(GTT)9	-	Introns/Intergenic	-	-	TTTGCTGATCTAGT ACGTGTGTGG	GTAGTGCTTCTTCCC GTTGATCC	177	A	A	A	RM28207
PMS6075	Os_Chr12	18356678	18356693	(CT)8	(CT)8	(CT)8	(CT)8	(CT)6	(CT)8	(CT)8	(CT)8	(CT)8	(CT)8	(CT)8	LOC_Os12g30560	CDS	expressed protein	FRAME_SHIFT	CCTTTCTTCATCCCT CTCCC	CACCGTTGTCCACCA TCTT	205	P	A	A	NA
PMS6076	Os_Chr12	18383713	18383733	(CGC)7	(CGC)7	(CGC)7	(CGC)7	(CGC)7	(CGC)7	(CGC)7	(CGC)7	(CGC)7	(CGC)7	(CGC)6	LOC_Os12g30610	URR	expressed protein	-	TTTGAGAACACGAA AACCCC	ATGGAGTGGAGGAG AGGATG	152	A	A	A	NA
PMS6077	Os_Chr12	18578389	18578406	(CCG)6	(CCG)6	(CCG)6	(CCG)6	(CCG)6	(CCG)6	(CCG)5	(CCG)6	(CCG)6	(CCG)6	(CCG)6	LOC_Os12g30920	CDS	OsFBA4 - F-box and FBA domain containing protein, expressed	CODON_DELETION	GAGATAACGCTGGT CAACCC	AGCTGAGCCTCTCTG CCTC	118	A	A	A	NA
PMS6078	Os_Chr12	18624332	18624353	(AG)11	(AG)11	(AG)11	(AG)11	(AG)11	(AG)11	(AG)11	(AG)11	(AG)11	(AG)11	(AG)7	-	Introns/Intergenic	-	-	GGGAGGGAGCAGA GGATAAG	CCAGCAAAAATCAGG CCTAC	151	A	A	A	NA
PMS6079	Os_Chr12	18764999	18765022	(ATCT)6	(ATCT)6	(ATCT)6	(ATCT)5	(ATCT)5	(ATCT)6	(ATCT)6	(ATCT)6	(ATCT)6	(ATCT)6	(ATCT)6	-	Introns/Intergenic	-	-	CACCGTTGATTACG CAGATG	CGTGGAGGCTCCTTT TTGTA	253	P	A	A	NA
PMS6080	Os_Chr12	18904013	18904033	(GCA)7	(GCA)7	(GCA)7	(GCA)7	(GCA)7	(GCA)7	(GCA)5	(GCA)7	(GCA)7	(GCA)6	(GCA)7	LOC_Os12g31430	CDS	helix-loop-helix DNA-binding domain containing protein, expressed	CODON_DELETION	GCTGGTGACTCGCT TCGT	GGTTGTGGTGGTGG TGGT	188	A	P	A	NA
PMS6081	Os_Chr12	18922042	18922065	(CCG)8	(CCG)8	(CCG)8	(CCG)8	(CCG)8	(CCG)6	(CCG)8	(CCG)6	(CCG)6	(CCG)8	(CCG)6	-	Introns/Intergenic	-	-	CTCCTCTGCCTCCT CCTCTC	TAATCCCTGTAACCC GAGCA	192	P	A	A	NA
PMS6082	Os_Chr12	18943558	18943572	(GCG)5	(GCG)5	(GCG)5	(GCG)5	(GCG)5	(GCG)5	(GCG)5	(GCG)5	(GCG)5	(GCG)7	(GCG)5	LOC_Os12g31490	CDS	expressed protein	CODON_INSERTION	ATAGGCAACAGGTG GGTGAG	GCGCGAGAGAGAGA GATCC	251	A	A	A	NA
PMS6083	Os_Chr12	18972583	18972604	(TA)11	(TA)8	(TA)11	(TA)11	(TA)11	(TA)11	(TA)11	(TA)11	(TA)11	(TA)8	(TA)11	-	Introns/Intergenic	-	-	TGGATCTCAGCTTG TTGCTG	AAATTTCAACAATGG TGCCC	140	P	A	A	NA

Polymorphic SSR (PMS) markers	Chromosomes	Start physical positions (bp)	End physical positions (bp)	Nipponbare	Kasalath	Nagina22	IR64	Pokkali	Bala	Tainung67	Azucena	Moroberekan	*Oryza rufipogon*	*Oryza nivara*	MSU gene locus IDs	Structural annotation	Functional annotation	SSR functional effects	Forward primers (5'-3')	Reverse primers (5'-3')	Amplified product size (bp)	Markers exhibiting polymorphism within *indica* rice	Transcrption factor genes-derived markers	Known cloned genes-derived markers	PMS markers corresponding with RM markers
PMS6084	Os_Chr12	18972999	18973044	(TA)23	(TA)23	(TA)23	(TA)23	(TA)23	(TA)10	(TA)16	(TA)23	(TA)23	(TA)17	(TA)23	-	Introns/Intergenic	-	-	ATGGTGCCCCATATGAGTGT	GTGTTTTACCCCGACCCTCT	146	P	A	A	NA
PMS6085	Os_Chr12	19102775	19102802	(TTAT)7	(TTAT)7	(TTAT)7	(TTAT)7	(TTAT)7	(TTAT)7	(TTAT)7	(TTAT)7	(TTAT)7	(TTAT)7	(TTAT)5	-	Introns/Intergenic	-	-	TGTTGGGCCTCTTTAGAGTTTGC	CCAACCTTCTATCCCAAACACACC	280	A	A	A	RM28255
PMS6086	Os_Chr12	19114951	19114974	(AG)12	(AG)11	(AG)12	(AG)12	(AG)12	(AG)12	(AG)12	(AG)12	(AG)12	(AG)12	(AG)12	-	Introns/Intergenic	-	-	CATCCGGAGGAAGTTTGAAAGAAGG	CAAGGGCAACCTCTTCCACTACGC	385	P	A	A	RM5341
PMS6087	Os_Chr12	19115285	19115314	(TC)15	(TC)15	(TC)15	(TC)15	(TC)15	(TC)7	(TC)15	(TC)15	(TC)15	(TC)15	(TC)15	-	Introns/Intergenic	-	-	GGCTCACCTCGTTCTCGATCC	CATAAATAAATAGGGCGCCACACC	185	P	A	A	RM1246
PMS6088	Os_Chr12	19167185	19167214	(AT)15	(AT)15	(AT)15	(AT)15	(AT)15	(AT)15	(AT)15	(AT)13	(AT)7	(AT)15	(AT)15	-	Introns/Intergenic	-	-	AGGGACAATCCAAACTGATAGAGG	CGCTAGAAATTAGTCACGGTCTCG	333	A	A	A	RM2972
PMS6089	Os_Chr12	19185798	19185811	(GT)7	(GT)7	(GT)7	(GT)7	(GT)7	(GT)7	(GT)7	(GT)8	(GT)8	(GT)7	(GT)7	LOC_Os12g31860	URR	ureide permease, putative, expressed	-	TGCTAATCTTGTTGGCCCTT	CCCAATCTCCATTCTCCTCA	221	A	A	A	NA
PMS6090	Os_Chr12	19193115	19193192	(TA)39	(TA)39	(TA)39	(TA)39	(TA)39	(TA)39	(TA)39	(TA)26	(TA)6	(TA)24	(TA)39	-	Introns/Intergenic	-	-	TGAGAAGCACTCCAAACACG	ATAATTCGCCTCTGCTCTCG	231	A	A	A	NA
PMS6091	Os_Chr12	19221559	19221578	(GA)10	(GA)10	(GA)10	(GA)10	(GA)10	(GA)10	(GA)10	(GA)10	(GA)10	(GA)10	(GA)8	-	Introns/Intergenic	-	-	CCTCCTCCACTTGCTCGG	CGGTTTCTCTCCACCTTCCT	116	A	A	A	NA
PMS6092	Os_Chr12	19332955	19332966	(AT)6	(AT)8	(AT)6	(AT)6	(AT)6	(AT)6	(AT)6	(AT)6	(AT)6	(AT)6	(AT)6	-	Introns/Intergenic	-	-	CTGGCTTCACCATTAAACCC	CCCTGTTTCCACCACCTCTA	241	P	A	A	NA
PMS6093	Os_Chr12	19339672	19339685	(TA)7	(TA)7	(TA)7	(TA)7	(TA)7	(TA)9	(TA)7	(TA)7	(TA)7	(TA)7	(TA)7	-	Introns/Intergenic	-	-	CCGATCGATTCAACAGGAGT	AAATCAAATTCCCCACGACA	255	P	A	A	NA
PMS6094	Os_Chr12	19376672	19376689	(GGC)6	(GGC)6	(GGC)6	(GGC)6	(GGC)6	(GGC)6	(GGC)6	(GGC)5	(GGC)6	(GGC)6	(GGC)6	-	Introns/Intergenic	-	-	GGAAGGTCAGTGAGGAACGA	CTCAATCTCTCAGGTCGATGC	225	A	A	A	NA
PMS6095	Os_Chr12	19455051	19455112	(TC)31	(TC)31	(TC)31	(TC)31	(TC)31	(TC)31	(TC)31	(TC)31	(TC)32	(TC)31	(TC)31	-	Introns/Intergenic	-	-	TGCACCACCTATTTCAATCCACTCC	CCTTCCTCAAGGGAAATCACAGAAGC	256	A	A	A	RM28277
PMS6096	Os_Chr12	19473042	19473068	(TTA)9	(TTA)9	(TTA)9	(TTA)9	(TTA)9	(TTA)9	(TTA)9	(TTA)5	(TTA)9	(TTA)9	(TTA)9	-	Introns/Intergenic	-	-	AACGACGGGTAAGCTAGGGT	AAAATTGCATCGTGGGTTTT	220	A	A	A	NA

Polymorphic SSR (PMS) markers	Chromosomes	Start physical positions (bp)	End physical positions (bp)	Nipponbare	Kasalath	Nagina22	IR64	Pokkali	Bala	Tainung67	Azucena	Moroberekan	*Oryza rufipogon*	*Oryza nivara*	MSU gene locus IDs	Structural annotation	Functional annotation	SSR functional effects	Forward primers (5'-3')	Reverse primers (5'-3')	Amplified product size (bp)	Markers exhibiting polymorphism within *indica* rice	Transcrption factor genes-derived markers	Known cloned genes-derived markers	PMS markers corresponding with RM markers
PMS6097	Os_Chr12	19578279	19578304	(TA)13	(TA)13	(TA)10	(TA)13	(TA)13	(TA)13	(TA)13	(TA)13	(TA)13	(TA)13	(TA)13	-	Introns/Intergenic	-	-	CTGTACCAGAAACAGGATCAAACC	ATGTGTTAACTGGAGACGAACG	309	P	A	A	RM28286
PMS6098	Os_Chr12	19581271	19581300	(TA)15	(TA)15	(TA)15	(TA)8	(TA)15	(TA)15	(TA)15	(TA)15	(TA)15	(TA)15	(TA)15	-	Introns/Intergenic	-	-	ATTTATTTTGGGACGGAGGG	CGGAGAAGACACGAAAGGAG	178	P	A	A	NA
PMS6099	Os_Chr12	19587217	19587237	(GGC)7	(GGC)7	(GGC)7	(GGC)7	(GGC)7	(GGC)7	(GGC)7	(GGC)6	(GGC)7	(GGC)7	(GGC)7	-	Introns/Intergenic	-	-	CTCTTCCCTACCTCTCCCAGATCC	ACCGGCGAACTCCTTTACATGC	142	A	A	A	RM28290
PMS6100	Os_Chr12	19628018	19628056	(TTC)13	(TTC)13	(TTC)13	(TTC)10	(TTC)13	(TTC)13	(TTC)13	(TTC)13	(TTC)13	(TTC)9	(TTC)13	-	Introns/Intergenic	-	-	ATCAACTCCGATCCTCTCCTCACC	ACCCGCCATGAACTCACTCG	175	P	A	A	RM28291
PMS6101	Os_Chr12	19735961	19735976	(GA)8	(GA)8	(GA)8	(GA)8	(GA)7	(GA)8	(GA)8	(GA)8	(GA)8	(GA)8	(GA)8	-	Introns/Intergenic	-	-	GCAATGAGCCGTTCTTCTTC	GCTAGCACCCTAGCAACCTG	187	P	A	A	NA
PMS6102	Os_Chr12	19773461	19773474	(AT)7	(AT)7	(AT)7	(AT)7	(AT)7	(AT)7	(AT)6	(AT)6	(AT)7	(AT)7	(AT)7	-	Introns/Intergenic	-	-	AAGTGGATAAATCGTAATAAGCAAA	TCATGCATGTGGCATTTACA	249	A	A	A	NA
PMS6103	Os_Chr12	19894394	19894407	(CT)7	(CT)6	(CT)7	(CT)7	(CT)7	(CT)7	(CT)7	(CT)7	(CT)7	(CT)7	(CT)7	-	Introns/Intergenic	-	-	GCCCCATGTGTCAGTCTCTT	CTTCGATGGTGGCTACGTC	180	P	A	A	NA
PMS6104	Os_Chr12	19917845	19917868	(CGG)8	(CGG)8	(CGG)8	(CGG)8	(CGG)8	(CGG)8	(CGG)5	(CGG)8	(CGG)8	(CGG)8	(CGG)8	LOC_Os12g32980	URR	potyvirus VPg interacting protein, putative, expressed	-	TTGGCAGAAGAAGCTCACCT	CCACTCTCCCCCACTTGATA	246	A	A	A	NA
PMS6105	Os_Chr12	19927668	19927682	(CGG)5	(CGG)5	(CGG)5	(CGG)5	(CGG)5	(CGG)5	(CGG)5	(CGG)5	(CGG)5	(CGG)7	(CGG)5	LOC_Os12g32986	URR	heat shock protein, putative, expressed	-	CTCCTCCGCTACTCCTACCA	AGTGACCCGTAGTAACCCCC	215	A	A	A	NA
PMS6106	Os_Chr12	19948119	19948156	(GA)19	(GA)19	(GA)19	(GA)19	(GA)17	(GA)19	(GA)19	(GA)19	(GA)19	(GA)19	(GA)16	LOC_Os12g33020	URR	mitochondrial import inner membrane translocase subunit Tim17, putative, expressed	-	TATTTACCACTCCCTGCCACTGC	AGGGAAGGGTACAAGGGTGTCG	455	P	A	A	RM3448
PMS6107	Os_Chr12	20051758	20051795	(AT)19	(AT)19	(AT)19	(AT)19	(AT)19	(AT)6	(AT)19	(AT)19	(AT)19	(AT)19	(AT)19	-	Introns/Intergenic	-	-	CACTATAGCTAATCACGCACACC	AGAGAATTTGTCCTGGATAGGG	142	P	A	A	RM28313
PMS6108	Os_Chr12	20055695	20055718	(TC)12	(TC)12	(TC)12	(TC)12	(TC)12	(TC)12	(TC)12	(TC)12	(TC)10	(TC)12	(TC)12	-	Introns/Intergenic	-	-	GCGGCGTCGTCATCTCTT	CCATGCACAGAGATAGAGAAA	229	A	A	A	NA
PMS6109	Os_Chr12	20056246	20056265	(TA)10	(TA)10	(TA)10	(TA)10	(TA)10	(TA)6	(TA)10	(TA)10	(TA)10	(TA)10	(TA)10	-	Introns/Intergenic	-	-	CTTGTGACGACCGGAAAAAT	AGGTCGTGGGAGGCTTTAAT	178	P	A	A	NA

Polymorphic SSR (PMS) markers	Chromosomes	Start physical positions (bp)	End physical positions (bp)	Nipponbare	Kasalath	Nagina22	IR64	Pokkali	Bala	Tainung67	Azucena	Moroberekan	*Oryza rufipogon*	*Oryza nivara*	MSU gene locus IDs	Structural annotation	Functional annotation	SSR functional effects	Forward primers (5'-3')	Reverse primers (5'-3')	Amplified product size (bp)	Markers exhibiting polymorphism within *indica* rice	Transcrption factor genes-derived markers	Known cloned genes-derived markers	PMS markers corresponding with RM markers
PMS6110	Os_Chr12	20090881	20090896	(AT)8	(AT)8	(AT)8	(AT)8	(AT)8	(AT)8	(AT)8	(AT)6	(AT)8	(AT)8	(AT)8	-	Introns/Intergenic	-	-	ACTCCCTCCGTTTCACAATG	GACTTTCTAGCATTGCCCACA	134	A	A	A	NA
PMS6111	Os_Chr12	20200088	20200105	(AT)9	(AT)9	(AT)9	(AT)9	(AT)9	(AT)9	(AT)7	(AT)9	(AT)9	(AT)9	(AT)9	-	Introns/Intergenic	-	-	GGGTAGGACGTGAGGGGTAA	CGTATTTCATACCATGGTGTCTC	232	A	A	A	NA
PMS6112	Os_Chr12	20895840	20895855	(AT)8	(AT)6	(AT)8	(AT)8	(AT)8	(AT)8	(AT)8	(AT)8	(AT)8	(AT)8	(AT)8	-	Introns/Intergenic	-	-	TGAGTACACCAGTTTAATTGGAAA	GTATGTACGCCTCCCTCCAA	240	P	A	A	NA
PMS6113	Os_Chr12	20953131	20953206	(AT)38	(AT)38	(AT)38	(AT)38	(AT)38	(AT)9	(AT)38	(AT)38	(AT)32	(AT)31	(AT)7	-	Introns/Intergenic	-	-	AATCCCTCCGTTTCACAATG	GATGCCTTCCAAACCTACCA	225	P	A	A	NA
PMS6114	Os_Chr12	21025604	21025627	(AGAT)6	(AGAT)6	(AGAT)6	(AGAT)6	(AGAT)6	(AGAT)6	(AGAT)6	(AGAT)6	(AGAT)5	(AGAT)6	(AGAT)5	-	Introns/Intergenic	-	-	AGAGCGCCATGTGGTAACTT	CGCCTATATGGGTCAAAGGA	205	A	A	A	NA
PMS6115	Os_Chr12	21081882	21081905	(TA)12	(TA)12	(TA)7	(TA)12	(TA)12	(TA)12	(TA)6	(TA)11	(TA)9	(TA)9	(TA)8	-	Introns/Intergenic	-	-	CCGGTGCATGCAGATAATTT	AAGTGGAATGCAGAAGCAGC	199	P	A	A	NA
PMS6116	Os_Chr12	21122150	21122163	(TC)7	(TC)7	(TC)7	(TC)6	(TC)7	(TC)7	(TC)7	(TC)7	(TC)7	(TC)7	(TC)7	-	Introns/Intergenic	-	-	AGTCCTCTTTTGGCTTTGGG	GAGGTAGACGACGACCTGACA	152	P	A	A	NA
PMS6117	Os_Chr12	21253117	21253131	(AGC)5	(AGC)5	(AGC)5	(AGC)7	(AGC)5	(AGC)5	(AGC)5	(AGC)5	(AGC)5	(AGC)7	(AGC)5	-	Introns/Intergenic	-	-	AATACCAACAACGGGCACTC	GACTGAGTCGAGCGAGAACG	221	P	A	A	NA
PMS6118	Os_Chr12	21339765	21339782	(CCG)6	(CCG)6	(CCG)6	(CCG)6	(CCG)6	(CCG)6	(CCG)6	(CCG)6	(CCG)6	(CCG)6	(CCG)5	-	Introns/Intergenic	-	-	TTTAGGGTTTACGGCAGTCC	TCTTCTCTCCCCCTTCTTCG	250	A	A	A	NA
PMS6119	Os_Chr12	21439780	21439795	(AG)8	(AG)8	(AG)7	(AG)8	(AG)8	(AG)8	(AG)8	(AG)8	(AG)8	(AG)8	(AG)8	-	Introns/Intergenic	-	-	ACCGTGTCTCGTGTGTCTTG	ACTACCATCCTGGTGCCAAC	194	P	A	A	NA
PMS6120	Os_Chr12	21488137	21488164	(GT)14	(GT)12	(GT)12	(GT)14	(GT)14	(GT)14	(GT)14	(GT)14	(GT)14	(GT)11	(GT)12	LOC_Os12g35350	URR	kelch repeat protein, putative, expressed	-	CCTTTCTGGCTTTCAGCAAC	AAAGAATCAGCAACCTCCGA	180	P	A	A	NA
PMS6121	Os_Chr12	21625434	21625445	(AG)6	(AG)7	(AG)7	(AG)6	(AG)6	(AG)6	(AG)6	(AG)6	(AG)6	(AG)6	(AG)6	-	Introns/Intergenic	-	-	GCCTTTCAAAGCATGCACTA	TCGTAATATTTGGTGGCACG	274	P	A	A	NA
PMS6122	Os_Chr12	21629584	21629604	(GCG)7	(GCG)8	(GCG)7	(GCG)7	(GCG)7	(GCG)8	(GCG)7	(GCG)7	(GCG)7	(GCG)6	(GCG)7	LOC_Os12g35570	URR	tRNA synthetase, putative, expressed	-	AGCTTGTCGTAGTCGATCCC	GCCAGAGCACGAGGTTTATC	228	P	A	A	NA

Polymorphic SSR (PMS) markers	Chromosomes	Start physical positions (bp)	End physical positions (bp)	Nipponbare	Kasalath	Nagina22	IR64	Pokkali	Bala	Tainung67	Azucena	Moroberekan	*Oryza rufipogon*	*Oryza nivara*	MSU gene locus IDs	Structural annotation	Functional annotation	SSR functional effects	Forward primers (5'-3')	Reverse primers (5'-3')	Amplified product size (bp)	Markers exhibiting polymorphism within *indica* rice	Transcrption factor genes-derived markers	Known cloned genes-derived markers	PMS markers corresponding with RM markers
PMS6123	Os_Chr12	21648382	21648396	(TCT)5	(TCT)5	(TCT)5	(TCT)5	(TCT)5	(TCT)5	(TCT)5	(TCT)5	(TCT)5	(TCT)7	(TCT)5	-	Introns/Intergenic	-	-	TTCTTGTCCTCACCGAATCC	TCCAATTCCCAAATCTGAGC	191	A	A	A	NA
PMS6124	Os_Chr12	21648983	21649003	(GCG)7	(GCG)7	(GCG)7	(GCG)7	(GCG)7	(GCG)7	(GCG)7	(GCG)7	(GCG)7	(GCG)5	(GCG)7	LOC_Os12g35610	CDS	Osrboh9	CODON_CHANGE_PLUS_CODON_DELETION	ACGCGGTTCAAGGAGGACAACG	GGCGCTGGAGTTGAGGAACTGG	360	A	A	P	RM28390
PMS6125	Os_Chr12	21731480	21731519	(TA)20	(TA)20	(TA)10	(TA)20	(TA)20	(TA)10	(TA)20	(TA)10	(TA)7	(TA)20	(TA)20	-	Introns/Intergenic	-	-	GCTTGTTGTTGGGACTGGTT	GACTGCCATTTTGCTTGGTT	272	P	A	A	NA
PMS6126	Os_Chr12	21753683	21753697	(AAT)5	(AAT)5	(AAT)5	(AAT)5	(AAT)5	(AAT)6	(AAT)5	(AAT)5	(AAT)6	(AAT)5	(AAT)6	-	Introns/Intergenic	-	-	CGGGCATGTATTTTAGGGTG	AACAAGCAGTGATGACAAGGAA	235	P	A	A	NA
PMS6127	Os_Chr12	21757084	21757121	(TA)19	(TA)19	(TA)10	(TA)19	(TA)19	(TA)19	(TA)19	(TA)19	(TA)19	(TA)19	(TA)19	-	Introns/Intergenic	-	-	CACATGTGTGGTGCTGCATA	CGGAGGCCTCACCTAATGTA	144	P	A	A	NA
PMS6128	Os_Chr12	21869773	21869790	(GGC)6	(GGC)6	(GGC)6	(GGC)6	(GGC)6	(GGC)6	(GGC)6	(GGC)6	(GGC)6	(GGC)5	(GGC)6	LOC_Os12g35890	CDS	FAD dependent oxidoreductase domain containing protein, expressed	CODON_DELETION	CATCTCCCACACCAAAATCC	ACCTCTCCAGGAGCAGCAC	178	A	A	A	NA
PMS6129	Os_Chr12	21935013	21935026	(CT)7	(CT)7	(CT)7	(CT)7	(CT)6	(CT)7	(CT)7	(CT)7	(CT)6	(CT)7	(CT)7	-	Introns/Intergenic	-	-	CGGCTCCTTTCTCTCTTCCT	GTCACTCCTGTGCCACCGT	229	P	A	A	NA
PMS6130	Os_Chr12	22010549	22010563	(GGA)5	(GGA)6	(GGA)5	(GGA)5	(GGA)5	(GGA)5	(GGA)5	(GGA)5	(GGA)6	(GGA)5	(GGA)5	LOC_Os12g36000	CDS	expressed protein	CODON_CHANGE_PLUS_CODON_INSERTION	CCAGATGGAGATGGAATGCT	AGGATCGGGAGGAGGATGT	144	P	A	A	NA
PMS6131	Os_Chr12	22027863	22027906	(TA)22	(TA)22	(TA)22	(TA)22	(TA)22	(TA)22	(TA)6	(TA)22	(TA)22	(TA)6	(TA)6	-	Introns/Intergenic	-	-	GGCCTCACCTGATGTGTCTT	CTCCGAAATTTCCAACCTGA	159	A	A	A	NA
PMS6132	Os_Chr12	22053764	22053785	(TA)11	(TA)11	(TA)11	(TA)11	(TA)11	(TA)11	(TA)11	(TA)7	(TA)11	(TA)11	(TA)11	-	Introns/Intergenic	-	-	TATGGGTCATAACTGAGCCACTCC	CACGTACACCTACTTCTTGCTTGC	306	A	A	A	RM28409
PMS6133	Os_Chr12	22116318	22116365	(TA)24	(TA)24	(TA)24	(TA)24	(TA)24	(TA)24	(TA)15	(TA)24	(TA)24	(TA)24	(TA)13	-	Introns/Intergenic	-	-	GGGAAATGTGATCTCTCCCA	AATTTTGTGCATTTGAAACCA	220	A	A	A	NA
PMS6134	Os_Chr12	22177232	22177249	(GCA)6	(GCA)6	(GCA)6	(GCA)5	(GCA)6	(GCA)6	(GCA)5	(GCA)6	(GCA)6	(GCA)5	(GCA)6	LOC_Os12g36180	CDS	auxilin, putative, expressed	CODON_DELETION	CTCGAGCTCCCATACCAAAG	AGAGCTGCAGTGGAGAGAGC	208	P	A	A	NA
PMS6135	Os_Chr12	22427317	22427331	(GGC)5	(GGC)5	(GGC)5	(GGC)5	(GGC)5	(GGC)5	(GGC)5	(GGC)5	(GGC)5	(GGC)8	(GGC)5	-	Introns/Intergenic	-	-	GGCATGCGTAGAAGGTAGGA	AGAGGCATCTCCGAATCTCA	189	A	A	A	NA

Polymorphic SSR (PMS) markers	Chromosomes	Start physical positions (bp)	End physical positions (bp)	Nipponbare	Kasalath	Nagina22	IR64	Pokkali	Bala	Tainung67	Azucena	Moroberekan	*Oryza rufipogon*	*Oryza nivara*	MSU gene locus IDs	Structural annotation	Functional annotation	SSR functional effects	Forward primers (5'-3')	Reverse primers (5'-3')	Amplified product size (bp)	Markers exhibiting polymorphism within *indica* rice	Transcrption factor genes-derived markers	Known cloned genes-derived markers	PMS markers corresponding with RM markers
PMS6136	Os_Chr12	22534606	22534623	(CGG)6	(CGG)6	(CGG)6	(CGG)6	(CGG)6	(CGG)6	(CGG)5	(CGG)6	(CGG)7	(CGG)6	(CGG)6	-	Introns/Intergenic	-	-	TCTCGCAAGAACAGAGAGCA	CACCGTGCGGTACACCTAC	236	A	A	A	NA
PMS6137	Os_Chr12	22610174	22610194	(ATA)7	(ATA)7	(ATA)7	(ATA)5	(ATA)7	(ATA)6	(ATA)5	(ATA)7	(ATA)7	(ATA)6	(ATA)7	-	Introns/Intergenic	-	-	AGCGCATCTCATCGAATCTT	TGTCCAATTTAACTATGCTCTCCA	101	P	A	A	NA
PMS6138	Os_Chr12	22610617	22610652	(TA)18	(TA)12	(TA)18	(TA)10	(TA)10	(TA)14	(TA)18	(TA)18	(TA)18	(TA)18	(TA)18	-	Introns/Intergenic	-	-	TTTGGGCTACCTCATTGGAT	TCTCGTTAGGAGTGGTCGCT	215	P	A	A	NA
PMS6139	Os_Chr12	22636599	22636618	(TA)10	(TA)10	(TA)10	(TA)10	(TA)10	(TA)9	(TA)10	(TA)10	(TA)10	(TA)10	(TA)10	-	Introns/Intergenic	-	-	CAGATTGGCCGTATGGTGAGC	TTAGCACATGTTTGACCGTTCG	446	P	A	A	RM28445
PMS6140	Os_Chr12	22671182	22671199	(CCA)6	(CCA)6	(CCA)6	(CCA)6	(CCA)6	(CCA)6	(CCA)5	(CCA)6	(CCA)6	(CCA)6	(CCA)6	LOC_Os12g36980	CDS	expressed protein	CODON_DELETION	CTCCCCAACCCCATCACT	AACCTCGACGAGAAATCGAA	280	A	A	A	NA
PMS6141	Os_Chr12	22707180	22707191	(AG)6	(AG)6	(AG)6	(AG)6	(AG)6	(AG)7	(AG)6	(AG)6	(AG)6	(AG)6	(AG)7	-	Introns/Intergenic	-	-	ACGCATACACACAGGGACAA	GGAGCCATGCATTAGTCTGG	246	P	A	A	NA
PMS6142	Os_Chr12	22742487	22742552	(ATA)22	(ATA)22	(ATA)16	(ATA)22	(ATA)22	(ATA)22	(ATA)10	(ATA)12	(ATA)22	(ATA)22	(ATA)22	-	Introns/Intergenic	-	-	GGTGTCCACATCGTTATGTCA	GCCCAGAAGATCACGGATAG	233	P	A	A	NA
PMS6143	Os_Chr12	22785691	22785714	(CCG)8	(CCG)8	(CCG)8	(CCG)8	(CCG)8	(CCG)8	(CCG)8	(CCG)8	(CCG)8	(CCG)8	(CCG)5	LOC_Os12g37160	URR	expressed protein	-	CGTCTGGGAGTTCACCCTAA	CCCCCTACCTCTCTTCGTTC	157	A	A	A	NA
PMS6144	Os_Chr12	22839956	22839967	(TA)6	(TA)8	(TA)6	(TA)8	(TA)8	(TA)8	(TA)8	(TA)6	(TA)6	(TA)8	(TA)8	-	Introns/Intergenic	-	-	TTAGCTAGCCACTACCAAGAGAGG	GTCCTTTCGACAGTACATCTGG	128	P	A	A	RM28457
PMS6145	Os_Chr12	22897955	22897968	(CT)7	(CT)7	(CT)7	(CT)6	(CT)7	(CT)7	(CT)7	(CT)7	(CT)7	(CT)7	(CT)7	-	Introns/Intergenic	-	-	TTCCAAATCAAATTCCTCCG	GAGCGGAGGAGGAGGGAG	261	P	A	A	NA
PMS6146	Os_Chr12	22904540	22904559	(CT)10	(CT)10	(CT)8	(CT)10	(CT)7	(CT)10	(CT)10	(CT)10	(CT)10	(CT)7	(CT)10	-	Introns/Intergenic	-	-	ACACACCGAGTGAAGCAGAG	CTCCGAAATCTCACTCAGGC	141	P	A	A	NA
PMS6147	Os_Chr12	22942639	22942971	(ATT)111	(ATT)111	(ATT)12	(ATT)8	(ATT)8	(ATT)10	(ATT)111	(ATT)111	(ATT)5	(ATT)8	(ATT)31	-	Introns/Intergenic	-	-	AGGTACAAACTAAGCCTCCAT	TGTGAATTTTCCATGTATCATC	427	P	A	A	NA
PMS6148	Os_Chr12	22946205	22946220	(AC)8	(AC)8	(AC)8	(AC)9	(AC)8	(AC)8	(AC)8	(AC)8	(AC)8	(AC)8	(AC)8	-	Introns/Intergenic	-	-	TACTGTAGCAGGGAATGGGC	GGCTGCTTGTTTTAGCTTCG	271	P	A	A	NA

Polymorphic SSR (PMS) markers	Chromosomes	Start physical positions (bp)	End physical positions (bp)	Nipponbare	Kasalath	Nagina22	IR64	Pokkali	Bala	Tainung67	Azucena	Moroberekan	*Oryza rufipogon*	*Oryza nivara*	MSU gene locus IDs	Structural annotation	Functional annotation	SSR functional effects	Forward primers (5'-3')	Reverse primers (5'-3')	Amplified product size (bp)	Markers exhibiting polymorphism within *indica* rice	Transcrption factor genes-derived markers	Known cloned genes-derived markers	PMS markers corresponding with RM markers
PMS6149	Os_Chr12	22950992	22951009	(GCG)6	(GCG)5	(GCG)6	(GCG)6	(GCG)6	(GCG)6	(GCG)6	(GCG)6	(GCG)6	(GCG)6	(GCG)6	LOC_Os12g37380	CDS	RNA pseudouridine synthase, putative, expressed	CODON_DELETION	CGAGCGATAGGTCTCCTCCT	CTACCCCTCCTCCTCCACTC	118	P	A	A	NA
PMS6150	Os_Chr12	22975558	22975590	(GCT)11	(GCT)11	(GCT)11	(GCT)7	(GCT)11	(GCT)11	(GCT)11	(GCT)11	(GCT)11	(GCT)7	(GCT)7	LOC_Os12g37430	CDS	PAPA-1-like conserved region family protein, expressed	CODON_DELETION	CCGACGAAGAAGACGAGGAGTAGCC	AGGCCGGAGAGCAATCATGTCG	76	P	A	A	RM28466
PMS6151	Os_Chr12	22999697	22999716	(AT)10	(AT)10	(AT)7	(AT)10	(AT)10	(AT)10	(AT)10	(AT)10	(AT)10	(AT)10	(AT)10	-	Introns/Intergenic	-	-	AGGCCATGGAGAACGATTTA	TCCATCCTTCACACCTCACA	277	P	A	A	NA
PMS6152	Os_Chr12	23025369	23025384	(CT)8	(CT)8	(CT)8	(CT)6	(CT)6	(CT)8	(CT)8	(CT)8	(CT)8	(CT)6	(CT)8	LOC_Os12g37519	URR	retrotransposon protein, putative, unclassified, expressed	-	AAACCTCCCTCCTTTTTCCA	AGGTTTTGGGATGTAGGGCT	124	P	A	A	NA
PMS6153	Os_Chr12	23116233	23116247	(TCA)5	(TCA)5	(TCA)5	(TCA)6	(TCA)5	(TCA)5	(TCA)5	(TCA)5	(TCA)5	(TCA)6	(TCA)5	LOC_Os12g37650	DRR	DUF538 domain containing protein, putative, expressed	-	CCAAGGTCGATGAGGAGAAG	AGTGAGATCTCGTGCTGCTG	231	P	A	A	NA
PMS6154	Os_Chr12	23120233	23120244	(TA)6	(TA)6	(TA)7	(TA)6	(TA)7	(TA)6	(TA)6	(TA)6	(TA)6	(TA)6	(TA)6	-	Introns/Intergenic	-	-	TCGTGCAATCCTACGTGTGT	CAAATTCATGTGGATGCCAG	136	P	A	A	NA
PMS6155	Os_Chr12	23121425	23121442	(AT)9	(AT)9	(AT)9	(AT)9	(AT)9	(AT)9	(AT)9	(AT)9	(AT)9	(AT)9	(AT)6	-	Introns/Intergenic	-	-	GTGAACGGGATCAACAGCTT	AATTTTGTGCATTTGAAACCA	112	A	A	A	NA
PMS6156	Os_Chr12	23122399	23122416	(CCG)6	(CCG)6	(CCG)6	(CCG)6	(CCG)6	(CCG)6	(CCG)5	(CCG)6	(CCG)6	(CCG)6	(CCG)6	LOC_Os12g37660	CDS	pectinesterase, putative, expressed	CODON_DELETION	CACGTAGTGCTGCACGTTCT	ACCAGCAATCCTTCACCATC	131	A	A	A	NA
PMS6157	Os_Chr12	23140853	23140864	(AT)6	(AT)6	(AT)7	(AT)6	(AT)6	(AT)6	(AT)6	(AT)6	(AT)6	(AT)6	(AT)6	-	Introns/Intergenic	-	-	GCGAAGAAGTGTGCAACAAA	TGGATGTGGGTGAAGCAATA	139	P	A	A	NA
PMS6158	Os_Chr12	23143530	23143567	(AT)19	(AT)19	(AT)19	(AT)15	(AT)19	(AT)19	(AT)19	(AT)19	(AT)19	(AT)19	(AT)10	-	Introns/Intergenic	-	-	TACTGGAGCCACTGGTTATTAGC	CTTAATTTACCGCAGCAACG	532	P	A	A	RM28484
PMS6159	Os_Chr12	23264583	23264600	(GAG)6	(GAG)6	(GAG)6	(GAG)6	(GAG)9	(GAG)6	(GAG)6	(GAG)6	(GAG)6	(GAG)6	(GAG)6	-	Introns/Intergenic	-	-	GAGGCCTAGGGTTTCGTACA	ACCTCTTGTCCGTTGTGAGC	232	P	A	A	NA
PMS6160	Os_Chr12	23338287	23338304	(TCG)6	(TCG)6	(TCG)6	(TCG)5	(TCG)6	(TCG)6	(TCG)6	(TCG)6	(TCG)6	(TCG)6	(TCG)6	LOC_Os12g37980	CDS	protein kinase domain containing protein, expressed	CODON_DELETION	ATCATCATCTTGGTCCCAGC	GAGCTGAAGGAGTGCCTCG	220	P	A	A	NA
PMS6161	Os_Chr12	23394797	23394820	(CGG)8	(CGG)8	(CGG)8	(CGG)8	(CGG)8	(CGG)7	(CGG)8	(CGG)8	(CGG)8	(CGG)8	(CGG)8	LOC_Os12g38090	CDS	OsFBX456 - F-box domain containing protein, expressed	CODON_DELETION	GTCGGAGTTGGAGTTTCCAG	ACCTACAAGGTCGCTCGCTA	220	P	A	A	NA

Polymorphic SSR (PMS) markers	Chromosomes	Start physical positions (bp)	End physical positions (bp)	Nipponbare	Kasalath	Nagina22	IR64	Pokkali	Bala	Tainung67	Azucena	Moroberekan	*Oryza rufipogon*	*Oryza nivara*	MSU gene locus IDs	Structural annotation	Functional annotation	SSR functional effects	Forward primers (5'-3')	Reverse primers (5'-3')	Amplified product size (bp)	Markers exhibiting polymorphism within *indica* rice	Transcrption factor genes-derived markers	Known cloned genes-derived markers	PMS markers corresponding with RM markers
PMS6162	Os_Chr12	23427005	23427038	(TA)17	(TA)17	(TA)17	(TA)17	(TA)17	(TA)17	(TA)8	(TA)17	(TA)6	(TA)17	(TA)7	-	Introns/Intergenic	-	-	GGATGCAATCACACGAGCTA	ATTCGATCGACCACCTGTGT	234	A	A	A	NA
PMS6163	Os_Chr12	23458171	23458198	(AG)14	(AG)14	(AG)14	(AG)14	(AG)14	(AG)14	(AG)14	(AG)14	(AG)14	(AG)14	(AG)15	-	Introns/Intergenic	-	-	TCACATGTCACATCGTTCCA	GGCCCAGTGTTTGTTCTCTC	196	A	A	A	NA
PMS6164	Os_Chr12	23492506	23492520	(CGG)5	(CGG)5	(CGG)5	(CGG)5	(CGG)6	(CGG)5	(CGG)5	(CGG)5	(CGG)5	(CGG)5	(CGG)5	LOC_Os12g38250	CDS	expressed protein	CODON_INSERTION	AGATGGAGGATCACCCGTTC	CCGGCTATGAAGAAGACAGC	102	P	A	A	NA
PMS6165	Os_Chr12	23502152	23502167	(TG)8	(TG)8	(TG)8	(TG)8	(TG)6	(TG)8	(TG)8	(TG)8	(TG)8	(TG)8	(TG)6	LOC_Os12g38270	DRR	metallothionein, putative, expressed	-	ATCCCTGCAACTGCTAAAGG	TCAGGTGCTCCAATCACAAG	248	P	A	A	NA
PMS6166	Os_Chr12	23528491	23528505	(AAG)5	(AAG)5	(AAG)5	(AAG)5	(AAG)5	(AAG)5	(AAG)5	(AAG)5	(AAG)5	(AAG)6	(AAG)5	-	Introns/Intergenic	-	-	TCCAATTTTTCAAGCCCAAG	GGAATGGCCTCCCATTTTAT	243	A	A	A	NA
PMS6167	Os_Chr12	23664218	23664238	(GCG)7	(GCG)7	(GCG)7	(GCG)7	(GCG)7	(GCG)8	(GCG)7	(GCG)5	(GCG)7	(GCG)7	(GCG)7	LOC_Os12g38540	CDS	expressed protein	CODON_DELETION	CCTTCCTCGATTCCTCCTCT	CGTATTCCTCCTCCTCCTCC	250	P	A	A	NA
PMS6168	Os_Chr12	23682875	23682895	(TGC)7	(TGC)7	(TGC)7	(TGC)7	(TGC)7	(TGC)6	(TGC)7	(TGC)7	(TGC)7	(TGC)7	(TGC)7	-	Introns/Intergenic	-	-	TTCAGAGCATGTATGTGAGTGAGC	AAGCTCGGAAACAATCAAGAGG	145	P	A	A	RM28519
PMS6169	Os_Chr12	23767176	23767189	(CG)7	(CG)7	(CG)7	(CG)7	(CG)7	(CG)7	(CG)7	(CG)7	(CG)7	(CG)7	(CG)6	-	Introns/Intergenic	-	-	AGTCTCTTTTGGCTTTGGGC	AGGAGATCCCGGAGATTGTT	258	A	A	A	NA
PMS6170	Os_Chr12	23831925	23831945	(GGC)7	(GGC)6	(GGC)7	(GGC)7	(GGC)7	(GGC)7	(GGC)6	(GGC)7	(GGC)7	(GGC)7	(GGC)7	LOC_Os12g38790	CDS	targeting protein for Xklp2 containing protein, expressed	CODON_DELETION	GAGGAATCGGGAGAACGTC	GTCCTCGTCCTCTTCTTCCC	267	P	A	A	NA
PMS6171	Os_Chr12	24000127	24000156	(TA)15	(TA)15	(TA)13	(TA)11	(TA)15	(TA)15	(TA)15	(TA)12	(TA)9	(TA)12	(TA)15	-	Introns/Intergenic	-	-	AGAAAATCGCGCAAGAAAAA	TACACGTGCATGATCGAATG	280	P	A	A	NA
PMS6172	Os_Chr12	24018177	24018192	(TA)8	(TA)8	(TA)8	(TA)8	(TA)8	(TA)8	(TA)8	(TA)6	(TA)8	(TA)8	(TA)8	-	Introns/Intergenic	-	-	TCAATTTCAACAATGGTGCC	GCATACGTATACGGCGCAC	186	A	A	A	NA
PMS6173	Os_Chr12	24057504	24057517	(AT)7	(AT)9	(AT)9	(AT)7	(AT)7	(AT)7	(AT)7	(AT)7	(AT)7	(AT)9	(AT)7	-	Introns/Intergenic	-	-	TTGCAAGGTGTGGACAAAAG	AAAGATGGAGCCGATGTGAG	272	P	A	A	NA
PMS6174	Os_Chr12	24095027	24095100	(AT)37	(AT)37	(AT)6	(AT)15	(AT)37	(AT)32	(AT)37	(AT)37	(AT)37	(AT)15	(AT)37	-	Introns/Intergenic	-	-	CTGGCTTTTCCAGATTTCCA	TCAATTTCAACAATGGTGCC	262	P	A	A	NA

Polymorphic SSR (PMS) markers	Chromosomes	Start physical positions (bp)	End physical positions (bp)	Nipponbare	Kasalath	Nagina22	IR64	Pokkali	Bala	Tainung67	Azucena	Moroberekan	*Oryza rufipogon*	*Oryza nivara*	MSU gene locus IDs	Structural annotation	Functional annotation	SSR functional effects	Forward primers (5'-3')	Reverse primers (5'-3')	Amplified product size (bp)	Markers exhibiting polymorphism within *indica* rice	Transcrption factor genes-derived markers	Known cloned genes-derived markers	PMS markers corresponding with RM markers
PMS6175	Os_Chr12	24095495	24095508	(TA)7	(TA)7	(TA)7	(TA)7	(TA)7	(TA)7	(TA)7	(TA)7	(TA)7	(TA)9	(TA)7	-	Introns/Intergenic	-	-	ATGGTGCCCCATAT GAGTGT	CCCACCTGCAAAGTT TCATT	230	A	A	A	NA
PMS6176	Os_Chr12	24098335	24098366	(TA)16	(TA)16	(TA)16	(TA)16	(TA)16	(TA)16	(TA)16	(TA)15	(TA)16	(TA)16	(TA)16	-	Introns/Intergenic	-	-	TCAATTTCAACAATG GTGCC	ACACGAACAGATCCA GGTCC	197	A	A	A	NA
PMS6177	Os_Chr12	24110536	24110559	(CT)12	(CT)12	(CT)12	(CT)12	(CT)12	(CT)13	(CT)12	(CT)12	(CT)12	(CT)12	(CT)12	-	Introns/Intergenic	-	-	TTGGCTCTCGTGAA AGACCT	AATCTGAGGCTAAAA GGCCA	115	P	A	A	NA
PMS6178	Os_Chr12	24234017	24234034	(CGA)6	(CGA)6	(CGA)6	(CGA)6	(CGA)6	(CGA)6	(CGA)6	(CGA)5	(CGA)6	(CGA)5	(CGA)6	-	Introns/Intergenic	-	-	GAGCTGGGTGAGC GAGTC	AGTCCGCATCGTCGT TGT	217	A	A	A	NA
PMS6179	Os_Chr12	24379740	24379763	(GA)12	(GA)13	(GA)12	(GA)13	(GA)13	(GA)13	(GA)12	(GA)12	(GA)12	(GA)12	(GA)11	-	Introns/Intergenic	-	-	CACCTGATGCTCCC TTGAAT	CAGGGATAGTCGAA CTCCCA	260	P	A	A	NA
PMS6180	Os_Chr12	24485169	24485182	(TA)7	(TA)7	(TA)7	(TA)7	(TA)7	(TA)7	(TA)7	(TA)7	(TA)6	(TA)7	(TA)7	-	Introns/Intergenic	-	-	CCATGTGTCAGCCT CTGCTA	TGCACCAGTCTTTGA GACCA	190	A	A	A	NA
PMS6181	Os_Chr12	24595591	24595606	(TC)8	(TC)8	(TC)8	(TC)8	(TC)8	(TC)8	(TC)8	(TC)8	(TC)8	(TC)6	(TC)8	-	Introns/Intergenic	-	-	TCTTCGACGAGGAG GACG	AGGCCATGGACGAC ACTAAC	257	A	A	A	NA
PMS6182	Os_Chr12	24625146	24625157	(TA)6	(TA)7	(TA)7	(TA)7	(TA)7	(TA)7	(TA)6	(TA)6	(TA)6	(TA)7	(TA)6	-	Introns/Intergenic	-	-	TGGAGTGAAAACAA ATGGCA	GCACTGTATCAGCAA AACTCCA	242	A	A	A	NA
PMS6183	Os_Chr12	24716726	24716740	(GCA)5	(GCA)6	(GCA)6	(GCA)6	(GCA)6	(GCA)6	(GCA)5	(GCA)5	(GCA)5	(GCA)6	(GCA)6	-	Introns/Intergenic	-	-	GGTGAAGACGAGG ATGGCTA	ATCCTAGGTCGGGAT CGTCT	225	A	A	A	NA
PMS6184	Os_Chr12	24724495	24724512	(CTG)6	(CTG)6	(CTG)6	(CTG)6	(CTG)6	(CTG)6	(CTG)5	(CTG)6	(CTG)6	(CTG)6	(CTG)6	LOC_Os12g39990	CDS	dof zinc finger domain containing protein, putative, expressed	CODON_DELETION	CGCCGTGTTGTAGT TGTTGT	GCTTAGAGCATCAGC AATCG	252	A	A	A	NA
PMS6185	Os_Chr12	24793227	24793244	(TAA)6	(TAA)5	(TAA)6	(TAA)6	(TAA)6	(TAA)5	(TAA)6	(TAA)5	(TAA)6	(TAA)5	(TAA)5	LOC_Os12g40100	DRR	expressed protein	-	ATTTTTATCTCGCG CGTTTG	CCAAGGAGCTAACC CTCCTC	167	P	A	A	NA
PMS6186	Os_Chr12	24809559	24809574	(AG)8	(AG)6	(AG)6	(AG)8	(AG)8	(AG)7	(AG)8	(AG)8	(AG)8	(AG)8	(AG)6	-	Introns/Intergenic	-	-	GACTTCTACGCCTC GCCTC	CGTGGCAGTTTAGTC AGCAA	263	P	A	A	NA
PMS6187	Os_Chr12	24892741	24892802	(AT)31	(AT)31	(AT)31	(AT)10	(AT)10	(AT)31	(AT)31	(AT)31	(AT)9	(AT)31	(AT)31	-	Introns/Intergenic	-	-	GTGCCAAGAAGTTG CAGACA	ATTAGATACCATGGT GCCCG	182	P	A	A	NA

**Polymorphic SSR (PMS) markers**	**Chromosomes**	**Start physical positions (bp)**	**End physical positions (bp)**	**Nipponbare**	**Kasalath**	**Nagina22**	**IR64**	**Pokkali**	**Bala**	**Tainung67**	**Azucena**	**Moroberekan**	***Oryza rufipogon***	***Oryza nivara***	**MSU gene locus IDs**	**Structural annotation**	**Functional annotation**	**SSR functional effects**	**Forward primers (5'-3')**	**Reverse primers (5'-3')**	**Amplified product size (bp)**	**Markers exhibiting polymorphism within *indica* rice**	**Transcrption factor genes-derived markers**	**Known cloned genes-derived markers**	**PMS markers corresponding with RM markers**
PMS6188	Os_Chr12	25015101	25015121	(CCT)7	(CCT)5	(CCT)7	(CCT)7	(CCT)7	(CCT)7	(CCT)7	(CCT)7	(CCT)7	(CCT)7	(CCT)7	-	Introns/Intergenic	-	-	GCTTTGGTTTATCGGGTGAA	GAGCTCCATCATCGGGAATA	195	P	A	A	NA
PMS6189	Os_Chr12	25060107	25060136	(TA)15	(TA)15	(TA)15	(TA)15	(TA)15	(TA)15	(TA)10	(TA)15	(TA)15	(TA)15	(TA)12	-	Introns/Intergenic	-	-	TTGGAGCCCATACTCCATGT	AAAAGGCAGCGTATTGTCAGA	223	A	A	A	NA
PMS6190	Os_Chr12	25103369	25103395	(CCG)9	(CCG)9	(CCG)9	(CCG)9	(CCG)9	(CCG)9	(CCG)8	(CCG)9	(CCG)9	(CCG)9	(CCG)9	LOC_Os12g40570	CDS	WRKY94, expressed	CODON_DELETION	CTGCTGCTCTCCAAGAATCC	TGCTCAGGAGCATAGCTTCA	187	A	P	A	NA
PMS6191	Os_Chr12	25114843	25114858	(AT)8	(AT)8	(AT)8	(AT)8	(AT)8	(AT)8	(AT)8	(AT)8	(AT)7	(AT)8	(AT)8	-	Introns/Intergenic	-	-	TGGAAGACGAGTCTACAGTCCA	ACTAGTATGATGGCCCACGC	259	A	A	A	NA
PMS6192	Os_Chr12	25276858	25276887	(TC)15	(TC)14	(TC)15	(TC)15	(TC)15	(TC)15	(TC)15	(TC)15	(TC)15	(TC)15	(TC)15	LOC_Os12g40830	DRR	kinase, pfkB family, putative, expressed	-	CTTCCTGGCTTCAACTCTGG	CGGAACTGCCGTTTATTCAT	250	P	A	A	NA
PMS6193	Os_Chr12	25289271	25289288	(TA)9	(TA)10	(TA)9	(TA)6	(TA)9	(TA)9	(TA)9	(TA)6	(TA)9	(TA)9	(TA)9	-	Introns/Intergenic	-	-	ACTATGTTGCTTCGCATCCC	TGTAACCGACCAAGATTAATTCC	102	P	A	A	NA
PMS6194	Os_Chr12	25289919	25289930	(CT)6	(CT)6	(CT)6	(CT)6	(CT)6	(CT)6	(CT)6	(CT)6	(CT)6	(CT)7	(CT)6	-	Introns/Intergenic	-	-	ATCCTCCTCCTCTCGCATCT	ACGCCGAACTAAACACAACC	206	A	A	A	NA
PMS6195	Os_Chr12	25331228	25331241	(AT)7	(AT)7	(AT)7	(AT)6	(AT)7	(AT)7	(AT)7	(AT)7	(AT)7	(AT)7	(AT)7	-	Introns/Intergenic	-	-	CGATACCGGGATACACCATC	GCTGTGAGCCTGTGAATCTG	200	P	A	A	NA
PMS6196	Os_Chr12	25335917	25335934	(CTG)6	(CTG)6	(CTG)6	(CTG)6	(CTG)6	(CTG)6	(CTG)5	(CTG)6	(CTG)6	(CTG)6	(CTG)6	LOC_Os12g40920	CDS	bZIP transcription factor domain containing protein, expressed	CODON_DELETION	TCAGGTGTATCAGACGCAGC	CTTCTGCAGGTTCTCCAAGC	165	A	P	A	NA
PMS6197	Os_Chr12	25463864	25463885	(AG)11	(AG)13	(AG)13	(AG)11	(AG)11	(AG)11	(AG)11	(AG)10	(AG)10	(AG)11	(AG)11	-	Introns/Intergenic	-	-	ACAGTGACTTTGGCCGTGTT	CGCGTGTTGTATGGTTTCAC	169	P	A	A	NA
PMS6198	Os_Chr12	25476546	25476569	(TCT)8	(TCT)8	(TCT)8	(TCT)9	(TCT)8	(TCT)9	(TCT)8	(TCT)8	(TCT)8	(TCT)8	(TCT)8	-	Introns/Intergenic	-	-	GCCTTAGCTTCTCCCTGCTT	TGGGAAGCAGAAGAGTTTTTG	243	P	A	A	NA
PMS6199	Os_Chr12	25482314	25482331	(TC)9	(TC)9	(TC)9	(TC)9	(TC)9	(TC)9	(TC)9	(TC)9	(TC)9	(TC)7	(TC)9	LOC_Os12g41124	URR	expressed protein	-	TGACCTGTTCTGTCCCTTGA	TGCAATGCAAGCTAGGTGAC	245	A	A	A	NA
PMS6200	Os_Chr12	25488752	25488772	(GTC)7	(GTC)7	(GTC)7	(GTC)7	(GTC)7	(GTC)7	(GTC)7	(GTC)5	(GTC)7	(GTC)7	(GTC)7	LOC_Os12g41140	CDS	expressed protein	CODON_DELETION	TGCAGATGGTGAGGAAGTTG	ACAAACACAACGCCCAGAAG	138	A	A	A	NA

Polymorphic SSR (PMS) markers	Chromosomes	Start physical positions (bp)	End physical positions (bp)	Nipponbare	Kasalath	Nagina22	IR64	Pokkali	Bala	Tainung67	Azucena	Moroberekan	*Oryza rufipogon*	*Oryza nivara*	MSU gene locus IDs	Structural annotation	Functional annotation	SSR functional effects	Forward primers (5'-3')	Reverse primers (5'-3')	Amplified product size (bp)	Markers exhibiting polymorphism within *indica* rice	Transcrption factor genes-derived markers	Known cloned genes-derived markers	PMS markers corresponding with RM markers
PMS6201	Os_Chr12	25493323	25493348	(AG)13	(AG)13	(AG)11	(AG)13	(AG)13	(AG)13	(AG)13	(AG)13	(AG)11	(AG)13	(AG)13	-	Introns/Intergenic	-	-	TCACTCAGGGACAAGCTCAA	GTTTCGTCCCTTCGTTCTTG	117	P	A	A	NA
PMS6202	Os_Chr12	25590816	25590855	(TA)20	(TA)20	(TA)20	(TA)20	(TA)11	(TA)20	(TA)20	(TA)20	(TA)20	(TA)20	(TA)20	-	Introns/Intergenic	-	-	TTTACAGAGGTGGGAGGTGG	GCGGCAACAATTTTCTTTTG	238	P	A	A	NA
PMS6203	Os_Chr12	25630447	25630526	(TA)40	(TA)38	(TA)40	(TA)6	(TA)40	(TA)7	(TA)40	(TA)40	(TA)40	(TA)40	(TA)10	-	Introns/Intergenic	-	-	ACTTTGGCATCTGTGTCCCTTCC	GGGCTTACCCAAGTGCCTAGC	394	P	A	A	RM28673
PMS6204	Os_Chr12	25678473	25678487	(CGA)5	(CGA)5	(CGA)5	(CGA)5	(CGA)5	(CGA)5	(CGA)5	(CGA)5	(CGA)5	(CGA)5	(CGA)6	LOC_Os12g41450	CDS	F-box domain containing protein, expressed	CODON_CHANGE_PLUS_CODON_INSERTION	ACACCATCGAGCACGTCAG	ACGAGAGCTGGTACTTTGGC	261	A	A	A	NA
PMS6205	Os_Chr12	25697378	25697389	(CT)6	(CT)6	(CT)6	(CT)6	(CT)6	(CT)6	(CT)6	(CT)6	(CT)6	(CT)8	(CT)6	-	Introns/Intergenic	-	-	CAAAGACTCAAATGTGGCCC	CACTATCGATCCACCATCCC	233	A	A	A	NA
PMS6206	Os_Chr12	25770870	25770947	(TTA)26	(TTA)26	(TTA)14	(TTA)8	(TTA)7	(TTA)26	(TTA)7	(TTA)8	(TTA)8	(TTA)26	(TTA)26	-	Introns/Intergenic	-	-	TCCGAGAGGGTACGTGTCATGC	GGAGCTGCAGTGACTTGAGATACTCG	423	P	A	A	RM28682
PMS6207	Os_Chr12	25779770	25779787	(GAC)6	(GAC)5	(GAC)6	(GAC)6	(GAC)6	(GAC)6	(GAC)6	(GAC)6	(GAC)6	(GAC)6	(GAC)6	-	Introns/Intergenic	-	-	CCATTTGGATTCTGGACCAC	TAATAAGTTTGGCCGGAGGC	160	P	A	A	NA
PMS6208	Os_Chr12	25838893	25838910	(CGG)6	(CGG)5	(CGG)6	(CGG)6	(CGG)6	(CGG)6	(CGG)6	(CGG)6	(CGG)6	(CGG)6	(CGG)6	LOC_Os12g41720	CDS	PLA IIIA/PLP7, putative, expressed	CODON_DELETION	GTCCATGGATGGCAAATGTA	AGCTTGTGAAGGAGCACGAG	273	P	A	A	NA
PMS6209	Os_Chr12	25845452	25845475	(TA)12	(TA)12	(TA)12	(TA)12	(TA)12	(TA)12	(TA)6	(TA)8	(TA)12	(TA)12	(TA)12	-	Introns/Intergenic	-	-	CCACATAGGTCTGCAGCATT	CGGTACTAGCCGTATGCCTC	240	A	A	A	NA
PMS6210	Os_Chr12	25853127	25853144	(AT)9	(AT)9	(AT)9	(AT)9	(AT)8	(AT)9	(AT)7	(AT)9	(AT)9	(AT)9	(AT)9	-	Introns/Intergenic	-	-	GGCATACGGCTAGTACCGAG	GATCGCTGTGAAACGTGCTA	150	P	A	A	NA
PMS6211	Os_Chr12	25853322	25853357	(TA)18	(TA)14	(TA)18	(TA)18	(TA)18	(TA)18	(TA)18	(TA)18	(TA)11	(TA)18	(TA)18	-	Introns/Intergenic	-	-	TTACTCTCGTTCCACACCCC	CTTGCCACCTACCACCTCAT	216	P	A	A	NA
PMS6212	Os_Chr12	25906902	25906939	(CT)19	(CT)19	(CT)19	(CT)19	(CT)19	(CT)19	(CT)19	(CT)14	(CT)19	(CT)19	(CT)19	LOC_Os12g41820	URR	heat shock protein DnaJ, putative, expressed	-	AACAGCTCCAAGAACCCAATCC	GGGATTTCTCGTCGTTTCTTGC	162	A	A	A	RM1310
PMS6213	Os_Chr12	25935445	25935462	(ACG)6	(ACG)5	(ACG)6	(ACG)6	(ACG)6	(ACG)5	(ACG)6	(ACG)6	(ACG)6	(ACG)6	(ACG)5	-	Introns/Intergenic	-	-	CGTGAGCCAATCTGTTTCAG	TGGAAGTTACAACGACGCAG	221	P	A	A	NA

Polymorphic SSR (PMS) markers	Chromosomes	Start physical positions (bp)	End physical positions (bp)	Nipponbare	Kasalath	Nagina22	IR64	Pokkali	Bala	Tainung67	Azucena	Moroberekan	*Oryza rufipogon*	*Oryza nivara*	MSU gene locus IDs	Structural annotation	Functional annotation	SSR functional effects	Forward primers (5'-3')	Reverse primers (5'-3')	Amplified product size (bp)	Markers exhibiting polymorphism within *indica* rice	Transcrption factor genes-derived markers	Known cloned genes-derived markers	PMS markers corresponding with RM markers
PMS6214	Os_Chr12	25999038	25999073	(TC)18	(TC)17	(TC)18	(TC)18	(TC)18	(TC)16	(TC)18	(TC)18	(TC)18	(TC)18	(TC)17	-	Introns/Intergenic	-	-	TTGCTACTACCACAACAGGGTTCC	GCAGCCACAGCTTTGAATAGAGC	144	P	A	A	RM1300
PMS6215	Os_Chr12	26021801	26021824	(CCG)8	(CCG)7	(CCG)8	(CCG)8	(CCG)8	(CCG)6	(CCG)8	(CCG)8	(CCG)8	(CCG)6	(CCG)8	LOC_Os12g41962	URR	nucleolar protein NOP5, putative, expressed	-	TCTCGCCTATTTACCATACTGC	AACCCTCAACTGGAATTACACC	464	P	A	A	RM6189
PMS6216	Os_Chr12	26138732	26138752	(CCG)7	(CCG)7	(CCG)7	(CCG)7	(CCG)7	(CCG)5	(CCG)7	(CCG)7	(CCG)7	(CCG)7	(CCG)7	LOC_Os12g42170	CDS	expressed protein	CODON_DELETION	ACTCGTGGATATCGTGGCTC	GCCGAGTACATCAGCCTCC	166	P	A	A	NA
PMS6217	Os_Chr12	26153134	26153221	(TA)44	(TA)44	(TA)44	(TA)44	(TA)44	(TA)44	(TA)6	(TA)44	(TA)8	(TA)6	(TA)6	-	Introns/Intergenic	-	-	GCCTTCTCTGCTCGCTACTG	GCATTTGGGTCAGCAACTCT	256	A	A	A	NA
PMS6218	Os_Chr12	26163276	26163299	(AAG)8	(AAG)7	(AAG)7	(AAG)8	(AAG)8	(AAG)6	(AAG)8	(AAG)8	(AAG)8	(AAG)8	(AAG)8	-	Introns/Intergenic	-	-	TGCACAACAGGAGTTCAGAAAGC	GACATGTCTCAACGGACGTAGGC	189	P	A	A	RM6953
PMS6219	Os_Chr12	26166381	26166424	(TA)22	(TA)22	(TA)22	(TA)22	(TA)22	(TA)8	(TA)22	(TA)22	(TA)22	(TA)22	(TA)15	-	Introns/Intergenic	-	-	GGGATCCATCTCCATAGCAA	CTCAGTGACATCTCAAGAAATCG	149	P	A	A	NA
PMS6220	Os_Chr12	26173441	26173472	(CT)16	(CT)13	(CT)16	(CT)16	(CT)16	(CT)16	(CT)16	(CT)16	(CT)16	(CT)16	(CT)16	-	Introns/Intergenic	-	-	ATCGCCGAGATCATCGTAAG	AACAGAGCCTTCACCTCGAA	250	P	A	A	NA
PMS6221	Os_Chr12	26181700	26181727	(TA)14	(TA)14	(TA)14	(TA)14	(TA)14	(TA)14	(TA)9	(TA)14	(TA)6	(TA)6	(TA)14	-	Introns/Intergenic	-	-	GGTAACCCTTATGGATATGAGC	TAGCAGCATCTGTCTGTATAGC	338	A	A	A	RM28726
PMS6222	Os_Chr12	26187459	26187524	(TA)33	(TA)33	(TA)33	(TA)33	(TA)33	(TA)33	(TA)7	(TA)33	(TA)33	(TA)33	(TA)33	-	Introns/Intergenic	-	-	TTTATTCCTGTTCTGTCGGAGAGTGC	ACCTCAGCAACTCAAAGTATGCAAGC	387	A	A	A	RM2734
PMS6223	Os_Chr12	26298382	26298399	(CGC)6	(CGC)6	(CGC)6	(CGC)6	(CGC)6	(CGC)6	(CGC)6	(CGC)6	(CGC)6	(CGC)6	(CGC)5	LOC_Os12g42290	CDS	expressed protein	CODON_DELETION	TACATCTCGCAGGGCTTCTT	GAGTAATGGTGGTGGCTGGT	168	A	A	A	NA
PMS6224	Os_Chr12	26327596	26327611	(TA)8	(TA)8	(TA)8	(TA)8	(TA)8	(TA)9	(TA)8	(TA)8	(TA)8	(TA)8	(TA)8	-	Introns/Intergenic	-	-	CTCCCACTCCCATACGACAC	CAAGGCTGAGGGTTGAAAAA	186	P	A	A	NA
PMS6225	Os_Chr12	26330406	26330473	(TA)34	(TA)14	(TA)34	(TA)10	(TA)34	(TA)34	(TA)34	(TA)34	(TA)34	(TA)34	(TA)34	-	Introns/Intergenic	-	-	TTTTTGACGGACGATGGAAG	GCATTGACGTCATCCTTGTG	209	P	A	A	NA
PMS6226	Os_Chr12	26374780	26374799	(CT)10	(CT)10	(CT)10	(CT)9	(CT)10	(CT)10	(CT)10	(CT)10	(CT)10	(CT)10	(CT)9	-	Introns/Intergenic	-	-	TGATTTTCCTTTTTGTCGGC	GCTTGGATGTTTGGAATGTTT	114	P	A	A	NA

Polymorphic SSR (PMS) markers	Chromosomes	Start physical positions (bp)	End physical positions (bp)	Nipponbare	Kasalath	Nagina22	IR64	Pokkali	Bala	Tainung67	Azucena	Moroberekan	*Oryza rufipogon*	*Oryza nivara*	MSU gene locus IDs	Structural annotation	Functional annotation	SSR functional effects	Forward primers (5'-3')	Reverse primers (5'-3')	Amplified product size (bp)	Markers exhibiting polymorphism within *indica* rice	Transcrption factor genes-derived markers	Known cloned genes-derived markers	PMS markers corresponding with RM markers
PMS6227	Os_Chr12	26464108	26464179	(TA)36	(TA)36	(TA)36	(TA)36	(TA)36	(TA)36	(TA)36	(TA)7	(TA)19	(TA)36	(TA)36	-	Introns/Intergenic	-	-	AGGCTTTCTAGTGTTGCTCACA	ATGATGATCGATGCTGATGG	278	A	A	A	NA
PMS6228	Os_Chr12	26487777	26487788	(TA)6	(TA)6	(TA)6	(TA)6	(TA)6	(TA)6	(TA)6	(TA)6	(TA)6	(TA)7	(TA)6	-	Introns/Intergenic	-	-	TTGGATGAGAAAGATGTATAGGCA	TTGGGAATTAAACTAGGCCA	204	A	A	A	NA
PMS6229	Os_Chr12	26507037	26507057	(GGT)7	(GGT)6	(GGT)7	(GGT)7	(GGT)7	(GGT)7	(GGT)7	(GGT)7	(GGT)7	(GGT)7	(GGT)7	LOC_Os12g42650	CDS	pollen preferential protein, putative, expressed	CODON_DELETION	ACCTCCTCACCCTCCTCGT	CCACCTCTCTCTCTCGCATC	244	P	A	A	NA
PMS6230	Os_Chr12	26542373	26542426	(AT)27	(AT)27	(AT)24	(AT)8	(AT)24	(AT)27	(AT)27	(AT)27	(AT)27	(AT)27	(AT)9	-	Introns/Intergenic	-	-	CGCGATACCGATCCACTAAT	CAGCCGAATAAACAGTAAACTGAA	256	P	A	A	NA
PMS6231	Os_Chr12	26616840	26616860	(CAC)7	(CAC)8	(CAC)8	(CAC)8	(CAC)8	(CAC)7	(CAC)7	(CAC)7	(CAC)7	(CAC)8	(CAC)7	LOC_Os12g42830	CDS	expressed protein	CODON_INSERTION	AACTGCTCTGGCTGGAAAAA	TTCCTCGATGTTCTCTTCCC	220	P	A	A	NA
PMS6232	Os_Chr12	26640549	26640569	(CCG)7	(CCG)7	(CCG)7	(CCG)7	(CCG)7	(CCG)7	(CCG)7	(CCG)5	(CCG)7	(CCG)7	(CCG)7	-	Introns/Intergenic	-	-	TCATAGAACTCCCCGAGCTG	GACAACCTCTCCGACGACAC	239	A	A	A	NA
PMS6233	Os_Chr12	26757685	26757705	(GGC)7	(GGC)7	(GGC)7	(GGC)7	(GGC)7	(GGC)7	(GGC)7	(GGC)5	(GGC)7	(GGC)7	(GGC)7	LOC_Os12g43090	CDS	expressed protein	CODON_DELETION	CTTCATCACCGCCTCCGTTCC	AGCTCTCTCCCGCTCTGGATGC	205	A	A	A	RM28778
PMS6234	Os_Chr12	26762288	26762320	(GCG)11	(GCG)11	(GCG)11	(GCG)11	(GCG)8	(GCG)11	(GCG)11	(GCG)11	(GCG)11	(GCG)11	(GCG)11	LOC_Os12g43100	URR	deoxyhypusine hydroxylase, putative, expressed	-	CAGAGGAACCCCTCCATCTC	TAGTGGGTTTCATGGGCTTC	222	P	A	A	NA
PMS6235	Os_Chr12	26845249	26845262	(GA)7	(GA)7	(GA)7	(GA)6	(GA)7	(GA)7	(GA)7	(GA)7	(GA)7	(GA)7	(GA)7	-	Introns/Intergenic	-	-	GGGAGAGAAGGAGAGGGAGA	CCTTGGTCTAAAAGGAGCCC	225	P	A	A	NA
PMS6236	Os_Chr12	26902227	26902244	(GCA)6	(GCA)6	(GCA)6	(GCA)6	(GCA)6	(GCA)6	(GCA)6	(GCA)6	(GCA)6	(GCA)5	(GCA)6	LOC_Os12g43363	URR	fatty acid hydroxylase, putative, expressed	-	TCCCACCACCTTGAGAAGAG	GAGATCGAGACGGGAATCTG	213	A	A	A	NA
PMS6237	Os_Chr12	26931816	26931831	(AT)8	(AT)8	(AT)8	(AT)6	(AT)8	(AT)8	(AT)8	(AT)8	(AT)8	(AT)8	(AT)8	-	Introns/Intergenic	-	-	AAACCTTGAGAGGCCCAAAC	CAAAGCGTAGGGCATCATCT	121	P	A	A	NA
PMS6238	Os_Chr12	26984734	26984748	(CGC)5	(CGC)6	(CGC)5	(CGC)5	(CGC)5	(CGC)5	(CGC)5	(CGC)5	(CGC)5	(CGC)5	(CGC)5	LOC_Os12g43500	CDS	expressed protein	CODON_INSERTION	AGGCGATGAGAGTTGTCAGG	CGTGTCACCCATCCTTCTCT	272	P	A	A	NA
PMS6239	Os_Chr12	27063735	27063755	(CGG)7	(CGG)7	(CGG)7	(CGG)5	(CGG)7	(CGG)5	(CGG)7	(CGG)7	(CGG)7	(CGG)6	(CGG)7	-	Introns/Intergenic	-	-	GGAATCCTGACAAGCGAGAG	CTTAAAGGCACAGGCAAAGC	250	P	A	A	NA

Polymorphic SSR (PMS) markers	Chromosomes	Start physical positions (bp)	End physical positions (bp)	Nipponbare	Kasalath	Nagina22	IR64	Pokkali	Bala	Tainung67	Azucena	Moroberekan	*Oryza rufipogon*	*Oryza nivara*	MSU gene locus IDs	Structural annotation	Functional annotation	SSR functional effects	Forward primers (5'-3')	Reverse primers (5'-3')	Amplified product size (bp)	Markers exhibiting polymorphism within *indica* rice	Transcrption factor genes-derived markers	Known cloned genes-derived markers	PMS markers corresponding with RM markers
PMS6240	Os_Chr12	27088039	27088053	(ACT)5	(ACT)6	(ACT)5	(ACT)5	(ACT)5	(ACT)5	(ACT)5	(ACT)5	(ACT)5	(ACT)5	(ACT)6	-	Introns/Intergenic	-	-	CCGTGTGCGGTAGTAGACAA	GCTGCTCCTCCTCCTCCTAT	240	P	A	A	NA
PMS6241	Os_Chr12	27182444	27182465	(CT)11	(CT)15	(CT)11	(CT)11	(CT)11	(CT)15	(CT)11	(CT)11	(CT)11	(CT)11	(CT)11	-	Introns/Intergenic	-	-	AATTTCTCTCGGCCCATCTT	GGAGAGGGAGGACTTGGCT	212	P	A	A	NA
PMS6242	Os_Chr12	27294049	27294072	(CTC)8	(CTC)5	(CTC)5	(CTC)5	(CTC)5	(CTC)5	(CTC)8	(CTC)8	(CTC)8	(CTC)5	(CTC)5	LOC_Os12g43990	CDS	expressed protein	CODON_DELETION	ATGGATCTTGCACCACAACA	ACCGCCTCAGAATGTTCATC	171	A	A	A	NA
PMS6243	Os_Chr12	27430297	27430312	(GA)8	(GA)9	(GA)8	(GA)8	(GA)8	(GA)8	(GA)8	(GA)8	(GA)8	(GA)8	(GA)9	LOC_Os12g44230	URR	expressed protein	-	AGAAGGAAGGACCGAAGGAG	TGACTGGAGTCCGTGCATAG	275	P	A	A	NA
PMS6244	Os_Chr12	27466213	27466244	(CT)16	(CT)16	(CT)16	(CT)16	(CT)16	(CT)18	(CT)16	(CT)16	(CT)16	(CT)16	(CT)16	-	Introns/Intergenic	-	-	AGGACCCAATCCAAAGTTTGTCC	AGCAGAGGCATAAATGCAAACC	231	P	A	A	RM28825

PMS: Polymorphic SSRs, P: Present and A: Absent

Table S3. Characteristics of a high-density genetic linkage map constructed using a 190 F_4 rice mapping population (IR 64 x Sonasal)

**Linkage groups (LGs)/chromosomes**	***In silico* polymorphic SSR markers mapped**	**Map length covered (cM)**	**Mean inter-marker distance (cM)**
Os_Chr01	485	232.216	0.479
Os_Chr02	397	199.941	0.504
Os_Chr03	420	201.173	0.479
Os_Chr04	297	153.484	0.517
Os_Chr05	302	161.194	0.534
Os_Chr06	301	149.191	0.496
Os_Chr07	282	162.086	0.575
Os_Chr08	270	161.380	0.598
Os_Chr09	218	133.084	0.610
Os_Chr10	235	150.177	0.639
Os_Chr11	288	172.137	0.598
Os_Chr12	296	184.120	0.622
**Total**	**3791**	**2060.183**	**0.543**

Table S4. Comparative overview between traditional QTL-seq and high-resolution QTL region-specific integrated SSR- and SNP marker-based QTL-seq strategies

**Characteristics**	**Conventional QTL-seq**	**QTL region-specific integrated SSR- and SNP marker-based QTL-seq**
**Whole genome resequencing**	Essential	Not essential<br>Needs targeted resequencing of only known QTL region
**Overall sequencing genome coverage produced**	Low (~10-20X) genome coverage (resolution)	High (~100X) genome coverage (resolution)
**Simultaneous use of SNP and SSR markers for QTL-seq analysis**	Not feasible (able to detect only SNPs due to low genome coverage)<br>Use Δ(SNP-index)-based QTL detection method	Feasible (able to detect both SSRs and SNPs due to high genome coverage)<br>Use Δ(SNP/SSR-index)-based QTL detection method
**Previous information on QTLs from conventional SSR marker-based QTL mapping**	Not required	Required (known strong trait-associated major QTL information from SSR marker-based traditional QTL mapping)
**Selection of homozygous individuals from a mapping population for constituting bulks with extreme (high and low) phenotypic trait values**	Selection is solely based on phenotyping information therefore, this approach likely selects inaccurate extreme bulks and detects erroneous QTLs.	Selection is based on both phenotyping and genotyping information of genome-wide and known QTL-linked markers (identified through SSR marker-based traditional QTL mapping), therefore this approach most likely selects accurate extreme bulks and detects non-erroneous QTLs.
**Possibility of spurious QTL detection**	Possibly prone to detect spurious QTLs.<br>It could be due to:<br>- Bulking of homozygous mapping individuals exhibiting extreme phenotypic trait values with almost similar genetic constitution types (selected primarily by phenotyping) resulting over-estimation of Δ(SNP-index)-based QTL detection in QTL-seq.<br>- Faulty selection of homozygous mapping individuals exhibiting extreme phenotypic trait values for bulking, which majorly relies on phenotyping data.	Less prone to detect spurious QTLs.<br>Since, this approach involves bulking of desirable low and high homozygous mapping individuals (extreme phenotypic trait value) with varied genetic constitution types (selected using both phenotyping and genotyping data) and utilizes QTL information of already known QTL identified by SSR marker-based traditional QTL mapping.
**Genomics-assisted breeding applications**	Deals with identification of novel unknown major QTLs governing multiple complex quantitative traits.	Deals with identification/revalidation of known major QTLs (identified through SSR marker-based traditional QTL mapping) and fine-mapping of known QTLs controlling diverse complex quantitative traits.
